# Cellular Response to Ciprofloxacin in Low-Level Quinolone-Resistant *Escherichia coli*

**DOI:** 10.3389/fmicb.2017.01370

**Published:** 2017-07-19

**Authors:** Jesús Machuca, Esther Recacha, Alejandra Briales, Paula Díaz-de-Alba, Jesús Blazquez, Álvaro Pascual, José-Manuel Rodríguez-Martínez

**Affiliations:** ^1^Unidad Intercentros de Enfermedades Infecciosas, Microbiología y Medicina Preventiva, Hospital Universitario Virgen Macarena y Virgen del Rocío Seville, Spain; ^2^Instituto de Biomedicina de Sevilla, Hospital Universitario Virgen del Rocío/Consejo Superior de Investigaciones Científicas/Universidad de Sevilla Sevilla, Spain; ^3^Red Española de Investigación en Patología Infecciosa, Instituto de Salud Carlos III Madrid, Spain; ^4^Departamento de Microbiología, Universidad de Sevilla Sevilla, Spain; ^5^Centro Nacional de Biotecnología, Consejo Superior de Investigaciones Científicas Madrid, Spain

**Keywords:** ciprofloxacin, low-level quinolone resistance, survival, transcriptomic, global response, sensitization

## Abstract

Bactericidal activity of quinolones has been related to a combination of DNA fragmentation, reactive oxygen species (ROS) production and programmed cell death (PCD) systems. The underlying molecular systems responsible for reducing bactericidal effect during antimicrobial therapy in low-level quinolone resistance (LLQR) phenotypes need to be clarified. To do this and also define possible new antimicrobial targets, the transcriptome profile of isogenic *Escherichia coli* harboring quinolone resistance mechanisms in the presence of a clinical relevant concentration of ciprofloxacin was evaluated. A marked differential response to ciprofloxacin of either up- or downregulation was observed in LLQR strains. Multiple genes implicated in ROS modulation (related to the TCA cycle, aerobic respiration and detoxification systems) were upregulated (*sdhC* up to 63.5-fold) in mutants with LLQR. SOS system components were downregulated (*recA* up to 30.7-fold). *yihE*, a protective kinase coding for PCD, was also upregulated (up to 5.2-fold). SdhC inhibition sensitized LLQR phenotypes (up to ΔLog = 2.3 after 24 h). At clinically relevant concentrations of ciprofloxacin, gene expression patterns in critical systems to bacterial survival and mutant development were significantly modified in LLQR phenotypes. Chemical inhibition of SdhC (succinate dehydrogenase) validated modulation of ROS as an interesting target for bacterial sensitization.

## Introduction

Enterobacteriaceae, particularly *Escherichia coli*, are among the most common organisms causing community, nosocomial, and opportunistic infections (Vila et al., 2016). Fluoroquinolones are commonly used for empirical and directed therapy in infections caused by *E. coli* (Vila et al., 2016). Over the past three decades, quinolone resistance in Enterobacteriaceae from human and veterinary isolates has increased (Rodríguez-Martínez et al., 2016b). Known mechanisms of fluoroquinolone resistance occur principally through chromosomal mutations in genes encoding the quinolone targets (DNA gyrase and topoisomerase IV), and to a lesser extent through decreased permeability (implicating upregulation of the AcrAB-TolC multidrug efflux pump) (Blair et al., 2015; Hooper and Jacoby, 2015). Additionally, plasmid-mediated quinolone resistance (PMQR) mechanisms (Qnr proteins that protect the quinolone targets; the acetylation of ciprofloxacin and norfloxacin by Aac(6′)-Ib-cr; and the plasmid-mediated efflux pumps, QepA and OqxAB) have also been described and are epidemiologically relevant (Rodríguez-Martínez et al., 2011, 2016b; Jacoby et al., 2014). All these determinants (chromosomal or plasmid mediated) on their own confer low-level quinolone resistance (LLQR), and multiple mechanisms must be combined to achieve clinical levels of resistance (Morgan-Linnell and Zechiedrich, 2007; Morgan-Linnell et al., 2009; Briales et al., 2011; Machuca et al., 2014).

It is of crucial medical importance to understand the molecular mechanisms that promote the development of antimicrobial resistance as well as to clarify the molecular responses to antimicrobials that lead to the survival or death of the cell (Palmer and Kishony, 2013; Baym et al., 2016). The bactericidal activity of quinolones in bacteria has been related to a combination of DNA fragmentation, reactive oxygen species (ROS) production and programmed cell death (PCD) systems mediated by toxin-antitoxin (TA) modules such as *mazEF* (Drlica et al., 2009; Erental et al., 2014; Zhao et al., 2015). The SOS system response has also been postulated as a formidable strategy against aggressions such as antimicrobial exposure, inducing the transient expression of mutational machinery (Baharoglu and Mazel, 2014). The intensity and role of all these processes implicated in survival and mutant emergence would be proportional to drug concentration (and a function of susceptibility levels directly related to MIC values).

The impact of LLQR mechanisms on the decreased bactericidal effect of these antimicrobial agents and their increased influence on the emergence of high-level resistance seems to be clear. However, the underlying molecular systems responsible for this phenomenon in clinical setting still need to be clarified. In order to do this in terms of genetic expression, this study evaluated the transcriptome profiling of isogenic *E. coli* harboring frequent quinolone resistance mechanisms (chromosomal and plasmid-mediated) in the presence of clinically significant concentrations of ciprofloxacin (breakpoint for reduced susceptibility which is near to 1/2 of serum Cmax) (Mandell et al., 2010). We also evaluated enhancing ciprofloxacin activity by targeting ROS modulation, which was greatly altered in LLQR strains.

## Materials and Methods

### Bacterial Strains

*Escherichia coli* ATCC^®^ 25922^TM^ was used as the background strain. This microorganism is a recommended CLSI control strain used worldwide for antimicrobial susceptibility testing (including quinolones). First, four LLQR isogenic strains were tested using a global transcriptomic approach: ATCC 25922 (wild-type, MIC for ciprofloxacin 0.004 μg/mL); EC14 (*E. coli* ATCC 25922 coding for QnrS1, MIC for ciprofloxacin 0.125 μg/mL); EC19 (*E. coli* ATCC 25922 with deleted *marR* gene and coding for QnrS1, MIC for ciprofloxacin 0.5 μg/mL); and EC24 (*E. coli* ATCC 25922 with the Ser83Leu substitution in GyrA and coding for QnrS1, MIC for ciprofloxacin 1 μg/mL) (**Table 1**; Machuca et al., 2014). All of these were susceptible to quinolones according to CLSI breakpoints (CLSI). Second, *E. coli* ATCC 25922 and two isogenic mutants harboring chromosomal and/or PMQR mechanisms (EC04: *E. coli* ATCC 25922 with the Ser83Leu substitutions in GyrA and Ser80Arg in ParC, MIC for ciprofloxacin 0.5 μg/mL; and EC59: *E. coli* ATCC 25922 with the Ser83Leu and Asp87Arg substitutions in GyrA and Ser80Arg in ParC, deleted *marR* gene and coding for QnrS1, MIC for ciprofloxacin 32 μg/mL) were used for *sdhC* (succinate dehydrogenase complex subunit C) or *cyoA* (cytochrome oxidase subunit II) inactivation, as described (**Table 1**; Datsenko and Wanner, 2000; Machuca et al., 2014). *qnrS1* gene was cloned into pBK-CMV vector as described previously (Machuca et al., 2014). Empty pBK-CMV vector was used as control and also introduced into *E. coli* ATCC 25922 and EC04 for comparison.

**Table 1:** Bacterial strains used in this study and ciprofloxacin susceptibility.

Strain	Genetic description	CIP^a^	Use in this study	Reference
*E. coli* ATCC 25922	Wild-type *E. coli*	0.004	Transcriptomic assays	7
EC14	*E. coli* ATCC 25922 coding for QnrS1	0.125	Transcriptomic assays	7
EC19	*E. coli* ATCC 25922 with deleted marR gene and coding for QnrS1	0.5	Transcriptomic assays	7
EC24	*E. coli* ATCC 25922 with the substitution Ser83Leu in GyrA and coding for QnrS1	1	Transcriptomic assays	7
EC04	*E. coli* ATCC 25922 with the Ser83Leu substitution in GyrA and Ser80Arg in ParC	0.5	Viability assays (Killing curves)	7
EC59	*E. coli* ATCC 25922 with substitutions Ser83Leu+Asp87Asn in GyrA, Ser80Arg in ParC, the deleted marR gene and coding for QnrS1	32	Viability assays (Killing curves)	7
*E. coli* ATCC 25922 Δ*sdhC*	*E. coli* ATCC 25922 with sdhC deleted	0.004	Viability assays (Killing curves)	This study
*E. coli* ATCC 25922 Δ*cyoA*	*E. coli* ATCC 25922 with cyoA deleted	0.004	Viability assays (Killing curves)	This study
EC04 Δ*sdhC*	*E. coli* ATCC 25922 with substitutions Ser83Leu in GyrA and Ser80Arg in ParC with deleted sdhC	0.5	Viability assays (Killing curves)	This study
EC04 Δ*cyoA*	*E. coli* ATCC 25922 with substitutions Ser83Leu in GyrA and Ser80Arg in ParC with deleted cyoA	0.5	Viability assays (Killing curves)	This study
EC59 Δ*sdhC*	*E. coli* ATCC 25922 with substitutions Ser83Leu+Asp87Asn in GyrA, Ser80Arg in ParC, deleted marR gene and coding for QnrS1 with deleted sdhC	32	Viability assays (Killing curves)	This study
EC59 Δ*cyoA*	*E. coli* ATCC 25922 with the substitutions Ser83Leu+Asp87Asn in GyrA, Ser80Arg in ParC, deleted marR gene and coding for QnrS1	32	Viability assays (Killing curves)	This study
	With deleted cyoA			

*^a^MIC (μg/mL) of ciprofloxacin.*

### Experimental Conditions and Microarray RNA Analysis

*Escherichia coli* ATCC 25922 (wild-type) and isogenic EC14, EC19, EC24 (LLQR) strains were tested to evaluate the global response to relevant fixed concentrations of ciprofloxacin (1 μg/mL, the breakpoint for reduced susceptibility according to CLSI and near to 1/2 of serum Cmax) (Mandell et al., 2010). Transcriptomic profile of *E. coli* ATCC 25922 was also compared to EC14 in absence of ciprofloxacin as control. All were susceptible to quinolones according to CLSI breakpoints (CLSI). Cultures were started from single colonies and grown overnight in 25 ml of LB (Luria Bertani medium, Becton Dickinson, Le Pont-de-Claix, France). These cells were diluted 1:100 and grown to cell concentrations of 4x10^8^ cells/ml (OD_600nm_ = 0.4, exponential phase) for treatment. Three biological replicates per genotype were incubated at 1 μg/mL ciprofloxacin for 60 min (i.e., 250xMIC for *E. coli* ATCC 25922, 8xMIC for EC14, 2xMIC for EC19 and 1xMIC for EC24). Approximately 10^9^ cells (2 ml) were taken for RNA isolation. The pellet was processed immediately. The pellet was pre-incubated in RNAprotect Bacteria Reagent (Qiagen, Hilden, Germany). RNA extraction was performed using the RNeasy Mini Kit (Qiagen, Hilden, Germany). Contaminating DNA was removed from RNA samples with TURBO DNA-free (Ambion, United States). The RNA concentration was measured using NanoDrop ND-1000 (Thermo Scientific). The integrity of the RNA samples was analyzed with the BioAnalyzer 2100 (Agilent Technologies) using the RNAnano 6000 kit (Agilent Technologies). Labeling, microarray hybridization, scanning, and data processing were performed in the Genomics Unit of the Centro Nacional de Biotecnología^1^. The data obtained from each LLQR mutant was always compared with the *E. coli* ATCC 25922 wild-type strain. The FIESTA program was used for analysis^2^ and the *p*-value was determined according to the FDR algorithm (*p* < 0.05 was considered significant). Microarray RNA data are available at GEO NCBI^3^ (accession number GSE86341).

### Annotation of Gene Functions and Regulation

Gene functions (COG, Clusters of Orthologous Groups) (Tatusov et al., 2000) were identified using the following bioinformatics resources for *E. coli*: ECOCYC^4^, ECOGENE^5^, Gene Expression Database^6^, KEGG^7^, BPROM^8^, and DAVID^9^. Venn diagrams were created using the http://bioinfogp.cnb.csic.es/tools/venny/index.html website.

### Microarray RNA Data Validation

Real time RT-PCR was used, as described (Rodríguez-Martínez et al., 2012), to confirm specific transcriptome data (*sdhC, sucD, nouH, recA, focA, tnaA* genes), both in presence and absence of ciprofloxacin. The LightCycler FastStart DNA Master SYBR Green I Kit (Roche) was used for amplification. To normalize expression levels, target gene transcripts were calculated relative to the *mdh* gene, using the 2^-ΔΔCT^ method. The primers used for gene amplification are indicated in Supplementary Table 1.

### Antimicrobial Susceptibility Testing

Antimicrobial susceptibility was determined by microdilution according to CLSI guidelines (CLSI). The quinolones tested were ciprofloxacin, norfloxacin, ofloxacin, levofloxacin, moxifloxacin and nalidixic acid (Sigma–Aldrich, Madrid, Spain).

### Time-Kill Curve Assays

Potentiating antibacterial activity by enhancing microbial ROS production has been demonstrated previously (Brynildsen et al., 2013). To test the impact of ROS modulation (by *sdhC* or *cyoA* deletion) on bacterial viability in *E. coli* ATCC 25922, EC04 and EC59 strains, time-kill curves were assayed in MHB (Mueller-Hinton broth, Becton Dickinson, Le Pont-de-Claix, France) at ciprofloxacin and ofloxacin concentrations of 2xMIC (**Table 1**). Growth in drug-free broth was evaluated in parallel, as a control. Cultures were incubated at 37°C, and shaken at 180rpm. An initial inoculum of 10^6^ CFU/mL from a fresh overnight culture was used in all experiments. Bacterial concentrations were determined at 0, 1, 2, 3, 4, and 24 h by colony counting on drug-free agar.

In parallel, the time courses of *E. coli* ATCC 25922, EC04 and EC59 cells treated with carboxin (500 μM), an inhibitor of succinate dehydrogenase (Brynildsen et al., 2013), and a fluoroquinolone (ciprofloxacin or ofloxacin at 2xMIC), were compared with treatment with fluoroquinolones alone. Carboxin (Sigma–Aldrich, Madrid, Spain) was dissolved in 100% ethanol (Brynildsen et al., 2013).

## Results

### Global Expression Profiles

**FIGURE 1:**
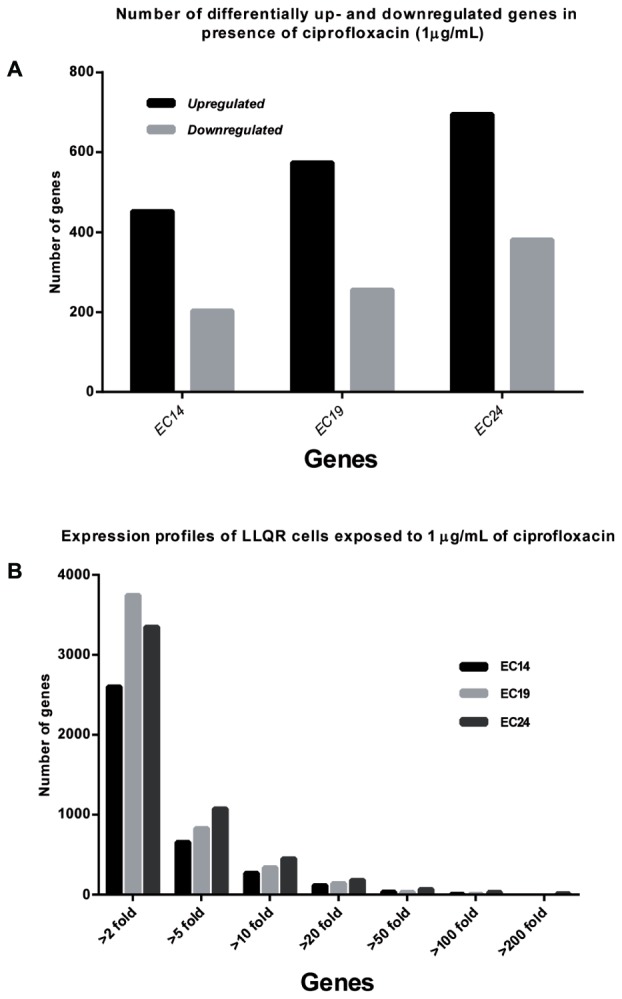
Global expression profiles of cells with low-level quinolone resistance (LLQR) exposed to 1 μg/mL of ciprofloxacin and compared with the wild-type cells in the same conditions. **(A)** Number of differentially up- and downregulated genes (≥ or ≤5-fold) in the presence of ciprofloxacin (1 μg/mL) in LLQR cells compared to wild-type cells in the same conditions. **(B)** Number of genes up- or downregulated, grouped according to the factor of differential expression in LLQR cells compared to the wild-type cells in the same conditions. Genes were included when differences were significant (*p* < 0.05). EC14 means *E. coli* ATCC 25922 pBK-QnrS1; EC19 means *E. coli* ATCC 25922 Δ*marR* pBK-QnrS1; and EC24 means *E. coli* ATCC 25922 S83L pBK-QnrS1.

In order to establish the role of LLQR phenotypes on the differential expression in response to quinolones, the expression profiles of isogenic *E. coli* carrying LLQR harboring frequent chromosomal and PMQR mechanisms (EC14 coding for QnrS1; EC19 with deleted *marR* gene and coding for QnrS1; and EC24 with the Ser83Leu substitution in GyrA and coding for QnrS1) (Machuca et al., 2014) were evaluated in the presence of significant clinical concentrations of ciprofloxacin (1 μg/mL, the breakpoint for reduced susceptibility according to CLSI and near to 1/2 of serum Cmax) (Mandell et al., 2010; Clinical and Laboratory Standards Institute [CLSI], 2016) and compared with those of wild-type *E. coli* ATCC 25922 (**Figure 1** and Supplementary Tables 2, 3). The MIC values of ciprofloxacin for these susceptible LLQR strains (EC14, EC19, EC24) were 32-, 125- and 250-fold higher compared to the wild-type strain. As expected, significant differences were observed in the numbers of differentially up- and downregulated genes (>5-fold, *p* < 0.05). Most genes were upregulated and the differences were proportional to the MIC values for strains with LLQR (EC14: 452/203 genes; EC19: 574/256 genes; EC24: 695/381 genes, upregulated and downregulated, respectively) (**Figure 1A**). Expression of >2500 genes, whether by upregulation or downregulation, varied more than > 2-fold, reflecting the marked differential response to ciprofloxacin in LLQR strains. Under these this clinically significant concentration of ciprofloxacin, variations of > 10-fold for EC14/EC19/EC24 (271/339/450 genes), of > 50-fold (34/32/68 genes) and of > 100-fold (8/8/33 genes) were observed (**Figure 1B**). Just a few genes had differential expression of more than 200-fold for LLQR cells compared to the wild-type (16 genes for EC24).

**FIGURE 2:**
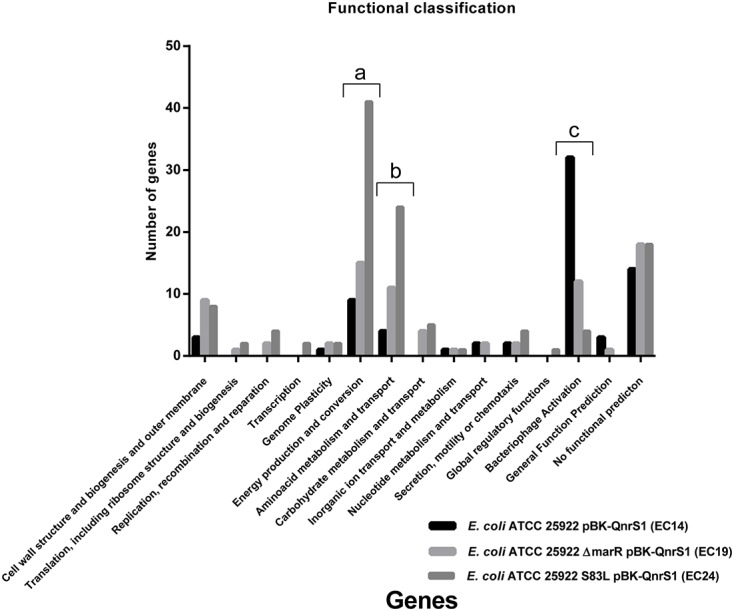
Gene expression classified by COG (Cluster of Orthologous Groups) (Tatusov et al., 2000) for functional categories (greater than or equal to 30-fold, *p*-value < 0.05).

**Figure 2** shows gene expression classified according to the COG functional categories (Tatusov et al., 2000) (showing values equal to or more than 30-fold, that is, large differences, *p*-value < 0.05) (see also Supplementary Table 3). Among these genes, four functional groups were chiefly affected, related to the number of genes involved. “Energy production and conversion genes (EPC)” and “Amino acid metabolism and transport genes (AMT)” tended to be genes upregulated at higher levels (≥30-fold). These differences were proportional to MIC values for strains with LLQR (EC14: 9/4 genes; EC19: 15/11 genes; EC24: 41/24 genes for EPC and AMT genes, respectively) (**Figure 2**). A reduction of genes related to “Bacteriophage Activation (BA)” was observed counter to MIC values for LLQR strains (mostly upregulated in EC14 and EC19) (**Figure 2**), while there was a considerable number of highly altered genes without functional prediction (both up- and downregulated).

**FIGURE 3:**
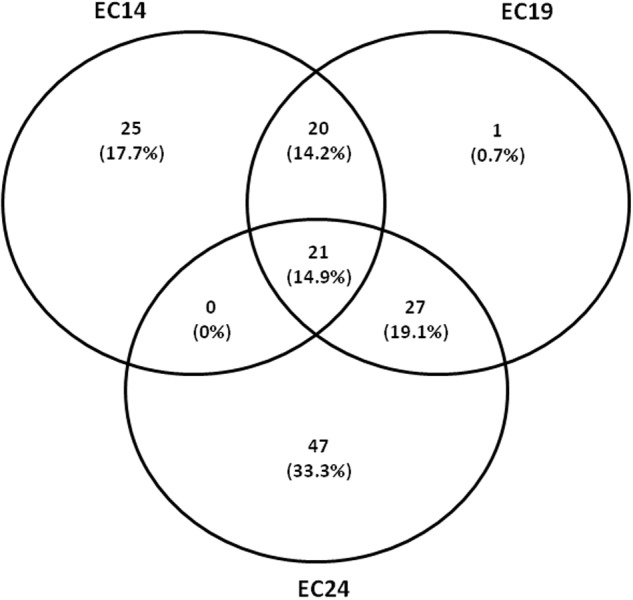
Overlap between differently expressed genes (greater than or equal to 30-fold, *p*-value < 0.05) on exposure to ciprofloxacin (1 μg/mL) between low-level quinolone resistant (LLQR) cells compared to the wild-type cells in the same conditions. Venn diagram shows the overlap. The numbers indicated on the diagram refer to the number of genes with significantly altered expression levels in one or both conditions. LLQR phenotypes: EC14 means *E. coli* ATCC 25922 pBK-QnrS1; EC19 means *E. coli* ATCC 25922 Δ*marR* pBK-QnrS1; and EC24 means *E. coli* ATCC 25922 S83L pBK-QnrS1.

**Figure 3** shows differentially expressed genes equal to or greater than 30-fold (*p*-value < 0.05) (regarded as major differences in terms of expression). Interestingly, the strains with LLQR were proportionally reorganized in response to ciprofloxacin stress (**Figure 3**). In this regard, 141 genes showed altered expression for at least one strain with LLQR, and 14.9% of these were shared in all three strains. In total, under ciprofloxacin-induced stress, 25 and 47 genes were specifically affected in EC14 and EC24, respectively, while neither of these strains shared any genes with altered expression. EC19, on the other hand, shared 20 and 27 genes with EC14 and EC24, respectively. These data indicate that, with respect to EC14 and EC24, EC19 is at a transitional stage in its response to ciprofloxacin (see also Supplementary Table 3).

The transcriptome microarray data for six specific genes was validated by quantitative RT-PCR, confirming both the upregulation and downregulation results in presence of ciprofloxacin (Supplementary Table 1). No significant differences were observed for these genes in absence of ciprofloxacin (data not shown). No significant differences were observed when *E. coli* ATCC 25922 and EC14 were compared in absence of ciprofloxacin (data not shown).

### Increased ROS Modulation in Isogenic *E. coli* with LLQR

**FIGURE 4:**
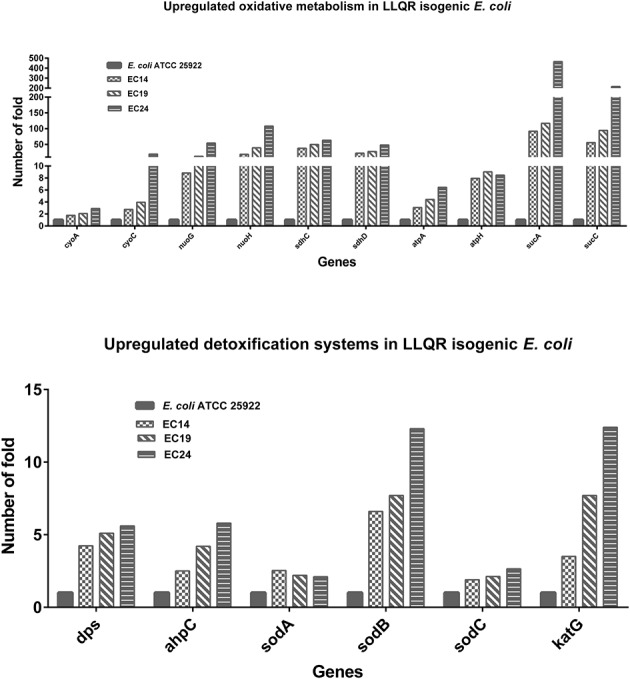
Impact on ROS response of low-level quinolone-resistant (LLQR) cells exposed to 1 μg/mL of ciprofloxacin compared to wild-type cells in the same conditions. LLQR phenotypes: EC14 means *E. coli* ATCC 25922 pBK-QnrS1; EC19 means *E. coli* ATCC 25922 ΔmarR pBK-QnrS1; and EC24 means *E. coli* ATCC 25922 S83L pBK-QnrS1. All indicated genes show a significantly different pattern of expression between LLQR strains and wild-type *E. coli* (*p*-value < 0.05). Standard deviations were within 10% of the means.

Multiple genes implicated in oxidative metabolism and modulation of ROS [related to the TCA cycle (*sdhC*: Succinate dehydrogenase; *pta*: Phosphotransacetylase; *sucA-D*: Succinyl CoA synthase complex; up to 495-fold), aerobic respiration (*cyoA-E*: Cytochrome oxidase; nuoG or nuoH: Ubiquinone oxidoreductase; *atpH*: ATP synthase; up to 108-fold), glucose metabolism (*gnd*: Gluconate-P dehydrogenase; *zwf*: Glucose-6-phosphate 1-dehydrogenase; up to 5.5-fold) or detoxification systems (*dps*: Stress-induced Fe-binding, *ahpC*: Alkyl hydroperoxide reductase, *sodB*: Superoxide dismutase, *katG*: Catalase-peroxidase; up to 12.4-fold)] were highly upregulated in LLQR mutants (**Figure 4** and Supplementary Table 2). Proteome profiling based on LC-MS/MS (liquid chromatography–mass spectrometry) showed a similar trend with proteins related to ROS modulation over-represented in LLQR mutants (Supplementary Table 4). Differences observed were proportional to MIC values for strains with LLQR. These data indicate that strains with LLQR would produce lower levels of ROS in the presence of clinical concentrations of ciprofloxacin (Brynildsen et al., 2013; Dwyer et al., 2014).

### Moderate SOS Response in Isogenic *E. coli* with LLQR

**FIGURE 5:**
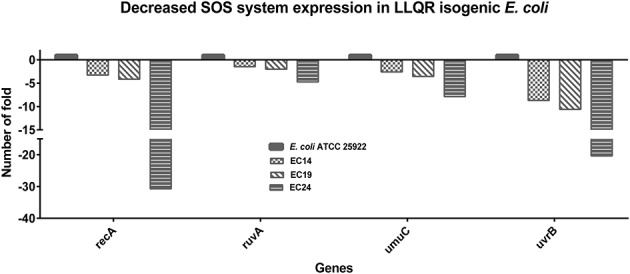
Impact on SOS response of low-level quinolone-resistant (LLQR) cells exposed to 1 μg/mL of ciprofloxacin, compared to wild-type cells in the same conditions. LLQR phenotypes: EC14 means *E. coli* ATCC 25922 pBK-QnrS1; EC19 means *E. coli* ATCC 25922 ΔmarR pBK-QnrS1; and EC24 means *E. coli* ATCC 25922 S83L pBK-QnrS1. All indicated genes shown a pattern of expression significantly different among LLQR strains and wild-type *E. coli* (*p*-value < 0.05). Standard deviations were within 10% of the means.

Genes involved in DNA repair and the SOS response are highly upregulated in response to quinolones (Dwyer et al., 2007; Baharoglu and Mazel, 2014). In our experiment, the relative concentrations of ciprofloxacin used were 250xMIC for *E. coli* ATCC 25922, 8xMIC for EC14, 2xMIC for EC19 and 1xMIC for EC24. Under this clinically significant concentration of ciprofloxacin, a clear reduction of SOS system gene expression was observed in strains with LLQR [related to SOS system activation (*recA*: the multifunctional DNA recombination and repair protein and master regulator of the SOS system; up to -30.7-fold), DNA repair by homologous recombination (*ruvA*: Holliday junction recognition protein; up to -4.75-fold), DNA nucleotide excision repair (*uvrB*: Excision nuclease subunit B; up to -20.3-fold) or DNA repair by translesion synthesis (*umuC*: Translesion DNA polymerase V; up to -7.9-fold)] (**Figure 5** and Supplementary Table 2).

### Impact of LLQR Phenotypes on Other Bacterial Survival Systems

The PCD and TA systems have been linked to cell death in response to quinolones (Erental et al., 2012, 2014). We analyzed patterns of expression of multiple TA systems (both type I and type II) and found both upregulated (*gnsA*, *ymcE*, *yjhQ*, *yjhX*, or *yihE*) and downregulated genes (*hokA*, *hokD*, *symE*, *chpBK*, *yhaV*, *yefM*, *yoeB*, or *yfjF*) in strains with LLQR (Yamaguchi and Inouye, 2011). Interestingly, the *yihE* gene (coding for a key kinase regulator that protects *E. coli* from antimicrobials like quinolones by antagonizing the MazEF TA module) was upregulated in LLQR strains (Supplementary Figure 1 and Table 2) (Dorsey-Oresto et al., 2013).

Altered expression of downstream genes linked to the *mazEF* pathway involved in PCD control was also detected (Erental et al., 2014). *yfiD*, *clpX*, *clpP*, and *yfbU* were upregulated; *slyD* and *ygcR* were downregulated in strains with LLQR. Interestingly, genes for the major quinolone target in Gram-negative bacteria, the DNA gyrase subunits, tended to be downregulated (*gyrA*: DNA gyrase, subunit A, up to -7.18-fold and *gyrB*: DNA gyrase, subunit B, up to -8.9-fold) in strains with LLQR. Finally, several genes implicated in the DNA mismatch repair system tended to be downregulated (*mutH*: up to -2.84-fold; *mutL*: up to -2.14-fold; *mutM*: up to -5.57-fold; *mutY*: up to -4.03-fold) in LLQR strains. *mutS* was not significantly affected (Supplementary Figure 1 and Table 2).

### Sensitization of LLQR Phenotypes by Targeting Oxidative Metabolism

**FIGURE 6:**
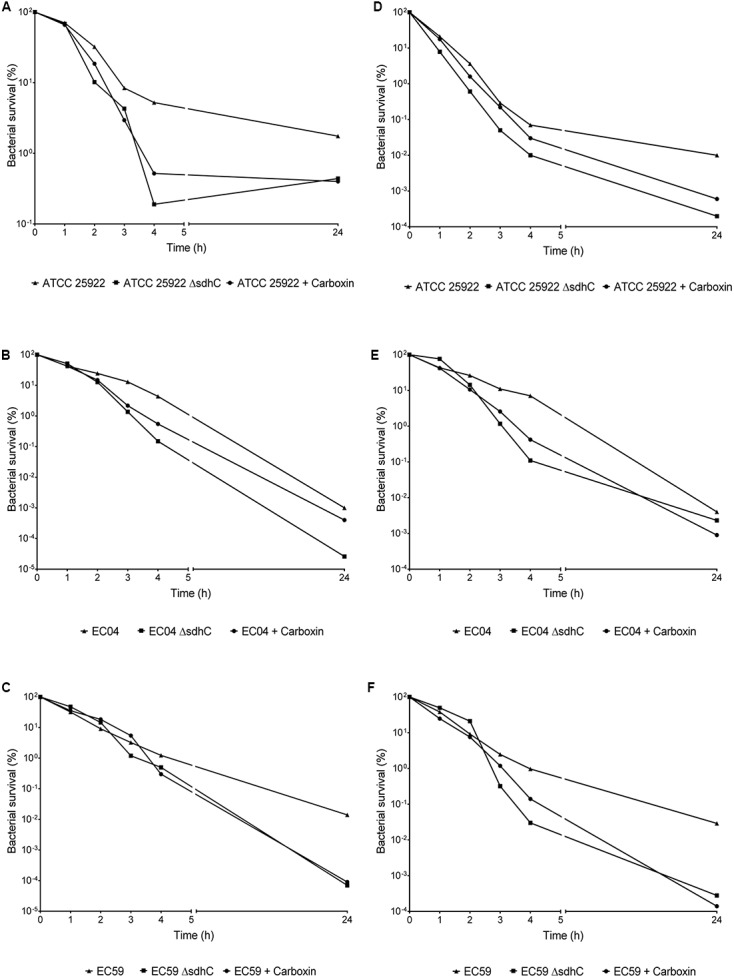
Evaluation of susceptibility to killing by ciprofloxacin **(A–C)**, ofloxacin **(D–F)**, and combination treatments with carboxin (500 μM). Fluoroquinolone concentrations tested were equivalent to 2xMIC values. Data are plotted as mean ± standard deviation.

The transcriptomic data obtained indicated that oxidative metabolism (related to the endogenous production of ROS and detoxification systems) plays an important and differential role in quinolone response in strains with LLQR. We tested the impact of *sdhC* (Succinate dehydrogenase component) and *cyoA* (Cytochrome O oxidase component) deletions on bacterial viability. Inhibiting these two targets has previously been shown to enhance endogenous microbial ROS production and potentiate the antibacterial activity of quinolones in full susceptible wild-type *E. coli* (Brynildsen et al., 2013). Here, we tested its putative role in terms of LLQR phenotype sensitization. In terms of time-kill curves (assayed in MHB at ciprofloxacin and ofloxacin concentrations of 2xMIC), *sdhC* deletion increased sensitivity to fluoroquinolones (both ciprofloxacin and ofloxacin) in wild-type and LLQR strains (**Figure 6**). A marked reduction in viable bacteria counts was observed for ciprofloxacin after a short incubation (*E. coli* ATCC 25922: ΔLog = 1.4, EC04: ΔLog = 1.5, EC59: ΔLog = 0.4; after 4 h) and a long one (*E. coli* ATCC 25922: ΔLog = 0.6, EC04: ΔLog = 1.4, EC59: ΔLog = 2.3; after 24 h). Although it has been suggested that *cyoA* is implicated in the quinolone sensitization process in *E. coli* (Brynildsen et al., 2013), only a minor impact was observed in our study in terms of expression (data not shown).

We also wondered whether chemical inhibition of this validated target (*sdhC*) would increase sensitivity to these bactericidal antimicrobial agents. We treated wild-type and LLQR strains with carboxin, an inhibitor of succinate dehydrogenase (Brynildsen et al., 2013), and measured their susceptibility to ciprofloxacin and ofloxacin, respectively. The addition of carboxin alone had no effect on the growth of wild-type cells (data not shown). However, wild-type and LLQR cells treated with fluoroquinolones and carboxin demonstrated increased sensitivity compared to cells treated with fluoroquinolones alone. For ciprofloxacin, this reduction in viability was observed after a short period of incubation (*E. coli* ATCC 25922: ΔLog = 1.1, EC04: ΔLog = 0.9, EC59: ΔLog = 0.6; after 4 h) and a long one (*E. coli* ATCC 25922: ΔLog = 0.6, EC04: ΔLog = 0.3, EC59: ΔLog = 2.2; after 24 h) (**Figure 6**).

## Discussion

Using a transcriptomic approach, we provided evidence of the differential response at clinically significant concentrations of ciprofloxacin (1 μg/mL, the breakpoint for reduced susceptibility according to CLSI and near to 1/2 of serum Cmax) (Mandell et al., 2010) in *E. coli* strains with LLQR harboring frequent quinolone resistance mechanisms (chromosomal and plasmid-mediated). This reduced susceptibility observed in LLQR phenotypes (directly related to MIC value and independently of the subjacent molecular mechanism) led to modulation of multiple systems contributing to bacterial survival under this therapeutic concentration. Additionally, this approach resulted to be useful to start the study of new potential targets for LLQR strains sensitization by targeting ROS modulation.

Ciprofloxacin caused a major reprogramming of gene expression across the genome: hundreds of genes exhibited upregulation or downregulation (**Figures 1**, **2** and Supplementary Tables 2, 3), supporting previous literature for quinolones and other antimicrobials (Shaw et al., 2003; Peter et al., 2004; Kohanski et al., 2007, 2010; Ferrándiz et al., 2016). Although it has been reported that both specific GyrA substitutions (Asp87Gly), *marR* gene deletion or *qnrS* expression may modify moderately the expression of several genes in bacteria (Seoane and Levy, 1995; Okumura et al., 2011; Kwak et al., 2013; Webber et al., 2013), this aspect had not a relevant impact in the interpretation of our results. Phenotypes with LLQR possessed a marked differential response to ciprofloxacin, and the differences observed were proportional to the MIC values for LLQR strains. One of the processes most affected was related to oxidative metabolism. It has already been shown that the antibacterial activity of antibiotics such as quinolones can be increased by potentiating endogenous microbial ROS production (Wang and Zhao, 2009; Brynildsen et al., 2013). Our results are consistent with these studies and indicate that strains with LLQR would produce lower levels of ROS (Brynildsen et al., 2013; Dwyer et al., 2014). At clinically significant concentrations of ciprofloxacin, the quinolone-induced complex redox alterations downstream of their target-specific interactions that contribute to cellular damage and death would be reduced in strains with LLQR (Erental et al., 2012; Brynildsen et al., 2013; Dorsey-Oresto et al., 2013; Dwyer et al., 2014; Händel et al., 2014, 2016; Méhi et al., 2014; Ferrándiz et al., 2016). In this context, resistance would be the result on interaction at the genetic and gene expression levels (Händel et al., 2014; Freihofer et al., 2016).

In *E. coli*, RecA-LexA coordinates the DNA damage response that allows two opposing responses: life, mediated by the SOS; and death, mediated by the PCD (Baharoglu and Mazel, 2014; Erental et al., 2014). The choice seems to depend on the degree of DNA damage to the cell (and is indirectly a function of MIC values) (Rodríguez-Martínez et al., 2016a). At 1 μg/mL of ciprofloxacin, LLQR strains would be affected by stress caused by moderate DNA damage compared to the stress in wild-type *E. coli* caused by a severe DNA damage. Moderate SOS response in LLQR strains could lead to survival, while massive DNA damage in wild-type *E. coli* could lead to dead population under exacerbated SOS response (Erental et al., 2014). At clinically significant concentrations of ciprofloxacin, the evolution to clinical resistance (ciprofloxacin MIC higher than 2 μg/mL) would be favored in the strain with LLQR, allowing survival and favoring processes of recombination and DNA repair, such as translesion synthesis (Morgan-Linnell and Zechiedrich, 2007; Morgan-Linnell et al., 2009; Baharoglu and Mazel, 2014; Erental et al., 2014). These data would support the higher frequency of mutants observed in LLQR strains (Briales et al., 2011; Machuca et al., 2014).

Furthermore, the expression profile was clearly altered in these LLQR strains, and involved even TA system genes, PCD genes and MMR genes. Interestingly, the *yihE* gene (coding for a key kinase regulator that protects *E. coli* from antimicrobials like quinolones by antagonizing the MazEF TA module) was upregulated in LLQR strains (Dorsey-Oresto et al., 2013), increasing the level of protection against PCD processes. Downregulation of the DNA gyrase genes (both *gyrA* and *gyrB*) also inferred the differential degree of stress in strains with LLQR compared to the wild-type strains (Supplementary Figure 1).

The redox stress component of antibiotic lethality contributes to cell death (usually without MICs values modification) (Wang and Zhao, 2009; Brynildsen et al., 2013). More specifically, antimicrobials like quinolones produce alterations to the central metabolism, cellular respiration and iron metabolism initiated by drug-mediated disruptions of target-specific processes and resulting in cellular damage (Dwyer et al., 2014; Zhao and Drlica, 2014; Lobritz et al., 2015; Zhao et al., 2015). Drug tolerance in pathogenic clinical isolates involves mutations in the oxidative stress response and detoxifying genes (McMurry et al., 1998; Koutsolioutsou et al., 2005; Páez et al., 2010; Thomas et al., 2013). Our transcriptomic data indicated that oxidative metabolism (related both to endogenous ROS modulation and detoxification systems) plays an important differential role in the response of quinolones in LLQR strains. Here we hypothesized that, against quinolones, the deletion or inhibition of the central components of the TCA cycle would lead to sensitization in strains with LLQR similar to that observed in fully susceptible wild-type *E. coli*, as previously described (Brynildsen et al., 2013).

Our results show that inhibiting succinate dehydrogenase (predictable according to transcriptomic comparisons) is sufficient to increase sensitivity to quinolones in LLQR *E. coli*. Although carboxin may not be useful as an antibiotic adjuvant because of its toxicity^10^, the data reinforce the potential usefulness of this strategy.

In summary, the global response to ciprofloxacin is significantly altered in LLQR *E. coli* and affects critical systems for survival and the emergence of antimicrobial resistance at clinically relevant concentrations. This approach validated ROS modulation as an interesting target in bacterial re-sensitization after drug resistance development.

## Author Contributions

J-MR-M and AP designed the study. J-MR-M, JM, ER, AB, and PD-d-A performed transcriptomic assays, further analysis and viability assays. J-MR-M, AP, and JB. contributed ideas and edited the manuscript. All authors read, commented on, and approved the final manuscript.

## Conflict of Interest Statement

The authors declare that the research was conducted in the absence of any commercial or financial relationships that could be construed as a potential conflict of interest.

## Footnotes

1 http://wwwuser.cnb.csic.es/~genomica/servicos.htm

2 http://bioinfogp.cnb.csic.es

3 http://www.ncbi.nlm.nih.gov/geo/

4 http://ecocyc.org/gene?orgid=ECOLI&id=PD00205#tab=REGULON

5 http://ecogene.org/

6 https://genexpdb.ou.edu/main/

7 http://www.genome.jp/kegg-bin/get_htext?htext=ko00002&filedir=%2fkegg%2fbrite%2fko&length=

8 http://linux1.softberry.com/berry.phtml

9 https://david.ncifcrf.gov/gene2gene.jsp

10 https://www.epa.gov/sites/production/files/2015-07/documents/epa-507.pdf
