# Supplementary material for: Cellular Response to Ciprofloxacin in Low-Level Quinolone-Resistant Escherichia coli
Source: Front Microbiol. 2017 Jul 19;8:1370. doi: 10.3389/fmicb.2017.01370 (PMC5516121; doi:10.3389/fmicb.2017.01370)
Supplement: Supplementary file 1 [file Image_1.PDF]

Supplementary Figure 1. Impact on other bacterial survival systems of low-level quinolone resistance (LLQR) cells exposed to 1 mg/L of ciprofloxacin and compared to wild-type cells in the same conditions. LLQR phenotypes: EC14 means *E. coli* ATCC 25922 pBK-QnrS1; EC19 means *E. coli* ATCC 25922 ΔmarR pBK-QnrS1; and EC24 means *E. coli* ATCC 25922 S83L pBK-QnrS1. * means a significantly different pattern of expression between LLQR strains and wild-type *E. coli* (*p* value <0.05).

(A)

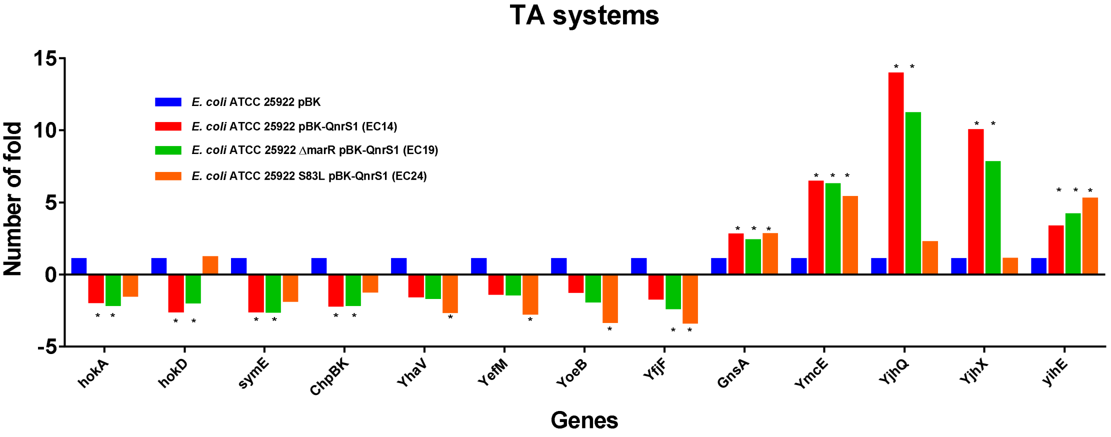

(B)

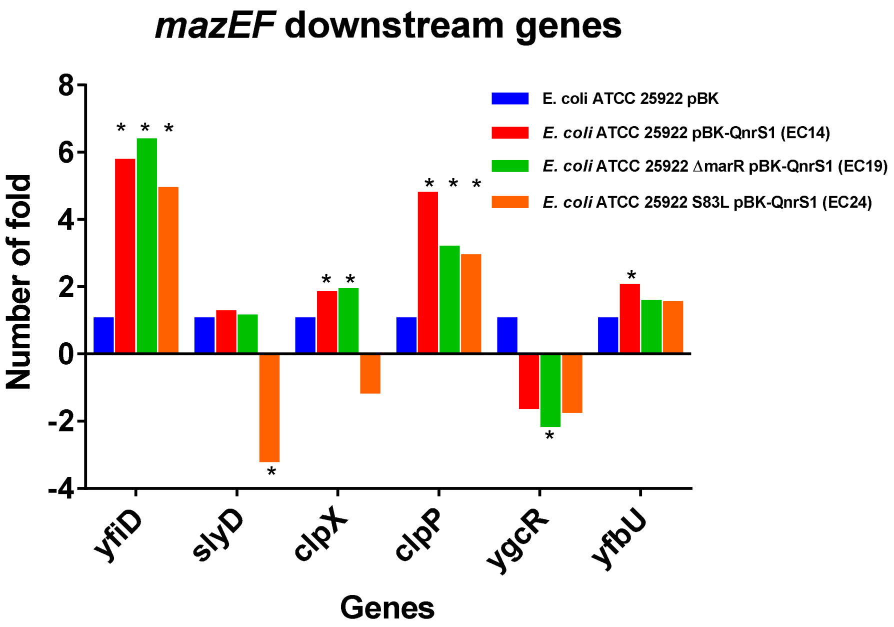

(C)

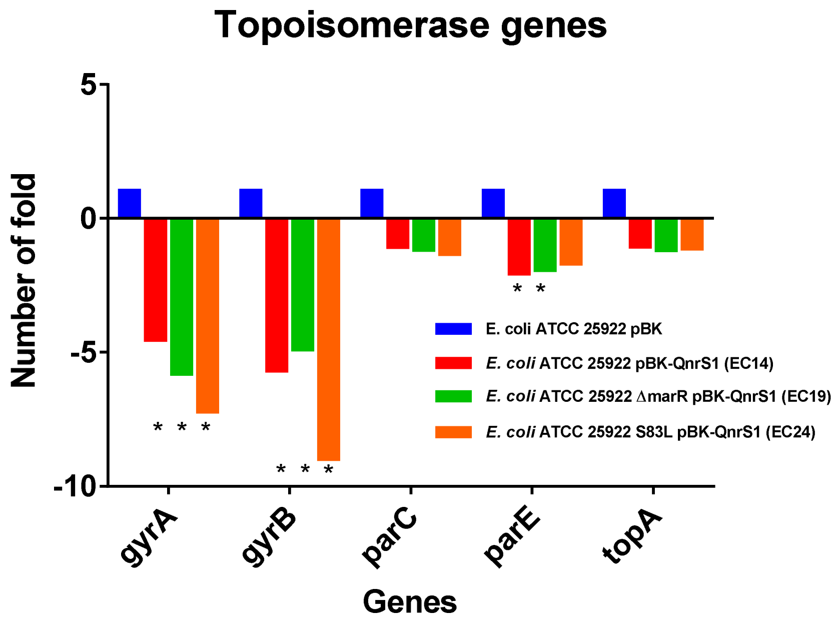
Topoisomerase genes
5
0
-5
-10
Number of fold
E. coli ATCC 25922 pBK
E. coli ATCC 25922 pBK-QnrS1 (EC14)
E. coli ATCC 25922 ΔmarR pBK-QnrS1 (EC19)
E. coli ATCC 25922 S83L pBK-QnrS1 (EC24)
gyrA
gyrB
parC
parE
topA
Genes

(D)

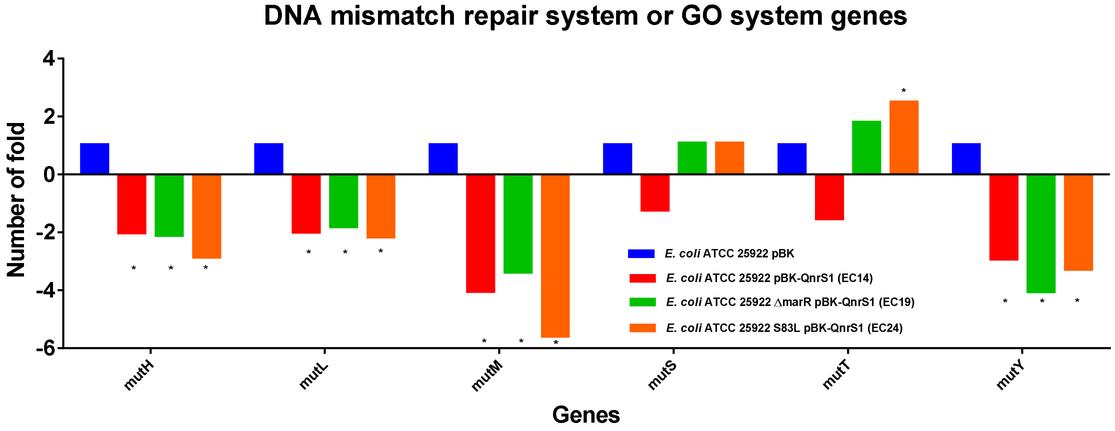
DNA mismatch repair system or GO system genes
4
2
0
-2
-4
-6
Number of fold
E. coli ATCC 25922 pBK
E. coli ATCC 25922 pBK-QnrS1 (EC14)
E. coli ATCC 25922 ΔmarR pBK-QnrS1 (EC19)
E. coli ATCC 25922 S83L pBK-QnrS1 (EC24)
mutH
mutL
mutM
mutS
mutT
mutY
Genes
