# Supplementary material for: Cellular Response to Ciprofloxacin in Low-Level Quinolone-Resistant Escherichia coli
Source: Front Microbiol. 2017 Jul 19;8:1370. doi: 10.3389/fmicb.2017.01370 (PMC5516121; doi:10.3389/fmicb.2017.01370)
Supplement: Supplementary file 2 [file Table_1.DOCX]

**Supplementary Table 1.** Oligonucleotides and vectors used in this study.

| **Primer** | **Sequence** | **Size (bp)** | **Gene (Function)** | **RT-PCR result^a^** | **Source or reference** |
| --- | --- | --- | --- | --- | --- |
| **RT-PCR validation** | |  |  |  |  |
| sdhC-RTF | 5´- CTTTGTTGCAGTGGGCATCC -3´ | 142 | *sdhC* (Succinate dehydrogenase cytochrome b556) | +142.6 | This study |
| sdhC-RTR | 5´- GTGATACGCCAGAGCGGTAA -3´ |  |  |  | This study |
| sucD-RTF | 5´- GCTGGATATGCTGACCGTGA-3´ | 166 | *sucD* (Succinyl CoA synthase alpha-subunit) | +130.2 | This study |
| sucD-RTR | 5´- CAGTGTACCGGAACGGGAAA-3´ |  |  |  | This study |
| nuoH-RTF | 5´- GATCCTGCTGGTGGTTGTCA-3´ | 197 | *nuoH* (NADH:ubiquinone oxidoreductase subunit H) | +233.8 | This study |
| nuoH-RTR | 5´- CCAGGGTAAAGATGACGCGA-3´ |  |  |  | This study |
| recA-RTF | 5´- TTCCGGTAAAACCACGCTGA -3´ | 189 | *recA* (Multifunctional  DNA recombination  and repair protein) | -52.6 | This study |
| recA-RTR | 5´- AGGGCGTCACAGATTTCCAG -3´ |  |  |  | This study |
| focA-RTF | 5´- GGGGTCAGTTGGCGAAAAAC -3´ | 184 | *focA* (Formate channel) | +7.4 | This study |
| focA-RTR | 5´- CCAAGACAGACGGCCTCAAT -3´ |  |  |  | This study |
| tnaA-RTF | 5´- CGCCAAGAAAGATGCGATGG -3´ | 173 | *tnaA* (Tryptophanase activity) | +180.5 | This study |
| tnaA-RTR | 5´- CGTCATACAGACCTACCGCC -3´ |  |  |  | This study |
| **Inactivation of chromosomal genes** | |  |  |  |  |
| H1-sdhC-P1 | 5’-GCCCGTAGTCCCCAGGGAATAATAAGAACAGCATGTGGGCGTTATTCATGATAAGAAATGTGAAAAAACA-GTGTAGGCTGGAGCTGCTTC -3’ |  | *sdhC* chromosomal inactivation |  | This study |
| H2-sdhC-P2 | 5’-GCCATTGCGTCCTAATGCGGAGGCGTTGCTTACCATACGAGGACTCCTGCGAGAAGTGAAAGCACGACAG-ATGGGAATTAGCCATGGTCC -3’ |  | *sdhC* chromosomal inactivation |  | This study |
| Pre-H1-sdhC | 5’-AGCAGACCGGAGGAAGGAAATCC -3’ |  | *sdhC* chromosomal inactivation |  | This study |
| Pre-H2-sdhC | 5’- GGTAGCGCGAACGAGGATGAAAT -3’ |  | *sdhC* chromosomal inactivation |  | This study |
| H1-cyoA-P1 | 5’-CCACACACTTTAAACGCCACCAGATCCCGTGGAATTGAGGTCGTTAAATGAGACTCAGGAAATACAATAA-GTGTAGGCTGGAGCTGCTTC -3’ |  | *cyoA* chromosomal inactivation |  | This study |
| H2-cyoA-P2 | 5’-CCGAACATCTTTATTCTTCCTCAACCCCTTTAATGGGCGGATTCCGCGTGGCTCATGTCCATGCCTTCCA-ATGGGAATTAGCCATGGTCC -3’ |  | *cyoA* chromosomal inactivation |  | This study |
| Pre-H1-cyoA | 5’- GACCTGGCAGCCAAATCCAAGTA -3’ |  | *cyoA* chromosomal inactivation |  | This study |
| Pre-H2-cyoA | 5’- AGGCGTTTATGGTCGACGGAGGT -3’ |  | *cyoA* chromosomal inactivation |  | This study |
| K1 | 5´- CAGTCATAGCCGAATAGCCT-3´ |  | Chromosomal inactivation |  | 14 |
| K2 | 5´- CGGTGCCCTGAATGAACTGC-3´ |  | Chromosomal inactivation |  | 14 |
| **Plasmids** |  |  |  |  | 14 |
| pBK-CMV |  |  | Cloning vector |  | Stratagene |
| pKD4 |  |  | Chromosomal inactivation |  | 14 |
| pKOBEG |  |  | Chromosomal inactivation/ helper vector |  | 14 |
| pCP20 |  |  | Chromosomal inactivation/ resolution vector |  | 14 |

^a^Shown RT-PCR results correspond to the relative expression of EC24 compared to *E. coli* ATCC 25922
