# Supplementary material for: Cellular Response to Ciprofloxacin in Low-Level Quinolone-Resistant Escherichia coli
Source: Front Microbiol. 2017 Jul 19;8:1370. doi: 10.3389/fmicb.2017.01370 (PMC5516121; doi:10.3389/fmicb.2017.01370)
Supplement: Supplementary file 3 [file Table_2.DOC]

**Supplementary Table 2**. Groups of genes analyzed in the transcriptomic response of strains with LLQR (compared to *E. coli* ATCC 25922) after 1 hour of exposure to 1 mg/L of ciprofloxacin.

| **Role** | **Protein or Function** | **EC14** | **EC19** | **EC24** |
| --- | --- | --- | --- | --- |
| **Oxidative metabolism** |  |  |  |  |
|  |  |  |  |  |
| *cyoA* | Cytochrome O oxidase | **1.8** | **2.1** | **2.9** |
| *cyoB* |  | **2.9** | **4.9** | **14.8** |
| *cyoC* |  | **2.7** | **3.9** | **19.4** |
| *cyoD* |  | **3.9** | **4.3** | **12.9** |
| *cyoE* |  | **2.9** | **4.5** | **7.1** |
| *nuoA* | NADH:ubiquinone oxidoreductase | **1.5** | 1.5 | **2.6** |
| *nuoB* |  | 1.4 | **1.7** | **2.5** |
| *nuoC* |  | **6.2** | **7.7** | **34.6** |
| *nuoD* |  |  |  |  |
| *nuoE* |  | **6.9** | **9.2** | **31.3** |
| *nuoF* |  | **16.1** | **26.6** | **72.9** |
| *nuoG* |  | **8.8** | **12.4** | **54.1** |
| *nuoH* |  | **18.8** | **39.1** | **107.9** |
| *nuoI* |  | **7.2** | **10.1** | **37.7** |
| *nuoJ* |  | **7.9** | **15.0** | **47.2** |
| *nuoK* |  | **9.9** | **22.5** | **55.1** |
| *nuoL* |  | **6.3** | **12.7** | **26.3** |
| *nuoM* |  | **6.6** | **11.3** | **35.4** |
| *nuoN* |  | **3.3** | **6.3** | **14.2** |
| *sdhA* | Succinate dehydrogenase | **10.9** | **11.5** | **19.5** |
| *sdhB* |  | **6.1** | **7.0** | **11.5** |
| *sdhC* |  | **37.9** | **49.6** | **63.5** |
| *sdhD* |  | **22.2** | **27.4** | **48.2** |
| *atpA* | ATP synthase | **3.1** | **4.4** | **6.4** |
| *atpB* |  | **2.2** | **2.3** | -1.0 |
| *atpC* |  | 1.4 | 1.2 | **2.6** |
| *atpD* |  | **3.2** | **4.4** | **5.5** |
| *atpE* |  | **4.4** | **5.0** | **4.9** |
| *atpF* |  | **1.7** | **1.8** | 1.5 |
| *atpG* |  | **3.8** | **5.7** | **4.4** |
| *atpH* |  | **7.9** | **9.0** | **8.5** |
| *atpI* |  | -1.5 | **-1.6** | **-2.0** |
| *sucA* | 2-oxoglutarate dehydrogenase | **91.7** | **116.8** | **465.0** |
| *sucB* |  | **45.7** | **84.2** | **190.5** |
| *sucC* | Succinyl CoA synthase | **55.8** | **94.5** | **215.9** |
| *sucD* |  | **36.2** | **54.9** | **112.2** |
| *pfkB* | 6-phosphofructokinase | **3.7** | **6.0** | **6.0** |
| *fumA* | Fumarases | **14.1** | **22.5** | **18.2** |
| *fumB* |  | **3.1** | **3.6** | **2.1** |
| *fumC* |  | **8.1** | **12.5** | **19.9** |
| *gnd* | 6-phosphogluconate dehydrogenase | **2.8** | **5.5** | **1.9** |
| *pta* | Phosphotransacetylase | **8.5** | **13.7** | **17.4** |
| *zwf* | Glucose-6-phosphate 1-dehydrogenase | **1.9** | **2.8** | **2.3** |
|  |  |  |  |  |
| **Detoxification systems** |  |  |  |  |
|  |  |  |  |  |
| *soxS* | Global transcription regulator for superoxide response | **2.6** | 1.8 | 1.5 |
| *soxR* | Redox-sensitive transcriptional activator for soxS | **-2.3** | **-2.7** | **-3.7** |
| *oxyS* | OxyS sRNA activates genes that detoxify oxidative damage |  |  |  |
| *oxyR* | Oxidative and nitrosative stress transcriptional regulator | **-2.2** | **-1.6** | **-1.6** |
| *dps* | Stress-induced Fe-binding | **4.2** | **5.1** | **5.6** |
| *ahpC* | Alkyl hydroperoxide reductase | **2.5** | **4.2** | **5.8** |
| *ahpF* |  | **-1.9** | -1.4 | **-1.8** |
| *sodA* | Superoxide dismutases | **2.5** | **2.2** | **2.1** |
| *sodB* |  | **6.6** | **7.7** | **12.3** |
| *sodC* |  | **1.9** | **2.1** | **2.7** |
| *katE* | Catalases | -1.09 | 1.7 | 1.6 |
| *katG* |  | **3.5** | **7.7** | **12.4** |
| *katP* |  | -1.1 | -1.5 | -1.3 |
|  |  |  |  |  |
| **SOS system** |  |  |  |  |
|  |  |  |  |  |
| *cho* | Endonuclease involved in excision repair | **-1.9** | **-1.6** | -1.6 |
| *dinB* | DNA polymerase IV | **-3.4** | **-3.0** | **-5.5** |
| *dinD* | DNA-damage-inducible protein | -1.2 | 1.2 | 1.4 |
| *dinF* | Oxidative stress resistance protein | **-3,1** | **-3.2** | **-2.5** |
| *dinG* | DNA helicase | **4.4** | **6.3** | **7.2** |
| *dinI* | Stabilizes RecA filaments | **-2.3** | **-2.0** | **-15.4** |
| *dinJ* | Antitoxin of TA component | 1.1 | -1.4 | 1.2 |
| *dinQ* | UV-inducible membrane toxin |  |  |  |
| *ftsI* | Transpeptidase PBP3 | **-1.9** | -1.8 | **-1.9** |
| *ftsK* | DNA translocase | 1.1 | **1.9** | **3.3** |
| *ftsZ* | Septal ring GTPase | 1.2 | 1.1 | **2.9** |
| *hokE* | Small toxic membrane polypeptide | -1.1 | -1.1 | -1.9 |
| *lexA* | Global regulator (repressor) for SOS regulon | 1.0 | 1.2 | **-1.9** |
| *phr* | DNA photolyase | -1.3 | -1.4 | 1.0 |
| *polB* | DNA polymerase II | **-5.2** | **-4.7** | **-4.3** |
| *ptrA* | Protease III | **-1.4** | -1.6 | 1.3 |
| *recA* | Multifunctional DNA recombination and repair protein/SOS activation | **-3.3** | **-4.1** | **-30.7** |
| *recB* | RecBCD Exonuclease | **-1.5** | **-1.9** | 1.1 |
| *recC* |  | -1.2 | -1.1 | -1.4 |
| *recN* | Recombination and repair | **-3.0** | **-1.9** | **-2.9** |
| *recX* | Blocks RecA filament extension; inhibitor of RecA ATPase | **-4.6** | **-8.2** | **-23.4** |
| *rpsU* | Ribosomal protein | **-1.6** | **-1.9** | **-2.9** |
| *ruvA* | Holliday junction recognition factor | **-1.5** | **-2.0** | **-4.7** |
| *ssb* | Single-strand DNA-binding protein | **-3.5** | **-3.4** | **-8.2** |
| *sulA* | Inhibits cell division and ftsZ ring formation | **2.6** | **3.7** | -1.0 |
| *umuC* | Translesion DNA polymerase | **-2.6** | **-3.5** | **-7.9** |
| *umuD* |  | **-1.4** | **-1.7** | **-5.7** |
| *uvrA* | Excision nuclease | **-5.9** | **-5.5** | **-11.1** |
| *uvrB* |  | **-8.7** | **-10.6** | **-20.4** |
| *uvrC* |  | **-1.7** | -1.1 | 1.1 |
| *uvrD* | DNA helicase II | **-1.8** | -1.5 | **-2.3** |
| *yafN* | Antitoxin | 1.0 | 1.0 | 1.1 |
| *ybfE* | CopB family protein | **-1.9** | **-3.3** | **-5.6** |
| *yebG* | DNA damage-inducible gene | **-2.0** | -1.4 | **-7.9** |
|  |  |  |  |  |
| **TA systems** |  |  |  |  |
|  |  |  |  |  |
| *hokA* | Type I toxin–antitoxin systems | **-1.8** | **-2.0** | -1.3 |
| *hokD* |  | **-2.5** | **-1.9** | 1.1 |
| *symE* |  | **-2.5** | **-2.5** | -1.7 |
| *ChpBK* | Type II toxin–antitoxin systems | **-2.1** | **-2.0** | -1.1 |
| *YhaV* |  | -1.4 | -1.5 | **-2.5** |
| *YefM* |  | -1.3 | -1.3 | **-2.6** |
| *YoeB* |  | -1.1 | -1.8 | **-3.2** |
| *YfjF* |  | -1.6 | **-2.2** | **-3.3** |
| *GnsA* |  | **2.7** | **2.3** | **2.7** |
| *YmcE* |  | **6.4** | **6.2** | **5.3** |
| *YjhQ* |  | **13.9** | **11.1** | **2.2** |
| *YjhX* |  | **9.9** | **7.7** | 1.0 |
| *yihE* |  | **3.3** | **4.1** | **5.2** |
|  |  |  |  |  |
| ***mazEF* pathway** |  |  |  |  |
|  |  |  |  |  |
| *yfiD* | required for MazF-mediated cell death after DNA damage | **5.7** | **6.3** | **4.9** |
| *slyD* |  | 1.2 | 1.1 | **-3.1** |
| *clpX* |  | **1.8** | **1.9** | -1.1 |
| *clpP* |  | **4.7** | **3.1** | **2.9** |
| *ygcR* |  | -1.5 | **-2.1** | -1.6 |
| *yfbU* |  | **2.0** | 1.5 | 1.4 |
|  |  |  |  |  |
| **Topoisomerases** |  |  |  |  |
|  |  |  |  |  |
| *gyrA* | Type II topoisomerase (DNA gyrase) | **-4.5** | **-5.8** | **-7.2** |
| *gyrB* |  | **-5.6** | **-4.9** | **-8.9** |
| *parC* | Type II topoisomerase (Topoisomerase IV) | -1.0 | -1.1 | -1.3 |
| *parE* |  | **-2.0** | **-1.9** | -1.6 |
| *topA* | Topoisomerase I | -1.0 | -1.1 | -1.1 |
|  |  |  |  |  |
| **DNA mismatch repair system** |  |  |  |  |
|  |  |  |  |  |
| *mutH* | DNA repair | **-2.0** | **-2.1** | **-2.8** |
| *mutL* |  | **-1.9** | **-1.8** | **-2.1** |
| *mutM* |  | **-4.0** | **-3.4** | **-5.6** |
| *mutS* |  | -1.2 | 1.1 | 1.1 |
| *mutT* |  | -1.5 | 1.8 | **2.5** |
| *mutY* |  | **-2.9** | **-4.0** | **-3.3** |
|  |  |  |  |  |

Protein definition or functions were performed mainly with EcoGene 3.0 (<http://ecogene.org/>)

Significant differences (p value <0.05) are indicated with **underlined bold** numbers.

EC14 means *E. coli* ATCC 25922 pBK-QnrS1

EC19 means *E. coli* ATCC 25922 Δ*marR* pBK-QnrS1

EC24 means *E. coli* ATCC 25922 S83L pBK-QnrS1
