# Supplementary material for: Cellular Response to Ciprofloxacin in Low-Level Quinolone-Resistant Escherichia coli
Source: Front Microbiol. 2017 Jul 19;8:1370. doi: 10.3389/fmicb.2017.01370 (PMC5516121; doi:10.3389/fmicb.2017.01370)
Supplement: Supplementary file 4 [file Table_3.pdf]

Supplementary Table 3. Global data for transcriptomic assays

E. coli ATCC 25922 pBK-QnrS1 (EC14) versus *E. coli* ATCC 25922 pBK-CMV

Significant differential expression (greater than or equal to two-fold, P value <0,05)

Gene Name (ProbeID)	Fold Change	logSignal (A)	logRatio (M)	logControl	StdErr (logControl)	logExperiment	StdErr (logExperiment)	pval (limma)	FDR (limma)	Oligo	Systematic Name	Description
aaeA	-16.08	9.83	-4.01	11.83	0.1	7.82	0.1	0.00000220	0.00026540	A_07_P012680	b3241	cmr|putative membrane protein [b3241]
aaeB	-4.94	8.03	-2.31	9.18	0.1	6.88	0.0	0.00003059	0.00078194	A_07_P012673	b3240	cmr|orf, hypothetical protein [b3240]
aaeX	-4.10	8.13	-2.04	9.15	0.0	7.11	0.1	0.00004379	0.00094590	A_07_P010572	b3242	cmr|orf, hypothetical protein [b3242]
ABE-0285085	-3.42	8.51	-1.77	9.40	0.1	7.63	0.2	0.00033640	0.00367028	A_07_P010678	ABE-0285085	asap|IS1 transposase InsAB', C-ter fragment (pseudogene) [b4564]
ABH-0247453	-25.66	11.59	-4.68	13.94	0.0	9.25	0.1	0.00000074	0.00019431	A_07_P048654	ABH-0247453	asap|predicted protein []
ABR-0081747	-4.30	12.62	-2.10	13.67	0.0	11.57	0.0	0.00003172	0.00079523	A_07_P035421	ABR-0081747	asap|toxic membrane protein []
ABR-0081751	-3.96	8.80	-1.99	9.79	0.1	7.81	0.1	0.00010635	0.00164315	A_07_P041043	ABR-0081751	asap|predicted ferredoxin []
ABR-0081753	+6.78	10.99	+2.76	9.61	0.1	12.37	0.0	0.00001117	0.00048574	A_07_P041110	ABR-0081753	asap|predicted protein []
ABR-0081754	+2.65	7.54	+1.41	6.84	0.4	8.24	0.0	0.00577464	0.02947988	A_07_P041114	ABR-0081754	asap|predicted protein []
ACA-0244210	-3.16	11.50	-1.66	12.33	0.1	10.67	0.4	0.00246207	0.01543104	A_07_P059088	ACA-0244210	asap|predicted protein []
ACA-0285117	-2.79	11.88	-1.48	12.62	0.2	11.14	0.2	0.00104963	0.00821752	A_07_P062850	ACA-0285117	asap|toxic polypeptide, small []
accB	+4.40	10.79	+2.14	9.72	0.1	11.85	0.0	0.00003995	0.00090512	A_07_P012745	b3255	cmr|acetylCoA carboxylase, BCCP subunit; carrier of biotin [b3255]
accC	+2.00	10.52	+1.00	10.02	0.1	11.02	0.0	0.00190573	0.01273506	A_07_P012747	b3256	cmr|acetyl CoA carboxylase, biotin carboxylase subunit [b3256]
aceA	+6.63	9.18	+2.73	7.82	0.0	10.54	0.0	0.00000871	0.00042757	A_07_P008720	b4015	cmr|isocitrate lyase [b4015]
aceA	+6.26	8.97	+2.65	7.64	0.3	10.29	0.1	0.00008519	0.00142692	A_07_P037462	c_4972	cmr|Isocitrate lyase [c_4972]
aceB	+23.04	9.49	+4.53	7.23	0.0	11.76	0.0	0.00000071	0.00019431	A_07_P008715	b4014	cmr|malate synthase A [b4014]
aceE	+27.60	11.24	+4.79	8.85	0.0	13.64	0.1	0.00000063	0.00019431	A_07_P002535	b0114	cmr|pyruvate dehydrogenase [b0114]
aceF	+7.87	11.69	+2.98	10.20	0.1	13.18	0.0	0.00000907	0.00043556	A_07_P002536	b0115	cmr|pyruvate dehydrogenase [b0115]
ackA	+4.66	12.75	+2.22	11.64	0.1	13.87	0.0	0.00002945	0.00076000	A_07_P013766	b2296	cmr|acetate kinase [b2296]
acnA	+2.72	9.01	+1.44	8.29	0.1	9.74	0.1	0.00034781	0.00375837	A_07_P014361	b1276	cmr|aconitate hydrase 1 [b1276]
acnB	+7.16	10.24	+2.84	8.82	0.0	11.66	0.1	0.00000809	0.00041095	A_07_P002554	b0118	cmr|aconitate hydrase B [b0118]
acpD	+3.43	8.65	+1.78	7.76	0.1	9.53	0.1	0.00012727	0.00183667	A_07_P052470	Z2315	cmr|acyl carrier protein phosphodiesterase [Z2315]
acpD	+3.03	9.19	+1.60	8.39	0.2	9.99	0.0	0.00029674	0.00335322	A_07_P063175	ECs2014	cmr|acyl carrier protein phosphodiesterase [ECs2014]
acpP	+2.17	16.08	+1.12	15.52	0.0	16.64	0.1	0.00115284	0.00883229	A_07_P001671	b1094	cmr|acyl carrier protein [b1094]
acpS	+2.59	8.37	+1.37	7.68	0.1	9.06	0.0	0.00043162	0.00435473	A_07_P019720	b2563	cmr|CoA:apo- [b2563]
acpS	+2.98	8.44	+1.58	7.65	0.0	9.23	0.1	0.00015331	0.00211360	A_07_P053485	ECs3429	cmr|CoA:apo- [ECs3429]
acpS	+2.08	8.36	+1.05	7.83	0.1	8.89	0.0	0.00139171	0.01006017	A_07_P042915	Z3844	cmr|CoA:apo- [Z3844]
acs	+10.15	8.58	+3.34	6.91	0.1	10.26	0.1	0.00000660	0.00038136	A_07_P052329	Z5668	cmr|acetyl-CoA synthetase [Z5668]
acs	+6.57	8.49	+2.72	7.13	0.2	9.85	0.2	0.00009019	0.00146989	A_07_P008986	b4069	cmr|acetyl-CoA synthetase [b4069]
actP	+2.18	7.66	+1.12	7.10	0.2	8.22	0.1	0.00370526	0.02100672	A_07_P063696	ECs5049	cmr|putative transport protein [ECs5049]
actP	+2.68	7.56	+1.42	6.85	0.1	8.28	0.0	0.00042444	0.00430129	A_07_P052321	Z5666	cmr|putative transport protein [Z5666]
ade	+2.52	8.18	+1.33	7.52	0.0	8.85	0.1	0.00043204	0.00435473	A_07_P017261	b3665	cmr|probable adenine deaminase [b3665]
adhE	+2.03	8.13	+1.02	7.62	0.1	8.64	0.0	0.00130811	0.00968257	A_07_P007143	b1241	cmr|CoA-linked acetaldehyde dehydrogenase and iron-dependent alcohol dehydrogenase; pyruvate-formate-lyase deactivase [b1241]
aefA	-2.69	8.75	-1.43	9.47	0.0	8.04	0.0	0.00020331	0.00257851	A_07_P031187	c_0584	cmr|Potassium efflux system kefA [c_0584]
aes	-19.95	10.61	-4.32	12.77	0.1	8.45	0.0	0.00000141	0.00022371	A_07_P021486	b0476	cmr|putative lipase [b0476]
agaB	+2.11	7.34	+1.08	6.80	0.0	7.88	0.0	0.00083427	0.00700522	A_07_P000415	b3138	cmr|PTS system, cytoplasmic, N-acetylgalactosamine-specific IIB component 1 [b3138]
agaS	+13.70	8.73	+3.78	6.84	0.1	10.62	0.0	0.00000308	0.00028863	A_07_P034110	c_3893	cmr|Putative tagatose-6-phosphate ketose/aldose isomerase [c_3893]
agaS	+9.24	8.61	+3.21	7.01	0.1	10.22	0.0	0.00000492	0.00034758	A_07_P000404	b3136	cmr|putative tagatose-6-phosphate aldose/ketose isomerase [b3136]
agaV	+9.38	8.11	+3.23	6.50	0.1	9.73	0.1	0.00000531	0.00034950	A_07_P000390	b3133	cmr|PTS system, cytoplasmic, N-acetylgalactosamine-specific IIB component 2 [b3133]
agaW	+4.73	8.64	+2.24	7.52	0.3	9.76	0.2	0.00025275	0.00301002	A_07_P000391	b3134	cmr|PTS system N-acetylgalactosameine-specific IIC component 2 [b3134]
agp	+2.27	9.54	+1.18	8.95	0.3	10.13	0.1	0.00317787	0.01874573	A_07_P033935	c_1137	cmr|Glucose-1-phosphatase precursor [c_1137]
agp	+2.62	9.40	+1.39	8.71	0.1	10.10	0.2	0.00089902	0.00739123	A_07_P001241	b1002	cmr|periplasmic glucose-1-phosphatase [b1002]
ahpC	+2.50	15.39	+1.32	14.73	0.1	16.05	0.1	0.00039188	0.00405638	A_07_P016781	b0605	cmr|alkyl hydroperoxide reductase, C22 subunit; detoxification of hydroperoxides [b0605]
aidB	-2.92	9.26	-1.55	10.03	0.3	8.48	0.0	0.00192466	0.01280417	A_07_P011655	b4187	cmr|putative acyl coenzyme A dehydrogenase [b4187]
aldA	+3.12	8.28	+1.64	7.46	0.1	9.10	0.2	0.00033709	0.00367526	A_07_P009166	b1415	cmr|aldehyde dehydrogenase, NAD-linked [b1415]
allA	+2.76	8.86	+1.47	8.12	0.5	9.59	0.1	0.00695603	0.03382203	A_07_P016314	b0505	cmr|orf, hypothetical protein [b0505]
allB	+3.22	9.19	+1.69	8.34	0.2	10.03	0.1	0.00034946	0.00376840	A_07_P016349	b0512	cmr|putative hydrolase [b0512]
allD	+4.77	8.12	+2.25	6.99	0.0	9.24	0.2	0.00005402	0.00107118	A_07_P016372	b0517	cmr|putative malate dehydrogenase [b0517]
alr	-2.53	10.01	-1.34	10.68	0.0	9.34	0.1	0.00044888	0.00448424	A_07_P008909	b4053	cmr|alanine racemase 1 [b4053]
alsB	+9.06	9.29	+3.18	7.70	0.1	10.88	0.0	0.00000426	0.00033352	A_07_P011177	b4088	cmr|putative LACI-type transcriptional regulator [b4088]
amiA	-6.34	12.23	-2.66	13.56	0.0	10.90	0.1	0.00001089	0.00047910	A_07_P042594	c_2969	cmr|Probable N-acetylmuramoyl-L-alanine amidase amiA precursor [c_2969]
amiA	-6.35	12.20	-2.67	13.53	0.0	10.86	0.0	0.00001047	0.00047118	A_07_P052703	Z3700	cmr|N-acetylmuramoyl-l-alanine amidase I [Z3700]
amiA	-5.66	11.72	-2.50	12.97	0.0	10.47	0.0	0.00001332	0.00052521	A_07_P013234	b2435	cmr|N-acetylmuramoyl-l-alanine amidase I [b2435]
amiB	-2.54	10.66	-1.34	11.33	0.1	9.98	0.0	0.00030309	0.00339633	A_07_P011563	b4169	cmr|N-acetylmuramoyl-l-alanine amidase II; a murein hydrolase [b4169]
amn	+6.05	9.03	+2.60	7.73	0.4	10.33	0.0	0.00027270	0.00315462	A_07_P015257	b1982	cmr|AMP nucleosidase [b1982]
ampD	+2.85	12.12	+1.51	11.36	0.1	12.87	0.0	0.00019589	0.00250745	A_07_P041423	c_0129	cmr|AmpD protein [c_0129]
ampD	+3.65	10.90	+1.87	9.97	0.0	11.84	0.2	0.00016366	0.00221361	A_07_P002511	b0110	cmr|regulates ampC [b0110]
ampE	+2.06	9.14	+1.04	8.62	0.0	9.66	0.1	0.00121441	0.00913944	A_07_P002518	b0111	cmr|regulates ampC [b0111]
amyA	-2.57	8.04	-1.36	8.72	0.2	7.36	0.0	0.00106954	0.00833602	A_07_P044659	Z3017	cmr|cytoplasmic alpha-amylase [Z3017]
anmK	-2.02	9.52	-1.01	10.02	0.1	9.01	0.1	0.00200074	0.01316324	A_07_P020831	b1640	cmr|orf, hypothetical protein [b1640]
ansB	+15.30	12.19	+3.94	10.22	0.1	14.15	0.0	0.00000152	0.00022581	A_07_P011926	b2957	cmr|periplasmic L-asparaginase II [b2957]
apbA	+2.67	9.54	+1.41	8.84	0.1	10.25	0.0	0.00028897	0.00328960	A_07_P031138	c_0536	cmr|2-dehydropantoate 2-reductase [c_0536]
aphA	+2.22	8.25	+1.15	7.67	0.2	8.82	0.1	0.00259418	0.01604388	A_07_P008919	b4055	cmr|diadenosine tetraphosphatase [b4055]
apt	+7.43	12.30	+2.89	10.85	0.0	13.74	0.1	0.00000681	0.00038477	A_07_P021451	b0469	cmr|adenine phosphoribosyltransferase [b0469]
araA	+4.53	8.35	+2.18	7.26	0.1	9.44	0.0	0.00003715	0.00087028	A_07_P010999	b0062	cmr|L-arabinose isomerase [b0062]
araB	+2.83	8.05	+1.50	7.29	0.0	8.80	0.2	0.00078245	0.00665883	A_07_P011001	b0063	cmr|L-ribulokinase [b0063]
araC	+2.15	11.06	+1.10	10.50	0.0	11.61	0.0	0.00073874	0.00636601	A_07_P011010	b0064	cmr|transcriptional regulator for ara operon [b0064]
argE	+3.12	8.62	+1.64	7.80	0.2	9.44	0.1	0.00048189	0.00470068	A_07_P039521	c_4916	cmr|Acetylornithine deacetylase [c_4916]
argE	+3.25	7.85	+1.70	6.99	0.2	8.70	0.1	0.00032654	0.00359518	A_07_P008486	b3957	cmr|acetylornithine deacetylase [b3957]
argG	+2.09	7.96	+1.06	7.42	0.2	8.49	0.0	0.00254197	0.01579499	A_07_P000576	b3172	cmr|argininosuccinate synthetase [b3172]
argG	+2.06	8.57	+1.05	8.04	0.1	9.09	0.1	0.00181163	0.01228992	A_07_P041781	c_3929	cmr|Argininosuccinate synthase [c_3929]
argO	-2.03	7.90	-1.02	8.41	0.0	7.39	0.2	0.00362200	0.02069114	A_07_P011764	b2923	cmr|orf, hypothetical protein [b2923]
argR	-2.36	11.47	-1.24	12.08	0.0	10.85	0.0	0.00044476	0.00444871	A_07_P012656	b3237	cmr|repressor of arg regulon; cer-mediated site specific recombination [b3237]
arnT	+2.02	8.18	+1.01	7.67	0.2	8.69	0.1	0.00388747	0.02182751	A_07_P001072	b2257	cmr|orf, hypothetical protein [b2257]
aroD	+2.05	7.38	+1.03	6.86	0.1	7.89	0.1	0.00191791	0.01278929	A_07_P018517	b1693	cmr|3-dehydroquinate dehydratase [b1693]
aroL	-2.45	9.99	-1.29	10.63	0.1	9.34	0.0	0.00049035	0.00476059	A_07_P021052	b0388	cmr|shikimate kinase II [b0388]
aroM	-2.39	7.93	-1.26	8.56	0.0	7.30	0.1	0.00050690	0.00488112	A_07_P031086	c_0498	cmr|AroM protein [c_0498]
aroM	-2.77	7.82	-1.47	8.56	0.0	7.09	0.1	0.00021620	0.00268655	A_07_P043806	Z0486	cmr|protein of aro operon, regulated by aroR [Z0486]
arrD	+15.18	9.92	+3.92	7.96	0.1	11.88	0.1	0.00000237	0.00027039	A_07_P016536	b0555	cmr|bacteriophage lambda lysozyme homolog [b0555]
artI	+2.97	8.53	+1.57	7.75	0.1	9.32	0.1	0.00032893	0.00361129	A_07_P004766	b0863	cmr|arginine 3rd transport system periplasmic binding protein [b0863]
artP	+6.73	9.24	+2.75	7.87	0.2	10.62	0.1	0.00003721	0.00087055	A_07_P004773	b0864	cmr|ATP-binding component of 3rd arginine transport system [b0864]
asd	+3.63	9.52	+1.86	8.59	0.1	10.45	0.0	0.00006644	0.00120815	A_07_P018194	b3433	cmr|aspartate-semialdehyde dehydrogenase [b3433]
asnB	+2.66	9.43	+1.41	8.72	0.1	10.13	0.1	0.00041609	0.00423720	A_07_P046247	Z0821	cmr|asparagine synthetase B [Z0821]
asnB	+2.33	9.32	+1.22	8.71	0.2	9.93	0.1	0.00189978	0.01272086	A_07_P056567	ECs0704	cmr|asparagine synthetase B [ECs0704]
aspA	+5.99	15.44	+2.58	14.15	0.0	16.73	0.0	0.00001089	0.00047910	A_07_P011426	b4139	cmr|aspartate ammonia-lyase [b4139]
aspC	+5.75	9.54	+2.52	8.28	0.2	10.80	0.0	0.00002937	0.00076000	A_07_P052047	Z1275	cmr|aspartate aminotransferase [Z1275]
aspC	+6.18	10.90	+2.63	9.58	0.1	12.21	0.0	0.00001596	0.00056395	A_07_P005088	b0928	cmr|aspartate aminotransferase [b0928]
asr	-5.81	10.46	-2.54	11.73	0.0	9.19	0.1	0.00002243	0.00067002	A_07_P020616	b1597	cmr|acid shock protein [b1597]
astA	+3.05	8.31	+1.61	7.51	0.0	9.11	0.1	0.00016911	0.00225435	A_07_P018783	b1747	cmr|orf, hypothetical protein [b1747]
astC	+2.07	7.10	+1.05	6.58	0.1	7.62	0.1	0.00114177	0.00876450	A_07_P018786	b1748	cmr|acetylornithine delta-aminotransferase [b1748]
atpA	+3.07	14.98	+1.62	14.18	0.0	15.79	0.0	0.00012124	0.00178598	A_07_P005591	b3734	cmr|membrane-bound ATP synthase, F1 sector, alpha-subunit [b3734]
atpB	+2.15	12.51	+1.10	11.96	0.0	13.06	0.0	0.00072413	0.00627400	A_07_P005614	b3738	cmr|membrane-bound ATP synthase, F0 sector, subunit a [b3738]
atpD	+3.17	11.27	+1.67	10.44	0.0	12.10	0.0	0.00009419	0.00150861	A_07_P005582	b3732	cmr|membrane-bound ATP synthase, F1 sector, beta-subunit [b3732]
atpE	+4.40	12.62	+2.14	11.55	0.0	13.69	0.0	0.00003335	0.00081556	A_07_P005607	b3737	cmr|membrane-bound ATP synthase, F0 sector, subunit c [b3737]
atpG	+3.77	12.10	+1.92	11.14	0.0	13.06	0.0	0.00005306	0.00105884	A_07_P005587	b3733	cmr|membrane-bound ATP synthase, F1 sector, gamma-subunit [b3733]
atpH	+7.95	13.09	+2.99	11.60	0.1	14.59	0.1	0.00000792	0.00041039	A_07_P005596	b3735	cmr|membrane-bound ATP synthase, F1 sector, delta-subunit [b3735]
azoR	+2.60	8.88	+1.38	8.19	0.2	9.57	0.1	0.00122668	0.00920538	A_07_P009153	b1412	cmr|acyl carrier protein phosphodiesterase [b1412]
b0322	-5.16	9.24	-2.37	10.43	0.1	8.06	0.1	0.00004256	0.00093353	A_07_P008305	b0322	cmr|orf, hypothetical protein [b0322]
b0359	-2.25	8.60	-1.17	9.18	0.3	8.01	0.1	0.00402529	0.02229975	A_07_P020924	b0359	cmr|putative transferase [b0359]
b0359	-2.32	7.78	-1.22	8.38	0.1	7.17	0.0	0.00068608	0.00602876	A_07_P010289	b0359	cmr|putative transferase [b0359]
b0362	-2.23	7.10	-1.16	7.68	0.3	6.52	0.2	0.00463381	0.02479764	A_07_P020932	b0362	cmr|orf, hypothetical protein [b0362]
b0374	-5.61	8.35	-2.49	9.60	0.1	7.11	0.0	0.00001907	0.00061714	A_07_P020983	b0374	cmr|putative flagellin structural protein [b0374]
b0395	+2.57	7.55	+1.36	6.87	0.0	8.23	0.1	0.00054608	0.00516365	A_07_P021086	b0395	cmr|orf, hypothetical protein [b0395]
b0538	+2.25	7.97	+1.17	7.39	0.0	8.56	0.2	0.00156643	0.01104171	A_07_P016479	b0538	cmr|putative sensory transduction regulator [b0538]
b0671	-3.14	8.15	-1.65	8.98	0.0	7.33	0.2	0.00056765	0.00528823	A_07_P009885	b0671	cmr|putative RNA [b0671]
b0701	+2.17	8.91	+1.12	8.35	0.1	9.47	0.3	0.00711103	0.03444804	A_07_P010022	b0701	cmr|protein in rhs element [b0701]
b0725	+12.15	8.71	+3.60	6.91	0.0	10.51	0.1	0.00000313	0.00028944	A_07_P010142	b0725	cmr|orf, hypothetical protein [b0725]
b1170	-3.45	8.19	-1.78	9.09	0.1	7.30	0.0	0.00007884	0.00134340	A_07_P006805	b1170	cmr|putative part of putative ATP-binding component of a transport system [b1170]
b1543	-2.76	7.36	-1.46	8.09	0.0	6.63	0.2	0.00096859	0.00775143	A_07_P020361	b1543	cmr|putative transport protein [b1543]
b1995	-4.93	8.18	-2.30	9.34	0.0	7.03	0.1	0.00004494	0.00095618	A_07_P015310	b1995	cmr|orf, hypothetical protein [b1995]
b2087	+2.15	7.46	+1.11	6.91	0.1	8.02	0.0	0.00123195	0.00922733	A_07_P003208	b2087	cmr|split galactitol utilization operon repressor, interrupted [b2087]
b2680	-2.16	7.79	-1.11	8.34	0.2	7.23	0.0	0.00343917	0.01990336	A_07_P001871	b2680	cmr|orf, hypothetical protein [b2680]
b3016	-2.16	9.55	-1.11	10.11	0.0	9.00	0.1	0.00089191	0.00735583	A_07_P012215	b3016	cmr|orf, hypothetical protein [b3016]
b3111	+5.79	9.07	+2.53	7.80	0.0	10.34	0.1	0.00002355	0.00068545	A_07_P000282	b3111	cmr|putative L-serine dehydratase [b3111]
b3112	+4.39	8.38	+2.13	7.31	0.1	9.45	0.1	0.00005645	0.00109452	A_07_P000290	b3112	cmr|putative L-serine dehydratase [b3112]
b3763	+2.55	8.51	+1.35	7.83	0.0	9.19	0.1	0.00030764	0.00342294	A_07_P005708	b3763	cmr|regulator of pssA [b3763]
b3837	-3.65	11.98	-1.87	12.91	0.0	11.04	0.1	0.00007228	0.00126864	A_07_P018994	b3837	cmr|putative histone [b3837]
b3841	-3.54	10.13	-1.83	11.05	0.0	9.22	0.1	0.00007592	0.00131203	A_07_P019013	b3841	cmr|orf, hypothetical protein [b3841]
b3914	-2.01	12.33	-1.01	12.83	0.0	11.82	0.0	0.00113026	0.00870163	A_07_P019349	b3914	cmr|orf, hypothetical protein [b3914]
b4285	-2.60	15.23	-1.38	15.92	0.0	14.54	0.2	0.00092963	0.00755603	A_07_P003953	b4285	cmr|putative transposase [b4285]
bamD	-2.20	11.75	-1.14	12.32	0.1	11.19	0.0	0.00067416	0.00595430	A_07_P019860	b2595	cmr|orf, hypothetical protein [b2595]
basR	+2.37	9.78	+1.25	9.15	0.1	10.40	0.2	0.00189154	0.01267793	A_07_P037777	c_5118	cmr|Transcriptional Regulatory protein basR/pmrA [c_5118]
basS	-2.56	12.63	-1.36	13.31	0.5	11.95	0.1	0.01062794	0.04700177	A_07_P011296	b4112	cmr|sensor protein for basR [b4112]
bax	-6.61	12.16	-2.72	13.53	0.0	10.80	0.0	0.00000853	0.00042498	A_07_P007778	b3570	cmr|putative ATP-binding protein [b3570]
bcp	+11.85	10.07	+3.57	8.28	0.1	11.85	0.0	0.00000266	0.00027706	A_07_P013456	b2480	cmr|bacterioferritin comigratory protein [b2480]
bcsA	-3.05	8.67	-1.61	9.48	0.1	7.87	0.1	0.00020561	0.00259797	A_07_P007596	b3533	cmr|putative cellulose synthase [b3533]
bcsF	-3.29	7.94	-1.72	8.80	0.1	7.08	0.1	0.00011046	0.00167703	A_07_P007618	b3537	cmr|orf, hypothetical protein [b3537]
betA	+2.55	9.08	+1.35	8.41	0.0	9.76	0.1	0.00059532	0.00547004	A_07_P043676	Z0398	cmr|choline dehydrogenase, a flavoprotein [Z0398]
betA	+3.67	9.64	+1.88	8.70	0.2	10.58	0.1	0.00031830	0.00352414	A_07_P008247	b0311	cmr|choline dehydrogenase, a flavoprotein [b0311]
betA	+3.98	10.31	+1.99	9.31	0.2	11.30	0.0	0.00009776	0.00154382	A_07_P030984	c_0431	cmr|Choline dehydrogenase [c_0431]
betB	+2.90	12.14	+1.53	11.37	0.1	12.90	0.1	0.00018926	0.00244038	A_07_P008251	b0312	cmr|NAD+-dependent betaine aldehyde dehydrogenase [b0312]
bfr	-2.16	9.65	-1.11	10.21	0.1	9.10	0.2	0.00211430	0.01376089	A_07_P017709	b3336	cmr|bacterioferrin, an iron storage homoprotein [b3336]
bhsA	-9.66	11.09	-3.27	12.73	0.1	9.46	0.0	0.00000390	0.00032155	A_07_P001765	b1112	cmr|orf, hypothetical protein [b1112]
bioB	+2.00	7.43	+1.00	6.93	0.2	7.93	0.0	0.00389569	0.02185785	A_07_P004326	b0775	cmr|biotin synthesis, sulfur insertion? [b0775]
birA	-2.70	11.22	-1.44	11.94	0.0	10.51	0.0	0.00021972	0.00271742	A_07_P008550	b3973	cmr|biotin- [b3973]
bolA	+12.50	9.95	+3.64	8.13	0.0	11.78	0.0	0.00000208	0.00026053	A_07_P021288	b0435	cmr|possible regulator of murein genes [b0435]
borD	+2.16	9.18	+1.11	8.63	0.0	9.74	0.0	0.00073483	0.00634270	A_07_P016549	b0557	cmr|bacteriophage lambda Bor protein homolog [b0557]
bssS	-10.62	13.10	-3.41	14.80	0.0	11.39	0.0	0.00000287	0.00028251	A_07_P001504	b1060	cmr|orf, hypothetical protein [b1060]
btuB	-5.02	11.33	-2.33	12.49	0.0	10.17	0.1	0.00002328	0.00068274	A_07_P008533	b3966	cmr|outer membrane receptor for transport of vitamin B12, E colicins, and bacteriophage BF23 [b3966]
btuE	-2.07	10.79	-1.05	11.31	0.1	10.26	0.2	0.00428059	0.02334963	A_07_P035160	c_2106	cmr|Vitamin B12 transport periplasmic protein btuE [c_2106]
c_0036	+2.42	8.73	+1.28	8.10	0.2	9.37	0.1	0.00102614	0.00807690	A_07_P035658	c_0036	cmr|Hypothetical protein [c_0036]
c_0059	-2.43	11.77	-1.28	12.41	0.1	11.13	0.1	0.00100872	0.00798031	A_07_P035683	c_0059	cmr|Putative antitoxin of gyrase inhibiting toxin-antitoxin system [c_0059]
c_0062	-2.74	7.76	-1.45	8.48	0.1	7.03	0.2	0.00088084	0.00727980	A_07_P035698	c_0062	cmr|Hypothetical protein [c_0062]
c_0086	-2.26	10.58	-1.18	11.17	0.2	9.99	0.0	0.00133286	0.00975571	A_07_P035735	c_0086	cmr|Transposase [c_0086]
c_0088	-2.75	9.14	-1.46	9.87	0.1	8.40	0.1	0.00026470	0.00309464	A_07_P035738	c_0088	cmr|Hypothetical protein [c_0088]
c_0120	-4.85	9.83	-2.28	10.97	0.0	8.69	0.1	0.00004695	0.00098031	A_07_P035766	c_0120	cmr|Hypothetical protein [c_0120]
c_0138	-2.95	9.16	-1.56	9.94	0.0	8.38	0.3	0.00188395	0.01265396	A_07_P035783	c_0138	cmr|Unknown protein of IS629 encoded within prophage [c_0138]
c_0226	-2.11	9.43	-1.08	9.96	0.1	8.89	0.1	0.00120437	0.00909682	A_07_P035924	c_0226	cmr|Putative cell cycle protein mesJ [c_0226]
c_0256	-2.24	8.14	-1.17	8.72	0.1	7.55	0.0	0.00080857	0.00682945	A_07_P035961	c_0256	cmr|Hypothetical protein [c_0256]
c_0257	-6.24	10.70	-2.64	12.02	0.0	9.38	0.0	0.00001054	0.00047145	A_07_P035967	c_0257	cmr|Unknown in ISEc8 [c_0257]
c_0258	-3.00	11.46	-1.59	12.25	0.2	10.67	0.3	0.00132772	0.00973617	A_07_P035972	c_0258	cmr|Unknown in ISEc8 [c_0258]
c_0260	-2.43	7.83	-1.28	8.47	0.1	7.19	0.1	0.00089799	0.00739046	A_07_P035978	c_0260	cmr|Hypothetical protein [c_0260]
c_0261	-2.64	7.73	-1.40	8.43	0.0	7.03	0.1	0.00030620	0.00341417	A_07_P035987	c_0261	cmr|Hypothetical protein [c_0261]
c_0264	-3.10	8.09	-1.63	8.91	0.0	7.28	0.1	0.00013542	0.00192967	A_07_P036004	c_0264	cmr|Hypothetical protein [c_0264]
c_0274	-2.21	8.34	-1.14	8.91	0.1	7.77	0.2	0.00162382	0.01133721	A_07_P036041	c_0274	cmr|Hypothetical protein [c_0274]
c_0279	-3.27	7.80	-1.71	8.65	0.2	6.94	0.3	0.00133984	0.00979769	A_07_P036068	c_0279	cmr|Hypothetical protein ykfF [c_0279]
c_0281	-2.48	10.41	-1.31	11.07	0.0	9.75	0.1	0.00042408	0.00430129	A_07_P036079	c_0281	cmr|Hypothetical protein [c_0281]
c_0286	-4.41	9.75	-2.14	10.82	0.1	8.68	0.0	0.00003331	0.00081556	A_07_P036101	c_0286	cmr|Conserved hypothetical protein [c_0286]
c_0289	-4.66	8.37	-2.22	9.48	0.1	7.26	0.1	0.00004710	0.00098224	A_07_P036114	c_0289	cmr|Hypothetical protein [c_0289]
c_0301	-2.44	7.54	-1.29	8.18	0.0	6.90	0.1	0.00065632	0.00585114	A_07_P036159	c_0301	cmr|Hypothetical protein [c_0301]
c_0305	-2.14	10.25	-1.10	10.80	0.3	9.70	0.0	0.00729231	0.03517466	A_07_P036170	c_0305	cmr|Hypothetical protein [c_0305]
c_0309	-3.29	8.26	-1.72	9.11	0.1	7.40	0.0	0.00010537	0.00163463	A_07_P036181	c_0309	cmr|Conserved hypothetical protein [c_0309]
c_0313	-2.82	12.97	-1.49	13.72	0.3	12.22	0.3	0.00403642	0.02233720	A_07_P036201	c_0313	cmr|Hypothetical protein [c_0313]
c_0316	-2.96	8.56	-1.56	9.34	0.1	7.78	0.1	0.00017123	0.00227395	A_07_P036219	c_0316	cmr|Hypothetical protein [c_0316]
c_0328	-2.81	8.16	-1.49	8.91	0.0	7.41	0.1	0.00020740	0.00261010	A_07_P036267	c_0328	cmr|Hypothetical protein [c_0328]
c_0331	+2.62	8.29	+1.39	7.60	0.0	8.99	0.0	0.00023073	0.00281851	A_07_P036277	c_0331	cmr|Putative ribokinase [c_0331]
c_0337	-8.57	10.44	-3.10	11.99	0.0	8.89	0.2	0.00001356	0.00052725	A_07_P030738	c_0337	cmr|Putative conserved protein [c_0337]
c_0339	-2.64	9.40	-1.40	10.10	0.1	8.70	0.0	0.00030504	0.00340368	A_07_P030741	c_0339	cmr|Hypothetical protein [c_0339]
c_0340	-3.07	8.70	-1.62	9.51	0.1	7.89	0.1	0.00018156	0.00237762	A_07_P030749	c_0340	cmr|Conserved hypothetical protein [c_0340]
c_0348	-2.08	9.26	-1.06	9.79	0.0	8.73	0.1	0.00136343	0.00991481	A_07_P030761	c_0348	cmr|Hypothetical protein [c_0348]
c_0349	-3.47	7.98	-1.79	8.88	0.2	7.08	0.1	0.00028803	0.00328124	A_07_P030767	c_0349	cmr|Putative Transposase within prophage [c_0349]
c_0352	-4.68	9.79	-2.23	10.90	0.1	8.67	0.0	0.00002667	0.00073020	A_07_P030781	c_0352	cmr|Partial Transposase [c_0352]
c_0355	-3.62	9.32	-1.86	10.25	0.2	8.39	0.2	0.00037099	0.00392997	A_07_P030788	c_0355	cmr|Hypothetical protein [c_0355]
c_0357	-2.38	8.22	-1.25	8.85	0.1	7.60	0.4	0.00766725	0.03655758	A_07_P030794	c_0357	cmr|Hypothetical protein [c_0357]
c_0358	-2.44	8.57	-1.29	9.21	0.1	7.93	0.1	0.00062207	0.00563493	A_07_P030798	c_0358	cmr|Hypothetical protein [c_0358]
c_0365	-4.16	8.34	-2.06	9.37	0.1	7.32	0.1	0.00004787	0.00099032	A_07_P030828	c_0365	cmr|Hypothetical protein [c_0365]
c_0385	-3.65	8.26	-1.87	9.19	0.0	7.33	0.0	0.00006422	0.00118391	A_07_P030859	c_0385	cmr|Hypothetical protein [c_0385]
c_0386	+2.00	9.98	+1.00	9.48	0.1	10.48	0.0	0.00154186	0.01093470	A_07_P041475	c_0386	cmr|Hypothetical protein yafA [c_0386]
c_0396	-2.48	7.63	-1.31	8.29	0.0	6.98	0.1	0.00043303	0.00435910	A_07_P030886	c_0396	cmr|Insertion element IS1 1/2/3/5/6 protein insA [c_0396]
c_0397	-2.77	7.99	-1.47	8.73	0.0	7.26	0.1	0.00027043	0.00313734	A_07_P030892	c_0397	cmr|InsB protein [c_0397]
c_0410	+4.08	8.65	+2.03	7.64	0.0	9.67	0.0	0.00003972	0.00090487	A_07_P030916	c_0410	cmr|Hypothetical protein ycjY [c_0410]
c_0412	-2.09	9.79	-1.06	10.32	0.1	9.26	0.0	0.00113391	0.00872521	A_07_P030929	c_0412	cmr|Hypothetical transcriptional regulator ycjZ [c_0412]
c_0413	+3.16	7.48	+1.66	6.65	0.2	8.31	0.1	0.00061065	0.00556489	A_07_P030931	c_0413	cmr|Putative aldo/keto reductase [c_0413]
c_0414	+3.73	8.37	+1.90	7.42	0.0	9.32	0.2	0.00011679	0.00174120	A_07_P030940	c_0414	cmr|2,5-diketo-D-gluconic acid reductase A [c_0414]
c_0427	-3.54	8.06	-1.82	8.97	0.3	7.15	0.0	0.00088054	0.00727980	A_07_P030962	c_0427	cmr|Conserved hypothetical protein [c_0427]
c_0444	+2.01	7.77	+1.01	7.26	0.2	8.27	0.0	0.00430865	0.02346263	A_07_P031002	c_0444	cmr|Carbamate kinase-like protein yahI [c_0444]
c_0481	-2.65	8.82	-1.40	9.52	0.0	8.12	0.0	0.00022165	0.00273628	A_07_P031085	c_0481	cmr|Hypothetical protein [c_0481]
c_0487	-3.20	11.67	-1.68	12.51	0.0	10.83	0.1	0.00010677	0.00164437	A_07_P041502	c_0487	cmr|D-alanine--D-alanine ligase A [c_0487]
c_0503	+2.36	9.39	+1.24	8.77	0.1	10.01	0.1	0.00098623	0.00783803	A_07_P031097	c_0503	cmr|Hypothetical protein yajF [c_0503]
c_0550	+3.82	7.62	+1.93	6.65	0.1	8.58	0.1	0.00007556	0.00131017	A_07_P031150	c_0550	cmr|Hypothetical protein [c_0550]
c_0552	+2.07	7.07	+1.05	6.55	0.1	7.60	0.0	0.00116466	0.00890119	A_07_P031156	c_0552	cmr|Hypothetical protein [c_0552]
c_0583	-3.49	10.61	-1.80	11.51	0.1	9.71	0.0	0.00012441	0.00181695	A_07_P031182	c_0583	cmr|Hypothetical protein [c_0583]
c_0608	-2.32	10.24	-1.21	10.85	0.0	9.64	0.0	0.00047317	0.00465311	A_07_P031211	c_0608	cmr|Hypothetical protein [c_0608]
c_0638	-2.33	8.44	-1.22	9.05	0.1	7.83	0.1	0.00108042	0.00840005	A_07_P031250	c_0638	cmr|Hypothetical protein [c_0638]
c_0640	+2.59	7.79	+1.37	7.10	0.1	8.47	0.0	0.00037748	0.00396055	A_07_P031257	c_0640	cmr|Conserved hypothetical protein [c_0640]
c_0697	-2.79	8.78	-1.48	9.52	0.1	8.04	0.2	0.00045565	0.00453750	A_07_P031357	c_0697	cmr|Conserved hypothetical protein [c_0697]
c_0719	-2.58	8.08	-1.37	8.77	0.1	7.40	0.1	0.00049063	0.00476059	A_07_P033287	c_0719	cmr|Conserved hypothetical protein [c_0719]
c_0778	+7.10	8.15	+2.83	6.73	0.0	9.56	0.2	0.00001972	0.00062719	A_07_P033361	c_0778	cmr|Hypothetical protein [c_0778]
c_0784	-6.02	8.62	-2.59	9.92	0.0	7.33	0.2	0.00006854	0.00122908	A_07_P033375	c_0784	cmr|Hypothetical protein [c_0784]
c_0888	+2.23	8.53	+1.16	7.95	0.0	9.11	0.0	0.00063352	0.00570950	A_07_P042104	c_0888	cmr|Hypothetical protein ybiI [c_0888]
c_0897	+17.64	13.21	+4.14	11.14	0.1	15.28	0.1	0.00000209	0.00026053	A_07_P033506	c_0897	cmr|Hypothetical protein [c_0897]
c_0953	+2.00	7.09	+1.00	6.59	0.2	7.59	0.2	0.00654861	0.03227968	A_07_P033627	c_0953	cmr|Terminase, endonuclease subunit [c_0953]
c_1166	+3.03	8.79	+1.60	7.99	0.2	9.59	0.1	0.00057619	0.00534565	A_07_P033974	c_1166	cmr|Conserved hypothetical protein [c_1166]
c_1172	-4.71	8.05	-2.23	9.17	0.1	6.93	0.0	0.00003094	0.00078443	A_07_P034010	c_1172	cmr|Hypothetical protein [c_1172]
c_1173	-3.08	11.64	-1.62	12.46	0.1	10.83	0.3	0.00084253	0.00704450	A_07_P034004	c_1173	cmr|Hypothetical protein [c_1173]
c_1174	-2.80	9.75	-1.49	10.49	0.1	9.01	0.1	0.00035343	0.00379046	A_07_P040226	c_1174	cmr|Hypothetical protein [c_1174]
c_1174	-3.62	9.78	-1.86	10.70	0.2	8.85	0.3	0.00058340	0.00539033	A_07_P040226	c_1174	cmr|Hypothetical protein [c_1174]
c_1174	-3.56	9.58	-1.83	10.50	0.1	8.67	0.3	0.00062276	0.00563493	A_07_P040226	c_1174	cmr|Hypothetical protein [c_1174]
c_1174	-3.47	9.63	-1.79	10.53	0.2	8.73	0.5	0.00394459	0.02201473	A_07_P040226	c_1174	cmr|Hypothetical protein [c_1174]
c_1174	-3.39	9.66	-1.76	10.54	0.2	8.78	0.4	0.00279979	0.01698430	A_07_P040226	c_1174	cmr|Hypothetical protein [c_1174]
c_1175	+3.43	7.76	+1.78	6.87	0.1	8.65	0.2	0.00027580	0.00318111	A_07_P040232	c_1175	cmr|Putative aminotransferase [c_1175]
c_1186	-2.19	9.03	-1.13	9.60	0.1	8.47	0.1	0.00092040	0.00750815	A_07_P040258	c_1186	cmr|Putative beta-ketoacyl-ACP synthase [c_1186]
c_1197	-2.19	9.00	-1.13	9.56	0.0	8.43	0.2	0.00164534	0.01143172	A_07_P040310	c_1197	cmr|Putative enzyme [c_1197]
c_1198	-2.69	8.74	-1.43	9.45	0.0	8.02	0.2	0.00052319	0.00499828	A_07_P040314	c_1198	cmr|Conserved hypothetical protein [c_1198]
c_1199	-2.64	10.70	-1.40	11.41	0.0	10.00	0.0	0.00025102	0.00299696	A_07_P040317	c_1199	cmr|Putative acyl carrier protein [c_1199]
c_1202	-2.59	7.59	-1.37	8.28	0.0	6.91	0.0	0.00025967	0.00307016	A_07_P040334	c_1202	cmr|Conserved hypothetical protein [c_1202]
c_1203	-3.32	9.40	-1.73	10.27	0.1	8.54	0.0	0.00008761	0.00144743	A_07_P040338	c_1203	cmr|Putative O-methyltransferase [c_1203]
c_1204	-3.83	9.79	-1.94	10.76	0.1	8.82	0.0	0.00008318	0.00140064	A_07_P040344	c_1204	cmr|Conserved hypothetical protein [c_1204]
c_1204	-4.57	10.12	-2.19	11.22	0.0	9.02	0.1	0.00002756	0.00073830	A_07_P040344	c_1204	cmr|Conserved hypothetical protein [c_1204]
c_1204	-3.72	10.13	-1.89	11.07	0.1	9.18	0.0	0.00007479	0.00129967	A_07_P040344	c_1204	cmr|Conserved hypothetical protein [c_1204]
c_1204	-4.06	9.89	-2.02	10.90	0.1	8.88	0.0	0.00005793	0.00111357	A_07_P040344	c_1204	cmr|Conserved hypothetical protein [c_1204]
c_1204	-4.32	9.98	-2.11	11.04	0.1	8.93	0.1	0.00005705	0.00110340	A_07_P040344	c_1204	cmr|Conserved hypothetical protein [c_1204]
c_1209	-2.42	9.59	-1.27	10.23	0.2	8.95	0.1	0.00113682	0.00873918	A_07_P040366	c_1209	cmr|Hypothetical protein [c_1209]
c_1214	+2.83	12.42	+1.50	11.67	0.1	13.17	0.1	0.00021751	0.00269643	A_07_P040383	c_1214	cmr|Cea protein [c_1214]
c_1215	-4.45	12.59	-2.15	13.67	0.0	11.52	0.0	0.00002748	0.00073821	A_07_P040390	c_1215	cmr|Entry exclusion protein 2 [c_1215]
c_1219	-2.22	8.30	-1.15	8.88	0.0	7.73	0.2	0.00307548	0.01823741	A_07_P040407	c_1219	cmr|Putative Transposase [c_1219]
c_1225	-2.43	10.09	-1.28	10.73	0.1	9.45	0.0	0.00065722	0.00585582	A_07_P040430	c_1225	cmr|Transposase insE for insertion sequence IS3A/B/C/D/E/fA/fB [c_1225]
c_1235	-10.31	8.83	-3.37	10.51	0.0	7.14	0.1	0.00000412	0.00032560	A_07_P040469	c_1235	cmr|Conserved hypothetical protein [c_1235]
c_1236	-4.88	9.14	-2.29	10.29	0.0	8.00	0.5	0.00146771	0.01050345	A_07_P040474	c_1236	cmr|Conserved hypothetical protein [c_1236]
c_1253	-5.00	9.55	-2.32	10.72	0.1	8.39	0.1	0.00002892	0.00075516	A_07_P040560	c_1253	cmr|ATP binding cassette [c_1253]
c_1257	-23.41	11.02	-4.55	13.29	0.1	8.74	0.0	0.00000088	0.00019431	A_07_P040573	c_1257	cmr|Putative conserved protein [c_1257]
c_1258	-2.24	7.77	-1.17	8.35	0.4	7.18	0.0	0.00758089	0.03625561	A_07_P040577	c_1258	cmr|Hypothetical protein [c_1258]
c_1261	-2.11	9.49	-1.08	10.03	0.0	8.96	0.0	0.00081180	0.00684205	A_07_P040595	c_1261	cmr|Hypothetical protein [c_1261]
c_1267	-3.53	8.48	-1.82	9.39	0.5	7.57	0.1	0.00397885	0.02211190	A_07_P040614	c_1267	cmr|Hypothetical protein [c_1267]
c_1268	-2.08	8.45	-1.06	8.98	0.0	7.92	0.1	0.00126934	0.00944895	A_07_P040616	c_1268	cmr|Hypothetical protein [c_1268]
c_1271	-2.35	8.50	-1.23	9.12	0.1	7.89	0.0	0.00049393	0.00478257	A_07_P040631	c_1271	cmr|Hypothetical protein [c_1271]
c_1272	-4.34	8.07	-2.12	9.13	0.1	7.01	0.0	0.00003790	0.00087581	A_07_P040636	c_1272	cmr|Hypothetical protein yeeP [c_1272]
c_1273	+4.01	11.44	+2.00	10.43	0.1	12.44	0.1	0.00006645	0.00120815	A_07_P040643	c_1273	cmr|Antigen 43 precursor [c_1273]
c_1284	-2.84	11.51	-1.50	12.26	0.2	10.75	0.5	0.01033846	0.04588910	A_07_P040687	c_1284	cmr|Hypothetical protein yeeT [c_1284]
c_1286	-5.42	10.23	-2.44	11.45	0.0	9.01	0.6	0.00131315	0.00970625	A_07_P040696	c_1286	cmr|Hypothetical protein [c_1286]
c_1294	-3.68	7.70	-1.88	8.64	0.0	6.76	0.0	0.00005423	0.00107304	A_07_P040732	c_1294	cmr|Hypothetical protein [c_1294]
c_1363	+2.29	8.02	+1.20	7.42	0.1	8.62	0.1	0.00064173	0.00576675	A_07_P040789	c_1363	cmr|Hypothetical protein [c_1363]
c_1406	-3.46	8.26	-1.79	9.16	0.1	7.37	0.2	0.00018722	0.00242003	A_07_P040862	c_1406	cmr|Hypothetical protein [c_1406]
c_1421	-9.27	9.73	-3.21	11.34	0.1	8.12	0.0	0.00000594	0.00036511	A_07_P040925	c_1421	cmr|Hypothetical protein [c_1421]
c_1436	-2.08	8.97	-1.06	9.50	0.2	8.44	0.0	0.00385479	0.02169043	A_07_P042220	c_1436	cmr|Probable lysozyme from lambdoid prophage Qin [c_1436]
c_1445	+2.61	10.68	+1.39	9.98	0.2	11.37	0.1	0.00093603	0.00758059	A_07_P041010	c_1445	cmr|Putative DNA packaging protein of prophage terminase [c_1445]
c_1446	+3.01	10.88	+1.59	10.09	0.3	11.68	0.1	0.00184013	0.01242327	A_07_P041015	c_1446	cmr|Putative DNA packaging protein of prophage [c_1446]
c_1448	+23.01	11.32	+4.52	9.05	0.1	13.58	0.0	0.00000082	0.00019431	A_07_P041021	c_1448	cmr|Putative capsid assembly protein of prophage [c_1448]
c_1453	+12.23	11.45	+3.61	9.64	0.1	13.25	0.2	0.00000783	0.00041039	A_07_P038108	c_1453	cmr|Putative head-tail joining protein of prophage [c_1453]
c_1456	-2.07	11.26	-1.05	11.78	0.2	10.73	0.2	0.00725882	0.03504535	A_07_P038121	c_1456	cmr|Putative tail fiber component V of prophage [c_1456]
c_1478	+3.08	8.67	+1.62	7.86	0.1	9.48	0.0	0.00013550	0.00192967	A_07_P038214	c_1478	cmr|Hypothetical protein [c_1478]
c_1484	-2.25	9.11	-1.17	9.70	0.4	8.53	0.1	0.00790421	0.03747183	A_07_P038229	c_1484	cmr|Unknown protein encoded by prophage [c_1484]
c_1487	-2.18	10.41	-1.12	10.98	0.1	9.85	0.1	0.00117338	0.00894178	A_07_P038235	c_1487	cmr|Hypothetical protein [c_1487]
c_1499	+2.37	8.59	+1.25	7.97	0.0	9.21	0.1	0.00061432	0.00558100	A_07_P038295	c_1499	cmr|Putative capsid protein of prophage [c_1499]
c_1501	+3.04	8.97	+1.60	8.17	0.0	9.78	0.1	0.00018346	0.00238910	A_07_P038305	c_1501	cmr|Hypothetical protein [c_1501]
c_1516	+2.50	7.34	+1.32	6.68	0.2	8.00	0.1	0.00151988	0.01081784	A_07_P038337	c_1516	cmr|Hypothetical protein [c_1516]
c_1519	+14.69	8.96	+3.88	7.02	0.3	10.89	0.2	0.00004663	0.00097506	A_07_P038347	c_1519	cmr|Prophage lambda integrase [c_1519]
c_1522	+112.15	11.07	+6.81	7.66	0.4	14.47	0.1	0.00000258	0.00027706	A_07_P038354	c_1522	cmr|Hypothetical protein [c_1522]
c_1523	+68.86	12.31	+6.11	9.26	0.1	15.36	0.1	0.00000025	0.00019431	A_07_P035459	c_1523	cmr|Unknown protein encoded by bacteriophage [c_1523]
c_1524	+84.18	11.31	+6.40	8.12	0.0	14.51	0.0	0.00000011	0.00019431	A_07_P038365	c_1524	cmr|Hypothetical protein [c_1524]
c_1525	+85.60	12.65	+6.42	9.44	0.0	15.86	0.0	0.00000013	0.00019431	A_07_P038369	c_1525	cmr|Hypothetical protein [c_1525]
c_1526	+83.64	10.95	+6.39	7.76	0.1	14.15	0.1	0.00000024	0.00019431	A_07_P038374	c_1526	cmr|Hypothetical protein [c_1526]
c_1527	+65.16	10.66	+6.03	7.65	0.3	13.67	0.0	0.00000246	0.00027471	A_07_P038377	c_1527	cmr|Hypothetical protein [c_1527]
c_1528	+117.66	11.09	+6.88	7.65	0.3	14.53	0.1	0.00000081	0.00019431	A_07_P038381	c_1528	cmr|Hypothetical protein [c_1528]
c_1531	+134.56	11.60	+7.07	8.06	0.0	15.13	0.0	0.00000007	0.00019431	A_07_P038395	c_1531	cmr|Hypothetical protein [c_1531]
c_1532	+41.70	11.68	+5.38	8.99	0.3	14.37	0.2	0.00000618	0.00036832	A_07_P038400	c_1532	cmr|Unknown protein encoded within prophage [c_1532]
c_1533	+5.71	13.07	+2.51	11.81	0.3	14.33	0.1	0.00010487	0.00162985	A_07_P038402	c_1533	cmr|Hypothetical protein [c_1533]
c_1536	+5.71	14.23	+2.51	12.98	0.1	15.49	0.1	0.00002464	0.00070547	A_07_P038406	c_1536	cmr|Putative recombination protein Bet of prophage [c_1536]
c_1538	+31.45	13.16	+4.98	10.67	0.0	15.65	0.0	0.00000044	0.00019431	A_07_P038419	c_1538	cmr|Hypothetical protein [c_1538]
c_1540	+8.29	14.93	+3.05	13.40	0.1	16.45	0.0	0.00000542	0.00034972	A_07_P038423	c_1540	cmr|Lambda Regulatory protein CIII [c_1540]
c_1541	+20.00	14.41	+4.32	12.24	0.0	16.57	0.0	0.00000089	0.00019431	A_07_P038427	c_1541	cmr|Putative single-stranded DNA binding protein of prophage [c_1541]
c_1544	-2.07	8.48	-1.05	9.01	0.0	7.96	0.0	0.00095699	0.00769111	A_07_P038443	c_1544	cmr|Hypothetical protein [c_1544]
c_1546	-5.53	12.18	-2.47	13.42	0.0	10.95	0.1	0.00002038	0.00063193	A_07_P038451	c_1546	cmr|Repressor protein [c_1546]
c_1547	+5.28	13.02	+2.40	11.82	0.1	14.22	0.1	0.00003088	0.00078408	A_07_P038456	c_1547	cmr|Hypothetical protein [c_1547]
c_1548	+5.05	12.47	+2.34	11.30	0.2	13.64	0.0	0.00005610	0.00109168	A_07_P038465	c_1548	cmr|Putative Regulatory protein CII of bacteriophage [c_1548]

c_1549	+10.42	13.56	+3.38	11.87	0.0	15.25	0.0	0.00000282	0.00028143	A_07_P038470	c_1549	cmr|Putative replication protein O of bacteriophage [c_1549]
c_1550	+7.03	15.04	+2.81	13.63	0.1	16.45	0.0	0.00001063	0.00047201	A_07_P038471	c_1550	cmr|Putative replication protein P of bacteriophage [c_1550]
c_1554	+61.33	10.90	+5.94	7.93	0.2	13.87	0.2	0.00000092	0.00019431	A_07_P038485	c_1554	cmr|Unknown protein encoded within prophage [c_1554]
c_1555	+47.13	10.13	+5.56	7.35	0.1	12.91	0.0	0.00000035	0.00019431	A_07_P038490	c_1555	cmr|Putative DNA N-6-adenine-methyltransferase of bacteriophage [c_1555]
c_1556	+152.03	10.91	+7.25	7.28	0.2	14.53	0.0	0.00000015	0.00019431	A_07_P038492	c_1556	cmr|Hypothetical protein [c_1556]
c_1565	+4.38	12.58	+2.13	11.51	0.2	13.65	0.0	0.00016624	0.00223475	A_07_P038514	c_1565	cmr|Partial tonB-like membrane protein encoded within prophage [c_1565]
c_1567	+6.07	10.16	+2.60	8.86	0.1	11.46	0.0	0.00001316	0.00052315	A_07_P038518	c_1567	cmr|Hypothetical protein [c_1567]
c_1569	+2.29	10.50	+1.20	9.90	0.0	11.09	0.1	0.00064805	0.00580043	A_07_P038528	c_1569	cmr|Putative DNA packaging protein of prophage terminase [c_1569]
c_1570	+3.99	11.21	+2.00	10.21	0.4	12.21	0.1	0.00118025	0.00897241	A_07_P038532	c_1570	cmr|Putative DNA packaging protein of prophage [c_1570]
c_1571	+9.96	9.76	+3.32	8.10	0.0	11.42	0.1	0.00000509	0.00034758	A_07_P038538	c_1571	cmr|Putative capsid protein of prophage [c_1571]
c_1572	+60.26	10.81	+5.91	7.86	0.1	13.77	0.0	0.00000022	0.00019431	A_07_P038541	c_1572	cmr|Putative capsid assembly protein of prophage [c_1572]
c_1574	+25.07	10.65	+4.65	8.32	0.1	12.97	0.0	0.00000090	0.00019431	A_07_P038551	c_1574	cmr|Putative capsid protein of prophage [c_1574]
c_1575	+23.31	9.61	+4.54	7.34	0.2	11.88	0.1	0.00000321	0.00029243	A_07_P038556	c_1575	cmr|Putative capsid protein of prophage [c_1575]
c_1576	+15.42	9.62	+3.95	7.65	0.2	11.59	0.0	0.00000786	0.00041039	A_07_P038564	c_1576	cmr|Hypothetical protein [c_1576]
c_1577	+20.87	9.22	+4.38	7.03	0.1	11.41	0.1	0.00000189	0.00025064	A_07_P038569	c_1577	cmr|Putative head-tail joining protein of prophage [c_1577]
c_1579	+9.79	11.19	+3.29	9.55	0.1	12.84	0.0	0.00000627	0.00036832	A_07_P038580	c_1579	cmr|Putative tail component of prophage [c_1579]
c_1580	+47.82	9.82	+5.58	7.03	0.0	12.61	0.2	0.00000070	0.00019431	A_07_P038581	c_1580	cmr|Tail protein [c_1580]
c_1583	+13.48	9.77	+3.75	7.89	0.0	11.65	0.1	0.00000238	0.00027039	A_07_P038594	c_1583	cmr|Putative tail component of prophage [c_1583]
c_1585	+5.71	10.15	+2.51	8.89	0.2	11.41	0.0	0.00005566	0.00108730	A_07_P038605	c_1585	cmr|Putative tail component of prophage [c_1585]
c_1587	+2.97	9.75	+1.57	8.96	0.1	10.53	0.3	0.00146740	0.01050345	A_07_P038615	c_1587	cmr|Putative tail component of prophage [c_1587]
c_1588	+2.39	11.31	+1.26	10.68	0.3	11.93	0.1	0.00470663	0.02512617	A_07_P038617	c_1588	cmr|Putative tail component of prophage [c_1588]
c_1590	+2.68	9.68	+1.42	8.97	0.1	10.40	0.2	0.00068310	0.00601156	A_07_P038626	c_1590	cmr|Putative tail component of prophage [c_1590]
c_1591	+3.27	9.57	+1.71	8.72	0.3	10.43	0.2	0.00171954	0.01183830	A_07_P038637	c_1591	cmr|Hypothetical protein [c_1591]
c_1593	+4.57	8.27	+2.19	7.17	0.2	9.36	0.0	0.00006914	0.00123557	A_07_P038645	c_1593	cmr|Hypothetical protein [c_1593]
c_1596	-3.15	8.87	-1.66	9.70	0.0	8.05	0.1	0.00016016	0.00218551	A_07_P038656	c_1596	cmr|Hypothetical protein [c_1596]
c_1605	-2.56	11.85	-1.36	12.52	0.1	11.17	0.1	0.00065167	0.00582613	A_07_P038687	c_1605	cmr|Hypothetical protein [c_1605]
c_1640	-3.43	11.96	-1.78	12.85	0.1	11.07	0.1	0.00018399	0.00239209	A_07_P038726	c_1640	cmr|Putative Na [c_1640]
c_1657	+2.61	7.88	+1.38	7.19	0.1	8.57	0.1	0.00040724	0.00416878	A_07_P038777	c_1657	cmr|Protein ycgS [c_1657]
c_1660	-2.64	9.68	-1.40	10.38	0.0	8.98	0.0	0.00026068	0.00307888	A_07_P038788	c_1660	cmr|Hypothetical protein [c_1660]
c_1714	-5.80	10.99	-2.54	12.25	0.1	9.72	0.0	0.00001601	0.00056395	A_07_P038863	c_1714	cmr|Hypothetical protein [c_1714]
c_1755	+2.05	8.93	+1.04	8.41	0.0	9.44	0.1	0.00132361	0.00972818	A_07_P038912	c_1755	cmr|Conserved hypothetical protein [c_1755]
c_1819	+2.53	8.92	+1.34	8.25	0.2	9.59	0.1	0.00087705	0.00726750	A_07_P034790	c_1819	cmr|Putative conserved protein [c_1819]
c_1825	+3.91	10.23	+1.97	9.25	0.3	11.22	0.2	0.00044208	0.00442752	A_07_P034801	c_1825	cmr|Hypothetical protein [c_1825]
c_1884	+2.37	7.16	+1.24	6.54	0.3	7.78	0.0	0.00342932	0.01985699	A_07_P034884	c_1884	cmr|Hypothetical protein [c_1884]
c_1887	-2.08	8.33	-1.06	8.86	0.1	7.80	0.1	0.00126651	0.00943235	A_07_P034897	c_1887	cmr|Hypothetical protein [c_1887]
c_1935	-2.86	8.89	-1.52	9.65	0.2	8.13	0.0	0.00036974	0.00392997	A_07_P034987	c_1935	cmr|Chaperone protein fimC precursor [c_1935]
c_1955	-2.07	6.97	-1.05	7.49	0.1	6.44	0.2	0.00313742	0.01855583	A_07_P035036	c_1955	cmr|6-phospho-beta-glucosidase bglA [c_1955]
c_1956	-2.46	11.29	-1.30	11.94	0.2	10.64	0.1	0.00181709	0.01230990	A_07_P035043	c_1956	cmr|Putative outer membrane protein yieC precursor [c_1956]
c_1989	-10.96	10.50	-3.45	12.23	0.1	8.77	0.1	0.00000368	0.00030737	A_07_P035078	c_1989	cmr|Putative acid shock protein [c_1989]
c_2059	+2.21	9.33	+1.14	8.76	0.1	9.90	0.1	0.00137422	0.00997041	A_07_P035140	c_2059	cmr|Protein ydhR precursor [c_2059]
c_2148	+2.78	7.97	+1.47	7.24	0.4	8.71	0.1	0.00470243	0.02512617	A_07_P035198	c_2148	cmr|Succinylornithine transaminase [c_2148]
c_2155	+3.23	9.12	+1.69	8.28	0.2	9.97	0.1	0.00055263	0.00520656	A_07_P042371	c_2155	cmr|Protein ynjB [c_2155]
c_2158	+7.24	9.77	+2.86	8.34	0.0	11.20	0.0	0.00000662	0.00038136	A_07_P041171	c_2158	cmr|Putative thiosulfate sulfurtransferase ynjE precursor [c_2158]
c_2255	-16.55	10.32	-4.05	12.35	0.0	8.30	0.1	0.00000177	0.00024644	A_07_P035318	c_2255	cmr|Hypothetical protein [c_2255]
c_2273	-2.42	9.90	-1.27	10.54	0.0	9.27	0.1	0.00043298	0.00435910	A_07_P035336	c_2273	cmr|High-affinity zinc uptake system membrane protein znuB [c_2273]
c_2348	+22.28	9.69	+4.48	7.45	0.4	11.93	0.1	0.00001880	0.00061398	A_07_P035389	c_2348	cmr|Outer membrane porin protein nmpC precursor [c_2348]
c_2392	-2.88	9.46	-1.53	10.23	0.2	8.70	0.4	0.00565433	0.02897843	A_07_P031413	c_2392	cmr|Putative P4-family integrase [c_2392]
c_2409	+2.37	7.71	+1.25	7.09	0.1	8.33	0.0	0.00044014	0.00441938	A_07_P031493	c_2409	cmr|Hypothetical protein [c_2409]
c_2418	-2.89	9.05	-1.53	9.82	0.3	8.29	0.1	0.00123068	0.00922659	A_07_P031531	c_2418	cmr|Prophage P4 integrase [c_2418]
c_2423	-2.06	8.02	-1.04	8.54	0.1	7.50	0.1	0.00243279	0.01529017	A_07_P031557	c_2423	cmr|Putataive AraC type regulator [c_2423]
c_2455	-2.90	13.20	-1.53	13.96	0.1	12.43	0.1	0.00034552	0.00373874	A_07_P031671	c_2455	cmr|Putative peptide synthetase [c_2455]
c_2458	-3.06	9.56	-1.61	10.37	0.1	8.75	0.2	0.00028353	0.00325162	A_07_P031686	c_2458	cmr|Putative peptide synthetase [c_2458]
c_2460	+3.64	7.47	+1.86	6.54	0.2	8.41	0.1	0.00022080	0.00272866	A_07_P031697	c_2460	cmr|Putative polyketide synthase [c_2460]
c_2461	+2.26	8.26	+1.18	7.67	0.1	8.85	0.2	0.00156620	0.01104171	A_07_P031704	c_2461	cmr|Hypothetical protein [c_2461]
c_2481	-4.02	8.38	-2.01	9.39	0.0	7.38	0.0	0.00003799	0.00087581	A_07_P031773	c_2481	cmr|Hypothetical protein [c_2481]
c_2482	-6.86	8.34	-2.78	9.73	0.1	6.95	0.1	0.00001274	0.00051704	A_07_P031779	c_2482	cmr|Putative outer membrane receptor for iron compound [c_2482]
c_2484	-5.76	11.54	-2.53	12.80	0.0	10.28	0.1	0.00001445	0.00054604	A_07_P031782	c_2484	cmr|Hypothetical protein ybdM [c_2484]
c_2489	-2.44	7.83	-1.29	8.47	0.1	7.18	0.0	0.00065846	0.00586029	A_07_P031799	c_2489	cmr|Putative transferase [c_2489]
c_2495	-2.69	9.58	-1.43	10.30	0.2	8.87	0.1	0.00092727	0.00755215	A_07_P031824	c_2495	cmr|Putative phosphotriesterase-related protein [c_2495]
c_2500	-3.96	8.73	-1.98	9.72	0.1	7.74	0.0	0.00006010	0.00114415	A_07_P031846	c_2500	cmr|Hypothetical protein [c_2500]
c_2501	-2.03	9.02	-1.02	9.53	0.0	8.51	0.1	0.00159129	0.01116952	A_07_P031851	c_2501	cmr|Hypothetical protein [c_2501]
c_2512	+2.87	8.46	+1.52	7.70	0.2	9.22	0.0	0.00040910	0.00418234	A_07_P031888	c_2512	cmr|Transposase [c_2512]
c_2514	+4.10	8.76	+2.04	7.74	0.1	9.78	0.1	0.00004642	0.00097317	A_07_P031900	c_2514	cmr|Hypothetical protein [c_2514]
c_2520	+8.81	10.91	+3.14	9.34	0.1	12.48	0.0	0.00000536	0.00034972	A_07_P031907	c_2520	cmr|Conserved hypothetical protein [c_2520]
c_2523	+2.79	9.92	+1.48	9.18	0.0	10.66	0.1	0.00037336	0.00394177	A_07_P031921	c_2523	cmr|Conserved hypothetical protein [c_2523]
c_2524	+4.85	9.40	+2.28	8.26	0.2	10.53	0.1	0.00011255	0.00169568	A_07_P031927	c_2524	cmr|Hypothetical protein [c_2524]
c_2525	+5.90	9.37	+2.56	8.08	0.3	10.65	0.1	0.00010178	0.00159601	A_07_P031934	c_2525	cmr|Hypothetical protein [c_2525]
c_2528	-2.77	9.06	-1.47	9.80	0.1	8.33	0.1	0.00025108	0.00299696	A_07_P031949	c_2528	cmr|Hypothetical protein [c_2528]
c_2558	+4.71	10.67	+2.24	9.56	0.2	11.79	0.0	0.00008006	0.00135829	A_07_P031994	c_2558	cmr|Mannose-1-phosphate guanylyltransferase [c_2558]
c_2559	+2.24	11.51	+1.16	10.93	0.2	12.09	0.0	0.00242223	0.01522987	A_07_P031999	c_2559	cmr|Hypothetical protein [c_2559]
c_2562	-2.59	12.90	-1.37	13.58	0.0	12.21	0.1	0.00027825	0.00319999	A_07_P032014	c_2562	cmr|Glycosyl transferase [c_2562]
c_2566	-2.35	11.64	-1.24	12.25	0.0	11.02	0.1	0.00046950	0.00463439	A_07_P032030	c_2566	cmr|Hypothetical protein [c_2566]
c_2662	-2.09	8.40	-1.06	8.93	0.1	7.87	0.0	0.00162942	0.01136119	A_07_P032204	c_2662	cmr|Hypothetical protein [c_2662]
c_2670	-2.76	9.38	-1.46	10.11	0.2	8.65	0.1	0.00064418	0.00577889	A_07_P032229	c_2670	cmr|Putative conserved protein [c_2670]
c_2729	-3.42	8.74	-1.77	9.63	0.1	7.86	0.0	0.00009950	0.00156646	A_07_P032291	c_2729	cmr|Hypothetical protein [c_2729]
c_2897	+2.23	7.46	+1.16	6.88	0.2	8.04	0.1	0.00291415	0.01745165	A_07_P032543	c_2897	cmr|Type 1 fimbriae Regulatory protein fimB [c_2897]
c_3030	-2.36	8.21	-1.24	8.83	0.1	7.59	0.0	0.00076650	0.00656218	A_07_P030168	c_3030	cmr|SinI homolog [c_3030]
c_3031	-5.16	8.81	-2.37	9.99	0.0	7.63	0.0	0.00001813	0.00060098	A_07_P030171	c_3031	cmr|SinH homolog [c_3031]
c_3053	-4.60	12.08	-2.20	13.19	0.2	10.98	0.0	0.00006430	0.00118410	A_07_P041244	c_3053	cmr|Protein yfhF [c_3053]
c_3116	-2.49	11.38	-1.32	12.03	0.0	10.72	0.0	0.00032938	0.00361129	A_07_P030232	c_3116	cmr|Ribosomal large subunit pseudouridine synthase D [c_3116]
c_3156	+3.42	8.56	+1.77	7.67	0.1	9.44	0.1	0.00016472	0.00222409	A_07_P030311	c_3156	cmr|Putative tail fiber component K of prophage [c_3156]
c_3158	+8.14	8.55	+3.02	7.04	0.1	10.06	0.3	0.00004053	0.00091288	A_07_P030320	c_3158	cmr|Putative tail component of prophage [c_3158]
c_3159	+4.19	10.42	+2.07	9.39	0.5	11.46	0.1	0.00170948	0.01178890	A_07_P030328	c_3159	cmr|Putative tail component of prophage [c_3159]
c_3160	+3.19	9.99	+1.67	9.15	0.4	10.83	0.2	0.00395909	0.02205659	A_07_P030331	c_3160	cmr|Putative tail component of prophage [c_3160]
c_3161	+8.64	8.45	+3.11	6.90	0.2	10.01	0.1	0.00001470	0.00054604	A_07_P030336	c_3161	cmr|Putative tail component of prophage [c_3161]
c_3162	+2.44	10.91	+1.28	10.27	0.1	11.56	0.1	0.00077832	0.00663808	A_07_P030343	c_3162	cmr|Putative tail component of prophage [c_3162]
c_3163	+14.52	8.87	+3.86	6.94	0.2	10.80	0.0	0.00000496	0.00034758	A_07_P030350	c_3163	cmr|Putative tail component of prophage [c_3163]
c_3164	+38.04	9.60	+5.25	6.98	0.1	12.23	0.0	0.00000052	0.00019431	A_07_P030353	c_3164	cmr|Putative tail component of prophage [c_3164]
c_3165	+10.51	9.68	+3.39	7.98	0.6	11.37	0.1	0.00053588	0.00507637	A_07_P030358	c_3165	cmr|Putative tail fiber component Z of prophage [c_3165]
c_3176	+16.58	11.69	+4.05	9.67	0.1	13.72	0.1	0.00000240	0.00027039	A_07_P030411	c_3176	cmr|GnsB protein [c_3176]
c_3214	-2.30	8.42	-1.20	9.02	0.0	7.82	0.3	0.00336946	0.01960412	A_07_P030524	c_3214	cmr|Hypothetical protein [c_3214]
c_3224	-2.00	8.93	-1.00	9.43	0.2	8.43	0.1	0.00396671	0.02208340	A_07_P030537	c_3224	cmr|Conserved hypothetical protein [c_3224]
c_3225	+2.50	8.44	+1.32	7.78	0.0	9.11	0.1	0.00040186	0.00414060	A_07_P030542	c_3225	cmr|Conserved hypothetical protein [c_3225]
c_3252	-5.69	12.08	-2.51	13.33	0.0	10.82	0.2	0.00005271	0.00105307	A_07_P041659	c_3252	cmr|Regulatory protein recX [c_3252]
c_3270	-2.72	8.54	-1.44	9.26	0.0	7.82	0.2	0.00044198	0.00442752	A_07_P030625	c_3270	cmr|Hypothetical protein [c_3270]
c_3273	-3.28	13.19	-1.71	14.05	0.1	12.33	0.1	0.00011158	0.00169030	A_07_P030635	c_3273	cmr|Hypothetical protein ygjM [c_3273]
c_3276	-2.13	8.37	-1.09	8.92	0.0	7.83	0.1	0.00088138	0.00728041	A_07_P030650	c_3276	cmr|Hypothetical protein [c_3276]
c_3305	-2.49	7.85	-1.32	8.51	0.1	7.19	0.2	0.00135308	0.00986703	A_07_P030690	c_3305	cmr|Hypothetical protein [c_3305]
c_3343	-2.29	7.54	-1.20	8.14	0.1	6.94	0.1	0.00112483	0.00867256	A_07_P032631	c_3343	cmr|Hypothetical protein [c_3343]
c_3389	-2.27	8.72	-1.18	9.31	0.2	8.13	0.0	0.00137316	0.00996727	A_07_P032681	c_3389	cmr|Hypothetical protein [c_3389]
c_3394	-4.96	8.22	-2.31	9.37	0.1	7.06	0.2	0.00008874	0.00145375	A_07_P032706	c_3394	cmr|Hypothetical protein [c_3394]
c_3395	-2.55	7.45	-1.35	8.12	0.1	6.77	0.1	0.00037059	0.00392997	A_07_P032713	c_3395	cmr|Hypothetical protein [c_3395]
c_3396	-5.02	8.66	-2.33	9.82	0.0	7.50	0.1	0.00002213	0.00066628	A_07_P032716	c_3396	cmr|Hypothetical protein [c_3396]
c_3397	-2.81	7.75	-1.49	8.49	0.0	7.00	0.0	0.00017137	0.00227395	A_07_P032722	c_3397	cmr|Hypothetical protein [c_3397]
c_3432	-2.37	11.51	-1.25	12.13	0.1	10.88	0.0	0.00072202	0.00626395	A_07_P032816	c_3432	cmr|Transposase [c_3432]
c_3506	+6.42	10.63	+2.68	9.29	0.1	11.97	0.0	0.00001477	0.00054604	A_07_P032900	c_3506	cmr|Hypothetical protein [c_3506]
c_3507	+12.04	9.27	+3.59	7.48	0.3	11.07	0.0	0.00001614	0.00056732	A_07_P032905	c_3507	cmr|Hypothetical protein [c_3507]
c_3508	+4.47	8.86	+2.16	7.78	0.0	9.94	0.3	0.00023586	0.00285424	A_07_P032907	c_3508	cmr|Hypothetical protein [c_3508]
c_3513	-2.56	12.61	-1.36	13.29	0.1	11.93	0.4	0.00629175	0.03135720	A_07_P032930	c_3513	cmr|Putative conserved protein [c_3513]
c_3518	-4.29	8.37	-2.10	9.42	0.1	7.32	0.0	0.00003868	0.00088510	A_07_P032938	c_3518	cmr|Hypothetical protein [c_3518]
c_3523	-2.13	7.12	-1.09	7.67	0.2	6.58	0.0	0.00251977	0.01569898	A_07_P032958	c_3523	cmr|Conserved hypothetical protein [c_3523]
c_3557	-5.29	9.26	-2.40	10.46	0.1	8.06	0.1	0.00002256	0.00067139	A_07_P032997	c_3557	cmr|ShiA homolog [c_3557]
c_3560	-3.91	8.18	-1.97	9.17	0.1	7.20	0.0	0.00008652	0.00143544	A_07_P033008	c_3560	cmr|Unknown protein encoded by ISEc8 within prophage [c_3560]
c_3561	-2.31	10.14	-1.21	10.74	0.0	9.54	0.2	0.00138485	0.01002730	A_07_P033015	c_3561	cmr|Unknown protein encoded by ISEc8 [c_3561]
c_3564	-3.37	9.74	-1.75	10.62	0.4	8.87	0.3	0.00452332	0.02435539	A_07_P033028	c_3564	cmr|Hypothetical protein [c_3564]
c_3565	-5.81	10.16	-2.54	11.43	0.2	8.89	0.0	0.00005330	0.00106045	A_07_P033031	c_3565	cmr|Putative response regulator [c_3565]
c_3575	-2.54	8.45	-1.35	9.13	0.2	7.78	0.2	0.00200891	0.01320585	A_07_P033084	c_3575	cmr|Transposase insF for insertion sequence IS3A/B/C/D/E/fA [c_3575]
c_3576	-3.13	8.77	-1.65	9.59	0.0	7.95	0.1	0.00012782	0.00183942	A_07_P033087	c_3576	cmr|Unknown in IS [c_3576]
c_3579	-5.08	10.15	-2.34	11.32	0.0	8.98	0.1	0.00002584	0.00072358	A_07_P033108	c_3579	cmr|Hypothetical protein [c_3579]
c_3580	-8.44	11.74	-3.08	13.28	0.0	10.20	0.0	0.00000483	0.00034614	A_07_P033102	c_3580	cmr|Hypothetical protein [c_3580]
c_3581	-5.93	11.50	-2.57	12.78	0.0	10.21	0.1	0.00001482	0.00054604	A_07_P033112	c_3581	cmr|Hypothetical protein [c_3581]
c_3594	-10.62	10.94	-3.41	12.64	0.1	9.24	0.1	0.00000494	0.00034758	A_07_P033172	c_3594	cmr|Putative Transposase [c_3594]
c_3595	-6.28	10.87	-2.65	12.19	0.0	9.54	0.2	0.00003731	0.00087155	A_07_P033176	c_3595	cmr|Transposase [c_3595]
c_3596	-7.01	11.56	-2.81	12.96	0.1	10.15	0.0	0.00000789	0.00041039	A_07_P033185	c_3596	cmr|Hypothetical protein in IS [c_3596]
c_3597	-3.53	10.24	-1.82	11.15	0.0	9.33	0.0	0.00006310	0.00116867	A_07_P033190	c_3597	cmr|Transposase [c_3597]
c_3598	-4.29	9.53	-2.10	10.58	0.0	8.48	0.1	0.00004043	0.00091288	A_07_P033191	c_3598	cmr|Hypothetical protein [c_3598]
c_3598	-5.13	9.74	-2.36	10.92	0.1	8.55	0.0	0.00002199	0.00066568	A_07_P033191	c_3598	cmr|Hypothetical protein [c_3598]
c_3598	-4.50	9.73	-2.17	10.82	0.1	8.65	0.1	0.00004518	0.00095882	A_07_P033191	c_3598	cmr|Hypothetical protein [c_3598]
c_3598	-5.48	9.71	-2.45	10.94	0.0	8.49	0.0	0.00001516	0.00055633	A_07_P033191	c_3598	cmr|Hypothetical protein [c_3598]
c_3598	-5.10	9.64	-2.35	10.81	0.1	8.46	0.1	0.00003235	0.00079881	A_07_P033191	c_3598	cmr|Hypothetical protein [c_3598]
c_3600	-2.14	10.09	-1.09	10.64	0.0	9.54	0.2	0.00289411	0.01739116	A_07_P033203	c_3600	cmr|Hypothetical protein [c_3600]
c_3601	-11.66	9.48	-3.54	11.25	0.1	7.71	0.2	0.00001349	0.00052666	A_07_P033207	c_3601	cmr|Hypothetical protein [c_3601]
c_3602	-3.87	11.97	-1.95	12.94	0.2	10.99	0.1	0.00019646	0.00250894	A_07_P033212	c_3602	cmr|Hypothetical protein [c_3602]
c_3603	-2.07	10.41	-1.05	10.93	0.1	9.88	0.1	0.00153880	0.01091792	A_07_P033216	c_3603	cmr|Hypothetical protein [c_3603]
c_3604	-2.24	8.37	-1.16	8.95	0.0	7.78	0.1	0.00063614	0.00571983	A_07_P033222	c_3604	cmr|Hypothetical protein [c_3604]
c_3605	+2.26	10.61	+1.18	10.02	0.1	11.20	0.1	0.00101482	0.00801272	A_07_P033227	c_3605	cmr|Hypothetical protein [c_3605]
c_3606	+3.89	9.71	+1.96	8.73	0.1	10.69	0.1	0.00005903	0.00112656	A_07_P033231	c_3606	cmr|Hypothetical protein [c_3606]
c_3609	-2.28	8.94	-1.19	9.53	0.1	8.35	0.4	0.00942330	0.04268139	A_07_P033238	c_3609	cmr|Hypothetical protein [c_3609]
c_3612	-2.47	10.91	-1.30	11.56	0.2	10.26	0.1	0.00087203	0.00723355	A_07_P033247	c_3612	cmr|Transposase insC for insertion element IS2A/D/F/H/I/K [c_3612]
c_3614	-2.96	9.50	-1.56	10.28	0.1	8.71	0.1	0.00035151	0.00378015	A_07_P033257	c_3614	cmr|Hypothetical protein [c_3614]
c_3615	-2.14	8.86	-1.10	9.41	0.0	8.32	0.0	0.00075730	0.00650463	A_07_P033261	c_3615	cmr|Unknown in ISEc8 [c_3615]
c_3616	-3.93	10.71	-1.97	11.70	0.1	9.72	0.0	0.00008572	0.00143229	A_07_P033266	c_3616	cmr|Unknown in ISEc8 [c_3616]
c_3617	-2.68	8.28	-1.42	8.99	0.1	7.57	0.0	0.00029748	0.00335322	A_07_P036281	c_3617	cmr|Unknown in putative ISEc8 [c_3617]
c_3618	-10.11	9.52	-3.34	11.19	0.1	7.85	0.0	0.00000380	0.00031465	A_07_P036289	c_3618	cmr|Hypothetical protein [c_3618]
c_3618	-10.73	9.57	-3.42	11.29	0.1	7.86	0.0	0.00000443	0.00033535	A_07_P036289	c_3618	cmr|Hypothetical protein [c_3618]
c_3618	-9.75	9.21	-3.29	10.86	0.1	7.57	0.1	0.00000518	0.00034758	A_07_P036289	c_3618	cmr|Hypothetical protein [c_3618]
c_3618	-11.17	9.63	-3.48	11.37	0.1	7.89	0.1	0.00000339	0.00029832	A_07_P036289	c_3618	cmr|Hypothetical protein [c_3618]
c_3618	-9.02	9.27	-3.17	10.86	0.0	7.68	0.1	0.00000730	0.00040349	A_07_P036289	c_3618	cmr|Hypothetical protein [c_3618]
c_3620	-5.09	10.78	-2.35	11.95	0.1	9.60	0.0	0.00001976	0.00062719	A_07_P036293	c_3620	cmr|Hypothetical protein [c_3620]
c_3630	-3.84	9.38	-1.94	10.35	0.1	8.41	0.1	0.00006362	0.00117563	A_07_P036335	c_3630	cmr|Hypothetical protein yjhS precursor [c_3630]
c_3631	-3.84	9.28	-1.94	10.25	0.1	8.31	0.2	0.00018493	0.00240029	A_07_P036340	c_3631	cmr|Hypothetical protein [c_3631]
c_3639	+3.48	9.02	+1.80	8.12	0.0	9.92	0.1	0.00008579	0.00143229	A_07_P036375	c_3639	cmr|N-acetylneuraminate lyase subunit [c_3639]
c_3641	-3.06	9.58	-1.61	10.39	0.2	8.78	0.2	0.00086129	0.00716707	A_07_P036385	c_3641	cmr|Unknown in ISEc8 [c_3641]
c_3643	-2.08	10.95	-1.05	11.48	0.0	10.42	0.1	0.00105307	0.00824036	A_07_P036391	c_3643	cmr|Unknown in ISEc8 [c_3643]
c_3646	-3.43	12.32	-1.78	13.21	0.3	11.44	0.3	0.00269198	0.01651697	A_07_P036409	c_3646	cmr|Hypothetical protein [c_3646]
c_3652	-3.36	9.17	-1.75	10.04	0.1	8.29	0.1	0.00013841	0.00196138	A_07_P036440	c_3652	cmr|Hypothetical protein yfjI [c_3652]
c_3654	-4.04	8.12	-2.01	9.13	0.0	7.11	0.2	0.00009699	0.00154244	A_07_P036449	c_3654	cmr|Hypothetical protein yeeP [c_3654]
c_3655	+3.74	11.21	+1.90	10.26	0.1	12.17	0.1	0.00012776	0.00183942	A_07_P036451	c_3655	cmr|Antigen 43 precursor [c_3655]
c_3658	-34.46	14.06	-5.11	16.62	0.0	11.51	0.0	0.00000041	0.00019431	A_07_P036466	c_3658	cmr|Hypothetical protein [c_3658]
c_3659	-35.89	9.92	-5.17	12.50	0.0	7.34	0.0	0.00000036	0.00019431	A_07_P036474	c_3659	cmr|Unknown protein encoded by ISEc8 [c_3659]
c_3660	-28.57	10.11	-4.84	12.53	0.0	7.70	0.0	0.00000048	0.00019431	A_07_P036480	c_3660	cmr|Unknown protein encoded by ISEc8 [c_3660]
c_3662	-4.74	11.21	-2.25	12.33	0.1	10.09	0.2	0.00006188	0.00115851	A_07_P036488	c_3662	cmr|Unknown protein encoded by ISEc8 [c_3662]
c_3663	-5.94	8.53	-2.57	9.82	0.1	7.24	0.1	0.00001796	0.00059912	A_07_P036493	c_3663	cmr|Hypothetical protein [c_3663]
c_3666	-2.65	7.97	-1.41	8.68	0.0	7.27	0.1	0.00029775	0.00335322	A_07_P036509	c_3666	cmr|Hypothetical protein ykfF [c_3666]
c_3670	-3.07	9.16	-1.62	9.97	0.0	8.35	0.2	0.00048546	0.00472965	A_07_P036530	c_3670	cmr|Hypothetical protein [c_3670]
c_3681	-13.24	11.82	-3.73	13.68	0.0	9.96	0.0	0.00000185	0.00025064	A_07_P036583	c_3681	cmr|Hypothetical protein [c_3681]
c_3682	-18.88	10.66	-4.24	12.78	0.0	8.55	0.0	0.00000094	0.00019431	A_07_P036586	c_3682	cmr|Hypothetical protein [c_3682]
c_3683	-61.63	11.89	-5.95	14.86	0.0	8.92	0.1	0.00000021	0.00019431	A_07_P036593	c_3683	cmr|Hypothetical protein [c_3683]
c_3686	+8.10	10.19	+3.02	8.68	0.1	11.70	0.0	0.00001013	0.00046432	A_07_P036604	c_3686	cmr|Hypothetical protein yrbH [c_3686]
c_3689	+3.73	8.98	+1.90	8.04	0.1	9.93	0.1	0.00011009	0.00167356	A_07_P036619	c_3689	cmr|3-deoxy-manno-octulosonate cytidylyltransferase [c_3689]
c_3693	+4.90	9.28	+2.29	8.14	0.2	10.43	0.1	0.00011787	0.00175230	A_07_P036636	c_3693	cmr|Hypothetical protein [c_3693]
c_3693	+4.32	9.71	+2.11	8.66	0.3	10.77	0.1	0.00030719	0.00342042	A_07_P036636	c_3693	cmr|Hypothetical protein [c_3693]
c_3693	+4.88	9.56	+2.29	8.42	0.0	10.71	0.0	0.00002021	0.00063193	A_07_P036636	c_3693	cmr|Hypothetical protein [c_3693]
c_3693	+4.65	9.52	+2.22	8.41	0.1	10.62	0.0	0.00002868	0.00075016	A_07_P036636	c_3693	cmr|Hypothetical protein [c_3693]
c_3693	+5.01	9.60	+2.32	8.44	0.1	10.76	0.0	0.00003479	0.00083884	A_07_P036636	c_3693	cmr|Hypothetical protein [c_3693]
c_3694	+3.30	11.51	+1.72	10.64	0.0	12.37	0.0	0.00008598	0.00143251	A_07_P036641	c_3694	cmr|Hypothetical protein [c_3694]
c_3695	+5.36	10.16	+2.42	8.95	0.2	11.37	0.2	0.00020928	0.00262336	A_07_P036649	c_3695	cmr|Hypothetical protein [c_3695]
c_3774	-2.31	7.90	-1.21	8.51	0.0	7.30	0.0	0.00050885	0.00488792	A_07_P036820	c_3774	cmr|Ferric enterobactin transport ATP-binding protein fepC [c_3774]
c_3819	-5.87	15.03	-2.55	16.30	0.0	13.75	0.1	0.00001309	0.00052207	A_07_P036906	c_3819	cmr|Putative conserved protein [c_3819]
c_3873	+17.65	9.40	+4.14	7.33	0.1	11.47	0.0	0.00000124	0.00021441	A_07_P034083	c_3873	cmr|Putative conserved protein [c_3873]
c_3891	+2.89	8.19	+1.53	7.43	0.3	8.96	0.3	0.00358837	0.02058117	A_07_P034105	c_3891	cmr|Putative phosphotransferase system enzyme subunit [c_3891]
c_3928	+2.00	7.40	+1.00	6.90	0.0	7.90	0.1	0.00124156	0.00928541	A_07_P034139	c_3928	cmr|Hypothetical protein [c_3928]
c_3976	+5.37	8.19	+2.42	6.97	0.1	9.40	0.1	0.00002164	0.00065900	A_07_P041300	c_3976	cmr|Hypothetical protein yhcI [c_3976]
c_4013	-2.73	11.16	-1.45	11.88	0.0	10.43	0.1	0.00023555	0.00285318	A_07_P034204	c_4013	cmr|Hypothetical protein [c_4013]
c_4014	-7.61	8.76	-2.93	10.22	0.1	7.29	0.1	0.00001144	0.00049545	A_07_P034207	c_4014	cmr|Hypothetical protein [c_4014]
c_4016	-6.26	8.56	-2.65	9.88	0.0	7.23	0.2	0.00003128	0.00078813	A_07_P034216	c_4016	cmr|Ribose transport ATP-binding protein rbsA [c_4016]
c_4053	-2.29	11.28	-1.19	11.88	0.0	10.68	0.1	0.00067564	0.00595930	A_07_P041813	c_4053	cmr|Zn [c_4053]
c_4059	+3.33	7.70	+1.74	6.83	0.3	8.56	0.0	0.00061757	0.00560084	A_07_P034273	c_4059	cmr|Hypothetical protein [c_4059]
c_4067	+2.16	7.32	+1.11	6.76	0.2	7.87	0.1	0.00288845	0.01737417	A_07_P034282	c_4067	cmr|Hypothetical protein [c_4067]
c_4084	+2.95	7.76	+1.56	6.98	0.0	8.54	0.1	0.00019787	0.00252367	A_07_P034310	c_4084	cmr|Hypothetical protein [c_4084]
c_4086	+2.06	7.52	+1.04	7.00	0.0	8.04	0.0	0.00094782	0.00763687	A_07_P034312	c_4086	cmr|Hypothetical protein [c_4086]
c_4209	-2.19	10.23	-1.13	10.80	0.0	9.67	0.1	0.00122602	0.00920482	A_07_P034472	c_4209	cmr|Putative minor fimbrial subunit precursor [c_4209]
c_4277	+2.03	7.78	+1.02	7.27	0.3	8.30	0.0	0.00873074	0.04038097	A_07_P034581	c_4277	cmr|Putative phosphotransferase system enzyme subunit [c_4277]
c_4278	+3.07	8.69	+1.62	7.88	0.3	9.50	0.0	0.00101889	0.00803271	A_07_P034587	c_4278	cmr|Putative phosphotransferase system enzyme subunit [c_4278]
c_4360	-2.60	9.48	-1.38	10.16	0.1	8.79	0.0	0.00029643	0.00335277	A_07_P039592	c_4360	cmr|Hypothetical protein [c_4360]
c_4422	-2.48	12.32	-1.31	12.98	0.2	11.67	0.0	0.00068642	0.00602876	A_07_P039723	c_4422	cmr|Hypothetical protein [c_4422]
c_4437	+2.74	7.49	+1.45	6.76	0.0	8.22	0.2	0.00056391	0.00527744	A_07_P039756	c_4437	cmr|Hypothetical protein [c_4437]
c_4437	+2.59	7.67	+1.38	6.98	0.1	8.36	0.1	0.00041837	0.00425500	A_07_P039756	c_4437	cmr|Hypothetical protein [c_4437]
c_4437	+3.24	7.83	+1.70	6.98	0.1	8.68	0.0	0.00010729	0.00164539	A_07_P039756	c_4437	cmr|Hypothetical protein [c_4437]
c_4437	+2.74	7.68	+1.45	6.95	0.1	8.41	0.2	0.00071650	0.00622895	A_07_P039756	c_4437	cmr|Hypothetical protein [c_4437]
c_4437	+2.56	7.81	+1.36	7.13	0.2	8.49	0.2	0.00158342	0.01112418	A_07_P039756	c_4437	cmr|Hypothetical protein [c_4437]
c_4481	-2.11	10.36	-1.08	10.89	0.1	9.82	0.1	0.00130882	0.00968327	A_07_P039839	c_4481	cmr|Conserved hypothetical protein [c_4481]
c_4487	+2.05	7.67	+1.04	7.16	0.1	8.19	0.0	0.00132913	0.00973744	A_07_P039870	c_4487	cmr|Putative phosphotransferase system [c_4487]
c_4493	-2.38	8.41	-1.25	9.03	0.0	7.78	0.1	0.00050563	0.00487485	A_07_P039886	c_4493	cmr|Hypothetical protein [c_4493]
c_4523	-3.81	8.40	-1.93	9.36	0.2	7.43	0.0	0.00017581	0.00231945	A_07_P040008	c_4523	cmr|Conserved hypothetical protein [c_4523]
c_4528	-2.09	7.70	-1.06	8.24	0.1	7.17	0.0	0.00096407	0.00772037	A_07_P040032	c_4528	cmr|Ethanolamine utilization protein eutN [c_4528]
c_4548	-2.62	9.33	-1.39	10.02	0.2	8.63	0.0	0.00110096	0.00853027	A_07_P040129	c_4548	cmr|Hypothetical protein [c_4548]
c_4576	+2.35	8.02	+1.23	7.40	0.0	8.64	0.1	0.00073327	0.00633274	A_07_P038942	c_4576	cmr|Hypothetical protein yeeV [c_4576]
c_4580	-3.52	9.00	-1.81	9.91	0.2	8.10	0.1	0.00032200	0.00355257	A_07_P038963	c_4580	cmr|Hypothetical protein [c_4580]
c_4762	-2.62	7.97	-1.39	8.67	0.2	7.28	0.0	0.00055343	0.00520817	A_07_P039201	c_4762	cmr|Putative permease [c_4762]
c_4776	+2.53	8.41	+1.34	7.74	0.3	9.08	0.0	0.00189005	0.01267793	A_07_P039258	c_4776	cmr|Hypothetical protein [c_4776]
c_4777	+3.53	7.85	+1.82	6.94	0.0	8.76	0.2	0.00017666	0.00232751	A_07_P039263	c_4777	cmr|Putative conserved protein [c_4777]
c_4778	+2.45	8.07	+1.29	7.43	0.1	8.72	0.1	0.00068658	0.00602876	A_07_P039269	c_4778	cmr|Putative conserved protein [c_4778]
c_4779	+3.19	8.35	+1.67	7.51	0.1	9.18	0.2	0.00029411	0.00333849	A_07_P039274	c_4779	cmr|Putative transcriptional regulator [c_4779]
c_4780	+2.42	12.64	+1.27	12.00	0.0	13.27	0.0	0.00037301	0.00394177	A_07_P039276	c_4780	cmr|Hypothetical protein [c_4780]
c_4836	+5.75	11.34	+2.52	10.08	0.0	12.60	0.1	0.00001834	0.00060558	A_07_P039365	c_4836	cmr|Putative lipase [c_4836]
c_4837	-21.77	12.52	-4.44	14.74	0.0	10.29	0.0	0.00000072	0.00019431	A_07_P039369	c_4837	cmr|Hypothetical protein [c_4837]
c_4838	-4.67	12.24	-2.22	13.35	0.1	11.13	0.2	0.00006854	0.00122908	A_07_P039373	c_4838	cmr|Hypothetical protein [c_4838]
c_4839	-19.72	11.40	-4.30	13.55	0.1	9.25	0.0	0.00000122	0.00021323	A_07_P039376	c_4839	cmr|Hypothetical protein [c_4839]
c_4858	+2.42	7.30	+1.28	6.66	0.0	7.94	0.0	0.00036284	0.00387759	A_07_P039424	c_4858	cmr|Hypothetical protein [c_4858]
c_4924	-3.37	7.82	-1.75	8.70	0.0	6.95	0.0	0.00007425	0.00129689	A_07_P039536	c_4924	cmr|Putative hippuricase [c_4924]
c_4925	-2.92	8.28	-1.54	9.05	0.1	7.50	0.1	0.00027246	0.00315418	A_07_P039541	c_4925	cmr|Putative citrate permease [c_4925]
c_4928	-2.84	8.92	-1.51	9.67	0.1	8.17	0.0	0.00020983	0.00262814	A_07_P037396	c_4928	cmr|tRNA [c_4928]
c_4938	+3.12	7.30	+1.64	6.48	0.0	8.12	0.0	0.00011823	0.00175446	A_07_P037425	c_4938	cmr|Hypothetical protein [c_4938]
c_4942	+3.63	7.79	+1.86	6.86	0.2	8.72	0.5	0.00411103	0.02272614	A_07_P037427	c_4942	cmr|Hypothetical protein [c_4942]
c_4953	+2.30	9.54	+1.20	8.94	0.2	10.14	0.0	0.00117774	0.00896134	A_07_P037440	c_4953	cmr|NADH pyrophosphatase [c_4953]
c_4986	-2.04	7.64	-1.03	8.16	0.1	7.13	0.2	0.00348791	0.02010465	A_07_P037506	c_4986	cmr|D-glucitol-6-phosphate dehydrogenase [c_4986]
c_5012	+2.24	11.21	+1.16	10.63	0.1	11.79	0.0	0.00066812	0.00592275	A_07_P037546	c_5012	cmr|Hypothetical protein [c_5012]
c_5019	-4.70	8.85	-2.23	9.96	0.0	7.73	0.3	0.00027234	0.00315418	A_07_P035565	c_5019	cmr|Hypothetical protein yjbO [c_5019]
c_5033	-2.03	8.33	-1.02	8.84	0.1	7.82	0.0	0.00198592	0.01309861	A_07_P037610	c_5033	cmr|Hypothetical protein [c_5033]
c_5035	+2.43	8.11	+1.28	7.47	0.1	8.75	0.0	0.00075786	0.00650583	A_07_P037619	c_5035	cmr|Putative 2-oxoglutarate dehydrogenase [c_5035]
c_5055	+3.61	8.58	+1.85	7.65	0.0	9.50	0.1	0.00008885	0.00145399	A_07_P037658	c_5055	cmr|Hypothetical protein [c_5055]
c_5058	-2.44	8.98	-1.28	9.62	0.2	8.34	0.1	0.00171514	0.01182274	A_07_P037663	c_5058	cmr|Putative transcriptional regulator [c_5058]
c_5061	-2.12	8.37	-1.08	8.92	0.2	7.83	0.1	0.00461841	0.02473656	A_07_P037671	c_5061	cmr|Hypothetical protein [c_5061]
c_5061	-2.71	8.60	-1.44	9.32	0.1	7.88	0.1	0.00055942	0.00525198	A_07_P037671	c_5061	cmr|Hypothetical protein [c_5061]
c_5062	+4.00	8.52	+2.00	7.52	0.1	9.52	0.1	0.00013732	0.00194847	A_07_P037679	c_5062	cmr|Putative symporter yjcG [c_5062]
c_5088	-2.41	7.61	-1.27	8.24	0.0	6.98	0.2	0.00080939	0.00683275	A_07_P037733	c_5088	cmr|Hypothetical protein [c_5088]
c_5153	-3.78	9.81	-1.92	10.77	0.0	8.85	0.0	0.00004899	0.00100424	A_07_P037845	c_5153	cmr|Hypothetical protein [c_5153]
c_5154	-3.11	9.07	-1.64	9.89	0.1	8.25	0.1	0.00022337	0.00274527	A_07_P037850	c_5154	cmr|Hypothetical protein [c_5154]
c_5166	-4.02	9.52	-2.01	10.52	0.0	8.51	0.4	0.00079857	0.00677409	A_07_P037902	c_5166	cmr|Partial Transposase [c_5166]
c_5207	-2.39	11.48	-1.26	12.11	0.2	10.86	0.1	0.00202867	0.01329867	A_07_P038058	c_5207	cmr|Hypothetical protein [c_5207]
c_5233	-2.33	8.49	-1.22	9.10	0.1	7.88	0.1	0.00062327	0.00563628	A_07_P036933	c_5233	cmr|Hypothetical protein [c_5233]
c_5269	-2.07	7.96	-1.05	8.49	0.0	7.44	0.1	0.00140249	0.01012879	A_07_P036952	c_5269	cmr|Hypothetical protein [c_5269]
c_5293	+3.85	8.38	+1.94	7.41	0.0	9.35	0.2	0.00025929	0.00307016	A_07_P036987	c_5293	cmr|Hypothetical protein [c_5293]
c_5295	-7.09	10.41	-2.83	11.82	0.0	8.99	0.1	0.00001025	0.00046640	A_07_P036994	c_5295	cmr|Hypothetical protein [c_5295]
c_5296	-15.18	12.16	-3.92	14.12	0.0	10.20	0.2	0.00000653	0.00038079	A_07_P037000	c_5296	cmr|Hypothetical protein [c_5296]
c_5297	-3.54	8.96	-1.82	9.87	0.2	8.05	0.2	0.00043753	0.00439592	A_07_P037001	c_5297	cmr|Hypothetical protein [c_5297]
c_5334	+3.86	8.73	+1.95	7.76	0.6	9.71	0.1	0.00364540	0.02076451	A_07_P037056	c_5334	cmr|Hypothetical protein [c_5334]
c_5341	-39.25	12.29	-5.29	14.93	0.1	9.64	0.1	0.00000078	0.00019431	A_07_P042071	c_5341	cmr|Mg [c_5341]
c_5343	-40.65	9.65	-5.35	12.33	0.1	6.98	0.1	0.00000056	0.00019431	A_07_P037072	c_5343	cmr|Hypothetical protein [c_5343]
c_5356	-2.83	7.68	-1.50	8.43	0.1	6.93	0.1	0.00026161	0.00308181	A_07_P037109	c_5356	cmr|Hypothetical protein [c_5356]
c_5375	-2.29	9.81	-1.20	10.41	0.4	9.21	0.1	0.00730859	0.03522085	A_07_P037145	c_5375	cmr|Hypothetical protein [c_5375]
c_5448	-2.37	7.87	-1.24	8.49	0.0	7.25	0.2	0.00134247	0.00981235	A_07_P037329	c_5448	cmr|Hypothetical protein [c_5448]
c_5455	+6.33	9.10	+2.66	7.77	0.1	10.43	0.1	0.00002028	0.00063193	A_07_P037341	c_5455	cmr|Hypothetical protein [c_5455]
caiA	-6.26	12.08	-2.65	13.41	0.0	10.76	0.0	0.00000976	0.00045680	A_07_P010884	b0039	cmr|probable carnitine operon oxidoreductase [b0039]
caiT	+2.00	6.79	+1.00	6.29	0.0	7.28	0.0	0.00110998	0.00858747	A_07_P010887	b0040	cmr|probable carnitine transporter [b0040]
carA	+4.81	8.83	+2.27	7.70	0.2	9.97	0.1	0.00006544	0.00119937	A_07_P010846	b0032	cmr|carbamoyl-phosphate synthetase, glutamine [b0032]
carB	+8.71	10.16	+3.12	8.60	0.2	11.72	0.1	0.00001921	0.00061714	A_07_P010855	b0033	cmr|carbamoyl-phosphate synthase large subunit [b0033]
cbl	-5.16	13.04	-2.37	14.23	0.0	11.86	0.0	0.00001835	0.00060558	A_07_P015271	b1987	cmr|transcriptional regulator cys regulon; accessory regulatory circuit affecting cysM [b1987]
cbpA	+6.40	8.67	+2.68	7.33	0.2	10.01	0.0	0.00003939	0.00090005	A_07_P001235	b1000	cmr|curved DNA-binding protein; functions closely related to DnaJ [b1000]
cbpM	+4.90	8.38	+2.29	7.23	0.0	9.53	0.0	0.00002002	0.00063193	A_07_P001226	b0999	cmr|orf, hypothetical protein [b0999]
cbrA	-3.65	7.76	-1.87	8.70	0.4	6.83	0.1	0.00131909	0.00971976	A_07_P017384	b3690	cmr|orf, hypothetical protein [b3690]
ccmG	-2.28	9.64	-1.19	10.23	0.1	9.04	0.2	0.00145687	0.01044487	A_07_P000765	b2195	cmr|disulfide oxidoreductase [b2195]
cdh	-2.41	8.13	-1.27	8.76	0.1	7.49	0.0	0.00059663	0.00547399	A_07_P019370	b3918	cmr|CDP-diacylglycerol phosphotidylhydrolase [b3918]
cdsA	-2.19	11.26	-1.13	11.83	0.2	10.69	0.0	0.00203345	0.01331170	A_07_P002831	b0175	cmr|CDP-diglyceride synthetase [b0175]
cedA	-4.71	11.18	-2.24	12.30	0.1	10.07	0.0	0.00002592	0.00072358	A_07_P018702	b1731	cmr|orf, hypothetical protein [b1731]
chaC	-2.13	8.03	-1.09	8.58	0.2	7.49	0.1	0.00244928	0.01537539	A_07_P007044	b1218	cmr|cation transport regulator [b1218]
cheZ	-3.02	11.76	-1.60	12.56	0.1	10.96	0.1	0.00026422	0.00309464	A_07_P016084	b1881	cmr|chemotactic response; CheY protein phophatase; antagonist of CheY as switch regulator [b1881]
chpB	-2.08	10.85	-1.05	11.37	0.0	10.32	0.1	0.00159278	0.01117504	A_07_P003680	b4225	cmr|probable growth inhibitor, PemK-like, autoregulated [b4225]
clpA	+3.99	9.95	+1.99	8.95	0.0	10.95	0.1	0.00008816	0.00145271	A_07_P004862	b0882	cmr|ATP-binding component of serine protease [b0882]
clpP	+4.74	12.22	+2.24	11.09	0.1	13.34	0.0	0.00002762	0.00073830	A_07_P021300	b0437	cmr|ATP-dependent proteolytic subunit of clpA-clpP serine protease, heat shock protein F21.5 [b0437]
clpS	+3.66	10.47	+1.87	9.53	0.1	11.40	0.0	0.00009328	0.00149973	A_07_P004856	b0881	cmr|orf, hypothetical protein [b0881]
cmr	-2.31	8.13	-1.21	8.73	0.1	7.53	0.0	0.00056846	0.00529264	A_07_P004661	b0842	cmr|proton motive force efflux pump [b0842]
cmtA	-2.94	11.34	-1.55	12.11	0.4	10.56	0.1	0.00272924	0.01664824	A_07_P011811	b2933	cmr|PTS system, mannitol-specific enzyme II component, cryptic [b2933]
cobS	-3.30	8.77	-1.72	9.63	0.1	7.91	0.2	0.00055408	0.00521111	A_07_P015292	b1992	cmr|cobalamin 5-phosphate synthase [b1992]
cobU	-2.87	9.32	-1.52	10.08	0.0	8.56	0.0	0.00015453	0.00212664	A_07_P015296	b1993	cmr|cobinamide kinase/cobinamide phosphate guanylyltransferase [b1993]
codA	+4.72	8.89	+2.24	7.77	0.0	10.01	0.2	0.00008894	0.00145399	A_07_P008378	b0337	cmr|cytosine deaminase [b0337]
codB	+3.89	7.83	+1.96	6.85	0.0	8.81	0.2	0.00014676	0.00204652	A_07_P031022	c_0455	cmr|Cytosine permease [c_0455]

codB	+2.09	7.73	+1.06	7.19	0.1	8.26	0.1	0.00282372	0.01707992	A_07_P008371	b0336	cmr|cytosine permease/transport [b0336]
cpdA	-2.04	12.73	-1.03	13.24	0.1	12.21	0.1	0.00116605	0.00890744	A_07_P012295	b3032	cmr|regulator of lacZ [b3032]
cpxR	+3.83	10.88	+1.94	9.91	0.0	11.85	0.0	0.00004520	0.00095882	A_07_P019340	b3912	cmr|transcriptional regulator in 2-component system [b3912]
cpxR	+3.48	11.24	+1.80	10.34	0.0	12.14	0.0	0.00006620	0.00120763	A_07_P041979	c_4864	cmr|Transcriptional Regulatory protein cpxR [c_4864]
creD	-3.82	9.12	-1.93	10.09	0.1	8.15	0.0	0.00005717	0.00110431	A_07_P017684	b4400	cmr|tolerance to colicin E2 [b4400]
crp	-2.44	12.94	-1.28	13.59	0.0	12.30	0.0	0.00034200	0.00370834	A_07_P017815	b3357	cmr|cyclic AMP receptor protein [b3357]
crr	+2.65	10.24	+1.41	9.54	0.0	10.94	0.1	0.00031070	0.00345228	A_07_P013145	b2417	cmr|PTS system, glucose-specific IIA component [b2417]
csdA	-3.20	11.28	-1.68	12.11	0.0	10.44	0.1	0.00012054	0.00178030	A_07_P006050	b2810	cmr|orf, hypothetical protein [b2810]
csiD	-3.97	9.02	-1.99	10.02	0.1	8.03	0.0	0.00006667	0.00120840	A_07_P020146	b2659	cmr|orf, hypothetical protein [b2659]
csiE	+3.57	7.93	+1.84	7.01	0.1	8.85	0.0	0.00008296	0.00139835	A_07_P019579	b2535	cmr|orf, hypothetical protein [b2535]
cspB	+2.15	11.49	+1.10	10.94	0.1	12.04	0.0	0.00088915	0.00733692	A_07_P020429	b1557	cmr|cold shock protein; may affect transcription [b1557]
cspC	+3.44	14.09	+1.78	13.20	0.1	14.98	0.0	0.00014268	0.00200567	A_07_P015809	b1823	cmr|cold shock protein [b1823]
cspD	+22.92	10.20	+4.52	7.94	0.1	12.45	0.0	0.00000110	0.00020547	A_07_P004854	b0880	cmr|cold shock protein [b0880]
cspE	+6.54	12.96	+2.71	11.61	0.1	14.32	0.1	0.00001278	0.00051714	A_07_P035444	c_0714	cmr|Cold shock-like protein cspE [c_0714]
cspE	+5.88	12.71	+2.55	11.43	0.1	13.98	0.2	0.00004632	0.00097245	A_07_P009684	b0623	cmr|cold shock protein [b0623]
cstA	+6.30	10.15	+2.66	8.82	0.4	11.48	0.1	0.00026794	0.00311300	A_07_P016748	b0598	cmr|carbon starvation protein [b0598]
cvpA	+6.06	12.90	+2.60	11.60	0.1	14.20	0.0	0.00001564	0.00056283	A_07_P013855	b2313	cmr|membrane protein required for colicin V production [b2313]
cvpA	+5.31	11.02	+2.41	9.82	0.1	12.23	0.0	0.00001947	0.00062241	A_07_P042620	Z3575	cmr|membrane protein required for colicin V production [Z3575]
cvrA	-2.94	12.72	-1.55	13.49	0.2	11.94	0.3	0.00251109	0.01565726	A_07_P006906	b1191	cmr|orf, hypothetical protein [b1191]
cyaY	+2.85	10.47	+1.51	9.72	0.1	11.23	0.0	0.00017144	0.00227395	A_07_P005904	b3807	cmr|orf, hypothetical protein [b3807]
cyaY	+2.38	11.50	+1.25	10.87	0.1	12.12	0.1	0.00069705	0.00609686	A_07_P041940	c_4726	cmr|CyaY protein [c_4726]
cybC	+2.56	11.65	+1.36	10.97	0.2	12.33	0.0	0.00075604	0.00649734	A_07_P037063	c_5335	cmr|Soluble cytochrome b562 precursor [c_5335]
cybC	+3.04	10.51	+1.60	9.71	0.0	11.31	0.1	0.00013737	0.00194847	A_07_P003731	b4236	cmr|cytochrome b [b4236]
cydA	+11.22	11.99	+3.49	10.24	0.0	13.73	0.1	0.00000440	0.00033535	A_07_P010185	b0733	cmr|cytochrome d terminal oxidase, polypeptide subunit I [b0733]
cydB	+8.49	11.65	+3.09	10.11	0.1	13.20	0.0	0.00000519	0.00034758	A_07_P010187	b0734	cmr|cytochrome d terminal oxidase polypeptide subunit II [b0734]
cyoB	+2.95	13.90	+1.56	13.12	0.0	14.68	0.1	0.00015079	0.00208799	A_07_P021268	b0431	cmr|cytochrome o ubiquinol oxidase subunit I [b0431]
cyoC	+2.76	12.71	+1.46	11.98	0.0	13.44	0.1	0.00021587	0.00268458	A_07_P021261	b0430	cmr|cytochrome o ubiquinol oxidase subunit III [b0430]
cyoD	+3.89	8.69	+1.96	7.71	0.2	9.67	0.0	0.00010062	0.00157948	A_07_P051941	Z0532	cmr|cytochrome o ubiquinol oxidase subunit IV [Z0532]
cyoD	+3.19	11.29	+1.68	10.45	0.1	12.13	0.0	0.00011753	0.00174890	A_07_P021258	b0429	cmr|cytochrome o ubiquinol oxidase subunit IV [b0429]
cyoE	+2.88	10.53	+1.52	9.77	0.0	11.29	0.0	0.00015989	0.00218551	A_07_P021254	b0428	cmr|protoheme IX farnesyltransferase [b0428]
cysA	-3.43	9.29	-1.78	10.18	0.2	8.40	0.1	0.00019199	0.00246351	A_07_P013166	b2422	cmr|ATP-binding component of sulfate permease A protein; chromate resistance [b2422]
cysB	-2.41	9.30	-1.27	9.94	0.0	8.67	0.1	0.00046899	0.00463227	A_07_P014359	b1275	cmr|positive transcriptional regulator for cysteine regulon [b1275]
cysC	-4.19	9.41	-2.07	10.44	0.0	8.37	0.1	0.00004371	0.00094590	A_07_P002196	b2750	cmr|adenosine 5-phosphosulfate kinase [b2750]
cysD	-2.11	9.97	-1.08	10.51	0.1	9.43	0.1	0.00135926	0.00990294	A_07_P002209	b2752	cmr|ATP:sulfurylase [b2752]
cysH	-3.65	10.82	-1.87	11.76	0.0	9.89	0.1	0.00009217	0.00148959	A_07_P002256	b2762	cmr|3-phosphoadenosine 5-phosphosulfate reductase [b2762]
cysI	-5.44	11.51	-2.44	12.74	0.0	10.29	0.1	0.00002647	0.00072866	A_07_P002265	b2763	cmr|sulfite reductase, alpha subunit [b2763]
cysJ	-4.25	11.19	-2.09	12.23	0.0	10.14	0.1	0.00003522	0.00084015	A_07_P032576	c_3323	cmr|Sulfite reductase [c_3323]
cysJ	-2.07	10.05	-1.05	10.58	0.0	9.53	0.1	0.00103769	0.00814024	A_07_P002266	b2764	cmr|sulfite reductase [b2764]
cysM	-2.13	10.19	-1.09	10.74	0.1	9.65	0.1	0.00097747	0.00779599	A_07_P030051	c_2955	cmr|Cysteine synthase B [c_2955]
cysN	-2.88	7.80	-1.53	8.56	0.2	7.04	0.1	0.00039934	0.00412279	A_07_P002205	b2751	cmr|ATP-sulfurylase [b2751]
cysN	-4.75	9.11	-2.25	10.24	0.1	7.99	0.1	0.00003576	0.00084433	A_07_P043152	Z4059	cmr|ATP-sulfurylase [Z4059]
cysN	-5.14	9.64	-2.36	10.83	0.1	8.46	0.1	0.00003792	0.00087581	A_07_P030710	c_3318	cmr|Sulfate adenylyltransferase subunit 1 [c_3318]
cysN	-4.38	9.27	-2.13	10.33	0.0	8.20	0.2	0.00006985	0.00124629	A_07_P056037	ECs3605	cmr|ATP-sulfurylase [ECs3605]
cysP	-4.48	11.61	-2.16	12.70	0.0	10.53	0.2	0.00008108	0.00137285	A_07_P013181	b2425	cmr|thiosulfate binding protein [b2425]
cysP	-5.90	12.48	-2.56	13.77	0.0	11.20	0.1	0.00001351	0.00052666	A_07_P030063	c_2959	cmr|Thiosulfate-binding protein precursor [c_2959]
cysP	-4.54	11.14	-2.18	12.23	0.0	10.04	0.1	0.00004067	0.00091477	A_07_P042814	Z3690	cmr|thiosulfate binding protein [Z3690]
cysU	-3.81	12.00	-1.93	12.96	0.1	11.03	0.0	0.00005893	0.00112656	A_07_P013177	b2424	cmr|sulfate, thiosulfate transport system permease T protein [b2424]
cysU	-4.62	11.09	-2.21	12.19	0.0	9.98	0.2	0.00006160	0.00115828	A_07_P042582	c_2958	cmr|Sulfate transport system permease protein cysT [c_2958]
cysW	-12.38	10.92	-3.63	12.73	0.0	9.10	0.1	0.00000230	0.00026836	A_07_P013174	b2423	cmr|sulfate transport system permease W protein [b2423]
dadA	+10.10	9.98	+3.34	8.31	0.0	11.65	0.0	0.00000300	0.00028496	A_07_P006899	b1189	cmr|D-amino acid dehydrogenase subunit [b1189]
dadA	+8.46	10.12	+3.08	8.58	0.1	11.66	0.1	0.00001174	0.00050228	A_07_P038722	c_1638	cmr|D-amino acid dehydrogenase small subunit [c_1638]
dadX	+5.04	9.37	+2.33	8.21	0.0	10.54	0.0	0.00001857	0.00061157	A_07_P006905	b1190	cmr|alanine racemase 2, catabolic [b1190]
damX	-2.31	10.91	-1.21	11.52	0.0	10.31	0.1	0.00056994	0.00530327	A_07_P017966	b3388	cmr|putative membrane protein; interferes with cell division [b3388]
dapA	+2.74	11.49	+1.45	10.76	0.0	12.21	0.1	0.00023797	0.00287537	A_07_P013447	b2478	cmr|dihydrodipicolinate synthase [b2478]
dapB	+3.64	9.33	+1.86	8.39	0.0	10.26	0.1	0.00006743	0.00121740	A_07_P010842	b0031	cmr|dihydrodipicolinate reductase [b0031]
dapD	+2.78	8.62	+1.48	7.89	0.0	9.36	0.1	0.00024466	0.00293818	A_07_P041442	c_0201	cmr|2,3,4,5-tetrahydropyridine-2,6-dicarboxylate N-succinyltransferase [c_0201]
dapE	-4.24	10.91	-2.09	11.95	0.1	9.87	0.0	0.00003602	0.00084763	A_07_P013420	b2472	cmr|N-succinyl-diaminopimelate deacylase [b2472]
dcd	-3.54	12.31	-1.82	13.22	0.0	11.39	0.0	0.00006817	0.00122531	A_07_P003102	b2065	cmr|2-deoxycytidine 5-triphosphate deaminase [b2065]
dctA	+17.35	10.51	+4.12	8.46	0.1	12.57	0.0	0.00000152	0.00022581	A_07_P007574	b3528	cmr|uptake of C4-dicarboxylic acids [b3528]
dcuA	+3.24	10.23	+1.69	9.38	0.1	11.08	0.1	0.00014143	0.00199367	A_07_P011423	b4138	cmr|anaerobic dicarboxylate transport [b4138]
dcuB	-2.56	8.28	-1.36	8.96	0.0	7.61	0.2	0.00102756	0.00808088	A_07_P011354	b4123	cmr|anaerobic dicarboxylate transport [b4123]
dcuC	+2.11	8.05	+1.08	7.52	0.0	8.59	0.1	0.00087549	0.00725841	A_07_P009671	b0621	cmr|transport of dicarboxylates [b0621]
dcyD	-6.15	11.41	-2.62	12.72	0.0	10.10	0.0	0.00001054	0.00047145	A_07_P014958	b1919	cmr|putative 1-aminocyclopropane-1-carboxylate deaminase [b1919]
ddl	-2.94	11.59	-1.56	12.37	0.0	10.81	0.0	0.00014337	0.00201178	A_07_P062950	ECs0431	cmr|D-alanine-D-alanine ligase A [ECs0431]
ddl	-2.66	11.53	-1.41	12.24	0.0	10.83	0.1	0.00040431	0.00415498	A_07_P051920	Z0477	cmr|putative enzyme Cell envelop: Murein sacculus, peptidoglycan [Z0477]
ddlA	-4.06	9.81	-2.02	10.82	0.1	8.80	0.0	0.00005623	0.00109182	A_07_P021019	b0381	cmr|D-alanine-D-alanine ligase A [b0381]
deaD	-14.08	11.48	-3.82	13.39	0.0	9.57	0.1	0.00000182	0.00025064	A_07_P000534	b3162	cmr|inducible ATP-independent RNA helicase [b3162]
degP	-2.10	11.14	-1.07	11.68	0.0	10.61	0.0	0.00078568	0.00668277	A_07_P002766	b0161	cmr|periplasmic serine protease Do; heat shock protein HtrA [b0161]
degQ	+3.81	8.77	+1.93	7.80	0.0	9.73	0.1	0.00006245	0.00116220	A_07_P041306	c_3989	cmr|Protease degQ precursor [c_3989]
degS	-3.60	9.39	-1.85	10.31	0.1	8.47	0.0	0.00006994	0.00124629	A_07_P034177	c_3990	cmr|Protease degS precursor [c_3990]
deoB	+4.53	11.00	+2.18	9.91	0.1	12.09	0.0	0.00003022	0.00077353	A_07_P017598	b4383	cmr|phosphopentomutase [b4383]
deoC	-6.14	14.49	-2.62	15.80	0.0	13.18	0.0	0.00001067	0.00047201	A_07_P017589	b4381	cmr|2-deoxyribose-5-phosphate aldolase [b4381]
deoD	+2.15	11.15	+1.10	10.60	0.0	11.71	0.1	0.00080621	0.00682418	A_07_P017603	b4384	cmr|purine-nucleoside phosphorylase [b4384]
deoR	-8.03	10.23	-3.01	11.74	0.1	8.73	0.3	0.00006115	0.00115710	A_07_P042124	c_0925	cmr|Deoxyribose operon repressor [c_0925]
deoR	-11.73	10.57	-3.55	12.34	0.1	8.79	0.1	0.00000311	0.00028944	A_07_P004651	b0840	cmr|transcriptional repressor for deo operon, tsx, nupG [b0840]
dhaK	+3.89	8.89	+1.96	7.91	0.1	9.87	0.0	0.00007676	0.00132177	A_07_P006953	b1200	cmr|putative dihydroxyacetone kinase [b1200]
dhaL	+2.72	7.70	+1.45	6.98	0.1	8.43	0.1	0.00047985	0.00468366	A_07_P006948	b1199	cmr|putative dihydroxyacetone kinase [b1199]
diaA	+2.61	9.41	+1.38	8.72	0.1	10.10	0.1	0.00042591	0.00430780	A_07_P000466	b3149	cmr|orf, hypothetical protein [b3149]
dinB	-3.37	12.07	-1.75	12.95	0.1	11.19	0.0	0.00012234	0.00179502	A_07_P007867	b0231	cmr|damage-inducible protein P; putative tRNA synthetase [b0231]
dinF	-3.12	10.11	-1.64	10.93	0.0	9.29	0.0	0.00010654	0.00164437	A_07_P008863	b4044	cmr|DNA-damage-inducible protein F [b4044]
dinG	+4.36	11.26	+2.12	10.20	0.1	12.33	0.1	0.00006253	0.00116220	A_07_P004448	b0799	cmr|probably ATP-dependent helicase [b0799]
dinI	-2.31	14.34	-1.21	14.94	0.0	13.73	0.1	0.00049084	0.00476059	A_07_P001507	b1061	cmr|damage-inducible protein I [b1061]
djlB	-3.83	11.81	-1.94	12.78	0.1	10.84	0.0	0.00006046	0.00114826	A_07_P009800	b0646	cmr|putative enzyme of polynucleotide modification [b0646]
dkgA	+2.85	8.75	+1.51	7.99	0.0	9.50	0.0	0.00015105	0.00208974	A_07_P012193	b3012	cmr|orf, hypothetical protein [b3012]
dkgB	-3.84	8.94	-1.94	9.91	0.1	7.97	0.1	0.00008853	0.00145271	A_07_P002965	b0207	cmr|putative aldose reductase [b0207]
dmsA	+5.26	10.01	+2.39	8.81	0.2	11.21	0.0	0.00004400	0.00094686	A_07_P033816	c_1031	cmr|Anaerobic dimethyl sulfoxide reductase chain A precursor [c_1031]
dmsA	+4.75	8.27	+2.25	7.15	0.1	9.39	0.1	0.00004053	0.00091288	A_07_P004916	b0894	cmr|anaerobic dimethyl sulfoxide reductase subunit A [b0894]
dmsB	+3.71	8.43	+1.89	7.49	0.1	9.38	0.1	0.00009178	0.00148821	A_07_P004924	b0895	cmr|anaerobic dimethyl sulfoxide reductase subunit B [b0895]
dmsC	+2.38	7.78	+1.25	7.15	0.1	8.40	0.1	0.00059996	0.00549480	A_07_P052033	Z1242	cmr|anaerobic dimethyl sulfoxide reductase subunit C [Z1242]
dmsC	+2.88	8.13	+1.52	7.37	0.0	8.90	0.0	0.00016021	0.00218551	A_07_P004926	b0896	cmr|anaerobic dimethyl sulfoxide reductase subunit C [b0896]
dnaA	-2.07	14.01	-1.05	14.54	0.0	13.49	0.1	0.00109608	0.00849666	A_07_P005441	b3702	cmr|DNA biosynthesis; initiation of chromosome replication; can be transcription regulator [b3702]
dnaB	-2.22	9.30	-1.15	9.88	0.0	8.73	0.0	0.00054978	0.00518615	A_07_P008903	b4052	cmr|replicative DNA helicase; part of primosome [b4052]
dnaC	+2.00	11.91	+1.00	11.41	0.1	12.41	0.1	0.00210341	0.01370701	A_07_P017504	b4361	cmr|chromosome replication; initiation and chain elongation [b4361]
dnaG	-5.32	11.70	-2.41	12.91	0.0	10.49	0.0	0.00001587	0.00056388	A_07_P000065	b3066	cmr|DNA biosynthesis; DNA primase [b3066]
dnaN	-3.06	13.24	-1.61	14.05	0.0	12.44	0.1	0.00012751	0.00183834	A_07_P005436	b3701	cmr|DNA polymerase III, beta-subunit [b3701]
dnaQ	+3.07	9.18	+1.62	8.37	0.0	9.99	0.0	0.00011279	0.00169761	A_07_P051886	Z0241	cmr|DNA polymerase III, epsilon subunit [Z0241]
dnaQ	+3.08	9.80	+1.62	8.99	0.1	10.61	0.0	0.00023399	0.00284423	A_07_P003004	b0215	cmr|DNA polymerase III, epsilon subunit [b0215]
dnaX	+6.83	11.14	+2.77	9.76	0.1	12.53	0.0	0.00000887	0.00042982	A_07_P021457	b0470	cmr|DNA polymerase III, tau and gamma subunits; DNA elongation factor III [b0470]
dps	+4.24	11.69	+2.08	10.64	0.1	12.73	0.1	0.00008620	0.00143428	A_07_P004515	b0812	cmr|global regulator, starvation conditions [b0812]
dsbA	+4.11	9.47	+2.04	8.45	0.1	10.49	0.0	0.00004476	0.00095355	A_07_P019081	b3860	cmr|protein disulfide isomerase I, essential for cytochrome c synthesis and formate-dependent reduction [b3860]
dsdX	+2.38	7.57	+1.25	6.95	0.3	8.20	0.1	0.00335565	0.01955992	A_07_P014107	b2365	cmr|transport system permease [b2365]
dtd	+3.00	9.81	+1.58	9.02	0.3	10.60	0.1	0.00136593	0.00992393	A_07_P019212	b3887	cmr|orf, hypothetical protein [b3887]
dusB	+3.72	11.89	+1.89	10.94	0.1	12.83	0.1	0.00008195	0.00138289	A_07_P012768	b3260	cmr|putative dehydrogenase [b3260]
E1A_r60_a104	-2.21	9.95	-1.14	10.52	0.1	9.37	0.3	0.00581046	0.02964352	(+)E1A_r60_a104	E1A_r60_a104	
E1A_r60_a104	-2.26	10.00	-1.18	10.59	0.0	9.41	0.1	0.00064411	0.00577889	(+)E1A_r60_a104	E1A_r60_a104	
E1A_r60_a104	-3.20	10.17	-1.68	11.01	0.3	9.33	0.0	0.00057391	0.00533670	(+)E1A_r60_a104	E1A_r60_a104	
E1A_r60_a104	-2.23	9.74	-1.15	10.32	0.1	9.16	0.1	0.00116791	0.00891302	(+)E1A_r60_a104	E1A_r60_a104	
E1A_r60_a104	-2.13	10.03	-1.09	10.57	0.1	9.48	0.2	0.00278233	0.01690661	(+)E1A_r60_a104	E1A_r60_a104	
E1A_r60_a104	-2.16	10.14	-1.11	10.70	0.3	9.59	0.1	0.00582787	0.02964589	(+)E1A_r60_a104	E1A_r60_a104	
E1A_r60_a107	-2.94	9.73	-1.56	10.51	0.3	8.96	0.3	0.00402784	0.02229975	(+)E1A_r60_a107	E1A_r60_a107	
E1A_r60_a107	-2.15	9.12	-1.10	9.68	0.1	8.57	0.0	0.00104769	0.00821045	(+)E1A_r60_a107	E1A_r60_a107	
E1A_r60_a135	-2.47	9.56	-1.30	10.21	0.3	8.91	0.2	0.00365353	0.02080082	(+)E1A_r60_a135	E1A_r60_a135	
E1A_r60_a20	-2.43	10.25	-1.28	10.89	0.1	9.61	0.0	0.00041082	0.00419719	(+)E1A_r60_a20	E1A_r60_a20	
E1A_r60_a20	-2.09	10.11	-1.06	10.64	0.3	9.58	0.2	0.00685332	0.03344661	(+)E1A_r60_a20	E1A_r60_a20	
E1A_r60_a20	-2.03	10.31	-1.02	10.82	0.3	9.80	0.1	0.00768295	0.03662136	(+)E1A_r60_a20	E1A_r60_a20	
E1A_r60_a20	-2.16	10.32	-1.11	10.88	0.0	9.77	0.1	0.00136401	0.00991481	(+)E1A_r60_a20	E1A_r60_a20	
E1A_r60_a22	-2.02	10.98	-1.02	11.49	0.1	10.47	0.2	0.00458755	0.02460027	(+)E1A_r60_a22	E1A_r60_a22	
E1A_r60_a97	-2.26	8.22	-1.18	8.81	0.2	7.63	0.0	0.00218404	0.01409245	(+)E1A_r60_a97	E1A_r60_a97	
E1A_r60_n11	-3.11	8.19	-1.64	9.01	0.2	7.37	0.1	0.00077007	0.00658197	(+)E1A_r60_n11	E1A_r60_n11	
eaeH	-2.16	12.33	-1.11	12.89	0.1	11.78	0.2	0.00281771	0.01706630	A_07_P008177	b0297	cmr|attaching and effacing protein, pathogenesis factor [b0297]
ebgR	+2.20	9.19	+1.13	8.62	0.3	9.76	0.0	0.00519127	0.02711724	A_07_P041749	c_3832	cmr|Ebg operon repressor [c_3832]
ECs0016	-3.96	12.44	-1.98	13.44	0.0	11.45	0.0	0.00004206	0.00093353	A_07_P058872	ECs0016	cmr|Gef protein [ECs0016]
ECs0031	+2.34	9.98	+1.23	9.37	0.2	10.60	0.2	0.00223052	0.01433372	A_07_P062890	ECs0031	cmr|putative FKBX-type 16KD peptidyl-prolyl cis-trans isomerase [ECs0031]
ECs0052	-2.21	11.93	-1.14	12.50	0.0	11.36	0.0	0.00059481	0.00547004	A_07_P059413	ECs0052	cmr|CcdA-like protein [ECs0052]
ECs0127	-2.06	9.91	-1.04	10.44	0.2	9.39	0.0	0.00230787	0.01468676	A_07_P062900	ECs0127	cmr|hypothetical protein [ECs0127]
ECs0132	-2.41	8.58	-1.27	9.21	0.1	7.94	0.1	0.00092771	0.00755215	A_07_P059452	ECs0132	cmr|hypothetical protein [ECs0132]
ECs0211	+3.12	9.38	+1.64	8.56	0.0	10.20	0.1	0.00012866	0.00184826	A_07_P062916	ECs0211	cmr|DNA polymerase III epsilon subunit [ECs0211]
ECs0278	+3.09	13.44	+1.63	12.63	0.0	14.25	0.0	0.00011209	0.00169030	A_07_P053587	ECs0278	cmr|replication protein [ECs0278]
ECs0309	+2.18	7.73	+1.12	7.17	0.2	8.29	0.1	0.00401692	0.02227627	A_07_P053736	ECs0309	cmr|putative transcriptional regulator [ECs0309]
ECs0325	-2.43	8.29	-1.28	8.93	0.2	7.65	0.3	0.00327560	0.01919557	A_07_P053765	ECs0325	cmr|hypothetical protein [ECs0325]
ECs0330	-3.21	8.74	-1.68	9.58	0.1	7.90	0.1	0.00017284	0.00229052	A_07_P053789	ECs0330	cmr|hypothetical protein [ECs0330]
ECs0332	+3.45	8.46	+1.79	7.57	0.3	9.36	0.1	0.00064963	0.00581122	A_07_P053796	ECs0332	cmr|hypothetical protein [ECs0332]
ECs0357	+2.66	9.14	+1.41	8.43	0.0	9.84	0.0	0.00021437	0.00267014	A_07_P053871	ECs0357	cmr|choline dehydrogenase [ECs0357]
ECs0440	-2.74	7.96	-1.45	8.68	0.2	7.23	0.2	0.00171965	0.01183830	A_07_P054001	ECs0440	cmr|aroM protein [ECs0440]
ECs0449	-5.27	11.65	-2.40	12.85	0.0	10.45	0.1	0.00002686	0.00073391	A_07_P054018	ECs0449	cmr|positive response regulator for pho regulon PhoB [ECs0449]
ECs0483	+3.67	8.77	+1.87	7.83	0.2	9.71	0.0	0.00026118	0.00308044	A_07_P062971	ECs0483	cmr|cytochrome o ubiquinol oxidase subunit IV [ECs0483]
ECs0551	-3.17	10.64	-1.67	11.48	0.1	9.81	0.1	0.00015056	0.00208675	A_07_P054138	ECs0551	cmr|hypothetical protein [ECs0551]
ECs0573	+2.25	7.50	+1.17	6.92	0.0	8.08	0.0	0.00053244	0.00505894	A_07_P054146	ECs0573	cmr|allantoinase [ECs0573]
ECs0679	-2.69	10.30	-1.43	11.01	0.0	9.59	0.2	0.00061403	0.00558100	A_07_P054223	ECs0679	cmr|minor lipoprotein [ECs0679]
ECs0711	-2.16	10.26	-1.11	10.81	0.1	9.70	0.0	0.00140827	0.01016201	A_07_P056574	ECs0711	cmr|hypothetical protein [ECs0711]
ECs0778	-2.10	8.97	-1.07	9.50	0.1	8.43	0.0	0.00090116	0.00739560	A_07_P056671	ECs0778	cmr|quinolinate synthetase A protein [ECs0778]
ECs0794	+2.50	10.08	+1.32	9.42	0.1	10.75	0.0	0.00051120	0.00490750	A_07_P056677	ECs0794	cmr|putative phosphatase [ECs0794]
ECs0805	+164.56	11.31	+7.36	7.63	0.0	14.99	0.1	0.00000013	0.00019431	A_07_P056700	ECs0805	cmr|hypothetical C4-type zinc finger protein TraR-family [ECs0805]
ECs0806	+12.84	11.77	+3.68	9.93	0.3	13.61	0.2	0.00003555	0.00084290	A_07_P056701	ECs0806	cmr|hypothetical protein [ECs0806]
ECs0807	+42.89	10.56	+5.42	7.85	0.0	13.27	0.2	0.00000127	0.00021491	A_07_P056708	ECs0807	cmr|hypothetical protein [ECs0807]
ECs0808	+9.96	12.17	+3.32	10.52	0.2	13.83	0.2	0.00004304	0.00093596	A_07_P056712	ECs0808	cmr|hypothetical protein [ECs0808]
ECs0809	+6.25	13.12	+2.64	11.80	0.1	14.44	0.1	0.00001732	0.00058395	A_07_P056720	ECs0809	cmr|exonuclease [ECs0809]
ECs0810	+27.97	10.56	+4.81	8.15	0.1	12.96	0.1	0.00000062	0.00019431	A_07_P056725	ECs0810	cmr|NinE protein [ECs0810]
ECs0819	+24.51	10.29	+4.62	7.99	0.1	12.60	0.1	0.00000148	0.00022429	A_07_P056765	ECs0819	cmr|endolysin [ECs0819]
ECs0820	+14.27	8.77	+3.83	6.85	0.1	10.69	0.0	0.00000240	0.00027039	A_07_P056766	ECs0820	cmr|putative endopeptidase [ECs0820]
ECs0825	-3.19	8.57	-1.68	9.41	0.2	7.73	0.1	0.00025975	0.00307016	A_07_P056791	ECs0825	cmr|putative terminase large subunit [ECs0825]
ECs0827	-2.05	9.90	-1.04	10.42	0.1	9.38	0.1	0.00286931	0.01731483	A_07_P056801	ECs0827	cmr|putative portal protein [ECs0827]
ECs0828	-2.70	8.18	-1.43	8.90	0.1	7.47	0.2	0.00064463	0.00577966	A_07_P056809	ECs0828	cmr|hypothetical protein [ECs0828]
ECs0839	+2.52	9.64	+1.33	8.97	0.2	10.31	0.1	0.00120470	0.00909682	A_07_P056861	ECs0839	cmr|putative minor tail protein [ECs0839]
ECs0840	+7.62	9.58	+2.93	8.11	0.2	11.04	0.0	0.00001938	0.00062155	A_07_P056866	ECs0840	cmr|putative tail assembly protein [ECs0840]
ECs0841	+3.08	8.69	+1.62	7.88	0.2	9.50	0.2	0.00096893	0.00775143	A_07_P056872	ECs0841	cmr|putative tail assembly protein [ECs0841]
ECs0878	+2.85	10.13	+1.51	9.38	0.0	10.89	0.1	0.00020856	0.00262051	A_07_P056933	ECs0878	cmr|putative enzyme [ECs0878]
ECs0901	+2.40	7.26	+1.27	6.62	0.1	7.89	0.2	0.00178083	0.01216899	A_07_P056937	ECs0901	cmr|hypothetical protein [ECs0901]
ECs0906	+2.43	10.63	+1.28	9.99	0.2	11.27	0.0	0.00124787	0.00931993	A_07_P056943	ECs0906	cmr|hypothetical protein [ECs0906]
ECs0950	-2.02	11.51	-1.01	12.02	0.1	11.00	0.2	0.00245419	0.01539194	A_07_P056991	ECs0950	cmr|hypothetical protein [ECs0950]
ECs0981	+2.81	7.98	+1.49	7.23	0.2	8.72	0.1	0.00053277	0.00505906	A_07_P063053	ECs0981	cmr|anaerobic dimethyl sulfoxide reductase subunit C [ECs0981]
ECs1011	+4.91	9.74	+2.30	8.59	0.1	10.89	0.0	0.00002655	0.00072942	A_07_P063067	ECs1011	cmr|aspartate aminotransferase [ECs1011]
ECs1015	+5.36	9.25	+2.42	8.04	0.0	10.46	0.1	0.00001876	0.00061398	A_07_P063074	ECs1015	cmr|aminopeptidase N [ECs1015]
ECs1081	-3.82	9.15	-1.93	10.11	0.1	8.18	0.0	0.00005492	0.00107955	A_07_P057173	ECs1081	cmr|hypothetical protein [ECs1081]
ECs1092	+6.84	11.43	+2.77	10.05	0.3	12.82	0.0	0.00007088	0.00125539	A_07_P057221	ECs1092	cmr|putative lipoprotein Rz1 protein precursor [ECs1092]
ECs1093	+2.67	7.32	+1.41	6.61	0.0	8.03	0.1	0.00028768	0.00327971	A_07_P057227	ECs1093	cmr|putative endopeptidase [ECs1093]
ECs1105	-2.88	12.22	-1.53	12.98	0.1	11.46	0.3	0.00217496	0.01405686	A_07_P057269	ECs1105	cmr|putative terminase small subunit [ECs1105]
ECs1106	+4.06	9.16	+2.02	8.15	0.0	10.17	0.0	0.00003737	0.00087155	A_07_P057274	ECs1106	cmr|putative terminase large subunit [ECs1106]
ECs1107	+7.66	8.71	+2.94	7.24	0.0	10.17	0.1	0.00000724	0.00040140	A_07_P057276	ECs1107	cmr|putative portal protein [ECs1107]
ECs1108	+45.22	10.81	+5.50	8.06	0.2	13.56	0.1	0.00000094	0.00019431	A_07_P057281	ECs1108	cmr|putative head-tail preconnector protein [ECs1108]
ECs1109	+39.03	10.31	+5.29	7.67	0.3	12.95	0.1	0.00000220	0.00026540	A_07_P057290	ECs1109	cmr|putative head decoration protein [ECs1109]
ECs1110	+27.81	9.24	+4.80	6.84	0.0	11.64	0.2	0.00000197	0.00025681	A_07_P057293	ECs1110	cmr|putative major head protein [ECs1110]
ECs1115	+3.01	8.36	+1.59	7.57	0.1	9.16	0.1	0.00038712	0.00402821	A_07_P057316	ECs1115	cmr|putative minor tail protein [ECs1115]
ECs1119	-2.11	10.77	-1.08	11.31	0.2	10.24	0.1	0.00336452	0.01958264	A_07_P057336	ECs1119	cmr|hypothetical protein [ECs1119]
ECs1160	-2.02	10.48	-1.01	10.99	0.0	9.97	0.3	0.00858206	0.03982196	A_07_P061927	ECs1160	cmr|putative integrase [ECs1160]
ECs1163	+42.62	9.84	+5.41	7.13	0.0	12.54	0.1	0.00000031	0.00019431	A_07_P061944	ECs1163	cmr|hypothetical protein [ECs1163]
ECs1170	+59.08	10.61	+5.88	7.66	0.2	13.55	0.1	0.00000104	0.00020391	A_07_P061980	ECs1170	cmr|C4-type zinc finger protein [ECs1170]
ECs1173	+7.59	10.37	+2.92	8.91	0.6	11.83	0.1	0.00065453	0.00584189	A_07_P061991	ECs1173	cmr|hypothetical protein [ECs1173]
ECs1174	+19.62	12.65	+4.29	10.50	0.3	14.80	0.2	0.00001473	0.00054604	A_07_P061996	ECs1174	cmr|exonuclease [ECs1174]
ECs1175	+8.74	15.25	+3.13	13.69	0.0	16.82	0.0	0.00000442	0.00033535	A_07_P062005	ECs1175	cmr|recombination protein Bet [ECs1175]
ECs1176	+35.96	13.05	+5.17	10.47	0.1	15.64	0.1	0.00000057	0.00019431	A_07_P062008	ECs1176	cmr|host-nuclease inhibitor protein Gam [ECs1176]
ECs1177	+2.83	14.38	+1.50	13.63	0.0	15.13	0.0	0.00017068	0.00227100	A_07_P062011	ECs1177	cmr|Kil protein [ECs1177]
ECs1179	+21.08	13.97	+4.40	11.77	0.2	16.17	0.0	0.00000204	0.00025889	A_07_P062021	ECs1179	cmr|single strand binding protein Ea10 [ECs1179]
ECs1185	-4.10	13.22	-2.04	14.24	0.0	12.20	0.0	0.00003527	0.00084015	A_07_P062053	ECs1185	cmr|putative cI repressor protein [ECs1185]
ECs1186	+11.56	11.53	+3.53	9.77	0.2	13.30	0.0	0.00000679	0.00038477	A_07_P062059	ECs1186	cmr|putative regulatory protein [ECs1186]
ECs1187	+7.41	12.07	+2.89	10.63	0.1	13.52	0.1	0.00000818	0.00041270	A_07_P062064	ECs1187	cmr|regulatory protein CII [ECs1187]
ECs1197	+57.27	9.94	+5.84	7.02	0.0	12.86	0.1	0.00000025	0.00019431	A_07_P062106	ECs1197	cmr|NinE protein [ECs1197]
ECs1207	-2.00	8.25	-1.00	8.75	0.1	7.75	0.1	0.00177437	0.01213541	A_07_P062157	ECs1207	cmr|hypothetical protein [ECs1207]
ECs1234	+2.32	7.53	+1.21	6.92	0.1	8.13	0.3	0.00272304	0.01663479	A_07_P062282	ECs1234	cmr|putative outer membrane protein [ECs1234]
ECs1249	-2.34	12.04	-1.23	12.66	0.2	11.43	0.1	0.00192438	0.01280417	A_07_P062357	ECs1249	cmr|hypothetical protein [ECs1249]
ECs1273	-3.38	7.85	-1.76	8.72	0.6	6.97	0.0	0.00816684	0.03837586	A_07_P062382	ECs1273	cmr|FidL-like protein [ECs1273]
ECs1276	-2.59	8.95	-1.37	9.64	0.4	8.27	0.0	0.00643008	0.03186500	A_07_P062400	ECs1276	cmr|putative chaperone protein [ECs1276]
ECs1283	-2.38	8.29	-1.25	8.92	0.2	7.67	0.0	0.00193226	0.01282529	A_07_P062434	ECs1283	cmr|hemolysin activator-related protein [ECs1283]
ECs1299	-2.25	11.11	-1.17	11.69	0.4	10.53	0.1	0.01117072	0.04896207	A_07_P062501	ECs1299	cmr|putative integrase [ECs1299]
ECs1300	+3.57	8.66	+1.84	7.75	0.2	9.58	0.2	0.00046683	0.00461378	A_07_P062509	ECs1300	cmr|putative membrane protein [ECs1300]
ECs1328	-2.59	8.18	-1.38	8.87	0.1	7.49	0.0	0.00038603	0.00402231	A_07_P062631	ECs1328	cmr|hypothetical protein [ECs1328]
ECs1338	-3.05	9.78	-1.61	10.59	0.1	8.98	0.1	0.00028359	0.00325162	A_07_P062685	ECs1338	cmr|hypothetical protein [ECs1338]
ECs1339	-2.94	8.79	-1.56	9.57	0.3	8.01	0.1	0.00090378	0.00739560	A_07_P062686	ECs1339	cmr|hypothetical protein [ECs1339]
ECs1359	-2.30	8.30	-1.20	8.90	0.1	7.70	0.1	0.00065424	0.00584189	A_07_P060342	ECs1359	cmr|hypothetical protein [ECs1359]
ECs1363	-12.59	9.92	-3.65	11.75	0.1	8.09	0.3	0.00001719	0.00058318	A_07_P060356	ECs1363	cmr|hypothetical protein [ECs1363]
ECs1369	-2.53	7.70	-1.34	8.37	0.1	7.03	0.1	0.00059619	0.00547315	A_07_P060383	ECs1369	cmr|hypothetical protein [ECs1369]
ECs1380	-8.39	9.97	-3.07	11.50	0.1	8.43	0.1	0.00000624	0.00036832	A_07_P060427	ECs1380	cmr|putative transposase OrfB protein of insertion sequence IS629 [ECs1380]
ECs1381	-2.94	9.87	-1.56	10.65	0.1	9.09	0.1	0.00041201	0.00420666	A_07_P060432	ECs1381	cmr|putative transposase OrfA protein of insertion sequence IS629 [ECs1381]
ECs1387	-2.45	8.52	-1.29	9.17	0.0	7.88	0.1	0.00039574	0.00409097	A_07_P060457	ECs1387	cmr|hypothetical protein [ECs1387]
ECs1392	-4.71	8.93	-2.24	10.05	0.0	7.81	0.3	0.00017996	0.00236303	A_07_P060482	ECs1392	cmr|hypothetical protein [ECs1392]
ECs1395	-2.55	7.65	-1.35	8.32	0.1	6.97	0.1	0.00072447	0.00627400	A_07_P060497	ECs1395	cmr|hypothetical protein [ECs1395]
ECs1494	-2.09	9.67	-1.06	10.20	0.2	9.13	0.0	0.00288192	0.01736433	A_07_P063103	ECs1494	cmr|hypothetical protein [ECs1494]
ECs1511	-2.48	12.33	-1.31	12.98	0.1	11.67	0.4	0.00762094	0.03637851	A_07_P060633	ECs1511	cmr|hypothetical protein [ECs1511]
ECs1518	+17.31	10.17	+4.11	8.12	0.3	12.23	0.0	0.00001004	0.00046359	A_07_P060667	ECs1518	cmr|hypothetical protein [ECs1518]
ECs1526	-5.92	8.67	-2.57	9.95	0.0	7.39	0.1	0.00001250	0.00051423	A_07_P060699	ECs1526	cmr|hypothetical protein [ECs1526]
ECs1537	-3.10	11.82	-1.63	12.64	0.1	11.00	0.4	0.00231620	0.01472790	A_07_P060748	ECs1537	cmr|hypothetical protein [ECs1537]
ECs1558	+4.01	8.97	+2.00	7.97	0.3	9.97	0.1	0.00052965	0.00503852	A_07_P060837	ECs1558	cmr|putative tail assembly protein [ECs1558]
ECs1595	-3.83	11.29	-1.94	12.25	0.3	10.32	0.4	0.00304539	0.01807261	A_07_P061000	ECs1595	cmr|hypothetical protein [ECs1595]
ECs1607	-3.43	13.82	-1.78	14.71	0.0	12.93	0.2	0.00036405	0.00388583	A_07_P061030	ECs1607	cmr|hypothetical protein [ECs1607]
ECs1608	+3.27	8.51	+1.71	7.65	0.1	9.36	0.1	0.00023140	0.00282329	A_07_P061034	ECs1608	cmr|isocitrate dehydrogenase [ECs1608]
ECs1609	+17.16	8.86	+4.10	6.81	0.1	10.91	0.2	0.00000457	0.00033765	A_07_P061036	ECs1609	cmr|intergrase [ECs1609]
ECs1610	+95.49	10.66	+6.58	7.37	0.3	13.94	0.0	0.00000093	0.00019431	A_07_P061042	ECs1610	cmr|excisionase [ECs1610]
ECs1612	+5.64	12.66	+2.50	11.41	0.0	13.90	0.0	0.00001412	0.00053964	A_07_P061055	ECs1612	cmr|replication protein P [ECs1612]
ECs1623	+20.88	9.65	+4.38	7.45	0.4	11.84	0.0	0.00003064	0.00078195	A_07_P061089	ECs1623	cmr|putative endopeptidase [ECs1623]
ECs1625	+2.36	7.56	+1.24	6.94	0.0	8.17	0.2	0.00143959	0.01035874	A_07_P061095	ECs1625	cmr|Bor protein precursor [ECs1625]
ECs1638	+35.31	10.61	+5.14	8.04	0.1	13.18	0.0	0.00000052	0.00019431	A_07_P061143	ECs1638	cmr|minor tail protein [ECs1638]
ECs1639	+5.90	11.21	+2.56	9.93	0.0	12.49	0.1	0.00001524	0.00055785	A_07_P061148	ECs1639	cmr|minor tail protein [ECs1639]
ECs1640	+28.79	9.67	+4.85	7.24	0.1	12.09	0.1	0.00000064	0.00019431	A_07_P061154	ECs1640	cmr|major tail protein [ECs1640]
ECs1641	+26.59	9.54	+4.73	7.17	0.1	11.91	0.2	0.00000286	0.00028251	A_07_P061159	ECs1641	cmr|minor tail protein [ECs1641]
ECs1642	+7.62	8.32	+2.93	6.85	0.0	9.78	0.2	0.00001809	0.00060095	A_07_P061162	ECs1642	cmr|minor tail protein [ECs1642]
ECs1643	+6.36	10.19	+2.67	8.86	0.0	11.53	0.0	0.00001017	0.00046432	A_07_P061170	ECs1643	cmr|tail length tape measure protein precursor [ECs1643]
ECs1644	+6.43	10.05	+2.68	8.71	0.2	11.40	0.2	0.00012273	0.00179744	A_07_P061173	ECs1644	cmr|minor tail protein [ECs1644]
ECs1645	+4.27	9.75	+2.10	8.70	0.2	10.79	0.1	0.00018629	0.00241201	A_07_P061180	ECs1645	cmr|minor tail protein [ECs1645]
ECs1666	-4.62	9.71	-2.21	10.82	0.2	8.61	0.1	0.00006738	0.00121740	A_07_P058060	ECs1666	cmr|putative transposase OrfB protein of insertion sequence IS629 [ECs1666]
ECs1702	-2.23	8.11	-1.15	8.69	0.1	7.53	0.2	0.00219478	0.01415011	A_07_P058124	ECs1702	cmr|trehalase [ECs1702]
ECs1724	+3.38	9.35	+1.76	8.47	0.0	10.23	0.0	0.00007581	0.00131154	A_07_P058147	ECs1724	cmr|hypothetical protein [ECs1724]
ECs1726	+3.37	11.12	+1.75	10.24	0.1	12.00	0.0	0.00016061	0.00218551	A_07_P063128	ECs1726	cmr|nitrate/nitrite response regulator protein NarL [ECs1726]
ECs1743	+21.39	11.02	+4.42	8.81	0.3	13.23	0.0	0.00000616	0.00036832	A_07_P063132	ECs1743	cmr|oligopeptide transport periplasmic binding protein [ECs1743]
ECs1751	+10.22	10.24	+3.35	8.56	0.0	11.91	0.0	0.00000299	0.00028496	A_07_P058156	ECs1751	cmr|hypothetical protein [ECs1751]
ECs1773	-2.43	15.57	-1.28	16.21	0.0	14.93	0.0	0.00036682	0.00391011	A_07_P058245	ECs1773	cmr|putative prophage maintenance protein [ECs1773]
ECs1827	-4.05	9.31	-2.02	10.32	0.0	8.30	0.2	0.00022439	0.00275355	A_07_P058453	ECs1827	cmr|IS630 hypothetical protein [ECs1827]
ECs1833	+3.69	8.61	+1.88	7.67	0.1	9.55	0.1	0.00006503	0.00119336	A_07_P058463	ECs1833	cmr|tryptophan synthase beta protein [ECs1833]
ECs1839	-2.31	8.96	-1.21	9.57	0.1	8.36	0.0	0.00089888	0.00739123	A_07_P058472	ECs1839	cmr|hypothetical protein [ECs1839]
ECs1839	-2.36	8.76	-1.24	9.38	0.0	8.14	0.1	0.00052261	0.00499575	A_07_P058472	ECs1839	cmr|hypothetical protein [ECs1839]
ECs1839	-2.39	9.06	-1.26	9.69	0.0	8.43	0.0	0.00037277	0.00394177	A_07_P058472	ECs1839	cmr|hypothetical protein [ECs1839]
ECs1839	-2.38	9.07	-1.25	9.70	0.0	8.45	0.0	0.00037871	0.00396960	A_07_P058472	ECs1839	cmr|hypothetical protein [ECs1839]
ECs1900	-2.76	12.10	-1.47	12.83	0.0	11.36	0.2	0.00059551	0.00547004	A_07_P058536	ECs1900	cmr|putative enzyme [ECs1900]
ECs1906	+2.17	8.45	+1.12	7.90	0.2	9.01	0.1	0.00234582	0.01486822	A_07_P058547	ECs1906	cmr|hypothetical protein [ECs1906]
ECs1930	-2.21	10.16	-1.14	10.73	0.0	9.59	0.2	0.00184752	0.01244116	A_07_P058575	ECs1930	cmr|hypothetical protein [ECs1930]
ECs1951	+56.52	10.09	+5.82	7.18	0.2	13.00	0.1	0.00000099	0.00020340	A_07_P058631	ECs1951	cmr|hypothetical protein [ECs1951]
ECs2049	-2.32	7.66	-1.21	8.27	0.1	7.05	0.0	0.00095827	0.00769355	A_07_P058915	ECs2049	cmr|hypothetical protein [ECs2049]
ECs2081	-2.18	10.06	-1.12	10.62	0.1	9.50	0.1	0.00133959	0.00979769	A_07_P058916	ECs2081	cmr|hypothetical protein [ECs2081]
ECs2113	+2.11	8.29	+1.08	7.75	0.1	8.83	0.1	0.00114409	0.00877803	A_07_P054316	ECs2113	cmr|putative type 1 fimbrial protein precursor [ECs2113]
ECs2139	+2.37	7.87	+1.25	7.24	0.3	8.49	0.2	0.00467360	0.02498512	A_07_P054346	ECs2139	cmr|multiple antibiotic resistance protein [ECs2139]
ECs2177	+3.21	8.34	+1.68	7.50	0.0	9.18	0.3	0.00060345	0.00551728	A_07_P054468	ECs2177	cmr|putative portal protein [ECs2177]
ECs2183	+9.60	9.65	+3.26	8.01	0.1	11.28	0.0	0.00000490	0.00034758	A_07_P054491	ECs2183	cmr|putative lipoprotein Rz1 precursor [ECs2183]
ECs2184	+12.93	8.89	+3.69	7.05	0.0	10.74	0.0	0.00000188	0.00025064	A_07_P054498	ECs2184	cmr|putative endopeptidase [ECs2184]
ECs2234	+4.76	8.35	+2.25	7.22	0.0	9.48	0.0	0.00002191	0.00066473	A_07_P054691	ECs2234	cmr|putative host specificity protein [ECs2234]
ECs2241	-2.64	11.84	-1.40	12.54	0.2	11.14	0.1	0.00156265	0.01102754	A_07_P054726	ECs2241	cmr|putative tail protein [ECs2241]
ECs2319	+2.52	8.51	+1.33	7.84	0.3	9.17	0.1	0.00328762	0.01925605	A_07_P054872	ECs2319	cmr|mannose-6-phosphate isomerase [ECs2319]
ECs2356	+2.23	9.57	+1.15	9.00	0.0	10.15	0.0	0.00056480	0.00527744	A_07_P054895	ECs2356	cmr|hypothetical protein [ECs2356]
ECs2431	+4.04	8.70	+2.01	7.69	0.2	9.70	0.1	0.00026646	0.00310509	A_07_P054909	ECs2431	cmr|hypothetical protein [ECs2431]
ECs2471	+8.13	11.20	+3.02	9.69	0.2	12.71	0.0	0.00001780	0.00059515	A_07_P054922	ECs2471	cmr|hypothetical protein [ECs2471]
ECs2477	-3.10	9.19	-1.63	10.00	0.1	8.37	0.3	0.00099521	0.00788955	A_07_P054931	ECs2477	cmr|putative transposase OrfA protein of insertion sequence IS629 [ECs2477]
ECs2478	-2.72	8.43	-1.44	9.15	0.1	7.71	0.4	0.00693111	0.03376342	A_07_P054936	ECs2478	cmr|putative transposase OrfB protein of insertion sequence IS629 [ECs2478]
ECs2526	+3.65	8.26	+1.87	7.32	0.3	9.19	0.0	0.00038260	0.00399182	A_07_P054984	ECs2526	cmr|hypothetical protein [ECs2526]
ECs2547	-2.35	9.24	-1.23	9.85	0.0	8.62	0.0	0.00041874	0.00425603	A_07_P054991	ECs2547	cmr|hypothetical protein [ECs2547]
ECs2555	-5.65	10.34	-2.50	11.59	0.2	9.09	0.1	0.00004535	0.00095882	A_07_P063252	ECs2555	cmr|protease II [ECs2555]
ECs2619	-2.29	10.88	-1.20	11.48	0.2	10.28	0.1	0.00235605	0.01490900	A_07_P055016	ECs2619	cmr|putative membrane protein [ECs2619]
ECs2658	-3.77	9.92	-1.91	10.87	0.1	8.96	0.1	0.00007092	0.00125539	A_07_P055157	ECs2658	cmr|hypothetical protein [ECs2658]
ECs2668	+4.51	9.99	+2.17	8.90	0.0	11.08	0.0	0.00002816	0.00074298	A_07_P063276	ECs2668	cmr|putative transport system permease protein [ECs2668]
ECs2700	-2.25	8.23	-1.17	8.81	0.0	7.64	0.1	0.00096215	0.00770892	A_07_P063287	ECs2700	cmr|hypothetical protein [ECs2700]
ECs2720	+4.56	8.94	+2.19	7.85	0.0	10.04	0.1	0.00003855	0.00088510	A_07_P055249	ECs2720	cmr|putative host specificity protein [ECs2720]
ECs2720	+4.35	8.94	+2.12	7.88	0.1	10.00	0.1	0.00008627	0.00143428	A_07_P055249	ECs2720	cmr|putative host specificity protein [ECs2720]
ECs2734	+5.37	10.85	+2.42	9.64	0.1	12.06	0.0	0.00001695	0.00058050	A_07_P055282	ECs2734	cmr|putative head completion protein [ECs2734]
ECs2748	+2.58	8.28	+1.37	7.60	0.0	8.96	0.3	0.00351794	0.02022393	A_07_P055335	ECs2748	cmr|hypothetical protein [ECs2748]
ECs2754	-2.29	15.46	-1.19	16.06	0.1	14.87	0.0	0.00054731	0.00516907	A_07_P055361	ECs2754	cmr|prophage maintenance protein [ECs2754]
ECs2773	-2.34	11.19	-1.23	11.81	0.0	10.58	0.0	0.00044512	0.00444948	A_07_P055453	ECs2773	cmr|putative integrase [ECs2773]
ECs2782	-8.54	8.61	-3.09	10.16	0.1	7.07	0.1	0.00000712	0.00039666	A_07_P055462	ECs2782	cmr|hypothetical protein [ECs2782]
ECs2792	-8.02	8.55	-3.00	10.06	0.0	7.05	0.0	0.00000533	0.00034950	A_07_P055475	ECs2792	cmr|hypothetical protein [ECs2792]
ECs2793	-3.61	8.68	-1.85	9.60	0.1	7.75	0.4	0.00121028	0.00912855	A_07_P055479	ECs2793	cmr|hypothetical protein [ECs2793]
ECs2795	-7.45	10.66	-2.90	12.10	0.1	9.21	0.1	0.00001200	0.00050646	A_07_P055487	ECs2795	cmr|putative transposase OrfB protein of insertion sequence IS629 [ECs2795]
ECs2800	+2.25	9.46	+1.17	8.88	0.2	10.05	0.1	0.00220375	0.01419051	A_07_P055513	ECs2800	cmr|hypothetical protein [ECs2800]

ECs2825	+6.84	8.67	+2.77	7.28	0.2	10.05	0.0	0.00005026	0.00101969	A_07_P062811	ECs2825	cmr|N- [ECs2825]
ECs2828	-2.26	8.93	-1.18	9.52	0.1	8.34	0.0	0.00056586	0.00528183	A_07_P055560	ECs2828	cmr|regulator of length of O-antigen component of lipopolysaccharide chains [ECs2828]
ECs2829	-4.03	8.73	-2.01	9.73	0.0	7.72	0.0	0.00004252	0.00093353	A_07_P055562	ECs2829	cmr|UDP-glucose 6-dehydrogenase [ECs2829]
ECs2835	+2.10	10.27	+1.07	9.74	0.1	10.81	0.1	0.00114753	0.00879585	A_07_P055581	ECs2835	cmr|phosphomannomutase [ECs2835]
ECs2845	-2.80	10.47	-1.49	11.22	0.0	9.73	0.3	0.00198508	0.01309851	A_07_P055631	ECs2845	cmr|putative glycosyl transferase [ECs2845]
ECs2905	-3.03	8.58	-1.60	9.38	0.1	7.78	0.2	0.00057798	0.00535596	A_07_P062818	ECs2905	cmr|hypothetical protein [ECs2905]
ECs2933	-3.84	9.14	-1.94	10.11	0.1	8.17	0.1	0.00006569	0.00120118	A_07_P055796	ECs2933	cmr|putative transposase OrfA protein of insertion sequence IS629 [ECs2933]
ECs2978	-2.02	8.63	-1.02	9.14	0.1	8.12	0.1	0.00162210	0.01133021	A_07_P052883	ECs2978	cmr|hypothetical protein [ECs2978]
ECs2981	+34.60	9.06	+5.11	6.50	0.2	11.62	0.0	0.00000093	0.00019431	A_07_P052891	ECs2981	cmr|putative DNA methylase [ECs2981]
ECs2985	+9.24	13.08	+3.21	11.47	0.0	14.68	0.0	0.00000397	0.00032449	A_07_P052912	ECs2985	cmr|Ren protein [ECs2985]
ECs2986	+7.23	14.54	+2.85	13.11	0.1	15.96	0.1	0.00001029	0.00046680	A_07_P052916	ECs2986	cmr|phage replication protein P [ECs2986]
ECs2987	+13.89	11.35	+3.80	9.45	0.2	13.25	0.0	0.00000453	0.00033765	A_07_P052921	ECs2987	cmr|phage replication protein O [ECs2987]
ECs2988	+4.86	9.54	+2.28	8.39	0.2	10.68	0.0	0.00008858	0.00145271	A_07_P052928	ECs2988	cmr|regulatory protein CII [ECs2988]
ECs2996	+16.55	13.74	+4.05	11.71	0.1	15.76	0.0	0.00000131	0.00021824	A_07_P052967	ECs2996	cmr|putative single-stranded DNA binding protein [ECs2996]
ECs2997	+11.70	13.60	+3.55	11.82	0.1	15.37	0.0	0.00000354	0.00030339	A_07_P052972	ECs2997	cmr|regulatory protein cIII [ECs2997]
ECs2998	+3.08	13.78	+1.62	12.97	0.0	14.59	0.0	0.00011299	0.00169761	A_07_P052976	ECs2998	cmr|Kil protein [ECs2998]
ECs2999	+30.13	12.73	+4.91	10.27	0.2	15.19	0.0	0.00000119	0.00021323	A_07_P052981	ECs2999	cmr|host-nuclease inhibitor protein gam [ECs2999]
ECs3000	+23.29	11.41	+4.54	9.14	0.0	13.68	0.1	0.00000136	0.00022125	A_07_P052986	ECs3000	cmr|putative host-nuclease inhibitor protein Gam [ECs3000]
ECs3001	+7.56	13.54	+2.92	12.08	0.1	15.00	0.1	0.00001238	0.00051423	A_07_P052993	ECs3001	cmr|recombination protein Bet [ECs3001]
ECs3002	+5.88	12.34	+2.56	11.06	0.0	13.62	0.1	0.00001547	0.00056253	A_07_P053000	ECs3002	cmr|exonuclease [ECs3002]
ECs3003	+40.59	11.98	+5.34	9.31	0.1	14.65	0.3	0.00000501	0.00034758	A_07_P053004	ECs3003	cmr|hypothetical protein [ECs3003]
ECs3004	+53.38	10.60	+5.74	7.73	0.2	13.47	0.2	0.00000147	0.00022429	A_07_P053006	ECs3004	cmr|hypothetical protein [ECs3004]
ECs3005	+37.52	11.64	+5.23	9.03	0.3	14.26	0.2	0.00000606	0.00036814	A_07_P053011	ECs3005	cmr|hypothetical protein [ECs3005]
ECs3006	+64.74	9.77	+6.02	6.77	0.1	12.78	0.1	0.00000031	0.00019431	A_07_P053018	ECs3006	cmr|putative C4-type zinc finger protein [ECs3006]
ECs3042	+14.47	10.08	+3.86	8.15	0.1	12.00	0.1	0.00000185	0.00025064	A_07_P053100	ECs3042	cmr|galactose-binding transport protein [ECs3042]
ECs3104	+2.42	10.42	+1.28	9.78	0.0	11.06	0.0	0.00035054	0.00377532	A_07_P053132	ECs3104	cmr|outer membrane protein C OmpC [ECs3104]
ECs3152	+2.02	8.91	+1.01	8.41	0.3	9.42	0.1	0.00798753	0.03777581	A_07_P053169	ECs3152	cmr|2-oxoglutarate decarboxylase [ECs3152]
ECs3160	+3.66	7.70	+1.87	6.77	0.1	8.64	0.1	0.00010823	0.00165600	A_07_P062826	ECs3160	cmr|NADH dehydrogenase I chain N [ECs3160]
ECs3163	+10.44	8.79	+3.38	7.10	0.1	10.48	0.3	0.00003442	0.00083231	A_07_P053173	ECs3163	cmr|NADH dehydrogenase I chain K [ECs3163]
ECs3167	+8.06	9.77	+3.01	8.26	0.1	11.27	0.1	0.00001206	0.00050646	A_07_P053176	ECs3167	cmr|NADH dehydrogenase I chain G [ECs3167]
ECs3169	+5.45	11.63	+2.45	10.41	0.2	12.86	0.1	0.00007987	0.00135656	A_07_P063350	ECs3169	cmr|NADH dehydrogenase I chain E [ECs3169]
ECs3179	+3.34	10.23	+1.74	9.37	0.0	11.10	0.0	0.00007634	0.00131755	A_07_P053181	ECs3179	cmr|hypothetical protein [ECs3179]
ECs3196	+5.83	9.18	+2.54	7.90	0.0	10.45	0.1	0.00002699	0.00073391	A_07_P053197	ECs3196	cmr|amidophosphoribosyltransferase [ECs3196]
ECs3197	+5.55	10.93	+2.47	9.69	0.0	12.17	0.0	0.00001362	0.00052801	A_07_P053205	ECs3197	cmr|membrane protein required for colicin V production [ECs3197]
ECs3243	-2.70	8.14	-1.43	8.86	0.1	7.43	0.2	0.00092896	0.00755454	A_07_P053337	ECs3243	cmr|sucrose-6 phosphate hydrolase [ECs3243]
ECs3296	-4.87	11.15	-2.28	12.30	0.1	10.01	0.1	0.00003186	0.00079613	A_07_P053399	ECs3296	cmr|thiosulfate binding protein [ECs3296]
ECs3306	-5.23	11.79	-2.39	12.99	0.0	10.60	0.1	0.00002209	0.00066624	A_07_P063373	ECs3306	cmr|N-acetylmuramoyl-l-alanine amidase I [ECs3306]
ECs3370	+4.74	11.25	+2.25	10.12	0.1	12.37	0.0	0.00004444	0.00094837	A_07_P053430	ECs3370	cmr|IMP dehydrogenase [ECs3370]
ECs3382	-2.32	11.49	-1.22	12.10	0.1	10.88	0.2	0.00155263	0.01097764	A_07_P053440	ECs3382	cmr|putative anaerobic dimethyl sulfoxide reductase chain C [ECs3382]
ECs3398	-5.31	13.60	-2.41	14.81	0.0	12.40	0.0	0.00001676	0.00058010	A_07_P053458	ECs3398	cmr|putative ATP synthase beta subunit [ECs3398]
ECs3442	+4.18	8.71	+2.06	7.68	0.3	9.74	0.0	0.00053507	0.00507319	A_07_P053491	ECs3442	cmr|ATP-dependent RNA helicase [ECs3442]
ECs3450	+2.73	9.45	+1.45	8.73	0.0	10.18	0.1	0.00038797	0.00402870	A_07_P053496	ECs3450	cmr|hypothetical protein [ECs3450]
ECs3492	-3.48	8.70	-1.80	9.60	0.0	7.80	0.1	0.00014289	0.00200686	A_07_P055876	ECs3492	cmr|hypothetical protein [ECs3492]
ECs3521	-2.21	9.17	-1.14	9.74	0.1	8.60	0.2	0.00363596	0.02074077	A_07_P055974	ECs3521	cmr|hypothetical protein [ECs3521]
ECs3535	+2.79	8.33	+1.48	7.59	0.0	9.07	0.2	0.00046241	0.00457870	A_07_P055985	ECs3535	cmr|hypothetical protein [ECs3535]
ECs3569	-3.19	8.06	-1.68	8.90	0.1	7.22	0.2	0.00061077	0.00556489	A_07_P055993	ECs3569	cmr|electron transport protein HydN [ECs3569]
ECs3585	+2.15	9.31	+1.10	8.75	0.1	9.86	0.1	0.00121244	0.00913448	A_07_P056003	ECs3585	cmr|hydrogenase isoenzyme HypD [ECs3585]
ECs3607	-2.11	7.48	-1.08	8.02	0.1	6.94	0.0	0.00113979	0.00875357	A_07_P063410	ECs3607	cmr|alkaline phosphatase isozyme conversion [ECs3607]
ECs3649	+3.15	8.06	+1.65	7.23	0.1	8.88	0.0	0.00012560	0.00182757	A_07_P063411	ECs3649	cmr|putative transport protein [ECs3649]
ECs3663	+3.87	11.51	+1.95	10.53	0.0	12.48	0.0	0.00004347	0.00094275	A_07_P056139	ECs3663	cmr|L-fuculokinase [ECs3663]
ECs3670	-2.17	9.35	-1.12	9.91	0.1	8.79	0.1	0.00155412	0.01097764	A_07_P056142	ECs3670	cmr|hypothetical protein [ECs3670]
ECs3683	+2.40	7.85	+1.26	7.22	0.2	8.49	0.1	0.00135229	0.00986580	A_07_P056146	ECs3683	cmr|prepilin peptidase dependent protein A [ECs3683]
ECs3758	-2.96	8.80	-1.56	9.58	0.2	8.02	0.1	0.00045989	0.00456818	A_07_P056278	ECs3758	cmr|putative oxidoreductase Fe-S subunit [ECs3758]
ECs3764	-3.52	9.27	-1.82	10.17	0.2	8.36	0.0	0.00014428	0.00202100	A_07_P056285	ECs3764	cmr|ssDNA exonuclease RecJ [ECs3764]
ECs3772	+2.27	7.84	+1.18	7.25	0.1	8.43	0.0	0.00067970	0.00598833	A_07_P056288	ECs3772	cmr|hypothetical protein [ECs3772]
ECs3774	+3.18	8.55	+1.67	7.71	0.1	9.38	0.1	0.00025353	0.00301482	A_07_P063454	ECs3774	cmr|glycine decarboxylase [ECs3774]
ECs3778	+2.49	11.95	+1.31	11.29	0.0	12.61	0.0	0.00029545	0.00334773	A_07_P056295	ECs3778	cmr|2-octaprenyl-6-methoxyphenol 4-monoxygenase [ECs3778]
ECs3792	-2.33	7.19	-1.22	7.80	0.2	6.58	0.4	0.00798410	0.03777094	A_07_P056301	ECs3792	cmr|putative transcriptional regulator LYSR-type [ECs3792]
ECs3799	+2.11	7.27	+1.08	6.74	0.1	7.81	0.2	0.00395595	0.02205159	A_07_P056312	ECs3799	cmr|hypothetical protein [ECs3799]
ECs3800	+7.47	8.28	+2.90	6.83	0.1	9.73	0.0	0.00000878	0.00042939	A_07_P056318	ECs3800	cmr|putative transporter [ECs3800]
ECs3801	+2.89	8.30	+1.53	7.53	0.1	9.06	0.0	0.00021258	0.00265188	A_07_P056323	ECs3801	cmr|hypothetical lipoprotein [ECs3801]
ECs3802	+2.14	7.29	+1.10	6.74	0.0	7.84	0.0	0.00068820	0.00603958	A_07_P056330	ECs3802	cmr|putative ABC transporter ATP-binding subunit [ECs3802]
ECs3837	-2.01	12.11	-1.00	12.61	0.1	11.61	0.0	0.00172526	0.01186652	A_07_P063461	ECs3837	cmr|adenine glycosylase [ECs3837]
ECs3844	-4.92	9.67	-2.30	10.82	0.0	8.52	0.1	0.00002943	0.00076000	A_07_P056361	ECs3844	cmr|hypothetical protein [ECs3844]
ECs3853	-2.00	8.43	-1.00	8.93	0.0	7.93	0.0	0.00106076	0.00827993	A_07_P056407	ECs3853	cmr|putative transposase [ECs3853]
ECs3858	-2.65	7.56	-1.41	8.26	0.5	6.85	0.2	0.01018590	0.04533978	A_07_P056433	ECs3858	cmr|hypothetical protein [ECs3858]
ECs3869	-2.56	11.82	-1.36	12.50	0.1	11.15	0.1	0.00032927	0.00361129	A_07_P056468	ECs3869	cmr|hypothetical protein [ECs3869]
ECs3870	-3.23	9.05	-1.69	9.89	0.3	8.20	0.1	0.00131797	0.00971976	A_07_P056475	ECs3870	cmr|hypothetical protein [ECs3870]
ECs3874	+2.14	9.62	+1.10	9.07	0.2	10.17	0.0	0.00226472	0.01450600	A_07_P056483	ECs3874	cmr|hypothetical protein [ECs3874]
ECs3916	-2.65	8.15	-1.40	8.85	0.1	7.44	0.1	0.00035114	0.00377879	A_07_P056524	ECs3916	cmr|putative ABC-type iron-siderophore transport system ATP-binding protein [ECs3916]
ECs3930	-2.40	13.15	-1.26	13.78	0.1	12.52	0.2	0.00111264	0.00860386	A_07_P056534	ECs3930	cmr|hypothetical protein [ECs3930]
ECs3966	+2.17	8.96	+1.11	8.40	0.0	9.52	0.0	0.00071400	0.00621064	A_07_P059647	ECs3966	cmr|putative enzyme [ECs3966]
ECs3995	+14.38	9.18	+3.85	7.25	0.2	11.10	0.0	0.00000613	0.00036832	A_07_P059659	ECs3995	cmr|putative kinase [ECs3995]
ECs4006	+4.64	9.22	+2.21	8.11	0.1	10.32	0.1	0.00003798	0.00087581	A_07_P059666	ECs4006	cmr|putative hydrolase [ECs4006]
ECs4014	+2.58	7.79	+1.37	7.10	0.1	8.47	0.2	0.00110162	0.00853121	A_07_P059680	ECs4014	cmr|N-acetyl-galactosamine / galactosamine PTS system enzyme IIA component [ECs4014]
ECs4014	+2.64	7.85	+1.40	7.15	0.3	8.55	0.1	0.00220217	0.01418618	A_07_P059680	ECs4014	cmr|N-acetyl-galactosamine / galactosamine PTS system enzyme IIA component [ECs4014]
ECs4014	+2.52	8.23	+1.33	7.56	0.1	8.89	0.1	0.00062968	0.00568443	A_07_P059680	ECs4014	cmr|N-acetyl-galactosamine / galactosamine PTS system enzyme IIA component [ECs4014]
ECs4023	-30.04	11.84	-4.91	14.29	0.1	9.39	0.1	0.00000060	0.00019431	A_07_P059688	ECs4023	cmr|truncated putative fimbrial protein [ECs4023]
ECs4052	+2.09	8.27	+1.07	7.74	0.1	8.80	0.2	0.00290426	0.01741803	A_07_P059701	ECs4052	cmr|argininosuccinate synthetase [ECs4052]
ECs4086	+4.49	9.83	+2.17	8.74	0.0	10.91	0.0	0.00002693	0.00073391	A_07_P063500	ECs4086	cmr|hypothetical protein [ECs4086]
ECs4094	+3.11	7.89	+1.64	7.07	0.0	8.71	0.1	0.00012156	0.00178700	A_07_P059712	ECs4094	cmr|hypothetical protein [ECs4094]
ECs4098	+2.04	7.67	+1.03	7.15	0.0	8.19	0.0	0.00103267	0.00811298	A_07_P059716	ECs4098	cmr|N-acetylneuraminate lyase [ECs4098]
ECs4115	-3.37	8.32	-1.75	9.20	0.1	7.45	0.2	0.00034044	0.00369388	A_07_P058957	ECs4115	cmr|hypothetical protein [ECs4115]
ECs4150	+2.96	10.91	+1.57	10.13	0.0	11.70	0.1	0.00015917	0.00217908	A_07_P059737	ECs4150	cmr|hypothetical protein [ECs4150]
ECs4237	+2.04	7.18	+1.03	6.67	0.1	7.69	0.1	0.00199118	0.01312227	A_07_P059765	ECs4237	cmr|hypothetical protein [ECs4237]
ECs4253	-2.36	9.32	-1.24	9.94	0.1	8.70	0.3	0.00349972	0.02014611	A_07_P063536	ECs4253	cmr|hypothetical protein [ECs4253]
ECs4260	+3.57	8.14	+1.83	7.23	0.2	9.06	0.1	0.00018400	0.00239209	A_07_P063542	ECs4260	cmr|positive regulator of mal regulon [ECs4260]
ECs4325	-2.92	8.32	-1.55	9.09	0.0	7.54	0.0	0.00014797	0.00205794	A_07_P059892	ECs4325	cmr|putative O-methyltransferase [ECs4325]
ECs4329	-3.10	10.49	-1.63	11.30	0.0	9.67	0.0	0.00010809	0.00165548	A_07_P059912	ECs4329	cmr|putative acyl carrier protein [ECs4329]
ECs4331	-2.33	7.85	-1.22	8.46	0.2	7.24	0.2	0.00261667	0.01616670	A_07_P059921	ECs4331	cmr|putative surfactin synthetase [ECs4331]
ECs4332	-2.27	7.53	-1.18	8.12	0.0	6.94	0.0	0.00052102	0.00498353	A_07_P059928	ECs4332	cmr|putative [ECs4332]
ECs4337	+2.05	7.48	+1.03	6.96	0.0	7.99	0.0	0.00094090	0.00760835	A_07_P059955	ECs4337	cmr|hypothetical lipoprotein [ECs4337]
ECs4339	+2.29	7.10	+1.19	6.51	0.2	7.70	0.1	0.00144895	0.01040556	A_07_P059965	ECs4339	cmr|putative beta-hydroxydecanoyl-ACP dehydrase [ECs4339]
ECs4353	-2.58	8.61	-1.37	9.30	0.1	7.93	0.2	0.00102922	0.00808988	A_07_P060001	ECs4353	cmr|putative sugar kinase [ECs4353]
ECs4367	+6.22	11.85	+2.64	10.53	0.1	13.17	0.0	0.00001898	0.00061693	A_07_P060029	ECs4367	cmr|universal stress protein UspA [ECs4367]
ECs4407	-2.42	9.40	-1.27	10.03	0.1	8.76	0.1	0.00065903	0.00586205	A_07_P060081	ECs4407	cmr|hypothetical protein [ECs4407]
ECs4457	-2.86	9.21	-1.52	9.97	0.3	8.46	0.3	0.00649773	0.03207913	A_07_P060122	ECs4457	cmr|putative transcriptional regulator [ECs4457]
ECs4475	+3.19	8.05	+1.67	7.21	0.1	8.88	0.1	0.00011744	0.00174890	A_07_P060156	ECs4475	cmr|mannitol-specific PTS system enzyme IIABC components [ECs4475]
ECs4505	-3.96	10.11	-1.99	11.10	0.2	9.12	0.3	0.00049106	0.00476059	A_07_P060214	ECs4505	cmr|lipopolysaccharide core biosynthesis protein RfaP [ECs4505]
ECs4587	-2.13	7.76	-1.09	8.31	0.0	7.22	0.1	0.00093066	0.00756053	A_07_P057552	ECs4587	cmr|hypothetical protein [ECs4587]
ECs4630	-2.52	7.41	-1.34	8.08	0.0	6.74	0.2	0.00121540	0.00914253	A_07_P062852	ECs4630	cmr|hypothetical protein [ECs4630]
ECs4643	+2.37	8.40	+1.24	7.77	0.2	9.02	0.0	0.00212384	0.01380587	A_07_P057644	ECs4643	cmr|hypothetical protein [ECs4643]
ECs4786	+2.93	8.43	+1.55	7.66	0.2	9.21	0.0	0.00047865	0.00468356	A_07_P063635	ECs4786	cmr|DNA polymerase I [ECs4786]
ECs4787	+4.11	9.46	+2.04	8.44	0.0	10.48	0.1	0.00004281	0.00093353	A_07_P057780	ECs4787	cmr|essential GTPase for cell cycle [ECs4787]
ECs4819	+2.68	11.01	+1.42	10.30	0.0	11.72	0.0	0.00021523	0.00267878	A_07_P057834	ECs4819	cmr|formate dehydrogenase-O iron-sulfur subunit [ECs4819]
ECs4832	+2.13	7.69	+1.09	7.14	0.0	8.23	0.0	0.00073110	0.00632113	A_07_P057869	ECs4832	cmr|positive regulator for rhaBAD operon [ECs4832]
ECs4847	+2.94	8.34	+1.55	7.57	0.2	9.12	0.1	0.00070347	0.00614276	A_07_P057876	ECs4847	cmr|hypothetical protein [ECs4847]
ECs4848	+2.38	9.60	+1.25	8.97	0.1	10.22	0.0	0.00043716	0.00439510	A_07_P057881	ECs4848	cmr|putative regulator [ECs4848]
ECs4866	-2.59	9.57	-1.37	10.26	0.1	8.89	0.1	0.00040676	0.00416878	A_07_P063657	ECs4866	cmr|hypothetical protein [ECs4866]
ECs4892	-3.10	12.44	-1.63	13.26	0.5	11.62	0.2	0.00856238	0.03977756	A_07_P057920	ECs4892	cmr|putative amino acid amidohydrolase [ECs4892]
ECs4893	-3.31	12.02	-1.73	12.88	0.3	11.15	0.1	0.00117798	0.00896134	A_07_P057923	ECs4893	cmr|putative membrane transport / symporter protein [ECs4893]
ECs4893	-2.15	11.93	-1.10	12.48	0.1	11.38	0.1	0.00132564	0.00973002	A_07_P057923	ECs4893	cmr|putative membrane transport / symporter protein [ECs4893]
ECs4896	-2.85	9.11	-1.51	9.86	0.1	8.35	0.1	0.00041964	0.00426244	A_07_P057927	ECs4896	cmr|tRNA [ECs4896]
ECs4921	+6.25	9.52	+2.64	8.20	0.0	10.84	0.0	0.00001032	0.00046700	A_07_P057952	ECs4921	cmr|endonuclease V [ECs4921]
ECs4956	-2.07	9.20	-1.05	9.72	0.0	8.67	0.1	0.00180110	0.01225015	A_07_P061239	ECs4956	cmr|hypothetical protein [ECs4956]
ECs4963	+2.64	8.15	+1.40	7.45	0.1	8.85	0.1	0.00056693	0.00528775	A_07_P061274	ECs4963	cmr|hypothetical protein [ECs4963]
ECs4978	-2.09	8.70	-1.06	9.23	0.2	8.17	0.1	0.00310066	0.01836601	A_07_P061350	ECs4978	cmr|hypothetical protein [ECs4978]
ECs5003	-2.11	7.62	-1.08	8.16	0.1	7.08	0.3	0.00524741	0.02732890	A_07_P061472	ECs5003	cmr|putative sorbitol-6-phosphate 2-dehydrogenase [ECs5003]
ECs5043	-2.64	8.32	-1.40	9.02	0.1	7.62	0.1	0.00045061	0.00449586	A_07_P063693	ECs5043	cmr|hypothetical protein [ECs5043]
ECs5051	+10.86	8.62	+3.44	6.90	0.2	10.35	0.1	0.00001535	0.00055949	A_07_P063704	ECs5051	cmr|acetyl-CoA synthetase [ECs5051]
ECs5065	-2.50	7.86	-1.32	8.52	0.0	7.20	0.0	0.00029832	0.00335727	A_07_P061508	ECs5065	cmr|hypothetical protein [ECs5065]
ECs5129	-3.22	9.54	-1.69	10.39	0.0	8.70	0.1	0.00010981	0.00167356	A_07_P058973	ECs5129	cmr|suppressor of groEL [ECs5129]
ECs5165	+11.02	11.05	+3.46	9.32	0.0	12.78	0.0	0.00000282	0.00028143	A_07_P058980	ECs5165	cmr|hypothetical protein [ECs5165]
ECs5273	-2.14	7.36	-1.09	7.91	0.0	6.82	0.1	0.00080478	0.00681577	A_07_P061747	ECs5273	cmr|major type 1 subunit fimbrin [ECs5273]
ECs5282	+4.60	8.31	+2.20	7.21	0.1	9.41	0.0	0.00003278	0.00080633	A_07_P061751	ECs5282	cmr|D-mannonate oxidoreductase [ECs5282]
ECs5296	+2.74	7.76	+1.45	7.03	0.1	8.49	0.0	0.00028603	0.00327271	A_07_P061800	ECs5296	cmr|hypothetical protein [ECs5296]
ECs5338	+4.80	10.35	+2.26	9.22	0.1	11.48	0.0	0.00002536	0.00071770	A_07_P061864	ECs5338	cmr|hypothetical protein [ECs5338]
ECs5344	+2.09	8.70	+1.06	8.17	0.2	9.23	0.1	0.00290293	0.01741803	A_07_P061868	ECs5344	cmr|hypothetical protein [ECs5344]
ECs5345	+5.21	8.88	+2.38	7.69	0.1	10.07	0.1	0.00002925	0.00076000	A_07_P061871	ECs5345	cmr|putative lipoate-protein ligase A [ECs5345]
ECs5350	+2.11	7.67	+1.08	7.13	0.0	8.21	0.0	0.00081088	0.00684165	A_07_P062875	ECs5350	cmr|soluble lytic murein transglycosylase [ECs5350]
edd	+2.56	8.40	+1.36	7.72	0.1	9.07	0.2	0.00084412	0.00705035	A_07_P015931	b1851	cmr|6-phosphogluconate dehydratase [b1851]
elaA	+2.42	8.02	+1.28	7.39	0.0	8.66	0.1	0.00064304	0.00577530	A_07_P032393	c_2811	cmr|Protein elaA [c_2811]
elbB	+4.80	10.08	+2.26	8.95	0.1	11.22	0.0	0.00002625	0.00072778	A_07_P012521	b3209	cmr|sigma cross-reacting protein 27A [b3209]
emrA	-2.00	11.03	-1.00	11.53	0.0	10.53	0.1	0.00132054	0.00971976	A_07_P001896	b2685	cmr|multidrug resistance secretion protein [b2685]
emrD	-5.87	9.97	-2.55	11.24	0.0	8.69	0.3	0.00012603	0.00183113	A_07_P017297	b3673	cmr|2-module integral membrane pump; multidrug resistance [b3673]
emtA	-2.32	10.53	-1.21	11.14	0.2	9.93	0.0	0.00160614	0.01123368	A_07_P006916	b1193	cmr|murein transglycosylase E [b1193]
eno	+3.90	10.35	+1.96	9.37	0.1	11.33	0.0	0.00005551	0.00108557	A_07_P002342	b2779	cmr|enolase [b2779]
epd	+3.18	10.63	+1.67	9.80	0.2	11.47	0.0	0.00035616	0.00381456	A_07_P011783	b2927	cmr|D-erythrose 4-phosphate dehydrogenase [b2927]
eptA	+2.34	8.46	+1.23	7.85	0.0	9.08	0.1	0.00049506	0.00478762	A_07_P011307	b4114	cmr|orf, hypothetical protein [b4114]
eptB	+2.32	7.99	+1.22	7.38	0.0	8.59	0.1	0.00083915	0.00702801	A_07_P007657	b3546	cmr|orf, hypothetical protein [b3546]
EQC	-2.09	9.05	-1.06	9.59	0.2	8.52	0.2	0.00582192	0.02964589	(+)eQC-42	EQC	
erpA	-12.08	11.18	-3.59	12.98	0.1	9.38	0.1	0.00000297	0.00028496	A_07_P002744	b0156	cmr|orf, hypothetical protein [b0156]
essD	+22.15	9.78	+4.47	7.55	0.2	12.01	0.0	0.00000428	0.00033352	A_07_P016531	b0554	cmr|orf, hypothetical protein [b0554]
essQ	+2.53	7.70	+1.34	7.03	0.2	8.37	0.1	0.00162713	0.01135023	A_07_P010455	b1556	cmr|orf, hypothetical protein [b1556]
ETG10_234183	-2.63	12.08	-1.39	12.77	0.2	11.38	0.2	0.00211567	0.01376413	ETG10_234183	ETG10_234183	
ETG10_234183	-2.91	12.53	-1.54	13.31	0.2	11.76	0.5	0.00622418	0.03105973	ETG10_234183	ETG10_234183	
ETG10_236652	+2.61	7.47	+1.39	6.78	0.1	8.17	0.0	0.00042201	0.00428377	ETG10_236652	ETG10_236652	
etpO	-2.07	9.18	-1.05	9.70	0.1	8.65	0.2	0.00336110	0.01957722	A_07_P048849	ABH-0244075	asap|type II secretion protein [L7044]
eutA	+2.27	7.33	+1.19	6.74	0.1	7.92	0.2	0.00294589	0.01761493	A_07_P013311	b2451	cmr|orf, hypothetical protein [b2451]
eutC	+2.14	7.29	+1.10	6.74	0.1	7.84	0.1	0.00124206	0.00928541	A_07_P042830	Z3705	cmr|ethanolamine ammonia-lyase, light chain [Z3705]
exoD	+6.21	13.03	+2.63	11.71	0.0	14.34	0.2	0.00005105	0.00103301	A_07_P016473	b0539	cmr|putative exonuclease [b0539]
exuR	-2.41	12.59	-1.27	13.22	0.1	11.95	0.2	0.00109157	0.00847006	A_07_P000199	b3094	cmr|negative regulator of exu regulon, exuT, uxaAC, and uxuB [b3094]
exuT	+6.58	8.61	+2.72	7.25	0.1	9.97	0.1	0.00001473	0.00054604	A_07_P000192	b3093	cmr|transport of hexuronates [b3093]
fabB	+7.57	12.20	+2.92	10.74	0.0	13.66	0.0	0.00000589	0.00036511	A_07_P013901	b2323	cmr|3-oxoacyl- [b2323]
fabD	+5.00	12.81	+2.32	11.65	0.1	13.97	0.1	0.00002697	0.00073391	A_07_P001661	b1092	cmr|malonyl-CoA- [b1092]
fabG	+5.97	12.37	+2.58	11.08	0.1	13.66	0.1	0.00002062	0.00063650	A_07_P001668	b1093	cmr|3-oxoacyl- [b1093]
fabH	+3.80	12.63	+1.93	11.66	0.1	13.59	0.3	0.00039229	0.00405794	A_07_P001656	b1091	cmr|3-oxoacyl- [b1091]
fabZ	-2.09	14.37	-1.06	14.90	0.0	13.84	0.0	0.00087923	0.00727980	A_07_P002859	b0180	cmr|(3R)-hydroxymyristol acyl carrier protein dehydratase [b0180]
fadI	-3.34	11.34	-1.74	12.21	0.2	10.47	0.0	0.00034038	0.00369388	A_07_P053270	ECs3225	cmr|putative acyltransferase [ECs3225]
fadI	-3.40	10.78	-1.77	11.67	0.1	9.90	0.1	0.00009887	0.00155813	A_07_P014000	b2342	cmr|putative acyltransferase [b2342]
fadI	-2.93	11.08	-1.55	11.86	0.4	10.31	0.1	0.00254529	0.01580792	A_07_P042685	Z3605	cmr|putative acyltransferase [Z3605]
fadJ	+2.07	11.06	+1.05	10.53	0.0	11.59	0.0	0.00090348	0.00739560	A_07_P013995	b2341	cmr|putative enzyme [b2341]
fadL	+5.84	8.20	+2.55	6.93	0.1	9.48	0.1	0.00002052	0.00063471	A_07_P032506	c_2889	cmr|Long-chain fatty acid transport protein precursor [c_2889]
fbaA	+9.27	11.27	+3.21	9.67	0.1	12.88	0.1	0.00000597	0.00036562	A_07_P011772	b2925	cmr|fructose-bisphosphate aldolase, class II [b2925]
fbaB	+2.23	10.10	+1.16	9.52	0.0	10.68	0.0	0.00061149	0.00556489	A_07_P003250	b2097	cmr|orf, hypothetical protein [b2097]
fbp	-2.53	14.13	-1.34	14.80	0.0	13.46	0.0	0.00027061	0.00313734	A_07_P003714	b4232	cmr|fructose-bisphosphatase [b4232]
fdhE	+2.22	9.89	+1.15	9.32	0.2	10.47	0.1	0.00144569	0.01039310	A_07_P039385	c_4841	cmr|Protein fdhE [c_4841]
fdhE	+2.02	10.02	+1.01	9.52	0.1	10.53	0.1	0.00209039	0.01364472	A_07_P039385	c_4841	cmr|Protein fdhE [c_4841]
fdhE	+2.10	10.30	+1.07	9.77	0.0	10.84	0.0	0.00082053	0.00690825	A_07_P039385	c_4841	cmr|Protein fdhE [c_4841]
fdnG	+3.44	9.71	+1.78	8.82	0.2	10.60	0.2	0.00040855	0.00417949	A_07_P009461	b1474	cmr|formate dehydrogenase-N, nitrate-inducible, alpha subunit [b1474]
fdnH	+3.10	8.51	+1.63	7.69	0.1	9.32	0.1	0.00020594	0.00260014	A_07_P009468	b1475	cmr|formate dehydrogenase-N, nitrate-inducible, iron-sulfur beta subunit [b1475]
fdoG	+2.86	8.55	+1.52	7.79	0.0	9.31	0.1	0.00024255	0.00291707	A_07_P019246	b3894	cmr|formate dehydrogenase-O, major subunit [b3894]
fdoH	+3.36	9.53	+1.75	8.66	0.2	10.41	0.2	0.00058420	0.00539455	A_07_P019241	b3893	cmr|formate dehydrogenase-O, iron-sulfur subunit [b3893]
fdoH	+3.34	10.14	+1.74	9.26	0.1	11.01	0.0	0.00007682	0.00132177	A_07_P039386	c_4843	cmr|Formate dehydrogenase-O beta subunit [c_4843]
fdoH	+2.65	11.07	+1.41	10.36	0.0	11.77	0.0	0.00021702	0.00269339	A_07_P047064	Z5435	cmr|formate dehydrogenase-O, iron-sulfur subunit [Z5435]
fdoI	+4.80	9.81	+2.26	8.67	0.1	10.94	0.1	0.00002635	0.00072866	A_07_P019239	b3892	cmr|formate dehydrogenase, cytochrome B556 [b3892]
fecB	-2.32	9.95	-1.22	10.56	0.1	9.34	0.1	0.00083922	0.00702801	A_07_P003977	b4290	cmr|citrate-dependent iron transport, periplasmic protein [b4290]
fecI	-2.39	8.64	-1.26	9.26	0.1	8.01	0.2	0.00109393	0.00848418	A_07_P003994	b4293	cmr|probable RNA polymerase sigma factor [b4293]
fepE	-19.90	11.28	-4.31	13.43	0.0	9.12	0.2	0.00000237	0.00027039	A_07_P031316	c_0674	cmr|Ferric enterobactin transport protein fepE [c_0674]
ffh	-2.68	13.72	-1.42	14.43	0.0	13.00	0.0	0.00022177	0.00273628	A_07_P019926	b2610	cmr|GTP-binding export factor binds to signal sequence, GTP and RNA [b2610]
fhuD	-2.25	7.90	-1.17	8.48	0.0	7.31	0.0	0.00054860	0.00517818	A_07_P002724	b0152	cmr|hydroxamate-dependent iron uptake, cytoplasmic membrane component [b0152]
fimA	-3.78	9.03	-1.92	9.99	0.0	8.07	0.1	0.00005837	0.00111936	A_07_P004096	b4314	cmr|major type 1 subunit fimbrin [b4314]
fimC	-2.53	8.36	-1.34	9.03	0.1	7.69	0.1	0.00050673	0.00488112	A_07_P004109	b4316	cmr|periplasmic chaperone, required for type 1 fimbriae [b4316]
fimD	-12.14	9.45	-3.60	11.25	0.0	7.65	0.2	0.00000749	0.00040630	A_07_P004111	b4317	cmr|outer membrane protein; export and assembly of type 1 fimbriae, interrupted [b4317]
fimF	-16.44	9.29	-4.04	11.31	0.2	7.27	0.1	0.00000406	0.00032474	A_07_P004120	b4318	cmr|fimbrial morphology [b4318]
fimF	-8.58	8.26	-3.10	9.82	0.1	6.71	0.1	0.00000758	0.00040630	A_07_P041401	c_5397	cmr|FimF protein precursor [c_5397]
fimG	-8.60	10.88	-3.10	12.43	0.1	9.32	0.1	0.00001105	0.00048316	A_07_P004124	b4319	cmr|fimbrial morphology [b4319]
fimH	-3.66	7.83	-1.87	8.77	0.1	6.90	0.0	0.00007113	0.00125539	A_07_P004128	b4320	cmr|minor fimbrial subunit, D-mannose specific adhesin [b4320]
fimI	-2.02	9.13	-1.01	9.64	0.1	8.63	0.1	0.00262177	0.01618712	A_07_P004101	b4315	cmr|fimbrial protein [b4315]
fis	+4.74	10.54	+2.24	9.41	0.1	11.66	0.0	0.00002913	0.00075920	A_07_P012772	b3261	cmr|site-specific DNA inversion stimulation factor; DNA-binding protein; a trans activator for transcription [b3261]
fklB	+2.42	11.68	+1.28	11.04	0.1	12.32	0.1	0.00077145	0.00659019	A_07_P011753	b4207	cmr|FKBP-type 22KD peptidyl-prolyl cis-trans isomerase [b4207]
fkpB	+4.25	10.50	+2.09	9.46	0.0	11.55	0.0	0.00003384	0.00082101	A_07_P010829	b0028	cmr|probable FKBX-type 16KD peptidyl-prolyl cis-trans isomerase [b0028]
flgK	-2.05	9.47	-1.04	9.99	0.1	8.96	0.2	0.00229730	0.01464911	A_07_P001612	b1082	cmr|flagellar biosynthesis, hook-filament junction protein 1 [b1082]
flgL	-2.31	8.59	-1.21	9.19	0.2	7.98	0.1	0.00157810	0.01109673	A_07_P001620	b1083	cmr|flagellar biosynthesis; hook-filament junction protein [b1083]
flhD	+2.72	8.55	+1.44	7.83	0.0	9.27	0.1	0.00025783	0.00305666	A_07_P016140	b1892	cmr|regulator of flagellar biosynthesis, acting on class 2 operons; transcriptional initiation factor [b1892]
fliA	-3.91	7.96	-1.97	8.94	0.1	6.97	0.1	0.00008935	0.00145767	A_07_P014973	b1922	cmr|flagellar biosynthesis; alternative sigma factor 28; regulation of flagellar operons [b1922]
fliI	-2.95	12.39	-1.56	13.16	0.3	11.61	0.1	0.00122183	0.00918208	A_07_P044691	Z3031	cmr|flagellum-specific ATP synthase [Z3031]
fliI	-2.80	12.49	-1.49	13.23	0.4	11.75	0.1	0.00371157	0.02103173	A_07_P055201	ECs2680	cmr|flagellum-specific ATP synthase [ECs2680]
fliI	-2.32	12.02	-1.22	12.63	0.4	11.41	0.1	0.01000488	0.04476184	A_07_P015066	b1941	cmr|flagellum-specific ATP synthase [b1941]
fliI	-2.81	11.78	-1.49	12.53	0.2	11.03	0.0	0.00042456	0.00430129	A_07_P035407	c_2358	cmr|Flagellum-specific ATP synthase [c_2358]
fliY	-6.88	12.76	-2.78	14.15	0.0	11.36	0.0	0.00000760	0.00040630	A_07_P041209	c_2335	cmr|Cystine-binding periplasmic protein precursor [c_2335]
fliY	-6.03	11.64	-2.59	12.93	0.1	10.34	0.0	0.00001250	0.00051423	A_07_P014964	b1920	cmr|putative periplasmic binding transport protein [b1920]
flu	+3.06	10.51	+1.62	9.70	0.5	11.31	0.0	0.00723627	0.03496863	A_07_P015330	b2000	cmr|outer membrane fluffing protein, similar to adhesin [b2000]
fmt	-2.71	11.97	-1.44	12.69	0.0	11.25	0.2	0.00044950	0.00448761	A_07_P012874	b3288	cmr|10-formyltetrahydrofolate:L-methionyl-tRNA [b3288]
fnr	-10.73	13.62	-3.42	15.33	0.0	11.91	0.2	0.00001341	0.00052642	A_07_P014647	b1334	cmr|transcriptional regulation of aerobic, anaerobic respiration, osmotic balance [b1334]
focA	+3.50	10.70	+1.81	9.80	0.1	11.60	0.1	0.00011177	0.00169030	A_07_P004969	b0904	cmr|probable formate transporter [b0904]
focA	+40.43	12.66	+5.34	9.99	0.0	15.33	0.1	0.00000065	0.00019431	A_07_P040486	c_1239	cmr|F1C major fimbrial subunit precursor [c_1239]
focC	+6.88	9.56	+2.78	8.16	0.1	10.95	0.0	0.00001310	0.00052207	A_07_P040500	c_1241	cmr|F1C periplasmic chaperone [c_1241]
focD	+3.56	10.43	+1.83	9.52	0.3	11.35	0.1	0.00052728	0.00502204	A_07_P040505	c_1242	cmr|F1C fimbrial usher [c_1242]
focF	+3.53	9.81	+1.82	8.90	0.2	10.72	0.1	0.00018230	0.00238186	A_07_P040507	c_1243	cmr|F1C minor fimbrial subunit F precursor [c_1243]
focX	-2.87	10.63	-1.52	11.39	0.2	9.87	0.1	0.00055293	0.00520656	A_07_P040530	c_1247	cmr|Putative Regulatory protein [c_1247]
folA	+2.54	9.24	+1.35	8.56	0.1	9.91	0.1	0.00043446	0.00437074	A_07_P010929	b0048	cmr|dihydrofolate reductase type I; trimethoprim resistance [b0048]
folA	+2.36	10.35	+1.24	9.73	0.1	10.97	0.0	0.00067081	0.00593666	A_07_P035679	c_0058	cmr|Dihydrofolate reductase [c_0058]
folA	+2.49	9.20	+1.32	8.54	0.0	9.86	0.1	0.00035211	0.00378335	A_07_P049144	Z0055	cmr|dihydrofolate reductase type I trimethoprim resistance [Z0055]
folA	+2.62	9.29	+1.39	8.60	0.0	9.98	0.0	0.00023169	0.00282329	A_07_P059409	ECs0051	cmr|dihydrofolate reductase type I; trimethoprim resistance [ECs0051]
fpr	+3.18	9.38	+1.67	8.54	0.1	10.21	0.1	0.00033822	0.00367995	A_07_P039438	c_4876	cmr|Ferredoxin--NADP reductase [c_4876]
fpr	+3.89	8.89	+1.96	7.91	0.1	9.87	0.1	0.00010565	0.00163561	A_07_P019396	b3924	cmr|ferredoxin-NADP reductase [b3924]
frdB	+2.66	12.30	+1.41	11.59	0.1	13.01	0.0	0.00030073	0.00337715	A_07_P011497	b4153	cmr|fumarate reductase, anaerobic, iron-sulfur protein subunit [b4153]
frdC	+2.61	11.48	+1.38	10.79	0.2	12.17	0.0	0.00084519	0.00705338	A_07_P011491	b4152	cmr|fumarate reductase, anaerobic, membrane anchor polypeptide [b4152]
frdD	+2.39	9.69	+1.26	9.06	0.2	10.32	0.0	0.00085517	0.00712456	A_07_P011487	b4151	cmr|fumarate reductase, anaerobic, membrane anchor polypeptide [b4151]
frmR	+6.50	10.64	+2.70	9.29	0.1	11.99	0.1	0.00001700	0.00058050	A_07_P020911	b0357	cmr|putative alpha helix chain [b0357]
frr	-2.15	13.50	-1.10	14.05	0.0	12.95	0.0	0.00070711	0.00617112	A_07_P002818	b0172	cmr|ribosome releasing factor [b0172]
fruR	+2.89	10.03	+1.53	9.26	0.1	10.79	0.1	0.00021876	0.00270977	A_07_P011076	b0080	cmr|transcriptional repressor of fru operon and others [b0080]
fryB	-2.13	7.38	-1.09	7.92	0.0	6.83	0.0	0.00081774	0.00688848	A_07_P014219	b2387	cmr|putative PTS system enzyme IIB component [b2387]
ftnA	+17.48	10.12	+4.13	8.05	0.0	12.18	0.0	0.00000107	0.00020503	A_07_P016193	b1905	cmr|cytoplasmic ferritin [b1905]
ftnB	+4.17	10.14	+2.06	9.11	0.1	11.17	0.1	0.00004097	0.00091779	A_07_P016180	b1902	cmr|ferritin-like protein [b1902]
ftsE	-8.42	11.03	-3.07	12.57	0.0	9.50	0.0	0.00000524	0.00034781	A_07_P007255	b3463	cmr|ATP-binding component of a membrane-associated complex involved in cell division [b3463]
ftsY	-4.40	13.17	-2.14	14.24	0.1	12.10	0.1	0.00005157	0.00103969	A_07_P007256	b3464	cmr|cell division membrane protein [b3464]
fucA	+2.64	10.69	+1.40	9.99	0.0	11.39	0.0	0.00023000	0.00281576	A_07_P005999	b2800	cmr|L-fuculose-1-phosphate aldolase [b2800]
fucK	+3.29	9.36	+1.72	8.50	0.1	10.22	0.0	0.00018109	0.00237584	A_07_P006014	b2803	cmr|L-fuculokinase [b2803]
fucK	+3.39	11.60	+1.76	10.72	0.3	12.49	0.0	0.00076736	0.00656593	A_07_P043254	Z4120	cmr|L-fuculokinase [Z4120]
fucO	+4.09	9.78	+2.03	8.77	0.0	10.80	0.1	0.00004092	0.00091779	A_07_P005991	b2799	cmr|L-1,2-propanediol oxidoreductase [b2799]
fucP	-2.14	10.38	-1.10	10.93	0.3	9.84	0.0	0.00393053	0.02197522	A_07_P006001	b2801	cmr|fucose permease [b2801]
fucR	+4.13	8.28	+2.05	7.26	0.1	9.31	0.0	0.00004770	0.00098950	A_07_P006024	b2805	cmr|positive regulator of the fuc operon [b2805]
fucU	+3.26	11.16	+1.71	10.30	0.1	12.01	0.0	0.00009335	0.00149973	A_07_P006018	b2804	cmr|protein of fucose operon [b2804]
fumA	+14.13	10.51	+3.82	8.60	0.0	12.42	0.0	0.00000166	0.00023773	A_07_P020691	b1612	cmr|fumarase A = fumarate hydratase Class I; aerobic isozyme [b1612]
fumB	+3.06	10.37	+1.61	9.56	0.4	11.17	0.0	0.00215863	0.01397427	A_07_P011347	b4122	cmr|fumarase B= fumarate hydratase Class I; anaerobic isozyme [b4122]
fumC	+5.11	8.64	+2.35	7.46	0.0	9.81	0.1	0.00002107	0.00064399	A_07_P042312	c_2003	cmr|Fumarate hydratase class II [c_2003]
fumC	+8.08	8.82	+3.02	7.32	0.1	10.33	0.0	0.00000566	0.00035797	A_07_P020689	b1611	cmr|fumarase C= fumarate hydratase Class II; isozyme [b1611]
galF	+3.14	10.29	+1.65	9.46	0.1	11.11	0.0	0.00019018	0.00245020	A_07_P015526	b2042	cmr|homolog of Salmonella UTP--glucose-1-P uridyltransferase, probably a UDP-gal transferase [b2042]
galK	+7.17	11.57	+2.84	10.15	0.1	13.00	0.1	0.00000898	0.00043361	A_07_P004240	b0757	cmr|galactokinase [b0757]
galM	+6.86	10.77	+2.78	9.38	0.1	12.16	0.1	0.00001329	0.00052521	A_07_P004231	b0756	cmr|galactose-1-epimerase [b0756]
galS	+2.87	9.57	+1.52	8.80	0.2	10.33	0.0	0.00064761	0.00579974	A_07_P032245	c_2685	cmr|Mgl repressor and galactose ultrainduction factor [c_2685]
galT	+2.25	10.30	+1.17	9.72	0.0	10.89	0.0	0.00055909	0.00525198	A_07_P004241	b0758	cmr|galactose-1-phosphate uridylyltransferase [b0758]
gamW	+20.43	13.28	+4.35	11.10	0.2	15.45	0.0	0.00000404	0.00032474	A_07_P038414	c_1537	cmr|Putative host-nuclease inhibitor protein Gam of bacteriphage [c_1537]
gapA	+14.88	10.66	+3.90	8.71	0.1	12.61	0.1	0.00000354	0.00030339	A_07_P015590	b1779	cmr|glyceraldehyde-3-phosphate dehydrogenase A [b1779]
gcd	-2.59	8.73	-1.37	9.42	0.2	8.05	0.1	0.00093147	0.00756060	A_07_P002585	b0124	cmr|glucose dehydrogenase [b0124]
gcvH	+76.66	11.01	+6.26	7.88	0.0	14.14	0.0	0.00000015	0.00019431	A_07_P041711	c_3484	cmr|Glycine cleavage system H protein [c_3484]
gcvH	+48.16	10.28	+5.59	7.49	0.2	13.08	0.0	0.00000075	0.00019431	A_07_P006489	b2904	cmr|in glycine cleavage complex, carrier of aminomethyl moiety via covalently bound lipoyl cofactor [b2904]
gcvH	+44.64	11.31	+5.48	8.57	0.3	14.05	0.0	0.00000188	0.00025064	A_07_P041275	c_3484	cmr|Glycine cleavage system H protein [c_3484]
gcvP	+3.23	8.70	+1.69	7.86	0.1	9.55	0.1	0.00023559	0.00285318	A_07_P032879	c_3483	cmr|Glycine dehydrogenase [c_3483]
gcvP	+3.73	8.60	+1.90	7.65	0.2	9.55	0.1	0.00016661	0.00223623	A_07_P052779	Z4240	cmr|glycine decarboxylase, P protein of glycine cleavage [Z4240]
gcvP	+5.62	8.29	+2.49	7.04	0.0	9.53	0.1	0.00002015	0.00063193	A_07_P006481	b2903	cmr|glycine decarboxylase, P protein of glycine cleavage system [b2903]
gcvR	+6.92	9.17	+2.79	7.78	0.1	10.57	0.1	0.00000882	0.00042982	A_07_P013453	b2479	cmr|transcriptional regulation of gcv operon [b2479]
gcvT	+46.68	10.72	+5.54	7.95	0.0	13.49	0.1	0.00000028	0.00019431	A_07_P006491	b2905	cmr|aminomethyltransferase [b2905]
gef	-4.59	12.55	-2.20	13.65	0.0	11.45	0.0	0.00002726	0.00073404	A_07_P048482	Z0016	cmr|Gef protein interferes with membrane function when [Z0016]
gef	-2.42	10.72	-1.28	11.36	0.1	10.08	0.0	0.00044094	0.00442459	A_07_P048478	Z0016	cmr|Gef protein interferes with membrane function when [Z0016]
gfcA	-3.40	11.94	-1.77	12.83	0.0	11.06	0.2	0.00031542	0.00349707	A_07_P005379	b0987	cmr|orf, hypothetical protein [b0987]
gfcD	-2.06	15.14	-1.04	15.66	0.0	14.62	0.2	0.00323447	0.01901548	A_07_P005364	b0984	cmr|orf, hypothetical protein [b0984]
ggt	-2.25	7.58	-1.17	8.16	0.0	6.99	0.1	0.00087198	0.00723355	A_07_P034524	c_4236	cmr|Gamma-glutamyltranspeptidase precursor [c_4236]
glcB	+3.34	9.20	+1.74	8.33	0.1	10.08	0.0	0.00009231	0.00148959	A_07_P012016	b2976	cmr|malate synthase G [b2976]
glcB	+4.78	11.22	+2.26	10.09	0.1	12.34	0.1	0.00004896	0.00100424	A_07_P041726	c_3705	cmr|Malate synthase G [c_3705]
glcD	+4.07	8.64	+2.02	7.62	0.0	9.65	0.0	0.00004252	0.00093353	A_07_P012031	b2979	cmr|glycolate oxidase subunit D [b2979]
glcF	+11.09	9.31	+3.47	7.58	0.1	11.05	0.1	0.00000593	0.00036511	A_07_P012026	b2978	cmr|glycolate oxidase iron-sulfur subunit [b2978]
glcG	+39.37	9.66	+5.30	7.01	0.0	12.31	0.0	0.00000030	0.00019431	A_07_P012024	b2977	cmr|orf, hypothetical protein [b2977]
gldA	+6.23	12.06	+2.64	10.74	0.2	13.38	0.1	0.00003134	0.00078813	A_07_P019501	b3945	cmr|glycerol dehydrogenase, [b3945]
glgA	+4.45	10.50	+2.15	9.42	0.1	11.58	0.1	0.00004587	0.00096667	A_07_P018171	b3429	cmr|glycogen synthase [b3429]
glgB	+2.58	9.19	+1.37	8.50	0.1	9.87	0.1	0.00044167	0.00442752	A_07_P018189	b3432	cmr|1,4-alpha-glucan branching enzyme [b3432]
glgC	+6.25	9.90	+2.64	8.57	0.1	11.22	0.0	0.00001456	0.00054604	A_07_P018179	b3430	cmr|glucose-1-phosphate adenylyltransferase [b3430]
glgP	+2.70	8.17	+1.43	7.46	0.3	8.89	0.1	0.00185063	0.01245671	A_07_P018166	b3428	cmr|glycogen phosphorylase [b3428]
glgS	+5.19	9.98	+2.38	8.79	0.0	11.17	0.0	0.00001740	0.00058395	A_07_P012366	b3049	cmr|glycogen biosynthesis, rpoS dependent [b3049]
glgX	+4.10	10.89	+2.04	9.87	0.1	11.91	0.1	0.00006778	0.00122099	A_07_P018182	b3431	cmr|part of glycogen operon, a glycosyl hydrolase, debranching enzyme [b3431]
glmM	+3.27	11.18	+1.71	10.32	0.1	12.03	0.0	0.00010259	0.00160236	A_07_P000591	b3176	cmr|similar to phosphoglucomutases and phosphomannomutases [b3176]
glnA	-4.89	13.16	-2.29	14.30	0.0	12.01	0.2	0.00005624	0.00109182	A_07_P019129	b3870	cmr|glutamine synthetase [b3870]
glnB	+3.68	11.06	+1.88	10.12	0.1	12.00	0.0	0.00006001	0.00114386	A_07_P019668	b2553	cmr|regulatory protein P-II for glutamine synthetase [b2553]
glnH	-3.21	14.65	-1.68	15.49	0.0	13.81	0.2	0.00030440	0.00340122	A_07_P004510	b0811	cmr|periplasmic glutamine-binding protein; permease [b0811]
gloA	+4.38	9.53	+2.13	8.47	0.2	10.60	0.1	0.00011152	0.00169030	A_07_P020890	b1651	cmr|lactoylglutathione lyase [b1651]
glpB	-2.16	10.46	-1.11	11.02	0.1	9.91	0.2	0.00197928	0.01307121	A_07_P000998	b2242	cmr|sn-glycerol-3-phosphate dehydrogenase [b2242]

glpE	-2.13	9.81	-1.09	10.36	0.0	9.26	0.1	0.00100097	0.00793120	A_07_P018153	b3425	cmr|protein of glp regulon [b3425]
glpF	+10.50	9.75	+3.39	8.05	0.1	11.44	0.0	0.00000328	0.00029330	A_07_P019412	b3927	cmr|facilitated diffusion of glycerol [b3927]
glpK	+2.01	9.90	+1.01	9.40	0.1	10.41	0.1	0.00143802	0.01035215	A_07_P019409	b3926	cmr|glycerol kinase [b3926]
glpT	+6.19	8.95	+2.63	7.63	0.1	10.26	0.2	0.00004123	0.00092082	A_07_P063326	ECs3125	cmr|sn-glycerol-3-phosphate permease [ECs3125]
glpT	+5.08	9.04	+2.35	7.87	0.1	10.21	0.0	0.00002284	0.00067725	A_07_P052656	Z3498	cmr|sn-glycerol-3-phosphate permease [Z3498]
glpT	+4.24	9.44	+2.08	8.39	0.2	10.48	0.0	0.00007958	0.00135303	A_07_P000990	b2240	cmr|sn-glycerol-3-phosphate permease [b2240]
gltA	+19.85	13.20	+4.31	11.04	0.1	15.35	0.1	0.00000273	0.00028012	A_07_P056596	ECs0745	cmr|citrate synthase [ECs0745]
gltA	+9.71	10.83	+3.28	9.19	0.2	12.47	0.1	0.00001454	0.00054604	A_07_P010118	b0720	cmr|citrate synthase [b0720]
gltA	+18.48	13.83	+4.21	11.72	0.1	15.93	0.0	0.00000132	0.00021824	A_07_P046286	Z0873	cmr|citrate synthase [Z0873]
gltD	+2.30	8.71	+1.20	8.11	0.1	9.31	0.1	0.00097072	0.00775788	A_07_P012545	b3213	cmr|glutamate synthase, small subunit [b3213]
gltK	+2.83	8.31	+1.50	7.56	0.1	9.06	0.0	0.00020281	0.00257504	A_07_P009831	b0653	cmr|glutamate/aspartate transport system permease [b0653]
gltL	+3.05	8.77	+1.61	7.97	0.1	9.57	0.1	0.00015262	0.00210589	A_07_P009830	b0652	cmr|ATP-binding protein of glutamate/aspartate transport system [b0652]
gltP	+3.20	8.66	+1.68	7.82	0.2	9.50	0.0	0.00047020	0.00463832	A_07_P009026	b4077	cmr|glutamate-aspartate symport protein [b4077]
glvG	-3.24	9.45	-1.70	10.30	0.4	8.61	0.0	0.00226923	0.01451717	A_07_P010606	ABE-0285077	asap|C-terminal fragment of predicted 6-phospho-beta-glucosidase (pseudogene) [b4556]
glyA	+9.22	11.83	+3.20	10.23	0.1	13.43	0.1	0.00000747	0.00040630	A_07_P019657	b2551	cmr|serine hydroxymethyltransferase [b2551]
gmhA	+3.69	9.34	+1.89	8.40	0.0	10.28	0.0	0.00005502	0.00108015	A_07_P053536	ECs0249	cmr|phosphoheptose isomerase [ECs0249]
gmhA	+3.25	9.24	+1.70	8.39	0.2	10.10	0.1	0.00041460	0.00423040	A_07_P043366	Z0280	cmr|phosphoheptose isomerase [Z0280]
gmhA	+5.41	11.54	+2.44	10.32	0.0	12.76	0.0	0.00001573	0.00056283	A_07_P030845	c_0372	cmr|Phosphoheptose isomerase [c_0372]
gmk	-13.48	12.61	-3.75	14.48	0.0	10.73	0.0	0.00000176	0.00024644	A_07_P017181	b3648	cmr|guanylate kinase [b3648]
gnd	+2.82	8.54	+1.50	7.79	0.4	9.29	0.0	0.00248644	0.01554664	A_07_P015470	b2029	cmr|gluconate-6-phosphate dehydrogenase, decarboxylating [b2029]
gnd	+2.80	10.09	+1.48	9.35	0.1	10.83	0.1	0.00035858	0.00383789	A_07_P042488	c_2556	cmr|6-phosphogluconate dehydrogenase, decarboxylating [c_2556]
gnsA	+2.72	11.11	+1.45	10.39	0.1	11.83	0.0	0.00031563	0.00349707	A_07_P010343	ABE-0285038	asap|predicted regulator of phosphatidylethanolamine synthesis [b4517]
gnsB	+14.71	10.00	+3.88	8.07	0.5	11.94	0.0	0.00009282	0.00149582	A_07_P020394	b1550	cmr|orf, hypothetical protein [b1550]
gntX	+3.10	11.79	+1.63	10.98	0.1	12.61	0.0	0.00019812	0.00252367	A_07_P018091	b3413	cmr|orf, hypothetical protein [b3413]
gntY	-3.65	10.94	-1.87	11.87	0.1	10.00	0.2	0.00024665	0.00295753	A_07_P018096	b3414	cmr|orf, hypothetical protein [b3414]
gntY	-3.26	11.27	-1.70	12.12	0.1	10.42	0.0	0.00016924	0.00225435	A_07_P018096	b3414	cmr|orf, hypothetical protein [b3414]
gntY	-2.90	11.21	-1.54	11.97	0.1	10.44	0.0	0.00017691	0.00232877	A_07_P018096	b3414	cmr|orf, hypothetical protein [b3414]
gntY	-3.49	11.11	-1.80	12.02	0.1	10.21	0.1	0.00010703	0.00164539	A_07_P018096	b3414	cmr|orf, hypothetical protein [b3414]
gntY	-3.16	11.10	-1.66	11.93	0.1	10.27	0.0	0.00018167	0.00237762	A_07_P018096	b3414	cmr|orf, hypothetical protein [b3414]
gph	+4.97	9.87	+2.31	8.72	0.1	11.03	0.0	0.00002588	0.00072358	A_07_P017954	b3385	cmr|phosphoglycolate phosphatase [b3385]
gpmA	+3.20	7.85	+1.68	7.01	0.2	8.69	0.2	0.00076106	0.00652976	A_07_P004227	b0755	cmr|phosphoglyceromutase 1 [b0755]
greB	-2.75	10.86	-1.46	11.59	0.1	10.13	0.1	0.00040567	0.00416132	A_07_P018057	b3406	cmr|transcription elongation factor and transcript cleavage [b3406]
groL	+4.22	12.63	+2.08	11.59	0.0	13.67	0.1	0.00004000	0.00090512	A_07_P011446	b4143	cmr|GroEL, chaperone Hsp60, peptide-dependent ATPase, heat shock protein [b4143]
groS	+5.01	13.34	+2.32	12.17	0.1	14.50	0.0	0.00002517	0.00071646	A_07_P011443	b4142	cmr|GroES, 10 Kd chaperone binds to Hsp60 in pres. Mg-ATP, suppressing its ATPase activity [b4142]
grxB	+9.27	9.69	+3.21	8.08	0.2	11.29	0.0	0.00001273	0.00051704	A_07_P001521	b1064	cmr|glutaredoxin 2 [b1064]
gshA	-3.47	10.31	-1.79	11.21	0.0	9.41	0.1	0.00010631	0.00164315	A_07_P001912	b2688	cmr|gamma-glutamate-cysteine ligase [b2688]
gshB	+2.46	11.33	+1.30	10.68	0.1	11.98	0.0	0.00040572	0.00416132	A_07_P011876	b2947	cmr|glutathione synthetase [b2947]
gspD	-2.87	7.81	-1.52	8.57	0.0	7.05	0.2	0.00040273	0.00414691	A_07_P013055	b3325	cmr|putative general protein secretion protein [b3325]
gspL	-2.94	9.82	-1.56	10.60	0.1	9.04	0.1	0.00030397	0.00340122	A_07_P013091	b3333	cmr|putative general protein secretion protein [b3333]
gspM	-3.59	7.80	-1.84	8.72	0.1	6.88	0.2	0.00025942	0.00307016	A_07_P017699	b3334	cmr|putative general secretion [b3334]
gst	+9.67	10.30	+3.27	8.66	0.1	11.94	0.0	0.00000602	0.00036752	A_07_P020808	b1635	cmr|glutathionine S-transferase [b1635]
guaA	+2.38	10.68	+1.25	10.05	0.1	11.30	0.1	0.00071081	0.00619446	A_07_P013594	b2507	cmr|GMP synthetase [b2507]
guaB	+3.92	11.28	+1.97	10.29	0.0	12.27	0.0	0.00004122	0.00092082	A_07_P030153	c_3027	cmr|Inosine-5-monophosphate dehydrogenase [c_3027]
guaB	+4.40	11.30	+2.14	10.23	0.1	12.37	0.0	0.00006555	0.00120005	A_07_P042850	Z3772	cmr|IMP dehydrogenase [Z3772]
gudP	+3.50	7.54	+1.81	6.64	0.1	8.44	0.1	0.00017079	0.00227100	A_07_P002391	b2789	cmr|putative transport protein [b2789]
gyrA	-4.50	12.96	-2.17	14.04	0.0	11.88	0.1	0.00003358	0.00081707	A_07_P000942	b2231	cmr|DNA gyrase, subunit A, type II topoisomerase [b2231]
gyrB	-5.65	12.53	-2.50	13.78	0.0	11.28	0.0	0.00001281	0.00051714	A_07_P005429	b3699	cmr|DNA gyrase subunit B, type II topoisomerase, ATPase activity [b3699]
hchA	+2.15	10.24	+1.11	9.69	0.3	10.79	0.0	0.00432030	0.02350338	A_07_P015194	b1967	cmr|orf, hypothetical protein [b1967]
hda	-3.77	10.72	-1.92	11.68	0.0	9.76	0.0	0.00004956	0.00100951	A_07_P013536	b2496	cmr|putative DNA replication factor [b2496]
hdeA	+3.10	8.23	+1.63	7.41	0.1	9.05	0.0	0.00019561	0.00250584	A_07_P007483	b3510	cmr|orf, hypothetical protein [b3510]
hdhA	+2.24	9.69	+1.16	9.11	0.1	10.27	0.1	0.00122866	0.00921583	A_07_P035106	c_2011	cmr|7-alpha-hydroxysteroid dehydrogenase [c_2011]
hemC	+4.70	11.59	+2.23	10.47	0.1	12.70	0.1	0.00002969	0.00076491	A_07_P039097	c_4724	cmr|Porphobilinogen deaminase [c_4724]
hemC	+3.42	9.77	+1.77	8.88	0.1	10.66	0.3	0.00067735	0.00597099	A_07_P005892	b3805	cmr|porphobilinogen deaminase = hydroxymethylbilane synthase [b3805]
hemE	+4.29	9.24	+2.10	8.19	0.1	10.29	0.0	0.00004879	0.00100402	A_07_P008648	b3997	cmr|uroporphyrinogen decarboxylase [b3997]
hemF	-2.22	11.37	-1.15	11.95	0.0	10.80	0.4	0.00864329	0.04005886	A_07_P013240	b2436	cmr|coproporphyrinogen III oxidase [b2436]
hemN	+2.32	8.20	+1.21	7.59	0.0	8.80	0.1	0.00087017	0.00722955	A_07_P041357	c_4816	cmr|Oxygen-independent coproporphyrinogen III oxidase [c_4816]
hepA	-4.18	10.67	-2.06	11.70	0.1	9.64	0.0	0.00003864	0.00088510	A_07_P010981	b0059	cmr|probable ATP-dependent RNA helicase [b0059]
hflB	-3.13	13.17	-1.65	13.99	0.0	12.35	0.0	0.00011208	0.00169030	A_07_P000601	b3178	cmr|degrades sigma32, integral membrane peptidase, cell division protein [b3178]
hflK	-2.26	12.12	-1.18	12.71	0.1	11.54	0.1	0.00072212	0.00626395	A_07_P011588	b4174	cmr|protease specific for phage lambda cII repressor [b4174]
hha	-4.61	10.04	-2.21	11.15	0.1	8.94	0.1	0.00004098	0.00091779	A_07_P021406	b0460	cmr|haemolysin expression modulating protein [b0460]
hisA	+4.57	8.92	+2.19	7.82	0.1	10.01	0.1	0.00005447	0.00107551	A_07_P015442	b2024	cmr|N- [b2024]
hisA	+4.77	8.80	+2.25	7.67	0.2	9.92	0.1	0.00006998	0.00124629	A_07_P051776	Z3186	cmr|N- [Z3186]
hisB	+8.20	9.00	+3.04	7.48	0.2	10.52	0.0	0.00001228	0.00051423	A_07_P015435	b2022	cmr|imidazoleglycerolphosphate dehydratase and histidinol-phosphate phosphatase [b2022]
hisC	+7.24	8.86	+2.86	7.43	0.1	10.28	0.1	0.00001723	0.00058333	A_07_P015429	b2021	cmr|histidinol-phosphate aminotransferase [b2021]
hisD	+8.49	9.38	+3.09	7.83	0.1	10.92	0.1	0.00000886	0.00042982	A_07_P015421	b2020	cmr|L-histidinal:NAD+ oxidoreductase; L-histidinol:NAD+ oxidoreductase [b2020]
hisF	+9.23	9.76	+3.21	8.16	0.6	11.36	0.1	0.00048796	0.00474808	A_07_P015446	b2025	cmr|imidazole glycerol phosphate synthase subunit in heterodimer with HisH = imidazole glycerol phsphate synthase holoenzyme [b2025]
hisG	+3.82	8.19	+1.93	7.22	0.0	9.16	0.1	0.00005751	0.00110693	A_07_P031978	c_2546	cmr|ATP phosphoribosyltransferase [c_2546]
hisG	+2.42	7.68	+1.28	7.04	0.2	8.32	0.0	0.00089680	0.00738456	A_07_P015420	b2019	cmr|ATP phosphoribosyltransferase [b2019]
hisH	+13.76	9.38	+3.78	7.49	0.1	11.27	0.1	0.00000327	0.00029330	A_07_P015440	b2023	cmr|glutamine amidotransferase subunit of heterodimer with HisF = imidazole glycerol phosphate synthase holoenzyme [b2023]
hisI	+3.83	8.10	+1.94	7.13	0.2	9.07	0.0	0.00013556	0.00192967	A_07_P042478	c_2553	cmr|Histidine biosynthesis bifunctional protein hisIE [c_2553]
hisJ	+4.46	10.86	+2.16	9.78	0.0	11.94	0.1	0.00003254	0.00080182	A_07_P013834	b2309	cmr|histidine-binding periplasmic protein of high-affinity histidine transport system [b2309]
hisM	+2.46	8.98	+1.30	8.33	0.1	9.63	0.1	0.00082745	0.00695536	A_07_P013822	b2307	cmr|histidine transport, membrane protein M [b2307]
hlyB	-2.32	8.38	-1.22	8.99	0.0	7.77	0.1	0.00067385	0.00595430	A_07_P033071	c_3573	cmr|Hemolysin B [c_3573]
hlyC	+3.58	9.16	+1.84	8.24	0.1	10.08	0.1	0.00012544	0.00182701	A_07_P033053	c_3569	cmr|Hemolysin C [c_3569]
hmp	+7.83	9.04	+2.97	7.56	0.2	10.52	0.0	0.00001250	0.00051423	A_07_P019661	b2552	cmr|dihydropteridine reductase, ferrisiderophore reductase activity [b2552]
hmpA	+10.00	9.38	+3.32	7.72	0.4	11.04	0.1	0.00008336	0.00140212	A_07_P041601	c_3075	cmr|Flavohemoprotein [c_3075]
hofH	-2.10	8.06	-1.07	8.60	0.2	7.53	0.2	0.00721878	0.03491522	A_07_P034333	c_4100	cmr|Putative general secretion pathway protein H precursor [c_4100]
hokD	-2.47	15.61	-1.31	16.26	0.0	14.95	0.0	0.00030452	0.00340122	A_07_P020454	b1562	cmr|polypeptide destructive to membrane potential [b1562]
holC	+5.90	10.12	+2.56	8.84	0.2	11.40	0.1	0.00004243	0.00093353	A_07_P003845	b4259	cmr|DNA polymerase III, chi subunit [b4259]
holE	+5.56	9.06	+2.48	7.82	0.1	10.29	0.0	0.00002209	0.00066624	A_07_P015890	b1842	cmr|DNA polymerase III, theta subunit [b1842]
holE	+4.20	9.32	+2.07	8.28	0.3	10.35	0.0	0.00042602	0.00430780	A_07_P035500	c_2252	cmr|DNA polymerase III, theta subunit [c_2252]
hpf	+13.82	11.98	+3.79	10.09	0.0	13.88	0.1	0.00000177	0.00024644	A_07_P012495	b3203	cmr|probable sigma-54 modulation protein [b3203]
hpt	-2.87	12.71	-1.52	13.47	0.0	11.95	0.0	0.00014795	0.00205794	A_07_P002588	b0125	cmr|hypoxanthine phosphoribosyltransferase [b0125]
hrpB	-2.68	10.21	-1.42	10.92	0.0	9.49	0.2	0.00051186	0.00491089	A_07_P035880	c_0181	cmr|ATP-dependent helicase hrpB [c_0181]
hscA	+2.03	11.00	+1.02	10.49	0.1	11.51	0.0	0.00124767	0.00931993	A_07_P019531	b2526	cmr|heat shock protein, chaperone, member of Hsp70 protein family [b2526]
hslU	+2.28	8.27	+1.19	7.67	0.2	8.86	0.0	0.00115471	0.00884231	A_07_P019432	b3931	cmr|heat shock protein hslVU, ATPase subunit, homologous to chaperones [b3931]
hslV	+3.91	11.76	+1.97	10.78	0.1	12.75	0.1	0.00008173	0.00138058	A_07_P019439	b3932	cmr|heat shock protein hslVU, proteasome-related peptidase subunit [b3932]
hspQ	-4.54	13.85	-2.18	14.94	0.1	12.76	0.1	0.00003110	0.00078710	A_07_P005278	b0966	cmr|orf, hypothetical protein [b0966]
htpG	+3.46	8.87	+1.79	7.97	0.2	9.77	0.0	0.00021039	0.00263076	A_07_P021471	b0473	cmr|chaperone Hsp90, heat shock protein C 62.5 [b0473]
htrG	-4.61	10.10	-2.20	11.20	0.1	8.99	0.1	0.00004726	0.00098397	A_07_P000008	b3055	cmr|orf, hypothetical protein [b3055]
hupA	+16.81	12.69	+4.07	10.65	0.0	14.72	0.0	0.00000121	0.00021323	A_07_P008662	b4000	cmr|DNA-binding protein HU-alpha [b4000]
hupB	+3.09	12.16	+1.63	11.35	0.1	12.98	0.1	0.00016794	0.00224832	A_07_P021314	b0440	cmr|DNA-binding protein HU-beta, NS1 [b0440]
hybA	+2.61	10.50	+1.38	9.81	0.1	11.19	0.1	0.00030391	0.00340122	A_07_P012111	b2996	cmr|hydrogenase-2 small subunit [b2996]
hybB	+5.95	11.25	+2.57	9.96	0.0	12.53	0.0	0.00001169	0.00050224	A_07_P012108	b2995	cmr|probable cytochrome Ni/Fe component of hydrogenase-2 [b2995]
hybC	+3.28	10.08	+1.71	9.22	0.0	10.94	0.1	0.00011187	0.00169030	A_07_P012105	b2994	cmr|probable large subunit, hydrogenase-2 [b2994]
hybD	+2.38	10.81	+1.25	10.18	0.2	11.44	0.0	0.00093339	0.00756708	A_07_P012100	b2993	cmr|probable processing element for hydrogenase-2 [b2993]
hydN	-3.65	8.14	-1.87	9.07	0.0	7.20	0.2	0.00026442	0.00309464	A_07_P043108	Z4021	cmr|involved in electron transport from formate to [Z4021]
hydN	-4.78	8.46	-2.26	9.58	0.0	7.33	0.1	0.00002665	0.00073020	A_07_P002014	b2713	cmr|involved in electron transport from formate to hydrogen, Fe-S centers [b2713]
hydN	-4.35	8.32	-2.12	9.38	0.1	7.26	0.1	0.00005211	0.00104646	A_07_P030619	c_3269	cmr|Electron transport protein hydN [c_3269]
hypB	+2.64	9.94	+1.40	9.24	0.1	10.64	0.1	0.00031931	0.00353038	A_07_P002083	b2727	cmr|guanine-nucleotide binding protein, functions as nickel donor for large subunit of hydrogenase 3 [b2727]
hypC	+2.13	12.30	+1.09	11.76	0.2	12.85	0.0	0.00177969	0.01216649	A_07_P002090	b2728	cmr|pleiotrophic effects on 3 hydrogenase isozymes [b2728]
hypD	+2.56	9.17	+1.36	8.49	0.1	9.85	0.1	0.00057455	0.00533670	A_07_P002091	b2729	cmr|pleiotrophic effects on 3 hydrogenase isozymes [b2729]
iap	-2.75	7.81	-1.46	8.54	0.1	7.08	0.4	0.00452729	0.02436236	A_07_P041682	c_3320	cmr|Alkaline phosphatase isozyme conversion protein precursor [c_3320]
ibpB	-3.78	10.61	-1.92	11.57	0.1	9.65	0.1	0.00008574	0.00143229	A_07_P017361	b3686	cmr|heat shock protein [b3686]
icd	+2.68	9.93	+1.42	9.22	0.1	10.64	0.2	0.00084538	0.00705338	A_07_P006631	b1136	cmr|isocitrate dehydrogenase, specific for NADP+ [b1136]
icdA	+5.30	8.44	+2.41	7.24	0.0	9.64	0.1	0.00002248	0.00067042	A_07_P050489	Z1865	cmr|isocitrate dehydrogenase, specific for NADP+ [Z1865]
icdA	+15.00	13.47	+3.91	11.51	0.0	15.42	0.1	0.00000289	0.00028300	A_07_P038343	c_1517	cmr|Isocitrate dehydrogenase [c_1517]
iclR	+7.49	8.47	+2.90	7.01	0.1	9.92	0.0	0.00000742	0.00040580	A_07_P008735	b4018	cmr|repressor of aceBA operon [b4018]
iclR	+12.70	8.95	+3.67	7.12	0.1	10.79	0.0	0.00000219	0.00026540	A_07_P041378	c_4975	cmr|Acetate operon repressor [c_4975]
idnO	-3.03	11.67	-1.60	12.47	0.3	10.87	0.2	0.00173536	0.01192035	A_07_P003879	b4266	cmr|5-keto-D-gluconate 5-reductase [b4266]
ihfA	+3.34	12.33	+1.74	11.46	0.0	13.20	0.1	0.00009415	0.00150861	A_07_P018614	b1712	cmr|integration host factor [b1712]
ihfB	+4.55	9.87	+2.18	8.78	0.0	10.96	0.1	0.00002860	0.00074930	A_07_P005009	b0912	cmr|integration host factor [b0912]
ilvB	+2.58	10.70	+1.37	10.01	0.2	11.38	0.0	0.00066917	0.00592567	A_07_P017291	b3671	cmr|acetolactate synthase I,valine-sensitive, large subunit [b3671]
ilvD	+3.95	9.04	+1.98	8.04	0.0	10.03	0.0	0.00003977	0.00090487	A_07_P039071	c_4693	cmr|Dihydroxy-acid dehydratase [c_4693]
ilvM	+3.89	8.35	+1.96	7.37	0.0	9.33	0.1	0.00004761	0.00098891	A_07_P005727	b3769	cmr|acetolactate synthase II, valine insensitive, small subunit [b3769]
ilvN	+2.22	8.68	+1.15	8.11	0.0	9.26	0.1	0.00070140	0.00613147	A_07_P038986	c_4595	cmr|Acetolactate synthase isozyme I small subunit [c_4595]
inaA	-2.76	7.82	-1.46	8.55	0.0	7.09	0.1	0.00021274	0.00265188	A_07_P000971	b2237	cmr|pH-inducible protein involved in stress response [b2237]
infB	-2.33	13.05	-1.22	13.66	0.0	12.44	0.1	0.00049932	0.00481991	A_07_P000561	b3168	cmr|protein chain initiation factor IF-2 [b3168]
infC	-3.27	14.77	-1.71	15.63	0.0	13.91	0.0	0.00008682	0.00143726	A_07_P018636	b1718	cmr|protein chain initiation factor IF-3 [b1718]
insA	-3.41	9.09	-1.77	9.97	0.1	8.20	0.1	0.00009048	0.00147268	A_07_P008028	b0265	cmr|IS1 protein InsA [b0265]
insA	-4.05	10.16	-2.02	11.17	0.0	9.15	0.1	0.00007937	0.00135096	A_07_P003998	b4294	cmr|IS1 protein InsA [b4294]
insB	-2.80	9.45	-1.48	10.19	0.3	8.71	0.2	0.00322447	0.01897086	A_07_P005381	b0988	cmr|IS1 protein InsB [b0988]
insB	-3.66	9.51	-1.87	10.45	0.0	8.57	0.3	0.00038104	0.00398343	A_07_P010675	b0021	cmr|IS1 protein InsB [b0021]
insD	-2.87	12.25	-1.52	13.01	0.2	11.49	0.3	0.00179736	0.01225015	A_07_P020927	b0361	cmr|IS2 hypothetical protein [b0361]
insG	-20.13	10.77	-4.33	12.94	0.0	8.61	0.1	0.00000103	0.00020391	A_07_P003924	b4278	cmr|IS4 hypothetical protein [b4278]
iroN	-5.50	8.86	-2.46	10.09	0.1	7.63	0.3	0.00011805	0.00175334	A_07_P040541	c_1250	cmr|Siderophore receptor IroN [c_1250]
iscA	-4.70	11.42	-2.23	12.53	0.1	10.30	0.1	0.00006458	0.00118644	A_07_P019544	b2528	cmr|putative regulator [b2528]
iscR	-3.31	10.69	-1.73	11.55	0.1	9.82	0.1	0.00012327	0.00180375	A_07_P019560	b2531	cmr|orf, hypothetical protein [b2531]
iscS	-2.12	12.19	-1.08	12.74	0.1	11.65	0.1	0.00096057	0.00770017	A_07_P019553	b2530	cmr|putative aminotransferase [b2530]
iscX	-2.28	12.45	-1.19	13.04	0.1	11.85	0.0	0.00055147	0.00519904	A_07_P013676	b2524	cmr|orf, hypothetical protein [b2524]
ispG	+2.09	10.08	+1.07	9.54	0.2	10.61	0.1	0.00201586	0.01322404	A_07_P013631	b2515	cmr|orf, hypothetical protein [b2515]
iucC	-2.09	8.38	-1.06	8.91	0.1	7.84	0.1	0.00116126	0.00888380	A_07_P036313	c_3625	cmr|IucC protein [c_3625]
iutA	-2.44	8.29	-1.29	8.93	0.0	7.64	0.2	0.00086890	0.00722279	A_07_P036302	c_3623	cmr|IutA protein [c_3623]
katG	+3.42	10.20	+1.77	9.31	0.1	11.08	0.0	0.00008109	0.00137285	A_07_P019488	b3942	cmr|catalase; hydroperoxidase HPI [b3942]
kbaZ	+4.97	8.17	+2.31	7.01	0.1	9.32	0.0	0.00002838	0.00074722	A_07_P000381	b3132	cmr|putative tagatose 6-phosphate kinase 2 [b3132]
kbl	+16.32	11.09	+4.03	9.07	0.1	13.10	0.1	0.00000363	0.00030703	A_07_P017030	b3617	cmr|2-amino-3-ketobutyrate CoA ligase [b3617]
kdpA	-2.70	8.68	-1.43	9.40	0.1	7.96	0.0	0.00025130	0.00299733	A_07_P010006	b0698	cmr|ATPase of high-affinity potassium transport system, A chain [b0698]
kdsA	-2.32	11.91	-1.21	12.51	0.0	11.30	0.1	0.00049464	0.00478647	A_07_P007028	b1215	cmr|2-dehydro-3-deoxyphosphooctulonate aldolase [b1215]
kdsC	-2.09	13.04	-1.07	13.57	0.0	12.51	0.2	0.00181527	0.01230406	A_07_P012468	b3198	cmr|orf, hypothetical protein [b3198]
kefA	-2.56	8.72	-1.36	9.40	0.0	8.04	0.1	0.00041635	0.00423720	A_07_P021433	b0465	cmr|putative alpha helix protein [b0465]
kpsC	+2.00	7.42	+1.00	6.92	0.0	7.92	0.1	0.00195408	0.01294824	A_07_P036625	c_3690	cmr|KpsC protein [c_3690]
kpsE	+2.96	9.24	+1.56	8.46	0.1	10.02	0.1	0.00028602	0.00327271	A_07_P036606	c_3687	cmr|KpsE protein [c_3687]
kpsS	-2.06	8.57	-1.04	9.09	0.2	8.05	0.1	0.00266590	0.01640169	A_07_P036626	c_3691	cmr|KpsS protein [c_3691]
kpsT	+2.87	9.99	+1.52	9.23	0.1	10.75	0.3	0.00117822	0.00896134	A_07_P036658	c_3697	cmr|KpsT protein [c_3697]
ksgA	-2.53	11.81	-1.34	12.48	0.0	11.14	0.1	0.00032451	0.00357524	A_07_P010944	b0051	cmr|S-adenosylmethionine-6-N,N-adenosyl [b0051]
L7002	-4.30	12.40	-2.10	13.45	0.4	11.35	0.2	0.00121065	0.00912855	A_07_P048661	ABH-0243999	asap|unknown [L7002]
L7055	-2.85	10.97	-1.51	11.73	0.1	10.22	0.2	0.00050036	0.00482696	A_07_P048893	ABH-0244096	asap|replication protein [L7055]
L7079	-2.00	11.65	-1.00	12.15	0.0	11.15	0.1	0.00233153	0.01478359	A_07_P048996	ABH-0244148	asap|unknown [L7079]
lacI	+2.81	8.40	+1.49	7.65	0.1	9.14	0.1	0.00039006	0.00404551	A_07_P008419	b0345	cmr|transcriptional repressor of the lac operon [b0345]
lacZ	+2.39	7.68	+1.26	7.05	0.2	8.31	0.1	0.00118095	0.00897338	A_07_P031027	c_0459	cmr|Beta-galactosidase [c_0459]
lamB	+14.52	9.75	+3.86	7.82	0.1	11.68	0.1	0.00000210	0.00026053	A_07_P008822	b4036	cmr|phage lambda receptor protein; maltose high-affinity receptor [b4036]
lamB	+14.30	9.25	+3.84	7.33	0.0	11.17	0.1	0.00000274	0.00028012	A_07_P037538	c_5006	cmr|Maltoporin precursor [c_5006]
leuB	+2.14	9.36	+1.10	8.81	0.0	9.91	0.2	0.00275200	0.01675359	A_07_P035745	c_0090	cmr|3-isopropylmalate dehydrogenase [c_0090]
leuE	-9.69	8.94	-3.28	10.57	0.1	7.30	0.2	0.00001564	0.00056283	A_07_P015685	b1798	cmr|orf, hypothetical protein [b1798]
ligB	-2.88	8.07	-1.53	8.83	0.0	7.30	0.1	0.00030196	0.00338846	A_07_P017180	b3647	cmr|putative enzyme [b3647]
ligT	-2.81	10.45	-1.49	11.20	0.1	9.71	0.1	0.00040385	0.00415342	A_07_P002698	b0147	cmr|orf, hypothetical protein [b0147]
lipA	+2.30	12.86	+1.20	12.26	0.0	13.46	0.1	0.00060601	0.00553423	A_07_P009709	b0628	cmr|lipoate synthesis, sulfur insertion? [b0628]
lipB	-7.99	12.43	-3.00	13.93	0.0	10.93	0.0	0.00000513	0.00034758	A_07_P009718	b0630	cmr|protein of lipoate biosynthesis [b0630]
livK	-4.49	8.09	-2.17	9.17	0.1	7.00	0.2	0.00007699	0.00132177	A_07_P034548	c_4248	cmr|Leucine-specific binding protein precursor [c_4248]
lldD	+4.56	8.65	+2.19	7.56	0.5	9.75	0.1	0.00107515	0.00836324	A_07_P016968	b3605	cmr|L-lactate dehydrogenase [b3605]
lldP	+2.29	7.12	+1.20	6.52	0.1	7.72	0.1	0.00094727	0.00763638	A_07_P016959	b3603	cmr|L-lactate permease [b3603]
lldR	+3.30	8.21	+1.72	7.35	0.1	9.07	0.1	0.00016858	0.00225120	A_07_P016965	b3604	cmr|transcriptional regulator [b3604]
lolA	+2.32	9.41	+1.21	8.80	0.0	10.01	0.0	0.00051250	0.00491401	A_07_P004904	b0891	cmr|periplasmic protein effects translocation of lipoproteins from inner membrane to outer [b0891]
lolC	-2.26	9.95	-1.18	10.54	0.2	9.36	0.0	0.00119454	0.00904608	A_07_P040818	c_1391	cmr|Lipoprotein releasing system transmembrane protein lolC [c_1391]
lpcA	+5.18	10.73	+2.37	9.54	0.0	11.91	0.0	0.00001716	0.00058318	A_07_P007824	b0222	cmr|phosphoheptose isomerase [b0222]
lpxA	-2.59	11.68	-1.37	12.36	0.0	10.99	0.0	0.00025108	0.00299696	A_07_P002865	b0181	cmr|UDP-N-acetylglucosamine acetyltransferase; lipid A biosynthesis [b0181]
lpxB	-2.42	10.59	-1.27	11.22	0.1	9.95	0.1	0.00080810	0.00682912	A_07_P002870	b0182	cmr|tetraacyldisaccharide-1-P; lipid A biosynthesis, penultimate step [b0182]
lpxC	-6.01	14.46	-2.59	15.76	0.0	13.17	0.0	0.00001094	0.00047993	A_07_P002451	b0096	cmr|UDP-3-O-acyl N-acetylglucosamine deacetylase; lipid A biosynthesis [b0096]
lpxK	-4.40	10.09	-2.14	11.16	0.0	9.02	0.1	0.00004195	0.00093286	A_07_P005022	b0915	cmr|putative EC 1.2 enzyme [b0915]
lpxL	-3.90	11.64	-1.96	12.62	0.0	10.65	0.0	0.00004405	0.00094686	A_07_P001480	b1054	cmr|heat shock protein [b1054]
lpxP	-2.33	11.53	-1.22	12.14	0.0	10.92	0.1	0.00048620	0.00473388	A_07_P014174	b2378	cmr|putative heat shock protein [b2378]
lrhA	-4.38	11.83	-2.13	12.90	0.0	10.77	0.0	0.00002789	0.00073912	A_07_P013734	b2289	cmr|NADH dehydrogenase transcriptional regulator, LysR family [b2289]
lrp	+3.59	9.83	+1.84	8.90	0.1	10.75	0.1	0.00016627	0.00223475	A_07_P004893	b0889	cmr|regulator for leucine [b0889]
lsrK	-2.15	9.23	-1.11	9.79	0.3	8.68	0.1	0.00664756	0.03266514	A_07_P020203	b1511	cmr|putative kinase [b1511]
ltaE	+3.19	9.10	+1.67	8.26	0.0	9.94	0.0	0.00010256	0.00160236	A_07_P004805	b0870	cmr|putative arylsulfatase [b0870]
lysS	-2.78	11.64	-1.47	12.38	0.1	10.90	0.0	0.00020470	0.00259073	A_07_P006416	b2890	cmr|lysine tRNA synthetase, constitutive; suppressor of ColE1 mutation in primer RNA [b2890]
lyxK	+2.37	7.50	+1.25	6.87	0.1	8.12	0.1	0.00067415	0.00595430	A_07_P039671	c_4403	cmr|Cryptic L-xylulose kinase [c_4403]
maa	-7.32	9.57	-2.87	11.01	0.1	8.14	0.0	0.00000978	0.00045680	A_07_P021405	b0459	cmr|putative transferase [b0459]
macB	-2.50	8.55	-1.32	9.21	0.0	7.88	0.1	0.00037915	0.00397162	A_07_P004848	b0879	cmr|putative ATP-binding component of a transport system [b0879]
maeA	+2.52	8.54	+1.33	7.87	0.1	9.20	0.1	0.00057557	0.00534303	A_07_P009486	b1479	cmr|NAD-linked malate dehydrogenase [b1479]
maeB	+2.10	10.55	+1.07	10.02	0.2	11.09	0.0	0.00229563	0.01464440	A_07_P013371	b2463	cmr|putative multimodular enzyme [b2463]
malE	+6.32	10.18	+2.66	8.85	0.1	11.51	0.0	0.00001716	0.00058318	A_07_P008811	b4034	cmr|periplasmic maltose-binding protein; substrate recognition for transport and chemotaxis [b4034]
malI	-2.28	10.11	-1.19	10.70	0.1	9.51	0.0	0.00098079	0.00781063	A_07_P020735	b1620	cmr|repressor of malX and Y genes [b1620]
malI	-2.56	10.23	-1.36	10.91	0.1	9.55	0.2	0.00108460	0.00842841	A_07_P042320	c_2012	cmr|Maltose regulon Regulatory protein malI [c_2012]
malK	+2.67	7.30	+1.41	6.59	0.0	8.01	0.0	0.00023413	0.00284423	A_07_P008816	b4035	cmr|ATP-binding component of transport system for maltose [b4035]
malP	+6.10	9.15	+2.61	7.85	0.1	10.46	0.1	0.00003540	0.00084182	A_07_P018111	b3417	cmr|maltodextrin phosphorylase [b3417]
malT	+3.71	8.20	+1.89	7.25	0.0	9.14	0.0	0.00005391	0.00107039	A_07_P034438	c_4196	cmr|MalT Regulatory protein [c_4196]
malT	+2.39	7.80	+1.26	7.17	0.0	8.43	0.2	0.00081156	0.00684205	A_07_P018118	b3418	cmr|positive regulator of mal regulon [b3418]
malT	+2.77	8.18	+1.47	7.44	0.1	8.91	0.0	0.00033321	0.00364059	A_07_P052157	Z4774	cmr|positive regulator of mal regulon [Z4774]
malX	+3.80	9.78	+1.93	8.81	0.0	10.74	0.0	0.00004926	0.00100666	A_07_P020740	b1621	cmr|PTS system, maltose and glucose-specific II ABC [b1621]
malY	+2.35	9.10	+1.23	8.48	0.1	9.71	0.1	0.00117144	0.00893567	A_07_P042322	c_2014	cmr|MalY protein [c_2014]
manA	+2.37	8.82	+1.25	8.20	0.1	9.45	0.1	0.00093165	0.00756060	A_07_P020700	b1613	cmr|mannose-6-phosphate isomerase [b1613]
manA	+2.66	8.73	+1.41	8.02	0.1	9.44	0.0	0.00033738	0.00367597	A_07_P044397	Z2616	cmr|mannose-6-phosphate isomerase [Z2616]
manA	+2.32	8.73	+1.22	8.12	0.0	9.34	0.1	0.00051652	0.00494954	A_07_P035096	c_2005	cmr|Mannose-6-phosphate isomerase [c_2005]
manB	+2.03	10.47	+1.02	9.96	0.1	10.98	0.1	0.00223846	0.01436435	A_07_P045001	Z3194	cmr|phosphomannomutase [Z3194]
manB	+2.50	12.60	+1.32	11.94	0.1	13.26	0.0	0.00053522	0.00507319	A_07_P031986	c_2557	cmr|Phosphomannomutase [c_2557]
manX	+7.29	9.72	+2.87	8.29	0.0	11.16	0.2	0.00001659	0.00057541	A_07_P015778	b1817	cmr|PTS enzyme IIAB, mannose-specific [b1817]
manY	+3.06	9.29	+1.62	8.48	0.1	10.10	0.1	0.00019173	0.00246301	A_07_P015784	b1818	cmr|PTS enzyme IIC, mannose-specific [b1818]
manZ	+3.25	9.41	+1.70	8.56	0.2	10.25	0.1	0.00037537	0.00395400	A_07_P015786	b1819	cmr|PTS enzyme IID, mannose-specific [b1819]
marA	+6.16	10.41	+2.62	9.10	0.1	11.73	0.0	0.00001913	0.00061714	A_07_P020303	b1531	cmr|multiple antibiotic resistance; transcriptional activator of defense systems [b1531]
marB	+2.16	8.72	+1.11	8.16	0.1	9.27	0.1	0.00126315	0.00941621	A_07_P020307	b1532	cmr|multiple antibiotic resistance protein [b1532]
marR	+2.71	11.63	+1.44	10.91	0.2	12.35	0.1	0.00143201	0.01031835	A_07_P020299	b1530	cmr|multiple antibiotic resistance protein; repressor of mar operon [b1530]
matA	-2.13	9.36	-1.09	9.91	0.3	8.82	0.1	0.00895348	0.04122947	A_07_P041479	c_0405	cmr|Hypothetical protein ykgK [c_0405]
mchD	-2.50	11.64	-1.32	12.30	0.4	10.98	0.0	0.00451509	0.02431941	A_07_P040445	c_1230	cmr|MchD protein [c_1230]
mdaB	+5.71	9.81	+2.51	8.55	0.2	11.06	0.2	0.00009718	0.00154382	A_07_P012273	b3028	cmr|modulator of drug activity B [b3028]
mdh	+6.74	11.61	+2.75	10.24	0.0	12.99	0.0	0.00000798	0.00041039	A_07_P012651	b3236	cmr|malate dehydrogenase [b3236]
mdoC	-2.69	8.02	-1.43	8.73	0.0	7.31	0.1	0.00023076	0.00281851	A_07_P001448	b1047	cmr|orf, hypothetical protein [b1047]
mdoG	-2.52	10.95	-1.33	11.62	0.0	10.29	0.1	0.00040389	0.00415342	A_07_P001451	b1048	cmr|periplasmic glucans biosynthesis protein [b1048]
mdtH	-2.77	9.53	-1.47	10.26	0.3	8.79	0.2	0.00227272	0.01453359	A_07_P001526	b1065	cmr|orf, hypothetical protein [b1065]
mdtK	-3.18	11.17	-1.67	12.01	0.0	10.34	0.0	0.00009294	0.00149610	A_07_P018376	b1663	cmr|putative transport protein [b1663]
mdtL	-2.24	7.98	-1.16	8.56	0.3	7.40	0.1	0.00644569	0.03192183	A_07_P005475	b3710	cmr|putative transport protein [b3710]
mdtQ	-2.16	7.07	-1.11	7.62	0.0	6.51	0.0	0.00066069	0.00587078	A_07_P003459	b2139	cmr|orf, hypothetical protein [b2139]
mdtQ	-2.23	8.52	-1.16	9.10	0.1	7.94	0.1	0.00107124	0.00834272	A_07_P010504	b2139	cmr|orf, hypothetical protein [b2139]
melA	+2.85	8.83	+1.51	8.08	0.1	9.59	0.2	0.00069434	0.00608327	A_07_P011333	b4119	cmr|alpha-galactosidase [b4119]
menB	+5.33	9.64	+2.41	8.43	0.1	10.85	0.1	0.00001893	0.00061693	A_07_P001100	b2262	cmr|dihydroxynaphtoic acid synthetase [b2262]
menC	-2.39	12.52	-1.26	13.15	0.1	11.89	0.1	0.00108530	0.00842964	A_07_P001094	b2261	cmr|o-succinylbenzoyl-CoA synthase; conversion of chorismate to 2-o-succinylbenzoyl-CoA [b2261]
menD	+2.25	8.95	+1.17	8.37	0.2	9.54	0.1	0.00163683	0.01139267	A_07_P032389	c_2808	cmr|Menaquinone biosynthesis protein menD [c_2808]
metB	+2.53	8.89	+1.34	8.22	0.2	9.56	0.1	0.00091526	0.00747785	A_07_P019474	b3939	cmr|cystathionine gamma-synthase [b3939]
metH	+2.49	8.12	+1.32	7.46	0.3	8.78	0.1	0.00253811	0.01578201	A_07_P057969	ECs4937	cmr|B12-dependent homocysteine-N5-methyltetrahydrofolate transmethylase [ECs4937]
metH	+2.99	8.48	+1.58	7.69	0.0	9.27	0.0	0.00013299	0.00190006	A_07_P047219	Z5610	cmr|B12-dependent homocysteine-N5-methyltetrahydrofolate transmethylase, repressor of metE and [Z5610]
metH	+2.52	9.11	+1.33	8.44	0.1	9.77	0.1	0.00069653	0.00609566	A_07_P037467	c_4976	cmr|5-methyltetrahydrofolate--homocysteine methyltransferase [c_4976]
metN	-3.64	10.73	-1.87	11.66	0.1	9.80	0.1	0.00009758	0.00154382	A_07_P002955	b0199	cmr|ATP-binding component of a transporter [b0199]
mfd	+2.16	7.72	+1.11	7.16	0.1	8.27	0.0	0.00119228	0.00903331	A_07_P040806	c_1389	cmr|Transcription-repair coupling factor [c_1389]
mglA	+5.38	8.39	+2.43	7.17	0.1	9.60	0.1	0.00002243	0.00067002	A_07_P003507	b2149	cmr|ATP-binding component of methyl-galactoside transport and galactose taxis [b2149]
mglB	+13.26	9.19	+3.73	7.32	0.3	11.05	0.0	0.00001472	0.00054604	A_07_P003515	b2150	cmr|galactose-binding transport protein; receptor for galactose taxis [b2150]
mglB	+15.81	10.19	+3.98	8.19	0.0	12.18	0.0	0.00000138	0.00022125	A_07_P032239	c_2684	cmr|D-galactose-binding periplasmic protein precursor [c_2684]
mglB	+17.45	10.29	+4.12	8.22	0.1	12.35	0.0	0.00000117	0.00021114	A_07_P045485	Z3405	cmr|galactose-binding transport protein receptor for galactose taxis [Z3405]
mglC	+2.87	8.62	+1.52	7.86	0.0	9.38	0.1	0.00021945	0.00271616	A_07_P003504	b2148	cmr|methyl-galactoside transport and galactose taxis [b2148]
mgsA	+5.31	10.33	+2.41	9.12	0.2	11.53	0.1	0.00006353	0.00117531	A_07_P005264	b0963	cmr|methylglyoxal synthase [b0963]
mgtA	-37.54	11.77	-5.23	14.38	0.0	9.15	0.1	0.00000059	0.00019431	A_07_P003761	b4242	cmr|Mg2+ transport ATPase, P-type 1 [b4242]
miaA	+3.11	13.07	+1.64	12.26	0.0	13.89	0.0	0.00010557	0.00163561	A_07_P011571	b4171	cmr|delta [b4171]
minD	+2.18	10.21	+1.13	9.65	0.2	10.78	0.2	0.00337708	0.01962668	A_07_P006827	b1175	cmr|cell division inhibitor, a membrane ATPase, activates minC [b1175]
minE	+3.31	11.96	+1.73	11.09	0.1	12.82	0.0	0.00011655	0.00173927	A_07_P006821	b1174	cmr|cell division topological specificity factor, reverses MinC inhibition of ftsZ ring formation [b1174]
mioC	-4.91	13.92	-2.30	15.07	0.0	12.77	0.0	0.00002079	0.00063916	A_07_P005634	b3742	cmr|initiation of chromosome replication [b3742]
mipA	+4.48	11.81	+2.16	10.73	0.1	12.90	0.0	0.00003767	0.00087581	A_07_P015602	b1782	cmr|orf, hypothetical protein [b1782]
mltD	+3.16	10.65	+1.66	9.82	0.2	11.48	0.0	0.00023069	0.00281851	A_07_P002982	b0211	cmr|transcriptional regulator for nitrite reductase [b0211]
mntH	-7.86	9.79	-2.97	11.28	0.2	8.31	0.1	0.00003191	0.00079613	A_07_P014241	b2392	cmr|putative transport system permease [b2392]
moaB	+2.58	10.27	+1.37	9.59	0.1	10.96	0.1	0.00044460	0.00444871	A_07_P004361	b0782	cmr|molybdopterin biosynthesis, protein B [b0782]
mobA	+3.44	8.19	+1.78	7.30	0.4	9.08	0.1	0.00182312	0.01232958	A_07_P039291	c_4801	cmr|Molybdopterin-guanine dinucleotide biosynthesis protein A [c_4801]
moeA	-2.11	11.36	-1.08	11.90	0.0	10.82	0.1	0.00097952	0.00780447	A_07_P004586	b0827	cmr|molybdopterin biosynthesis [b0827]
moeB	-2.39	9.06	-1.26	9.69	0.1	8.43	0.1	0.00077461	0.00661362	A_07_P004583	b0826	cmr|molybdopterin biosynthesis [b0826]
mokC	-3.55	12.36	-1.83	13.27	0.1	11.44	0.0	0.00012129	0.00178598	A_07_P010256	b0018	cmr|Gef protein interferes with membrane function when in excess [b0018]
mokC	-2.41	10.92	-1.27	11.55	0.1	10.28	0.0	0.00040051	0.00412947	A_07_P010779	b0018	cmr|Gef protein interferes with membrane function when in excess [b0018]
mokP	-2.60	15.08	-1.38	15.77	0.0	14.39	0.4	0.00414157	0.02281485	A_07_P044279	Z6063	cmr|putative cell killing protein encoded within cryptic [Z6063]
motB	-2.96	8.42	-1.57	9.20	0.0	7.63	0.2	0.00033299	0.00364059	A_07_P016121	b1889	cmr|enables flagellar motor rotation, linking torque machinery to cell wall [b1889]
mpl	-2.24	12.34	-1.16	12.92	0.0	11.75	0.0	0.00059428	0.00547004	A_07_P003716	b4233	cmr|putative ligase [b4233]
mraZ	-3.20	12.31	-1.68	13.15	0.0	11.47	0.1	0.00012419	0.00181541	A_07_P011082	b0081	cmr|orf, hypothetical protein [b0081]
mrdA	-2.10	8.81	-1.07	9.35	0.1	8.28	0.1	0.00145651	0.01044487	A_07_P009743	b0635	cmr|cell elongation, e phase; peptidoglycan synthetase; penicillin-binding protein 2 [b0635]
msbA	-4.43	10.26	-2.15	11.33	0.0	9.18	0.0	0.00002776	0.00073912	A_07_P005017	b0914	cmr|ATP-binding transport protein; multicopy suppressor of htrB [b0914]
mscL	-2.02	12.30	-1.02	12.81	0.0	11.79	0.1	0.00123814	0.00926487	A_07_P034268	c_4051	cmr|Large-conductance mechanosensitive channel [c_4051]
msrA	+3.49	8.69	+1.80	7.78	0.1	9.59	0.0	0.00012626	0.00183207	A_07_P003647	b4219	cmr|peptide methionine sulfoxide reductase [b4219]
msrB	+6.54	13.44	+2.71	12.09	0.1	14.80	0.1	0.00001334	0.00052521	A_07_P015584	b1778	cmr|orf, hypothetical protein [b1778]
msyB	+2.68	10.26	+1.42	9.55	0.1	10.97	0.0	0.00042529	0.00430593	A_07_P040763	c_1318	cmr|Acidic protein msyB [c_1318]
mtfA	+2.64	7.68	+1.40	6.98	0.1	8.38	0.0	0.00033204	0.00363536	A_07_P015234	b1976	cmr|orf, hypothetical protein [b1976]
mtlA	+2.79	7.99	+1.48	7.25	0.0	8.73	0.1	0.00033997	0.00369388	A_07_P049516	Z5023	cmr|PTS system, mannitol-specific enzyme IIABC components [Z5023]
mtlA	+3.51	8.27	+1.81	7.37	0.1	9.18	0.2	0.00018306	0.00238590	A_07_P039704	c_4416	cmr|PTS system, mannitol-specific IIABC component [c_4416]
mtlD	+2.58	8.79	+1.37	8.11	0.0	9.47	0.2	0.00056596	0.00528183	A_07_P039708	c_4417	cmr|Mannitol-1-phosphate 5-dehydrogenase [c_4417]
murI	-3.44	10.51	-1.78	11.40	0.1	9.62	0.1	0.00014487	0.00202744	A_07_P008540	b3967	cmr|glutamate racemase, required for biosynthesis of D-glutamate and peptidoglycan [b3967]
mutH	-2.00	8.47	-1.00	8.97	0.0	7.97	0.1	0.00169517	0.01170047	A_07_P041691	c_3426	cmr|DNA mismatch repair protein mutH [c_3426]
mutM	-4.02	9.79	-2.01	10.80	0.0	8.79	0.1	0.00006458	0.00118644	A_07_P017118	b3635	cmr|formamidopyrimidine DNA glycosylase [b3635]
mutY	-2.91	12.02	-1.54	12.79	0.5	11.24	0.0	0.00636244	0.03164936	A_07_P011946	b2961	cmr|adenine glycosylase; G.C --> T.A transversions [b2961]
nac	-10.12	9.31	-3.34	10.98	0.1	7.64	0.0	0.00000593	0.00036511	A_07_P015279	b1988	cmr|nitrogen assimilation control protein [b1988]
nadA	-2.27	8.47	-1.18	9.06	0.2	7.88	0.1	0.00214651	0.01390728	A_07_P033430	c_0825	cmr|Quinolinate synthetase A [c_0825]
nadA	-2.04	8.70	-1.03	9.22	0.0	8.19	0.1	0.00163609	0.01139259	A_07_P046361	Z0919	cmr|quinolinate synthetase, A protein [Z0919]
nadA	-2.09	7.90	-1.06	8.43	0.1	7.37	0.1	0.00158224	0.01112091	A_07_P010232	b0750	cmr|quinolinate synthetase, A protein [b0750]
nadE	+5.78	9.53	+2.53	8.26	0.0	10.79	0.1	0.00001397	0.00053770	A_07_P018749	b1740	cmr|NAD synthetase, prefers NH3 over glutamine [b1740]
nadR	+2.03	9.17	+1.02	8.66	0.3	9.68	0.1	0.00998600	0.04470339	A_07_P017631	b4390	cmr|probable nadAB transcriptional regulator [b4390]
nagA	+2.43	9.44	+1.28	8.80	0.2	10.08	0.3	0.00365441	0.02080082	A_07_P009903	b0677	cmr|N-acetylglucosamine-6-phosphate deacetylase [b0677]
nagB	+2.20	8.40	+1.14	7.84	0.1	8.97	0.1	0.00112795	0.00868811	A_07_P009910	b0678	cmr|glucosamine-6-phosphate deaminase [b0678]
nagE	-2.05	11.96	-1.04	12.48	0.0	11.44	0.0	0.00093463	0.00757320	A_07_P009911	b0679	cmr|PTS system, N-acetylglucosamine-specific enzyme IIABC [b0679]
nagZ	+4.52	9.08	+2.18	8.00	0.0	10.17	0.0	0.00002780	0.00073912	A_07_P001737	b1107	cmr|orf, hypothetical protein [b1107]
nanA	+3.00	8.58	+1.58	7.78	0.4	9.37	0.2	0.00401424	0.02226925	A_07_P041797	c_3979	cmr|N-acetylneuraminate lyase subunit [c_3979]
nanA	+2.02	7.70	+1.01	7.20	0.1	8.21	0.1	0.00241642	0.01521155	A_07_P012599	b3225	cmr|N-acetylneuraminate lyase [b3225]
nanE	+2.91	8.15	+1.54	7.38	0.0	8.92	0.0	0.00015057	0.00208675	A_07_P012586	b3223	cmr|putative enzyme [b3223]
nanK	+2.85	7.43	+1.51	6.67	0.0	8.18	0.1	0.00021006	0.00262893	A_07_P012584	b3222	cmr|putative NAGC-like transcriptional regulator [b3222]
napB	+2.63	8.90	+1.40	8.20	0.1	9.60	0.1	0.00038737	0.00402821	A_07_P000804	b2203	cmr|cytochrome c-type protein [b2203]
napF	-4.02	11.13	-2.01	12.13	0.1	10.13	0.0	0.00004892	0.00100424	A_07_P000827	b2208	cmr|ferredoxin-type protein: electron transfer [b2208]
narL	+2.89	10.98	+1.53	10.22	0.2	11.75	0.0	0.00032707	0.00359843	A_07_P052433	Z1996	cmr|pleiotrophic regulation of anaerobic respiration: response regulator [Z1996]

narP	+2.81	8.13	+1.49	7.38	0.3	8.88	0.2	0.00179841	0.01225015	A_07_P000752	b2193	cmr|nitrate/nitrite response regulator [b2193]
ndk	+9.19	11.48	+3.20	9.88	0.1	13.08	0.0	0.00000529	0.00034950	A_07_P013646	b2518	cmr|nucleoside diphosphate kinase [b2518]
nemA	+4.44	10.47	+2.15	9.39	0.0	11.54	0.0	0.00003007	0.00077228	A_07_P020882	b1650	cmr|N-ethylmaleimide reductase [b1650]
nfi	+7.47	9.81	+2.90	8.36	0.1	11.26	0.1	0.00000805	0.00041039	A_07_P037441	c_4955	cmr|Endonuclease V [c_4955]
nfi	+6.35	9.39	+2.67	8.05	0.0	10.72	0.1	0.00001145	0.00049545	A_07_P008655	b3998	cmr|endonuclease V [b3998]
nfi	+3.56	9.41	+1.83	8.50	0.3	10.33	0.2	0.00132405	0.00972818	A_07_P047202	Z5574	cmr|endonuclease V [Z5574]
nfo	+3.03	8.50	+1.60	7.70	0.1	9.30	0.1	0.00040715	0.00416878	A_07_P003556	b2159	cmr|endonuclease IV [b2159]
nhaA	-7.26	11.57	-2.86	13.00	0.0	10.14	0.0	0.00000666	0.00038136	A_07_P010784	b0019	cmr|Na+/H antiporter, pH dependent [b0019]
nhaR	-3.26	9.55	-1.71	10.41	0.1	8.70	0.1	0.00015405	0.00212192	A_07_P035651	c_0025	cmr|Transcriptional activator protein nhaR [c_0025]
nikA	+2.62	7.70	+1.39	7.01	0.2	8.40	0.0	0.00099420	0.00788955	A_07_P007316	b3476	cmr|periplasmic binding protein for nickel [b3476]
nlpA	-4.40	8.58	-2.14	9.65	0.1	7.51	0.1	0.00005542	0.00108517	A_07_P017245	b3661	cmr|lipoprotein-28 [b3661]
nlpI	-5.78	14.84	-2.53	16.11	0.0	13.57	0.0	0.00001303	0.00052185	A_07_P000538	b3163	cmr|putative control proteins [b3163]
nmpC	+17.13	10.40	+4.10	8.36	0.5	12.45	0.0	0.00009575	0.00152759	A_07_P038497	c_1560	cmr|Outer membrane porin protein nmpC precursor [c_1560]
nohA	+7.14	10.37	+2.84	8.95	0.0	11.79	0.0	0.00000700	0.00039376	A_07_P038523	c_1568	cmr|Prophage Qin DNA packaging protein NU1 homolog [c_1568]
nrdA	+50.08	10.76	+5.65	7.93	0.1	13.58	0.1	0.00000034	0.00019431	A_07_P000956	b2234	cmr|ribonucleoside diphosphate reductase 1, alpha subunit, B1 [b2234]
nrdB	+12.18	10.65	+3.61	8.85	0.1	12.45	0.1	0.00000264	0.00027706	A_07_P000962	b2235	cmr|ribonucleoside-diphosphate reductase 1, beta subunit, B2 [b2235]
nrdD	+26.15	12.76	+4.71	10.41	0.1	15.12	0.1	0.00000105	0.00020472	A_07_P003742	b4238	cmr|anaerobic ribonucleoside-triphosphate reductase [b4238]
nrdD	+24.22	13.21	+4.60	10.91	0.0	15.51	0.1	0.00000127	0.00021491	A_07_P042066	c_5337	cmr|Anaerobic ribonucleoside-triphosphate reductase [c_5337]
nrdG	+11.44	10.17	+3.52	8.41	0.1	11.93	0.0	0.00000361	0.00030683	A_07_P003737	b4237	cmr|anaerobic ribonucleotide reductase activating protein [b4237]
nrdR	-3.38	13.39	-1.76	14.27	0.1	12.52	0.0	0.00008374	0.00140699	A_07_P021179	b0413	cmr|orf, hypothetical protein [b0413]
nudB	+2.87	10.81	+1.52	10.05	0.1	11.57	0.1	0.00025418	0.00302024	A_07_P016003	b1865	cmr|dATP pyrophosphohydrolase [b1865]
nudB	+2.92	11.13	+1.54	10.36	0.1	11.90	0.0	0.00026659	0.00310509	A_07_P016003	b1865	cmr|dATP pyrophosphohydrolase [b1865]
nudB	+2.79	11.06	+1.48	10.32	0.0	11.80	0.0	0.00018260	0.00238373	A_07_P016003	b1865	cmr|dATP pyrophosphohydrolase [b1865]
nudB	+3.25	11.03	+1.70	10.18	0.0	11.88	0.1	0.00012189	0.00179019	A_07_P016003	b1865	cmr|dATP pyrophosphohydrolase [b1865]
nudB	+2.87	10.86	+1.52	10.10	0.0	11.62	0.1	0.00017515	0.00231344	A_07_P016003	b1865	cmr|dATP pyrophosphohydrolase [b1865]
nudC	+2.14	9.65	+1.10	9.11	0.1	10.20	0.0	0.00118980	0.00902756	A_07_P008643	b3996	cmr|orf, hypothetical protein [b3996]
nudC	+2.18	9.59	+1.13	9.02	0.1	10.15	0.1	0.00090540	0.00740498	A_07_P063685	ECs4919	cmr|hypothetical protein [ECs4919]
nudE	-2.29	10.61	-1.20	11.21	0.1	10.01	0.1	0.00073152	0.00632113	A_07_P018014	b3397	cmr|orf, hypothetical protein [b3397]
nudE	-2.19	11.01	-1.13	11.57	0.1	10.44	0.1	0.00078930	0.00670628	A_07_P059766	ECs4239	cmr|hypothetical protein [ECs4239]
nudE	-2.15	11.55	-1.10	12.10	0.0	11.00	0.1	0.00099489	0.00788955	A_07_P046016	Z4751	cmr|orf, hypothetical protein [Z4751]
nudI	+2.78	8.21	+1.48	7.47	0.1	8.95	0.0	0.00022409	0.00275206	A_07_P001045	b2251	cmr|orf, hypothetical protein [b2251]
nudK	+2.41	9.14	+1.27	8.51	0.1	9.78	0.4	0.00782807	0.03723420	A_07_P013393	b2467	cmr|orf, hypothetical protein [b2467]
nuoC	+6.15	10.89	+2.62	9.58	0.0	12.20	0.0	0.00001043	0.00047057	A_07_P013718	b2286	cmr|NADH dehydrogenase I chain C, D [b2286]
nuoE	+5.24	12.78	+2.39	11.59	0.0	13.98	0.0	0.00001624	0.00056947	A_07_P042543	c_2826	cmr|NADH dehydrogenase I chain E [c_2826]
nuoE	+6.88	11.13	+2.78	9.74	0.0	12.53	0.0	0.00000741	0.00040580	A_07_P013712	b2285	cmr|NADH dehydrogenase I chain E [b2285]
nuoE	+4.42	11.16	+2.14	10.09	0.1	12.23	0.0	0.00005581	0.00108886	A_07_P052679	Z3544	cmr|NADH dehydrogenase I chain E [Z3544]
nuoF	+16.05	10.48	+4.00	8.48	0.1	12.48	0.0	0.00000281	0.00028143	A_07_P013708	b2284	cmr|NADH dehydrogenase I chain F [b2284]
nuoG	+6.15	8.62	+2.62	7.31	0.1	9.93	0.3	0.00013043	0.00186858	A_07_P013701	b2283	cmr|NADH dehydrogenase I chain G [b2283]
nuoG	+8.84	9.75	+3.14	8.18	0.0	11.32	0.2	0.00001201	0.00050646	A_07_P045556	Z3542	cmr|NADH dehydrogenase I chain G [Z3542]
nuoH	+18.80	9.64	+4.23	7.52	0.0	11.75	0.1	0.00000164	0.00023759	A_07_P013700	b2282	cmr|NADH dehydrogenase I chain H [b2282]
nuoI	+7.16	9.41	+2.84	7.99	0.7	10.83	0.0	0.00192746	0.01280417	A_07_P013694	b2281	cmr|NADH dehydrogenase I chain I [b2281]
nuoJ	+7.91	9.55	+2.98	8.06	0.3	11.04	0.1	0.00007038	0.00124928	A_07_P013686	b2280	cmr|NADH dehydrogenase I chain J [b2280]
nuoK	+9.89	8.68	+3.31	7.03	0.0	10.34	0.2	0.00001452	0.00054604	A_07_P001181	b2279	cmr|NADH dehydrogenase I chain K [b2279]
nuoK	+9.72	8.84	+3.28	7.20	0.0	10.48	0.2	0.00001564	0.00056283	A_07_P045553	Z3538	cmr|NADH dehydrogenase I chain K [Z3538]
nuoL	+6.25	8.79	+2.64	7.47	0.4	10.11	0.0	0.00018912	0.00244038	A_07_P001178	b2278	cmr|NADH dehydrogenase I chain L [b2278]
nuoM	+6.62	8.54	+2.73	7.18	0.2	9.90	0.0	0.00003127	0.00078813	A_07_P001174	b2277	cmr|NADH dehydrogenase I chain M [b2277]
nuoN	+3.33	7.59	+1.73	6.72	0.1	8.46	0.1	0.00014739	0.00205361	A_07_P051791	Z3534	cmr|NADH dehydrogenase I chain N [Z3534]
nuoN	+2.70	7.74	+1.43	7.03	0.2	8.46	0.1	0.00088384	0.00729692	A_07_P042539	c_2817	cmr|NADH dehydrogenase I chain N [c_2817]
nuoN	+2.41	7.81	+1.27	7.17	0.2	8.45	0.2	0.00228626	0.01459056	A_07_P001166	b2276	cmr|NADH dehydrogenase I chain N [b2276]
nupC	+2.58	8.30	+1.37	7.62	0.4	8.99	0.2	0.00567604	0.02908023	A_07_P014250	b2393	cmr|permease of transport system for 3 nucleosides [b2393]
nupG	+4.40	10.07	+2.14	9.00	0.1	11.14	0.0	0.00003507	0.00083975	A_07_P011964	b2964	cmr|transport of nucleosides, permease protein [b2964]
nusA	-2.85	14.77	-1.51	15.52	0.1	14.01	0.1	0.00023169	0.00282329	A_07_P000566	b3169	cmr|transcription pausing; L factor [b3169]
nusB	-6.99	12.53	-2.81	13.93	0.0	11.12	0.1	0.00000948	0.00044666	A_07_P021194	b0416	cmr|transcription termination; L factor [b0416]
nusG	+2.36	10.21	+1.24	9.58	0.2	10.83	0.0	0.00145006	0.01040556	A_07_P008575	b3982	cmr|component in transcription antitermination [b3982]
obgE	-2.11	10.18	-1.08	10.72	0.1	9.64	0.0	0.00084412	0.00705035	A_07_P012391	b3183	cmr|putative GTP-binding factor [b3183]
ompC	+5.17	9.97	+2.37	8.79	0.1	11.16	0.0	0.00002033	0.00063193	A_07_P032359	c_2758	cmr|Outer membrane protein C precursor [c_2758]
ompC	+4.54	9.14	+2.18	8.05	0.1	10.23	0.1	0.00004795	0.00099062	A_07_P000863	b2215	cmr|outer membrane protein 1b [b2215]
ompC	+2.32	10.38	+1.21	9.77	0.1	10.98	0.0	0.00084123	0.00703741	A_07_P045517	Z3473	cmr|outer membrane protein 1b [Z3473]
ompF	+6.72	10.16	+2.75	8.79	0.1	11.54	0.0	0.00001234	0.00051423	A_07_P005092	b0929	cmr|outer membrane protein 1a [b0929]
ompT	+2.12	8.18	+1.08	7.64	0.3	8.72	0.1	0.00836015	0.03904543	A_07_P031283	c_0652	cmr|Protease VII precursor [c_0652]
ompW	+35.91	9.54	+5.17	6.96	0.0	12.13	0.0	0.00000034	0.00019431	A_07_P007216	b1256	cmr|putative outer membrane protein [b1256]
ompW	+81.78	11.83	+6.35	8.65	0.0	15.00	0.1	0.00000015	0.00019431	A_07_P038872	c_1722	cmr|Outer membrane protein W precursor [c_1722]
ompX	-8.05	13.81	-3.01	15.32	0.0	12.31	0.0	0.00000518	0.00034758	A_07_P004524	b0814	cmr|outer membrane protein X [b0814]
oppA	+16.39	10.14	+4.03	8.12	0.2	12.15	0.0	0.00000556	0.00035456	A_07_P007151	b1243	cmr|oligopeptide transport; periplasmic binding protein [b1243]
oppA	+20.77	11.14	+4.38	8.95	0.0	13.33	0.0	0.00000085	0.00019431	A_07_P052437	Z2019	cmr|oligopeptide transport periplasmic binding protein [Z2019]
oppC	+2.55	7.89	+1.35	7.21	0.0	8.57	0.1	0.00035526	0.00380746	A_07_P007165	b1245	cmr|homolog of Salmonella oligopeptide transport permease protein [b1245]
oppD	+4.85	9.65	+2.28	8.51	0.2	10.79	0.1	0.00013905	0.00196871	A_07_P007166	b1246	cmr|homolog of Salmonella ATP-binding protein of oligopeptide ABC transport system [b1246]
oppF	+5.31	8.71	+2.41	7.50	0.3	9.91	0.0	0.00011319	0.00169854	A_07_P007171	b1247	cmr|homolog of Salmonella ATP-binding protein of oligopeptide ABC transport system [b1247]
osmB	-5.84	8.95	-2.55	10.22	0.1	7.67	0.0	0.00001527	0.00055785	A_07_P014396	b1283	cmr|osmotically inducible lipoprotein [b1283]
osmC	-2.34	10.91	-1.22	11.52	0.0	10.29	0.0	0.00047810	0.00468112	A_07_P009499	b1482	cmr|osmotically inducible protein [b1482]
osmE	+2.32	8.12	+1.22	7.52	0.1	8.73	0.1	0.00065802	0.00585967	A_07_P018744	b1739	cmr|activator of ntrL gene [b1739]
osmY	+2.08	8.62	+1.06	8.10	0.1	9.15	0.2	0.00304110	0.01806079	A_07_P017561	b4376	cmr|hyperosmotically inducible periplasmic protein [b4376]
oxyR	-2.24	13.78	-1.16	14.36	0.0	13.20	0.0	0.00056380	0.00527744	A_07_P008508	b3961	cmr|activator, hydrogen peroxide-inducible genes [b3961]
pal	+2.55	11.45	+1.35	10.78	0.1	12.13	0.0	0.00036801	0.00391653	A_07_P010223	b0741	cmr|peptidoglycan-associated lipoprotein [b0741]
panB	-2.58	10.60	-1.37	11.28	0.0	9.91	0.1	0.00029118	0.00331235	A_07_P002634	b0134	cmr|3-methyl-2-oxobutanoate hydroxymethyltransferase [b0134]
panB	-3.38	12.53	-1.76	13.41	0.0	11.65	0.1	0.00008665	0.00143594	A_07_P035825	c_0165	cmr|3-methyl-2-oxobutanoate hydroxymethyltransferase [c_0165]
panC	-2.08	9.58	-1.05	10.11	0.1	9.05	0.0	0.00144973	0.01040556	A_07_P049200	Z0144	cmr|pantothenate synthetase [Z0144]
panD	+3.69	11.56	+1.88	10.62	0.0	12.50	0.0	0.00005258	0.00105181	A_07_P002617	b0131	cmr|aspartate 1-decarboxylase [b0131]
panE	+2.18	8.95	+1.13	8.39	0.1	9.51	0.0	0.00134374	0.00981706	A_07_P021236	b0425	cmr|involved in thiamin biosynthesis, alternative pyrimidine biosynthesis [b0425]
panF	+2.11	8.39	+1.08	7.85	0.0	8.93	0.1	0.00105817	0.00826384	A_07_P012756	b3258	cmr|sodium/pantothenate symporter [b3258]
papA	+11.49	9.00	+3.52	7.24	0.2	10.76	0.2	0.00002704	0.00073404	A_07_P033164	c_3592	cmr|PapA protein [c_3592]
papC	+4.03	8.97	+2.01	7.97	0.1	9.98	0.1	0.00006678	0.00120840	A_07_P033151	c_3590	cmr|PapC protein [c_3590]
papD_2	+2.71	9.37	+1.44	8.65	0.1	10.09	0.1	0.00049009	0.00476059	A_07_P037957	c_5185	cmr|PapD protein [c_5185]
papE	+2.21	9.28	+1.14	8.71	0.1	9.85	0.1	0.00144027	0.01035891	A_07_P033134	c_3585	cmr|PapE protein [c_3585]
papH	+3.03	10.43	+1.60	9.62	0.3	11.23	0.0	0.00128454	0.00953955	A_07_P033156	c_3591	cmr|PapH protein [c_3591]
papH_2	+6.26	8.94	+2.65	7.62	0.2	10.27	0.1	0.00004446	0.00094837	A_07_P037969	c_5187	cmr|PapH protein [c_5187]
papI_2	+3.11	7.89	+1.63	7.07	0.2	8.71	0.1	0.00060411	0.00552011	A_07_P037980	c_5189	cmr|PapI protein [c_5189]
papK	+2.30	9.56	+1.20	8.96	0.2	10.16	0.0	0.00222564	0.01431980	A_07_P033137	c_3586	cmr|PapK protein [c_3586]
parE	-2.02	11.44	-1.02	11.94	0.3	10.93	0.0	0.00637151	0.03168450	A_07_P012284	b3030	cmr|DNA topoisomerase IV subunit B [b3030]
pbpG	+2.17	7.61	+1.12	7.05	0.3	8.17	0.0	0.00430773	0.02346263	A_07_P003433	b2134	cmr|penicillin-binding protein 7 [b2134]
pck	+4.61	14.13	+2.21	13.03	0.1	15.24	0.1	0.00004630	0.00097245	A_07_P018041	b3403	cmr|phosphoenolpyruvate carboxykinase [b3403]
pcm	-2.39	11.08	-1.25	11.71	0.2	10.46	0.1	0.00094291	0.00761295	A_07_P002163	b2743	cmr|L-isoaspartate protein carboxylmethyltransferase type II [b2743]
pdhR	+2.09	11.82	+1.06	11.28	0.1	12.35	0.1	0.00102525	0.00807477	A_07_P002527	b0113	cmr|transcriptional regulator for pyruvate dehydrogenase complex [b0113]
pdxB	+2.86	10.64	+1.52	9.89	0.0	11.40	0.2	0.00038633	0.00402277	A_07_P013888	b2320	cmr|erythronate-4-phosphate dehyrogenase [b2320]
pdxJ	+3.53	8.99	+1.82	8.08	0.0	9.90	0.0	0.00007021	0.00124769	A_07_P019721	b2564	cmr|pyridoxine biosynthesis [b2564]
pepA	+2.14	12.33	+1.10	11.78	0.0	12.87	0.0	0.00074428	0.00640677	A_07_P003848	b4260	cmr|aminopeptidase A/I [b4260]
pepB	+3.02	9.36	+1.60	8.56	0.0	10.15	0.1	0.00016925	0.00225435	A_07_P013674	b2523	cmr|putative peptidase [b2523]
pepD	+10.85	10.48	+3.44	8.76	0.1	12.20	0.0	0.00000435	0.00033435	A_07_P007896	b0237	cmr|aminoacyl-histidine dipeptidase [b0237]
pepE	+4.95	8.12	+2.31	6.96	0.1	9.27	0.1	0.00003219	0.00079793	A_07_P008748	b4021	cmr|peptidase E, a dipeptidase where amino-terminal residue is aspartate [b4021]
pepN	+5.98	9.11	+2.58	7.82	0.1	10.40	0.0	0.00001598	0.00056395	A_07_P051734	Z1280	cmr|aminopeptidase N [Z1280]
pepT	+4.66	9.20	+2.22	8.09	0.1	10.31	0.2	0.00009097	0.00147798	A_07_P006587	b1127	cmr|putative peptidase T [b1127]
perM	-2.69	9.36	-1.43	10.07	0.1	8.64	0.1	0.00056482	0.00527744	A_07_P030120	c_3009	cmr|Putative permease perM [c_3009]
pfkB	+3.22	9.11	+1.69	8.26	0.1	9.95	0.1	0.00026477	0.00309464	A_07_P041160	c_2121	cmr|6-phosphofructokinase isozyme 2 [c_2121]
pfkB	+3.73	9.26	+1.90	8.31	0.1	10.21	0.0	0.00007728	0.00132533	A_07_P018663	b1723	cmr|6-phosphofructokinase II; suppressor of pfkA [b1723]
pflA	-2.87	12.93	-1.52	13.69	0.0	12.17	0.1	0.00016825	0.00224864	A_07_P004958	b0902	cmr|pyruvate formate lyase activating enzyme 1 [b0902]
pflB	+8.96	10.90	+3.16	9.32	0.1	12.48	0.1	0.00000610	0.00036832	A_07_P004961	b0903	cmr|formate acetyltransferase 1 [b0903]
pgaD	-4.96	9.06	-2.31	10.22	0.0	7.91	0.3	0.00016697	0.00223914	A_07_P001332	b1021	cmr|orf, hypothetical protein [b1021]
pgi	+2.81	8.97	+1.49	8.22	0.3	9.71	0.0	0.00245102	0.01538016	A_07_P008767	b4025	cmr|glucosephosphate isomerase [b4025]
pgk	+4.09	12.73	+2.03	11.71	0.1	13.74	0.0	0.00004322	0.00093866	A_07_P011776	b2926	cmr|phosphoglycerate kinase [b2926]
pgl	+5.67	9.19	+2.50	7.94	0.0	10.44	0.0	0.00001264	0.00051704	A_07_P004288	b0767	cmr|putative isomerase [b0767]
pheS	+3.75	11.21	+1.91	10.26	0.0	12.17	0.2	0.00014064	0.00198761	A_07_P018621	b1714	cmr|phenylalanine tRNA synthetase, alpha-subunit [b1714]
pheT	+2.72	10.23	+1.44	9.51	0.1	10.95	0.1	0.00035229	0.00378335	A_07_P018619	b1713	cmr|phenylalanine tRNA synthetase, beta-subunit [b1713]
phnI	-2.26	11.29	-1.17	11.88	0.1	10.71	0.1	0.00078215	0.00665883	A_07_P011231	b4099	cmr|phosphonate metabolism [b4099]
phoA	-2.59	8.59	-1.37	9.28	0.2	7.91	0.1	0.00076368	0.00654155	A_07_P021029	b0383	cmr|alkaline phosphatase [b0383]
phoB	-5.63	11.81	-2.49	13.06	0.1	10.56	0.1	0.00002330	0.00068274	A_07_P031103	c_0508	cmr|Phosphate regulon transcriptional Regulatory protein phoB [c_0508]
phoB	-5.64	11.51	-2.50	12.76	0.0	10.26	0.0	0.00001293	0.00051944	A_07_P021109	b0399	cmr|positive response regulator for pho regulon, sensor is PhoR [b0399]
phoB	-5.21	11.73	-2.38	12.92	0.0	10.54	0.1	0.00002026	0.00063193	A_07_P043823	Z0497	cmr|positive response regulator for pho regulon, sensor [Z0497]
phoR	-3.16	8.25	-1.66	9.08	0.1	7.42	0.1	0.00020272	0.00257504	A_07_P021115	b0400	cmr|positive and negative sensor protein for pho regulon [b0400]
phoU	+2.14	8.70	+1.10	8.16	0.2	9.25	0.2	0.00386221	0.02172443	A_07_P005542	b3724	cmr|negative regulator for pho regulon and putative enzyme in phosphate metabolism [b3724]
pinR	-2.71	8.49	-1.44	9.20	0.1	7.77	0.0	0.00035336	0.00379046	A_07_P014850	b1374	cmr|putative transposon resolvase [b1374]
pmbA	+2.49	8.70	+1.32	8.04	0.2	9.36	0.4	0.00710987	0.03444804	A_07_P003729	b4235	cmr|maturation of antibiotic MccB17, see tld genes [b4235]
pntA	+4.90	8.57	+2.29	7.42	0.2	9.72	0.0	0.00008477	0.00142126	A_07_P020648	b1603	cmr|pyridine nucleotide transhydrogenase, alpha subunit [b1603]
polA	+3.66	8.60	+1.87	7.66	0.2	9.53	0.1	0.00022615	0.00277298	A_07_P019099	b3863	cmr|DNA polymerase I, 3 --> 5 polymerase, 5 --> 3 and 3 --> 5 exonuclease [b3863]
polA	+2.90	8.68	+1.54	7.91	0.4	9.45	0.1	0.00302621	0.01798592	A_07_P052260	Z5398	cmr|DNA polymerase I, 3 --> 5 polymerase [Z5398]
polB	-5.18	10.47	-2.37	11.66	0.0	9.29	0.1	0.00002414	0.00069739	A_07_P010989	b0060	cmr|DNA polymerase II [b0060]
potA	-2.36	10.73	-1.24	11.35	0.0	10.11	0.1	0.00047964	0.00468366	A_07_P006583	b1126	cmr|ATP-binding component of spermidine/putrescine transport [b1126]
potC	-3.46	11.88	-1.79	12.77	0.0	10.98	0.0	0.00006863	0.00122932	A_07_P006571	b1124	cmr|spermidine/putrescine transport system permease [b1124]
ppdA	+2.88	8.05	+1.53	7.28	0.2	8.81	0.0	0.00058019	0.00537009	A_07_P043261	Z4143	cmr|prepilin peptidase dependent protein A [Z4143]
ppdA	+2.74	8.23	+1.46	7.50	0.1	8.96	0.2	0.00053438	0.00507128	A_07_P032800	c_3420	cmr|Prepilin peptidase dependent protein A precursor [c_3420]
pphB	-3.41	8.00	-1.77	8.88	0.3	7.12	0.1	0.00066113	0.00587078	A_07_P002119	b2734	cmr|protein phosphatase 2 [b2734]
ppiB	+2.69	12.90	+1.43	12.18	0.1	13.61	0.0	0.00037011	0.00392997	A_07_P016413	b0525	cmr|peptidyl-prolyl cis-trans isomerase B [b0525]
ppiC	+2.18	9.35	+1.12	8.79	0.0	9.91	0.1	0.00078205	0.00665883	A_07_P041923	c_4697	cmr|Peptidyl-prolyl cis-trans isomerase C [c_4697]
ppiC	+2.15	8.25	+1.11	7.70	0.1	8.81	0.0	0.00090232	0.00739560	A_07_P005759	b3775	cmr|peptidyl-prolyl cis-trans isomerase C [b3775]
pps	+3.21	13.28	+1.68	12.44	0.0	14.12	0.1	0.00011949	0.00176668	A_07_P018562	b1702	cmr|phosphoenolpyruvate synthase [b1702]
ppx	-2.04	8.75	-1.03	9.27	0.0	8.24	0.1	0.00132907	0.00973744	A_07_P013570	b2502	cmr|exopolyphosphatase [b2502]
prfA	-2.41	10.05	-1.27	10.69	0.1	9.42	0.1	0.00082288	0.00692435	A_07_P007006	b1211	cmr|peptide chain release factor RF-1 [b1211]
prfB	-2.81	13.88	-1.49	14.62	0.0	13.13	0.2	0.00065522	0.00584467	A_07_P006421	b2891	cmr|peptide chain release factor RF-2 [b2891]
prfB	-2.64	14.38	-1.40	15.07	0.0	13.68	0.1	0.00029452	0.00334071	A_07_P032852	c_5622	cmr|Peptide chain release factor 2 [c_5622]
prfH	-8.22	10.49	-3.04	12.01	0.0	8.97	0.0	0.00000514	0.00034758	A_07_P030853	c_0382	cmr|Peptide chain release factor homolog [c_0382]
prfH	-6.36	10.94	-2.67	12.28	0.2	9.61	0.0	0.00002717	0.00073404	A_07_P007894	b0236	cmr|probable peptide chain release factor [b0236]
prfH	-6.82	9.29	-2.77	10.67	0.1	7.90	0.0	0.00000982	0.00045743	A_07_P062922	ECs0263	cmr|probable peptide chain release factor [ECs0263]
prfH	-6.43	9.33	-2.69	10.67	0.0	7.99	0.1	0.00001686	0.00058050	A_07_P051892	Z0297	cmr|probable peptide chain release factor [Z0297]
priA	-2.23	8.55	-1.15	9.12	0.1	7.97	0.0	0.00119666	0.00905341	A_07_P019451	b3935	cmr|primosomal protein N [b3935]
priA	-2.94	9.99	-1.56	10.76	0.2	9.21	0.3	0.00297784	0.01777220	A_07_P039449	c_4888	cmr|Primosomal protein N [c_4888]
prmC	-2.03	9.94	-1.02	10.45	0.1	9.43	0.1	0.00176962	0.01211343	A_07_P007012	b1212	cmr|possible protoporphyrinogen oxidase [b1212]
proP	-5.86	10.17	-2.55	11.45	0.1	8.90	0.1	0.00001484	0.00054604	A_07_P011292	b4111	cmr|low-affinity transport system; proline permease II [b4111]
proV	-3.07	8.69	-1.62	9.50	0.0	7.88	0.1	0.00015594	0.00214424	A_07_P030555	c_3230	cmr|Glycine betaine/L-proline transport ATP-binding protein proV [c_3230]
proW	-2.78	8.52	-1.47	9.26	0.0	7.78	0.1	0.00022252	0.00274273	A_07_P001861	b2678	cmr|high-affinity transport system for glycine betaine and proline [b2678]
proX	+2.62	8.19	+1.39	7.49	0.5	8.88	0.0	0.00977943	0.04395301	A_07_P001866	b2679	cmr|high-affinity transport system for glycine betaine and proline [b2679]
proX	+4.13	8.83	+2.04	7.81	0.2	9.86	0.0	0.00016096	0.00218777	A_07_P030557	c_3232	cmr|Glycine betaine-binding periplasmic protein precursor [c_3232]
prpR	-2.86	11.11	-1.52	11.87	0.3	10.35	0.0	0.00131514	0.00971180	A_07_P031011	c_0450	cmr|Propionate catabolism operon Regulatory protein [c_0450]
psiF	-2.30	8.77	-1.20	9.37	0.2	8.17	0.1	0.00213757	0.01386644	A_07_P062746	ECs0434	cmr|phosphate starvation-induced protein psiF [ECs0434]
psiF	-2.25	8.86	-1.17	9.45	0.1	8.27	0.0	0.00069415	0.00608327	A_07_P051681	Z0480	cmr|induced by phosphate starvation [Z0480]
pspB	-8.04	10.20	-3.01	11.71	0.1	8.70	0.0	0.00000775	0.00041039	A_07_P014506	b1305	cmr|phage shock protein [b1305]
pspC	-18.09	10.48	-4.18	12.57	0.0	8.40	0.1	0.00000201	0.00025681	A_07_P014514	b1306	cmr|phage shock protein: activates phage shock-protein expression [b1306]
pspE	+2.85	11.00	+1.51	10.25	0.1	11.76	0.1	0.00038818	0.00402870	A_07_P014522	b1308	cmr|phage shock protein [b1308]
pspE	+2.47	11.14	+1.31	10.49	0.1	11.80	0.0	0.00036817	0.00391653	A_07_P014522	b1308	cmr|phage shock protein [b1308]
pstC	-3.82	10.31	-1.93	11.27	0.1	9.34	0.1	0.00008397	0.00140944	A_07_P005556	b3727	cmr|high-affinity phosphate-specific transport system, cytoplasmic membrane component [b3727]
pta	+8.56	10.50	+3.10	8.96	0.1	12.05	0.1	0.00000797	0.00041039	A_07_P042552	c_2840	cmr|Phosphate acetyltransferase [c_2840]
pta	+8.49	9.55	+3.09	8.01	0.3	11.09	0.1	0.00004414	0.00094686	A_07_P013771	b2297	cmr|phosphotransacetylase [b2297]
pth	+4.73	11.11	+2.24	9.99	0.1	12.23	0.1	0.00002979	0.00076634	A_07_P006971	b1204	cmr|peptidyl-tRNA hydrolase [b1204]
ptrB	-4.36	8.33	-2.12	9.39	0.1	7.27	0.2	0.00011606	0.00173362	A_07_P015904	b1845	cmr|protease II [b1845]
ptrB	-10.69	9.81	-3.42	11.52	0.0	8.10	0.2	0.00000800	0.00041039	A_07_P035322	c_2256	cmr|Protease II [c_2256]
ptrB	-14.04	10.24	-3.81	12.14	0.2	8.33	0.2	0.00002170	0.00065941	A_07_P052572	Z2896	cmr|protease II [Z2896]
ptsH	+2.11	11.50	+1.08	10.96	0.0	12.04	0.1	0.00102195	0.00805287	A_07_P013132	b2415	cmr|PTS system protein HPr [b2415]
ptsI	+6.54	12.92	+2.71	11.57	0.1	14.28	0.2	0.00003653	0.00085835	A_07_P013136	b2416	cmr|PEP-protein phosphotransferase system enzyme I [b2416]
ptsI	+6.71	13.47	+2.75	12.10	0.1	14.84	0.1	0.00001595	0.00056395	A_07_P013136	b2416	cmr|PEP-protein phosphotransferase system enzyme I [b2416]
ptsI	+8.53	12.74	+3.09	11.19	0.1	14.28	0.0	0.00000635	0.00037147	A_07_P013136	b2416	cmr|PEP-protein phosphotransferase system enzyme I [b2416]
ptsI	+6.53	13.52	+2.71	12.17	0.1	14.87	0.0	0.00000985	0.00045743	A_07_P013136	b2416	cmr|PEP-protein phosphotransferase system enzyme I [b2416]
ptsI	+6.38	13.49	+2.67	12.16	0.1	14.83	0.0	0.00001769	0.00059256	A_07_P013136	b2416	cmr|PEP-protein phosphotransferase system enzyme I [b2416]
purB	+3.18	11.41	+1.67	10.57	0.0	12.24	0.0	0.00010311	0.00160656	A_07_P006606	b1131	cmr|adenylosuccinate lyase [b1131]
purC	+15.02	10.12	+3.91	8.16	0.1	12.07	0.0	0.00000200	0.00025681	A_07_P013440	b2476	cmr|phosphoribosylaminoimidazole-succinocarboxamide synthetase = SAICAR synthetase [b2476]
purD	+19.08	10.09	+4.25	7.96	0.5	12.21	0.0	0.00004214	0.00093353	A_07_P008690	b4005	cmr|phosphoribosylglycinamide synthetase = GAR synthetase [b4005]
purE	+5.92	9.77	+2.57	8.49	0.3	11.05	0.1	0.00010303	0.00160656	A_07_P016404	b0523	cmr|phosphoribosylaminoimidazole carboxylase = AIR carboxylase, catalytic subunit [b0523]
purF	+4.66	8.94	+2.22	7.82	0.1	10.05	0.2	0.00008705	0.00143969	A_07_P013849	b2312	cmr|amidophosphoribosyltransferase = PRPP amidotransferase [b2312]
purF	+7.33	10.16	+2.87	8.72	0.1	11.60	0.0	0.00000737	0.00040556	A_07_P032426	c_2856	cmr|Amidophosphoribosyltransferase [c_2856]
purF	+4.88	9.11	+2.29	7.97	0.2	10.26	0.1	0.00010668	0.00164437	A_07_P042612	Z3574	cmr|amidophosphoribosyltransferase = PRPP amidotransferase [Z3574]
purH	+11.78	8.71	+3.56	6.93	0.1	10.49	0.1	0.00000543	0.00034972	A_07_P057962	ECs4929	cmr|phosphoribosylaminoimidazolecarboxamideformyltra nsferase [ECs4929]
purH	+34.26	10.24	+5.10	7.69	0.0	12.79	0.1	0.00000045	0.00019431	A_07_P037451	c_4964	cmr|Purine biosynthesis protein PurH [c_4964]
purH	+12.19	8.82	+3.61	7.02	0.1	10.62	0.0	0.00000260	0.00027706	A_07_P047211	Z5583	cmr|phosphoribosylaminoimidazolecarboxamide formyltransferase = AICAR formyltransferase IMP cyclohydrolase [Z5583]
purH	+11.96	8.90	+3.58	7.11	0.1	10.69	0.0	0.00000264	0.00027706	A_07_P008693	b4006	cmr|phosphoribosylaminoimidazolecarboxamideformyltransferase = AICAR formyltransferase; IMP cyclohydrolase [b4006]
purK	+4.37	9.38	+2.13	8.32	0.1	10.44	0.1	0.00009825	0.00154998	A_07_P016397	b0522	cmr|phosphoribosylaminoimidazole carboxylase = AIR carboxylase, CO [b0522]
purL	+9.40	9.23	+3.23	7.61	0.3	10.84	0.0	0.00003554	0.00084290	A_07_P019686	b2557	cmr|phosphoribosylformyl-glycineamide synthetase = FGAM synthetase [b2557]
purM	+23.48	11.34	+4.55	9.06	0.0	13.62	0.1	0.00000075	0.00019431	A_07_P013551	b2499	cmr|phosphoribosylaminoimidazole synthetase = AIR synthetase [b2499]
purN	+13.35	10.60	+3.74	8.73	0.1	12.47	0.0	0.00000336	0.00029719	A_07_P013559	b2500	cmr|phosphoribosylglycinamide formyltransferase 1 [b2500]
purN	+11.56	11.45	+3.53	9.68	0.1	13.21	0.1	0.00000348	0.00030339	A_07_P030133	c_3018	cmr|Phosphoribosylglycinamide formyltransferase [c_3018]
purT	+15.70	9.48	+3.97	7.49	0.0	11.47	0.0	0.00000142	0.00022424	A_07_P015921	b1849	cmr|phosphoribosylglycinamide formyltransferase 2 [b1849]
putA	+5.17	10.36	+2.37	9.17	0.2	11.54	0.0	0.00005250	0.00105155	A_07_P041092	c_1151	cmr|PutA protein [c_1151]
putA	+21.48	9.78	+4.42	7.57	0.2	11.99	0.1	0.00000263	0.00027706	A_07_P001304	b1014	cmr|proline dehydrogenase, P5C dehydrogenase [b1014]
putP	+3.99	9.58	+2.00	8.58	0.5	10.58	0.0	0.00200164	0.01316364	A_07_P001306	b1015	cmr|major sodium/proline symporter [b1015]
pykA	+6.60	10.50	+2.72	9.14	0.0	11.86	0.0	0.00000872	0.00042757	A_07_P015946	b1854	cmr|pyruvate kinase II, glucose stimulated [b1854]
pyrB	+9.92	9.71	+3.31	8.06	0.3	11.37	0.0	0.00002852	0.00074846	A_07_P003778	b4245	cmr|aspartate carbamoyltransferase, catalytic subunit [b4245]
pyrC	+4.23	8.69	+2.08	7.65	0.0	9.73	0.0	0.00003346	0.00081556	A_07_P001513	b1062	cmr|dihydro-orotase [b1062]
pyrD	+2.96	9.22	+1.57	8.43	0.1	10.00	0.1	0.00019812	0.00252367	A_07_P005175	b0945	cmr|dihydro-orotate dehydrogenase [b0945]
pyrF	+3.24	9.99	+1.70	9.14	0.1	10.84	0.0	0.00013968	0.00197581	A_07_P014388	b1281	cmr|orotidine-5-phosphate decarboxylase [b1281]
pyrG	+4.05	11.37	+2.02	10.36	0.1	12.37	0.0	0.00004229	0.00093353	A_07_P002348	b2780	cmr|CTP synthetase [b2780]
pyrH	-2.45	12.49	-1.29	13.13	0.0	11.84	0.0	0.00033810	0.00367995	A_07_P002814	b0171	cmr|uridylate kinase [b0171]
pyrI	+6.06	8.98	+2.60	7.68	0.1	10.28	0.0	0.00001448	0.00054604	A_07_P003774	b4244	cmr|aspartate carbamoyltransferase, regulatory subunit [b4244]
qor	+4.75	8.91	+2.25	7.79	0.1	10.04	0.1	0.00003988	0.00090512	A_07_P008897	b4051	cmr|quinone oxidoreductase [b4051]
quuQ	-2.00	8.94	-1.00	9.43	0.2	8.44	0.0	0.00569906	0.02918868	A_07_P020436	b1559	cmr|orf, hypothetical protein [b1559]
raiA	+29.10	13.85	+4.86	11.42	0.1	16.28	0.0	0.00000090	0.00019431	A_07_P019869	b2597	cmr|putative yhbH sigma 54 modulator [b2597]
rarD	-2.14	9.22	-1.10	9.77	0.1	8.67	0.0	0.00104128	0.00816430	A_07_P005965	b3819	cmr|orf, hypothetical protein [b3819]
rbfA	-2.83	12.99	-1.50	13.74	0.0	12.24	0.0	0.00016249	0.00220162	A_07_P000559	b3167	cmr|ribosome-binding factor A [b3167]
rbn	+3.55	9.22	+1.83	8.31	0.1	10.14	0.1	0.00017474	0.00230993	A_07_P039358	c_4833	cmr|Ribonuclease BN [c_4833]
rbsB	+3.97	14.29	+1.99	13.29	0.0	15.28	0.0	0.00004380	0.00094590	A_07_P005677	b3751	cmr|D-ribose periplasmic binding protein [b3751]
rbsC	+7.95	10.25	+2.99	8.75	0.0	11.74	0.0	0.00000544	0.00034972	A_07_P005671	b3750	cmr|D-ribose high-affinity transport system [b3750]
rcsB	+2.12	10.24	+1.08	9.70	0.0	10.78	0.1	0.00127825	0.00950623	A_07_P032365	c_2760	cmr|Capsular synthesis regulator component B [c_2760]
rcsD	+6.84	9.95	+2.77	8.57	0.2	11.34	0.0	0.00002130	0.00064982	A_07_P000870	b2216	cmr|putative 2-component sensor protein [b2216]
rcsF	+2.34	11.22	+1.23	10.61	0.1	11.84	0.0	0.00053891	0.00509893	A_07_P041460	c_0237	cmr|Protein rcsF [c_0237]
rdgC	+4.14	10.32	+2.05	9.29	0.3	11.34	0.0	0.00024952	0.00298515	A_07_P021080	b0393	cmr|orf, hypothetical protein [b0393]
rdoA	+3.26	10.58	+1.71	9.73	0.1	11.43	0.0	0.00009766	0.00154382	A_07_P019079	b3859	cmr|orf, hypothetical protein [b3859]
recA	-3.27	14.69	-1.71	15.55	0.0	13.84	0.1	0.00010685	0.00164437	A_07_P001941	b2699	cmr|DNA strand exchange and renaturation, DNA-dependent ATPase, DNA- and ATP-dependent coprotease [b2699]
recF	-3.86	11.12	-1.95	12.10	0.0	10.15	0.2	0.00012265	0.00179744	A_07_P005432	b3700	cmr|ssDNA and dsDNA binding, ATP binding [b3700]
recJ	-2.54	9.23	-1.35	9.90	0.1	8.55	0.2	0.00135159	0.00986532	A_07_P045645	Z4230	cmr|ssDNA exonuclease, 5 --> 3 specific [Z4230]
recJ	-2.84	8.29	-1.51	9.04	0.1	7.54	0.1	0.00033880	0.00368379	A_07_P006426	b2892	cmr|ssDNA exonuclease, 5 --> 3 specific [b2892]
recJ	-4.31	11.10	-2.11	12.16	0.0	10.05	0.0	0.00003237	0.00079881	A_07_P032859	c_3472	cmr|Single-stranded-DNA-specific exonuclease recJ [c_3472]
recN	-3.03	13.88	-1.60	14.68	0.0	13.08	0.0	0.00011524	0.00172297	A_07_P019956	b2616	cmr|protein used in recombination and DNA repair [b2616]
recR	+8.63	11.87	+3.11	10.32	0.1	13.43	0.0	0.00000682	0.00038477	A_07_P021466	b0472	cmr|recombination and repair [b0472]
recX	-4.32	11.38	-2.11	12.44	0.0	10.33	0.1	0.00003863	0.00088510	A_07_P063405	ECs3555	cmr|regulatory protein OraA [ECs3555]
recX	-4.57	11.60	-2.19	12.69	0.0	10.50	0.2	0.00007017	0.00124769	A_07_P001938	b2698	cmr|regulator, OraA protein [b2698]
recX	-3.00	10.77	-1.58	11.56	0.1	9.98	0.2	0.00058126	0.00537678	A_07_P052735	Z4001	cmr|regulator, OraA protein [Z4001]
rep	+3.09	8.96	+1.63	8.15	0.1	9.78	0.1	0.00023461	0.00284787	A_07_P005771	b3778	cmr|rep helicase, a single-stranded DNA dependent ATPase [b3778]
rfaH	-2.63	9.52	-1.40	10.22	0.0	8.82	0.1	0.00027793	0.00319869	A_07_P019019	b3842	cmr|transcriptional activator affecting biosynthesis of lipopolysaccharide core, F pilin, and haemolysin [b3842]
rfaI	-9.02	12.44	-3.17	14.03	0.0	10.85	0.1	0.00000451	0.00033765	A_07_P039805	c_4453	cmr|Lipopolysaccharide 1,3-galactosyltransferase [c_4453]
rfaP	-3.08	12.01	-1.62	12.83	0.0	11.20	0.1	0.00017590	0.00231945	A_07_P039808	c_4454	cmr|Lipopolysaccharide core biosynthesis protein rfaP [c_4454]
rfaP	-3.20	12.36	-1.68	13.20	0.2	11.52	0.0	0.00030711	0.00342042	A_07_P039808	c_4454	cmr|Lipopolysaccharide core biosynthesis protein rfaP [c_4454]
rfaP	-2.70	12.08	-1.43	12.80	0.0	11.36	0.0	0.00021054	0.00263076	A_07_P039808	c_4454	cmr|Lipopolysaccharide core biosynthesis protein rfaP [c_4454]
rfaP	-3.09	12.41	-1.63	13.22	0.0	11.59	0.1	0.00011951	0.00176668	A_07_P039808	c_4454	cmr|Lipopolysaccharide core biosynthesis protein rfaP [c_4454]
rfaP	-2.92	12.25	-1.55	13.02	0.0	11.47	0.1	0.00015771	0.00216480	A_07_P039808	c_4454	cmr|Lipopolysaccharide core biosynthesis protein rfaP [c_4454]
rffA	+2.36	8.31	+1.24	7.69	0.1	8.93	0.0	0.00046235	0.00457870	A_07_P005832	b3791	cmr|putative regulator [b3791]
rffC	+2.68	8.03	+1.42	7.32	0.4	8.74	0.1	0.00522529	0.02723171	A_07_P005826	b3790	cmr|orf, hypothetical protein [b3790]
rffM	+2.14	7.64	+1.10	7.09	0.1	8.19	0.1	0.00099510	0.00788955	A_07_P005860	b3794	cmr|probable UDP-N-acetyl-D-mannosaminuronic acid transferase; synthesis of enterobacterial common antigen [b3794]
rhaS	+2.79	7.55	+1.48	6.81	0.3	8.29	0.0	0.00174778	0.01199888	A_07_P019305	b3905	cmr|positive regulator for rhaBAD operon [b3905]
rhaS	+2.16	7.51	+1.11	6.96	0.2	8.07	0.0	0.00332798	0.01943461	A_07_P047099	Z5449	cmr|positive regulator for rhaBAD operon [Z5449]
rhlB	+2.76	9.04	+1.47	8.31	0.0	9.78	0.1	0.00032396	0.00357174	A_07_P005782	b3780	cmr|putative ATP-dependent RNA helicase [b3780]
rhlE	+2.05	10.08	+1.04	9.56	0.1	10.60	0.0	0.00149320	0.01066650	A_07_P004438	b0797	cmr|putative ATP-dependent RNA helicase [b0797]
rho	-3.24	10.24	-1.69	11.09	0.1	9.39	0.0	0.00014363	0.00201369	A_07_P005791	b3783	cmr|transcription termination factor Rho; polarity suppressor [b3783]
ribD	-2.58	10.98	-1.37	11.67	0.0	10.30	0.0	0.00024886	0.00297954	A_07_P021185	b0414	cmr|bifunctional pyrimidine deaminase/reductase in pathway of riboflavin synthesis [b0414]
ribE	-4.51	12.81	-2.17	13.90	0.0	11.72	0.1	0.00002926	0.00076000	A_07_P021187	b0415	cmr|riboflavin synthase, beta chain [b0415]
ribE	-4.31	12.99	-2.11	14.04	0.0	11.93	0.0	0.00003384	0.00082101	A_07_P021187	b0415	cmr|riboflavin synthase, beta chain [b0415]
ribE	-4.67	12.74	-2.22	13.85	0.0	11.63	0.1	0.00002566	0.00072274	A_07_P021187	b0415	cmr|riboflavin synthase, beta chain [b0415]
ribE	-4.58	12.67	-2.20	13.77	0.0	11.58	0.0	0.00002459	0.00070547	A_07_P021187	b0415	cmr|riboflavin synthase, beta chain [b0415]
ribE	-4.73	12.73	-2.24	13.85	0.0	11.61	0.0	0.00002300	0.00067944	A_07_P021187	b0415	cmr|riboflavin synthase, beta chain [b0415]
rimI	-2.10	11.72	-1.07	12.26	0.0	11.19	0.0	0.00080706	0.00682752	A_07_P017550	b4373	cmr|acyltransferase for 30S ribosomal subunit protein S18; acetylation of N-terminal alanine [b4373]
rimJ	+3.62	10.49	+1.86	9.56	0.1	11.42	0.1	0.00014603	0.00203963	A_07_P001534	b1066	cmr|acetylation of N-terminal alanine of 30S ribosomal subunit protein S5 [b1066]
rimN	+2.46	8.67	+1.30	8.02	0.3	9.33	0.0	0.00315062	0.01862685	A_07_P012843	b3282	cmr|orf, hypothetical protein [b3282]
rlmL	+2.75	8.51	+1.46	7.78	0.2	9.24	0.1	0.00047186	0.00464602	A_07_P005186	b0948	cmr|putative oxidoreductase [b0948]
rlpA	+2.65	9.84	+1.41	9.14	0.1	10.55	0.0	0.00026191	0.00308181	A_07_P009735	b0633	cmr|a minor lipoprotein [b0633]
rlpB	-2.53	10.36	-1.34	11.03	0.1	9.69	0.0	0.00035058	0.00377532	A_07_P044023	Z0788	cmr|a minor lipoprotein [Z0788]
rlpB	-3.33	12.43	-1.73	13.30	0.1	11.56	0.0	0.00009199	0.00148849	A_07_P009773	b0641	cmr|a minor lipoprotein [b0641]
rluA	-2.51	9.73	-1.33	10.39	0.2	9.07	0.0	0.00119591	0.00905214	A_07_P010976	b0058	cmr|orf, hypothetical protein [b0058]
rluD	-2.65	10.47	-1.41	11.18	0.3	9.77	0.0	0.00127534	0.00948910	A_07_P042940	Z3888	cmr|suppressor of ftsH mutation [Z3888]
rluE	-2.37	11.26	-1.24	11.88	0.0	10.63	0.1	0.00051765	0.00495438	A_07_P006629	b1135	cmr|orf, hypothetical protein [b1135]
rmf	+25.77	11.15	+4.69	8.80	0.1	13.49	0.0	0.00000068	0.00019431	A_07_P005212	b0953	cmr|ribosome modulation factor [b0953]
rnc	-3.24	12.06	-1.69	12.91	0.0	11.21	0.1	0.00011518	0.00172297	A_07_P019740	b2567	cmr|RNase III, ds RNA [b2567]
rne	+2.77	8.18	+1.47	7.45	0.1	8.92	0.1	0.00027536	0.00317893	A_07_P001621	b1084	cmr|RNase E, membrane attachment, mRNA turnover, maturation 5S RNA [b1084]
rnhB	-4.01	11.18	-2.01	12.18	0.1	10.18	0.0	0.00005329	0.00106045	A_07_P002875	b0183	cmr|RNAse HII, degrades RNA of DNA-RNA hybrids [b0183]
rnk	+5.61	10.20	+2.49	8.95	0.2	11.44	0.1	0.00007826	0.00133632	A_07_P016807	b0610	cmr|regulator of nucleoside diphosphate kinase [b0610]
rnpA	-2.15	14.35	-1.10	14.90	0.0	13.80	0.0	0.00073125	0.00632113	A_07_P005448	b3704	cmr|RNase P, protein component; protein C5; processes tRNA, 4.5S RNA [b3704]
rob	+2.94	11.90	+1.56	11.12	0.1	12.68	0.1	0.00026432	0.00309464	A_07_P017663	b4396	cmr|right origin-binding protein [b4396]
rpe	+4.50	10.25	+2.17	9.16	0.1	11.33	0.0	0.00005485	0.00107946	A_07_P017959	b3386	cmr|D-ribulose-5-phosphate 3-epimerase [b3386]
rpiR	+10.15	9.35	+3.34	7.68	0.1	11.03	0.0	0.00000400	0.00032449	A_07_P037745	c_5095	cmr|Protein rpiR [c_5095]
rplB	+2.26	16.85	+1.18	16.26	0.0	17.44	0.1	0.00061415	0.00558100	A_07_P013013	b3317	cmr|50S ribosomal subunit protein L2 [b3317]
rplI	-2.48	15.05	-1.31	15.71	0.0	14.40	0.0	0.00031229	0.00346495	A_07_P011735	b4203	cmr|50S ribosomal subunit protein L9 [b4203]
rplN	+2.65	16.37	+1.41	15.66	0.0	17.07	0.0	0.00022312	0.00274437	A_07_P012976	b3310	cmr|50S ribosomal subunit protein L14 [b3310]
rplR	-2.40	16.43	-1.27	17.06	0.1	15.80	0.1	0.00046399	0.00459145	A_07_P012950	b3304	cmr|50S ribosomal subunit protein L18 [b3304]
rplS	-5.70	12.95	-2.51	14.21	0.0	11.69	0.0	0.00001265	0.00051704	A_07_P019906	b2606	cmr|50S ribosomal subunit protein L19 [b2606]
rplT	-5.48	13.66	-2.46	14.89	0.1	12.43	0.0	0.00001642	0.00057181	A_07_P018627	b1716	cmr|50S ribosomal subunit protein L20, and regulator [b1716]
rplY	+2.55	14.11	+1.35	13.44	0.1	14.79	0.0	0.00034820	0.00376000	A_07_P000722	b2185	cmr|50S ribosomal subunit protein L25 [b2185]
rplY	+2.90	13.99	+1.54	13.22	0.1	14.76	0.1	0.00022750	0.00278734	A_07_P035511	c_2722	cmr|50S ribosomal protein L25 [c_2722]
rpmE	-5.28	15.20	-2.40	16.40	0.0	13.99	0.0	0.00001573	0.00056283	A_07_P019460	b3936	cmr|50S ribosomal subunit protein L31 [b3936]
rpmG	-3.07	14.17	-1.62	14.98	0.0	13.36	0.1	0.00016493	0.00222503	A_07_P017122	b3636	cmr|50S ribosomal subunit protein L33 [b3636]
rpoB	+4.06	10.81	+2.02	9.80	0.0	11.82	0.1	0.00004411	0.00094686	A_07_P008600	b3987	cmr|RNA polymerase, beta subunit [b3987]
rpoC	+2.25	10.57	+1.17	9.99	0.1	11.16	0.1	0.00072018	0.00625405	A_07_P008605	b3988	cmr|RNA polymerase, beta prime subunit [b3988]
rpoD	-4.46	14.52	-2.16	15.60	0.0	13.44	0.1	0.00003133	0.00078813	A_07_P000070	b3067	cmr|RNA polymerase, sigma [b3067]
rpoE	-5.35	12.07	-2.42	13.28	0.0	10.86	0.0	0.00001583	0.00056378	A_07_P019770	b2573	cmr|RNA polymerase, sigma-E factor; heat shock and oxidative stress [b2573]
rpoH	-2.59	11.96	-1.37	12.64	0.0	11.27	0.0	0.00024105	0.00291040	A_07_P007245	b3461	cmr|RNA polymerase, sigma [b3461]
rpoN	+2.06	9.76	+1.04	9.24	0.0	10.28	0.3	0.00991105	0.04444308	A_07_P012490	b3202	cmr|RNA polymerase, sigma [b3202]

rpoZ	-4.23	13.64	-2.08	14.68	0.0	12.60	0.0	0.00003409	0.00082567	A_07_P017187	b3649	cmr|RNA polymerase, omega subunit [b3649]
rppH	-3.93	12.37	-1.97	13.35	0.1	11.38	0.0	0.00004657	0.00097501	A_07_P006135	b2830	cmr|putative invasion protein [b2830]
rpsA	-2.18	14.67	-1.12	15.23	0.0	14.11	0.0	0.00062260	0.00563493	A_07_P005002	b0911	cmr|30S ribosomal subunit protein S1 [b0911]
rpsB	-2.82	14.58	-1.49	15.33	0.0	13.83	0.0	0.00017339	0.00229590	A_07_P002803	b0169	cmr|30S ribosomal subunit protein S2 [b0169]
rpsC	-2.32	16.72	-1.21	17.32	0.0	16.11	0.1	0.00052507	0.00501311	A_07_P012998	b3314	cmr|30S ribosomal subunit protein S3 [b3314]
rpsG	-2.08	16.35	-1.06	16.88	0.0	15.82	0.0	0.00085214	0.00710601	A_07_P017731	b3341	cmr|30S ribosomal subunit protein S7, initiates assembly [b3341]
rpsI	-3.32	13.60	-1.73	14.47	0.1	12.74	0.4	0.00163480	0.01138865	A_07_P012625	b3230	cmr|30S ribosomal subunit protein S9 [b3230]
rpsO	+3.06	13.35	+1.61	12.55	0.1	14.16	0.0	0.00012937	0.00185677	A_07_P000550	b3165	cmr|30S ribosomal subunit protein S15 [b3165]
rpsQ	-4.89	15.70	-2.29	16.84	0.0	14.55	0.1	0.00002237	0.00067002	A_07_P012981	b3311	cmr|30S ribosomal subunit protein S17 [b3311]
rpsS	+2.27	15.72	+1.19	15.13	0.0	16.31	0.1	0.00061818	0.00560315	A_07_P013010	b3316	cmr|30S ribosomal subunit protein S19 [b3316]
rpsT	-3.85	13.66	-1.95	14.64	0.0	12.69	0.1	0.00004930	0.00100666	A_07_P010803	b0023	cmr|30S ribosomal subunit protein S20 [b0023]
rraA	+2.71	10.30	+1.44	9.59	0.1	11.02	0.0	0.00023687	0.00286428	A_07_P019421	b3929	cmr|menaquinone biosynthesis, unknown [b3929]
rrmJ	-3.16	12.17	-1.66	13.00	0.0	11.34	0.0	0.00009672	0.00153974	A_07_P000609	b3179	cmr|cell division protein [b3179]
rsd	+5.82	10.46	+2.54	9.19	0.1	11.73	0.1	0.00002073	0.00063877	A_07_P008637	b3995	cmr|putative transcriptional regulator [b3995]
rseA	-6.01	13.26	-2.59	14.55	0.0	11.96	0.0	0.00001067	0.00047201	A_07_P019761	b2572	cmr|sigma-E factor, negative regulatory protein [b2572]
rseB	-2.39	9.97	-1.26	10.60	0.2	9.34	0.1	0.00101769	0.00802729	A_07_P019758	b2571	cmr|regulates activity of sigma-E factor [b2571]
rseP	-2.16	12.18	-1.11	12.74	0.0	11.63	0.0	0.00075283	0.00647325	A_07_P002836	b0176	cmr|orf, hypothetical protein [b0176]
rsmE	+4.43	11.22	+2.15	10.14	0.1	12.29	0.1	0.00005163	0.00103969	A_07_P011872	b2946	cmr|orf, hypothetical protein [b2946]
rstB	-2.00	8.89	-1.00	9.39	0.1	8.39	0.1	0.00295702	0.01767474	A_07_P035086	c_2001	cmr|Sensor protein rstB [c_2001]
rstB	-2.21	8.81	-1.14	9.39	0.1	8.24	0.1	0.00184600	0.01244116	A_07_P020676	b1609	cmr|sensor histidine protein kinase [b1609]
rsuA	-2.88	12.25	-1.53	13.01	0.1	11.49	0.1	0.00018279	0.00238430	A_07_P000715	b2183	cmr|16S pseudouridylate 516 synthase [b2183]
rsxA	-18.34	10.78	-4.20	12.88	0.0	8.68	0.1	0.00000111	0.00020547	A_07_P020768	b1627	cmr|orf, hypothetical protein [b1627]
rsxB	-7.62	10.26	-2.93	11.73	0.0	8.80	0.0	0.00000659	0.00038136	A_07_P020774	b1628	cmr|orf, hypothetical protein [b1628]
rsxC	-4.07	9.75	-2.02	10.76	0.0	8.74	0.1	0.00004731	0.00098397	A_07_P020776	b1629	cmr|putative membrane protein [b1629]
rsxD	-2.54	8.10	-1.35	8.78	0.1	7.43	0.0	0.00041498	0.00423153	A_07_P020785	b1630	cmr|orf, hypothetical protein [b1630]
rtcA	-2.37	9.02	-1.24	9.64	0.4	8.40	0.0	0.00536171	0.02783211	A_07_P018126	b3420	cmr|RNA 3-terminal phosphate cyclase [b3420]
rusA	+62.89	9.87	+5.97	6.89	0.0	12.86	0.0	0.00000016	0.00019431	A_07_P016520	b0550	cmr|endodeoxyribonuclease RUS [b0550]
rutB	-2.85	11.02	-1.51	11.78	0.0	10.26	0.4	0.00427093	0.02331535	A_07_P001287	b1011	cmr|putative synthetase [b1011]
rutR	-2.11	9.40	-1.08	9.95	0.1	8.86	0.0	0.00091186	0.00745396	A_07_P001300	b1013	cmr|putative tet operon regulator [b1013]
ruvB	-2.39	11.69	-1.26	12.32	0.0	11.06	0.0	0.00037711	0.00396055	A_07_P015980	b1860	cmr|Holliday junction helicase subunit A; branch migration; repair [b1860]
rzpD	+24.13	11.25	+4.59	8.95	0.4	13.54	0.0	0.00002646	0.00072866	A_07_P010301	b0556	cmr|bacteriophage lambda endopeptidase homolog [b0556]
rzpD	+11.77	9.74	+3.56	7.96	0.2	11.52	0.0	0.00001206	0.00050646	A_07_P016543	b0556	cmr|bacteriophage lambda endopeptidase homolog [b0556]
rzpQ	-2.28	7.62	-1.19	8.21	0.4	7.02	0.0	0.00896823	0.04126118	A_07_P020510	b1573	cmr|orf, hypothetical protein [b1573]
rzpR	+2.31	7.87	+1.21	7.27	0.1	8.47	0.2	0.00121259	0.00913448	A_07_P014786	b1362	cmr|putative Rac prophage endopeptidase [b1362]
sad	-2.08	9.49	-1.06	10.02	0.2	8.96	0.0	0.00444914	0.02406294	A_07_P020272	b1525	cmr|putative aldehyde dehydrogenase [b1525]
sat	-11.02	10.44	-3.46	12.17	0.1	8.71	0.1	0.00000355	0.00030339	A_07_P036297	c_3619	cmr|Aecreted auto transpoter toxin [c_3619]
sbcB	+2.27	8.86	+1.18	8.27	0.2	9.45	0.0	0.00247431	0.01548501	A_07_P044967	Z3173	cmr|exonuclease I, 3 --> 5 specific deoxyribophosphodiesterase [Z3173]
sbcB	+2.10	9.16	+1.07	8.63	0.1	9.70	0.1	0.00120758	0.00911419	A_07_P055542	ECs2813	cmr|exonuclease I [ECs2813]
sbcB	+2.11	10.28	+1.08	9.74	0.0	10.81	0.2	0.00237912	0.01503690	A_07_P031971	c_2539	cmr|Exodeoxyribonuclease I [c_2539]
sbmA	-2.78	9.29	-1.47	10.03	0.1	8.55	0.1	0.00043202	0.00435473	A_07_P020999	b0377	cmr|sensitivity to microcin B17, possibly envelop protein [b0377]
sbp	-5.78	11.03	-2.53	12.30	0.1	9.77	0.2	0.00006029	0.00114635	A_07_P019364	b3917	cmr|periplasmic sulfate-binding protein [b3917]
sdaA	+2.77	10.58	+1.47	9.85	0.1	11.32	0.1	0.00029731	0.00335322	A_07_P015761	b1814	cmr|L-serine deaminase [b1814]
sdaC	+2.31	11.41	+1.21	10.80	0.1	12.01	0.1	0.00063562	0.00571983	A_07_P002426	b2796	cmr|probable serine transporter [b2796]
sdhA	+9.99	11.67	+3.32	10.01	0.1	13.33	0.1	0.00000559	0.00035512	A_07_P010135	b0723	cmr|succinate dehydrogenase, flavoprotein subunit [b0723]
sdhA	+10.87	10.12	+3.44	8.40	0.2	11.84	0.1	0.00001273	0.00051704	A_07_P051706	Z0877	cmr|succinate dehydrogenase, flavoprotein subunit [Z0877]
sdhA	+10.40	10.26	+3.38	8.57	0.0	11.95	0.0	0.00000323	0.00029256	A_07_P063016	ECs0748	cmr|succinate dehydrogenase flavoprotein subunit [ECs0748]
sdhB	+6.09	11.62	+2.61	10.32	0.1	12.92	0.0	0.00002023	0.00063193	A_07_P010139	b0724	cmr|succinate dehydrogenase, iron sulfur protein [b0724]
sdhC	+37.88	12.43	+5.24	9.81	0.1	15.05	0.0	0.00000050	0.00019431	A_07_P010122	b0721	cmr|succinate dehydrogenase, cytochrome b556 [b0721]
sdhD	+22.16	12.14	+4.47	9.91	0.1	14.38	0.0	0.00000109	0.00020547	A_07_P010127	b0722	cmr|succinate dehydrogenase, hydrophobic subunit [b0722]
sdiA	-6.33	9.83	-2.66	11.16	0.1	8.50	0.1	0.00001698	0.00058050	A_07_P014943	b1916	cmr|transcriptional regulator of ftsQAZ gene cluster [b1916]
sdiA	-4.92	8.16	-2.30	9.31	0.1	7.01	0.0	0.00002304	0.00067944	A_07_P035369	c_2330	cmr|Regulatory protein sdiA [c_2330]
secB	+4.53	11.12	+2.18	10.03	0.1	12.20	0.1	0.00003500	0.00083975	A_07_P016988	b3609	cmr|protein export; molecular chaperone; may bind to signel sequence [b3609]
secE	-3.53	13.72	-1.82	14.63	0.0	12.81	0.0	0.00006163	0.00115828	A_07_P008570	b3981	cmr|preprotein translocase [b3981]
secG	-2.28	11.82	-1.19	12.41	0.1	11.22	0.2	0.00227766	0.01455673	A_07_P000590	b3175	cmr|protein export - membrane protein [b3175]
secY	+2.26	14.97	+1.18	14.38	0.1	15.56	0.1	0.00093650	0.00758059	A_07_P012927	b3300	cmr|putative ATPase subunit of translocase [b3300]
selA	+2.08	10.13	+1.05	9.60	0.0	10.65	0.1	0.00103455	0.00811965	A_07_P016899	b3591	cmr|selenocysteine synthase: L-seryl-tRNA [b3591]
selA	+2.04	10.05	+1.03	9.54	0.1	10.57	0.0	0.00105798	0.00826384	A_07_P016899	b3591	cmr|selenocysteine synthase: L-seryl-tRNA [b3591]
selD	+5.36	10.36	+2.42	9.15	0.0	11.58	0.1	0.00002089	0.00063998	A_07_P018866	b1764	cmr|selenophosphate synthase, H [b1764]
serS	+4.68	8.70	+2.23	7.59	0.2	9.81	0.0	0.00006163	0.00115828	A_07_P004911	b0893	cmr|serine tRNA synthetase; also charges selenocystein tRNA with serine [b0893]
sfaB	+7.84	8.70	+2.97	7.21	0.1	10.18	0.0	0.00000712	0.00039666	A_07_P040481	c_1238	cmr|Putative F1C and S fimbrial switch Regulatory [c_1238]
sfaC	+3.80	7.91	+1.93	6.94	0.1	8.87	0.0	0.00005512	0.00108065	A_07_P040480	c_1237	cmr|Putative F1C and S fimbrial switch Regulatory [c_1237]
sfaD	+4.95	8.96	+2.31	7.81	0.0	10.12	0.1	0.00003462	0.00083595	A_07_P040493	c_1240	cmr|Putative minor F1C fimbrial subunit precursor [c_1240]
sfsA	+3.48	10.92	+1.80	10.02	0.0	11.82	0.1	0.00007566	0.00131047	A_07_P035869	c_0179	cmr|Sugar fermentation stimulation protein A [c_0179]
sfsA	+2.10	10.16	+1.07	9.63	0.1	10.70	0.0	0.00088035	0.00727980	A_07_P002692	b0146	cmr|probable regulator for maltose metabolism [b0146]
sgbH	+2.57	7.53	+1.36	6.85	0.1	8.21	0.2	0.00107145	0.00834272	A_07_P016850	b3581	cmr|probable 3-hexulose 6-phosphate synthase [b3581]
sgbU	+2.22	7.63	+1.15	7.06	0.2	8.21	0.1	0.00174831	0.01199888	A_07_P039678	c_4405	cmr|Putative hexulose-6-phosphate isomerase [c_4405]
sgcX	+2.81	7.49	+1.49	6.74	0.0	8.23	0.1	0.00019649	0.00250894	A_07_P004054	b4305	cmr|putative lyase/synthase [b4305]
sitA	-2.07	9.42	-1.05	9.95	0.1	8.90	0.1	0.00106763	0.00832527	A_07_P038676	c_1600	cmr|SitA protein [c_1600]
sitB	-2.56	8.32	-1.36	9.00	0.1	7.64	0.1	0.00047595	0.00466588	A_07_P038673	c_1599	cmr|SitB protein [c_1599]
sitC	-4.47	8.33	-2.16	9.41	0.2	7.25	0.3	0.00063612	0.00571983	A_07_P038667	c_1598	cmr|SitC protein [c_1598]
skp	+8.19	12.72	+3.03	11.20	0.0	14.24	0.0	0.00000484	0.00034614	A_07_P002847	b0178	cmr|histone-like protein, located in outer membrane or nucleoid [b0178]
slpA	+2.51	10.37	+1.33	9.70	0.5	11.03	0.0	0.00886234	0.04088152	A_07_P051860	Z0033	cmr|probable FKBX-type 16KD peptidyl-prolyl cis-trans isomerase [Z0033]
slt	+2.30	8.11	+1.20	7.51	0.3	8.71	0.0	0.00495496	0.02616938	A_07_P017645	b4392	cmr|soluble lytic murein transglycosylase [b4392]
slt	+2.34	7.90	+1.23	7.29	0.0	8.52	0.1	0.00052780	0.00502395	A_07_P017645	b4392	cmr|soluble lytic murein transglycosylase [b4392]
slt	+2.25	8.06	+1.17	7.48	0.1	8.65	0.1	0.00094559	0.00763066	A_07_P017645	b4392	cmr|soluble lytic murein transglycosylase [b4392]
slt	+2.14	8.06	+1.09	7.51	0.1	8.61	0.0	0.00083837	0.00702801	A_07_P051845	Z5994	cmr|soluble lytic murein transglycosylase [Z5994]
smg	+5.69	9.56	+2.51	8.30	0.1	10.81	0.1	0.00002521	0.00071646	A_07_P034260	c_4045	cmr|Protein smg [c_4045]
smg	+3.19	9.00	+1.67	8.17	0.2	9.84	0.1	0.00029722	0.00335322	A_07_P012853	b3284	cmr|orf, hypothetical protein [b3284]
smg	+2.41	10.98	+1.27	10.34	0.3	11.61	0.0	0.00225922	0.01447669	A_07_P045982	Z4655	cmr|orf, hypothetical protein [Z4655]
smpA	-4.85	12.12	-2.28	13.26	0.1	10.98	0.0	0.00002944	0.00076000	A_07_P019965	b2617	cmr|small membrane protein A [b2617]
smpB	-2.80	12.36	-1.48	13.10	0.0	11.61	0.1	0.00018606	0.00241091	A_07_P019980	b2620	cmr|small protein B [b2620]
sodA	+2.09	9.83	+1.06	9.30	0.0	10.36	0.0	0.00090299	0.00739560	A_07_P041367	c_4859	cmr|Superoxide dismutase [c_4859]
sodA	+2.53	7.94	+1.34	7.27	0.1	8.61	0.1	0.00070327	0.00614276	A_07_P047103	Z5453	cmr|superoxide dismutase, manganese [Z5453]
sodA	+2.04	8.38	+1.03	7.87	0.3	8.89	0.2	0.00939413	0.04259139	A_07_P019316	b3908	cmr|superoxide dismutase, manganese [b3908]
sodB	+6.67	12.61	+2.74	11.24	0.1	13.98	0.0	0.00001251	0.00051423	A_07_P018342	b1656	cmr|superoxide dismutase, iron [b1656]
sodB	+4.74	12.92	+2.25	11.80	0.0	14.05	0.1	0.00002602	0.00072493	A_07_P042333	c_2050	cmr|Superoxide dismutase [c_2050]
soxR	-2.28	8.81	-1.19	9.41	0.1	8.22	0.0	0.00086549	0.00719822	A_07_P008959	b4063	cmr|redox-sensing activator of soxS [b4063]
soxS	+2.65	11.23	+1.40	10.52	0.1	11.93	0.1	0.00059029	0.00544760	A_07_P008951	b4062	cmr|regulation of superoxide response regulon [b4062]
speB	-2.62	9.45	-1.39	10.14	0.0	8.75	0.1	0.00026764	0.00311206	A_07_P011831	b2937	cmr|agmatinase [b2937]
speD	+2.30	10.19	+1.20	9.58	0.2	10.79	0.1	0.00150225	0.01072141	A_07_P002565	b0120	cmr|S-adenosylmethionine decarboxylase [b0120]
speF	+3.33	8.42	+1.74	7.55	0.4	9.29	0.1	0.00211789	0.01377283	A_07_P009981	b0693	cmr|ornithine decarboxylase isozyme, inducible [b0693]
speG	-2.81	10.79	-1.49	11.54	0.1	10.05	0.0	0.00028659	0.00327435	A_07_P020551	b1584	cmr|spermidine N1-acetyltransferase [b1584]
spoT	-2.80	10.07	-1.49	10.81	0.1	9.33	0.1	0.00027541	0.00317893	A_07_P017193	b3650	cmr|bifunctional (p)ppGpp synthetase II and guanosine-3',5'-bis pyrophosphate 3'-pyrophosphohydrolase [b3650]
spr	+2.44	12.38	+1.29	11.73	0.1	13.02	0.1	0.00043116	0.00435418	A_07_P000674	b2175	cmr|putative lipoprotein [b2175]
srlB	+2.93	7.35	+1.55	6.58	0.2	8.13	0.0	0.00064532	0.00578255	A_07_P001966	b2704	cmr|PTS system, glucitol/sorbitol-specific enzyme IIA component [b2704]
srlE	+2.37	7.25	+1.24	6.63	0.1	7.87	0.0	0.00067470	0.00595430	A_07_P030595	c_3257	cmr|PTS system, glucitol/sorbitol-specific IIBC component [c_3257]
srlE	+2.45	7.47	+1.29	6.83	0.0	8.12	0.1	0.00049580	0.00479178	A_07_P001964	b2703	cmr|PTS system, glucitol/sorbitol-specific IIB component and second of two IIC components [b2703]
srmB	+3.91	8.93	+1.97	7.95	0.3	9.91	0.0	0.00026684	0.00310509	A_07_P042926	Z3859	cmr|ATP-dependent RNA helicase [Z3859]
srmB	+3.89	9.38	+1.96	8.40	0.0	10.36	0.0	0.00004432	0.00094837	A_07_P030217	c_3100	cmr|ATP-dependent RNA helicase srmB [c_3100]
ssb	-3.46	12.98	-1.79	13.87	0.0	12.08	0.0	0.00007147	0.00125866	A_07_P008940	b4059	cmr|ssDNA-binding protein [b4059]
ssbW	+19.42	13.85	+4.28	11.71	0.0	15.99	0.0	0.00000088	0.00019431	A_07_P051338	Z1440	cmr|putative single-stranded DNA binding protein [Z1440]
sseA	+9.67	11.65	+3.27	10.01	0.1	13.28	0.0	0.00000801	0.00041039	A_07_P013661	b2521	cmr|putative thiosulfate sulfurtransferase [b2521]
sspA	-4.34	13.57	-2.12	14.63	0.0	12.51	0.1	0.00003776	0.00087581	A_07_P012616	b3229	cmr|regulator of transcription; stringent starvation protein A [b3229]
sspB	-5.35	13.96	-2.42	15.17	0.0	12.75	0.0	0.00001630	0.00056947	A_07_P012612	b3228	cmr|stringent starvation protein B [b3228]
sstT	+2.17	8.26	+1.12	7.70	0.1	8.81	0.2	0.00228004	0.01455673	A_07_P000173	b3089	cmr|putative transport protein [b3089]
stpA	-2.16	10.67	-1.11	11.22	0.1	10.11	0.1	0.00093211	0.00756060	A_07_P020199	b2669	cmr|DNA-binding protein; H-NS-like protein; chaperone activity; RNA splicing? [b2669]
sucA	+91.76	10.65	+6.52	7.39	0.0	13.91	0.0	0.00000012	0.00019431	A_07_P010149	b0726	cmr|2-oxoglutarate dehydrogenase [b0726]
sucB	+45.69	12.07	+5.51	9.32	0.0	14.83	0.0	0.00000026	0.00019431	A_07_P010151	b0727	cmr|2-oxoglutarate dehydrogenase [b0727]
sucC	+55.82	11.22	+5.80	8.32	0.1	14.12	0.0	0.00000030	0.00019431	A_07_P010159	b0728	cmr|succinyl-CoA synthetase, beta subunit [b0728]
sucD	+36.23	10.53	+5.18	7.94	0.0	13.12	0.1	0.00000039	0.00019431	A_07_P010165	b0729	cmr|succinyl-CoA synthetase, alpha subunit [b0729]
sugE	-3.26	11.86	-1.71	12.71	0.1	11.00	0.1	0.00020118	0.00255851	A_07_P035585	c_5236	cmr|SugE protein [c_5236]
sugE	-3.41	9.53	-1.77	10.42	0.1	8.65	0.1	0.00009199	0.00148849	A_07_P048623	Z5755	cmr|suppresses groEL, may be chaperone [Z5755]
sugE	-4.10	10.25	-2.04	11.27	0.2	9.24	0.0	0.00020926	0.00262336	A_07_P010653	b4148	cmr|suppresses groEL, may be chaperone [b4148]
sugE	-12.28	8.96	-3.62	10.77	0.1	7.16	0.2	0.00000626	0.00036832	A_07_P011472	b4148	cmr|suppresses groEL, may be chaperone [b4148]
sulA	+2.65	15.01	+1.41	14.31	0.0	15.72	0.3	0.00196910	0.01302035	A_07_P005240	b0958	cmr|suppressor of lon; inhibits cell division and ftsZ ring formation [b0958]
sxy	-2.09	10.24	-1.06	10.77	0.1	9.71	0.0	0.00129644	0.00961427	A_07_P005243	b0959	cmr|orf, hypothetical protein [b0959]
tadA	-4.08	9.27	-2.03	10.29	0.0	8.26	0.1	0.00005689	0.00110173	A_07_P019700	b2559	cmr|putative deaminase [b2559]
talA	+2.22	8.13	+1.15	7.55	0.3	8.70	0.0	0.00464674	0.02485839	A_07_P013377	b2464	cmr|transaldolase A [b2464]
talB	+2.86	9.96	+1.52	9.20	0.1	10.72	0.0	0.00028694	0.00327593	A_07_P010731	b0008	cmr|transaldolase B [b0008]
tatA	-2.12	13.98	-1.09	14.53	0.0	13.44	0.0	0.00074192	0.00638990	A_07_P018990	b3836	cmr|orf, hypothetical protein [b3836]
tatB	-3.52	13.50	-1.81	14.41	0.0	12.60	0.0	0.00006673	0.00120840	A_07_P018999	b3838	cmr|orf, hypothetical protein [b3838]
tauA	-2.28	8.54	-1.19	9.14	0.3	7.95	0.0	0.00511323	0.02675396	A_07_P031067	c_0472	cmr|Taurine-binding periplasmic protein precursor [c_0472]
tdcA	+9.64	10.10	+3.27	8.46	0.2	11.73	0.4	0.00011928	0.00176667	A_07_P000316	b3118	cmr|transcriptional activator of tdc operon [b3118]
tdcB	+6.84	8.51	+2.77	7.12	0.1	9.90	0.1	0.00001321	0.00052395	A_07_P000312	b3117	cmr|threonine dehydratase, catabolic [b3117]
tdcC	+7.70	8.72	+2.94	7.24	0.3	10.19	0.1	0.00004948	0.00100904	A_07_P000306	b3116	cmr|anaerobically inducible L-threonine, L-serine permease [b3116]
tdcD	+5.18	7.46	+2.37	6.27	0.1	8.64	0.0	0.00002012	0.00063193	A_07_P000305	b3115	cmr|putative kinase [b3115]
tdcD	+13.92	9.13	+3.80	7.23	0.0	11.02	0.2	0.00000776	0.00041039	A_07_P045889	Z4467	cmr|putative kinase [Z4467]
tdcE	+2.77	9.09	+1.47	8.35	0.0	9.82	0.1	0.00032007	0.00353373	A_07_P034076	c_3872	cmr|Keto-acid formate acetyltransferase [c_3872]
tdh	+8.45	10.23	+3.08	8.69	0.0	11.77	0.0	0.00000478	0.00034614	A_07_P017024	b3616	cmr|threonine dehydrogenase [b3616]
terB	+2.21	8.59	+1.15	8.01	0.0	9.16	0.2	0.00275664	0.01676994	A_07_P046904	Z1173	cmr|putative phage inhibition, colicin resistance and tellurite [Z1173]
tfaR	-2.72	9.52	-1.45	10.25	0.0	8.80	0.1	0.00024272	0.00291707	A_07_P014841	b1373	cmr|orf, hypothetical protein [b1373]
tfaX	-2.09	7.49	-1.07	8.02	0.1	6.95	0.1	0.00154778	0.01096185	A_07_P016578	b0563	cmr|orf, hypothetical protein [b0563]
tgt	+9.05	10.62	+3.18	9.03	0.0	12.21	0.0	0.00000433	0.00033435	A_07_P021144	b0406	cmr|tRNA-guanine transglycosylase [b0406]
thiD	-5.39	10.00	-2.43	11.22	0.2	8.79	0.3	0.00033286	0.00364059	A_07_P003278	b2103	cmr|phosphomethylpyrimidine kinase [b2103]
thiG	+2.09	7.93	+1.06	7.40	0.0	8.46	0.1	0.00173298	0.01190921	A_07_P010631	b3991	cmr|thiamin biosynthesis, thiazole moiety [b3991]
thiK	+2.26	10.84	+1.17	10.25	0.0	11.42	0.1	0.00059909	0.00549159	A_07_P001731	b1106	cmr|putative beta-glucosidase [b1106]
thiM	-4.48	9.10	-2.16	10.18	0.0	8.02	0.2	0.00012725	0.00183667	A_07_P003284	b2104	cmr|hydoxyethylthiazole kinase [b2104]
thrA	+2.31	7.78	+1.21	7.18	0.1	8.38	0.1	0.00126505	0.00942593	A_07_P010704	b0002	cmr|aspartokinase I, homoserine dehydrogenase I [b0002]
thrB	+3.44	8.17	+1.78	7.27	0.2	9.06	0.1	0.00038180	0.00398874	A_07_P010710	b0003	cmr|homoserine kinase [b0003]
thrC	+3.89	9.62	+1.96	8.65	0.0	10.60	0.1	0.00009055	0.00147268	A_07_P035606	c_0005	cmr|Threonine synthase [c_0005]
thyA	+3.37	9.44	+1.75	8.56	0.1	10.31	0.1	0.00013156	0.00188305	A_07_P006119	b2827	cmr|thymidylate synthetase [b2827]
tktA	+6.81	11.60	+2.77	10.22	0.0	12.98	0.0	0.00000911	0.00043585	A_07_P032946	c_3520	cmr|Transketolase 1 [c_3520]
tktA	+3.25	8.19	+1.70	7.34	0.3	9.04	0.0	0.00100283	0.00794100	A_07_P011821	b2935	cmr|transketolase 1 isozyme [b2935]
tnaA	+185.98	10.41	+7.54	6.64	0.2	14.18	0.0	0.00000022	0.00019431	A_07_P057646	ECs4645	cmr|tryptophanase [ECs4645]
tnaA	+75.28	11.68	+6.23	8.56	0.1	14.80	0.1	0.00000037	0.00019431	A_07_P005461	b3708	cmr|tryptophanase [b3708]
tnaA	+153.65	10.56	+7.26	6.93	0.1	14.19	0.0	0.00000014	0.00019431	A_07_P049911	Z5203	cmr|tryptophanase [Z5203]
tnaB	+12.56	9.35	+3.65	7.52	0.3	11.18	0.0	0.00002526	0.00071646	A_07_P041909	c_4632	cmr|Low affinity tryptophan permease [c_4632]
tnaB	+3.98	8.02	+1.99	7.02	0.3	9.02	0.1	0.00036522	0.00389567	A_07_P005466	b3709	cmr|low affinity tryptophan permease [b3709]
tolB	+5.93	10.92	+2.57	9.64	0.2	12.20	0.0	0.00003600	0.00084763	A_07_P010216	b0740	cmr|periplasmic protein involved in the tonb-independent uptake of group A colicins [b0740]
tolC	+3.41	10.50	+1.77	9.61	0.0	11.38	0.0	0.00007176	0.00126242	A_07_P012308	b3035	cmr|outer membrane channel; specific tolerance to colicin E1; segregation of daughter chromosomes [b3035]
torD	-2.00	7.96	-1.00	8.46	0.1	7.46	0.2	0.00327607	0.01919557	A_07_P051271	Z1416	cmr|part of trimethylamine-N-oxide oxidoreductase [Z1416]
torS	-2.99	12.41	-1.58	13.20	0.1	11.62	0.0	0.00019174	0.00246301	A_07_P001197	b0993	cmr|sensor protein torS [b0993]
tpiA	+2.97	12.74	+1.57	11.95	0.0	13.53	0.1	0.00014613	0.00203963	A_07_P019375	b3919	cmr|triosephosphate isomerase [b3919]
tpx	+5.64	10.85	+2.50	9.60	0.0	12.10	0.1	0.00001575	0.00056283	A_07_P014605	b1324	cmr|thiol peroxidase [b1324]
treB	+2.35	8.19	+1.23	7.58	0.1	8.81	0.1	0.00073665	0.00635495	A_07_P003751	b4240	cmr|PTS system enzyme II, trehalose specific [b4240]
treR	-2.16	11.81	-1.11	12.36	0.1	11.26	0.0	0.00080398	0.00681264	A_07_P003760	b4241	cmr|repressor of treA,B,C [b4241]
trg	-2.08	9.75	-1.05	10.27	0.1	9.22	0.1	0.00132466	0.00972818	A_07_P009199	b1421	cmr|methyl-accepting chemotaxis protein III, ribose sensor receptor [b1421]
trkH	-2.50	8.92	-1.32	9.58	0.1	8.26	0.2	0.00092193	0.00751675	A_07_P019052	b3849	cmr|potassium uptake, requires TrkE [b3849]
trmA	-2.93	9.15	-1.55	9.93	0.1	8.38	0.2	0.00047535	0.00466588	A_07_P047177	Z5526	cmr|tRNA [Z5526]
trmD	-2.28	16.22	-1.19	16.82	0.0	15.63	0.1	0.00059234	0.00546330	A_07_P019915	b2607	cmr|tRNA methyltransferase; tRNA [b2607]
trmH	-4.16	10.12	-2.05	11.15	0.1	9.09	0.1	0.00006103	0.00115629	A_07_P017198	b3651	cmr|putative RNA methylase [b3651]
trmI	+6.22	12.78	+2.64	11.46	0.0	14.10	0.1	0.00001187	0.00050521	A_07_P011943	b2960	cmr|orf, hypothetical protein [b2960]
trmJ	-5.39	13.82	-2.43	15.04	0.0	12.61	0.0	0.00001565	0.00056283	A_07_P019564	b2532	cmr|putative ATP synthase beta subunit [b2532]
trpB	+3.84	8.62	+1.94	7.65	0.1	9.59	0.0	0.00006599	0.00120525	A_07_P014291	b1261	cmr|tryptophan synthase, beta protein [b1261]
trpB	+3.86	9.29	+1.95	8.31	0.0	10.26	0.0	0.00004525	0.00095882	A_07_P038879	c_1726	cmr|Tryptophan synthase beta chain [c_1726]
trpB	+2.65	8.63	+1.41	7.93	0.1	9.34	0.1	0.00050868	0.00488792	A_07_P044133	Z2550	cmr|tryptophan synthase, beta protein [Z2550]
trpR	+2.11	8.50	+1.08	7.96	0.0	9.04	0.0	0.00082597	0.00694666	A_07_P017646	b4393	cmr|regulator for trp operon and aroH; trp aporepressor [b4393]
trxA	-6.31	14.00	-2.66	15.33	0.0	12.67	0.0	0.00000937	0.00044530	A_07_P057729	ECs4714	cmr|thioredoxin 1 [ECs4714]
trxA	-7.73	13.63	-2.95	15.10	0.0	12.15	0.0	0.00000587	0.00036511	A_07_P005789	b3781	cmr|thioredoxin 1 [b3781]
trxA	-7.18	13.92	-2.84	15.35	0.1	12.50	0.0	0.00000758	0.00040630	A_07_P035554	c_4701	cmr|Thioredoxin 1 [c_4701]
trxB	+4.09	12.70	+2.03	11.69	0.1	13.72	0.1	0.00006174	0.00115851	A_07_P033812	c_1025	cmr|Thioredoxin reductase [c_1025]
tsgA	-3.34	8.10	-1.74	8.97	0.1	7.22	0.1	0.00009465	0.00151434	A_07_P017847	b3364	cmr|putative transport [b3364]
ttdR	-4.32	9.65	-2.11	10.71	0.1	8.60	0.2	0.00015909	0.00217908	A_07_P000033	b3060	cmr|putative transcriptional regulator LYSR-type [b3060]
tuf	+2.40	16.31	+1.26	15.67	0.1	16.94	0.0	0.00057869	0.00535939	A_07_P059760	ECs4190	cmr|protein chain elongation factor EF-Tu [ECs4190]
tuf	+2.48	16.82	+1.31	16.17	0.0	17.48	0.0	0.00029942	0.00336717	A_07_P046005	Z4697	cmr|protein chain elongation factor EF-Tu [Z4697]
tuf	+2.04	16.47	+1.03	15.96	0.0	16.99	0.0	0.00098339	0.00782734	A_07_P057936	ECs4903	cmr|protein chain elongation factor EF-Tu [ECs4903]
tufA	+2.45	16.70	+1.29	16.05	0.0	17.34	0.0	0.00032733	0.00359882	A_07_P017725	b3339	cmr|protein chain elongation factor EF-Tu [b3339]
tyrB	+2.24	8.04	+1.16	7.46	0.3	8.62	0.3	0.01011396	0.04509606	A_07_P037596	c_5031	cmr|Aromatic-amino-acid aminotransferase [c_5031]
tyrS	+3.47	8.39	+1.79	7.49	0.1	9.28	0.1	0.00009586	0.00152759	A_07_P020819	b1637	cmr|tyrosine tRNA synthetase [b1637]
ubiE	-3.77	13.74	-1.92	14.70	0.0	12.78	0.1	0.00006260	0.00116220	A_07_P018975	b3833	cmr|2-octaprenyl-6-methoxy-1,4-benzoquinone --> 2-octaprenyl-3-methyl-6-methoxy-1,4-benzoquinone [b3833]
ubiH	+2.62	11.91	+1.39	11.21	0.1	12.60	0.0	0.00029964	0.00336721	A_07_P045655	Z4244	cmr|2-octaprenyl-6-methoxyphenol--> 2-octaprenyl-6-methoxy-1, 4-benzoquinone [Z4244]
ubiH	+2.39	11.90	+1.26	11.27	0.0	12.53	0.0	0.00037546	0.00395400	A_07_P032895	c_3489	cmr|2-octaprenyl-6-methoxyphenol hydroxylase [c_3489]
ucpA	+4.17	8.83	+2.06	7.80	0.2	9.86	0.1	0.00012688	0.00183667	A_07_P013188	b2426	cmr|putative oxidoreductase [b2426]
ucpA	+5.31	11.46	+2.41	10.26	0.1	12.67	0.0	0.00002461	0.00070547	A_07_P041240	c_2960	cmr|Oxidoreductase ucpA [c_2960]
ugd	-2.62	8.97	-1.39	9.67	0.1	8.28	0.0	0.00026120	0.00308044	A_07_P015464	b2028	cmr|UDP-glucose 6-dehydrogenase [b2028]
ugd	-4.22	8.56	-2.08	9.60	0.1	7.52	0.1	0.00006241	0.00116220	A_07_P044992	Z3190	cmr|UDP-glucose 6-dehydrogenase [Z3190]
ugpB	+2.00	8.12	+1.00	7.62	0.1	8.62	0.1	0.00227880	0.01455673	A_07_P018281	b3453	cmr|sn-glycerol 3-phosphate transport system; periplasmic binding protein [b3453]
ugpC	+2.90	8.27	+1.54	7.50	0.0	9.03	0.1	0.00016049	0.00218551	A_07_P018267	b3450	cmr|ATP-binding component of sn-glycerol 3-phosphate transport system [b3450]
uhpT	-8.39	8.59	-3.07	10.12	0.0	7.05	0.0	0.00000471	0.00034354	A_07_P017270	b3666	cmr|hexose phosphate transport protein [b3666]
uhpT	-10.55	8.84	-3.40	10.54	0.0	7.14	0.1	0.00000508	0.00034758	A_07_P038972	c_4590	cmr|Hexose phosphate transport protein [c_4590]
ulaD	+2.05	9.21	+1.03	8.70	0.3	9.73	0.1	0.00561809	0.02881149	A_07_P011698	b4196	cmr|probable hexulose-6-phosphate synthase [b4196]
umuC	-2.60	11.87	-1.38	12.56	0.1	11.18	0.0	0.00026512	0.00309642	A_07_P006875	b1184	cmr|SOS mutagenesis and repair [b1184]
ung	-2.95	10.32	-1.56	11.10	0.1	9.54	0.1	0.00030249	0.00339199	A_07_P041614	c_3105	cmr|Uracil-DNA glycosylase [c_3105]
upp	+10.78	12.49	+3.43	10.78	0.2	14.21	0.1	0.00000961	0.00045183	A_07_P013547	b2498	cmr|uracil phosphoribosyltransferase [b2498]
uspA	+8.03	12.47	+3.01	10.97	0.0	13.98	0.1	0.00000508	0.00034758	A_07_P007413	b3495	cmr|universal stress protein; broad regulatory function? [b3495]
uspA	+6.82	11.53	+2.77	10.14	0.0	12.91	0.1	0.00000938	0.00044530	A_07_P049374	Z4895	cmr|universal stress protein broad regulatory function? [Z4895]
uspA	+6.92	12.00	+2.79	10.60	0.1	13.39	0.1	0.00001985	0.00062874	A_07_P034623	c_4293	cmr|Universal stress protein A [c_4293]
uspF	+17.19	11.33	+4.10	9.28	0.1	13.38	0.1	0.00000147	0.00022429	A_07_P014857	b1376	cmr|putative filament protein [b1376]
uvrA	-5.86	13.35	-2.55	14.63	0.0	12.08	0.1	0.00001396	0.00053770	A_07_P008933	b4058	cmr|excision nuclease subunit A [b4058]
uvrB	-8.71	13.26	-3.12	14.82	0.0	11.70	0.0	0.00000445	0.00033554	A_07_P033475	c_0860	cmr|Excinuclease ABC subunit B [c_0860]
uvrB	-6.34	12.69	-2.66	14.02	0.1	11.36	0.1	0.00002039	0.00063193	A_07_P004347	b0779	cmr|DNA repair; excision nuclease subunit B [b0779]
uvrY	-2.13	9.90	-1.09	10.44	0.0	9.35	0.1	0.00100322	0.00794100	A_07_P014931	b1914	cmr|putative 2-component transcriptional regulator [b1914]
uxaA	+6.48	8.24	+2.70	6.89	0.3	9.59	0.0	0.00007544	0.00130949	A_07_P000185	b3091	cmr|altronate hydrolase [b3091]
uxaA	+9.36	9.43	+3.23	7.82	0.1	11.05	0.1	0.00000455	0.00033765	A_07_P034058	c_3849	cmr|Altronate hydrolase [c_3849]
uxaC	+23.49	9.59	+4.55	7.32	0.0	11.87	0.0	0.00000070	0.00019431	A_07_P000188	b3092	cmr|uronate isomerase [b3092]
uxuB	+2.03	9.40	+1.02	8.89	0.1	9.91	0.1	0.00130641	0.00967457	A_07_P037204	c_5403	cmr|D-mannonate oxidoreductase [c_5403]
uxuB	+3.67	8.14	+1.87	7.20	0.1	9.08	0.0	0.00007440	0.00129725	A_07_P047511	Z5921	cmr|mannonate oxidoreductase [Z5921]
vacJ	+4.99	8.18	+2.32	7.02	0.1	9.34	0.0	0.00002233	0.00067002	A_07_P032512	c_2890	cmr|VacJ lipoprotein precursor [c_2890]
vacJ	+3.68	8.80	+1.88	7.86	0.3	9.74	0.2	0.00069363	0.00608327	A_07_P014018	b2346	cmr|lipoprotein precursor [b2346]
viaA	-2.01	9.24	-1.01	9.75	0.2	8.74	0.2	0.00834807	0.03900062	A_07_P005646	b3745	cmr|orf, hypothetical protein [b3745]
waaP	-4.75	10.06	-2.25	11.19	0.2	8.94	0.0	0.00006289	0.00116627	A_07_P049579	Z5054	cmr|putative LPS biosynthesis enzyme [Z5054]
waaQ	-2.00	10.96	-1.00	11.46	0.1	10.46	0.1	0.00297908	0.01777288	A_07_P049589	Z5056	cmr|putative LPS biosynthesis enzyme [Z5056]
wbbJ	+2.31	7.81	+1.21	7.20	0.1	8.41	0.0	0.00071188	0.00619557	A_07_P015485	b2033	cmr|putative O-acetyl transferase [b2033]
wbdN	-3.41	10.44	-1.77	11.32	0.1	9.55	0.0	0.00007779	0.00133123	A_07_P045051	Z3204	cmr|glycosyl transferase [Z3204]
wcaK	+2.16	7.39	+1.11	6.83	0.1	7.95	0.1	0.00101760	0.00802729	A_07_P032034	c_2570	cmr|Colanic acid biosynthesis protein wcaK [c_2570]
wrbA	+3.12	9.51	+1.64	8.69	0.1	10.33	0.1	0.00030461	0.00340122	A_07_P001254	b1004	cmr|trp repressor binding protein; affects association of trp repressor and operator [b1004]
wzyE	+2.34	8.25	+1.23	7.63	0.2	8.86	0.1	0.00132034	0.00971976	A_07_P005852	b3793	cmr|TDP-Fuc4NAc:lipidII transferase; synthesis of enterobacterial common antigen [b3793]
wzzB	-2.93	11.38	-1.55	12.15	0.0	10.60	0.0	0.00014208	0.00199905	A_07_P031985	c_2554	cmr|Chain length determinant protein [c_2554]
wzzB	-2.54	8.97	-1.34	9.64	0.2	8.30	0.1	0.00118844	0.00902162	A_07_P044990	Z3189	cmr|regulator of length of O-antigen component of [Z3189]
wzzE	-2.34	8.48	-1.22	9.10	0.2	7.87	0.0	0.00132980	0.00973786	A_07_P005801	b3785	cmr|putative transport protein [b3785]
xerC	+2.27	8.03	+1.18	7.44	0.3	8.62	0.0	0.00346557	0.01999336	A_07_P049986	Z5328	cmr|site-specific recombinase, acts on cer sequence of [Z5328]
xerC	+2.07	8.31	+1.05	7.78	0.0	8.83	0.2	0.00267201	0.01642642	A_07_P039112	c_4732	cmr|Integrase/recombinase xerC [c_4732]
xerD	-2.61	11.98	-1.38	12.68	0.0	11.29	0.1	0.00029356	0.00333465	A_07_P006436	b2894	cmr|site-specific recombinase [b2894]
xseA	-2.10	11.41	-1.07	11.95	0.3	10.88	0.1	0.00754737	0.03612822	A_07_P013602	b2509	cmr|exonuclease VII, large subunit [b2509]
xseA	-2.07	9.78	-1.05	10.30	0.1	9.25	0.1	0.00131113	0.00969583	A_07_P030157	c_3028	cmr|Exodeoxyribonuclease VII large subunit [c_3028]
xthA	+2.03	10.16	+1.02	9.65	0.0	10.67	0.1	0.00112198	0.00866326	A_07_P044436	Z2781	cmr|exonuclease III [Z2781]
yaaA	+2.35	9.18	+1.23	8.57	0.1	9.80	0.1	0.00097147	0.00775997	A_07_P010722	b0006	cmr|orf, hypothetical protein [b0006]
yaaH	-2.35	8.60	-1.23	9.21	0.1	7.98	0.0	0.00077551	0.00661768	A_07_P010741	b0010	cmr|orf, hypothetical protein [b0010]
yaaY	-3.70	8.41	-1.89	9.35	0.0	7.47	0.3	0.00034645	0.00374618	A_07_P010806	b0024	cmr|orf, hypothetical protein [b0024]
yabI	-3.45	8.36	-1.78	9.25	0.1	7.46	0.3	0.00056764	0.00528823	A_07_P035723	c_0081	cmr|Hypothetical protein yabI [c_0081]
yabI	-3.16	8.43	-1.66	9.26	0.1	7.60	0.0	0.00011198	0.00169030	A_07_P035723	c_0081	cmr|Hypothetical protein yabI [c_0081]
yabI	-3.08	8.16	-1.62	8.97	0.0	7.35	0.1	0.00014173	0.00199583	A_07_P035723	c_0081	cmr|Hypothetical protein yabI [c_0081]
yabI	-3.66	8.13	-1.87	9.07	0.0	7.20	0.2	0.00015157	0.00209512	A_07_P035723	c_0081	cmr|Hypothetical protein yabI [c_0081]
yabI	-3.24	8.31	-1.70	9.16	0.0	7.46	0.2	0.00024138	0.00291212	A_07_P035723	c_0081	cmr|Hypothetical protein yabI [c_0081]
yacL	+2.50	13.06	+1.32	12.40	0.1	13.72	0.0	0.00053770	0.00509054	A_07_P002559	b0119	cmr|orf, hypothetical protein [b0119]
yadC	-19.53	11.50	-4.29	13.64	0.0	9.35	0.0	0.00000093	0.00019431	A_07_P035826	c_0166	cmr|Hypothetical fimbrial-like protein yadC precursor [c_0166]
yadD	-2.11	10.63	-1.07	11.17	0.0	10.09	0.0	0.00076157	0.00653062	A_07_P035809	c_0162	cmr|Hypothetical protein yadD [c_0162]
yadH	-2.26	8.65	-1.17	9.23	0.1	8.06	0.1	0.00087088	0.00723162	A_07_P049187	Z0139	cmr|orf, hypothetical protein [Z0139]
yadH	-2.00	8.84	-1.00	9.34	0.0	8.34	0.1	0.00181180	0.01228992	A_07_P002602	b0128	cmr|orf, hypothetical protein [b0128]
yadS	-2.48	8.20	-1.31	8.86	0.1	7.55	0.2	0.00179934	0.01225015	A_07_P002749	b0157	cmr|orf, hypothetical protein [b0157]
yafE	+2.11	7.72	+1.08	7.18	0.0	8.26	0.1	0.00132045	0.00971976	A_07_P041461	c_0247	cmr|Hypothetical protein yafE [c_0247]
yafJ	+2.08	10.39	+1.05	9.87	0.0	10.92	0.0	0.00090310	0.00739560	A_07_P007830	b0223	cmr|putative amidotransferase [b0223]
yafK	-7.92	9.73	-2.98	11.22	0.2	8.23	0.1	0.00002622	0.00072778	A_07_P007831	b0224	cmr|orf, hypothetical protein [b0224]
yafP	-2.19	9.91	-1.13	10.48	0.1	9.35	0.1	0.00095748	0.00769113	A_07_P007881	b0234	cmr|orf, hypothetical protein [b0234]
yafY	-2.63	8.50	-1.40	9.20	0.4	7.80	0.0	0.00616345	0.03084453	A_07_P007962	b0251	cmr|orf, hypothetical protein [b0251]
yahA	-4.24	8.68	-2.08	9.73	0.1	7.64	0.0	0.00005451	0.00107551	A_07_P008268	b0315	cmr|orf, hypothetical protein [b0315]
yahM	-8.29	9.60	-3.05	11.12	0.1	8.07	0.1	0.00000583	0.00036511	A_07_P008330	b0327	cmr|orf, hypothetical protein [b0327]
yaiA	-5.86	12.02	-2.55	13.29	0.1	10.74	0.1	0.00003577	0.00084433	A_07_P021060	b0389	cmr|orf, hypothetical protein [b0389]
yaiI	+3.25	10.16	+1.70	9.32	0.5	11.01	0.0	0.00481491	0.02557557	A_07_P021047	b0387	cmr|orf, hypothetical protein [b0387]
yaiL	+2.76	7.89	+1.47	7.16	0.2	8.63	0.0	0.00088081	0.00727980	A_07_P031039	c_0462	cmr|Hypothetical protein yaiL [c_0462]
yaiO	-2.68	8.00	-1.42	8.71	0.2	7.29	0.1	0.00054648	0.00516435	A_07_P020918	b0358	cmr|orf, hypothetical protein [b0358]
yajC	+7.60	9.85	+2.93	8.39	0.3	11.32	0.1	0.00006129	0.00115791	A_07_P021148	b0407	cmr|orf, hypothetical protein [b0407]
ybaA	-2.23	8.34	-1.16	8.92	0.2	7.77	0.0	0.00181903	0.01231780	A_07_P021388	b0456	cmr|orf, hypothetical protein [b0456]
ybaB	+4.96	12.35	+2.31	11.19	0.0	13.50	0.0	0.00001976	0.00062719	A_07_P021461	b0471	cmr|orf, hypothetical protein [b0471]
ybaC	-13.32	10.54	-3.74	12.41	0.1	8.67	0.1	0.00000317	0.00029057	A_07_P031201	c_0596	cmr|Acetyl esterase [c_0596]
ybaK	+2.98	9.03	+1.58	8.24	0.1	9.82	0.0	0.00020341	0.00257851	A_07_P021513	b0481	cmr|orf, hypothetical protein [b0481]
ybaL	-3.33	10.41	-1.73	11.28	0.1	9.54	0.1	0.00020737	0.00261010	A_07_P021498	b0478	cmr|putative transport protein [b0478]
ybaQ	-9.53	10.66	-3.25	12.29	0.1	9.03	0.2	0.00001367	0.00052871	A_07_P021524	b0483	cmr|orf, hypothetical protein [b0483]
ybaR	-2.17	9.89	-1.12	10.45	0.2	9.33	0.1	0.00358689	0.02058019	A_07_P031208	c_0604	cmr|Copper-transporting P-type ATPase [c_0604]
ybbJ	-2.72	10.54	-1.45	11.26	0.0	9.82	0.0	0.00020380	0.00258139	A_07_P031218	c_0609	cmr|Hypothetical protein ybbJ [c_0609]
ybbT	+5.47	8.95	+2.45	7.72	0.3	10.18	0.0	0.00010005	0.00157212	A_07_P031224	c_0620	cmr|Ureidoglycolate hydrolase [c_0620]
ybcO	+7.19	12.25	+2.85	10.83	0.0	13.68	0.1	0.00000945	0.00044666	A_07_P016514	b0549	cmr|orf, hypothetical protein [b0549]
ybcU	+2.56	8.01	+1.36	7.33	0.1	8.69	0.1	0.00037759	0.00396055	A_07_P038504	c_1564	cmr|Bor protein homolog from lambdoid prophage DLP12 [c_1564]
ybdD	+4.73	8.64	+2.24	7.52	0.0	9.76	0.0	0.00002337	0.00068274	A_07_P010317	ABE-0285033	asap|conserved protein [b4512]
ybdM	-2.52	7.98	-1.33	8.64	0.1	7.31	0.2	0.00129500	0.00960813	A_07_P016765	b0601	cmr|orf, hypothetical protein [b0601]
ybdN	-5.58	10.63	-2.48	11.87	0.0	9.39	0.1	0.00002584	0.00072358	A_07_P016768	b0602	cmr|orf, hypothetical protein [b0602]
ybeA	-3.81	12.25	-1.93	13.22	0.0	11.29	0.0	0.00004878	0.00100402	A_07_P009747	b0636	cmr|orf, hypothetical protein [b0636]
ybeB	-3.74	13.29	-1.90	14.24	0.0	12.34	0.0	0.00004963	0.00100955	A_07_P009752	b0637	cmr|orf, hypothetical protein [b0637]
ybeL	+2.65	11.08	+1.41	10.38	0.3	11.79	0.1	0.00191148	0.01276266	A_07_P009784	b0643	cmr|putative alpha helical protein [b0643]
ybeY	+2.80	10.27	+1.49	9.52	0.1	11.01	0.2	0.00061053	0.00556489	A_07_P009856	b0659	cmr|orf, hypothetical protein [b0659]
ybeZ	+2.71	10.42	+1.44	9.71	0.0	11.14	0.1	0.00024712	0.00296089	A_07_P009865	b0660	cmr|putative ATP-binding protein in pho regulon [b0660]
ybfA	-8.86	11.00	-3.15	12.58	0.0	9.43	0.2	0.00001187	0.00050521	A_07_P010014	b0699	cmr|orf, hypothetical protein [b0699]
ybgA	-5.68	9.54	-2.51	10.80	0.1	8.29	0.1	0.00001649	0.00057297	A_07_P033376	c_0786	cmr|Hypothetical protein ybgA [c_0786]
ybgE	+3.57	8.45	+1.84	7.54	0.1	9.37	0.1	0.00010733	0.00164539	A_07_P010194	b0735	cmr|orf, hypothetical protein [b0735]
ybgO	+2.14	7.48	+1.10	6.93	0.1	8.03	0.0	0.00095513	0.00768114	A_07_P010097	b0716	cmr|orf, hypothetical protein [b0716]
ybhA	+2.80	10.14	+1.49	9.39	0.2	10.88	0.0	0.00047091	0.00463958	A_07_P004285	b0766	cmr|putative phosphatase [b0766]
ybhA	+2.55	10.03	+1.35	9.35	0.1	10.71	0.0	0.00047969	0.00468366	A_07_P046367	Z0936	cmr|putative phosphatase [Z0936]
ybhC	+3.18	8.14	+1.67	7.30	0.0	8.97	0.0	0.00009584	0.00152759	A_07_P051991	Z0943	cmr|putative pectinesterase [Z0943]

ybhI	-2.74	10.13	-1.45	10.86	0.2	9.41	0.1	0.00119085	0.00903117	A_07_P004302	b0770	cmr|putative membrane pump protein [b0770]
ybhQ	+2.02	10.28	+1.01	9.78	0.1	10.79	0.0	0.00183269	0.01238361	A_07_P004406	b0791	cmr|orf, hypothetical protein [b0791]
ybiB	+2.30	11.72	+1.20	11.12	0.1	12.32	0.1	0.00094221	0.00761113	A_07_P004455	b0800	cmr|putative enzyme [b0800]
ybiB	+2.69	10.06	+1.43	9.35	0.1	10.78	0.0	0.00024534	0.00294404	A_07_P046583	Z1021	cmr|putative enzyme [Z1021]
ybiB	+3.55	11.13	+1.83	10.21	0.1	12.04	0.0	0.00012135	0.00178598	A_07_P041584	c_0884	cmr|Hypothetical protein ybiB [c_0884]
ybiC	+3.47	8.76	+1.80	7.86	0.1	9.66	0.1	0.00013574	0.00193052	A_07_P042097	c_0885	cmr|Hypothetical oxidoreductase ybiC [c_0885]
ybiH	+2.65	7.67	+1.41	6.96	0.2	8.37	0.0	0.00112574	0.00867527	A_07_P004432	b0796	cmr|putative transcriptional regulator [b0796]
ybiJ	-4.64	9.84	-2.21	10.95	0.2	8.73	0.1	0.00010899	0.00166313	A_07_P004463	b0802	cmr|orf, hypothetical protein [b0802]
ybiN	+2.23	8.26	+1.16	7.68	0.1	8.84	0.0	0.00068183	0.00600378	A_07_P033505	c_0892	cmr|Hypothetical protein ybiN [c_0892]
ybiO	-2.94	9.82	-1.56	10.60	0.1	9.04	0.0	0.00019180	0.00246301	A_07_P004491	b0808	cmr|putative transport protein [b0808]
ybiR	-2.25	9.95	-1.17	10.54	0.1	9.37	0.1	0.00108695	0.00843837	A_07_P004542	b0818	cmr|orf, hypothetical protein [b0818]
ybiS	-2.19	10.67	-1.13	11.23	0.0	10.10	0.1	0.00073822	0.00636505	A_07_P004547	b0819	cmr|orf, hypothetical protein [b0819]
ybiS	-2.34	12.34	-1.23	12.96	0.1	11.73	0.0	0.00046052	0.00457021	A_07_P042114	c_0905	cmr|Protein ybiS precursor [c_0905]
ybiT	-13.51	10.36	-3.76	12.23	0.1	8.48	0.2	0.00000856	0.00042498	A_07_P004555	b0820	cmr|putative ATP-binding component of a transport system [b0820]
ybjG	-30.06	10.94	-4.91	13.40	0.0	8.49	0.2	0.00000161	0.00023544	A_07_P004660	b0841	cmr|orf, hypothetical protein [b0841]
ybjL	-10.93	10.15	-3.45	11.87	0.1	8.42	0.2	0.00001801	0.00059959	A_07_P004688	b0847	cmr|putative transport protein [b0847]
ybjM	-3.67	9.15	-1.87	10.08	0.1	8.21	0.0	0.00006225	0.00116220	A_07_P004692	b0848	cmr|orf, hypothetical protein [b0848]
ybjN	+3.63	10.56	+1.86	9.63	0.1	11.49	0.0	0.00007693	0.00132177	A_07_P004718	b0853	cmr|putative sensory transduction regulator [b0853]
ybjP	+2.24	9.11	+1.16	8.52	0.1	9.69	0.1	0.00123438	0.00924113	A_07_P052022	Z1095	cmr|putative enzyme [Z1095]
ybjT	+2.40	7.72	+1.26	7.09	0.0	8.35	0.0	0.00037759	0.00396055	A_07_P004800	b0869	cmr|putative dTDP-glucose enzyme [b0869]
ybjX	-4.53	10.74	-2.18	11.83	0.0	9.65	0.1	0.00003155	0.00079211	A_07_P004839	b0877	cmr|putative enzyme [b0877]
ybjX	-4.97	12.47	-2.31	13.62	0.0	11.31	0.0	0.00001916	0.00061714	A_07_P042136	c_1012	cmr|Hypothetical protein ybjX [c_1012]
ycaD	-2.22	8.25	-1.15	8.82	0.1	7.67	0.0	0.00116740	0.00891302	A_07_P033821	c_1037	cmr|Hypothetical protein ycaD [c_1037]
ycaO	+2.01	8.93	+1.01	8.43	0.0	9.44	0.1	0.00217482	0.01405686	A_07_P004973	b0905	cmr|orf, hypothetical protein [b0905]
ycbG	+3.74	9.34	+1.90	8.38	0.0	10.29	0.0	0.00005164	0.00103969	A_07_P005226	b0956	cmr|putative dehydrogenase [b0956]
ycbJ	+2.51	9.06	+1.33	8.39	0.0	9.72	0.1	0.00061142	0.00556489	A_07_P005042	b0919	cmr|orf, hypothetical protein [b0919]
yccA	+2.03	11.11	+1.02	10.60	0.1	11.62	0.1	0.00177221	0.01212591	A_07_P005300	b0970	cmr|putative carrier/transport protein [b0970]
yccM	-2.42	7.84	-1.28	8.48	0.2	7.20	0.1	0.00168022	0.01161762	A_07_P001194	b0992	cmr|orf, hypothetical protein [b0992]
yccW	+2.00	9.59	+1.00	9.09	0.0	10.09	0.1	0.00143457	0.01033203	A_07_P005282	b0967	cmr|putative oxidoreductase [b0967]
yccX	-2.53	13.24	-1.34	13.91	0.1	12.57	0.0	0.00038524	0.00401672	A_07_P005289	b0968	cmr|orf, hypothetical protein [b0968]
ycdT	-7.95	10.59	-2.99	12.08	0.0	9.09	0.1	0.00001200	0.00050646	A_07_P001352	b1025	cmr|orf, hypothetical protein [b1025]
ycdZ	-4.35	9.85	-2.12	10.91	0.1	8.79	0.1	0.00006658	0.00120840	A_07_P001391	b1036	cmr|orf, hypothetical protein [b1036]
yceH	+2.67	8.43	+1.42	7.72	0.2	9.14	0.0	0.00049233	0.00477000	A_07_P040780	c_1334	cmr|Hypothetical protein yceH [c_1334]
yceH	+2.14	8.35	+1.10	7.80	0.1	8.90	0.1	0.00114540	0.00878383	A_07_P001540	b1067	cmr|orf, hypothetical protein [b1067]
ycfJ	-7.74	9.69	-2.95	11.16	0.1	8.21	0.1	0.00000804	0.00041039	A_07_P001753	b1110	cmr|orf, hypothetical protein [b1110]
ycfL	+4.35	9.69	+2.12	8.63	0.3	10.75	0.0	0.00029772	0.00335322	A_07_P001721	b1104	cmr|orf, hypothetical protein [b1104]
ycfP	+3.19	13.84	+1.67	13.01	0.1	14.68	0.1	0.00016810	0.00224857	A_07_P001743	b1108	cmr|orf, hypothetical protein [b1108]
ycfQ	-7.73	10.97	-2.95	12.44	0.1	9.49	0.1	0.00000848	0.00042498	A_07_P001759	b1111	cmr|orf, hypothetical protein [b1111]
ycfU	-2.04	9.49	-1.03	10.01	0.1	8.98	0.0	0.00113736	0.00873918	A_07_P052403	Z1757	cmr|orf, hypothetical protein [Z1757]
ycfZ	-2.09	7.59	-1.06	8.12	0.0	7.06	0.1	0.00155417	0.01097764	A_07_P001806	b1121	cmr|homolog of virulence factor [b1121]
ycgK	+2.91	10.15	+1.54	9.38	0.1	10.92	0.0	0.00016269	0.00220238	A_07_P006843	b1178	cmr|orf, hypothetical protein [b1178]
ycgZ	+6.24	9.46	+2.64	8.14	0.2	10.78	0.0	0.00004970	0.00100963	A_07_P042226	c_1607	cmr|Hypothetical protein ycgZ [c_1607]
ychA	+3.06	10.12	+1.62	9.31	0.2	10.93	0.1	0.00036303	0.00387759	A_07_P007024	b1214	cmr|orf, hypothetical protein [b1214]
ychM	-2.28	9.13	-1.19	9.72	0.0	8.54	0.0	0.00052686	0.00502151	A_07_P038791	c_1664	cmr|Putative sulfate transporter ychM [c_1664]
ychN	+3.02	9.25	+1.59	8.45	0.3	10.04	0.1	0.00097468	0.00778163	A_07_P007047	b1219	cmr|orf, hypothetical protein [b1219]
ychN	+2.91	9.16	+1.54	8.39	0.2	9.93	0.0	0.00055626	0.00522848	A_07_P050787	Z1994	cmr|orf, hypothetical protein [Z1994]
ychO	-2.86	8.19	-1.52	8.94	0.2	7.43	0.2	0.00141589	0.01021151	A_07_P007051	b1220	cmr|putative factor [b1220]
yciG	-2.98	12.85	-1.58	13.64	0.2	12.06	0.3	0.00199382	0.01313037	A_07_P010380	b1259	cmr|orf, hypothetical protein [b1259]
yciH	+3.75	11.77	+1.91	10.82	0.1	12.73	0.1	0.00008848	0.00145271	A_07_P041123	c_1751	cmr|Protein yciH [c_1751]
yciH	+4.47	12.04	+2.16	10.96	0.0	13.12	0.0	0.00002760	0.00073830	A_07_P014394	b1282	cmr|orf, hypothetical protein [b1282]
yciI	+12.16	10.08	+3.60	8.27	0.1	11.88	0.0	0.00000463	0.00033872	A_07_P007191	b1251	cmr|orf, hypothetical protein [b1251]
yciI	+11.46	10.35	+3.52	8.59	0.1	12.11	0.0	0.00000304	0.00028634	A_07_P050796	Z2029	cmr|orf, hypothetical protein [Z2029]
yciK	+2.69	10.94	+1.43	10.23	0.1	11.66	0.0	0.00028041	0.00322248	A_07_P014337	b1271	cmr|putative oxidoreductase [b1271]
yciO	-2.56	8.82	-1.36	9.50	0.0	8.15	0.1	0.00038230	0.00399135	A_07_P044117	Z2543	cmr|orf, hypothetical protein [Z2543]
yciO	-3.53	10.60	-1.82	11.51	0.0	9.69	0.0	0.00006134	0.00115791	A_07_P038891	c_1732	cmr|Protein yciO [c_1732]
yciO	-2.67	9.67	-1.42	10.38	0.0	8.96	0.1	0.00031859	0.00352489	A_07_P014319	b1267	cmr|orf, hypothetical protein [b1267]
yciQ	-2.27	7.47	-1.18	8.06	0.0	6.88	0.3	0.00398987	0.02214967	A_07_P014324	b1268	cmr|orf, hypothetical protein [b1268]
yciW	-2.27	12.36	-1.18	12.95	0.3	11.77	0.1	0.00308684	0.01829102	A_07_P014418	b1287	cmr|putative oxidoreductase [b1287]
ycjX	-2.08	11.83	-1.05	12.36	0.0	11.31	0.2	0.00190194	0.01272597	A_07_P048461	Z2458	cmr|putative EC 2.1 enzymes [Z2458]
ydbJ	+2.50	10.45	+1.32	9.79	0.1	11.11	0.1	0.00037353	0.00394177	A_07_P010409	ABE-0285050	asap|predicted protein [b4529]
ydbK	+4.89	8.92	+2.29	7.78	0.1	10.07	0.0	0.00003337	0.00081556	A_07_P014866	b1378	cmr|putative oxidoreductase, Fe-S subunit [b1378]
ydcH	+8.43	9.06	+3.08	7.52	0.0	10.60	0.0	0.00000502	0.00034758	A_07_P009222	b1426	cmr|orf, hypothetical protein [b1426]
ydcI	-2.11	8.66	-1.08	9.20	0.1	8.12	0.0	0.00091776	0.00749052	A_07_P009204	b1422	cmr|putative transcriptional regulator LYSR-type [b1422]
ydcY	+4.68	12.07	+2.23	10.96	0.0	13.19	0.1	0.00002724	0.00073404	A_07_P009325	b1446	cmr|orf, hypothetical protein [b1446]
yddA	-2.29	7.56	-1.20	8.15	0.3	6.96	0.1	0.00362174	0.02069114	A_07_P009570	b1496	cmr|putative ATP-binding component of a transport system [b1496]
yddB	-2.27	7.54	-1.18	8.14	0.4	6.95	0.0	0.00708866	0.03438197	A_07_P048054	Z2213	cmr|orf, hypothetical protein [Z2213]
ydeA	-4.26	8.49	-2.09	9.53	0.2	7.44	0.0	0.00010327	0.00160656	A_07_P020286	b1528	cmr|putative resistance / regulatory protein [b1528]
ydfG	+2.64	8.39	+1.40	7.69	0.0	9.09	0.1	0.00033480	0.00365537	A_07_P020342	b1539	cmr|putative oxidoreductase [b1539]
ydfM	-8.19	8.69	-3.03	10.21	0.1	7.18	0.1	0.00000664	0.00038136	A_07_P031275	c_0649	cmr|Tail fiber assembly protein homolog from lambdoid [c_0649]
ydfZ	+3.13	11.45	+1.64	10.63	0.2	12.27	0.0	0.00025692	0.00304818	A_07_P020351	b1541	cmr|orf, hypothetical protein [b1541]
ydgH	+3.18	9.61	+1.67	8.78	0.2	10.45	0.0	0.00036162	0.00386772	A_07_P020654	b1604	cmr|orf, hypothetical protein [b1604]
ydgJ	+2.46	8.44	+1.30	7.79	0.2	9.09	0.2	0.00268868	0.01650314	A_07_P035111	c_2016	cmr|Hypothetical oxidoreductase ydgJ [c_2016]
ydgJ	+2.10	7.76	+1.07	7.22	0.0	8.30	0.1	0.00092859	0.00755454	A_07_P020751	b1624	cmr|orf, hypothetical protein [b1624]
ydgK	-3.13	9.21	-1.65	10.03	0.1	8.39	0.1	0.00024212	0.00291652	A_07_P020765	b1626	cmr|orf, hypothetical protein [b1626]
ydhI	-3.58	8.22	-1.84	9.14	0.2	7.31	0.0	0.00018902	0.00244038	A_07_P020850	b1643	cmr|orf, hypothetical protein [b1643]
ydhJ	-2.78	7.94	-1.48	8.68	0.1	7.20	0.1	0.00032756	0.00359886	A_07_P020852	b1644	cmr|putative membrane protein [b1644]
ydhJ	-2.41	7.81	-1.27	8.45	0.1	7.18	0.1	0.00067437	0.00595430	A_07_P020852	b1644	cmr|putative membrane protein [b1644]
ydhM	+2.20	8.12	+1.14	7.55	0.4	8.69	0.1	0.01057925	0.04681273	A_07_P042330	c_2042	cmr|Hypothetical transcriptional regulator ydhM [c_2042]
ydhM	+3.43	9.16	+1.78	8.27	0.2	10.05	0.1	0.00034389	0.00372619	A_07_P020877	b1649	cmr|orf, hypothetical protein [b1649]
ydhR	+2.39	8.97	+1.26	8.34	0.1	9.59	0.1	0.00098992	0.00786343	A_07_P018387	b1667	cmr|orf, hypothetical protein [b1667]
ydhV	+2.44	8.25	+1.29	7.61	0.1	8.89	0.1	0.00086088	0.00716707	A_07_P018419	b1673	cmr|orf, hypothetical protein [b1673]
ydhZ	+2.43	8.46	+1.28	7.82	0.1	9.10	0.1	0.00048884	0.00475369	A_07_P018430	b1675	cmr|orf, hypothetical protein [b1675]
ydiA	+4.46	8.20	+2.16	7.12	0.1	9.28	0.1	0.00003779	0.00087581	A_07_P018569	b1703	cmr|orf, hypothetical protein [b1703]
ydiI	+2.52	9.25	+1.33	8.59	0.1	9.92	0.0	0.00051760	0.00495438	A_07_P018484	b1686	cmr|orf, hypothetical protein [b1686]
ydiJ	+2.89	9.50	+1.53	8.73	0.3	10.27	0.1	0.00209469	0.01366715	A_07_P018489	b1687	cmr|putative oxidase [b1687]
ydiU	-2.38	11.27	-1.25	11.89	0.2	10.64	0.1	0.00154506	0.01094754	A_07_P018581	b1706	cmr|orf, hypothetical protein [b1706]
ydiV	-4.54	8.95	-2.18	10.04	0.1	7.86	0.1	0.00003861	0.00088510	A_07_P018587	b1707	cmr|orf, hypothetical protein [b1707]
ydjA	+7.70	10.78	+2.95	9.31	0.2	12.25	0.1	0.00003187	0.00079613	A_07_P044447	Z2798	cmr|orf, hypothetical protein [Z2798]
ydjA	+16.98	10.44	+4.09	8.40	0.1	12.49	0.1	0.00000162	0.00023544	A_07_P035242	c_2168	cmr|Protein ydjA [c_2168]
ydjA	+15.38	10.26	+3.94	8.29	0.1	12.23	0.1	0.00000222	0.00026540	A_07_P035242	c_2168	cmr|Protein ydjA [c_2168]
ydjA	+14.95	10.20	+3.90	8.25	0.1	12.15	0.1	0.00000223	0.00026540	A_07_P035242	c_2168	cmr|Protein ydjA [c_2168]
ydjA	+15.32	10.28	+3.94	8.31	0.1	12.25	0.0	0.00000258	0.00027706	A_07_P035242	c_2168	cmr|Protein ydjA [c_2168]
ydjA	+12.89	10.36	+3.69	8.51	0.2	12.20	0.0	0.00000585	0.00036511	A_07_P018872	b1765	cmr|orf, hypothetical protein [b1765]
ydjA	+14.49	10.55	+3.86	8.62	0.1	12.47	0.1	0.00000230	0.00026836	A_07_P035242	c_2168	cmr|Protein ydjA [c_2168]
ydjE	-3.90	8.18	-1.96	9.16	0.1	7.19	0.5	0.00262618	0.01620104	A_07_P041186	c_2173	cmr|Hypothetical metabolite transport protein ydjE [c_2173]
ydjE	-2.58	8.12	-1.37	8.81	0.0	7.44	0.0	0.00026679	0.00310509	A_07_P018893	b1769	cmr|putative transport protein [b1769]
ydjF	-3.91	10.03	-1.97	11.01	0.1	9.04	0.2	0.00016028	0.00218551	A_07_P018898	b1770	cmr|putative DEOR-type transcriptional regulator [b1770]
ydjM	-2.87	8.64	-1.52	9.40	0.1	7.88	0.0	0.00018693	0.00241824	A_07_P018690	b1728	cmr|orf, hypothetical protein [b1728]
ydjN	-4.43	10.82	-2.15	11.90	0.0	9.75	0.0	0.00002852	0.00074846	A_07_P018691	b1729	cmr|part of a kinase [b1729]
ydjN	-3.63	9.83	-1.86	10.76	0.0	8.90	0.1	0.00010997	0.00167356	A_07_P042351	c_2128	cmr|Hypothetical symporter ydjN [c_2128]
yeaC	+3.68	12.38	+1.88	11.44	0.1	13.32	0.1	0.00009143	0.00148396	A_07_P015578	b1777	cmr|orf, hypothetical protein [b1777]
yeaD	+4.37	9.22	+2.13	8.16	0.2	10.28	0.1	0.00012713	0.00183667	A_07_P035263	c_2185	cmr|Unknown protein from 2D-page [c_2185]
yeaD	+4.31	9.31	+2.11	8.26	0.3	10.36	0.1	0.00026229	0.00308398	A_07_P035263	c_2185	cmr|Unknown protein from 2D-page [c_2185]
yeaD	+3.88	9.54	+1.96	8.56	0.1	10.52	0.2	0.00019093	0.00245787	A_07_P035263	c_2185	cmr|Unknown protein from 2D-page [c_2185]
yeaD	+3.99	9.31	+2.00	8.31	0.0	10.31	0.0	0.00004279	0.00093353	A_07_P035263	c_2185	cmr|Unknown protein from 2D-page [c_2185]
yeaD	+3.52	8.66	+1.82	7.75	0.0	9.57	0.1	0.00007461	0.00129798	A_07_P015593	b1780	cmr|orf, hypothetical protein [b1780]
yeaD	+4.05	9.55	+2.02	8.54	0.0	10.56	0.1	0.00005425	0.00107304	A_07_P035263	c_2185	cmr|Unknown protein from 2D-page [c_2185]
yeaJ	-6.46	8.85	-2.69	10.20	0.1	7.51	0.0	0.00001290	0.00051944	A_07_P015623	b1786	cmr|orf, hypothetical protein [b1786]
'yeaJ	-2.18	9.65	-1.12	10.21	0.0	9.08	0.0	0.00066768	0.00592222	A_07_P044476	Z2826	cmr|orf Unknown function [Z2826]
yeaL	-2.42	9.33	-1.28	9.97	0.2	8.69	0.2	0.00168570	0.01164021	A_07_P015638	b1789	cmr|orf, hypothetical protein [b1789]
yeaO	+2.26	9.46	+1.18	8.87	0.1	10.05	0.1	0.00128387	0.00953902	A_07_P015655	b1792	cmr|orf, hypothetical protein [b1792]
yeaT	-3.12	7.68	-1.64	8.50	0.0	6.86	0.0	0.00010326	0.00160656	A_07_P015689	b1799	cmr|putative transcriptional regulator LYSR-type [b1799]
yeaY	-2.04	10.12	-1.03	10.63	0.1	9.60	0.1	0.00249073	0.01556729	A_07_P015721	b1806	cmr|putative outer membrane protein [b1806]
yebB	-2.20	11.10	-1.14	11.67	0.3	10.53	0.2	0.00948813	0.04290094	A_07_P035344	c_2276	cmr|Hypothetical protein yebB [c_2276]
yebE	-8.25	11.11	-3.04	12.63	0.0	9.58	0.0	0.00000523	0.00034781	A_07_P015906	b1846	cmr|orf, hypothetical protein [b1846]
yebF	-4.28	15.38	-2.10	16.43	0.0	14.34	0.0	0.00003087	0.00078408	A_07_P015914	b1847	cmr|orf, hypothetical protein [b1847]
yebG	-2.02	15.52	-1.01	16.03	0.0	15.02	0.0	0.00112270	0.00866463	A_07_P015916	b1848	cmr|orf, hypothetical protein [b1848]
yebK	+3.66	8.52	+1.87	7.58	0.1	9.46	0.2	0.00013690	0.00194533	A_07_P015941	b1853	cmr|orf, hypothetical protein [b1853]
yebN	-2.80	10.98	-1.49	11.72	0.1	10.23	0.0	0.00020675	0.00260608	A_07_P015797	b1821	cmr|orf, hypothetical protein [b1821]
yebO	-6.31	9.99	-2.66	11.32	0.0	8.66	0.1	0.00001480	0.00054604	A_07_P015812	b1825	cmr|orf, hypothetical protein [b1825]
yebR	+2.53	8.68	+1.34	8.01	0.1	9.35	0.2	0.00161855	0.01131048	A_07_P015837	b1832	cmr|orf, hypothetical protein [b1832]
yebW	-2.43	9.32	-1.28	9.96	0.1	8.68	0.3	0.00249226	0.01557068	A_07_P010475	b1837	cmr|orf, hypothetical protein [b1837]
yebY	+3.64	10.31	+1.87	9.38	0.0	11.24	0.0	0.00006165	0.00115828	A_07_P015874	b1839	cmr|orf, hypothetical protein [b1839]
yecC	-4.48	8.42	-2.16	9.50	0.1	7.34	0.0	0.00005596	0.00109042	A_07_P014947	b1917	cmr|putative ATP-binding component of a transport system [b1917]
yecH	+2.07	7.52	+1.05	7.00	0.2	8.04	0.0	0.00432253	0.02350739	A_07_P052586	Z2962	cmr|orf, hypothetical protein [Z2962]
yecH	+2.27	7.93	+1.18	7.34	0.3	8.52	0.0	0.00474457	0.02527011	A_07_P035364	c_2322	cmr|Hypothetical protein yecH [c_2322]
yecI	+3.67	10.47	+1.88	9.53	0.0	11.40	0.0	0.00006057	0.00114898	A_07_P035358	c_2315	cmr|Ferritin-like protein 2 [c_2315]
yecN	+2.11	8.81	+1.08	8.27	0.0	9.35	0.1	0.00106294	0.00829283	A_07_P016023	b1869	cmr|orf, hypothetical protein [b1869]
yecR	-3.71	7.96	-1.89	8.91	0.0	7.02	0.1	0.00006228	0.00116220	A_07_P016190	b1904	cmr|orf, hypothetical protein [b1904]
yecS	-5.67	9.08	-2.50	10.33	0.1	7.83	0.1	0.00003217	0.00079793	A_07_P014955	b1918	cmr|putative transport system permease protein [b1918]
yecS	-4.94	9.33	-2.31	10.48	0.0	8.18	0.2	0.00006188	0.00115851	A_07_P042434	c_2332	cmr|Hypothetical amino-acid ABC transporter permease protein yecS [c_2332]
yedE	+4.49	10.10	+2.17	9.01	0.0	11.18	0.0	0.00003083	0.00078408	A_07_P015006	b1929	cmr|putative transport system permease protein [b1929]
yedE	+5.23	9.80	+2.39	8.60	0.1	10.99	0.0	0.00002724	0.00073404	A_07_P052596	Z3019	cmr|putative transport system permease protein [Z3019]
yedE	+12.68	10.64	+3.66	8.81	0.1	12.47	0.0	0.00000276	0.00028062	A_07_P035376	c_2344	cmr|Hypothetical protein yedE [c_2344]
yedF	+11.11	10.15	+3.47	8.41	0.2	11.89	0.0	0.00001468	0.00054604	A_07_P015012	b1930	cmr|orf, hypothetical protein [b1930]
yedJ	-2.33	8.24	-1.22	8.85	0.0	7.63	0.1	0.00047645	0.00466781	A_07_P052607	Z3055	cmr|orf, hypothetical protein [Z3055]
yedJ	-4.63	10.21	-2.21	11.31	0.0	9.10	0.1	0.00002770	0.00073912	A_07_P041216	c_2381	cmr|Hypothetical protein yedJ [c_2381]
yedJ	-4.47	9.13	-2.16	10.21	0.0	8.05	0.1	0.00004172	0.00093045	A_07_P015168	b1962	cmr|orf, hypothetical protein [b1962]
yedR	-2.88	8.25	-1.53	9.01	0.1	7.49	0.0	0.00023553	0.00285318	A_07_P015174	b1963	cmr|orf, hypothetical protein [b1963]
yedW	-2.16	8.28	-1.11	8.84	0.1	7.73	0.1	0.00134803	0.00984389	A_07_P015203	b1969	cmr|putative 2-component transcriptional regulator [b1969]
yeeA	+3.19	8.08	+1.67	7.24	0.1	8.91	0.0	0.00018218	0.00238186	A_07_P031967	c_2536	cmr|Hypothetical protein yeeA [c_2536]
yeeO	-6.51	8.39	-2.70	9.75	0.0	7.04	0.0	0.00000906	0.00043556	A_07_P015266	b1985	cmr|orf, hypothetical protein [b1985]
yeeY	-2.28	9.83	-1.19	10.42	0.1	9.23	0.0	0.00052690	0.00502151	A_07_P015404	b2015	cmr|putative transcriptional regulator LYSR-type [b2015]
yeeZ	-2.71	12.36	-1.44	13.08	0.0	11.64	0.1	0.00027722	0.00319517	A_07_P015407	b2016	cmr|putative enzyme of sugar metabolism [b2016]
yegB	-2.00	8.06	-1.00	8.55	0.2	7.56	0.1	0.00625655	0.03120151	A_07_P042496	c_2603	cmr|Hypothetical transport protein yegB [c_2603]
yegE	-2.24	9.30	-1.16	9.88	0.1	8.72	0.3	0.00679630	0.03324044	A_07_P003112	b2067	cmr|putative sensor-type protein [b2067]
yegI	-2.11	10.11	-1.08	10.64	0.1	9.57	0.1	0.00126200	0.00941208	A_07_P003126	b2070	cmr|putative chaperonin [b2070]
yegL	-2.10	9.91	-1.07	10.45	0.1	9.38	0.0	0.00122159	0.00918208	A_07_P032081	c_2599	cmr|Hypothetical protein yegL [c_2599]
yegP	+2.16	7.87	+1.11	7.32	0.2	8.43	0.1	0.00214829	0.01391304	A_07_P032098	c_2606	cmr|Hypothetical protein yegP [c_2606]
yegQ	-5.05	10.49	-2.34	11.66	0.1	9.33	0.1	0.00002639	0.00072866	A_07_P003182	b2081	cmr|orf, hypothetical protein [b2081]
yegS	-2.17	9.57	-1.12	10.13	0.2	9.01	0.2	0.00391403	0.02191410	A_07_P032127	c_2614	cmr|Hypothetical protein yegS [c_2614]
yegW	-2.06	8.67	-1.04	9.19	0.1	8.15	0.0	0.00185675	0.01248726	A_07_P003270	b2101	cmr|putative transcriptional regulator [b2101]
yegX	-2.37	11.38	-1.24	12.00	0.1	10.75	0.1	0.00095922	0.00769519	A_07_P003271	b2102	cmr|orf, hypothetical protein [b2102]
yegX	-3.66	8.41	-1.87	9.34	0.1	7.47	0.0	0.00008650	0.00143544	A_07_P051783	Z3266	cmr|orf, hypothetical protein [Z3266]
yehD	+5.15	7.79	+2.36	6.60	0.0	8.97	0.1	0.00002448	0.00070547	A_07_P003318	b2111	cmr|putative fimbrial-like protein [b2111]
yehL	-3.25	8.25	-1.70	9.10	0.0	7.40	0.3	0.00066265	0.00588091	A_07_P003356	b2119	cmr|orf, hypothetical protein [b2119]
yehL	-5.58	9.08	-2.48	10.32	0.1	7.84	0.2	0.00004832	0.00099713	A_07_P041221	c_2649	cmr|Hypothetical protein yehL [c_2649]
yehS	-3.39	9.08	-1.76	9.96	0.0	8.20	0.0	0.00007811	0.00133518	A_07_P003385	b2124	cmr|orf, hypothetical protein [b2124]
yehX	-2.21	7.66	-1.14	8.24	0.0	7.09	0.0	0.00062542	0.00564928	A_07_P032191	c_2659	cmr|Hypothetical ABC transporter ATP-binding protein yehX [c_2659]
yeiC	+5.04	7.76	+2.33	6.60	0.3	8.93	0.1	0.00012722	0.00183667	A_07_P032264	c_2701	cmr|Hypothetical sugar kinase yeiC [c_2701]
yeiP	+4.81	9.10	+2.27	7.97	0.0	10.23	0.0	0.00002412	0.00069739	A_07_P000651	b2171	cmr|putative elongation factor [b2171]
yeiQ	+7.46	9.29	+2.90	7.84	0.0	10.74	0.1	0.00000862	0.00042621	A_07_P000660	b2172	cmr|putative oxidoreductase [b2172]
yeiT	+2.67	8.67	+1.42	7.96	0.2	9.38	0.2	0.00159737	0.01119846	A_07_P003491	b2146	cmr|putative oxidoreductase [b2146]
yejG	-4.70	12.45	-2.23	13.57	0.0	11.34	0.0	0.00002296	0.00067941	A_07_P000703	b2181	cmr|orf, hypothetical protein [b2181]
yejK	+4.18	10.28	+2.06	9.25	0.2	11.31	0.0	0.00018583	0.00240997	A_07_P000729	b2186	cmr|orf, hypothetical protein [b2186]
yejL	-2.12	12.09	-1.08	12.63	0.1	11.55	0.1	0.00119733	0.00905414	A_07_P000733	b2187	cmr|orf, hypothetical protein [b2187]
yejM	-2.28	8.55	-1.19	9.15	0.2	7.96	0.1	0.00240665	0.01516218	A_07_P000736	b2188	cmr|putative sulfatase [b2188]
yfaE	+14.19	9.86	+3.83	7.95	0.2	11.77	0.0	0.00000713	0.00039666	A_07_P000966	b2236	cmr|orf, hypothetical protein [b2236]
yfbB	+2.62	8.53	+1.39	7.83	0.0	9.22	0.1	0.00037734	0.00396055	A_07_P001104	b2263	cmr|putative enzyme [b2263]
yfbR	+2.14	7.67	+1.10	7.12	0.1	8.22	0.1	0.00147928	0.01057665	A_07_P013744	b2291	cmr|putative alpha helix protein [b2291]
yfbU	+2.00	9.98	+1.00	9.48	0.0	10.48	0.1	0.00132700	0.00973544	A_07_P013758	b2294	cmr|orf, hypothetical protein [b2294]
yfcE	+2.22	11.93	+1.15	11.35	0.1	12.50	0.0	0.00094946	0.00764622	A_07_P013789	b2300	cmr|orf, hypothetical protein [b2300]
yfcF	+2.13	8.20	+1.09	7.65	0.1	8.74	0.0	0.00095117	0.00765605	A_07_P013791	b2301	cmr|orf, hypothetical protein [b2301]
yfdE	-4.18	9.36	-2.06	10.39	0.2	8.33	0.1	0.00018448	0.00239640	A_07_P014140	b2371	cmr|putative enzyme [b2371]
yfdZ	+2.96	8.60	+1.57	7.82	0.0	9.39	0.1	0.00015009	0.00208564	A_07_P014179	b2379	cmr|putative aminotransferase [b2379]
yfeA	-2.65	8.52	-1.41	9.23	0.1	7.82	0.1	0.00047961	0.00468366	A_07_P014259	b2395	cmr|orf, hypothetical protein [b2395]
yfeN	-2.08	11.11	-1.06	11.63	0.2	10.58	0.2	0.00848145	0.03951816	A_07_P013096	b2408	cmr|putative sugar hydrolase [b2408]
yffB	-3.27	10.95	-1.71	11.81	0.0	10.10	0.1	0.00010241	0.00160236	A_07_P013411	b2471	cmr|orf, hypothetical protein [b2471]
yffL	-2.05	9.57	-1.03	10.09	0.2	9.05	0.0	0.00281837	0.01706630	A_07_P013272	b2443	cmr|orf, hypothetical protein [b2443]
yfgC	+2.14	8.50	+1.10	7.95	0.0	9.05	0.1	0.00083660	0.00702103	A_07_P013530	b2494	cmr|orf, hypothetical protein [b2494]
yfgC	+2.36	9.54	+1.24	8.92	0.2	10.16	0.1	0.00192672	0.01280417	A_07_P042608	c_3011	cmr|Hypothetical protein yfgC precursor [c_3011]
yfgD	+3.93	8.41	+1.98	7.42	0.1	9.40	0.0	0.00007451	0.00129774	A_07_P013532	b2495	cmr|putative oxidoreductase [b2495]
yfgG	+2.61	10.33	+1.38	9.63	0.1	11.02	0.1	0.00059925	0.00549159	A_07_P013578	b2504	cmr|orf, hypothetical protein [b2504]
yfgI	-2.10	10.75	-1.07	11.28	0.2	10.21	0.1	0.00230337	0.01467505	A_07_P030147	c_3025	cmr|Hypothetical protein yfgI [c_3025]
yfgM	+3.64	10.91	+1.86	9.98	0.0	11.84	0.0	0.00005736	0.00110534	A_07_P013623	b2513	cmr|orf, hypothetical protein [b2513]
yfhB	-6.78	9.92	-2.76	11.30	0.1	8.54	0.1	0.00002268	0.00067364	A_07_P019703	b2560	cmr|orf, hypothetical protein [b2560]
yfiC	+2.93	8.66	+1.55	7.88	0.2	9.43	0.0	0.00047218	0.00464624	A_07_P019777	b2575	cmr|putative enzyme [b2575]
yfiD	+5.72	11.46	+2.52	10.20	0.1	12.72	0.0	0.00001737	0.00058395	A_07_P019799	b2579	cmr|putative formate acetyltransferase [b2579]
yfiF	+7.54	11.02	+2.91	9.56	0.1	12.48	0.0	0.00000864	0.00042621	A_07_P019806	b2581	cmr|orf, hypothetical protein [b2581]
yfiQ	+3.57	9.09	+1.84	8.17	0.0	10.01	0.1	0.00007396	0.00129530	A_07_P019821	b2584	cmr|orf, hypothetical protein [b2584]
ygaA	-3.86	9.07	-1.95	10.04	0.1	8.09	0.0	0.00005897	0.00112656	A_07_P030601	c_3263	cmr|Hypothetical sigma-54-dependent transcriptional regulator ygaA [c_3263]
ygaD	+7.43	10.32	+2.89	8.88	0.0	11.77	0.1	0.00000817	0.00041270	A_07_P001946	b2700	cmr|orf, hypothetical protein [b2700]
ygaF	-3.30	8.38	-1.72	9.24	0.6	7.52	0.1	0.00900240	0.04138212	A_07_P020152	b2660	cmr|orf, hypothetical protein [b2660]
ygaF	-2.24	9.00	-1.17	9.58	0.1	8.42	0.1	0.00083875	0.00702801	A_07_P030517	c_3208	cmr|Hypothetical protein ygaF [c_3208]
ygaT	-3.03	8.34	-1.60	9.14	0.0	7.54	0.0	0.00011835	0.00175449	A_07_P041636	c_3207	cmr|Hypothetical protein ygaT [c_3207]
ygaV	+2.41	8.51	+1.27	7.88	0.2	9.15	0.2	0.00197548	0.01305157	A_07_P020189	b2667	cmr|orf, hypothetical protein [b2667]
ygaW	-6.03	11.68	-2.59	12.97	0.1	10.38	0.0	0.00001351	0.00052666	A_07_P001825	b2670	cmr|orf, hypothetical protein [b2670]
ygaZ	-2.24	9.92	-1.16	10.50	0.2	9.34	0.1	0.00203909	0.01333202	A_07_P001881	b2682	cmr|orf, hypothetical protein [b2682]
ygbE	-2.51	8.74	-1.33	9.40	0.0	8.07	0.0	0.00029639	0.00335277	A_07_P030701	c_3316	cmr|Hypothetical protein ygbE [c_3316]
ygbJ	+2.42	8.51	+1.27	7.87	0.1	9.14	0.1	0.00062520	0.00564928	A_07_P030675	c_3297	cmr|Hypothetical oxidoreductase ygbJ [c_3297]
ygcG	-4.27	9.34	-2.09	10.39	0.1	8.30	0.0	0.00004234	0.00093353	A_07_P032628	c_3342	cmr|Hypothetical protein ygcG [c_3342]
ygcN	-2.10	9.27	-1.07	9.81	0.0	8.74	0.2	0.00184697	0.01244116	A_07_P002278	b2766	cmr|orf, hypothetical protein [b2766]
ygdE	-2.03	9.07	-1.02	9.58	0.1	8.56	0.1	0.00153430	0.01089259	A_07_P006026	b2806	cmr|orf, hypothetical protein [b2806]
ygdH	+2.22	9.43	+1.15	8.85	0.1	10.00	0.2	0.00362027	0.02069114	A_07_P002424	b2795	cmr|orf, hypothetical protein [b2795]
ygdL	-2.27	10.91	-1.18	11.50	0.1	10.32	0.0	0.00057437	0.00533670	A_07_P032656	c_3383	cmr|Hypothetical protein ygdL [c_3383]
ygdQ	-2.64	8.17	-1.40	8.87	0.1	7.47	0.0	0.00026461	0.00309464	A_07_P006141	b2832	cmr|putative transport protein [b2832]
ygeK	-2.40	11.27	-1.26	11.90	0.2	10.64	0.1	0.00219770	0.01416318	A_07_P006257	b2855	cmr|putative 2-component transcriptional regulator [b2855]
ygeR	-13.56	9.89	-3.76	11.77	0.0	8.01	0.1	0.00000368	0.00030737	A_07_P006295	b2865	cmr|putative lipoprotein [b2865]
ygfA	-3.00	9.43	-1.58	10.23	0.1	8.64	0.3	0.00125004	0.00933173	A_07_P006525	b2912	cmr|putative ligase [b2912]
ygfX	-2.57	11.35	-1.36	12.04	0.0	10.67	0.0	0.00026355	0.00309415	A_07_P006446	b2896	cmr|orf, hypothetical protein [b2896]
yggD	-2.19	8.58	-1.13	9.15	0.0	8.02	0.0	0.00063391	0.00570950	A_07_P011793	b2929	cmr|putative transcriptional regulator [b2929]
yggD	-2.86	9.77	-1.52	10.53	0.0	9.01	0.1	0.00018124	0.00237586	A_07_P032921	c_3512	cmr|Hypothetical protein yggD [c_3512]
yggL	+3.82	13.00	+1.93	12.03	0.0	13.96	0.0	0.00004543	0.00095882	A_07_P011938	b2959	cmr|orf, hypothetical protein [b2959]
yggP	-2.40	8.27	-1.26	8.90	0.1	7.64	0.0	0.00045704	0.00454842	A_07_P011806	b2932	cmr|orf, hypothetical protein [b2932]
yggX	+3.15	10.78	+1.66	9.96	0.1	11.61	0.0	0.00011012	0.00167356	A_07_P011954	b2962	cmr|orf, hypothetical protein [b2962]
yghB	-7.24	11.27	-2.86	12.70	0.0	9.84	0.1	0.00000823	0.00041394	A_07_P012180	b3009	cmr|orf, hypothetical protein [b3009]
yghU	+2.33	8.06	+1.22	7.45	0.1	8.67	0.0	0.00056473	0.00527744	A_07_P012078	b2989	cmr|orf, hypothetical protein [b2989]
yghU	+3.37	8.84	+1.75	7.96	0.1	9.71	0.1	0.00009358	0.00150183	A_07_P041281	c_3726	cmr|Hypothetical GST-like protein yghU [c_3726]
yghZ	+2.83	9.51	+1.50	8.76	0.1	10.26	0.0	0.00021709	0.00269339	A_07_P012136	b3001	cmr|putative reductase [b3001]
ygiB	+2.70	11.93	+1.43	11.21	0.2	12.64	0.1	0.00050832	0.00488792	A_07_P012316	b3037	cmr|orf, hypothetical protein [b3037]
ygiC	+2.43	8.43	+1.28	7.79	0.3	9.07	0.0	0.00192703	0.01280417	A_07_P012323	b3038	cmr|putative synthetase/amidase [b3038]
ygiF	-3.99	11.43	-2.00	12.43	0.0	10.44	0.1	0.00006376	0.00117677	A_07_P000003	b3054	cmr|orf, hypothetical protein [b3054]
ygiF	-3.11	12.72	-1.64	13.54	0.0	11.90	0.0	0.00011300	0.00169761	A_07_P041286	c_3802	cmr|Hypothetical protein ygiF [c_3802]
ygiN	+13.33	10.59	+3.74	8.72	0.1	12.46	0.1	0.00000369	0.00030737	A_07_P012278	b3029	cmr|orf, hypothetical protein [b3029]
ygiP	-4.75	9.74	-2.25	10.86	0.0	8.61	0.1	0.00004191	0.00093286	A_07_P036891	c_3810	cmr|Hypothetical transcriptional regulator ygiP [c_3810]
ygjH	-2.55	12.52	-1.35	13.19	0.0	11.84	0.4	0.00572323	0.02927440	A_07_P000096	b3074	cmr|putative tRNA synthetase [b3074]
ygjO	+2.90	8.66	+1.54	7.89	0.4	9.43	0.1	0.00286514	0.01730292	A_07_P045877	Z4437	cmr|putative enzyme [Z4437]
ygjP	-5.28	9.27	-2.40	10.47	0.1	8.07	0.0	0.00002371	0.00068742	A_07_P000154	b3085	cmr|orf, hypothetical protein [b3085]
ygjP	-7.13	10.92	-2.83	12.34	0.0	9.50	0.1	0.00001063	0.00047201	A_07_P034044	c_3843	cmr|Hypothetical protein ygjP [c_3843]
ygjQ	-3.09	8.15	-1.63	8.96	0.0	7.33	0.0	0.00010538	0.00163463	A_07_P041292	c_3844	cmr|Hypothetical protein ygjQ [c_3844]
ygjR	+3.56	8.97	+1.83	8.06	0.1	9.89	0.0	0.00007139	0.00125864	A_07_P000165	b3087	cmr|orf, hypothetical protein [b3087]
ygjU	+3.12	9.26	+1.64	8.44	0.1	10.08	0.1	0.00029556	0.00334773	A_07_P034047	c_3847	cmr|Hypothetical symporter ygjU [c_3847]
yhaG	+2.22	9.35	+1.15	8.78	0.2	9.93	0.1	0.00189443	0.01269186	A_07_P034086	c_3883	cmr|D-galactarate dehydratase [c_3883]
yhaG	+4.20	9.45	+2.07	8.41	0.2	10.48	0.1	0.00014115	0.00199309	A_07_P045896	Z4480	cmr|putative hydrolase [Z4480]
yhaJ	+2.47	9.72	+1.31	9.06	0.0	10.37	0.0	0.00033044	0.00362039	A_07_P000254	b3105	cmr|putative transcriptional regulator LYSR-type [b3105]
yhaL	+2.88	10.72	+1.53	9.95	0.4	11.48	0.0	0.00345649	0.01995830	A_07_P000264	b3107	cmr|orf, hypothetical protein [b3107]
yhbU	+2.47	10.87	+1.31	10.22	0.2	11.52	0.0	0.00092482	0.00753640	A_07_P000511	b3158	cmr|putative collagenase [b3158]
yhbV	+2.02	9.32	+1.02	8.81	0.1	9.83	0.1	0.00159836	0.01119918	A_07_P000516	b3159	cmr|orf, hypothetical protein [b3159]
yhcB	+5.32	12.80	+2.41	11.60	0.0	14.01	0.1	0.00001863	0.00061218	A_07_P012640	b3233	cmr|orf, hypothetical protein [b3233]
yhcH	+3.92	8.04	+1.97	7.06	0.0	9.03	0.1	0.00005802	0.00111391	A_07_P034165	c_3975	cmr|Hypothetical protein yhcH [c_3975]
yhcH	+3.16	8.14	+1.66	7.31	0.3	8.97	0.1	0.00063371	0.00570950	A_07_P045957	Z4579	cmr|orf, hypothetical protein [Z4579]
yhcI	+3.06	7.82	+1.61	7.02	0.2	8.63	0.1	0.00085527	0.00712456	A_07_P051815	Z4580	cmr|putative NAGC-like transcriptional regulator [Z4580]
yhcJ	+5.35	8.59	+2.42	7.38	0.2	9.80	0.1	0.00007107	0.00125539	A_07_P041791	c_3977	cmr|Hypothetical protein yhcJ [c_3977]
yhcJ	+4.94	8.37	+2.31	7.22	0.1	9.53	0.2	0.00005190	0.00104364	A_07_P041304	c_3977	cmr|Hypothetical protein yhcJ [c_3977]
yhcK	-2.07	9.70	-1.05	10.22	0.0	9.17	0.0	0.00094158	0.00760999	A_07_P034168	c_3980	cmr|Hypothetical transcriptional regulator yhcK [c_3980]
yhcO	-2.26	8.25	-1.18	8.84	0.1	7.66	0.1	0.00107398	0.00835826	A_07_P012666	b3239	cmr|orf, hypothetical protein [b3239]
yhcR	-2.90	7.64	-1.54	8.41	0.1	6.87	0.2	0.00053221	0.00505894	A_07_P035544	c_3997	cmr|Hypothetical protein yhcR [c_3997]
yhcR	-3.73	8.32	-1.90	9.27	0.0	7.38	0.1	0.00006897	0.00123392	A_07_P048607	Z4601	cmr|orf, hypothetical protein [Z4601]
yhdE	+2.45	9.14	+1.29	8.49	0.2	9.79	0.1	0.00131411	0.00970876	A_07_P012715	b3248	cmr|orf, hypothetical protein [b3248]
yhdH	+7.24	9.96	+2.86	8.53	0.3	11.39	0.2	0.00007430	0.00129689	A_07_P034191	c_4009	cmr|Protein yhdH [c_4009]
yhdH	+4.97	8.61	+2.31	7.45	0.2	9.76	0.1	0.00008593	0.00143251	A_07_P012736	b3253	cmr|putative dehydrogenase [b3253]
yhdZ	+3.51	8.30	+1.81	7.39	0.1	9.20	0.1	0.00015036	0.00208675	A_07_P012823	b3271	cmr|putative ATP-binding component of a transport system [b3271]
yheF	-3.87	8.07	-1.95	9.04	0.1	7.09	0.1	0.00008903	0.00145399	A_07_P034326	c_4096	cmr|Probable general secretion pathway protein D precursor [c_4096]
yheO	-2.15	10.52	-1.11	11.08	0.0	9.97	0.0	0.00071141	0.00619497	A_07_P017760	b3346	cmr|orf, hypothetical protein [b3346]
yheS	-2.86	11.02	-1.52	11.78	0.0	10.27	0.0	0.00016158	0.00219117	A_07_P017786	b3352	cmr|putative ATP-binding component of a transport system [b3352]
yhfC	-3.95	8.50	-1.98	9.49	0.0	7.51	0.2	0.00020908	0.00262336	A_07_P041829	c_4139	cmr|Hypothetical protein yhfC [c_4139]
yhfS	-2.05	11.10	-1.03	11.61	0.0	10.58	0.0	0.00095526	0.00768114	A_07_P017907	b3376	cmr|orf, hypothetical protein [b3376]
yhfS	-2.53	8.28	-1.34	8.95	0.2	7.61	0.1	0.00149684	0.01068764	A_07_P034375	c_4146	cmr|Hypothetical protein yhfS [c_4146]
yhfU	-2.18	8.98	-1.13	9.54	0.2	8.42	0.0	0.00274830	0.01674509	A_07_P017919	b3378	cmr|orf, hypothetical protein [b3378]
yhfZ	+2.22	8.21	+1.15	7.63	0.0	8.78	0.2	0.00147284	0.01053537	A_07_P017944	b3383	cmr|orf, hypothetical protein [b3383]
yhgF	+2.06	10.02	+1.04	9.50	0.0	10.54	0.0	0.00098488	0.00783521	A_07_P018065	b3407	cmr|orf, hypothetical protein [b3407]
yhgH	+3.18	11.93	+1.67	11.09	0.0	12.76	0.0	0.00009961	0.00156674	A_07_P041315	c_4190	cmr|Hypothetical protein yhgH [c_4190]
yhhM	+2.24	7.61	+1.16	7.03	0.2	8.19	0.1	0.00231987	0.01473934	A_07_P007271	b3467	cmr|putative receptor [b3467]
yhhM	+2.04	7.72	+1.03	7.20	0.3	8.23	0.1	0.00601140	0.03031503	A_07_P007271	b3467	cmr|putative receptor [b3467]
yhhM	+2.11	7.86	+1.08	7.32	0.0	8.40	0.1	0.00139740	0.01009668	A_07_P007271	b3467	cmr|putative receptor [b3467]
yhhS	-2.22	8.33	-1.15	8.90	0.1	7.75	0.0	0.00102654	0.00807690	A_07_P007303	b3473	cmr|putative transport [b3473]
yhhW	+2.29	9.00	+1.20	8.40	0.0	9.60	0.0	0.00046068	0.00457021	A_07_P018221	b3439	cmr|orf, hypothetical protein [b3439]
yhiD	-3.37	14.27	-1.75	15.14	0.0	13.39	0.0	0.00008170	0.00138058	A_07_P007475	b3508	cmr|putative transport ATPase [b3508]
yhiN	-3.30	10.90	-1.72	11.76	0.1	10.04	0.1	0.00014561	0.00203595	A_07_P007396	b3492	cmr|orf, hypothetical protein [b3492]
yhiQ	-2.25	8.06	-1.17	8.65	0.1	7.47	0.1	0.00079704	0.00676475	A_07_P007422	b3497	cmr|orf, hypothetical protein [b3497]
yhiR	-3.89	12.59	-1.96	13.57	0.0	11.61	0.0	0.00004921	0.00100666	A_07_P007435	b3499	cmr|orf, hypothetical protein [b3499]
yhiW	-2.32	8.78	-1.21	9.39	0.0	8.17	0.1	0.00097613	0.00778927	A_07_P039549	c_4326	cmr|Hypothetical transcriptional regulator yhiW [c_4326]
yhjJ	-2.70	9.22	-1.43	9.93	0.0	8.50	0.0	0.00020640	0.00260387	A_07_P049426	Z4941	cmr|orf, hypothetical protein [Z4941]
yhjJ	-2.52	9.99	-1.33	10.65	0.0	9.32	0.1	0.00047572	0.00466588	A_07_P039556	c_4339	cmr|Protein yhjJ precursor [c_4339]
yhjQ	-4.30	8.80	-2.10	9.85	0.1	7.74	0.2	0.00017446	0.00230819	A_07_P007604	b3534	cmr|orf, hypothetical protein [b3534]
yhjR	-2.71	8.66	-1.44	9.38	0.0	7.94	0.2	0.00065454	0.00584189	A_07_P007606	b3535	cmr|orf, hypothetical protein [b3535]
yiaC	-2.17	10.24	-1.12	10.80	0.1	9.68	0.2	0.00216106	0.01398425	A_07_P007676	b3550	cmr|orf, hypothetical protein [b3550]
yiaC	-2.30	9.43	-1.20	10.03	0.1	8.83	0.0	0.00097039	0.00775788	A_07_P039621	c_4368	cmr|Hypothetical acetyltransferase yiaC [c_4368]

yiaF	+2.17	10.20	+1.12	9.65	0.0	10.76	0.0	0.00069576	0.00609231	A_07_P007698	b3554	cmr|orf, hypothetical protein [b3554]
yiaK	+2.78	8.43	+1.48	7.69	0.0	9.16	0.1	0.00028150	0.00323262	A_07_P007803	b3575	cmr|putative dehydrogenase [b3575]
yibF	+3.07	8.30	+1.62	7.49	0.0	9.11	0.1	0.00019354	0.00248131	A_07_P016905	b3592	cmr|putative S-transferase [b3592]
yibK	+2.85	9.60	+1.51	8.85	0.0	10.36	0.0	0.00015908	0.00217908	A_07_P016974	b3606	cmr|orf, hypothetical protein [b3606]
yidC	-2.50	12.63	-1.32	13.29	0.1	11.97	0.0	0.00039168	0.00405638	A_07_P005451	b3705	cmr|60 KD inner-membrane protein [b3705]
yidD	-2.73	13.42	-1.45	14.15	0.1	12.70	0.0	0.00021108	0.00263544	A_07_P021528	ABE-0285078	asap|predicted protein [b4557]
yidF	+2.73	8.79	+1.45	8.07	0.0	9.51	0.2	0.00072381	0.00627400	A_07_P017305	b3674	cmr|putative transcriptional regulator [b3674]
yidS	-3.59	8.11	-1.84	9.04	0.0	7.19	0.1	0.00010721	0.00164539	A_07_P039007	c_4611	cmr|Hypothetical protein yidS precursor [c_4611]
yidS	-4.17	7.71	-2.06	8.74	0.1	6.68	0.1	0.00006769	0.00122079	A_07_P051822	Z5186	cmr|orf, hypothetical protein [Z5186]
yieF	+2.00	11.21	+1.00	10.71	0.2	11.71	0.1	0.00313039	0.01852121	A_07_P005486	b3713	cmr|orf, hypothetical protein [b3713]
yieG	-2.01	8.45	-1.01	8.96	0.1	7.95	0.1	0.00226920	0.01451717	A_07_P005491	b3714	cmr|putative membrane / transport protein [b3714]
yieN	-2.39	10.48	-1.25	11.10	0.1	9.85	0.1	0.00068457	0.00602118	A_07_P041346	c_4674	cmr|Hypothetical protein yieN [c_4674]
yifE	+5.06	9.75	+2.34	8.58	0.2	10.92	0.0	0.00005218	0.00104650	A_07_P005711	b3764	cmr|orf, hypothetical protein [b3764]
yifK	+3.68	8.69	+1.88	7.75	0.2	9.63	0.0	0.00017860	0.00234911	A_07_P041930	c_4717	cmr|Probable transport protein yifK [c_4717]
yifL	+2.99	10.00	+1.58	9.20	0.0	10.79	0.0	0.00012138	0.00178598	A_07_P010621	ABE-0285079	asap|predicted lipoprotein [b4558]
yigI	-2.57	10.35	-1.36	11.04	0.1	9.67	0.1	0.00037688	0.00396055	A_07_P005970	b3820	cmr|orf, hypothetical protein [b3820]
yigM	-2.61	8.10	-1.38	8.79	0.1	7.41	0.1	0.00057750	0.00535466	A_07_P018941	b3827	cmr|orf, hypothetical protein [b3827]
yigP	-3.41	12.92	-1.77	13.81	0.0	12.04	0.0	0.00007244	0.00126998	A_07_P018976	b3834	cmr|orf, hypothetical protein [b3834]
yigZ	-2.07	10.56	-1.05	11.09	0.1	10.04	0.0	0.00176800	0.01210758	A_07_P041354	c_4795	cmr|Hypothetical protein yigZ [c_4795]
yihA	+4.13	9.60	+2.04	8.58	0.3	10.62	0.0	0.00046163	0.00457676	A_07_P047013	Z5400	cmr|orf, hypothetical protein [Z5400]
yihA	+3.13	10.33	+1.65	9.50	0.0	11.15	0.0	0.00010902	0.00166313	A_07_P019103	b3865	cmr|orf, hypothetical protein [b3865]
yihA	+4.69	9.94	+2.23	8.83	0.1	11.06	0.0	0.00004444	0.00094837	A_07_P039310	c_4812	cmr|Probable GTP-binding protein engB [c_4812]
yihD	+36.56	10.04	+5.19	7.44	0.3	12.63	0.0	0.00000408	0.00032474	A_07_P019075	b3858	cmr|orf, hypothetical protein [b3858]
yihI	+7.26	8.73	+2.86	7.30	0.1	10.16	0.2	0.00001919	0.00061714	A_07_P019108	b3866	cmr|orf, hypothetical protein [b3866]
yihX	+3.21	9.91	+1.68	9.06	0.1	10.75	0.1	0.00014145	0.00199367	A_07_P019205	b3885	cmr|putative phosphatase [b3885]
yiiD	+2.48	8.56	+1.31	7.91	0.2	9.22	0.1	0.00082862	0.00696146	A_07_P019216	b3888	cmr|putative acetyltransferase [b3888]
yiiD	+2.78	9.20	+1.48	8.46	0.3	9.93	0.1	0.00179341	0.01223375	A_07_P041956	c_4835	cmr|Hypothetical protein yiiD [c_4835]
yiiE	-10.17	8.93	-3.35	10.61	0.1	7.26	0.0	0.00000419	0.00032993	A_07_P019225	b3889	cmr|orf, hypothetical protein [b3889]
yiiQ	+3.55	8.39	+1.83	7.48	0.1	9.31	0.0	0.00009234	0.00148959	A_07_P019377	b3920	cmr|orf, hypothetical protein [b3920]
yiiS	+3.49	8.56	+1.80	7.65	0.1	9.46	0.1	0.00016625	0.00223475	A_07_P039426	c_4874	cmr|Hypothetical protein yiiS [c_4874]
yiiS	+3.87	8.08	+1.95	7.11	0.0	9.06	0.1	0.00005853	0.00112095	A_07_P019386	b3922	cmr|orf, hypothetical protein [b3922]
yiiS	+2.50	8.34	+1.32	7.68	0.3	9.00	0.1	0.00222932	0.01433372	A_07_P047106	Z5467	cmr|orf, hypothetical protein [Z5467]
yiiT	+2.64	9.62	+1.40	8.92	0.1	10.32	0.0	0.00032001	0.00353373	A_07_P047111	Z5468	cmr|putative regulator [Z5468]
yiiT	+2.21	9.54	+1.14	8.97	0.0	10.11	0.0	0.00059469	0.00547004	A_07_P039431	c_4875	cmr|Hypothetical protein yiiT [c_4875]
yiiU	+2.86	12.60	+1.51	11.84	0.1	13.35	0.0	0.00026306	0.00309077	A_07_P019420	b3928	cmr|orf, hypothetical protein [b3928]
yiiX	-2.76	9.64	-1.46	10.37	0.1	8.90	0.1	0.00028637	0.00327425	A_07_P052282	Z5492	cmr|orf, hypothetical protein [Z5492]
yiiX	-3.38	8.91	-1.76	9.79	0.0	8.03	0.1	0.00013007	0.00186501	A_07_P041984	c_4890	cmr|Hypothetical protein yiiX precursor [c_4890]
yiiX	-2.32	7.83	-1.21	8.44	0.0	7.22	0.2	0.00128861	0.00956524	A_07_P019465	b3937	cmr|orf, hypothetical protein [b3937]
yijF	-3.16	12.98	-1.66	13.81	0.2	12.15	0.4	0.00329507	0.01928715	A_07_P039476	c_4902	cmr|Hypothetical protein yijF precursor [c_4902]
yjaA	-3.81	10.09	-1.93	11.06	0.1	9.13	0.1	0.00012470	0.00181953	A_07_P008699	b4011	cmr|orf, hypothetical protein [b4011]
yjaB	-2.42	12.79	-1.27	13.43	0.1	12.15	0.2	0.00151648	0.01080827	A_07_P008704	b4012	cmr|orf, hypothetical protein [b4012]
yjaG	+4.98	10.37	+2.32	9.21	0.1	11.53	0.0	0.00002794	0.00073943	A_07_P008660	b3999	cmr|orf, hypothetical protein [b3999]
yjbG	-2.02	11.77	-1.01	12.28	0.1	11.26	0.1	0.00156677	0.01104171	A_07_P008783	b4028	cmr|orf, hypothetical protein [b4028]
yjbJ	-2.20	9.81	-1.14	10.38	0.0	9.24	0.1	0.00117309	0.00894178	A_07_P008870	b4045	cmr|orf, hypothetical protein [b4045]
yjbJ	-4.04	10.98	-2.02	11.99	0.1	9.97	0.0	0.00004241	0.00093353	A_07_P037555	c_5016	cmr|Protein yjbJ [c_5016]
yjbQ	+4.79	9.19	+2.26	8.06	0.2	10.32	0.0	0.00009557	0.00152759	A_07_P008923	b4056	cmr|orf, hypothetical protein [b4056]
yjcB	-13.30	9.80	-3.73	11.66	0.1	7.93	0.2	0.00001168	0.00050224	A_07_P008942	b4060	cmr|orf, hypothetical protein [b4060]
yjcB	-12.34	9.56	-3.63	11.37	0.1	7.75	0.1	0.00000544	0.00034972	A_07_P035567	c_5050	cmr|Hypothetical protein yjcB [c_5050]
yjcC	-2.74	8.07	-1.45	8.79	0.1	7.34	0.0	0.00025579	0.00303705	A_07_P052319	Z5660	cmr|orf, hypothetical protein [Z5660]
yjcC	-2.33	7.74	-1.22	8.35	0.1	7.13	0.1	0.00090055	0.00739560	A_07_P041382	c_5052	cmr|Hypothetical protein yjcC [c_5052]
yjcD	+2.58	8.75	+1.37	8.07	0.1	9.43	0.2	0.00079143	0.00672076	A_07_P008961	b4064	cmr|orf, hypothetical protein [b4064]
yjcH	+2.60	9.23	+1.38	8.54	0.1	9.92	0.0	0.00037383	0.00394212	A_07_P037683	c_5063	cmr|Hypothetical protein yjcH [c_5063]
yjcH	+2.16	9.42	+1.11	8.87	0.0	9.98	0.3	0.00377461	0.02130779	A_07_P037683	c_5063	cmr|Hypothetical protein yjcH [c_5063]
yjcH	+2.16	9.30	+1.11	8.74	0.1	9.85	0.1	0.00110963	0.00858747	A_07_P037683	c_5063	cmr|Hypothetical protein yjcH [c_5063]
yjcH	+2.17	9.32	+1.12	8.76	0.0	9.88	0.2	0.00230366	0.01467505	A_07_P037683	c_5063	cmr|Hypothetical protein yjcH [c_5063]
yjcO	-2.21	9.46	-1.14	10.03	0.0	8.89	0.1	0.00070995	0.00619249	A_07_P009034	b4078	cmr|orf, hypothetical protein [b4078]
yjcO	-2.49	9.73	-1.31	10.39	0.1	9.08	0.0	0.00037118	0.00392997	A_07_P009034	b4078	cmr|orf, hypothetical protein [b4078]
yjcO	-2.25	9.66	-1.17	10.25	0.0	9.08	0.0	0.00056055	0.00525943	A_07_P009034	b4078	cmr|orf, hypothetical protein [b4078]
yjcO	-2.02	9.58	-1.01	10.09	0.0	9.07	0.0	0.00104906	0.00821710	A_07_P009034	b4078	cmr|orf, hypothetical protein [b4078]
yjdB	+2.52	7.67	+1.33	7.01	0.2	8.34	0.1	0.00164164	0.01141493	A_07_P037782	c_5119	cmr|Hypothetical protein yjdB [c_5119]
yjdC	+4.08	9.05	+2.03	8.04	0.0	10.07	0.0	0.00003564	0.00084367	A_07_P011409	b4135	cmr|orf, hypothetical protein [b4135]
yjdJ	+2.10	8.26	+1.07	7.73	0.0	8.80	0.2	0.00315582	0.01864361	A_07_P047348	Z5729	cmr|orf, hypothetical protein [Z5729]
yjdM	+2.28	8.31	+1.19	7.71	0.2	8.90	0.0	0.00180750	0.01227664	A_07_P011276	b4108	cmr|orf, hypothetical protein [b4108]
yjdN	-2.79	8.66	-1.48	9.40	0.1	7.92	0.0	0.00025252	0.00300958	A_07_P011271	b4107	cmr|orf, hypothetical protein [b4107]
yjeI	+4.45	11.34	+2.15	10.27	0.0	12.42	0.0	0.00003207	0.00079793	A_07_P011455	b4144	cmr|orf, hypothetical protein [b4144]
yjeK	-2.23	8.85	-1.16	9.43	0.1	8.27	0.0	0.00061132	0.00556489	A_07_P011461	b4146	cmr|orf, hypothetical protein [b4146]
yjeS	-11.64	9.73	-3.54	11.50	0.0	7.96	0.1	0.00000480	0.00034614	A_07_P011550	b4166	cmr|orf, hypothetical protein [b4166]
yjfC	-2.12	7.50	-1.08	8.04	0.3	6.96	0.1	0.00523484	0.02727243	A_07_P011646	b4186	cmr|putative synthetase/amidase [b4186]
yjfN	+10.76	10.57	+3.43	8.85	0.0	12.28	0.0	0.00000296	0.00028496	A_07_P011660	b4188	cmr|orf, hypothetical protein [b4188]
yjfO	+11.70	12.32	+3.55	10.55	0.1	14.09	0.1	0.00000756	0.00040630	A_07_P035589	c_5277	cmr|Hypothetical lipoprotein yjfO precursor [c_5277]
yjfO	+10.24	10.53	+3.36	8.85	0.1	12.21	0.1	0.00000678	0.00038477	A_07_P011661	b4189	cmr|orf, hypothetical protein [b4189]
yjfO	+12.08	11.13	+3.59	9.33	0.1	12.92	0.0	0.00000294	0.00028496	A_07_P010658	b4189	cmr|orf, hypothetical protein [b4189]
yjfO	+10.13	10.77	+3.34	9.10	0.2	12.44	0.0	0.00001112	0.00048491	A_07_P048630	Z5796	cmr|orf, hypothetical protein [Z5796]
yjgA	-6.08	12.65	-2.60	13.96	0.0	11.35	0.1	0.00001171	0.00050224	A_07_P037054	c_5332	cmr|Hypothetical protein yjgA [c_5332]
yjgA	-5.05	12.54	-2.34	13.71	0.0	11.37	0.1	0.00002087	0.00063998	A_07_P003722	b4234	cmr|putative alpha helix protein [b4234]
yjgF	+35.40	11.26	+5.15	8.68	0.2	13.83	0.0	0.00000115	0.00021042	A_07_P037066	c_5342	cmr|Protein yjgF [c_5342]
yjgF	+22.20	9.99	+4.47	7.75	0.1	12.22	0.0	0.00000085	0.00019431	A_07_P003767	b4243	cmr|orf, hypothetical protein [b4243]
yjgP	-3.15	10.39	-1.66	11.22	0.1	9.56	0.1	0.00015184	0.00209697	A_07_P003851	b4261	cmr|orf, hypothetical protein [b4261]
yjgQ	-3.50	11.24	-1.81	12.15	0.0	10.34	0.0	0.00007407	0.00129569	A_07_P003856	b4262	cmr|orf, hypothetical protein [b4262]
yjgR	+5.66	8.99	+2.50	7.74	0.0	10.24	0.0	0.00001403	0.00053879	A_07_P037116	c_5364	cmr|Hypothetical protein yjgR [c_5364]
yjhD	-3.03	9.13	-1.60	9.93	0.3	8.33	0.4	0.00694566	0.03380291	A_07_P003936	b4281	cmr|orf, hypothetical protein [b4281]
yjhQ	+13.87	9.32	+3.79	7.42	0.1	11.22	0.1	0.00000257	0.00027706	A_07_P004061	b4307	cmr|orf, hypothetical protein [b4307]
yjhQ	+11.99	9.42	+3.58	7.63	0.4	11.21	0.0	0.00006636	0.00120815	A_07_P004061	b4307	cmr|orf, hypothetical protein [b4307]
yjhQ	+13.90	9.48	+3.80	7.58	0.2	11.38	0.0	0.00000856	0.00042498	A_07_P004061	b4307	cmr|orf, hypothetical protein [b4307]
yjhQ	+9.51	9.54	+3.25	7.92	0.3	11.17	0.0	0.00003209	0.00079793	A_07_P004061	b4307	cmr|orf, hypothetical protein [b4307]
yjhQ	+10.74	9.62	+3.42	7.91	0.1	11.33	0.0	0.00000334	0.00029719	A_07_P004061	b4307	cmr|orf, hypothetical protein [b4307]
yjhR	-2.64	11.85	-1.40	12.55	0.1	11.15	0.4	0.00344659	0.01992771	A_07_P004068	b4308	cmr|putative frameshift suppressor [b4308]
yjhU	-2.27	9.78	-1.18	10.37	0.1	9.19	0.1	0.00076303	0.00653952	A_07_P004004	b4295	cmr|orf, hypothetical protein [b4295]
yjhX	+9.93	10.36	+3.31	8.71	0.0	12.02	0.0	0.00000315	0.00029018	A_07_P010688	ABE-0285087	asap|conserved protein [b4566]
yjiG	+5.12	8.63	+2.36	7.45	0.0	9.81	0.1	0.00002539	0.00071770	A_07_P004174	b4329	cmr|orf, hypothetical protein [b4329]
yjiH	+3.12	7.81	+1.64	6.99	0.1	8.63	0.0	0.00016515	0.00222615	A_07_P004179	b4330	cmr|orf, hypothetical protein [b4330]
yjiL	+2.24	9.18	+1.16	8.60	0.2	9.76	0.3	0.00788191	0.03742245	A_07_P004196	b4334	cmr|putative enzyme [b4334]
yjiM	+5.00	8.80	+2.32	7.64	0.1	9.96	0.0	0.00003498	0.00083975	A_07_P004201	b4335	cmr|orf, hypothetical protein [b4335]
yjiX	+3.11	9.47	+1.64	8.66	0.1	10.29	0.0	0.00016636	0.00223475	A_07_P017462	b4353	cmr|orf, hypothetical protein [b4353]
yjjI	+4.53	10.60	+2.18	9.51	0.0	11.69	0.0	0.00002727	0.00073404	A_07_P017585	b4380	cmr|orf, hypothetical protein [b4380]
yjjK	+4.70	10.15	+2.23	9.03	0.1	11.26	0.0	0.00003527	0.00084015	A_07_P017637	b4391	cmr|putative ATP-binding component of a transport system [b4391]
yjjK	+4.48	10.11	+2.16	9.03	0.0	11.19	0.1	0.00003510	0.00083975	A_07_P017637	b4391	cmr|putative ATP-binding component of a transport system [b4391]
yjjK	+4.23	9.96	+2.08	8.92	0.0	11.00	0.1	0.00004397	0.00094686	A_07_P017637	b4391	cmr|putative ATP-binding component of a transport system [b4391]
yjjK	+4.28	10.12	+2.10	9.07	0.0	11.17	0.0	0.00003223	0.00079793	A_07_P017637	b4391	cmr|putative ATP-binding component of a transport system [b4391]
yjjK	+4.52	10.20	+2.18	9.11	0.0	11.29	0.0	0.00002582	0.00072358	A_07_P017637	b4391	cmr|putative ATP-binding component of a transport system [b4391]
yjjN	-2.32	7.47	-1.22	8.08	0.0	6.86	0.1	0.00089564	0.00737883	A_07_P017488	b4358	cmr|putative oxidoreductase [b4358]
yjjN	-2.38	7.58	-1.25	8.21	0.0	6.96	0.0	0.00041590	0.00423720	A_07_P017488	b4358	cmr|putative oxidoreductase [b4358]
yjjQ	-2.31	7.29	-1.21	7.89	0.2	6.68	0.0	0.00097819	0.00779781	A_07_P042086	c_5444	cmr|Hypothetical protein yjjQ [c_5444]
yjjQ	-3.05	7.48	-1.61	8.29	0.0	6.68	0.2	0.00060030	0.00549480	A_07_P017521	b4365	cmr|putative regulator [b4365]
ykfJ	-10.25	11.42	-3.36	13.10	0.0	9.74	0.0	0.00000350	0.00030339	A_07_P007889	b0235	cmr|orf, hypothetical protein [b0235]
ykgE	-2.05	10.89	-1.04	11.41	0.3	10.37	0.0	0.00852239	0.03965027	A_07_P008225	b0306	cmr|putative dehydrogenase subunit [b0306]
ykgJ	-3.75	8.46	-1.91	9.42	0.3	7.51	0.1	0.00077925	0.00664237	A_07_P008131	b0288	cmr|putative ferredoxin [b0288]
ykiA	-2.06	7.65	-1.05	8.17	0.2	7.13	0.0	0.00249534	0.01557755	A_07_P021074	b0392	cmr|orf, hypothetical protein [b0392]
ylaB	-2.32	11.16	-1.21	11.76	0.1	10.55	0.2	0.00146122	0.01047130	A_07_P021391	b0457	cmr|orf, hypothetical protein [b0457]
ylaC	-13.96	11.94	-3.80	13.84	0.0	10.04	0.2	0.00000400	0.00032449	A_07_P021396	b0458	cmr|orf, hypothetical protein [b0458]
yliG	+2.26	9.13	+1.17	8.54	0.1	9.71	0.1	0.00105423	0.00824530	A_07_P004626	b0835	cmr|orf, hypothetical protein [b0835]
yliG	+2.63	10.55	+1.39	9.86	0.0	11.25	0.0	0.00023226	0.00282805	A_07_P042116	c_0920	cmr|Hypothetical protein yliG [c_0920]
ymcE	+6.37	9.65	+2.67	8.31	0.0	10.98	0.0	0.00001009	0.00046432	A_07_P001189	b0991	cmr|suppresses fabA and ts growth mutation [b0991]
ymdF	-2.15	12.54	-1.11	13.09	0.0	11.98	0.0	0.00072574	0.00628152	A_07_P010350	ABE-0285039	asap|conserved protein [b4518]
ymfA	-4.66	8.59	-2.22	9.70	0.0	7.48	0.0	0.00002458	0.00070547	A_07_P040833	c_1397	cmr|Hypothetical protein ymfA [c_1397]
ymfC	-3.22	13.67	-1.69	14.52	0.0	12.83	0.0	0.00008820	0.00145271	A_07_P050485	Z1864	cmr|orf, hypothetical protein [Z1864]
ymgA	+7.02	8.08	+2.81	6.68	0.1	9.49	0.1	0.00001439	0.00054604	A_07_P006777	b1165	cmr|orf, hypothetical protein [b1165]
ymgB	+11.70	10.42	+3.55	8.65	0.2	12.20	0.1	0.00000994	0.00046030	A_07_P038703	c_1609	cmr|Hypothetical protein ymgB [c_1609]
ymgD	-3.12	8.63	-1.64	9.45	0.3	7.81	0.1	0.00103452	0.00811965	A_07_P006807	b1171	cmr|orf, hypothetical protein [b1171]
ymgG	-3.27	12.81	-1.71	13.67	0.0	11.96	0.1	0.00012847	0.00184717	A_07_P006812	b1172	cmr|orf, hypothetical protein [b1172]
ymjA	+2.31	7.96	+1.21	7.36	0.3	8.56	0.1	0.00344180	0.01990730	A_07_P014457	b1295	cmr|orf, hypothetical protein [b1295]
ynaI	-9.22	10.37	-3.20	11.97	0.1	8.77	0.1	0.00000761	0.00040630	A_07_P014632	b1330	cmr|orf, hypothetical protein [b1330]
ynbE	+2.08	7.63	+1.05	7.10	0.0	8.16	0.1	0.00150391	0.01072842	A_07_P034809	c_1829	cmr|Hypothetical protein ynbE [c_1829]
yneG	-2.00	8.08	-1.00	8.58	0.2	7.58	0.0	0.00272833	0.01664824	A_07_P035023	c_1946	cmr|Hypothetical protein yneG [c_1946]
yneH	-2.86	10.25	-1.51	11.00	0.1	9.49	0.1	0.00037056	0.00392997	A_07_P020270	b1524	cmr|putative glutaminase [b1524]
yneI	-2.15	9.54	-1.11	10.09	0.0	8.98	0.0	0.00071789	0.00623756	A_07_P035026	c_1948	cmr|Aldehyde-dehydrogenase like protein yneI [c_1948]
yneJ	+2.26	7.92	+1.17	7.33	0.1	8.50	0.0	0.00090291	0.00739560	A_07_P035034	c_1949	cmr|Hypothetical transcriptional regulator yneJ [c_1949]
ynfB	-4.43	14.07	-2.15	15.14	0.0	13.00	0.1	0.00003346	0.00081556	A_07_P020550	b1583	cmr|orf, hypothetical protein [b1583]
ynfE	+2.76	7.61	+1.46	6.88	0.3	8.34	0.0	0.00191603	0.01278216	A_07_P020568	b1587	cmr|putative oxidoreductase, major subunit [b1587]
ynfE	+4.20	8.29	+2.07	7.25	0.0	9.32	0.1	0.00004287	0.00093353	A_07_P042301	c_1977	cmr|Putative dimethyl sulfoxide reductase chain ynfE precursor [c_1977]
ynfF	+2.15	9.09	+1.10	8.54	0.3	9.64	0.1	0.00551450	0.02841910	A_07_P020575	b1588	cmr|putative oxidoreductase, major subunit [b1588]
ynfG	+5.58	8.56	+2.48	7.32	0.3	9.80	0.1	0.00012522	0.00182539	A_07_P020576	b1589	cmr|putative oxidoreductase, Fe-S subunit [b1589]
ynfM	-2.65	11.79	-1.41	12.49	0.4	11.09	0.0	0.00334249	0.01950487	A_07_P020611	b1596	cmr|putative transport protein [b1596]
yniA	+4.06	8.43	+2.02	7.42	0.0	9.44	0.1	0.00006818	0.00122531	A_07_P018674	b1725	cmr|orf, hypothetical protein [b1725]
ynjB	+2.62	7.80	+1.39	7.10	0.0	8.50	0.1	0.00027775	0.00319869	A_07_P018816	b1754	cmr|orf, hypothetical protein [b1754]
ynjE	+4.32	9.16	+2.11	8.11	0.4	10.22	0.1	0.00059507	0.00547004	A_07_P018832	b1757	cmr|putative thiosulfate sulfur transferase [b1757]
yoaB	+5.31	9.23	+2.41	8.03	0.0	10.44	0.0	0.00001686	0.00058050	A_07_P015736	b1809	cmr|orf, hypothetical protein [b1809]
yobB	+2.93	8.81	+1.55	8.04	0.1	9.59	0.0	0.00016131	0.00218930	A_07_P015892	b1843	cmr|orf, hypothetical protein [b1843]
yobH	-2.47	7.57	-1.31	8.22	0.2	6.92	0.0	0.00100920	0.00798031	A_07_P010464	ABE-0285057	asap|predicted protein [b4536]
yohK	-2.02	7.45	-1.02	7.96	0.0	6.94	0.0	0.00101102	0.00799073	A_07_P003473	b2142	cmr|putative seritonin transporter [b2142]
yohM	-2.06	10.33	-1.04	10.85	0.3	9.81	0.2	0.00833014	0.03892837	A_07_P045159	Z3274	cmr|orf, hypothetical protein [Z3274]
ypfG	-4.66	10.03	-2.22	11.14	0.0	8.92	0.0	0.00002330	0.00068274	A_07_P042601	c_2991	cmr|Hypothetical protein ypfG precursor [c_2991]
ypfG	-2.74	9.78	-1.46	10.51	0.1	9.06	0.1	0.00038817	0.00402870	A_07_P013386	b2466	cmr|orf, hypothetical protein [b2466]
ypfN	-2.22	9.64	-1.15	10.22	0.0	9.06	0.0	0.00056388	0.00527744	A_07_P010535	ABE-0285068	asap|predicted protein [b4547]
yphE	-3.23	12.98	-1.69	13.82	0.0	12.13	0.0	0.00009758	0.00154382	A_07_P019639	b2547	cmr|putative ATP-binding component of a transport system [b2547]
yphF	-2.06	8.01	-1.05	8.53	0.2	7.49	0.0	0.00359956	0.02061632	A_07_P019645	b2548	cmr|putative LACI-type transcriptional regulator [b2548]
yqaA	-2.67	10.08	-1.42	10.79	0.1	9.37	0.0	0.00034465	0.00373190	A_07_P001920	b2689	cmr|orf, hypothetical protein [b2689]
yqaB	-6.55	11.29	-2.71	12.64	0.0	9.93	0.0	0.00000939	0.00044530	A_07_P030582	c_3247	cmr|Hypothetical protein yqaB [c_3247]
yqaB	-4.89	11.00	-2.29	12.15	0.0	9.85	0.1	0.00002355	0.00068545	A_07_P001923	b2690	cmr|putative phosphatase [b2690]
yqcA	+3.73	9.63	+1.90	8.68	0.1	10.58	0.1	0.00009729	0.00154382	A_07_P002396	b2790	cmr|orf, hypothetical protein [b2790]
yqcC	+2.28	8.50	+1.19	7.91	0.1	9.10	0.1	0.00061695	0.00559838	A_07_P002409	b2792	cmr|orf, hypothetical protein [b2792]
yqeB	-2.72	8.51	-1.44	9.24	0.2	7.79	0.1	0.00080747	0.00682752	A_07_P006342	b2875	cmr|putative synthases [b2875]
yqeC	-2.54	8.69	-1.35	9.37	0.2	8.02	0.1	0.00141659	0.01021191	A_07_P006347	b2876	cmr|orf, hypothetical protein [b2876]
yqfA	+2.01	9.43	+1.01	8.93	0.1	9.94	0.0	0.00148444	0.01060874	A_07_P006461	b2899	cmr|putative oxidoreductase [b2899]
yqfB	+2.04	7.54	+1.03	7.03	0.1	8.06	0.2	0.00473002	0.02520968	A_07_P045648	Z4238	cmr|orf, hypothetical protein [Z4238]
yqfB	+2.12	7.49	+1.09	6.95	0.1	8.04	0.2	0.00255272	0.01584154	A_07_P045648	Z4238	cmr|orf, hypothetical protein [Z4238]
yqfB	+2.81	10.18	+1.49	9.43	0.1	10.92	0.1	0.00028717	0.00327619	A_07_P006468	b2900	cmr|orf, hypothetical protein [b2900]
yqfB	+2.08	7.81	+1.06	7.28	0.1	8.34	0.2	0.00334497	0.01951211	A_07_P045648	Z4238	cmr|orf, hypothetical protein [Z4238]
yqfB	+2.22	7.76	+1.15	7.19	0.1	8.34	0.2	0.00262711	0.01620104	A_07_P045648	Z4238	cmr|orf, hypothetical protein [Z4238]
yqfB	+2.72	10.50	+1.45	9.78	0.1	11.22	0.0	0.00023330	0.00283859	A_07_P032874	c_3481	cmr|Hypothetical protein yqfB [c_3481]
yqfE	-2.42	12.69	-1.27	13.33	0.2	12.05	0.0	0.00091762	0.00749052	A_07_P006538	b2915	cmr|orf, hypothetical protein [b2915]
yqgE	+2.86	10.52	+1.52	9.76	0.1	11.28	0.1	0.00048238	0.00470257	A_07_P011885	b2948	cmr|orf, hypothetical protein [b2948]
yqhA	-2.81	8.99	-1.49	9.74	0.0	8.25	0.0	0.00016060	0.00218551	A_07_P012144	b3002	cmr|orf, hypothetical protein [b3002]
yqhD	+2.81	10.37	+1.49	9.63	0.2	11.12	0.0	0.00045824	0.00455748	A_07_P012186	b3011	cmr|putative oxidoreductase [b3011]
yqjA	-9.14	11.24	-3.19	12.84	0.0	9.65	0.0	0.00000408	0.00032474	A_07_P000202	b3095	cmr|orf, hypothetical protein [b3095]
yqjA	-11.45	10.81	-3.52	12.57	0.0	9.05	0.1	0.00000264	0.00027706	A_07_P041768	c_3853	cmr|Hypothetical protein yqjA [c_3853]
yqjB	-4.07	10.37	-2.03	11.39	0.1	9.36	0.1	0.00008827	0.00145271	A_07_P000206	b3096	cmr|orf, hypothetical protein [b3096]
yqjC	+3.87	10.59	+1.95	9.62	0.1	11.57	0.0	0.00005335	0.00106045	A_07_P000212	b3097	cmr|orf, hypothetical protein [b3097]
yqjI	+5.47	11.64	+2.45	10.42	0.1	12.87	0.0	0.00001734	0.00058395	A_07_P000083	b3071	cmr|orf, hypothetical protein [b3071]
yraP	+4.02	10.23	+2.01	9.23	0.0	11.24	0.0	0.00004282	0.00093353	A_07_P000475	b3150	cmr|putative periplasmic protein [b3150]
yrbA	+3.30	10.39	+1.72	9.53	0.1	11.25	0.0	0.00011328	0.00169854	A_07_P012426	b3190	cmr|orf, hypothetical protein [b3190]
yrbB	+2.07	8.65	+1.05	8.13	0.2	9.18	0.1	0.00350282	0.02015319	A_07_P035537	c_3950	cmr|Hypothetical protein yrbB [c_3950]
yrbE	-3.86	10.98	-1.95	11.95	0.1	10.00	0.0	0.00005103	0.00103301	A_07_P012449	b3194	cmr|orf, hypothetical protein [b3194]
yrbF	-2.58	11.04	-1.37	11.73	0.2	10.36	0.2	0.00122328	0.00918860	A_07_P012451	b3195	cmr|putative ATP-binding component of a transport system [b3195]
yrbK	+2.51	12.47	+1.33	11.81	0.0	13.13	0.0	0.00031071	0.00345228	A_07_P012474	b3199	cmr|orf, hypothetical protein [b3199]
yrbL	+4.28	9.65	+2.10	8.61	0.3	10.70	0.0	0.00024195	0.00291652	A_07_P052110	Z4570	cmr|orf, hypothetical protein [Z4570]
yrbL	+4.59	10.35	+2.20	9.25	0.0	11.45	0.0	0.00002514	0.00071646	A_07_P012512	b3207	cmr|orf, hypothetical protein [b3207]
yrdD	+2.28	8.98	+1.19	8.38	0.0	9.57	0.1	0.00063065	0.00568996	A_07_P012850	b3283	cmr|putative DNA topoisomerase [b3283]
yrfF	-4.46	9.51	-2.16	10.59	0.0	8.43	0.2	0.00012662	0.00183570	A_07_P034400	c_4169	cmr|Putative membrane protein igaA homolog [c_4169]
yrfF	-3.06	8.95	-1.61	9.76	0.0	8.14	0.2	0.00037942	0.00397175	A_07_P018019	b3398	cmr|putative dehydrogenase [b3398]
ysaA	-2.28	8.54	-1.19	9.13	0.0	7.94	0.0	0.00047049	0.00463832	A_07_P007793	b3573	cmr|orf, hypothetical protein [b3573]
ytfE	+2.10	7.77	+1.07	7.23	0.0	8.30	0.1	0.00093975	0.00760301	A_07_P037039	c_5308	cmr|Hypothetical protein ytfE [c_5308]
ytfH	-3.16	10.44	-1.66	11.27	0.2	9.61	0.1	0.00039984	0.00412524	A_07_P003611	b4212	cmr|orf, hypothetical protein [b4212]
ytfH	-2.74	10.21	-1.45	10.94	0.0	9.49	0.1	0.00026556	0.00309930	A_07_P035591	c_5311	cmr|Hypothetical protein ytfH [c_5311]
ytfL	-2.01	9.08	-1.00	9.59	0.1	8.58	0.2	0.00338833	0.01968112	A_07_P003641	b4218	cmr|putative transport protein [b4218]
ytfQ	+2.39	8.95	+1.26	8.32	0.2	9.58	0.1	0.00157667	0.01109658	A_07_P037048	c_5325	cmr|ABC transporter Periplasmic binding protein ytfQ precursor [c_5325]
Z0024	-2.23	8.25	-1.16	8.83	0.3	7.67	0.1	0.00489128	0.02592874	A_07_P049126	Z0024	cmr|putative type-1 fimbrial protein [Z0024]
Z0056	-2.35	12.07	-1.24	12.69	0.1	11.45	0.0	0.00047557	0.00466588	A_07_P049148	Z0056	cmr|putative antitoxin of gyrase inhibiting toxin-antitoxin system [Z0056]
Z0202	-2.13	11.19	-1.09	11.74	0.0	10.65	0.0	0.00078870	0.00670479	A_07_P049248	Z0202	cmr|orf Unknown function [Z0202]
Z0309	-4.91	12.42	-2.30	13.56	0.0	11.27	0.0	0.00002030	0.00063193	A_07_P043395	Z0309	cmr|putative cI repressor protein for prophage CP-933H [Z0309]
Z0311	+12.08	13.77	+3.59	11.97	0.0	15.57	0.1	0.00000272	0.00028012	A_07_P043403	Z0311	cmr|partial O replication protein for prophage CP-933H [Z0311]
Z0312	+2.44	14.77	+1.29	14.12	0.0	15.41	0.1	0.00061518	0.00558557	A_07_P043406	Z0312	cmr|partial O replication protein for prophage CP-933H [Z0312]
Z0319	-2.19	9.06	-1.13	9.63	0.2	8.50	0.2	0.00265065	0.01633339	A_07_P043443	Z0319	cmr|unknown protein from prophage CP-933H [Z0319]
Z0370	+3.31	8.43	+1.73	7.56	0.2	9.29	0.0	0.00022281	0.00274273	A_07_P043596	Z0370	cmr|orf Unknown function [Z0370]
Z0395	-2.08	8.81	-1.06	9.34	0.2	8.28	0.1	0.00493578	0.02610312	A_07_P043668	Z0395	cmr|orf Unknown function [Z0395]
Z0666	+2.39	7.44	+1.26	6.81	0.0	8.07	0.1	0.00045361	0.00451997	A_07_P043951	Z0666	cmr|orf Unknown function [Z0666]
Z0949	+19.20	11.20	+4.26	9.07	0.3	13.33	0.4	0.00005135	0.00103783	A_07_P046386	Z0949	cmr|unknown protein encoded by prophage CP-933K [Z0949]
Z0950	+59.01	10.59	+5.88	7.65	0.1	13.53	0.2	0.00000090	0.00019431	A_07_P046393	Z0950	cmr|unknown protein encoded by prophage CP-933K [Z0950]
Z0951	+7.25	13.37	+2.86	11.94	0.1	14.80	0.3	0.00009771	0.00154382	A_07_P046396	Z0951	cmr|putative exonuclease encoded by prophage CP-933K [Z0951]
Z0952	+8.89	13.11	+3.15	11.53	0.1	14.68	0.0	0.00000517	0.00034758	A_07_P046403	Z0952	cmr|putative Bet recombination protein of prophage CP-933K [Z0952]
Z0960	+27.61	10.13	+4.79	7.73	0.1	12.52	0.1	0.00000137	0.00022125	A_07_P046435	Z0960	cmr|putative lysozyme protein R of prophage CP-933K [Z0960]
Z0965	-2.22	9.85	-1.15	10.43	0.1	9.28	0.0	0.00083981	0.00702923	A_07_P046451	Z0965	cmr|unknown protein encoded by prophage CP-933K [Z0965]
Z0966	-2.64	8.22	-1.40	8.92	0.1	7.52	0.1	0.00050848	0.00488792	A_07_P046460	Z0966	cmr|unknown protein encoded by prophage CP-933K [Z0966]
Z0966	-2.93	8.18	-1.55	8.96	0.1	7.40	0.1	0.00027382	0.00316517	A_07_P046460	Z0966	cmr|unknown protein encoded by prophage CP-933K [Z0966]
Z0966	-3.41	8.10	-1.77	8.98	0.0	7.21	0.1	0.00013193	0.00188652	A_07_P046460	Z0966	cmr|unknown protein encoded by prophage CP-933K [Z0966]
Z0966	-2.62	8.07	-1.39	8.77	0.0	7.38	0.2	0.00060146	0.00550224	A_07_P046460	Z0966	cmr|unknown protein encoded by prophage CP-933K [Z0966]
Z0966	-3.30	8.20	-1.72	9.06	0.1	7.34	0.1	0.00012608	0.00183113	A_07_P046460	Z0966	cmr|unknown protein encoded by prophage CP-933K [Z0966]
Z0971	+2.19	7.31	+1.13	6.75	0.0	7.88	0.1	0.00079947	0.00677807	A_07_P046483	Z0971	cmr|putative tail component of prophage CP-933K [Z0971]
Z0974	-2.30	9.81	-1.20	10.41	0.3	9.20	0.0	0.00397875	0.02211190	A_07_P046497	Z0974	cmr|putative tail component of prophage CP-933K [Z0974]
Z0977	+5.33	9.55	+2.41	8.35	0.3	10.76	0.2	0.00039883	0.00412020	A_07_P046512	Z0977	cmr|putative tail component of prophage CP-933K [Z0977]
Z0979	+4.31	8.84	+2.11	7.79	0.0	9.89	0.1	0.00003967	0.00090487	A_07_P046522	Z0979	cmr|putative tail component of prophage CP-933K [Z0979]
Z1046	+2.36	7.36	+1.24	6.74	0.0	7.98	0.0	0.00039155	0.00405638	A_07_P046587	Z1046	cmr|orf Unknown function [Z1046]
Z1051	+2.47	10.18	+1.30	9.53	0.1	10.84	0.1	0.00066107	0.00587078	A_07_P046593	Z1051	cmr|orf Unknown function [Z1051]
Z1058	-2.07	7.29	-1.05	7.81	0.0	6.76	0.0	0.00090235	0.00739560	A_07_P052018	Z1058	cmr|orf, hypothetical protein [Z1058]
Z1098	-2.16	11.48	-1.11	12.03	0.1	10.92	0.0	0.00136054	0.00990669	A_07_P046651	Z1098	cmr|orf Unknown function [Z1098]
Z1120	-2.01	10.97	-1.01	11.47	0.1	10.46	0.0	0.00167783	0.01160625	A_07_P046671	Z1120	cmr|putative P4-family integrase [Z1120]
Z1121	+2.66	8.57	+1.41	7.86	0.3	9.28	0.2	0.00271778	0.01661055	A_07_P046679	Z1121	cmr|orf Unknown function [Z1121]
Z1124	+2.19	6.96	+1.13	6.40	0.1	7.53	0.2	0.00218253	0.01408847	A_07_P046689	Z1124	cmr|putative prophage regulatory protein [Z1124]
Z1129	-2.92	13.40	-1.55	14.17	0.3	12.62	0.1	0.00095945	0.00769519	A_07_P046708	Z1129	cmr|putative helicase [Z1129]
Z1134	-2.36	7.93	-1.24	8.55	0.0	7.31	0.0	0.00042980	0.00434325	A_07_P046735	Z1134	cmr|unknown in IS600 [Z1134]
Z1150	-2.51	8.30	-1.33	8.97	0.2	7.64	0.1	0.00076977	0.00658197	A_07_P046801	Z1150	cmr|unknown in IS [Z1150]
Z1160	-3.11	9.74	-1.64	10.55	0.2	8.92	0.1	0.00045343	0.00451997	A_07_P046840	Z1160	cmr|unknown in ISEc8 [Z1160]
Z1161	-2.44	8.87	-1.29	9.51	0.0	8.22	0.1	0.00066920	0.00592567	A_07_P046841	Z1161	cmr|unknown in ISEc8 [Z1161]
Z1193	-2.09	7.80	-1.06	8.33	0.2	7.26	0.1	0.00553146	0.02846227	A_07_P046975	Z1193	cmr|orf Unknown function [Z1193]
Z1199	-2.52	9.62	-1.33	10.29	0.1	8.96	0.0	0.00045896	0.00456178	A_07_P050887	Z1199	cmr|unknown in IS [Z1199]
Z1203	-2.22	10.65	-1.15	11.23	0.2	10.08	0.1	0.00206209	0.01347675	A_07_P050909	Z1203	cmr|unknown [Z1203]
Z1204	-2.05	8.64	-1.03	9.16	0.0	8.13	0.1	0.00112179	0.00866326	A_07_P050912	Z1204	cmr|unknown [Z1204]
Z1210	-2.51	7.65	-1.33	8.32	0.1	6.99	0.2	0.00165807	0.01150422	A_07_P050942	Z1210	cmr|putative histone [Z1210]
Z1214	+2.68	9.07	+1.42	8.36	0.1	9.78	0.0	0.00026174	0.00308181	A_07_P050956	Z1214	cmr|orf Unknown function [Z1214]
Z1340	-2.42	9.94	-1.28	10.58	0.4	9.30	0.1	0.00894630	0.04122051	A_07_P051102	Z1340	cmr|unknown protein encoded by cryptic prophage CP-933M [Z1340]
Z1352	-3.58	9.17	-1.84	10.09	0.4	8.25	0.1	0.00138701	0.01003542	A_07_P051141	Z1352	cmr|putative endolysin of cryptic prophage CP-933M [Z1352]
Z1373	-2.12	11.11	-1.08	11.65	0.1	10.56	0.1	0.00151862	0.01081374	A_07_P051220	Z1373	cmr|unknown protein encoded by cryptic prophage CP-933M [Z1373]
Z1428	+45.67	10.00	+5.51	7.24	0.1	12.76	0.0	0.00000040	0.00019431	A_07_P048529	Z1428	cmr|unknown protein encoded by bacteriophage BP-933W [Z1428]
Z1429	-2.60	8.44	-1.38	9.13	0.0	7.75	0.1	0.00028377	0.00325162	A_07_P051303	Z1429	cmr|unknown protein encoded by bacteriophage BP-933W [Z1429]
Z1433	+56.21	11.60	+5.81	8.69	0.2	14.50	0.1	0.00000093	0.00019431	A_07_P051325	Z1433	cmr|unknown protein encoded by bacteriophage BP-933W [Z1433]
Z1434	+52.93	10.37	+5.73	7.50	0.3	13.23	0.2	0.00000230	0.00026836	A_07_P051326	Z1434	cmr|unknown protein encoded by bacteriophage BP-933W [Z1434]
Z1444	-3.12	12.66	-1.64	13.48	0.1	11.84	0.1	0.00016403	0.00221677	A_07_P051356	Z1444	cmr|putative serine/threonine kinase encoded by bacteriophage BP-933W [Z1444]
Z1449	+7.12	11.18	+2.83	9.77	0.2	12.60	0.0	0.00002361	0.00068568	A_07_P051385	Z1449	cmr|putative regulatory protein CII of bacteriophage BP-933W [Z1449]
Z1454	+59.98	10.27	+5.91	7.31	0.3	13.22	0.0	0.00000200	0.00025681	A_07_P051405	Z1454	cmr|putative DNA N-6-adenine-methyltransferase of bacteriophage BP-933W [Z1454]
Z1457	-2.83	11.01	-1.50	11.76	0.4	10.26	0.1	0.00326149	0.01913860	A_07_P051411	Z1457	cmr|putative DNA-binding protein Roi of bacteriophage BP-933W [Z1457]
Z1473	+15.20	9.15	+3.93	7.19	0.3	11.11	0.0	0.00001949	0.00062241	A_07_P051457	Z1473	cmr|putative endopeptidase Rz of bacteriophage BP-933W [Z1473]
Z1475	-2.39	9.26	-1.26	9.89	0.3	8.63	0.2	0.00466199	0.02493152	A_07_P051463	Z1475	cmr|putative terminase small subunit of bacteriophage BP-933W [Z1475]
Z1534	-2.12	8.80	-1.08	9.34	0.2	8.26	0.0	0.00260952	0.01613044	A_07_P051615	Z1534	cmr|putative chaperone [Z1534]
Z1660	-3.29	8.43	-1.72	9.29	0.0	7.57	0.1	0.00011484	0.00172031	A_07_P050081	Z1660	cmr|transposase for IS629 [Z1660]
Z1778	-2.09	11.81	-1.07	12.35	0.1	11.28	0.1	0.00105620	0.00825663	A_07_P050169	Z1778	cmr|unknown protein encoded by prophage CP-933N [Z1778]
Z1779	-2.10	7.61	-1.07	8.14	0.1	7.08	0.2	0.00335693	0.01956012	A_07_P050172	Z1779	cmr|unknown protein encoded by prophage CP-933N [Z1779]
Z1781	+21.31	10.13	+4.41	7.93	0.2	12.34	0.1	0.00000248	0.00027471	A_07_P050182	Z1781	cmr|unknown protein encoded by prophage CP-933N [Z1781]
Z1794	-2.67	8.82	-1.42	9.53	0.1	8.12	0.0	0.00026812	0.00311300	A_07_P050226	Z1794	cmr|putative holin protein [Z1794]
Z1805	-2.40	11.53	-1.26	12.16	0.4	10.90	0.3	0.01049221	0.04650600	A_07_P050276	Z1805	cmr|unknown protein encoded by prophage CP-933N [Z1805]
Z1819	+3.82	9.02	+1.93	8.05	0.5	9.99	0.2	0.00337643	0.01962668	A_07_P050332	Z1819	cmr|putative tail component K homolog encoded by [Z1819]
Z1856	-3.22	7.74	-1.69	8.59	0.0	6.90	0.2	0.00040565	0.00416132	A_07_P050473	Z1856	cmr|unknown protein encoded by prophage CP-933C [Z1856]
Z1866	+16.26	8.98	+4.02	6.97	0.1	10.99	0.2	0.00000783	0.00041039	A_07_P050491	Z1866	cmr|putative integrase of prophage CP-933X [Z1866]
Z1867	+104.78	10.92	+6.71	7.57	0.1	14.28	0.0	0.00000011	0.00019431	A_07_P050497	Z1867	cmr|putative integrase of prophage CP-933X [Z1867]
Z1869	+6.17	12.44	+2.63	11.13	0.1	13.76	0.0	0.00001435	0.00054604	A_07_P052425	Z1869	cmr|putative replication protein P of prophage CP-933X [Z1869]
Z1871	-2.16	8.52	-1.11	9.07	0.0	7.96	0.1	0.00094719	0.00763638	A_07_P050509	Z1871	cmr|unknown protein encoded by prophage CP-933X [Z1871]
Z1891	+24.01	10.55	+4.59	8.26	0.1	12.84	0.1	0.00000085	0.00019431	A_07_P050583	Z1891	cmr|putative tail component of prophage CP-933X [Z1891]
Z1893	+5.94	11.21	+2.57	9.92	0.1	12.49	0.1	0.00002337	0.00068274	A_07_P050588	Z1893	cmr|putative tail component of prophage CP-933X [Z1893]
Z1894	+24.34	9.70	+4.61	7.40	0.1	12.00	0.1	0.00000091	0.00019431	A_07_P050594	Z1894	cmr|putative tail component of prophage CP-933X [Z1894]
Z1895	+24.61	9.30	+4.62	6.99	0.3	11.62	0.2	0.00001694	0.00058050	A_07_P050599	Z1895	cmr|putative tail component of prophage CP-933X [Z1895]
Z1896	+5.02	8.28	+2.33	7.12	0.3	9.44	0.1	0.00019887	0.00253112	A_07_P050602	Z1896	cmr|putative tail component of prophage CP-933X [Z1896]
Z1898	+7.75	10.60	+2.95	9.12	0.4	12.07	0.1	0.00024235	0.00291707	A_07_P050610	Z1898	cmr|putative tail component of prophage CP-933X [Z1898]
Z1915	+5.05	8.94	+2.34	7.77	0.0	10.11	0.1	0.00003709	0.00087015	A_07_P050656	Z1915	cmr|putative tail protein [Z1915]
Z1919	-2.37	7.76	-1.24	8.38	0.4	7.14	0.1	0.00725740	0.03504535	A_07_P050674	Z1919	cmr|unknown protein encoded by prophage CP-933X [Z1919]
Z1957	-5.96	10.15	-2.57	11.44	0.1	8.86	0.0	0.00001412	0.00053964	A_07_P050726	Z1957	cmr|transposase for IS629 [Z1957]
Z2005	-2.67	12.53	-1.41	13.24	0.2	11.82	0.5	0.01006809	0.04494246	A_07_P050791	Z2005	cmr|orf Unknown function [Z2005]
Z2054	-2.59	15.49	-1.37	16.17	0.1	14.80	0.3	0.00155307	0.01097764	A_07_P047630	Z2054	cmr|putative killer protein encoded by prophage CP-933O [Z2054]
Z2074	-4.21	10.94	-2.07	11.98	0.1	9.90	0.0	0.00004783	0.00099032	A_07_P047687	Z2074	cmr|putative IS encoded protein within CP-933O [Z2074]
Z2076	-2.09	11.18	-1.06	11.71	0.1	10.65	0.1	0.00123192	0.00922733	A_07_P047700	Z2076	cmr|unknown protein encoded by prophage CP-933O [Z2076]
Z2080	-7.96	11.50	-2.99	13.00	0.1	10.01	0.0	0.00000625	0.00036832	A_07_P047717	Z2080	cmr|putative IS encoded protein within CP-933O [Z2080]
Z2118	+2.41	7.38	+1.27	6.74	0.1	8.01	0.0	0.00056298	0.00527744	A_07_P047862	Z2118	cmr|putative endopeptidase Rz of prophage CP-933O [Z2118]
Z2119	-4.04	8.69	-2.01	9.69	0.0	7.68	0.0	0.00004001	0.00090512	A_07_P047868	Z2119	cmr|unknown protein encoded within prophage CP-933O [Z2119]
Z2131	+3.95	9.45	+1.98	8.46	0.2	10.44	0.1	0.00016105	0.00218777	A_07_P047904	Z2131	cmr|putative terminase large subunit of prophage CP-933O [Z2131]
Z2133	+9.21	8.61	+3.20	7.01	0.1	10.22	0.0	0.00000616	0.00036832	A_07_P047906	Z2133	cmr|putative capsid assembly protein of prophage CP-933O [Z2133]
Z2136	+16.45	9.30	+4.04	7.28	0.4	11.32	0.2	0.00007196	0.00126447	A_07_P047913	Z2136	cmr|partial putative major capsid protein of prophage [Z2136]
Z2140	+3.33	7.83	+1.74	6.97	0.0	8.70	0.0	0.00007845	0.00133820	A_07_P047929	Z2140	cmr|putative tail component of prophage CP-933O [Z2140]
Z2142	-2.66	11.83	-1.41	12.54	0.2	11.12	0.3	0.00304423	0.01807256	A_07_P047939	Z2142	cmr|putative tail component of prophage CP-933O [Z2142]
Z2144	-2.53	10.81	-1.34	11.48	0.3	10.14	0.2	0.00421745	0.02312764	A_07_P047949	Z2144	cmr|putative tail component of prophage CP-933O [Z2144]
Z2181	-2.61	7.76	-1.38	8.45	0.2	7.07	0.3	0.00257129	0.01593793	A_07_P048026	Z2181	cmr|orf Unknown function [Z2181]

Z2297	-2.14	10.96	-1.10	11.51	0.1	10.41	0.2	0.00253752	0.01578201	A_07_P048153	Z2297	cmr|orf Unknown function [Z2297]
Z2298	-3.16	9.05	-1.66	9.88	0.1	8.22	0.1	0.00020782	0.00261329	A_07_P048160	Z2298	cmr|orf Unknown function [Z2298]
Z2360	+31.47	10.18	+4.98	7.69	0.5	12.67	0.3	0.00004287	0.00093353	A_07_P048290	Z2360	cmr|putative capsid protein of prophage CP-933R [Z2360]
Z2361	+43.56	11.10	+5.44	8.37	0.0	13.82	0.1	0.00000034	0.00019431	A_07_P048291	Z2361	cmr|putative capsid assembly protein of prophage CP-933R [Z2361]
Z2391	+52.43	9.78	+5.71	6.93	0.2	12.64	0.0	0.00000058	0.00019431	A_07_P048371	Z2391	cmr|unknown protein encoded within prophage CP-933R [Z2391]
Z2519	+2.31	8.16	+1.21	7.55	0.3	8.76	0.0	0.00386704	0.02173248	A_07_P044110	Z2519	cmr|orf Unknown function [Z2519]
Z2519	+2.12	8.41	+1.08	7.87	0.1	8.95	0.1	0.00101229	0.00799671	A_07_P044110	Z2519	cmr|orf Unknown function [Z2519]
Z2519	+2.15	8.30	+1.11	7.74	0.2	8.85	0.1	0.00312712	0.01850877	A_07_P044110	Z2519	cmr|orf Unknown function [Z2519]
Z2519	+2.11	8.23	+1.08	7.69	0.1	8.77	0.1	0.00159754	0.01119846	A_07_P044110	Z2519	cmr|orf Unknown function [Z2519]
Z2561	-4.24	9.04	-2.09	10.09	0.1	8.00	0.0	0.00004539	0.00095882	A_07_P044143	Z2561	cmr|putative transposase [Z2561]
Z2664	+2.22	9.23	+1.15	8.66	0.0	9.81	0.0	0.00059272	0.00546354	A_07_P044420	Z2664	cmr|orf Unknown function [Z2664]
Z2754	+2.90	8.89	+1.54	8.12	0.4	9.66	0.1	0.00389587	0.02185785	A_07_P044434	Z2754	cmr|orf, hypothetical protein [Z2754]
Z2804	-3.86	8.99	-1.95	9.96	0.1	8.01	0.1	0.00010204	0.00159849	A_07_P044458	Z2804	cmr|unknown protein encoded within IS629 [Z2804]
Z2806	-4.36	8.42	-2.13	9.48	0.2	7.36	0.0	0.00007641	0.00131755	A_07_P044461	Z2806	cmr|putative transposase [Z2806]
Z2836	-2.22	8.57	-1.15	9.15	0.1	7.99	0.2	0.00165870	0.01150422	A_07_P044493	Z2836	cmr|orf Unknown function [Z2836]
Z3083	-2.61	12.05	-1.39	12.75	0.2	11.36	0.3	0.00371235	0.02103173	A_07_P044754	Z3083	cmr|putative tail fiber component M of prophage [Z3083]
Z3087	-3.94	11.70	-1.98	12.69	0.6	10.71	0.2	0.00678042	0.03319372	A_07_P044772	Z3087	cmr|putative tail fiber component V of prophage [Z3087]
Z3099	-2.32	11.61	-1.21	12.21	0.0	11.00	0.1	0.00071096	0.00619446	A_07_P044814	Z3099	cmr|putative DNA packaging protein of prophage CP-933U [Z3099]
Z3108	-2.85	8.20	-1.51	8.95	0.0	7.44	0.1	0.00025321	0.00301324	A_07_P044835	Z3108	cmr|unknown protein encoded within prophage CP-933U [Z3108]
Z3144	-5.79	8.75	-2.53	10.02	0.0	7.49	0.1	0.00002626	0.00072778	A_07_P044923	Z3144	cmr|orf Unknown function [Z3144]
Z3159	-7.91	8.70	-2.98	10.19	0.1	7.21	0.3	0.00006255	0.00116220	A_07_P044945	Z3159	cmr|putative outer membrane receptor for iron compound [Z3159]
Z3162	-4.07	9.38	-2.02	10.39	0.1	8.37	0.1	0.00008856	0.00145271	A_07_P044951	Z3162	cmr|IS629 transposase [Z3162]
Z3249	+2.14	7.93	+1.10	7.39	0.1	8.48	0.1	0.00139168	0.01006017	A_07_P045124	Z3249	cmr|orf, hypothetical protein [Z3249]
Z3297	-4.80	9.89	-2.26	11.02	0.0	8.76	0.1	0.00002551	0.00071967	A_07_P045211	Z3297	cmr|putative transposase for IS629 [Z3297]
Z3316	-2.55	14.24	-1.35	14.92	0.1	13.57	0.3	0.00308247	0.01827197	A_07_P045251	Z3316	cmr|unknown protein encoded within prophage CP-933V [Z3316]
Z3320	-2.26	10.61	-1.17	11.20	0.2	10.03	0.1	0.00132477	0.00972818	A_07_P045261	Z3320	cmr|unknown protein encoded within prophage CP-933V [Z3320]
Z3349	+29.49	9.06	+4.88	6.62	0.1	11.50	0.3	0.00000556	0.00035456	A_07_P045345	Z3349	cmr|putative DNA methyltransferase encoded within prophage CP-933V [Z3349]
Z3354	+8.63	13.17	+3.11	11.62	0.0	14.73	0.0	0.00000459	0.00033793	A_07_P045360	Z3354	cmr|putative exclusion protein ren of prophage CP-933V [Z3354]
Z3355	+7.14	14.70	+2.84	13.28	0.0	16.11	0.2	0.00002817	0.00074298	A_07_P045362	Z3355	cmr|putative DNA replication protein P of prophage [Z3355]
Z3356	+11.42	11.82	+3.51	10.06	0.1	13.57	0.1	0.00000431	0.00033415	A_07_P045369	Z3356	cmr|putative DNA replication protein O of prophage [Z3356]
Z3357	+4.65	9.71	+2.22	8.60	0.1	10.82	0.1	0.00004624	0.00097245	A_07_P045373	Z3357	cmr|putative regulatory protein CII of prophage CP-933V [Z3357]
Z3364	+3.96	14.02	+1.99	13.02	0.0	15.01	0.0	0.00004281	0.00093353	A_07_P045400	Z3364	cmr|putative host killing protein Kil of prophage [Z3364]
Z3365	+25.70	11.54	+4.68	9.20	0.1	13.88	0.0	0.00000091	0.00019431	A_07_P045401	Z3365	cmr|putative host-nuclease inhibitor protein Gam of prophage [Z3365]
Z3366	+6.97	15.19	+2.80	13.79	0.0	16.59	0.0	0.00000786	0.00041039	A_07_P045406	Z3366	cmr|putative recombination protein Bet of prophage CP-933V [Z3366]
Z3367	+6.78	12.28	+2.76	10.90	0.1	13.66	0.0	0.00001017	0.00046432	A_07_P045415	Z3367	cmr|putative exonuclease of prophage CP-933V [Z3367]
Z3390	-2.22	10.20	-1.15	10.78	0.3	9.62	0.1	0.00534715	0.02777476	A_07_P045451	Z3390	cmr|putative hydroxylase [Z3390]
Z3603	-2.10	9.12	-1.07	9.66	0.3	8.59	0.0	0.00458362	0.02459489	A_07_P042677	Z3603	cmr|orf, hypothetical protein [Z3603]
Z3657	-3.54	10.50	-1.82	11.41	0.1	9.59	0.3	0.00058329	0.00539033	A_07_P042781	Z3657	cmr|orf, hypothetical protein [Z3657]
Z3783	-2.85	11.86	-1.51	12.61	0.1	11.10	0.1	0.00037355	0.00394177	A_07_P042860	Z3783	cmr|putative dimethyl sulfoxide reductase subunit C [Z3783]
Z3799	-6.29	13.75	-2.65	15.07	0.0	12.42	0.1	0.00001577	0.00056283	A_07_P042878	Z3799	cmr|putative ATP synthase beta subunit [Z3799]
Z3799	-5.00	13.54	-2.32	14.70	0.0	12.38	0.0	0.00001869	0.00061299	A_07_P042878	Z3799	cmr|putative ATP synthase beta subunit [Z3799]
Z3799	-5.34	13.80	-2.42	15.01	0.0	12.60	0.0	0.00001631	0.00056947	A_07_P042878	Z3799	cmr|putative ATP synthase beta subunit [Z3799]
Z3799	-5.37	14.10	-2.43	15.31	0.1	12.89	0.0	0.00002784	0.00073912	A_07_P042878	Z3799	cmr|putative ATP synthase beta subunit [Z3799]
Z3799	-5.00	13.80	-2.32	14.96	0.1	12.64	0.0	0.00002471	0.00070600	A_07_P042878	Z3799	cmr|putative ATP synthase beta subunit [Z3799]
Z3866	+2.48	7.87	+1.31	7.21	0.0	8.52	0.1	0.00034897	0.00376574	A_07_P042932	Z3866	cmr|orf Unknown function [Z3866]
Z3923	-4.66	10.28	-2.22	11.39	0.1	9.16	0.0	0.00003497	0.00083975	A_07_P042966	Z3923	cmr|orf Unknown function [Z3923]
Z3924	-6.04	10.86	-2.59	12.16	0.0	9.56	0.1	0.00001245	0.00051423	A_07_P042971	Z3924	cmr|partial putative transposase [Z3924]
Z3925	-4.22	9.49	-2.08	10.53	0.1	8.45	0.1	0.00007106	0.00125539	A_07_P042976	Z3925	cmr|partial putative transposase [Z3925]
Z3974	+3.10	8.18	+1.63	7.36	0.3	9.00	0.1	0.00113443	0.00872521	A_07_P043090	Z3974	cmr|orf Unknown function [Z3974]
Z4065	-3.71	9.93	-1.89	10.88	0.4	8.98	0.1	0.00164220	0.01141493	A_07_P043170	Z4065	cmr|orf hypothetical protein [Z4065]
Z4105	+2.62	7.87	+1.39	7.17	0.2	8.56	0.2	0.00198500	0.01309851	A_07_P052741	Z4105	cmr|putative transport protein [Z4105]
Z4127	-2.06	9.51	-1.04	10.03	0.0	8.99	0.1	0.00121324	0.00913496	A_07_P043257	Z4127	cmr|orf, hypothetical protein [Z4127]
Z4268	+8.71	8.34	+3.12	6.78	0.3	9.90	0.2	0.00007736	0.00132538	A_07_P045673	Z4268	cmr|orf hypothetical protein [Z4268]
Z4269	+4.18	8.36	+2.06	7.33	0.1	9.39	0.0	0.00003797	0.00087581	A_07_P045678	Z4269	cmr|orf hypothetical protein [Z4269]
Z4270	+2.40	7.23	+1.26	6.60	0.1	7.86	0.1	0.00052686	0.00502151	A_07_P045685	Z4270	cmr|putative ATP-binding protein of ABC transport system [Z4270]
Z4321	-2.57	10.94	-1.36	11.62	0.2	10.26	0.2	0.00281591	0.01706455	A_07_P045744	Z4321	cmr|putative PagC-like membrane protein [Z4321]
Z4324	-2.45	8.64	-1.29	9.29	0.4	7.99	0.0	0.00642944	0.03186500	A_07_P045757	Z4324	cmr|putative transposase [Z4324]
Z4332	-2.12	9.36	-1.09	9.91	0.2	8.82	0.0	0.00301145	0.01791847	A_07_P045787	Z4332	cmr|putative cytotoxin [Z4332]
Z4340	-2.98	11.84	-1.58	12.63	0.0	11.05	0.1	0.00015657	0.00215104	A_07_P045803	Z4340	cmr|unknown protein encoded by ISEc8 [Z4340]
Z4385	-2.70	8.33	-1.43	9.05	0.0	7.62	0.0	0.00022281	0.00274273	A_07_P045844	Z4385	cmr|putative ATP-binding protein of ABC transporter family [Z4385]
Z4400	-2.62	13.23	-1.39	13.93	0.1	12.54	0.0	0.00038035	0.00397892	A_07_P045859	Z4400	cmr|orf Unknown function [Z4400]
Z4400	-2.52	13.78	-1.33	14.45	0.0	13.12	0.0	0.00029305	0.00333126	A_07_P045859	Z4400	cmr|orf Unknown function [Z4400]
Z4400	-2.64	13.50	-1.40	14.20	0.0	12.80	0.1	0.00031152	0.00345878	A_07_P045859	Z4400	cmr|orf Unknown function [Z4400]
Z4400	-2.47	13.67	-1.31	14.32	0.0	13.02	0.1	0.00049763	0.00480654	A_07_P045859	Z4400	cmr|orf Unknown function [Z4400]
Z4488	+2.01	8.21	+1.01	7.70	0.1	8.71	0.1	0.00231358	0.01471716	A_07_P045910	Z4488	cmr|putative phosphotransferase system enzyme subunit [Z4488]
Z4495	+2.29	8.04	+1.20	7.45	0.3	8.64	0.2	0.00702396	0.03412069	A_07_P045911	Z4495	cmr|putative galactosamine-6-phosphate isomerase [Z4495]
Z4501	-25.09	11.99	-4.65	14.32	0.0	9.67	0.2	0.00000144	0.00022429	A_07_P045934	Z4501	cmr|orf hypothetical protein [Z4501]
Z4854	-2.96	10.26	-1.57	11.04	0.0	9.47	0.1	0.00019627	0.00250894	A_07_P046157	Z4854	cmr|putative acyl carrier protein [Z4854]
Z4856	-2.22	7.62	-1.15	8.20	0.1	7.05	0.2	0.00172727	0.01187519	A_07_P046166	Z4856	cmr|putative enzyme [Z4856]
Z4862	+2.21	7.40	+1.14	6.83	0.0	7.97	0.1	0.00074516	0.00641085	A_07_P046200	Z4862	cmr|orf Unknown function [Z4862]
Z4863	+2.00	7.37	+1.00	6.87	0.1	7.87	0.0	0.00130413	0.00966225	A_07_P046205	Z4863	cmr|putative 3-oxoacyl- [Z4863]
Z4866	-2.06	9.10	-1.04	9.63	0.1	8.58	0.1	0.00163219	0.01137546	A_07_P046220	Z4866	cmr|putative beta-ketoacyl-ACP synthase [Z4866]
Z4878	-3.15	8.77	-1.66	9.60	0.1	7.94	0.1	0.00013363	0.00190740	A_07_P049346	Z4878	cmr|putative xylulose kinase [Z4878]
Z4950	-3.83	9.96	-1.94	10.93	0.2	8.99	0.2	0.00036745	0.00391414	A_07_P049440	Z4950	cmr|orf Unknown function [Z4950]
Z4999	-2.38	8.08	-1.25	8.70	0.1	7.45	0.1	0.00089390	0.00736840	A_07_P049484	Z4999	cmr|orf Unknown function [Z4999]
Z5522	-2.13	12.02	-1.09	12.56	0.1	11.47	0.3	0.00788718	0.03743065	A_07_P047170	Z5522	cmr|putative hippuricase [Z5522]
Z5523	-2.47	12.06	-1.30	12.72	0.1	11.41	0.1	0.00119916	0.00906366	A_07_P047173	Z5523	cmr|putative citrate permease [Z5523]
Z5618	-2.14	7.54	-1.10	8.09	0.1	6.99	0.1	0.00111754	0.00863748	A_07_P047247	Z5618	cmr|D-glucitol-6-phosphate dehydrogenase [Z5618]
Z5852	-18.99	14.23	-4.25	16.35	0.0	12.10	0.0	0.00000103	0.00020391	A_07_P047387	Z5852	cmr|orf Unknown function [Z5852]
Z5940	+2.77	7.96	+1.47	7.23	0.0	8.70	0.0	0.00017957	0.00235985	A_07_P047549	Z5940	cmr|orf Unknown function [Z5940]
Z5987	+2.39	8.58	+1.26	7.95	0.0	9.21	0.1	0.00046682	0.00461378	A_07_P050868	Z5987	cmr|orf Unknown function [Z5987]
Z5988	+4.99	8.80	+2.32	7.64	0.0	9.96	0.1	0.00003020	0.00077353	A_07_P050871	Z5988	cmr|putative lipoate-protein ligase A [Z5988]
Z5988	+6.75	8.56	+2.75	7.18	0.2	9.93	0.1	0.00005733	0.00110534	A_07_P050871	Z5988	cmr|putative lipoate-protein ligase A [Z5988]
Z5988	+4.61	8.66	+2.20	7.55	0.1	9.76	0.1	0.00005459	0.00107563	A_07_P050871	Z5988	cmr|putative lipoate-protein ligase A [Z5988]
Z5988	+5.60	8.82	+2.48	7.58	0.1	10.06	0.0	0.00001900	0.00061693	A_07_P050871	Z5988	cmr|putative lipoate-protein ligase A [Z5988]
Z5988	+4.73	8.80	+2.24	7.68	0.1	9.93	0.1	0.00003335	0.00081556	A_07_P050871	Z5988	cmr|putative lipoate-protein ligase A [Z5988]
Z6030	+6.39	8.60	+2.67	7.27	0.3	9.94	0.1	0.00010875	0.00166231	A_07_P044196	Z6030	cmr|putative tail component of cryptic prophage CP-933P [Z6030]
Z6036	-2.29	10.54	-1.19	11.13	0.0	9.94	0.0	0.00047521	0.00466588	A_07_P044215	Z6036	cmr|putative tail assembly protein of cryptic prophage [Z6036]
Z6060	-4.25	8.02	-2.09	9.06	0.4	6.98	0.0	0.00112332	0.00866511	A_07_P044262	Z6060	cmr|putative Q antiterminator encoded by prophage CP-933P [Z6060]
zapA	+2.08	11.41	+1.06	10.88	0.1	11.94	0.0	0.00098608	0.00783803	A_07_P006520	b2910	cmr|orf, hypothetical protein [b2910]
zntA	+3.08	9.39	+1.62	8.58	0.0	10.20	0.1	0.00020523	0.00259529	A_07_P034561	c_4262	cmr|Lead, cadmium, zinc and mercury transporting ATPase [c_4262]
zntR	-2.62	11.29	-1.39	11.99	0.1	10.60	0.0	0.00037081	0.00392997	A_07_P052121	Z4662	cmr|putative transcriptional regulator [Z4662]
znuC	-2.88	9.03	-1.53	9.80	0.0	8.27	0.0	0.00016756	0.00224522	A_07_P015968	b1858	cmr|putative ATP-binding component of a transport system [b1858]

E. coli ATCC 25922 ΔmarR pBK-QnrS1 (EC19) versus *E. coli* ATCC 25922 pBK-CMV

Significant differential expression (greater than or equal to two-fold, P value <0,05)

Gene Name (ProbeID)	Fold Change	logSignal (A)	logRatio (M)	logControl	StdErr (logControl)	logExperiment	StdErr (logExperiment)	pval (limma)	FDR (limma)	pval (RankProd)	FDR (RankProd)	Oligo	Systematic Name	Description
aaeA	-22.98	9.57	-4.52	11.83	0.1	7.31	0.1	0.00000025	0.00004211	0.00000889	0.00777778	A_07_P012680	b3241	cmr|putative membrane protein [b3241]
aaeB	-4.76	8.06	-2.25	9.18	0.1	6.93	0.2	0.00002685	0.00047665	0.00372586	0.20297578	A_07_P012673	b3240	cmr|orf, hypothetical protein [b3240]
aaeX	-4.49	8.06	-2.17	9.15	0.0	6.98	0.0	0.00001461	0.00031702	0.00448742	0.21873065	A_07_P010572	b3242	cmr|orf, hypothetical protein [b3242]
ABE-0285085	-3.66	8.47	-1.87	9.40	0.1	7.53	0.1	0.00006973	0.00093397	0.00973641	0.31737060	A_07_P010678	ABE-0285085	asap|IS1 transposase InsAB', C-ter fragment (pseudogene) [b4564]
ABH-0247453	-34.16	11.39	-5.09	13.94	0.0	8.84	0.1	0.00000009	0.00003427	0.00000254	0.00400000	A_07_P048654	ABH-0247453	asap|predicted protein []
ABH-0285233	-2.20	9.08	-1.14	9.65	0.1	8.51	0.4	0.00588241	0.02592801	0.05585175	0.61107019	A_07_P048532	ABH-0285233	asap|non-LEE-encoded type III secreted effector []
ABR-0081747	-5.03	12.51	-2.33	13.67	0.0	11.34	0.2	0.00001612	0.00034029	0.00301575	0.18403101	A_07_P035421	ABR-0081747	asap|toxic membrane protein []
ABR-0081751	-4.70	8.67	-2.23	9.79	0.1	7.56	0.2	0.00005011	0.00074154	0.00390180	0.20614094	A_07_P041043	ABR-0081751	asap|predicted ferredoxin []
ABR-0081753	+12.78	11.45	+3.68	9.61	0.1	13.29	0.1	0.00000077	0.00006301	0.00154789	0.11772947	A_07_P041110	ABR-0081753	asap|predicted protein []
ABR-0081754	+3.77	7.79	+1.91	6.84	0.4	8.75	0.1	0.00056011	0.00422026	0.01763211	0.35139241	A_07_P041114	ABR-0081754	asap|predicted protein []
ACA-0244204	-2.12	8.62	-1.08	9.17	0.2	8.08	0.1	0.00215976	0.01173970	0.06152439	0.63600788	A_07_P059059	ACA-0244204	asap|Hemolysin C []
ACA-0244210	-3.64	11.40	-1.86	12.33	0.1	10.46	0.3	0.00018526	0.00189276	0.01012576	0.32534694	A_07_P059088	ACA-0244210	asap|predicted protein []
ACA-0285117	-3.43	11.73	-1.78	12.62	0.2	10.84	0.1	0.00009614	0.00116257	0.01227325	0.33840630	A_07_P062850	ACA-0285117	asap|toxic polypeptide, small []
accB	+4.00	10.72	+2.00	9.72	0.1	11.72	0.2	0.00007675	0.00099210	0.01556911	0.33716644	A_07_P012745	b3255	cmr|acetylCoA carboxylase, BCCP subunit; carrier of biotin [b3255]
accC	+2.63	10.71	+1.40	10.02	0.1	11.41	0.2	0.00049052	0.00384788	0.03520325	0.47738157	A_07_P012747	b3256	cmr|acetyl CoA carboxylase, biotin carboxylase subunit [b3256]
aceA	+8.39	9.35	+3.07	7.82	0.0	10.88	0.1	0.00000227	0.00010914	0.00360264	0.17345566	A_07_P008720	b4015	cmr|isocitrate lyase [b4015]
aceA	+7.98	9.14	+3.00	7.64	0.3	10.64	0.5	0.00009455	0.00114776	0.00393102	0.18096491	A_07_P037462	c_4972	cmr|Isocitrate lyase [c_4972]
aceB	+38.05	9.85	+5.25	7.23	0.0	12.48	0.1	0.00000008	0.00003427	0.00007050	0.02265306	A_07_P008715	b4014	cmr|malate synthase A [b4014]
aceE	+47.02	11.63	+5.56	8.85	0.0	14.40	0.1	0.00000005	0.00003344	0.00002731	0.01228571	A_07_P002535	b0114	cmr|pyruvate dehydrogenase [b0114]
aceF	+10.21	11.88	+3.35	10.20	0.1	13.55	0.2	0.00000343	0.00013521	0.00251080	0.14695167	A_07_P002536	b0115	cmr|pyruvate dehydrogenase [b0115]
ackA	+6.30	12.97	+2.66	11.64	0.1	14.30	0.0	0.00000479	0.00016636	0.00640307	0.22911364	A_07_P013766	b2296	cmr|acetate kinase [b2296]
acnA	+4.95	9.45	+2.31	8.29	0.1	10.60	0.2	0.00001857	0.00037436	0.01037729	0.28072165	A_07_P014361	b1276	cmr|aconitate hydrase 1 [b1276]
acnB	+8.79	10.39	+3.14	8.82	0.0	11.96	0.1	0.00000165	0.00009590	0.00336573	0.16929712	A_07_P002554	b0118	cmr|aconitate hydrase B [b0118]
acpD	+3.67	8.69	+1.88	7.76	0.1	9.63	0.0	0.00004370	0.00066995	0.01875826	0.35928224	A_07_P052470	Z2315	cmr|acyl carrier protein phosphodiesterase [Z2315]
acpD	+3.58	9.31	+1.84	8.39	0.2	10.23	0.1	0.00007575	0.00098524	0.01962525	0.36827175	A_07_P063175	ECs2014	cmr|acyl carrier protein phosphodiesterase [ECs2014]
acpP	+2.57	16.20	+1.36	15.52	0.0	16.88	0.1	0.00025930	0.00240996	0.03660950	0.48394626	A_07_P001671	b1094	cmr|acyl carrier protein [b1094]
acpS	+4.35	8.74	+2.12	7.68	0.1	9.80	0.1	0.00003894	0.00061546	0.01372332	0.31680352	A_07_P019720	b2563	cmr|CoA:apo- [b2563]
acpS	+5.45	8.87	+2.45	7.65	0.0	10.10	0.2	0.00001266	0.00029313	0.00850737	0.25609943	A_07_P053485	ECs3429	cmr|CoA:apo- [ECs3429]
acpS	+3.75	8.79	+1.91	7.83	0.1	9.74	0.2	0.00009050	0.00111579	0.01810595	0.35455224	A_07_P042915	Z3844	cmr|CoA:apo- [Z3844]
acpT	-2.48	10.46	-1.31	11.11	0.0	9.80	0.2	0.00082641	0.00565446	0.03864456	0.53323401	A_07_P007313	b3475	cmr|orf, hypothetical protein [b3475]
acrE	-2.02	9.62	-1.01	10.13	0.0	9.11	0.2	0.00209626	0.01146076	0.07281250	0.66960280	A_07_P012792	b3265	cmr|transmembrane protein affects septum formation and cell membrane permeability [b3265]
acrF	-2.68	9.63	-1.42	10.34	0.4	8.92	0.3	0.00368600	0.01778149	0.02742696	0.47399561	A_07_P012796	b3266	cmr|integral transmembrane protein; acridine resistance [b3266]
acs	+11.92	8.70	+3.58	6.91	0.1	10.49	0.4	0.00000951	0.00024416	0.00173209	0.12283784	A_07_P052329	Z5668	cmr|acetyl-CoA synthetase [Z5668]
acs	+13.77	9.02	+3.78	7.13	0.2	10.91	0.1	0.00000204	0.00010341	0.00130208	0.11081081	A_07_P008986	b4069	cmr|acetyl-CoA synthetase [b4069]
actP	+2.88	7.86	+1.53	7.10	0.2	8.62	0.2	0.00069270	0.00489273	0.02942645	0.44249284	A_07_P063696	ECs5049	cmr|putative transport protein [ECs5049]
actP	+3.38	7.73	+1.76	6.85	0.1	8.61	0.1	0.00008779	0.00109090	0.02139482	0.38672790	A_07_P052321	Z5666	cmr|putative transport protein [Z5666]
actP	+3.06	8.23	+1.62	7.42	0.4	9.03	0.2	0.00159375	0.00924197	0.02689278	0.42767677	A_07_P008980	b4067	cmr|putative transport protein [b4067]
ade	+4.38	8.58	+2.13	7.52	0.0	9.65	0.1	0.00001771	0.00036352	0.01337081	0.31325893	A_07_P017261	b3665	cmr|probable adenine deaminase [b3665]
adhE	+3.60	8.55	+1.85	7.62	0.1	9.47	0.1	0.00005783	0.00081514	0.01941247	0.36646283	A_07_P007143	b1241	cmr|CoA-linked acetaldehyde dehydrogenase and iron-dependent alcohol dehydrogenase; pyruvate-formate-lyase deactivase [b1241]
aefA	-2.43	8.83	-1.28	9.47	0.0	8.19	0.1	0.00043708	0.00355256	0.04018547	0.54307296	A_07_P031187	c_0584	cmr|Potassium efflux system kefA [c_0584]
aes	-19.09	10.64	-4.25	12.77	0.1	8.51	0.0	0.00000029	0.00004211	0.00002223	0.01346154	A_07_P021486	b0476	cmr|putative lipase [b0476]
agaB	+2.33	7.41	+1.22	6.80	0.0	8.02	0.4	0.00518647	0.02346429	0.04667619	0.53445091	A_07_P000415	b3138	cmr|PTS system, cytoplasmic, N-acetylgalactosamine-specific IIB component 1 [b3138]
agaS	+19.45	8.98	+4.28	6.84	0.1	11.12	0.1	0.00000050	0.00005264	0.00053862	0.06784000	A_07_P034110	c_3893	cmr|Putative tagatose-6-phosphate ketose/aldose isomerase [c_3893]
agaS	+14.85	8.95	+3.89	7.01	0.1	10.90	0.1	0.00000069	0.00006127	0.00107025	0.09970414	A_07_P000404	b3136	cmr|putative tagatose-6-phosphate aldose/ketose isomerase [b3136]
agaV	+13.38	8.37	+3.74	6.50	0.1	10.24	0.4	0.00000687	0.00020075	0.00139799	0.11523560	A_07_P000390	b3133	cmr|PTS system, cytoplasmic, N-acetylgalactosamine-specific IIB component 2 [b3133]
agaW	+10.61	9.22	+3.41	7.52	0.3	10.92	0.2	0.00000668	0.00019764	0.00224403	0.13747082	A_07_P000391	b3134	cmr|PTS system N-acetylgalactosameine-specific IIC component 2 [b3134]
agp	+4.78	10.08	+2.26	8.95	0.3	11.21	0.1	0.00005168	0.00075483	0.01115600	0.28840722	A_07_P033935	c_1137	cmr|Glucose-1-phosphatase precursor [c_1137]
agp	+6.03	10.00	+2.59	8.71	0.1	11.30	0.1	0.00000897	0.00023724	0.00698933	0.23869848	A_07_P001241	b1002	cmr|periplasmic glucose-1-phosphatase [b1002]
ahpC	+4.16	15.76	+2.06	14.73	0.1	16.79	0.0	0.00002118	0.00040971	0.01469004	0.32712871	A_07_P016781	b0605	cmr|alkyl hydroperoxide reductase, C22 subunit; detoxification of hydroperoxides [b0605]
aidB	-3.49	9.13	-1.80	10.03	0.3	8.23	0.0	0.00036704	0.00311187	0.01149835	0.33277574	A_07_P011655	b4187	cmr|putative acyl coenzyme A dehydrogenase [b4187]
aldA	+5.54	8.70	+2.47	7.46	0.1	9.93	0.0	0.00000745	0.00021277	0.00823742	0.25085106	A_07_P009166	b1415	cmr|aldehyde dehydrogenase, NAD-linked [b1415]
allA	+2.71	8.84	+1.44	8.12	0.5	9.56	0.2	0.00448159	0.02087519	0.03530678	0.47755155	A_07_P016314	b0505	cmr|orf, hypothetical protein [b0505]
allB	+5.22	9.54	+2.38	8.34	0.2	10.73	0.1	0.00001849	0.00037316	0.00925368	0.26781250	A_07_P016349	b0512	cmr|putative hydrolase [b0512]
allD	+8.19	8.51	+3.03	6.99	0.0	10.03	0.1	0.00000197	0.00010186	0.00376524	0.17801802	A_07_P016372	b0517	cmr|putative malate dehydrogenase [b0517]
alr	-2.72	9.96	-1.44	10.68	0.0	9.24	0.0	0.00015799	0.00168751	0.02803163	0.47557112	A_07_P008909	b4053	cmr|alanine racemase 1 [b4053]
alsB	+18.77	9.82	+4.23	7.70	0.1	11.93	0.1	0.00000035	0.00004756	0.00058626	0.07210938	A_07_P011177	b4088	cmr|putative LACI-type transcriptional regulator [b4088]
alsC	+2.24	9.70	+1.16	9.12	0.4	10.28	0.1	0.00749382	0.03128686	0.05345465	0.56864189	A_07_P011170	b4086	cmr|putative transport system permease protein [b4086]
alsE	+2.67	10.11	+1.42	9.40	0.1	10.82	0.0	0.00021456	0.00210860	0.03380462	0.46974404	A_07_P011164	b4085	cmr|putative epimerase [b4085]
alsK	+2.56	9.48	+1.36	8.80	0.2	10.16	0.3	0.00123580	0.00761208	0.03760925	0.48694079	A_07_P011158	b4084	cmr|putative NAGC-like transcriptional regulator [b4084]
amiA	-7.62	12.09	-2.93	13.56	0.0	10.63	0.0	0.00000214	0.00010587	0.00068534	0.08174242	A_07_P042594	c_2969	cmr|Probable N-acetylmuramoyl-L-alanine amidase amiA precursor [c_2969]
amiA	-7.28	12.10	-2.86	13.53	0.0	10.67	0.0	0.00000250	0.00011564	0.00081936	0.08896552	A_07_P052703	Z3700	cmr|N-acetylmuramoyl-l-alanine amidase I [Z3700]
amiA	-6.27	11.65	-2.65	12.97	0.0	10.33	0.1	0.00000674	0.00019841	0.00140943	0.12536723	A_07_P013234	b2435	cmr|N-acetylmuramoyl-l-alanine amidase I [b2435]
amiB	-2.47	10.68	-1.30	11.33	0.1	10.02	0.1	0.00040250	0.00334584	0.03762132	0.52743544	A_07_P011563	b4169	cmr|N-acetylmuramoyl-l-alanine amidase II; a murein hydrolase [b4169]
amn	+6.98	9.13	+2.80	7.73	0.4	10.53	0.2	0.00007407	0.00096772	0.00525089	0.20823678	A_07_P015257	b1982	cmr|AMP nucleosidase [b1982]
ampD	+2.73	12.09	+1.45	11.36	0.1	12.81	0.2	0.00040718	0.00336868	0.03256733	0.46109712	A_07_P041423	c_0129	cmr|AmpD protein [c_0129]
ampD	+3.96	10.96	+1.98	9.97	0.0	11.95	0.1	0.00002787	0.00048979	0.01631669	0.34297730	A_07_P002511	b0110	cmr|regulates ampC [b0110]
ampE	+2.56	9.30	+1.36	8.62	0.0	9.97	0.1	0.00032013	0.00280126	0.03703887	0.48514143	A_07_P002518	b0111	cmr|regulates ampC [b0111]
amtB	-2.19	10.84	-1.13	11.41	0.0	10.28	0.3	0.00318791	0.01587041	0.05572980	0.61058455	A_07_P031178	c_0570	cmr|Probable ammonium transporter [c_0570]
amyA	-2.72	9.17	-1.44	9.89	0.2	8.45	0.1	0.00078279	0.00539515	0.02792492	0.47529730	A_07_P014999	b1927	cmr|cytoplasmic alpha-amylase [b1927]
anmK	+2.06	10.26	+1.04	9.74	0.1	10.78	0.0	0.00157484	0.00914241	0.05988504	0.59710576	A_07_P052526	Z2654	cmr|orf, hypothetical protein [Z2654]
anmK	+2.12	10.13	+1.08	9.59	0.1	10.67	0.1	0.00122976	0.00758080	0.05584858	0.57923584	A_07_P063211	ECs2349	cmr|hypothetical protein [ECs2349]
ansB	+40.81	12.89	+5.35	10.22	0.1	15.57	0.2	0.00000011	0.00003545	0.00005526	0.02071429	A_07_P011926	b2957	cmr|periplasmic L-asparaginase II [b2957]
apaG	-2.17	9.47	-1.12	10.03	0.1	8.91	0.2	0.00239039	0.01268674	0.05722497	0.61624487	A_07_P010939	b0050	cmr|orf, hypothetical protein [b0050]
apbA	+2.76	9.57	+1.46	8.84	0.1	10.30	0.0	0.00017638	0.00182810	0.03157393	0.45647383	A_07_P031138	c_0536	cmr|2-dehydropantoate 2-reductase [c_0536]
aphA	+4.59	8.77	+2.20	7.67	0.2	9.87	0.1	0.00003992	0.00062694	0.01223260	0.29905280	A_07_P008919	b4055	cmr|diadenosine tetraphosphatase [b4055]
aphA	+3.93	10.59	+1.98	9.60	0.1	11.58	0.1	0.00004822	0.00071961	0.01641895	0.34329349	A_07_P042001	c_5045	cmr|Class B acid phosphatase precursor [c_5045]
apt	+4.05	11.86	+2.02	10.85	0.0	12.87	0.0	0.00002125	0.00040998	0.01541667	0.33664355	A_07_P021451	b0469	cmr|adenine phosphoribosyltransferase [b0469]
araA	+8.57	8.81	+3.10	7.26	0.1	10.36	0.0	0.00000202	0.00010341	0.00348006	0.17121875	A_07_P010999	b0062	cmr|L-arabinose isomerase [b0062]
araB	+2.58	7.98	+1.37	7.29	0.0	8.66	0.3	0.00125678	0.00771115	0.03669017	0.48419950	A_07_P011001	b0063	cmr|L-ribulokinase [b0063]
araC	+2.42	11.14	+1.27	10.50	0.0	11.78	0.1	0.00052484	0.00401973	0.04166349	0.50770124	A_07_P011010	b0064	cmr|transcriptional regulator for ara operon [b0064]
araF	+2.44	8.27	+1.29	7.63	0.1	8.92	0.1	0.00055073	0.00416662	0.04054433	0.50025862	A_07_P016171	b1901	cmr|L-arabinose-binding periplasmic protein [b1901]
argE	+3.86	8.77	+1.95	7.80	0.2	9.75	0.1	0.00006300	0.00086859	0.01686420	0.34571615	A_07_P039521	c_4916	cmr|Acetylornithine deacetylase [c_4916]
argE	+3.36	7.87	+1.75	6.99	0.2	8.74	0.0	0.00012698	0.00142599	0.02151423	0.38799542	A_07_P008486	b3957	cmr|acetylornithine deacetylase [b3957]
argG	+2.45	8.07	+1.29	7.42	0.2	8.71	0.0	0.00067428	0.00481225	0.04081491	0.50007004	A_07_P000576	b3172	cmr|argininosuccinate synthetase [b3172]
argG	+2.48	8.67	+1.31	8.01	0.1	9.32	0.1	0.00042246	0.00347138	0.03963224	0.49482157	A_07_P045946	Z4534	cmr|argininosuccinate synthetase [Z4534]
argG	+2.45	8.69	+1.29	8.04	0.1	9.33	0.0	0.00035096	0.00299810	0.04055069	0.49994518	A_07_P041781	c_3929	cmr|Argininosuccinate synthase [c_3929]
argI	-2.06	9.98	-1.04	10.50	0.1	9.46	0.1	0.00181650	0.01024317	0.06816565	0.65599022	A_07_P003818	b4254	cmr|ornithine carbamoyltransferase 1 [b4254]
argR	-3.68	11.14	-1.88	12.08	0.0	10.21	0.2	0.00007539	0.00098262	0.00975419	0.31729339	A_07_P012656	b3237	cmr|repressor of arg regulon; cer-mediated site specific recombination [b3237]
ariR	+2.13	9.91	+1.09	9.36	0.2	10.45	0.1	0.00262182	0.01363658	0.05684642	0.58419713	A_07_P006784	b1166	cmr|orf, hypothetical protein [b1166]
arnC	+2.76	9.64	+1.46	8.91	0.2	10.37	0.0	0.00031384	0.00276655	0.03214558	0.45883953	A_07_P001060	b2254	cmr|putative sugar transferase [b2254]
arnF	-2.67	8.30	-1.42	9.01	0.1	7.60	0.3	0.00091094	0.00609775	0.02993585	0.48638803	A_07_P010515	b2258	cmr|putative transport/receptor protein [b2258]
arnT	+2.69	8.39	+1.43	7.67	0.2	9.10	0.1	0.00052333	0.00401527	0.03403392	0.46961437	A_07_P001072	b2257	cmr|orf, hypothetical protein [b2257]
aroA	+2.44	8.93	+1.29	8.28	0.1	9.57	0.2	0.00078560	0.00540818	0.04108295	0.50296267	A_07_P033835	c_1046	cmr|3-phosphoshikimate 1-carboxyvinyltransferase [c_1046]
aroD	+2.51	7.52	+1.33	6.86	0.1	8.19	0.0	0.00035602	0.00303253	0.03812055	0.48715097	A_07_P018517	b1693	cmr|3-dehydroquinate dehydratase [b1693]
aroG	+2.54	8.09	+1.35	7.42	0.2	8.76	0.0	0.00045177	0.00362894	0.03729040	0.48641259	A_07_P010251	b0754	cmr|3-deoxy-D-arabinoheptulosonate-7-phosphate synthase [b0754]
aroH	+2.32	9.43	+1.21	8.82	0.4	10.04	0.3	0.01297006	0.04853831	0.05324886	0.56914460	A_07_P018571	b1704	cmr|3-deoxy-D-arabinoheptulosonate-7-phosphate synthase [b1704]
aroK	-2.56	11.72	-1.36	12.39	0.0	11.04	0.1	0.00026313	0.00243694	0.03396024	0.50920952	A_07_P017976	b3390	cmr|shikimate kinase I [b3390]
arrD	+12.76	9.80	+3.67	7.96	0.1	11.63	0.1	0.00000074	0.00006247	0.00150279	0.11771144	A_07_P016536	b0555	cmr|bacteriophage lambda lysozyme homolog [b0555]
artI	+5.39	8.96	+2.43	7.75	0.1	10.18	0.2	0.00001698	0.00035267	0.00867060	0.25903226	A_07_P004766	b0863	cmr|arginine 3rd transport system periplasmic binding protein [b0863]
artP	+7.65	9.33	+2.94	7.87	0.2	10.80	0.1	0.00000750	0.00021366	0.00413872	0.18564103	A_07_P004773	b0864	cmr|ATP-binding component of 3rd arginine transport system [b0864]
artQ	+2.41	8.23	+1.27	7.59	0.2	8.87	0.1	0.00117340	0.00730198	0.04268674	0.51146119	A_07_P033780	c_0995	cmr|Arginine transport system permease protein artQ [c_0995]
asd	+4.90	9.74	+2.29	8.59	0.1	10.88	0.2	0.00002307	0.00043145	0.01059769	0.28327674	A_07_P018194	b3433	cmr|aspartate-semialdehyde dehydrogenase [b3433]
asnA	-7.40	11.20	-2.89	12.65	0.0	9.76	0.2	0.00000511	0.00017328	0.00076029	0.08673913	A_07_P005641	b3744	cmr|asparagine synthetase A [b3744]
aspA	+6.78	15.53	+2.76	14.15	0.0	16.91	0.1	0.00000457	0.00016295	0.00552782	0.21435961	A_07_P011426	b4139	cmr|aspartate ammonia-lyase [b4139]
aspC	+6.20	9.60	+2.63	8.28	0.2	10.91	0.3	0.00002887	0.00050012	0.00666921	0.23230088	A_07_P052047	Z1275	cmr|aspartate aminotransferase [Z1275]
aspC	+9.42	11.20	+3.24	9.58	0.1	12.82	0.1	0.00000237	0.00011227	0.00290904	0.15631399	A_07_P005088	b0928	cmr|aspartate aminotransferase [b0928]
asr	-7.25	10.30	-2.86	11.73	0.0	8.87	0.2	0.00000880	0.00023401	0.00081364	0.08958042	A_07_P020616	b1597	cmr|acid shock protein [b1597]
astA	+4.40	8.58	+2.14	7.51	0.0	9.64	0.2	0.00005468	0.00078482	0.01328506	0.31171386	A_07_P018783	b1747	cmr|orf, hypothetical protein [b1747]
atoC	+3.42	8.56	+1.77	7.67	0.2	9.44	0.0	0.00010509	0.00124498	0.02133384	0.38651323	A_07_P000888	b2220	cmr|response regulator of ato, ornithine decarboxylase antizyme [b2220]
atoS	+2.52	9.15	+1.33	8.48	0.1	9.81	0.0	0.00027150	0.00249826	0.03804688	0.48660439	A_07_P000883	b2219	cmr|sensor protein AtoS for response regulator AtoC [b2219]
atpA	+4.43	15.25	+2.15	14.18	0.0	16.32	0.1	0.00001621	0.00034168	0.01302337	0.30786787	A_07_P005591	b3734	cmr|membrane-bound ATP synthase, F1 sector, alpha-subunit [b3734]
atpB	+2.29	12.56	+1.19	11.96	0.0	13.15	0.1	0.00070636	0.00496691	0.04692391	0.53495293	A_07_P005614	b3738	cmr|membrane-bound ATP synthase, F0 sector, subunit a [b3738]
atpD	+4.42	11.51	+2.14	10.44	0.0	12.58	0.1	0.00002036	0.00039679	0.01308498	0.30839820	A_07_P005582	b3732	cmr|membrane-bound ATP synthase, F1 sector, beta-subunit [b3732]
atpE	+5.00	12.71	+2.32	11.55	0.0	13.87	0.2	0.00002599	0.00046542	0.01005399	0.27673077	A_07_P005607	b3737	cmr|membrane-bound ATP synthase, F0 sector, subunit c [b3737]
atpG	+5.72	12.40	+2.52	11.14	0.0	13.66	0.3	0.00002725	0.00048212	0.00772612	0.24623482	A_07_P005587	b3733	cmr|membrane-bound ATP synthase, F1 sector, gamma-subunit [b3733]
atpH	+9.02	13.18	+3.17	11.60	0.1	14.77	0.1	0.00000193	0.00010107	0.00319741	0.16559211	A_07_P005596	b3735	cmr|membrane-bound ATP synthase, F1 sector, delta-subunit [b3735]
azoR	+2.90	8.96	+1.54	8.19	0.2	9.73	0.2	0.00042576	0.00349110	0.02904726	0.44057803	A_07_P009153	b1412	cmr|acyl carrier protein phosphodiesterase [b1412]
b0255	-2.54	10.98	-1.34	11.65	0.2	10.31	0.0	0.00052323	0.00401527	0.03547891	0.51577101	A_07_P007981	b0255	cmr|IS911 hypothetical protein, variant [b0255]
b0257	-2.72	15.79	-1.44	16.51	0.0	15.07	0.1	0.00025491	0.00238178	0.02762005	0.47524590	A_07_P007992	b0257	cmr|putative transposase [b0257]
b0322	-5.51	9.20	-2.46	10.43	0.1	7.96	0.1	0.00001156	0.00027749	0.00227261	0.15831858	A_07_P008305	b0322	cmr|orf, hypothetical protein [b0322]
b0359	-2.31	8.58	-1.21	9.18	0.3	7.97	0.1	0.00213642	0.01164156	0.04744538	0.57681853	A_07_P020924	b0359	cmr|putative transferase [b0359]
b0359	-2.21	7.81	-1.14	8.38	0.1	7.24	0.1	0.00111960	0.00707343	0.05482914	0.60833686	A_07_P010289	b0359	cmr|putative transferase [b0359]
b0362	-2.14	7.13	-1.10	7.68	0.3	6.58	0.1	0.00310301	0.01553432	0.05892658	0.62348118	A_07_P020932	b0362	cmr|orf, hypothetical protein [b0362]
b0374	-6.91	8.20	-2.79	9.60	0.1	6.81	0.1	0.00000411	0.00015128	0.00100927	0.10121019	A_07_P020983	b0374	cmr|putative flagellin structural protein [b0374]
b0395	+2.20	7.44	+1.14	6.87	0.0	8.01	0.0	0.00066382	0.00475922	0.05072980	0.55852448	A_07_P021086	b0395	cmr|orf, hypothetical protein [b0395]
b0501	-2.55	9.23	-1.35	9.90	0.5	8.56	0.0	0.00560317	0.02495386	0.03376842	0.50826960	A_07_P016291	b0501	cmr|orf, hypothetical protein [b0501]
b0663	-2.19	7.53	-1.13	8.09	0.0	6.96	0.4	0.00682664	0.02911120	0.05415777	0.60515259	A_07_P009880	b0663	cmr|putative RNA [b0663]
b0671	-4.12	7.96	-2.04	8.98	0.0	6.94	0.2	0.00003803	0.00060518	0.00620300	0.25767810	A_07_P009885	b0671	cmr|putative RNA [b0671]
b0725	+11.48	8.67	+3.52	6.91	0.0	10.43	0.4	0.00001790	0.00036590	0.00186547	0.12938326	A_07_P010142	b0725	cmr|orf, hypothetical protein [b0725]
b1170	-3.72	8.14	-1.89	9.09	0.1	7.19	0.2	0.00008955	0.00110664	0.00901931	0.30537634	A_07_P006805	b1170	cmr|putative part of putative ATP-binding component of a transport system [b1170]
b1364	-2.28	10.02	-1.19	10.62	0.0	9.43	0.0	0.00050192	0.00391007	0.04930259	0.58538462	A_07_P014798	b1364	cmr|orf, hypothetical protein [b1364]
b1543	-2.52	7.43	-1.33	8.09	0.0	6.76	0.2	0.00055653	0.00420042	0.03496888	0.51118849	A_07_P020361	b1543	cmr|putative transport protein [b1543]
b1995	-4.11	8.32	-2.04	9.34	0.0	7.30	0.1	0.00002553	0.00046151	0.00630907	0.25733161	A_07_P015310	b1995	cmr|orf, hypothetical protein [b1995]
b2680	-2.13	7.80	-1.09	8.34	0.2	7.25	0.1	0.00338037	0.01658994	0.05928417	0.62684352	A_07_P001871	b2680	cmr|orf, hypothetical protein [b2680]
b3000	-2.17	7.77	-1.12	8.33	0.2	7.21	0.1	0.00232079	0.01240276	0.05753874	0.61667120	A_07_P012133	b3000	cmr|orf, hypothetical protein [b3000]
b3016	-2.58	9.42	-1.37	10.11	0.0	8.74	0.0	0.00022417	0.00217051	0.03279980	0.50577865	A_07_P012215	b3016	cmr|orf, hypothetical protein [b3016]
b3111	+30.92	10.28	+4.95	7.80	0.0	12.75	0.1	0.00000011	0.00003548	0.00012894	0.03274194	A_07_P000282	b3111	cmr|putative L-serine dehydratase [b3111]
b3112	+21.78	9.54	+4.44	7.31	0.1	11.76	0.1	0.00000027	0.00004211	0.00037983	0.05588785	A_07_P000290	b3112	cmr|putative L-serine dehydratase [b3112]
b3436	-2.01	7.38	-1.01	7.88	0.1	6.88	0.1	0.00225257	0.01211220	0.07350610	0.67205575	A_07_P018209	b3436	cmr|low-affinity gluconate transport permease protein, interrupted [b3436]
b3695	+2.35	8.19	+1.23	7.57	0.1	8.81	0.2	0.00114620	0.00718671	0.04469957	0.52323420	A_07_P005410	b3695	cmr|regulator protein for dgo operon [b3695]
b3762	+2.40	7.80	+1.26	7.17	0.1	8.44	0.2	0.00076000	0.00527345	0.04244411	0.51049656	A_07_P005705	b3762	cmr|orf, hypothetical protein [b3762]
b3763	+2.89	8.60	+1.53	7.83	0.0	9.37	0.2	0.00033969	0.00292453	0.02939723	0.44247610	A_07_P005708	b3763	cmr|regulator of pssA [b3763]
b3814	+2.06	9.28	+1.04	8.76	0.1	9.80	0.1	0.00135218	0.00813168	0.05996824	0.59717900	A_07_P005938	b3814	cmr|orf, hypothetical protein [b3814]
b3837	-2.86	12.15	-1.51	12.91	0.0	11.40	0.1	0.00013328	0.00148186	0.02378303	0.44470309	A_07_P018994	b3837	cmr|putative histone [b3837]
b3841	-3.00	10.25	-1.58	11.05	0.0	9.46	0.1	0.00010142	0.00121246	0.02001778	0.40665806	A_07_P019013	b3841	cmr|orf, hypothetical protein [b3841]
b3914	-2.11	12.29	-1.07	12.83	0.0	11.76	0.1	0.00116466	0.00727060	0.06327109	0.64433376	A_07_P019349	b3914	cmr|orf, hypothetical protein [b3914]
b4285	-4.76	14.79	-2.25	15.92	0.0	13.67	0.2	0.00002895	0.00050086	0.00383575	0.20402027	A_07_P003953	b4285	cmr|putative transposase [b4285]
basR	+2.49	9.81	+1.32	9.15	0.1	10.47	0.0	0.00044064	0.00356882	0.03898247	0.49296386	A_07_P037777	c_5118	cmr|Transcriptional Regulatory protein basR/pmrA [c_5118]
basS	-3.73	12.36	-1.90	13.31	0.5	11.41	0.4	0.00254753	0.01335163	0.00958905	0.31386694	A_07_P011296	b4112	cmr|sensor protein for basR [b4112]
bax	-8.14	12.01	-3.02	13.53	0.0	10.50	0.0	0.00000177	0.00009825	0.00052210	0.07210526	A_07_P007778	b3570	cmr|putative ATP-binding protein [b3570]
bcp	+12.99	10.13	+3.70	8.28	0.1	11.98	0.3	0.00000271	0.00012064	0.00143229	0.11505102	A_07_P013456	b2480	cmr|bacterioferritin comigratory protein [b2480]
bcsA	-2.37	8.85	-1.25	9.48	0.1	8.23	0.2	0.00070282	0.00494641	0.04290460	0.55871795	A_07_P007596	b3533	cmr|putative cellulose synthase [b3533]
bcsF	-2.16	8.24	-1.11	8.80	0.1	7.69	0.1	0.00090114	0.00605586	0.05860836	0.62304524	A_07_P007618	b3537	cmr|orf, hypothetical protein [b3537]
betA	+3.38	9.28	+1.76	8.41	0.0	10.16	0.1	0.00008598	0.00108038	0.02164126	0.38806378	A_07_P043676	Z0398	cmr|choline dehydrogenase, a flavoprotein [Z0398]
betA	+5.19	9.89	+2.38	8.70	0.2	11.08	0.1	0.00003470	0.00056726	0.00941883	0.26864130	A_07_P008247	b0311	cmr|choline dehydrogenase, a flavoprotein [b0311]
betA	+4.95	10.47	+2.31	9.31	0.2	11.62	0.1	0.00002784	0.00048967	0.01040015	0.28037671	A_07_P030984	c_0431	cmr|Choline dehydrogenase [c_0431]
betB	+2.79	12.11	+1.48	11.37	0.1	12.85	0.0	0.00015409	0.00165376	0.03074568	0.44903525	A_07_P008251	b0312	cmr|NAD+-dependent betaine aldehyde dehydrogenase [b0312]
betT	-2.72	10.97	-1.44	11.69	0.4	10.25	0.0	0.00175767	0.00998653	0.02735963	0.47387239	A_07_P051906	Z0401	cmr|high-affinity choline transport [Z0401]
bfr	-2.38	9.58	-1.25	10.21	0.1	8.96	0.0	0.00045092	0.00362761	0.04319995	0.55932566	A_07_P017709	b3336	cmr|bacterioferrin, an iron storage homoprotein [b3336]
bglJ	-2.03	7.89	-1.02	8.40	0.1	7.38	0.0	0.00124532	0.00765274	0.07175940	0.66732428	A_07_P017526	b4366	cmr|2-component transcriptional regulator [b4366]
bhsA	-21.35	10.52	-4.42	12.73	0.1	8.31	0.2	0.00000049	0.00005264	0.00001651	0.01300000	A_07_P001765	b1112	cmr|orf, hypothetical protein [b1112]
birA	-4.20	10.91	-2.07	11.94	0.0	9.87	0.1	0.00002185	0.00041549	0.00585938	0.25136240	A_07_P008550	b3973	cmr|biotin- [b3973]
bolA	+14.34	10.05	+3.84	8.13	0.0	11.98	0.0	0.00000040	0.00004930	0.00116679	0.10497143	A_07_P021288	b0435	cmr|possible regulator of murein genes [b0435]
bssS	-9.90	13.15	-3.31	14.80	0.0	11.49	0.1	0.00000134	0.00008557	0.00021977	0.04739726	A_07_P001504	b1060	cmr|orf, hypothetical protein [b1060]
btuB	-6.14	11.19	-2.62	12.49	0.0	9.88	0.5	0.00019524	0.00196595	0.00150597	0.12679144	A_07_P008533	b3966	cmr|outer membrane receptor for transport of vitamin B12, E colicins, and bacteriophage BF23 [b3966]
btuE	+2.18	9.05	+1.13	8.48	0.1	9.61	0.1	0.00099628	0.00650846	0.05235772	0.56421629	A_07_P044422	Z2739	cmr|vitamin B12 transport [Z2739]
btuE	+2.47	8.96	+1.30	8.31	0.1	9.62	0.1	0.00050733	0.00393662	0.03991679	0.49719146	A_07_P044422	Z2739	cmr|vitamin B12 transport [Z2739]
btuE	-2.60	10.63	-1.38	11.31	0.1	9.94	0.3	0.00108716	0.00692964	0.03133003	0.49623742	A_07_P035160	c_2106	cmr|Vitamin B12 transport periplasmic protein btuE [c_2106]
c_0036	+3.33	8.96	+1.74	8.10	0.2	9.83	0.1	0.00012296	0.00139175	0.02203316	0.39329932	A_07_P035658	c_0036	cmr|Hypothetical protein [c_0036]
c_0059	-2.30	11.81	-1.20	12.41	0.1	11.21	0.1	0.00087529	0.00591949	0.04761369	0.57663846	A_07_P035683	c_0059	cmr|Putative antitoxin of gyrase inhibiting toxin-antitoxin system [c_0059]
c_0062	-2.73	7.76	-1.45	8.48	0.1	7.03	0.1	0.00024126	0.00228540	0.02708206	0.47270510	A_07_P035698	c_0062	cmr|Hypothetical protein [c_0062]
c_0086	-2.50	10.51	-1.32	11.17	0.2	9.84	0.4	0.00394398	0.01878789	0.03470719	0.51307981	A_07_P035735	c_0086	cmr|Transposase [c_0086]
c_0088	-3.43	8.98	-1.78	9.87	0.1	8.09	0.1	0.00007290	0.00095734	0.01216527	0.33839223	A_07_P035738	c_0088	cmr|Hypothetical protein [c_0088]
c_0100	-2.44	11.39	-1.28	12.03	0.0	10.75	0.5	0.00618393	0.02696945	0.03980818	0.54029310	A_07_P041416	c_0100	cmr|S-adenosyl-methyltransferase mraW [c_0100]
c_0120	-5.23	9.78	-2.39	10.97	0.0	8.59	0.2	0.00002764	0.00048679	0.00267276	0.16967742	A_07_P035766	c_0120	cmr|Hypothetical protein [c_0120]
c_0138	-3.61	9.01	-1.85	9.94	0.0	8.09	0.2	0.00006491	0.00088731	0.01037729	0.32224852	A_07_P035783	c_0138	cmr|Unknown protein of IS629 encoded within prophage [c_0138]
c_0163	-2.02	9.74	-1.01	10.24	0.1	9.23	0.2	0.00303078	0.01527901	0.07138974	0.66664294	A_07_P035811	c_0163	cmr|Hypothetical protein [c_0163]
c_0192	-2.02	7.58	-1.01	8.08	0.0	7.07	0.1	0.00182749	0.01029029	0.07292175	0.66904429	A_07_P035893	c_0192	cmr|Hypothetical protein [c_0192]
c_0226	-2.83	9.21	-1.50	9.96	0.1	8.47	0.1	0.00016170	0.00171435	0.02430323	0.44962397	A_07_P035924	c_0226	cmr|Putative cell cycle protein mesJ [c_0226]
c_0253	-2.49	8.63	-1.32	9.28	0.0	7.97	0.0	0.00027668	0.00253932	0.03706682	0.52480216	A_07_P035949	c_0253	cmr|Hypothetical protein [c_0253]
c_0256	-2.78	7.98	-1.47	8.72	0.1	7.25	0.0	0.00018424	0.00188477	0.02624111	0.46735294	A_07_P035961	c_0256	cmr|Hypothetical protein [c_0256]
c_0257	-7.52	10.56	-2.91	12.02	0.0	9.11	0.1	0.00000321	0.00013128	0.00072218	0.08299270	A_07_P035967	c_0257	cmr|Unknown in ISEc8 [c_0257]
c_0258	-4.31	11.20	-2.11	12.25	0.2	10.14	0.2	0.00005496	0.00078754	0.00558435	0.24354571	A_07_P035972	c_0258	cmr|Unknown in ISEc8 [c_0258]
c_0260	-2.96	7.69	-1.57	8.47	0.1	6.90	0.1	0.00017223	0.00179690	0.02099784	0.41636020	A_07_P035978	c_0260	cmr|Hypothetical protein [c_0260]
c_0261	-2.23	7.85	-1.16	8.43	0.0	7.27	0.0	0.00056367	0.00423782	0.05310340	0.59846815	A_07_P035987	c_0261	cmr|Hypothetical protein [c_0261]
c_0264	-3.43	8.02	-1.78	8.91	0.0	7.13	0.0	0.00004644	0.00069738	0.01219639	0.33746924	A_07_P036004	c_0264	cmr|Hypothetical protein [c_0264]
c_0274	-2.53	8.24	-1.34	8.91	0.1	7.57	0.0	0.00030480	0.00271268	0.03498158	0.51089981	A_07_P036041	c_0274	cmr|Hypothetical protein [c_0274]
c_0277	-2.08	7.47	-1.05	8.00	0.0	6.94	0.0	0.00100507	0.00654555	0.06666413	0.64988235	A_07_P036056	c_0277	cmr|Hypothetical protein [c_0277]
c_0279	-3.49	7.75	-1.80	8.65	0.2	6.85	0.1	0.00015065	0.00162457	0.01164888	0.33467153	A_07_P036068	c_0279	cmr|Hypothetical protein ykfF [c_0279]
c_0280	-2.65	10.32	-1.40	11.02	0.4	9.62	0.1	0.00172723	0.00984915	0.02991997	0.48663223	A_07_P036072	c_0280	cmr|Hypothetical protein [c_0280]
c_0281	-2.83	10.32	-1.50	11.07	0.0	9.57	0.3	0.00048711	0.00383450	0.02360010	0.44286055	A_07_P036079	c_0281	cmr|Hypothetical protein [c_0281]
c_0284	-3.58	9.80	-1.84	10.72	0.1	8.88	0.1	0.00005273	0.00076604	0.01069931	0.32456647	A_07_P036095	c_0284	cmr|Conserved hypothetical protein [c_0284]
c_0286	-4.61	9.72	-2.20	10.82	0.1	8.62	0.1	0.00002215	0.00041939	0.00416984	0.21177419	A_07_P036101	c_0286	cmr|Conserved hypothetical protein [c_0286]
c_0287	-6.35	12.32	-2.67	13.65	0.1	10.98	0.0	0.00000553	0.00017942	0.00138402	0.12522989	A_07_P036106	c_0287	cmr|Hypothetical protein [c_0287]
c_0289	-4.47	8.40	-2.16	9.48	0.1	7.32	0.1	0.00002464	0.00045104	0.00462589	0.22272171	A_07_P036114	c_0289	cmr|Hypothetical protein [c_0289]
c_0297	-2.08	10.37	-1.05	10.90	0.3	9.85	0.1	0.00552769	0.02470981	0.06644499	0.65178193	A_07_P036146	c_0297	cmr|Hypothetical protein [c_0297]
c_0301	-2.43	7.55	-1.28	8.18	0.0	6.91	0.1	0.00044254	0.00358038	0.04041349	0.54335611	A_07_P036159	c_0301	cmr|Hypothetical protein [c_0301]
c_0304	-2.31	7.60	-1.21	8.21	0.1	7.00	0.1	0.00071095	0.00498360	0.04688707	0.57581123	A_07_P036164	c_0304	cmr|Hypothetical protein [c_0304]
c_0305	-4.56	9.71	-2.19	10.80	0.3	8.61	0.5	0.00093184	0.00618762	0.00453506	0.22037037	A_07_P036170	c_0305	cmr|Hypothetical protein [c_0305]
c_0309	-2.75	8.38	-1.46	9.11	0.1	7.66	0.0	0.00018821	0.00191593	0.02711446	0.47222345	A_07_P036181	c_0309	cmr|Conserved hypothetical protein [c_0309]
c_0310	-2.21	8.11	-1.15	8.68	0.2	7.54	0.2	0.00264500	0.01373901	0.05425432	0.60537208	A_07_P036189	c_0310	cmr|Hypothetical protein [c_0310]
c_0313	-4.30	12.67	-2.10	13.72	0.3	11.61	0.3	0.00018503	0.00189159	0.00572218	0.24886740	A_07_P036201	c_0313	cmr|Hypothetical protein [c_0313]
c_0315	-3.25	10.37	-1.70	11.22	0.2	9.52	0.0	0.00020045	0.00200247	0.01505018	0.36908100	A_07_P036212	c_0315	cmr|Hypothetical protein [c_0315]
c_0316	-2.51	8.68	-1.33	9.34	0.1	8.02	0.3	0.00131852	0.00797966	0.03669207	0.52278733	A_07_P036219	c_0316	cmr|Hypothetical protein [c_0316]
c_0319	+2.86	8.93	+1.52	8.17	0.1	9.68	0.0	0.00013868	0.00152367	0.02953697	0.44373092	A_07_P036230	c_0319	cmr|Putative oligogalacturonide lyase [c_0319]
c_0328	-2.92	8.13	-1.55	8.91	0.0	7.36	0.1	0.00017324	0.00180391	0.02153138	0.42215442	A_07_P036267	c_0328	cmr|Hypothetical protein [c_0328]
c_0331	+4.01	8.60	+2.00	7.60	0.0	9.60	0.1	0.00002381	0.00044188	0.01586319	0.33933424	A_07_P036277	c_0331	cmr|Putative ribokinase [c_0331]
c_0332	+2.13	9.16	+1.09	8.61	0.0	9.70	0.2	0.00202682	0.01116916	0.05452807	0.57118430	A_07_P030713	c_0332	cmr|Putative L-fucose permease [c_0332]
c_0337	-7.17	10.57	-2.84	11.99	0.0	9.15	0.1	0.00000355	0.00013789	0.00085366	0.09142857	A_07_P030738	c_0337	cmr|Putative conserved protein [c_0337]
c_0339	-4.21	9.07	-2.07	10.10	0.1	8.03	0.1	0.00002571	0.00046319	0.00584286	0.25133880	A_07_P030741	c_0339	cmr|Hypothetical protein [c_0339]
c_0340	-3.21	8.66	-1.68	9.51	0.0	7.82	0.2	0.00015661	0.00167393	0.01541159	0.37271889	A_07_P030749	c_0340	cmr|Conserved hypothetical protein [c_0340]
c_0348	-2.52	9.12	-1.34	9.79	0.0	8.45	0.0	0.00025679	0.00239370	0.03552020	0.51541935	A_07_P030761	c_0348	cmr|Hypothetical protein [c_0348]
c_0349	-3.84	7.91	-1.94	8.88	0.2	6.93	0.3	0.00024648	0.00232227	0.00842607	0.29878378	A_07_P030767	c_0349	cmr|Putative Transposase within prophage [c_0349]
c_0352	-5.51	9.67	-2.46	10.90	0.1	8.44	0.1	0.00000834	0.00022675	0.00222370	0.15699552	A_07_P030781	c_0352	cmr|Partial Transposase [c_0352]
c_0355	-5.06	9.08	-2.34	10.25	0.2	7.91	0.2	0.00005434	0.00078198	0.00318534	0.19214559	A_07_P030788	c_0355	cmr|Hypothetical protein [c_0355]
c_0357	-2.86	8.09	-1.52	8.85	0.1	7.33	0.3	0.00085615	0.00581755	0.02242251	0.43368550	A_07_P030794	c_0357	cmr|Hypothetical protein [c_0357]
c_0358	-2.80	8.47	-1.49	9.21	0.1	7.73	0.1	0.00023864	0.00227020	0.02486090	0.45512791	A_07_P030798	c_0358	cmr|Hypothetical protein [c_0358]
c_0361	-2.65	12.28	-1.41	12.98	0.3	11.57	0.0	0.00107460	0.00687464	0.02999555	0.48685567	A_07_P030810	c_0361	cmr|Putative cytoplasmic membrane export protein [c_0361]
c_0365	-4.37	8.31	-2.13	9.37	0.1	7.24	0.1	0.00002131	0.00041024	0.00506669	0.23054913	A_07_P030828	c_0365	cmr|Hypothetical protein [c_0365]
c_0385	-3.39	8.31	-1.76	9.19	0.0	7.43	0.0	0.00005502	0.00078754	0.01290968	0.34332770	A_07_P030859	c_0385	cmr|Hypothetical protein [c_0385]
c_0393	-2.21	7.98	-1.14	8.55	0.2	7.41	0.1	0.00156232	0.00908314	0.05506733	0.60883427	A_07_P030878	c_0393	cmr|Haemoglobin protease [c_0393]
c_0396	-2.07	7.77	-1.05	8.29	0.0	7.24	0.1	0.00117271	0.00730198	0.06704395	0.65116595	A_07_P030886	c_0396	cmr|Insertion element IS1 1/2/3/5/6 protein insA [c_0396]
c_0397	-3.28	7.87	-1.71	8.73	0.0	7.02	0.0	0.00006032	0.00084197	0.01437881	0.35990461	A_07_P030892	c_0397	cmr|InsB protein [c_0397]
c_0410	+5.09	8.81	+2.35	7.64	0.0	9.99	0.0	0.00000859	0.00022953	0.00969830	0.27120782	A_07_P030916	c_0410	cmr|Hypothetical protein ycjY [c_0410]
c_0413	+3.30	7.51	+1.72	6.65	0.2	8.37	0.4	0.00118945	0.00738206	0.02354294	0.40911700	A_07_P030931	c_0413	cmr|Putative aldo/keto reductase [c_0413]
c_0414	+5.59	8.66	+2.48	7.42	0.0	9.91	0.2	0.00001298	0.00029667	0.00811230	0.24945312	A_07_P030940	c_0414	cmr|2,5-diketo-D-gluconic acid reductase A [c_0414]
c_0427	-2.80	8.23	-1.49	8.97	0.3	7.49	0.2	0.00186327	0.01045079	0.02455602	0.45164720	A_07_P030962	c_0427	cmr|Conserved hypothetical protein [c_0427]
c_0429	-2.37	9.17	-1.25	9.79	0.0	8.55	0.0	0.00037936	0.00319396	0.04368585	0.56054605	A_07_P030975	c_0429	cmr|Conserved hypothetical protein [c_0429]
c_0467	-2.27	8.58	-1.18	9.17	0.1	7.99	0.0	0.00067174	0.00479850	0.05040015	0.58647450	A_07_P031049	c_0467	cmr|Hypothetical protein yaiO [c_0467]
c_0481	-2.67	8.82	-1.41	9.52	0.0	8.11	0.1	0.00020382	0.00202457	0.02941374	0.48138254	A_07_P031085	c_0481	cmr|Hypothetical protein [c_0481]
c_0487	-3.05	11.70	-1.61	12.51	0.0	10.90	0.0	0.00009069	0.00111721	0.01873666	0.39437166	A_07_P041502	c_0487	cmr|D-alanine--D-alanine ligase A [c_0487]
c_0503	+2.38	9.40	+1.25	8.77	0.1	10.02	0.1	0.00082600	0.00565414	0.04257241	0.51047982	A_07_P031097	c_0503	cmr|Hypothetical protein yajF [c_0503]
c_0550	+2.32	7.26	+1.21	6.65	0.1	7.86	0.1	0.00060068	0.00443370	0.04538364	0.52849112	A_07_P031150	c_0550	cmr|Hypothetical protein [c_0550]
c_0583	-4.16	10.48	-2.06	11.51	0.1	9.45	0.1	0.00003293	0.00054976	0.00607342	0.25430851	A_07_P031182	c_0583	cmr|Hypothetical protein [c_0583]
c_0608	-3.15	10.41	-1.66	11.24	0.1	9.58	0.3	0.00059751	0.00442070	0.01697917	0.38025605	A_07_P031211	c_0608	cmr|Hypothetical protein [c_0608]
c_0608	-2.25	9.73	-1.17	10.32	0.3	9.15	0.1	0.00222208	0.01199330	0.05124936	0.59198092	A_07_P031211	c_0608	cmr|Hypothetical protein [c_0608]
c_0638	-2.35	8.43	-1.24	9.05	0.1	7.82	0.1	0.00092723	0.00617004	0.04334604	0.55846154	A_07_P031250	c_0638	cmr|Hypothetical protein [c_0638]
c_0640	+2.79	7.84	+1.48	7.10	0.1	8.58	0.2	0.00034627	0.00296932	0.03103277	0.45071956	A_07_P031257	c_0640	cmr|Conserved hypothetical protein [c_0640]
c_0697	-3.19	8.68	-1.67	9.52	0.1	7.85	0.2	0.00016621	0.00175269	0.01571646	0.37377644	A_07_P031357	c_0697	cmr|Conserved hypothetical protein [c_0697]
c_0719	-2.56	8.09	-1.36	8.77	0.1	7.41	0.1	0.00028991	0.00262169	0.03331428	0.50676329	A_07_P033287	c_0719	cmr|Conserved hypothetical protein [c_0719]
c_0761	+2.45	7.64	+1.29	6.99	0.0	8.28	0.0	0.00031134	0.00275220	0.04068153	0.50077404	A_07_P033336	c_0761	cmr|Putative dihydrodipicolinate synthase [c_0761]
c_0778	+37.34	9.34	+5.22	6.73	0.0	11.95	0.1	0.00000006	0.00003344	0.00007431	0.02340000	A_07_P033361	c_0778	cmr|Hypothetical protein [c_0778]
c_0784	-6.07	8.62	-2.60	9.92	0.0	7.31	0.3	0.00002007	0.00039347	0.00157965	0.12886010	A_07_P033375	c_0784	cmr|Hypothetical protein [c_0784]
c_0795	+2.06	7.63	+1.04	7.11	0.0	8.15	0.0	0.00108489	0.00692357	0.05897866	0.59370844	A_07_P033384	c_0795	cmr|Putative conserved protein [c_0795]
c_0813	-2.27	8.19	-1.18	8.78	0.4	7.59	0.2	0.00820557	0.03364282	0.05014355	0.58695911	A_07_P033406	c_0813	cmr|Hypothetical protein [c_0813]
c_0897	+24.35	13.44	+4.61	11.14	0.1	15.74	0.4	0.00000264	0.00011947	0.00025978	0.04595506	A_07_P033506	c_0897	cmr|Hypothetical protein [c_0897]
c_0938	-2.73	7.70	-1.45	8.42	0.3	6.97	0.0	0.00125915	0.00772048	0.02683498	0.47100334	A_07_P033552	c_0938	cmr|Hypothetical protein [c_0938]
c_0940	-2.33	7.63	-1.22	8.24	0.0	7.02	0.1	0.00056673	0.00425291	0.04630843	0.57317610	A_07_P033562	c_0940	cmr|Hypothetical protein ybiI [c_0940]
c_0947	-2.54	11.84	-1.35	12.51	0.2	11.17	0.3	0.00165643	0.00952130	0.03368839	0.50755024	A_07_P033599	c_0947	cmr|Probable capsid portal protein [c_0947]
c_1005	-2.46	7.84	-1.30	8.49	0.1	7.19	0.1	0.00051927	0.00399580	0.03902503	0.53473455	A_07_P041077	c_1005	cmr|NADH oxidoreductase hcr [c_1005]
c_1020	-2.71	9.18	-1.44	9.90	0.1	8.46	0.0	0.00019111	0.00193740	0.02758511	0.47568456	A_07_P033805	c_1020	cmr|Hypothetical protein [c_1020]
c_1094	-2.35	10.72	-1.23	11.33	0.3	10.10	0.0	0.00160038	0.00927702	0.04361852	0.56059592	A_07_P033878	c_1094	cmr|Hypothetical protein [c_1094]
c_1111	-2.71	9.54	-1.44	10.26	0.3	8.82	0.1	0.00100463	0.00654555	0.02764672	0.47466739	A_07_P033904	c_1111	cmr|Hypothetical protein [c_1111]
c_1138	+2.66	9.26	+1.41	8.56	0.3	9.97	0.1	0.00166841	0.00957964	0.03534489	0.47724700	A_07_P033937	c_1138	cmr|Hypothetical protein [c_1138]
c_1166	+5.44	9.21	+2.44	7.99	0.2	10.43	0.1	0.00002390	0.00044210	0.00861725	0.25841905	A_07_P033974	c_1166	cmr|Conserved hypothetical protein [c_1166]
c_1172	-4.54	8.08	-2.18	9.17	0.1	6.99	0.1	0.00002339	0.00043630	0.00440930	0.21761755	A_07_P034010	c_1172	cmr|Hypothetical protein [c_1172]
c_1173	-6.18	11.14	-2.63	12.46	0.1	9.83	0.5	0.00017393	0.00180865	0.00147485	0.12551351	A_07_P034004	c_1173	cmr|Hypothetical protein [c_1173]
c_1174	-3.47	9.81	-1.80	10.70	0.2	8.91	0.3	0.00042433	0.00348135	0.01215257	0.33863717	A_07_P040226	c_1174	cmr|Hypothetical protein [c_1174]
c_1174	-3.15	9.67	-1.65	10.50	0.1	8.84	0.2	0.00022394	0.00216966	0.01685785	0.37969957	A_07_P040226	c_1174	cmr|Hypothetical protein [c_1174]
c_1174	-2.21	9.96	-1.14	10.53	0.2	9.39	0.4	0.00728257	0.03058331	0.05183943	0.59099203	A_07_P040226	c_1174	cmr|Hypothetical protein [c_1174]
c_1174	-2.42	9.90	-1.27	10.54	0.2	9.27	0.3	0.00439179	0.02054201	0.03869792	0.53303587	A_07_P040226	c_1174	cmr|Hypothetical protein [c_1174]
c_1175	+5.89	8.15	+2.56	6.87	0.1	9.43	0.2	0.00000996	0.00025049	0.00714685	0.24145923	A_07_P040232	c_1175	cmr|Putative aminotransferase [c_1175]
c_1186	-2.29	9.00	-1.19	9.60	0.1	8.41	0.0	0.00059276	0.00438964	0.04907711	0.58447050	A_07_P040258	c_1186	cmr|Putative beta-ketoacyl-ACP synthase [c_1186]

c_1187	+3.06	7.17	+1.61	6.36	0.4	7.98	0.0	0.00080370	0.00552067	0.02663173	0.42741081	A_07_P040261	c_1187	cmr|3-oxoacyl- [c_1187]
c_1189	+2.03	7.61	+1.02	7.10	0.0	8.12	0.1	0.00142754	0.00844932	0.06099149	0.60128366	A_07_P040271	c_1189	cmr|Putative 3-oxoacyl- [c_1189]
c_1190	+2.69	7.72	+1.43	7.01	0.0	8.44	0.1	0.00022453	0.00217267	0.03303100	0.46556849	A_07_P040280	c_1190	cmr|Conserved hypothetical protein [c_1190]
c_1198	-2.42	8.81	-1.27	9.45	0.0	8.18	0.1	0.00041749	0.00343417	0.04053227	0.54356048	A_07_P040314	c_1198	cmr|Conserved hypothetical protein [c_1198]
c_1199	-3.52	10.50	-1.81	11.41	0.0	9.59	0.3	0.00032643	0.00283787	0.01158981	0.33419414	A_07_P040317	c_1199	cmr|Putative acyl carrier protein [c_1199]
c_1202	-2.35	7.66	-1.24	8.28	0.0	7.05	0.1	0.00060450	0.00445358	0.04425749	0.56238095	A_07_P040334	c_1202	cmr|Conserved hypothetical protein [c_1202]
c_1203	-2.20	9.70	-1.14	10.27	0.1	9.13	0.2	0.00134273	0.00809032	0.05487170	0.60795215	A_07_P040338	c_1203	cmr|Putative O-methyltransferase [c_1203]
c_1204	-2.39	10.13	-1.26	10.76	0.1	9.50	0.1	0.00060308	0.00444514	0.04185722	0.54962469	A_07_P040344	c_1204	cmr|Conserved hypothetical protein [c_1204]
c_1204	-3.25	10.37	-1.70	11.22	0.0	9.52	0.1	0.00008818	0.00109403	0.01506733	0.36892691	A_07_P040344	c_1204	cmr|Conserved hypothetical protein [c_1204]
c_1204	-2.52	10.41	-1.33	11.07	0.1	9.74	0.1	0.00043739	0.00355327	0.03520262	0.51270120	A_07_P040344	c_1204	cmr|Conserved hypothetical protein [c_1204]
c_1204	-3.60	9.98	-1.85	10.90	0.1	9.05	0.3	0.00021800	0.00213179	0.01049035	0.32320939	A_07_P040344	c_1204	cmr|Conserved hypothetical protein [c_1204]
c_1204	-3.24	10.19	-1.69	11.04	0.1	9.34	0.0	0.00008226	0.00104105	0.01516387	0.36956656	A_07_P040344	c_1204	cmr|Conserved hypothetical protein [c_1204]
c_1206	-2.14	9.06	-1.10	9.61	0.0	8.51	0.2	0.00146660	0.00862539	0.05980501	0.62855140	A_07_P040351	c_1206	cmr|Hypothetical protein [c_1206]
c_1209	-2.56	9.55	-1.36	10.23	0.2	8.87	0.2	0.00117909	0.00733158	0.03263910	0.50627586	A_07_P040366	c_1209	cmr|Hypothetical protein [c_1209]
c_1214	+2.95	12.45	+1.56	11.67	0.1	13.23	0.0	0.00011486	0.00132092	0.02767658	0.43314115	A_07_P040383	c_1214	cmr|Cea protein [c_1214]
c_1215	-4.52	12.58	-2.18	13.67	0.0	11.49	0.0	0.00001356	0.00030459	0.00445185	0.21767081	A_07_P040390	c_1215	cmr|Entry exclusion protein 2 [c_1215]
c_1219	-2.53	8.21	-1.34	8.88	0.0	7.54	0.2	0.00045928	0.00367611	0.03612424	0.51750682	A_07_P040407	c_1219	cmr|Putative Transposase [c_1219]
c_1220	-3.07	8.93	-1.62	9.74	0.2	8.12	0.1	0.00032254	0.00281803	0.01770389	0.38658807	A_07_P040413	c_1220	cmr|Phospho-2-dehydro-3-deoxyheptonate aldolase, Trp-sensitive [c_1220]
c_1225	-2.46	10.08	-1.30	10.73	0.1	9.43	0.1	0.00054184	0.00411349	0.03768674	0.52741333	A_07_P040430	c_1225	cmr|Transposase insE for insertion sequence IS3A/B/C/D/E/fA/fB [c_1225]
c_1235	-11.05	8.78	-3.47	10.51	0.0	7.04	0.1	0.00000101	0.00007407	0.00012513	0.03862745	A_07_P040469	c_1235	cmr|Conserved hypothetical protein [c_1235]
c_1236	-7.06	8.88	-2.82	10.29	0.0	7.47	0.0	0.00000273	0.00012064	0.00097307	0.10013072	A_07_P040474	c_1236	cmr|Conserved hypothetical protein [c_1236]
c_1249	-2.70	7.87	-1.43	8.58	0.1	7.15	0.1	0.00030899	0.00273608	0.02850165	0.47839019	A_07_P040540	c_1249	cmr|Hypothetical protein [c_1249]
c_1253	-4.66	9.61	-2.22	10.72	0.1	8.49	0.2	0.00003365	0.00055720	0.00404535	0.20813725	A_07_P040560	c_1253	cmr|ATP binding cassette [c_1253]
c_1257	-23.00	11.03	-4.52	13.29	0.1	8.77	0.1	0.00000022	0.00004002	0.00000921	0.00878788	A_07_P040573	c_1257	cmr|Putative conserved protein [c_1257]
c_1258	-2.96	7.57	-1.57	8.35	0.4	6.78	0.4	0.00361801	0.01758082	0.02067200	0.41302030	A_07_P040577	c_1258	cmr|Hypothetical protein [c_1258]
c_1267	-3.44	8.50	-1.78	9.39	0.5	7.61	0.4	0.00460613	0.02137938	0.01185658	0.33694946	A_07_P040614	c_1267	cmr|Hypothetical protein [c_1267]
c_1268	-2.75	8.25	-1.46	8.98	0.0	7.52	0.0	0.00015475	0.00165961	0.02664444	0.46975364	A_07_P040616	c_1268	cmr|Hypothetical protein [c_1268]
c_1271	-2.03	8.61	-1.02	9.12	0.1	8.10	0.1	0.00148001	0.00869077	0.07131987	0.66638576	A_07_P040631	c_1271	cmr|Hypothetical protein [c_1271]
c_1272	-4.68	8.02	-2.23	9.13	0.1	6.90	0.0	0.00001406	0.00031050	0.00395135	0.20736667	A_07_P040636	c_1272	cmr|Hypothetical protein yeeP [c_1272]
c_1273	+2.75	11.16	+1.46	10.43	0.1	11.89	0.1	0.00023131	0.00222327	0.03210747	0.45912807	A_07_P040643	c_1273	cmr|Antigen 43 precursor [c_1273]
c_1284	-5.17	11.07	-2.37	12.26	0.2	9.89	0.0	0.00002188	0.00041549	0.00276994	0.17444000	A_07_P040687	c_1284	cmr|Hypothetical protein yeeT [c_1284]
c_1286	-7.64	9.98	-2.93	11.45	0.0	8.51	0.2	0.00000488	0.00016794	0.00063961	0.07992063	A_07_P040696	c_1286	cmr|Hypothetical protein [c_1286]
c_1294	-3.93	7.65	-1.97	8.64	0.0	6.66	0.1	0.00002816	0.00049161	0.00740218	0.27550827	A_07_P040732	c_1294	cmr|Hypothetical protein [c_1294]
c_1323	+2.05	7.81	+1.04	7.29	0.1	8.32	0.1	0.00121835	0.00753406	0.06002731	0.59663510	A_07_P040769	c_1323	cmr|Cytochrome b561 homolog 2 [c_1323]
c_1337	-2.65	7.64	-1.41	8.34	0.0	6.94	0.0	0.00019136	0.00193876	0.02981263	0.48538780	A_07_P040785	c_1337	cmr|Hypothetical protein [c_1337]
c_1406	-3.06	8.35	-1.62	9.16	0.1	7.55	0.1	0.00010397	0.00123441	0.01831809	0.38867925	A_07_P040862	c_1406	cmr|Hypothetical protein [c_1406]
c_1413	-2.13	10.13	-1.09	10.68	0.2	9.59	0.2	0.00378201	0.01815367	0.06169906	0.63531066	A_07_P040889	c_1413	cmr|Unknown protein encoded within prophage [c_1413]
c_1413	-3.01	10.02	-1.59	10.82	0.1	9.23	0.1	0.00012169	0.00137842	0.01963097	0.40191157	A_07_P040889	c_1413	cmr|Unknown protein encoded within prophage [c_1413]
c_1413	-2.33	10.11	-1.22	10.72	0.3	9.50	0.1	0.00161992	0.00936961	0.04660506	0.57458888	A_07_P040889	c_1413	cmr|Unknown protein encoded within prophage [c_1413]
c_1417	-2.37	9.30	-1.25	9.92	0.4	8.67	0.1	0.00514141	0.02328724	0.04317772	0.55949794	A_07_P040905	c_1417	cmr|Hypothetical protein [c_1417]
c_1421	-12.97	9.49	-3.70	11.34	0.1	7.64	0.0	0.00000065	0.00005973	0.00005653	0.02282051	A_07_P040925	c_1421	cmr|Hypothetical protein [c_1421]
c_1436	-2.74	8.77	-1.45	9.50	0.2	8.04	0.0	0.00049589	0.00387652	0.02710938	0.47265781	A_07_P042220	c_1436	cmr|Probable lysozyme from lambdoid prophage Qin [c_1436]
c_1444	-2.32	10.95	-1.21	11.56	0.2	10.34	0.1	0.00106656	0.00683435	0.04648247	0.57397647	A_07_P041004	c_1444	cmr|Prophage Qin DNA packaging protein NU1 homolog [c_1444]
c_1445	+2.19	10.55	+1.13	9.98	0.2	11.11	0.1	0.00167289	0.00959142	0.05271977	0.56540872	A_07_P041010	c_1445	cmr|Putative DNA packaging protein of prophage terminase [c_1445]
c_1446	+2.35	10.70	+1.23	10.09	0.3	11.32	0.0	0.00304880	0.01533556	0.04514863	0.52653333	A_07_P041015	c_1446	cmr|Putative DNA packaging protein of prophage [c_1446]
c_1448	+16.87	11.09	+4.08	9.05	0.1	13.13	0.2	0.00000064	0.00005892	0.00077299	0.08278912	A_07_P041021	c_1448	cmr|Putative capsid assembly protein of prophage [c_1448]
c_1453	+9.76	11.28	+3.29	9.64	0.1	12.93	0.1	0.00000208	0.00010447	0.00272612	0.15328571	A_07_P038108	c_1453	cmr|Putative head-tail joining protein of prophage [c_1453]
c_1457	-2.39	8.87	-1.26	9.50	0.5	8.24	0.2	0.00963082	0.03810697	0.04156822	0.54857502	A_07_P038133	c_1457	cmr|Hypothetical protein [c_1457]
c_1484	-2.70	8.98	-1.43	9.70	0.4	8.26	0.2	0.00217741	0.01181128	0.02720655	0.47278146	A_07_P038229	c_1484	cmr|Unknown protein encoded by prophage [c_1484]
c_1487	-2.72	10.26	-1.44	10.98	0.1	9.54	0.0	0.00023244	0.00223011	0.02765879	0.47435730	A_07_P038235	c_1487	cmr|Hypothetical protein [c_1487]
c_1496	-2.34	9.52	-1.23	10.13	0.5	8.90	0.0	0.00827161	0.03384310	0.04448107	0.56385668	A_07_P038279	c_1496	cmr|Unknown protein encoded by prophage [c_1496]
c_1501	+2.81	8.91	+1.49	8.17	0.0	9.66	0.1	0.00018131	0.00186421	0.03066057	0.44945996	A_07_P038305	c_1501	cmr|Hypothetical protein [c_1501]
c_1506	-2.98	8.04	-1.58	8.83	0.0	7.25	0.2	0.00018609	0.00190000	0.02009972	0.40779639	A_07_P038330	c_1506	cmr|Unknown protein encoded by prophage [c_1506]
c_1514	-2.35	12.13	-1.24	12.75	0.0	11.52	0.2	0.00110264	0.00699719	0.04430132	0.56203062	A_07_P038334	c_1514	cmr|Hypothetical protein [c_1514]
c_1519	+11.52	8.78	+3.53	7.02	0.3	10.54	0.2	0.00001253	0.00029182	0.00186547	0.12881579	A_07_P038347	c_1519	cmr|Prophage lambda integrase [c_1519]
c_1522	+49.53	10.48	+5.63	7.66	0.4	13.30	0.3	0.00000116	0.00007956	0.00001842	0.00935484	A_07_P038354	c_1522	cmr|Hypothetical protein [c_1522]
c_1523	+45.54	12.01	+5.51	9.26	0.1	14.77	0.2	0.00000009	0.00003427	0.00003303	0.01405405	A_07_P035459	c_1523	cmr|Unknown protein encoded by bacteriophage [c_1523]
c_1524	+25.47	10.45	+4.67	8.12	0.0	12.79	0.8	0.00003460	0.00056633	0.00021087	0.04000000	A_07_P038365	c_1524	cmr|Hypothetical protein [c_1524]
c_1525	+37.38	12.05	+5.22	9.44	0.0	14.66	0.2	0.00000022	0.00003960	0.00006098	0.02181818	A_07_P038369	c_1525	cmr|Hypothetical protein [c_1525]
c_1526	+47.56	10.55	+5.57	7.76	0.1	13.33	0.2	0.00000014	0.00003548	0.00002414	0.01187500	A_07_P038374	c_1526	cmr|Hypothetical protein [c_1526]
c_1527	+30.29	10.11	+4.92	7.65	0.3	12.57	0.1	0.00000084	0.00006592	0.00013148	0.03234375	A_07_P038377	c_1527	cmr|Hypothetical protein [c_1527]
c_1528	+37.02	10.26	+5.21	7.65	0.3	12.86	0.9	0.00004461	0.00067949	0.00006098	0.02133333	A_07_P038381	c_1528	cmr|Hypothetical protein [c_1528]
c_1531	+71.59	11.14	+6.16	8.06	0.0	14.22	0.2	0.00000006	0.00003344	0.00000635	0.00666667	A_07_P038395	c_1531	cmr|Hypothetical protein [c_1531]
c_1532	+18.33	11.09	+4.20	8.99	0.3	13.19	0.1	0.00000171	0.00009748	0.00065358	0.07566176	A_07_P038400	c_1532	cmr|Unknown protein encoded within prophage [c_1532]
c_1533	+2.08	12.34	+1.06	11.81	0.3	12.87	0.1	0.00387837	0.01854039	0.05993140	0.59718987	A_07_P038402	c_1533	cmr|Hypothetical protein [c_1533]
c_1536	+3.08	13.79	+1.62	12.98	0.1	14.60	0.2	0.00015546	0.00166500	0.02576855	0.42437238	A_07_P038406	c_1536	cmr|Putative recombination protein Bet of prophage [c_1536]
c_1538	+20.85	12.87	+4.38	10.67	0.0	15.06	0.1	0.00000027	0.00004211	0.00043445	0.06107143	A_07_P038419	c_1538	cmr|Hypothetical protein [c_1538]
c_1540	+4.72	14.52	+2.24	13.40	0.1	15.64	0.0	0.00001264	0.00029313	0.01157203	0.29290997	A_07_P038423	c_1540	cmr|Lambda Regulatory protein CIII [c_1540]
c_1541	+8.94	13.82	+3.16	12.24	0.0	15.41	0.5	0.00005442	0.00078251	0.00319614	0.16662252	A_07_P038427	c_1541	cmr|Putative single-stranded DNA binding protein of prophage [c_1541]
c_1542	-2.71	15.17	-1.44	15.89	0.0	14.45	0.1	0.00018152	0.00186421	0.02827426	0.47558761	A_07_P038431	c_1542	cmr|Lambda ant-restriction protein [c_1542]
c_1546	-4.02	12.42	-2.01	13.42	0.0	11.41	0.1	0.00003119	0.00053034	0.00700330	0.26762136	A_07_P038451	c_1546	cmr|Repressor protein [c_1546]
c_1547	+4.32	12.87	+2.11	11.82	0.1	13.93	0.1	0.00002376	0.00044169	0.01368839	0.31646109	A_07_P038456	c_1547	cmr|Hypothetical protein [c_1547]
c_1548	+2.73	12.03	+1.45	11.30	0.2	12.75	0.3	0.00130654	0.00793298	0.03314914	0.46639857	A_07_P038465	c_1548	cmr|Putative Regulatory protein CII of bacteriophage [c_1548]
c_1549	+6.95	13.27	+2.80	11.87	0.0	14.67	0.0	0.00000285	0.00012365	0.00521913	0.20855330	A_07_P038470	c_1549	cmr|Putative replication protein O of bacteriophage [c_1549]
c_1550	+4.05	14.64	+2.02	13.63	0.1	15.65	0.2	0.00005542	0.00079029	0.01558244	0.33699176	A_07_P038471	c_1550	cmr|Putative replication protein P of bacteriophage [c_1550]
c_1554	+41.30	10.62	+5.37	7.93	0.2	13.30	0.1	0.00000012	0.00003548	0.00005399	0.02073171	A_07_P038485	c_1554	cmr|Unknown protein encoded within prophage [c_1554]
c_1555	+23.29	9.62	+4.54	7.35	0.1	11.89	0.3	0.00000068	0.00006127	0.00031949	0.05239583	A_07_P038490	c_1555	cmr|Putative DNA N-6-adenine-methyltransferase of bacteriophage [c_1555]
c_1556	+100.86	10.61	+6.66	7.28	0.2	13.94	0.1	0.00000003	0.00003344	0.00000064	0.00125000	A_07_P038492	c_1556	cmr|Hypothetical protein [c_1556]
c_1565	+3.99	12.51	+2.00	11.51	0.2	13.51	0.3	0.00024458	0.00230877	0.01545351	0.33604972	A_07_P038514	c_1565	cmr|Partial tonB-like membrane protein encoded within prophage [c_1565]
c_1567	+4.74	9.98	+2.25	8.86	0.1	11.10	0.3	0.00009809	0.00118435	0.01105945	0.28732673	A_07_P038518	c_1567	cmr|Hypothetical protein [c_1567]
c_1570	+2.93	10.99	+1.55	10.21	0.4	11.76	0.0	0.00153853	0.00896468	0.02936484	0.44325983	A_07_P038532	c_1570	cmr|Putative DNA packaging protein of prophage [c_1570]
c_1571	+7.02	9.51	+2.81	8.10	0.0	10.91	0.1	0.00000326	0.00013195	0.00517467	0.20836317	A_07_P038538	c_1571	cmr|Putative capsid protein of prophage [c_1571]
c_1572	+42.15	10.55	+5.40	7.86	0.1	13.25	0.2	0.00000016	0.00003548	0.00004764	0.01973684	A_07_P038541	c_1572	cmr|Putative capsid assembly protein of prophage [c_1572]
c_1574	+18.06	10.41	+4.17	8.32	0.1	12.50	0.2	0.00000062	0.00005819	0.00063199	0.07481203	A_07_P038551	c_1574	cmr|Putative capsid protein of prophage [c_1574]
c_1575	+17.97	9.42	+4.17	7.34	0.2	11.51	0.3	0.00000248	0.00011555	0.00064533	0.07582090	A_07_P038556	c_1575	cmr|Putative capsid protein of prophage [c_1575]
c_1576	+11.05	9.38	+3.47	7.65	0.2	11.11	0.1	0.00000395	0.00014758	0.00202553	0.13177686	A_07_P038564	c_1576	cmr|Hypothetical protein [c_1576]
c_1577	+12.51	8.85	+3.65	7.03	0.1	10.68	0.2	0.00000158	0.00009392	0.00153709	0.11804878	A_07_P038569	c_1577	cmr|Putative head-tail joining protein of prophage [c_1577]
c_1579	+8.11	11.06	+3.02	9.55	0.1	12.57	0.3	0.00001003	0.00025069	0.00375127	0.17842900	A_07_P038580	c_1579	cmr|Putative tail component of prophage [c_1579]
c_1580	+37.55	9.64	+5.23	7.03	0.0	12.26	0.2	0.00000015	0.00003548	0.00007685	0.02372549	A_07_P038581	c_1580	cmr|Tail protein [c_1580]
c_1583	+8.95	9.47	+3.16	7.89	0.0	11.05	0.1	0.00000175	0.00009825	0.00322980	0.16672131	A_07_P038594	c_1583	cmr|Putative tail component of prophage [c_1583]
c_1585	+3.11	9.71	+1.64	8.89	0.2	10.53	0.2	0.00048931	0.00384271	0.02537221	0.42315678	A_07_P038605	c_1585	cmr|Putative tail component of prophage [c_1585]
c_1587	+2.49	9.62	+1.32	8.96	0.1	10.28	0.1	0.00061776	0.00452296	0.03910696	0.49334936	A_07_P038615	c_1587	cmr|Putative tail component of prophage [c_1587]
c_1590	+2.12	9.51	+1.08	8.97	0.1	10.06	0.1	0.00143197	0.00846284	0.05630081	0.58200919	A_07_P038626	c_1590	cmr|Putative tail component of prophage [c_1590]
c_1591	+2.28	9.31	+1.19	8.72	0.3	9.90	0.1	0.00334406	0.01643911	0.04963415	0.55500000	A_07_P038637	c_1591	cmr|Hypothetical protein [c_1591]
c_1593	+3.12	7.99	+1.64	7.17	0.2	8.81	0.2	0.00031656	0.00278278	0.02523183	0.42126193	A_07_P038645	c_1593	cmr|Hypothetical protein [c_1593]
c_1596	-3.17	8.87	-1.66	9.70	0.0	8.04	0.2	0.00012656	0.00142224	0.01622586	0.37789941	A_07_P038656	c_1596	cmr|Hypothetical protein [c_1596]
c_1605	-4.16	11.49	-2.06	12.52	0.1	10.47	0.0	0.00002668	0.00047513	0.00608105	0.25395225	A_07_P038687	c_1605	cmr|Hypothetical protein [c_1605]
c_1611	-2.22	8.36	-1.15	8.93	0.3	7.78	0.1	0.00301130	0.01521009	0.05223069	0.59330447	A_07_P038708	c_1611	cmr|Hypothetical protein [c_1611]
c_1640	-4.00	11.85	-2.00	12.85	0.1	10.85	0.1	0.00005581	0.00079521	0.00699441	0.26793187	A_07_P038726	c_1640	cmr|Putative Na [c_1640]
c_1657	+5.23	8.38	+2.39	7.19	0.1	9.58	0.1	0.00001317	0.00029928	0.00919970	0.26822222	A_07_P038777	c_1657	cmr|Protein ycgS [c_1657]
c_1660	-3.62	9.45	-1.85	10.38	0.0	8.53	0.2	0.00011571	0.00132585	0.01034045	0.32365805	A_07_P038788	c_1660	cmr|Hypothetical protein [c_1660]
c_1714	-6.19	10.94	-2.63	12.25	0.1	9.62	0.1	0.00000833	0.00022675	0.00152376	0.12760638	A_07_P038863	c_1714	cmr|Hypothetical protein [c_1714]
c_1755	+2.21	8.98	+1.14	8.41	0.0	9.55	0.0	0.00060575	0.00445856	0.05038554	0.55864085	A_07_P038912	c_1755	cmr|Conserved hypothetical protein [c_1755]
c_1766	-2.33	8.65	-1.22	9.26	0.4	8.04	0.2	0.00587739	0.02592801	0.04396913	0.56234768	A_07_P034731	c_1766	cmr|Putative membrane transport protein [c_1766]
c_1800	+2.51	8.75	+1.33	8.09	0.1	9.42	0.1	0.00034988	0.00299377	0.03848831	0.48907183	A_07_P034765	c_1800	cmr|Conserved hypothetical protein [c_1800]
c_1812	-3.41	9.49	-1.77	10.38	0.2	8.61	0.2	0.00020074	0.00200374	0.01278519	0.34232993	A_07_P034785	c_1812	cmr|Hypothetical protein [c_1812]
c_1819	+2.49	8.91	+1.32	8.25	0.2	9.57	0.2	0.00087652	0.00592275	0.03952680	0.49428912	A_07_P034790	c_1819	cmr|Putative conserved protein [c_1819]
c_1825	+5.12	10.43	+2.36	9.25	0.3	11.60	0.1	0.00004019	0.00062962	0.00965638	0.27099822	A_07_P034801	c_1825	cmr|Hypothetical protein [c_1825]
c_1935	-3.84	8.68	-1.94	9.65	0.2	7.71	0.3	0.00019392	0.00195589	0.00839050	0.29819413	A_07_P034987	c_1935	cmr|Chaperone protein fimC precursor [c_1935]
c_1956	-4.17	10.91	-2.06	11.94	0.2	9.88	0.1	0.00010381	0.00123350	0.00629192	0.25864230	A_07_P035043	c_1956	cmr|Putative outer membrane protein yieC precursor [c_1956]
c_1989	-9.66	10.59	-3.27	12.23	0.1	8.95	0.1	0.00000187	0.00009959	0.00022929	0.04878378	A_07_P035078	c_1989	cmr|Putative acid shock protein [c_1989]
c_2059	+2.01	9.26	+1.00	8.76	0.1	9.76	0.1	0.00274436	0.01410089	0.06430831	0.61886919	A_07_P035140	c_2059	cmr|Protein ydhR precursor [c_2059]
c_2148	+4.02	8.24	+2.01	7.24	0.4	9.24	0.2	0.00050248	0.00391058	0.01544398	0.33677285	A_07_P035198	c_2148	cmr|Succinylornithine transaminase [c_2148]
c_2155	+4.66	9.39	+2.22	8.28	0.2	10.50	0.1	0.00003590	0.00058029	0.01174733	0.29450637	A_07_P042371	c_2155	cmr|Protein ynjB [c_2155]
c_2158	+9.34	9.95	+3.22	8.34	0.0	11.56	0.1	0.00000134	0.00008557	0.00296557	0.15720539	A_07_P041171	c_2158	cmr|Putative thiosulfate sulfurtransferase ynjE precursor [c_2158]
c_2225	-2.11	7.62	-1.08	8.16	0.2	7.08	0.3	0.00556420	0.02483072	0.06394309	0.64574727	A_07_P035300	c_2225	cmr|Hypothetical protein [c_2225]
c_2255	-20.20	10.18	-4.34	12.35	0.0	8.01	0.1	0.00000019	0.00003706	0.00001651	0.01238095	A_07_P035318	c_2255	cmr|Hypothetical protein [c_2255]
c_2273	-2.31	9.94	-1.21	10.54	0.0	9.33	0.0	0.00048258	0.00381030	0.04759019	0.57724191	A_07_P035336	c_2273	cmr|High-affinity zinc uptake system membrane protein znuB [c_2273]
c_2348	+30.03	9.90	+4.91	7.45	0.4	12.36	0.3	0.00000413	0.00015132	0.00013211	0.03200000	A_07_P035389	c_2348	cmr|Outer membrane porin protein nmpC precursor [c_2348]
c_2405	+2.26	8.03	+1.18	7.44	0.1	8.62	0.1	0.00093493	0.00620521	0.04854611	0.54750000	A_07_P031475	c_2405	cmr|Hypothetical protein [c_2405]
c_2409	+2.73	7.81	+1.45	7.09	0.1	8.54	0.0	0.00017747	0.00183705	0.03250889	0.46151488	A_07_P031493	c_2409	cmr|Hypothetical protein [c_2409]
c_2412	-3.78	9.16	-1.92	10.12	0.4	8.20	0.1	0.00034373	0.00295415	0.00871507	0.30222467	A_07_P031509	c_2412	cmr|Hypothetical protein [c_2412]
c_2418	-3.32	8.95	-1.73	9.82	0.3	8.09	0.2	0.00039369	0.00329173	0.01338161	0.34765677	A_07_P031531	c_2418	cmr|Prophage P4 integrase [c_2418]
c_2423	-2.32	7.93	-1.21	8.54	0.1	7.33	0.0	0.00064284	0.00464471	0.04700775	0.57505051	A_07_P031557	c_2423	cmr|Putataive AraC type regulator [c_2423]
c_2450	+2.90	12.01	+1.53	11.24	0.0	12.77	0.1	0.00015643	0.00167317	0.02872269	0.43946550	A_07_P031648	c_2450	cmr|Hypothetical protein [c_2450]
c_2451	+3.16	10.58	+1.66	9.75	0.0	11.41	0.1	0.00008787	0.00109097	0.02422066	0.41314193	A_07_P031651	c_2451	cmr|Putative thioesterase [c_2451]
c_2454	+3.87	8.55	+1.95	7.57	0.0	9.52	0.1	0.00003748	0.00059842	0.01700394	0.34677461	A_07_P031667	c_2454	cmr|Hypothetical protein [c_2454]
c_2455	-4.46	12.89	-2.16	13.96	0.1	11.81	0.2	0.00003130	0.00053044	0.00488122	0.22804154	A_07_P031671	c_2455	cmr|Putative peptide synthetase [c_2455]
c_2458	-2.86	9.61	-1.52	10.37	0.1	8.85	0.1	0.00015379	0.00165162	0.02339304	0.44160671	A_07_P031686	c_2458	cmr|Putative peptide synthetase [c_2458]
c_2460	+6.80	7.92	+2.76	6.54	0.2	9.31	0.1	0.00000817	0.00022493	0.00539825	0.21089330	A_07_P031697	c_2460	cmr|Putative polyketide synthase [c_2460]
c_2461	+4.69	8.78	+2.23	7.67	0.1	9.90	0.1	0.00001959	0.00038789	0.01173844	0.29475279	A_07_P031704	c_2461	cmr|Hypothetical protein [c_2461]
c_2463	+2.61	8.18	+1.39	7.49	0.1	8.87	0.1	0.00043918	0.00356045	0.03575203	0.47863946	A_07_P031708	c_2463	cmr|Putative transacylase [c_2463]
c_2466	+2.59	9.29	+1.37	8.60	0.4	9.97	0.2	0.00333375	0.01641745	0.03717099	0.48646717	A_07_P031725	c_2466	cmr|Hypothetical protein [c_2466]
c_2467	+3.40	9.54	+1.76	8.66	0.1	10.42	0.0	0.00006399	0.00087841	0.02122523	0.38498848	A_07_P031728	c_2467	cmr|Putative 3-hydroxyacyl-CoA dehydrogenase [c_2467]
c_2467	+2.39	9.72	+1.26	9.09	0.4	10.35	0.0	0.00341103	0.01672999	0.04467988	0.52339286	A_07_P031728	c_2467	cmr|Putative 3-hydroxyacyl-CoA dehydrogenase [c_2467]
c_2467	+2.93	9.83	+1.55	9.06	0.3	10.61	0.3	0.00174661	0.00994168	0.02936484	0.44283525	A_07_P031728	c_2467	cmr|Putative 3-hydroxyacyl-CoA dehydrogenase [c_2467]
c_2467	+2.50	9.60	+1.32	8.94	0.3	10.26	0.0	0.00103688	0.00669591	0.03944995	0.49490040	A_07_P031728	c_2467	cmr|Putative 3-hydroxyacyl-CoA dehydrogenase [c_2467]
c_2467	+3.10	9.91	+1.63	9.09	0.4	10.72	0.1	0.00103523	0.00669352	0.02627096	0.42684211	A_07_P031728	c_2467	cmr|Putative 3-hydroxyacyl-CoA dehydrogenase [c_2467]
c_2468	+4.26	8.24	+2.09	7.20	0.1	9.29	0.2	0.00003656	0.00058730	0.01414190	0.32174855	A_07_P031733	c_2468	cmr|Putative polyketide synthase [c_2468]
c_2470	+3.07	8.98	+1.62	8.17	0.2	9.79	0.0	0.00016819	0.00176652	0.02577807	0.42408568	A_07_P031741	c_2470	cmr|Putative peptide/polyketide synthase [c_2470]
c_2481	-6.22	8.07	-2.64	9.39	0.0	6.75	0.2	0.00000912	0.00023883	0.00148310	0.12553763	A_07_P031773	c_2481	cmr|Hypothetical protein [c_2481]
c_2482	-6.86	8.34	-2.78	9.73	0.1	6.95	0.0	0.00000334	0.00013377	0.00104548	0.10287500	A_07_P031779	c_2482	cmr|Putative outer membrane receptor for iron compound [c_2482]
c_2484	-5.20	11.62	-2.38	12.80	0.0	10.43	0.1	0.00000858	0.00022953	0.00268483	0.16975904	A_07_P031782	c_2484	cmr|Hypothetical protein ybdM [c_2484]
c_2485	-2.08	10.61	-1.06	11.14	0.1	10.08	0.2	0.00269736	0.01391457	0.06613885	0.65121326	A_07_P031787	c_2485	cmr|Hypothetical protein ybdN [c_2485]
c_2489	-2.40	7.84	-1.26	8.47	0.1	7.21	0.1	0.00059604	0.00441187	0.04160633	0.54861809	A_07_P031799	c_2489	cmr|Putative transferase [c_2489]
c_2492	+2.21	8.13	+1.14	7.56	0.0	8.70	0.1	0.00086261	0.00585134	0.05107215	0.55916551	A_07_P031808	c_2492	cmr|Putative carbohydrate kinase [c_2492]
c_2494	+2.99	7.95	+1.58	7.16	0.2	8.74	0.0	0.00017949	0.00185302	0.02703633	0.42779899	A_07_P031818	c_2494	cmr|Hypothetical protein [c_2494]
c_2496	-2.48	9.91	-1.31	10.57	0.0	9.25	0.1	0.00039822	0.00332076	0.03720020	0.52527354	A_07_P031827	c_2496	cmr|Hypothetical protein [c_2496]
c_2497	-2.33	10.28	-1.22	10.89	0.0	9.66	0.1	0.00046278	0.00369283	0.04620173	0.57275591	A_07_P031834	c_2497	cmr|Transposase [c_2497]
c_2500	-5.71	8.47	-2.51	9.72	0.1	7.21	0.3	0.00003357	0.00055697	0.00203061	0.14732719	A_07_P031846	c_2500	cmr|Hypothetical protein [c_2500]
c_2501	-2.26	8.94	-1.18	9.53	0.0	8.35	0.2	0.00091596	0.00611315	0.05105056	0.59055107	A_07_P031851	c_2501	cmr|Hypothetical protein [c_2501]
c_2512	+2.71	8.42	+1.44	7.70	0.2	9.14	0.1	0.00042714	0.00349708	0.03343750	0.46753108	A_07_P031888	c_2512	cmr|Transposase [c_2512]
c_2514	+6.46	9.09	+2.69	7.74	0.1	10.43	0.0	0.00000413	0.00015132	0.00608041	0.22577830	A_07_P031900	c_2514	cmr|Hypothetical protein [c_2514]
c_2522	-3.06	10.00	-1.61	10.80	0.0	9.19	0.0	0.00008684	0.00108440	0.01829713	0.38875843	A_07_P031916	c_2522	cmr|Conserved hypothetical protein [c_2522]
c_2528	-3.24	8.95	-1.70	9.80	0.1	8.10	0.1	0.00010763	0.00126648	0.01476435	0.36548742	A_07_P031949	c_2528	cmr|Hypothetical protein [c_2528]
c_2558	+5.88	10.83	+2.55	9.56	0.2	12.11	0.1	0.00001683	0.00035043	0.00737741	0.24452632	A_07_P031994	c_2558	cmr|Mannose-1-phosphate guanylyltransferase [c_2558]
c_2562	-2.79	12.85	-1.48	13.58	0.0	12.11	0.1	0.00020273	0.00201497	0.02537411	0.45918391	A_07_P032014	c_2562	cmr|Glycosyl transferase [c_2562]
c_2566	-2.61	11.56	-1.39	12.25	0.0	10.87	0.1	0.00022964	0.00221673	0.03186992	0.49876740	A_07_P032030	c_2566	cmr|Hypothetical protein [c_2566]
c_2669	-4.15	9.35	-2.05	10.38	0.5	8.32	0.1	0.00088812	0.00598822	0.00639926	0.25701531	A_07_P032221	c_2669	cmr|Hypothetical protein [c_2669]
c_2670	-3.08	9.30	-1.62	10.11	0.2	8.49	0.3	0.00057666	0.00430688	0.01764101	0.38628651	A_07_P032229	c_2670	cmr|Putative conserved protein [c_2670]
c_2729	-3.58	8.71	-1.84	9.63	0.1	7.79	0.1	0.00005426	0.00078198	0.01045859	0.32286275	A_07_P032291	c_2729	cmr|Hypothetical protein [c_2729]
c_2755	-3.08	10.50	-1.62	11.31	0.1	9.69	0.1	0.00011952	0.00135862	0.01817772	0.38831750	A_07_P032344	c_2755	cmr|Hypothetical protein [c_2755]
c_2891	-2.68	10.90	-1.42	11.61	0.0	10.18	0.3	0.00131114	0.00794857	0.02933753	0.48163712	A_07_P032517	c_2891	cmr|Hypothetical protein [c_2891]
c_2895	-3.40	10.54	-1.77	11.43	0.0	9.66	0.2	0.00014103	0.00154404	0.01283473	0.34307301	A_07_P032532	c_2895	cmr|yapH homolog [c_2895]
c_2896	-2.01	11.16	-1.01	11.66	0.1	10.65	0.2	0.00323869	0.01605494	0.07252096	0.67005282	A_07_P032539	c_2896	cmr|Hypothetical protein [c_2896]
c_2897	+2.72	7.60	+1.44	6.88	0.2	8.32	0.0	0.00042845	0.00350235	0.03318407	0.46647321	A_07_P032543	c_2897	cmr|Type 1 fimbriae Regulatory protein fimB [c_2897]
c_2911	-2.14	8.05	-1.10	8.60	0.0	7.50	0.0	0.00077416	0.00535752	0.06056593	0.63274718	A_07_P042574	c_2911	cmr|YfdX protein precursor [c_2911]
c_3030	-2.36	8.21	-1.24	8.83	0.1	7.59	0.1	0.00078222	0.00539515	0.04420922	0.56313107	A_07_P030168	c_3030	cmr|SinI homolog [c_3030]
c_3031	-5.45	8.77	-2.45	9.99	0.0	7.54	0.1	0.00000834	0.00022675	0.00227134	0.15893333	A_07_P030171	c_3031	cmr|SinH homolog [c_3031]
c_3053	-5.05	12.02	-2.34	13.19	0.2	10.85	0.1	0.00002101	0.00040692	0.00296684	0.18246094	A_07_P041244	c_3053	cmr|Protein yfhF [c_3053]
c_3116	-3.61	11.11	-1.85	12.03	0.0	10.18	0.0	0.00003741	0.00059799	0.01019309	0.32485830	A_07_P030232	c_3116	cmr|Ribosomal large subunit pseudouridine synthase D [c_3116]
c_3156	+2.13	8.21	+1.09	7.67	0.1	8.76	0.1	0.00124606	0.00765430	0.05540079	0.57610964	A_07_P030311	c_3156	cmr|Putative tail fiber component K of prophage [c_3156]
c_3158	+4.18	8.07	+2.06	7.04	0.1	9.10	0.3	0.00010760	0.00126648	0.01427337	0.32194842	A_07_P030320	c_3158	cmr|Putative tail component of prophage [c_3158]
c_3159	+2.91	10.16	+1.54	9.39	0.5	10.93	0.4	0.00677485	0.02892955	0.03122269	0.45264273	A_07_P030328	c_3159	cmr|Putative tail component of prophage [c_3159]
c_3161	+6.64	8.26	+2.73	6.90	0.2	9.63	0.2	0.00001118	0.00027255	0.00570567	0.21909756	A_07_P030336	c_3161	cmr|Putative tail component of prophage [c_3161]
c_3163	+10.03	8.60	+3.33	6.94	0.2	10.26	0.2	0.00000516	0.00017391	0.00248666	0.14773585	A_07_P030350	c_3163	cmr|Putative tail component of prophage [c_3163]
c_3164	+20.63	9.16	+4.37	6.98	0.1	11.34	0.3	0.00000198	0.00010186	0.00045668	0.06252174	A_07_P030353	c_3164	cmr|Putative tail component of prophage [c_3164]
c_3165	+5.50	9.21	+2.46	7.98	0.6	10.44	0.1	0.00075642	0.00525554	0.00820122	0.25120623	A_07_P030358	c_3165	cmr|Putative tail fiber component Z of prophage [c_3165]
c_3170	-2.16	7.50	-1.11	8.06	0.0	6.95	0.0	0.00076961	0.00533070	0.05935976	0.62722148	A_07_P030384	c_3170	cmr|Putative capsid protein of prophage [c_3170]
c_3176	+22.85	11.92	+4.51	9.67	0.1	14.18	0.0	0.00000021	0.00003960	0.00034045	0.05360000	A_07_P030411	c_3176	cmr|GnsB protein [c_3176]
c_3214	-2.01	8.52	-1.01	9.02	0.0	8.02	0.0	0.00129101	0.00785683	0.07301004	0.66946418	A_07_P030524	c_3214	cmr|Hypothetical protein [c_3214]
c_3224	-2.10	8.90	-1.07	9.43	0.2	8.36	0.0	0.00191708	0.01066141	0.06408981	0.64598592	A_07_P030537	c_3224	cmr|Conserved hypothetical protein [c_3224]
c_3225	+2.36	8.40	+1.24	7.78	0.0	9.02	0.1	0.00057437	0.00429996	0.04342543	0.51599245	A_07_P030542	c_3225	cmr|Conserved hypothetical protein [c_3225]
c_3242	-2.91	8.94	-1.54	9.71	0.1	8.17	0.5	0.00427627	0.02010921	0.02241425	0.43405904	A_07_P030574	c_3242	cmr|Hypothetical protein [c_3242]
c_3243	-2.03	9.09	-1.02	9.60	0.1	8.58	0.1	0.00207985	0.01140546	0.06978023	0.65904019	A_07_P030576	c_3243	cmr|Hypothetical protein [c_3243]
c_3252	-8.38	11.80	-3.07	13.33	0.0	10.26	0.0	0.00000159	0.00009392	0.00047764	0.06899083	A_07_P041659	c_3252	cmr|Regulatory protein recX [c_3252]
c_3270	-3.57	8.34	-1.84	9.26	0.0	7.42	0.0	0.00003805	0.00060518	0.01065485	0.32446809	A_07_P030625	c_3270	cmr|Hypothetical protein [c_3270]
c_3273	-4.72	12.93	-2.24	14.05	0.1	11.81	0.2	0.00002388	0.00044210	0.00379637	0.20539519	A_07_P030635	c_3273	cmr|Hypothetical protein ygjM [c_3273]
c_3274	-2.08	12.69	-1.05	13.22	0.0	12.17	0.1	0.00111221	0.00704458	0.06662093	0.65026658	A_07_P030642	c_3274	cmr|Hypothetical protein [c_3274]
c_3276	-2.19	8.35	-1.13	8.92	0.0	7.79	0.0	0.00066923	0.00478490	0.05632241	0.61281272	A_07_P030650	c_3276	cmr|Hypothetical protein [c_3276]
c_3292	+2.51	11.24	+1.33	10.58	0.3	11.90	0.1	0.00115979	0.00724307	0.03897167	0.49322347	A_07_P030663	c_3292	cmr|Potential molybdenum-pterin-binding-protein [c_3292]
c_3305	-3.08	7.70	-1.62	8.51	0.1	6.89	0.0	0.00009945	0.00119708	0.01825521	0.38839189	A_07_P030690	c_3305	cmr|Hypothetical protein [c_3305]
c_3309	-2.73	10.63	-1.45	11.36	0.2	9.91	0.1	0.00047759	0.00378609	0.02758321	0.47617325	A_07_P030697	c_3309	cmr|Hypothetical protein [c_3309]
c_3343	-2.43	7.49	-1.28	8.14	0.1	6.85	0.2	0.00095926	0.00633003	0.03898310	0.53462544	A_07_P032631	c_3343	cmr|Hypothetical protein [c_3343]
c_3379	-2.10	9.50	-1.07	10.04	0.1	8.96	0.1	0.00171857	0.00981186	0.06390244	0.64658098	A_07_P032655	c_3379	cmr|Hypothetical protein [c_3379]
c_3391	+2.09	7.90	+1.07	7.37	0.1	8.44	0.0	0.00111780	0.00707056	0.05746697	0.58560518	A_07_P032689	c_3391	cmr|Secreted protein Hcp [c_3391]
c_3391	+2.05	7.76	+1.04	7.24	0.3	8.28	0.1	0.00644007	0.02780162	0.06277248	0.60743085	A_07_P032689	c_3391	cmr|Secreted protein Hcp [c_3391]
c_3394	-5.33	8.17	-2.41	9.37	0.1	6.96	0.2	0.00002462	0.00045104	0.00259400	0.17159664	A_07_P032706	c_3394	cmr|Hypothetical protein [c_3394]
c_3395	-2.09	7.59	-1.07	8.12	0.1	7.06	0.2	0.00229506	0.01228192	0.06476372	0.64780178	A_07_P032713	c_3395	cmr|Hypothetical protein [c_3395]
c_3396	-4.26	8.78	-2.09	9.82	0.0	7.73	0.1	0.00002534	0.00045915	0.00548780	0.24338028	A_07_P032716	c_3396	cmr|Hypothetical protein [c_3396]
c_3397	-2.87	7.73	-1.52	8.49	0.0	6.97	0.0	0.00012170	0.00137842	0.02339240	0.44212485	A_07_P032722	c_3397	cmr|Hypothetical protein [c_3397]
c_3398	-2.62	10.75	-1.39	11.44	0.2	10.05	0.0	0.00058751	0.00436104	0.03092988	0.49437563	A_07_P032729	c_3398	cmr|Hypothetical protein [c_3398]
c_3432	-2.76	11.40	-1.46	12.13	0.1	10.67	0.3	0.00088897	0.00599144	0.02554116	0.46061856	A_07_P032816	c_3432	cmr|Transposase [c_3432]
c_3506	+6.16	10.60	+2.62	9.29	0.1	11.91	0.2	0.00001088	0.00026728	0.00665079	0.23268889	A_07_P032900	c_3506	cmr|Hypothetical protein [c_3506]
c_3507	+11.47	9.24	+3.52	7.48	0.3	11.00	0.3	0.00001392	0.00030904	0.00186039	0.12960177	A_07_P032905	c_3507	cmr|Hypothetical protein [c_3507]
c_3508	+6.17	9.09	+2.63	7.78	0.0	10.40	0.0	0.00000440	0.00015907	0.00662348	0.23328859	A_07_P032907	c_3508	cmr|Hypothetical protein [c_3508]
c_3509	+2.38	7.40	+1.25	6.77	0.0	8.02	0.1	0.00060729	0.00446453	0.04291603	0.51264795	A_07_P032913	c_3509	cmr|Putative ATP-binding protein of ABC transport system [c_3509]
c_3513	-3.35	12.42	-1.74	13.29	0.1	11.54	0.2	0.00011025	0.00128478	0.01352388	0.34847791	A_07_P032930	c_3513	cmr|Putative conserved protein [c_3513]
c_3518	-3.74	8.47	-1.90	9.42	0.1	7.52	0.1	0.00004421	0.00067646	0.00913681	0.30671642	A_07_P032938	c_3518	cmr|Hypothetical protein [c_3518]
c_3523	-2.00	7.17	-1.00	7.67	0.2	6.67	0.0	0.00301865	0.01524235	0.07454205	0.67564191	A_07_P032958	c_3523	cmr|Conserved hypothetical protein [c_3523]
c_3557	-4.29	9.41	-2.10	10.46	0.1	8.36	0.2	0.00003607	0.00058187	0.00556784	0.24350000	A_07_P032997	c_3557	cmr|ShiA homolog [c_3557]
c_3560	-4.02	8.17	-2.01	9.17	0.1	7.16	0.0	0.00003642	0.00058676	0.00692835	0.26735294	A_07_P033008	c_3560	cmr|Unknown protein encoded by ISEc8 within prophage [c_3560]
c_3564	-3.54	9.71	-1.82	10.62	0.4	8.80	0.0	0.00068068	0.00483817	0.01085683	0.32434535	A_07_P033028	c_3564	cmr|Hypothetical protein [c_3564]
c_3565	-4.98	10.28	-2.32	11.43	0.2	9.12	0.2	0.00004521	0.00068369	0.00324060	0.18966543	A_07_P033031	c_3565	cmr|Putative response regulator [c_3565]
c_3566	-2.01	7.83	-1.01	8.33	0.2	7.32	0.1	0.00244758	0.01291377	0.07278646	0.67014620	A_07_P033036	c_3566	cmr|Hypothetical protein [c_3566]
c_3575	-2.59	8.44	-1.37	9.13	0.2	7.75	0.0	0.00056631	0.00425177	0.03234502	0.50270484	A_07_P033084	c_3575	cmr|Transposase insF for insertion sequence IS3A/B/C/D/E/fA [c_3575]
c_3576	-2.29	8.99	-1.20	9.59	0.0	8.40	0.0	0.00050822	0.00393965	0.04881034	0.58261562	A_07_P033087	c_3576	cmr|Unknown in IS [c_3576]
c_3579	-4.43	10.25	-2.15	11.32	0.0	9.18	0.1	0.00002033	0.00039679	0.00475102	0.22598187	A_07_P033108	c_3579	cmr|Hypothetical protein [c_3579]
c_3580	-10.36	11.59	-3.37	13.28	0.0	9.90	0.1	0.00000110	0.00007696	0.00016324	0.04015625	A_07_P033102	c_3580	cmr|Hypothetical protein [c_3580]
c_3581	-4.39	11.71	-2.13	12.78	0.0	10.65	0.1	0.00001863	0.00037505	0.00488059	0.22869048	A_07_P033112	c_3581	cmr|Hypothetical protein [c_3581]
c_3594	-11.74	10.87	-3.55	12.64	0.1	9.09	0.0	0.00000069	0.00006127	0.00008003	0.03000000	A_07_P033172	c_3594	cmr|Putative Transposase [c_3594]
c_3595	-10.24	10.51	-3.36	12.19	0.0	8.84	0.3	0.00000628	0.00019141	0.00019118	0.04560606	A_07_P033176	c_3595	cmr|Transposase [c_3595]
c_3596	-8.68	11.41	-3.12	12.96	0.1	9.85	0.1	0.00000210	0.00010502	0.00040333	0.06350000	A_07_P033185	c_3596	cmr|Hypothetical protein in IS [c_3596]
c_3597	-5.43	9.92	-2.44	11.15	0.0	8.70	0.1	0.00000985	0.00024973	0.00232470	0.16052632	A_07_P033190	c_3597	cmr|Transposase [c_3597]
c_3598	-5.38	9.37	-2.43	10.58	0.0	8.15	0.1	0.00000810	0.00022454	0.00239139	0.16369565	A_07_P033191	c_3598	cmr|Hypothetical protein [c_3598]
c_3598	-6.60	9.55	-2.72	10.92	0.1	8.19	0.1	0.00000554	0.00017942	0.00116743	0.11139394	A_07_P033191	c_3598	cmr|Hypothetical protein [c_3598]
c_3598	-6.28	9.50	-2.65	10.82	0.1	8.17	0.1	0.00000546	0.00017880	0.00142785	0.12351648	A_07_P033191	c_3598	cmr|Hypothetical protein [c_3598]
c_3598	-8.02	9.44	-3.00	10.94	0.0	7.94	0.0	0.00000182	0.00009954	0.00056847	0.07649573	A_07_P033191	c_3598	cmr|Hypothetical protein [c_3598]
c_3598	-6.96	9.41	-2.80	10.81	0.1	8.02	0.0	0.00000302	0.00012594	0.00098450	0.10000000	A_07_P033191	c_3598	cmr|Hypothetical protein [c_3598]
c_3600	-2.57	9.96	-1.36	10.64	0.0	9.27	0.2	0.00047920	0.00379316	0.03409108	0.51068506	A_07_P033203	c_3600	cmr|Hypothetical protein [c_3600]
c_3601	-9.08	9.66	-3.18	11.25	0.1	8.07	0.0	0.00000157	0.00009392	0.00034045	0.05583333	A_07_P033207	c_3601	cmr|Hypothetical protein [c_3601]
c_3602	-4.05	11.94	-2.02	12.94	0.2	10.93	0.1	0.00006046	0.00084243	0.00675940	0.26341584	A_07_P033212	c_3602	cmr|Hypothetical protein [c_3602]
c_3603	-3.05	10.13	-1.61	10.93	0.1	9.32	0.4	0.00173790	0.00989906	0.01901677	0.39550859	A_07_P033216	c_3603	cmr|Hypothetical protein [c_3603]
c_3604	-2.40	8.32	-1.26	8.95	0.0	7.68	0.2	0.00066439	0.00476110	0.04135671	0.54900506	A_07_P033222	c_3604	cmr|Hypothetical protein [c_3604]
c_3606	+4.79	9.86	+2.26	8.73	0.1	10.99	0.1	0.00001500	0.00032347	0.01117759	0.28801964	A_07_P033231	c_3606	cmr|Hypothetical protein [c_3606]
c_3609	-2.80	8.79	-1.48	9.53	0.1	8.05	0.3	0.00076433	0.00529881	0.02561738	0.46146453	A_07_P033238	c_3609	cmr|Hypothetical protein [c_3609]
c_3612	-2.60	10.87	-1.38	11.56	0.2	10.18	0.2	0.00082584	0.00565414	0.03170160	0.49811377	A_07_P033247	c_3612	cmr|Transposase insC for insertion element IS2A/D/F/H/I/K [c_3612]
c_3614	-3.59	9.36	-1.84	10.28	0.1	8.43	0.1	0.00007989	0.00101674	0.01030615	0.32322709	A_07_P033257	c_3614	cmr|Hypothetical protein [c_3614]
c_3616	-2.76	10.96	-1.47	11.70	0.1	10.23	0.0	0.00024430	0.00230872	0.02642149	0.46897407	A_07_P033266	c_3616	cmr|Unknown in ISEc8 [c_3616]
c_3617	-2.48	8.34	-1.31	8.99	0.1	7.68	0.1	0.00040646	0.00336751	0.03800114	0.52899204	A_07_P036281	c_3617	cmr|Unknown in putative ISEc8 [c_3617]
c_3618	-9.26	9.58	-3.21	11.19	0.1	7.98	0.1	0.00000151	0.00009241	0.00029662	0.05367816	A_07_P036289	c_3618	cmr|Hypothetical protein [c_3618]
c_3618	-9.33	9.67	-3.22	11.29	0.1	8.06	0.1	0.00000177	0.00009825	0.00029281	0.05423529	A_07_P036289	c_3618	cmr|Hypothetical protein [c_3618]
c_3618	-8.38	9.32	-3.07	10.86	0.1	7.79	0.0	0.00000184	0.00009954	0.00047891	0.06854545	A_07_P036289	c_3618	cmr|Hypothetical protein [c_3618]
c_3618	-10.39	9.68	-3.38	11.37	0.1	7.99	0.1	0.00000112	0.00007798	0.00016133	0.04031746	A_07_P036289	c_3618	cmr|Hypothetical protein [c_3618]
c_3618	-8.55	9.31	-3.10	10.86	0.0	7.76	0.0	0.00000154	0.00009303	0.00041603	0.06421569	A_07_P036289	c_3618	cmr|Hypothetical protein [c_3618]
c_3620	-6.54	10.60	-2.71	11.95	0.1	9.24	0.2	0.00000801	0.00022294	0.00122015	0.11434524	A_07_P036293	c_3620	cmr|Hypothetical protein [c_3620]
c_3630	-3.64	9.42	-1.86	10.35	0.1	8.48	0.1	0.00004213	0.00064958	0.01004129	0.32395492	A_07_P036335	c_3630	cmr|Hypothetical protein yjhS precursor [c_3630]
c_3631	-3.68	9.31	-1.88	10.25	0.1	8.37	0.2	0.00012020	0.00136440	0.00925749	0.30879237	A_07_P036340	c_3631	cmr|Hypothetical protein [c_3631]
c_3636	+2.35	8.18	+1.23	7.56	0.0	8.80	0.0	0.00041120	0.00339303	0.04414380	0.52138035	A_07_P036358	c_3636	cmr|Hypothetical protein [c_3636]
c_3638	+2.51	8.60	+1.32	7.93	0.1	9.26	0.2	0.00100411	0.00654555	0.03895960	0.49346742	A_07_P036368	c_3638	cmr|Hypothetical protein yhcI [c_3638]
c_3639	+5.94	9.41	+2.57	8.12	0.0	10.69	0.0	0.00000490	0.00016801	0.00709604	0.24129590	A_07_P036375	c_3639	cmr|N-acetylneuraminate lyase subunit [c_3639]
c_3641	-4.06	9.38	-2.02	10.39	0.2	8.37	0.3	0.00014757	0.00159788	0.00698298	0.26814634	A_07_P036385	c_3641	cmr|Unknown in ISEc8 [c_3641]
c_3643	-2.31	10.88	-1.21	11.48	0.0	10.27	0.1	0.00066705	0.00477580	0.04809705	0.58070552	A_07_P036391	c_3643	cmr|Unknown in ISEc8 [c_3643]
c_3646	-4.18	12.18	-2.06	13.21	0.3	11.15	0.2	0.00018976	0.00192999	0.00594703	0.25237197	A_07_P036409	c_3646	cmr|Hypothetical protein [c_3646]
c_3648	-2.23	7.64	-1.15	8.21	0.0	7.06	0.0	0.00059978	0.00443275	0.05385480	0.60434070	A_07_P036418	c_3648	cmr|Hypothetical protein [c_3648]
c_3652	-2.73	9.32	-1.45	10.04	0.1	8.59	0.1	0.00021834	0.00213217	0.02730882	0.47351322	A_07_P036440	c_3652	cmr|Hypothetical protein yfjI [c_3652]
c_3654	-3.20	8.29	-1.68	9.13	0.0	7.45	0.1	0.00007783	0.00100024	0.01572917	0.37351433	A_07_P036449	c_3654	cmr|Hypothetical protein yeeP [c_3654]
c_3655	+4.53	11.35	+2.18	10.26	0.1	12.44	0.2	0.00004593	0.00069200	0.01248539	0.30148773	A_07_P036451	c_3655	cmr|Antigen 43 precursor [c_3655]
c_3658	-35.74	14.04	-5.16	16.62	0.0	11.46	0.0	0.00000006	0.00003344	0.00000254	0.00500000	A_07_P036466	c_3658	cmr|Hypothetical protein [c_3658]
c_3659	-36.27	9.91	-5.18	12.50	0.0	7.32	0.2	0.00000012	0.00003548	0.00000191	0.00428571	A_07_P036474	c_3659	cmr|Unknown protein encoded by ISEc8 [c_3659]
c_3660	-40.62	9.86	-5.34	12.53	0.0	7.19	0.1	0.00000006	0.00003344	0.00000127	0.00400000	A_07_P036480	c_3660	cmr|Unknown protein encoded by ISEc8 [c_3660]
c_3661	-2.14	8.79	-1.10	9.34	0.0	8.24	0.2	0.00222082	0.01199062	0.05936484	0.62685446	A_07_P036481	c_3661	cmr|Hypothetical protein [c_3661]
c_3662	-3.47	11.44	-1.79	12.33	0.1	10.54	0.1	0.00006923	0.00092999	0.01185023	0.33737794	A_07_P036488	c_3662	cmr|Unknown protein encoded by ISEc8 [c_3662]
c_3663	-4.84	8.68	-2.27	9.82	0.1	7.54	0.0	0.00001315	0.00029912	0.00343242	0.19369176	A_07_P036493	c_3663	cmr|Hypothetical protein [c_3663]
c_3666	-2.70	7.96	-1.43	8.68	0.0	7.24	0.1	0.00026573	0.00245372	0.02813897	0.47585392	A_07_P036509	c_3666	cmr|Hypothetical protein ykfF [c_3666]
c_3668	-2.41	9.03	-1.27	9.66	0.1	8.39	0.1	0.00042903	0.00350320	0.04201220	0.55120000	A_07_P036519	c_3668	cmr|Hypothetical protein yafZ [c_3668]

c_3670	-3.58	9.05	-1.84	9.97	0.0	8.13	0.1	0.00003973	0.00062482	0.01053481	0.32331384	A_07_P036530	c_3670	cmr|Hypothetical protein [c_3670]
c_3675	-2.90	9.59	-1.54	10.36	0.0	8.82	0.1	0.00013159	0.00146726	0.02238694	0.43406404	A_07_P036560	c_3675	cmr|Hypothetical protein [c_3675]
c_3681	-11.23	11.94	-3.49	13.68	0.0	10.20	0.1	0.00000078	0.00006333	0.00010544	0.03688889	A_07_P036583	c_3681	cmr|Hypothetical protein [c_3681]
c_3682	-15.53	10.81	-3.96	12.78	0.0	8.83	0.1	0.00000041	0.00004972	0.00003176	0.01666667	A_07_P036586	c_3682	cmr|Hypothetical protein [c_3682]
c_3683	-48.00	12.07	-5.59	14.86	0.0	9.28	0.2	0.00000007	0.00003344	0.00000064	0.00333333	A_07_P036593	c_3683	cmr|Hypothetical protein [c_3683]
c_3686	+7.73	10.15	+2.95	8.68	0.1	11.63	0.0	0.00000326	0.00013195	0.00426766	0.18873596	A_07_P036604	c_3686	cmr|Hypothetical protein yrbH [c_3686]
c_3689	+4.66	9.15	+2.22	8.04	0.1	10.26	0.0	0.00001795	0.00036652	0.01197917	0.29561129	A_07_P036619	c_3689	cmr|3-deoxy-manno-octulosonate cytidylyltransferase [c_3689]
c_3693	+3.76	9.09	+1.91	8.14	0.2	10.05	0.5	0.00122096	0.00754130	0.01757812	0.35076046	A_07_P036636	c_3693	cmr|Hypothetical protein [c_3693]
c_3693	+4.14	9.68	+2.05	8.66	0.3	10.71	0.3	0.00026949	0.00248118	0.01431720	0.32247496	A_07_P036636	c_3693	cmr|Hypothetical protein [c_3693]
c_3693	+4.69	9.53	+2.23	8.42	0.0	10.65	0.0	0.00001154	0.00027747	0.01161331	0.29348315	A_07_P036636	c_3693	cmr|Hypothetical protein [c_3693]
c_3693	+5.19	9.59	+2.38	8.41	0.1	10.78	0.1	0.00001246	0.00029182	0.00942264	0.26826401	A_07_P036636	c_3693	cmr|Hypothetical protein [c_3693]
c_3693	+5.64	9.69	+2.50	8.44	0.1	10.94	0.1	0.00001282	0.00029422	0.00792302	0.24948000	A_07_P036636	c_3693	cmr|Hypothetical protein [c_3693]
c_3694	+2.61	11.34	+1.38	10.64	0.0	12.03	0.2	0.00037046	0.00313406	0.03554433	0.47707587	A_07_P036641	c_3694	cmr|Hypothetical protein [c_3694]
c_3695	+6.32	10.28	+2.66	8.95	0.2	11.61	0.1	0.00001787	0.00036585	0.00635671	0.22901602	A_07_P036649	c_3695	cmr|Hypothetical protein [c_3695]
c_3703	-2.63	10.69	-1.40	11.38	0.4	9.99	0.3	0.00415636	0.01962150	0.03069868	0.49368744	A_07_P036678	c_3703	cmr|Transposase insG for insertion sequence element IS4 [c_3703]
c_3712	-2.84	9.10	-1.51	9.86	0.5	8.35	0.1	0.00365790	0.01771453	0.02358422	0.44309069	A_07_P036681	c_3712	cmr|Putative saframycin Mx1 synthetase B [c_3712]
c_3766	-2.04	10.18	-1.03	10.69	0.0	9.67	0.0	0.00118679	0.00737365	0.07088351	0.66467540	A_07_P036785	c_3766	cmr|Hypothetical protein [c_3766]
c_3772	-2.00	7.80	-1.00	8.30	0.3	7.30	0.2	0.01000337	0.03922098	0.07631669	0.67959842	A_07_P036806	c_3772	cmr|Putative iron compound permease protein of ABC [c_3772]
c_3774	-2.13	7.96	-1.09	8.51	0.0	7.42	0.2	0.00141979	0.00841610	0.06133638	0.63615283	A_07_P036820	c_3774	cmr|Ferric enterobactin transport ATP-binding protein fepC [c_3774]
c_3819	-7.63	14.84	-2.93	16.30	0.0	13.37	0.3	0.00002140	0.00041080	0.00067010	0.08178295	A_07_P036906	c_3819	cmr|Putative conserved protein [c_3819]
c_3873	+85.55	10.54	+6.42	7.33	0.1	13.75	0.4	0.00000025	0.00004211	0.00000254	0.00363636	A_07_P034083	c_3873	cmr|Putative conserved protein [c_3873]
c_3890	+4.35	8.96	+2.12	7.90	0.7	10.02	0.3	0.00354636	0.01728069	0.01345084	0.31373333	A_07_P034096	c_3890	cmr|PTS system, N-acetylgalactosamine-specific IID component [c_3890]
c_3891	+5.95	8.72	+2.57	7.43	0.3	10.00	0.1	0.00004764	0.00071298	0.00715066	0.24107066	A_07_P034105	c_3891	cmr|Putative phosphotransferase system enzyme subunit [c_3891]
c_3928	+2.40	7.53	+1.26	6.90	0.0	8.16	0.1	0.00056498	0.00424382	0.04212462	0.51094761	A_07_P034139	c_3928	cmr|Hypothetical protein [c_3928]
c_3976	+7.03	8.38	+2.81	6.97	0.1	9.79	0.0	0.00000299	0.00012554	0.00509909	0.20744186	A_07_P041300	c_3976	cmr|Hypothetical protein yhcI [c_3976]
c_4010	+2.37	9.35	+1.25	8.73	0.1	9.97	0.1	0.00069963	0.00493057	0.04356136	0.51721719	A_07_P034196	c_4010	cmr|Hypothetical protein [c_4010]
c_4013	-2.87	11.12	-1.52	11.88	0.0	10.37	0.3	0.00050293	0.00391210	0.02266514	0.43623472	A_07_P034204	c_4013	cmr|Hypothetical protein [c_4013]
c_4014	-9.53	8.60	-3.25	10.22	0.1	6.97	0.1	0.00000165	0.00009590	0.00026931	0.05435897	A_07_P034207	c_4014	cmr|Hypothetical protein [c_4014]
c_4015	-3.21	8.27	-1.68	9.11	0.1	7.42	0.3	0.00032027	0.00280126	0.01530488	0.37242658	A_07_P034215	c_4015	cmr|Ribose transport system permease protein rbsC [c_4015]
c_4016	-8.91	8.30	-3.15	9.88	0.0	6.72	0.0	0.00000133	0.00008557	0.00034235	0.05556701	A_07_P034216	c_4016	cmr|Ribose transport ATP-binding protein rbsA [c_4016]
c_4064	-2.34	8.70	-1.23	9.31	0.0	8.09	0.2	0.00098345	0.00645412	0.04609248	0.57275454	A_07_P034279	c_4064	cmr|Hypothetical protein [c_4064]
c_4074	-2.05	7.50	-1.04	8.02	0.1	6.98	0.1	0.00166014	0.00953567	0.06805386	0.65531498	A_07_P034295	c_4074	cmr|Hypothetical protein [c_4074]
c_4088	-2.89	8.64	-1.53	9.40	0.0	7.87	0.3	0.00064316	0.00464494	0.02282901	0.43725061	A_07_P034318	c_4088	cmr|Hypothetical protein [c_4088]
c_4209	-2.31	10.19	-1.21	10.80	0.0	9.59	0.1	0.00053957	0.00410825	0.04745109	0.57644290	A_07_P034472	c_4209	cmr|Putative minor fimbrial subunit precursor [c_4209]
c_4223	+2.58	8.60	+1.37	7.91	0.0	9.28	0.1	0.00028083	0.00256758	0.03627858	0.48078283	A_07_P034506	c_4223	cmr|Putative conserved protein [c_4223]
c_4277	+2.94	8.05	+1.56	7.27	0.3	8.83	0.0	0.00067879	0.00483352	0.02887576	0.44009681	A_07_P034581	c_4277	cmr|Putative phosphotransferase system enzyme subunit [c_4277]
c_4284	-3.38	10.35	-1.76	11.23	0.1	9.47	0.0	0.00006186	0.00085663	0.01295795	0.34345118	A_07_P034612	c_4284	cmr|Putative aldolase [c_4284]
c_4352	-2.08	7.91	-1.05	8.43	0.0	7.38	0.0	0.00096313	0.00634991	0.06662030	0.65066377	A_07_P039574	c_4352	cmr|Hypothetical protein [c_4352]
c_4360	-4.39	9.10	-2.14	10.16	0.1	8.03	0.5	0.00070340	0.00494828	0.00549543	0.24303371	A_07_P039592	c_4360	cmr|Hypothetical protein [c_4360]
c_4422	-2.84	12.23	-1.51	12.98	0.2	11.47	0.0	0.00021976	0.00214234	0.02404345	0.44797633	A_07_P039723	c_4422	cmr|Hypothetical protein [c_4422]
c_4437	+3.89	7.74	+1.96	6.76	0.0	8.72	0.1	0.00003111	0.00052951	0.01692454	0.34650195	A_07_P039756	c_4437	cmr|Hypothetical protein [c_4437]
c_4437	+3.56	7.90	+1.83	6.98	0.1	8.81	0.1	0.00005920	0.00083145	0.01982914	0.36989336	A_07_P039756	c_4437	cmr|Hypothetical protein [c_4437]
c_4437	+3.46	7.88	+1.79	6.98	0.1	8.78	0.0	0.00005088	0.00074789	0.02065231	0.37808140	A_07_P039756	c_4437	cmr|Hypothetical protein [c_4437]
c_4437	+3.27	7.81	+1.71	6.95	0.1	8.66	0.1	0.00009916	0.00119443	0.02303417	0.40564877	A_07_P039756	c_4437	cmr|Hypothetical protein [c_4437]
c_4437	+3.04	7.93	+1.60	7.13	0.2	8.74	0.2	0.00056730	0.00425363	0.02677846	0.42845528	A_07_P039756	c_4437	cmr|Hypothetical protein [c_4437]
c_4481	-2.21	10.32	-1.15	10.89	0.1	9.75	0.2	0.00147108	0.00864850	0.05310340	0.59889685	A_07_P039839	c_4481	cmr|Conserved hypothetical protein [c_4481]
c_4486	+2.47	7.30	+1.30	6.65	0.2	7.95	0.0	0.00073685	0.00514135	0.04032266	0.49948072	A_07_P039865	c_4486	cmr|PTS system, fructose-like-2 IIB component 1 [c_4486]
c_4486	+2.43	7.32	+1.28	6.68	0.1	7.96	0.1	0.00064617	0.00466377	0.04135988	0.50478295	A_07_P039865	c_4486	cmr|PTS system, fructose-like-2 IIB component 1 [c_4486]
c_4486	+2.44	7.27	+1.29	6.63	0.0	7.91	0.0	0.00031262	0.00275733	0.04068280	0.50039844	A_07_P039865	c_4486	cmr|PTS system, fructose-like-2 IIB component 1 [c_4486]
c_4486	+2.41	7.37	+1.27	6.73	0.2	8.00	0.3	0.00242815	0.01283662	0.04392658	0.51959429	A_07_P039865	c_4486	cmr|PTS system, fructose-like-2 IIB component 1 [c_4486]
c_4487	+3.08	7.97	+1.62	7.16	0.1	8.78	0.0	0.00010830	0.00127097	0.02563135	0.42433228	A_07_P039870	c_4487	cmr|Putative phosphotransferase system [c_4487]
c_4488	+2.21	7.38	+1.14	6.81	0.0	7.95	0.2	0.00130358	0.00791804	0.05146659	0.55997927	A_07_P039873	c_4488	cmr|Putative transcriptional Antiterminator [c_4488]
c_4493	-2.24	8.45	-1.16	9.03	0.0	7.87	0.2	0.00099344	0.00650068	0.05178544	0.59209150	A_07_P039886	c_4493	cmr|Hypothetical protein [c_4493]
c_4523	-3.33	8.49	-1.74	9.36	0.2	7.62	0.2	0.00033643	0.00290553	0.01389355	0.35394822	A_07_P040008	c_4523	cmr|Conserved hypothetical protein [c_4523]
c_4548	-4.41	8.95	-2.14	10.02	0.2	7.88	0.3	0.00013843	0.00152299	0.00517530	0.23413793	A_07_P040129	c_4548	cmr|Hypothetical protein [c_4548]
c_4580	-3.99	8.91	-2.00	9.91	0.2	7.92	0.1	0.00006641	0.00090442	0.00718686	0.27134293	A_07_P038963	c_4580	cmr|Hypothetical protein [c_4580]
c_4688	+3.08	8.08	+1.62	7.27	0.6	8.89	0.2	0.00532571	0.02397710	0.02729167	0.42968000	A_07_P039063	c_4688	cmr|Hypothetical protein [c_4688]
c_4735	+2.46	9.16	+1.30	8.51	0.1	9.81	0.1	0.00045548	0.00364940	0.03998666	0.49727488	A_07_P039123	c_4735	cmr|Conserved hypothetical protein [c_4735]
c_4758	+2.35	8.28	+1.23	7.66	0.3	8.90	0.1	0.00226267	0.01215403	0.04575521	0.52890602	A_07_P039181	c_4758	cmr|PTS system, glucose-specific IIBC component [c_4758]
c_4762	-2.21	8.10	-1.14	8.67	0.2	7.53	0.3	0.00484344	0.02226426	0.05514863	0.60802521	A_07_P039201	c_4762	cmr|Putative permease [c_4762]
c_4769	+2.09	7.47	+1.06	6.94	0.2	8.00	0.1	0.00204941	0.01127391	0.05811801	0.58956830	A_07_P039231	c_4769	cmr|Hypothetical protein [c_4769]
c_4770	+2.81	8.07	+1.49	7.33	0.0	8.81	0.1	0.00021347	0.00209925	0.03065993	0.44986952	A_07_P039236	c_4770	cmr|Hypothetical protein [c_4770]
c_4776	+4.51	8.82	+2.17	7.74	0.3	9.91	0.1	0.00006881	0.00092591	0.01261751	0.30328244	A_07_P039258	c_4776	cmr|Hypothetical protein [c_4776]
c_4777	+7.86	8.43	+2.97	6.94	0.0	9.92	0.2	0.00000415	0.00015177	0.00401169	0.18307246	A_07_P039263	c_4777	cmr|Putative conserved protein [c_4777]
c_4778	+3.29	8.28	+1.72	7.43	0.1	9.14	0.1	0.00010378	0.00123350	0.02252731	0.39985344	A_07_P039269	c_4778	cmr|Putative conserved protein [c_4778]
c_4779	+3.83	8.48	+1.94	7.51	0.1	9.45	0.1	0.00004185	0.00064800	0.01728468	0.34799233	A_07_P039274	c_4779	cmr|Putative transcriptional regulator [c_4779]
c_4780	+3.44	12.89	+1.78	12.00	0.0	13.78	0.1	0.00005899	0.00082929	0.02101690	0.38253179	A_07_P039276	c_4780	cmr|Hypothetical protein [c_4780]
c_4836	+9.89	11.73	+3.31	10.08	0.0	13.38	0.2	0.00000262	0.00011934	0.00259464	0.14800725	A_07_P039365	c_4836	cmr|Putative lipase [c_4836]
c_4837	-24.91	12.42	-4.64	14.74	0.0	10.10	0.1	0.00000015	0.00003548	0.00000699	0.00733333	A_07_P039369	c_4837	cmr|Hypothetical protein [c_4837]
c_4838	-3.51	12.45	-1.81	13.35	0.1	11.54	0.1	0.00006439	0.00088167	0.01129192	0.33167910	A_07_P039373	c_4838	cmr|Hypothetical protein [c_4838]
c_4839	-23.26	11.28	-4.54	13.55	0.1	9.01	0.1	0.00000019	0.00003706	0.00000921	0.00878788	A_07_P039376	c_4839	cmr|Hypothetical protein [c_4839]
c_4858	+2.08	7.19	+1.06	6.66	0.0	7.72	0.1	0.00152936	0.00893653	0.05747332	0.58529107	A_07_P039424	c_4858	cmr|Hypothetical protein [c_4858]
c_4924	-3.00	7.91	-1.59	8.70	0.0	7.11	0.1	0.00011127	0.00129378	0.01974276	0.40315175	A_07_P039536	c_4924	cmr|Putative hippuricase [c_4924]
c_4925	-2.38	8.42	-1.25	9.05	0.1	7.80	0.2	0.00110936	0.00703699	0.04366425	0.56072594	A_07_P039541	c_4925	cmr|Putative citrate permease [c_4925]
c_4928	-3.60	8.75	-1.85	9.67	0.1	7.82	0.2	0.00009887	0.00119275	0.01052083	0.32351562	A_07_P037396	c_4928	cmr|tRNA [c_4928]
c_4986	-2.35	7.54	-1.23	8.16	0.1	6.93	0.0	0.00055898	0.00421682	0.04522675	0.56918465	A_07_P037506	c_4986	cmr|D-glucitol-6-phosphate dehydrogenase [c_4986]
c_5019	-4.96	8.81	-2.31	9.96	0.0	7.65	0.2	0.00003008	0.00051644	0.00320884	0.18992481	A_07_P035565	c_5019	cmr|Hypothetical protein yjbO [c_5019]
c_5029	-2.55	7.66	-1.35	8.33	0.0	6.98	0.1	0.00031110	0.00275220	0.03498539	0.51048193	A_07_P037590	c_5029	cmr|Putative transporter [c_5029]
c_5033	-2.13	8.29	-1.09	8.84	0.1	7.75	0.1	0.00133635	0.00805492	0.06072980	0.63319868	A_07_P037610	c_5033	cmr|Hypothetical protein [c_5033]
c_5035	+2.55	8.14	+1.35	7.47	0.1	8.82	0.0	0.00040108	0.00333578	0.03748984	0.48659522	A_07_P037619	c_5035	cmr|Putative 2-oxoglutarate dehydrogenase [c_5035]
c_5055	+2.60	8.34	+1.38	7.65	0.0	9.03	0.2	0.00041560	0.00342042	0.03583016	0.47927782	A_07_P037658	c_5055	cmr|Hypothetical protein [c_5055]
c_5058	-2.60	8.93	-1.38	9.62	0.2	8.24	0.2	0.00115467	0.00721967	0.03234502	0.50320158	A_07_P037663	c_5058	cmr|Putative transcriptional regulator [c_5058]
c_5061	-3.07	8.87	-1.62	9.68	0.7	8.06	0.3	0.00846561	0.03436004	0.01778011	0.38664365	A_07_P037671	c_5061	cmr|Hypothetical protein [c_5061]
c_5061	-2.98	8.57	-1.58	9.36	0.2	7.78	0.1	0.00021106	0.00208075	0.01992696	0.40586028	A_07_P037671	c_5061	cmr|Hypothetical protein [c_5061]
c_5062	+6.38	8.85	+2.67	7.52	0.1	10.19	0.1	0.00000761	0.00021549	0.00631542	0.22910138	A_07_P037679	c_5062	cmr|Putative symporter yjcG [c_5062]
c_5088	-2.64	7.54	-1.40	8.24	0.0	6.84	0.0	0.00020171	0.00200865	0.03035569	0.49017436	A_07_P037733	c_5088	cmr|Hypothetical protein [c_5088]
c_5135	-2.18	7.30	-1.12	7.86	0.2	6.74	0.1	0.00267822	0.01383394	0.05706745	0.61665752	A_07_P037796	c_5135	cmr|Hypothetical protein [c_5135]
c_5153	-4.45	9.69	-2.15	10.77	0.0	8.62	0.2	0.00002800	0.00049142	0.00473450	0.22656535	A_07_P037845	c_5153	cmr|Hypothetical protein [c_5153]
c_5154	-3.12	9.07	-1.64	9.89	0.1	8.25	0.2	0.00031525	0.00277437	0.01693471	0.37980057	A_07_P037850	c_5154	cmr|Hypothetical protein [c_5154]
c_5166	-3.19	9.68	-1.67	10.52	0.0	8.85	0.2	0.00016398	0.00173498	0.01609057	0.37641902	A_07_P037902	c_5166	cmr|Partial Transposase [c_5166]
c_5207	-2.43	11.47	-1.28	12.11	0.2	10.84	0.3	0.00245524	0.01294373	0.04021341	0.54251928	A_07_P038058	c_5207	cmr|Hypothetical protein [c_5207]
c_5233	-2.22	8.53	-1.15	9.10	0.1	7.95	0.0	0.00069560	0.00490746	0.05403709	0.60466240	A_07_P036933	c_5233	cmr|Hypothetical protein [c_5233]
c_5295	-6.31	10.49	-2.66	11.82	0.0	9.16	0.1	0.00000514	0.00017372	0.00139037	0.12437500	A_07_P036994	c_5295	cmr|Hypothetical protein [c_5295]
c_5296	-15.98	12.12	-4.00	14.12	0.0	10.12	0.1	0.00000049	0.00005264	0.00003176	0.01724138	A_07_P037000	c_5296	cmr|Hypothetical protein [c_5296]
c_5297	-3.27	9.02	-1.71	9.87	0.2	8.16	0.2	0.00026382	0.00244187	0.01414444	0.35744783	A_07_P037001	c_5297	cmr|Hypothetical protein [c_5297]
c_5321	-2.87	8.97	-1.52	9.73	0.2	8.21	0.5	0.00328103	0.01622380	0.02336573	0.44268351	A_07_P037042	c_5321	cmr|Putative conserved protein [c_5321]
c_5334	+2.63	8.46	+1.40	7.76	0.6	9.16	0.2	0.00791924	0.03268166	0.03691374	0.48511686	A_07_P037056	c_5334	cmr|Hypothetical protein [c_5334]
c_5341	-78.91	11.78	-6.30	14.93	0.1	8.63	0.1	0.00000002	0.00003344	0.00000000	0.00000000	A_07_P042071	c_5341	cmr|Mg [c_5341]
c_5343	-40.00	9.66	-5.32	12.33	0.1	7.00	0.2	0.00000010	0.00003427	0.00000127	0.00333333	A_07_P037072	c_5343	cmr|Hypothetical protein [c_5343]
c_5356	-2.56	7.76	-1.36	8.43	0.1	7.08	0.0	0.00029383	0.00264683	0.03331872	0.50585342	A_07_P037109	c_5356	cmr|Hypothetical protein [c_5356]
c_5375	-2.56	9.73	-1.36	10.41	0.4	9.05	0.3	0.00442881	0.02066605	0.03202109	0.50063555	A_07_P037145	c_5375	cmr|Hypothetical protein [c_5375]
c_5376	-2.71	10.49	-1.44	11.21	0.2	9.77	0.1	0.00043574	0.00354356	0.02832190	0.47588047	A_07_P037149	c_5376	cmr|Hypothetical protein [c_5376]
c_5416	+2.30	8.46	+1.20	7.86	0.2	9.06	0.1	0.00113202	0.00712615	0.04697091	0.53510130	A_07_P037232	c_5416	cmr|Hypothetical protein [c_5416]
c_5455	+4.16	8.80	+2.06	7.77	0.1	9.83	0.4	0.00029957	0.00267978	0.01462843	0.32714489	A_07_P037341	c_5455	cmr|Hypothetical protein [c_5455]
caiA	-9.51	11.78	-3.25	13.41	0.0	10.16	0.4	0.00001880	0.00037681	0.00027947	0.05432099	A_07_P010884	b0039	cmr|probable carnitine operon oxidoreductase [b0039]
caiC	+2.59	8.56	+1.38	7.87	0.1	9.25	0.1	0.00033281	0.00287739	0.03612487	0.48036318	A_07_P035675	c_0046	cmr|Probable crotonobetaine/carnitine-CoA ligase [c_0046]
caiT	+2.10	6.82	+1.07	6.29	0.0	7.36	0.3	0.00379076	0.01818085	0.05697726	0.58477836	A_07_P010887	b0040	cmr|probable carnitine transporter [b0040]
carA	+4.97	8.86	+2.31	7.70	0.2	10.01	0.3	0.00006654	0.00090540	0.01036585	0.28089501	A_07_P010846	b0032	cmr|carbamoyl-phosphate synthetase, glutamine [b0032]
carB	+15.05	10.55	+3.91	8.60	0.2	12.51	0.1	0.00000141	0.00008809	0.00102579	0.09670659	A_07_P010855	b0033	cmr|carbamoyl-phosphate synthase large subunit [b0033]
cbl	-5.81	12.96	-2.54	14.23	0.0	11.69	0.3	0.00003048	0.00052217	0.00184896	0.14200000	A_07_P015271	b1987	cmr|transcriptional regulator cys regulon; accessory regulatory circuit affecting cysM [b1987]
cbpA	+5.66	8.58	+2.50	7.33	0.2	9.83	0.6	0.00068225	0.00484496	0.00804052	0.24968442	A_07_P001235	b1000	cmr|curved DNA-binding protein; functions closely related to DnaJ [b1000]
cbpM	+7.60	8.70	+2.93	7.23	0.0	10.16	0.1	0.00000295	0.00012541	0.00438135	0.18950549	A_07_P001226	b0999	cmr|orf, hypothetical protein [b0999]
ccmD	+2.20	7.94	+1.13	7.37	0.0	8.50	0.1	0.00087096	0.00590290	0.05162411	0.56014473	A_07_P032305	c_2735	cmr|Heme exporter protein D [c_2735]
cdaR	-2.39	9.46	-1.26	10.08	0.0	8.83	0.2	0.00089905	0.00604647	0.04183562	0.54979967	A_07_P002772	b0162	cmr|orf, hypothetical protein [b0162]
cdh	-2.40	8.13	-1.26	8.76	0.1	7.50	0.2	0.00081298	0.00557469	0.04210429	0.55194838	A_07_P019370	b3918	cmr|CDP-diacylglycerol phosphotidylhydrolase [b3918]
cdsA	-2.46	11.17	-1.30	11.83	0.2	10.52	0.4	0.00377216	0.01811930	0.03714685	0.52499102	A_07_P002831	b0175	cmr|CDP-diglyceride synthetase [b0175]
cedA	-4.67	11.19	-2.22	12.30	0.1	10.08	0.2	0.00002949	0.00050857	0.00402248	0.20900990	A_07_P018702	b1731	cmr|orf, hypothetical protein [b1731]
chaA	-2.76	9.45	-1.46	10.18	0.0	8.72	0.3	0.00066332	0.00475776	0.02683244	0.47148438	A_07_P007032	b1216	cmr|sodium-calcium/proton antiporter [b1216]
chaB	-2.08	8.93	-1.06	9.46	0.0	8.40	0.1	0.00100857	0.00655968	0.06600229	0.65190715	A_07_P007036	b1217	cmr|cation transport regulator [b1217]
chbC	-2.04	8.78	-1.03	9.29	0.2	8.26	0.3	0.00840993	0.03420749	0.06736598	0.65268308	A_07_P018731	b1737	cmr|PEP-dependent phosphotransferase enzyme II for cellobiose, arbutin, and salicin [b1737]
cheZ	-3.62	11.63	-1.86	12.56	0.1	10.70	0.1	0.00005151	0.00075300	0.00991806	0.32129630	A_07_P016084	b1881	cmr|chemotactic response; CheY protein phophatase; antagonist of CheY as switch regulator [b1881]
chpB	-2.03	10.86	-1.02	11.37	0.0	10.35	0.0	0.00116813	0.00728355	0.07096926	0.66508333	A_07_P003680	b4225	cmr|probable growth inhibitor, PemK-like, autoregulated [b4225]
chuS	+2.45	8.13	+1.29	7.48	0.2	8.77	0.1	0.00066807	0.00477877	0.04035823	0.49913590	A_07_P034657	c_4307	cmr|Putative heme/hemoglobin transport protein [c_4307]
clpA	+5.44	10.17	+2.44	8.95	0.0	11.39	0.1	0.00000941	0.00024318	0.00859057	0.25811069	A_07_P004862	b0882	cmr|ATP-binding component of serine protease [b0882]
clpB	+2.51	9.46	+1.32	8.79	0.1	10.12	0.2	0.00068863	0.00487053	0.03910506	0.49411717	A_07_P019845	b2592	cmr|heat shock protein [b2592]
clpP	+3.14	11.92	+1.65	11.09	0.1	12.74	0.5	0.00158294	0.00918269	0.02509210	0.41981934	A_07_P021300	b0437	cmr|ATP-dependent proteolytic subunit of clpA-clpP serine protease, heat shock protein F21.5 [b0437]
clpS	+3.12	10.36	+1.64	9.53	0.1	11.18	0.2	0.00019965	0.00199696	0.02509527	0.41942675	A_07_P004856	b0881	cmr|orf, hypothetical protein [b0881]
cmr	-2.62	8.04	-1.39	8.73	0.1	7.34	0.0	0.00023333	0.00223585	0.03105310	0.49433771	A_07_P004661	b0842	cmr|proton motive force efflux pump [b0842]
cobB	+2.10	8.76	+1.07	8.23	0.0	9.29	0.2	0.00201659	0.01112176	0.05705539	0.58367771	A_07_P052409	Z1761	cmr|putative nicotinic acid mononucleotide:5,6-dimethylbenzimidazole [Z1761]
cobS	-2.87	8.87	-1.52	9.63	0.1	8.11	0.1	0.00022283	0.00216294	0.02333079	0.44255422	A_07_P015292	b1992	cmr|cobalamin 5-phosphate synthase [b1992]
cobT	-2.03	10.00	-1.02	10.51	0.3	9.49	0.3	0.01214882	0.04594162	0.07191756	0.66761203	A_07_P015288	b1991	cmr|nicotinate-nucleotide dimethylbenzimidazole-P phophoribosyl transferase [b1991]
cobU	-2.55	9.40	-1.35	10.08	0.0	8.73	0.1	0.00033358	0.00288247	0.03461954	0.51371348	A_07_P015296	b1993	cmr|cobinamide kinase/cobinamide phosphate guanylyltransferase [b1993]
codA	+6.62	9.14	+2.73	7.77	0.0	10.50	0.1	0.00000558	0.00017942	0.00567264	0.21836186	A_07_P008378	b0337	cmr|cytosine deaminase [b0337]
codB	+5.22	8.05	+2.38	6.85	0.0	9.24	0.2	0.00001606	0.00033988	0.00934070	0.26884826	A_07_P031022	c_0455	cmr|Cytosine permease [c_0455]
codB	+2.43	7.83	+1.28	7.19	0.1	8.47	0.1	0.00064068	0.00463553	0.04142403	0.50517428	A_07_P008371	b0336	cmr|cytosine permease/transport [b0336]
cpdA	-2.35	12.63	-1.23	13.24	0.1	12.01	0.4	0.00404772	0.01920077	0.04463161	0.56485531	A_07_P012295	b3032	cmr|regulator of lacZ [b3032]
cpxR	+3.95	10.90	+1.98	9.91	0.0	11.89	0.1	0.00002594	0.00046513	0.01639037	0.34315160	A_07_P019340	b3912	cmr|transcriptional regulator in 2-component system [b3912]
cpxR	+3.71	11.29	+1.89	10.34	0.0	12.23	0.2	0.00006682	0.00090763	0.01777566	0.35202516	A_07_P041979	c_4864	cmr|Transcriptional Regulatory protein cpxR [c_4864]
creD	-4.05	9.08	-2.02	10.09	0.1	8.07	0.0	0.00002503	0.00045582	0.00663681	0.26386364	A_07_P017684	b4400	cmr|tolerance to colicin E2 [b4400]
crp	-2.20	13.02	-1.13	13.59	0.0	12.45	0.1	0.00091511	0.00611315	0.05619538	0.61270083	A_07_P017815	b3357	cmr|cyclic AMP receptor protein [b3357]
crr	+3.70	10.48	+1.89	9.54	0.0	11.42	0.1	0.00003538	0.00057430	0.01837525	0.35453431	A_07_P013145	b2417	cmr|PTS system, glucose-specific IIA component [b2417]
csdA	-3.46	11.22	-1.79	12.11	0.0	10.32	0.1	0.00006549	0.00089423	0.01180577	0.33672101	A_07_P006050	b2810	cmr|orf, hypothetical protein [b2810]
csgA	+2.25	8.06	+1.17	7.48	0.3	8.65	0.1	0.00297198	0.01507177	0.05052655	0.55823860	A_07_P040743	c_1306	cmr|Major curlin subunit precursor [c_1306]
csgB	+2.08	7.62	+1.06	7.09	0.1	8.15	0.1	0.00134995	0.00812449	0.05814787	0.58949131	A_07_P001417	b1041	cmr|minor curlin subunit precursor, similar ro CsgA [b1041]
csgG	-2.16	9.69	-1.11	10.24	0.3	9.13	0.1	0.00351460	0.01716843	0.05702553	0.61705155	A_07_P001396	b1037	cmr|curli production assembly/transport component, 2nd curli operon [b1037]
csiD	-2.79	9.28	-1.48	10.02	0.1	8.54	0.2	0.00038791	0.00324823	0.02570503	0.46251429	A_07_P020146	b2659	cmr|orf, hypothetical protein [b2659]
csiE	+5.07	8.18	+2.34	7.01	0.1	9.35	0.1	0.00001216	0.00028817	0.00986979	0.27357394	A_07_P019579	b2535	cmr|orf, hypothetical protein [b2535]
cspC	+4.40	14.27	+2.14	13.20	0.1	15.34	0.3	0.00011363	0.00131450	0.01287475	0.30573152	A_07_P015809	b1823	cmr|cold shock protein [b1823]
cspD	+51.91	10.78	+5.70	7.94	0.1	13.63	0.1	0.00000005	0.00003344	0.00001842	0.01035714	A_07_P004854	b0880	cmr|cold shock protein [b0880]
cspE	+6.64	12.97	+2.73	11.61	0.1	14.34	0.1	0.00000408	0.00015128	0.00572027	0.21859223	A_07_P035444	c_0714	cmr|Cold shock-like protein cspE [c_0714]
cspE	+6.47	12.77	+2.69	11.43	0.1	14.12	0.0	0.00000446	0.00016034	0.00604738	0.22561611	A_07_P009684	b0623	cmr|cold shock protein [b0623]
cspF	-2.05	9.10	-1.04	9.62	0.2	8.58	0.2	0.00485591	0.02230857	0.06663681	0.65001859	A_07_P020435	b1558	cmr|cold shock protein [b1558]
cstA	+11.23	10.57	+3.49	8.82	0.4	12.31	0.0	0.00000975	0.00024876	0.00203633	0.13193416	A_07_P016748	b0598	cmr|carbon starvation protein [b0598]
cusA	-2.26	8.54	-1.18	9.13	0.2	7.95	0.1	0.00106472	0.00683365	0.05040968	0.58615214	A_07_P031301	c_0661	cmr|Putative cation efflux system protein cusA [c_0661]
cusF	-2.29	9.60	-1.19	10.20	0.3	9.01	0.2	0.00467191	0.02160826	0.04792810	0.57910975	A_07_P016630	b0573	cmr|orf, hypothetical protein [b0573]
cvpA	+4.38	12.67	+2.13	11.60	0.1	13.73	0.1	0.00002579	0.00046404	0.01345528	0.31337278	A_07_P013855	b2313	cmr|membrane protein required for colicin V production [b2313]
cvpA	+3.91	10.80	+1.97	9.82	0.1	11.79	0.1	0.00003891	0.00061546	0.01663808	0.34512516	A_07_P042620	Z3575	cmr|membrane protein required for colicin V production [Z3575]
cvrA	-3.84	12.52	-1.94	13.49	0.2	11.55	0.4	0.00075194	0.00523364	0.00868140	0.30238938	A_07_P006906	b1191	cmr|orf, hypothetical protein [b1191]
cyaY	+3.08	10.53	+1.62	9.72	0.1	11.34	0.1	0.00010528	0.00124621	0.02562436	0.42466316	A_07_P005904	b3807	cmr|orf, hypothetical protein [b3807]
cyaY	+2.64	11.58	+1.40	10.87	0.1	12.28	0.1	0.00031238	0.00275682	0.03446011	0.47218451	A_07_P041940	c_4726	cmr|CyaY protein [c_4726]
cybC	+2.75	11.70	+1.46	10.97	0.2	12.43	0.0	0.00032379	0.00282385	0.03205030	0.45998177	A_07_P037063	c_5335	cmr|Soluble cytochrome b562 precursor [c_5335]
cybC	+3.51	10.61	+1.81	9.71	0.0	11.52	0.1	0.00006700	0.00090852	0.02023819	0.37397887	A_07_P003731	b4236	cmr|cytochrome b [b4236]
cydA	+26.33	12.60	+4.72	10.24	0.0	14.96	0.1	0.00000013	0.00003548	0.00021532	0.04035714	A_07_P010185	b0733	cmr|cytochrome d terminal oxidase, polypeptide subunit I [b0733]
cydB	+24.44	12.42	+4.61	10.11	0.1	14.72	0.0	0.00000013	0.00003548	0.00026105	0.04566667	A_07_P010187	b0734	cmr|cytochrome d terminal oxidase polypeptide subunit II [b0734]
cydC	+2.05	9.15	+1.03	8.63	0.2	9.67	0.1	0.00332157	0.01637796	0.06186865	0.60575871	A_07_P004879	b0886	cmr|ATP-binding component of cytochrome-related transport [b0886]
cyoA	+2.08	11.89	+1.05	11.37	0.0	12.42	0.2	0.00189423	0.01056400	0.05965447	0.59669632	A_07_P021271	b0432	cmr|cytochrome o ubiquinol oxidase subunit II [b0432]
cyoB	+4.95	14.28	+2.31	13.12	0.0	15.43	0.1	0.00001507	0.00032455	0.01042048	0.28044444	A_07_P021268	b0431	cmr|cytochrome o ubiquinol oxidase subunit I [b0431]
cyoC	+3.96	12.97	+1.99	11.98	0.0	13.96	0.2	0.00006373	0.00087552	0.01585874	0.33970068	A_07_P021261	b0430	cmr|cytochrome o ubiquinol oxidase subunit III [b0430]
cyoD	+4.07	8.72	+2.02	7.71	0.2	9.74	0.4	0.00029483	0.00264941	0.01498730	0.33001399	A_07_P051941	Z0532	cmr|cytochrome o ubiquinol oxidase subunit IV [Z0532]
cyoD	+4.28	11.50	+2.10	10.45	0.1	12.55	0.0	0.00002050	0.00039839	0.01411077	0.32150507	A_07_P021258	b0429	cmr|cytochrome o ubiquinol oxidase subunit IV [b0429]
cyoE	+4.46	10.84	+2.16	9.77	0.0	11.92	0.1	0.00001743	0.00036012	0.01286395	0.30593656	A_07_P021254	b0428	cmr|protoheme IX farnesyltransferase [b0428]
cysA	-3.63	9.25	-1.86	10.18	0.2	8.32	0.0	0.00006324	0.00087038	0.01016451	0.32526423	A_07_P013166	b2422	cmr|ATP-binding component of sulfate permease A protein; chromate resistance [b2422]
cysC	-4.89	9.30	-2.29	10.44	0.0	8.15	0.1	0.00001606	0.00033988	0.00338415	0.19445255	A_07_P002196	b2750	cmr|adenosine 5-phosphosulfate kinase [b2750]
cysD	-3.14	9.68	-1.65	10.51	0.1	8.86	0.0	0.00010296	0.00122803	0.01705030	0.37755274	A_07_P002209	b2752	cmr|ATP:sulfurylase [b2752]
cysH	-4.81	10.62	-2.27	11.76	0.0	9.49	0.0	0.00001128	0.00027354	0.00354929	0.19676056	A_07_P002256	b2762	cmr|3-phosphoadenosine 5-phosphosulfate reductase [b2762]
cysI	-6.09	11.43	-2.61	12.74	0.0	10.13	0.0	0.00000472	0.00016577	0.00153392	0.12777778	A_07_P002265	b2763	cmr|sulfite reductase, alpha subunit [b2763]
cysJ	-7.07	10.82	-2.82	12.23	0.0	9.41	0.1	0.00000466	0.00016486	0.00092289	0.09751678	A_07_P032576	c_3323	cmr|Sulfite reductase [c_3323]
cysJ	-2.20	10.01	-1.14	10.58	0.0	9.44	0.2	0.00138152	0.00824826	0.05419779	0.60517021	A_07_P002266	b2764	cmr|sulfite reductase [b2764]
cysM	-2.15	8.66	-1.10	9.21	0.2	8.11	0.2	0.00223180	0.01202925	0.06018801	0.62963455	A_07_P053391	ECs3292	cmr|cysteine synthase B [ECs3292]
cysM	-2.15	8.69	-1.10	9.24	0.4	8.14	0.1	0.00854094	0.03459441	0.05778265	0.61886395	A_07_P042806	Z3686	cmr|cysteine synthase B, O-acetylserine sulfhydrolase B [Z3686]
cysM	-2.34	10.12	-1.23	10.74	0.1	9.51	0.2	0.00094277	0.00624679	0.04507940	0.56823859	A_07_P030051	c_2955	cmr|Cysteine synthase B [c_2955]
cysN	-2.28	7.97	-1.19	8.56	0.2	7.38	0.1	0.00136899	0.00819026	0.05036268	0.58647189	A_07_P002205	b2751	cmr|ATP-sulfurylase [b2751]
cysN	-4.36	9.17	-2.12	10.24	0.1	8.11	0.0	0.00001934	0.00038447	0.00501778	0.22965116	A_07_P043152	Z4059	cmr|ATP-sulfurylase [Z4059]
cysN	-5.61	9.58	-2.49	10.83	0.1	8.34	0.2	0.00001396	0.00030964	0.00206809	0.14867580	A_07_P030710	c_3318	cmr|Sulfate adenylyltransferase subunit 1 [c_3318]
cysN	-4.81	9.20	-2.26	10.33	0.0	8.07	0.2	0.00003227	0.00054107	0.00356644	0.19701754	A_07_P056037	ECs3605	cmr|ATP-sulfurylase [ECs3605]
cysP	-5.22	11.50	-2.38	12.70	0.0	10.31	0.1	0.00001598	0.00033988	0.00263783	0.17090535	A_07_P013181	b2425	cmr|thiosulfate binding protein [b2425]
cysP	-7.71	12.29	-2.95	13.77	0.0	10.82	0.1	0.00000283	0.00012340	0.00063770	0.08032000	A_07_P030063	c_2959	cmr|Thiosulfate-binding protein precursor [c_2959]
cysP	-5.78	10.96	-2.53	12.23	0.0	9.70	0.0	0.00000556	0.00017942	0.00187564	0.14334951	A_07_P042814	Z3690	cmr|thiosulfate binding protein [Z3690]
cysU	-5.67	11.71	-2.50	12.96	0.1	10.46	0.1	0.00001145	0.00027642	0.00206872	0.14804545	A_07_P013177	b2424	cmr|sulfate, thiosulfate transport system permease T protein [b2424]
cysU	-5.87	10.91	-2.55	12.19	0.0	9.64	0.1	0.00000837	0.00022676	0.00181847	0.14103448	A_07_P042582	c_2958	cmr|Sulfate transport system permease protein cysT [c_2958]
cysW	-13.71	10.84	-3.78	12.73	0.0	8.95	0.1	0.00000060	0.00005750	0.00004700	0.02114286	A_07_P013174	b2423	cmr|sulfate transport system permease W protein [b2423]
dadA	+11.46	10.07	+3.52	8.31	0.0	11.83	0.0	0.00000066	0.00006014	0.00192581	0.13012876	A_07_P006899	b1189	cmr|D-amino acid dehydrogenase subunit [b1189]
dadA	+9.93	10.24	+3.31	8.58	0.1	11.89	0.0	0.00000167	0.00009634	0.00258067	0.14882784	A_07_P038722	c_1638	cmr|D-amino acid dehydrogenase small subunit [c_1638]
dadX	+6.51	9.56	+2.70	8.21	0.0	10.91	0.1	0.00000566	0.00018051	0.00588796	0.22177033	A_07_P006905	b1190	cmr|alanine racemase 2, catabolic [b1190]
dam	-2.42	9.57	-1.28	10.20	0.1	8.93	0.4	0.00513878	0.02328200	0.04073996	0.54356780	A_07_P017965	b3387	cmr|DNA adenine methylase [b3387]
dam	-2.03	9.76	-1.02	10.27	0.0	9.25	0.3	0.00442541	0.02065627	0.07177083	0.66703660	A_07_P017965	b3387	cmr|DNA adenine methylase [b3387]
damX	-3.09	10.70	-1.63	11.52	0.0	9.89	0.1	0.00008622	0.00108122	0.01774327	0.38637621	A_07_P017966	b3388	cmr|putative membrane protein; interferes with cell division [b3388]
dapA	+2.99	11.55	+1.58	10.76	0.0	12.34	0.0	0.00009977	0.00119993	0.02699886	0.42806647	A_07_P013447	b2478	cmr|dihydrodipicolinate synthase [b2478]
dapB	+3.98	9.39	+1.99	8.39	0.0	10.39	0.1	0.00003200	0.00053978	0.01626397	0.34324397	A_07_P010842	b0031	cmr|dihydrodipicolinate reductase [b0031]
dapD	+2.74	8.62	+1.46	7.89	0.0	9.34	0.2	0.00030152	0.00269055	0.03216717	0.45873188	A_07_P041442	c_0201	cmr|2,3,4,5-tetrahydropyridine-2,6-dicarboxylate N-succinyltransferase [c_0201]
dapD	+2.15	8.02	+1.11	7.47	0.1	8.57	0.1	0.00106350	0.00682858	0.05388084	0.56818486	A_07_P002786	b0166	cmr|2,3,4,5-tetrahydropyridine-2-carboxylate N-succinyltransferase [b0166]
dapE	-4.66	10.84	-2.22	11.95	0.1	9.73	0.2	0.00002419	0.00044487	0.00402693	0.20855263	A_07_P013420	b2472	cmr|N-succinyl-diaminopimelate deacylase [b2472]
dcd	-4.68	12.11	-2.23	13.22	0.0	10.99	0.3	0.00008551	0.00107531	0.00418318	0.21108974	A_07_P003102	b2065	cmr|2-deoxycytidine 5-triphosphate deaminase [b2065]
dcm	-2.00	9.96	-1.00	10.46	0.0	9.46	0.3	0.00676386	0.02889830	0.07398120	0.67405093	A_07_P015161	b1961	cmr|DNA cytosine methylase [b1961]
DCP_20_0	-2.12	9.93	-1.09	10.47	0.2	9.38	0.1	0.00241704	0.01278694	0.06158092	0.63575738	DCP_20_0	DCP_20_0	
DCP_20_1	-2.71	9.95	-1.44	10.67	0.0	9.23	0.0	0.00017443	0.00181154	0.02795605	0.47531317	DCP_20_1	DCP_20_1	
DCP_20_5	-2.18	10.08	-1.13	10.64	0.2	9.52	0.1	0.00267228	0.01381689	0.05707635	0.61633059	DCP_20_5	DCP_20_5	
DCP_20_5	-2.44	10.23	-1.29	10.87	0.2	9.59	0.1	0.00113965	0.00715494	0.03895706	0.53520070	DCP_20_5	DCP_20_5	
DCP_22_0	-2.57	10.57	-1.36	11.25	0.1	9.89	0.1	0.00036260	0.00307752	0.03434896	0.51114367	DCP_22_0	DCP_22_0	
DCP_22_0	-2.01	10.62	-1.01	11.12	0.1	10.12	0.1	0.00265337	0.01375978	0.07364647	0.67255800	DCP_22_0	DCP_22_0	
DCP_22_2	-2.31	10.31	-1.21	10.91	0.3	9.70	0.0	0.00180305	0.01018193	0.04759146	0.57681293	DCP_22_2	DCP_22_2	
DCP_22_2	-2.06	10.29	-1.04	10.82	0.3	9.77	0.2	0.00461667	0.02140307	0.06740346	0.65264453	DCP_22_2	DCP_22_2	
DCP_22_4	-2.28	10.39	-1.19	10.99	0.2	9.80	0.0	0.00085239	0.00580198	0.04939660	0.58517682	DCP_22_4	DCP_22_4	
DCP_22_6	-2.44	10.54	-1.29	11.18	0.3	9.89	0.2	0.00359638	0.01750326	0.03932736	0.53747396	DCP_22_6	DCP_22_6	
DCP_22_6	-2.61	10.34	-1.38	11.03	0.1	9.64	0.1	0.00046602	0.00371502	0.03179243	0.49854582	DCP_22_6	DCP_22_6	
DCP_22_9	-2.03	8.88	-1.02	9.39	0.1	8.37	0.0	0.00141447	0.00839404	0.07215701	0.66825882	DCP_22_9	DCP_22_9	
dctA	+17.92	10.54	+4.16	8.46	0.1	12.62	0.1	0.00000044	0.00005132	0.00067264	0.07618705	A_07_P007574	b3528	cmr|uptake of C4-dicarboxylic acids [b3528]
dcuA	+6.84	10.77	+2.77	9.38	0.1	12.16	0.1	0.00000897	0.00023724	0.00526994	0.20794486	A_07_P011423	b4138	cmr|anaerobic dicarboxylate transport [b4138]
dcuB	-2.74	8.24	-1.45	8.96	0.0	7.51	0.0	0.00016418	0.00173600	0.02766197	0.47389554	A_07_P011354	b4123	cmr|anaerobic dicarboxylate transport [b4123]
dcuC	+2.66	8.22	+1.41	7.52	0.0	8.93	0.2	0.00042819	0.00350235	0.03434388	0.47100174	A_07_P009671	b0621	cmr|transport of dicarboxylates [b0621]
dcyD	-4.69	11.60	-2.23	12.72	0.0	10.49	0.1	0.00001255	0.00029182	0.00384972	0.20407407	A_07_P014958	b1919	cmr|putative 1-aminocyclopropane-1-carboxylate deaminase [b1919]
ddl	-2.87	11.61	-1.52	12.37	0.0	10.85	0.1	0.00013644	0.00151031	0.02329332	0.44291063	A_07_P062950	ECs0431	cmr|D-alanine-D-alanine ligase A [ECs0431]
ddl	-2.72	11.52	-1.44	12.24	0.0	10.79	0.2	0.00049667	0.00388070	0.02782330	0.47459372	A_07_P051920	Z0477	cmr|putative enzyme Cell envelop: Murein sacculus, peptidoglycan [Z0477]
ddlA	-4.34	9.76	-2.12	10.82	0.1	8.70	0.2	0.00005115	0.00074921	0.00532139	0.23868946	A_07_P021019	b0381	cmr|D-alanine-D-alanine ligase A [b0381]
deaD	-20.31	11.22	-4.34	13.39	0.0	9.04	0.0	0.00000018	0.00003706	0.00001651	0.01181818	A_07_P000534	b3162	cmr|inducible ATP-independent RNA helicase [b3162]
degQ	+2.84	8.56	+1.51	7.80	0.0	9.31	0.5	0.00284168	0.01453049	0.03124238	0.45251150	A_07_P041306	c_3989	cmr|Protease degQ precursor [c_3989]
degS	-4.40	9.25	-2.14	10.31	0.1	8.18	0.1	0.00003056	0.00052302	0.00502604	0.22936232	A_07_P034177	c_3990	cmr|Protease degS precursor [c_3990]
deoB	+3.64	10.84	+1.86	9.91	0.1	11.77	0.7	0.00449220	0.02091844	0.01964558	0.36777646	A_07_P017598	b4383	cmr|phosphopentomutase [b4383]
deoC	-9.20	14.20	-3.20	15.80	0.0	12.60	0.3	0.00000557	0.00017942	0.00031314	0.05417582	A_07_P017589	b4381	cmr|2-deoxyribose-5-phosphate aldolase [b4381]
deoD	+2.18	11.16	+1.12	10.60	0.0	11.73	0.0	0.00173850	0.00989906	0.05220147	0.56368999	A_07_P017603	b4384	cmr|purine-nucleoside phosphorylase [b4384]
deoR	-9.26	10.13	-3.21	11.74	0.1	8.53	0.0	0.00000150	0.00009204	0.00027248	0.05362500	A_07_P042124	c_0925	cmr|Deoxyribose operon repressor [c_0925]
deoR	-10.72	10.63	-3.42	12.34	0.1	8.92	0.0	0.00000089	0.00006827	0.00013338	0.03750000	A_07_P004651	b0840	cmr|transcriptional repressor for deo operon, tsx, nupG [b0840]
dhaK	+8.14	9.42	+3.03	7.91	0.1	10.94	0.2	0.00000489	0.00016794	0.00386369	0.17997041	A_07_P006953	b1200	cmr|putative dihydroxyacetone kinase [b1200]
dhaL	+4.48	8.06	+2.16	6.98	0.1	9.14	0.1	0.00002548	0.00046116	0.01269436	0.30466463	A_07_P006948	b1199	cmr|putative dihydroxyacetone kinase [b1199]
dhaM	+2.01	7.32	+1.01	6.82	0.1	7.83	0.1	0.00181153	0.01022251	0.06215130	0.60626394	A_07_P006942	b1198	cmr|putative PTS system enzyme I [b1198]
diaA	+3.19	9.55	+1.67	8.72	0.1	10.39	0.1	0.00008879	0.00109894	0.02376334	0.41113187	A_07_P000466	b3149	cmr|orf, hypothetical protein [b3149]
dinB	-3.00	12.15	-1.59	12.95	0.1	11.36	0.2	0.00029614	0.00265821	0.01939151	0.39908497	A_07_P007867	b0231	cmr|damage-inducible protein P; putative tRNA synthetase [b0231]
dinF	-3.18	10.09	-1.67	10.93	0.0	9.26	0.4	0.00085738	0.00582084	0.01554624	0.37367939	A_07_P008863	b4044	cmr|DNA-damage-inducible protein F [b4044]
dinG	+6.29	11.53	+2.65	10.20	0.1	12.85	0.1	0.00000693	0.00020202	0.00627858	0.22829099	A_07_P004448	b0799	cmr|probably ATP-dependent helicase [b0799]
dinI	-2.00	14.44	-1.00	14.94	0.0	13.95	0.1	0.00197427	0.01094470	0.07536649	0.67726598	A_07_P001507	b1061	cmr|damage-inducible protein I [b1061]
dipZ	+2.46	8.32	+1.30	7.67	0.1	8.97	0.1	0.00044419	0.00358698	0.04003938	0.49714511	A_07_P011411	b4136	cmr|thiol:disulfide interchange protein; copper tolerance [b4136]
djlB	-4.06	11.77	-2.02	12.78	0.1	10.76	0.2	0.00005953	0.00083457	0.00663681	0.26453165	A_07_P009800	b0646	cmr|putative enzyme of polynucleotide modification [b0646]
dkgA	+4.33	9.05	+2.11	7.99	0.0	10.10	0.1	0.00002006	0.00039347	0.01367696	0.31712813	A_07_P012193	b3012	cmr|orf, hypothetical protein [b3012]
dkgB	-3.51	9.01	-1.81	9.91	0.1	8.10	0.2	0.00010672	0.00125952	0.01115917	0.33024436	A_07_P002965	b0207	cmr|putative aldose reductase [b0207]
dmsA	+10.08	10.48	+3.33	8.81	0.2	12.15	0.1	0.00000204	0.00010341	0.00249174	0.14748120	A_07_P033816	c_1031	cmr|Anaerobic dimethyl sulfoxide reductase chain A precursor [c_1031]
dmsA	+6.29	8.47	+2.65	7.15	0.1	9.80	0.5	0.00010862	0.00127246	0.00665269	0.23223947	A_07_P004916	b0894	cmr|anaerobic dimethyl sulfoxide reductase subunit A [b0894]
dmsB	+7.43	8.93	+2.89	7.49	0.1	10.38	0.2	0.00000495	0.00016913	0.00444741	0.19079019	A_07_P004924	b0895	cmr|anaerobic dimethyl sulfoxide reductase subunit B [b0895]
dmsC	+4.74	8.27	+2.25	7.15	0.1	9.40	0.1	0.00001648	0.00034504	0.01153519	0.29291935	A_07_P052033	Z1242	cmr|anaerobic dimethyl sulfoxide reductase subunit C [Z1242]
dmsC	+5.61	8.62	+2.49	7.37	0.0	9.86	0.0	0.00000631	0.00019141	0.00797066	0.24948310	A_07_P004926	b0896	cmr|anaerobic dimethyl sulfoxide reductase subunit C [b0896]
dmsD	+2.31	8.38	+1.21	7.78	0.1	8.99	0.1	0.00058284	0.00434069	0.04584350	0.52914956	A_07_P020586	b1591	cmr|putative oxidoreductase component [b1591]
dnaA	-2.08	14.01	-1.06	14.54	0.0	13.48	0.1	0.00125929	0.00772048	0.06628303	0.65181761	A_07_P005441	b3702	cmr|DNA biosynthesis; initiation of chromosome replication; can be transcription regulator [b3702]
dnaB	-2.40	9.25	-1.26	9.88	0.0	8.62	0.2	0.00109941	0.00698512	0.04252350	0.55651704	A_07_P008903	b4052	cmr|replicative DNA helicase; part of primosome [b4052]
dnaG	-6.66	11.54	-2.74	12.91	0.0	10.17	0.0	0.00000493	0.00016890	0.00114583	0.11000000	A_07_P000065	b3066	cmr|DNA biosynthesis; DNA primase [b3066]
dnaK	+2.48	11.33	+1.31	10.68	0.2	11.99	0.2	0.00097072	0.00639188	0.03995617	0.49728854	A_07_P010763	b0014	cmr|chaperone Hsp70; DNA biosynthesis; autoregulated heat shock proteins [b0014]
dnaN	-3.38	13.17	-1.76	14.05	0.0	12.29	0.0	0.00005092	0.00074790	0.01293191	0.34333895	A_07_P005436	b3701	cmr|DNA polymerase III, beta-subunit [b3701]
dnaQ	+2.08	8.90	+1.06	8.37	0.0	9.43	0.4	0.00965485	0.03817948	0.05988186	0.59745247	A_07_P051886	Z0241	cmr|DNA polymerase III, epsilon subunit [Z0241]
dnaQ	+3.06	9.80	+1.61	8.99	0.1	10.60	0.1	0.00019948	0.00199659	0.02617378	0.42658385	A_07_P003004	b0215	cmr|DNA polymerase III, epsilon subunit [b0215]
dnaX	+4.90	10.90	+2.29	9.76	0.1	12.05	0.2	0.00003296	0.00054976	0.01048336	0.28069728	A_07_P021457	b0470	cmr|DNA polymerase III, tau and gamma subunits; DNA elongation factor III [b0470]
dos	-2.05	7.51	-1.03	8.01	0.1	6.99	0.2	0.00294427	0.01495309	0.06897675	0.65776499	A_07_P009534	b1489	cmr|putative enzyme [b1489]
dpiB	-3.24	9.44	-1.70	10.28	0.4	8.59	0.3	0.00075337	0.00524407	0.01490981	0.36735524	A_07_P009663	b0619	cmr|putative sensor-type protein [b0619]
dppA	+2.46	8.03	+1.30	7.39	0.1	8.68	0.1	0.00050443	0.00392186	0.04005335	0.49692671	A_07_P007652	b3544	cmr|dipeptide transport protein [b3544]
dppD	+2.11	8.54	+1.07	8.01	0.1	9.08	0.1	0.00438886	0.02053437	0.05703125	[illegible]	A_07_P009583	c_4356	cmr|Dipeptide transport ATP-binding protein dppD [c_4356]
dppD	+2.29	8.07	+1.20	7.47	0.0	8.67	0.1	0.00050820	0.00393965	0.04658854	0.53383552	A_07_P007640	b3541	cmr|putative ATP-binding component of dipeptide transport system [b3541]
dppF	-2.46	8.24	-1.30	8.89	0.0	7.59	0.1	0.00393689	0.01876518	[illegible]	0.53194908	A_07_P007633	b3540	cmr|putative ATP-binding component of dipeptide transport system [b3540]
dps	+2.36	11.82	+1.24	10.64	0.1	13.00	0.0	[illegible]	[illegible]	[illegible]	[illegible]	A_07_P004515	b0812	cmr|global regulator, starvation conditions [b0812]
dsbA	+6.22	9.77	+2.64	8.45	0.1	11.09	0.1	0.00000606	0.00018784	0.00650089	0.23156109	A_07_P019081	b3860	cmr|protein disulfide isomerase I, essential for cytochrome c synthesis and formate-dependent reduction [b3860]

dsdX	+3.74	7.90	+1.90	6.95	0.3	8.85	0.2	0.00030662	0.00272199	0.01810658	0.35412422	A_07_P014107	b2365	cmr|transport system permease [b2365]
dtd	+4.22	10.05	+2.08	9.02	0.3	11.09	0.2	0.00017668	0.00183002	0.01427020	0.32280172	A_07_P019212	b3887	cmr|orf, hypothetical protein [b3887]
dusA	-2.04	8.81	-1.03	9.33	0.1	8.30	0.2	0.00401449	0.01906612	0.07009909	0.66125824	A_07_P008886	b4049	cmr|orf, hypothetical protein [b4049]
dusB	+2.52	11.61	+1.33	10.94	0.1	12.27	0.4	0.00279414	0.01431532	0.04033981	0.49930031	A_07_P012768	b3260	cmr|putative dehydrogenase [b3260]
E1A_r60_a104	-2.24	10.45	-1.17	11.04	0.3	9.87	0.3	0.00549885	0.02459485	0.05149708	0.59266813	(+)E1A_r60_a104	E1A_r60_a104	
E1A_r60_a104	-2.48	9.33	-1.31	9.99	0.4	8.68	0.0	0.00287579	0.01466208	0.03674289	0.52303797	(+)E1A_r60_a104	E1A_r60_a104	
E1A_r60_a104	-2.12	10.05	-1.08	10.59	0.0	9.51	0.2	0.00179384	0.01014814	0.06197027	0.63685379	(+)E1A_r60_a104	E1A_r60_a104	
E1A_r60_a104	-2.29	9.60	-1.19	10.19	0.5	9.00	0.0	0.00920517	0.03680429	0.04665396	0.57474178	(+)E1A_r60_a104	E1A_r60_a104	
E1A_r60_a104	-4.50	9.92	-2.17	11.01	0.3	8.84	0.1	0.00006152	0.00085336	0.00470084	0.22564024	(+)E1A_r60_a104	E1A_r60_a104	
E1A_r60_a104	-3.35	9.45	-1.74	10.32	0.1	8.57	0.1	0.00007119	0.00094658	0.01347497	0.34835796	(+)E1A_r60_a104	E1A_r60_a104	
E1A_r60_a104	-2.79	9.65	-1.48	10.39	0.6	8.91	0.1	0.00808799	0.03323866	0.02393928	0.44709371	(+)E1A_r60_a104	E1A_r60_a104	
E1A_r60_a104	-2.50	9.74	-1.32	10.40	0.4	9.08	0.4	0.00918236	0.03674987	0.03418445	0.51111111	(+)E1A_r60_a104	E1A_r60_a104	
E1A_r60_a104	-2.14	10.02	-1.10	10.57	0.1	9.48	0.5	0.01343664	0.04994015	0.05805513	0.61925474	(+)E1A_r60_a104	E1A_r60_a104	
E1A_r60_a104	-2.41	10.06	-1.27	10.70	0.3	9.43	0.4	0.00745333	0.03116738	0.03979230	0.54101036	(+)E1A_r60_a104	E1A_r60_a104	
E1A_r60_a107	-3.38	9.63	-1.76	10.51	0.3	8.76	0.2	0.00035734	0.00303839	0.01306974	0.34525168	(+)E1A_r60_a107	E1A_r60_a107	
E1A_r60_a107	-2.08	8.70	-1.06	9.23	0.2	8.18	0.1	0.00180672	0.01019897	0.06546875	0.65030915	(+)E1A_r60_a107	E1A_r60_a107	
E1A_r60_a107	-2.28	9.30	-1.19	9.89	0.5	8.70	0.1	0.01031189	0.04026417	0.04843432	0.58121189	(+)E1A_r60_a107	E1A_r60_a107	
E1A_r60_a107	-2.30	8.69	-1.20	9.29	0.5	8.09	0.0	0.00793262	0.03271972	0.04737170	0.57636785	(+)E1A_r60_a107	E1A_r60_a107	
E1A_r60_a107	-2.22	8.95	-1.15	9.52	0.4	8.37	0.3	0.01305239	0.04874212	0.05164444	0.59263120	(+)E1A_r60_a107	E1A_r60_a107	
E1A_r60_a107	-2.18	9.09	-1.12	9.65	0.3	8.52	0.2	0.00415479	0.01962000	0.05514736	0.60843728	(+)E1A_r60_a107	E1A_r60_a107	
E1A_r60_a135	-2.29	8.92	-1.20	9.52	0.1	8.32	0.0	0.00068350	0.00484533	0.04869347	0.58298859	(+)E1A_r60_a135	E1A_r60_a135	
E1A_r60_a135	-2.34	9.12	-1.23	9.73	0.1	8.50	0.1	0.00060655	0.00446243	0.04523183	0.56879393	(+)E1A_r60_a135	E1A_r60_a135	
E1A_r60_a135	-3.42	9.41	-1.78	10.30	0.7	8.52	0.1	0.00533795	0.02401652	0.01233549	0.33658579	(+)E1A_r60_a135	E1A_r60_a135	
E1A_r60_a135	-3.11	9.48	-1.64	10.30	0.1	8.66	0.3	0.00042917	0.00350320	0.01703252	0.37875706	(+)E1A_r60_a135	E1A_r60_a135	
E1A_r60_a135	-2.78	9.02	-1.47	9.76	0.2	8.28	0.0	0.00032175	0.00281267	0.02548590	0.46014908	(+)E1A_r60_a135	E1A_r60_a135	
E1A_r60_a20	-2.00	10.16	-1.00	10.67	0.3	9.66	0.2	0.00848181	0.03439917	0.07558117	0.67764806	(+)E1A_r60_a20	E1A_r60_a20	
E1A_r60_a20	-2.87	10.11	-1.52	10.87	0.3	9.35	0.2	0.00100870	0.00655968	0.02311611	0.44060533	(+)E1A_r60_a20	E1A_r60_a20	
E1A_r60_a20	-2.11	10.11	-1.07	10.64	0.1	9.57	0.1	0.00179503	0.01015122	0.06356898	0.64611362	(+)E1A_r60_a20	E1A_r60_a20	
E1A_r60_a20	-2.35	9.79	-1.23	10.41	0.1	9.18	0.1	0.00048937	0.00384271	0.04474784	0.56587149	(+)E1A_r60_a20	E1A_r60_a20	
E1A_r60_a20	-2.18	9.83	-1.12	10.39	0.2	9.27	0.0	0.00186484	0.01045585	0.05736979	0.61569870	(+)E1A_r60_a20	E1A_r60_a20	
E1A_r60_a20	-2.15	9.95	-1.10	10.50	0.1	9.40	0.3	0.00359537	0.01750326	0.05993267	0.62905333	(+)E1A_r60_a20	E1A_r60_a20	
E1A_r60_a20	-2.57	10.21	-1.36	10.89	0.1	9.53	0.5	0.00469711	0.02170570	0.03338351	0.50586141	(+)E1A_r60_a20	E1A_r60_a20	
E1A_r60_a20	-2.58	9.96	-1.37	10.64	0.3	9.28	0.1	0.00091569	0.00611315	0.03264037	0.50579724	(+)E1A_r60_a20	E1A_r60_a20	
E1A_r60_a20	-2.58	10.13	-1.37	10.82	0.3	9.45	0.0	0.00092067	0.00613938	0.03309134	0.50630709	(+)E1A_r60_a20	E1A_r60_a20	
E1A_r60_a20	-2.62	10.18	-1.39	10.88	0.0	9.49	0.2	0.00048598	0.00382757	0.03088288	0.49412602	(+)E1A_r60_a20	E1A_r60_a20	
E1A_r60_a22	-2.11	10.92	-1.08	11.46	0.1	10.38	0.2	0.00288096	0.01467889	0.06233168	0.63889974	(+)E1A_r60_a22	E1A_r60_a22	
E1A_r60_a22	-2.14	10.70	-1.10	11.24	0.0	10.15	0.0	0.00078308	0.00539515	0.06089177	0.63404762	(+)E1A_r60_a22	E1A_r60_a22	
E1A_r60_a22	-2.66	10.80	-1.41	11.50	0.4	10.09	0.1	0.00253371	0.01328788	0.02922701	0.48082550	(+)E1A_r60_a22	E1A_r60_a22	
E1A_r60_a22	-2.55	10.87	-1.35	11.54	0.3	10.19	0.1	0.00108689	0.00692964	0.03473704	0.51207865	(+)E1A_r60_a22	E1A_r60_a22	
E1A_r60_a22	-2.48	10.56	-1.31	11.21	0.1	9.90	0.1	0.00049826	0.00389116	0.03801575	0.52872792	(+)E1A_r60_a22	E1A_r60_a22	
E1A_r60_a22	-2.33	10.73	-1.22	11.34	0.2	10.12	0.2	0.00161925	0.00936916	0.04618585	0.57301024	(+)E1A_r60_a22	E1A_r60_a22	
E1A_r60_a22	-2.75	10.78	-1.46	11.51	0.3	10.05	0.1	0.00113185	0.00712615	0.02657457	0.46904709	(+)E1A_r60_a22	E1A_r60_a22	
E1A_r60_a22	-2.35	10.65	-1.23	11.26	0.0	10.03	0.2	0.00115015	0.00720286	0.04533346	0.56870916	(+)E1A_r60_a22	E1A_r60_a22	
E1A_r60_a22	-2.43	10.63	-1.28	11.27	0.1	9.99	0.1	0.00057885	0.00431914	0.04037221	0.54326496	(+)E1A_r60_a22	E1A_r60_a22	
E1A_r60_a22	-2.31	10.65	-1.21	11.26	0.2	10.05	0.1	0.00153628	0.00896468	0.04747840	0.57632999	(+)E1A_r60_a22	E1A_r60_a22	
E1A_r60_a22	-2.68	10.60	-1.42	11.31	0.2	9.89	0.0	0.00051447	0.00396851	0.02912919	0.47971757	(+)E1A_r60_a22	E1A_r60_a22	
E1A_r60_a22	-2.85	10.59	-1.51	11.35	0.1	9.83	0.0	0.00018056	0.00186075	0.02369728	0.44415476	(+)E1A_r60_a22	E1A_r60_a22	
E1A_r60_a22	-2.92	10.71	-1.54	11.49	0.1	9.94	0.1	0.00020795	0.00205389	0.02156504	0.42176398	(+)E1A_r60_a22	E1A_r60_a22	
E1A_r60_a22	-2.23	10.66	-1.16	11.24	0.1	10.08	0.3	0.00218366	0.01183460	0.05400343	0.60471550	(+)E1A_r60_a22	E1A_r60_a22	
E1A_r60_a22	-2.03	10.73	-1.02	11.24	0.2	10.22	0.0	0.00297812	0.01509091	0.07065739	0.66413731	(+)E1A_r60_a22	E1A_r60_a22	
E1A_r60_a22	-2.10	10.65	-1.07	11.19	0.0	10.12	0.2	0.00163574	0.00943339	0.06341209	0.64535229	(+)E1A_r60_a22	E1A_r60_a22	
E1A_r60_a22	-2.57	10.65	-1.36	11.33	0.1	9.97	0.1	0.00028436	0.00258935	0.03293445	0.50587317	(+)E1A_r60_a22	E1A_r60_a22	
E1A_r60_a97	-2.23	7.83	-1.16	8.41	0.3	7.25	0.1	0.00422149	0.01988127	0.05278328	0.59785612	(+)E1A_r60_a97	E1A_r60_a97	
E1A_r60_a97	-2.08	7.99	-1.06	8.52	0.1	7.46	0.1	0.00133391	0.00804329	0.06567200	0.65150599	(+)E1A_r60_a97	E1A_r60_a97	
E1A_r60_a97	-2.68	8.09	-1.42	8.81	0.2	7.38	0.0	0.00052493	0.00401973	0.02876270	0.47919577	(+)E1A_r60_a97	E1A_r60_a97	
E1A_r60_n11	+2.22	7.76	+1.15	7.19	0.2	8.33	0.0	0.00103009	0.00666690	0.05065549	0.55809657	(+)E1A_r60_n11	E1A_r60_n11	
E1A_r60_n11	+2.84	8.12	+1.51	7.37	0.2	8.87	0.4	0.00305345	0.01534424	0.03090955	0.45059259	(+)E1A_r60_n11	E1A_r60_n11	
E1A_r60_n11	-3.29	8.15	-1.72	9.01	0.2	7.29	0.2	0.00029090	0.00262909	0.01407139	0.35674718	(+)E1A_r60_n11	E1A_r60_n11	
eaeH	-2.58	12.20	-1.37	12.89	0.1	11.52	0.1	0.00035854	0.00304634	0.03294842	0.50559454	A_07_P008177	b0297	cmr|attaching and effacing protein, pathogenesis factor [b0297]
ebgR	+2.22	9.20	+1.15	8.62	0.3	9.77	0.1	0.00325172	0.01608894	0.05212779	0.56366758	A_07_P041749	c_3832	cmr|Ebg operon repressor [c_3832]
eco	+2.19	9.04	+1.13	8.48	0.2	9.61	0.0	0.00144861	0.00853689	0.05153519	0.55995169	A_07_P000831	b2209	cmr|ecotin, a serine protease inhibitor [b2209]
eco	+2.30	12.92	+1.20	12.32	0.4	13.53	0.2	0.00755325	0.03148488	0.05135290	0.55990305	A_07_P045511	Z3467	cmr|ecotin, a serine protease inhibitor [Z3467]
eco	+2.61	13.08	+1.39	12.39	0.1	13.78	0.2	0.00044385	0.00358698	0.03541095	0.47732021	A_07_P032326	c_2750	cmr|Ecotin precursor [c_2750]
ECs0016	-3.69	12.49	-1.88	13.44	0.0	11.55	0.1	0.00004978	0.00073942	0.00929942	0.30823158	A_07_P058872	ECs0016	cmr|Gef protein [ECs0016]
ECs0031	+2.51	10.03	+1.33	9.37	0.2	10.70	0.0	0.00061364	0.00449774	0.03887894	0.49284219	A_07_P062890	ECs0031	cmr|putative FKBX-type 16KD peptidyl-prolyl cis-trans isomerase [ECs0031]
ECs0052	-2.25	11.92	-1.17	12.50	0.0	11.33	0.0	0.00053382	0.00406992	0.05165396	0.59230881	A_07_P059413	ECs0052	cmr|CcdA-like protein [ECs0052]
ECs0127	-2.40	9.81	-1.26	10.44	0.2	9.18	0.2	0.00126149	0.00772497	0.04097434	0.54530854	A_07_P062900	ECs0127	cmr|hypothetical protein [ECs0127]
ECs0132	-2.64	8.51	-1.40	9.21	0.1	7.82	0.0	0.00026112	0.00242251	0.03043763	0.49049130	A_07_P059452	ECs0132	cmr|hypothetical protein [ECs0132]
ECs0192	-2.06	11.00	-1.04	11.52	0.1	10.48	0.1	0.00183856	0.01034164	0.06703951	0.65152469	A_07_P059513	ECs0192	cmr|hypothetical protein [ECs0192]
ECs0211	+2.59	9.24	+1.37	8.56	0.0	9.93	0.1	0.00033756	0.00291050	0.03600356	0.47996613	A_07_P062916	ECs0211	cmr|DNA polymerase III epsilon subunit [ECs0211]
ECs0218	-2.23	8.39	-1.16	8.97	0.4	7.81	0.2	0.00669527	0.02867161	0.05290587	0.59838362	A_07_P059547	ECs0218	cmr|IcmF-like protein [ECs0218]
ECs0228	-2.37	10.15	-1.24	10.77	0.3	9.52	0.2	0.00246307	0.01297376	0.04328633	0.55860656	A_07_P059596	ECs0228	cmr|hypothetical protein [ECs0228]
ECs0290	-2.14	11.38	-1.10	11.93	0.1	10.83	0.1	0.00120662	0.00747030	0.05995935	0.62849534	A_07_P053647	ECs0290	cmr|hypothetical protein [ECs0290]
ECs0296	-3.35	9.76	-1.75	10.63	0.2	8.89	0.1	0.00016130	0.00171124	0.01323044	0.34543947	A_07_P053677	ECs0296	cmr|putative Ogr family transcription activator [ECs0296]
ECs0325	-2.75	8.20	-1.46	8.93	0.2	7.47	0.0	0.00028487	0.00259096	0.02652439	0.46921348	A_07_P053765	ECs0325	cmr|hypothetical protein [ECs0325]
ECs0326	-2.27	8.12	-1.18	8.72	0.0	7.53	0.1	0.00063177	0.00459849	0.04986598	0.58588806	A_07_P053768	ECs0326	cmr|hypothetical protein [ECs0326]
ECs0330	-3.24	8.73	-1.70	9.58	0.1	7.88	0.0	0.00007365	0.00096468	0.01512195	0.36911628	A_07_P053789	ECs0330	cmr|hypothetical protein [ECs0330]
ECs0332	+4.88	8.71	+2.29	7.57	0.3	9.86	0.2	0.00010994	0.00128313	0.01074695	0.28484848	A_07_P053796	ECs0332	cmr|hypothetical protein [ECs0332]
ECs0335	+2.16	7.48	+1.11	6.92	0.1	8.03	0.1	0.00101159	0.00657578	0.05330348	0.56934193	A_07_P053815	ECs0335	cmr|putative oxidoreductase [ECs0335]
ECs0357	+3.07	9.24	+1.62	8.43	0.0	10.05	0.2	0.00030398	0.00270694	0.02650407	0.42710338	A_07_P053871	ECs0357	cmr|choline dehydrogenase [ECs0357]
ECs0423	-2.04	10.39	-1.03	10.90	0.1	9.87	0.1	0.00170368	0.00973955	0.06914761	0.65740338	A_07_P053991	ECs0423	cmr|5-aminolevulinate dehydratase [ECs0423]
ECs0449	-7.63	11.38	-2.93	12.85	0.0	9.92	0.0	0.00000215	0.00010587	0.00069106	0.08119403	A_07_P054018	ECs0449	cmr|positive response regulator for pho regulon PhoB [ECs0449]
ECs0483	+3.49	8.73	+1.80	7.83	0.2	9.64	0.7	0.00658270	0.02827779	0.02118077	0.38462514	A_07_P062971	ECs0483	cmr|cytochrome o ubiquinol oxidase subunit IV [ECs0483]
ECs0547	-2.18	10.24	-1.13	10.80	0.1	9.68	0.3	0.00327481	0.01619812	0.05732978	0.61568895	A_07_P054119	ECs0547	cmr|hypothetical protein [ECs0547]
ECs0551	-2.55	10.80	-1.35	11.48	0.1	10.13	0.0	0.00028746	0.00260332	0.03420033	0.51086338	A_07_P054138	ECs0551	cmr|hypothetical protein [ECs0551]
ECs0573	+2.60	7.60	+1.38	6.92	0.0	8.29	0.1	0.00031892	0.00279411	0.03590066	0.47940628	A_07_P054146	ECs0573	cmr|allantoinase [ECs0573]
ECs0679	-2.20	10.45	-1.13	11.01	0.0	9.88	0.0	0.00063622	0.00461448	0.05629700	0.61295989	A_07_P054223	ECs0679	cmr|minor lipoprotein [ECs0679]
ECs0694	+2.83	11.27	+1.50	10.52	0.2	12.02	0.1	0.00030216	0.00269224	0.03031885	0.44652947	A_07_P056560	ECs0694	cmr|putative periplasmic binding transport protein [ECs0694]
ECs0711	-3.61	9.89	-1.85	10.81	0.1	8.96	0.2	0.00016643	0.00175383	0.00991870	0.32065708	A_07_P056574	ECs0711	cmr|hypothetical protein [ECs0711]
ECs0742	+2.13	7.17	+1.09	6.63	0.1	7.72	0.1	0.00109917	0.00698512	0.05462779	0.57184840	A_07_P056583	ECs0742	cmr|putative chaperone [ECs0742]
ECs0778	-3.20	8.66	-1.68	9.50	0.1	7.82	0.2	0.00014446	0.00156823	0.01571202	0.37423601	A_07_P056671	ECs0778	cmr|quinolinate synthetase A protein [ECs0778]
ECs0794	+2.04	9.94	+1.03	9.42	0.1	10.45	0.1	0.00202955	0.01117636	0.06136751	0.60347908	A_07_P056677	ECs0794	cmr|putative phosphatase [ECs0794]
ECs0805	+107.44	11.01	+6.75	7.63	0.0	14.38	0.2	0.00000004	0.00003344	0.00000064	0.00166667	A_07_P056700	ECs0805	cmr|hypothetical C4-type zinc finger protein TraR-family [ECs0805]
ECs0806	+5.73	11.19	+2.52	9.93	0.3	12.45	0.2	0.00005770	0.00081407	0.00775915	0.24629032	A_07_P056701	ECs0806	cmr|hypothetical protein [ECs0806]
ECs0807	+17.00	9.90	+4.09	7.85	0.0	11.94	0.2	0.00000108	0.00007677	0.00081237	0.08470199	A_07_P056708	ECs0807	cmr|hypothetical protein [ECs0807]
ECs0808	+4.67	11.63	+2.22	10.52	0.2	12.74	0.2	0.00010542	0.00124701	0.01176956	0.29459459	A_07_P056712	ECs0808	cmr|hypothetical protein [ECs0808]
ECs0809	+3.57	12.72	+1.84	11.80	0.1	13.63	0.0	0.00004771	0.00071332	0.01937754	0.36624250	A_07_P056720	ECs0809	cmr|exonuclease [ECs0809]
ECs0810	+16.70	10.18	+4.06	8.15	0.1	12.21	0.1	0.00000046	0.00005132	0.00080285	0.08426667	A_07_P056725	ECs0810	cmr|NinE protein [ECs0810]
ECs0819	+22.14	10.22	+4.47	7.99	0.1	12.45	0.2	0.00000052	0.00005341	0.00035696	0.05509804	A_07_P056765	ECs0819	cmr|endolysin [ECs0819]
ECs0820	+12.89	8.70	+3.69	6.85	0.1	10.54	0.1	0.00000077	0.00006301	0.00142721	0.11582474	A_07_P056766	ECs0820	cmr|putative endopeptidase [ECs0820]
ECs0821	-2.54	10.13	-1.35	10.80	0.2	9.45	0.0	0.00058367	0.00434242	0.03430767	0.51149621	A_07_P056771	ECs0821	cmr|putative lipoprotein Rz1 precursor [ECs0821]
ECs0825	-2.52	8.74	-1.34	9.41	0.2	8.07	0.1	0.00048784	0.00383643	0.03553798	0.51520258	A_07_P056791	ECs0825	cmr|putative terminase large subunit [ECs0825]
ECs0827	-2.26	9.83	-1.18	10.42	0.1	9.24	0.3	0.00286751	0.01463408	0.04838923	0.58155725	A_07_P056801	ECs0827	cmr|putative portal protein [ECs0827]
ECs0828	-2.64	8.20	-1.40	8.90	0.1	7.49	0.1	0.00027739	0.00254324	0.03009146	0.48740741	A_07_P056809	ECs0828	cmr|hypothetical protein [ECs0828]
ECs0836	-3.53	9.75	-1.82	10.66	0.6	8.84	0.0	0.00324174	0.01606236	0.01106009	0.32792844	A_07_P056847	ECs0836	cmr|putative minor tail protein [ECs0836]
ECs0840	+5.47	9.34	+2.45	8.11	0.2	10.57	0.1	0.00002264	0.00042679	0.00845211	0.25492337	A_07_P056866	ECs0840	cmr|putative tail assembly protein [ECs0840]
ECs0841	+2.61	8.57	+1.38	7.88	0.2	9.26	0.2	0.00071962	0.00503543	0.03611535	0.48064243	A_07_P056872	ECs0841	cmr|putative tail assembly protein [ECs0841]
ECs0878	+4.28	10.42	+2.10	9.38	0.0	11.47	0.1	0.00001939	0.00038447	0.01396278	0.31952035	A_07_P056933	ECs0878	cmr|putative enzyme [ECs0878]
ECs0901	+2.36	7.24	+1.24	6.62	0.1	7.87	0.2	0.00153812	0.00896468	0.04455094	0.52266021	A_07_P056937	ECs0901	cmr|hypothetical protein [ECs0901]
ECs0906	+2.17	10.55	+1.12	9.99	0.2	11.10	0.1	0.00188334	0.01051561	0.05394754	0.56850736	A_07_P056943	ECs0906	cmr|hypothetical protein [ECs0906]
ECs0950	-2.69	11.30	-1.43	12.02	0.1	10.59	0.0	0.00018626	0.00190047	0.02909489	0.48015723	A_07_P056991	ECs0950	cmr|hypothetical protein [ECs0950]
ECs0952	+2.13	8.69	+1.09	8.15	0.1	9.24	0.1	0.00164716	0.00948535	0.05539888	0.57647059	A_07_P057004	ECs0952	cmr|hypothetical protein [ECs0952]
ECs0981	+5.37	8.44	+2.43	7.23	0.2	9.66	0.1	0.00002006	0.00039347	0.00875826	0.25967985	A_07_P063053	ECs0981	cmr|anaerobic dimethyl sulfoxide reductase subunit C [ECs0981]
ECs1011	+6.41	9.93	+2.68	8.59	0.1	11.27	0.1	0.00000701	0.00020362	0.00610455	0.22561033	A_07_P063067	ECs1011	cmr|aspartate aminotransferase [ECs1011]
ECs1015	+7.70	9.51	+2.94	8.04	0.0	10.98	0.3	0.00001331	0.00030070	0.00433181	0.18891967	A_07_P063074	ECs1015	cmr|aminopeptidase N [ECs1015]
ECs1061	+2.11	7.41	+1.07	6.87	0.1	7.95	0.1	0.00111280	0.00704458	0.05656885	0.58324820	A_07_P057089	ECs1061	cmr|hypothetical protein [ECs1061]
ECs1071	-2.88	10.40	-1.52	11.16	0.4	9.64	0.0	0.00200255	0.01105861	0.02243712	0.43343558	A_07_P057126	ECs1071	cmr|hypothetical protein [ECs1071]
ECs1081	-4.64	9.01	-2.21	10.11	0.1	7.90	0.1	0.00001605	0.00033988	0.00396786	0.20754153	A_07_P057173	ECs1081	cmr|hypothetical protein [ECs1081]
ECs1092	+5.26	11.25	+2.40	10.05	0.3	12.44	0.2	0.00009217	0.00113011	0.00920541	0.26789279	A_07_P057221	ECs1092	cmr|putative lipoprotein Rz1 protein precursor [ECs1092]
ECs1093	+2.05	7.13	+1.04	6.61	0.0	7.65	0.0	0.00108047	0.00690376	0.05904662	0.59401278	A_07_P057227	ECs1093	cmr|putative endopeptidase [ECs1093]
ECs1098	-2.10	7.51	-1.07	8.05	0.4	6.98	0.1	0.00861171	0.03483629	0.06130589	0.63667546	A_07_P057241	ECs1098	cmr|hypothetical protein [ECs1098]
ECs1105	-4.49	11.90	-2.17	12.98	0.1	10.82	0.2	0.00004518	0.00068369	0.00475483	0.22548193	A_07_P057269	ECs1105	cmr|putative terminase small subunit [ECs1105]
ECs1106	+3.07	8.96	+1.62	8.15	0.0	9.76	0.0	0.00008139	0.00103335	0.02553163	0.42491543	A_07_P057274	ECs1106	cmr|putative terminase large subunit [ECs1106]
ECs1107	+5.81	8.51	+2.54	7.24	0.0	9.78	0.2	0.00000996	0.00025049	0.00742315	0.24501048	A_07_P057276	ECs1107	cmr|putative portal protein [ECs1107]
ECs1108	+27.96	10.47	+4.81	8.06	0.2	12.87	0.0	0.00000023	0.00004002	0.00017276	0.03726027	A_07_P057281	ECs1108	cmr|putative head-tail preconnector protein [ECs1108]
ECs1109	+29.97	10.12	+4.91	7.67	0.3	12.57	0.1	0.00000040	0.00004930	0.00013656	0.03257576	A_07_P057290	ECs1109	cmr|putative head decoration protein [ECs1109]
ECs1110	+18.81	8.96	+4.23	6.84	0.0	11.07	0.1	0.00000022	0.00004002	0.00060722	0.07242424	A_07_P057293	ECs1110	cmr|putative major head protein [ECs1110]
ECs1112	+2.22	8.28	+1.15	7.71	0.1	8.86	0.0	0.00071315	0.00499460	0.05001651	0.55769122	A_07_P057302	ECs1112	cmr|putative minor tail protein [ECs1112]
ECs1119	-2.65	10.61	-1.41	11.31	0.2	9.90	0.4	0.00303173	0.01527901	0.02868648	0.47893955	A_07_P057336	ECs1119	cmr|hypothetical protein [ECs1119]
ECs1125	-2.09	7.37	-1.06	7.90	0.1	6.84	0.1	0.00153063	0.00893653	0.06561611	0.65136192	A_07_P061904	ECs1125	cmr|hypothetical protein [ECs1125]
ECs1156	-6.16	9.66	-2.62	10.97	0.0	8.35	0.8	0.00121979	0.00753702	0.00145516	0.12451087	A_07_P061921	ECs1156	cmr|hypothetical protein [ECs1156]
ECs1160	-2.74	10.26	-1.45	10.99	0.0	9.53	0.1	0.00017415	0.00180973	0.02679942	0.47143017	A_07_P061927	ECs1160	cmr|putative integrase [ECs1160]
ECs1160	-2.28	10.00	-1.19	10.60	0.1	9.41	0.1	0.00068353	0.00484533	0.04971100	0.58581587	A_07_P061927	ECs1160	cmr|putative integrase [ECs1160]
ECs1163	+22.95	9.39	+4.52	7.13	0.0	11.65	0.0	0.00000014	0.00003548	0.00032393	0.05204082	A_07_P061944	ECs1163	cmr|hypothetical protein [ECs1163]
ECs1170	+29.78	10.11	+4.90	7.66	0.2	12.56	0.2	0.00000073	0.00006202	0.00013656	0.03208955	A_07_P061980	ECs1170	cmr|C4-type zinc finger protein [ECs1170]
ECs1173	+3.12	9.73	+1.64	8.91	0.6	10.55	0.3	0.00512656	0.02323331	0.02682546	0.42746964	A_07_P061991	ECs1173	cmr|hypothetical protein [ECs1173]
ECs1174	+10.11	12.17	+3.34	10.50	0.3	13.84	0.1	0.00000674	0.00019841	0.00252795	0.14686347	A_07_P061996	ECs1174	cmr|exonuclease [ECs1174]
ECs1175	+4.86	14.83	+2.28	13.69	0.0	15.97	0.1	0.00001725	0.00035688	0.01077617	0.28466443	A_07_P062005	ECs1175	cmr|recombination protein Bet [ECs1175]
ECs1176	+18.15	12.56	+4.18	10.47	0.1	14.65	0.0	0.00000030	0.00004295	0.00064850	0.07562963	A_07_P062008	ECs1176	cmr|host-nuclease inhibitor protein Gam [ECs1176]
ECs1179	+11.93	13.56	+3.58	11.77	0.2	15.34	0.0	0.00000123	0.00008178	0.00171621	0.12281818	A_07_P062021	ECs1179	cmr|single strand binding protein Ea10 [ECs1179]
ECs1181	-3.13	14.53	-1.65	15.36	0.1	13.71	0.2	0.00016215	0.00171792	0.01739329	0.38192469	A_07_P062031	ECs1181	cmr|putative anti-termination protein N [ECs1181]
ECs1185	-2.49	13.58	-1.31	14.24	0.0	12.93	0.1	0.00032848	0.00285409	0.03741552	0.52595536	A_07_P062053	ECs1185	cmr|putative cI repressor protein [ECs1185]
ECs1186	+7.08	11.18	+2.82	9.77	0.2	12.59	0.2	0.00001498	0.00032347	0.00517467	0.20783163	A_07_P062059	ECs1186	cmr|putative regulatory protein [ECs1186]
ECs1187	+4.82	11.76	+2.27	10.63	0.1	12.90	0.2	0.00002881	0.00050012	0.01078951	0.28453936	A_07_P062064	ECs1187	cmr|regulatory protein CII [ECs1187]
ECs1197	+30.02	9.48	+4.91	7.02	0.0	11.93	0.2	0.00000029	0.00004218	0.00013021	0.03253968	A_07_P062106	ECs1197	cmr|NinE protein [ECs1197]
ECs1202	-2.04	10.79	-1.03	11.30	0.3	10.28	0.2	0.01078145	0.04174696	0.07169525	0.66791124	A_07_P062135	ECs1202	cmr|hypothetical protein [ECs1202]
ECs1204	-2.77	10.84	-1.47	11.58	0.0	10.11	0.6	0.00746154	0.03119343	0.02635544	0.46885876	A_07_P062145	ECs1204	cmr|hypothetical protein [ECs1204]
ECs1205	-2.21	9.13	-1.14	9.70	0.1	8.56	0.1	0.00106564	0.00683382	0.05501524	0.60868587	A_07_P062148	ECs1205	cmr|Shiga toxin 2 subunit A [ECs1205]
ECs1219	-2.80	8.82	-1.48	9.56	0.0	8.07	0.5	0.00293612	0.01492133	0.02531695	0.45920507	A_07_P062208	ECs1219	cmr|putative small subunit terminase [ECs1219]
ECs1240	-3.11	9.23	-1.64	10.05	0.1	8.41	0.1	0.00014523	0.00157469	0.01768039	0.38661111	A_07_P062314	ECs1240	cmr|hypothetical protein [ECs1240]
ECs1249	-3.04	11.86	-1.60	12.66	0.2	11.05	0.5	0.00254987	0.01335793	0.01779535	0.38644138	A_07_P062357	ECs1249	cmr|hypothetical protein [ECs1249]
ECs1276	-3.14	8.81	-1.65	9.64	0.4	7.99	0.1	0.00136401	0.00817160	0.01648247	0.37772926	A_07_P062400	ECs1276	cmr|putative chaperone protein [ECs1276]
ECs1283	-2.86	8.16	-1.52	8.92	0.2	7.40	0.1	0.00043425	0.00353321	0.02316565	0.44101572	A_07_P062434	ECs1283	cmr|hemolysin activator-related protein [ECs1283]
ECs1299	-3.16	10.87	-1.66	11.69	0.4	10.04	0.0	0.00102899	0.00666690	0.01643102	0.37709913	A_07_P062501	ECs1299	cmr|putative integrase [ECs1299]
ECs1300	+7.34	9.18	+2.88	7.75	0.2	10.62	0.1	0.00000919	0.00023947	0.00480246	0.20216578	A_07_P062509	ECs1300	cmr|putative membrane protein [ECs1300]
ECs1305	-3.26	12.89	-1.70	13.75	0.6	12.04	0.6	0.01120798	0.04302815	0.01574632	0.37279699	A_07_P062533	ECs1305	cmr|hypothetical protein [ECs1305]
ECs1338	-3.67	9.65	-1.88	10.59	0.1	8.71	0.1	0.00005985	0.00083757	0.00952744	0.31250000	A_07_P062685	ECs1338	cmr|hypothetical protein [ECs1338]
ECs1339	-3.04	8.77	-1.60	9.57	0.3	7.96	0.2	0.00053725	0.00409412	0.01858295	0.39218499	A_07_P062686	ECs1339	cmr|hypothetical protein [ECs1339]
ECs1359	-3.21	8.05	-1.68	8.90	0.1	7.21	0.0	0.00007381	0.00096602	0.01565993	0.37356061	A_07_P060342	ECs1359	cmr|hypothetical protein [ECs1359]
ECs1363	-10.95	10.02	-3.45	11.75	0.1	8.30	0.0	0.00000098	0.00007230	0.00011115	0.03571429	A_07_P060356	ECs1363	cmr|hypothetical protein [ECs1363]
ECs1369	-2.67	7.66	-1.41	8.37	0.1	6.95	0.2	0.00048496	0.00382331	0.03038364	0.49012295	A_07_P060383	ECs1369	cmr|hypothetical protein [ECs1369]
ECs1375	-2.08	8.99	-1.06	9.52	0.0	8.47	0.2	0.00163553	0.00943339	0.06601499	0.65162382	A_07_P060402	ECs1375	cmr|hypothetical protein [ECs1375]
ECs1380	-9.21	9.90	-3.20	11.50	0.1	8.30	0.2	0.00000405	0.00015126	0.00032647	0.05586957	A_07_P060427	ECs1380	cmr|putative transposase OrfB protein of insertion sequence IS629 [ECs1380]
ECs1381	-3.03	9.85	-1.60	10.65	0.1	9.05	0.2	0.00025971	0.00241229	0.01890943	0.39589096	A_07_P060432	ECs1381	cmr|putative transposase OrfA protein of insertion sequence IS629 [ECs1381]
ECs1392	-4.83	8.91	-2.27	10.05	0.0	7.78	0.1	0.00001356	0.00030459	0.00356771	0.19639860	A_07_P060482	ECs1392	cmr|hypothetical protein [ECs1392]
ECs1395	-2.67	7.61	-1.42	8.32	0.1	6.90	0.1	0.00041509	0.00341797	0.02885099	0.47965153	A_07_P060497	ECs1395	cmr|hypothetical protein [ECs1395]
ECs1397	-2.98	11.11	-1.58	11.90	0.1	10.32	0.0	0.00013777	0.00151791	0.02030678	0.40883632	A_07_P060501	ECs1397	cmr|hypothetical protein [ECs1397]
ECs1400	+2.24	8.03	+1.16	7.45	0.4	8.61	0.1	0.00701024	0.02972510	0.05239393	0.56422025	A_07_P060520	ECs1400	cmr|hypothetical protein [ECs1400]
ECs1445	+2.48	7.97	+1.31	7.31	0.0	8.63	0.1	0.00029795	0.00267133	0.03919080	0.49361600	A_07_P063092	ECs1445	cmr|hypothetical protein [ECs1445]
ECs1511	-4.22	11.94	-2.08	12.98	0.1	10.90	0.2	0.00005390	0.00077860	0.00598704	0.25338710	A_07_P060633	ECs1511	cmr|hypothetical protein [ECs1511]
ECs1513	-2.25	7.94	-1.17	8.53	0.5	7.36	0.1	0.01059942	0.04116362	0.05019881	0.58629822	A_07_P060641	ECs1513	cmr|hypothetical protein [ECs1513]
ECs1514	-2.69	11.52	-1.43	12.24	0.0	10.81	0.0	0.00017073	0.00178845	0.02905361	0.48048319	A_07_P060649	ECs1514	cmr|hypothetical protein [ECs1514]
ECs1518	+8.77	9.68	+3.13	8.12	0.3	11.25	0.2	0.00001374	0.00030776	0.00325965	0.16608414	A_07_P060667	ECs1518	cmr|hypothetical protein [ECs1518]
ECs1526	-8.85	8.38	-3.15	9.95	0.0	6.81	0.0	0.00000140	0.00008756	0.00035950	0.05717172	A_07_P060699	ECs1526	cmr|hypothetical protein [ECs1526]
ECs1527	-2.27	8.51	-1.18	9.10	0.1	7.92	0.3	0.00267195	0.01381689	0.05020135	0.58589325	A_07_P060707	ECs1527	cmr|hypothetical protein [ECs1527]
ECs1537	-3.27	11.78	-1.71	12.64	0.1	10.93	0.4	0.00099495	0.00650516	0.01491679	0.36695312	A_07_P060748	ECs1537	cmr|hypothetical protein [ECs1537]
ECs1539	-2.64	7.81	-1.40	8.51	0.4	7.11	0.1	0.00304604	0.01532658	0.02955475	0.48268672	A_07_P060754	ECs1539	cmr|hypothetical protein [ECs1539]
ECs1544	-2.34	8.97	-1.23	9.58	0.3	8.35	0.1	0.00334441	0.01643911	0.04505526	0.56838942	A_07_P060776	ECs1544	cmr|putative portal protein [ECs1544]
ECs1558	+3.01	8.76	+1.59	7.97	0.3	9.55	0.3	0.00137981	0.00824114	0.02771024	0.43280754	A_07_P060837	ECs1558	cmr|putative tail assembly protein [ECs1558]
ECs1567	-2.14	9.00	-1.10	9.54	0.2	8.45	0.2	0.00224639	0.01208718	0.05950902	0.62669565	A_07_P060877	ECs1567	cmr|hypothetical protein [ECs1567]
ECs1595	-6.06	10.95	-2.60	12.25	0.3	9.65	0.0	0.00002513	0.00045661	0.00157076	0.12947644	A_07_P061000	ECs1595	cmr|hypothetical protein [ECs1595]
ECs1607	-5.03	13.54	-2.33	14.71	0.0	12.38	0.1	0.00001321	0.00029969	0.00297637	0.18233463	A_07_P061030	ECs1607	cmr|hypothetical protein [ECs1607]
ECs1608	+6.11	8.96	+2.61	7.65	0.1	10.26	0.1	0.00000812	0.00022454	0.00680259	0.23590308	A_07_P061034	ECs1608	cmr|isocitrate dehydrogenase [ECs1608]
ECs1609	+12.58	8.64	+3.65	6.81	0.1	10.46	0.2	0.00000237	0.00011227	0.00159299	0.11886256	A_07_P061036	ECs1609	cmr|intergrase [ECs1609]
ECs1610	+55.51	10.26	+5.79	7.37	0.3	13.16	0.1	0.00000021	0.00003870	0.00001651	0.01040000	A_07_P061042	ECs1610	cmr|excisionase [ECs1610]
ECs1612	+2.72	12.13	+1.44	11.41	0.0	12.85	0.1	0.00020451	0.00202631	0.03255716	0.46178378	A_07_P061055	ECs1612	cmr|replication protein P [ECs1612]
ECs1615	-2.02	8.24	-1.02	8.74	0.1	7.73	0.0	0.00128982	0.00785283	0.07162919	0.66769094	A_07_P061064	ECs1615	cmr|hypothetical protein [ECs1615]
ECs1623	+19.28	9.59	+4.27	7.45	0.4	11.72	0.2	0.00000610	0.00018856	0.00048526	0.06211382	A_07_P061089	ECs1623	cmr|putative endopeptidase [ECs1623]
ECs1625	+2.25	7.52	+1.17	6.94	0.0	8.11	0.1	0.00077706	0.00537131	0.04899835	0.55141530	A_07_P061095	ECs1625	cmr|Bor protein precursor [ECs1625]
ECs1630	-2.56	8.77	-1.36	9.45	0.5	8.09	0.1	0.00627202	0.02726303	0.03217416	0.50203171	A_07_P061109	ECs1630	cmr|terminase large subunit [ECs1630]
ECs1638	+20.51	10.22	+4.36	8.04	0.1	12.40	0.1	0.00000027	0.00004211	0.00045668	0.06198276	A_07_P061143	ECs1638	cmr|minor tail protein [ECs1638]
ECs1639	+3.91	10.92	+1.97	9.93	0.0	11.90	0.1	0.00003366	0.00055720	0.01636814	0.34314248	A_07_P061148	ECs1639	cmr|minor tail protein [ECs1639]
ECs1640	+16.14	9.25	+4.01	7.24	0.1	11.26	0.1	0.00000042	0.00005012	0.00089876	0.09070513	A_07_P061154	ECs1640	cmr|major tail protein [ECs1640]
ECs1641	+16.88	9.21	+4.08	7.17	0.1	11.25	0.0	0.00000032	0.00004517	0.00076918	0.08351724	A_07_P061159	ECs1641	cmr|minor tail protein [ECs1641]
ECs1642	+4.21	7.89	+2.07	6.85	0.0	8.93	0.2	0.00006767	0.00091533	0.01419779	0.32255411	A_07_P061162	ECs1642	cmr|minor tail protein [ECs1642]
ECs1643	+4.86	10.00	+2.28	8.86	0.0	11.14	0.2	0.00002127	0.00040998	0.01077109	0.28500840	A_07_P061170	ECs1643	cmr|tail length tape measure protein precursor [ECs1643]
ECs1644	+4.62	9.81	+2.21	8.71	0.2	10.92	0.2	0.00007617	0.00098704	0.01195185	0.29540031	A_07_P061173	ECs1644	cmr|minor tail protein [ECs1644]
ECs1645	+3.22	9.54	+1.69	8.70	0.2	10.38	0.2	0.00044776	0.00360591	0.02405615	0.41078091	A_07_P061180	ECs1645	cmr|minor tail protein [ECs1645]
ECs1653	-2.26	8.44	-1.18	9.03	0.4	7.85	0.1	0.00696371	0.02957556	0.04850800	0.58165270	A_07_P061214	ECs1653	cmr|hypothetical protein [ECs1653]
ECs1666	-6.64	9.45	-2.73	10.82	0.2	8.09	0.2	0.00001926	0.00038387	0.00117315	0.11059880	A_07_P058060	ECs1666	cmr|putative transposase OrfB protein of insertion sequence IS629 [ECs1666]
ECs1702	-2.16	8.13	-1.11	8.69	0.1	7.58	0.1	0.00143774	0.00849372	0.05877795	0.62358491	A_07_P058124	ECs1702	cmr|trehalase [ECs1702]
ECs1724	+3.91	9.45	+1.97	8.47	0.0	10.43	0.1	0.00002992	0.00051429	0.01668382	0.34516426	A_07_P058147	ECs1724	cmr|hypothetical protein [ECs1724]
ECs1743	+60.82	11.77	+5.93	8.81	0.3	14.73	0.2	0.00000020	0.00003706	0.00001143	0.00947368	A_07_P063132	ECs1743	cmr|oligopeptide transport periplasmic binding protein [ECs1743]
ECs1751	+11.37	10.31	+3.51	8.56	0.0	12.07	0.1	0.00000080	0.00006443	0.00196329	0.13153191	A_07_P058156	ECs1751	cmr|hypothetical protein [ECs1751]
ECs1769	+2.05	8.00	+1.04	7.48	0.1	8.52	0.1	0.00160588	0.00930203	0.06083905	0.60166457	A_07_P058224	ECs1769	cmr|putative phage replication protein [ECs1769]
ECs1773	-2.15	15.65	-1.10	16.21	0.0	15.10	0.0	0.00076827	0.00532382	0.05969576	0.62824198	A_07_P058245	ECs1773	cmr|putative prophage maintenance protein [ECs1773]
ECs1780	-5.47	10.82	-2.45	12.04	0.0	9.59	0.8	0.00148203	0.00869341	0.00246888	0.16682403	A_07_P058276	ECs1780	cmr|putative DNA methylase [ECs1780]
ECs1798	-4.49	9.44	-2.17	10.52	0.2	8.35	0.4	0.00051238	0.00396018	0.00487424	0.22907463	A_07_P058341	ECs1798	cmr|hypothetical protein [ECs1798]
ECs1827	-5.21	9.13	-2.38	10.32	0.0	7.94	0.1	0.00000901	0.00023748	0.00266578	0.17060976	A_07_P058453	ECs1827	cmr|IS630 hypothetical protein [ECs1827]
ECs1833	+29.96	10.12	+4.90	7.67	0.1	12.57	0.0	0.00000009	0.00003427	0.00014799	0.03426471	A_07_P058463	ECs1833	cmr|tryptophan synthase beta protein [ECs1833]
ECs1839	-2.24	8.98	-1.17	9.57	0.1	8.40	0.1	0.00093070	0.00618686	0.05177591	0.59241279	A_07_P058472	ECs1839	cmr|hypothetical protein [ECs1839]
ECs1839	-2.25	8.79	-1.17	9.38	0.0	8.20	0.1	0.00057475	0.00430081	0.05146913	0.59277981	A_07_P058472	ECs1839	cmr|hypothetical protein [ECs1839]
ECs1839	-2.55	9.01	-1.35	9.69	0.0	8.34	0.0	0.00023745	0.00226157	0.03463859	0.51351224	A_07_P058472	ECs1839	cmr|hypothetical protein [ECs1839]
ECs1839	-2.39	8.95	-1.26	9.57	0.0	8.32	0.1	0.00047473	0.00377104	0.04232279	0.55435108	A_07_P058472	ECs1839	cmr|hypothetical protein [ECs1839]
ECs1839	-2.25	9.11	-1.17	9.70	0.0	8.52	0.1	0.00068008	0.00483817	0.05077617	0.58867452	A_07_P058472	ECs1839	cmr|hypothetical protein [ECs1839]
ECs1900	-2.69	12.12	-1.43	12.83	0.0	11.41	0.2	0.00031679	0.00278319	0.02937246	0.48170833	A_07_P058536	ECs1900	cmr|putative enzyme [ECs1900]
ECs1906	+2.97	8.68	+1.57	7.90	0.2	9.47	0.0	0.00020083	0.00200374	0.02756606	0.43227092	A_07_P058547	ECs1906	cmr|hypothetical protein [ECs1906]
ECs1930	-2.80	9.99	-1.49	10.73	0.0	9.25	0.3	0.00064142	0.00463874	0.02542048	0.45949483	A_07_P058575	ECs1930	cmr|hypothetical protein [ECs1930]
ECs1930	-4.12	10.12	-2.04	11.14	0.4	9.10	0.3	0.00072726	0.00508433	0.00673399	0.26373134	A_07_P058575	ECs1930	cmr|hypothetical protein [ECs1930]
ECs1930	-3.99	10.01	-2.00	11.01	0.8	9.01	0.1	0.00665322	0.02853397	0.00756542	0.27829439	A_07_P058575	ECs1930	cmr|hypothetical protein [ECs1930]
ECs1951	+20.47	9.36	+4.36	7.18	0.2	11.53	0.5	0.00000917	0.00023946	0.00044080	0.06087719	A_07_P058631	ECs1951	cmr|hypothetical protein [ECs1951]
ECs1974	-2.05	9.28	-1.04	9.80	0.1	8.77	0.1	0.00169054	0.00967148	0.06859947	0.65775274	A_07_P058697	ECs1974	cmr|putative portal protein [ECs1974]
ECs1981	-3.07	10.52	-1.62	11.33	0.3	9.72	0.0	0.00046153	0.00368850	0.01794271	0.38644323	A_07_P058732	ECs1981	cmr|putative tail assembly chaperon [ECs1981]
ECs1985	-3.64	11.56	-1.86	12.49	0.1	10.63	0.1	0.00005639	0.00080052	0.01015561	0.32564155	A_07_P058751	ECs1985	cmr|putative minor tail protein [ECs1985]
ECs2032	+2.08	8.50	+1.06	7.97	0.0	9.03	0.2	0.00203995	0.01122576	0.05966400	0.59641270	A_07_P054237	ECs2032	cmr|ribosomal-protein-serine N-acetyltransferase [ECs2032]
ECs2049	-2.66	7.56	-1.41	8.27	0.1	6.86	0.3	0.00174777	0.00994469	0.03007940	0.48771370	A_07_P058915	ECs2049	cmr|hypothetical protein [ECs2049]
ECs2112	-2.27	7.96	-1.18	8.55	0.1	7.37	0.3	0.00256069	0.01339832	0.04952934	0.58498875	A_07_P054315	ECs2112	cmr|putative fimbrial chaperone protein precursor [ECs2112]
ECs2128	+3.10	9.84	+1.63	9.03	0.0	10.66	0.3	0.00031444	0.00277030	0.02557228	0.42514256	A_07_P063195	ECs2128	cmr|altronate oxidoreductase [ECs2128]
ECs2139	+6.79	8.62	+2.76	7.24	0.3	10.01	0.0	0.00001645	0.00034504	0.00544461	0.21217822	A_07_P054346	ECs2139	cmr|multiple antibiotic resistance protein [ECs2139]
ECs2171	+2.58	8.16	+1.37	7.48	0.2	8.85	0.1	0.00055946	0.00421847	0.03651677	0.48312605	A_07_P054460	ECs2171	cmr|putative minor tail protein [ECs2171]
ECs2177	+2.15	8.05	+1.11	7.50	0.0	8.61	0.2	0.00167238	0.00959142	0.05493204	0.57350796	A_07_P054468	ECs2177	cmr|putative portal protein [ECs2177]
ECs2183	+8.49	9.56	+3.09	8.01	0.1	11.10	0.1	0.00000308	0.00012777	0.00350864	0.17208723	A_07_P054491	ECs2183	cmr|putative lipoprotein Rz1 precursor [ECs2183]
ECs2184	+8.69	8.61	+3.12	7.05	0.0	10.17	0.2	0.00000435	0.00015783	0.00339304	0.16905063	A_07_P054498	ECs2184	cmr|putative endopeptidase [ECs2184]
ECs2234	+2.82	7.97	+1.49	7.22	0.0	8.72	0.0	0.00013667	0.00151031	0.03024327	0.44583333	A_07_P054691	ECs2234	cmr|putative host specificity protein [ECs2234]
ECs2235	+2.38	7.66	+1.25	7.04	0.2	8.29	0.0	0.00063293	0.00460268	0.04292937	0.51241850	A_07_P054700	ECs2235	cmr|putative host specificity protein [ECs2235]
ECs2241	-3.18	11.70	-1.67	12.54	0.2	10.87	0.4	0.00140496	0.00835653	0.01548145	0.37383436	A_07_P054726	ECs2241	cmr|putative tail protein [ECs2241]
ECs2297	+2.04	8.31	+1.03	7.80	0.3	8.82	0.2	0.00775549	0.03219150	0.06504383	0.62404022	A_07_P054856	ECs2297	cmr|putative oxidoreductase component [ECs2297]
ECs2319	+3.23	8.68	+1.69	7.84	0.3	9.53	0.1	0.00045141	0.00362894	0.02395643	0.41086057	A_07_P054872	ECs2319	cmr|mannose-6-phosphate isomerase [ECs2319]
ECs2320	+2.17	8.99	+1.12	8.43	0.2	9.54	0.1	0.00225046	0.01210495	0.05386052	0.56835121	A_07_P054879	ECs2320	cmr|hypothetical protein [ECs2320]
ECs2356	+2.69	9.71	+1.43	9.00	0.0	10.42	0.1	0.00023721	0.00226072	0.03329522	0.46720143	A_07_P054895	ECs2356	cmr|hypothetical protein [ECs2356]
ECs2417	+2.22	8.94	+1.15	8.37	0.0	9.52	0.1	0.00084518	0.00576041	0.05031314	0.55862482	A_07_P054897	ECs2417	cmr|vitamin B12 transport [ECs2417]
ECs2431	+9.33	9.30	+3.22	7.69	0.2	10.91	0.1	0.00000596	0.00018605	0.00295732	0.15783051	A_07_P054909	ECs2431	cmr|hypothetical protein [ECs2431]
ECs2467	+2.19	9.02	+1.13	8.45	0.3	9.58	0.1	0.00349318	0.01707971	0.05196456	0.56228866	A_07_P063226	ECs2467	cmr|NADP-specific glutamate dehydrogenase [ECs2467]
ECs2471	+10.11	11.36	+3.34	9.69	0.2	13.02	0.1	0.00000253	0.00011625	0.00245109	0.14673004	A_07_P054922	ECs2471	cmr|hypothetical protein [ECs2471]
ECs2477	-4.92	8.85	-2.30	10.00	0.1	7.71	0.3	0.00006166	0.00085460	0.00351753	0.19708185	A_07_P054931	ECs2477	cmr|putative transposase OrfA protein of insertion sequence IS629 [ECs2477]
ECs2478	-4.00	8.15	-2.00	9.15	0.1	7.15	0.0	0.00003756	0.00059916	0.00707762	0.26850602	A_07_P054936	ECs2478	cmr|putative transposase OrfB protein of insertion sequence IS629 [ECs2478]
ECs2489	+2.21	7.66	+1.14	7.09	0.0	8.24	0.2	0.00106887	0.00684636	0.05016705	0.55739591	A_07_P054948	ECs2489	cmr|hypothetical protein [ECs2489]
ECs2495	-2.28	9.45	-1.19	10.04	0.1	8.85	0.3	0.00209204	0.01144841	0.04968750	0.58597753	A_07_P054951	ECs2495	cmr|hypothetical protein [ECs2495]
ECs2517	+2.25	8.09	+1.17	7.51	0.3	8.68	0.2	0.00397244	0.01890633	0.05013402	0.55781625	A_07_P063247	ECs2517	cmr|putative enzyme [ECs2517]
ECs2526	+6.76	8.70	+2.76	7.32	0.3	10.08	0.1	0.00001721	0.00035655	0.00560023	0.21663391	A_07_P054984	ECs2526	cmr|hypothetical protein [ECs2526]
ECs2547	-2.18	9.29	-1.12	9.85	0.0	8.73	0.1	0.00097415	0.00640909	0.05692518	0.61681349	A_07_P054991	ECs2547	cmr|hypothetical protein [ECs2547]
ECs2555	-5.66	10.34	-2.50	11.59	0.2	9.09	0.4	0.00011474	0.00132092	0.00199822	0.14769953	A_07_P063252	ECs2555	cmr|protease II [ECs2555]
ECs2614	+2.16	7.95	+1.11	7.39	0.2	8.50	0.3	0.00563598	0.02505163	0.05363440	0.56939987	A_07_P063266	ECs2614	cmr|hypothetical protein [ECs2614]
ECs2619	-2.01	10.97	-1.01	11.48	0.2	10.47	0.2	0.00701490	0.02973682	0.07361979	0.67270459	A_07_P055016	ECs2619	cmr|putative membrane protein [ECs2619]
ECs2641	-2.51	8.48	-1.33	9.14	0.2	7.82	0.2	0.00123809	0.00762321	0.03600419	0.51861848	A_07_P055118	ECs2641	cmr|putative tail protein [ECs2641]
ECs2658	-5.10	9.70	-2.35	10.87	0.1	8.52	0.5	0.00025026	0.00234812	0.00323171	0.18985075	A_07_P055157	ECs2658	cmr|hypothetical protein [ECs2658]
ECs2666	-2.62	7.80	-1.39	8.49	0.1	7.10	0.0	0.00023530	0.00224686	0.03113694	0.49517172	A_07_P055169	ECs2666	cmr|cytoplasmic alpha-amylase [ECs2666]
ECs2668	+3.22	9.75	+1.69	8.90	0.0	10.59	0.4	0.00069572	0.00490746	0.02370554	0.41058306	A_07_P063276	ECs2668	cmr|putative transport system permease protein [ECs2668]
ECs2694	-2.18	7.97	-1.12	8.53	0.2	7.41	0.4	0.00721994	0.03037077	0.05784235	0.61908226	A_07_P055206	ECs2694	cmr|hypothetical protein [ECs2694]
ECs2700	-2.27	8.22	-1.18	8.81	0.0	7.63	0.0	0.00053187	0.00405706	0.04988758	0.58570470	A_07_P063287	ECs2700	cmr|hypothetical protein [ECs2700]
ECs2720	+4.17	8.88	+2.06	7.85	0.0	9.91	0.1	0.00002680	0.00047665	0.01476372	0.32691983	A_07_P055249	ECs2720	cmr|putative host specificity protein [ECs2720]
ECs2720	+2.55	8.55	+1.35	7.88	0.1	9.23	0.3	0.00124867	0.00766733	0.03781504	0.48800000	A_07_P055249	ECs2720	cmr|putative host specificity protein [ECs2720]
ECs2727	-3.33	10.19	-1.74	11.06	0.7	9.32	0.1	0.00486793	0.02234424	0.01354421	0.34843137	A_07_P055278	ECs2727	cmr|putative minor tail protein [ECs2727]
ECs2734	+3.10	10.45	+1.63	9.64	0.1	11.27	0.2	0.00026154	0.00242357	0.02561230	0.42491043	A_07_P055282	ECs2734	cmr|putative head completion protein [ECs2734]
ECs2746	-2.02	8.50	-1.01	9.01	0.3	8.00	0.2	0.00789677	0.03261459	0.07033155	0.66226077	A_07_P055321	ECs2746	cmr|hypothetical protein [ECs2746]
ECs2754	-2.59	15.37	-1.37	16.06	0.1	14.69	0.4	0.00351074	0.01715491	0.03289444	0.50575195	A_07_P055361	ECs2754	cmr|prophage maintenance protein [ECs2754]

ECs2771	-2.45	8.10	-1.29	8.75	0.3	7.46	0.0	0.00199255	0.01101912	0.03836573	0.53078207	A_07_P055443	ECs2771	cmr|hypothetical protein [ECs2771]
ECs2773	-3.27	10.95	-1.71	11.81	0.0	10.10	0.1	0.00009303	0.00113624	0.01456047	0.36157729	A_07_P055453	ECs2773	cmr|putative integrase [ECs2773]
ECs2782	-7.90	8.67	-2.98	10.16	0.1	7.18	0.1	0.00000223	0.00010815	0.00058943	0.07733333	A_07_P055462	ECs2782	cmr|hypothetical protein [ECs2782]
ECs2792	-7.36	8.62	-2.88	10.06	0.0	7.18	0.0	0.00000240	0.00011310	0.00078443	0.08758865	A_07_P055475	ECs2792	cmr|hypothetical protein [ECs2792]
ECs2793	-3.49	8.70	-1.80	9.60	0.1	7.80	0.5	0.00127265	0.00777212	0.01096418	0.32631380	A_07_P055479	ECs2793	cmr|hypothetical protein [ECs2793]
ECs2795	-8.48	10.56	-3.08	12.10	0.1	9.02	0.2	0.00000487	0.00016794	0.00043318	0.06621359	A_07_P055487	ECs2795	cmr|putative transposase OrfB protein of insertion sequence IS629 [ECs2795]
ECs2796	-6.27	11.79	-2.65	13.11	0.0	10.46	0.0	0.00000411	0.00015128	0.00142403	0.12455556	A_07_P055493	ECs2796	cmr|hypothetical protein [ECs2796]
ECs2797	-2.91	9.98	-1.54	10.76	0.1	9.21	0.2	0.00023492	0.00224565	0.02153328	0.42166667	A_07_P055498	ECs2797	cmr|hypothetical protein [ECs2797]
ECs2798	-2.18	10.70	-1.12	11.26	0.1	10.14	0.1	0.00099124	0.00648904	0.05731961	0.61600000	A_07_P055501	ECs2798	cmr|hypothetical protein [ECs2798]
ECs2814	+2.34	7.50	+1.23	6.88	0.0	8.11	0.3	0.00230613	0.01233699	0.04516260	0.52630644	A_07_P055548	ECs2814	cmr|hypothetical protein [ECs2814]
ECs2825	+8.14	8.79	+3.03	7.28	0.2	10.30	0.1	0.00000923	0.00023970	0.00376524	0.17748503	A_07_P062811	ECs2825	cmr|N- [ECs2825]
ECs2827	+2.39	7.39	+1.26	6.76	0.1	8.02	0.1	0.00050057	0.00390530	0.04284616	0.51298099	A_07_P055553	ECs2827	cmr|phosphoribosyl-amp cyclohydrolase [ECs2827]
ECs2828	-2.13	8.98	-1.09	9.52	0.1	8.43	0.2	0.00239789	0.01271122	0.06154789	0.63583333	A_07_P055560	ECs2828	cmr|regulator of length of O-antigen component of lipopolysaccharide chains [ECs2828]
ECs2829	-3.42	8.85	-1.77	9.73	0.0	7.96	0.2	0.00010834	0.00127097	0.01232025	0.33733913	A_07_P055562	ECs2829	cmr|UDP-glucose 6-dehydrogenase [ECs2829]
ECs2845	-3.26	10.37	-1.70	11.22	0.0	9.51	0.1	0.00006995	0.00093569	0.01480882	0.36601256	A_07_P055631	ECs2845	cmr|putative glycosyl transferase [ECs2845]
ECs2905	-3.13	8.55	-1.65	9.38	0.1	7.73	0.3	0.00034892	0.00298717	0.01658028	0.37777135	A_07_P062818	ECs2905	cmr|hypothetical protein [ECs2905]
ECs2907	-2.21	8.94	-1.14	9.52	0.1	8.37	0.3	0.00359648	0.01750326	0.05222752	0.59412572	A_07_P055723	ECs2907	cmr|hydoxyethylthiazole kinase [ECs2907]
ECs2910	-2.05	8.32	-1.04	8.84	0.2	7.80	0.0	0.00228787	0.01224763	0.06844766	0.65669714	A_07_P055739	ECs2910	cmr|putative outer membrane protein [ECs2910]
ECs2912	-2.06	9.97	-1.04	10.49	0.0	9.45	0.2	0.00188318	0.01051561	0.06792810	0.65490508	A_07_P055744	ECs2912	cmr|hypothetical protein [ECs2912]
ECs2918	-2.48	8.90	-1.31	9.55	0.1	8.25	0.1	0.00040644	0.00336751	0.03817518	0.52861038	A_07_P055764	ECs2918	cmr|hypothetical protein [ECs2918]
ECs2933	-4.42	9.04	-2.14	10.11	0.1	7.96	0.1	0.00002219	0.00041939	0.00480691	0.22726727	A_07_P055796	ECs2933	cmr|putative transposase OrfA protein of insertion sequence IS629 [ECs2933]
ECs2946	-2.50	10.64	-1.32	11.30	0.6	9.98	0.0	0.01198888	0.04543883	0.03508702	0.51149074	A_07_P055824	ECs2946	cmr|putative tail assembly protein [ECs2946]
ECs2963	-2.88	11.31	-1.53	12.08	0.0	10.55	0.1	0.00018346	0.00187806	0.02265561	0.43658507	A_07_P052840	ECs2963	cmr|putative terminase large subunit [ECs2963]
ECs2975	-2.52	10.08	-1.33	10.75	0.1	9.42	0.0	0.00030648	0.00272199	0.03607215	0.51817518	A_07_P052866	ECs2975	cmr|antitermination protein [ECs2975]
ECs2978	-2.54	8.46	-1.35	9.14	0.1	7.79	0.0	0.00028576	0.00259753	0.03464240	0.51308561	A_07_P052883	ECs2978	cmr|hypothetical protein [ECs2978]
ECs2981	+13.07	8.36	+3.71	6.50	0.2	10.21	0.7	0.00008847	0.00109678	0.00141832	0.11569948	A_07_P052891	ECs2981	cmr|putative DNA methylase [ECs2981]
ECs2985	+6.42	12.82	+2.68	11.47	0.0	14.16	0.2	0.00001043	0.00025872	0.00602071	0.22569048	A_07_P052912	ECs2985	cmr|Ren protein [ECs2985]
ECs2986	+4.12	14.13	+2.04	13.11	0.1	15.15	0.0	0.00002245	0.00042379	0.01511052	0.33179916	A_07_P052916	ECs2986	cmr|phage replication protein P [ECs2986]
ECs2987	+5.92	10.74	+2.57	9.45	0.2	12.02	0.3	0.00004476	0.00068082	0.00725165	0.24239915	A_07_P052921	ECs2987	cmr|phage replication protein O [ECs2987]
ECs2988	+3.25	9.25	+1.70	8.39	0.2	10.10	0.2	0.00027822	0.00254813	0.02304179	0.40532961	A_07_P052928	ECs2988	cmr|regulatory protein CII [ECs2988]
ECs2996	+10.03	13.37	+3.33	11.71	0.1	15.04	0.0	0.00000104	0.00007542	0.00258702	0.14864964	A_07_P052967	ECs2996	cmr|putative single-stranded DNA binding protein [ECs2996]
ECs2997	+5.88	13.10	+2.56	11.82	0.1	14.38	0.4	0.00006317	0.00087006	0.00742823	0.24466527	A_07_P052972	ECs2997	cmr|regulatory protein cIII [ECs2997]
ECs2999	+11.27	12.02	+3.49	10.27	0.2	13.77	0.6	0.00009347	0.00113992	0.00196519	0.13110169	A_07_P052981	ECs2999	cmr|host-nuclease inhibitor protein gam [ECs2999]
ECs3000	+13.36	11.01	+3.74	9.14	0.0	12.88	0.1	0.00000060	0.00005729	0.00139037	0.11521053	A_07_P052986	ECs3000	cmr|putative host-nuclease inhibitor protein Gam [ECs3000]
ECs3001	+3.79	13.04	+1.92	12.08	0.1	14.00	0.1	0.00005067	0.00074622	0.01728023	0.34879487	A_07_P052993	ECs3001	cmr|recombination protein Bet [ECs3001]
ECs3002	+2.72	11.79	+1.45	11.06	0.0	12.51	0.1	0.00022114	0.00215178	0.03255843	0.46138614	A_07_P053000	ECs3002	cmr|exonuclease [ECs3002]
ECs3003	+21.83	11.53	+4.45	9.31	0.1	13.76	0.2	0.00000042	0.00004998	0.00036014	0.05504854	A_07_P053004	ECs3003	cmr|hypothetical protein [ECs3003]
ECs3004	+21.07	9.93	+4.40	7.73	0.2	12.13	0.3	0.00000206	0.00010411	0.00043636	0.06079646	A_07_P053006	ECs3004	cmr|hypothetical protein [ECs3004]
ECs3005	+21.79	11.25	+4.45	9.03	0.3	13.47	0.3	0.00000369	0.00014246	0.00036522	0.05424528	A_07_P053011	ECs3005	cmr|hypothetical protein [ECs3005]
ECs3006	+28.28	9.18	+4.82	6.77	0.1	11.59	0.2	0.00000033	0.00004520	0.00016959	0.03760563	A_07_P053018	ECs3006	cmr|putative C4-type zinc finger protein [ECs3006]
ECs3042	+27.58	10.54	+4.79	8.15	0.1	12.93	0.2	0.00000025	0.00004211	0.00017721	0.03671053	A_07_P053100	ECs3042	cmr|galactose-binding transport protein [ECs3042]
ECs3098	+2.26	13.08	+1.18	12.50	0.1	13.67	0.2	0.00096434	0.00635518	0.04751778	0.53976912	A_07_P053126	ECs3098	cmr|ecotin, a serine protease inhibitor [ECs3098]
ECs3104	+2.33	10.39	+1.22	9.78	0.0	11.00	0.1	0.00057539	0.00430357	0.04496888	0.52560505	A_07_P053132	ECs3104	cmr|outer membrane protein C OmpC [ECs3104]
ECs3152	+2.19	8.75	+1.13	8.18	0.0	9.31	0.0	0.00066086	0.00474229	0.05142276	0.55988935	A_07_P053169	ECs3152	cmr|2-oxoglutarate decarboxylase [ECs3152]
ECs3152	+2.46	8.69	+1.30	8.04	0.0	9.34	0.2	0.00052906	0.00404719	0.03998730	0.49689029	A_07_P053169	ECs3152	cmr|2-oxoglutarate decarboxylase [ECs3152]
ECs3152	+2.52	8.82	+1.33	8.15	0.2	9.48	0.2	0.00081632	0.00559521	0.03839113	0.48902104	A_07_P053169	ECs3152	cmr|2-oxoglutarate decarboxylase [ECs3152]
ECs3160	+5.73	8.03	+2.52	6.77	0.1	9.28	0.1	0.00000849	0.00022916	0.00770389	0.24702648	A_07_P062826	ECs3160	cmr|NADH dehydrogenase I chain N [ECs3160]
ECs3163	+26.31	9.46	+4.72	7.10	0.1	11.82	0.1	0.00000016	0.00003548	0.00020325	0.03950617	A_07_P053173	ECs3163	cmr|NADH dehydrogenase I chain K [ECs3163]
ECs3167	+12.69	10.09	+3.67	8.26	0.1	11.93	0.4	0.00000681	0.00019979	0.00148183	0.11665000	A_07_P053176	ECs3167	cmr|NADH dehydrogenase I chain G [ECs3167]
ECs3169	+7.42	11.86	+2.89	10.41	0.2	13.30	0.0	0.00000796	0.00022222	0.00447027	0.19073171	A_07_P063350	ECs3169	cmr|NADH dehydrogenase I chain E [ECs3169]
ECs3179	+3.41	10.25	+1.77	9.37	0.0	11.14	0.2	0.00008388	0.00105814	0.02139990	0.38637615	A_07_P053181	ECs3179	cmr|hypothetical protein [ECs3179]
ECs3189	-2.78	8.58	-1.47	9.32	0.3	7.85	0.1	0.00090774	0.00608664	0.02529853	0.45940023	A_07_P053190	ECs3189	cmr|hypothetical protein [ECs3189]
ECs3190	+2.48	7.49	+1.31	6.83	0.0	8.14	0.2	0.00054163	0.00411349	0.03948615	0.49496019	A_07_P053195	ECs3190	cmr|ATP-binding component of histidine transport [ECs3190]
ECs3196	+4.57	9.00	+2.19	7.90	0.0	10.10	0.1	0.00001777	0.00036419	0.01230691	0.29947450	A_07_P053197	ECs3196	cmr|amidophosphoribosyltransferase [ECs3196]
ECs3197	+4.22	10.73	+2.08	9.69	0.0	11.77	0.1	0.00002886	0.00050012	0.01445694	0.32469330	A_07_P053205	ECs3197	cmr|membrane protein required for colicin V production [ECs3197]
ECs3236	-2.63	8.95	-1.40	9.65	0.4	8.25	0.0	0.00220897	0.01194579	0.03010925	0.48719424	A_07_P053303	ECs3236	cmr|putativa replication protein [ECs3236]
ECs3243	-2.08	8.33	-1.06	8.86	0.1	7.80	0.1	0.00196570	0.01090487	0.06587398	0.65104834	A_07_P053337	ECs3243	cmr|sucrose-6 phosphate hydrolase [ECs3243]
ECs3296	-6.26	10.97	-2.65	12.30	0.1	9.65	0.0	0.00000473	0.00016577	0.00142403	0.12525140	A_07_P053399	ECs3296	cmr|thiosulfate binding protein [ECs3296]
ECs3306	-5.75	11.72	-2.52	12.99	0.0	10.46	0.0	0.00000583	0.00018275	0.00191247	0.14545894	A_07_P063373	ECs3306	cmr|N-acetylmuramoyl-l-alanine amidase I [ECs3306]
ECs3311	+2.14	7.36	+1.10	6.81	0.0	7.91	0.1	0.00090470	0.00607145	0.05415269	0.56990642	A_07_P053415	ECs3311	cmr|ethanolamine ammonia-lyase light chain [ECs3311]
ECs3370	+4.75	11.25	+2.25	10.12	0.1	12.37	0.1	0.00002874	0.00049935	0.01153455	0.29337641	A_07_P053430	ECs3370	cmr|IMP dehydrogenase [ECs3370]
ECs3382	-2.65	11.39	-1.41	12.10	0.1	10.69	0.1	0.00025197	0.00235847	0.02969322	0.48444560	A_07_P053440	ECs3382	cmr|putative anaerobic dimethyl sulfoxide reductase chain C [ECs3382]
ECs3398	-9.71	13.17	-3.28	14.81	0.0	11.53	0.3	0.00001067	0.00026319	0.00025089	0.05129870	A_07_P053458	ECs3398	cmr|putative ATP synthase beta subunit [ECs3398]
ECs3442	+3.25	8.53	+1.70	7.68	0.3	9.38	0.1	0.00056128	0.00422410	0.02332508	0.40848721	A_07_P053491	ECs3442	cmr|ATP-dependent RNA helicase [ECs3442]
ECs3450	+2.89	9.49	+1.53	8.73	0.0	10.26	0.5	0.00310463	0.01553432	0.02996126	0.44459001	A_07_P053496	ECs3450	cmr|hypothetical protein [ECs3450]
ECs3489	-2.44	11.79	-1.29	12.44	0.3	11.15	0.2	0.00189486	0.01056400	0.03962907	0.53972318	A_07_P055872	ECs3489	cmr|hypothetical protein [ECs3489]
ECs3492	-3.98	8.60	-1.99	9.60	0.0	7.61	0.1	0.00002814	0.00049161	0.00720084	0.27122010	A_07_P055876	ECs3492	cmr|hypothetical protein [ECs3492]
ECs3535	+3.02	8.39	+1.59	7.59	0.0	9.19	0.2	0.00019251	0.00194658	0.02681720	0.42777102	A_07_P055985	ECs3535	cmr|hypothetical protein [ECs3535]
ECs3569	-3.46	8.00	-1.79	8.90	0.1	7.11	0.0	0.00005502	0.00078754	0.01185658	0.33634234	A_07_P055993	ECs3569	cmr|electron transport protein HydN [ECs3569]
ECs3586	+2.32	11.30	+1.21	10.69	0.1	11.90	0.2	0.00109295	0.00695496	0.04620173	0.53094891	A_07_P056010	ECs3586	cmr|HypE protein [ECs3586]
ECs3591	+3.70	8.74	+1.89	7.79	0.0	9.68	0.5	0.00132479	0.00801289	0.01786268	0.35286073	A_07_P056017	ECs3591	cmr|hypothetical protein [ECs3591]
ECs3594	-2.44	9.40	-1.29	10.04	0.1	8.75	0.1	0.00050640	0.00393463	0.03994855	0.54079966	A_07_P056034	ECs3594	cmr|transcriptional regulator [ECs3594]
ECs3610	-2.42	9.02	-1.27	9.66	0.3	8.38	0.1	0.00182266	0.01027329	0.04047383	0.54323956	A_07_P056055	ECs3610	cmr|hypothetical protein [ECs3610]
ECs3622	-2.31	8.40	-1.21	9.00	0.5	7.80	0.1	0.01026192	0.04008028	0.04671748	0.57462500	A_07_P056090	ECs3622	cmr|hypothetical protein [ECs3622]
ECs3649	+2.47	7.88	+1.30	7.23	0.1	8.53	0.2	0.00067812	0.00483092	0.04014672	0.49769291	A_07_P063411	ECs3649	cmr|putative transport protein [ECs3649]
ECs3663	+5.74	11.79	+2.52	10.53	0.0	13.05	0.1	0.00000832	0.00022675	0.00760353	0.24784679	A_07_P056139	ECs3663	cmr|L-fuculokinase [ECs3663]
ECs3670	-2.42	9.27	-1.28	9.91	0.1	8.64	0.1	0.00044505	0.00359144	0.04080221	0.54393734	A_07_P056142	ECs3670	cmr|hypothetical protein [ECs3670]
ECs3683	+2.87	7.98	+1.52	7.22	0.2	8.75	0.0	0.00027752	0.00254324	0.02939533	0.44287081	A_07_P056146	ECs3683	cmr|prepilin peptidase dependent protein A [ECs3683]
ECs3734	-2.15	8.88	-1.10	9.43	0.2	8.33	0.1	0.00277533	0.01423748	0.05826601	0.62066306	A_07_P056259	ECs3734	cmr|type III secretion protein EivF [ECs3734]
ECs3758	-3.11	8.76	-1.64	9.58	0.2	7.95	0.1	0.00018059	0.00186075	0.01706491	0.37681627	A_07_P056278	ECs3758	cmr|putative oxidoreductase Fe-S subunit [ECs3758]
ECs3764	-3.46	9.28	-1.79	10.17	0.2	8.38	0.1	0.00011662	0.00133244	0.01186992	0.33551167	A_07_P056285	ECs3764	cmr|ssDNA exonuclease RecJ [ECs3764]
ECs3771	+3.03	9.27	+1.60	8.47	0.2	10.07	0.1	0.00025789	0.00239826	0.02665142	0.42685656	A_07_P063449	ECs3771	cmr|putative oxidoreductase [ECs3771]
ECs3772	+2.07	7.77	+1.05	7.25	0.1	8.30	0.0	0.00122744	0.00757240	0.05869157	0.59271328	A_07_P056288	ECs3772	cmr|hypothetical protein [ECs3772]
ECs3774	+6.98	9.11	+2.80	7.71	0.1	10.52	0.1	0.00000441	0.00015907	0.00513211	0.20771208	A_07_P063454	ECs3774	cmr|glycine decarboxylase [ECs3774]
ECs3778	+2.26	11.88	+1.18	11.29	0.0	12.47	0.0	0.00054133	0.00411349	0.04798844	0.54393808	A_07_P056295	ECs3778	cmr|2-octaprenyl-6-methoxyphenol 4-monoxygenase [ECs3778]
ECs3792	-2.01	7.30	-1.00	7.80	0.2	6.80	0.3	0.00688415	0.02931845	0.07213605	0.66845792	A_07_P056301	ECs3792	cmr|putative transcriptional regulator LYSR-type [ECs3792]
ECs3799	+2.12	7.28	+1.08	6.74	0.1	7.82	0.1	0.00144275	0.00851376	0.05520452	0.57520847	A_07_P056312	ECs3799	cmr|hypothetical protein [ECs3799]
ECs3800	+10.08	8.50	+3.33	6.83	0.1	10.16	0.1	0.00000139	0.00008723	0.00251524	0.14666667	A_07_P056318	ECs3800	cmr|putative transporter [ECs3800]
ECs3801	+4.85	8.67	+2.28	7.53	0.1	9.81	0.1	0.00001637	0.00034464	0.01090638	0.28523256	A_07_P056323	ECs3801	cmr|hypothetical lipoprotein [ECs3801]
ECs3802	+2.19	7.30	+1.13	6.74	0.0	7.87	0.0	0.00065923	0.00473490	0.05099022	0.55943554	A_07_P056330	ECs3802	cmr|putative ABC transporter ATP-binding subunit [ECs3802]
ECs3810	+3.19	8.78	+1.67	7.94	0.6	9.62	0.1	0.00300656	0.01519593	0.02469131	0.41576471	A_07_P056341	ECs3810	cmr|transketolase 1 isozyme [ECs3810]
ECs3844	-5.27	9.62	-2.40	10.82	0.0	8.42	0.0	0.00000751	0.00021366	0.00262449	0.17216667	A_07_P056361	ECs3844	cmr|hypothetical protein [ECs3844]
ECs3850	-2.00	7.41	-1.00	7.91	0.4	6.91	0.1	0.01311864	0.04894311	0.07108168	0.66574063	A_07_P056394	ECs3850	cmr|putative virulence-related membrane protein [ECs3850]
ECs3860	-3.02	9.33	-1.59	10.13	0.3	8.53	0.0	0.00050219	0.00391025	0.01923844	0.39801577	A_07_P056442	ECs3860	cmr|putative adherence factor [ECs3860]
ECs3869	-3.13	11.68	-1.65	12.50	0.1	10.86	0.0	0.00007988	0.00101674	0.01701283	0.37939093	A_07_P056468	ECs3869	cmr|hypothetical protein [ECs3869]
ECs3870	-3.39	9.01	-1.76	9.89	0.3	8.13	0.1	0.00040870	0.00337771	0.01247840	0.33756014	A_07_P056475	ECs3870	cmr|hypothetical protein [ECs3870]
ECs3874	+2.39	9.70	+1.26	9.07	0.2	10.33	0.1	0.00109314	0.00695496	0.04325711	0.51476946	A_07_P056483	ECs3874	cmr|hypothetical protein [ECs3874]
ECs3878	+2.82	11.55	+1.50	10.80	0.0	12.30	0.3	0.00048376	0.00381767	0.03069360	0.44869081	A_07_P056488	ECs3878	cmr|hydrogenase 2 maturation protease HybD [ECs3878]
ECs3916	-2.54	8.18	-1.34	8.85	0.1	7.50	0.0	0.00029464	0.00264925	0.03484820	0.51275701	A_07_P056524	ECs3916	cmr|putative ABC-type iron-siderophore transport system ATP-binding protein [ECs3916]
ECs3930	-2.62	13.09	-1.39	13.78	0.1	12.40	0.0	0.00023533	0.00224686	0.03133003	0.49673716	A_07_P056534	ECs3930	cmr|hypothetical protein [ECs3930]
ECs3995	+78.34	10.40	+6.29	7.25	0.2	13.54	0.1	0.00000006	0.00003344	0.00000508	0.00571429	A_07_P059659	ECs3995	cmr|putative kinase [ECs3995]
ECs4006	+4.58	9.21	+2.20	8.11	0.1	10.31	0.1	0.00002383	0.00044188	0.01228404	0.29938080	A_07_P059666	ECs4006	cmr|putative hydrolase [ECs4006]
ECs4013	+3.79	7.80	+1.92	6.84	0.2	8.76	0.2	0.00016468	0.00174012	0.01763402	0.35098609	A_07_P059671	ECs4013	cmr|N-acetylgalactosamine-specific PTS system enzyme IID component [ECs4013]
ECs4014	+6.62	8.47	+2.73	7.10	0.1	9.83	0.2	0.00001271	0.00029373	0.00573806	0.21874092	A_07_P059680	ECs4014	cmr|N-acetyl-galactosamine / galactosamine PTS system enzyme IIA component [ECs4014]
ECs4014	+3.59	8.68	+1.84	7.76	0.1	9.60	0.2	0.00009339	0.00113982	0.01970528	0.36801898	A_07_P059680	ECs4014	cmr|N-acetyl-galactosamine / galactosamine PTS system enzyme IIA component [ECs4014]
ECs4014	+4.18	8.18	+2.06	7.15	0.3	9.21	0.3	0.00033708	0.00290798	0.01449822	0.32515670	A_07_P059680	ECs4014	cmr|N-acetyl-galactosamine / galactosamine PTS system enzyme IIA component [ECs4014]
ECs4014	+2.89	8.52	+1.53	7.76	0.1	9.29	0.4	0.00140761	0.00836504	0.02966146	0.44348528	A_07_P059680	ECs4014	cmr|N-acetyl-galactosamine / galactosamine PTS system enzyme IIA component [ECs4014]
ECs4014	+4.27	8.61	+2.10	7.56	0.1	9.65	0.1	0.00003950	0.00062195	0.01380018	0.31764620	A_07_P059680	ECs4014	cmr|N-acetyl-galactosamine / galactosamine PTS system enzyme IIA component [ECs4014]
ECs4023	-32.65	11.78	-5.03	14.29	0.1	9.27	0.1	0.00000009	0.00003427	0.00000318	0.00454545	A_07_P059688	ECs4023	cmr|truncated putative fimbrial protein [ECs4023]
ECs4052	+2.83	8.49	+1.50	7.74	0.1	9.24	0.0	0.00019629	0.00196842	0.03002541	0.44428571	A_07_P059701	ECs4052	cmr|argininosuccinate synthetase [ECs4052]
ECs4086	+3.51	9.65	+1.81	8.74	0.0	10.55	0.1	0.00004735	0.00070931	0.02028709	0.37356725	A_07_P063500	ECs4086	cmr|hypothetical protein [ECs4086]
ECs4094	+4.24	8.11	+2.08	7.07	0.0	9.16	0.0	0.00001848	0.00037316	0.01423336	0.32243165	A_07_P059712	ECs4094	cmr|hypothetical protein [ECs4094]
ECs4098	+2.99	7.94	+1.58	7.15	0.0	8.73	0.1	0.00014144	0.00154753	0.02700140	0.42767606	A_07_P059716	ECs4098	cmr|N-acetylneuraminate lyase [ECs4098]
ECs4115	-4.50	8.11	-2.17	9.20	0.1	7.03	0.2	0.00002624	0.00046900	0.00444931	0.21822430	A_07_P058957	ECs4115	cmr|hypothetical protein [ECs4115]
ECs4150	+2.53	10.80	+1.34	10.13	0.0	11.47	0.2	0.00085313	0.00580363	0.03798145	0.48735126	A_07_P059737	ECs4150	cmr|hypothetical protein [ECs4150]
ECs4196	-2.24	9.85	-1.16	10.44	0.2	9.27	0.1	0.00184277	0.01035794	0.05228786	0.59352559	A_07_P063525	ECs4196	cmr|hypothetical protein [ECs4196]
ECs4237	+2.39	7.30	+1.26	6.67	0.1	7.92	0.0	0.00041156	0.00339421	0.04241108	0.51087988	A_07_P059765	ECs4237	cmr|hypothetical protein [ECs4237]
ECs4253	-2.89	9.17	-1.53	9.94	0.1	8.41	0.5	0.00318230	0.01585526	0.02280805	0.43791463	A_07_P063536	ECs4253	cmr|hypothetical protein [ECs4253]
ECs4260	+4.40	8.29	+2.14	7.23	0.2	9.36	0.5	0.00048185	0.00380646	0.01285188	0.30611195	A_07_P063542	ECs4260	cmr|positive regulator of mal regulon [ECs4260]
ECs4282	+2.73	8.55	+1.45	7.83	0.1	9.28	0.1	0.00028755	0.00260332	0.03274644	0.46280072	A_07_P059833	ECs4282	cmr|putative ATP-dependent DNA helicase [ECs4282]
ECs4292	-2.69	10.69	-1.43	11.41	0.5	9.98	0.2	0.00787207	0.03252107	0.02783473	0.47427489	A_07_P059864	ECs4292	cmr|hypothetical membrane protein [ECs4292]
ECs4325	-2.04	8.58	-1.03	9.09	0.0	8.06	0.1	0.00126043	0.00772450	0.06965701	0.65906250	A_07_P059892	ECs4325	cmr|putative O-methyltransferase [ECs4325]
ECs4329	-3.93	10.32	-1.97	11.30	0.0	9.33	0.0	0.00002480	0.00045357	0.00747586	0.27564403	A_07_P059912	ECs4329	cmr|putative acyl carrier protein [ECs4329]
ECs4331	-2.05	7.94	-1.03	8.46	0.2	7.42	0.2	0.00435358	0.02039497	0.06708778	0.65118989	A_07_P059921	ECs4331	cmr|putative surfactin synthetase [ECs4331]
ECs4332	-2.47	7.47	-1.31	8.12	0.0	6.82	0.2	0.00099625	0.00650846	0.03712589	0.52516622	A_07_P059928	ECs4332	cmr|putative [ECs4332]
ECs4334	+2.04	8.47	+1.03	7.96	0.1	8.98	0.1	0.00267386	0.01381863	0.06230246	0.60623609	A_07_P059939	ECs4334	cmr|hypothetical protein [ECs4334]
ECs4335	+2.09	7.92	+1.06	7.39	0.0	8.45	0.1	0.00100237	0.00654014	0.05710810	0.58345879	A_07_P059941	ECs4335	cmr|hypothetical protein [ECs4335]
ECs4337	+2.15	7.51	+1.10	6.96	0.0	8.06	0.4	0.00577740	0.02558586	0.05493140	0.57388188	A_07_P059955	ECs4337	cmr|hypothetical lipoprotein [ECs4337]
ECs4338	+2.52	7.71	+1.33	7.05	0.1	8.38	0.1	0.00048096	0.00380401	0.03803100	0.48679675	A_07_P059960	ECs4338	cmr|putative 3-oxoacyl- [ECs4338]
ECs4339	+2.54	7.18	+1.35	6.51	0.2	7.85	0.3	0.00211849	0.01155702	0.03847624	0.48931341	A_07_P059965	ECs4339	cmr|putative beta-hydroxydecanoyl-ACP dehydrase [ECs4339]
ECs4341	-2.10	8.79	-1.07	9.32	0.0	8.25	0.0	0.00090872	0.00609065	0.06443407	0.64738354	A_07_P059972	ECs4341	cmr|putative 3-oxoacyl- [ECs4341]
ECs4350	+2.86	7.59	+1.52	6.83	0.2	8.35	0.0	0.00030736	0.00272536	0.02974657	0.44391469	A_07_P059987	ECs4350	cmr|putative phosphotransferase system enzyme IIA [ECs4350]
ECs4367	+12.26	12.34	+3.62	10.53	0.1	14.15	0.1	0.00000105	0.00007542	0.00165206	0.12041667	A_07_P060029	ECs4367	cmr|universal stress protein UspA [ECs4367]
ECs4407	-2.78	9.30	-1.47	10.03	0.1	8.56	0.2	0.00026525	0.00245180	0.02575838	0.46241733	A_07_P060081	ECs4407	cmr|hypothetical protein [ECs4407]
ECs4458	-2.91	9.63	-1.54	10.40	0.3	8.86	0.5	0.00777489	0.03222538	0.02118839	0.41803258	A_07_P060126	ECs4458	cmr|putative permease [ECs4458]
ECs4475	+4.17	8.24	+2.06	7.21	0.1	9.27	0.1	0.00003411	0.00056193	0.01464304	0.32700709	A_07_P060156	ECs4475	cmr|mannitol-specific PTS system enzyme IIABC components [ECs4475]
ECs4503	-2.03	9.78	-1.02	10.29	0.1	9.27	0.1	0.00173019	0.00985948	0.07158473	0.66767180	A_07_P060202	ECs4503	cmr|lipopolysaccharide core biosynthesis protein WaaY [ECs4503]
ECs4505	-3.65	10.17	-1.87	11.10	0.2	9.23	0.3	0.00038193	0.00321037	0.00946011	0.31158996	A_07_P060214	ECs4505	cmr|lipopolysaccharide core biosynthesis protein RfaP [ECs4505]
ECs4506	-2.35	10.37	-1.23	10.99	0.1	9.75	0.4	0.00511001	0.02317834	0.04538808	0.56803657	A_07_P060217	ECs4506	cmr|glucosyltransferase I [ECs4506]
ECs4551	-2.00	8.43	-1.00	8.93	0.2	7.94	0.1	0.00311551	0.01558153	0.07470592	0.67518370	A_07_P057374	ECs4551	cmr|hypothetical protein [ECs4551]
ECs4579	-2.08	9.34	-1.06	9.87	0.4	8.81	0.1	0.01050366	0.04086227	0.06669715	0.64980198	A_07_P057512	ECs4579	cmr|hypothetical protein [ECs4579]
ECs4586	-2.19	9.32	-1.13	9.88	0.3	8.75	0.1	0.00365178	0.01770167	0.05597497	0.61114424	A_07_P057546	ECs4586	cmr|hypothetical protein [ECs4586]
ECs4586	-2.37	9.22	-1.24	9.84	0.3	8.59	0.0	0.00171186	0.00977922	0.04296176	0.55807756	A_07_P057546	ECs4586	cmr|hypothetical protein [ECs4586]
ECs4586	-2.07	9.25	-1.05	9.77	0.3	8.72	0.1	0.00370105	0.01782482	0.06524390	0.64971537	A_07_P057546	ECs4586	cmr|hypothetical protein [ECs4586]
ECs4587	-2.44	7.66	-1.29	8.31	0.0	7.02	0.1	0.00051689	0.00397939	0.03925432	0.53694179	A_07_P057552	ECs4587	cmr|hypothetical protein [ECs4587]
ECs4592	-2.67	8.08	-1.41	8.78	0.3	7.37	0.3	0.00258540	0.01349367	0.02821075	0.47502674	A_07_P057572	ECs4592	cmr|hypothetical protein [ECs4592]
ECs4594	+2.51	8.56	+1.33	7.89	0.2	9.22	0.1	0.00061150	0.00448415	0.03876397	0.49178082	A_07_P063596	ECs4594	cmr|putative permease transporter [ECs4594]
ECs4630	-2.43	7.44	-1.28	8.08	0.0	6.80	0.1	0.00034527	0.00296290	0.04036014	0.54356715	A_07_P062852	ECs4630	cmr|hypothetical protein [ECs4630]
ECs4742	+2.91	8.80	+1.54	8.03	0.1	9.57	0.1	0.00014203	0.00155099	0.02836636	0.43827282	A_07_P057739	ECs4742	cmr|putative phosphatase [ECs4742]
ECs4786	+3.71	8.60	+1.89	7.66	0.2	9.55	0.3	0.00042922	0.00350320	0.01768102	0.35147727	A_07_P063635	ECs4786	cmr|DNA polymerase I [ECs4786]
ECs4787	+4.47	9.52	+2.16	8.44	0.0	10.60	0.0	0.00001444	0.00031530	0.01290841	0.30560902	A_07_P057780	ECs4787	cmr|essential GTPase for cell cycle [ECs4787]
ECs4819	+3.64	11.23	+1.86	10.30	0.0	12.16	0.1	0.00004073	0.00063615	0.01905170	0.36225845	A_07_P057834	ECs4819	cmr|formate dehydrogenase-O iron-sulfur subunit [ECs4819]
ECs4820	+2.12	11.02	+1.09	10.48	0.1	11.56	0.1	0.00095611	0.00631683	0.05516514	0.57517881	A_07_P057838	ECs4820	cmr|formate dehydrogenase-O major subunit [ECs4820]
ECs4832	+2.90	7.91	+1.54	7.14	0.0	8.68	0.0	0.00011400	0.00131780	0.02852388	0.43898338	A_07_P057869	ECs4832	cmr|positive regulator for rhaBAD operon [ECs4832]
ECs4847	+4.63	8.67	+2.21	7.57	0.2	9.78	0.3	0.00013737	0.00151557	0.01163046	0.29297600	A_07_P057876	ECs4847	cmr|hypothetical protein [ECs4847]
ECs4848	+3.90	9.95	+1.96	8.97	0.1	10.94	0.3	0.00011171	0.00129703	0.01623158	0.34302013	A_07_P057881	ECs4848	cmr|putative regulator [ECs4848]
ECs4866	-3.30	9.40	-1.72	10.26	0.1	8.54	0.0	0.00007333	0.00096133	0.01415587	0.35602236	A_07_P063657	ECs4866	cmr|hypothetical protein [ECs4866]
ECs4892	-3.99	12.26	-2.00	13.26	0.5	11.26	0.3	0.00151961	0.00888737	0.00746126	0.27575117	A_07_P057920	ECs4892	cmr|putative amino acid amidohydrolase [ECs4892]
ECs4893	-2.29	11.45	-1.20	12.05	0.2	10.85	0.3	0.00397786	0.01892068	0.04817200	0.58071975	A_07_P057923	ECs4893	cmr|putative membrane transport / symporter protein [ECs4893]
ECs4893	-4.69	11.76	-2.23	12.88	0.3	10.65	0.1	0.00013181	0.00146867	0.00415333	0.21230519	A_07_P057923	ECs4893	cmr|putative membrane transport / symporter protein [ECs4893]
ECs4893	-2.98	11.69	-1.58	12.48	0.1	10.90	0.1	0.00016816	0.00176652	0.02058308	0.41281529	A_07_P057923	ECs4893	cmr|putative membrane transport / symporter protein [ECs4893]
ECs4893	-2.31	11.50	-1.21	12.10	0.1	10.89	0.0	0.00051662	0.00397929	0.04776613	0.57759601	A_07_P057923	ECs4893	cmr|putative membrane transport / symporter protein [ECs4893]
ECs4893	-2.03	11.47	-1.02	11.98	0.4	10.96	0.1	0.00957553	0.03798364	0.07199251	0.66791397	A_07_P057923	ECs4893	cmr|putative membrane transport / symporter protein [ECs4893]
ECs4896	-3.37	8.99	-1.75	9.86	0.1	8.11	0.3	0.00050104	0.00390706	0.01316946	0.34614357	A_07_P057927	ECs4896	cmr|tRNA [ECs4896]
ECs4921	+5.14	9.38	+2.36	8.20	0.0	10.56	0.1	0.00000910	0.00023883	0.00947599	0.26832734	A_07_P057952	ECs4921	cmr|endonuclease V [ECs4921]
ECs4956	-2.13	9.17	-1.09	9.72	0.0	8.63	0.5	0.01175805	0.04470386	0.06160823	0.63562254	A_07_P061239	ECs4956	cmr|hypothetical protein [ECs4956]
ECs4978	-2.30	8.63	-1.20	9.23	0.2	8.03	0.1	0.00115195	0.00721126	0.04722624	0.57504254	A_07_P061350	ECs4978	cmr|hypothetical protein [ECs4978]
ECs4980	-3.50	10.09	-1.81	10.99	0.4	9.19	0.3	0.00105029	0.00676135	0.01170732	0.33573770	A_07_P061358	ECs4980	cmr|hypothetical protein [ECs4980]
ECs4994	-2.22	9.43	-1.15	10.00	0.4	8.85	0.2	0.00944792	0.03761962	0.05445058	0.60541667	A_07_P061427	ECs4994	cmr|hypothetical protein [ECs4994]
ECs5000	-2.14	7.43	-1.09	7.97	0.3	6.88	0.2	0.00731370	0.03067313	0.06135925	0.63555263	A_07_P061459	ECs5000	cmr|sorbose-permease PTS system IIC component [ECs5000]
ECs5003	-2.15	7.61	-1.11	8.16	0.1	7.06	0.4	0.00624639	0.02717412	0.05674924	0.61533058	A_07_P061472	ECs5003	cmr|putative sorbitol-6-phosphate 2-dehydrogenase [ECs5003]
ECs5043	-2.49	8.36	-1.32	9.02	0.1	7.70	0.1	0.00060027	0.00443275	0.03741044	0.52635389	A_07_P063693	ECs5043	cmr|hypothetical protein [ECs5043]
ECs5051	+13.46	8.78	+3.75	6.90	0.2	10.65	0.2	0.00000245	0.00011451	0.00137449	0.11510638	A_07_P063704	ECs5051	cmr|acetyl-CoA synthetase [ECs5051]
ECs5057	-2.24	9.12	-1.17	9.70	0.4	8.54	0.1	0.00857277	0.03469656	0.05091336	0.58983076	A_07_P063709	ECs5057	cmr|cytochrome c-type biogenesis protein NrfF [ECs5057]
ECs5065	-2.89	7.75	-1.53	8.52	0.0	6.99	0.1	0.00012784	0.00143388	0.02277121	0.43774115	A_07_P061508	ECs5065	cmr|hypothetical protein [ECs5065]
ECs5104	+2.74	8.10	+1.45	7.37	0.0	8.82	0.0	0.00015352	0.00164984	0.03222624	0.45915837	A_07_P061567	ECs5104	cmr|fumarase B [ECs5104]
ECs5129	-3.49	9.48	-1.80	10.39	0.0	8.58	0.1	0.00004991	0.00073997	0.01138148	0.33306691	A_07_P058973	ECs5129	cmr|suppressor of groEL [ECs5129]
ECs5165	+13.24	11.18	+3.73	9.32	0.0	13.05	0.1	0.00000082	0.00006559	0.00141768	0.11625000	A_07_P058980	ECs5165	cmr|hypothetical protein [ECs5165]
ECs5249	+2.43	7.10	+1.28	6.46	0.2	7.74	0.2	0.00087492	0.00591949	0.04181339	0.50834749	A_07_P061654	ECs5249	cmr|putative resolvase [ECs5249]
ECs5256	-2.41	9.24	-1.27	9.88	0.1	8.61	0.1	0.00065042	0.00468443	0.04141832	0.54843566	A_07_P061690	ECs5256	cmr|hypothetical protein [ECs5256]
ECs5282	+7.60	8.67	+2.93	7.21	0.1	10.14	0.2	0.00000530	0.00017598	0.00427210	0.18840336	A_07_P061751	ECs5282	cmr|D-mannonate oxidoreductase [ECs5282]
ECs5296	+2.88	7.79	+1.53	7.03	0.1	8.56	0.1	0.00022541	0.00217985	0.02897485	0.43990357	A_07_P061800	ECs5296	cmr|hypothetical protein [ECs5296]
ECs5301	+2.18	8.44	+1.13	7.88	0.1	9.01	0.2	0.00220949	0.01194579	0.05368902	0.56959569	A_07_P061804	ECs5301	cmr|hypothetical protein [ECs5301]
ECs5337	+2.51	8.49	+1.33	7.83	0.2	9.16	0.1	0.00111498	0.00705553	0.03952744	0.49390476	A_07_P061859	ECs5337	cmr|putative activating enzyme [ECs5337]
ECs5338	+5.78	10.49	+2.53	9.22	0.1	11.75	0.3	0.00004061	0.00063499	0.00755335	0.24723493	A_07_P061864	ECs5338	cmr|hypothetical protein [ECs5338]
ECs5344	+2.62	8.86	+1.39	8.17	0.2	9.56	0.3	0.00209082	0.01144571	0.03629764	0.48063078	A_07_P061868	ECs5344	cmr|hypothetical protein [ECs5344]
ECs5345	+5.92	8.97	+2.57	7.69	0.1	10.26	0.1	0.00000839	0.00022708	0.00713732	0.24165591	A_07_P061871	ECs5345	cmr|putative lipoate-protein ligase A [ECs5345]
ECs5350	+2.49	7.79	+1.31	7.13	0.0	8.45	0.0	0.00028273	0.00258051	0.03929688	0.49416134	A_07_P062875	ECs5350	cmr|soluble lytic murein transglycosylase [ECs5350]
edd	+2.15	8.27	+1.11	7.72	0.1	8.83	0.0	0.00091068	0.00609775	0.05337843	0.56898443	A_07_P015931	b1851	cmr|6-phosphogluconate dehydratase [b1851]
elaA	+2.99	8.18	+1.58	7.39	0.0	8.97	0.1	0.00011938	0.00135835	0.02699505	0.42843750	A_07_P032393	c_2811	cmr|Protein elaA [c_2811]
elbB	+4.56	10.05	+2.19	8.95	0.1	11.14	0.3	0.00013016	0.00145337	0.01186357	0.29553797	A_07_P012521	b3209	cmr|sigma cross-reacting protein 27A [b3209]
emrA	-2.32	10.92	-1.22	11.53	0.0	10.31	0.1	0.00071040	0.00498201	0.04716654	0.57520527	A_07_P001896	b2685	cmr|multidrug resistance secretion protein [b2685]
emrD	-6.31	9.91	-2.66	11.24	0.0	8.59	0.0	0.00000383	0.00014478	0.00140943	0.12466292	A_07_P017297	b3673	cmr|2-module integral membrane pump; multidrug resistance [b3673]
emrE	-2.44	8.75	-1.28	9.39	0.2	8.10	0.2	0.00201681	0.01112176	0.03979040	0.54145203	A_07_P052427	Z1870	cmr|methylviologen resistance protein encoded within prophage CP-933X [Z1870]
emrE	-2.38	8.21	-1.25	8.84	0.1	7.59	0.1	0.00065310	0.00469949	0.04299606	0.55806265	A_07_P016482	b0543	cmr|methylviologen resistance [b0543]
emtA	-3.07	10.33	-1.62	11.14	0.2	9.52	0.0	0.00021630	0.00211777	0.01813199	0.38839456	A_07_P006916	b1193	cmr|murein transglycosylase E [b1193]
endA	-2.49	10.10	-1.32	10.75	0.1	9.44	0.1	0.00052583	0.00402465	0.03763847	0.52720641	A_07_P011868	b2945	cmr|DNA-specific endonuclease I [b2945]
eno	+6.66	10.74	+2.74	9.37	0.1	12.11	0.1	0.00000547	0.00017880	0.00566502	0.21860294	A_07_P002342	b2779	cmr|enolase [b2779]
entB	-2.20	8.94	-1.13	9.51	0.0	8.38	0.2	0.00184040	0.01034832	0.05607533	0.61181566	A_07_P016731	b0595	cmr|2,3-dihydro-2,3-dihydroxybenzoate synthetase, isochroismatase [b0595]
epd	+3.73	10.75	+1.90	9.80	0.2	11.70	0.2	0.00019530	0.00196595	0.01812436	0.35403226	A_07_P011783	b2927	cmr|D-erythrose 4-phosphate dehydrogenase [b2927]
eptA	+2.06	8.37	+1.04	7.85	0.0	8.89	0.0	0.00102994	0.00666690	0.05887957	0.59346991	A_07_P011307	b4114	cmr|orf, hypothetical protein [b4114]
eptB	+3.62	8.31	+1.86	7.38	0.0	9.23	0.1	0.00005376	0.00077723	0.01918890	0.36354994	A_07_P007657	b3546	cmr|orf, hypothetical protein [b3546]
erpA	-14.23	11.06	-3.83	12.98	0.1	9.15	0.4	0.00000602	0.00018702	0.00005399	0.02236842	A_07_P002744	b0156	cmr|orf, hypothetical protein [b0156]
essD	+15.77	9.53	+3.98	7.55	0.2	11.52	0.2	0.00000229	0.00010966	0.00092670	0.09292994	A_07_P016531	b0554	cmr|orf, hypothetical protein [b0554]
essQ	+2.05	7.55	+1.03	7.03	0.2	8.06	0.2	0.00512082	0.02321400	0.06243775	0.60605425	A_07_P010455	b1556	cmr|orf, hypothetical protein [b1556]
ETG02_36680	-2.27	7.19	-1.18	7.78	0.3	6.60	0.1	0.00233000	0.01243793	0.04909235	0.58421013	ETG02_36680	ETG02_36680	
ETG10_234183	-2.28	11.39	-1.19	11.98	0.1	10.79	0.2	0.00104287	0.00672630	0.04835810	0.58162720	ETG10_234183	ETG10_234183	
ETG10_234183	-3.82	11.81	-1.93	12.77	0.2	10.84	0.2	0.00017053	0.00178746	0.00862043	0.30294643	ETG10_234183	ETG10_234183	
ETG10_234183	-5.74	12.04	-2.52	13.31	0.2	10.78	0.0	0.00001112	0.00027182	0.00193407	0.14500000	ETG10_234183	ETG10_234183	
ETG10_234183	-5.49	11.64	-2.46	12.86	0.2	10.41	0.3	0.00004999	0.00074046	0.00227325	0.15766520	ETG10_234183	ETG10_234183	
ETG10_234183	-2.38	11.59	-1.25	12.21	0.0	10.96	0.0	0.00037869	0.00319003	0.04328760	0.55816544	ETG10_234183	ETG10_234183	
ETG10_236652	+2.72	7.62	+1.44	6.90	0.1	8.34	0.0	0.00025025	0.00234812	0.03281186	0.46330942	ETG10_236652	ETG10_236652	
etpO	-2.17	9.14	-1.12	9.70	0.1	8.58	0.1	0.00121470	0.00751445	0.05786649	0.61849966	A_07_P048849	ABH-0244075	asap|type II secretion protein [L7044]
eutA	+2.53	7.41	+1.34	6.74	0.1	8.08	0.0	0.00028682	0.00260187	0.03788427	0.48809329	A_07_P013311	b2451	cmr|orf, hypothetical protein [b2451]
eutC	+2.45	7.39	+1.29	6.74	0.1	8.04	0.1	0.00058099	0.00433102	0.04073361	0.50024181	A_07_P042830	Z3705	cmr|ethanolamine ammonia-lyase, light chain [Z3705]
eutD	+2.49	8.07	+1.32	7.42	0.4	8.73	0.2	0.00401101	0.01905578	0.04246824	0.51039695	A_07_P013350	b2458	cmr|ethanolamine utilization; homolog of Salmonella acetyl/butyryl P transferase [b2458]
eutQ	+2.95	8.31	+1.56	7.53	0.1	9.09	0.1	0.00013476	0.00149621	0.02773437	0.43275520	A_07_P013359	b2460	cmr|orf, hypothetical protein [b2460]
evgS	-2.04	8.08	-1.03	8.59	0.2	7.56	0.1	0.00387116	0.01851944	0.06818471	0.65577276	A_07_P042758	Z3632	cmr|putative sensor for regulator EvgA [Z3632]
exoD	+5.40	12.92	+2.43	11.71	0.0	14.14	0.1	0.00001134	0.00027458	0.00872396	0.26013258	A_07_P016473	b0539	cmr|putative exonuclease [b0539]
exuR	-2.17	12.66	-1.12	13.22	0.1	12.10	0.1	0.00094313	0.00624679	0.05747269	0.61638283	A_07_P000199	b3094	cmr|negative regulator of exu regulon, exuT, uxaAC, and uxuB [b3094]
exuT	+11.27	9.00	+3.49	7.25	0.1	10.75	0.1	0.00000109	0.00007696	0.00197853	0.13033473	A_07_P000192	b3093	cmr|transport of hexuronates [b3093]
fabB	+10.05	12.40	+3.33	10.74	0.0	14.07	0.1	0.00000104	0.00007542	0.00250445	0.14712687	A_07_P013901	b2323	cmr|3-oxoacyl- [b2323]
fabD	+5.30	12.85	+2.41	11.65	0.1	14.06	0.1	0.00001228	0.00028963	0.00900216	0.26442164	A_07_P001661	b1092	cmr|malonyl-CoA- [b1092]
fabG	+2.16	7.09	+1.11	6.53	0.2	7.64	0.0	0.00142456	0.00843485	0.05381479	0.56863087	A_07_P046211	Z4865	cmr|putative beta-ketoacyl-ACP reductase [Z4865]
fabG	+2.23	7.35	+1.16	6.77	0.1	7.92	0.2	0.00157273	0.00913690	0.05073234	0.55816212	A_07_P059966	ECs4340	cmr|putative 3-oxoacyl- [ECs4340]
fabG	+6.54	12.43	+2.71	11.08	0.1	13.79	0.1	0.00000979	0.00024876	0.00588732	0.22227818	A_07_P001668	b1093	cmr|3-oxoacyl- [b1093]
fabH	+4.76	12.79	+2.25	11.66	0.1	13.91	0.1	0.00002591	0.00046513	0.01133829	0.29073290	A_07_P001656	b1091	cmr|3-oxoacyl- [b1091]
fabZ	-2.23	14.33	-1.16	14.90	0.0	13.75	0.1	0.00068793	0.00486779	0.05282965	0.59795111	A_07_P002859	b0180	cmr|(3R)-hydroxymyristol acyl carrier protein dehydratase [b0180]
fadB	-3.11	11.06	-1.64	11.88	0.8	10.24	0.1	0.01307821	0.04881538	0.01733549	0.38118715	A_07_P039288	c_4793	cmr|Fatty oxidation complex alpha subunit [c_4793]
fadB	-2.04	10.72	-1.03	11.24	0.0	10.21	0.1	0.00153021	0.00893653	0.07043890	0.66287507	A_07_P039288	c_4793	cmr|Fatty oxidation complex alpha subunit [c_4793]
fadI	-2.93	11.43	-1.55	12.21	0.2	10.66	0.3	0.00098344	0.00645412	0.02164317	0.42224287	A_07_P053270	ECs3225	cmr|putative acyltransferase [ECs3225]
fadI	-2.96	10.88	-1.57	11.67	0.1	10.10	0.1	0.00013867	0.00152367	0.02069042	0.41286439	A_07_P014000	b2342	cmr|putative acyltransferase [b2342]
fadI	-2.55	11.18	-1.35	11.86	0.4	10.51	0.1	0.00243955	0.01288005	0.03426766	0.51138389	A_07_P042685	Z3605	cmr|putative acyltransferase [Z3605]
fadJ	+2.07	11.06	+1.05	10.53	0.0	11.58	0.1	0.00114954	0.00720187	0.05819106	0.58954955	A_07_P013995	b2341	cmr|putative enzyme [b2341]
fadL	+5.19	8.12	+2.37	6.93	0.1	9.31	0.1	0.00001106	0.00027075	0.00940358	0.26918182	A_07_P032506	c_2889	cmr|Long-chain fatty acid transport protein precursor [c_2889]
fbaA	+11.24	11.41	+3.49	9.67	0.1	13.16	0.5	0.00002274	0.00042819	0.00190104	0.13013043	A_07_P011772	b2925	cmr|fructose-bisphosphate aldolase, class II [b2925]
fbp	-2.58	14.11	-1.37	14.80	0.0	13.43	0.0	0.00021685	0.00212192	0.03310849	0.50607767	A_07_P003714	b4232	cmr|fructose-bisphosphatase [b4232]
fdhE	+2.19	10.41	+1.13	9.84	0.2	10.97	0.0	0.00153032	0.00893653	0.05282838	0.56580272	A_07_P039385	c_4841	cmr|Protein fdhE [c_4841]
fdhE	+2.74	10.04	+1.46	9.32	0.2	10.77	0.2	0.00051472	0.00396851	0.03211700	0.45884755	A_07_P039385	c_4841	cmr|Protein fdhE [c_4841]
fdhE	+2.41	10.15	+1.27	9.52	0.1	10.79	0.2	0.00103027	0.00666690	0.04241870	0.51058104	A_07_P039385	c_4841	cmr|Protein fdhE [c_4841]
fdhE	+2.84	10.52	+1.50	9.77	0.0	11.27	0.1	0.00013670	0.00151031	0.02985391	0.44383381	A_07_P039385	c_4841	cmr|Protein fdhE [c_4841]
fdnG	+3.76	9.78	+1.91	8.82	0.2	10.73	0.2	0.00009641	0.00116488	0.01770389	0.35148802	A_07_P009461	b1474	cmr|formate dehydrogenase-N, nitrate-inducible, alpha subunit [b1474]
fdnH	+3.83	8.66	+1.94	7.69	0.1	9.63	0.1	0.00003994	0.00062694	0.01728214	0.34838668	A_07_P009468	b1475	cmr|formate dehydrogenase-N, nitrate-inducible, iron-sulfur beta subunit [b1475]
fdoG	+2.93	11.17	+1.55	10.39	0.0	11.94	0.2	0.00028070	0.00256758	0.02869220	0.43985394	A_07_P047068	Z5436	cmr|formate dehydrogenase-O, major subunit [Z5436]
fdoG	+3.99	8.79	+2.00	7.79	0.0	9.79	0.2	0.00007591	0.00098604	0.01594512	0.33924324	A_07_P019246	b3894	cmr|formate dehydrogenase-O, major subunit [b3894]
fdoH	+5.48	9.89	+2.46	8.66	0.2	11.11	0.2	0.00002933	0.00050641	0.00844131	0.25508637	A_07_P019241	b3893	cmr|formate dehydrogenase-O, iron-sulfur subunit [b3893]
fdoH	+2.70	9.98	+1.43	9.26	0.0	10.70	0.5	0.00466568	0.02159216	0.03495744	0.47568712	A_07_P039386	c_4843	cmr|Formate dehydrogenase-O beta subunit [c_4843]
fdoH	+4.01	11.37	+2.00	10.36	0.0	12.37	0.1	0.00003519	0.00057232	0.01598704	0.33921833	A_07_P047064	Z5435	cmr|formate dehydrogenase-O, iron-sulfur subunit [Z5435]
fdoI	+6.29	10.00	+2.65	8.67	0.1	11.33	0.1	0.00000569	0.00018088	0.00632495	0.22891954	A_07_P019239	b3892	cmr|formate dehydrogenase, cytochrome B556 [b3892]
fecA	-2.58	9.76	-1.37	10.44	0.1	9.07	0.1	0.00030626	0.00272199	0.03320948	0.50663760	A_07_P003984	b4291	cmr|outer membrane receptor; citrate-dependent iron transport, outer membrane receptor [b4291]
fecB	-3.47	9.66	-1.79	10.56	0.1	8.76	0.0	0.00005429	0.00078198	0.01173082	0.33519056	A_07_P003977	b4290	cmr|citrate-dependent iron transport, periplasmic protein [b4290]
fecI	-2.73	8.54	-1.45	9.26	0.1	7.81	0.2	0.00062875	0.00458505	0.02657393	0.46956229	A_07_P003994	b4293	cmr|probable RNA polymerase sigma factor [b4293]
fepA	-3.33	9.49	-1.74	10.36	0.1	8.62	0.6	0.00280741	0.01437393	0.01431720	0.35950558	A_07_P016680	b0584	cmr|outer membrane receptor for ferric enterobactin [b0584]
fepA	-3.56	8.48	-1.83	9.40	0.5	7.57	0.1	0.00112595	0.00710213	0.01079522	0.32559387	A_07_P031310	c_0669	cmr|Ferrienterobactin receptor precursor [c_0669]
fepE	-18.64	11.32	-4.22	13.43	0.0	9.21	0.1	0.00000031	0.00004374	0.00002223	0.01400000	A_07_P031316	c_0674	cmr|Ferric enterobactin transport protein fepE [c_0674]
ffh	-3.14	13.60	-1.65	14.43	0.0	12.78	0.2	0.00022242	0.00216028	0.01667111	0.37711207	A_07_P019926	b2610	cmr|GTP-binding export factor binds to signal sequence, GTP and RNA [b2610]
fhlA	+2.20	7.93	+1.14	7.36	0.2	8.50	0.1	0.00138268	0.00825206	0.05184070	0.56133425	A_07_P002101	b2731	cmr|formate hydrogen-lyase transcriptional activator for fdhF, hyc and hyp operons [b2731]
fhuD	-2.05	7.96	-1.03	8.48	0.0	7.44	0.1	0.00121891	0.00753458	0.06898501	0.65744552	A_07_P002724	b0152	cmr|hydroxamate-dependent iron uptake, cytoplasmic membrane component [b0152]
fimA	-3.14	9.17	-1.65	9.99	0.0	8.34	0.2	0.00026131	0.00242287	0.01705348	0.37709270	A_07_P004096	b4314	cmr|major type 1 subunit fimbrin [b4314]
fimC	-2.49	8.37	-1.31	9.03	0.1	7.71	0.2	0.00095475	0.00631051	0.03795160	0.52970745	A_07_P004109	b4316	cmr|periplasmic chaperone, required for type 1 fimbriae [b4316]
fimD	-16.16	9.25	-4.01	11.25	0.0	7.24	0.1	0.00000041	0.00004972	0.00003176	0.01851852	A_07_P004111	b4317	cmr|outer membrane protein; export and assembly of type 1 fimbriae, interrupted [b4317]
fimF	-15.06	9.36	-3.91	11.31	0.2	7.40	0.1	0.00000069	0.00006128	0.00003430	0.01741935	A_07_P004120	b4318	cmr|fimbrial morphology [b4318]
fimF	-7.27	8.38	-2.86	9.82	0.1	6.95	0.1	0.00000375	0.00014286	0.00084223	0.09082192	A_07_P041401	c_5397	cmr|FimF protein precursor [c_5397]
fimG	-11.31	10.68	-3.50	12.43	0.1	8.93	0.6	0.00007804	0.00100121	0.00013720	0.03661017	A_07_P004124	b4319	cmr|fimbrial morphology [b4319]
fimH	-3.65	7.83	-1.87	8.77	0.1	6.90	0.1	0.00005896	0.00082929	0.01008575	0.32472393	A_07_P004128	b4320	cmr|minor fimbrial subunit, D-mannose specific adhesin [b4320]
fimI	-2.39	9.01	-1.26	9.64	0.1	8.39	0.0	0.00054157	0.00411349	0.04278900	0.55859867	A_07_P004101	b4315	cmr|fimbrial protein [b4315]

fis	+2.89	10.18	+1.53	9.41	0.1	10.94	0.3	0.00048743	0.00383512	0.02931911	0.44299424	A_07_P012772	b3261	cmr|site-specific DNA inversion stimulation factor; DNA-binding protein; a trans activator for transcription [b3261]
fklB	+2.76	11.77	+1.46	11.04	0.1	12.51	0.1	0.00018150	0.00186421	0.03143420	0.45487132	A_07_P011753	b4207	cmr|FKBP-type 22KD peptidyl-prolyl cis-trans isomerase [b4207]
fkpB	+4.66	10.57	+2.22	9.46	0.0	11.68	0.1	0.00002055	0.00039896	0.01192073	0.29555906	A_07_P010829	b0028	cmr|probable FKBX-type 16KD peptidyl-prolyl cis-trans isomerase [b0028]
flgK	-2.70	9.28	-1.43	9.99	0.1	8.56	0.2	0.00045683	0.00365837	0.02883892	0.47995772	A_07_P001612	b1082	cmr|flagellar biosynthesis, hook-filament junction protein 1 [b1082]
flhD	+2.52	8.50	+1.34	7.83	0.0	9.17	0.3	0.00099486	0.00650516	0.03833651	0.48872065	A_07_P016140	b1892	cmr|regulator of flagellar biosynthesis, acting on class 2 operons; transcriptional initiation factor [b1892]
fliA	-4.51	7.85	-2.17	8.94	0.1	6.77	0.1	0.00002524	0.00045780	0.00444106	0.21850000	A_07_P014973	b1922	cmr|flagellar biosynthesis; alternative sigma factor 28; regulation of flagellar operons [b1922]
fliE	-3.89	7.73	-1.96	8.71	0.3	6.75	0.1	0.00019642	0.00196842	0.00782076	0.28436490	A_07_P015049	b1937	cmr|flagellar biosynthesis; basal-body component, possibly at [b1937]
fliI	-2.60	12.47	-1.38	13.16	0.3	11.78	0.1	0.00119865	0.00742973	0.03215130	0.50217262	A_07_P044691	Z3031	cmr|flagellum-specific ATP synthase [Z3031]
fliI	-3.16	12.40	-1.66	13.23	0.4	11.57	0.2	0.00129527	0.00787670	0.01654789	0.37757971	A_07_P055201	ECs2680	cmr|flagellum-specific ATP synthase [ECs2680]
fliI	-2.56	11.95	-1.36	12.63	0.4	11.27	0.0	0.00265915	0.01378518	0.03331682	0.50631274	A_07_P015066	b1941	cmr|flagellum-specific ATP synthase [b1941]
fliI	-2.71	11.81	-1.44	12.53	0.2	11.09	0.1	0.00033156	0.00286975	0.02772866	0.47349241	A_07_P035407	c_2358	cmr|Flagellum-specific ATP synthase [c_2358]
fliY	-8.70	12.59	-3.12	14.15	0.0	11.02	0.3	0.00000664	0.00019716	0.00041286	0.06435644	A_07_P041209	c_2335	cmr|Cystine-binding periplasmic protein precursor [c_2335]
fliY	-7.34	11.50	-2.88	12.93	0.1	10.06	0.0	0.00000262	0.00011934	0.00081936	0.08958333	A_07_P014964	b1920	cmr|putative periplasmic binding transport protein [b1920]
flk	-3.21	8.68	-1.68	9.52	0.2	7.84	0.3	0.00052930	0.00404719	0.01509654	0.36906832	A_07_P013891	b2321	cmr|cell division protein [b2321]
flu	+2.34	10.31	+1.23	9.70	0.5	10.92	0.1	0.01299494	0.04860830	0.05060340	0.55830413	A_07_P015330	b2000	cmr|outer membrane fluffing protein, similar to adhesin [b2000]
fmt	-2.42	12.05	-1.28	12.69	0.0	11.41	0.1	0.00052296	0.00401527	0.04018039	0.54347079	A_07_P012874	b3288	cmr|10-formyltetrahydrofolate:L-methionyl-tRNA [b3288]
fnr	-8.06	13.83	-3.01	15.33	0.0	12.32	0.1	0.00000271	0.00012064	0.00051448	0.07168142	A_07_P014647	b1334	cmr|transcriptional regulation of aerobic, anaerobic respiration, osmotic balance [b1334]
focA	+4.99	10.96	+2.32	9.80	0.1	12.12	0.1	0.00001449	0.00031589	0.01022167	0.27939236	A_07_P004969	b0904	cmr|probable formate transporter [b0904]
focA	+69.57	13.05	+6.12	9.99	0.0	16.11	0.1	0.00000003	0.00003344	0.00000635	0.00625000	A_07_P040486	c_1239	cmr|F1C major fimbrial subunit precursor [c_1239]
focB	-2.65	12.99	-1.40	13.69	0.1	12.28	0.2	0.00043046	0.00350857	0.03098514	0.49475659	A_07_P013519	b2492	cmr|probable formate transporter [b2492]
focC	+8.38	9.70	+3.07	8.16	0.1	11.23	0.1	0.00000380	0.00014395	0.00362614	0.17352584	A_07_P040500	c_1241	cmr|F1C periplasmic chaperone [c_1241]
focD	+5.73	10.78	+2.52	9.52	0.3	12.04	0.2	0.00005259	0.00076528	0.00761687	0.24725773	A_07_P040505	c_1242	cmr|F1C fimbrial usher [c_1242]
focF	+7.07	10.31	+2.82	8.90	0.2	11.72	0.1	0.00000578	0.00018187	0.00512068	0.20778351	A_07_P040507	c_1243	cmr|F1C minor fimbrial subunit F precursor [c_1243]
focG	+6.51	10.66	+2.70	9.31	0.8	12.01	0.1	0.00131105	0.00794857	0.00616425	0.22569767	A_07_P040512	c_1244	cmr|F1C minor fimbrial subunit protein G presursor [c_1244]
focH	+5.76	10.08	+2.53	8.82	0.1	11.34	0.2	0.00001555	0.00033216	0.00764990	0.24731006	A_07_P040520	c_1245	cmr|F1C Putative fimbrial adhesin precursor [c_1245]
folA	+2.10	9.10	+1.07	8.56	0.1	9.63	0.0	0.00104338	0.00672686	0.05700013	0.58463192	A_07_P010929	b0048	cmr|dihydrofolate reductase type I; trimethoprim resistance [b0048]
folA	+2.03	10.24	+1.02	9.73	0.1	10.75	0.1	0.00198825	0.01100668	0.06193534	0.60603480	A_07_P035679	c_0058	cmr|Dihydrofolate reductase [c_0058]
folA	+2.34	9.21	+1.23	8.60	0.0	9.82	0.2	0.00073522	0.00513543	0.04478659	0.52386330	A_07_P059409	ECs0051	cmr|dihydrofolate reductase type I; trimethoprim resistance [ECs0051]
folX	+2.15	9.41	+1.10	8.86	0.1	9.96	0.1	0.00104063	0.00671463	0.05450140	0.57128495	A_07_P013803	b2303	cmr|D-erythro-7,8-dihydroneopterin tri P epimerase [b2303]
fpr	+3.21	9.39	+1.68	8.54	0.1	10.23	0.1	0.00012566	0.00141516	0.02348577	0.40947951	A_07_P039438	c_4876	cmr|Ferredoxin--NADP reductase [c_4876]
fpr	+5.11	9.09	+2.35	7.91	0.1	10.26	0.1	0.00001826	0.00037077	0.00963542	0.27137746	A_07_P019396	b3924	cmr|ferredoxin-NADP reductase [b3924]
frdA	+2.40	14.78	+1.26	14.14	0.0	15.41	0.0	0.00036212	0.00307507	0.04233168	0.51109663	A_07_P011501	b4154	cmr|fumarate reductase, anaerobic, flavoprotein subunit [b4154]
frdB	+4.28	12.64	+2.10	11.59	0.1	13.69	0.0	0.00002162	0.00041206	0.01397548	0.31934688	A_07_P011497	b4153	cmr|fumarate reductase, anaerobic, iron-sulfur protein subunit [b4153]
frdC	+3.82	11.76	+1.93	10.79	0.2	12.73	0.0	0.00007141	0.00094797	0.01719258	0.34836551	A_07_P011491	b4152	cmr|fumarate reductase, anaerobic, membrane anchor polypeptide [b4152]
frdD	+3.90	10.04	+1.96	9.06	0.2	11.03	0.1	0.00004983	0.00073949	0.01655996	0.34441215	A_07_P011487	b4151	cmr|fumarate reductase, anaerobic, membrane anchor polypeptide [b4151]
frmR	+6.88	10.68	+2.78	9.29	0.1	12.07	0.1	0.00000518	0.00017426	0.00527630	0.20767500	A_07_P020911	b0357	cmr|putative alpha helix chain [b0357]
frr	-2.96	13.28	-1.57	14.07	0.0	12.50	0.3	0.00050689	0.00393517	0.02097815	0.41649433	A_07_P059508	ECs0174	cmr|ribosome releasing factor [ECs0174]
frr	-2.64	13.95	-1.40	14.64	0.0	13.25	0.0	0.00020026	0.00200181	0.03132368	0.49713710	A_07_P049243	Z0183	cmr|ribosome releasing factor [Z0183]
frr	-2.21	13.81	-1.14	14.38	0.0	13.24	0.1	0.00067045	0.00479144	0.05517785	0.60749650	A_07_P035911	c_0208	cmr|Ribosome recycling factor [c_0208]
frr	-2.84	13.30	-1.51	14.05	0.0	12.55	0.0	0.00012522	0.00141122	0.02394944	0.44675355	A_07_P002818	b0172	cmr|ribosome releasing factor [b0172]
fruA	-2.20	8.98	-1.14	9.55	0.0	8.41	0.1	0.00069539	0.00490746	0.05549225	0.60882927	A_07_P000634	b2167	cmr|PTS system, fructose-specific transport protein [b2167]
fruA	-2.11	9.18	-1.08	9.72	0.1	8.64	0.1	0.00124030	0.00763383	0.06383003	0.64626367	A_07_P042526	c_2702	cmr|PTS system, fructose-specific IIBC component [c_2702]
fruR	+3.41	10.15	+1.77	9.26	0.1	11.04	0.1	0.00008685	0.00108440	0.02159362	0.38765108	A_07_P011076	b0080	cmr|transcriptional repressor of fru operon and others [b0080]
fsaA	+2.15	7.85	+1.11	7.30	0.1	8.41	0.0	0.00096905	0.00638357	0.05338034	0.56861976	A_07_P004579	b0825	cmr|putative transaldolase [b0825]
ftnA	+18.92	10.17	+4.24	8.05	0.0	12.29	0.1	0.00000026	0.00004211	0.00057292	0.07158730	A_07_P016193	b1905	cmr|cytoplasmic ferritin [b1905]
ftnB	+5.95	10.40	+2.57	9.11	0.1	11.68	0.1	0.00000820	0.00022525	0.00718051	0.24155983	A_07_P016180	b1902	cmr|ferritin-like protein [b1902]
ftsE	-8.98	10.99	-3.17	12.57	0.0	9.40	0.1	0.00000162	0.00009465	0.00033155	0.05553191	A_07_P007255	b3463	cmr|ATP-binding component of a membrane-associated complex involved in cell division [b3463]
ftsL	-2.32	10.80	-1.22	11.41	0.2	10.19	0.3	0.00483942	0.02225231	0.04691502	0.57570538	A_07_P011094	b0083	cmr|cell division protein; ingrowth of wall at septum [b0083]
ftsN	-2.25	11.52	-1.17	12.10	0.1	10.93	0.1	0.00074840	0.00521135	0.05178925	0.59170537	A_07_P019442	b3933	cmr|essential cell division protein [b3933]
ftsQ	-2.31	11.46	-1.21	12.06	0.0	10.85	0.1	0.00052068	0.00400270	0.04720274	0.57520124	A_07_P002438	b0093	cmr|cell division protein; ingrowth of wall at septum [b0093]
ftsW	+2.23	8.79	+1.16	8.21	0.1	9.36	0.2	0.00173029	0.00985948	0.05038554	0.55824771	A_07_P011122	b0089	cmr|cell division; membrane protein involved in shape determination [b0089]
ftsY	-6.05	12.94	-2.60	14.24	0.1	11.64	0.3	0.00002835	0.00049426	0.00160696	0.12908163	A_07_P007256	b3464	cmr|cell division membrane protein [b3464]
fucA	+3.92	10.97	+1.97	9.99	0.0	11.96	0.0	0.00002501	0.00045582	0.01649898	0.34405298	A_07_P005999	b2800	cmr|L-fuculose-1-phosphate aldolase [b2800]
fucK	+4.23	9.54	+2.08	8.50	0.1	10.58	0.1	0.00003138	0.00053108	0.01427337	0.32241033	A_07_P006014	b2803	cmr|L-fuculokinase [b2803]
fucK	+5.42	11.94	+2.44	10.72	0.3	13.16	0.1	0.00005615	0.00079813	0.00866235	0.25927757	A_07_P043254	Z4120	cmr|L-fuculokinase [Z4120]
fucO	+5.90	10.05	+2.56	8.77	0.0	11.33	0.2	0.00000914	0.00023912	0.00722180	0.24243070	A_07_P005991	b2799	cmr|L-1,2-propanediol oxidoreductase [b2799]
fucP	-2.98	10.15	-1.57	10.93	0.3	9.36	0.3	0.00131879	0.00797966	0.02023692	0.40899872	A_07_P006001	b2801	cmr|fucose permease [b2801]
fucR	+5.40	8.47	+2.43	7.26	0.1	9.69	0.1	0.00000977	0.00024876	0.00872713	0.25973535	A_07_P006024	b2805	cmr|positive regulator of the fuc operon [b2805]
fucU	+4.58	11.40	+2.20	10.30	0.1	12.50	0.0	0.00001344	0.00030273	0.01222116	0.29923795	A_07_P006018	b2804	cmr|protein of fucose operon [b2804]
fumA	+22.52	10.85	+4.49	8.60	0.0	13.10	0.0	0.00000015	0.00003548	0.00035188	0.05485149	A_07_P020691	b1612	cmr|fumarase A = fumarate hydratase Class I; aerobic isozyme [b1612]
fumB	+3.58	10.48	+1.84	9.56	0.4	11.40	0.1	0.00046255	0.00369283	0.01954395	0.36762246	A_07_P011347	b4122	cmr|fumarase B= fumarate hydratase Class I; anaerobic isozyme [b4122]
fumC	+7.45	8.91	+2.90	7.46	0.0	10.36	0.1	0.00000351	0.00013758	0.00465003	0.19627346	A_07_P042312	c_2003	cmr|Fumarate hydratase class II [c_2003]
fumC	+12.73	9.15	+3.67	7.32	0.1	10.99	0.1	0.00000085	0.00006636	0.00154027	0.11771845	A_07_P020689	b1611	cmr|fumarase C= fumarate hydratase Class II; isozyme [b1611]
fur	-2.20	12.61	-1.14	13.18	0.0	12.04	0.1	0.00070670	0.00496708	0.05582190	0.61116829	A_07_P009932	b0683	cmr|negative regulator [b0683]
gabT	-2.93	10.66	-1.55	11.43	0.6	9.88	0.0	0.00655660	0.02821185	0.02083968	0.41426768	A_07_P041262	c_3210	cmr|4-aminobutyrate aminotransferase [c_3210]
galF	+3.18	10.30	+1.67	9.46	0.1	11.13	0.0	0.00011003	0.00128322	0.02390434	0.41131148	A_07_P015526	b2042	cmr|homolog of Salmonella UTP--glucose-1-P uridyltransferase, probably a UDP-gal transferase [b2042]
galK	+9.65	11.79	+3.27	10.15	0.1	13.42	0.0	0.00000119	0.00008082	0.00274454	0.15322695	A_07_P004240	b0757	cmr|galactokinase [b0757]
galM	+10.84	11.10	+3.44	9.38	0.1	12.82	0.1	0.00000119	0.00008082	0.00213034	0.13469880	A_07_P004231	b0756	cmr|galactose-1-epimerase [b0756]
galP	-2.21	7.40	-1.15	7.97	0.0	6.83	0.1	0.00068725	0.00486692	0.05408473	0.60476562	A_07_P011856	b2943	cmr|galactose-proton symport of transport system [b2943]
galS	+3.06	9.61	+1.61	8.80	0.2	10.42	0.1	0.00029841	0.00267394	0.02616870	0.42694301	A_07_P032245	c_2685	cmr|Mgl repressor and galactose ultrainduction factor [c_2685]
galT	+2.13	10.26	+1.09	9.72	0.0	10.81	0.4	0.00712342	0.03010768	0.05663872	0.58282353	A_07_P004241	b0758	cmr|galactose-1-phosphate uridylyltransferase [b0758]
gamW	+13.76	12.99	+3.78	11.10	0.2	14.88	0.1	0.00000157	0.00009392	0.00125445	0.10972222	A_07_P038414	c_1537	cmr|Putative host-nuclease inhibitor protein Gam of bacteriphage [c_1537]
gapA	+27.39	11.10	+4.78	8.71	0.1	13.49	0.1	0.00000014	0.00003548	0.00018293	0.03740260	A_07_P015590	b1779	cmr|glyceraldehyde-3-phosphate dehydrogenase A [b1779]
garP	-2.13	7.88	-1.09	8.43	0.1	7.34	0.1	0.00134805	0.00811924	0.06129573	0.63699010	A_07_P000357	b3127	cmr|putative transport protein [b3127]
gcd	-2.87	8.66	-1.52	9.42	0.2	7.90	0.0	0.00029158	0.00263253	0.02294271	0.43836165	A_07_P002585	b0124	cmr|glucose dehydrogenase [b0124]
gcvH	+177.19	11.61	+7.47	7.88	0.0	15.34	0.1	0.00000001	0.00003344	0.00000000	0.00000000	A_07_P041711	c_3484	cmr|Glycine cleavage system H protein [c_3484]
gcvH	+92.42	10.75	+6.53	7.49	0.2	14.02	0.1	0.00000005	0.00003344	0.00000318	0.00416667	A_07_P006489	b2904	cmr|in glycine cleavage complex, carrier of aminomethyl moiety via covalently bound lipoyl cofactor [b2904]
gcvH	+102.63	11.91	+6.68	8.57	0.3	15.25	0.1	0.00000008	0.00003427	0.00000064	0.00142857	A_07_P041275	c_3484	cmr|Glycine cleavage system H protein [c_3484]
gcvP	+8.12	9.37	+3.02	7.86	0.1	10.88	0.1	0.00000320	0.00013126	0.00375635	0.17813253	A_07_P032879	c_3483	cmr|Glycine dehydrogenase [c_3483]
gcvP	+8.51	9.19	+3.09	7.65	0.2	10.74	0.0	0.00000300	0.00012561	0.00346926	0.17122257	A_07_P052779	Z4240	cmr|glycine decarboxylase, P protein of glycine cleavage [Z4240]
gcvP	+12.72	8.87	+3.67	7.04	0.0	10.71	0.0	0.00000051	0.00005264	0.00153519	0.11848039	A_07_P006481	b2903	cmr|glycine decarboxylase, P protein of glycine cleavage system [b2903]
gcvR	+7.59	9.24	+2.92	7.78	0.1	10.70	0.1	0.00000409	0.00015128	0.00443280	0.19068306	A_07_P013453	b2479	cmr|transcriptional regulation of gcv operon [b2479]
gcvT	+66.83	10.98	+6.06	7.95	0.0	14.01	0.2	0.00000007	0.00003344	0.00000826	0.00722222	A_07_P006491	b2905	cmr|aminomethyltransferase [b2905]
gdhA	+2.75	8.81	+1.46	8.08	0.0	9.54	0.1	0.00020228	0.00201310	0.03188135	0.45923147	A_07_P052546	Z2793	cmr|NADP-specific glutamate dehydrogenase [Z2793]
gdhA	+2.13	9.19	+1.09	8.65	0.3	9.74	0.3	0.00689728	0.02934094	0.05866108	0.59278562	A_07_P018851	b1761	cmr|NADP-specific glutamate dehydrogenase [b1761]
gef	-3.75	12.69	-1.91	13.65	0.0	11.74	0.1	0.00003371	0.00055746	0.00879700	0.30240175	A_07_P048482	Z0016	cmr|Gef protein interferes with membrane function when [Z0016]
gef	-2.63	10.66	-1.40	11.36	0.1	9.96	0.2	0.00039892	0.00332485	0.03142848	0.49679719	A_07_P048478	Z0016	cmr|Gef protein interferes with membrane function when [Z0016]
gfcA	-3.41	11.94	-1.77	12.83	0.0	11.05	0.0	0.00004950	0.00073662	0.01248412	0.33713551	A_07_P005379	b0987	cmr|orf, hypothetical protein [b0987]
gfcD	-5.39	14.44	-2.43	15.66	0.0	13.23	0.5	0.00019074	0.00193615	0.00249682	0.16799145	A_07_P005364	b0984	cmr|orf, hypothetical protein [b0984]
ggt	-2.03	7.65	-1.02	8.16	0.0	7.14	0.0	0.00119368	0.00740477	0.07110772	0.66519311	A_07_P034524	c_4236	cmr|Gamma-glutamyltranspeptidase precursor [c_4236]
gidB	-2.21	10.46	-1.14	11.04	0.1	9.89	0.2	0.00167592	0.00960525	0.05510099	0.60835203	A_07_P005623	b3740	cmr|glucose-inhibited division; chromosome replication? [b3740]
glcB	+3.49	9.23	+1.80	8.33	0.1	10.14	0.1	0.00007734	0.00099640	0.02043255	0.37536756	A_07_P012016	b2976	cmr|malate synthase G [b2976]
glcB	+4.89	11.23	+2.29	10.09	0.1	12.38	0.2	0.00003145	0.00053123	0.01061166	0.28316949	A_07_P041726	c_3705	cmr|Malate synthase G [c_3705]
glcF	+8.59	9.13	+3.10	7.58	0.1	10.68	0.1	0.00000288	0.00012407	0.00338034	0.16895238	A_07_P012026	b2978	cmr|glycolate oxidase iron-sulfur subunit [b2978]
glcG	+35.14	9.58	+5.13	7.01	0.0	12.15	0.2	0.00000011	0.00003548	0.00009273	0.02654545	A_07_P012024	b2977	cmr|orf, hypothetical protein [b2977]
gldA	+9.40	12.35	+3.23	10.74	0.2	13.97	0.1	0.00000285	0.00012365	0.00281377	0.15435540	A_07_P019501	b3945	cmr|glycerol dehydrogenase, [b3945]
glgA	+6.94	10.82	+2.79	9.42	0.1	12.22	0.1	0.00000559	0.00017942	0.00530996	0.20796020	A_07_P018171	b3429	cmr|glycogen synthase [b3429]
glgB	+5.09	9.68	+2.35	8.50	0.1	10.85	0.1	0.00001176	0.00028105	0.00978341	0.27310284	A_07_P018189	b3432	cmr|1,4-alpha-glucan branching enzyme [b3432]
glgC	+9.85	10.23	+3.30	8.57	0.1	11.88	0.1	0.00000192	0.00010099	0.00262386	0.14859712	A_07_P018179	b3430	cmr|glucose-1-phosphate adenylyltransferase [b3430]
glgP	+3.02	8.25	+1.59	7.46	0.3	9.05	0.5	0.00353327	0.01724670	0.02716972	0.42861723	A_07_P018166	b3428	cmr|glycogen phosphorylase [b3428]
glgS	+7.24	10.22	+2.86	8.79	0.0	11.65	0.1	0.00000290	0.00012407	0.00480501	0.20173333	A_07_P012366	b3049	cmr|glycogen biosynthesis, rpoS dependent [b3049]
glgX	+7.05	11.28	+2.82	9.87	0.1	12.69	0.0	0.00000302	0.00012594	0.00503112	0.20844737	A_07_P018182	b3431	cmr|part of glycogen operon, a glycosyl hydrolase, debranching enzyme [b3431]
glmM	+3.70	11.27	+1.89	10.32	0.1	12.21	0.2	0.00006809	0.00091941	0.01843115	0.35517748	A_07_P000591	b3176	cmr|similar to phosphoglucomutases and phosphomannomutases [b3176]
glnA	-3.46	13.41	-1.79	14.30	0.0	12.51	0.1	0.00005039	0.00074420	0.01194296	0.33516934	A_07_P019129	b3870	cmr|glutamine synthetase [b3870]
glnB	+3.09	10.93	+1.63	10.12	0.1	11.74	0.2	0.00017203	0.00179690	0.02558562	0.42491561	A_07_P019668	b2553	cmr|regulatory protein P-II for glutamine synthetase [b2553]
glnD	+2.25	8.47	+1.17	7.89	0.1	9.06	0.1	0.00088722	0.00598473	0.04909680	0.55173448	A_07_P002791	b0167	cmr|protein PII; uridylyltransferase acts on regulator of glnA [b0167]
gloA	+3.80	9.43	+1.93	8.47	0.2	10.39	0.1	0.00008625	0.00108122	0.01728849	0.34762452	A_07_P020890	b1651	cmr|lactoylglutathione lyase [b1651]
glpB	-2.25	11.39	-1.17	11.97	0.0	10.80	0.1	0.00064682	0.00466489	0.05154345	0.59233577	A_07_P041234	c_2783	cmr|Anaerobic glycerol-3-phosphate dehydrogenase subunit B [c_2783]
glpB	-2.53	10.35	-1.34	11.02	0.1	9.68	0.0	0.00030110	0.00268893	0.03576156	0.51654128	A_07_P000998	b2242	cmr|sn-glycerol-3-phosphate dehydrogenase [b2242]
glpE	-2.04	9.84	-1.03	10.36	0.0	9.33	0.1	0.00168846	0.00967008	0.07019436	0.66176048	A_07_P018153	b3425	cmr|protein of glp regulon [b3425]
glpF	+13.78	9.94	+3.79	8.05	0.1	11.83	0.1	0.00000061	0.00005750	0.00125889	0.10950276	A_07_P019412	b3927	cmr|facilitated diffusion of glycerol [b3927]
glpK	+3.91	10.38	+1.97	9.40	0.1	11.36	0.1	0.00003456	0.00056633	0.01676766	0.34508497	A_07_P019409	b3926	cmr|glycerol kinase [b3926]
glpQ	+2.54	8.30	+1.35	7.63	0.2	8.97	0.1	0.00051156	0.00395777	0.03790396	0.48794767	A_07_P000982	b2239	cmr|glycerophosphodiester phosphodiesterase, periplasmic [b2239]
glpT	+10.35	9.32	+3.37	7.63	0.1	11.00	0.2	0.00000271	0.00012064	0.00237424	0.14321839	A_07_P063326	ECs3125	cmr|sn-glycerol-3-phosphate permease [ECs3125]
glpT	+6.77	9.25	+2.76	7.87	0.1	10.63	0.0	0.00000352	0.00013758	0.00546939	0.21261728	A_07_P052656	Z3498	cmr|sn-glycerol-3-phosphate permease [Z3498]
glpT	+5.57	9.63	+2.48	8.39	0.2	10.87	0.0	0.00001185	0.00028261	0.00806784	0.24954813	A_07_P000990	b2240	cmr|sn-glycerol-3-phosphate permease [b2240]
gltA	+34.23	13.59	+5.10	11.04	0.1	16.14	0.1	0.00000013	0.00003548	0.00009337	0.02625000	A_07_P056596	ECs0745	cmr|citrate synthase [ECs0745]
gltA	+20.30	11.36	+4.34	9.19	0.2	13.53	0.1	0.00000046	0.00005132	0.00047256	0.06148760	A_07_P010118	b0720	cmr|citrate synthase [b0720]
gltA	+31.28	14.21	+4.97	11.72	0.1	16.69	0.1	0.00000016	0.00003548	0.00011941	0.03133333	A_07_P046286	Z0873	cmr|citrate synthase [Z0873]
gltD	+2.09	8.64	+1.06	8.11	0.1	9.17	0.1	0.00115693	0.00722808	0.05776550	0.58788623	A_07_P012545	b3213	cmr|glutamate synthase, small subunit [b3213]
gltI	+2.46	11.54	+1.30	10.89	0.1	12.19	0.2	0.00091036	0.00609775	0.04069741	0.50018735	A_07_P009841	b0655	cmr|putative periplasmic binding transport protein [b0655]
gltK	+4.46	8.64	+2.16	7.56	0.1	9.72	0.1	0.00002514	0.00045661	0.01279789	0.30575114	A_07_P009831	b0653	cmr|glutamate/aspartate transport system permease [b0653]
gltL	+4.17	9.00	+2.06	7.97	0.1	10.03	0.2	0.00003717	0.00059471	0.01475356	0.32761636	A_07_P009830	b0652	cmr|ATP-binding protein of glutamate/aspartate transport system [b0652]
gltP	+3.01	8.61	+1.59	7.82	0.2	9.41	0.3	0.00120146	0.00744423	0.02784489	0.43362018	A_07_P009026	b4077	cmr|glutamate-aspartate symport protein [b4077]
glvG	-4.06	9.29	-2.02	10.30	0.4	8.28	0.0	0.00034375	0.00295415	0.00692835	0.26800983	A_07_P010606	ABE-0285077	asap|C-terminal fragment of predicted 6-phospho-beta-glucosidase (pseudogene) [b4556]
glyA	+8.77	11.80	+3.13	10.23	0.1	13.36	0.2	0.00000659	0.00019656	0.00331936	0.16803859	A_07_P019657	b2551	cmr|serine hydroxymethyltransferase [b2551]
gmhA	+3.44	9.29	+1.78	8.40	0.0	10.18	0.2	0.00013279	0.00147858	0.02054497	0.37655413	A_07_P053536	ECs0249	cmr|phosphoheptose isomerase [ECs0249]
gmhA	+3.87	9.37	+1.95	8.39	0.2	10.35	0.0	0.00007725	0.00099606	0.01674162	0.34500000	A_07_P043366	Z0280	cmr|phosphoheptose isomerase [Z0280]
gmhA	+5.75	11.58	+2.52	10.32	0.0	12.84	0.0	0.00000571	0.00018115	0.00755780	0.24686722	A_07_P030845	c_0372	cmr|Phosphoheptose isomerase [c_0372]
gmk	-13.38	12.61	-3.74	14.48	0.0	10.74	0.1	0.00000055	0.00005504	0.00005145	0.02250000	A_07_P017181	b3648	cmr|guanylate kinase [b3648]
gnd	+5.21	8.98	+2.38	7.79	0.4	10.17	0.2	0.00011152	0.00129577	0.00937818	0.26894353	A_07_P015470	b2029	cmr|gluconate-6-phosphate dehydrogenase, decarboxylating [b2029]
gnd	+4.80	10.48	+2.26	9.35	0.1	11.61	0.2	0.00002730	0.00048247	0.01116044	0.28804918	A_07_P042488	c_2556	cmr|6-phosphogluconate dehydrogenase, decarboxylating [c_2556]
gnsA	+2.31	10.99	+1.21	10.39	0.1	11.59	0.0	0.00063591	0.00461448	0.04581682	0.52922964	A_07_P010343	ABE-0285038	asap|predicted regulator of phosphatidylethanolamine synthesis [b4517]
gnsB	+18.04	10.15	+4.17	8.07	0.5	12.24	0.0	0.00000939	0.00024309	0.00060213	0.07236641	A_07_P020394	b1550	cmr|orf, hypothetical protein [b1550]
gntX	+2.68	11.69	+1.42	10.98	0.1	12.40	0.1	0.00040524	0.00336503	0.03401232	0.47014047	A_07_P018091	b3413	cmr|orf, hypothetical protein [b3413]
gntY	-3.26	11.02	-1.70	11.87	0.1	10.16	0.1	0.00007988	0.00101674	0.01490536	0.36782132	A_07_P018096	b3414	cmr|orf, hypothetical protein [b3414]
gntY	-3.39	11.24	-1.76	12.12	0.1	10.36	0.0	0.00007977	0.00101674	0.01289634	0.34355330	A_07_P018096	b3414	cmr|orf, hypothetical protein [b3414]
gntY	-3.17	11.14	-1.66	11.97	0.1	10.31	0.1	0.00011484	0.00132092	0.01634782	0.37683748	A_07_P018096	b3414	cmr|orf, hypothetical protein [b3414]
gntY	-3.78	11.06	-1.92	12.02	0.1	10.10	0.1	0.00004499	0.00068369	0.00848704	0.29959641	A_07_P018096	b3414	cmr|orf, hypothetical protein [b3414]
gntY	-3.36	11.05	-1.75	11.93	0.1	10.18	0.1	0.00008370	0.00105675	0.01320884	0.34602329	A_07_P018096	b3414	cmr|orf, hypothetical protein [b3414]
gor	-2.21	11.32	-1.14	11.89	0.5	10.75	0.0	0.01105222	0.04253391	0.05435595	0.60564756	A_07_P007436	b3500	cmr|glutathione oxidoreductase [b3500]
gph	+6.44	10.06	+2.69	8.72	0.1	11.40	0.2	0.00000778	0.00021851	0.00607787	0.22621749	A_07_P017954	b3385	cmr|phosphoglycolate phosphatase [b3385]
gpmA	+5.81	8.28	+2.54	7.01	0.2	9.54	0.1	0.00001879	0.00037681	0.00750635	0.24620833	A_07_P004227	b0755	cmr|phosphoglyceromutase 1 [b0755]
gpsA	+3.16	10.80	+1.66	9.97	0.1	11.63	0.1	0.00010788	0.00126841	0.02446202	0.41411828	A_07_P016982	b3608	cmr|glycerol-3-phosphate dehydrogenase [b3608]
greB	-2.85	10.84	-1.51	11.59	0.1	10.08	0.1	0.00017389	0.00180865	0.02332444	0.44296743	A_07_P018057	b3406	cmr|transcription elongation factor and transcript cleavage [b3406]
groL	+7.75	13.07	+2.95	11.59	0.0	14.54	0.2	0.00000836	0.00022676	0.00430894	0.18896936	A_07_P011446	b4143	cmr|GroEL, chaperone Hsp60, peptide-dependent ATPase, heat shock protein [b4143]
groS	+10.27	13.85	+3.36	12.17	0.1	15.53	0.2	0.00000380	0.00014395	0.00236662	0.14330769	A_07_P011443	b4142	cmr|GroES, 10 Kd chaperone binds to Hsp60 in pres. Mg-ATP, suppressing its ATPase activity [b4142]
grxB	+17.71	10.15	+4.15	8.08	0.2	12.23	0.1	0.00000082	0.00006559	0.00071837	0.07964789	A_07_P001521	b1064	cmr|glutaredoxin 2 [b1064]
gshA	-3.35	10.34	-1.74	11.21	0.0	9.47	0.1	0.00005868	0.00082629	0.01328887	0.34639073	A_07_P001912	b2688	cmr|gamma-glutamate-cysteine ligase [b2688]
gshB	+2.73	11.41	+1.45	10.68	0.1	12.13	0.1	0.00023932	0.00227257	0.03204395	0.46031022	A_07_P011876	b2947	cmr|glutathione synthetase [b2947]
gspD	-2.82	7.82	-1.50	8.57	0.0	7.08	0.1	0.00017886	0.00184898	0.02485010	0.45545984	A_07_P013055	b3325	cmr|putative general protein secretion protein [b3325]
gspE	-3.44	7.85	-1.78	8.74	0.7	6.96	0.1	0.00441529	0.02062895	0.01192073	0.33514286	A_07_P013056	b3326	cmr|putative general secretion pathway for protein export [b3326]
gspI	-2.25	7.83	-1.17	8.42	0.2	7.25	0.0	0.00119736	0.00742466	0.05133003	0.59204396	A_07_P013076	b3330	cmr|putative export protein for general secretion pathway [b3330]
gspL	-3.20	9.76	-1.68	10.60	0.1	8.92	0.1	0.00011352	0.00131450	0.01549797	0.37366003	A_07_P013091	b3333	cmr|putative general protein secretion protein [b3333]
gspM	-3.76	7.77	-1.91	8.72	0.1	6.81	0.1	0.00004779	0.00071390	0.00880208	0.30191721	A_07_P017699	b3334	cmr|putative general secretion [b3334]
gst	+7.52	10.12	+2.91	8.66	0.1	11.58	0.3	0.00002569	0.00046319	0.00455221	0.19318059	A_07_P020808	b1635	cmr|glutathionine S-transferase [b1635]
guaA	+3.01	10.85	+1.59	10.05	0.1	11.64	0.1	0.00016796	0.00176640	0.02679688	0.42831472	A_07_P013594	b2507	cmr|GMP synthetase [b2507]
guaB	+3.38	11.17	+1.76	10.29	0.0	12.05	0.1	0.00006958	0.00093316	0.02185531	0.39101136	A_07_P030153	c_3027	cmr|Inosine-5-monophosphate dehydrogenase [c_3027]
guaB	+3.78	11.19	+1.92	10.23	0.1	12.15	0.2	0.00008115	0.00103116	0.01743267	0.34918575	A_07_P042850	Z3772	cmr|IMP dehydrogenase [Z3772]
gyrA	-5.76	12.78	-2.53	14.04	0.0	11.52	0.4	0.00006573	0.00089669	0.00192391	0.14492823	A_07_P000942	b2231	cmr|DNA gyrase, subunit A, type II topoisomerase [b2231]
gyrB	-4.86	12.64	-2.28	13.78	0.0	11.50	0.1	0.00001385	0.00030876	0.00339621	0.19443636	A_07_P005429	b3699	cmr|DNA gyrase subunit B, type II topoisomerase, ATPase activity [b3699]
hchA	+3.17	10.52	+1.67	9.69	0.3	11.35	0.1	0.00032926	0.00285769	0.02464431	0.41586281	A_07_P015194	b1967	cmr|orf, hypothetical protein [b1967]
hda	-4.10	10.66	-2.04	11.68	0.0	9.64	0.2	0.00004217	0.00064958	0.00630081	0.25833333	A_07_P013536	b2496	cmr|putative DNA replication factor [b2496]
hdeA	+5.27	8.61	+2.40	7.41	0.1	9.81	0.1	0.00001427	0.00031323	0.00898882	0.26452336	A_07_P007483	b3510	cmr|orf, hypothetical protein [b3510]
hdeB	+2.02	7.30	+1.02	6.79	0.1	7.80	0.1	0.00132890	0.00802484	0.06207381	0.60625931	A_07_P007477	b3509	cmr|orf, hypothetical protein [b3509]
hdhA	+2.98	9.89	+1.57	9.11	0.1	10.68	0.0	0.00013756	0.00151663	0.02716209	0.42892678	A_07_P035106	c_2011	cmr|7-alpha-hydroxysteroid dehydrogenase [c_2011]
hemB	-2.25	10.24	-1.17	10.82	0.0	9.65	0.1	0.00065790	0.00472969	0.05152884	0.59260044	A_07_P020966	b0369	cmr|5-aminolevulinate dehydratase = porphobilinogen synthase [b0369]
hemC	+3.72	11.42	+1.89	10.47	0.1	12.37	0.5	0.00132605	0.00801741	0.01782139	0.35248744	A_07_P039097	c_4724	cmr|Porphobilinogen deaminase [c_4724]
hemC	+3.72	9.83	+1.90	8.88	0.1	10.78	0.2	0.00012928	0.00144554	0.01798590	0.35307980	A_07_P005892	b3805	cmr|porphobilinogen deaminase = hydroxymethylbilane synthase [b3805]
hemD	+2.06	10.80	+1.04	10.28	0.1	11.32	0.1	0.00164293	0.00946791	0.05972879	0.59668147	A_07_P005889	b3804	cmr|uroporphyrinogen III synthase [b3804]
hemE	+4.30	9.24	+2.10	8.19	0.1	10.29	0.1	0.00002648	0.00047222	0.01392149	0.31950437	A_07_P008648	b3997	cmr|uroporphyrinogen decarboxylase [b3997]
hemF	-4.84	10.81	-2.27	11.95	0.0	9.68	0.2	0.00002637	0.00047080	0.00360074	0.19752613	A_07_P013240	b2436	cmr|coproporphyrinogen III oxidase [b2436]
hepA	-6.33	10.37	-2.66	11.70	0.1	9.04	0.1	0.00000448	0.00016072	0.00138974	0.12502857	A_07_P010981	b0059	cmr|probable ATP-dependent RNA helicase [b0059]
hflB	-4.11	12.98	-2.04	13.99	0.0	11.96	0.1	0.00002298	0.00043128	0.00636052	0.25742931	A_07_P000601	b3178	cmr|degrades sigma32, integral membrane peptidase, cell division protein [b3178]
hflK	-2.36	12.09	-1.24	12.71	0.1	11.47	0.3	0.00206584	0.01134840	0.04482978	0.56599840	A_07_P011588	b4174	cmr|protease specific for phage lambda cII repressor [b4174]
hha	-3.89	10.17	-1.96	11.15	0.1	9.19	0.1	0.00003473	0.00056727	0.00770008	0.28127610	A_07_P021406	b0460	cmr|haemolysin expression modulating protein [b0460]
hinT	+2.05	11.17	+1.03	10.65	0.1	11.68	0.2	0.00272976	0.01403571	0.06124936	0.60307067	A_07_P001720	b1103	cmr|orf, hypothetical protein [b1103]
hisA	+4.34	8.88	+2.12	7.82	0.1	9.94	0.0	0.00002432	0.00044632	0.01364266	0.31679941	A_07_P015442	b2024	cmr|N- [b2024]
hisA	+4.95	8.83	+2.31	7.67	0.2	9.98	0.2	0.00003575	0.00057902	0.01038872	0.28054889	A_07_P051776	Z3186	cmr|N- [Z3186]
hisB	+8.11	8.99	+3.02	7.48	0.2	10.50	0.1	0.00000513	0.00017372	0.00390244	0.18017595	A_07_P015435	b2022	cmr|imidazoleglycerolphosphate dehydratase and histidinol-phosphate phosphatase [b2022]
hisC	+6.09	8.73	+2.61	7.43	0.1	10.03	0.1	0.00000855	0.00022936	0.00695249	0.23899563	A_07_P015429	b2021	cmr|histidinol-phosphate aminotransferase [b2021]
hisD	+7.57	9.29	+2.92	7.83	0.1	10.75	0.1	0.00000442	0.00015907	0.00423780	0.18794366	A_07_P015421	b2020	cmr|L-histidinal:NAD+ oxidoreductase; L-histidinol:NAD+ oxidoreductase [b2020]
hisF	+9.00	9.74	+3.17	8.16	0.6	11.33	0.1	0.00011483	0.00132092	0.00319677	0.16610561	A_07_P015446	b2025	cmr|imidazole glycerol phosphate synthase subunit in heterodimer with HisH = imidazole glycerol phsphate synthase holoenzyme [b2025]
hisG	+3.29	8.08	+1.72	7.22	0.0	8.94	0.1	0.00007789	0.00100024	0.02250953	0.39998871	A_07_P031978	c_2546	cmr|ATP phosphoribosyltransferase [c_2546]
hisG	+2.20	7.61	+1.14	7.04	0.2	8.18	0.1	0.00128610	0.00783911	0.05139164	0.55993772	A_07_P015420	b2019	cmr|ATP phosphoribosyltransferase [b2019]
hisH	+14.16	9.40	+3.82	7.49	0.1	11.31	0.1	0.00000073	0.00006202	0.00119538	0.10632768	A_07_P015440	b2023	cmr|glutamine amidotransferase subunit of heterodimer with HisF = imidazole glycerol phosphate synthase holoenzyme [b2023]
hisI	+2.41	7.58	+1.27	6.95	0.2	8.22	0.1	0.00068286	0.00484533	0.04202934	0.51018504	A_07_P044983	Z3188	cmr|phosphoribosyl-amp cyclohydrolase phosphoribosyl-ATP pyrophosphatase [Z3188]
hisI	+4.44	8.21	+2.15	7.13	0.2	9.28	0.0	0.00003126	0.00053038	0.01283981	0.30628788	A_07_P042478	c_2553	cmr|Histidine biosynthesis bifunctional protein hisIE [c_2553]
hisJ	+4.64	10.89	+2.21	9.78	0.0	11.99	0.1	0.00001390	0.00030902	0.01191883	0.29644550	A_07_P013834	b2309	cmr|histidine-binding periplasmic protein of high-affinity histidine transport system [b2309]
hisM	+2.97	9.12	+1.57	8.33	0.1	9.90	0.2	0.00035219	0.00300373	0.02797510	0.43435897	A_07_P013822	b2307	cmr|histidine transport, membrane protein M [b2307]
hisQ	+2.02	7.65	+1.02	7.14	0.2	8.16	0.0	0.00303159	0.01527901	0.06362424	0.61378676	A_07_P013826	b2308	cmr|histidine transport system permease protein [b2308]
hisQ	+2.61	8.54	+1.38	7.84	0.0	9.23	0.1	0.00023273	0.00223145	0.03565549	0.47816014	A_07_P032423	c_2850	cmr|Histidine transport system permease protein hisQ [c_2850]
hlyB	-2.34	8.37	-1.23	8.99	0.0	7.76	0.1	0.00062171	0.00454361	0.04568788	0.56907437	A_07_P033071	c_3573	cmr|Hemolysin B [c_3573]
hlyC	+5.00	9.40	+2.32	8.24	0.1	10.56	0.1	0.00001938	0.00038447	0.01014164	0.27768696	A_07_P033053	c_3569	cmr|Hemolysin C [c_3569]
hmp	+9.59	9.19	+3.26	7.56	0.2	10.82	0.0	0.00000197	0.00010186	0.00276677	0.15338028	A_07_P019661	b2552	cmr|dihydropteridine reductase, ferrisiderophore reductase activity [b2552]
hmpA	+12.15	9.52	+3.60	7.72	0.4	11.32	0.1	0.00000987	0.00024987	0.00158854	0.11909524	A_07_P041601	c_3075	cmr|Flavohemoprotein [c_3075]
hns	+2.51	10.48	+1.33	9.82	0.1	11.15	0.1	0.00060741	0.00446453	0.03869347	0.49128226	A_07_P007124	b1237	cmr|DNA-binding protein HLP-II [b1237]
hofM	+2.35	7.69	+1.23	7.07	0.1	8.30	0.0	0.00044776	0.00360591	0.04429243	0.52196108	A_07_P018002	b3395	cmr|orf, hypothetical protein [b3395]
hokA	-2.04	10.50	-1.03	11.02	0.0	9.99	0.1	0.00128445	0.00783511	0.06988567	0.65964029	A_07_P010587	ABE-0047285	asap|toxic polypeptide, small [b4455]
holC	+3.77	9.80	+1.92	8.84	0.2	10.76	0.6	0.00206247	0.01133388	0.01743140	0.34960510	A_07_P003845	b4259	cmr|DNA polymerase III, chi subunit [b4259]
holE	+5.03	8.98	+2.33	7.82	0.1	10.15	0.1	0.00001477	0.00031981	0.01001334	0.27657895	A_07_P015890	b1842	cmr|DNA polymerase III, theta subunit [b1842]
holE	+3.17	9.12	+1.66	8.28	0.3	9.95	0.0	0.00051121	0.00395777	0.02492823	0.41841151	A_07_P035500	c_2252	cmr|DNA polymerase III, theta subunit [c_2252]
hpf	+20.65	12.27	+4.37	10.09	0.0	14.45	0.0	0.00000017	0.00003582	0.00045859	0.06170940	A_07_P012495	b3203	cmr|probable sigma-54 modulation protein [b3203]
hpt	-3.56	12.56	-1.83	13.47	0.0	11.64	0.3	0.00023154	0.00222327	0.01059769	0.32335271	A_07_P002588	b0125	cmr|hypoxanthine phosphoribosyltransferase [b0125]
hrpB	-3.02	10.12	-1.60	10.92	0.0	9.32	0.2	0.00021607	0.00211683	0.01893610	0.39592297	A_07_P035880	c_0181	cmr|ATP-dependent helicase hrpB [c_0181]
hscB	-2.56	10.74	-1.35	11.41	0.1	10.06	0.2	0.00087466	0.00591949	0.03484883	0.51228758	A_07_P019538	b2527	cmr|orf, hypothetical protein [b2527]
hslJ	-2.52	9.20	-1.33	9.86	0.1	8.53	0.0	0.00036368	0.00308504	0.03565358	0.51592831	A_07_P014873	b1379	cmr|heat shock protein hslJ [b1379]
hslU	+3.30	8.54	+1.72	7.67	0.2	9.40	0.0	0.00010591	0.00125089	0.02240727	0.39862147	A_07_P019432	b3931	cmr|heat shock protein hslVU, ATPase subunit, homologous to chaperones [b3931]
hslV	+3.26	11.63	+1.70	10.78	0.1	12.48	0.4	0.00098982	0.00648512	0.02348196	0.41032186	A_07_P019439	b3932	cmr|heat shock protein hslVU, proteasome-related peptidase subunit [b3932]
hspQ	-4.90	13.80	-2.29	14.94	0.1	12.65	0.1	0.00001255	0.00029182	0.00334985	0.19389706	A_07_P005278	b0966	cmr|orf, hypothetical protein [b0966]
htpG	+5.23	9.17	+2.39	7.97	0.2	10.36	0.3	0.00010070	0.00120567	0.00912602	0.26706320	A_07_P021471	b0473	cmr|chaperone Hsp90, heat shock protein C 62.5 [b0473]
htrC	-2.26	7.80	-1.18	8.39	0.4	7.22	0.0	0.00769778	0.03198571	0.04912030	0.58366038	A_07_P047192	Z5563	cmr|heat shock protein C [Z5563]
htrG	-3.40	10.31	-1.77	11.20	0.1	9.43	0.1	0.00009909	0.00119443	0.01273565	0.34275214	A_07_P000008	b3055	cmr|orf, hypothetical protein [b3055]
hupA	+31.72	13.15	+4.99	10.65	0.0	15.64	0.1	0.00000014	0.00003548	0.00012449	0.03213115	A_07_P008662	b4000	cmr|DNA-binding protein HU-alpha [b4000]
hupB	+5.23	12.54	+2.39	11.35	0.1	13.74	0.1	0.00001078	0.00026552	0.00918953	0.26842301	A_07_P021314	b0440	cmr|DNA-binding protein HU-beta, NS1 [b0440]
hybA	+2.38	13.12	+1.25	12.50	0.1	13.75	0.2	0.00108661	0.00692964	0.04288554	0.51267274	A_07_P036723	c_3733	cmr|Hydrogenase-2 operon protein hybA precursor [c_3733]
hybA	+5.18	11.00	+2.37	9.81	0.1	12.18	0.2	0.00001805	0.00036761	0.00946646	0.26854054	A_07_P012111	b2996	cmr|hydrogenase-2 small subunit [b2996]
hybB	+13.60	11.84	+3.77	9.96	0.0	13.73	0.0	0.00000046	0.00005132	0.00134909	0.11358289	A_07_P012108	b2995	cmr|probable cytochrome Ni/Fe component of hydrogenase-2 [b2995]
hybC	+4.98	10.38	+2.32	9.22	0.1	11.54	0.2	0.00001847	0.00037316	0.01012767	0.27778746	A_07_P012105	b2994	cmr|probable large subunit, hydrogenase-2 [b2994]
hybD	+2.99	10.97	+1.58	10.18	0.2	11.76	0.5	0.00276359	0.01418185	0.02844258	0.43858962	A_07_P012100	b2993	cmr|probable processing element for hydrogenase-2 [b2993]
hybD	+3.22	12.02	+1.69	11.18	0.2	12.87	0.1	0.00023358	0.00223690	0.02359502	0.40911894	A_07_P045813	Z4347	cmr|probable processing element for hydrogenase-2 [Z4347]
hybE	+2.98	9.28	+1.58	8.49	0.0	10.06	0.2	0.00026633	0.00245782	0.02733613	0.42995005	A_07_P012094	b2992	cmr|member of hyb operon [b2992]
hybF	+2.38	10.79	+1.25	10.16	0.2	11.41	0.0	0.00073068	0.00510603	0.04326156	0.51443353	A_07_P012088	b2991	cmr|may modulate levels of hydrogenase-2 [b2991]
hybG	+2.16	9.99	+1.11	9.44	0.1	10.55	0.1	0.00131045	0.00794857	0.05370871	0.56942088	A_07_P012083	b2990	cmr|hydrogenase-2 operon protein: may effect maturation of large subunit of hydrogenase-2 [b2990]
hybO	+2.16	13.12	+1.11	12.56	0.0	13.67	0.2	0.00145842	0.00858370	0.05437500	0.57072000	A_07_P012116	b2997	cmr|putative hydrogenase subunit [b2997]
hycB	-2.03	7.24	-1.02	7.75	0.2	6.73	0.0	0.00227522	0.01220896	0.07109057	0.66542806	A_07_P002069	b2724	cmr|probable small subunit of hydrogenase-3, iron-sulfur protein [b2724]
hydN	-4.28	8.02	-2.10	9.07	0.0	6.97	0.3	0.00009585	0.00116076	0.00574123	0.24900826	A_07_P043108	Z4021	cmr|involved in electron transport from formate to [Z4021]
hydN	-5.26	8.39	-2.40	9.58	0.0	7.19	0.0	0.00000754	0.00021396	0.00265689	0.17073469	A_07_P002014	b2713	cmr|involved in electron transport from formate to hydrogen, Fe-S centers [b2713]
hydN	-4.85	8.24	-2.28	9.38	0.1	7.10	0.0	0.00001233	0.00028963	0.00342162	0.19518116	A_07_P030619	c_3269	cmr|Electron transport protein hydN [c_3269]
hypB	+2.42	12.83	+1.28	12.20	0.2	13.47	0.2	0.00163243	0.00942465	0.04234121	0.51081992	A_07_P041672	c_3287	cmr|Hydrogenase isoenzymes nickel incorporation protein hypB [c_3287]
hypB	+3.37	10.12	+1.75	9.24	0.1	11.00	0.1	0.00007882	0.00100804	0.02202490	0.39359818	A_07_P002083	b2727	cmr|guanine-nucleotide binding protein, functions as nickel donor for large subunit of hydrogenase 3 [b2727]
hypC	+3.18	12.59	+1.67	11.76	0.2	13.42	0.2	0.00024582	0.00231743	0.02450838	0.41401288	A_07_P002090	b2728	cmr|pleiotrophic effects on 3 hydrogenase isozymes [b2728]
hypD	+2.44	9.13	+1.29	8.49	0.1	9.77	0.3	0.00206162	0.01133314	0.04175114	0.50798300	A_07_P002091	b2729	cmr|pleiotrophic effects on 3 hydrogenase isozymes [b2729]
hypD	+2.68	9.85	+1.42	9.14	0.1	10.56	0.2	0.00049223	0.00385941	0.03388084	0.47038801	A_07_P043118	Z4038	cmr|pleiotrophic effects on 3 hydrogenase isozymes [Z4038]
hypE	+2.05	12.05	+1.04	11.53	0.0	12.57	0.2	0.00208245	0.01141181	0.06178925	0.60611215	A_07_P002097	b2730	cmr|plays structural role in maturation of all 3 hydrogenases [b2730]
hypE	+2.35	11.20	+1.23	10.58	0.0	11.82	0.2	0.00099050	0.00648688	0.04458778	0.52270290	A_07_P043125	Z4039	cmr|plays structural role in maturation of all [Z4039]
iaaA	+2.03	9.93	+1.02	9.42	0.1	10.44	0.1	0.00199260	0.01101912	0.06236916	0.60613580	A_07_P004591	b0828	cmr|putative asparaginase [b0828]
iap	-3.19	7.70	-1.67	8.54	0.1	6.86	0.1	0.00012876	0.00144178	0.01609311	0.37591988	A_07_P041682	c_3320	cmr|Alkaline phosphatase isozyme conversion protein precursor [c_3320]
iap	-2.14	7.55	-1.10	8.10	0.2	7.00	0.1	0.00136516	0.00817540	0.05997586	0.62825017	A_07_P052740	Z4061	cmr|alkaline phosphatase isozyme conversion, aminopeptidase [Z4061]
icd	+5.65	10.47	+2.50	9.22	0.1	11.72	0.2	0.00001748	0.00036012	0.00793064	0.24922156	A_07_P006631	b1136	cmr|isocitrate dehydrogenase, specific for NADP+ [b1136]
icdA	+11.09	8.97	+3.47	7.24	0.0	10.71	0.1	0.00000089	0.00006827	0.00205602	0.13158537	A_07_P050489	Z1865	cmr|isocitrate dehydrogenase, specific for NADP+ [Z1865]
icdA	+29.28	13.95	+4.87	11.51	0.0	16.39	0.2	0.00000017	0.00003548	0.00015117	0.03449275	A_07_P038343	c_1517	cmr|Isocitrate dehydrogenase [c_1517]
iclR	+9.19	8.61	+3.20	7.01	0.1	10.21	0.1	0.00000184	0.00009954	0.00292873	0.15683673	A_07_P008735	b4018	cmr|repressor of aceBA operon [b4018]
iclR	+14.30	9.04	+3.84	7.12	0.1	10.96	0.1	0.00000046	0.00005132	0.00115536	0.10454023	A_07_P041378	c_4975	cmr|Acetate operon repressor [c_4975]
idnO	-3.56	11.56	-1.83	12.47	0.3	10.64	0.3	0.00077994	0.00538566	0.01127922	0.33192523	A_07_P003879	b4266	cmr|5-keto-D-gluconate 5-reductase [b4266]
ihfA	+3.98	12.45	+1.99	11.46	0.0	13.45	0.1	0.00002747	0.00048490	0.01615790	0.34238223	A_07_P018614	b1712	cmr|integration host factor [b1712]
ihfB	+5.70	10.03	+2.51	8.78	0.0	11.29	0.1	0.00000611	0.00018856	0.00771087	0.24674797	A_07_P005009	b0912	cmr|integration host factor [b0912]
ilvB	+2.53	10.68	+1.34	10.01	0.2	11.35	0.2	0.00082740	0.00565880	0.03846100	0.48951496	A_07_P017291	b3671	cmr|acetolactate synthase I,valine-sensitive, large subunit [b3671]
ilvC	+2.10	7.08	+1.07	6.54	0.0	7.61	0.0	0.00088370	0.00596613	0.05583016	0.57942650	A_07_P052249	Z5285	cmr|ketol-acid reductoisomerase [Z5285]
ilvD	+4.84	9.18	+2.28	8.04	0.0	10.32	0.0	0.00001023	0.00025442	0.01084159	0.28448333	A_07_P039071	c_4693	cmr|Dihydroxy-acid dehydratase [c_4693]
ilvD	+2.01	7.49	+1.00	6.99	0.0	8.00	0.0	0.00137639	0.00822395	0.06222624	0.60586889	A_07_P005736	b3771	cmr|dihydroxyacid dehydratase [b3771]
ilvM	+3.90	8.35	+1.96	7.37	0.0	9.33	0.1	0.00002846	0.00049572	0.01674162	0.34590551	A_07_P005727	b3769	cmr|acetolactate synthase II, valine insensitive, small subunit [b3769]
ilvN	+3.14	8.93	+1.65	8.11	0.0	9.76	0.0	0.00007578	0.00098524	0.02445567	0.41445640	A_07_P038986	c_4595	cmr|Acetolactate synthase isozyme I small subunit [c_4595]
imp	+2.18	10.48	+1.12	9.91	0.1	11.04	0.1	0.00130934	0.00794687	0.05296875	0.56692046	A_07_P010957	b0054	cmr|organic solvent tolerance [b0054]
inaA	-2.33	7.94	-1.22	8.55	0.0	7.33	0.0	0.00046229	0.00369269	0.04623666	0.57273800	A_07_P000971	b2237	cmr|pH-inducible protein involved in stress response [b2237]
infB	-2.79	12.92	-1.48	13.66	0.0	12.18	0.1	0.00017903	0.00184949	0.02571646	0.46219178	A_07_P000561	b3168	cmr|protein chain initiation factor IF-2 [b3168]
infC	-2.58	14.94	-1.37	15.63	0.0	14.26	0.1	0.00025063	0.00235010	0.03280932	0.50543053	A_07_P018636	b1718	cmr|protein chain initiation factor IF-3 [b1718]
insA	-3.13	9.15	-1.65	9.97	0.1	8.33	0.1	0.00010699	0.00126182	0.01708397	0.37670868	A_07_P008028	b0265	cmr|IS1 protein InsA [b0265]
insA	-3.62	10.24	-1.86	11.17	0.0	9.31	0.2	0.00010943	0.00127906	0.00985201	0.31981443	A_07_P003998	b4294	cmr|IS1 protein InsA [b4294]
insB	-3.03	9.39	-1.60	10.19	0.3	8.59	0.3	0.00135300	0.00813347	0.01823107	0.38840325	A_07_P005381	b0988	cmr|IS1 protein InsB [b0988]
insB	-3.32	9.58	-1.73	10.45	0.0	8.72	0.1	0.00007068	0.00094225	0.01385988	0.35366288	A_07_P010675	b0021	cmr|IS1 protein InsB [b0021]
insC	-2.07	9.61	-1.05	10.14	0.1	9.09	0.1	0.00128133	0.00781908	0.06655361	0.65203485	A_07_P015315	b1997	cmr|IS2 hypothetical protein [b1997]
insD	-4.32	11.96	-2.11	13.01	0.2	10.90	0.2	0.00007809	0.00100121	0.00528074	0.23754286	A_07_P020927	b0361	cmr|IS2 hypothetical protein [b0361]
insG	-26.55	10.57	-4.73	12.94	0.0	8.21	0.1	0.00000016	0.00003548	0.00000508	0.00571429	A_07_P003924	b4278	cmr|IS4 hypothetical protein [b4278]
iraP	+2.02	10.82	+1.02	10.31	0.0	11.33	0.1	0.00137052	0.00819500	0.06193915	0.60569565	A_07_P021023	b0382	cmr|orf, hypothetical protein [b0382]
iroN	-5.29	8.89	-2.40	10.09	0.1	7.69	0.3	0.00005765	0.00081407	0.00262195	0.17271967	A_07_P040541	c_1250	cmr|Siderophore receptor IroN [c_1250]

iscA	-4.38	11.47	-2.13	12.53	0.1	10.40	0.2	0.00004036	0.00063161	0.00492696	0.22814706	A_07_P019544	b2528	cmr|putative regulator [b2528]
iscR	-3.98	10.55	-1.99	11.55	0.1	9.56	0.1	0.00003099	0.00052867	0.00711319	0.26920673	A_07_P019560	b2531	cmr|orf, hypothetical protein [b2531]
iscS	-2.32	12.13	-1.21	12.74	0.1	11.52	0.1	0.00051379	0.00396851	0.04698552	0.57522551	A_07_P019553	b2530	cmr|putative aminotransferase [b2530]
iscX	-2.94	12.26	-1.56	13.04	0.1	11.48	0.4	0.00116660	0.00727690	0.02163872	0.42267990	A_07_P013676	b2524	cmr|orf, hypothetical protein [b2524]
iutA	-2.06	8.41	-1.04	8.93	0.0	7.89	0.1	0.00147745	0.00868272	0.06863122	0.65765673	A_07_P036302	c_3623	cmr|IutA protein [c_3623]
katG	+7.65	10.78	+2.94	9.31	0.1	12.24	0.0	0.00000229	0.00010966	0.00431339	0.18863889	A_07_P019488	b3942	cmr|catalase; hydroperoxidase HPI [b3942]
kbaZ	+4.81	8.14	+2.27	7.01	0.1	9.28	0.1	0.00002298	0.00043128	0.01097752	0.28614238	A_07_P000381	b3132	cmr|putative tagatose 6-phosphate kinase 2 [b3132]
kbl	+24.09	11.37	+4.59	9.07	0.1	13.66	0.4	0.00000352	0.00013758	0.00026105	0.04516484	A_07_P017030	b3617	cmr|2-amino-3-ketobutyrate CoA ligase [b3617]
kdpA	-3.09	8.58	-1.63	9.40	0.1	7.77	0.1	0.00010038	0.00120402	0.01785315	0.38662999	A_07_P010006	b0698	cmr|ATPase of high-affinity potassium transport system, A chain [b0698]
kdsA	-2.28	11.92	-1.19	12.51	0.0	11.32	0.0	0.00050169	0.00391007	0.04945567	0.58499624	A_07_P007028	b1215	cmr|2-dehydro-3-deoxyphosphooctulonate aldolase [b1215]
kdsC	-2.44	12.93	-1.29	13.57	0.0	12.29	0.1	0.00034652	0.00296986	0.03979738	0.54061260	A_07_P012468	b3198	cmr|orf, hypothetical protein [b3198]
kefA	-2.71	8.68	-1.44	9.40	0.0	7.95	0.1	0.00017255	0.00179905	0.02807546	0.47580194	A_07_P021433	b0465	cmr|putative alpha helix protein [b0465]
kefB	-2.27	8.58	-1.18	9.17	0.0	7.99	0.0	0.00051064	0.00395648	0.05053608	0.58718819	A_07_P017779	b3350	cmr|K+ efflux; NEM-activable K+/H+ antiporter [b3350]
kefG	-2.38	11.25	-1.25	11.87	0.1	10.62	0.1	0.00047497	0.00377105	0.04323425	0.55930978	A_07_P017784	b3351	cmr|putative NAD [b3351]
kpsC	+2.35	7.54	+1.23	6.92	0.0	8.15	0.0	0.00039951	0.00332795	0.04438707	0.52190441	A_07_P036625	c_3690	cmr|KpsC protein [c_3690]
kpsE	+3.76	9.41	+1.91	8.46	0.1	10.37	0.1	0.00006641	0.00090442	0.01792556	0.35277500	A_07_P036606	c_3687	cmr|KpsE protein [c_3687]
kpsT	+3.61	10.15	+1.85	9.23	0.1	11.08	0.1	0.00004092	0.00063841	0.01904853	0.36263603	A_07_P036658	c_3697	cmr|KpsT protein [c_3697]
ksgA	-4.25	11.44	-2.09	12.48	0.0	10.39	0.5	0.00075986	0.00527345	0.00624682	0.25881579	A_07_P010944	b0051	cmr|S-adenosylmethionine-6-N,N-adenosyl [b0051]
L7002	-2.68	12.74	-1.42	13.45	0.4	12.03	0.2	0.00324432	0.01606689	0.02816502	0.47578326	A_07_P048661	ABH-0243999	asap|unknown [L7002]
L7009	-2.26	7.67	-1.18	8.26	0.5	7.08	0.2	0.01087705	0.04204475	0.05093369	0.58963235	A_07_P048693	ABH-0244019	asap|replication protein [L7009]
L7055	-3.77	10.77	-1.91	11.73	0.1	9.81	0.0	0.00003333	0.00055352	0.00879256	0.30357456	A_07_P048893	ABH-0244096	asap|replication protein [L7055]
L7073	-2.06	10.26	-1.04	10.78	0.1	9.74	0.2	0.00217767	0.01181128	0.06767213	0.65323728	A_07_P048969	ABH-0244142	asap|unknown [L7073]
L7079	-3.18	11.31	-1.67	12.15	0.0	10.48	0.0	0.00006844	0.00092248	0.01601753	0.37638806	A_07_P048996	ABH-0244148	asap|unknown [L7079]
L7082	-4.98	9.30	-2.32	10.46	0.3	8.15	0.1	0.00007552	0.00098348	0.00333587	0.19380074	A_07_P049011	ABH-0244151	asap|unknown [L7082]
lacI	+3.48	8.55	+1.80	7.65	0.1	9.45	0.1	0.00007462	0.00097352	0.02079649	0.38027875	A_07_P008419	b0345	cmr|transcriptional repressor of the lac operon [b0345]
lacZ	+4.50	8.14	+2.17	7.05	0.2	9.22	0.2	0.00005746	0.00081349	0.01270008	0.30433790	A_07_P031027	c_0459	cmr|Beta-galactosidase [c_0459]
lacZ	+2.47	7.48	+1.30	6.83	0.3	8.13	0.2	0.00299193	0.01513170	0.04237424	0.51082695	A_07_P008412	b0344	cmr|beta-D-galactosidase [b0344]
lamB	+34.70	10.38	+5.12	7.82	0.1	12.94	0.3	0.00000036	0.00004777	0.00007876	0.02384615	A_07_P008822	b4036	cmr|phage lambda receptor protein; maltose high-affinity receptor [b4036]
lamB	+40.48	10.00	+5.34	7.33	0.0	12.67	0.2	0.00000010	0.00003427	0.00005018	0.02025641	A_07_P037538	c_5006	cmr|Maltoporin precursor [c_5006]
ldhA	+2.41	8.97	+1.27	8.34	0.2	9.61	0.1	0.00095931	0.00633003	0.04250826	0.51009909	A_07_P014878	b1380	cmr|fermentative D-lactate dehydrogenase, NAD-dependent [b1380]
leuE	-10.83	8.86	-3.44	10.57	0.1	7.14	0.1	0.00000209	0.00010461	0.00012767	0.03792453	A_07_P015685	b1798	cmr|orf, hypothetical protein [b1798]
lhr	-2.27	9.42	-1.18	10.01	0.3	8.82	0.1	0.00246112	0.01296781	0.04964494	0.58591454	A_07_P018330	b1653	cmr|member of ATP-dependent helicase superfamily II [b1653]
ligB	-2.45	8.18	-1.30	8.83	0.0	7.53	0.2	0.00106605	0.00683382	0.03790015	0.52992895	A_07_P017180	b3647	cmr|putative enzyme [b3647]
ligT	-2.79	10.46	-1.48	11.20	0.1	9.72	0.0	0.00017125	0.00179152	0.02523120	0.45870670	A_07_P002698	b0147	cmr|orf, hypothetical protein [b0147]
lipA	+2.49	12.92	+1.31	12.26	0.0	13.57	0.1	0.00038188	0.00321037	0.03931212	0.49395850	A_07_P009709	b0628	cmr|lipoate synthesis, sulfur insertion? [b0628]
lipB	-8.20	12.41	-3.04	13.93	0.0	10.89	0.0	0.00000168	0.00009634	0.00050114	0.07044643	A_07_P009718	b0630	cmr|protein of lipoate biosynthesis [b0630]
livK	-4.12	8.15	-2.04	9.17	0.1	7.13	0.2	0.00004413	0.00067579	0.00630399	0.25779221	A_07_P034548	c_4248	cmr|Leucine-specific binding protein precursor [c_4248]
lldD	+4.52	8.64	+2.18	7.56	0.5	9.73	0.1	0.00035614	0.00303253	0.01232787	0.29952160	A_07_P016968	b3605	cmr|L-lactate dehydrogenase [b3605]
lldP	+3.24	7.37	+1.70	6.52	0.1	8.22	0.0	0.00009416	0.00114522	0.02359375	0.40954796	A_07_P016959	b3603	cmr|L-lactate permease [b3603]
lldR	+5.03	8.51	+2.33	7.35	0.1	9.68	0.1	0.00001905	0.00038105	0.01002731	0.27647986	A_07_P016965	b3604	cmr|transcriptional regulator [b3604]
lolA	+2.82	9.55	+1.50	8.80	0.0	10.30	0.0	0.00013537	0.00150088	0.03015053	0.44488285	A_07_P004904	b0891	cmr|periplasmic protein effects translocation of lipoproteins from inner membrane to outer [b0891]
lolC	-2.24	9.95	-1.16	10.54	0.2	9.37	0.0	0.00099714	0.00651142	0.05222879	0.59371119	A_07_P040818	c_1391	cmr|Lipoprotein releasing system transmembrane protein lolC [c_1391]
lon	+2.23	11.00	+1.16	10.42	0.1	11.58	0.2	0.00186587	0.01045789	0.05064278	0.55834734	A_07_P043874	Z0545	cmr|DNA-binding, ATP-dependent protease La heat shock K-protein [Z0545]
lpcA	+3.91	10.52	+1.97	9.54	0.0	11.51	0.4	0.00045155	0.00362894	0.01588288	0.33929444	A_07_P007824	b0222	cmr|phosphoheptose isomerase [b0222]
lpxA	-2.74	11.63	-1.46	12.36	0.0	10.91	0.1	0.00018207	0.00186867	0.02692962	0.47213808	A_07_P002865	b0181	cmr|UDP-N-acetylglucosamine acetyltransferase; lipid A biosynthesis [b0181]
lpxB	-2.21	10.65	-1.15	11.22	0.1	10.08	0.1	0.00087789	0.00592945	0.05438262	0.60551627	A_07_P002870	b0182	cmr|tetraacyldisaccharide-1-P; lipid A biosynthesis, penultimate step [b0182]
lpxC	-7.54	14.30	-2.91	15.76	0.0	12.84	0.2	0.00000763	0.00021562	0.00071011	0.08281481	A_07_P002451	b0096	cmr|UDP-3-O-acyl N-acetylglucosamine deacetylase; lipid A biosynthesis [b0096]
lpxH	-3.03	9.36	-1.60	10.16	0.3	8.56	0.0	0.00056024	0.00422026	0.01885036	0.39517976	A_07_P016406	b0524	cmr|orf, hypothetical protein [b0524]
lpxK	-5.26	9.96	-2.39	11.16	0.0	8.77	0.0	0.00000815	0.00022481	0.00264926	0.17094262	A_07_P005022	b0915	cmr|putative EC 1.2 enzyme [b0915]
lpxL	-4.19	11.59	-2.07	12.62	0.0	10.55	0.1	0.00003109	0.00052951	0.00581491	0.25082192	A_07_P001480	b1054	cmr|heat shock protein [b1054]
lpxP	-3.17	11.31	-1.66	12.14	0.0	10.48	0.0	0.00007273	0.00095677	0.01629510	0.37727941	A_07_P014174	b2378	cmr|putative heat shock protein [b2378]
lrhA	-4.49	11.82	-2.17	12.90	0.0	10.73	0.0	0.00001382	0.00030876	0.00458079	0.22190769	A_07_P013734	b2289	cmr|NADH dehydrogenase transcriptional regulator, LysR family [b2289]
lrp	+4.93	10.06	+2.30	8.90	0.1	11.21	0.0	0.00001257	0.00029182	0.01043826	0.27996593	A_07_P004893	b0889	cmr|regulator for leucine [b0889]
lsrA	-2.06	8.12	-1.05	8.64	0.4	7.60	0.0	0.00755613	0.03148853	0.06627096	0.65210625	A_07_P020212	b1513	cmr|putative ATP-binding component of a transport system [b1513]
lsrK	-3.56	8.87	-1.83	9.79	0.3	7.95	0.0	0.00022992	0.00221785	0.01078570	0.32593090	A_07_P020203	b1511	cmr|putative kinase [b1511]
ltaE	+3.41	9.15	+1.77	8.26	0.0	10.03	0.2	0.00009602	0.00116203	0.02104357	0.38257506	A_07_P004805	b0870	cmr|putative arylsulfatase [b0870]
lysC	+2.31	7.51	+1.21	6.91	0.2	8.11	0.0	0.00091524	0.00611315	0.04638910	0.53193736	A_07_P008761	b4024	cmr|aspartokinase III, lysine sensitive [b4024]
lysS	-3.46	11.48	-1.79	12.38	0.1	10.58	0.3	0.00038471	0.00322860	0.01218750	0.33781690	A_07_P006416	b2890	cmr|lysine tRNA synthetase, constitutive; suppressor of ColE1 mutation in primer RNA [b2890]
lysU	+4.68	10.53	+2.23	9.41	0.2	11.64	0.1	0.00003300	0.00054981	0.01179052	0.29465079	A_07_P011381	b4129	cmr|lysine tRNA synthetase, inducible; heat shock protein [b4129]
lyxK	+3.86	7.85	+1.95	6.87	0.1	8.82	0.1	0.00003218	0.00054058	0.01701220	0.34604651	A_07_P039671	c_4403	cmr|Cryptic L-xylulose kinase [c_4403]
maa	-11.27	9.26	-3.49	11.01	0.1	7.51	0.4	0.00000852	0.00022936	0.00011115	0.03500000	A_07_P021405	b0459	cmr|putative transferase [b0459]
macB	-2.63	8.51	-1.40	9.21	0.0	7.81	0.0	0.00020530	0.00203026	0.03085938	0.49425229	A_07_P004848	b0879	cmr|putative ATP-binding component of a transport system [b0879]
maeA	+4.15	8.90	+2.05	7.87	0.1	9.92	0.1	0.00004876	0.00072623	0.01475864	0.32726761	A_07_P009486	b1479	cmr|NAD-linked malate dehydrogenase [b1479]
maeB	+3.21	10.86	+1.68	10.02	0.2	11.70	0.1	0.00015900	0.00169372	0.02402693	0.41072747	A_07_P013371	b2463	cmr|putative multimodular enzyme [b2463]
malE	+25.22	11.18	+4.66	8.85	0.1	13.50	0.2	0.00000028	0.00004211	0.00023819	0.04310345	A_07_P008811	b4034	cmr|periplasmic maltose-binding protein; substrate recognition for transport and chemotaxis [b4034]
malF	+2.39	7.93	+1.26	7.30	0.0	8.56	0.1	0.00040951	0.00338264	0.04250445	0.51044241	A_07_P008806	b4033	cmr|part of maltose permease, periplasmic [b4033]
malI	-2.25	10.12	-1.17	10.70	0.1	9.53	0.0	0.00085717	0.00582084	0.05161014	0.59266958	A_07_P020735	b1620	cmr|repressor of malX and Y genes [b1620]
malI	-2.27	10.32	-1.18	10.91	0.1	9.73	0.2	0.00103882	0.00670568	0.04934705	0.58547099	A_07_P042320	c_2012	cmr|Maltose regulon Regulatory protein malI [c_2012]
malK	+5.28	7.79	+2.40	6.59	0.0	8.99	0.2	0.00002992	0.00051429	0.00905234	0.26540037	A_07_P008816	b4035	cmr|ATP-binding component of transport system for maltose [b4035]
malM	+2.29	8.32	+1.20	7.72	0.1	8.92	0.2	0.00154571	0.00900319	0.04800241	0.54331416	A_07_P008827	b4037	cmr|periplasmic protein of mal regulon [b4037]
malP	+13.88	9.74	+3.79	7.85	0.1	11.64	0.1	0.00000087	0.00006750	0.00123730	0.10943820	A_07_P018111	b3417	cmr|maltodextrin phosphorylase [b3417]
malQ	+3.92	9.23	+1.97	8.25	0.1	10.22	0.1	0.00004647	0.00069738	0.01653328	0.34431217	A_07_P018108	b3416	cmr|4-alpha-glucanotransferase [b3416]
malT	+4.22	8.29	+2.08	7.25	0.0	9.33	0.6	0.00115321	0.00721338	0.01373539	0.31661786	A_07_P034438	c_4196	cmr|MalT Regulatory protein [c_4196]
malT	+4.42	8.24	+2.14	7.17	0.0	9.31	0.1	0.00002419	0.00044487	0.01309388	0.30814649	A_07_P018118	b3418	cmr|positive regulator of mal regulon [b3418]
malT	+5.21	8.63	+2.38	7.44	0.1	9.82	0.1	0.00001546	0.00033209	0.00937182	0.26925182	A_07_P052157	Z4774	cmr|positive regulator of mal regulon [Z4774]
malX	+3.06	9.62	+1.61	8.81	0.0	10.43	0.2	0.00023036	0.00221839	0.02613631	0.42730010	A_07_P020740	b1621	cmr|PTS system, maltose and glucose-specific II ABC [b1621]
malY	+3.01	9.27	+1.59	8.48	0.1	10.07	0.0	0.00014279	0.00155578	0.02665015	0.42727088	A_07_P042322	c_2014	cmr|MalY protein [c_2014]
manA	+2.59	8.89	+1.37	8.20	0.1	9.57	0.2	0.00049327	0.00386563	0.03610391	0.48089679	A_07_P020700	b1613	cmr|mannose-6-phosphate isomerase [b1613]
manA	+3.07	8.83	+1.62	8.02	0.1	9.64	0.1	0.00011710	0.00133575	0.02565930	0.42434874	A_07_P044397	Z2616	cmr|mannose-6-phosphate isomerase [Z2616]
manA	+3.01	8.92	+1.59	8.12	0.0	9.71	0.0	0.00009281	0.00113535	0.02655932	0.42711951	A_07_P035096	c_2005	cmr|Mannose-6-phosphate isomerase [c_2005]
manB	+2.20	12.50	+1.14	11.94	0.1	13.07	0.3	0.00363002	0.01762833	0.05234629	0.56447945	A_07_P031986	c_2557	cmr|Phosphomannomutase [c_2557]
manX	+10.14	9.96	+3.34	8.29	0.0	11.63	0.1	0.00000128	0.00008337	0.00254065	0.14705882	A_07_P015778	b1817	cmr|PTS enzyme IIAB, mannose-specific [b1817]
manY	+4.62	9.59	+2.21	8.48	0.1	10.69	0.1	0.00002145	0.00041085	0.01210302	0.29773437	A_07_P015784	b1818	cmr|PTS enzyme IIC, mannose-specific [b1818]
manZ	+3.92	9.54	+1.97	8.56	0.2	10.53	0.1	0.00007393	0.00096669	0.01627160	0.34294511	A_07_P015786	b1819	cmr|PTS enzyme IID, mannose-specific [b1819]
marA	+16.77	11.14	+4.07	9.10	0.1	13.17	0.3	0.00000327	0.00013195	0.00081428	0.08434211	A_07_P020303	b1531	cmr|multiple antibiotic resistance; transcriptional activator of defense systems [b1531]
marB	+8.31	9.69	+3.06	8.16	0.1	11.22	0.1	0.00000222	0.00010802	0.00365091	0.17418182	A_07_P020307	b1532	cmr|multiple antibiotic resistance protein [b1532]
marB	+5.24	8.78	+2.39	7.59	0.0	9.98	0.2	0.00002157	0.00041173	0.00924987	0.26819521	A_07_P048006	Z2169	cmr|multiple antibiotic resistance protein [Z2169]
marR	+10.74	12.62	+3.42	10.91	0.2	14.33	0.1	0.00000337	0.00013444	0.00217924	0.13615079	A_07_P020299	b1530	cmr|multiple antibiotic resistance protein; repressor of mar operon [b1530]
matA	-2.24	9.68	-1.16	10.26	0.4	9.10	0.2	0.00617113	0.02692859	0.05145706	0.59307467	A_07_P008164	b0294	cmr|putative regulator [b0294]
matA	-2.09	9.38	-1.07	9.91	0.3	8.84	0.2	0.00719933	0.03034706	0.06216019	0.63838878	A_07_P041479	c_0405	cmr|Hypothetical protein ykgK [c_0405]
matC	-3.00	9.06	-1.58	9.85	0.1	8.27	0.1	0.00021844	0.00213217	0.02016705	0.40811054	A_07_P008155	b0292	cmr|orf, hypothetical protein [b0292]
mchD	-2.01	11.79	-1.01	12.30	0.4	11.29	0.0	0.00918280	0.03674987	0.07327680	0.67073837	A_07_P040445	c_1230	cmr|MchD protein [c_1230]
mchF	-2.10	10.62	-1.07	11.16	0.1	10.08	0.3	0.00350426	0.01712857	0.06392149	0.64635838	A_07_P040453	c_1232	cmr|Probable microcin H47 secretion ATP-binding protein [c_1232]
mdaB	+6.24	9.87	+2.64	8.55	0.2	11.19	0.1	0.00001114	0.00027193	0.00641070	0.22886621	A_07_P012273	b3028	cmr|modulator of drug activity B [b3028]
mdh	+11.52	12.00	+3.53	10.24	0.0	13.76	0.1	0.00000071	0.00006188	0.00192581	0.12957265	A_07_P012651	b3236	cmr|malate dehydrogenase [b3236]
mdoC	-2.12	8.19	-1.09	8.73	0.0	7.65	0.1	0.00098590	0.00646480	0.06181339	0.63607190	A_07_P001448	b1047	cmr|orf, hypothetical protein [b1047]
mdoG	-2.00	11.12	-1.00	11.62	0.0	10.62	0.0	0.00139918	0.00832840	0.07444677	0.67594579	A_07_P001451	b1048	cmr|periplasmic glucans biosynthesis protein [b1048]
mdtI	+2.16	7.74	+1.11	7.18	0.1	8.29	0.3	0.00312969	0.01563754	0.05596735	0.57932281	A_07_P020626	b1599	cmr|possible chaperone [b1599]
mdtK	-3.70	11.06	-1.89	12.01	0.0	10.12	0.0	0.00003304	0.00054981	0.00929116	0.30860759	A_07_P018376	b1663	cmr|putative transport protein [b1663]
mdtL	-2.27	7.97	-1.18	8.56	0.3	7.38	0.2	0.00588358	0.02592801	0.04998603	0.58555060	A_07_P005475	b3710	cmr|putative transport protein [b3710]
mdtQ	-2.07	7.10	-1.05	7.62	0.0	6.57	0.1	0.00113988	0.00715494	0.06696710	0.65122298	A_07_P003459	b2139	cmr|orf, hypothetical protein [b2139]
mdtQ	-2.42	8.46	-1.27	9.10	0.1	7.82	0.0	0.00045239	0.00363081	0.04093115	0.54519459	A_07_P010504	b2139	cmr|orf, hypothetical protein [b2139]
melA	+3.20	8.92	+1.68	8.08	0.1	9.76	0.2	0.00015918	0.00169450	0.02376715	0.41074643	A_07_P011333	b4119	cmr|alpha-galactosidase [b4119]
menB	+8.69	9.99	+3.12	8.43	0.1	11.55	0.2	0.00000275	0.00012089	0.00330475	0.16783871	A_07_P001100	b2262	cmr|dihydroxynaphtoic acid synthetase [b2262]
menC	-2.12	12.61	-1.08	13.15	0.1	12.07	0.4	0.01300041	0.04861721	0.06451664	0.64779974	A_07_P001094	b2261	cmr|o-succinylbenzoyl-CoA synthase; conversion of chorismate to 2-o-succinylbenzoyl-CoA [b2261]
menD	+3.05	8.84	+1.61	8.03	0.1	9.64	0.1	0.00016483	0.00174046	0.02621443	0.42680455	A_07_P001109	b2264	cmr|2-oxoglutarate decarboxylase; SHCHC synthase [b2264]
menD	+2.11	8.91	+1.08	8.37	0.2	9.45	0.2	0.00318024	0.01585490	0.05734502	0.58474093	A_07_P032389	c_2808	cmr|Menaquinone biosynthesis protein menD [c_2808]
metB	+2.83	8.97	+1.50	8.22	0.2	9.72	0.2	0.00064035	0.00463527	0.03079903	0.44939759	A_07_P019474	b3939	cmr|cystathionine gamma-synthase [b3939]
metH	+3.58	8.38	+1.84	7.46	0.3	9.30	0.3	0.00044664	0.00360203	0.01942772	0.36631138	A_07_P057969	ECs4937	cmr|B12-dependent homocysteine-N5-methyltetrahydrofolate transmethylase [ECs4937]
metH	+4.52	8.78	+2.18	7.69	0.0	9.86	0.0	0.00001435	0.00031409	0.01251461	0.30173047	A_07_P047219	Z5610	cmr|B12-dependent homocysteine-N5-methyltetrahydrofolate transmethylase, repressor of metE and [Z5610]
metH	+2.77	7.94	+1.47	7.20	0.1	8.67	0.3	0.00082562	0.00565414	0.03225038	0.45908680	A_07_P008740	b4019	cmr|B12-dependent homocysteine-N5-methyltetrahydrofolate transmethylase, repressor of metE and metF [b4019]
metH	+4.75	9.57	+2.25	8.44	0.1	10.69	0.1	0.00002071	0.00040158	0.01145071	0.29218801	A_07_P037467	c_4976	cmr|5-methyltetrahydrofolate--homocysteine methyltransferase [c_4976]
metN	-4.73	10.54	-2.24	11.66	0.1	9.42	0.2	0.00002812	0.00049161	0.00383003	0.20440678	A_07_P002955	b0199	cmr|ATP-binding component of a transporter [b0199]
mglA	+11.66	8.95	+3.54	7.17	0.1	10.72	0.3	0.00000429	0.00015581	0.00183626	0.12848889	A_07_P003507	b2149	cmr|ATP-binding component of methyl-galactoside transport and galactose taxis [b2149]
mglB	+21.58	9.54	+4.43	7.32	0.3	11.75	0.1	0.00000092	0.00006969	0.00040587	0.05756757	A_07_P003515	b2150	cmr|galactose-binding transport protein; receptor for galactose taxis [b2150]
mglB	+28.71	10.62	+4.84	8.19	0.0	13.04	0.1	0.00000012	0.00003548	0.00016133	0.03628571	A_07_P032239	c_2684	cmr|D-galactose-binding periplasmic protein precursor [c_2684]
mglB	+39.48	10.87	+5.30	8.22	0.1	13.53	0.2	0.00000020	0.00003706	0.00006352	0.02127660	A_07_P045485	Z3405	cmr|galactose-binding transport protein receptor for galactose taxis [Z3405]
mglC	+8.64	9.42	+3.11	7.86	0.0	10.97	0.1	0.00000169	0.00009680	0.00340066	0.16889590	A_07_P003504	b2148	cmr|methyl-galactoside transport and galactose taxis [b2148]
mgsA	+6.96	10.52	+2.80	9.12	0.2	11.92	0.1	0.00000948	0.00024416	0.00504954	0.20811518	A_07_P005264	b0963	cmr|methylglyoxal synthase [b0963]
mgtA	-69.36	11.32	-6.12	14.38	0.0	8.26	0.1	0.00000002	0.00003344	0.00000064	0.00500000	A_07_P003761	b4242	cmr|Mg2+ transport ATPase, P-type 1 [b4242]
miaA	+2.65	12.96	+1.40	12.26	0.0	13.66	0.2	0.00060922	0.00447365	0.03449378	0.47223478	A_07_P011571	b4171	cmr|delta [b4171]
minD	+3.01	10.44	+1.59	9.65	0.2	11.24	0.1	0.00027215	0.00250274	0.02712589	0.42878514	A_07_P006827	b1175	cmr|cell division inhibitor, a membrane ATPase, activates minC [b1175]
minE	+5.68	12.35	+2.50	11.09	0.1	13.60	0.1	0.00000792	0.00022158	0.00780234	0.24666667	A_07_P006821	b1174	cmr|cell division topological specificity factor, reverses MinC inhibition of ftsZ ring formation [b1174]
mioC	-7.11	13.65	-2.83	15.07	0.0	12.24	0.1	0.00000458	0.00016295	0.00087081	0.09263514	A_07_P005634	b3742	cmr|initiation of chromosome replication [b3742]
mipA	+4.08	11.75	+2.03	10.73	0.1	12.76	0.1	0.00003080	0.00052589	0.01518928	0.33306407	A_07_P015602	b1782	cmr|orf, hypothetical protein [b1782]
mltA	-2.10	10.53	-1.07	11.07	0.1	9.99	0.0	0.00134944	0.00812449	0.06357088	0.64571613	A_07_P006061	b2813	cmr|membrane-bound lytic murein transglycosylase A [b2813]
mltD	+2.37	10.44	+1.24	9.82	0.2	11.07	0.3	0.00241629	0.01278694	0.04431021	0.52178010	A_07_P002982	b0211	cmr|transcriptional regulator for nitrite reductase [b0211]
mnmA	-2.53	11.94	-1.34	12.61	0.0	11.27	0.1	0.00036765	0.00311361	0.03578760	0.51644363	A_07_P006616	b1133	cmr|orf, hypothetical protein [b1133]
mntH	-7.68	9.81	-2.94	11.28	0.2	8.34	0.0	0.00000536	0.00017738	0.00067073	0.08123077	A_07_P014241	b2392	cmr|putative transport system permease [b2392]
moaB	+3.35	10.46	+1.74	9.59	0.1	11.33	0.2	0.00011308	0.00131105	0.02204332	0.39303511	A_07_P004361	b0782	cmr|molybdopterin biosynthesis, protein B [b0782]
moaC	+2.12	11.23	+1.08	10.69	0.3	11.77	0.1	0.00428750	0.02014998	0.05838669	0.59039178	A_07_P004368	b0783	cmr|molybdopterin biosynthesis, protein C [b0783]
MoaE	+2.37	10.98	+1.24	10.36	0.0	11.60	0.2	0.00068054	0.00483817	0.04360455	0.51733986	A_07_P041580	c_0867	cmr|Molybdopterin converting factor subunit 2 [c_0867]
mobA	+3.86	8.28	+1.95	7.30	0.4	9.25	0.1	0.00045252	0.00363081	0.01664444	0.34480263	A_07_P039291	c_4801	cmr|Molybdopterin-guanine dinucleotide biosynthesis protein A [c_4801]
modA	+2.02	9.19	+1.01	8.68	0.1	9.69	0.1	0.00145224	0.00855373	0.06231834	0.60601606	A_07_P041574	c_0840	cmr|Molybdate-binding periplasmic protein precursor [c_0840]
moeA	-2.10	11.36	-1.07	11.90	0.0	10.83	0.1	0.00109185	0.00695496	0.06404980	0.64599616	A_07_P004586	b0827	cmr|molybdopterin biosynthesis [b0827]
moeB	-3.20	8.85	-1.68	9.69	0.1	8.01	0.4	0.00073704	0.00514135	0.01600102	0.37656203	A_07_P004583	b0826	cmr|molybdopterin biosynthesis [b0826]
mokC	-4.48	12.19	-2.16	13.27	0.1	11.11	0.5	0.00038808	0.00324823	0.00460874	0.22257669	A_07_P010256	b0018	cmr|Gef protein interferes with membrane function when in excess [b0018]
mokC	-2.53	10.88	-1.34	11.55	0.1	10.21	0.2	0.00056081	0.00422257	0.03622650	0.51802906	A_07_P010779	b0018	cmr|Gef protein interferes with membrane function when in excess [b0018]
molR_C	+2.87	7.81	+1.52	7.05	0.2	8.57	0.6	0.00840445	0.03420749	0.03093877	0.45060130	A_07_P045191	Z3285	cmr|putative regulator [Z3285]
motA	-2.08	7.47	-1.06	8.00	0.0	6.94	0.1	0.00112016	0.00707411	0.06634591	0.65121571	A_07_P016130	b1890	cmr|proton conductor component of motor; no effect on switching [b1890]
motB	-3.70	8.26	-1.89	9.20	0.0	7.31	0.0	0.00003416	0.00056196	0.00917111	0.30721277	A_07_P016121	b1889	cmr|enables flagellar motor rotation, linking torque machinery to cell wall [b1889]
mpl	-2.23	12.34	-1.16	12.92	0.0	11.76	0.0	0.00060018	0.00443275	0.05307038	0.59895341	A_07_P003716	b4233	cmr|putative ligase [b4233]
mraZ	-3.50	12.24	-1.81	13.15	0.0	11.34	0.1	0.00005529	0.00078914	0.01141705	0.33164207	A_07_P011082	b0081	cmr|orf, hypothetical protein [b0081]
mrcA	-2.11	10.16	-1.08	10.70	0.1	9.62	0.1	0.00124122	0.00763651	0.06250572	0.63901948	A_07_P018008	b3396	cmr|peptidoglycan synthetase; penicillin-binding protein 1A [b3396]
mrcB	-3.15	11.08	-1.65	11.90	0.5	10.25	0.5	0.00692650	0.02945729	0.01594766	0.37586826	A_07_P041431	c_0183	cmr|Penicillin-binding protein 1B [c_0183]
msbA	-5.28	10.13	-2.40	11.33	0.0	8.93	0.0	0.00000730	0.00020980	0.00258511	0.17172996	A_07_P005017	b0914	cmr|ATP-binding transport protein; multicopy suppressor of htrB [b0914]
msrA	+3.62	8.71	+1.86	7.78	0.1	9.64	0.1	0.00006711	0.00090923	0.01908791	0.36250905	A_07_P003647	b4219	cmr|peptide methionine sulfoxide reductase [b4219]
msrB	+7.19	13.51	+2.85	12.09	0.1	14.93	0.1	0.00000297	0.00012541	0.00484566	0.20289894	A_07_P015584	b1778	cmr|orf, hypothetical protein [b1778]
msyB	+2.54	10.22	+1.34	9.55	0.1	10.89	0.1	0.00046010	0.00368076	0.03802020	0.48705452	A_07_P040763	c_1318	cmr|Acidic protein msyB [c_1318]
mtfA	+2.93	7.75	+1.55	6.98	0.1	8.53	0.3	0.00099972	0.00652550	0.02880589	0.43988361	A_07_P015234	b1976	cmr|orf, hypothetical protein [b1976]
mtlA	+3.59	8.17	+1.84	7.25	0.0	9.09	0.1	0.00004514	0.00068369	0.01948107	0.36687799	A_07_P049516	Z5023	cmr|PTS system, mannitol-specific enzyme IIABC components [Z5023]
mtlA	+4.16	8.40	+2.06	7.37	0.0	9.43	0.0	0.00002128	0.00040998	0.01469385	0.32675141	A_07_P039704	c_4416	cmr|PTS system, mannitol-specific IIABC component [c_4416]
mtlD	+2.83	8.86	+1.50	8.11	0.0	9.61	0.2	0.00034533	0.00296290	0.03045605	0.44771242	A_07_P039708	c_4417	cmr|Mannitol-1-phosphate 5-dehydrogenase [c_4417]
mtr	+7.41	8.51	+2.89	7.06	0.0	9.95	0.1	0.00000268	0.00012042	0.00464050	0.19639785	A_07_P000526	b3161	cmr|tryptophan-specific transport protein [b3161]
mtr	+16.11	9.96	+4.01	7.96	0.0	11.97	0.1	0.00000033	0.00004563	0.00087462	0.08883871	A_07_P041776	c_3914	cmr|Tryptophan-specific transport protein [c_3914]
mukB	+2.62	9.70	+1.39	9.01	0.1	10.39	0.1	0.00023706	0.00226063	0.03527693	0.47755804	A_07_P005066	b0924	cmr|kinesin-like cell division protein involved in chromosome partitioning [b0924]
murD	+2.00	7.79	+1.00	7.29	0.0	8.29	0.0	0.00132935	0.00802484	0.06274390	0.60752768	A_07_P049185	Z0098	cmr|UDP-N-acetylmuramoylalanine-D-glutamate ligase [Z0098]
murI	-3.57	10.48	-1.84	11.40	0.1	9.56	0.1	0.00008655	0.00108321	0.01086763	0.32405303	A_07_P008540	b3967	cmr|glutamate racemase, required for biosynthesis of D-glutamate and peptidoglycan [b3967]
murQ	-2.26	10.08	-1.18	10.67	0.3	9.49	0.0	0.00303395	0.01528382	0.05111662	0.59088106	A_07_P013199	b2428	cmr|putative regulator [b2428]
mutH	-2.09	8.44	-1.07	8.97	0.0	7.90	0.2	0.00270748	0.01396220	0.06367442	0.64551835	A_07_P041691	c_3426	cmr|DNA mismatch repair protein mutH [c_3426]
mutM	-3.36	9.92	-1.75	10.80	0.0	9.05	0.2	0.00012598	0.00141677	0.01345084	0.34830592	A_07_P017118	b3635	cmr|formamidopyrimidine DNA glycosylase [b3635]
mutY	-2.60	11.53	-1.38	12.22	0.1	10.84	0.0	0.00026734	0.00246573	0.03284235	0.50544477	A_07_P052786	Z4306	cmr|adenine glycosylase G.C --> T.A transversions [Z4306]
mutY	-4.03	11.78	-2.01	12.79	0.5	10.78	0.1	0.00071872	0.00503139	0.00700394	0.26699758	A_07_P011946	b2961	cmr|adenine glycosylase; G.C --> T.A transversions [b2961]
nac	-9.29	9.38	-3.22	10.98	0.1	7.77	0.1	0.00000263	0.00011937	0.00030170	0.05337079	A_07_P015279	b1988	cmr|nitrogen assimilation control protein [b1988]
nadA	-2.54	8.39	-1.35	9.06	0.2	7.72	0.1	0.00090567	0.00607538	0.03482978	0.51296539	A_07_P033430	c_0825	cmr|Quinolinate synthetase A [c_0825]
nadA	-2.99	8.42	-1.58	9.22	0.0	7.63	0.1	0.00015288	0.00164413	0.02028963	0.40953846	A_07_P046361	Z0919	cmr|quinolinate synthetase, A protein [Z0919]
nadE	+6.24	9.58	+2.64	8.26	0.0	10.90	0.1	0.00000523	0.00017498	0.00638465	0.22897494	A_07_P018749	b1740	cmr|NAD synthetase, prefers NH3 over glutamine [b1740]
nagA	+2.69	9.51	+1.43	8.80	0.2	10.23	0.2	0.00052411	0.00401856	0.03396024	0.47024626	A_07_P009903	b0677	cmr|N-acetylglucosamine-6-phosphate deacetylase [b0677]
nagB	+2.07	8.36	+1.05	7.84	0.1	8.88	0.1	0.00179587	0.01015231	0.05877096	0.59313462	A_07_P009910	b0678	cmr|glucosamine-6-phosphate deaminase [b0678]
nagC	+2.24	8.26	+1.16	7.68	0.1	8.84	0.1	0.00077166	0.00534259	0.04929497	0.55238434	A_07_P009896	b0676	cmr|transcriptional repressor of nag [b0676]
nagK	+2.57	9.84	+1.36	9.16	0.1	10.52	0.0	0.00031484	0.00277226	0.03677718	0.48413043	A_07_P001798	b1119	cmr|putative NAGC-like transcriptional regulator [b1119]
nagZ	+5.60	9.24	+2.48	8.00	0.0	10.48	0.1	0.00000893	0.00023708	0.00802210	0.25009901	A_07_P001737	b1107	cmr|orf, hypothetical protein [b1107]
nanA	+3.71	8.73	+1.89	7.78	0.4	9.67	0.1	0.00056737	0.00425363	0.01820757	0.35390123	A_07_P041797	c_3979	cmr|N-acetylneuraminate lyase subunit [c_3979]
nanE	+3.37	8.26	+1.75	7.38	0.0	9.13	0.0	0.00005119	0.00074921	0.02157965	0.38872998	A_07_P012586	b3223	cmr|putative enzyme [b3223]
nanK	+3.56	7.59	+1.83	6.67	0.0	8.50	0.0	0.00004100	0.00063851	0.01983613	0.36914894	A_07_P012584	b3222	cmr|putative NAGC-like transcriptional regulator [b3222]
napA	+2.02	10.04	+1.02	9.53	0.1	10.55	0.1	0.00222468	0.01200322	0.06290079	0.60792511	A_07_P032316	c_2745	cmr|Periplasmic nitrate reductase precursor [c_2745]
napA	+2.26	10.06	+1.18	9.48	0.0	10.65	0.1	0.00070994	0.00498096	0.04810722	0.54410920	A_07_P032316	c_2745	cmr|Periplasmic nitrate reductase precursor [c_2745]
napB	+3.36	9.08	+1.75	8.20	0.1	9.95	0.3	0.00042314	0.00347436	0.02159045	0.38848000	A_07_P000804	b2203	cmr|cytochrome c-type protein [b2203]
napF	-3.74	11.18	-1.90	12.13	0.1	10.23	0.1	0.00004604	0.00069298	0.00887005	0.30161987	A_07_P000827	b2208	cmr|ferredoxin-type protein: electron transfer [b2208]
napH	+2.73	9.06	+1.45	8.34	0.1	9.79	0.1	0.00019107	0.00193740	0.03236408	0.46028907	A_07_P000808	b2204	cmr|ferredoxin-type protein: electron transfer [b2204]
narP	+2.99	8.17	+1.58	7.38	0.3	8.96	0.1	0.00045270	0.00363081	0.02757812	0.43202985	A_07_P000752	b2193	cmr|nitrate/nitrite response regulator [b2193]
ndk	+8.49	11.42	+3.09	9.88	0.1	12.96	0.3	0.00001000	0.00025069	0.00356390	0.17317901	A_07_P013646	b2518	cmr|nucleoside diphosphate kinase [b2518]
nemA	+3.01	10.19	+1.59	9.39	0.0	10.98	0.0	0.00009448	0.00114770	0.02638529	0.42606154	A_07_P020882	b1650	cmr|N-ethylmaleimide reductase [b1650]
nfi	+6.02	9.66	+2.59	8.36	0.1	10.95	0.2	0.00001080	0.00026565	0.00695376	0.23851852	A_07_P037441	c_4955	cmr|Endonuclease V [c_4955]
nfi	+5.89	9.33	+2.56	8.05	0.0	10.61	0.1	0.00000575	0.00018174	0.00723514	0.24236170	A_07_P008655	b3998	cmr|endonuclease V [b3998]
nfi	+3.45	9.39	+1.79	8.50	0.3	10.28	0.1	0.00030163	0.00269055	0.02081809	0.38023202	A_07_P047202	Z5574	cmr|endonuclease V [Z5574]
nfo	+2.17	8.26	+1.12	7.70	0.1	8.82	0.4	0.00751690	0.03137488	0.05484566	0.57336653	A_07_P003556	b2159	cmr|endonuclease IV [b2159]
nfsB	+2.60	10.17	+1.38	9.48	0.0	10.86	0.1	0.00024818	0.00233418	0.03569487	0.47828085	A_07_P016654	b0578	cmr|oxygen-insensitive NAD [b0578]
nhaA	-7.80	11.51	-2.96	13.00	0.0	10.03	0.1	0.00000272	0.00012064	0.00061293	0.07845528	A_07_P010784	b0019	cmr|Na+/H antiporter, pH dependent [b0019]
nhaR	-3.18	9.57	-1.67	10.41	0.1	8.74	0.1	0.00011453	0.00132092	0.01612424	0.37608889	A_07_P035651	c_0025	cmr|Transcriptional activator protein nhaR [c_0025]
nikA	+3.30	7.87	+1.72	7.01	0.2	8.73	0.1	0.00018312	0.00187662	0.02271341	0.40179775	A_07_P007316	b3476	cmr|periplasmic binding protein for nickel [b3476]
nikD	+2.02	7.93	+1.02	7.42	0.1	8.44	0.1	0.00217860	0.01181128	0.06218115	0.60580446	A_07_P007331	b3479	cmr|ATP-binding protein of nickel transport system [b3479]
nlpA	-3.45	8.75	-1.78	9.65	0.1	7.86	0.1	0.00008517	0.00107193	0.01206872	0.33809609	A_07_P017245	b3661	cmr|lipoprotein-28 [b3661]
nlpI	-6.83	14.72	-2.77	16.11	0.0	13.33	0.3	0.00001971	0.00038945	0.00098196	0.10038961	A_07_P000538	b3163	cmr|putative control proteins [b3163]
nmpC	+32.56	10.87	+5.03	8.36	0.5	13.38	0.1	0.00000480	0.00016636	0.00010226	0.02775862	A_07_P038497	c_1560	cmr|Outer membrane porin protein nmpC precursor [c_1560]
nohA	+5.60	10.20	+2.48	8.95	0.0	11.44	0.0	0.00000631	0.00019141	0.00798971	0.24958333	A_07_P038523	c_1568	cmr|Prophage Qin DNA packaging protein NU1 homolog [c_1568]
nrdA	+68.48	10.98	+6.10	7.93	0.1	14.03	0.1	0.00000003	0.00003344	0.00000635	0.00588235	A_07_P000956	b2234	cmr|ribonucleoside diphosphate reductase 1, alpha subunit, B1 [b2234]
nrdB	+14.64	10.78	+3.87	8.85	0.1	12.72	0.1	0.00000047	0.00005206	0.00109820	0.10111111	A_07_P000962	b2235	cmr|ribonucleoside-diphosphate reductase 1, beta subunit, B2 [b2235]
nrdD	+23.69	12.69	+4.57	10.41	0.1	14.98	0.0	0.00000015	0.00003548	0.00029535	0.04946809	A_07_P003742	b4238	cmr|anaerobic ribonucleoside-triphosphate reductase [b4238]
nrdD	+24.61	13.22	+4.62	10.91	0.0	15.53	0.2	0.00000029	0.00004211	0.00025025	0.04477273	A_07_P042066	c_5337	cmr|Anaerobic ribonucleoside-triphosphate reductase [c_5337]
nrdG	+8.85	9.99	+3.15	8.41	0.1	11.56	0.2	0.00000497	0.00016940	0.00323234	0.16630719	A_07_P003737	b4237	cmr|anaerobic ribonucleotide reductase activating protein [b4237]
nrdR	-4.90	13.12	-2.29	14.27	0.1	11.98	0.1	0.00001553	0.00033216	0.00337335	0.19454212	A_07_P021179	b0413	cmr|orf, hypothetical protein [b0413]
nudB	+3.16	10.88	+1.66	10.05	0.1	11.71	0.0	0.00009191	0.00112960	0.02422447	0.41275974	A_07_P016003	b1865	cmr|dATP pyrophosphohydrolase [b1865]
nudB	+3.24	11.20	+1.70	10.36	0.1	12.05	0.2	0.00020432	0.00202571	0.02351245	0.40949115	A_07_P016003	b1865	cmr|dATP pyrophosphohydrolase [b1865]
nudB	+2.03	10.83	+1.02	10.32	0.0	11.34	0.4	0.01278001	0.04792962	0.06442581	0.61962126	A_07_P016003	b1865	cmr|dATP pyrophosphohydrolase [b1865]
nudB	+3.23	11.03	+1.69	10.18	0.0	11.87	0.1	0.00007007	0.00093648	0.02322599	0.40765886	A_07_P016003	b1865	cmr|dATP pyrophosphohydrolase [b1865]
nudB	+2.23	10.68	+1.16	10.10	0.0	11.26	0.3	0.00414201	0.01958311	0.05046939	0.55839072	A_07_P016003	b1865	cmr|dATP pyrophosphohydrolase [b1865]
nudE	-2.84	10.45	-1.51	11.21	0.1	9.70	0.2	0.00039473	0.00329861	0.02404980	0.44756501	A_07_P018014	b3397	cmr|orf, hypothetical protein [b3397]
nudE	-2.36	10.95	-1.24	11.57	0.1	10.34	0.1	0.00048176	0.00380646	0.04425305	0.56277868	A_07_P059766	ECs4239	cmr|hypothetical protein [ECs4239]
nudE	-2.68	11.39	-1.42	12.10	0.0	10.67	0.0	0.00017274	0.00179989	0.02938897	0.48147763	A_07_P046016	Z4751	cmr|orf, hypothetical protein [Z4751]
nudI	+3.75	8.43	+1.91	7.47	0.1	9.38	0.1	0.00006099	0.00084745	0.01815041	0.35322621	A_07_P001045	b2251	cmr|orf, hypothetical protein [b2251]
nuoC	+7.21	11.00	+2.85	9.58	0.0	12.43	0.5	0.00007824	0.00100225	0.00505526	0.20780679	A_07_P013718	b2286	cmr|NADH dehydrogenase I chain C, D [b2286]
nuoE	+7.94	13.09	+2.99	11.59	0.0	14.58	0.0	0.00000198	0.00010186	0.00399708	0.18293605	A_07_P042543	c_2826	cmr|NADH dehydrogenase I chain E [c_2826]
nuoE	+11.33	11.49	+3.50	9.74	0.0	13.25	0.0	0.00000070	0.00006128	0.00196646	0.13063291	A_07_P013712	b2285	cmr|NADH dehydrogenase I chain E [b2285]
nuoE	+9.25	11.69	+3.21	10.09	0.1	13.30	0.2	0.00000348	0.00013688	0.00285887	0.15574394	A_07_P052679	Z3544	cmr|NADH dehydrogenase I chain E [Z3544]
nuoF	+26.57	10.85	+4.73	8.48	0.1	13.21	0.2	0.00000059	0.00005706	0.00020960	0.04024390	A_07_P013708	b2284	cmr|NADH dehydrogenase I chain F [b2284]
nuoG	+12.99	9.16	+3.70	7.31	0.1	11.01	0.0	0.00000075	0.00006289	0.00146532	0.11592965	A_07_P013701	b2283	cmr|NADH dehydrogenase I chain G [b2283]
nuoG	+12.44	10.00	+3.64	8.18	0.0	11.81	0.4	0.00001147	0.00027642	0.00154980	0.11730769	A_07_P045556	Z3542	cmr|NADH dehydrogenase I chain G [Z3542]
nuoH	+39.17	10.17	+5.29	7.52	0.0	12.81	0.1	0.00000006	0.00003344	0.00006352	0.02173913	A_07_P013700	b2282	cmr|NADH dehydrogenase I chain H [b2282]
nuoI	+10.09	9.66	+3.33	7.99	0.7	11.32	0.3	0.00034716	0.00297369	0.00247269	0.14746212	A_07_P013694	b2281	cmr|NADH dehydrogenase I chain I [b2281]
nuoJ	+15.01	10.01	+3.91	8.06	0.3	11.96	0.1	0.00000286	0.00012365	0.00100927	0.09689024	A_07_P013686	b2280	cmr|NADH dehydrogenase I chain J [b2280]
nuoK	+20.58	9.21	+4.36	7.03	0.0	11.39	0.0	0.00000018	0.00003688	0.00046939	0.06158333	A_07_P001181	b2279	cmr|NADH dehydrogenase I chain K [b2279]
nuoK	+22.54	9.45	+4.49	7.20	0.0	11.69	0.2	0.00000058	0.00005706	0.00032203	0.05226804	A_07_P045553	Z3538	cmr|NADH dehydrogenase I chain K [Z3538]
nuoL	+12.70	9.30	+3.67	7.47	0.4	11.13	0.1	0.00000815	0.00022481	0.00145071	0.11593909	A_07_P001178	b2278	cmr|NADH dehydrogenase I chain L [b2278]
nuoM	+11.35	8.93	+3.50	7.18	0.2	10.68	0.1	0.00000225	0.00010894	0.00197027	0.13033613	A_07_P001174	b2277	cmr|NADH dehydrogenase I chain M [b2277]
nuoN	+6.33	8.05	+2.66	6.72	0.1	9.38	0.0	0.00000542	0.00017834	0.00635544	0.22949541	A_07_P051791	Z3534	cmr|NADH dehydrogenase I chain N [Z3534]
nuoN	+4.80	8.16	+2.26	7.03	0.2	9.29	0.1	0.00002594	0.00046513	0.01106644	0.28703460	A_07_P042539	c_2817	cmr|NADH dehydrogenase I chain N [c_2817]
nuoN	+4.25	8.22	+2.09	7.17	0.2	9.26	0.2	0.00011526	0.00132359	0.01421367	0.32244957	A_07_P001166	b2276	cmr|NADH dehydrogenase I chain N [b2276]
nupC	+2.11	8.16	+1.08	7.62	0.4	8.70	0.1	0.00731353	0.03067313	0.05940612	0.59572611	A_07_P014250	b2393	cmr|permease of transport system for 3 nucleosides [b2393]
nupG	+6.39	10.34	+2.68	9.00	0.1	11.67	0.1	0.00000476	0.00016625	0.00617124	0.22542923	A_07_P011964	b2964	cmr|transport of nucleosides, permease protein [b2964]
nusA	-3.56	14.61	-1.83	15.52	0.1	13.69	0.3	0.00019363	0.00195541	0.01038110	0.32173228	A_07_P000566	b3169	cmr|transcription pausing; L factor [b3169]
nusB	-9.22	12.33	-3.20	13.93	0.0	10.72	0.3	0.00001148	0.00027642	0.00029853	0.05340909	A_07_P021194	b0416	cmr|transcription termination; L factor [b0416]
obgE	-3.64	9.79	-1.86	10.72	0.1	8.85	0.6	0.00239135	0.01268674	0.01077045	0.32609615	A_07_P012391	b3183	cmr|putative GTP-binding factor [b3183]
ompC	+4.05	9.80	+2.02	8.79	0.1	10.80	0.6	0.00117535	0.00731126	0.01544970	0.33643154	A_07_P032359	c_2758	cmr|Outer membrane protein C precursor [c_2758]
ompC	+5.47	9.27	+2.45	8.05	0.1	10.50	0.1	0.00000991	0.00025045	0.00835493	0.25344894	A_07_P000863	b2215	cmr|outer membrane protein 1b [b2215]
ompF	+4.47	9.87	+2.16	8.79	0.1	10.95	0.4	0.00033964	0.00292453	0.01234693	0.29952234	A_07_P005092	b0929	cmr|outer membrane protein 1a [b0929]
ompT	+3.07	8.45	+1.62	7.64	0.3	9.26	0.2	0.00092130	0.00614098	0.02614710	0.42703320	A_07_P031283	c_0652	cmr|Protease VII precursor [c_0652]
ompW	+55.19	9.85	+5.79	6.96	0.0	12.75	0.4	0.00000037	0.00004777	0.00001715	0.01038462	A_07_P007216	b1256	cmr|putative outer membrane protein [b1256]
ompW	+181.43	12.40	+7.50	8.65	0.0	16.15	0.2	0.00000001	0.00003344	0.00000000	0.00000000	A_07_P038872	c_1722	cmr|Outer membrane protein W precursor [c_1722]
ompX	-9.99	13.66	-3.32	15.32	0.0	12.00	0.1	0.00000120	0.00008111	0.00019182	0.04507463	A_07_P004524	b0814	cmr|outer membrane protein X [b0814]
oppA	+32.28	10.63	+5.01	8.12	0.2	13.13	0.2	0.00000056	0.00005541	0.00010036	0.02771930	A_07_P007151	b1243	cmr|oligopeptide transport; periplasmic binding protein [b1243]
oppA	+56.77	11.87	+5.83	8.95	0.0	14.78	0.0	0.00000003	0.00003344	0.00001334	0.01000000	A_07_P052437	Z2019	cmr|oligopeptide transport periplasmic binding protein [Z2019]
oppB	+2.76	9.43	+1.47	8.70	0.0	10.17	0.2	0.00030103	0.00268893	0.03186674	0.45944139	A_07_P007157	b1244	cmr|oligopeptide transport permease protein [b1244]
oppC	+4.01	8.22	+2.00	7.21	0.0	9.22	0.5	0.00066760	0.00477761	0.01505589	0.33106145	A_07_P007165	b1245	cmr|homolog of Salmonella oligopeptide transport permease protein [b1245]
oppD	+12.31	10.32	+3.62	8.51	0.2	12.13	0.0	0.00000179	0.00009900	0.00167810	0.12119266	A_07_P007166	b1246	cmr|homolog of Salmonella ATP-binding protein of oligopeptide ABC transport system [b1246]
oppF	+10.42	9.19	+3.38	7.50	0.3	10.88	0.2	0.00000912	0.00023883	0.00226435	0.13817829	A_07_P007171	b1247	cmr|homolog of Salmonella ATP-binding protein of oligopeptide ABC transport system [b1247]
osmB	-5.28	9.02	-2.40	10.22	0.1	7.82	0.2	0.00002287	0.00043013	0.00263592	0.17148760	A_07_P014396	b1283	cmr|osmotically inducible lipoprotein [b1283]
osmC	-2.09	10.99	-1.06	11.52	0.0	10.45	0.3	0.00593785	0.02612058	0.06525597	0.64942478	A_07_P009499	b1482	cmr|osmotically inducible protein [b1482]
osmE	+2.81	8.26	+1.49	7.52	0.1	9.01	0.5	0.00321406	0.01595819	0.03306911	0.46568873	A_07_P018744	b1739	cmr|activator of ntrL gene [b1739]
osmY	+2.71	8.82	+1.44	8.10	0.1	9.54	0.1	0.00035020	0.00299483	0.03325838	0.46710080	A_07_P017561	b4376	cmr|hyperosmotically inducible periplasmic protein [b4376]
paaF	-2.55	8.45	-1.35	9.13	0.6	7.78	0.0	0.00969192	0.03827176	0.03312691	0.50586809	A_07_P009069	b1393	cmr|putative enzyme [b1393]
pal	+2.09	11.31	+1.07	10.78	0.1	11.84	0.3	0.00353391	0.01724670	0.05792683	0.58876695	A_07_P010223	b0741	cmr|peptidoglycan-associated lipoprotein [b0741]
panB	-3.03	10.48	-1.60	11.28	0.0	9.68	0.1	0.00011362	0.00131450	0.01916603	0.39703947	A_07_P002634	b0134	cmr|3-methyl-2-oxobutanoate hydroxymethyltransferase [b0134]
panB	-3.30	12.55	-1.72	13.41	0.0	11.69	0.1	0.00007295	0.00095734	0.01414952	0.35700321	A_07_P035825	c_0165	cmr|3-methyl-2-oxobutanoate hydroxymethyltransferase [c_0165]
panD	+3.72	11.57	+1.89	10.62	0.0	12.52	0.0	0.00003140	0.00053108	0.01824251	0.35370690	A_07_P002617	b0131	cmr|aspartate 1-decarboxylase [b0131]
papA	+23.97	9.53	+4.58	7.24	0.2	11.83	0.2	0.00000083	0.00006559	0.00028074	0.04752688	A_07_P033164	c_3592	cmr|PapA protein [c_3592]
papC	+5.71	9.22	+2.51	7.97	0.1	10.48	0.0	0.00000717	0.00020686	0.00768991	0.24708163	A_07_P033151	c_3590	cmr|PapC protein [c_3590]
papD_2	+3.88	9.63	+1.95	8.65	0.1	10.61	0.2	0.00006976	0.00093397	0.01695694	0.34626459	A_07_P037957	c_5185	cmr|PapD protein [c_5185]
papE	+3.71	9.65	+1.89	8.71	0.1	10.60	0.2	0.00008738	0.00108817	0.01822536	0.35381011	A_07_P033134	c_3585	cmr|PapE protein [c_3585]
papH	+3.15	10.45	+1.65	9.62	0.3	11.28	0.2	0.00070887	0.00497570	0.02489774	0.41834578	A_07_P033156	c_3591	cmr|PapH protein [c_3591]
papH_2	+5.94	8.91	+2.57	7.62	0.2	10.19	0.4	0.00011622	0.00133068	0.00739520	0.24460084	A_07_P037969	c_5187	cmr|PapH protein [c_5187]
papI_2	+3.63	8.00	+1.86	7.07	0.2	8.93	0.0	0.00011648	0.00133244	0.01877033	0.35907655	A_07_P037980	c_5189	cmr|PapI protein [c_5189]
papJ	+2.56	9.20	+1.36	8.52	0.0	9.88	0.2	0.00048994	0.00384526	0.03700076	0.48504580	A_07_P033147	c_3588	cmr|PapJ protein [c_3588]
papK	+2.20	9.52	+1.13	8.96	0.2	10.09	0.3	0.00519099	0.02347799	0.05343750	0.56884381	A_07_P033137	c_3586	cmr|PapK protein [c_3586]
pck	+9.19	14.63	+3.20	13.03	0.1	16.23	0.1	0.00000159	0.00009392	0.00303036	0.15956522	A_07_P018041	b3403	cmr|phosphoenolpyruvate carboxykinase [b3403]
pcm	-2.28	11.12	-1.19	11.71	0.2	10.52	0.1	0.00103391	0.00668770	0.04909870	0.58384441	A_07_P002163	b2743	cmr|L-isoaspartate protein carboxylmethyltransferase type II [b2743]

pcnB	-2.11	11.59	-1.08	12.13	0.1	11.05	0.0	0.00101963	0.00661709	0.06291667	0.64197019	A_07_P002676	b0143	cmr|poly [b0143]
pdxB	+2.76	10.62	+1.47	9.89	0.0	11.35	0.2	0.00028651	0.00260187	0.03167048	0.45703025	A_07_P013888	b2320	cmr|erythronate-4-phosphate dehyrogenase [b2320]
pdxJ	+5.51	9.31	+2.46	8.08	0.0	10.54	0.1	0.00000951	0.00024416	0.00819042	0.25136452	A_07_P019721	b2564	cmr|pyridoxine biosynthesis [b2564]
pdxK	+2.15	9.96	+1.10	9.40	0.1	10.51	0.0	0.00085505	0.00581255	0.05384654	0.56858484	A_07_P013150	b2418	cmr|pyridoxal/pyridoxine/pyridoxamine kinase [b2418]
pdxY	+2.37	8.74	+1.24	8.12	0.3	9.36	0.1	0.00212805	0.01160110	0.04437754	0.52218236	A_07_P020811	b1636	cmr|pyridoxal kinase 2 / pyridoxine kinase [b1636]
pepB	+4.76	9.68	+2.25	8.56	0.0	10.81	0.2	0.00002036	0.00039679	0.01125762	0.28913540	A_07_P013674	b2523	cmr|putative peptidase [b2523]
pepD	+13.98	10.67	+3.81	8.76	0.1	12.57	0.0	0.00000057	0.00005636	0.00124238	0.10927374	A_07_P007896	b0237	cmr|aminoacyl-histidine dipeptidase [b0237]
pepE	+10.05	8.63	+3.33	6.96	0.1	10.29	0.1	0.00000137	0.00008682	0.00249746	0.14726592	A_07_P008748	b4021	cmr|peptidase E, a dipeptidase where amino-terminal residue is aspartate [b4021]
pepN	+8.81	9.39	+3.14	7.82	0.1	10.96	0.3	0.00001423	0.00031323	0.00333460	0.16826923	A_07_P051734	Z1280	cmr|aminopeptidase N [Z1280]
pepT	+7.74	9.56	+2.95	8.09	0.1	11.04	0.0	0.00000290	0.00012407	0.00413808	0.18614286	A_07_P006587	b1127	cmr|putative peptidase T [b1127]
perM	-3.44	9.18	-1.78	10.07	0.1	8.29	0.0	0.00006440	0.00088167	0.01225229	0.33842105	A_07_P030120	c_3009	cmr|Putative permease perM [c_3009]
pfkB	+5.58	9.50	+2.48	8.26	0.1	10.74	0.1	0.00001253	0.00029182	0.00807673	0.24884540	A_07_P041160	c_2121	cmr|6-phosphofructokinase isozyme 2 [c_2121]
pfkB	+6.05	9.61	+2.60	8.31	0.1	10.90	0.1	0.00000644	0.00019352	0.00693216	0.23881838	A_07_P018663	b1723	cmr|6-phosphofructokinase II; suppressor of pfkA [b1723]
pflA	-3.37	12.82	-1.75	13.69	0.0	11.94	0.1	0.00006673	0.00090725	0.01319169	0.34615000	A_07_P004958	b0902	cmr|pyruvate formate lyase activating enzyme 1 [b0902]
pflB	+14.84	11.26	+3.89	9.32	0.1	13.21	0.1	0.00000072	0.00006192	0.00108232	0.10023529	A_07_P004961	b0903	cmr|formate acetyltransferase 1 [b0903]
pgaD	-3.93	9.23	-1.97	10.22	0.0	8.24	0.5	0.00094607	0.00626098	0.00745871	0.27630588	A_07_P001332	b1021	cmr|orf, hypothetical protein [b1021]
pgi	+2.07	8.75	+1.05	8.22	0.3	9.27	0.1	0.00730844	0.03067313	0.06065485	0.60059748	A_07_P008767	b4025	cmr|glucosephosphate isomerase [b4025]
pgk	+4.76	12.84	+2.25	11.71	0.1	13.96	0.0	0.00001305	0.00029749	0.01141895	0.29185065	A_07_P011776	b2926	cmr|phosphoglycerate kinase [b2926]
pgl	+6.00	9.23	+2.59	7.94	0.0	10.52	0.3	0.00002566	0.00046319	0.00710938	0.24122845	A_07_P004288	b0767	cmr|putative isomerase [b0767]
pheS	+4.05	11.27	+2.02	10.26	0.0	12.28	0.0	0.00002141	0.00041080	0.01545668	0.33565517	A_07_P018621	b1714	cmr|phenylalanine tRNA synthetase, alpha-subunit [b1714]
pheT	+2.97	10.30	+1.57	9.51	0.1	11.08	0.0	0.00011716	0.00133575	0.02733994	0.42958084	A_07_P018619	b1713	cmr|phenylalanine tRNA synthetase, beta-subunit [b1713]
phnH	+2.25	8.45	+1.17	7.87	0.1	9.04	0.1	0.00072301	0.00505692	0.04879510	0.54952074	A_07_P037761	c_5106	cmr|PhnH protein [c_5106]
phnI	-3.83	10.91	-1.94	11.88	0.1	9.94	0.2	0.00007053	0.00094109	0.00806212	0.28979452	A_07_P011231	b4099	cmr|phosphonate metabolism [b4099]
phnI	-3.57	9.71	-1.84	10.63	0.3	8.80	0.1	0.00023004	0.00221785	0.01069296	0.32500000	A_07_P041400	c_5105	cmr|PhnI protein [c_5105]
phnJ	+2.25	7.45	+1.17	6.86	0.2	8.04	0.0	0.00115285	0.00721338	0.04909299	0.55208571	A_07_P011230	b4098	cmr|phosphonate metabolism [b4098]
phnK	+2.13	7.69	+1.09	7.14	0.2	8.23	0.3	0.00668870	0.02865544	0.05795605	0.58868387	A_07_P011223	b4097	cmr|ATP-binding component of phosphonate transport [b4097]
phoA	-2.83	8.53	-1.50	9.28	0.2	7.78	0.0	0.00025676	0.00239370	0.02428100	0.45027091	A_07_P021029	b0383	cmr|alkaline phosphatase [b0383]
phoB	-7.81	11.57	-2.96	13.06	0.1	10.09	0.1	0.00000250	0.00011564	0.00063453	0.08056452	A_07_P031103	c_0508	cmr|Phosphate regulon transcriptional Regulatory protein phoB [c_0508]
phoB	-8.01	11.26	-3.00	12.76	0.0	9.76	0.0	0.00000180	0.00009954	0.00056021	0.07603448	A_07_P021109	b0399	cmr|positive response regulator for pho regulon, sensor is PhoR [b0399]
phoB	-7.32	11.48	-2.87	12.92	0.0	10.05	0.1	0.00000375	0.00014286	0.00079713	0.08838028	A_07_P043823	Z0497	cmr|positive response regulator for pho regulon, sensor [Z0497]
phoR	-3.32	8.21	-1.73	9.08	0.1	7.35	0.1	0.00008353	0.00105543	0.01385480	0.35410714	A_07_P021115	b0400	cmr|positive and negative sensor protein for pho regulon [b0400]
phoU	+2.70	8.87	+1.43	8.16	0.2	9.59	0.2	0.00093094	0.00618686	0.03413364	0.46975524	A_07_P005542	b3724	cmr|negative regulator for pho regulon and putative enzyme in phosphate metabolism [b3724]
pinO	-2.17	10.81	-1.12	11.37	0.4	10.26	0.0	0.00908501	0.03645116	0.05842797	0.62154730	A_07_P041818	c_4093	cmr|PioO protein [c_4093]
pinR	-3.34	8.33	-1.74	9.20	0.1	7.46	0.0	0.00007898	0.00100904	0.01349657	0.34834426	A_07_P014850	b1374	cmr|putative transposon resolvase [b1374]
pmbA	+3.32	8.90	+1.73	8.04	0.2	9.77	0.2	0.00032446	0.00282692	0.02256034	0.39953881	A_07_P003729	b4235	cmr|maturation of antibiotic MccB17, see tld genes [b4235]
pntA	+6.21	8.74	+2.63	7.42	0.2	10.06	0.4	0.00013885	0.00152444	0.00680450	0.23545055	A_07_P020648	b1603	cmr|pyridine nucleotide transhydrogenase, alpha subunit [b1603]
pntB	+2.21	9.58	+1.14	9.01	0.2	10.15	0.1	0.00126513	0.00773824	0.05033854	0.55851304	A_07_P020644	b1602	cmr|pyridine nucleotide transhydrogenase, beta subunit [b1602]
pntB	+2.32	9.25	+1.21	8.65	0.0	9.86	0.1	0.00068978	0.00487650	0.04569106	0.52972018	A_07_P052517	Z2597	cmr|pyridine nucleotide transhydrogenase, beta subunit [Z2597]
polA	+6.60	9.02	+2.72	7.66	0.2	10.38	0.1	0.00000738	0.00021133	0.00580158	0.22009639	A_07_P019099	b3863	cmr|DNA polymerase I, 3 --> 5 polymerase, 5 --> 3 and 3 --> 5 exonuclease [b3863]
polA	+4.49	8.99	+2.17	7.91	0.4	10.08	0.0	0.00016069	0.00170704	0.01245236	0.30115207	A_07_P052260	Z5398	cmr|DNA polymerase I, 3 --> 5 polymerase [Z5398]
polB	-4.67	10.55	-2.22	11.66	0.0	9.44	0.2	0.00003483	0.00056774	0.00402058	0.20960265	A_07_P010989	b0060	cmr|DNA polymerase II [b0060]
potA	-2.82	10.60	-1.49	11.35	0.0	9.85	0.2	0.00024460	0.00230877	0.02441374	0.45060961	A_07_P006583	b1126	cmr|ATP-binding component of spermidine/putrescine transport [b1126]
potC	-5.77	11.51	-2.53	12.77	0.0	10.24	0.3	0.00003123	0.00053034	0.00195884	0.14616114	A_07_P006571	b1124	cmr|spermidine/putrescine transport system permease [b1124]
potD	+2.09	12.54	+1.06	12.01	0.0	13.07	0.1	0.00142163	0.00842385	0.05826283	0.58989711	A_07_P052411	Z1762	cmr|spermidine/putrescine periplasmic transport protein [Z1762]
potD	+2.21	12.42	+1.15	11.85	0.1	12.99	0.1	0.00117147	0.00730152	0.05102452	0.55942201	A_07_P001819	b1123	cmr|spermidine/putrescine periplasmic transport protein [b1123]
potE	+3.17	8.00	+1.67	7.17	0.2	8.84	0.1	0.00020269	0.00201497	0.02466019	0.41568522	A_07_P009978	b0692	cmr|putrescine transport protein [b0692]
ppc	+4.01	9.13	+2.01	8.13	0.1	10.13	0.1	0.00003460	0.00056633	0.01560340	0.33698217	A_07_P008482	b3956	cmr|phosphoenolpyruvate carboxylase [b3956]
ppdA	+2.71	8.00	+1.44	7.28	0.2	8.72	0.1	0.00054567	0.00413428	0.03293890	0.46468638	A_07_P043261	Z4143	cmr|prepilin peptidase dependent protein A [Z4143]
ppdA	+2.76	8.24	+1.46	7.50	0.1	8.97	0.1	0.00021829	0.00213217	0.03188326	0.45883912	A_07_P032800	c_3420	cmr|Prepilin peptidase dependent protein A precursor [c_3420]
ppdA	+2.11	8.22	+1.07	7.68	0.2	8.75	0.2	0.00317231	0.01582536	0.05779154	0.58777132	A_07_P006115	b2826	cmr|prepilin peptidase dependent protein A [b2826]
pphB	-3.58	7.97	-1.84	8.88	0.3	7.05	0.2	0.00035043	0.00299520	0.01037221	0.32272727	A_07_P002119	b2734	cmr|protein phosphatase 2 [b2734]
ppiB	+2.36	12.80	+1.24	12.18	0.1	13.42	0.1	0.00065556	0.00471498	0.04394627	0.51943694	A_07_P016413	b0525	cmr|peptidyl-prolyl cis-trans isomerase B [b0525]
ppiC	+2.06	8.22	+1.04	7.70	0.1	8.74	0.1	0.00160402	0.00929472	0.05999174	0.59703540	A_07_P005759	b3775	cmr|peptidyl-prolyl cis-trans isomerase C [b3775]
ppk	+2.96	9.74	+1.57	8.95	0.2	10.52	0.1	0.00028701	0.00260187	0.02802973	0.43435039	A_07_P013561	b2501	cmr|polyphosphate kinase [b2501]
pps	+6.20	13.75	+2.63	12.44	0.0	15.07	0.2	0.00001248	0.00029182	0.00656949	0.23242697	A_07_P018562	b1702	cmr|phosphoenolpyruvate synthase [b1702]
prfA	-2.32	10.08	-1.21	10.69	0.1	9.47	0.0	0.00074065	0.00515964	0.04707571	0.57543478	A_07_P007006	b1211	cmr|peptide chain release factor RF-1 [b1211]
prfB	-2.50	13.96	-1.32	14.62	0.0	13.30	0.2	0.00058921	0.00436953	0.03606009	0.51847489	A_07_P006421	b2891	cmr|peptide chain release factor RF-2 [b2891]
prfB	-2.85	14.32	-1.51	15.07	0.0	13.56	0.2	0.00024009	0.00227709	0.02339876	0.44118563	A_07_P032852	c_5622	cmr|Peptide chain release factor 2 [c_5622]
prfH	-9.37	10.40	-3.23	12.01	0.0	8.78	0.1	0.00000176	0.00009825	0.00029281	0.05488095	A_07_P030853	c_0382	cmr|Peptide chain release factor homolog [c_0382]
prfH	-9.50	10.65	-3.25	12.28	0.2	9.03	0.1	0.00000297	0.00012541	0.00027058	0.05392405	A_07_P007894	b0236	cmr|probable peptide chain release factor [b0236]
prfH	-9.44	9.05	-3.24	10.67	0.1	7.43	0.0	0.00000126	0.00008261	0.00028646	0.05500000	A_07_P062922	ECs0263	cmr|probable peptide chain release factor [ECs0263]
prfH	-6.60	9.31	-2.72	10.67	0.0	7.95	0.3	0.00002154	0.00041173	0.00116870	0.11084337	A_07_P051892	Z0297	cmr|probable peptide chain release factor [Z0297]
priA	-3.06	9.96	-1.61	10.76	0.2	9.15	0.3	0.00080002	0.00550019	0.01804243	0.38700272	A_07_P039449	c_4888	cmr|Primosomal protein N [c_4888]
prlC	+2.22	8.78	+1.15	8.20	0.0	9.35	0.1	0.00064203	0.00464101	0.04994347	0.55766667	A_07_P007426	b3498	cmr|oligopeptidase A [b3498]
prlC	+2.35	9.38	+1.24	8.76	0.0	10.00	0.1	0.00042967	0.00350504	0.04392276	0.51993985	A_07_P041321	c_4297	cmr|Oligopeptidase A [c_4297]
prmC	-2.18	9.89	-1.12	10.45	0.1	9.33	0.0	0.00087570	0.00591971	0.05724657	0.61605605	A_07_P007012	b1212	cmr|possible protoporphyrinogen oxidase [b1212]
proC	+2.02	9.25	+1.01	8.74	0.0	9.75	0.0	0.00124238	0.00763763	0.06180259	0.60548849	A_07_P021045	b0386	cmr|pyrroline-5-carboxylate reductase [b0386]
proP	-6.86	10.06	-2.78	11.45	0.1	8.67	0.2	0.00000636	0.00019181	0.00101499	0.10050314	A_07_P011292	b4111	cmr|low-affinity transport system; proline permease II [b4111]
proV	-2.08	8.97	-1.06	9.50	0.0	8.45	0.0	0.00105283	0.00677115	0.06660252	0.65129814	A_07_P030555	c_3230	cmr|Glycine betaine/L-proline transport ATP-binding protein proV [c_3230]
proV	-2.82	8.57	-1.50	9.32	0.4	7.83	0.1	0.00201832	0.01112623	0.02372523	0.44414982	A_07_P001859	b2677	cmr|ATP-binding component of transport system for glycine, betaine and proline [b2677]
proW	-2.46	8.60	-1.30	9.26	0.0	7.95	0.1	0.00046058	0.00368276	0.03911966	0.53556522	A_07_P001861	b2678	cmr|high-affinity transport system for glycine betaine and proline [b2678]
proX	+3.29	8.35	+1.72	7.49	0.5	9.21	0.0	0.00165174	0.00950126	0.02348196	0.40986696	A_07_P001866	b2679	cmr|high-affinity transport system for glycine betaine and proline [b2679]
proX	+5.23	9.01	+2.39	7.81	0.2	10.20	0.2	0.00005120	0.00074921	0.00932038	0.26875458	A_07_P030557	c_3232	cmr|Glycine betaine-binding periplasmic protein precursor [c_3232]
prpR	-3.40	10.13	-1.76	11.01	0.2	9.25	0.2	0.00021278	0.00209373	0.01304243	0.34510924	A_07_P008344	b0330	cmr|regulator for prp operon [b0330]
prpR	-3.41	10.99	-1.77	11.87	0.3	10.10	0.3	0.00063087	0.00459623	0.01234693	0.33631488	A_07_P031011	c_0450	cmr|Propionate catabolism operon Regulatory protein [c_0450]
psiF	-2.10	8.84	-1.07	9.37	0.2	8.30	0.1	0.00264678	0.01374372	0.06372967	0.64566281	A_07_P062746	ECs0434	cmr|phosphate starvation-induced protein psiF [ECs0434]
psiF	-2.03	8.94	-1.02	9.45	0.1	8.43	0.0	0.00138860	0.00828407	0.07172955	0.66783560	A_07_P051681	Z0480	cmr|induced by phosphate starvation [Z0480]
pspB	-8.59	10.15	-3.10	11.71	0.1	8.60	0.1	0.00000234	0.00011149	0.00044588	0.06750000	A_07_P014506	b1305	cmr|phage shock protein [b1305]
pspC	-15.95	10.57	-4.00	12.57	0.0	8.58	0.1	0.00000054	0.00005412	0.00003176	0.01785714	A_07_P014514	b1306	cmr|phage shock protein: activates phage shock-protein expression [b1306]
pspE	+5.59	11.49	+2.48	10.25	0.1	12.73	0.1	0.00001399	0.00030964	0.00802337	0.24964427	A_07_P014522	b1308	cmr|phage shock protein [b1308]
pspE	+2.87	11.25	+1.52	10.49	0.1	12.01	0.5	0.00401111	0.01905578	0.03042365	0.44765421	A_07_P014522	b1308	cmr|phage shock protein [b1308]
pspG	-2.02	9.84	-1.01	10.35	0.3	9.34	0.0	0.00699087	0.02965092	0.07249873	0.67024075	A_07_P048618	Z5648	cmr|orf, hypothetical protein [Z5648]
pssA	+2.92	9.30	+1.55	8.52	0.1	10.07	0.0	0.00014987	0.00161942	0.02828125	0.43781711	A_07_P019829	b2585	cmr|phosphatidylserine synthase; phospholipid synthesis [b2585]
pstA	-3.09	10.25	-1.63	11.06	0.1	9.44	0.2	0.00017086	0.00178857	0.01772739	0.38656510	A_07_P005555	b3726	cmr|high-affinity phosphate-specific transport system [b3726]
pstC	-4.40	10.20	-2.14	11.27	0.1	9.13	0.1	0.00002210	0.00041925	0.00489139	0.22784024	A_07_P005556	b3727	cmr|high-affinity phosphate-specific transport system, cytoplasmic membrane component [b3727]
pstS	-2.52	9.85	-1.34	10.52	0.1	9.18	0.1	0.00036762	0.00311361	0.03491870	0.51140465	A_07_P005561	b3728	cmr|high-affinity phosphate-specific transport system; periplasmic phosphate-binding protein [b3728]
pta	+13.72	10.84	+3.78	8.96	0.1	12.73	0.2	0.00000094	0.00007037	0.00127096	0.10994505	A_07_P042552	c_2840	cmr|Phosphate acetyltransferase [c_2840]
pta	+9.77	9.65	+3.29	8.01	0.3	11.30	0.2	0.00001276	0.00029390	0.00276042	0.15356890	A_07_P013771	b2297	cmr|phosphotransacetylase [b2297]
pth	+3.73	10.94	+1.90	9.99	0.1	11.89	0.0	0.00003536	0.00057430	0.01802147	0.35333748	A_07_P006971	b1204	cmr|peptidyl-tRNA hydrolase [b1204]
ptrB	-4.17	8.36	-2.06	9.39	0.1	7.33	0.2	0.00006854	0.00092303	0.00626016	0.25868766	A_07_P015904	b1845	cmr|protease II [b1845]
ptrB	-10.69	9.81	-3.42	11.52	0.0	8.10	0.1	0.00000093	0.00007024	0.00013084	0.03745455	A_07_P035322	c_2256	cmr|Protease II [c_2256]
ptrB	-19.35	10.01	-4.27	12.14	0.2	7.87	0.0	0.00000072	0.00006192	0.00002223	0.01458333	A_07_P052572	Z2896	cmr|protease II [Z2896]
ptsH	+2.24	11.54	+1.16	10.96	0.0	12.13	0.2	0.00100528	0.00654555	0.04914190	0.55145403	A_07_P013132	b2415	cmr|PTS system protein HPr [b2415]
ptsI	+8.99	13.15	+3.17	11.57	0.1	14.74	0.1	0.00000216	0.00010631	0.00318852	0.16677741	A_07_P013136	b2416	cmr|PEP-protein phosphotransferase system enzyme I [b2416]
ptsI	+7.73	13.57	+2.95	12.10	0.1	15.05	0.1	0.00000317	0.00013050	0.00412665	0.18616046	A_07_P013136	b2416	cmr|PEP-protein phosphotransferase system enzyme I [b2416]
ptsI	+7.73	12.67	+2.95	11.19	0.1	14.14	0.4	0.00004114	0.00063933	0.00423272	0.18824859	A_07_P013136	b2416	cmr|PEP-protein phosphotransferase system enzyme I [b2416]
ptsI	+7.79	13.65	+2.96	12.17	0.1	15.13	0.1	0.00000282	0.00012327	0.00404154	0.18337176	A_07_P013136	b2416	cmr|PEP-protein phosphotransferase system enzyme I [b2416]
ptsI	+7.34	13.60	+2.88	12.16	0.1	15.03	0.1	0.00000483	0.00016704	0.00452426	0.19251351	A_07_P013136	b2416	cmr|PEP-protein phosphotransferase system enzyme I [b2416]
ptsN	+2.82	11.28	+1.50	10.54	0.1	12.03	0.2	0.00038386	0.00322319	0.03097307	0.45068392	A_07_P012498	b3204	cmr|phosphotransferase system enzyme IIA, regulates N metabolism [b3204]
purA	-2.28	12.57	-1.19	13.16	0.1	11.97	0.5	0.00977948	0.03853055	0.04986408	0.58630321	A_07_P011601	b4177	cmr|adenylosuccinate synthetase [b4177]
purB	+2.14	11.12	+1.09	10.57	0.0	11.67	0.1	0.00112278	0.00708782	0.05442581	0.57087275	A_07_P006606	b1131	cmr|adenylosuccinate lyase [b1131]
purC	+19.20	10.29	+4.26	8.16	0.1	12.42	0.1	0.00000039	0.00004858	0.00057482	0.07125984	A_07_P013440	b2476	cmr|phosphoribosylaminoimidazole-succinocarboxamide synthetase = SAICAR synthetase [b2476]
purD	+19.98	10.12	+4.32	7.96	0.5	12.28	0.2	0.00000823	0.00022564	0.00046430	0.06194915	A_07_P008690	b4005	cmr|phosphoribosylglycinamide synthetase = GAR synthetase [b4005]
purE	+3.19	9.32	+1.67	8.49	0.3	10.16	0.3	0.00111264	0.00704458	0.02393356	0.41091603	A_07_P016404	b0523	cmr|phosphoribosylaminoimidazole carboxylase = AIR carboxylase, catalytic subunit [b0523]
purF	+3.20	8.66	+1.68	7.82	0.1	9.50	0.3	0.00045526	0.00364940	0.02399517	0.41063043	A_07_P013849	b2312	cmr|amidophosphoribosyltransferase = PRPP amidotransferase [b2312]
purF	+5.71	9.98	+2.51	8.72	0.1	11.24	0.0	0.00000620	0.00018975	0.00768356	0.24738241	A_07_P032426	c_2856	cmr|Amidophosphoribosyltransferase [c_2856]
purF	+3.49	8.87	+1.80	7.97	0.2	9.77	0.3	0.00065850	0.00473185	0.02014926	0.37321176	A_07_P042612	Z3574	cmr|amidophosphoribosyltransferase = PRPP amidotransferase [Z3574]
purH	+12.72	8.76	+3.67	6.93	0.1	10.60	0.2	0.00000110	0.00007696	0.00152630	0.11837438	A_07_P057962	ECs4929	cmr|phosphoribosylaminoimidazolecarboxamideformyltra nsferase [ECs4929]
purH	+34.86	10.25	+5.12	7.69	0.0	12.81	0.1	0.00000009	0.00003427	0.00008003	0.02377358	A_07_P037451	c_4964	cmr|Purine biosynthesis protein PurH [c_4964]
purH	+13.13	8.87	+3.72	7.02	0.1	10.73	0.1	0.00000069	0.00006127	0.00145516	0.11570707	A_07_P047211	Z5583	cmr|phosphoribosylaminoimidazolecarboxamide formyltransferase = AICAR formyltransferase IMP cyclohydrolase [Z5583]
purH	+13.21	8.97	+3.72	7.11	0.1	10.84	0.2	0.00000104	0.00007542	0.00143039	0.11548718	A_07_P008693	b4006	cmr|phosphoribosylaminoimidazolecarboxamideformyltransferase = AICAR formyltransferase; IMP cyclohydrolase [b4006]
purK	+2.96	9.10	+1.57	8.32	0.1	9.88	0.3	0.00067984	0.00483817	0.02800051	0.43432512	A_07_P016397	b0522	cmr|phosphoribosylaminoimidazole carboxylase = AIR carboxylase, CO [b0522]
purL	+7.18	9.03	+2.84	7.61	0.3	10.45	0.5	0.00015490	0.00166013	0.00506161	0.20752604	A_07_P019686	b2557	cmr|phosphoribosylformyl-glycineamide synthetase = FGAM synthetase [b2557]
purM	+22.95	11.32	+4.52	9.06	0.0	13.58	0.0	0.00000014	0.00003548	0.00033155	0.05272727	A_07_P013551	b2499	cmr|phosphoribosylaminoimidazole synthetase = AIR synthetase [b2499]
purN	+12.22	10.54	+3.61	8.73	0.1	12.35	0.0	0.00000094	0.00007037	0.00166349	0.12069124	A_07_P013559	b2500	cmr|phosphoribosylglycinamide formyltransferase 1 [b2500]
purN	+8.13	11.19	+3.02	9.68	0.1	12.70	0.0	0.00000203	0.00010341	0.00384146	0.18000000	A_07_P030133	c_3018	cmr|Phosphoribosylglycinamide formyltransferase [c_3018]
purT	+15.08	9.45	+3.91	7.49	0.0	11.41	0.0	0.00000036	0.00004756	0.00100483	0.09826087	A_07_P015921	b1849	cmr|phosphoribosylglycinamide formyltransferase 2 [b1849]
putA	+9.59	10.80	+3.26	9.17	0.2	12.43	0.1	0.00000341	0.00013521	0.00285950	0.15524138	A_07_P041092	c_1151	cmr|PutA protein [c_1151]
putA	+51.03	10.40	+5.67	7.57	0.2	13.24	0.2	0.00000016	0.00003548	0.00001842	0.01000000	A_07_P001304	b1014	cmr|proline dehydrogenase, P5C dehydrogenase [b1014]
putP	+8.47	10.12	+3.08	8.58	0.5	11.66	0.2	0.00007186	0.00095259	0.00355882	0.17346749	A_07_P001306	b1015	cmr|major sodium/proline symporter [b1015]
pykA	+8.51	10.68	+3.09	9.14	0.0	12.23	0.3	0.00000732	0.00020980	0.00357025	0.17295385	A_07_P015946	b1854	cmr|pyruvate kinase II, glucose stimulated [b1854]
pyrB	+17.76	10.13	+4.15	8.06	0.3	12.21	0.1	0.00000145	0.00009005	0.00070440	0.07865248	A_07_P003778	b4245	cmr|aspartate carbamoyltransferase, catalytic subunit [b4245]
pyrC	+4.97	8.80	+2.31	7.65	0.0	9.96	0.1	0.00001586	0.00033838	0.01030551	0.27974138	A_07_P001513	b1062	cmr|dihydro-orotase [b1062]
pyrD	+4.76	9.56	+2.25	8.43	0.1	10.69	0.1	0.00001553	0.00033216	0.01145960	0.29194175	A_07_P005175	b0945	cmr|dihydro-orotate dehydrogenase [b0945]
pyrF	+2.62	9.84	+1.39	9.14	0.1	10.53	0.0	0.00028365	0.00258732	0.03553036	0.47729522	A_07_P014388	b1281	cmr|orotidine-5-phosphate decarboxylase [b1281]
pyrG	+4.13	11.38	+2.05	10.36	0.1	12.40	0.2	0.00006227	0.00086079	0.01498476	0.33042017	A_07_P002348	b2780	cmr|CTP synthetase [b2780]
pyrH	-2.60	12.44	-1.38	13.13	0.0	11.75	0.2	0.00051155	0.00395777	0.03158092	0.49770771	A_07_P002814	b0171	cmr|uridylate kinase [b0171]
pyrI	+11.22	9.43	+3.49	7.68	0.1	11.17	0.1	0.00000093	0.00007006	0.00201791	0.13182573	A_07_P003774	b4244	cmr|aspartate carbamoyltransferase, regulatory subunit [b4244]
qor	+8.46	9.33	+3.08	7.79	0.1	10.87	0.1	0.00000217	0.00010631	0.00351435	0.17183230	A_07_P008897	b4051	cmr|quinone oxidoreductase [b4051]
quuQ	-2.43	8.79	-1.28	9.43	0.2	8.15	0.1	0.00149856	0.00878057	0.04019690	0.54276158	A_07_P020436	b1559	cmr|orf, hypothetical protein [b1559]
raiA	+45.75	14.18	+5.52	11.42	0.1	16.94	0.0	0.00000006	0.00003344	0.00003239	0.01416667	A_07_P019869	b2597	cmr|putative yhbH sigma 54 modulator [b2597]
rarA	+2.12	10.43	+1.08	9.89	0.1	10.97	0.2	0.00175516	0.00998282	0.05689977	0.58436399	A_07_P004906	b0892	cmr|putative polynucleotide enzyme [b0892]
rarD	-2.32	9.16	-1.21	9.77	0.1	8.55	0.1	0.00060996	0.00447491	0.04692835	0.57542056	A_07_P005965	b3819	cmr|orf, hypothetical protein [b3819]
ravA	-2.15	9.07	-1.10	9.62	0.0	8.52	0.5	0.01262704	0.04743502	0.05995363	0.62885410	A_07_P005654	b3746	cmr|putative 2-component regulator [b3746]
rbbA	+3.64	9.08	+1.86	8.15	0.1	10.02	0.2	0.00015212	0.00163810	0.01890561	0.36035109	A_07_P007367	b3486	cmr|putative ATP-binding component of a transport system, fragment 1 [b3486]
rbfA	-4.80	12.61	-2.26	13.74	0.0	11.48	0.4	0.00010938	0.00127906	0.00382368	0.20476190	A_07_P000559	b3167	cmr|ribosome-binding factor A [b3167]
rbn	+6.17	9.62	+2.63	8.31	0.1	10.94	0.1	0.00001016	0.00025323	0.00651931	0.23169300	A_07_P039358	c_4833	cmr|Ribonuclease BN [c_4833]
rbsB	+6.26	14.62	+2.65	13.29	0.0	15.94	0.3	0.00003582	0.00057956	0.00637957	0.22931507	A_07_P005677	b3751	cmr|D-ribose periplasmic binding protein [b3751]
rbsC	+11.14	10.49	+3.48	8.75	0.0	12.23	0.0	0.00000076	0.00006301	0.00204840	0.13217213	A_07_P005671	b3750	cmr|D-ribose high-affinity transport system [b3750]
rbsK	+2.62	10.58	+1.39	9.88	0.0	11.27	0.1	0.00025431	0.00237897	0.03516959	0.47733621	A_07_P005685	b3752	cmr|ribokinase [b3752]
rbsR	+2.25	10.19	+1.17	9.60	0.0	10.77	0.2	0.00163043	0.00941651	0.04948488	0.55411807	A_07_P005686	b3753	cmr|regulator for rbs operon [b3753]
rcnA	-3.63	7.93	-1.86	8.86	0.7	7.00	0.0	0.00434822	0.02038663	0.01021278	0.32482828	A_07_P003293	b2106	cmr|orf, hypothetical protein [b2106]
rcsB	+2.29	10.30	+1.19	9.70	0.0	10.90	0.1	0.00059134	0.00438119	0.04680323	0.53473875	A_07_P032365	c_2760	cmr|Capsular synthesis regulator component B [c_2760]
rcsD	+4.54	9.66	+2.18	8.57	0.2	10.75	0.4	0.00035666	0.00303529	0.01213161	0.29797192	A_07_P000870	b2216	cmr|putative 2-component sensor protein [b2216]
rcsF	+2.19	11.18	+1.13	10.61	0.1	11.74	0.1	0.00113462	0.00713681	0.05244474	0.56438141	A_07_P041460	c_0237	cmr|Protein rcsF [c_0237]
rdgC	+2.66	10.00	+1.41	9.29	0.3	10.71	0.1	0.00095780	0.00632537	0.03521850	0.47717728	A_07_P021080	b0393	cmr|orf, hypothetical protein [b0393]
rdoA	+3.92	10.71	+1.97	9.73	0.1	11.70	0.1	0.00003202	0.00053978	0.01662030	0.34521108	A_07_P019079	b3859	cmr|orf, hypothetical protein [b3859]
recA	-4.14	14.52	-2.05	15.55	0.0	13.50	0.5	0.00051398	0.00396851	0.00664761	0.26362720	A_07_P001941	b2699	cmr|DNA strand exchange and renaturation, DNA-dependent ATPase, DNA- and ATP-dependent coprotease [b2699]
recF	-3.20	11.26	-1.68	12.10	0.0	10.42	0.1	0.00008709	0.00108651	0.01578951	0.37325826	A_07_P005432	b3700	cmr|ssDNA and dsDNA binding, ATP binding [b3700]
recJ	-3.14	9.08	-1.65	9.90	0.1	8.25	0.1	0.00010879	0.00127349	0.01697917	0.37971591	A_07_P045645	Z4230	cmr|ssDNA exonuclease, 5 --> 3 specific [Z4230]
recJ	-2.71	8.32	-1.44	9.04	0.1	7.60	0.2	0.00047814	0.00378674	0.02869284	0.47853814	A_07_P006426	b2892	cmr|ssDNA exonuclease, 5 --> 3 specific [b2892]
recJ	-4.63	11.05	-2.21	12.16	0.0	9.95	0.0	0.00001288	0.00029471	0.00404090	0.20859016	A_07_P032859	c_3472	cmr|Single-stranded-DNA-specific exonuclease recJ [c_3472]
recR	+6.91	11.71	+2.79	10.32	0.1	13.11	0.2	0.00000900	0.00023748	0.00515180	0.20797436	A_07_P021466	b0472	cmr|recombination and repair [b0472]
recX	-7.85	10.95	-2.97	12.44	0.0	9.46	0.3	0.00001323	0.00029969	0.00059388	0.07727273	A_07_P063405	ECs3555	cmr|regulatory protein OraA [ECs3555]
recX	-8.17	11.18	-3.03	12.69	0.0	9.66	0.4	0.00002400	0.00044341	0.00049606	0.07036036	A_07_P001938	b2698	cmr|regulator, OraA protein [b2698]
recX	-3.05	10.76	-1.61	11.56	0.1	9.96	0.2	0.00027261	0.00250551	0.01851181	0.39120805	A_07_P052735	Z4001	cmr|regulator, OraA protein [Z4001]
rem	-4.01	10.16	-2.00	11.16	0.5	9.16	0.0	0.00079976	0.00550019	0.00726054	0.27281623	A_07_P020446	b1561	cmr|orf, hypothetical protein [b1561]
rep	+2.91	8.92	+1.54	8.15	0.1	9.69	0.0	0.00013818	0.00152138	0.02859439	0.43878168	A_07_P005771	b3778	cmr|rep helicase, a single-stranded DNA dependent ATPase [b3778]
rfaH	-3.71	9.27	-1.89	10.22	0.0	8.33	0.1	0.00004565	0.00068845	0.00941374	0.31071279	A_07_P019019	b3842	cmr|transcriptional activator affecting biosynthesis of lipopolysaccharide core, F pilin, and haemolysin [b3842]
rfaI	-10.33	12.34	-3.37	14.03	0.0	10.66	0.1	0.00000124	0.00008213	0.00016133	0.04096774	A_07_P039805	c_4453	cmr|Lipopolysaccharide 1,3-galactosyltransferase [c_4453]
rfaP	-2.65	12.12	-1.41	12.83	0.0	11.42	0.2	0.00035602	0.00303253	0.02970084	0.48406832	A_07_P039808	c_4454	cmr|Lipopolysaccharide core biosynthesis protein rfaP [c_4454]
rfaP	-3.14	12.37	-1.65	13.20	0.2	11.55	0.1	0.00024154	0.00228676	0.01664444	0.37759366	A_07_P039808	c_4454	cmr|Lipopolysaccharide core biosynthesis protein rfaP [c_4454]
rfaP	-4.27	11.75	-2.09	12.80	0.0	10.70	0.5	0.00081269	0.00557469	0.00617124	0.25703704	A_07_P039808	c_4454	cmr|Lipopolysaccharide core biosynthesis protein rfaP [c_4454]
rfaP	-3.01	12.43	-1.59	13.22	0.0	11.63	0.2	0.00015835	0.00168912	0.01942899	0.39881356	A_07_P039808	c_4454	cmr|Lipopolysaccharide core biosynthesis protein rfaP [c_4454]
rfaP	-2.73	12.30	-1.45	13.02	0.0	11.57	0.2	0.00037146	0.00313916	0.02697599	0.47190000	A_07_P039808	c_4454	cmr|Lipopolysaccharide core biosynthesis protein rfaP [c_4454]
rffA	+2.91	8.46	+1.54	7.69	0.1	9.23	0.1	0.00019216	0.00194432	0.02869919	0.43953307	A_07_P005832	b3791	cmr|putative regulator [b3791]
rffC	+2.66	8.02	+1.41	7.32	0.4	8.73	0.2	0.00315912	0.01576954	0.03612741	0.47999156	A_07_P005826	b3790	cmr|orf, hypothetical protein [b3790]
rffD	+2.15	7.82	+1.11	7.27	0.1	8.38	0.1	0.00094599	0.00626098	0.05372777	0.56923957	A_07_P005811	b3787	cmr|UDP-N-acetyl-D-mannosaminuronic acid dehydrogenase; synthesis of enterobacterial common antigen [b3787]
rffH	+2.22	7.46	+1.15	6.89	0.0	8.04	0.0	0.00063644	0.00461448	0.04996253	0.55748405	A_07_P005823	b3789	cmr|glucose-1-phosphate thymidylyltransferase [b3789]
rffM	+2.95	7.87	+1.56	7.09	0.1	8.65	0.1	0.00013158	0.00146726	0.02770135	0.43309831	A_07_P005860	b3794	cmr|probable UDP-N-acetyl-D-mannosaminuronic acid transferase; synthesis of enterobacterial common antigen [b3794]
rhaS	+2.90	7.58	+1.53	6.81	0.3	8.34	0.4	0.00290130	0.01477774	0.03066184	0.44864312	A_07_P019305	b3905	cmr|positive regulator for rhaBAD operon [b3905]
rhlB	+3.12	9.13	+1.64	8.31	0.0	9.95	0.3	0.00032392	0.00282385	0.02539825	0.42314286	A_07_P005782	b3780	cmr|putative ATP-dependent RNA helicase [b3780]
rho	-2.05	10.57	-1.03	11.09	0.1	10.05	0.1	0.00187689	0.01049353	0.06839685	0.65741148	A_07_P005791	b3783	cmr|transcription termination factor Rho; polarity suppressor [b3783]
rhsC	-2.99	7.84	-1.58	8.63	0.2	7.05	0.2	0.00047170	0.00375069	0.02029154	0.40905250	A_07_P010017	b0700	cmr|rhsC protein in rhs element [b0700]
ribD	-3.76	10.71	-1.91	11.67	0.0	9.76	0.3	0.00027421	0.00251881	0.00908283	0.30620985	A_07_P021185	b0414	cmr|bifunctional pyrimidine deaminase/reductase in pathway of riboflavin synthesis [b0414]
ribE	-3.93	12.91	-1.97	13.90	0.0	11.92	0.2	0.00005587	0.00079530	0.00733041	0.27348341	A_07_P021187	b0415	cmr|riboflavin synthase, beta chain [b0415]
ribE	-4.05	13.03	-2.02	14.04	0.0	12.03	0.1	0.00003326	0.00055288	0.00671049	0.26412500	A_07_P021187	b0415	cmr|riboflavin synthase, beta chain [b0415]
ribE	-4.40	12.78	-2.14	13.85	0.0	11.71	0.1	0.00001797	0.00036652	0.00481707	0.22706587	A_07_P021187	b0415	cmr|riboflavin synthase, beta chain [b0415]
ribE	-4.97	12.62	-2.31	13.77	0.0	11.46	0.0	0.00000930	0.00024121	0.00318534	0.19141221	A_07_P021187	b0415	cmr|riboflavin synthase, beta chain [b0415]
ribE	-4.78	12.72	-2.26	13.85	0.0	11.59	0.0	0.00001056	0.00026108	0.00354675	0.19731449	A_07_P021187	b0415	cmr|riboflavin synthase, beta chain [b0415]
rihB	+2.02	7.86	+1.02	7.35	0.1	8.37	0.0	0.00124199	0.00763763	0.06093115	0.60144201	A_07_P053101	ECs3054	cmr|hypothetical protein [ECs3054]
rimI	-3.01	11.46	-1.59	12.26	0.0	10.67	0.1	0.00014453	0.00156823	0.01936547	0.39959371	A_07_P017550	b4373	cmr|acyltransferase for 30S ribosomal subunit protein S18; acetylation of N-terminal alanine [b4373]
rimJ	+4.87	10.71	+2.29	9.56	0.1	11.85	0.0	0.00001603	0.00033988	0.01063326	0.28326565	A_07_P001534	b1066	cmr|acetylation of N-terminal alanine of 30S ribosomal subunit protein S5 [b1066]
rimM	-3.09	16.47	-1.63	17.28	0.0	15.66	0.8	0.01343602	0.04994015	0.01867759	0.39365462	A_07_P019918	b2608	cmr|orf, hypothetical protein [b2608]
rlmL	+2.65	8.48	+1.40	7.78	0.2	9.18	0.2	0.00057352	0.00429564	0.03459223	0.47276042	A_07_P005186	b0948	cmr|putative oxidoreductase [b0948]
rlpA	+2.94	9.92	+1.56	9.14	0.1	10.70	0.1	0.00017165	0.00179445	0.02786458	0.43349802	A_07_P009735	b0633	cmr|a minor lipoprotein [b0633]
rlpB	-3.51	10.13	-1.81	11.03	0.1	9.22	0.3	0.00038768	0.00324823	0.01170732	0.33512727	A_07_P044023	Z0788	cmr|a minor lipoprotein [Z0788]
rlpB	-3.17	12.46	-1.67	13.30	0.1	11.63	0.2	0.00014225	0.00155099	0.01607978	0.37672619	A_07_P009773	b0641	cmr|a minor lipoprotein [b0641]
rluA	-3.62	9.47	-1.86	10.39	0.2	8.54	0.0	0.00010337	0.00123197	0.01018864	0.32537525	A_07_P010976	b0058	cmr|orf, hypothetical protein [b0058]
rluD	-3.76	10.22	-1.91	11.18	0.3	9.27	0.1	0.00014184	0.00155076	0.00886878	0.30222944	A_07_P042940	Z3888	cmr|suppressor of ftsH mutation [Z3888]
rluD	-2.57	8.45	-1.36	9.13	0.0	7.77	0.2	0.00069190	0.00488925	0.03382558	0.50815840	A_07_P019852	b2594	cmr|suppressor of ftsH mutation [b2594]
rluD	-3.61	10.11	-1.85	11.04	0.2	9.19	0.1	0.00008626	0.00108122	0.01023628	0.32426559	A_07_P053505	ECs3457	cmr|suppressor of ftsH mutation [ECs3457]
rluE	-3.31	11.01	-1.73	11.88	0.0	10.15	0.1	0.00007213	0.00095356	0.01398247	0.35563813	A_07_P006629	b1135	cmr|orf, hypothetical protein [b1135]
rmf	+60.47	11.76	+5.92	8.80	0.1	14.72	0.0	0.00000003	0.00003344	0.00001143	0.00900000	A_07_P005212	b0953	cmr|ribosome modulation factor [b0953]
rmuC	+2.32	11.97	+1.21	11.36	0.1	12.58	0.0	0.00058657	0.00435609	0.04569995	0.52943341	A_07_P018970	b3832	cmr|putative alpha helix chain [b3832]
rnc	-3.58	11.99	-1.84	12.91	0.0	11.07	0.1	0.00005045	0.00074433	0.01030488	0.32383234	A_07_P019740	b2567	cmr|RNase III, ds RNA [b2567]
rne	+4.27	8.49	+2.09	7.45	0.1	9.54	0.2	0.00003857	0.00061155	0.01405805	0.32076812	A_07_P001621	b1084	cmr|RNase E, membrane attachment, mRNA turnover, maturation 5S RNA [b1084]
rnhB	-5.14	11.00	-2.36	12.18	0.1	9.82	0.1	0.00001426	0.00031323	0.00284680	0.17645669	A_07_P002875	b0183	cmr|RNase HII, degrades RNA of DNA-RNA hybrids [b0183]
rnk	+4.85	10.09	+2.28	8.95	0.2	11.23	0.1	0.00004096	0.00063841	0.01080666	0.28451505	A_07_P016807	b0610	cmr|regulator of nucleoside diphosphate kinase [b0610]
rnpA	-2.18	14.34	-1.12	14.90	0.0	13.78	0.1	0.00104400	0.00672810	0.05627858	0.61318339	A_07_P005448	b3704	cmr|RNase P, protein component; protein C5; processes tRNA, 4.5S RNA [b3704]
rob	+2.89	11.89	+1.53	11.12	0.1	12.65	0.1	0.00017548	0.00182122	0.02894563	0.43988417	A_07_P017663	b4396	cmr|right origin-binding protein [b4396]
rpe	+5.87	10.44	+2.55	9.16	0.1	11.72	0.1	0.00001205	0.00028605	0.00734566	0.24450317	A_07_P017959	b3386	cmr|D-ribulose-5-phosphate 3-epimerase [b3386]
rpiR	+2.51	9.02	+1.33	8.36	0.2	9.68	0.1	0.00065115	0.00468757	0.03910569	0.49372895	A_07_P011184	b4089	cmr|transcriptional repressor of rpiB expression [b4089]
rpiR	+16.84	9.72	+4.07	7.68	0.1	11.76	0.1	0.00000052	0.00005300	0.00079078	0.08355705	A_07_P037745	c_5095	cmr|Protein rpiR [c_5095]
rplA	+2.20	16.00	+1.14	15.43	0.1	16.57	0.1	0.00073883	0.00514923	0.05110328	0.55911744	A_07_P008585	b3984	cmr|50S ribosomal subunit protein L1, regulates synthesis of L1 and L11 [b3984]
rplB	+2.66	16.96	+1.41	16.26	0.0	17.67	0.2	0.00033975	0.00292453	0.03410887	0.46982502	A_07_P013013	b3317	cmr|50S ribosomal subunit protein L2 [b3317]
rplF	-2.04	16.11	-1.03	16.62	0.1	15.60	0.0	0.00133030	0.00802484	0.07021278	0.66153800	A_07_P012955	b3305	cmr|50S ribosomal subunit protein L6 [b3305]
rplI	-2.93	14.93	-1.55	15.71	0.0	14.16	0.4	0.00103019	0.00666690	0.02065295	0.41316391	A_07_P011735	b4203	cmr|50S ribosomal subunit protein L9 [b4203]
rplN	+2.55	16.34	+1.35	15.66	0.0	17.02	0.1	0.00025724	0.00239644	0.03697282	0.48508333	A_07_P012976	b3310	cmr|50S ribosomal subunit protein L14 [b3310]
rplR	-2.09	16.53	-1.06	17.06	0.1	16.00	0.0	0.00097149	0.00639429	0.06497777	0.64870640	A_07_P012950	b3304	cmr|50S ribosomal subunit protein L18 [b3304]
rplS	-11.83	12.42	-3.56	14.21	0.0	10.64	0.6	0.00005510	0.00078788	0.00010798	0.03617021	A_07_P019906	b2606	cmr|50S ribosomal subunit protein L19 [b2606]
rplT	-5.18	13.70	-2.37	14.89	0.1	12.52	0.0	0.00000870	0.00023173	0.00282584	0.17584980	A_07_P018627	b1716	cmr|50S ribosomal subunit protein L20, and regulator [b1716]
rplY	+2.99	14.23	+1.58	13.44	0.1	15.02	0.0	0.00011437	0.00132013	0.02679815	0.42790061	A_07_P000722	b2185	cmr|50S ribosomal subunit protein L25 [b2185]
rplY	+3.58	14.14	+1.84	13.22	0.1	15.06	0.2	0.00010850	0.00127193	0.01963986	0.36810714	A_07_P035511	c_2722	cmr|50S ribosomal protein L25 [c_2722]
rpmC	-2.17	16.53	-1.12	17.09	0.0	15.97	0.1	0.00084138	0.00573949	0.05703760	0.61675824	A_07_P012987	b3312	cmr|50S ribosomal subunit protein L29 [b3312]
rpmE	-4.92	15.25	-2.30	16.40	0.0	14.10	0.1	0.00001455	0.00031648	0.00320948	0.18925094	A_07_P019460	b3936	cmr|50S ribosomal subunit protein L31 [b3936]
rpmG	-3.03	14.18	-1.60	14.98	0.0	13.38	0.1	0.00010041	0.00120402	0.01915142	0.39725955	A_07_P017122	b3636	cmr|50S ribosomal subunit protein L33 [b3636]
rpoA	-2.05	15.19	-1.03	15.70	0.0	14.67	0.2	0.00202841	0.01117402	0.06883511	0.65721043	A_07_P012909	b3295	cmr|RNA polymerase, alpha subunit [b3295]
rpoB	+4.81	10.93	+2.27	9.80	0.0	12.07	0.2	0.00003432	0.00056336	0.01109756	0.28736842	A_07_P008600	b3987	cmr|RNA polymerase, beta subunit [b3987]
rpoC	+2.14	10.54	+1.10	9.99	0.1	11.09	0.5	0.01133088	0.04337307	0.05720719	0.58409209	A_07_P008605	b3988	cmr|RNA polymerase, beta prime subunit [b3988]
rpoD	-4.09	14.58	-2.03	15.60	0.0	13.57	0.2	0.00003947	0.00062195	0.00632685	0.25672680	A_07_P000070	b3067	cmr|RNA polymerase, sigma [b3067]
rpoE	-4.18	12.24	-2.06	13.28	0.0	11.21	0.1	0.00002311	0.00043163	0.00590892	0.25211382	A_07_P019770	b2573	cmr|RNA polymerase, sigma-E factor; heat shock and oxidative stress [b2573]
rpoH	-2.56	11.97	-1.36	12.64	0.0	11.29	0.1	0.00026460	0.00244765	0.03378938	0.50809933	A_07_P007245	b3461	cmr|RNA polymerase, sigma [b3461]
rpoN	+3.18	10.07	+1.67	9.24	0.0	10.91	0.2	0.00013307	0.00148058	0.02448361	0.41403867	A_07_P012490	b3202	cmr|RNA polymerase, sigma [b3202]
rpoZ	-4.94	13.52	-2.30	14.68	0.0	12.37	0.0	0.00000978	0.00024876	0.00320058	0.19087121	A_07_P017187	b3649	cmr|RNA polymerase, omega subunit [b3649]
rppH	-3.37	12.48	-1.75	13.35	0.1	11.60	0.1	0.00009200	0.00112981	0.01313580	0.34583612	A_07_P006135	b2830	cmr|putative invasion protein [b2830]
rpsA	-4.26	14.18	-2.09	15.23	0.0	13.14	0.7	0.00199627	0.01103557	0.00642340	0.25732824	A_07_P005002	b0911	cmr|30S ribosomal subunit protein S1 [b0911]
rpsB	-3.08	14.51	-1.62	15.33	0.0	13.70	0.0	0.00008769	0.00109056	0.01791476	0.38636986	A_07_P002803	b0169	cmr|30S ribosomal subunit protein S2 [b0169]
rpsC	-3.44	16.43	-1.78	17.32	0.0	15.54	0.2	0.00010963	0.00128044	0.01241171	0.33749568	A_07_P012998	b3314	cmr|30S ribosomal subunit protein S3 [b3314]
rpsG	-5.00	15.72	-2.32	16.88	0.0	14.56	1.0	0.00632563	0.02744301	0.00354230	0.19776596	A_07_P017731	b3341	cmr|30S ribosomal subunit protein S7, initiates assembly [b3341]
rpsI	-2.77	13.74	-1.47	14.47	0.1	13.00	0.1	0.00019513	0.00196595	0.02609121	0.46573696	A_07_P012625	b3230	cmr|30S ribosomal subunit protein S9 [b3230]
rpsL	-2.12	16.34	-1.08	16.88	0.1	15.80	0.3	0.00495608	0.02262353	0.06281822	0.64138132	A_07_P017738	b3342	cmr|30S ribosomal subunit protein S12 [b3342]
rpsO	+2.54	13.22	+1.35	12.55	0.1	13.89	0.4	0.00220379	0.01192730	0.03817327	0.48742903	A_07_P000550	b3165	cmr|30S ribosomal subunit protein S15 [b3165]
rpsQ	-4.51	15.75	-2.17	16.84	0.0	14.67	0.0	0.00001359	0.00030481	0.00440422	0.21805031	A_07_P012981	b3311	cmr|30S ribosomal subunit protein S17 [b3311]
rpsT	-4.56	13.54	-2.19	14.64	0.0	12.45	0.1	0.00001491	0.00032253	0.00429052	0.21512739	A_07_P010803	b0023	cmr|30S ribosomal subunit protein S20 [b0023]
rraA	+2.55	10.26	+1.35	9.59	0.1	10.94	0.2	0.00083930	0.00573025	0.03735201	0.48681291	A_07_P019421	b3929	cmr|menaquinone biosynthesis, unknown [b3929]
rrmJ	-3.16	12.17	-1.66	13.00	0.0	11.34	0.1	0.00009208	0.00112998	0.01650534	0.37715530	A_07_P000609	b3179	cmr|cell division protein [b3179]
rsd	+6.42	10.53	+2.68	9.19	0.1	11.87	0.1	0.00000572	0.00018115	0.00608168	0.22529412	A_07_P008637	b3995	cmr|putative transcriptional regulator [b3995]
rseA	-5.42	13.33	-2.44	14.55	0.0	12.11	0.3	0.00004438	0.00067775	0.00246888	0.16754310	A_07_P019761	b2572	cmr|sigma-E factor, negative regulatory protein [b2572]
rseB	-3.32	9.73	-1.73	10.60	0.2	8.87	0.6	0.00342159	0.01677132	0.01442264	0.35928797	A_07_P019758	b2571	cmr|regulates activity of sigma-E factor [b2571]
rseP	-2.04	12.23	-1.03	12.74	0.0	11.71	0.2	0.00325090	0.01608894	0.06844639	0.65708537	A_07_P002836	b0176	cmr|orf, hypothetical protein [b0176]
rsmE	+3.03	10.94	+1.60	10.14	0.1	11.74	0.4	0.00110194	0.00699554	0.02692073	0.42768920	A_07_P011872	b2946	cmr|orf, hypothetical protein [b2946]
rstB	-2.48	8.73	-1.31	9.39	0.1	8.08	0.1	0.00071197	0.00498854	0.03795478	0.52928255	A_07_P020676	b1609	cmr|sensor histidine protein kinase [b1609]
rsuA	-4.17	11.98	-2.06	13.01	0.1	10.95	0.1	0.00002345	0.00043689	0.00599530	0.25305630	A_07_P000715	b2183	cmr|16S pseudouridylate 516 synthase [b2183]
rsxA	-19.01	10.75	-4.25	12.88	0.0	8.63	0.1	0.00000028	0.00004211	0.00001715	0.01173913	A_07_P020768	b1627	cmr|orf, hypothetical protein [b1627]
rsxB	-9.89	10.08	-3.31	11.73	0.0	8.42	0.1	0.00000112	0.00007777	0.00020897	0.04633803	A_07_P020774	b1628	cmr|orf, hypothetical protein [b1628]
rsxC	-4.17	9.73	-2.06	10.76	0.0	8.70	0.1	0.00003034	0.00052027	0.00589812	0.25233696	A_07_P020776	b1629	cmr|putative membrane protein [b1629]
rsxD	-2.32	8.17	-1.21	8.78	0.1	7.56	0.1	0.00063261	0.00460251	0.04680259	0.57522248	A_07_P020785	b1630	cmr|orf, hypothetical protein [b1630]
rsxG	-2.02	8.88	-1.02	9.39	0.0	8.37	0.3	0.00451920	0.02103170	0.07205602	0.66810954	A_07_P020789	b1631	cmr|orf, hypothetical protein [b1631]
rtcA	-2.24	9.06	-1.16	9.64	0.4	8.48	0.1	0.00510611	0.02317029	0.05026550	0.58620741	A_07_P018126	b3420	cmr|RNA 3-terminal phosphate cyclase [b3420]
rtcB	-2.53	11.46	-1.34	12.13	0.1	10.79	0.1	0.00048473	0.00382331	0.03472434	0.51237113	A_07_P018134	b3421	cmr|orf, hypothetical protein [b3421]
rumB	-2.29	9.15	-1.19	9.75	0.1	8.56	0.3	0.00219433	0.01188425	0.04935467	0.58512048	A_07_P056960	ECs0939	cmr|putative enzyme [ECs0939]
rusA	+41.33	9.57	+5.37	6.89	0.0	12.26	0.1	0.00000008	0.00003427	0.00005208	0.02050000	A_07_P016520	b0550	cmr|endodeoxyribonuclease RUS [b0550]
rutB	-4.79	10.65	-2.26	11.78	0.0	9.52	0.2	0.00003700	0.00059268	0.00376397	0.20434483	A_07_P001287	b1011	cmr|putative synthetase [b1011]
rutF	-2.32	8.26	-1.22	8.86	0.4	7.65	0.1	0.00363205	0.01763275	0.04659235	0.57488245	A_07_P001269	b1007	cmr|orf, hypothetical protein [b1007]
rutR	-2.55	9.27	-1.35	9.95	0.1	8.60	0.1	0.00031235	0.00275682	0.03415777	0.51119772	A_07_P001300	b1013	cmr|putative tet operon regulator [b1013]
ruvA	-2.00	12.24	-1.00	12.74	0.0	11.74	0.1	0.00142969	0.00845252	0.07451537	0.67617867	A_07_P015985	b1861	cmr|Holliday junction helicase subunit B; branch migration; repair [b1861]
ruvB	-2.47	11.66	-1.30	12.32	0.0	11.01	0.1	0.00037777	0.00318394	0.03808562	0.52783451	A_07_P015980	b1860	cmr|Holliday junction helicase subunit A; branch migration; repair [b1860]
rzpD	+16.68	10.98	+4.06	8.95	0.4	13.01	0.1	0.00000752	0.00021366	0.00077045	0.08308219	A_07_P010301	b0556	cmr|bacteriophage lambda endopeptidase homolog [b0556]
rzpD	+10.83	9.68	+3.44	7.96	0.2	11.40	0.1	0.00000289	0.00012407	0.00219258	0.13644269	A_07_P016543	b0556	cmr|bacteriophage lambda endopeptidase homolog [b0556]
sad	-2.74	9.29	-1.46	10.02	0.2	8.56	0.0	0.00057834	0.00431741	0.02693852	0.47176863	A_07_P020272	b1525	cmr|putative aldehyde dehydrogenase [b1525]
sapA	-2.02	9.91	-1.01	10.41	0.0	9.40	0.1	0.00185079	0.01039559	0.07255462	0.66957796	A_07_P014455	b1294	cmr|homolog of Salmonella peptide transport periplasmic protein [b1294]
sapD	+2.03	8.09	+1.02	7.58	0.0	8.60	0.1	0.00169026	0.00967148	0.06213542	0.60648481	A_07_P014440	b1291	cmr|putative ATP-binding protein of peptide transport system [b1291]
sat	-9.96	10.51	-3.32	12.17	0.1	8.86	0.0	0.00000105	0.00007542	0.00020833	0.04685714	A_07_P036297	c_3619	cmr|Aecreted auto transpoter toxin [c_3619]
sbcB	+2.29	8.87	+1.20	8.27	0.2	9.47	0.2	0.00268838	0.01388190	0.04799860	0.54366187	A_07_P044967	Z3173	cmr|exonuclease I, 3 --> 5 specific deoxyribophosphodiesterase [Z3173]
sbcB	+2.03	9.14	+1.02	8.63	0.1	9.65	0.0	0.00131469	0.00796098	0.06073996	0.60106223	A_07_P055542	ECs2813	cmr|exonuclease I [ECs2813]

sbcB	+2.55	10.41	+1.35	9.74	0.0	11.09	0.0	0.00025176	0.00235792	0.03719830	0.48601660	A_07_P031971	c_2539	cmr|Exodeoxyribonuclease I [c_2539]
sbmA	-2.59	9.34	-1.37	10.03	0.1	8.65	0.2	0.00051214	0.00396018	0.03234057	0.50363007	A_07_P020999	b0377	cmr|sensitivity to microcin B17, possibly envelop protein [b0377]
sbp	-6.51	10.95	-2.70	12.30	0.1	9.59	0.1	0.00000612	0.00018856	0.00125127	0.11588235	A_07_P019364	b3917	cmr|periplasmic sulfate-binding protein [b3917]
sdaA	+2.44	10.49	+1.29	9.85	0.1	11.14	0.1	0.00037513	0.00316336	0.04077236	0.49993769	A_07_P015761	b1814	cmr|L-serine deaminase [b1814]
sdaC	+2.56	11.48	+1.35	10.80	0.1	12.16	0.2	0.00044088	0.00356882	0.03725038	0.48629353	A_07_P002426	b2796	cmr|probable serine transporter [b2796]
sdhA	+8.76	11.58	+3.13	10.01	0.1	13.14	0.3	0.00001344	0.00030273	0.00336636	0.16878981	A_07_P010135	b0723	cmr|succinate dehydrogenase, flavoprotein subunit [b0723]
sdhA	+11.54	10.17	+3.53	8.40	0.2	11.93	0.1	0.00000195	0.00010176	0.00190612	0.12935345	A_07_P051706	Z0877	cmr|succinate dehydrogenase, flavoprotein subunit [Z0877]
sdhA	+10.66	10.27	+3.41	8.57	0.0	11.98	0.2	0.00000245	0.00011451	0.00220274	0.13653543	A_07_P063016	ECs0748	cmr|succinate dehydrogenase flavoprotein subunit [ECs0748]
sdhB	+7.05	11.73	+2.82	10.32	0.1	13.13	0.1	0.00000616	0.00018932	0.00501080	0.20925729	A_07_P010139	b0724	cmr|succinate dehydrogenase, iron sulfur protein [b0724]
sdhC	+49.31	12.62	+5.62	9.81	0.1	15.43	0.1	0.00000006	0.00003344	0.00002414	0.01151515	A_07_P010122	b0721	cmr|succinate dehydrogenase, cytochrome b556 [b0721]
sdhD	+27.43	12.30	+4.78	9.91	0.1	14.68	0.0	0.00000013	0.00003548	0.00018483	0.03730769	A_07_P010127	b0722	cmr|succinate dehydrogenase, hydrophobic subunit [b0722]
sdiA	-3.49	10.26	-1.80	11.16	0.1	9.35	0.2	0.00012925	0.00144554	0.01143674	0.33160221	A_07_P014943	b1916	cmr|transcriptional regulator of ftsQAZ gene cluster [b1916]
sdiA	-4.40	8.24	-2.14	9.31	0.1	7.17	0.2	0.00003408	0.00056193	0.00497332	0.22961877	A_07_P035369	c_2330	cmr|Regulatory protein sdiA [c_2330]
secA	+2.96	9.07	+1.57	8.29	0.0	9.85	0.1	0.00014092	0.00154399	0.02752541	0.43206381	A_07_P002461	b0098	cmr|preprotein translocase; secretion protein [b0098]
secB	+7.92	11.52	+2.99	10.03	0.1	13.01	0.2	0.00000376	0.00014286	0.00394754	0.18119534	A_07_P016988	b3609	cmr|protein export; molecular chaperone; may bind to signel sequence [b3609]
secE	-4.07	13.62	-2.03	14.63	0.0	12.61	0.2	0.00004119	0.00063960	0.00637386	0.25730769	A_07_P008570	b3981	cmr|preprotein translocase [b3981]
secG	-2.04	11.90	-1.03	12.41	0.1	11.38	0.1	0.00154655	0.00900474	0.06928417	0.65790712	A_07_P000590	b3175	cmr|protein export - membrane protein [b3175]
secY	+2.65	15.08	+1.41	14.38	0.1	15.79	0.2	0.00055617	0.00419969	0.03464685	0.47309627	A_07_P012927	b3300	cmr|putative ATPase subunit of translocase [b3300]
selA	+2.13	10.08	+1.09	9.53	0.1	10.63	0.1	0.00101414	0.00658962	0.05478532	0.57311628	A_07_P016899	b3591	cmr|selenocysteine synthase: L-seryl-tRNA [b3591]
selA	+2.26	10.19	+1.18	9.60	0.0	10.78	0.1	0.00062336	0.00455203	0.04817137	0.54405308	A_07_P016899	b3591	cmr|selenocysteine synthase: L-seryl-tRNA [b3591]
selA	+2.17	9.95	+1.12	9.39	0.2	10.51	0.1	0.00187149	0.01047078	0.05433435	0.57067378	A_07_P016899	b3591	cmr|selenocysteine synthase: L-seryl-tRNA [b3591]
selB	+2.20	7.33	+1.14	6.76	0.3	7.90	0.0	0.00240044	0.01272050	0.05265180	0.56506476	A_07_P016891	b3590	cmr|selenocysteinyl-tRNA-specific translation factor [b3590]
selD	+4.77	10.28	+2.25	9.15	0.0	11.41	0.3	0.00005033	0.00074401	0.01097243	0.28648425	A_07_P018866	b1764	cmr|selenophosphate synthase, H [b1764]
serS	+7.75	9.06	+2.96	7.59	0.2	10.54	0.1	0.00000555	0.00017942	0.00403773	0.18372832	A_07_P004911	b0893	cmr|serine tRNA synthetase; also charges selenocystein tRNA with serine [b0893]
sfaB	+9.45	8.83	+3.24	7.21	0.1	10.45	0.0	0.00000133	0.00008557	0.00286522	0.15501718	A_07_P040481	c_1238	cmr|Putative F1C and S fimbrial switch Regulatory [c_1238]
sfaC	+4.55	8.03	+2.18	6.94	0.1	9.13	0.1	0.00001986	0.00039176	0.01242378	0.30092308	A_07_P040480	c_1237	cmr|Putative F1C and S fimbrial switch Regulatory [c_1237]
sfaD	+7.77	9.29	+2.96	7.81	0.0	10.77	0.1	0.00000251	0.00011582	0.00414063	0.18519886	A_07_P040493	c_1240	cmr|Putative minor F1C fimbrial subunit precursor [c_1240]
sfsA	+2.53	10.69	+1.34	10.02	0.0	11.36	0.6	0.01092416	0.04217507	0.04076347	0.50021824	A_07_P035869	c_0179	cmr|Sugar fermentation stimulation protein A [c_0179]
sfsA	+2.32	10.23	+1.21	9.63	0.1	10.84	0.1	0.00063713	0.00461617	0.04550114	0.52907681	A_07_P002692	b0146	cmr|probable regulator for maltose metabolism [b0146]
sgbH	+4.60	7.95	+2.20	6.85	0.1	9.05	0.1	0.00001880	0.00037681	0.01191946	0.29599369	A_07_P016850	b3581	cmr|probable 3-hexulose 6-phosphate synthase [b3581]
sgbU	+3.22	7.90	+1.69	7.06	0.2	8.75	0.0	0.00011563	0.00132585	0.02352325	0.40922652	A_07_P039678	c_4405	cmr|Putative hexulose-6-phosphate isomerase [c_4405]
sgcR	-2.74	8.35	-1.45	9.08	0.5	7.63	0.0	0.00510676	0.02317029	0.02617950	0.46678369	A_07_P004028	b4300	cmr|putative DEOR-type transcriptional regulator [b4300]
sgcX	+3.10	7.56	+1.63	6.74	0.0	8.38	0.3	0.00031769	0.00278650	0.02583587	0.42415016	A_07_P004054	b4305	cmr|putative lyase/synthase [b4305]
sitA	-2.55	9.27	-1.35	9.95	0.1	8.59	0.2	0.00044427	0.00358698	0.03359756	0.50715244	A_07_P038676	c_1600	cmr|SitA protein [c_1600]
sitB	-2.13	8.45	-1.09	9.00	0.1	7.91	0.0	0.00112854	0.00711563	0.06162221	0.63535036	A_07_P038673	c_1599	cmr|SitB protein [c_1599]
sitC	-4.76	8.28	-2.25	9.41	0.2	7.16	0.4	0.00025740	0.00239648	0.00410124	0.21032573	A_07_P038667	c_1598	cmr|SitC protein [c_1598]
skp	+6.65	12.57	+2.73	11.20	0.0	13.93	0.6	0.00030063	0.00268778	0.00603786	0.22579572	A_07_P002847	b0178	cmr|histone-like protein, located in outer membrane or nucleoid [b0178]
slpA	+2.74	10.43	+1.45	9.70	0.5	11.15	0.0	0.00297118	0.01507177	0.03409743	0.47007881	A_07_P051860	Z0033	cmr|probable FKBX-type 16KD peptidyl-prolyl cis-trans isomerase [Z0033]
slt	+2.71	8.23	+1.44	7.51	0.3	8.95	0.1	0.00128599	0.00783911	0.03397040	0.46997364	A_07_P017645	b4392	cmr|soluble lytic murein transglycosylase [b4392]
slt	+2.69	8.00	+1.43	7.29	0.0	8.72	0.1	0.00021512	0.00211279	0.03338605	0.46805877	A_07_P017645	b4392	cmr|soluble lytic murein transglycosylase [b4392]
slt	+2.43	8.41	+1.28	7.77	0.0	9.05	0.1	0.00041285	0.00340132	0.04121380	0.50417249	A_07_P017645	b4392	cmr|soluble lytic murein transglycosylase [b4392]
slt	+2.57	8.16	+1.36	7.48	0.1	8.84	0.1	0.00032951	0.00285827	0.03671240	0.48368201	A_07_P017645	b4392	cmr|soluble lytic murein transglycosylase [b4392]
slt	+2.31	8.11	+1.21	7.51	0.1	8.72	0.1	0.00054647	0.00413833	0.04590828	0.52950916	A_07_P051845	Z5994	cmr|soluble lytic murein transglycosylase [Z5994]
slt	+2.49	8.39	+1.32	7.73	0.1	9.05	0.2	0.00092988	0.00618506	0.03950521	0.49480509	A_07_P017645	b4392	cmr|soluble lytic murein transglycosylase [b4392]
slyB	-2.43	12.89	-1.28	13.53	0.0	12.25	0.4	0.00328813	0.01624870	0.04062373	0.54386054	A_07_P035132	c_2033	cmr|Outer membrane lipoprotein slyB precursor [c_2033]
smg	+5.86	9.58	+2.55	8.30	0.1	10.85	0.2	0.00001204	0.00028605	0.00737106	0.24483122	A_07_P034260	c_4045	cmr|Protein smg [c_4045]
smg	+3.65	9.10	+1.87	8.17	0.2	10.03	0.2	0.00013905	0.00152559	0.01889609	0.36060606	A_07_P012853	b3284	cmr|orf, hypothetical protein [b3284]
smg	+2.71	11.06	+1.44	10.34	0.3	11.78	0.0	0.00067649	0.00482179	0.03339685	0.46737778	A_07_P045982	Z4655	cmr|orf, hypothetical protein [Z4655]
smpA	-4.72	12.14	-2.24	13.26	0.1	11.02	0.0	0.00001400	0.00030964	0.00379764	0.20476027	A_07_P019965	b2617	cmr|small membrane protein A [b2617]
smpB	-3.53	12.19	-1.82	13.10	0.0	11.28	0.1	0.00006222	0.00086079	0.01126270	0.33205993	A_07_P019980	b2620	cmr|small protein B [b2620]
sodA	+2.18	9.86	+1.13	9.30	0.0	10.42	0.0	0.00070107	0.00493783	0.05153074	0.56029006	A_07_P041367	c_4859	cmr|Superoxide dismutase [c_4859]
sodA	+2.08	7.80	+1.06	7.27	0.1	8.33	0.3	0.00435251	0.02039497	0.05926766	0.59547543	A_07_P047103	Z5453	cmr|superoxide dismutase, manganese [Z5453]
sodA	+2.10	8.40	+1.07	7.87	0.3	8.94	0.1	0.00473968	0.02186391	0.06005272	0.59651104	A_07_P019316	b3908	cmr|superoxide dismutase, manganese [b3908]
sodB	+7.71	12.71	+2.95	11.24	0.1	14.19	0.2	0.00000470	0.00016577	0.00435213	0.18928177	A_07_P018342	b1656	cmr|superoxide dismutase, iron [b1656]
sodB	+4.22	12.84	+2.08	11.80	0.0	13.88	0.5	0.00051466	0.00396851	0.01368140	0.31676471	A_07_P042333	c_2050	cmr|Superoxide dismutase [c_2050]
sodC	+2.12	9.21	+1.09	8.67	0.2	9.75	0.1	0.00269425	0.01390305	0.05709413	0.58369481	A_07_P020863	b1646	cmr|superoxide dismutase precursor [b1646]
solA	+2.07	7.82	+1.05	7.29	0.1	8.34	0.1	0.00155618	0.00905078	0.05911712	0.59434227	A_07_P001500	b1059	cmr|sarcosine oxidase-like protein [b1059]
soxR	-2.73	8.68	-1.45	9.41	0.1	7.96	0.2	0.00057656	0.00430688	0.02817010	0.47535906	A_07_P008959	b4063	cmr|redox-sensing activator of soxS [b4063]
speA	+2.24	9.32	+1.16	8.74	0.1	9.90	0.1	0.00090291	0.00606204	0.04910061	0.55138374	A_07_P032963	c_3524	cmr|Biosynthetic arginine decarboxylase [c_3524]
speA	+2.15	9.76	+1.10	9.21	0.0	10.32	0.2	0.00135427	0.00813575	0.05377985	0.56902554	A_07_P011837	b2938	cmr|biosynthetic arginine decarboxylase [b2938]
speB	-2.36	9.52	-1.24	10.14	0.0	8.90	0.0	0.00038576	0.00323566	0.04392785	0.56273393	A_07_P011831	b2937	cmr|agmatinase [b2937]
speD	+3.82	10.55	+1.93	9.58	0.2	11.52	0.1	0.00009271	0.00113501	0.01716019	0.34860645	A_07_P002565	b0120	cmr|S-adenosylmethionine decarboxylase [b0120]
speF	+13.25	9.41	+3.73	7.55	0.4	11.28	0.4	0.00002971	0.00051171	0.00137767	0.11476190	A_07_P009981	b0693	cmr|ornithine decarboxylase isozyme, inducible [b0693]
speG	-3.23	10.69	-1.69	11.54	0.1	9.85	0.1	0.00012961	0.00144828	0.01540587	0.37315385	A_07_P020551	b1584	cmr|spermidine N1-acetyltransferase [b1584]
spr	+2.40	12.36	+1.26	11.73	0.1	12.99	0.1	0.00061595	0.00451258	0.04230501	0.51155914	A_07_P000674	b2175	cmr|putative lipoprotein [b2175]
srlA	+2.32	7.67	+1.22	7.06	0.0	8.28	0.3	0.00261033	0.01359031	0.04595211	0.52962665	A_07_P001960	b2702	cmr|PTS system, glucitol/sorbitol-specific IIC component, one of two [b2702]
srlA	+2.39	8.24	+1.25	7.61	0.2	8.86	0.2	0.00132802	0.00802315	0.04369284	0.51760722	A_07_P041662	c_3256	cmr|PTS system, glucitol/sorbitol-specific IIC2 component [c_3256]
srlA_1	+2.13	7.35	+1.09	6.80	0.0	7.90	0.0	0.00078331	0.00539515	0.05425622	0.57061456	A_07_P043096	Z4005	cmr|PTS system, glucitol/sorbitol-specific IIC component, one of [Z4005]
srlB	+3.73	7.53	+1.90	6.58	0.2	8.48	0.2	0.00019047	0.00193470	0.01773565	0.35167506	A_07_P001966	b2704	cmr|PTS system, glucitol/sorbitol-specific enzyme IIA component [b2704]
srlE	+2.72	7.35	+1.44	6.63	0.1	8.07	0.3	0.00080609	0.00553232	0.03354294	0.46817376	A_07_P030595	c_3257	cmr|PTS system, glucitol/sorbitol-specific IIBC component [c_3257]
srlE	+2.74	7.55	+1.45	6.83	0.0	8.28	0.2	0.00056391	0.00423782	0.03272739	0.46294699	A_07_P001964	b2703	cmr|PTS system, glucitol/sorbitol-specific IIB component and second of two IIC components [b2703]
srlE_1	+2.20	8.68	+1.14	8.11	0.1	9.25	0.2	0.00164875	0.00948802	0.05215765	0.56360329	A_07_P043105	Z4007	cmr|PTS system, glucitol/sorbitol-specific IIB component and second [Z4007]
srmB	+3.84	8.92	+1.94	7.95	0.3	9.89	0.2	0.00015814	0.00168799	0.01700457	0.34633894	A_07_P042926	Z3859	cmr|ATP-dependent RNA helicase [Z3859]
srmB	+3.72	9.35	+1.90	8.40	0.0	10.29	0.1	0.00003648	0.00058676	0.01824759	0.35337023	A_07_P030217	c_3100	cmr|ATP-dependent RNA helicase srmB [c_3100]
ssb	-3.45	12.98	-1.79	13.87	0.0	12.09	0.1	0.00006916	0.00092982	0.01190930	0.33542039	A_07_P008940	b4059	cmr|ssDNA-binding protein [b4059]
ssbW	+12.79	13.55	+3.68	11.71	0.0	15.39	0.2	0.00000117	0.00008016	0.00152439	0.11881188	A_07_P051338	Z1440	cmr|putative single-stranded DNA binding protein [Z1440]
sseA	+11.60	11.78	+3.54	10.01	0.1	13.55	0.1	0.00000172	0.00009748	0.00188770	0.12978166	A_07_P013661	b2521	cmr|putative thiosulfate sulfurtransferase [b2521]
sspA	-5.07	13.46	-2.34	14.63	0.0	12.29	0.1	0.00001284	0.00029436	0.00292048	0.18031373	A_07_P012616	b3229	cmr|regulator of transcription; stringent starvation protein A [b3229]
sspB	-6.30	13.85	-2.66	15.17	0.0	12.52	0.3	0.00001748	0.00036012	0.00136369	0.12410405	A_07_P012612	b3228	cmr|stringent starvation protein B [b3228]
sstT	+2.38	8.32	+1.25	7.70	0.1	8.95	0.1	0.00052964	0.00404719	0.04306148	0.51321726	A_07_P000173	b3089	cmr|putative transport protein [b3089]
stpA	-2.35	10.61	-1.23	11.22	0.1	9.99	0.2	0.00080283	0.00551710	0.04423971	0.56306386	A_07_P020199	b2669	cmr|DNA-binding protein; H-NS-like protein; chaperone activity; RNA splicing? [b2669]
sucA	+116.82	10.82	+6.87	7.39	0.0	14.25	0.5	0.00000048	0.00005238	0.00000000	0.00000000	A_07_P010149	b0726	cmr|2-oxoglutarate dehydrogenase [b0726]
sucB	+84.25	12.52	+6.40	9.32	0.0	15.71	0.2	0.00000004	0.00003344	0.00000381	0.00461538	A_07_P010151	b0727	cmr|2-oxoglutarate dehydrogenase [b0727]
sucC	+94.53	11.60	+6.56	8.32	0.1	14.88	0.0	0.00000002	0.00003344	0.00000254	0.00400000	A_07_P010159	b0728	cmr|succinyl-CoA synthetase, beta subunit [b0728]
sucD	+54.91	10.83	+5.78	7.94	0.0	13.72	0.3	0.00000027	0.00004211	0.00001842	0.01074074	A_07_P010165	b0729	cmr|succinyl-CoA synthetase, alpha subunit [b0729]
sugE	-5.05	11.54	-2.34	12.71	0.1	10.37	0.0	0.00001387	0.00030876	0.00303290	0.18365385	A_07_P035585	c_5236	cmr|SugE protein [c_5236]
sugE	-3.14	9.59	-1.65	10.42	0.1	8.77	0.0	0.00008255	0.00104397	0.01703760	0.37833568	A_07_P048623	Z5755	cmr|suppresses groEL, may be chaperone [Z5755]
sugE	-3.49	10.37	-1.80	11.27	0.2	9.47	0.0	0.00015938	0.00169545	0.01141260	0.33212569	A_07_P010653	b4148	cmr|suppresses groEL, may be chaperone [b4148]
sugE	-12.07	8.98	-3.59	10.77	0.1	7.18	0.1	0.00000076	0.00006289	0.00007749	0.02975610	A_07_P011472	b4148	cmr|suppresses groEL, may be chaperone [b4148]
sulA	+3.70	15.26	+1.89	14.31	0.0	16.20	0.0	0.00003479	0.00056765	0.01844322	0.35497555	A_07_P005240	b0958	cmr|suppressor of lon; inhibits cell division and ftsZ ring formation [b0958]
symE	-2.50	11.37	-1.32	12.03	0.5	10.71	0.2	0.00586348	0.02589470	0.03600546	0.51816271	A_07_P017432	b4347	cmr|orf, hypothetical protein [b4347]
tadA	-4.90	9.14	-2.29	10.29	0.0	7.99	0.3	0.00004192	0.00064831	0.00345084	0.19403571	A_07_P019700	b2559	cmr|putative deaminase [b2559]
talA	+2.51	8.22	+1.33	7.55	0.3	8.88	0.1	0.00162245	0.00938075	0.03969766	0.49524564	A_07_P013377	b2464	cmr|transaldolase A [b2464]
talB	+2.90	9.97	+1.54	9.20	0.1	10.74	0.0	0.00016970	0.00178003	0.02880399	0.44028155	A_07_P010731	b0008	cmr|transaldolase B [b0008]
talC	+2.63	10.97	+1.40	10.27	0.1	11.67	0.2	0.00060908	0.00447365	0.03516705	0.47771355	A_07_P039490	c_4905	cmr|Fructose-6-phosphate aldolase 2 [c_4905]
tatB	-3.79	13.45	-1.92	14.41	0.0	12.49	0.4	0.00049558	0.00387598	0.00905170	0.30581545	A_07_P018999	b3838	cmr|orf, hypothetical protein [b3838]
tauA	-3.01	8.34	-1.59	9.14	0.3	7.54	0.0	0.00064636	0.00466377	0.01901359	0.39596561	A_07_P031067	c_0472	cmr|Taurine-binding periplasmic protein precursor [c_0472]
tdcA	+13.69	10.35	+3.77	8.46	0.2	12.24	0.1	0.00000122	0.00008163	0.00133575	0.11306452	A_07_P000316	b3118	cmr|transcriptional activator of tdc operon [b3118]
tdcB	+17.73	9.20	+4.15	7.12	0.1	11.27	0.4	0.00000564	0.00018039	0.00069233	0.07785714	A_07_P000312	b3117	cmr|threonine dehydratase, catabolic [b3117]
tdcC	+52.74	10.10	+5.72	7.24	0.3	12.96	0.2	0.00000024	0.00004211	0.00001651	0.01083333	A_07_P000306	b3116	cmr|anaerobically inducible L-threonine, L-serine permease [b3116]
tdcD	+18.75	8.38	+4.23	6.27	0.1	10.50	0.4	0.00000267	0.00012042	0.00059832	0.07246154	A_07_P000305	b3115	cmr|putative kinase [b3115]
tdcD	+97.02	10.53	+6.60	7.23	0.0	13.83	0.1	0.00000001	0.00003344	0.00000254	0.00444444	A_07_P045889	Z4467	cmr|putative kinase [Z4467]
tdcE	+10.28	10.03	+3.36	8.35	0.0	11.71	0.4	0.00000980	0.00024876	0.00244982	0.14721374	A_07_P034076	c_3872	cmr|Keto-acid formate acetyltransferase [c_3872]
tdcE	+10.64	9.84	+3.41	8.14	0.1	11.55	0.2	0.00000323	0.00013159	0.00221481	0.13674510	A_07_P000296	b3114	cmr|probable formate acetyltransferase 3 [b3114]
tdcF	+8.18	10.13	+3.03	8.62	0.2	11.65	0.1	0.00000601	0.00018702	0.00380145	0.17865672	A_07_P000294	b3113	cmr|orf, hypothetical protein [b3113]
tdh	+14.97	10.64	+3.90	8.69	0.0	12.59	0.0	0.00000037	0.00004793	0.00103468	0.09696429	A_07_P017024	b3616	cmr|threonine dehydrogenase [b3616]
tehB	+2.21	9.08	+1.15	8.51	0.1	9.65	0.2	0.00140162	0.00833983	0.05111598	0.55886806	A_07_P009242	b1430	cmr|tellurite resistance [b1430]
tfaR	-4.31	9.19	-2.11	10.25	0.0	8.14	0.0	0.00001664	0.00034782	0.00534743	0.23917614	A_07_P014841	b1373	cmr|orf, hypothetical protein [b1373]
tgt	+9.84	10.68	+3.30	9.03	0.0	12.33	0.1	0.00000158	0.00009392	0.00261814	0.14880866	A_07_P021144	b0406	cmr|tRNA-guanine transglycosylase [b0406]
thiD	-7.21	9.79	-2.85	11.22	0.2	8.37	0.2	0.00001017	0.00025323	0.00077617	0.08791367	A_07_P003278	b2103	cmr|phosphomethylpyrimidine kinase [b2103]
thiG	+2.14	6.84	+1.10	6.29	0.2	7.39	0.0	0.00164229	0.00946767	0.05498222	0.57365142	A_07_P008618	b3991	cmr|thiamin biosynthesis, thiazole moiety [b3991]
thiG	+2.50	8.06	+1.32	7.40	0.0	8.72	0.3	0.00105088	0.00676135	0.03925114	0.49398082	A_07_P010631	b3991	cmr|thiamin biosynthesis, thiazole moiety [b3991]
thiK	+2.57	10.93	+1.36	10.25	0.0	11.61	0.2	0.00045066	0.00362743	0.03688770	0.48517962	A_07_P001731	b1106	cmr|putative beta-glucosidase [b1106]
thiM	-3.37	9.36	-1.75	10.24	0.6	8.49	0.1	0.00331686	0.01635987	0.01322853	0.34596346	A_07_P045138	Z3268	cmr|hydoxyethylthiazole kinase [Z3268]
thiM	-4.80	9.05	-2.26	10.18	0.0	7.92	0.3	0.00005453	0.00078334	0.00366489	0.20034722	A_07_P003284	b2104	cmr|hydoxyethylthiazole kinase [b2104]
thrA	+2.88	7.94	+1.53	7.18	0.1	8.70	0.2	0.00027954	0.00255874	0.02954840	0.44347950	A_07_P010704	b0002	cmr|aspartokinase I, homoserine dehydrogenase I [b0002]
thrB	+3.50	8.18	+1.81	7.27	0.2	9.08	0.2	0.00024535	0.00231443	0.02023565	0.37437133	A_07_P010710	b0003	cmr|homoserine kinase [b0003]
thrC	+3.50	9.55	+1.81	8.65	0.0	10.45	0.0	0.00004254	0.00065463	0.02026550	0.37360656	A_07_P035606	c_0005	cmr|Threonine synthase [c_0005]
thyA	+3.26	9.41	+1.70	8.56	0.1	10.26	0.0	0.00008457	0.00106601	0.02282774	0.40291480	A_07_P006119	b2827	cmr|thymidylate synthetase [b2827]
tilS	-2.40	8.78	-1.26	9.41	0.0	8.14	0.2	0.00111097	0.00704150	0.04263211	0.55701245	A_07_P002899	b0188	cmr|cell cycle protein [b0188]
tktA	+7.09	11.63	+2.83	10.22	0.0	13.04	0.5	0.00012787	0.00143388	0.00524327	0.20898734	A_07_P032946	c_3520	cmr|Transketolase 1 [c_3520]
tktA	+3.18	8.86	+1.67	8.03	0.6	9.70	0.1	0.00598733	0.02630882	0.02497967	0.41882854	A_07_P045696	Z4279	cmr|transketolase 1 isozyme [Z4279]
tktA	+5.45	8.56	+2.45	7.34	0.3	9.78	0.1	0.00005752	0.00081364	0.00837970	0.25371154	A_07_P011821	b2935	cmr|transketolase 1 isozyme [b2935]
tldD	+2.24	9.36	+1.17	8.78	0.4	9.95	0.0	0.00425300	0.02001174	0.05079141	0.55842179	A_07_P034184	c_3999	cmr|TldD protein [c_3999]
tnaA	+108.28	10.02	+6.76	6.64	0.2	13.39	0.2	0.00000007	0.00003344	0.00000000	0.00000000	A_07_P057646	ECs4645	cmr|tryptophanase [ECs4645]
tnaA	+53.25	11.43	+5.73	8.56	0.1	14.30	0.1	0.00000006	0.00003344	0.00001842	0.00966667	A_07_P005461	b3708	cmr|tryptophanase [b3708]
tnaA	+112.63	10.34	+6.82	6.93	0.1	13.75	0.1	0.00000002	0.00003344	0.00000000	0.00000000	A_07_P049911	Z5203	cmr|tryptophanase [Z5203]
tnaB	+4.82	8.66	+2.27	7.52	0.3	9.79	0.2	0.00011523	0.00132359	0.01087716	0.28494176	A_07_P041909	c_4632	cmr|Low affinity tryptophan permease [c_4632]
tnaB	+2.79	7.76	+1.48	7.02	0.3	8.50	0.1	0.00078337	0.00539515	0.03193534	0.45916895	A_07_P005466	b3709	cmr|low affinity tryptophan permease [b3709]
tolB	+5.76	10.90	+2.53	9.64	0.2	12.16	0.0	0.00001282	0.00029422	0.00766197	0.24719262	A_07_P010216	b0740	cmr|periplasmic protein involved in the tonb-independent uptake of group A colicins [b0740]
tolC	+2.25	10.20	+1.17	9.61	0.0	10.78	0.4	0.00780309	0.03229545	0.05059769	0.55863254	A_07_P012308	b3035	cmr|outer membrane channel; specific tolerance to colicin E1; segregation of daughter chromosomes [b3035]
tolQ	-2.05	11.18	-1.04	11.70	0.1	10.66	0.3	0.00719185	0.03032437	0.06926702	0.65814122	A_07_P010205	b0737	cmr|inner membrane protein, membrane-spanning, maintains integrity of cell envelope; tolerance to group A colicins [b0737]
torD	-2.08	7.89	-1.06	8.42	0.0	7.37	0.0	0.00100641	0.00655018	0.06612741	0.65150814	A_07_P061916	ECs1153	cmr|part of trimethylamine-N-oxide oxidoreductase [ECs1153]
torS	-2.56	12.53	-1.36	13.20	0.1	11.85	0.0	0.00030671	0.00272199	0.03432673	0.51129612	A_07_P001197	b0993	cmr|sensor protein torS [b0993]
tpiA	+4.95	13.11	+2.31	11.95	0.0	14.26	0.0	0.00000993	0.00025049	0.01043001	0.28022184	A_07_P019375	b3919	cmr|triosephosphate isomerase [b3919]
tpx	+9.35	11.22	+3.22	9.60	0.0	12.83	0.1	0.00000146	0.00009005	0.00287983	0.15527397	A_07_P014605	b1324	cmr|thiol peroxidase [b1324]
treB	+3.43	8.47	+1.78	7.58	0.1	9.36	0.2	0.00010552	0.00124725	0.02100991	0.38284722	A_07_P003751	b4240	cmr|PTS system enzyme II, trehalose specific [b4240]
treR	-3.60	11.44	-1.85	12.36	0.1	10.52	0.3	0.00023960	0.00227378	0.01056085	0.32348249	A_07_P003760	b4241	cmr|repressor of treA,B,C [b4241]
trmA	-3.11	9.11	-1.63	9.93	0.1	8.30	0.1	0.00014388	0.00156332	0.01758448	0.38558496	A_07_P047177	Z5526	cmr|tRNA [Z5526]
trmH	-2.75	10.42	-1.46	11.15	0.1	9.68	0.1	0.00019329	0.00195328	0.02635925	0.46839729	A_07_P017198	b3651	cmr|putative RNA methylase [b3651]
trmI	+6.02	12.75	+2.59	11.46	0.0	14.05	0.1	0.00000800	0.00022294	0.00696329	0.23832609	A_07_P011943	b2960	cmr|orf, hypothetical protein [b2960]
trmJ	-10.10	13.37	-3.34	15.04	0.0	11.70	0.4	0.00001935	0.00038447	0.00019627	0.04544118	A_07_P019564	b2532	cmr|putative ATP synthase beta subunit [b2532]
trpB	+26.35	10.01	+4.72	7.65	0.1	12.37	0.2	0.00000034	0.00004570	0.00021659	0.04011765	A_07_P014291	b1261	cmr|tryptophan synthase, beta protein [b1261]
trpB	+32.52	10.82	+5.02	8.31	0.0	13.34	0.0	0.00000007	0.00003370	0.00010226	0.02728814	A_07_P038879	c_1726	cmr|Tryptophan synthase beta chain [c_1726]
trpB	+22.36	10.17	+4.48	7.93	0.1	12.41	0.1	0.00000023	0.00004002	0.00036522	0.05476190	A_07_P044133	Z2550	cmr|tryptophan synthase, beta protein [Z2550]
trpC	+23.35	10.42	+4.55	8.15	0.1	12.70	0.2	0.00000028	0.00004211	0.00030234	0.05010526	A_07_P014296	b1262	cmr|N- [b1262]
trpD	+12.09	9.88	+3.60	8.08	0.3	11.67	0.1	0.00000479	0.00016636	0.00171748	0.12235294	A_07_P038884	c_1729	cmr|Anthranilate synthase component II [c_1729]
trpD	+28.40	9.35	+4.83	6.93	0.1	11.76	0.1	0.00000018	0.00003706	0.00017721	0.03720000	A_07_P014304	b1263	cmr|anthranilate synthase component II, glutamine amidotransferase and phosphoribosylanthranilate transferase [b1263]
trpE	+8.12	8.58	+3.02	7.07	0.3	10.09	0.3	0.00005074	0.00074664	0.00387640	0.18002950	A_07_P038886	c_1730	cmr|Anthranilate synthase component I [c_1730]
trpR	+6.54	9.32	+2.71	7.96	0.0	10.67	0.1	0.00000533	0.00017677	0.00586446	0.22194712	A_07_P017646	b4393	cmr|regulator for trp operon and aroH; trp aporepressor [b4393]
trxA	-5.89	14.05	-2.56	15.33	0.0	12.77	0.1	0.00000560	0.00017942	0.00176131	0.13865000	A_07_P057729	ECs4714	cmr|thioredoxin 1 [ECs4714]
trxA	-8.98	13.52	-3.17	15.10	0.0	11.94	0.3	0.00000652	0.00019522	0.00035061	0.05632653	A_07_P005789	b3781	cmr|thioredoxin 1 [b3781]
trxA	-6.39	14.01	-2.68	15.35	0.1	12.67	0.0	0.00000407	0.00015128	0.00132304	0.12110465	A_07_P035554	c_4701	cmr|Thioredoxin 1 [c_4701]
trxB	+5.65	12.94	+2.50	11.69	0.1	14.19	0.2	0.00001327	0.00030016	0.00788491	0.24877756	A_07_P033812	c_1025	cmr|Thioredoxin reductase [c_1025]
tsgA	-4.36	7.90	-2.12	8.97	0.1	6.84	0.1	0.00002707	0.00047936	0.00516895	0.23452450	A_07_P017847	b3364	cmr|putative transport [b3364]
ttdR	-3.87	9.73	-1.95	10.71	0.1	8.75	0.0	0.00004113	0.00063933	0.00795287	0.28717890	A_07_P000033	b3060	cmr|putative transcriptional regulator LYSR-type [b3060]
tuf	+2.06	16.19	+1.04	15.67	0.1	16.71	0.3	0.00573632	0.02545777	0.06497967	0.62380488	A_07_P059760	ECs4190	cmr|protein chain elongation factor EF-Tu [ECs4190]
tuf	+2.84	16.92	+1.51	16.17	0.0	17.67	0.2	0.00023572	0.00224917	0.03001969	0.44503766	A_07_P046005	Z4697	cmr|protein chain elongation factor EF-Tu [Z4697]
tufA	+2.45	16.70	+1.29	16.05	0.0	17.34	0.0	0.00030743	0.00272536	0.04044080	0.49937255	A_07_P017725	b3339	cmr|protein chain elongation factor EF-Tu [b3339]
tyrB	+2.66	8.17	+1.41	7.46	0.3	8.87	0.2	0.00150190	0.00879682	0.03485137	0.47506494	A_07_P037596	c_5031	cmr|Aromatic-amino-acid aminotransferase [c_5031]
tyrB	+2.09	7.55	+1.07	7.02	0.1	8.09	0.0	0.00113112	0.00712615	0.05726817	0.58433571	A_07_P008914	b4054	cmr|tyrosine aminotransferase, tyrosine repressible [b4054]
tyrS	+5.38	8.70	+2.43	7.49	0.1	9.92	0.2	0.00001462	0.00031702	0.00880462	0.26056391	A_07_P020819	b1637	cmr|tyrosine tRNA synthetase [b1637]
ubiB	+2.14	12.45	+1.09	11.90	0.1	13.00	0.2	0.00150951	0.00883482	0.05549225	0.57667987	A_07_P018983	b3835	cmr|orf, hypothetical protein [b3835]
ubiE	-2.54	14.02	-1.35	14.70	0.0	13.35	0.1	0.00039763	0.00332058	0.03438961	0.51126534	A_07_P018975	b3833	cmr|2-octaprenyl-6-methoxy-1,4-benzoquinone --> 2-octaprenyl-3-methyl-6-methoxy-1,4-benzoquinone [b3833]
ubiF	-2.04	9.23	-1.03	9.75	0.1	8.72	0.1	0.00221847	0.01198612	0.06892975	0.65771515	A_07_P044036	Z0811	cmr|orf, hypothetical protein [Z0811]
ubiH	+2.24	11.79	+1.17	11.21	0.1	12.38	0.2	0.00141079	0.00837854	0.04868521	0.54867573	A_07_P045655	Z4244	cmr|2-octaprenyl-6-methoxyphenol--> 2-octaprenyl-6-methoxy-1, 4-benzoquinone [Z4244]
ubiH	+2.50	11.93	+1.32	11.27	0.0	12.59	0.2	0.00073664	0.00514135	0.03910823	0.49297038	A_07_P032895	c_3489	cmr|2-octaprenyl-6-methoxyphenol hydroxylase [c_3489]
ucpA	+6.93	9.20	+2.79	7.80	0.2	10.60	0.1	0.00000577	0.00018187	0.00518610	0.20776081	A_07_P013188	b2426	cmr|putative oxidoreductase [b2426]
ucpA	+5.86	11.53	+2.55	10.26	0.1	12.81	0.5	0.00016733	0.00176096	0.00761623	0.24774793	A_07_P041240	c_2960	cmr|Oxidoreductase ucpA [c_2960]
ugd	-2.98	8.69	-1.58	9.47	0.1	7.90	0.0	0.00011102	0.00129181	0.01998984	0.40661499	A_07_P042482	c_2555	cmr|UDP-glucose 6-dehydrogenase [c_2555]
ugd	-2.09	9.14	-1.06	9.67	0.1	8.60	0.1	0.00126710	0.00774429	0.06527439	0.64919773	A_07_P015464	b2028	cmr|UDP-glucose 6-dehydrogenase [b2028]
ugd	-3.14	8.77	-1.65	9.60	0.1	7.95	0.1	0.00012457	0.00140493	0.01704332	0.37792958	A_07_P044992	Z3190	cmr|UDP-glucose 6-dehydrogenase [Z3190]
ugpB	+2.39	8.25	+1.26	7.62	0.1	8.88	0.0	0.00039819	0.00332076	0.04228976	0.51176787	A_07_P018281	b3453	cmr|sn-glycerol 3-phosphate transport system; periplasmic binding protein [b3453]
ugpC	+3.55	8.41	+1.83	7.50	0.0	9.32	0.2	0.00009375	0.00114240	0.01996126	0.37103896	A_07_P018267	b3450	cmr|ATP-binding component of sn-glycerol 3-phosphate transport system [b3450]
uhpT	-9.25	8.52	-3.21	10.12	0.0	6.91	0.0	0.00000121	0.00008146	0.00029027	0.05506024	A_07_P017270	b3666	cmr|hexose phosphate transport protein [b3666]
uhpT	-10.66	8.84	-3.41	10.54	0.0	7.13	0.1	0.00000109	0.00007696	0.00013720	0.03724138	A_07_P038972	c_4590	cmr|Hexose phosphate transport protein [c_4590]
ulaB	+2.83	7.68	+1.50	6.93	0.0	8.43	0.1	0.00020396	0.00202470	0.03009146	0.44484507	A_07_P011689	b4194	cmr|orf, hypothetical protein [b4194]
ulaC	+2.34	8.13	+1.23	7.52	0.2	8.75	0.2	0.00126429	0.00773824	0.04572599	0.52895665	A_07_P011693	b4195	cmr|putative PTS system enzyme II A component [b4195]
ulaD	+3.08	9.51	+1.62	8.70	0.3	10.32	0.3	0.00085206	0.00580198	0.02637195	0.42672148	A_07_P011698	b4196	cmr|probable hexulose-6-phosphate synthase [b4196]
umuC	-3.56	11.65	-1.83	12.56	0.1	10.73	0.0	0.00004186	0.00064800	0.01081237	0.32548757	A_07_P006875	b1184	cmr|SOS mutagenesis and repair [b1184]
ung	-2.49	10.44	-1.32	11.10	0.1	9.78	0.2	0.00055506	0.00419421	0.03668128	0.52310688	A_07_P041614	c_3105	cmr|Uracil-DNA glycosylase [c_3105]
upp	+10.76	12.49	+3.43	10.78	0.2	14.20	0.0	0.00000186	0.00009954	0.00215892	0.13541833	A_07_P013547	b2498	cmr|uracil phosphoribosyltransferase [b2498]
usg	-2.05	11.19	-1.04	11.71	0.0	10.67	0.2	0.00194642	0.01080552	0.06873349	0.65783587	A_07_P032443	c_2864	cmr|USG-1 protein [c_2864]
uspA	+15.19	12.93	+3.92	10.97	0.0	14.90	0.1	0.00000038	0.00004857	0.00100673	0.09723926	A_07_P007413	b3495	cmr|universal stress protein; broad regulatory function? [b3495]
uspA	+11.74	11.92	+3.55	10.14	0.0	13.69	0.1	0.00000071	0.00006179	0.00183054	0.12866071	A_07_P049374	Z4895	cmr|universal stress protein broad regulatory function? [Z4895]
uspA	+12.45	12.42	+3.64	10.60	0.1	14.24	0.1	0.00000125	0.00008213	0.00155043	0.11679426	A_07_P034623	c_4293	cmr|Universal stress protein A [c_4293]
uspD	+3.96	9.48	+1.98	8.48	0.0	10.47	0.1	0.00003221	0.00054058	0.01633765	0.34296000	A_07_P019392	b3923	cmr|putative regulator [b3923]
uspF	+27.92	11.68	+4.80	9.28	0.1	14.08	0.3	0.00000059	0.00005706	0.00017149	0.03750000	A_07_P014857	b1376	cmr|putative filament protein [b1376]
uvrA	-5.55	13.39	-2.47	14.63	0.0	12.16	0.1	0.00000705	0.00020411	0.00217099	0.15466063	A_07_P008933	b4058	cmr|excision nuclease subunit A [b4058]
uvrB	-9.07	13.23	-3.18	14.82	0.0	11.64	0.1	0.00000148	0.00009122	0.00030869	0.05400000	A_07_P033475	c_0860	cmr|Excinuclease ABC subunit B [c_0860]
uvrB	-10.60	12.32	-3.41	14.02	0.1	10.62	0.2	0.00000355	0.00013789	0.00014672	0.03786885	A_07_P004347	b0779	cmr|DNA repair; excision nuclease subunit B [b0779]
uxaA	+14.49	8.82	+3.86	6.89	0.3	10.75	0.2	0.00000328	0.00013204	0.00112487	0.10236994	A_07_P000185	b3091	cmr|altronate hydrolase [b3091]
uxaA	+21.78	10.04	+4.44	7.82	0.1	12.26	0.0	0.00000016	0.00003548	0.00040142	0.05745455	A_07_P034058	c_3849	cmr|Altronate hydrolase [c_3849]
uxaB	+2.32	7.90	+1.22	7.29	0.2	8.50	0.1	0.00085337	0.00580363	0.04551321	0.52882657	A_07_P020252	b1521	cmr|altronate oxidoreductase [b1521]
uxaB	+2.89	9.99	+1.53	9.23	0.0	10.76	0.2	0.00024186	0.00228836	0.02925559	0.44245917	A_07_P052450	Z2184	cmr|altronate oxidoreductase [Z2184]
uxaC	+55.22	10.21	+5.79	7.32	0.0	13.10	0.1	0.00000004	0.00003344	0.00001334	0.00954545	A_07_P000188	b3092	cmr|uronate isomerase [b3092]
uxuB	+2.69	9.60	+1.43	8.89	0.1	10.31	0.2	0.00037351	0.00315226	0.03377414	0.46973498	A_07_P037204	c_5403	cmr|D-mannonate oxidoreductase [c_5403]
uxuB	+6.05	8.50	+2.60	7.20	0.1	9.80	0.2	0.00000943	0.00024327	0.00680196	0.23640177	A_07_P047511	Z5921	cmr|mannonate oxidoreductase [Z5921]
vacJ	+2.67	7.73	+1.42	7.02	0.1	8.44	0.3	0.00098464	0.00645922	0.03426702	0.47076789	A_07_P032512	c_2890	cmr|VacJ lipoprotein precursor [c_2890]
vacJ	+2.60	8.55	+1.38	7.86	0.3	9.24	0.2	0.00153086	0.00893653	0.03625000	0.48080876	A_07_P014018	b2346	cmr|lipoprotein precursor [b2346]
valS	-2.13	11.09	-1.09	11.63	0.1	10.54	0.4	0.00799071	0.03293072	0.06130971	0.63629532	A_07_P003836	b4258	cmr|valine tRNA synthetase [b4258]
vsr	-2.21	9.03	-1.15	9.60	0.1	8.45	0.1	0.00103667	0.00669591	0.05444677	0.60580212	A_07_P015156	b1960	cmr|DNA mismatch endonuclease, patch repair protein [b1960]
waaP	-4.18	10.16	-2.06	11.19	0.2	9.12	0.1	0.00004651	0.00069738	0.00592035	0.25191892	A_07_P049579	Z5054	cmr|putative LPS biosynthesis enzyme [Z5054]
wbbK	-3.00	8.44	-1.58	9.23	0.3	7.64	0.0	0.00067232	0.00480046	0.01938326	0.39943717	A_07_P015476	b2032	cmr|putative glucose transferase [b2032]
wbdN	-3.42	10.44	-1.78	11.32	0.1	9.55	0.1	0.00005984	0.00083757	0.01231072	0.33766551	A_07_P045051	Z3204	cmr|glycosyl transferase [Z3204]
wrbA	+3.71	9.64	+1.89	8.69	0.1	10.58	0.1	0.00009385	0.00114270	0.01812754	0.35365551	A_07_P001254	b1004	cmr|trp repressor binding protein; affects association of trp repressor and operator [b1004]
wzb	-2.62	8.06	-1.39	8.76	0.1	7.37	0.0	0.00029161	0.00263253	0.03184324	0.49884577	A_07_P003081	b2061	cmr|probable protein-tyrosine-phosphatase [b2061]
wzyE	+2.92	8.41	+1.55	7.63	0.2	9.18	0.1	0.00031842	0.00279132	0.02890117	0.43963285	A_07_P005852	b3793	cmr|TDP-Fuc4NAc:lipidII transferase; synthesis of enterobacterial common antigen [b3793]
wzzB	-4.25	11.11	-2.09	12.15	0.0	10.06	0.0	0.00001758	0.00036128	0.00555069	0.24410615	A_07_P031985	c_2554	cmr|Chain length determinant protein [c_2554]
xapA	-2.13	11.18	-1.09	11.73	0.1	10.64	0.4	0.00719202	0.03032437	0.06104421	0.63521481	A_07_P014289	b2407	cmr|xanthosine phosphorylase [b2407]
xdhA	+2.43	8.79	+1.28	8.15	0.3	9.43	0.1	0.00172474	0.00984208	0.04216527	0.51104696	A_07_P006300	b2866	cmr|orf, hypothetical protein [b2866]
xerC	+2.81	8.19	+1.49	7.44	0.3	8.93	0.3	0.00167219	0.00959142	0.03118902	0.45257143	A_07_P049986	Z5328	cmr|site-specific recombinase, acts on cer sequence of [Z5328]
xerC	+2.67	8.08	+1.42	7.37	0.2	8.79	0.1	0.00039778	0.00332058	0.03422066	0.47054148	A_07_P005923	b3811	cmr|site-specific recombinase, acts on cer sequence of ColE1, effects chromosome segregation at cell division [b3811]
xerC	+3.51	8.69	+1.81	7.78	0.0	9.59	0.1	0.00006068	0.00084472	0.02025724	0.37389215	A_07_P039112	c_4732	cmr|Integrase/recombinase xerC [c_4732]
xerC	+2.47	8.55	+1.30	7.90	0.0	9.20	0.2	0.00050657	0.00393463	0.03991489	0.49756136	A_07_P057731	ECs4741	cmr|site-specific recombinase XerC [ECs4741]
xerD	-3.06	11.87	-1.61	12.68	0.0	11.06	0.1	0.00010722	0.00126354	0.01851118	0.39172043	A_07_P006436	b2894	cmr|site-specific recombinase [b2894]
xseA	-2.38	11.32	-1.25	11.95	0.3	10.70	0.4	0.01000454	0.03922098	0.04257431	0.55671927	A_07_P013602	b2509	cmr|exonuclease VII, large subunit [b2509]
xylG	+3.22	8.18	+1.68	7.34	0.0	9.03	0.1	0.00008731	0.00108817	0.02379065	0.41070175	A_07_P007761	b3567	cmr|putative ATP-binding protein of xylose transport system [b3567]
yaaA	+2.40	9.20	+1.26	8.57	0.1	9.83	0.1	0.00052047	0.00400270	0.04232660	0.51142748	A_07_P010722	b0006	cmr|orf, hypothetical protein [b0006]
yaaH	-2.89	8.45	-1.53	9.21	0.1	7.68	0.1	0.00023411	0.00224064	0.02259591	0.43596814	A_07_P010741	b0010	cmr|orf, hypothetical protein [b0010]
yaaY	-3.81	8.39	-1.93	9.35	0.0	7.43	0.1	0.00003930	0.00062005	0.00828697	0.29652273	A_07_P010806	b0024	cmr|orf, hypothetical protein [b0024]
yabI	-3.45	8.35	-1.79	9.25	0.1	7.46	0.3	0.00022056	0.00214748	0.01227896	0.33797203	A_07_P035723	c_0081	cmr|Hypothetical protein yabI [c_0081]
yabI	-3.77	8.30	-1.92	9.26	0.1	7.34	0.0	0.00003426	0.00056309	0.00883448	0.30171367	A_07_P035723	c_0081	cmr|Hypothetical protein yabI [c_0081]
yabI	-3.44	8.08	-1.78	8.97	0.0	7.19	0.1	0.00006932	0.00093035	0.01207254	0.33760213	A_07_P035723	c_0081	cmr|Hypothetical protein yabI [c_0081]
yabI	-3.41	8.19	-1.77	9.07	0.0	7.30	0.1	0.00005338	0.00077458	0.01276296	0.34290102	A_07_P035723	c_0081	cmr|Hypothetical protein yabI [c_0081]
yabI	-3.67	8.22	-1.88	9.16	0.0	7.28	0.3	0.00021160	0.00208349	0.00927973	0.30887949	A_07_P035723	c_0081	cmr|Hypothetical protein yabI [c_0081]
yacF	-2.00	10.06	-1.00	10.56	0.1	9.56	0.0	0.00176344	0.01001213	0.07481517	0.67539564	A_07_P002479	b0102	cmr|orf, hypothetical protein [b0102]
yacK	-3.15	10.16	-1.65	10.98	0.7	9.33	0.2	0.01129508	0.04329919	0.01665206	0.37722302	A_07_P051870	Z0133	cmr|orf, hypothetical protein [Z0133]
yacL	+2.18	12.96	+1.12	12.40	0.1	13.52	0.1	0.00108193	0.00691032	0.05272739	0.56510551	A_07_P002559	b0119	cmr|orf, hypothetical protein [b0119]
yadC	-23.06	11.38	-4.53	13.64	0.0	9.11	0.2	0.00000042	0.00005012	0.00001016	0.00842105	A_07_P035826	c_0166	cmr|Hypothetical fimbrial-like protein yadC precursor [c_0166]
yadD	-2.36	10.55	-1.24	11.17	0.0	9.93	0.0	0.00038885	0.00325295	0.04420224	0.56349798	A_07_P035809	c_0162	cmr|Hypothetical protein yadD [c_0162]
yadG	-2.13	10.04	-1.09	10.58	0.0	9.49	0.1	0.00077747	0.00537131	0.06151613	0.63634034	A_07_P002599	b0127	cmr|putative ATP-binding component of a transport system [b0127]
yadH	-2.39	8.61	-1.26	9.23	0.1	7.98	0.1	0.00063342	0.00460415	0.04295732	0.55848059	A_07_P049187	Z0139	cmr|orf, hypothetical protein [Z0139]
yadH	-2.01	8.83	-1.01	9.34	0.0	8.33	0.0	0.00128985	0.00785283	0.07278328	0.67050907	A_07_P002602	b0128	cmr|orf, hypothetical protein [b0128]
yadS	-2.18	8.30	-1.12	8.86	0.1	7.74	0.4	0.00837636	0.03413861	0.05472688	0.60805928	A_07_P002749	b0157	cmr|orf, hypothetical protein [b0157]
yaeQ	-2.21	9.89	-1.14	10.46	0.1	9.32	0.0	0.00067653	0.00482179	0.05484439	0.60807746	A_07_P002906	b0190	cmr|orf, hypothetical protein [b0190]
yafE	+2.67	7.89	+1.42	7.18	0.0	8.60	0.1	0.00020892	0.00206171	0.03402376	0.46988596	A_07_P041461	c_0247	cmr|Hypothetical protein yafE [c_0247]
yafE	+2.01	7.85	+1.00	7.35	0.0	8.35	0.0	0.00126099	0.00772494	0.06240854	0.60614436	A_07_P002978	b0210	cmr|putative biotin synthesis protein [b0210]
yafJ	+2.16	10.42	+1.11	9.87	0.0	10.98	0.1	0.00098287	0.00645412	0.05354675	0.56885290	A_07_P007830	b0223	cmr|putative amidotransferase [b0223]
yafK	-8.20	9.70	-3.04	11.22	0.2	8.18	0.1	0.00000662	0.00019698	0.00053544	0.07330435	A_07_P007831	b0224	cmr|orf, hypothetical protein [b0224]
yafP	-2.10	9.94	-1.07	10.48	0.1	9.41	0.0	0.00107651	0.00688126	0.06437182	0.64717114	A_07_P007881	b0234	cmr|orf, hypothetical protein [b0234]
yafX	-3.02	8.84	-1.59	9.64	0.2	8.04	0.2	0.00043090	0.00350963	0.01968559	0.40250649	A_07_P007946	b0248	cmr|orf, hypothetical protein [b0248]
yafY	-3.03	8.40	-1.60	9.20	0.4	7.60	0.0	0.00151019	0.00883554	0.01874809	0.39408545	A_07_P007962	b0251	cmr|orf, hypothetical protein [b0251]
yagU	+2.33	8.03	+1.22	7.42	0.1	8.64	0.0	0.00056165	0.00422489	0.04508194	0.52653561	A_07_P030901	c_0399	cmr|Hypothetical protein yagU [c_0399]
yagU	+2.28	7.76	+1.19	7.16	0.0	8.35	0.1	0.00068698	0.00486692	0.04726944	0.53733574	A_07_P043568	Z0353	cmr|orf, hypothetical protein [Z0353]
yahA	-5.38	8.51	-2.43	9.73	0.1	7.30	0.0	0.00000956	0.00024464	0.00241235	0.16441558	A_07_P008268	b0315	cmr|orf, hypothetical protein [b0315]
yahK	-2.02	7.58	-1.01	8.09	0.2	7.08	0.1	0.00394759	0.01879943	0.07279662	0.66984804	A_07_P008320	b0325	cmr|putative oxidoreductase [b0325]
yahM	-10.81	9.41	-3.43	11.12	0.1	7.69	0.0	0.00000083	0.00006559	0.00012513	0.03788462	A_07_P008330	b0327	cmr|orf, hypothetical protein [b0327]
yahN	+2.10	8.25	+1.07	7.72	0.3	8.79	0.1	0.00388024	0.01854039	0.05934705	0.59589286	A_07_P008332	b0328	cmr|putative cytochrome subunit of dehydrogenase [b0328]
yaiA	-3.97	12.30	-1.99	13.29	0.1	11.31	0.4	0.00043853	0.00355884	0.00765498	0.28093240	A_07_P021060	b0389	cmr|orf, hypothetical protein [b0389]
yaiE	+2.20	10.68	+1.14	10.11	0.0	11.25	0.2	0.00114068	0.00715494	0.05156568	0.55989655	A_07_P021069	b0391	cmr|orf, hypothetical protein [b0391]
yaiI	+2.97	10.10	+1.57	9.32	0.5	10.88	0.1	0.00321064	0.01595085	0.02910124	0.44097209	A_07_P021047	b0387	cmr|orf, hypothetical protein [b0387]
yaiL	+2.83	7.91	+1.50	7.16	0.2	8.66	0.0	0.00040614	0.00336751	0.03064024	0.45000000	A_07_P031039	c_0462	cmr|Hypothetical protein yaiL [c_0462]
yaiO	-2.94	7.93	-1.56	8.71	0.2	7.16	0.0	0.00018792	0.00191495	0.02116552	0.41810540	A_07_P020918	b0358	cmr|orf, hypothetical protein [b0358]
yaiZ	-2.24	11.92	-1.17	12.51	0.2	11.34	0.3	0.00530568	0.02391428	0.05170414	0.59245269	A_07_P035439	c_0486	cmr|Hypothetical protein yaiZ [c_0486]
yajC	+9.15	9.99	+3.19	8.39	0.3	11.59	0.1	0.00000637	0.00019181	0.00302020	0.15956376	A_07_P021148	b0407	cmr|orf, hypothetical protein [b0407]
ybaA	-2.48	8.27	-1.31	8.92	0.2	7.62	0.0	0.00067646	0.00482179	0.03851753	0.53194737	A_07_P021388	b0456	cmr|orf, hypothetical protein [b0456]
ybaB	+3.57	12.11	+1.84	11.19	0.0	13.03	0.1	0.00004505	0.00068369	0.01966590	0.36771971	A_07_P021461	b0471	cmr|orf, hypothetical protein [b0471]
ybaC	-14.58	10.47	-3.87	12.41	0.1	8.54	0.1	0.00000049	0.00005264	0.00004383	0.02090909	A_07_P031201	c_0596	cmr|Acetyl esterase [c_0596]
ybaK	+2.06	8.77	+1.04	8.24	0.1	9.29	0.1	0.00153759	0.00896468	0.05946900	0.59597708	A_07_P021513	b0481	cmr|orf, hypothetical protein [b0481]
ybaL	-2.80	10.53	-1.49	11.28	0.1	9.79	0.2	0.00049507	0.00387531	0.02448806	0.45092398	A_07_P021498	b0478	cmr|putative transport protein [b0478]
ybaQ	-10.71	10.58	-3.42	12.29	0.1	8.86	0.0	0.00000090	0.00006894	0.00013529	0.03736842	A_07_P021524	b0483	cmr|orf, hypothetical protein [b0483]
ybaV	+3.01	10.35	+1.59	9.56	0.3	11.15	0.1	0.00042326	0.00347436	0.02725038	0.42945946	A_07_P021322	b0442	cmr|orf, hypothetical protein [b0442]
ybaY	-2.41	10.13	-1.27	10.76	0.1	9.50	0.1	0.00058966	0.00437079	0.04138211	0.54887953	A_07_P021380	b0453	cmr|glycoprotein/polysaccharide metabolism [b0453]
ybbB	-2.34	8.47	-1.23	9.08	0.1	7.85	0.2	0.00157829	0.00915909	0.04581110	0.56970774	A_07_P016305	b0503	cmr|putative capsule anchoring protein [b0503]
ybbJ	-2.33	9.02	-1.22	9.63	0.1	8.42	0.2	0.00142935	0.00845252	0.04668699	0.57469898	A_07_P043936	Z0641	cmr|orf, hypothetical protein [Z0641]

ybbJ	-2.29	10.67	-1.19	11.26	0.0	10.07	0.0	0.00050020	0.00390441	0.04888211	0.58303030	A_07_P031218	c_0609	cmr|Hypothetical protein ybbJ [c_0609]
ybbT	+3.97	8.72	+1.99	7.72	0.3	9.71	0.2	0.00015039	0.00162391	0.01582698	0.33948229	A_07_P031224	c_0620	cmr|Ureidoglycolate hydrolase [c_0620]
ybbW	+3.08	8.45	+1.62	7.64	0.2	9.26	0.1	0.00021567	0.00211521	0.02586255	0.42414583	A_07_P016343	b0511	cmr|putative transport protein [b0511]
ybcJ	-2.17	9.72	-1.12	10.28	0.1	9.16	0.5	0.01307121	0.04880085	0.05890434	0.62366510	A_07_P016429	b0528	cmr|orf, hypothetical protein [b0528]
ybcM	-3.25	11.69	-1.70	12.54	0.0	10.84	0.0	0.00005994	0.00083813	0.01491870	0.36642746	A_07_P016496	b0546	cmr|putative ARAC-type regulatory protein [b0546]
ybcO	+4.80	11.96	+2.26	10.83	0.0	13.09	0.1	0.00001273	0.00029390	0.01100229	0.28631405	A_07_P016514	b0549	cmr|orf, hypothetical protein [b0549]
ybcU	+2.02	7.84	+1.02	7.33	0.1	8.35	0.1	0.00144852	0.00853689	0.06198996	0.60581626	A_07_P038504	c_1564	cmr|Bor protein homolog from lambdoid prophage DLP12 [c_1564]
ybcW	-2.15	10.90	-1.10	11.46	0.1	10.35	0.2	0.00252223	0.01324992	0.05985328	0.62863909	A_07_P016559	b0559	cmr|orf, hypothetical protein [b0559]
ybdD	+5.68	8.78	+2.51	7.52	0.0	10.03	0.1	0.00000807	0.00022400	0.00775661	0.24670707	A_07_P010317	ABE-0285033	asap|conserved protein [b4512]
ybdJ	-2.00	8.77	-1.00	9.27	0.0	8.27	0.0	0.00139598	0.00831253	0.07425051	0.67533218	A_07_P016663	b0580	cmr|orf, hypothetical protein [b0580]
ybdM	-2.47	7.99	-1.30	8.64	0.1	7.34	0.2	0.00061794	0.00452296	0.03802718	0.52842012	A_07_P016765	b0601	cmr|orf, hypothetical protein [b0601]
ybdN	-5.89	10.59	-2.56	11.87	0.0	9.31	0.1	0.00000617	0.00018940	0.00176512	0.13825871	A_07_P016768	b0602	cmr|orf, hypothetical protein [b0602]
ybeA	-4.00	12.22	-2.00	13.22	0.0	11.22	0.1	0.00003810	0.00060530	0.00694614	0.26738386	A_07_P009747	b0636	cmr|orf, hypothetical protein [b0636]
ybeB	-4.04	13.24	-2.01	14.24	0.0	12.23	0.1	0.00002500	0.00045582	0.00680450	0.26451852	A_07_P009752	b0637	cmr|orf, hypothetical protein [b0637]
ybeJ	+2.94	11.92	+1.56	11.14	0.1	12.70	0.0	0.00011657	0.00133244	0.02783664	0.43392079	A_07_P044035	Z0805	cmr|putative periplasmic binding transport protein [Z0805]
ybeL	+2.55	11.05	+1.35	10.38	0.3	11.73	0.2	0.00175638	0.00998282	0.03793128	0.48710440	A_07_P009784	b0643	cmr|putative alpha helical protein [b0643]
ybeY	+3.99	10.52	+2.00	9.52	0.1	11.52	0.1	0.00003563	0.00057774	0.01592035	0.33917456	A_07_P009856	b0659	cmr|orf, hypothetical protein [b0659]
ybeZ	+3.07	10.51	+1.62	9.71	0.0	11.32	0.1	0.00010030	0.00120402	0.02570376	0.42419287	A_07_P009865	b0660	cmr|putative ATP-binding protein in pho regulon [b0660]
ybfA	-7.31	11.14	-2.87	12.58	0.0	9.71	0.2	0.00000599	0.00018688	0.00078252	0.08800000	A_07_P010014	b0699	cmr|orf, hypothetical protein [b0699]
ybfE	-3.31	11.64	-1.73	12.50	0.1	10.77	0.0	0.00006699	0.00090852	0.01404599	0.35667742	A_07_P009941	b0685	cmr|orf, hypothetical protein [b0685]
ybfL	-2.80	10.53	-1.48	11.27	0.4	9.79	0.0	0.00192026	0.01067532	0.02477007	0.45452214	A_07_P010041	b0705	cmr|putative receptor protein [b0705]
ybgA	-6.50	9.45	-2.70	10.80	0.1	8.10	0.1	0.00000633	0.00019153	0.00124174	0.11568047	A_07_P033376	c_0786	cmr|Hypothetical protein ybgA [c_0786]
ybgC	-2.12	11.08	-1.08	11.62	0.1	10.53	0.0	0.00106541	0.00683382	0.06237551	0.63893299	A_07_P010198	b0736	cmr|orf, hypothetical protein [b0736]
ybgE	+9.14	9.13	+3.19	7.54	0.1	10.73	0.1	0.00000172	0.00009748	0.00306529	0.16086667	A_07_P010194	b0735	cmr|orf, hypothetical protein [b0735]
ybgO	+2.34	7.54	+1.23	6.93	0.1	8.15	0.3	0.00190200	0.01058953	0.04547320	0.52914265	A_07_P010097	b0716	cmr|orf, hypothetical protein [b0716]
ybgS	+2.05	7.46	+1.03	6.94	0.1	7.98	0.1	0.00210628	0.01149838	0.05948107	0.59571883	A_07_P010246	b0753	cmr|putative homeobox protein [b0753]
ybhA	+2.02	9.86	+1.01	9.35	0.1	10.37	0.2	0.00319093	0.01587525	0.06353532	0.61330472	A_07_P046367	Z0936	cmr|putative phosphatase [Z0936]
ybhC	+2.57	7.98	+1.36	7.30	0.0	8.66	0.3	0.00092192	0.00614247	0.03718115	0.48619601	A_07_P051991	Z0943	cmr|putative pectinesterase [Z0943]
ybhC	+2.14	8.53	+1.10	7.98	0.1	9.08	0.2	0.00239787	0.01271122	0.05598577	0.57913272	A_07_P004315	b0772	cmr|putative pectinesterase [b0772]
ybhI	-3.80	9.90	-1.92	10.86	0.2	8.93	0.1	0.00014984	0.00161942	0.00865981	0.30230599	A_07_P004302	b0770	cmr|putative membrane pump protein [b0770]
ybhN	-3.09	9.50	-1.63	10.32	0.0	8.69	0.2	0.00023482	0.00224565	0.01802464	0.38714870	A_07_P004392	b0788	cmr|orf, hypothetical protein [b0788]
ybhQ	+2.20	10.34	+1.14	9.78	0.1	10.91	0.1	0.00098069	0.00644673	0.05130589	0.55977824	A_07_P004406	b0791	cmr|orf, hypothetical protein [b0791]
ybiB	+4.09	12.14	+2.03	11.12	0.1	13.15	0.2	0.00004516	0.00068369	0.01522104	0.33329624	A_07_P004455	b0800	cmr|putative enzyme [b0800]
ybiB	+3.99	10.35	+1.99	9.35	0.1	11.34	0.1	0.00003284	0.00054895	0.01596354	0.33917679	A_07_P046583	Z1021	cmr|putative enzyme [Z1021]
ybiB	+5.17	11.40	+2.37	10.21	0.1	12.58	0.1	0.00001668	0.00034825	0.00944487	0.26841155	A_07_P041584	c_0884	cmr|Hypothetical protein ybiB [c_0884]
ybiC	+5.09	9.04	+2.35	7.86	0.1	10.21	0.1	0.00001519	0.00032668	0.00979738	0.27300885	A_07_P042097	c_0885	cmr|Hypothetical oxidoreductase ybiC [c_0885]
ybiC	+2.05	9.01	+1.04	8.49	0.0	9.53	0.1	0.00148168	0.00869341	0.06026486	0.59824086	A_07_P004457	b0801	cmr|putative dehydrogenase [b0801]
ybiH	+2.35	7.58	+1.23	6.96	0.2	8.20	0.3	0.00427985	0.02012001	0.04609566	0.53050439	A_07_P004432	b0796	cmr|putative transcriptional regulator [b0796]
ybiJ	-4.61	9.85	-2.20	10.95	0.2	8.75	0.0	0.00003067	0.00052425	0.00417492	0.21135048	A_07_P004463	b0802	cmr|orf, hypothetical protein [b0802]
ybiO	-4.49	9.52	-2.17	10.60	0.1	8.44	0.2	0.00005617	0.00079813	0.00474530	0.22639394	A_07_P004491	b0808	cmr|putative transport protein [b0808]
ybiR	-2.56	9.86	-1.36	10.54	0.1	9.18	0.2	0.00058458	0.00434342	0.03346354	0.50658654	A_07_P004542	b0818	cmr|orf, hypothetical protein [b0818]
ybiS	-3.18	10.40	-1.67	11.23	0.0	9.56	0.1	0.00010159	0.00121353	0.01606898	0.37703428	A_07_P004547	b0819	cmr|orf, hypothetical protein [b0819]
ybiS	-3.32	12.09	-1.73	12.96	0.1	11.23	0.1	0.00008685	0.00108440	0.01374555	0.35245928	A_07_P042114	c_0905	cmr|Protein ybiS precursor [c_0905]
ybiT	-13.54	10.35	-3.76	12.23	0.1	8.47	0.0	0.00000051	0.00005264	0.00004700	0.02176471	A_07_P004555	b0820	cmr|putative ATP-binding component of a transport system [b0820]
ybjG	-43.08	10.68	-5.43	13.40	0.0	7.97	0.1	0.00000007	0.00003344	0.00000064	0.00250000	A_07_P004660	b0841	cmr|orf, hypothetical protein [b0841]
ybjL	-12.66	10.04	-3.66	11.87	0.1	8.21	0.2	0.00000212	0.00010505	0.00005780	0.02275000	A_07_P004688	b0847	cmr|putative transport protein [b0847]
ybjM	-3.48	9.18	-1.80	10.08	0.1	8.28	0.1	0.00007188	0.00095259	0.01162792	0.33468007	A_07_P004692	b0848	cmr|orf, hypothetical protein [b0848]
ybjN	+3.04	10.43	+1.61	9.63	0.1	11.24	0.3	0.00046740	0.00372408	0.02651804	0.42689162	A_07_P004718	b0853	cmr|putative sensory transduction regulator [b0853]
ybjP	+2.30	9.13	+1.20	8.52	0.1	9.73	0.2	0.00107646	0.00688126	0.04669842	0.53431686	A_07_P052022	Z1095	cmr|putative enzyme [Z1095]
ybjT	+2.53	7.76	+1.34	7.09	0.0	8.43	0.2	0.00062009	0.00453656	0.03788046	0.48844390	A_07_P004800	b0869	cmr|putative dTDP-glucose enzyme [b0869]
ybjX	-5.14	10.65	-2.36	11.83	0.0	9.47	0.2	0.00002142	0.00041080	0.00280742	0.17539683	A_07_P004839	b0877	cmr|putative enzyme [b0877]
ybjX	-6.09	12.32	-2.61	13.62	0.0	11.02	0.1	0.00000580	0.00018220	0.00154789	0.12826316	A_07_P042136	c_1012	cmr|Hypothetical protein ybjX [c_1012]
ycaD	-2.28	8.23	-1.19	8.82	0.1	7.64	0.1	0.00103015	0.00666690	0.04975546	0.58546338	A_07_P033821	c_1037	cmr|Hypothetical protein ycaD [c_1037]
ycaO	+2.05	8.90	+1.03	8.38	0.1	9.42	0.1	0.00135606	0.00814256	0.06034045	0.59823678	A_07_P051726	Z1251	cmr|orf, hypothetical protein [Z1251]
ycaO	+2.76	9.16	+1.47	8.43	0.0	9.90	0.2	0.00028692	0.00260187	0.03157774	0.45611009	A_07_P004973	b0905	cmr|orf, hypothetical protein [b0905]
ycbF	-3.43	11.57	-1.78	12.46	0.1	10.68	0.3	0.00029387	0.00264683	0.01242505	0.33669535	A_07_P005167	b0944	cmr|putative chaperone [b0944]
ycbG	+4.43	9.46	+2.15	8.38	0.0	10.53	0.1	0.00001640	0.00034470	0.01303608	0.30770615	A_07_P005226	b0956	cmr|putative dehydrogenase [b0956]
ycbJ	+4.14	9.42	+2.05	8.39	0.0	10.44	0.1	0.00002684	0.00047665	0.01493458	0.32977560	A_07_P005042	b0919	cmr|orf, hypothetical protein [b0919]
ycbQ	-2.38	9.01	-1.25	9.64	0.4	8.39	0.0	0.00582968	0.02575990	0.04167365	0.54904603	A_07_P005137	b0938	cmr|putative fimbrial-like protein [b0938]
yccJ	+3.52	9.60	+1.82	8.69	0.3	10.50	0.1	0.00026536	0.00245180	0.01983041	0.36947929	A_07_P001246	b1003	cmr|orf, hypothetical protein [b1003]
yccX	-3.91	12.93	-1.97	13.91	0.1	11.94	0.2	0.00009164	0.00112718	0.00743712	0.27615566	A_07_P005289	b0968	cmr|orf, hypothetical protein [b0968]
ycdT	-2.40	11.28	-1.26	11.91	0.1	10.65	0.1	0.00058366	0.00434242	0.04098895	0.54504223	A_07_P041100	c_1164	cmr|Hypothetical protein ycdT [c_1164]
ycdT	-7.95	10.59	-2.99	12.08	0.0	9.09	0.1	0.00000211	0.00010505	0.00058054	0.07745763	A_07_P001352	b1025	cmr|orf, hypothetical protein [b1025]
ycdZ	-5.86	9.63	-2.55	10.91	0.1	8.36	0.5	0.00019172	0.00194108	0.00184007	0.14200980	A_07_P001391	b1036	cmr|orf, hypothetical protein [b1036]
yceA	-2.09	10.04	-1.07	10.57	0.1	9.50	0.1	0.00113600	0.00714267	0.06471989	0.64818702	A_07_P001482	b1055	cmr|orf, hypothetical protein [b1055]
yceF	+2.54	7.80	+1.34	7.13	0.0	8.47	0.1	0.00037199	0.00314193	0.03763783	0.48691044	A_07_P001639	b1087	cmr|orf, hypothetical protein [b1087]
yceH	+3.88	8.70	+1.96	7.72	0.2	9.68	0.1	0.00005108	0.00074921	0.01677782	0.34484334	A_07_P040780	c_1334	cmr|Hypothetical protein yceH [c_1334]
yceH	+2.86	8.56	+1.52	7.80	0.1	9.32	0.2	0.00030210	0.00269224	0.02988504	0.44387736	A_07_P001540	b1067	cmr|orf, hypothetical protein [b1067]
yceH	+2.15	8.09	+1.10	7.54	0.2	8.64	0.2	0.00238038	0.01266107	0.05525534	0.57535714	A_07_P052072	Z1704	cmr|orf, hypothetical protein [Z1704]
yceM	+2.09	10.19	+1.07	9.66	0.0	10.72	0.0	0.00093527	0.00620521	0.05675813	0.58367080	A_07_P001544	b1068	cmr|putative virulence factor [b1068]
yceQ	-2.00	8.80	-1.00	9.30	0.1	8.30	0.2	0.00460831	0.02138323	0.07464558	0.67541379	A_07_P001627	b1085	cmr|orf, hypothetical protein [b1085]
ycfJ	-7.12	9.75	-2.83	11.16	0.1	8.33	0.0	0.00000298	0.00012546	0.00092607	0.09720000	A_07_P001753	b1110	cmr|orf, hypothetical protein [b1110]
ycfL	+4.14	9.66	+2.05	8.63	0.3	10.68	0.5	0.00111862	0.00707230	0.01466972	0.32713881	A_07_P001721	b1104	cmr|orf, hypothetical protein [b1104]
ycfP	+2.90	13.78	+1.54	13.01	0.1	14.54	0.3	0.00073774	0.00514389	0.02972180	0.44396584	A_07_P001743	b1108	cmr|orf, hypothetical protein [b1108]
ycfQ	-8.22	10.92	-3.04	12.44	0.1	9.40	0.2	0.00000475	0.00016625	0.00045668	0.06783019	A_07_P001759	b1111	cmr|orf, hypothetical protein [b1111]
ycfZ	-2.10	7.58	-1.07	8.12	0.0	7.05	0.1	0.00144656	0.00853300	0.06364710	0.64565722	A_07_P001806	b1121	cmr|homolog of virulence factor [b1121]
ycgK	+4.81	10.51	+2.26	9.38	0.1	11.64	0.1	0.00001306	0.00029749	0.01117950	0.28759804	A_07_P006843	b1178	cmr|orf, hypothetical protein [b1178]
ycgM	+2.76	9.26	+1.47	8.53	0.1	9.99	0.1	0.00029898	0.00267603	0.03207571	0.45950864	A_07_P006851	b1180	cmr|putative isomerase [b1180]
ycgV	+2.12	7.42	+1.08	6.88	0.1	7.96	0.3	0.00303999	0.01530590	0.05647294	0.58302295	A_07_P006961	b1202	cmr|putative adhesion and penetration protein [b1202]
ycgZ	+6.19	9.46	+2.63	8.14	0.2	10.77	0.1	0.00001697	0.00035267	0.00664761	0.23309577	A_07_P042226	c_1607	cmr|Hypothetical protein ycgZ [c_1607]
ychA	+3.47	10.21	+1.80	9.31	0.2	11.11	0.1	0.00011741	0.00133755	0.02049416	0.37606061	A_07_P007024	b1214	cmr|orf, hypothetical protein [b1214]
ychH	+2.54	10.61	+1.35	9.94	0.0	11.29	0.0	0.00024754	0.00233088	0.03743648	0.48670520	A_07_P006978	b1205	cmr|orf, hypothetical protein [b1205]
ychM	-3.22	8.88	-1.69	9.72	0.0	8.04	0.1	0.00010443	0.00123820	0.01556402	0.37296804	A_07_P038791	c_1664	cmr|Putative sulfate transporter ychM [c_1664]
ychM	-2.99	8.80	-1.58	9.59	0.2	8.01	0.1	0.00039998	0.00333015	0.01941184	0.39898172	A_07_P050776	Z1977	cmr|orf, hypothetical protein [Z1977]
ychM	-2.48	8.76	-1.31	9.42	0.4	8.10	0.2	0.00336153	0.01650778	0.03733422	0.52575134	A_07_P006981	b1206	cmr|orf, hypothetical protein [b1206]
ychN	+3.40	9.34	+1.77	8.45	0.3	10.22	0.2	0.00047753	0.00378609	0.02159362	0.38809361	A_07_P007047	b1219	cmr|orf, hypothetical protein [b1219]
ychN	+2.64	9.09	+1.40	8.39	0.2	9.79	0.4	0.00261341	0.01360180	0.03583778	0.47897284	A_07_P050787	Z1994	cmr|orf, hypothetical protein [Z1994]
ychO	-2.60	8.26	-1.38	8.94	0.2	7.57	0.3	0.00286284	0.01461735	0.03085810	0.49473523	A_07_P007051	b1220	cmr|putative factor [b1220]
yciG	-4.30	12.58	-2.10	13.64	0.2	11.53	0.2	0.00009081	0.00111789	0.00556784	0.24417827	A_07_P010380	b1259	cmr|orf, hypothetical protein [b1259]
yciH	+2.53	11.49	+1.34	10.82	0.1	12.16	0.1	0.00046603	0.00371502	0.03799860	0.48717427	A_07_P041123	c_1751	cmr|Protein yciH [c_1751]
yciH	+2.74	11.69	+1.45	10.96	0.0	12.41	0.1	0.00018297	0.00187662	0.03205285	0.45959927	A_07_P014394	b1282	cmr|orf, hypothetical protein [b1282]
yciI	+15.42	10.25	+3.95	8.27	0.1	12.22	0.2	0.00000105	0.00007542	0.00096481	0.09493750	A_07_P007191	b1251	cmr|orf, hypothetical protein [b1251]
yciI	+15.10	10.55	+3.92	8.59	0.1	12.51	0.1	0.00000045	0.00005132	0.00100483	0.09765432	A_07_P050796	Z2029	cmr|orf, hypothetical protein [Z2029]
yciK	+2.87	10.99	+1.52	10.23	0.1	11.75	0.2	0.00031156	0.00275265	0.02974911	0.44353220	A_07_P014337	b1271	cmr|putative oxidoreductase [b1271]
yciM	-4.37	11.05	-2.13	12.12	0.4	9.99	0.3	0.00062163	0.00454361	0.00535442	0.23881020	A_07_P014385	b1280	cmr|putative heat shock protein [b1280]
yciN	+2.22	10.30	+1.15	9.73	0.0	10.88	0.0	0.00062683	0.00457316	0.04986789	0.55721789	A_07_P014346	b1273	cmr|orf, hypothetical protein [b1273]
yciO	-2.27	8.91	-1.18	9.50	0.0	8.32	0.1	0.00054188	0.00411349	0.05017086	0.58640683	A_07_P044117	Z2543	cmr|orf, hypothetical protein [Z2543]
yciO	-3.14	10.68	-1.65	11.51	0.0	9.85	0.2	0.00017562	0.00182148	0.01672955	0.37734957	A_07_P038891	c_1732	cmr|Protein yciO [c_1732]
yciO	-2.81	9.63	-1.49	10.38	0.0	8.89	0.2	0.00026834	0.00247207	0.02457698	0.45150525	A_07_P014319	b1267	cmr|orf, hypothetical protein [b1267]
yciW	-2.65	12.25	-1.41	12.95	0.3	11.54	0.2	0.00163478	0.00943339	0.03034426	0.49049281	A_07_P014418	b1287	cmr|putative oxidoreductase [b1287]
ycjD	-3.59	9.23	-1.85	10.15	0.1	8.30	0.1	0.00007642	0.00098942	0.01043128	0.32265226	A_07_P014428	b1289	cmr|orf, hypothetical protein [b1289]
ycjF	+2.87	7.82	+1.52	7.06	0.3	8.58	0.1	0.00113876	0.00715430	0.03002033	0.44462841	A_07_P042267	c_1794	cmr|Hypothetical protein ycjF [c_1794]
ycjX	-2.49	11.70	-1.32	12.36	0.0	11.04	0.1	0.00041856	0.00344113	0.03697282	0.52536101	A_07_P048461	Z2458	cmr|putative EC 2.1 enzymes [Z2458]
ydaC	-2.31	7.76	-1.21	8.36	0.3	7.16	0.1	0.00284543	0.01454495	0.04610391	0.57244479	A_07_P014713	b1347	cmr|orf, hypothetical protein [b1347]
ydaE	-2.38	8.98	-1.25	9.61	0.2	8.36	0.2	0.00110982	0.00703703	0.04342924	0.55861928	A_07_P010386	ABE-0285047	asap|Rac prophage; conserved protein [b4526]
ydaQ	-2.38	9.76	-1.25	10.39	0.1	9.14	0.0	0.00059835	0.00442480	0.04337652	0.55839738	A_07_P048430	Z2414	cmr|unknown protein encoded within prophage CP-933R [Z2414]
ydaV	-3.37	9.68	-1.75	10.56	0.1	8.81	0.3	0.00024769	0.00233088	0.01329141	0.34588430	A_07_P014777	b1360	cmr|putative DNA replication factor [b1360]
ydbH	+2.09	7.92	+1.07	7.39	0.1	8.45	0.0	0.00105061	0.00676135	0.05702680	0.58414444	A_07_P014885	b1381	cmr|orf, hypothetical protein [b1381]
ydbJ	+3.07	10.59	+1.62	9.79	0.1	11.40	0.1	0.00009527	0.00115468	0.02568661	0.42435467	A_07_P010409	ABE-0285050	asap|predicted protein [b4529]
ydbK	+4.86	8.92	+2.28	7.78	0.1	10.06	0.1	0.00001824	0.00037077	0.01067264	0.28335582	A_07_P014866	b1378	cmr|putative oxidoreductase, Fe-S subunit [b1378]
ydcH	+15.38	9.49	+3.94	7.52	0.0	11.47	0.1	0.00000045	0.00005132	0.00096227	0.09528302	A_07_P009222	b1426	cmr|orf, hypothetical protein [b1426]
ydcI	-2.12	8.65	-1.08	9.20	0.1	8.11	0.0	0.00089440	0.00602286	0.06250572	0.63943470	A_07_P009204	b1422	cmr|putative transcriptional regulator LYSR-type [b1422]
ydcM	-2.06	9.15	-1.04	9.67	0.1	8.63	0.4	0.00808694	0.03323866	0.06840892	0.65712630	A_07_P009252	b1432	cmr|putative virulence protein [b1432]
ydcY	+4.13	11.98	+2.04	10.96	0.0	13.01	0.0	0.00002003	0.00039347	0.01491870	0.32988764	A_07_P009325	b1446	cmr|orf, hypothetical protein [b1446]
yddA	-2.23	7.58	-1.15	8.15	0.3	7.00	0.1	0.00271935	0.01399726	0.05305323	0.59918938	A_07_P009570	b1496	cmr|putative ATP-binding component of a transport system [b1496]
yddB	-2.59	7.45	-1.37	8.14	0.4	6.76	0.1	0.00235532	0.01254469	0.03151804	0.49771314	A_07_P048054	Z2213	cmr|orf, hypothetical protein [Z2213]
yddM	+2.00	8.71	+1.00	8.20	0.1	9.21	0.1	0.00175621	0.00998282	0.06377668	0.61450428	A_07_P034960	c_1909	cmr|Hypothetical protein yddM [c_1909]
ydeA	-4.98	8.38	-2.32	9.53	0.2	7.22	0.0	0.00002181	0.00041529	0.00320821	0.19060377	A_07_P020286	b1528	cmr|putative resistance / regulatory protein [b1528]
ydeN	+2.60	8.59	+1.38	7.90	0.2	9.28	0.1	0.00049385	0.00386823	0.03591527	0.47919492	A_07_P009578	b1498	cmr|putative sulfatase [b1498]
ydeT	-2.20	9.34	-1.14	9.91	0.1	8.77	0.0	0.00093750	0.00621733	0.05530869	0.60808659	A_07_P009612	b1505	cmr|putative outer membrane protein [b1505]
ydeU	-2.02	9.08	-1.02	9.59	0.2	8.57	0.1	0.00253452	0.01328788	0.07255399	0.66996481	A_07_P032527	c_2894	cmr|Hypothetical protein ydeU [c_2894]
ydfG	+2.90	8.46	+1.54	7.69	0.0	9.23	0.1	0.00012377	0.00139716	0.02850927	0.43918787	A_07_P020342	b1539	cmr|putative oxidoreductase [b1539]
ydfH	-2.02	9.15	-1.01	9.66	0.1	8.64	0.1	0.00248250	0.01305865	0.07285315	0.66919487	A_07_P052445	Z2157	cmr|orf, hypothetical protein [Z2157]
ydfM	-7.98	8.71	-3.00	10.21	0.1	7.21	0.1	0.00000255	0.00011704	0.00058117	0.07689076	A_07_P031275	c_0649	cmr|Tail fiber assembly protein homolog from lambdoid [c_0649]
ydfZ	+2.77	11.37	+1.47	10.63	0.2	12.10	0.2	0.00042640	0.00349284	0.03210747	0.45954545	A_07_P020351	b1541	cmr|orf, hypothetical protein [b1541]
ydgA	+2.02	9.19	+1.01	8.69	0.1	9.70	0.2	0.00228163	0.01223501	0.06154599	0.60447910	A_07_P044404	Z2617	cmr|orf, hypothetical protein [Z2617]
ydgA	+2.48	9.49	+1.31	8.83	0.1	10.15	0.0	0.00031579	0.00277757	0.03952490	0.49465819	A_07_P035103	c_2006	cmr|Hypothetical protein ydgA [c_2006]
ydgH	+3.80	9.74	+1.93	8.78	0.2	10.71	0.1	0.00008859	0.00109740	0.01722815	0.34863753	A_07_P020654	b1604	cmr|orf, hypothetical protein [b1604]
ydgJ	+3.99	8.78	+2.00	7.79	0.2	9.78	0.1	0.00006335	0.00087114	0.01588605	0.33890244	A_07_P035111	c_2016	cmr|Hypothetical oxidoreductase ydgJ [c_2016]
ydgJ	+2.72	7.94	+1.45	7.22	0.0	8.67	0.1	0.00023139	0.00222327	0.03247332	0.46142599	A_07_P020751	b1624	cmr|orf, hypothetical protein [b1624]
ydgK	-3.43	9.14	-1.78	10.03	0.1	8.25	0.1	0.00007081	0.00094322	0.01214558	0.33904255	A_07_P020765	b1626	cmr|orf, hypothetical protein [b1626]
ydhF	+2.36	9.44	+1.24	8.82	0.1	10.06	0.1	0.00098886	0.00648153	0.04452045	0.52269202	A_07_P020869	b1647	cmr|orf, hypothetical protein [b1647]
ydhI	-3.61	8.22	-1.85	9.14	0.2	7.29	0.0	0.00008179	0.00103599	0.01029090	0.32468938	A_07_P020850	b1643	cmr|orf, hypothetical protein [b1643]
ydhJ	-2.17	7.68	-1.12	8.24	0.2	7.12	0.2	0.00366642	0.01773944	0.05786204	0.61887228	A_07_P020852	b1644	cmr|putative membrane protein [b1644]
ydhJ	-2.24	7.67	-1.17	8.26	0.2	7.09	0.1	0.00161761	0.00936312	0.05183244	0.59134058	A_07_P020852	b1644	cmr|putative membrane protein [b1644]
ydhJ	-2.71	7.96	-1.44	8.68	0.1	7.24	0.2	0.00031727	0.00278587	0.02760036	0.47542670	A_07_P020852	b1644	cmr|putative membrane protein [b1644]
ydhJ	-2.42	7.81	-1.27	8.45	0.1	7.18	0.1	0.00043055	0.00350857	0.04145008	0.54793451	A_07_P020852	b1644	cmr|putative membrane protein [b1644]
ydhP	-2.70	7.80	-1.43	8.51	0.3	7.08	0.3	0.00200054	0.01105143	0.02766578	0.47344565	A_07_P018348	b1657	cmr|putative transport protein [b1657]
ydhR	+2.30	8.94	+1.20	8.34	0.1	9.54	0.1	0.00078297	0.00539515	0.04689215	0.53497826	A_07_P018387	b1667	cmr|orf, hypothetical protein [b1667]
ydhS	-3.91	9.00	-1.97	9.98	0.5	8.01	0.1	0.00120552	0.00746644	0.00797066	0.28716247	A_07_P018392	b1668	cmr|orf, hypothetical protein [b1668]
ydhU	+2.09	8.32	+1.06	7.79	0.0	8.86	0.1	0.00137640	0.00822395	0.05799479	0.58869761	A_07_P018401	b1670	cmr|orf, hypothetical protein [b1670]
ydhV	+3.69	8.55	+1.88	7.61	0.1	9.49	0.6	0.00222678	0.01201042	0.01826092	0.35276074	A_07_P018419	b1673	cmr|orf, hypothetical protein [b1673]
ydhW	+2.48	7.99	+1.31	7.33	0.1	8.64	0.0	0.00031947	0.00279739	0.03936674	0.49425040	A_07_P018411	b1672	cmr|orf, hypothetical protein [b1672]
ydhX	+3.53	8.01	+1.82	7.10	0.2	8.92	0.1	0.00011554	0.00132585	0.02009909	0.37272085	A_07_P018406	b1671	cmr|putative oxidoreductase, Fe-S subunit [b1671]
ydhZ	+2.12	8.36	+1.08	7.82	0.1	8.91	0.3	0.00347967	0.01702419	0.05662665	0.58308044	A_07_P018430	b1675	cmr|orf, hypothetical protein [b1675]
ydiA	+6.87	8.51	+2.78	7.12	0.1	9.90	0.1	0.00000459	0.00016295	0.00530551	0.20830424	A_07_P018569	b1703	cmr|orf, hypothetical protein [b1703]
ydiI	+3.34	9.46	+1.74	8.59	0.1	10.33	0.2	0.00023038	0.00221839	0.02179306	0.39034130	A_07_P018484	b1686	cmr|orf, hypothetical protein [b1686]
ydiJ	+3.08	9.55	+1.62	8.73	0.3	10.36	0.1	0.00065997	0.00473805	0.02634591	0.42673868	A_07_P018489	b1687	cmr|putative oxidase [b1687]
ydiU	-2.38	11.27	-1.25	11.89	0.2	10.64	0.3	0.00249232	0.01310152	0.04145071	0.54748322	A_07_P018581	b1706	cmr|orf, hypothetical protein [b1706]
ydiV	-4.10	9.03	-2.03	10.04	0.1	8.01	0.0	0.00002306	0.00043145	0.00642848	0.25687817	A_07_P018587	b1707	cmr|orf, hypothetical protein [b1707]
ydiZ	+2.02	9.54	+1.02	9.04	0.0	10.05	0.0	0.00116633	0.00727690	0.06135798	0.60376250	A_07_P018667	b1724	cmr|orf, hypothetical protein [b1724]
ydjA	+9.85	10.96	+3.30	9.31	0.2	12.61	0.1	0.00000305	0.00012679	0.00264418	0.14921147	A_07_P044447	Z2798	cmr|orf, hypothetical protein [Z2798]
ydjA	+21.67	10.62	+4.44	8.40	0.1	12.84	0.1	0.00000027	0.00004211	0.00038554	0.05620370	A_07_P035242	c_2168	cmr|Protein ydjA [c_2168]
ydjA	+19.87	10.45	+4.31	8.29	0.1	12.60	0.1	0.00000028	0.00004211	0.00051131	0.06491935	A_07_P035242	c_2168	cmr|Protein ydjA [c_2168]
ydjA	+18.82	10.36	+4.23	8.25	0.1	12.48	0.1	0.00000029	0.00004218	0.00058689	0.07162791	A_07_P035242	c_2168	cmr|Protein ydjA [c_2168]
ydjA	+18.00	10.40	+4.17	8.31	0.1	12.48	0.0	0.00000037	0.00004793	0.00067010	0.07644928	A_07_P035242	c_2168	cmr|Protein ydjA [c_2168]
ydjA	+14.39	10.43	+3.85	8.51	0.2	12.36	0.0	0.00000087	0.00006760	0.00112106	0.10261628	A_07_P018872	b1765	cmr|orf, hypothetical protein [b1765]
ydjA	+17.31	10.67	+4.11	8.62	0.1	12.73	0.0	0.00000030	0.00004311	0.00075648	0.08270833	A_07_P035242	c_2168	cmr|Protein ydjA [c_2168]
ydjE	-3.25	8.31	-1.70	9.16	0.1	7.46	0.2	0.00028422	0.00258935	0.01455539	0.36202212	A_07_P041186	c_2173	cmr|Hypothetical metabolite transport protein ydjE [c_2173]
ydjE	-2.77	8.07	-1.47	8.81	0.0	7.34	0.1	0.00019609	0.00196842	0.02585938	0.46317406	A_07_P018893	b1769	cmr|putative transport protein [b1769]
ydjF	-3.52	10.10	-1.81	11.01	0.1	9.20	0.1	0.00007722	0.00099606	0.01119093	0.33056285	A_07_P018898	b1770	cmr|putative DEOR-type transcriptional regulator [b1770]
ydjM	-3.13	8.58	-1.65	9.40	0.1	7.75	0.3	0.00043359	0.00352973	0.01723768	0.37956643	A_07_P018690	b1728	cmr|orf, hypothetical protein [b1728]
ydjN	-7.71	10.42	-2.95	11.90	0.0	8.95	0.3	0.00001176	0.00028105	0.00065041	0.08000000	A_07_P018691	b1729	cmr|part of a kinase [b1729]
ydjN	-4.57	9.67	-2.19	10.76	0.0	8.57	0.2	0.00003215	0.00054058	0.00429179	0.21450794	A_07_P042351	c_2128	cmr|Hypothetical symporter ydjN [c_2128]
ydjR	-2.07	7.77	-1.05	8.30	0.0	7.24	0.1	0.00135659	0.00814265	0.06669842	0.64941249	A_07_P041169	c_2141	cmr|Hypothetical protein ydjR [c_2141]
yeaC	+5.15	12.62	+2.36	11.44	0.1	13.80	0.2	0.00002036	0.00039679	0.00951092	0.26883303	A_07_P015578	b1777	cmr|orf, hypothetical protein [b1777]
yeaD	+5.86	9.43	+2.55	8.16	0.2	10.71	0.6	0.00053100	0.00405438	0.00725800	0.24209746	A_07_P035263	c_2185	cmr|Unknown protein from 2D-page [c_2185]
yeaD	+7.74	9.73	+2.95	8.26	0.3	11.21	0.2	0.00001881	0.00037681	0.00428163	0.18829609	A_07_P035263	c_2185	cmr|Unknown protein from 2D-page [c_2185]
yeaD	+8.61	10.12	+3.11	8.56	0.1	11.67	0.1	0.00000324	0.00013193	0.00345910	0.17125786	A_07_P035263	c_2185	cmr|Unknown protein from 2D-page [c_2185]
yeaD	+8.42	9.85	+3.07	8.31	0.0	11.39	0.1	0.00000186	0.00009954	0.00357978	0.17288344	A_07_P035263	c_2185	cmr|Unknown protein from 2D-page [c_2185]
yeaD	+2.14	7.50	+1.09	6.95	0.2	8.05	0.3	0.00588420	0.02592801	0.05701601	0.58441406	A_07_P044473	Z2820	cmr|orf, hypothetical protein [Z2820]
yeaD	+6.98	9.15	+2.80	7.75	0.0	10.55	0.0	0.00000280	0.00012259	0.00524327	0.20845960	A_07_P015593	b1780	cmr|orf, hypothetical protein [b1780]
yeaD	+8.94	10.12	+3.16	8.54	0.0	11.70	0.2	0.00000315	0.00012975	0.00324251	0.16574675	A_07_P035263	c_2185	cmr|Unknown protein from 2D-page [c_2185]
yeaI	-2.16	8.24	-1.11	8.80	0.1	7.69	0.0	0.00101649	0.00660214	0.05827553	0.62034483	A_07_P015616	b1785	cmr|orf, hypothetical protein [b1785]
yeaJ	-5.72	8.94	-2.52	10.20	0.1	7.68	0.1	0.00000781	0.00021882	0.00200267	0.14733645	A_07_P015623	b1786	cmr|orf, hypothetical protein [b1786]
'yeaJ	-2.13	9.64	-1.09	10.18	0.1	9.09	0.2	0.00180230	0.01018137	0.06114012	0.63579260	A_07_P044476	Z2826	cmr|orf Unknown function [Z2826]
'yeaJ	-2.59	9.53	-1.37	10.21	0.0	8.84	0.1	0.00024834	0.00233429	0.03277058	0.50582353	A_07_P044476	Z2826	cmr|orf Unknown function [Z2826]
'yeaJ	-2.20	9.72	-1.14	10.29	0.3	9.15	0.2	0.00417243	0.01969146	0.05430386	0.60549575	A_07_P044476	Z2826	cmr|orf Unknown function [Z2826]
'yeaJ	-2.74	9.48	-1.45	10.21	0.0	8.76	0.0	0.00016562	0.00174763	0.02720592	0.47329282	A_07_P044476	Z2826	cmr|orf Unknown function [Z2826]
yeaL	-3.14	9.14	-1.65	9.97	0.2	8.32	0.0	0.00015277	0.00164405	0.01685849	0.37917143	A_07_P015638	b1789	cmr|orf, hypothetical protein [b1789]
yeaM	-2.07	9.78	-1.05	10.31	0.1	9.25	0.1	0.00121258	0.00750429	0.06660252	0.65089385	A_07_P015644	b1790	cmr|putative ARAC-type regulatory protein [b1790]
yeaO	+2.56	9.55	+1.36	8.87	0.1	10.23	0.1	0.00029580	0.00265664	0.03696519	0.48538782	A_07_P015655	b1792	cmr|orf, hypothetical protein [b1792]
yeaT	-3.33	7.63	-1.74	8.50	0.0	6.76	0.1	0.00007902	0.00100904	0.01340701	0.34774300	A_07_P015689	b1799	cmr|putative transcriptional regulator LYSR-type [b1799]
yebB	-2.61	10.98	-1.38	11.67	0.3	10.29	0.0	0.00128941	0.00785283	0.03139863	0.49682412	A_07_P035344	c_2276	cmr|Hypothetical protein yebB [c_2276]
yebE	-10.91	10.90	-3.45	12.63	0.0	9.18	0.1	0.00000098	0.00007230	0.00013021	0.03796296	A_07_P015906	b1846	cmr|orf, hypothetical protein [b1846]
yebF	-4.86	15.29	-2.28	16.43	0.0	14.15	0.0	0.00001003	0.00025069	0.00342353	0.19458484	A_07_P015914	b1847	cmr|orf, hypothetical protein [b1847]
yebK	+3.53	8.49	+1.82	7.58	0.1	9.40	0.1	0.00006756	0.00091460	0.02002477	0.37178066	A_07_P015941	b1853	cmr|orf, hypothetical protein [b1853]
yebN	-5.91	10.44	-2.56	11.72	0.1	9.16	0.1	0.00000637	0.00019181	0.00177401	0.13826733	A_07_P015797	b1821	cmr|orf, hypothetical protein [b1821]
yebO	-8.32	9.79	-3.06	11.32	0.0	8.26	0.1	0.00000222	0.00010802	0.00046240	0.06803738	A_07_P015812	b1825	cmr|orf, hypothetical protein [b1825]
yebR	+3.30	8.87	+1.72	8.01	0.1	9.74	0.2	0.00015857	0.00169023	0.02279345	0.40276094	A_07_P015837	b1832	cmr|orf, hypothetical protein [b1832]
yebW	-2.49	9.30	-1.32	9.96	0.1	8.65	0.1	0.00040703	0.00336868	0.03633638	0.51912886	A_07_P010475	b1837	cmr|orf, hypothetical protein [b1837]
yebW	-2.23	9.94	-1.16	10.52	0.1	9.36	0.3	0.00266378	0.01379735	0.05347879	0.60140714	A_07_P015861	b1837	cmr|orf, hypothetical protein [b1837]
yebW	-2.34	9.55	-1.22	10.17	0.2	8.94	0.3	0.00432311	0.02028707	0.04556085	0.56884219	A_07_P035495	c_2246	cmr|Hypothetical protein yebW [c_2246]
yebY	+3.26	10.23	+1.70	9.38	0.0	11.08	0.1	0.00010121	0.00121081	0.02329967	0.40849666	A_07_P015874	b1839	cmr|orf, hypothetical protein [b1839]
yebZ	-2.12	11.32	-1.08	11.86	0.1	10.77	0.3	0.00405388	0.01922420	0.06241425	0.63891417	A_07_P042419	c_2250	cmr|Hypothetical protein yebZ [c_2250]
yecC	-3.28	8.65	-1.71	9.50	0.1	7.79	0.1	0.00010260	0.00122473	0.01441438	0.35965135	A_07_P014947	b1917	cmr|putative ATP-binding component of a transport system [b1917]
yecH	+2.57	8.02	+1.36	7.34	0.3	8.70	0.2	0.00214522	0.01167852	0.03792175	0.48737959	A_07_P035364	c_2322	cmr|Hypothetical protein yecH [c_2322]
yecH	+2.32	7.73	+1.22	7.12	0.1	8.33	0.1	0.00070746	0.00497024	0.04567772	0.52995578	A_07_P016197	b1906	cmr|orf, hypothetical protein [b1906]
yecI	+5.76	10.79	+2.53	9.53	0.0	12.05	0.1	0.00000902	0.00023748	0.00762449	0.24699588	A_07_P035358	c_2315	cmr|Ferritin-like protein 2 [c_2315]
yecR	-3.59	7.99	-1.84	8.91	0.0	7.07	0.1	0.00004357	0.00066917	0.01030297	0.32442000	A_07_P016190	b1904	cmr|orf, hypothetical protein [b1904]
yecS	-4.69	9.22	-2.23	10.33	0.1	8.11	0.0	0.00001647	0.00034504	0.00393610	0.20725753	A_07_P014955	b1918	cmr|putative transport system permease protein [b1918]
yecS	-4.12	9.46	-2.04	10.48	0.0	8.44	0.2	0.00003649	0.00058676	0.00626588	0.25824607	A_07_P042434	c_2332	cmr|Hypothetical amino-acid ABC transporter permease protein yecS [c_2332]
yedD	+2.08	9.55	+1.06	9.02	0.0	10.08	0.1	0.00140765	0.00836504	0.05835620	0.59046272	A_07_P015001	b1928	cmr|orf, hypothetical protein [b1928]
yedE	+4.67	10.12	+2.22	9.01	0.0	11.23	0.1	0.00001555	0.00033216	0.01194931	0.29580189	A_07_P015006	b1929	cmr|putative transport system permease protein [b1929]
yedE	+3.11	9.42	+1.64	8.60	0.1	10.24	0.5	0.00311655	0.01558175	0.02611344	0.42737006	A_07_P052596	Z3019	cmr|putative transport system permease protein [Z3019]
yedE	+15.05	10.76	+3.91	8.81	0.1	12.72	0.1	0.00000063	0.00005892	0.00101499	0.09626506	A_07_P035376	c_2344	cmr|Hypothetical protein yedE [c_2344]
yedF	+12.36	10.23	+3.63	8.41	0.2	12.04	0.2	0.00000387	0.00014511	0.00161839	0.12018868	A_07_P015012	b1930	cmr|orf, hypothetical protein [b1930]
yedJ	-2.37	8.23	-1.25	8.85	0.0	7.61	0.1	0.00041248	0.00340005	0.04387386	0.56250000	A_07_P052607	Z3055	cmr|orf, hypothetical protein [Z3055]
yedJ	-4.71	10.20	-2.24	11.31	0.0	9.08	0.1	0.00001910	0.00038165	0.00380335	0.20436860	A_07_P041216	c_2381	cmr|Hypothetical protein yedJ [c_2381]
yedJ	-4.38	9.15	-2.13	10.21	0.0	8.08	0.1	0.00002425	0.00044556	0.00497777	0.22915205	A_07_P015168	b1962	cmr|orf, hypothetical protein [b1962]
yedR	-3.21	8.17	-1.68	9.01	0.1	7.33	0.1	0.00009992	0.00120090	0.01556974	0.37253799	A_07_P015174	b1963	cmr|orf, hypothetical protein [b1963]
yedU	+3.08	10.56	+1.62	9.75	0.4	11.37	0.2	0.00112956	0.00711920	0.02656822	0.42682653	A_07_P042474	c_2385	cmr|Protein yedU [c_2385]
yeeA	+3.80	8.20	+1.93	7.24	0.1	9.17	0.1	0.00006248	0.00086284	0.01753747	0.35039340	A_07_P031967	c_2536	cmr|Hypothetical protein yeeA [c_2536]
yeeD	-2.70	10.64	-1.43	11.36	0.1	9.92	0.1	0.00027677	0.00253932	0.02862868	0.47899044	A_07_P015390	b2012	cmr|orf, hypothetical protein [b2012]
yeeO	-6.77	8.37	-2.76	9.75	0.0	6.99	0.1	0.00000337	0.00013444	0.00108105	0.10506173	A_07_P015266	b1985	cmr|orf, hypothetical protein [b1985]
yeeP	-2.08	7.48	-1.06	8.01	0.0	6.96	0.1	0.00126795	0.00774643	0.06658346	0.65151647	A_07_P015325	b1999	cmr|putative histone [b1999]
yeeR	-2.14	10.53	-1.09	11.07	0.0	9.98	0.3	0.00280352	0.01435870	0.06001270	0.62821809	A_07_P015335	b2001	cmr|orf, hypothetical protein [b2001]
yeeY	-3.00	9.63	-1.58	10.42	0.1	8.84	0.1	0.00011418	0.00131889	0.01988249	0.40547927	A_07_P015404	b2015	cmr|putative transcriptional regulator LYSR-type [b2015]
yeeZ	-3.52	12.17	-1.82	13.08	0.0	11.26	0.2	0.00015635	0.00167317	0.01140244	0.33244444	A_07_P015407	b2016	cmr|putative enzyme of sugar metabolism [b2016]
yegB	-2.61	7.86	-1.38	8.55	0.2	7.17	0.1	0.00094157	0.00624169	0.03155678	0.49782565	A_07_P042496	c_2603	cmr|Hypothetical transport protein yegB [c_2603]
yegB	-2.19	7.33	-1.13	7.89	0.1	6.76	0.0	0.00086002	0.00583628	0.05592924	0.61149306	A_07_P045116	Z3246	cmr|putative transport protein [Z3246]
yegD	-2.45	9.34	-1.29	9.98	0.1	8.69	0.1	0.00042830	0.00350235	0.03866806	0.53309107	A_07_P003121	b2069	cmr|putative heat shock protein [b2069]
yegE	-2.53	9.21	-1.34	9.88	0.1	8.54	0.1	0.00029717	0.00266590	0.03495617	0.51147770	A_07_P003112	b2067	cmr|putative sensor-type protein [b2067]
yegI	-2.00	10.14	-1.00	10.64	0.1	9.64	0.3	0.00494719	0.02259605	0.07400089	0.67384037	A_07_P003126	b2070	cmr|putative chaperonin [b2070]
yegL	-2.39	9.82	-1.26	10.45	0.1	9.19	0.0	0.00048106	0.00380401	0.04282330	0.55858326	A_07_P032081	c_2599	cmr|Hypothetical protein yegL [c_2599]
yegL	-2.04	10.72	-1.03	11.24	0.2	10.21	0.2	0.00492188	0.02251311	0.07061738	0.66415771	A_07_P003144	b2073	cmr|orf, hypothetical protein [b2073]
yegQ	-7.05	10.25	-2.82	11.66	0.1	8.84	0.1	0.00000374	0.00014286	0.00095338	0.09940397	A_07_P003182	b2081	cmr|orf, hypothetical protein [b2081]
yegS	-2.31	9.53	-1.20	10.13	0.2	8.92	0.3	0.00321515	0.01595819	0.04556021	0.56928571	A_07_P032127	c_2614	cmr|Hypothetical protein yegS [c_2614]
yegW	-2.30	8.59	-1.20	9.19	0.1	7.99	0.0	0.00075700	0.00525729	0.04822663	0.58048930	A_07_P003270	b2101	cmr|putative transcriptional regulator [b2101]
yegX	-2.40	11.37	-1.26	12.00	0.1	10.74	0.1	0.00063660	0.00461448	0.04138847	0.54850168	A_07_P003271	b2102	cmr|orf, hypothetical protein [b2102]
yegX	-3.35	8.47	-1.74	9.34	0.1	7.60	0.2	0.00020502	0.00203005	0.01306974	0.34467337	A_07_P051783	Z3266	cmr|orf, hypothetical protein [Z3266]
yehD	+7.90	8.09	+2.98	6.60	0.0	9.59	0.1	0.00000310	0.00012804	0.00405678	0.18353448	A_07_P003318	b2111	cmr|putative fimbrial-like protein [b2111]
yehL	-3.73	8.15	-1.90	9.10	0.0	7.20	0.1	0.00005363	0.00077607	0.00930069	0.30762605	A_07_P003356	b2119	cmr|orf, hypothetical protein [b2119]
yehL	-5.64	9.07	-2.50	10.32	0.1	7.82	0.3	0.00005222	0.00076124	0.00203379	0.14688073	A_07_P041221	c_2649	cmr|Hypothetical protein yehL [c_2649]
yehP	-3.47	10.73	-1.80	11.63	0.6	9.84	0.2	0.00454759	0.02113260	0.01188516	0.33534050	A_07_P003369	b2121	cmr|orf, hypothetical protein [b2121]
yehS	-3.13	9.14	-1.65	9.96	0.0	8.32	0.1	0.00007763	0.00099930	0.01700013	0.37964539	A_07_P003385	b2124	cmr|orf, hypothetical protein [b2124]
yehX	-2.28	7.64	-1.19	8.24	0.0	7.05	0.1	0.00063568	0.00461448	0.05016768	0.58680535	A_07_P032191	c_2659	cmr|Hypothetical ABC transporter ATP-binding protein yehX [c_2659]
yeiC	+5.56	7.83	+2.48	6.60	0.3	9.07	0.3	0.00010371	0.00123350	0.00821519	0.25114563	A_07_P032264	c_2701	cmr|Hypothetical sugar kinase yeiC [c_2701]
yeiE	+2.22	9.32	+1.15	8.75	0.4	9.90	0.2	0.00842898	0.03424667	0.05374555	0.56904506	A_07_P003546	b2157	cmr|putative transcriptional regulator LYSR-type [b2157]
yeiP	+4.02	8.97	+2.01	7.97	0.0	9.97	0.0	0.00002376	0.00044169	0.01578189	0.33943989	A_07_P000651	b2171	cmr|putative elongation factor [b2171]
yeiQ	+9.88	9.49	+3.30	7.84	0.0	11.14	0.1	0.00000155	0.00009347	0.00258829	0.14818182	A_07_P000660	b2172	cmr|putative oxidoreductase [b2172]
yeiT	+4.59	9.06	+2.20	7.96	0.2	10.16	0.1	0.00003284	0.00054895	0.01226626	0.29941085	A_07_P003491	b2146	cmr|putative oxidoreductase [b2146]
yeiW	-2.13	8.19	-1.09	8.74	0.1	7.65	0.0	0.00105878	0.00680106	0.06135163	0.63589203	A_07_P010506	ABE-0285016	asap|conserved protein [b4502]
yejG	-6.00	12.28	-2.59	13.57	0.0	10.99	0.1	0.00000507	0.00017253	0.00168128	0.13301508	A_07_P000703	b2181	cmr|orf, hypothetical protein [b2181]
yejK	+4.31	10.30	+2.11	9.25	0.2	11.35	0.1	0.00007213	0.00095356	0.01388465	0.31912409	A_07_P000729	b2186	cmr|orf, hypothetical protein [b2186]
yejL	-2.22	12.05	-1.15	12.63	0.1	11.48	0.1	0.00083506	0.00570625	0.05399200	0.60501779	A_07_P000733	b2187	cmr|orf, hypothetical protein [b2187]
yfaE	+10.64	9.65	+3.41	7.95	0.2	11.36	0.2	0.00000684	0.00020009	0.00226880	0.13791506	A_07_P000966	b2236	cmr|orf, hypothetical protein [b2236]
yfaW	+2.62	7.73	+1.39	7.03	0.0	8.42	0.0	0.00020102	0.00200437	0.03502096	0.47613990	A_07_P001021	b2247	cmr|putative racemase [b2247]
yfbB	+3.34	8.70	+1.74	7.83	0.0	9.57	0.2	0.00019799	0.00198296	0.02239075	0.39877828	A_07_P001104	b2263	cmr|putative enzyme [b2263]
yfbM	+2.61	8.08	+1.38	7.39	0.1	8.78	0.0	0.00029229	0.00263563	0.03541794	0.47700599	A_07_P001148	b2272	cmr|orf, hypothetical protein [b2272]
yfcD	+2.06	12.80	+1.04	12.28	0.0	13.33	0.2	0.00233590	0.01245813	0.06092226	0.60173149	A_07_P013782	b2299	cmr|putative regulator [b2299]
yfcF	+2.15	8.20	+1.10	7.65	0.1	8.76	0.1	0.00110045	0.00698892	0.05380272	0.56888516	A_07_P013791	b2301	cmr|orf, hypothetical protein [b2301]
yfdE	-5.43	9.17	-2.44	10.39	0.2	7.95	0.2	0.00004005	0.00062797	0.00234375	0.16113537	A_07_P014140	b2371	cmr|putative enzyme [b2371]
yfdZ	+4.02	8.82	+2.01	7.82	0.0	9.83	0.2	0.00005683	0.00080535	0.01577553	0.33976744	A_07_P014179	b2379	cmr|putative aminotransferase [b2379]
yfeA	-2.03	8.72	-1.02	9.23	0.1	8.21	0.0	0.00163011	0.00941651	0.07122713	0.66591449	A_07_P014259	b2395	cmr|orf, hypothetical protein [b2395]
yfeD	-2.53	9.77	-1.34	10.44	0.0	9.10	0.3	0.00140799	0.00836504	0.03572790	0.51652893	A_07_P030017	c_2935	cmr|Hypothetical protein yfeD [c_2935]
yfeN	-3.27	10.78	-1.71	11.63	0.2	9.92	0.1	0.00022617	0.00218591	0.01439278	0.35968254	A_07_P013096	b2408	cmr|putative sugar hydrolase [b2408]
yffB	-3.29	10.95	-1.72	11.81	0.0	10.09	0.0	0.00005952	0.00083457	0.01414380	0.35800643	A_07_P013411	b2471	cmr|orf, hypothetical protein [b2471]
yffL	-2.97	9.30	-1.57	10.09	0.2	8.51	0.1	0.00033179	0.00287013	0.02062945	0.41321883	A_07_P013272	b2443	cmr|orf, hypothetical protein [b2443]
yfgC	+2.63	8.64	+1.40	7.95	0.0	9.34	0.1	0.00025625	0.00239143	0.03478468	0.47456672	A_07_P013530	b2494	cmr|orf, hypothetical protein [b2494]
yfgC	+3.08	9.74	+1.62	8.92	0.2	10.55	0.2	0.00040661	0.00336751	0.02607088	0.42711759	A_07_P042608	c_3011	cmr|Hypothetical protein yfgC precursor [c_3011]
yfgD	+5.03	8.59	+2.33	7.42	0.1	9.75	0.1	0.00001713	0.00035530	0.01008702	0.27715532	A_07_P013532	b2495	cmr|putative oxidoreductase [b2495]
yfgF	-2.82	9.11	-1.50	9.86	0.1	8.36	0.0	0.00020517	0.00203026	0.02423844	0.45001179	A_07_P013571	b2503	cmr|putative cytochrome C-type biogenesis protein [b2503]
yfgG	+2.79	10.37	+1.48	9.63	0.1	11.11	0.2	0.00036902	0.00312361	0.03097815	0.45034164	A_07_P013578	b2504	cmr|orf, hypothetical protein [b2504]
yfgI	-2.15	10.73	-1.10	11.28	0.2	10.18	0.1	0.00186801	0.01046503	0.05876651	0.62388402	A_07_P030147	c_3025	cmr|Hypothetical protein yfgI [c_3025]
yfgI	-2.38	11.19	-1.25	11.82	0.2	10.57	0.2	0.00126669	0.00774429	0.04295478	0.55890909	A_07_P013587	b2506	cmr|putative membrane protein [b2506]

yfgM	+3.83	10.95	+1.94	9.98	0.0	11.92	0.0	0.00002806	0.00049142	0.01723323	0.34829268	A_07_P013623	b2513	cmr|orf, hypothetical protein [b2513]
yfhB	-6.26	9.98	-2.65	11.30	0.1	8.65	0.1	0.00000621	0.00018975	0.00145262	0.12497268	A_07_P019703	b2560	cmr|orf, hypothetical protein [b2560]
yfiC	+2.24	8.47	+1.16	7.88	0.2	9.05	0.1	0.00138910	0.00828407	0.05004002	0.55716407	A_07_P019777	b2575	cmr|putative enzyme [b2575]
yfiD	+6.33	11.53	+2.66	10.20	0.1	12.86	0.4	0.00008899	0.00110064	0.00656949	0.23295045	A_07_P019799	b2579	cmr|putative formate acetyltransferase [b2579]
yfiF	+7.42	11.01	+2.89	9.56	0.1	12.45	0.1	0.00000375	0.00014286	0.00440549	0.19002740	A_07_P019806	b2581	cmr|orf, hypothetical protein [b2581]
yfiH	-2.18	9.36	-1.12	9.92	0.1	8.80	0.1	0.00091408	0.00611098	0.05708079	0.61595613	A_07_P019847	b2593	cmr|orf, hypothetical protein [b2593]
yfiQ	+7.07	9.58	+2.82	8.17	0.0	11.00	0.2	0.00000629	0.00019141	0.00502858	0.20889182	A_07_P019821	b2584	cmr|orf, hypothetical protein [b2584]
yfjF	-2.26	11.72	-1.17	12.30	0.1	11.13	0.3	0.00415273	0.01962000	0.05173272	0.59234909	A_07_P019968	b2618	cmr|orf, hypothetical protein [b2618]
yfjH	-2.09	8.01	-1.06	8.54	0.4	7.47	0.0	0.00821525	0.03367376	0.06399073	0.64581410	A_07_P019987	b2623	cmr|putative histone [b2623]
yfjI	-2.02	10.34	-1.02	10.84	0.1	9.83	0.0	0.00155536	0.00904937	0.07264545	0.67002343	A_07_P019999	b2625	cmr|orf, hypothetical protein [b2625]
yfjJ	-2.06	7.34	-1.04	7.86	0.4	6.82	0.0	0.01201814	0.04553879	0.06603722	0.65102693	A_07_P020002	b2626	cmr|orf, hypothetical protein [b2626]
ygaA	-3.26	9.19	-1.71	10.04	0.1	8.34	0.0	0.00007274	0.00095677	0.01468051	0.36398425	A_07_P030601	c_3263	cmr|Hypothetical sigma-54-dependent transcriptional regulator ygaA [c_3263]
ygaD	+6.49	10.22	+2.70	8.88	0.0	11.57	0.2	0.00001228	0.00028963	0.00611344	0.22540984	A_07_P001946	b2700	cmr|orf, hypothetical protein [b2700]
ygaP	-2.33	11.27	-1.22	11.88	0.5	10.66	0.1	0.00920548	0.03680429	0.04548209	0.56876092	A_07_P020192	b2668	cmr|orf, hypothetical protein [b2668]
ygaT	-2.49	8.48	-1.31	9.14	0.0	7.82	0.1	0.00038321	0.00321947	0.03796303	0.52892920	A_07_P041636	c_3207	cmr|Hypothetical protein ygaT [c_3207]
ygaV	+2.92	8.65	+1.55	7.88	0.2	9.43	0.1	0.00029887	0.00267603	0.02887957	0.43972921	A_07_P020189	b2667	cmr|orf, hypothetical protein [b2667]
ygaW	-7.61	11.51	-2.93	12.97	0.1	10.04	0.4	0.00004361	0.00066917	0.00064914	0.08047244	A_07_P001825	b2670	cmr|orf, hypothetical protein [b2670]
ygaZ	-2.01	10.00	-1.01	10.50	0.2	9.49	0.1	0.00322064	0.01598039	0.07154916	0.66773563	A_07_P001881	b2682	cmr|orf, hypothetical protein [b2682]
ygbE	-2.54	8.73	-1.34	9.40	0.0	8.06	0.1	0.00038711	0.00324531	0.03471735	0.51274859	A_07_P030701	c_3316	cmr|Hypothetical protein ygbE [c_3316]
ygbJ	+3.88	8.85	+1.96	7.87	0.1	9.83	0.2	0.00007611	0.00098704	0.01683562	0.34558018	A_07_P030675	c_3297	cmr|Hypothetical oxidoreductase ygbJ [c_3297]
ygbJ	+2.26	7.91	+1.18	7.32	0.1	8.49	0.0	0.00058819	0.00436400	0.04797574	0.54457823	A_07_P002127	b2736	cmr|putative dehydrogenase [b2736]
ygbK	+2.94	8.32	+1.56	7.54	0.2	9.09	0.2	0.00029429	0.00264913	0.02855373	0.43901367	A_07_P002131	b2737	cmr|orf, hypothetical protein [b2737]
ygbL	-2.78	11.79	-1.48	12.53	0.1	11.06	0.2	0.00044394	0.00358698	0.02589621	0.46330682	A_07_P002137	b2738	cmr|putative epimerase/aldolase [b2738]
ygbM	+2.43	7.70	+1.28	7.06	0.4	8.34	0.1	0.00411088	0.01945349	0.04304306	0.51338636	A_07_P002141	b2739	cmr|orf, hypothetical protein [b2739]
ygcG	-4.35	9.33	-2.12	10.39	0.1	8.27	0.2	0.00003412	0.00056193	0.00526613	0.23756447	A_07_P032628	c_3342	cmr|Hypothetical protein ygcG [c_3342]
ygcN	-2.12	9.27	-1.08	9.81	0.0	8.72	0.2	0.00176238	0.01000969	0.06150597	0.63665352	A_07_P002278	b2766	cmr|orf, hypothetical protein [b2766]
ygcP	-2.07	10.29	-1.05	10.82	0.0	9.77	0.1	0.00150695	0.00882312	0.06657647	0.65185323	A_07_P002288	b2768	cmr|putative anti-terminator regulatory protein [b2768]
ygcR	-2.08	10.62	-1.06	11.15	0.1	10.09	0.1	0.00184458	0.01036442	0.06504827	0.64858771	A_07_P002300	b2770	cmr|putative transport protein [b2770]
ygcY	+2.12	8.02	+1.08	7.48	0.0	8.56	0.1	0.00096158	0.00634234	0.05556657	0.57707124	A_07_P032636	c_3352	cmr|Glucarate dehydratase related protein [c_3352]
ygdH	+4.70	9.97	+2.23	8.85	0.1	11.09	0.0	0.00001671	0.00034847	0.01136433	0.29092683	A_07_P002424	b2795	cmr|orf, hypothetical protein [b2795]
ygdL	-2.94	10.72	-1.56	11.50	0.1	9.94	0.1	0.00014340	0.00156135	0.02110074	0.41787421	A_07_P032656	c_3383	cmr|Hypothetical protein ygdL [c_3383]
ygdQ	-2.56	8.19	-1.36	8.87	0.1	7.52	0.1	0.00032985	0.00285964	0.03334159	0.50571291	A_07_P006141	b2832	cmr|putative transport protein [b2832]
ygeK	-2.91	11.13	-1.54	11.90	0.2	10.36	0.2	0.00063174	0.00459849	0.02146087	0.42129676	A_07_P006257	b2855	cmr|putative 2-component transcriptional regulator [b2855]
ygeR	-13.39	9.90	-3.74	11.77	0.0	8.03	0.1	0.00000053	0.00005412	0.00005272	0.02243243	A_07_P006295	b2865	cmr|putative lipoprotein [b2865]
ygeT	+3.04	8.06	+1.61	7.26	0.1	8.86	0.1	0.00012380	0.00139716	0.02628493	0.42662887	A_07_P032831	c_3445	cmr|Xanthine dehydrogenase, FAD binding subunit [c_3445]
ygfA	-3.30	9.36	-1.72	10.23	0.1	8.50	0.0	0.00006018	0.00084075	0.01415523	0.35657600	A_07_P006525	b2912	cmr|putative ligase [b2912]
ygfB	-2.41	13.56	-1.27	14.20	0.0	12.93	0.2	0.00107334	0.00686939	0.04174733	0.54909774	A_07_P006514	b2909	cmr|orf, hypothetical protein [b2909]
ygfI	-2.04	7.10	-1.03	7.61	0.1	6.58	0.2	0.00219783	0.01189911	0.06939342	0.65775436	A_07_P006568	b2921	cmr|putative transcriptional regulator LYSR-type [b2921]
ygfJ	-2.18	9.42	-1.13	9.98	0.2	8.85	0.1	0.00178976	0.01012870	0.05716336	0.61642466	A_07_P006352	b2877	cmr|orf, hypothetical protein [b2877]
ygfX	-3.06	11.23	-1.61	12.04	0.0	10.42	0.4	0.00129506	0.00787670	0.01885036	0.39570667	A_07_P006446	b2896	cmr|orf, hypothetical protein [b2896]
ygfZ	+2.33	11.82	+1.22	11.21	0.1	12.42	0.0	0.00047815	0.00378674	0.04510607	0.52642698	A_07_P006456	b2898	cmr|orf, hypothetical protein [b2898]
yggD	-2.92	9.75	-1.55	10.53	0.0	8.98	0.1	0.00015984	0.00169918	0.02179306	0.42411619	A_07_P032921	c_3512	cmr|Hypothetical protein yggD [c_3512]
yggL	+4.58	13.13	+2.19	12.03	0.0	14.22	0.1	0.00001609	0.00033993	0.01218941	0.29892523	A_07_P011938	b2959	cmr|orf, hypothetical protein [b2959]
yggP	-2.44	8.26	-1.29	8.90	0.1	7.61	0.1	0.00056879	0.00426231	0.03945630	0.53876843	A_07_P011806	b2932	cmr|orf, hypothetical protein [b2932]
yggX	+3.14	10.78	+1.65	9.96	0.1	11.61	0.0	0.00007686	0.00099265	0.02424416	0.41220302	A_07_P011954	b2962	cmr|orf, hypothetical protein [b2962]
yghB	-7.68	11.23	-2.94	12.70	0.0	9.76	0.1	0.00000296	0.00012541	0.00068534	0.08112782	A_07_P012180	b3009	cmr|orf, hypothetical protein [b3009]
yghU	+3.15	8.28	+1.65	7.45	0.1	9.10	0.1	0.00009045	0.00111579	0.02439088	0.41425027	A_07_P012078	b2989	cmr|orf, hypothetical protein [b2989]
yghU	+5.16	9.14	+2.37	7.96	0.1	10.33	0.1	0.00000952	0.00024416	0.00940485	0.26872958	A_07_P041281	c_3726	cmr|Hypothetical GST-like protein yghU [c_3726]
yghZ	+4.59	9.86	+2.20	8.76	0.1	10.96	0.2	0.00002761	0.00048679	0.01207254	0.29744914	A_07_P012136	b3001	cmr|putative reductase [b3001]
ygiC	+3.23	8.64	+1.69	7.79	0.3	9.48	0.3	0.00086372	0.00585633	0.02392658	0.41124454	A_07_P012323	b3038	cmr|putative synthetase/amidase [b3038]
ygiF	-3.16	11.60	-1.66	12.43	0.0	10.77	0.2	0.00012346	0.00139625	0.01641578	0.37729927	A_07_P000003	b3054	cmr|orf, hypothetical protein [b3054]
ygiF	-3.39	12.66	-1.76	13.54	0.0	11.78	0.2	0.00008984	0.00110941	0.01241616	0.33703448	A_07_P041286	c_3802	cmr|Hypothetical protein ygiF [c_3802]
ygiN	+10.89	10.45	+3.44	8.72	0.1	12.17	0.2	0.00000262	0.00011934	0.00210048	0.13334677	A_07_P012278	b3029	cmr|orf, hypothetical protein [b3029]
ygiP	-3.43	9.97	-1.78	10.86	0.0	9.08	0.1	0.00005246	0.00076402	0.01217734	0.33813051	A_07_P036891	c_3810	cmr|Hypothetical transcriptional regulator ygiP [c_3810]
ygjH	-3.31	12.33	-1.73	13.19	0.0	11.47	0.3	0.00035182	0.00300256	0.01436039	0.36001592	A_07_P000096	b3074	cmr|putative tRNA synthetase [b3074]
ygjP	-5.77	9.20	-2.53	10.47	0.1	7.94	0.2	0.00001234	0.00028963	0.00191629	0.14504808	A_07_P000154	b3085	cmr|orf, hypothetical protein [b3085]
ygjP	-11.27	10.59	-3.49	12.34	0.0	8.84	0.3	0.00000329	0.00013224	0.00010036	0.03590909	A_07_P034044	c_3843	cmr|Hypothetical protein ygjP [c_3843]
ygjQ	-3.47	8.06	-1.80	8.96	0.0	7.17	0.0	0.00004560	0.00068835	0.01186865	0.33607914	A_07_P041292	c_3844	cmr|Hypothetical protein ygjQ [c_3844]
ygjQ	-2.89	8.20	-1.53	8.97	0.3	7.44	0.2	0.00113452	0.00713681	0.02185722	0.42483951	A_07_P000160	b3086	cmr|orf, hypothetical protein [b3086]
ygjR	+5.69	9.31	+2.51	8.06	0.1	10.57	0.1	0.00000779	0.00021851	0.00777312	0.24623742	A_07_P000165	b3087	cmr|orf, hypothetical protein [b3087]
ygjU	+3.27	9.30	+1.71	8.44	0.1	10.15	0.1	0.00014388	0.00156332	0.02283410	0.40257559	A_07_P034047	c_3847	cmr|Hypothetical symporter ygjU [c_3847]
yhaG	+2.23	9.36	+1.16	8.78	0.2	9.94	0.1	0.00136154	0.00815989	0.05003239	0.55747346	A_07_P034086	c_3883	cmr|D-galactarate dehydratase [c_3883]
yhaG	+4.21	9.45	+2.07	8.41	0.2	10.48	0.2	0.00007297	0.00095734	0.01442518	0.32444286	A_07_P045896	Z4480	cmr|putative hydrolase [Z4480]
yhaJ	+2.85	9.82	+1.51	9.06	0.0	10.58	0.1	0.00013419	0.00149094	0.02963478	0.44350760	A_07_P000254	b3105	cmr|putative transcriptional regulator LYSR-type [b3105]
yhaL	+3.09	10.77	+1.63	9.95	0.4	11.58	0.3	0.00237460	0.01263456	0.02579459	0.42391441	A_07_P000264	b3107	cmr|orf, hypothetical protein [b3107]
yhaR	+7.05	11.56	+2.82	10.15	0.1	12.97	0.1	0.00000373	0.00014286	0.00501905	0.20904762	A_07_P034071	c_3871	cmr|TdcF protein [c_3871]
yhbC	-2.60	13.50	-1.38	14.18	0.0	12.81	0.1	0.00032482	0.00282698	0.03268737	0.50553045	A_07_P000572	b3170	cmr|orf, hypothetical protein [b3170]
yhbO	+2.64	8.07	+1.40	7.37	0.0	8.77	0.0	0.00019574	0.00196808	0.03458778	0.47311034	A_07_P000486	b3153	cmr|orf, hypothetical protein [b3153]
yhbU	+2.02	10.72	+1.01	10.22	0.2	11.23	0.1	0.00291855	0.01485119	0.06362741	0.61344152	A_07_P000511	b3158	cmr|putative collagenase [b3158]
yhbW	+2.20	8.41	+1.14	7.85	0.4	8.98	0.1	0.00658049	0.02827779	0.05248349	0.56441257	A_07_P034111	c_3913	cmr|Hypothetical protein yhbW [c_3913]
yhbY	+2.06	11.28	+1.04	10.76	0.1	11.80	0.1	0.00177987	0.01008723	0.06052464	0.59968534	A_07_P000614	b3180	cmr|orf, hypothetical protein [b3180]
yhcB	+5.72	12.86	+2.52	11.60	0.0	14.11	0.1	0.00000854	0.00022936	0.00772040	0.24655172	A_07_P012640	b3233	cmr|orf, hypothetical protein [b3233]
yhcH	+2.03	7.75	+1.02	7.24	0.1	8.26	0.1	0.00144884	0.00853689	0.06117569	0.60272215	A_07_P012577	b3221	cmr|orf, hypothetical protein [b3221]
yhcH	+5.77	8.32	+2.53	7.06	0.0	9.59	0.1	0.00000705	0.00020411	0.00748031	0.24586639	A_07_P034165	c_3975	cmr|Hypothetical protein yhcH [c_3975]
yhcH	+3.63	8.24	+1.86	7.31	0.3	9.17	0.1	0.00018148	0.00186421	0.01858803	0.35645554	A_07_P045957	Z4579	cmr|orf, hypothetical protein [Z4579]
yhcI	+3.82	7.99	+1.93	7.02	0.2	8.95	0.2	0.00018320	0.00187662	0.01731136	0.34764031	A_07_P051815	Z4580	cmr|putative NAGC-like transcriptional regulator [Z4580]
yhcJ	+6.45	8.72	+2.69	7.38	0.2	10.06	0.1	0.00001090	0.00026728	0.00614710	0.22559441	A_07_P041791	c_3977	cmr|Hypothetical protein yhcJ [c_3977]
yhcJ	+7.53	8.68	+2.91	7.22	0.1	10.13	0.2	0.00000586	0.00018329	0.00436928	0.18950413	A_07_P041304	c_3977	cmr|Hypothetical protein yhcJ [c_3977]
yhcK	-2.32	9.62	-1.21	10.22	0.0	9.01	0.0	0.00047031	0.00374156	0.04714876	0.57543411	A_07_P034168	c_3980	cmr|Hypothetical transcriptional regulator yhcK [c_3980]
yhcR	-3.19	7.57	-1.67	8.41	0.1	6.74	0.0	0.00010051	0.00120434	0.01584858	0.37409295	A_07_P035544	c_3997	cmr|Hypothetical protein yhcR [c_3997]
yhcR	-3.84	8.30	-1.94	9.27	0.0	7.33	0.2	0.00008636	0.00108170	0.00793445	0.28783410	A_07_P048607	Z4601	cmr|orf, hypothetical protein [Z4601]
yhdE	+3.07	9.30	+1.62	8.49	0.2	10.11	0.1	0.00029213	0.00263563	0.02576537	0.42476440	A_07_P012715	b3248	cmr|orf, hypothetical protein [b3248]
yhdH	+10.86	10.25	+3.44	8.53	0.3	11.97	0.2	0.00000551	0.00017942	0.00209858	0.13376518	A_07_P034191	c_4009	cmr|Protein yhdH [c_4009]
yhdH	+6.04	8.75	+2.60	7.45	0.2	10.05	0.2	0.00002027	0.00039679	0.00690104	0.23826754	A_07_P012736	b3253	cmr|putative dehydrogenase [b3253]
yhdZ	+3.05	8.20	+1.61	7.39	0.1	9.00	0.0	0.00014211	0.00155099	0.02621824	0.42642562	A_07_P012823	b3271	cmr|putative ATP-binding component of a transport system [b3271]
yheF	-3.82	8.08	-1.93	9.04	0.1	7.11	0.0	0.00003602	0.00058156	0.00833143	0.29676471	A_07_P034326	c_4096	cmr|Probable general secretion pathway protein D precursor [c_4096]
yheM	-2.63	8.11	-1.39	8.81	0.0	7.41	0.4	0.00236679	0.01259730	0.03115282	0.49492432	A_07_P017750	b3344	cmr|orf, hypothetical protein [b3344]
yheN	-3.00	9.35	-1.58	10.14	0.0	8.56	0.3	0.00063041	0.00459501	0.02014799	0.40824968	A_07_P052135	Z4703	cmr|orf, hypothetical protein [Z4703]
yheN	-2.67	10.41	-1.42	11.12	0.3	9.70	0.1	0.00118483	0.00736436	0.02898056	0.48079031	A_07_P017753	b3345	cmr|orf, hypothetical protein [b3345]
yheO	-2.54	10.40	-1.35	11.08	0.0	9.73	0.2	0.00051564	0.00397371	0.03548082	0.51532288	A_07_P017760	b3346	cmr|orf, hypothetical protein [b3346]
yheS	-2.36	11.16	-1.24	11.78	0.0	10.54	0.1	0.00046905	0.00373344	0.04400025	0.56228896	A_07_P017786	b3352	cmr|putative ATP-binding component of a transport system [b3352]
yhfC	-5.14	8.31	-2.36	9.49	0.0	7.13	0.2	0.00001588	0.00033838	0.00277693	0.17418327	A_07_P041829	c_4139	cmr|Hypothetical protein yhfC [c_4139]
yhfL	-2.06	7.66	-1.04	8.18	0.0	7.14	0.2	0.00176444	0.01001419	0.06804370	0.65561812	A_07_P017874	b3369	cmr|orf, hypothetical protein [b3369]
yhfS	-3.18	10.78	-1.67	11.61	0.0	9.94	0.2	0.00019381	0.00195589	0.01635861	0.37653509	A_07_P017907	b3376	cmr|orf, hypothetical protein [b3376]
yhfS	-2.61	8.26	-1.39	8.95	0.2	7.57	0.3	0.00186914	0.01046503	0.03081047	0.49447503	A_07_P034375	c_4146	cmr|Hypothetical protein yhfS [c_4146]
yhfU	-2.67	8.83	-1.42	9.54	0.2	8.13	0.1	0.00055148	0.00417027	0.02929306	0.48140919	A_07_P017919	b3378	cmr|orf, hypothetical protein [b3378]
yhfZ	+2.35	8.25	+1.23	7.63	0.0	8.86	0.0	0.00040105	0.00333578	0.04439977	0.52166418	A_07_P017944	b3383	cmr|orf, hypothetical protein [b3383]
yhgF	+2.54	10.18	+1.34	9.50	0.0	10.85	0.0	0.00025771	0.00239798	0.03757812	0.48693827	A_07_P018065	b3407	cmr|orf, hypothetical protein [b3407]
yhgH	+2.05	7.72	+1.03	7.21	0.0	8.24	0.3	0.00367074	0.01774942	0.06032774	0.59848771	A_07_P041831	c_4190	cmr|Hypothetical protein yhgH [c_4190]
yhgH	+2.37	11.71	+1.24	11.09	0.0	12.34	0.2	0.00078806	0.00542276	0.04363885	0.51735693	A_07_P041315	c_4190	cmr|Hypothetical protein yhgH [c_4190]
yhhA	+2.17	8.68	+1.12	8.12	0.1	9.23	0.1	0.00114041	0.00715494	0.05332635	0.56881436	A_07_P018259	b3448	cmr|orf, hypothetical protein [b3448]
yhhI	-2.95	11.06	-1.56	11.84	0.5	10.28	0.1	0.00361266	0.01756570	0.02045351	0.41126437	A_07_P007358	b3484	cmr|putative receptor [b3484]
yhhJ	+2.63	8.03	+1.40	7.33	0.2	8.72	0.1	0.00052981	0.00404719	0.03533092	0.47746781	A_07_P007361	b3485	cmr|putative transporter [b3485]
yhhM	+2.33	7.64	+1.22	7.03	0.2	8.25	0.0	0.00114693	0.00718839	0.04596608	0.52940015	A_07_P007271	b3467	cmr|putative receptor [b3467]
yhhM	+2.60	8.01	+1.38	7.32	0.0	8.70	0.0	0.00021526	0.00211288	0.03549289	0.47719898	A_07_P007271	b3467	cmr|putative receptor [b3467]
yhhM	+2.05	8.20	+1.03	7.68	0.2	8.72	0.0	0.00216176	0.01174424	0.06089050	0.60179535	A_07_P007271	b3467	cmr|putative receptor [b3467]
yhhS	-2.43	8.26	-1.28	8.90	0.1	7.62	0.3	0.00187110	0.01047078	0.04100229	0.54475949	A_07_P007303	b3473	cmr|putative transport [b3473]
yhhU	-2.64	10.36	-1.40	11.06	0.1	9.66	0.3	0.00115652	0.00722808	0.03101944	0.49430162	A_07_P041853	c_4268	cmr|4-phosphopantetheinyl transferase acpT [c_4268]
yhhW	+2.19	8.97	+1.13	8.40	0.0	9.54	0.1	0.00087296	0.00591391	0.05115917	0.55895212	A_07_P018221	b3439	cmr|orf, hypothetical protein [b3439]
yhhX	+2.42	9.49	+1.28	8.86	0.3	10.13	0.0	0.00198368	0.01098886	0.04190041	0.50901235	A_07_P018230	b3440	cmr|putative regulator [b3440]
yhiD	-2.67	14.44	-1.42	15.14	0.0	13.73	0.2	0.00064859	0.00467558	0.02901486	0.48034700	A_07_P007475	b3508	cmr|putative transport ATPase [b3508]
yhiI	+2.53	11.22	+1.34	10.55	0.1	11.88	0.2	0.00053135	0.00405508	0.03822663	0.48771475	A_07_P007374	b3487	cmr|putative membrane protein [b3487]
yhiN	-3.78	10.80	-1.92	11.76	0.1	9.84	0.1	0.00004550	0.00068746	0.00883257	0.30230435	A_07_P007396	b3492	cmr|orf, hypothetical protein [b3492]
yhiP	+3.24	10.31	+1.69	9.46	0.0	11.15	0.0	0.00006269	0.00086499	0.02310722	0.40602679	A_07_P007419	b3496	cmr|putative transport protein [b3496]
yhiQ	-2.24	8.06	-1.16	8.65	0.1	7.48	0.1	0.00092425	0.00615538	0.05194995	0.59182344	A_07_P007422	b3497	cmr|orf, hypothetical protein [b3497]
yhiR	-5.29	12.37	-2.40	13.57	0.0	11.17	0.4	0.00008478	0.00106781	0.00262576	0.17153527	A_07_P007435	b3499	cmr|orf, hypothetical protein [b3499]
yhiW	-2.32	8.78	-1.21	9.39	0.0	8.17	0.3	0.00267158	0.01381689	0.04559007	0.56875594	A_07_P039549	c_4326	cmr|Hypothetical transcriptional regulator yhiW [c_4326]
yhjJ	-2.98	9.15	-1.57	9.93	0.0	8.36	0.1	0.00015055	0.00162457	0.02048399	0.41135204	A_07_P049426	Z4941	cmr|orf, hypothetical protein [Z4941]
yhjJ	-2.75	9.92	-1.46	10.65	0.0	9.19	0.1	0.00022055	0.00214748	0.02699949	0.47178690	A_07_P039556	c_4339	cmr|Protein yhjJ precursor [c_4339]
yhjQ	-5.69	8.59	-2.51	9.85	0.1	7.34	0.2	0.00002601	0.00046542	0.00201410	0.14680556	A_07_P007604	b3534	cmr|orf, hypothetical protein [b3534]
yhjR	-2.32	8.77	-1.21	9.38	0.0	8.17	0.2	0.00135441	0.00813575	0.04537729	0.56835322	A_07_P007606	b3535	cmr|orf, hypothetical protein [b3535]
yiaC	-3.20	9.97	-1.68	10.80	0.1	9.13	0.4	0.00105621	0.00678733	0.01623984	0.37710914	A_07_P007676	b3550	cmr|orf, hypothetical protein [b3550]
yiaC	-2.51	9.37	-1.33	10.03	0.1	8.70	0.0	0.00043904	0.00356045	0.03560722	0.51573137	A_07_P039621	c_4368	cmr|Hypothetical acetyltransferase yiaC [c_4368]
yiaF	+2.16	10.20	+1.11	9.65	0.0	10.76	0.1	0.00097766	0.00642954	0.05331110	0.56903729	A_07_P007698	b3554	cmr|orf, hypothetical protein [b3554]
yiaK	+3.97	8.68	+1.99	7.69	0.0	9.68	0.2	0.00004448	0.00067860	0.01617696	0.34232527	A_07_P007803	b3575	cmr|putative dehydrogenase [b3575]
yiaL	+2.10	7.72	+1.07	7.19	0.2	8.26	0.1	0.00257334	0.01344214	0.05662475	0.58344241	A_07_P039647	c_4397	cmr|Hypothetical protein yiaL [c_4397]
yiaN	+3.22	7.26	+1.69	6.41	0.0	8.10	0.1	0.00007204	0.00095356	0.02339240	0.40921111	A_07_P039665	c_4400	cmr|Hypothetical protein yiaN [c_4400]
yibA	-2.35	10.04	-1.24	10.66	0.0	9.42	0.3	0.00168978	0.00967148	0.04515689	0.56876000	A_07_P016913	b3594	cmr|orf, hypothetical protein [b3594]
yibF	+4.39	8.56	+2.13	7.49	0.0	9.63	0.1	0.00002047	0.00039827	0.01328061	0.31207463	A_07_P016905	b3592	cmr|putative S-transferase [b3592]
yibK	+2.10	9.38	+1.07	8.85	0.0	9.91	0.0	0.00090188	0.00605770	0.05635417	0.58217848	A_07_P016974	b3606	cmr|orf, hypothetical protein [b3606]
yibT	+2.18	9.55	+1.13	8.98	0.1	10.11	0.1	0.00102065	0.00662100	0.05228404	0.56419465	A_07_P010599	ABE-0285075	asap|predicted protein [b4554]
yicL	+2.59	8.44	+1.37	7.75	0.4	9.13	0.1	0.00253408	0.01328788	0.03769309	0.48682527	A_07_P041337	c_4582	cmr|Hypothetical transport protein yicL [c_4582]
yicL	+2.54	8.72	+1.34	8.05	0.1	9.40	0.1	0.00031118	0.00275220	0.03742251	0.48692562	A_07_P052211	Z5146	cmr|putative permease transporter [Z5146]
yicL	+2.26	8.46	+1.18	7.87	0.0	9.05	0.1	0.00055518	0.00419421	0.04797828	0.54421470	A_07_P017240	b3660	cmr|putative permease transporter [b3660]
yidC	-2.12	12.75	-1.08	13.29	0.1	12.21	0.2	0.00176964	0.01003645	0.06166286	0.63535340	A_07_P005451	b3705	cmr|60 KD inner-membrane protein [b3705]
yidD	-2.53	13.48	-1.34	14.15	0.1	12.81	0.1	0.00041062	0.00339000	0.03488885	0.51191985	A_07_P021528	ABE-0285078	asap|predicted protein [b4557]
yidF	+4.28	9.11	+2.10	8.07	0.0	10.16	0.1	0.00001918	0.00038277	0.01392213	0.31905386	A_07_P017305	b3674	cmr|putative transcriptional regulator [b3674]
yidS	-3.96	8.04	-1.98	9.04	0.0	7.05	0.0	0.00002413	0.00044487	0.00732088	0.27377672	A_07_P039007	c_4611	cmr|Hypothetical protein yidS precursor [c_4611]
yidS	-2.94	7.96	-1.56	8.74	0.1	7.18	0.1	0.00013667	0.00151031	0.02116552	0.41863065	A_07_P051822	Z5186	cmr|orf, hypothetical protein [Z5186]
yieF	+2.94	11.49	+1.56	10.71	0.2	12.27	0.1	0.00030766	0.00272585	0.02835874	0.43858546	A_07_P005486	b3713	cmr|orf, hypothetical protein [b3713]
yieG	-2.14	9.48	-1.10	10.03	0.1	8.93	0.2	0.00245014	0.01292295	0.06063516	0.63305040	A_07_P039030	c_4637	cmr|Hypothetical protein yieG [c_4637]
yieP	+2.92	10.45	+1.54	9.68	0.1	11.22	0.2	0.00023764	0.00226205	0.02883638	0.43992248	A_07_P005698	b3755	cmr|orf, hypothetical protein [b3755]
yifE	+6.88	9.97	+2.78	8.58	0.2	11.36	0.1	0.00000667	0.00019764	0.00526169	0.20814070	A_07_P005711	b3764	cmr|orf, hypothetical protein [b3764]
yifK	+2.02	7.11	+1.01	6.61	0.1	7.62	0.0	0.00182314	0.01027329	0.06263783	0.60687385	A_07_P005861	b3795	cmr|putative amino acid/amine transport protein [b3795]
yifK	+2.04	6.96	+1.03	6.45	0.0	7.47	0.0	0.00118955	0.00738206	0.05985264	0.59753963	A_07_P005861	b3795	cmr|putative amino acid/amine transport protein [b3795]
yifK	+5.32	8.95	+2.41	7.75	0.2	10.16	0.1	0.00002417	0.00044487	0.00892022	0.26299625	A_07_P041930	c_4717	cmr|Probable transport protein yifK [c_4717]
yifL	+3.76	10.16	+1.91	9.20	0.0	11.11	0.1	0.00004968	0.00073865	0.01786776	0.35251880	A_07_P010621	ABE-0285079	asap|predicted lipoprotein [b4558]
yigB	+2.18	9.08	+1.12	8.52	0.1	9.64	0.2	0.00141545	0.00839670	0.05254065	0.56425648	A_07_P049994	Z5329	cmr|putative phosphatase [Z5329]
yigB	+3.17	8.87	+1.67	8.04	0.0	9.70	0.1	0.00007652	0.00098987	0.02399136	0.41101197	A_07_P039117	c_4733	cmr|Hypothetical protein yigB [c_4733]
yigI	-3.03	10.24	-1.60	11.04	0.1	9.43	0.1	0.00015089	0.00162606	0.01893991	0.39547745	A_07_P005970	b3820	cmr|orf, hypothetical protein [b3820]
yigM	-2.53	8.12	-1.34	8.79	0.1	7.45	0.1	0.00034051	0.00292952	0.03485074	0.51183769	A_07_P018941	b3827	cmr|orf, hypothetical protein [b3827]
yigP	-3.81	12.85	-1.93	13.81	0.0	11.88	0.3	0.00022779	0.00220021	0.00869284	0.30211921	A_07_P018976	b3834	cmr|orf, hypothetical protein [b3834]
yihA	+4.67	9.69	+2.22	8.58	0.3	10.80	0.1	0.00010799	0.00126877	0.01165523	0.29313099	A_07_P047013	Z5400	cmr|orf, hypothetical protein [Z5400]
yihA	+3.69	10.45	+1.89	9.50	0.0	11.39	0.1	0.00004297	0.00066061	0.01849212	0.35548230	A_07_P019103	b3865	cmr|orf, hypothetical protein [b3865]
yihA	+3.94	9.82	+1.98	8.83	0.1	10.80	0.3	0.00023884	0.00227025	0.01582317	0.33986357	A_07_P039310	c_4812	cmr|Probable GTP-binding protein engB [c_4812]
yihD	+37.50	10.06	+5.23	7.44	0.3	12.67	0.3	0.00000135	0.00008597	0.00006479	0.02125000	A_07_P019075	b3858	cmr|orf, hypothetical protein [b3858]
yihI	+9.37	8.91	+3.23	7.30	0.1	10.52	0.2	0.00000243	0.00011427	0.00296494	0.15770270	A_07_P019108	b3866	cmr|orf, hypothetical protein [b3866]
yihX	+4.69	10.18	+2.23	9.06	0.1	11.29	0.0	0.00001277	0.00029390	0.01161458	0.29304487	A_07_P019205	b3885	cmr|putative phosphatase [b3885]
yiiD	+2.86	8.67	+1.51	7.91	0.2	9.42	0.1	0.00033082	0.00286649	0.02977833	0.44312854	A_07_P019216	b3888	cmr|putative acetyltransferase [b3888]
yiiD	+4.05	9.47	+2.02	8.46	0.3	10.47	0.2	0.00020602	0.00203613	0.01551893	0.33654270	A_07_P041956	c_4835	cmr|Hypothetical protein yiiD [c_4835]
yiiE	-10.22	8.93	-3.35	10.61	0.1	7.25	0.1	0.00000186	0.00009954	0.00018483	0.04476923	A_07_P019225	b3889	cmr|orf, hypothetical protein [b3889]
yiiQ	+2.60	8.17	+1.38	7.48	0.1	8.86	0.5	0.00414888	0.01960973	0.03764037	0.48654351	A_07_P019377	b3920	cmr|orf, hypothetical protein [b3920]
yiiS	+5.61	8.90	+2.49	7.65	0.1	10.14	0.2	0.00002157	0.00041173	0.00807292	0.24921569	A_07_P039426	c_4874	cmr|Hypothetical protein yiiS [c_4874]
yiiS	+8.08	8.61	+3.02	7.11	0.0	10.12	0.0	0.00000185	0.00009954	0.00385988	0.18032641	A_07_P019386	b3922	cmr|orf, hypothetical protein [b3922]
yiiS	+5.27	8.88	+2.40	7.68	0.3	10.08	0.2	0.00006043	0.00084243	0.00890307	0.26298311	A_07_P047106	Z5467	cmr|orf, hypothetical protein [Z5467]
yiiT	+5.08	10.09	+2.35	8.92	0.1	11.26	0.1	0.00001234	0.00028963	0.00984820	0.27345679	A_07_P047111	Z5468	cmr|putative regulator [Z5468]
yiiT	+4.45	10.04	+2.16	8.97	0.0	11.12	0.1	0.00001849	0.00037316	0.01287792	0.30534639	A_07_P039431	c_4875	cmr|Hypothetical protein yiiT [c_4875]
yiiU	+4.51	12.93	+2.17	11.84	0.1	14.01	0.2	0.00003763	0.00059968	0.01270452	0.30398176	A_07_P019420	b3928	cmr|orf, hypothetical protein [b3928]
yiiX	-2.82	9.62	-1.49	10.37	0.1	8.87	0.1	0.00017995	0.00185653	0.02436103	0.45016432	A_07_P052282	Z5492	cmr|orf, hypothetical protein [Z5492]
yiiX	-3.52	8.88	-1.82	9.79	0.0	7.97	0.1	0.00006413	0.00087946	0.01104484	0.32809434	A_07_P041984	c_4890	cmr|Hypothetical protein yiiX precursor [c_4890]
yiiX	-2.05	7.92	-1.04	8.44	0.0	7.40	0.0	0.00107126	0.00685888	0.06883067	0.65756675	A_07_P019465	b3937	cmr|orf, hypothetical protein [b3937]
yijF	-3.80	12.85	-1.92	13.81	0.2	11.89	0.0	0.00005345	0.00077495	0.00863186	0.30267261	A_07_P039476	c_4902	cmr|Hypothetical protein yijF precursor [c_4902]
yijF	-4.64	13.00	-2.21	14.11	0.4	11.89	0.2	0.00024976	0.00234620	0.00437309	0.21719243	A_07_P019499	b3944	cmr|orf, hypothetical protein [b3944]
yjaA	-6.11	9.75	-2.61	11.06	0.1	8.45	0.2	0.00001425	0.00031323	0.00159362	0.12866667	A_07_P008699	b4011	cmr|orf, hypothetical protein [b4011]
yjaB	-2.74	12.70	-1.45	13.43	0.1	11.97	0.6	0.00668606	0.02865544	0.02536776	0.45959724	A_07_P008704	b4012	cmr|orf, hypothetical protein [b4012]
yjaG	+3.60	10.14	+1.85	9.21	0.1	11.06	0.3	0.00017211	0.00179690	0.01880399	0.35928398	A_07_P008660	b3999	cmr|orf, hypothetical protein [b3999]
yjbG	-2.96	11.49	-1.57	12.28	0.1	10.71	0.1	0.00012354	0.00139625	0.02080285	0.41405815	A_07_P008783	b4028	cmr|orf, hypothetical protein [b4028]
yjbJ	-3.17	11.16	-1.67	11.99	0.1	10.32	0.1	0.00011249	0.00130510	0.01625127	0.37681885	A_07_P037555	c_5016	cmr|Protein yjbJ [c_5016]
yjbQ	+5.15	9.24	+2.37	8.06	0.2	10.43	0.2	0.00004203	0.00064895	0.00964050	0.27103571	A_07_P008923	b4056	cmr|orf, hypothetical protein [b4056]
yjcB	-10.60	9.96	-3.41	11.66	0.1	8.26	0.1	0.00000124	0.00008210	0.00014164	0.03716667	A_07_P008942	b4060	cmr|orf, hypothetical protein [b4060]
yjcB	-9.93	9.71	-3.31	11.37	0.1	8.06	0.1	0.00000185	0.00009954	0.00021214	0.04638889	A_07_P035567	c_5050	cmr|Hypothetical protein yjcB [c_5050]
yjcC	-2.28	8.20	-1.19	8.79	0.1	7.60	0.0	0.00060214	0.00444197	0.04943534	0.58519549	A_07_P052319	Z5660	cmr|orf, hypothetical protein [Z5660]
yjcE	+2.11	7.62	+1.08	7.08	0.3	8.16	0.2	0.00491085	0.02248224	0.05587716	0.57915076	A_07_P008970	b4065	cmr|orf, hypothetical protein [b4065]
yjcH	+2.84	9.29	+1.51	8.54	0.1	10.05	0.2	0.00046798	0.00372682	0.03014291	0.44518762	A_07_P037683	c_5063	cmr|Hypothetical protein yjcH [c_5063]
yjcH	+3.84	9.84	+1.94	8.87	0.0	10.81	0.1	0.00003660	0.00058739	0.01718496	0.34865979	A_07_P037683	c_5063	cmr|Hypothetical protein yjcH [c_5063]
yjcH	+2.68	9.45	+1.42	8.74	0.1	10.16	0.1	0.00026773	0.00246792	0.03364075	0.46870796	A_07_P037683	c_5063	cmr|Hypothetical protein yjcH [c_5063]
yjcH	+2.94	9.54	+1.56	8.76	0.0	10.32	0.1	0.00016122	0.00171124	0.02792048	0.43393880	A_07_P037683	c_5063	cmr|Hypothetical protein yjcH [c_5063]
yjcO	-2.23	9.45	-1.16	10.03	0.0	8.87	0.1	0.00062468	0.00455958	0.05350356	0.60125625	A_07_P009034	b4078	cmr|orf, hypothetical protein [b4078]
yjcO	-2.36	9.77	-1.24	10.39	0.1	9.15	0.1	0.00048925	0.00384271	0.04449251	0.56354787	A_07_P009034	b4078	cmr|orf, hypothetical protein [b4078]
yjcO	-2.47	9.67	-1.31	10.32	0.1	9.01	0.2	0.00069856	0.00492527	0.03805386	0.52785903	A_07_P009034	b4078	cmr|orf, hypothetical protein [b4078]
yjcO	-2.28	9.49	-1.19	10.09	0.0	8.90	0.3	0.00256557	0.01341728	0.04996126	0.58569620	A_07_P009034	b4078	cmr|orf, hypothetical protein [b4078]
yjcW	+3.32	9.90	+1.73	9.03	0.2	10.76	0.2	0.00033142	0.00286975	0.02253493	0.39953829	A_07_P037736	c_5093	cmr|D-allose transport ATP-binding protein alsA [c_5093]
yjdB	+3.17	7.84	+1.67	7.01	0.2	8.67	0.2	0.00028902	0.00261510	0.02444106	0.41465517	A_07_P037782	c_5119	cmr|Hypothetical protein yjdB [c_5119]
yjdC	+4.97	9.19	+2.31	8.04	0.0	10.35	0.1	0.00001166	0.00027951	0.01027248	0.28029463	A_07_P011409	b4135	cmr|orf, hypothetical protein [b4135]
yjdJ	+2.66	8.43	+1.41	7.73	0.0	9.14	0.0	0.00018826	0.00191593	0.03393483	0.47072247	A_07_P047348	Z5729	cmr|orf, hypothetical protein [Z5729]
yjdK	+2.13	8.29	+1.09	7.74	0.2	8.83	0.1	0.00162618	0.00939890	0.05593941	0.57941447	A_07_P011380	b4128	cmr|orf, hypothetical protein [b4128]
yjdN	-3.09	8.58	-1.63	9.40	0.1	7.77	0.0	0.00010369	0.00123350	0.01786458	0.38634615	A_07_P011271	b4107	cmr|orf, hypothetical protein [b4107]
yjeI	+4.88	11.41	+2.29	10.27	0.0	12.55	0.1	0.00001217	0.00028817	0.01081491	0.28425710	A_07_P011455	b4144	cmr|orf, hypothetical protein [b4144]
yjeK	-2.01	8.93	-1.01	9.43	0.1	8.42	0.1	0.00141133	0.00837859	0.07272040	0.67032201	A_07_P011461	b4146	cmr|orf, hypothetical protein [b4146]
yjeS	-11.62	9.73	-3.54	11.50	0.0	7.96	0.2	0.00000189	0.00010039	0.00011115	0.03645833	A_07_P011550	b4166	cmr|orf, hypothetical protein [b4166]
yjfC	-2.20	7.47	-1.14	8.04	0.3	6.91	0.3	0.00639315	0.02767496	0.05340765	0.60146638	A_07_P011646	b4186	cmr|putative synthetase/amidase [b4186]
yjfL	-2.10	7.21	-1.07	7.74	0.0	6.67	0.0	0.00088502	0.00597250	0.06393166	0.64604621	A_07_P011636	b4184	cmr|orf, hypothetical protein [b4184]
yjfN	+12.23	10.66	+3.61	8.85	0.0	12.47	0.1	0.00000074	0.00006247	0.00169588	0.12191781	A_07_P011660	b4188	cmr|orf, hypothetical protein [b4188]
yjfO	+16.41	12.57	+4.04	10.55	0.1	14.58	0.1	0.00000050	0.00005264	0.00086128	0.08862745	A_07_P035589	c_5277	cmr|Hypothetical lipoprotein yjfO precursor [c_5277]
yjfO	+11.55	10.61	+3.53	8.85	0.1	12.38	0.2	0.00000196	0.00010186	0.00190422	0.12978355	A_07_P011661	b4189	cmr|orf, hypothetical protein [b4189]
yjfO	+15.11	11.29	+3.92	9.33	0.1	13.25	0.1	0.00000047	0.00005187	0.00101181	0.09654545	A_07_P010658	b4189	cmr|orf, hypothetical protein [b4189]
yjfO	+12.04	10.89	+3.59	9.10	0.2	12.69	0.1	0.00000188	0.00010005	0.00164825	0.12069767	A_07_P048630	Z5796	cmr|orf, hypothetical protein [Z5796]
yjgA	-6.06	12.66	-2.60	13.96	0.0	11.36	0.0	0.00000457	0.00016295	0.00157647	0.12927083	A_07_P037054	c_5332	cmr|Hypothetical protein yjgA [c_5332]
yjgA	-4.84	12.57	-2.27	13.71	0.0	11.43	0.1	0.00001126	0.00027354	0.00342607	0.19402878	A_07_P003722	b4234	cmr|putative alpha helix protein [b4234]
yjgB	-3.58	9.36	-1.84	10.28	0.8	8.44	0.4	0.01142190	0.04367897	0.01036204	0.32369048	A_07_P003891	b4269	cmr|putative oxidoreductase [b4269]
yjgF	+54.84	11.57	+5.78	8.68	0.2	14.46	0.1	0.00000011	0.00003526	0.00001524	0.01043478	A_07_P037066	c_5342	cmr|Protein yjgF [c_5342]
yjgF	+21.99	9.98	+4.46	7.75	0.1	12.21	0.1	0.00000019	0.00003706	0.00040142	0.05798165	A_07_P003767	b4243	cmr|orf, hypothetical protein [b4243]
yjgP	-3.58	10.30	-1.84	11.22	0.1	9.38	0.2	0.00011999	0.00136297	0.01026677	0.32457831	A_07_P003851	b4261	cmr|orf, hypothetical protein [b4261]
yjgQ	-4.01	11.14	-2.01	12.15	0.0	10.14	0.0	0.00002701	0.00047893	0.00684261	0.26534483	A_07_P003856	b4262	cmr|orf, hypothetical protein [b4262]
yjgR	+2.59	8.56	+1.37	7.88	0.5	9.25	0.1	0.00534099	0.02401652	0.03747332	0.48678218	A_07_P003863	b4263	cmr|orf, hypothetical protein [b4263]
yjgR	+8.79	9.30	+3.14	7.74	0.0	10.87	0.2	0.00000410	0.00015128	0.00323742	0.16602606	A_07_P037116	c_5364	cmr|Hypothetical protein yjgR [c_5364]
yjhD	-3.03	9.13	-1.60	9.93	0.3	8.33	0.5	0.00402287	0.01910016	0.01837779	0.38942127	A_07_P003936	b4281	cmr|orf, hypothetical protein [b4281]
yjhQ	+9.47	9.04	+3.24	7.42	0.1	10.67	0.2	0.00000539	0.00017798	0.00279281	0.15428070	A_07_P004061	b4307	cmr|orf, hypothetical protein [b4307]
yjhQ	+11.11	9.37	+3.47	7.63	0.4	11.10	0.1	0.00001428	0.00031323	0.00198806	0.13041667	A_07_P004061	b4307	cmr|orf, hypothetical protein [b4307]
yjhQ	+10.69	9.29	+3.42	7.58	0.2	11.00	0.0	0.00000273	0.00012064	0.00222053	0.13656250	A_07_P004061	b4307	cmr|orf, hypothetical protein [b4307]
yjhQ	+6.55	9.27	+2.71	7.92	0.3	10.63	0.2	0.00004463	0.00067949	0.00598260	0.22479714	A_07_P004061	b4307	cmr|orf, hypothetical protein [b4307]
yjhQ	+9.69	9.54	+3.28	7.91	0.1	11.18	0.1	0.00000153	0.00009287	0.00274073	0.15355872	A_07_P004061	b4307	cmr|orf, hypothetical protein [b4307]
yjhR	-3.02	11.75	-1.59	12.55	0.1	10.96	0.2	0.00035186	0.00300256	0.01933562	0.39950131	A_07_P004068	b4308	cmr|putative frameshift suppressor [b4308]
yjhU	-2.18	9.81	-1.12	10.37	0.1	9.24	0.1	0.00125351	0.00769406	0.05702426	0.61746217	A_07_P004004	b4295	cmr|orf, hypothetical protein [b4295]
yjhX	+7.72	10.18	+2.95	8.71	0.0	11.66	0.2	0.00000368	0.00014246	0.00415714	0.18541076	A_07_P010688	ABE-0285087	asap|conserved protein [b4566]
yjiG	+6.49	8.80	+2.70	7.45	0.0	10.15	0.1	0.00000526	0.00017546	0.00613758	0.22577103	A_07_P004174	b4329	cmr|orf, hypothetical protein [b4329]
yjiH	+3.03	7.79	+1.60	6.99	0.1	8.59	0.2	0.00025077	0.00235010	0.02644817	0.42663934	A_07_P004179	b4330	cmr|orf, hypothetical protein [b4330]
yjiH	+2.16	7.37	+1.11	6.82	0.0	7.93	0.1	0.00089906	0.00604647	0.05354421	0.56920999	A_07_P037230	c_5410	cmr|Hypothetical protein yjiH [c_5410]
yjiL	+4.69	9.72	+2.23	8.60	0.2	10.83	0.2	0.00005770	0.00081407	0.01156822	0.29328502	A_07_P004196	b4334	cmr|putative enzyme [b4334]
yjiM	+9.51	9.27	+3.25	7.64	0.1	10.89	0.1	0.00000192	0.00010099	0.00285442	0.15604167	A_07_P004201	b4335	cmr|orf, hypothetical protein [b4335]
yjiX	+3.39	9.54	+1.76	8.66	0.1	10.42	0.0	0.00006792	0.00091783	0.02136687	0.38666667	A_07_P017462	b4353	cmr|orf, hypothetical protein [b4353]
yjjI	+5.49	10.74	+2.46	9.51	0.0	11.96	0.0	0.00000679	0.00019947	0.00827934	0.25164093	A_07_P017585	b4380	cmr|orf, hypothetical protein [b4380]
yjjK	+4.27	10.08	+2.09	9.03	0.1	11.13	0.3	0.00008162	0.00103465	0.01360645	0.31642541	A_07_P017637	b4391	cmr|putative ATP-binding component of a transport system [b4391]
yjjK	+5.23	10.22	+2.39	9.03	0.0	11.41	0.1	0.00000851	0.00022936	0.00928417	0.26820183	A_07_P017637	b4391	cmr|putative ATP-binding component of a transport system [b4391]
yjjK	+4.97	10.08	+2.31	8.92	0.0	11.24	0.1	0.00001038	0.00025778	0.01028201	0.27958549	A_07_P017637	b4391	cmr|putative ATP-binding component of a transport system [b4391]
yjjK	+4.67	10.18	+2.22	9.07	0.0	11.30	0.0	0.00001232	0.00028963	0.01182673	0.29508716	A_07_P017637	b4391	cmr|putative ATP-binding component of a transport system [b4391]
yjjK	+5.06	10.28	+2.34	9.11	0.0	11.45	0.1	0.00001121	0.00027288	0.00991489	0.27434095	A_07_P017637	b4391	cmr|putative ATP-binding component of a transport system [b4391]
yjjM	+2.73	7.95	+1.45	7.22	0.3	8.67	0.1	0.00144191	0.00851197	0.03368648	0.46893015	A_07_P017484	b4357	cmr|orf, hypothetical protein [b4357]
yjjN	-2.44	7.62	-1.29	8.26	0.0	6.98	0.1	0.00037135	0.00313916	0.03950775	0.53900347	A_07_P017488	b4358	cmr|putative oxidoreductase [b4358]
yjjN	-2.20	7.51	-1.14	8.08	0.0	6.94	0.0	0.00062192	0.00454361	0.05533981	0.60800419	A_07_P017488	b4358	cmr|putative oxidoreductase [b4358]
yjjN	-2.00	7.71	-1.00	8.21	0.0	7.20	0.1	0.00149230	0.00874714	0.07392022	0.67388535	A_07_P017488	b4358	cmr|putative oxidoreductase [b4358]
yjjQ	-2.14	7.34	-1.10	7.89	0.2	6.79	0.2	0.00216396	0.01175212	0.05944995	0.62691226	A_07_P042086	c_5444	cmr|Hypothetical protein yjjQ [c_5444]
yjjQ	-3.09	7.47	-1.63	8.29	0.0	6.66	0.1	0.00009464	0.00114798	0.01798590	0.38684426	A_07_P017521	b4365	cmr|putative regulator [b4365]
yjjW	+2.21	8.60	+1.14	8.03	0.1	9.17	0.1	0.00087351	0.00591505	0.05043953	0.55845288	A_07_P050864	Z5981	cmr|putative activating enzyme [Z5981]
yjjW	+2.31	8.28	+1.21	7.67	0.2	8.88	0.0	0.00089879	0.00604647	0.04631542	0.53186725	A_07_P017579	b4379	cmr|putative activating enzyme [b4379]
yjjX	-2.86	10.70	-1.52	11.46	0.2	9.94	0.0	0.00025596	0.00239014	0.02337716	0.44236779	A_07_P017655	b4394	cmr|orf, hypothetical protein [b4394]
ykfJ	-14.43	11.17	-3.85	13.10	0.0	9.25	0.0	0.00000040	0.00004930	0.00004319	0.02125000	A_07_P007889	b0235	cmr|orf, hypothetical protein [b0235]
ykgJ	-3.73	8.47	-1.90	9.42	0.3	7.52	0.2	0.00030624	0.00272199	0.00921557	0.30804671	A_07_P008131	b0288	cmr|putative ferredoxin [b0288]
ykgL	-2.18	8.01	-1.13	8.57	0.1	7.45	0.2	0.00147951	0.00869077	0.05642721	0.61352901	A_07_P043583	Z0363	cmr|orf, hypothetical protein [Z0363]
ykiA	-2.21	7.60	-1.14	8.17	0.2	7.03	0.3	0.00492821	0.02253072	0.05516578	0.60778866	A_07_P021074	b0392	cmr|orf, hypothetical protein [b0392]
ylaB	-2.10	11.23	-1.07	11.76	0.1	10.69	0.0	0.00123195	0.00759130	0.06425432	0.64681586	A_07_P021391	b0457	cmr|orf, hypothetical protein [b0457]
ylaC	-12.25	12.04	-3.61	13.84	0.0	10.23	0.2	0.00000166	0.00009590	0.00009083	0.03325581	A_07_P021396	b0458	cmr|orf, hypothetical protein [b0458]
ylbA	-3.16	11.80	-1.66	12.63	0.3	10.97	0.0	0.00053963	0.00410825	0.01633575	0.37711144	A_07_P016365	b0515	cmr|orf, hypothetical protein [b0515]
ylbA	+2.18	7.77	+1.12	7.21	0.1	8.33	0.1	0.00108379	0.00691937	0.05167556	0.56031680	A_07_P031240	c_0629	cmr|Hypothetical protein ylbA [c_0629]
yliJ	+2.20	10.14	+1.14	9.57	0.2	10.71	0.1	0.00188351	0.01051561	0.05249365	0.56413652	A_07_P004644	b0838	cmr|putative transferase [b0838]
ymcE	+6.19	9.63	+2.63	8.31	0.0	10.94	0.2	0.00001001	0.00025069	0.00659426	0.23278027	A_07_P001189	b0991	cmr|suppresses fabA and ts growth mutation [b0991]
ymdF	-3.45	12.20	-1.79	13.09	0.0	11.31	0.3	0.00032282	0.00281814	0.01231072	0.33825480	A_07_P010350	ABE-0285039	asap|conserved protein [b4518]
ymfA	-4.18	8.67	-2.06	9.70	0.0	7.64	0.1	0.00003247	0.00054377	0.00603341	0.25330667	A_07_P040833	c_1397	cmr|Hypothetical protein ymfA [c_1397]
ymfC	-6.05	13.22	-2.60	14.52	0.0	11.92	0.1	0.00000721	0.00020759	0.00162030	0.12949239	A_07_P050485	Z1864	cmr|orf, hypothetical protein [Z1864]
ymfE	+2.05	7.64	+1.04	7.12	0.0	8.16	0.0	0.00103589	0.00669502	0.05890816	0.59337812	A_07_P006641	b1138	cmr|orf, hypothetical protein [b1138]
ymfS	-4.35	8.94	-2.12	10.00	0.6	7.88	0.1	0.00167103	0.00959124	0.00549860	0.24249300	A_07_P006729	b1155	cmr|orf, hypothetical protein [b1155]
ymgA	+8.37	8.21	+3.07	6.68	0.1	9.74	0.1	0.00000369	0.00014246	0.00360391	0.17298780	A_07_P006777	b1165	cmr|orf, hypothetical protein [b1165]
ymgB	+15.56	10.63	+3.96	8.65	0.2	12.61	0.4	0.00001142	0.00027623	0.00093941	0.09360759	A_07_P038703	c_1609	cmr|Hypothetical protein ymgB [c_1609]
ymgD	-2.19	8.89	-1.13	9.45	0.3	8.32	0.2	0.00429942	0.02019393	0.05593051	0.61108258	A_07_P006807	b1171	cmr|orf, hypothetical protein [b1171]
ymgG	-4.05	12.66	-2.02	13.67	0.0	11.65	0.0	0.00002218	0.00041939	0.00672320	0.26396509	A_07_P006812	b1172	cmr|orf, hypothetical protein [b1172]
ynaI	-6.79	10.59	-2.76	11.97	0.1	9.21	0.1	0.00000529	0.00017598	0.00106072	0.10372671	A_07_P014632	b1330	cmr|orf, hypothetical protein [b1330]

ynaK	-5.94	8.79	-2.57	10.08	0.3	7.50	0.2	0.00003212	0.00054058	0.00165587	0.13166667	A_07_P014801	b1365	cmr|orf, hypothetical protein [b1365]
ynbE	+2.90	7.87	+1.54	7.10	0.0	8.64	0.1	0.00019000	0.00193111	0.02856707	0.43879024	A_07_P034809	c_1829	cmr|Hypothetical protein ynbE [c_1829]
ynbE	-3.13	8.55	-1.65	9.37	0.7	7.73	0.2	0.00873328	0.03525557	0.01659172	0.37694084	A_07_P048215	Z2327	cmr|orf, hypothetical protein [Z2327]
yncJ	-2.05	8.62	-1.03	9.13	0.1	8.10	0.0	0.00136874	0.00819026	0.06960493	0.65896572	A_07_P009272	b1436	cmr|orf, hypothetical protein [b1436]
yneH	-4.68	9.89	-2.23	11.00	0.1	8.78	0.4	0.00031745	0.00278588	0.00433054	0.21575949	A_07_P020270	b1524	cmr|putative glutaminase [b1524]
yneI	-3.23	9.25	-1.69	10.09	0.0	8.40	0.4	0.00070128	0.00493783	0.01574378	0.37329819	A_07_P035026	c_1948	cmr|Aldehyde-dehydrogenase like protein yneI [c_1948]
yneJ	-2.16	11.39	-1.11	11.94	0.0	10.83	0.1	0.00093152	0.00618762	0.05880653	0.62346801	A_07_P020279	b1526	cmr|putative transcriptional regulator LYSR-type [b1526]
ynfB	-5.24	13.95	-2.39	15.14	0.0	12.75	0.0	0.00000770	0.00021737	0.00266959	0.17016194	A_07_P020550	b1583	cmr|orf, hypothetical protein [b1583]
ynfE	+5.41	8.10	+2.44	6.88	0.3	9.31	0.2	0.00007463	0.00097352	0.00875000	0.25992453	A_07_P020568	b1587	cmr|putative oxidoreductase, major subunit [b1587]
ynfE	+7.04	8.66	+2.82	7.25	0.0	10.07	0.1	0.00000339	0.00013496	0.00506606	0.20716883	A_07_P042301	c_1977	cmr|Putative dimethyl sulfoxide reductase chain ynfE precursor [c_1977]
ynfF	+3.70	9.48	+1.89	8.54	0.3	10.42	0.1	0.00018648	0.00190154	0.01825775	0.35313268	A_07_P020575	b1588	cmr|putative oxidoreductase, major subunit [b1588]
ynfG	+11.05	9.05	+3.47	7.32	0.3	10.79	0.0	0.00000387	0.00014511	0.00204840	0.13163265	A_07_P020576	b1589	cmr|putative oxidoreductase, Fe-S subunit [b1589]
ynfH	+2.17	8.75	+1.12	8.19	0.2	9.31	0.1	0.00198433	0.01098886	0.05406504	0.56936455	A_07_P020582	b1590	cmr|putative DMSO reductase anchor subunit [b1590]
ynfL	-2.61	10.97	-1.39	11.67	0.2	10.28	0.1	0.00068132	0.00484060	0.03175622	0.49847458	A_07_P020609	b1595	cmr|putative transcriptional regulator LYSR-type [b1595]
ynfM	-4.13	11.47	-2.05	12.49	0.4	10.45	0.3	0.00044089	0.00356882	0.00668509	0.26378446	A_07_P020611	b1596	cmr|putative transport protein [b1596]
yniA	+9.58	9.05	+3.26	7.42	0.0	10.68	0.1	0.00000135	0.00008589	0.00279599	0.15391608	A_07_P018674	b1725	cmr|orf, hypothetical protein [b1725]
ynjA	+2.25	8.48	+1.17	7.90	0.1	9.06	0.1	0.00094706	0.00626493	0.04924225	0.55218661	A_07_P018811	b1753	cmr|orf, hypothetical protein [b1753]
ynjB	+3.69	8.05	+1.88	7.10	0.0	8.99	0.1	0.00003509	0.00057133	0.01850927	0.35537805	A_07_P018816	b1754	cmr|orf, hypothetical protein [b1754]
ynjE	+6.17	9.42	+2.63	8.11	0.4	10.73	0.1	0.00005523	0.00078910	0.00664380	0.23348214	A_07_P018832	b1757	cmr|putative thiosulfate sulfur transferase [b1757]
yoaB	+8.04	9.53	+3.01	8.03	0.0	11.04	0.0	0.00000183	0.00009954	0.00390117	0.18064706	A_07_P015736	b1809	cmr|orf, hypothetical protein [b1809]
yobB	+2.69	8.75	+1.43	8.04	0.1	9.46	0.1	0.00021968	0.00214234	0.03339431	0.46775801	A_07_P015892	b1843	cmr|orf, hypothetical protein [b1843]
yohF	-2.08	9.86	-1.05	10.39	0.0	9.33	0.1	0.00113773	0.00715066	0.06670795	0.64910383	A_07_P003448	b2137	cmr|putative oxidoreductase [b2137]
ypfG	-5.70	9.88	-2.51	11.14	0.0	8.62	0.1	0.00000778	0.00021851	0.00200584	0.14688372	A_07_P042601	c_2991	cmr|Hypothetical protein ypfG precursor [c_2991]
ypfG	-3.32	9.65	-1.73	10.51	0.1	8.78	0.1	0.00007107	0.00094588	0.01381415	0.35364228	A_07_P013386	b2466	cmr|orf, hypothetical protein [b2466]
yphE	-5.40	12.61	-2.43	13.82	0.0	11.39	0.3	0.00005361	0.00077607	0.00252858	0.16940426	A_07_P019639	b2547	cmr|putative ATP-binding component of a transport system [b2547]
yphF	-2.01	8.03	-1.00	8.53	0.2	7.53	0.1	0.00432552	0.02029232	0.07464304	0.67577918	A_07_P019645	b2548	cmr|putative LACI-type transcriptional regulator [b2548]
yqaA	-3.84	9.82	-1.94	10.79	0.1	8.85	0.2	0.00007240	0.00095580	0.00832444	0.29718821	A_07_P001920	b2689	cmr|orf, hypothetical protein [b2689]
yqaB	-7.67	11.18	-2.94	12.64	0.0	9.71	0.0	0.00000231	0.00011009	0.00067962	0.08167939	A_07_P030582	c_3247	cmr|Hypothetical protein yqaB [c_3247]
yqaB	-4.93	11.00	-2.30	12.15	0.0	9.84	0.2	0.00003681	0.00059023	0.00330158	0.19251852	A_07_P001923	b2690	cmr|putative phosphatase [b2690]
yqcA	+4.38	9.75	+2.13	8.68	0.1	10.81	0.1	0.00002302	0.00043145	0.01338605	0.31315007	A_07_P002396	b2790	cmr|orf, hypothetical protein [b2790]
yqeB	-2.49	8.58	-1.32	9.24	0.2	7.92	0.0	0.00060986	0.00447491	0.03690485	0.52486902	A_07_P006342	b2875	cmr|putative synthases [b2875]
yqeC	-2.81	8.62	-1.49	9.37	0.2	7.88	0.3	0.00132760	0.00802315	0.02499682	0.45549769	A_07_P006347	b2876	cmr|orf, hypothetical protein [b2876]
yqfA	+2.64	9.63	+1.40	8.93	0.1	10.33	0.1	0.00028109	0.00256847	0.03491552	0.47552768	A_07_P006461	b2899	cmr|putative oxidoreductase [b2899]
yqfB	+2.35	7.64	+1.23	7.03	0.1	8.26	0.0	0.00043810	0.00355725	0.04423971	0.52173034	A_07_P045648	Z4238	cmr|orf, hypothetical protein [Z4238]
yqfB	+2.53	7.62	+1.34	6.95	0.1	8.29	0.0	0.00037361	0.00315226	0.03790523	0.48756536	A_07_P045648	Z4238	cmr|orf, hypothetical protein [Z4238]
yqfB	+2.68	10.14	+1.42	9.43	0.1	10.86	0.0	0.00021144	0.00208317	0.03359057	0.46842338	A_07_P006468	b2900	cmr|orf, hypothetical protein [b2900]
yqfB	+2.54	10.45	+1.34	9.78	0.1	11.12	0.1	0.00032480	0.00282698	0.03753493	0.48677924	A_07_P032874	c_3481	cmr|Hypothetical protein yqfB [c_3481]
yqfE	-2.83	12.58	-1.50	13.33	0.2	11.83	0.2	0.00042597	0.00349110	0.02414126	0.44873672	A_07_P006538	b2915	cmr|orf, hypothetical protein [b2915]
yqgE	+3.20	10.60	+1.68	9.76	0.1	11.43	0.2	0.00016307	0.00172651	0.02388275	0.41138950	A_07_P011885	b2948	cmr|orf, hypothetical protein [b2948]
yqhA	-2.81	8.99	-1.49	9.74	0.0	8.25	0.0	0.00013718	0.00151457	0.02494411	0.45559165	A_07_P012144	b3002	cmr|orf, hypothetical protein [b3002]
yqhD	+3.58	10.55	+1.84	9.63	0.2	11.47	0.2	0.00013502	0.00149807	0.01954522	0.36720764	A_07_P012186	b3011	cmr|putative oxidoreductase [b3011]
yqiK	-2.07	7.56	-1.05	8.08	0.1	7.03	0.2	0.00388189	0.01854264	0.06504446	0.64896071	A_07_P036874	c_3799	cmr|Hypothetical protein yqiK [c_3799]
yqjA	-8.36	11.31	-3.06	12.84	0.0	9.78	0.1	0.00000204	0.00010341	0.00045287	0.06790476	A_07_P000202	b3095	cmr|orf, hypothetical protein [b3095]
yqjA	-9.51	10.95	-3.25	12.57	0.0	9.32	0.1	0.00000176	0.00009825	0.00024771	0.05200000	A_07_P041768	c_3853	cmr|Hypothetical protein yqjA [c_3853]
yqjB	-3.12	10.57	-1.64	11.39	0.1	9.74	0.1	0.00019576	0.00196808	0.01689660	0.37948645	A_07_P000206	b3096	cmr|orf, hypothetical protein [b3096]
yqjC	+2.75	10.35	+1.46	9.62	0.1	11.08	0.6	0.00587009	0.02591662	0.03393991	0.47037852	A_07_P000212	b3097	cmr|orf, hypothetical protein [b3097]
yqjI	+3.19	11.25	+1.67	10.42	0.1	12.09	0.1	0.00011083	0.00129056	0.02422574	0.41233514	A_07_P000083	b3071	cmr|orf, hypothetical protein [b3071]
yraP	+4.35	10.29	+2.12	9.23	0.0	11.35	0.1	0.00002504	0.00045582	0.01343432	0.31381306	A_07_P000475	b3150	cmr|putative periplasmic protein [b3150]
yrbA	+2.32	10.14	+1.21	9.53	0.1	10.74	0.2	0.00105497	0.00678214	0.04610582	0.53023375	A_07_P012426	b3190	cmr|orf, hypothetical protein [b3190]
yrbB	+2.05	8.65	+1.04	8.13	0.2	9.16	0.0	0.00247470	0.01303067	0.06097307	0.60147870	A_07_P035537	c_3950	cmr|Hypothetical protein yrbB [c_3950]
yrbD	-2.75	11.07	-1.46	11.80	0.0	10.34	0.2	0.00032624	0.00283779	0.02678290	0.47166667	A_07_P012441	b3193	cmr|orf, hypothetical protein [b3193]
yrbE	-4.02	10.95	-2.01	11.95	0.1	9.94	0.2	0.00007013	0.00093648	0.00674606	0.26354839	A_07_P012449	b3194	cmr|orf, hypothetical protein [b3194]
yrbF	-2.85	10.97	-1.51	11.73	0.2	10.21	0.3	0.00091390	0.00611098	0.02285760	0.43726610	A_07_P012451	b3195	cmr|putative ATP-binding component of a transport system [b3195]
yrbK	+2.22	12.38	+1.15	11.81	0.0	12.96	0.2	0.00133034	0.00802484	0.05082825	0.55804742	A_07_P012474	b3199	cmr|orf, hypothetical protein [b3199]
yrbL	+3.71	9.55	+1.89	8.61	0.3	10.50	0.1	0.00020428	0.00202571	0.01793763	0.35257179	A_07_P052110	Z4570	cmr|orf, hypothetical protein [Z4570]
yrbL	+3.87	10.22	+1.95	9.25	0.0	11.20	0.1	0.00002925	0.00050543	0.01692962	0.34615584	A_07_P012512	b3207	cmr|orf, hypothetical protein [b3207]
yrdA	+2.29	8.70	+1.19	8.10	0.2	9.29	0.2	0.00209648	0.01146076	0.04849339	0.54729749	A_07_P034249	c_4040	cmr|Protein yrdA [c_4040]
yrdA	+2.24	8.91	+1.16	8.32	0.3	9.49	0.0	0.00392623	0.01872602	0.05047193	0.55802669	A_07_P034249	c_4040	cmr|Protein yrdA [c_4040]
yrdA	+2.00	8.70	+1.00	8.20	0.1	9.20	0.2	0.00365365	0.01770167	0.06556593	0.62751976	A_07_P034249	c_4040	cmr|Protein yrdA [c_4040]
yrdD	+2.45	9.03	+1.29	8.38	0.0	9.68	0.2	0.00084821	0.00577854	0.04068153	0.50116588	A_07_P012850	b3283	cmr|putative DNA topoisomerase [b3283]
yrfD	+2.13	7.97	+1.09	7.42	0.1	8.51	0.1	0.00136921	0.00819026	0.05431466	0.57084780	A_07_P034394	c_4165	cmr|Hypothetical protein yrfD [c_4165]
yrfF	-3.86	9.61	-1.95	10.59	0.0	8.64	0.0	0.00002804	0.00049142	0.00794144	0.28742529	A_07_P034400	c_4169	cmr|Putative membrane protein igaA homolog [c_4169]
yrfF	-2.75	9.03	-1.46	9.76	0.0	8.30	0.3	0.00075474	0.00524629	0.02603087	0.46518729	A_07_P018019	b3398	cmr|putative dehydrogenase [b3398]
ytfH	-2.94	10.49	-1.56	11.27	0.2	9.72	0.0	0.00028399	0.00258898	0.02130907	0.41988736	A_07_P003611	b4212	cmr|orf, hypothetical protein [b4212]
ytfH	-2.68	10.23	-1.42	10.94	0.0	9.51	0.1	0.00022308	0.00216400	0.02850356	0.47791267	A_07_P035591	c_5311	cmr|Hypothetical protein ytfH [c_5311]
ytfK	+2.07	8.55	+1.05	8.03	0.4	9.08	0.1	0.00884795	0.03563625	0.06255081	0.60677757	A_07_P035596	c_5315	cmr|Hypothetical protein ytfK [c_5315]
ytfL	-2.12	9.05	-1.08	9.59	0.1	8.50	0.0	0.00109334	0.00695496	0.06216845	0.63805737	A_07_P003641	b4218	cmr|putative transport protein [b4218]
ytfQ	+6.65	9.69	+2.73	8.32	0.2	11.05	0.1	0.00000862	0.00023000	0.00571075	0.21875912	A_07_P037048	c_5325	cmr|ABC transporter Periplasmic binding protein ytfQ precursor [c_5325]
ytfQ	+2.23	8.15	+1.16	7.57	0.0	8.73	0.1	0.00098123	0.00644763	0.04948806	0.55375977	A_07_P003689	b4227	cmr|putative LACI-type transcriptional regulator [b4227]
Z0024	-2.28	8.23	-1.19	8.83	0.3	7.64	0.0	0.00236368	0.01258499	0.04972561	0.58554974	A_07_P049126	Z0024	cmr|putative type-1 fimbrial protein [Z0024]
Z0056	-2.41	12.05	-1.27	12.69	0.1	11.41	0.1	0.00040851	0.00337771	0.04069931	0.54394737	A_07_P049148	Z0056	cmr|putative antitoxin of gyrase inhibiting toxin-antitoxin system [Z0056]
Z0202	-2.54	11.07	-1.34	11.74	0.0	10.39	0.1	0.00028212	0.00257640	0.03491806	0.51187151	A_07_P049248	Z0202	cmr|orf Unknown function [Z0202]
Z0255	-2.22	9.78	-1.15	10.36	0.3	9.21	0.1	0.00424917	0.01999969	0.05366171	0.60260342	A_07_P049311	Z0255	cmr|orf Unknown function [Z0255]
Z0259	-2.01	9.48	-1.01	9.98	0.1	8.97	0.0	0.00169218	0.00967732	0.07333143	0.67084834	A_07_P049331	Z0259	cmr|orf Unknown function [Z0259]
Z0268	-2.09	10.26	-1.07	10.80	0.0	9.73	0.1	0.00100347	0.00654457	0.06483803	0.64813333	A_07_P043359	Z0268	cmr|orf Unknown function [Z0268]
Z0309	-3.79	12.60	-1.92	13.56	0.0	11.64	0.3	0.00012837	0.00143849	0.00878811	0.30408791	A_07_P043395	Z0309	cmr|putative cI repressor protein for prophage CP-933H [Z0309]
Z0310	+2.06	7.61	+1.04	7.09	0.1	8.14	0.2	0.00275069	0.01412489	0.05934959	0.59553856	A_07_P043398	Z0310	cmr|putative cII antiterminator protein for prophage CP-933H [Z0310]
Z0311	+5.59	13.22	+2.48	11.97	0.0	14.46	0.4	0.00007141	0.00094797	0.00822218	0.25087209	A_07_P043403	Z0311	cmr|partial O replication protein for prophage CP-933H [Z0311]
Z0325	-3.25	11.11	-1.70	11.96	0.0	10.26	0.5	0.00157397	0.00914077	0.01532139	0.37167951	A_07_P043462	Z0325	cmr|unknown protein encoded in prophage CP-933I [Z0325]
Z0332	-2.37	9.21	-1.24	9.83	0.1	8.59	0.1	0.00058432	0.00434342	0.04401232	0.56198702	A_07_P043492	Z0332	cmr|putative activator encoded in prophage CP-933I [Z0332]
Z0370	+3.72	8.51	+1.89	7.56	0.2	9.46	0.2	0.00013958	0.00153034	0.01790206	0.35275344	A_07_P043596	Z0370	cmr|orf Unknown function [Z0370]
Z0374	+2.39	7.56	+1.26	6.93	0.0	8.19	0.2	0.00084085	0.00573833	0.04285442	0.51268997	A_07_P043615	Z0374	cmr|orf Unknown function [Z0374]
Z0377	+2.03	7.83	+1.02	7.32	0.1	8.34	0.2	0.00243818	0.01287715	0.06310150	0.60949080	A_07_P043626	Z0377	cmr|putative dehydrogenase [Z0377]
Z0393	-2.45	8.42	-1.29	9.07	0.4	7.77	0.1	0.00379644	0.01820071	0.03803735	0.52809524	A_07_P043660	Z0393	cmr|orf Unknown function [Z0393]
Z0397	-2.14	10.08	-1.10	10.63	0.2	9.53	0.3	0.00741341	0.03101693	0.05947599	0.62676707	A_07_P043675	Z0397	cmr|orf Unknown function [Z0397]
Z0476	-3.10	12.25	-1.63	13.07	0.3	11.43	0.2	0.00063848	0.00462381	0.01781631	0.38636364	A_07_P048504	Z0476	cmr|orf Unknown function [Z0476]
Z0491	-2.19	10.47	-1.13	11.03	0.3	9.91	0.0	0.00267438	0.01381863	0.05665206	0.61512414	A_07_P043817	Z0491	cmr|orf Unknown function [Z0491]
Z0666	+2.58	7.50	+1.36	6.81	0.0	8.18	0.2	0.00057658	0.00430688	0.03668318	0.48451342	A_07_P043951	Z0666	cmr|orf Unknown function [Z0666]
Z0949	+11.72	10.84	+3.55	9.07	0.3	12.62	0.2	0.00000645	0.00019352	0.00177591	0.12538117	A_07_P046386	Z0949	cmr|unknown protein encoded by prophage CP-933K [Z0949]
Z0950	+25.48	9.98	+4.67	7.65	0.1	12.32	0.2	0.00000026	0.00004211	0.00022802	0.04174419	A_07_P046393	Z0950	cmr|unknown protein encoded by prophage CP-933K [Z0950]
Z0951	+4.97	13.10	+2.31	11.94	0.1	14.26	0.3	0.00007774	0.00099992	0.01028138	0.28005190	A_07_P046396	Z0951	cmr|putative exonuclease encoded by prophage CP-933K [Z0951]
Z0952	+3.94	12.52	+1.98	11.53	0.1	13.51	0.3	0.00023106	0.00222327	0.01566184	0.33778082	A_07_P046403	Z0952	cmr|putative Bet recombination protein of prophage CP-933K [Z0952]
Z0960	+26.37	10.09	+4.72	7.73	0.1	12.45	0.1	0.00000016	0.00003548	0.00020198	0.03975000	A_07_P046435	Z0960	cmr|putative lysozyme protein R of prophage CP-933K [Z0960]
Z0964	-2.26	8.67	-1.18	9.26	0.0	8.08	0.1	0.00068334	0.00484533	0.05032203	0.58643227	A_07_P046446	Z0964	cmr|putative DNA packaging protein of prophage CP-933K [Z0964]
Z0965	-2.52	9.76	-1.33	10.43	0.1	9.09	0.4	0.00239166	0.01268674	0.03618077	0.51784545	A_07_P046451	Z0965	cmr|unknown protein encoded by prophage CP-933K [Z0965]
Z0966	-2.48	8.26	-1.31	8.92	0.1	7.61	0.0	0.00032291	0.00281814	0.03723831	0.52534050	A_07_P046460	Z0966	cmr|unknown protein encoded by prophage CP-933K [Z0966]
Z0966	-2.72	8.23	-1.44	8.96	0.1	7.51	0.0	0.00024425	0.00230872	0.02768674	0.47328990	A_07_P046460	Z0966	cmr|unknown protein encoded by prophage CP-933K [Z0966]
Z0966	-3.49	8.08	-1.80	8.98	0.0	7.18	0.1	0.00005420	0.00078198	0.01151232	0.33256881	A_07_P046460	Z0966	cmr|unknown protein encoded by prophage CP-933K [Z0966]
Z0966	-2.63	8.07	-1.40	8.77	0.0	7.37	0.1	0.00022337	0.00216550	0.03098577	0.49426545	A_07_P046460	Z0966	cmr|unknown protein encoded by prophage CP-933K [Z0966]
Z0966	-3.13	8.24	-1.65	9.06	0.1	7.42	0.1	0.00011717	0.00133575	0.01703125	0.37926450	A_07_P046460	Z0966	cmr|unknown protein encoded by prophage CP-933K [Z0966]
Z0971	+2.34	7.36	+1.23	6.75	0.0	7.97	0.1	0.00058390	0.00434242	0.04416286	0.52121439	A_07_P046483	Z0971	cmr|putative tail component of prophage CP-933K [Z0971]
Z0974	-2.57	9.73	-1.36	10.41	0.3	9.05	0.2	0.00187911	0.01050221	0.03228277	0.50322772	A_07_P046497	Z0974	cmr|putative tail component of prophage CP-933K [Z0974]
Z0977	+3.45	9.24	+1.79	8.35	0.3	10.13	0.2	0.00060546	0.00445856	0.02083778	0.38015064	A_07_P046512	Z0977	cmr|putative tail component of prophage CP-933K [Z0977]
Z0979	+2.61	8.48	+1.38	7.79	0.0	9.17	0.0	0.00020900	0.00206171	0.03542619	0.47670940	A_07_P046522	Z0979	cmr|putative tail component of prophage CP-933K [Z0979]
Z1046	+2.91	7.51	+1.54	6.74	0.0	8.28	0.1	0.00012056	0.00136756	0.02843559	0.43891176	A_07_P046587	Z1046	cmr|orf Unknown function [Z1046]
Z1058	-2.04	7.30	-1.03	7.81	0.0	6.78	0.1	0.00122849	0.00757592	0.06970147	0.65869148	A_07_P052018	Z1058	cmr|orf, hypothetical protein [Z1058]
Z1098	-2.42	11.40	-1.27	12.03	0.1	10.76	0.2	0.00141911	0.00841525	0.04066057	0.54389125	A_07_P046651	Z1098	cmr|orf Unknown function [Z1098]
Z1099	+2.30	9.02	+1.20	8.42	0.1	9.62	0.2	0.00091737	0.00611993	0.04670986	0.53405955	A_07_P046659	Z1099	cmr|orf, hypothetical protein [Z1099]
Z1102	+3.05	7.78	+1.61	6.98	0.1	8.59	0.3	0.00058025	0.00432758	0.02638529	0.42649897	A_07_P046662	Z1102	cmr|putative nucleotide di-P-sugar epimerase or dehydratase [Z1102]
Z1121	+5.26	9.06	+2.39	7.86	0.3	10.26	0.1	0.00003832	0.00060814	0.00923844	0.26835793	A_07_P046679	Z1121	cmr|orf Unknown function [Z1121]
Z1124	+2.62	7.10	+1.39	6.40	0.1	7.79	0.2	0.00047277	0.00375737	0.03538427	0.47736932	A_07_P046689	Z1124	cmr|putative prophage regulatory protein [Z1124]
Z1125	-2.48	10.90	-1.31	11.55	0.5	10.24	0.1	0.00601181	0.02640168	0.03705983	0.52517552	A_07_P046692	Z1125	cmr|orf Unknown function [Z1125]
Z1129	-3.05	13.36	-1.61	14.17	0.3	12.56	0.5	0.00437015	0.02045695	0.01898692	0.39593377	A_07_P046708	Z1129	cmr|putative helicase [Z1129]
Z1131	-2.22	9.21	-1.15	9.78	0.0	8.63	0.1	0.00066527	0.00476526	0.05397294	0.60523504	A_07_P046719	Z1131	cmr|unknown protein encoded in ISEc8 [Z1131]
Z1133	+2.04	9.29	+1.03	8.77	0.1	9.80	0.1	0.00189874	0.01057812	0.06141959	0.60361423	A_07_P046726	Z1133	cmr|partial putative transposase [Z1133]
Z1134	-2.09	8.02	-1.06	8.55	0.0	7.49	0.0	0.00091321	0.00611033	0.06510544	0.64874684	A_07_P046735	Z1134	cmr|unknown in IS600 [Z1134]
Z1139	-4.04	9.46	-2.02	10.47	0.9	8.45	0.1	0.00813311	0.03338923	0.00706110	0.26852657	A_07_P046753	Z1139	cmr|putative diacylglycerol kinase [Z1139]
Z1150	-2.08	8.44	-1.06	8.97	0.2	7.91	0.0	0.00165874	0.00953107	0.06633384	0.65150343	A_07_P046801	Z1150	cmr|unknown in IS [Z1150]
Z1160	-3.49	9.65	-1.80	10.55	0.2	8.75	0.1	0.00013645	0.00151031	0.01132304	0.33197393	A_07_P046840	Z1160	cmr|unknown in ISEc8 [Z1160]
Z1161	-2.54	8.84	-1.35	9.51	0.0	8.16	0.0	0.00025453	0.00237968	0.03468559	0.51324248	A_07_P046841	Z1161	cmr|unknown in ISEc8 [Z1161]
Z1199	-3.78	9.33	-1.92	10.29	0.1	8.37	0.3	0.00019638	0.00196842	0.00889037	0.30165948	A_07_P050887	Z1199	cmr|unknown in IS [Z1199]
Z1203	-2.21	10.65	-1.15	11.23	0.2	10.08	0.1	0.00148797	0.00872499	0.05341082	0.60107219	A_07_P050909	Z1203	cmr|unknown [Z1203]
Z1210	-2.49	7.66	-1.32	8.32	0.1	7.00	0.3	0.00126510	0.00773824	0.03610899	0.51775956	A_07_P050942	Z1210	cmr|putative histone [Z1210]
Z1212	-5.69	11.99	-2.51	13.24	0.0	10.73	0.1	0.00000701	0.00020362	0.00196900	0.14622642	A_07_P050946	Z1212	cmr|unknown [Z1212]
Z1213	-3.42	9.78	-1.78	10.67	0.0	8.89	0.0	0.00004845	0.00072232	0.01232406	0.33685764	A_07_P050951	Z1213	cmr|unknown [Z1213]
Z1217	-2.24	8.30	-1.17	8.88	0.0	7.71	0.2	0.00145542	0.00856925	0.05181657	0.59158811	A_07_P050973	Z1217	cmr|putative DNA repair protein, RADC family [Z1217]
Z1335	-2.10	10.95	-1.07	11.49	0.1	10.42	0.3	0.00689714	0.02934094	0.06489837	0.64832487	A_07_P051076	Z1335	cmr|unknown protein encoded by cryptic prophage CP-933M [Z1335]
Z1340	-4.67	9.47	-2.22	10.58	0.4	8.36	0.3	0.00044682	0.00360203	0.00415968	0.21194175	A_07_P051102	Z1340	cmr|unknown protein encoded by cryptic prophage CP-933M [Z1340]
Z1352	-3.57	9.17	-1.84	10.09	0.4	8.26	0.5	0.00333777	0.01642830	0.01036585	0.32316832	A_07_P051141	Z1352	cmr|putative endolysin of cryptic prophage CP-933M [Z1352]
Z1364	-5.80	10.70	-2.53	11.97	1.1	9.44	0.1	0.00560414	0.02495386	0.00158155	0.12835052	A_07_P051194	Z1364	cmr|unknown protein encoded by cryptic prophage CP-933M [Z1364]
Z1373	-2.94	10.87	-1.56	11.65	0.1	10.09	0.1	0.00016687	0.00175732	0.02135671	0.41977528	A_07_P051220	Z1373	cmr|unknown protein encoded by cryptic prophage CP-933M [Z1373]
Z1428	+20.30	9.42	+4.34	7.24	0.1	11.59	0.2	0.00000065	0.00005973	0.00047574	0.06139344	A_07_P048529	Z1428	cmr|unknown protein encoded by bacteriophage BP-933W [Z1428]
Z1433	+35.48	11.26	+5.15	8.69	0.2	13.84	0.3	0.00000045	0.00005132	0.00008956	0.02611111	A_07_P051325	Z1433	cmr|unknown protein encoded by bacteriophage BP-933W [Z1433]
Z1434	+24.53	9.81	+4.62	7.50	0.3	12.12	0.0	0.00000051	0.00005264	0.00027121	0.04641304	A_07_P051326	Z1434	cmr|unknown protein encoded by bacteriophage BP-933W [Z1434]
Z1444	-3.52	12.57	-1.82	13.48	0.1	11.66	0.4	0.00054214	0.00411349	0.01138338	0.33250464	A_07_P051356	Z1444	cmr|putative serine/threonine kinase encoded by bacteriophage BP-933W [Z1444]
Z1449	+4.13	10.79	+2.05	9.77	0.2	11.81	0.2	0.00007617	0.00098704	0.01456110	0.32610242	A_07_P051385	Z1449	cmr|putative regulatory protein CII of bacteriophage BP-933W [Z1449]
Z1454	+39.67	9.97	+5.31	7.31	0.3	12.62	0.2	0.00000059	0.00005706	0.00006034	0.02209302	A_07_P051405	Z1454	cmr|putative DNA N-6-adenine-methyltransferase of bacteriophage BP-933W [Z1454]
Z1457	-3.53	10.85	-1.82	11.76	0.4	9.94	0.3	0.00111897	0.00707230	0.01084096	0.32448669	A_07_P051411	Z1457	cmr|putative DNA-binding protein Roi of bacteriophage BP-933W [Z1457]
Z1473	+13.94	9.09	+3.80	7.19	0.3	10.99	0.1	0.00000466	0.00016486	0.00127096	0.10934426	A_07_P051457	Z1473	cmr|putative endopeptidase Rz of bacteriophage BP-933W [Z1473]
Z1534	-2.35	8.73	-1.23	9.34	0.2	8.11	0.1	0.00142419	0.00843485	0.04426829	0.56206452	A_07_P051615	Z1534	cmr|putative chaperone [Z1534]
Z1543	-2.08	8.62	-1.05	9.15	0.3	8.10	0.1	0.00429379	0.02017349	0.06631542	0.65172909	A_07_P051649	Z1543	cmr|putative outer membrane transporter of ShlA/HecA/FhaA exoprotein [Z1543]
Z1660	-3.15	8.46	-1.66	9.29	0.0	7.63	0.0	0.00007269	0.00095677	0.01658410	0.37731214	A_07_P050081	Z1660	cmr|transposase for IS629 [Z1660]
Z1775	-2.60	11.93	-1.38	12.61	0.0	11.24	0.2	0.00054978	0.00416141	0.03162919	0.49747253	A_07_P050153	Z1775	cmr|unknown protein encoded by prophage CP-933N [Z1775]
Z1778	-2.29	11.75	-1.20	12.35	0.1	11.15	0.2	0.00117335	0.00730198	0.04874301	0.58225341	A_07_P050169	Z1778	cmr|unknown protein encoded by prophage CP-933N [Z1778]
Z1781	+10.85	9.65	+3.44	7.93	0.2	11.37	0.2	0.00000423	0.00015425	0.00214812	0.13528000	A_07_P050182	Z1781	cmr|unknown protein encoded by prophage CP-933N [Z1781]
Z1794	-2.66	8.83	-1.41	9.53	0.1	8.12	0.3	0.00101919	0.00661694	0.02891133	0.48014768	A_07_P050226	Z1794	cmr|putative holin protein [Z1794]
Z1805	-2.65	11.46	-1.41	12.16	0.4	10.75	0.1	0.00187402	0.01048120	0.02909489	0.47965445	A_07_P050276	Z1805	cmr|unknown protein encoded by prophage CP-933N [Z1805]
Z1809	+2.32	7.15	+1.21	6.55	0.1	7.76	0.4	0.00607056	0.02662578	0.04717924	0.53669798	A_07_P050293	Z1809	cmr|unknown protein encoded by prophage CP-933N [Z1809]
Z1843	-2.79	8.17	-1.48	8.91	0.5	7.43	0.1	0.00309795	0.01552327	0.02491679	0.45562137	A_07_P050409	Z1843	cmr|unknown protein encoded by prophage CP-933C [Z1843]
Z1852	-3.74	10.70	-1.90	11.65	0.3	9.75	0.2	0.00020148	0.00200768	0.00910760	0.30638889	A_07_P050455	Z1852	cmr|putative holin protein of prophage CP-933C [Z1852]
Z1856	-2.92	7.81	-1.54	8.59	0.0	7.04	0.2	0.00035741	0.00303839	0.02135607	0.42028750	A_07_P050473	Z1856	cmr|unknown protein encoded by prophage CP-933C [Z1856]
Z1866	+12.44	8.79	+3.64	6.97	0.1	10.61	0.1	0.00000116	0.00007956	0.00163173	0.12061033	A_07_P050491	Z1866	cmr|putative integrase of prophage CP-933X [Z1866]
Z1867	+47.19	10.35	+5.56	7.57	0.1	13.13	0.0	0.00000004	0.00003344	0.00002604	0.01205882	A_07_P050497	Z1867	cmr|putative integrase of prophage CP-933X [Z1867]
Z1869	+3.61	12.06	+1.85	11.13	0.1	12.99	0.1	0.00007270	0.00095677	0.01935722	0.36629808	A_07_P052425	Z1869	cmr|putative replication protein P of prophage CP-933X [Z1869]
Z1871	-2.73	8.60	-1.45	9.32	0.1	7.88	0.0	0.00019636	0.00196842	0.02799479	0.47545847	A_07_P050509	Z1871	cmr|unknown protein encoded by prophage CP-933X [Z1871]
Z1871	-2.31	8.40	-1.21	9.00	0.1	7.79	0.1	0.00090122	0.00605586	0.04776105	0.57797848	A_07_P050509	Z1871	cmr|unknown protein encoded by prophage CP-933X [Z1871]
Z1891	+16.95	10.30	+4.08	8.26	0.1	12.34	0.2	0.00000077	0.00006301	0.00078061	0.08304054	A_07_P050583	Z1891	cmr|putative tail component of prophage CP-933X [Z1891]
Z1893	+3.90	10.91	+1.96	9.92	0.1	11.89	0.1	0.00004204	0.00064895	0.01645897	0.34367374	A_07_P050588	Z1893	cmr|putative tail component of prophage CP-933X [Z1893]
Z1894	+16.18	9.41	+4.02	7.40	0.1	11.41	0.0	0.00000037	0.00004777	0.00087335	0.08928571	A_07_P050594	Z1894	cmr|putative tail component of prophage CP-933X [Z1894]
Z1895	+20.54	9.17	+4.36	6.99	0.3	11.35	0.2	0.00000226	0.00010913	0.00046684	0.06176471	A_07_P050599	Z1895	cmr|putative tail component of prophage CP-933X [Z1895]
Z1896	+3.97	8.11	+1.99	7.12	0.3	9.10	0.2	0.00026105	0.00242251	0.01631225	0.34334225	A_07_P050602	Z1896	cmr|putative tail component of prophage CP-933X [Z1896]
Z1898	+4.82	10.25	+2.27	9.12	0.4	11.39	0.2	0.00028457	0.00258977	0.01066692	0.28368243	A_07_P050610	Z1898	cmr|putative tail component of prophage CP-933X [Z1898]
Z1914	-2.01	9.75	-1.00	10.25	0.1	9.25	0.1	0.00225802	0.01213735	0.07307800	0.66969732	A_07_P050652	Z1914	cmr|putative minor tail fiber protein of prophage [Z1914]
Z1915	+2.84	8.53	+1.51	7.77	0.0	9.28	0.1	0.00016835	0.00176700	0.02975927	0.44326395	A_07_P050656	Z1915	cmr|putative tail protein [Z1915]
Z1919	-2.72	7.66	-1.44	8.38	0.4	6.94	0.3	0.00320068	0.01591143	0.02720655	0.47226020	A_07_P050674	Z1919	cmr|unknown protein encoded by prophage CP-933X [Z1919]
Z1922	-2.57	8.49	-1.36	9.18	0.3	7.81	0.0	0.00139538	0.00831210	0.03271659	0.50548577	A_07_P050689	Z1922	cmr|unknown protein encoded by prophage CP-933X [Z1922]
Z1957	-6.68	10.07	-2.74	11.44	0.1	8.70	0.2	0.00000710	0.00020499	0.00111026	0.10723926	A_07_P050726	Z1957	cmr|transposase for IS629 [Z1957]
Z1972	-2.33	8.73	-1.22	9.34	0.1	8.12	0.2	0.00122198	0.00754466	0.04639672	0.57381775	A_07_P050771	Z1972	cmr|partial putative adhesion protein [Z1972]
Z2005	-4.49	12.15	-2.17	13.24	0.2	11.07	0.3	0.00017801	0.00184143	0.00491806	0.22840708	A_07_P050791	Z2005	cmr|orf Unknown function [Z2005]
Z2060	-3.04	10.80	-1.60	11.60	0.5	9.99	0.2	0.00296154	0.01503109	0.01820122	0.38829268	A_07_P047656	Z2060	cmr|putative DNA adenine methyltransferase encoded by prophage [Z2060]
Z2074	-3.89	11.00	-1.96	11.98	0.1	10.02	0.2	0.00008150	0.00103391	0.00772929	0.28168981	A_07_P047687	Z2074	cmr|putative IS encoded protein within CP-933O [Z2074]
Z2076	-2.10	11.18	-1.07	11.71	0.1	10.64	0.3	0.00368217	0.01778010	0.06415269	0.64620601	A_07_P047700	Z2076	cmr|unknown protein encoded by prophage CP-933O [Z2076]
Z2080	-9.12	11.41	-3.19	13.00	0.1	9.81	0.2	0.00000519	0.00017426	0.00029662	0.05430233	A_07_P047717	Z2080	cmr|putative IS encoded protein within CP-933O [Z2080]
Z2099	-3.23	8.39	-1.69	9.24	0.0	7.55	0.3	0.00028706	0.00260187	0.01555450	0.37330793	A_07_P047791	Z2099	cmr|unknown protein encoded within prophage CP-933O [Z2099]
Z2115	-2.44	11.51	-1.29	12.15	0.3	10.87	0.1	0.00215570	0.01172343	0.04026105	0.54269692	A_07_P047851	Z2115	cmr|unknown protein encoded within prophage CP-933O [Z2115]
Z2118	+2.06	7.26	+1.04	6.74	0.1	7.79	0.1	0.00197135	0.01093234	0.05882813	0.59333120	A_07_P047862	Z2118	cmr|putative endopeptidase Rz of prophage CP-933O [Z2118]
Z2119	-2.43	9.05	-1.28	9.69	0.0	8.41	0.0	0.00033681	0.00290718	0.04045541	0.54345563	A_07_P047868	Z2119	cmr|unknown protein encoded within prophage CP-933O [Z2119]
Z2124	-2.57	10.53	-1.36	11.21	0.5	9.85	0.1	0.00776446	0.03221176	0.03263021	0.50663708	A_07_P047894	Z2124	cmr|unknown protein encoded within prophage CP-933O [Z2124]
Z2131	+2.45	9.11	+1.29	8.46	0.2	9.75	0.1	0.00092569	0.00616237	0.04041794	0.49948195	A_07_P047904	Z2131	cmr|putative terminase large subunit of prophage CP-933O [Z2131]
Z2133	+4.44	8.09	+2.15	7.01	0.1	9.17	0.4	0.00023159	0.00222327	0.01255145	0.30215596	A_07_P047906	Z2133	cmr|putative capsid assembly protein of prophage CP-933O [Z2133]
Z2136	+13.62	9.16	+3.77	7.28	0.4	11.04	0.0	0.00001189	0.00028316	0.00130081	0.11130435	A_07_P047913	Z2136	cmr|partial putative major capsid protein of prophage [Z2136]
Z2139	-2.06	9.68	-1.05	10.20	0.3	9.16	0.2	0.00582589	0.02575761	0.06791286	0.65515931	A_07_P047923	Z2139	cmr|putative tail component of prophage CP-933O [Z2139]
Z2140	+3.19	7.80	+1.67	6.97	0.0	8.64	0.1	0.00007243	0.00095580	0.02384464	0.41118291	A_07_P047929	Z2140	cmr|putative tail component of prophage CP-933O [Z2140]
Z2142	-2.79	11.80	-1.48	12.54	0.2	11.06	0.4	0.00226452	0.01215567	0.02577172	0.46212984	A_07_P047939	Z2142	cmr|putative tail component of prophage CP-933O [Z2142]
Z2144	-3.21	10.63	-1.68	11.48	0.3	9.79	0.1	0.00048530	0.00382411	0.01530742	0.37191358	A_07_P047949	Z2144	cmr|putative tail component of prophage CP-933O [Z2144]
Z2181	-2.54	7.78	-1.35	8.45	0.2	7.11	0.2	0.00127948	0.00781083	0.03364647	0.50740421	A_07_P048026	Z2181	cmr|orf Unknown function [Z2181]
Z2249	-3.19	10.49	-1.67	11.33	0.2	9.66	0.1	0.00022172	0.00215607	0.01623476	0.37754801	A_07_P048081	Z2249	cmr|orf Unknown function [Z2249]
Z2254	-2.56	7.99	-1.36	8.67	0.4	7.31	0.1	0.00268946	0.01388288	0.03353659	0.50671785	A_07_P048086	Z2254	cmr|partial H repeat-associated protein of Rhs element [Z2254]
Z2297	-2.07	10.98	-1.05	11.51	0.1	10.46	0.0	0.00148048	0.00869077	0.06710048	0.65091189	A_07_P048153	Z2297	cmr|orf Unknown function [Z2297]
Z2298	-3.93	8.89	-1.97	9.88	0.1	7.90	0.2	0.00006492	0.00088731	0.00765943	0.28044186	A_07_P048160	Z2298	cmr|orf Unknown function [Z2298]
Z2309	-2.20	10.89	-1.14	11.46	0.2	10.32	0.4	0.00825783	0.03380517	0.05506923	0.60842807	A_07_P048162	Z2309	cmr|orf Unknown function [Z2309]
Z2353	-3.59	8.36	-1.84	9.29	0.8	7.44	0.2	0.01109835	0.04268843	0.01057292	0.32322330	A_07_P048271	Z2353	cmr|putative tail component of prophage CP-933R [Z2353]
Z2360	+27.39	10.08	+4.78	7.69	0.5	12.47	0.2	0.00000472	0.00016577	0.00019182	0.03822785	A_07_P048290	Z2360	cmr|putative capsid protein of prophage CP-933R [Z2360]
Z2361	+28.32	10.79	+4.82	8.37	0.0	13.20	0.1	0.00000010	0.00003427	0.00017403	0.03702703	A_07_P048291	Z2361	cmr|putative capsid assembly protein of prophage CP-933R [Z2361]
Z2391	+17.77	9.00	+4.15	6.93	0.2	11.08	0.6	0.00003410	0.00056193	0.00065866	0.07569343	A_07_P048371	Z2391	cmr|unknown protein encoded within prophage CP-933R [Z2391]
Z2446	+2.58	8.83	+1.37	8.15	0.0	9.51	0.1	0.00024067	0.00228123	0.03613821	0.47973019	A_07_P048452	Z2446	cmr|orf Unknown function [Z2446]
Z2503	-2.43	10.33	-1.28	10.97	0.3	9.69	0.1	0.00160984	0.00932155	0.03961255	0.53996537	A_07_P044070	Z2503	cmr|partial putative membrane transport protein [Z2503]
Z2511	-2.09	7.74	-1.06	8.27	0.3	7.21	0.3	0.00815930	0.03348801	0.06528836	0.64892677	A_07_P044103	Z2511	cmr|orf Unknown function [Z2511]
Z2519	+2.25	8.14	+1.17	7.55	0.3	8.73	0.3	0.00615058	0.02688400	0.05129573	0.56005548	A_07_P044110	Z2519	cmr|orf Unknown function [Z2519]
Z2519	+2.22	8.41	+1.15	7.84	0.2	8.98	0.1	0.00117215	0.00730198	0.05079586	0.55808095	A_07_P044110	Z2519	cmr|orf Unknown function [Z2519]
Z2519	+2.05	8.39	+1.04	7.87	0.1	8.90	0.1	0.00190660	0.01061063	0.05953887	0.59591863	A_07_P044110	Z2519	cmr|orf Unknown function [Z2519]
Z2519	+2.31	8.35	+1.21	7.74	0.2	8.95	0.1	0.00109823	0.00698325	0.04637259	0.53213557	A_07_P044110	Z2519	cmr|orf Unknown function [Z2519]
Z2519	+2.33	8.30	+1.22	7.69	0.1	8.91	0.1	0.00083789	0.00572307	0.04572282	0.52930882	A_07_P044110	Z2519	cmr|orf Unknown function [Z2519]
Z2546	+2.43	9.65	+1.28	9.01	0.0	10.29	0.0	0.00032875	0.00285488	0.04126588	0.50402638	A_07_P044121	Z2546	cmr|anthranilate synthase component I [Z2546]
Z2547	+17.15	9.54	+4.10	7.49	0.2	11.59	0.4	0.00000547	0.00017880	0.00075584	0.08321678	A_07_P044129	Z2547	cmr|anthranilate synthase component I [Z2547]
Z2560	-2.09	9.08	-1.06	9.61	0.0	8.55	0.4	0.01031411	0.04026417	0.06571773	0.65113908	A_07_P044137	Z2560	cmr|orf Unknown function [Z2560]
Z2561	-4.07	9.07	-2.03	10.09	0.1	8.06	0.1	0.00003927	0.00062005	0.00665523	0.26326633	A_07_P044143	Z2561	cmr|putative transposase [Z2561]
Z2664	+2.43	9.30	+1.28	8.66	0.0	9.94	0.3	0.00135141	0.00813014	0.04171494	0.50793503	A_07_P044420	Z2664	cmr|orf Unknown function [Z2664]
Z2754	+7.14	9.54	+2.84	8.12	0.4	10.96	0.1	0.00005274	0.00076604	0.00504573	0.20850394	A_07_P044434	Z2754	cmr|orf, hypothetical protein [Z2754]
Z2790	-3.83	9.50	-1.94	10.47	0.5	8.53	0.2	0.00139054	0.00828952	0.00865218	0.30271111	A_07_P044445	Z2790	cmr|putative cytochrome oxidase [Z2790]
Z2804	-3.22	9.12	-1.69	9.96	0.1	8.28	0.1	0.00009289	0.00113544	0.01551956	0.37360856	A_07_P044458	Z2804	cmr|unknown protein encoded within IS629 [Z2804]
Z2806	-3.37	8.61	-1.75	9.48	0.2	7.73	0.2	0.00029463	0.00264925	0.01278074	0.34279387	A_07_P044461	Z2806	cmr|putative transposase [Z2806]
Z2851	+2.04	8.42	+1.03	7.90	0.2	8.93	0.1	0.00248798	0.01308308	0.06161077	0.60473815	A_07_P052567	Z2851	cmr|putative enzyme [Z2851]
Z2994	-2.66	9.09	-1.41	9.80	0.4	8.38	0.0	0.00263087	0.01367914	0.02909172	0.48060860	A_07_P044649	Z2994	cmr|unknown protein encoded by prophage CP-933T [Z2994]
Z3082	-2.18	9.70	-1.12	10.26	0.0	9.14	0.4	0.00698842	0.02964853	0.05803925	0.61950508	A_07_P044746	Z3082	cmr|putative tail fiber component L of prophage [Z3082]
Z3087	-3.89	11.71	-1.96	12.69	0.6	10.73	0.6	0.00790346	0.03262510	0.00728404	0.27304762	A_07_P044772	Z3087	cmr|putative tail fiber component V of prophage [Z3087]
Z3089	-2.89	9.09	-1.53	9.85	0.4	8.32	0.1	0.00228276	0.01223691	0.02173399	0.42349010	A_07_P044781	Z3089	cmr|putative tail fiber component Z of prophage [Z3089]
Z3099	-2.86	11.46	-1.51	12.21	0.0	10.70	0.3	0.00064962	0.00468081	0.02281441	0.43750305	A_07_P044814	Z3099	cmr|putative DNA packaging protein of prophage CP-933U [Z3099]
Z3108	-2.81	8.21	-1.49	8.95	0.0	7.46	0.3	0.00083319	0.00569594	0.02522231	0.45907514	A_07_P044835	Z3108	cmr|unknown protein encoded within prophage CP-933U [Z3108]
Z3144	-4.46	8.94	-2.16	10.02	0.0	7.86	0.3	0.00008743	0.00108817	0.00498476	0.22880466	A_07_P044923	Z3144	cmr|orf Unknown function [Z3144]
Z3159	-6.98	8.79	-2.80	10.19	0.1	7.39	0.1	0.00000671	0.00019817	0.00100229	0.10115385	A_07_P044945	Z3159	cmr|putative outer membrane receptor for iron compound [Z3159]
Z3162	-4.57	9.30	-2.19	10.39	0.1	8.20	0.1	0.00001966	0.00038882	0.00423336	0.21293930	A_07_P044951	Z3162	cmr|IS629 transposase [Z3162]
Z3249	+2.29	7.98	+1.20	7.39	0.1	8.58	0.1	0.00077665	0.00537131	0.04688643	0.53530094	A_07_P045124	Z3249	cmr|orf, hypothetical protein [Z3249]
Z3297	-5.48	9.79	-2.46	11.02	0.0	8.57	0.2	0.00002006	0.00039347	0.00223958	0.15741071	A_07_P045211	Z3297	cmr|putative transposase for IS629 [Z3297]
Z3316	-3.84	13.95	-1.94	14.92	0.1	12.98	0.3	0.00014981	0.00161942	0.00846481	0.29948315	A_07_P045251	Z3316	cmr|unknown protein encoded within prophage CP-933V [Z3316]
Z3320	-3.69	10.26	-1.88	11.20	0.2	9.32	0.2	0.00012576	0.00141526	0.00961700	0.31412863	A_07_P045261	Z3320	cmr|unknown protein encoded within prophage CP-933V [Z3320]
Z3349	+21.97	8.85	+4.46	6.62	0.1	11.08	0.2	0.00000063	0.00005869	0.00036331	0.05500000	A_07_P045345	Z3349	cmr|putative DNA methyltransferase encoded within prophage CP-933V [Z3349]
Z3354	+6.57	12.97	+2.72	11.62	0.0	14.33	0.1	0.00000543	0.00017834	0.00578760	0.22009662	A_07_P045360	Z3354	cmr|putative exclusion protein ren of prophage CP-933V [Z3354]
Z3355	+5.12	14.46	+2.36	13.28	0.0	15.64	0.2	0.00001750	0.00036012	0.00969639	0.27163701	A_07_P045362	Z3355	cmr|putative DNA replication protein P of prophage [Z3355]
Z3356	+7.38	11.50	+2.88	10.06	0.1	12.94	0.1	0.00000387	0.00014511	0.00445376	0.19054348	A_07_P045369	Z3356	cmr|putative DNA replication protein O of prophage [Z3356]
Z3357	+3.81	9.57	+1.93	8.60	0.1	10.53	0.1	0.00003884	0.00061515	0.01746634	0.34941550	A_07_P045373	Z3357	cmr|putative regulatory protein CII of prophage CP-933V [Z3357]
Z3364	+2.68	13.74	+1.42	13.02	0.0	14.45	0.0	0.00018134	0.00186421	0.03348704	0.46780834	A_07_P045400	Z3364	cmr|putative host killing protein Kil of prophage [Z3364]
Z3365	+12.20	11.00	+3.61	9.20	0.1	12.81	0.2	0.00000146	0.00009005	0.00163427	0.12023364	A_07_P045401	Z3365	cmr|putative host-nuclease inhibitor protein Gam of prophage [Z3365]
Z3366	+3.14	14.61	+1.65	13.79	0.0	15.44	0.3	0.00049524	0.00387531	0.02484820	0.41795940	A_07_P045406	Z3366	cmr|putative recombination protein Bet of prophage CP-933V [Z3366]
Z3367	+2.85	11.65	+1.51	10.90	0.1	12.41	0.1	0.00022205	0.00215796	0.02958079	0.44354286	A_07_P045415	Z3367	cmr|putative exonuclease of prophage CP-933V [Z3367]
Z3392	-4.13	10.15	-2.05	11.17	0.3	9.12	0.1	0.00009420	0.00114522	0.00639545	0.25751918	A_07_P045464	Z3392	cmr|putative isomerase-decarboxylase [Z3392]
Z3600	-2.99	7.98	-1.58	8.77	0.3	7.19	0.1	0.00075475	0.00524629	0.01957381	0.40126302	A_07_P042666	Z3600	cmr|putative fimbrial usher [Z3600]
Z3603	-2.70	8.94	-1.44	9.66	0.3	8.22	0.1	0.00070798	0.00497166	0.02820312	0.47540685	A_07_P042677	Z3603	cmr|orf, hypothetical protein [Z3603]
Z3614	-2.51	7.69	-1.33	8.36	0.3	7.03	0.0	0.00112380	0.00709143	0.03641832	0.51982774	A_07_P042696	Z3614	cmr|putative prophage DNA injection protein [Z3614]
Z3657	-5.28	10.21	-2.40	11.41	0.1	9.01	0.1	0.00001383	0.00030876	0.00252985	0.16877119	A_07_P042781	Z3657	cmr|orf, hypothetical protein [Z3657]
Z3673	-3.85	11.39	-1.95	12.36	0.0	10.41	0.4	0.00040640	0.00336751	0.00850864	0.29968680	A_07_P042804	Z3673	cmr|putative transcriptional regulator LYSR-type [Z3673]
Z3783	-3.76	11.66	-1.91	12.61	0.1	10.70	0.0	0.00005211	0.00076029	0.00879256	0.30291028	A_07_P042860	Z3783	cmr|putative dimethyl sulfoxide reductase subunit C [Z3783]
Z3799	-7.81	13.59	-2.96	15.07	0.0	12.11	0.1	0.00000274	0.00012064	0.00060404	0.07795082	A_07_P042878	Z3799	cmr|putative ATP synthase beta subunit [Z3799]
Z3799	-8.40	13.17	-3.07	14.70	0.0	11.63	0.0	0.00000160	0.00009401	0.00046430	0.06768519	A_07_P042878	Z3799	cmr|putative ATP synthase beta subunit [Z3799]
Z3799	-10.11	13.34	-3.34	15.01	0.0	11.67	0.3	0.00000565	0.00018039	0.00019690	0.04492754	A_07_P042878	Z3799	cmr|putative ATP synthase beta subunit [Z3799]
Z3799	-7.48	13.86	-2.90	15.31	0.1	12.41	0.1	0.00000523	0.00017498	0.00071202	0.08242647	A_07_P042878	Z3799	cmr|putative ATP synthase beta subunit [Z3799]
Z3799	-9.15	13.36	-3.19	14.96	0.1	11.77	0.3	0.00000825	0.00022576	0.00032965	0.05580645	A_07_P042878	Z3799	cmr|putative ATP synthase beta subunit [Z3799]
Z3866	+3.00	8.01	+1.59	7.21	0.0	8.80	0.1	0.00014351	0.00156146	0.02688770	0.42802831	A_07_P042932	Z3866	cmr|orf Unknown function [Z3866]
Z3923	-4.96	10.23	-2.31	11.39	0.1	9.08	0.1	0.00001827	0.00037077	0.00319677	0.19136882	A_07_P042966	Z3923	cmr|orf Unknown function [Z3923]
Z3924	-9.54	10.53	-3.25	12.16	0.0	8.90	0.1	0.00000127	0.00008289	0.00024898	0.05157895	A_07_P042971	Z3924	cmr|partial putative transposase [Z3924]
Z3925	-6.84	9.14	-2.77	10.53	0.1	7.75	0.4	0.00006135	0.00085173	0.00097180	0.10065789	A_07_P042976	Z3925	cmr|partial putative transposase [Z3925]
Z3934	-2.58	10.31	-1.37	10.99	0.2	9.62	0.1	0.00104579	0.00673690	0.03321519	0.50623427	A_07_P043004	Z3934	cmr|unknown protein encoded by prophage CP-933Y [Z3934]
Z3935	-2.66	10.12	-1.41	10.83	0.6	9.42	0.1	0.00965669	0.03817948	0.02899581	0.48053684	A_07_P043007	Z3935	cmr|unknown protein encoded by prophage CP-933Y [Z3935]
Z3945	-2.34	7.74	-1.23	8.35	0.4	7.13	0.3	0.00821879	0.03367390	0.04534553	0.56840764	A_07_P043049	Z3945	cmr|putative enzyme Integration, recombination [Z3945]
Z3974	+2.59	8.05	+1.37	7.36	0.3	8.74	0.2	0.00215099	0.01170185	0.03740727	0.48712986	A_07_P043090	Z3974	cmr|orf Unknown function [Z3974]
Z4048	-2.82	9.23	-1.50	9.98	0.2	8.48	0.2	0.00054119	0.00411349	0.02443788	0.45052693	A_07_P043149	Z4048	cmr|putative regulator [Z4048]
Z4065	-4.22	9.84	-2.08	10.88	0.4	8.80	0.3	0.00054537	0.00413402	0.00579459	0.25063187	A_07_P043170	Z4065	cmr|orf hypothetical protein [Z4065]
Z4105	+2.21	7.74	+1.15	7.17	0.2	8.32	0.1	0.00209932	0.01146981	0.05168001	0.55997935	A_07_P052741	Z4105	cmr|putative transport protein [Z4105]
Z4127	-2.09	9.50	-1.07	10.03	0.0	8.96	0.1	0.00131328	0.00795547	0.06457825	0.64759236	A_07_P043257	Z4127	cmr|orf, hypothetical protein [Z4127]
Z4237	+3.06	9.14	+1.61	8.34	0.1	9.95	0.2	0.00021577	0.00211521	0.02629954	0.42642636	A_07_P052774	Z4237	cmr|putative oxidoreductase [Z4237]
Z4268	+14.23	8.70	+3.83	6.78	0.3	10.61	0.1	0.00000295	0.00012541	0.00118775	0.10625000	A_07_P045673	Z4268	cmr|orf hypothetical protein [Z4268]
Z4269	+7.04	8.74	+2.82	7.33	0.1	10.15	0.1	0.00000343	0.00013521	0.00509337	0.20774611	A_07_P045678	Z4269	cmr|orf hypothetical protein [Z4269]
Z4270	+3.48	7.50	+1.80	6.60	0.1	8.39	0.0	0.00004625	0.00069547	0.02041095	0.37540888	A_07_P045685	Z4270	cmr|putative ATP-binding protein of ABC transport system [Z4270]
Z4315	-2.82	9.17	-1.49	9.92	0.1	8.43	0.3	0.00122384	0.00755314	0.02495236	0.45521437	A_07_P045721	Z4315	cmr|unknown protein encoded by ISEc8 [Z4315]
Z4324	-2.45	8.64	-1.29	9.29	0.4	7.99	0.1	0.00410929	0.01945178	0.03879383	0.53388986	A_07_P045757	Z4324	cmr|putative transposase [Z4324]
Z4332	-2.85	9.15	-1.51	9.91	0.2	8.40	0.1	0.00038157	0.00321037	0.02340574	0.44078947	A_07_P045787	Z4332	cmr|putative cytotoxin [Z4332]

Z4340	-3.21	11.79	-1.68	12.63	0.0	10.95	0.0	0.00006838	0.00092248	0.01558117	0.37224583	A_07_P045803	Z4340	cmr|unknown protein encoded by ISEc8 [Z4340]
Z4343	+2.58	10.15	+1.37	9.46	0.2	10.83	0.1	0.00052190	0.00401015	0.03670986	0.48405360	A_07_P045808	Z4343	cmr|orf, hypothetical protein [Z4343]
Z4385	-3.34	8.18	-1.74	9.05	0.0	7.31	0.0	0.00005674	0.00080476	0.01362932	0.35004894	A_07_P045844	Z4385	cmr|putative ATP-binding protein of ABC transporter family [Z4385]
Z4400	-3.53	13.02	-1.82	13.93	0.1	12.11	0.4	0.00047714	0.00378609	0.01081428	0.32492366	A_07_P045859	Z4400	cmr|orf Unknown function [Z4400]
Z4400	-3.08	13.64	-1.62	14.45	0.0	12.83	0.0	0.00008055	0.00102432	0.01816756	0.38862772	A_07_P045859	Z4400	cmr|orf Unknown function [Z4400]
Z4400	-4.17	13.17	-2.06	14.20	0.0	12.14	0.3	0.00010444	0.00123820	0.00630907	0.25666667	A_07_P045859	Z4400	cmr|orf Unknown function [Z4400]
Z4400	-6.44	13.86	-2.69	15.21	1.0	12.52	0.1	0.00355656	0.01732505	0.00130907	0.12052632	A_07_P045859	Z4400	cmr|orf Unknown function [Z4400]
Z4400	-2.71	13.60	-1.44	14.32	0.0	12.88	0.1	0.00023894	0.00227025	0.02762703	0.47484716	A_07_P045859	Z4400	cmr|orf Unknown function [Z4400]
Z4487	+3.87	7.66	+1.95	6.68	0.0	8.63	0.1	0.00002864	0.00049816	0.01674162	0.34545216	A_07_P045901	Z4487	cmr|putative phosphotransferase system enzyme subunit [Z4487]
Z4488	+3.72	8.65	+1.89	7.70	0.1	9.60	0.0	0.00005049	0.00074433	0.01813770	0.35341584	A_07_P045910	Z4488	cmr|putative phosphotransferase system enzyme subunit [Z4488]
Z4501	-35.26	11.75	-5.14	14.32	0.0	9.18	0.1	0.00000007	0.00003344	0.00000254	0.00444444	A_07_P045934	Z4501	cmr|orf hypothetical protein [Z4501]
Z4801	+2.66	8.47	+1.41	7.76	0.2	9.18	0.1	0.00034514	0.00296290	0.03429116	0.47068875	A_07_P046078	Z4801	cmr|putative ATP-dependent DNA helicase [Z4801]
Z4803	+2.33	7.75	+1.22	7.14	0.1	8.36	0.1	0.00084463	0.00575914	0.04551639	0.52847345	A_07_P046088	Z4803	cmr|putative ATP-dependent DNA helicase [Z4803]
Z4850	-2.09	8.46	-1.06	8.99	0.0	7.93	0.2	0.00218812	0.01185470	0.06586128	0.65133166	A_07_P046137	Z4850	cmr|putative O-methyltransferase [Z4850]
Z4854	-3.16	10.21	-1.66	11.04	0.0	9.38	0.1	0.00007843	0.00100395	0.01650025	0.37758721	A_07_P046157	Z4854	cmr|putative acyl carrier protein [Z4854]
Z4862	+2.44	7.47	+1.29	6.83	0.0	8.12	0.2	0.00109315	0.00695496	0.04123793	0.50407609	A_07_P046200	Z4862	cmr|orf Unknown function [Z4862]
Z4863	+2.88	7.63	+1.52	6.87	0.1	8.39	0.1	0.00014223	0.00155099	0.02911204	0.44071154	A_07_P046205	Z4863	cmr|putative 3-oxoacyl- [Z4863]
Z4864	+2.11	7.67	+1.07	7.13	0.2	8.20	0.2	0.00453425	0.02107684	0.05999682	0.59670878	A_07_P046210	Z4864	cmr|orf Unknown function [Z4864]
Z4875	+2.40	7.69	+1.26	7.06	0.2	8.32	0.2	0.00142894	0.00845252	0.04309832	0.51326778	A_07_P046232	Z4875	cmr|putative phosphotransferase system enzyme subunit [Z4875]
Z4878	-2.43	8.96	-1.28	9.60	0.1	8.32	0.4	0.00540315	0.02422876	0.04057165	0.54362553	A_07_P049346	Z4878	cmr|putative xylulose kinase [Z4878]
Z4883	-2.09	10.55	-1.07	11.08	0.1	10.01	0.1	0.00118900	0.00738206	0.06471100	0.64851050	A_07_P049368	Z4883	cmr|orf Unknown function [Z4883]
Z4950	-3.69	9.99	-1.88	10.93	0.2	9.04	0.1	0.00011941	0.00135835	0.00952236	0.31298539	A_07_P049440	Z4950	cmr|orf Unknown function [Z4950]
Z4999	-2.89	7.94	-1.53	8.70	0.1	7.17	0.2	0.00040274	0.00334608	0.02223831	0.43171393	A_07_P049484	Z4999	cmr|orf Unknown function [Z4999]
Z5088	+2.40	7.46	+1.27	6.83	0.0	8.09	0.1	0.00052427	0.00401856	0.04216908	0.51070000	A_07_P049618	Z5088	cmr|unknown protein encoded by IS911 within prophage [Z5088]
Z5200	-2.29	7.76	-1.20	8.36	0.1	7.16	0.1	0.00054456	0.00412987	0.04873793	0.58263478	A_07_P049909	Z5200	cmr|orf Unknown function [Z5200]
Z5522	-3.39	11.68	-1.76	12.56	0.1	10.80	0.0	0.00006094	0.00084745	0.01288808	0.34391525	A_07_P047170	Z5522	cmr|putative hippuricase [Z5522]
Z5523	-3.62	11.79	-1.86	12.72	0.1	10.86	0.4	0.00080460	0.00552445	0.01083714	0.32499048	A_07_P047173	Z5523	cmr|putative citrate permease [Z5523]
Z5618	-2.04	7.57	-1.03	8.09	0.1	7.06	0.3	0.00574300	0.02546982	0.06933943	0.65803496	A_07_P047247	Z5618	cmr|D-glucitol-6-phosphate dehydrogenase [Z5618]
Z5852	-32.11	13.85	-5.01	16.35	0.0	11.35	0.1	0.00000010	0.00003427	0.00000381	0.00461538	A_07_P047387	Z5852	cmr|orf Unknown function [Z5852]
Z5886	-2.07	10.59	-1.05	11.11	0.0	10.06	0.3	0.00466284	0.02158535	0.06763402	0.65367096	A_07_P047426	Z5886	cmr|orf Unknown function [Z5886]
Z5899	-2.29	8.36	-1.19	8.96	0.0	7.76	0.3	0.00253450	0.01328788	0.04945694	0.58457207	A_07_P047482	Z5899	cmr|putative ATP-dependent helicase [Z5899]
Z5940	+3.60	8.15	+1.85	7.23	0.0	9.08	0.1	0.00004144	0.00064284	0.01918128	0.36384337	A_07_P047549	Z5940	cmr|orf Unknown function [Z5940]
Z5987	+4.08	8.96	+2.03	7.95	0.0	9.98	0.2	0.00004437	0.00067775	0.01527058	0.33391667	A_07_P050868	Z5987	cmr|orf Unknown function [Z5987]
Z5988	+5.97	8.93	+2.58	7.64	0.0	10.22	0.2	0.00001056	0.00026108	0.00708206	0.24134199	A_07_P050871	Z5988	cmr|putative lipoate-protein ligase A [Z5988]
Z5988	+5.63	8.42	+2.49	7.18	0.2	9.67	0.3	0.00009252	0.00113352	0.00805132	0.24952756	A_07_P050871	Z5988	cmr|putative lipoate-protein ligase A [Z5988]
Z5988	+5.07	8.73	+2.34	7.55	0.1	9.90	0.0	0.00000921	0.00023970	0.00984629	0.27388693	A_07_P050871	Z5988	cmr|putative lipoate-protein ligase A [Z5988]
Z5988	+6.33	8.91	+2.66	7.58	0.1	10.24	0.1	0.00000657	0.00019615	0.00620490	0.22613426	A_07_P050871	Z5988	cmr|putative lipoate-protein ligase A [Z5988]
Z5988	+5.64	8.93	+2.50	7.68	0.1	10.18	0.2	0.00001436	0.00031409	0.00793636	0.24890438	A_07_P050871	Z5988	cmr|putative lipoate-protein ligase A [Z5988]
Z6024	+2.00	7.97	+1.00	7.47	0.0	8.47	0.1	0.00139180	0.00829394	0.06283600	0.60767199	A_07_P044177	Z6024	cmr|unknown protein encoded by cryptic prophage CP-933P [Z6024]
Z6030	+2.94	8.05	+1.56	7.27	0.3	8.82	0.5	0.00534362	0.02401652	0.02959794	0.44337774	A_07_P044196	Z6030	cmr|putative tail component of cryptic prophage CP-933P [Z6030]
Z6036	-2.25	10.55	-1.17	11.13	0.0	9.97	0.1	0.00077752	0.00537131	0.05127731	0.59186950	A_07_P044215	Z6036	cmr|putative tail assembly protein of cryptic prophage [Z6036]
Z6054	-2.49	9.88	-1.32	10.54	0.1	9.22	0.2	0.00089088	0.00600171	0.03742950	0.52568243	A_07_P044256	Z6054	cmr|conserved hypothetical YjhS family protein encoded by [Z6054]
Z6060	-3.77	8.11	-1.92	9.06	0.4	7.15	0.7	0.00776836	0.03221944	0.00816629	0.29287016	A_07_P044262	Z6060	cmr|putative Q antiterminator encoded by prophage CP-933P [Z6060]
zntA	+2.12	9.12	+1.08	8.58	0.0	9.66	0.3	0.00529656	0.02388003	0.05648438	0.58275885	A_07_P034561	c_4262	cmr|Lead, cadmium, zinc and mercury transporting ATPase [c_4262]
znuC	-2.20	9.23	-1.14	9.80	0.0	8.66	0.1	0.00076322	0.00529343	0.05494347	0.60831927	A_07_P015968	b1858	cmr|putative ATP-binding component of a transport system [b1858]
zwf	+2.80	9.48	+1.49	8.74	0.0	10.22	0.1	0.00014565	0.00157814	0.03066184	0.44906047	A_07_P015938	b1852	cmr|glucose-6-phosphate dehydrogenase [b1852]

E. coli ATCC 25922 ΔmarR S83L pBK-QnrS1 (EC24) versus *E. coli* ATCC 25922 pBK-CMV

Significant differential expression (equal to or greater than two-fold, P value <0.05)

Gene Name (ProbeID)	Fold Change	logSignal (A)	logRatio (M)	logControl	StdErr (logControl)	logExperiment	StdErr (logExperiment)	pval (limma)	FDR (limma)	pval (RankProd)	FDR (RankProd)	Oligo	Systematic Name	Description
aaeA	-28.43	9.42	-4.83	11.83	0.1	7.00	0.2	0.00000145	0.00015308	0.00001588	0.01000000	A_07_P012680	b3241	cmr|putative membrane protein [b3241]
aaeB	-5.17	8.00	-2.37	9.18	0.1	6.81	0.0	0.00003208	0.00062191	0.00582317	0.24711590	A_07_P012673	b3240	cmr|orf, hypothetical protein [b3240]
aaeX	-3.84	8.17	-1.94	9.15	0.0	7.20	0.0	0.00006848	0.00099742	0.01372015	0.37179002	A_07_P010572	b3242	cmr|orf, hypothetical protein [b3242]
ABE-0012311	+2.22	9.42	+1.15	8.84	0.1	9.99	0.1	0.00126760	0.00827408	0.05648501	0.59011281	A_07_P010617	ABE-0012311	asap|acetolactate synthase II, large subunit, C-ter fragment (pseudogene) [b3768]
ABE-0285070	-4.14	8.75	-2.05	9.77	0.2	7.72	0.2	0.00047217	0.00387253	0.01136560	0.34411538	A_07_P010568	ABE-0285070	asap|predicted protein, C-ter fragment (pseudogene) [b4549]
ABE-0285083	-12.68	11.96	-3.66	13.79	0.0	10.13	0.1	0.00000254	0.00018134	0.00029726	0.05318182	A_07_P010669	ABE-0285083	asap|KpLE2 phage-like element; predicted transposase fragment (pseudogene) [b4562]
ABE-0285085	-4.33	8.34	-2.12	9.40	0.1	7.29	0.2	0.00016210	0.00182033	0.00966717	0.31124744	A_07_P010678	ABE-0285085	asap|IS1 transposase InsAB', C-ter fragment (pseudogene) [b4564]
ABH-0247453	-24.83	11.62	-4.63	13.94	0.0	9.30	0.1	0.00000081	0.00012560	0.00003366	0.01827586	A_07_P048654	ABH-0247453	asap|predicted protein []
ABH-0285233	-3.29	8.79	-1.72	9.65	0.1	7.93	0.2	0.00033964	0.00305558	0.02267022	0.45008827	A_07_P048532	ABH-0285233	asap|non-LEE-encoded type III secreted effector []
ABR-0081722	+2.84	10.32	+1.51	9.56	0.0	11.07	0.1	0.00028106	0.00266247	0.03599212	0.47940778	A_07_P035523	ABR-0081722	asap|predicted protein [c5636]
ABR-0081747	-3.55	12.76	-1.83	13.67	0.0	11.85	0.1	0.00014560	0.00167812	0.01804370	0.40933718	A_07_P035421	ABR-0081747	asap|toxic membrane protein []
ABR-0081751	-6.31	8.46	-2.66	9.79	0.1	7.13	0.2	0.00005378	0.00084214	0.00314024	0.18656604	A_07_P041043	ABR-0081751	asap|predicted ferredoxin []
ABR-0081753	+8.65	11.17	+3.11	9.61	0.1	12.73	0.1	0.00000707	0.00028168	0.00485264	0.20931507	A_07_P041110	ABR-0081753	asap|predicted protein []
ACA-0244199	-2.28	8.70	-1.19	9.29	0.0	8.10	0.3	0.00464391	0.02238630	0.06431466	0.64908333	A_07_P059043	ACA-0244199	asap|Type II secretion pathway related protein []
ACA-0244202	-5.02	9.73	-2.33	10.89	0.0	8.57	0.0	0.00002518	0.00054604	0.00618458	0.25225389	A_07_P062880	ACA-0244202	asap|predicted protein []
ACA-0244210	-5.68	11.08	-2.51	12.33	0.1	9.82	1.0	0.00768294	0.03357207	0.00447663	0.21686154	A_07_P059088	ACA-0244210	asap|predicted protein []
ACA-0244225	-2.37	10.03	-1.25	10.65	0.1	9.41	0.4	0.00592417	0.02721628	0.05831682	0.62289009	A_07_P059144	ACA-0244225	asap|predicted protein []
ACA-0244228	-3.01	8.69	-1.59	9.49	0.2	7.89	0.1	0.00039158	0.00336992	0.02943026	0.48722397	A_07_P059159	ACA-0244228	asap|predicted protein []
ACA-0285117	-2.99	11.83	-1.58	12.62	0.2	11.04	0.3	0.00196833	0.01150478	0.03088796	0.49220648	A_07_P062850	ACA-0285117	asap|toxic polypeptide, small []
accA	-3.00	9.51	-1.58	10.31	0.1	8.72	0.1	0.00022771	0.00232347	0.02969258	0.48746611	A_07_P002882	b0185	cmr|acetylCoA carboxylase, carboxytransferase component, alpha subunit [b0185]
accB	+8.27	11.24	+3.05	9.72	0.1	12.77	0.0	0.00000739	0.00028887	0.00533600	0.21652062	A_07_P012745	b3255	cmr|acetylCoA carboxylase, BCCP subunit; carrier of biotin [b3255]
accC	+2.78	10.75	+1.48	10.02	0.1	11.49	0.1	0.00041631	0.00352830	0.03743902	0.48714050	A_07_P012747	b3256	cmr|acetyl CoA carboxylase, biotin carboxylase subunit [b3256]
accD	-3.23	11.74	-1.69	12.59	0.0	10.90	0.1	0.00015174	0.00173170	0.02387068	0.45775883	A_07_P013869	b2316	cmr|acetylCoA carboxylase, carboxytransferase component, beta subunit [b2316]
aceA	+12.24	9.62	+3.61	7.82	0.0	11.43	0.1	0.00000260	0.00018134	0.00241870	0.14424242	A_07_P008720	b4015	cmr|isocitrate lyase [b4015]
aceA	+14.10	9.55	+3.82	7.64	0.3	11.46	0.2	0.00001539	0.00040729	0.00178354	0.12480000	A_07_P037462	c_4972	cmr|Isocitrate lyase [c_4972]
aceB	+48.72	10.03	+5.61	7.23	0.0	12.84	0.3	0.00000102	0.00012946	0.00012259	0.02924242	A_07_P008715	b4014	cmr|malate synthase A [b4014]
aceE	+101.26	12.18	+6.66	8.85	0.0	15.51	0.0	0.00000009	0.00007032	0.00002668	0.01354839	A_07_P002535	b0114	cmr|pyruvate dehydrogenase [b0114]
aceF	+27.25	12.58	+4.77	10.20	0.1	14.97	0.1	0.00000087	0.00012765	0.00038681	0.05970588	A_07_P002536	b0115	cmr|pyruvate dehydrogenase [b0115]
ackA	+4.81	12.78	+2.27	11.64	0.1	13.91	0.0	0.00003360	0.00063438	0.01510480	0.33121170	A_07_P013766	b2296	cmr|acetate kinase [b2296]
acnA	+5.37	9.51	+2.42	8.29	0.1	10.72	0.1	0.00002454	0.00053591	0.01258829	0.29669162	A_07_P014361	b1276	cmr|aconitate hydrase 1 [b1276]
acnB	+10.46	10.52	+3.39	8.82	0.0	12.21	0.3	0.00002415	0.00053182	0.00332381	0.16826367	A_07_P002554	b0118	cmr|aconitate hydrase B [b0118]
acpD	+3.67	8.69	+1.88	7.76	0.1	9.63	0.1	0.00011421	0.00142293	0.02311293	0.39944018	A_07_P052470	Z2315	cmr|acyl carrier protein phosphodiesterase [Z2315]
acpD	+3.80	9.35	+1.93	8.39	0.2	10.31	0.0	0.00013216	0.00157032	0.02197853	0.39055305	A_07_P063175	ECs2014	cmr|acyl carrier protein phosphodiesterase [ECs2014]
acpS	+3.70	8.63	+1.89	7.68	0.1	9.57	0.1	0.00016121	0.00181157	0.02292873	0.39888398	A_07_P019720	b2563	cmr|CoA:apo- [b2563]
acpS	+5.14	8.83	+2.36	7.65	0.0	10.01	0.1	0.00003631	0.00065813	0.01352896	0.31004367	A_07_P053485	ECs3429	cmr|CoA:apo- [ECs3429]
acpS	+2.92	8.61	+1.55	7.83	0.1	9.38	0.0	0.00030417	0.00282365	0.03453316	0.47195313	A_07_P042915	Z3844	cmr|CoA:apo- [Z3844]
acrB	+2.84	10.58	+1.50	9.83	0.0	11.33	0.1	0.00031422	0.00289985	0.03641133	0.48213625	A_07_P021417	b0462	cmr|acridine efflux pump [b0462]
acrE	-3.74	9.18	-1.90	10.13	0.0	8.22	0.8	0.01203158	0.04799218	0.01591527	0.39090484	A_07_P012792	b3265	cmr|transmembrane protein affecting septum formation and cell membrane permeability [b3265]
acs	+24.42	9.22	+4.61	6.91	0.1	11.52	0.5	0.00002894	0.00058560	0.00053100	0.07025210	A_07_P052329	Z5668	cmr|acetyl-CoA synthetase [Z5668]
acs	+23.07	9.39	+4.53	7.13	0.2	11.66	0.0	0.00000229	0.00017963	0.00057609	0.07198413	A_07_P008986	b4069	cmr|acetyl-CoA synthetase [b4069]
actP	+5.68	8.35	+2.51	7.10	0.2	9.60	0.1	0.00009422	0.00125289	0.01112932	0.28215781	A_07_P063696	ECs5049	cmr|putative transport protein [ECs5049]
actP	+6.39	8.19	+2.68	6.85	0.1	9.53	0.4	0.00031591	0.00290970	0.00871951	0.25901887	A_07_P052321	Z5666	cmr|putative transport protein [Z5666]
actP	+6.37	8.75	+2.67	7.42	0.4	10.09	0.1	0.00020650	0.00215450	0.00889926	0.26091248	A_07_P008980	b4067	cmr|putative transport protein [b4067]
ade	+6.06	8.81	+2.60	7.52	0.0	10.11	0.0	0.00001359	0.00038685	0.01010036	0.27323024	A_07_P017261	b3665	cmr|probable adenine deaminase [b3665]
adhE	+2.88	8.39	+1.53	7.62	0.1	9.15	0.1	0.00030803	0.00285605	0.03527757	0.47633791	A_07_P007143	b1241	cmr|CoA-linked acetaldehyde dehydrogenase and iron-dependent alcohol dehydrogenase; pyruvate-formate-lyase deactivase [b1241]
adk	-2.15	10.18	-1.10	10.73	0.2	9.63	0.0	0.00232907	0.01305870	0.07391832	0.67192263	A_07_P021477	b0474	cmr|adenylate kinase activity; pleiotropic effects on glycerol-3-phosphate acyltransferase activity [b0474]
aes	-22.63	10.52	-4.50	12.77	0.1	8.27	0.0	0.00000102	0.00012946	0.00003747	0.01735294	A_07_P021486	b0476	cmr|putative lipase [b0476]
agaB	+4.54	7.89	+2.18	6.80	0.0	8.99	0.1	0.00004214	0.00072123	0.01653074	0.34335092	A_07_P000415	b3138	cmr|PTS system, cytoplasmic, N-acetylgalactosamine-specific IIB component 1 [b3138]
agaS	+26.86	9.21	+4.75	6.84	0.1	11.59	0.0	0.00000083	0.00012615	0.00041476	0.06219048	A_07_P034110	c_3893	cmr|Putative tagatose-6-phosphate ketose/aldose isomerase [c_3893]
agaS	+16.44	9.03	+4.04	7.01	0.1	11.05	0.1	0.00000205	0.00017536	0.00130843	0.10842105	A_07_P000404	b3136	cmr|putative tagatose-6-phosphate aldose/ketose isomerase [b3136]
agaV	+23.46	8.77	+4.55	6.50	0.1	11.05	0.3	0.00000497	0.00024001	0.00056911	0.07168000	A_07_P000390	b3133	cmr|PTS system, cytoplasmic, N-acetylgalactosamine-specific IIB component 2 [b3133]
agaW	+18.45	9.62	+4.21	7.52	0.3	11.72	0.1	0.00000564	0.00025427	0.00089113	0.08607362	A_07_P000391	b3134	cmr|PTS system N-acetylgalactosameine-specific IIC component 2 [b3134]
agp	+7.20	10.37	+2.85	8.95	0.3	11.80	0.2	0.00008200	0.00113151	0.00707063	0.23837259	A_07_P033935	c_1137	cmr|Glucose-1-phosphatase precursor [c_1137]
agp	+8.65	10.26	+3.11	8.71	0.1	11.82	0.1	0.00001442	0.00039421	0.00494284	0.21032432	A_07_P001241	b1002	cmr|periplasmic glucose-1-phosphatase [b1002]
ahpC	+5.87	16.01	+2.55	14.73	0.1	17.28	0.0	0.00001649	0.00042417	0.01072790	0.27779605	A_07_P016781	b0605	cmr|alkyl hydroperoxide reductase, C22 subunit; detoxification of hydroperoxides [b0605]
aidB	-3.46	9.14	-1.79	10.03	0.3	8.24	0.5	0.00447612	0.02174390	0.02010607	0.42951153	A_07_P011655	b4187	cmr|putative acyl coenzyme A dehydrogenase [b4187]
aldA	+8.29	8.99	+3.05	7.46	0.1	10.51	0.0	0.00000712	0.00028183	0.00536649	0.21719794	A_07_P009166	b1415	cmr|aldehyde dehydrogenase, NAD-linked [b1415]
allB	+6.06	9.64	+2.60	8.34	0.2	10.94	0.1	0.00003896	0.00068231	0.00996951	0.27155709	A_07_P016349	b0512	cmr|putative hydrolase [b0512]
allD	+19.26	9.13	+4.27	6.99	0.0	11.26	0.1	0.00000168	0.00015927	0.00084096	0.08487179	A_07_P016372	b0517	cmr|putative malate dehydrogenase [b0517]
alr	-3.45	9.79	-1.79	10.68	0.0	8.90	0.2	0.00019952	0.00210254	0.01901042	0.41918768	A_07_P008909	b4053	cmr|alanine racemase 1 [b4053]
alsB	+20.23	9.87	+4.34	7.70	0.1	12.04	0.1	0.00000114	0.00013852	0.00073869	0.08248227	A_07_P011177	b4088	cmr|putative LACI-type transcriptional regulator [b4088]
alsC	+2.63	9.81	+1.40	9.12	0.4	10.51	0.0	0.00463307	0.02236143	0.04201156	0.51036265	A_07_P011170	b4086	cmr|putative transport system permease protein [b4086]
alsE	+3.16	10.23	+1.66	9.40	0.1	11.06	0.1	0.00018961	0.00202942	0.03002414	0.44177570	A_07_P011164	b4085	cmr|putative epimerase [b4085]
alsK	+4.10	9.82	+2.03	8.80	0.2	10.84	0.0	0.00009398	0.00125073	0.01964367	0.37624088	A_07_P011158	b4084	cmr|putative NAGC-like transcriptional regulator [b4084]
amiA	-9.11	11.97	-3.19	13.56	0.0	10.37	0.4	0.00011200	0.00140397	0.00091336	0.10055944	A_07_P042594	c_2969	cmr|Probable N-acetylmuramoyl-L-alanine amidase amiA precursor [c_2969]
amiA	-6.59	12.17	-2.72	13.53	0.0	10.81	0.1	0.00001410	0.00039205	0.00272866	0.17679012	A_07_P052703	Z3700	cmr|N-acetylmuramoyl-l-alanine amidase I [Z3700]
amiA	-7.11	11.56	-2.83	12.97	0.0	10.15	0.2	0.00001753	0.00043403	0.00218305	0.15694064	A_07_P013234	b2435	cmr|N-acetylmuramoyl-l-alanine amidase I [b2435]
amiB	-2.77	10.59	-1.47	11.33	0.1	9.86	0.2	0.00061426	0.00472975	0.03713161	0.52571942	A_07_P011563	b4169	cmr|N-acetylmuramoyl-l-alanine amidase II; a murein hydrolase [b4169]
amn	+26.81	10.10	+4.74	7.73	0.4	12.47	0.0	0.00000851	0.00030484	0.00043509	0.06342593	A_07_P015257	b1982	cmr|AMP nucleosidase [b1982]
ampD	+6.25	12.68	+2.64	11.36	0.1	14.01	0.2	0.00003143	0.00061555	0.00947282	0.26775583	A_07_P041423	c_0129	cmr|AmpD protein [c_0129]
ampD	+7.35	11.41	+2.88	9.97	0.0	12.85	0.0	0.00000807	0.00029787	0.00693661	0.23638528	A_07_P002511	b0110	cmr|regulates ampC [b0110]
ampE	+2.43	9.26	+1.28	8.62	0.0	9.90	0.1	0.00094388	0.00659868	0.04795287	0.54510469	A_07_P002518	b0111	cmr|regulates ampC [b0111]
amyA	-3.81	8.93	-1.93	9.89	0.2	7.96	0.1	0.00026537	0.00255845	0.01449568	0.38036667	A_07_P014999	b1927	cmr|cytoplasmic alpha-amylase [b1927]
anmK	+2.70	10.46	+1.43	9.74	0.1	11.18	0.1	0.00054740	0.00432645	0.03936674	0.49782329	A_07_P052526	Z2654	cmr|orf, hypothetical protein [Z2654]
anmK	+2.69	10.30	+1.43	9.59	0.1	11.02	0.1	0.00055187	0.00435523	0.03999682	0.49937351	A_07_P063211	ECs2349	cmr|hypothetical protein [ECs2349]
ansA	+2.24	8.62	+1.16	8.04	0.2	9.20	0.2	0.00467702	0.02250458	0.05739139	0.59602243	A_07_P063239	ECs2474	cmr|cytoplasmic L-asparaginase I [ECs2474]
ansB	+54.77	13.11	+5.78	10.22	0.1	15.99	0.0	0.00000020	0.00008010	0.00010353	0.02859649	A_07_P011926	b2957	cmr|periplasmic L-asparaginase II [b2957]
ansP	-2.26	8.22	-1.18	8.81	0.0	7.64	0.1	0.00115899	0.00772199	0.06577045	0.65578847	A_07_P009360	b1453	cmr|L-asparagine permease [b1453]
apbA	+2.78	9.57	+1.47	8.84	0.1	10.31	0.0	0.00033222	0.00301296	0.03756860	0.48761748	A_07_P031138	c_0536	cmr|2-dehydropantoate 2-reductase [c_0536]
aphA	+5.76	8.93	+2.52	7.67	0.2	10.20	0.0	0.00004753	0.00077461	0.01102261	0.28035541	A_07_P008919	b4055	cmr|diadenosine tetraphosphatase [b4055]
aphA	+5.95	10.89	+2.57	9.60	0.1	12.17	0.4	0.00023056	0.00234535	0.01018801	0.27418803	A_07_P042001	c_5045	cmr|Class B acid phosphatase precursor [c_5045]
aqpZ	-2.26	7.66	-1.18	8.25	0.1	7.08	0.0	0.00107826	0.00730302	0.06610772	0.65707071	A_07_P004830	b0875	cmr|transmembrane water channel; aquaporin Z [b0875]
araA	+7.23	8.68	+2.85	7.26	0.1	10.11	0.2	0.00003697	0.00066202	0.00709096	0.23854701	A_07_P010999	b0062	cmr|L-arabinose isomerase [b0062]
araB	+3.82	8.26	+1.93	7.29	0.0	9.23	0.3	0.00039672	0.00340023	0.02198234	0.39018038	A_07_P011001	b0063	cmr|L-ribulokinase [b0063]
araC	+2.74	11.23	+1.46	10.50	0.0	11.96	0.0	0.00029169	0.00273188	0.03865028	0.49472358	A_07_P011010	b0064	cmr|transcriptional regulator for ara operon [b0064]
araF	+5.34	8.84	+2.42	7.63	0.1	10.05	0.1	0.00004296	0.00073197	0.01251651	0.29588589	A_07_P016171	b1901	cmr|L-arabinose-binding periplasmic protein [b1901]
arcA	-2.95	12.69	-1.56	13.47	0.0	11.91	0.1	0.00035019	0.00312194	0.03120681	0.49132000	A_07_P017689	b4401	cmr|negative response regulator of genes in aerobic pathways, [b4401]
arcB	+5.10	12.76	+2.35	11.58	0.5	13.93	0.1	0.00062061	0.00475701	0.01304052	0.30281711	A_07_P012528	b3210	cmr|aerobic respiration sensor-response protein; histidine protein kinase/phosphatase, sensor for arcA [b3210]
argE	+4.65	8.91	+2.22	7.80	0.2	10.02	0.0	0.00007249	0.00103660	0.01573488	0.33750681	A_07_P039521	c_4916	cmr|Acetylornithine deacetylase [c_4916]
argE	+3.25	7.84	+1.70	6.99	0.2	8.69	0.5	0.00437069	0.02133709	0.02911839	0.43827916	A_07_P008486	b3957	cmr|acetylornithine deacetylase [b3957]
argG	+2.53	8.09	+1.34	7.42	0.2	8.76	0.1	0.00120584	0.00795007	0.04409870	0.52123874	A_07_P000576	b3172	cmr|argininosuccinate synthetase [b3172]
argG	+2.24	8.62	+1.17	8.04	0.1	9.21	0.0	0.00103875	0.00711049	0.05488821	0.58271072	A_07_P041781	c_3929	cmr|Argininosuccinate synthase [c_3929]
argO	-2.26	7.82	-1.18	8.41	0.0	7.24	0.1	0.00127825	0.00832293	0.06518928	0.65288804	A_07_P011764	b2923	cmr|orf, hypothetical protein [b2923]
argR	-3.89	11.10	-1.96	12.08	0.0	10.12	0.1	0.00006715	0.00098530	0.01328379	0.36435540	A_07_P012656	b3237	cmr|repressor of arg regulon; cer-mediated site specific recombination [b3237]
argS	-6.25	11.63	-2.64	12.95	0.0	10.30	0.2	0.00002609	0.00055356	0.00321710	0.18690037	A_07_P016056	b1876	cmr|arginine tRNA synthetase [b1876]
arnA	+2.91	8.56	+1.54	7.79	0.1	9.33	0.3	0.00099733	0.00689682	0.03490917	0.47339363	A_07_P001065	b2255	cmr|putative transformylase [b2255]
arnC	+6.29	10.23	+2.65	8.91	0.2	11.56	0.0	0.00002783	0.00057043	0.00937691	0.26793103	A_07_P001060	b2254	cmr|putative sugar transferase [b2254]
arnT	+5.69	8.93	+2.51	7.67	0.2	10.18	0.2	0.00007767	0.00109371	0.01111280	0.28219355	A_07_P001072	b2257	cmr|orf, hypothetical protein [b2257]
aroA	+3.47	9.18	+1.79	8.28	0.1	10.08	0.0	0.00011645	0.00144104	0.02548463	0.41194045	A_07_P033835	c_1046	cmr|3-phosphoshikimate 1-carboxyvinyltransferase [c_1046]
aroG	+2.66	8.12	+1.41	7.42	0.2	8.83	0.0	0.00067143	0.00507975	0.04073615	0.50301961	A_07_P010251	b0754	cmr|3-deoxy-D-arabinoheptulosonate-7-phosphate synthase [b0754]
aroK	-3.08	11.58	-1.62	12.39	0.0	10.77	0.1	0.00028233	0.00267123	0.02774390	0.48211921	A_07_P017976	b3390	cmr|shikimate kinase I [b3390]
aroM	-2.57	7.88	-1.36	8.56	0.0	7.20	0.0	0.00044066	0.00368021	0.04644627	0.57488208	A_07_P031086	c_0498	cmr|AroM protein [c_0498]
aroM	-2.57	7.88	-1.36	8.56	0.0	7.20	0.2	0.00102216	0.00702132	0.04714240	0.57580295	A_07_P043806	Z0486	cmr|protein of aro operon, regulated by aroR [Z0486]
arrD	+5.00	9.12	+2.32	7.96	0.1	10.28	0.1	0.00004026	0.00069800	0.01415079	0.31964132	A_07_P016536	b0555	cmr|bacteriophage lambda lysozyme homolog [b0555]
artI	+7.39	9.19	+2.89	7.75	0.1	10.63	0.1	0.00001527	0.00040465	0.00673844	0.23419426	A_07_P004766	b0863	cmr|arginine 3rd transport system periplasmic binding protein [b0863]
artP	+7.96	9.36	+2.99	7.87	0.2	10.86	0.2	0.00003528	0.00065109	0.00586192	0.22238554	A_07_P004773	b0864	cmr|ATP-binding component of 3rd arginine transport system [b0864]
artQ	+2.61	8.29	+1.38	7.59	0.2	8.98	0.3	0.00374022	0.01895287	0.04355882	0.51796828	A_07_P033780	c_0995	cmr|Arginine transport system permease protein artQ [c_0995]
asd	+9.71	10.23	+3.28	8.59	0.1	11.87	0.0	0.00000465	0.00023240	0.00381860	0.18385321	A_07_P018194	b3433	cmr|aspartate-semialdehyde dehydrogenase [b3433]
asnA	-13.95	10.75	-3.80	12.65	0.0	8.85	0.3	0.00001250	0.00037048	0.00021596	0.05000000	A_07_P005641	b3744	cmr|asparagine synthetase A [b3744]
asnS	-2.48	10.21	-1.31	10.86	0.0	9.55	0.0	0.00052202	0.00417189	0.05152248	0.60175816	A_07_P005096	b0930	cmr|asparagine tRNA synthetase [b0930]
aspA	+10.66	15.86	+3.41	14.15	0.0	17.56	0.1	0.00000345	0.00020118	0.00311357	0.16560811	A_07_P011426	b4139	cmr|aspartate ammonia-lyase [b4139]
aspC	+4.24	9.32	+2.08	8.28	0.2	10.36	0.5	0.00123687	0.00812060	0.01845147	0.36312500	A_07_P052047	Z1275	cmr|aspartate aminotransferase [Z1275]
aspC	+9.55	11.21	+3.26	9.58	0.1	12.84	0.1	0.00000656	0.00027019	0.00391578	0.18513514	A_07_P005088	b0928	cmr|aspartate aminotransferase [b0928]
asr	-6.32	10.40	-2.66	11.73	0.0	9.07	0.1	0.00001332	0.00038273	0.00317962	0.18679104	A_07_P020616	b1597	cmr|acid shock protein [b1597]
astA	+8.45	9.05	+3.08	7.51	0.0	10.58	0.2	0.00001051	0.00034033	0.00513021	0.21367725	A_07_P018783	b1747	cmr|orf, hypothetical protein [b1747]
astC	+2.20	7.14	+1.14	6.58	0.1	7.71	0.1	0.00126116	0.00825255	0.05683689	0.59143424	A_07_P018786	b1748	cmr|acetylornithine delta-aminotransferase [b1748]
astD	+3.06	7.63	+1.61	6.82	0.0	8.44	0.0	0.00017523	0.00193062	0.03168509	0.45267695	A_07_P018776	b1746	cmr|putative aldehyde dehydrogenase [b1746]
atoC	+7.52	9.13	+2.91	7.67	0.2	10.58	0.0	0.00001847	0.00044665	0.00652884	0.23098876	A_07_P000888	b2220	cmr|response regulator of ato, ornithine decarboxylase antizyme [b2220]
atoS	+7.61	9.95	+2.93	8.48	0.1	11.41	0.1	0.00000846	0.00030484	0.00631606	0.22807339	A_07_P000883	b2219	cmr|sensor protein AtoS for response regulator AtoC [b2219]
atpA	+6.44	15.52	+2.69	14.18	0.0	16.86	0.0	0.00001254	0.00037048	0.00900788	0.26214418	A_07_P005591	b3734	cmr|membrane-bound ATP synthase, F1 sector, alpha-subunit [b3734]
atpC	+2.57	13.11	+1.36	12.43	0.1	13.79	0.2	0.00086260	0.00615350	0.04315803	0.51397882	A_07_P005580	b3731	cmr|membrane-bound ATP synthase, F1 sector, epsilon-subunit [b3731]
atpD	+5.52	11.67	+2.46	10.44	0.0	12.90	0.1	0.00002805	0.00057424	0.01187373	0.29073095	A_07_P005582	b3732	cmr|membrane-bound ATP synthase, F1 sector, beta-subunit [b3732]
atpE	+4.92	12.70	+2.30	11.55	0.0	13.85	0.1	0.00003865	0.00068020	0.01446011	0.32246459	A_07_P005607	b3737	cmr|membrane-bound ATP synthase, F0 sector, subunit c [b3737]
atpG	+4.43	12.21	+2.15	11.14	0.0	13.29	0.1	0.00005244	0.00082894	0.01701029	0.34825748	A_07_P005587	b3733	cmr|membrane-bound ATP synthase, F1 sector, gamma-subunit [b3733]
atpH	+8.48	13.14	+3.08	11.60	0.1	14.68	0.3	0.00003615	0.00065813	0.00519690	0.21307292	A_07_P005596	b3735	cmr|membrane-bound ATP synthase, F1 sector, delta-subunit [b3735]
atpI	-2.02	13.37	-1.01	13.87	0.0	12.86	0.0	0.00176330	0.01059944	0.08917937	0.72373196	A_07_P005616	b3739	cmr|membrane-bound ATP synthase, dispensable protein, affects expression of atpB [b3739]
azoR	+3.23	9.03	+1.69	8.19	0.2	9.88	0.1	0.00040756	0.00347035	0.02912919	0.43760496	A_07_P009153	b1412	cmr|acyl carrier protein phosphodiesterase [b1412]
b0257	-12.79	14.68	-3.68	16.51	0.0	12.84	0.1	0.00000373	0.00020748	0.00031314	0.05417582	A_07_P007992	b0257	cmr|putative transposase [b0257]
b0322	-6.29	9.10	-2.65	10.43	0.1	7.77	0.1	0.00002747	0.00056718	0.00324251	0.18699634	A_07_P008305	b0322	cmr|orf, hypothetical protein [b0322]
b0359	-2.73	8.46	-1.45	9.18	0.3	7.74	0.1	0.00138511	0.00883238	0.03881542	0.53559159	A_07_P020924	b0359	cmr|putative transferase [b0359]
b0359	-2.09	7.85	-1.06	8.38	0.1	7.32	0.1	0.00259398	0.01420014	0.08118394	0.69465217	A_07_P010289	b0359	cmr|putative transferase [b0359]
b0374	-8.06	8.09	-3.01	9.60	0.1	6.59	0.1	0.00001344	0.00038460	0.00149835	0.12751351	A_07_P020983	b0374	cmr|putative flagellin structural protein [b0374]
b0395	+2.02	7.38	+1.02	6.87	0.0	7.89	0.1	0.00274950	0.01486442	0.06719195	0.64741126	A_07_P021086	b0395	cmr|orf, hypothetical protein [b0395]
b0501	-3.66	8.97	-1.87	9.90	0.5	8.03	0.2	0.00281522	0.01511176	0.01581745	0.39217323	A_07_P016291	b0501	cmr|orf, hypothetical protein [b0501]
b0663	-2.08	7.56	-1.06	8.09	0.0	7.03	0.2	0.00439038	0.02141995	0.08268166	0.70250405	A_07_P009880	b0663	cmr|putative RNA [b0663]
b0671	-3.64	8.05	-1.87	8.98	0.0	7.11	0.1	0.00012182	0.00148910	0.01661268	0.39688923	A_07_P009885	b0671	cmr|putative RNA [b0671]
b0701	-2.21	7.77	-1.15	8.35	0.1	7.20	0.1	0.00185554	0.01100785	0.06960620	0.66016867	A_07_P010022	b0701	cmr|protein in rhs element [b0701]
b0725	+30.53	9.37	+4.93	6.91	0.0	11.84	0.4	0.00000744	0.00028934	0.00030996	0.05136842	A_07_P010142	b0725	cmr|orf, hypothetical protein [b0725]
b1170	-4.37	8.02	-2.13	9.09	0.1	6.96	0.1	0.00004753	0.00077461	0.00959477	0.30954918	A_07_P006805	b1170	cmr|putative part of putative ATP-binding component of a transport system [b1170]
b1364	-2.67	9.91	-1.42	10.62	0.0	9.20	0.2	0.00067561	0.00510403	0.04223450	0.56065767	A_07_P014798	b1364	cmr|orf, hypothetical protein [b1364]
b1458	-2.61	8.89	-1.38	9.58	0.1	8.20	0.1	0.00053417	0.00424957	0.04499682	0.57316343	A_07_P009383	b1458	cmr|orf, hypothetical protein [b1458]
b1543	-2.04	7.58	-1.03	8.09	0.0	7.07	0.0	0.00163025	0.00997925	0.08676004	0.71440900	A_07_P020361	b1543	cmr|putative transport protein [b1543]
b1995	-3.65	8.40	-1.87	9.34	0.0	7.47	0.1	0.00009745	0.00128285	0.01624301	0.39464506	A_07_P015310	b1995	cmr|orf, hypothetical protein [b1995]
b2999	-3.19	9.30	-1.67	10.14	0.0	8.46	0.0	0.00014445	0.00167009	0.02465257	0.46041518	A_07_P012127	b2999	cmr|orf, hypothetical protein [b2999]
b3016	-2.97	9.32	-1.57	10.11	0.0	8.54	0.3	0.00085858	0.00613593	0.02983930	0.48834719	A_07_P012215	b3016	cmr|orf, hypothetical protein [b3016]
b3036	+3.62	14.90	+1.86	13.97	0.0	15.83	0.1	0.00009092	0.00121866	0.02353468	0.40014039	A_07_P012314	b3036	cmr|orf, hypothetical protein [b3036]
b3111	+36.32	10.39	+5.18	7.80	0.0	12.99	0.2	0.00000062	0.00010566	0.00021786	0.04287500	A_07_P000282	b3111	cmr|putative L-serine dehydratase [b3111]
b3112	+51.76	10.16	+5.69	7.31	0.1	13.01	0.1	0.00000037	0.00009117	0.00011751	0.02983871	A_07_P000290	b3112	cmr|putative L-serine dehydratase [b3112]
b3435	-2.79	8.60	-1.48	9.34	0.2	7.86	0.4	0.00407329	0.02025111	0.03515879	0.51444238	A_07_P018205	b3435	cmr|low-affinity gluconate transport permease protein, fragment 2 [b3435]
b3436	-2.15	7.33	-1.11	7.88	0.1	6.77	0.2	0.00275414	0.01488028	0.07415333	0.67173188	A_07_P018209	b3436	cmr|low-affinity gluconate transport permease protein, interrupted [b3436]
b3694	+2.03	8.76	+1.02	8.25	0.0	9.27	0.1	0.00226161	0.01276227	0.06851880	0.65458738	A_07_P005403	b3694	cmr|putative FADA-type transcriptional regulator [b3694]
b3695	+2.44	8.22	+1.29	7.57	0.1	8.86	0.2	0.00149991	0.00933386	0.04811420	0.54614996	A_07_P005410	b3695	cmr|regulator protein for dgo operon [b3695]
b3762	+2.11	7.71	+1.08	7.17	0.1	8.25	0.2	0.00246080	0.01358920	0.06356834	0.63023929	A_07_P005705	b3762	cmr|orf, hypothetical protein [b3762]
b3763	+3.21	8.68	+1.68	7.83	0.0	9.52	0.2	0.00053313	0.00424558	0.02954205	0.43919736	A_07_P005708	b3763	cmr|regulator of pssA [b3763]
b3837	-3.63	11.98	-1.86	12.91	0.0	11.05	0.1	0.00008817	0.00119258	0.01631542	0.39457757	A_07_P018994	b3837	cmr|putative histone [b3837]
b3841	-3.97	10.05	-1.99	11.05	0.0	9.06	0.0	0.00005990	0.00090761	0.01260036	0.35361854	A_07_P019013	b3841	cmr|orf, hypothetical protein [b3841]
b3914	-3.63	11.90	-1.86	12.83	0.0	10.97	0.0	0.00008601	0.00117236	0.01625699	0.39437596	A_07_P019349	b3914	cmr|orf, hypothetical protein [b3914]
b4091	-3.01	9.34	-1.59	10.13	0.1	8.54	0.3	0.00112646	0.00756288	0.02850102	0.48249462	A_07_P011193	b4091	cmr|orf, hypothetical protein [b4091]
b4285	-28.71	13.50	-4.84	15.92	0.0	11.08	0.2	0.00000096	0.00012946	0.00001588	0.01041667	A_07_P003953	b4285	cmr|putative transposase [b4285]
basR	+4.23	10.20	+2.08	9.15	0.1	11.24	0.1	0.00009392	0.00125073	0.01854738	0.36364882	A_07_P037777	c_5118	cmr|Transcriptional Regulatory protein basR/pmrA [c_5118]
basS	-4.62	12.21	-2.21	13.31	0.5	11.10	0.4	0.00239380	0.01331259	0.00836763	0.29472036	A_07_P011296	b4112	cmr|sensor protein for basR [b4112]
bax	-4.04	12.52	-2.02	13.53	0.0	11.51	0.1	0.00006146	0.00091973	0.01192962	0.34589319	A_07_P007778	b3570	cmr|putative ATP-binding protein [b3570]
bcp	+12.58	10.11	+3.65	8.28	0.1	11.94	0.0	0.00000240	0.00017963	0.00230691	0.14412698	A_07_P013456	b2480	cmr|bacterioferritin comigratory protein [b2480]
bcr	-2.01	8.41	-1.00	8.91	0.0	7.91	0.3	0.00805486	0.03486823	0.08687691	0.71499739	A_07_P032275	c_2719	cmr|Bicyclomycin resistance protein [c_2719]
bcsC	+3.21	9.59	+1.68	8.75	0.1	10.43	0.2	0.00036900	0.00323294	0.02965320	0.43919097	A_07_P007585	b3530	cmr|putative oxidoreductase subunit [b3530]
bcsZ	+2.06	9.18	+1.04	8.66	0.0	9.70	0.0	0.00152512	0.00945346	0.06443598	0.63563910	A_07_P007586	b3531	cmr|putative endoglucanase [b3531]
betA	+4.33	9.46	+2.12	8.41	0.0	10.52	0.1	0.00004885	0.00078969	0.01763021	0.35585897	A_07_P043676	Z0398	cmr|choline dehydrogenase, a flavoprotein [Z0398]
betA	+8.08	10.21	+3.01	8.70	0.2	11.72	0.1	0.00002570	0.00055176	0.00569296	0.21968137	A_07_P008247	b0311	cmr|choline dehydrogenase, a flavoprotein [b0311]
betA	+9.53	10.94	+3.25	9.31	0.2	12.56	0.1	0.00001207	0.00036323	0.00397866	0.18532544	A_07_P030984	c_0431	cmr|Choline dehydrogenase [c_0431]
betB	+6.12	12.68	+2.61	11.37	0.1	13.98	0.4	0.00018003	0.00195827	0.00952490	0.26730838	A_07_P008251	b0312	cmr|NAD+-dependent betaine aldehyde dehydrogenase [b0312]
bhsA	-32.67	10.21	-5.03	12.73	0.1	7.70	0.3	0.00000286	0.00018535	0.00000889	0.00823529	A_07_P001765	b1112	cmr|orf, hypothetical protein [b1112]
birA	-2.60	11.25	-1.38	11.94	0.0	10.56	0.4	0.00419268	0.02064735	0.04500064	0.57274859	A_07_P008550	b3973	cmr|biotin- [b3973]
bolA	+6.98	9.53	+2.80	8.13	0.0	10.94	0.0	0.00000929	0.00032222	0.00765244	0.24739220	A_07_P021288	b0435	cmr|possible regulator of murein genes [b0435]
bssS	-10.94	13.08	-3.45	14.80	0.0	11.35	0.0	0.00000320	0.00019528	0.00058117	0.07820513	A_07_P001504	b1060	cmr|orf, hypothetical protein [b1060]
btuB	-4.50	11.41	-2.17	12.49	0.0	10.32	0.0	0.00003551	0.00065308	0.00874428	0.30190789	A_07_P008533	b3966	cmr|outer membrane receptor for transport of vitamin B12, E colicins, and bacteriophage BF23 [b3966]
btuE	+2.06	9.01	+1.05	8.48	0.1	9.53	0.0	0.00185833	0.01101870	0.06497396	0.63774938	A_07_P044422	Z2739	cmr|vitamin B12 transport [Z2739]
btuE	+2.24	8.77	+1.16	8.19	0.1	9.35	0.1	0.00155978	0.00961898	0.05574822	0.58669786	A_07_P044422	Z2739	cmr|vitamin B12 transport [Z2739]
btuE	-2.19	8.56	-1.13	9.12	0.0	7.99	0.3	0.00739439	0.03252788	0.07182482	0.66596584	A_07_P018601	b1710	cmr|vitamin B12 transport [b1710]
c_0002	-2.23	11.40	-1.16	11.98	0.2	10.82	0.1	0.00336461	0.01741940	0.06895389	0.65754694	A_07_P035604	c_0002	cmr|Hypothetical protein [c_0002]
c_0059	-3.51	11.51	-1.81	12.41	0.1	10.60	0.2	0.00026862	0.00257833	0.01844322	0.41422254	A_07_P035683	c_0059	cmr|Putative antitoxin of gyrase inhibiting toxin-antitoxin system [c_0059]
c_0062	-3.16	7.65	-1.66	8.48	0.1	6.82	0.2	0.00046401	0.00382483	0.02541222	0.46684947	A_07_P035698	c_0062	cmr|Hypothetical protein [c_0062]
c_0086	-3.21	10.33	-1.68	11.17	0.2	9.49	0.2	0.00053795	0.00427318	0.02462907	0.46052257	A_07_P035735	c_0086	cmr|Transposase [c_0086]
c_0088	-5.55	8.63	-2.47	9.87	0.1	7.39	0.0	0.00001944	0.00046369	0.00468051	0.22262840	A_07_P035738	c_0088	cmr|Hypothetical protein [c_0088]
c_0120	-6.11	9.67	-2.61	10.97	0.0	8.36	0.2	0.00004500	0.00075371	0.00348069	0.19783394	A_07_P035766	c_0120	cmr|Hypothetical protein [c_0120]
c_0138	-2.26	9.35	-1.18	9.94	0.0	8.76	0.1	0.00104915	0.00715678	0.06717670	0.65732132	A_07_P035783	c_0138	cmr|Unknown protein of IS629 encoded within prophage [c_0138]
c_0161	-2.17	7.26	-1.12	7.82	0.1	6.70	0.2	0.00321879	0.01686411	0.07168509	0.66663320	A_07_P035804	c_0161	cmr|Hypothetical protein [c_0161]
c_0192	-2.21	7.51	-1.15	8.08	0.0	6.93	0.0	0.00104336	0.00712961	0.06947790	0.65934901	A_07_P035893	c_0192	cmr|Hypothetical protein [c_0192]
c_0226	-3.07	9.15	-1.62	9.96	0.1	8.34	0.2	0.00048449	0.00393588	0.02714367	0.47855543	A_07_P035924	c_0226	cmr|Putative cell cycle protein mesJ [c_0226]
c_0253	-4.10	8.27	-2.03	9.28	0.0	7.25	0.1	0.00005724	0.00087928	0.01149454	0.34602294	A_07_P035949	c_0253	cmr|Hypothetical protein [c_0253]
c_0256	-2.46	8.07	-1.30	8.72	0.1	7.42	0.1	0.00085849	0.00613593	0.05237614	0.60278509	A_07_P035961	c_0256	cmr|Hypothetical protein [c_0256]
c_0257	-13.36	10.15	-3.74	12.02	0.0	8.28	0.1	0.00000266	0.00018406	0.00024835	0.05077922	A_07_P035967	c_0257	cmr|Unknown in ISEc8 [c_0257]
c_0258	-3.33	11.38	-1.74	12.25	0.2	10.51	0.3	0.00086598	0.00617479	0.02235455	0.44948914	A_07_P035972	c_0258	cmr|Unknown in ISEc8 [c_0258]
c_0259	-2.95	8.76	-1.56	9.54	0.0	7.98	0.1	0.00023167	0.00234711	0.03130462	0.49138584	A_07_P035982	c_0259	cmr|Hypothetical protein [c_0259]
c_0260	-2.70	7.75	-1.43	8.47	0.1	7.04	0.4	0.00335277	0.01737166	0.04024200	0.54618103	A_07_P035978	c_0260	cmr|Hypothetical protein [c_0260]
c_0261	-2.05	7.91	-1.03	8.43	0.0	7.40	0.1	0.00184596	0.01097122	0.08643166	0.71394544	A_07_P035987	c_0261	cmr|Hypothetical protein [c_0261]
c_0263	-2.42	7.28	-1.28	7.92	0.0	6.64	0.3	0.00392029	0.01962717	0.05277820	0.60432000	A_07_P035997	c_0263	cmr|Putative Transposase [c_0263]
c_0264	-3.35	8.04	-1.74	8.91	0.0	7.17	0.0	0.00011750	0.00144794	0.02122713	0.44031621	A_07_P036004	c_0264	cmr|Hypothetical protein [c_0264]
c_0273	-2.29	8.23	-1.20	8.83	0.1	7.64	0.0	0.00099997	0.00690783	0.06311928	0.64278784	A_07_P036036	c_0273	cmr|Putative radC-like protein yeeS [c_0273]
c_0274	-2.75	8.18	-1.46	8.91	0.1	7.45	0.2	0.00086921	0.00619503	0.03747713	0.52729223	A_07_P036041	c_0274	cmr|Hypothetical protein [c_0274]
c_0276	-2.82	9.32	-1.50	10.07	0.0	8.57	0.1	0.00034039	0.00305885	0.03535633	0.51398892	A_07_P036054	c_0276	cmr|Hypothetical protein [c_0276]
c_0278	-2.50	9.34	-1.32	10.00	0.0	8.68	0.4	0.00418973	0.02063927	0.04813453	0.57982402	A_07_P036062	c_0278	cmr|Hypothetical protein yafZ [c_0278]
c_0279	-2.78	7.91	-1.47	8.65	0.2	7.18	0.2	0.00126712	0.00827408	0.03725419	0.52603587	A_07_P036068	c_0279	cmr|Hypothetical protein ykfF [c_0279]
c_0280	-2.48	10.37	-1.31	11.02	0.4	9.71	0.3	0.00777353	0.03389269	0.05237614	0.60322604	A_07_P036072	c_0280	cmr|Hypothetical protein [c_0280]
c_0281	-3.00	10.27	-1.58	11.07	0.0	9.48	0.2	0.00043040	0.00361396	0.02933435	0.48665964	A_07_P036079	c_0281	cmr|Hypothetical protein [c_0281]
c_0287	-2.19	13.08	-1.13	13.65	0.1	12.52	0.3	0.00857162	0.03666165	0.07219004	0.66817166	A_07_P036106	c_0287	cmr|Hypothetical protein [c_0287]
c_0289	-5.53	8.25	-2.47	9.48	0.1	7.01	0.1	0.00003054	0.00060552	0.00476054	0.22306548	A_07_P036114	c_0289	cmr|Hypothetical protein [c_0289]
c_0297	-2.20	10.33	-1.14	10.90	0.3	9.76	0.3	0.01169639	0.04688085	0.07227198	0.66775235	A_07_P036146	c_0297	cmr|Hypothetical protein [c_0297]
c_0301	-2.47	7.53	-1.30	8.18	0.0	6.88	0.2	0.00105416	0.00717229	0.05164380	0.60139053	A_07_P036159	c_0301	cmr|Hypothetical protein [c_0301]
c_0304	-2.61	7.52	-1.38	8.21	0.1	6.83	0.1	0.00065516	0.00498057	0.04415079	0.56836468	A_07_P036164	c_0304	cmr|Hypothetical protein [c_0304]
c_0305	-3.89	9.82	-1.96	10.80	0.3	8.84	0.4	0.00189366	0.01116204	0.01389736	0.37465753	A_07_P036170	c_0305	cmr|Hypothetical protein [c_0305]
c_0309	-3.29	8.25	-1.72	9.11	0.1	7.40	0.2	0.00030950	0.00286300	0.02208079	0.44798969	A_07_P036181	c_0309	cmr|Conserved hypothetical protein [c_0309]
c_0313	-2.96	12.93	-1.57	13.72	0.3	12.15	0.1	0.00103013	0.00705826	0.03004700	0.48971014	A_07_P036201	c_0313	cmr|Hypothetical protein [c_0313]
c_0316	-2.08	8.81	-1.06	9.34	0.1	8.28	0.0	0.00152514	0.00945346	0.08131034	0.69497828	A_07_P036219	c_0316	cmr|Hypothetical protein [c_0316]
c_0319	+3.27	9.02	+1.71	8.17	0.1	9.88	0.2	0.00029668	0.00277317	0.02833778	0.43611926	A_07_P036230	c_0319	cmr|Putative oligogalacturonide lyase [c_0319]
c_0326	-2.02	10.31	-1.01	10.81	0.0	9.80	0.2	0.00516755	0.02438786	0.08776613	0.71930765	A_07_P036256	c_0326	cmr|Hypothetical protein [c_0326]
c_0327	+2.26	7.48	+1.17	6.90	0.1	8.07	0.1	0.00128579	0.00835817	0.05436801	0.57992547	A_07_P036262	c_0327	cmr|Conserved hypothetical protein [c_0327]
c_0331	+6.91	8.99	+2.79	7.60	0.0	10.39	0.1	0.00001414	0.00039205	0.00785823	0.24943548	A_07_P036277	c_0331	cmr|Putative ribokinase [c_0331]
c_0332	+7.96	10.11	+2.99	8.61	0.0	11.60	0.0	0.00000657	0.00027019	0.00583079	0.22227603	A_07_P030713	c_0332	cmr|Putative L-fucose permease [c_0332]
c_0337	-5.15	10.81	-2.36	11.99	0.0	9.63	0.1	0.00002583	0.00055176	0.00574759	0.24523035	A_07_P030738	c_0337	cmr|Putative conserved protein [c_0337]
c_0339	-2.46	9.45	-1.30	10.10	0.0	8.80	0.3	0.00230415	0.01295591	0.05238821	0.60248356	A_07_P030741	c_0339	cmr|Hypothetical protein [c_0339]
c_0340	-4.03	8.50	-2.01	9.51	0.0	7.49	0.3	0.00034191	0.00307078	0.01182609	0.34736940	A_07_P030749	c_0340	cmr|Conserved hypothetical protein [c_0340]
c_0341	-2.13	9.32	-1.09	9.86	0.0	8.77	0.1	0.00184673	0.01097166	0.07604294	0.67487035	A_07_P030753	c_0341	cmr|Hypothetical protein [c_0341]
c_0348	-2.11	9.25	-1.08	9.79	0.0	8.71	0.0	0.00132778	0.00855696	0.07967543	0.68885777	A_07_P030761	c_0348	cmr|Hypothetical protein [c_0348]
c_0349	-3.16	8.05	-1.66	8.88	0.2	7.22	0.2	0.00056832	0.00444936	0.02584985	0.47049711	A_07_P030767	c_0349	cmr|Putative Transposase within prophage [c_0349]
c_0352	-7.21	9.48	-2.85	10.90	0.1	8.05	0.1	0.00001082	0.00034789	0.00210366	0.15771429	A_07_P030781	c_0352	cmr|Partial Transposase [c_0352]
c_0355	-4.92	9.10	-2.30	10.25	0.2	7.95	0.1	0.00006070	0.00091356	0.00679497	0.26812030	A_07_P030788	c_0355	cmr|Hypothetical protein [c_0355]
c_0357	-2.42	8.21	-1.28	8.85	0.1	7.57	0.4	0.00528911	0.02487951	0.05252922	0.60366423	A_07_P030794	c_0357	cmr|Hypothetical protein [c_0357]
c_0358	-2.34	8.60	-1.23	9.21	0.1	7.99	0.1	0.00114534	0.00765378	0.05978913	0.63006693	A_07_P030798	c_0358	cmr|Hypothetical protein [c_0358]
c_0361	-2.98	12.19	-1.57	12.98	0.3	11.40	0.1	0.00149040	0.00930330	0.02958397	0.48720711	A_07_P030810	c_0361	cmr|Putative cytoplasmic membrane export protein [c_0361]
c_0365	-4.07	8.36	-2.03	9.37	0.1	7.35	0.1	0.00008354	0.00114971	0.01166095	0.34969524	A_07_P030828	c_0365	cmr|Hypothetical protein [c_0365]
c_0385	-3.52	8.29	-1.82	9.19	0.0	7.38	0.0	0.00009873	0.00129314	0.01825457	0.41115880	A_07_P030859	c_0385	cmr|Hypothetical protein [c_0385]
c_0386	+2.39	10.11	+1.26	9.48	0.1	10.74	0.1	0.00079306	0.00575390	0.04876334	0.54837857	A_07_P041475	c_0386	cmr|Hypothetical protein yafA [c_0386]
c_0393	-2.53	7.88	-1.34	8.55	0.2	7.21	0.0	0.00117526	0.00780071	0.04792556	0.58041538	A_07_P030878	c_0393	cmr|Haemoglobin protease [c_0393]
c_0396	-2.56	7.61	-1.36	8.29	0.0	6.93	0.1	0.00045683	0.00378148	0.04708905	0.57604507	A_07_P030886	c_0396	cmr|Insertion element IS1 1/2/3/5/6 protein insA [c_0396]
c_0397	-4.01	7.73	-2.00	8.73	0.0	6.72	0.1	0.00007628	0.00107909	0.01216781	0.34517117	A_07_P030892	c_0397	cmr|InsB protein [c_0397]
c_0409	+3.07	7.98	+1.62	7.17	0.3	8.79	0.4	0.00472740	0.02268462	0.03280234	0.46193202	A_07_P030911	c_0409	cmr|Putative oxidoreductase [c_0409]
c_0410	+4.85	8.78	+2.28	7.64	0.0	9.92	0.1	0.00003225	0.00062449	0.01491235	0.32882353	A_07_P030916	c_0410	cmr|Hypothetical protein ycjY [c_0410]
c_0412	-2.25	9.74	-1.17	10.32	0.1	9.15	0.2	0.00205722	0.01188584	0.06830793	0.65897059	A_07_P030929	c_0412	cmr|Hypothetical transcriptional regulator ycjZ [c_0412]
c_0413	+4.31	7.70	+2.11	6.65	0.1	8.76	0.3	0.00048516	0.00393937	0.01778265	0.35756066	A_07_P030931	c_0413	cmr|Putative aldo/keto reductase [c_0413]
c_0414	+6.18	8.74	+2.63	7.42	0.0	10.05	0.2	0.00003196	0.00062114	0.00968623	0.26895944	A_07_P030940	c_0414	cmr|2,5-diketo-D-gluconic acid reductase A [c_0414]
c_0427	-3.06	8.17	-1.62	8.97	0.3	7.36	0.4	0.00640538	0.02901217	0.02647802	0.47371591	A_07_P030962	c_0427	cmr|Conserved hypothetical protein [c_0427]
c_0468	-2.44	8.62	-1.29	9.26	0.2	7.97	0.5	0.01117280	0.04531286	0.05404154	0.60730193	A_07_P031051	c_0468	cmr|Putative transferase [c_0468]
c_0470	-2.85	9.91	-1.51	10.66	0.1	9.15	0.5	0.00833678	0.03588145	0.03534172	0.51425139	A_07_P031058	c_0470	cmr|Putative conserved protein [c_0470]
c_0481	-3.12	8.70	-1.64	9.52	0.0	7.88	0.1	0.00023276	0.00234931	0.02625508	0.47295195	A_07_P031085	c_0481	cmr|Hypothetical protein [c_0481]
c_0503	+2.28	9.36	+1.19	8.77	0.1	9.96	0.0	0.00142359	0.00900121	0.05360963	0.57573670	A_07_P031097	c_0503	cmr|Hypothetical protein yajF [c_0503]
c_0583	-3.03	10.71	-1.60	11.51	0.1	9.91	0.1	0.00034930	0.00311578	0.02890625	0.48466454	A_07_P031182	c_0583	cmr|Hypothetical protein [c_0583]
c_0608	-2.61	10.55	-1.38	11.24	0.1	9.86	0.3	0.00267354	0.01451457	0.04315930	0.56390041	A_07_P031211	c_0608	cmr|Hypothetical protein [c_0608]
c_0608	-2.58	10.17	-1.37	10.85	0.0	9.48	0.2	0.00145015	0.00910331	0.04510417	0.57267742	A_07_P031211	c_0608	cmr|Hypothetical protein [c_0608]
c_0638	-2.09	8.52	-1.06	9.05	0.1	7.99	0.1	0.00219482	0.01247029	0.08005208	0.69135491	A_07_P031250	c_0638	cmr|Hypothetical protein [c_0638]
c_0640	+2.42	7.74	+1.28	7.10	0.1	8.38	0.0	0.00071629	0.00533460	0.04798209	0.54504329	A_07_P031257	c_0640	cmr|Conserved hypothetical protein [c_0640]
c_0697	-4.42	8.45	-2.14	9.52	0.1	7.38	0.7	0.00359687	0.01838386	0.00924987	0.30723629	A_07_P031357	c_0697	cmr|Conserved hypothetical protein [c_0697]
c_0719	-3.44	7.87	-1.78	8.77	0.1	6.98	0.1	0.00017085	0.00190094	0.01960810	0.42231190	A_07_P033287	c_0719	cmr|Conserved hypothetical protein [c_0719]
c_0748	-2.00	8.99	-1.00	9.49	0.1	8.50	0.0	0.00208134	0.01199877	0.09079268	0.72450076	A_07_P041064	c_0748	cmr|2-octaprenyl-3-methyl-6-methoxy-1,4-benzoquinol hydroxylase [c_0748]
c_0761	+3.00	7.78	+1.58	6.99	0.0	8.57	0.1	0.00028309	0.00267294	0.03299098	0.46334523	A_07_P033336	c_0761	cmr|Putative dihydrodipicolinate synthase [c_0761]
c_0762	+2.05	7.54	+1.04	7.02	0.1	8.06	0.1	0.00247721	0.01366069	0.06535887	0.63873991	A_07_P033342	c_0762	cmr|Putative alcohol dehydrogenase [c_0762]
c_0778	+147.08	10.33	+7.20	6.73	0.0	13.93	0.0	0.00000006	0.00007032	0.00000889	0.00666667	A_07_P033361	c_0778	cmr|Hypothetical protein [c_0778]
c_0784	-6.33	8.59	-2.66	9.92	0.1	7.26	0.3	0.00010149	0.00131952	0.00314088	0.18590226	A_07_P033375	c_0784	cmr|Hypothetical protein [c_0784]
c_0795	+2.06	7.63	+1.04	7.11	0.0	8.15	0.2	0.00425041	0.02087288	0.06800749	0.65327029	A_07_P033384	c_0795	cmr|Putative conserved protein [c_0795]
c_0897	+53.48	14.01	+5.74	11.14	0.1	16.88	0.0	0.00000028	0.00009047	0.00010226	0.02927273	A_07_P033506	c_0897	cmr|Hypothetical protein [c_0897]
c_0937	-3.07	8.49	-1.62	9.30	0.5	7.68	0.3	0.00544177	0.02540548	0.02655805	0.47460840	A_07_P033548	c_0937	cmr|Hypothetical protein [c_0937]
c_0947	-2.86	11.75	-1.52	12.51	0.2	11.00	0.3	0.00146229	0.00915766	0.03470910	0.51214620	A_07_P033599	c_0947	cmr|Probable capsid portal protein [c_0947]
c_0957	-2.83	8.99	-1.50	9.74	0.0	8.23	0.1	0.00035949	0.00317256	0.03523310	0.51457328	A_07_P033650	c_0957	cmr|Fels-2 prophage: probable prophage lysozyme [c_0957]
c_0958	-2.28	9.16	-1.19	9.75	0.1	8.56	0.0	0.00102487	0.00703381	0.06434769	0.64817019	A_07_P033652	c_0958	cmr|Putative membrane protein [c_0958]

c_0965	-2.15	8.02	-1.10	8.57	0.1	7.47	0.2	0.00335318	0.01737166	0.07310976	0.66882045	A_07_P033690	c_0965	cmr|Phage baseplate assembly protein [c_0965]
c_1020	-5.09	8.72	-2.35	9.90	0.1	7.55	0.1	0.00003112	0.00061325	0.00611153	0.25057292	A_07_P033805	c_1020	cmr|Hypothetical protein [c_1020]
c_1094	-2.58	10.65	-1.37	11.33	0.3	9.96	0.1	0.00178674	0.01069540	0.04521596	0.57317230	A_07_P033878	c_1094	cmr|Hypothetical protein [c_1094]
c_1109	-2.45	7.36	-1.29	8.01	0.2	6.72	0.1	0.00136602	0.00873191	0.05187691	0.60099338	A_07_P033900	c_1109	cmr|Hypothetical protein [c_1109]
c_1138	+4.44	9.63	+2.15	8.56	0.3	10.71	0.1	0.00036663	0.00321752	0.01693407	0.34760104	A_07_P033937	c_1138	cmr|Hypothetical protein [c_1138]
c_1165	-4.05	10.54	-2.02	11.55	0.1	9.53	0.3	0.00033452	0.00302754	0.01211509	0.34617060	A_07_P033966	c_1165	cmr|Putative P4-family integrase [c_1165]
c_1166	+14.00	9.89	+3.81	7.99	0.2	11.79	0.1	0.00000552	0.00025298	0.00188135	0.12822511	A_07_P033974	c_1166	cmr|Conserved hypothetical protein [c_1166]
c_1172	-3.92	8.18	-1.97	9.17	0.1	7.20	0.2	0.00026658	0.00256661	0.01358359	0.36936097	A_07_P034010	c_1172	cmr|Hypothetical protein [c_1172]
c_1173	-7.27	11.03	-2.86	12.46	0.1	9.59	0.4	0.00014505	0.00167552	0.00196456	0.15014563	A_07_P034004	c_1173	cmr|Hypothetical protein [c_1173]
c_1174	-2.55	10.03	-1.35	10.70	0.2	9.35	0.3	0.00350400	0.01798140	0.04594830	0.57231804	A_07_P040226	c_1174	cmr|Hypothetical protein [c_1174]
c_1174	-2.12	9.96	-1.08	10.50	0.1	9.41	0.2	0.00266431	0.01448442	0.07835937	0.68272828	A_07_P040226	c_1174	cmr|Hypothetical protein [c_1174]
c_1175	+6.89	8.26	+2.79	6.87	0.1	9.66	0.3	0.00008006	0.00111049	0.00770198	0.24797546	A_07_P040232	c_1175	cmr|Putative aminotransferase [c_1175]
c_1187	+5.89	7.64	+2.56	6.36	0.4	8.92	0.0	0.00016727	0.00187302	0.01040650	0.27769492	A_07_P040261	c_1187	cmr|3-oxoacyl- [c_1187]
c_1189	+5.20	8.29	+2.38	7.10	0.0	9.48	0.0	0.00002203	0.00050626	0.01323869	0.30561584	A_07_P040271	c_1189	cmr|Putative 3-oxoacyl- [c_1189]
c_1190	+8.69	8.57	+3.12	7.01	0.0	10.13	0.1	0.00000852	0.00030484	0.00481898	0.20843407	A_07_P040280	c_1190	cmr|Conserved hypothetical protein [c_1190]
c_1191	+2.05	7.23	+1.04	6.71	0.1	7.74	0.2	0.00475415	0.02276887	0.06578887	0.64174721	A_07_P040284	c_1191	cmr|Conserved hypothetical protein [c_1191]
c_1192	+4.34	8.63	+2.12	7.57	0.1	9.68	0.0	0.00004550	0.00075592	0.01775152	0.35739130	A_07_P040286	c_1192	cmr|Conserved hypothetical protein [c_1192]
c_1193	+7.00	9.36	+2.81	7.96	0.1	10.76	0.2	0.00003170	0.00061914	0.00749174	0.24624217	A_07_P040293	c_1193	cmr|Hypothetical protein [c_1193]
c_1194	+4.78	7.97	+2.26	6.84	0.0	9.10	0.1	0.00003914	0.00068389	0.01511433	0.33050000	A_07_P040296	c_1194	cmr|Putative enzyme [c_1194]
c_1200	+2.01	10.26	+1.00	9.75	0.0	10.76	0.1	0.00245447	0.01356706	0.06988313	0.66080480	A_07_P040321	c_1200	cmr|Putative acyl carrier protein [c_1200]
c_1205	-2.17	9.21	-1.12	9.77	0.0	8.65	0.1	0.00115753	0.00771881	0.07366552	0.67156340	A_07_P040348	c_1205	cmr|Hypothetical protein [c_1205]
c_1209	-2.23	9.65	-1.16	10.23	0.2	9.07	0.4	0.01197511	0.04781538	0.06433117	0.64841869	A_07_P040366	c_1209	cmr|Hypothetical protein [c_1209]
c_1210	-2.76	8.04	-1.47	8.78	0.1	7.31	0.3	0.00180614	0.01078199	0.03774454	0.52916296	A_07_P040372	c_1210	cmr|Hypothetical protein [c_1210]
c_1215	-3.68	12.73	-1.88	13.67	0.0	11.79	0.1	0.00008572	0.00116948	0.01574759	0.39291601	A_07_P040390	c_1215	cmr|Entry exclusion protein 2 [c_1215]
c_1219	-2.58	8.19	-1.37	8.88	0.0	7.51	0.5	0.00761766	0.03332381	0.04539317	0.57127898	A_07_P040407	c_1219	cmr|Putative Transposase [c_1219]
c_1220	-2.07	9.22	-1.05	9.74	0.2	8.69	0.2	0.00875090	0.03719607	0.08568979	0.71267829	A_07_P040413	c_1220	cmr|Phospho-2-dehydro-3-deoxyheptonate aldolase, Trp-sensitive [c_1220]
c_1225	-2.71	10.02	-1.44	10.73	0.1	9.30	0.3	0.00175968	0.01059037	0.03892022	0.53562937	A_07_P040430	c_1225	cmr|Transposase insE for insertion sequence IS3A/B/C/D/E/fA/fB [c_1225]
c_1235	-12.55	8.68	-3.65	10.51	0.0	6.86	0.0	0.00000232	0.00017963	0.00032076	0.05430108	A_07_P040469	c_1235	cmr|Conserved hypothetical protein [c_1235]
c_1236	-7.97	8.79	-2.99	10.29	0.0	7.29	0.1	0.00000828	0.00030193	0.00149644	0.12874317	A_07_P040474	c_1236	cmr|Conserved hypothetical protein [c_1236]
c_1246	+7.11	10.49	+2.83	9.07	0.1	11.90	0.1	0.00001620	0.00041877	0.00737995	0.24409664	A_07_P040521	c_1246	cmr|Hypothetical protein [c_1246]
c_1253	-3.62	9.79	-1.86	10.72	0.1	8.86	0.1	0.00012043	0.00147672	0.01666921	0.39703480	A_07_P040560	c_1253	cmr|ATP binding cassette [c_1253]
c_1257	-20.51	11.11	-4.36	13.29	0.1	8.93	0.1	0.00000127	0.00014546	0.00004573	0.02000000	A_07_P040573	c_1257	cmr|Putative conserved protein [c_1257]
c_1258	-2.36	7.73	-1.24	8.35	0.4	7.11	0.0	0.00566401	0.02623543	0.05657965	0.61689058	A_07_P040577	c_1258	cmr|Hypothetical protein [c_1258]
c_1259	-2.13	10.01	-1.09	10.55	0.2	9.46	0.3	0.01075793	0.04409603	0.07886941	0.68452040	A_07_P040585	c_1259	cmr|Hypothetical protein [c_1259]
c_1261	-2.01	9.53	-1.00	10.03	0.0	9.03	0.2	0.00365562	0.01863115	0.09111979	0.72454040	A_07_P040595	c_1261	cmr|Hypothetical protein [c_1261]
c_1268	-2.05	8.46	-1.03	8.98	0.0	7.94	0.1	0.00234821	0.01312861	0.08428862	0.70775467	A_07_P040616	c_1268	cmr|Hypothetical protein [c_1268]
c_1272	-3.75	8.18	-1.91	9.13	0.1	7.22	0.1	0.00013683	0.00160881	0.01488758	0.38614498	A_07_P040636	c_1272	cmr|Hypothetical protein yeeP [c_1272]
c_1273	+7.59	11.90	+2.92	10.43	0.1	13.36	0.2	0.00002542	0.00054964	0.00644881	0.23022676	A_07_P040643	c_1273	cmr|Antigen 43 precursor [c_1273]
c_1278	-2.42	9.10	-1.27	9.73	0.0	8.46	0.3	0.00377927	0.01910752	0.05272993	0.60420670	A_07_P040661	c_1278	cmr|Hypothetical protein yafZ [c_1278]
c_1284	-4.43	11.18	-2.15	12.26	0.2	10.11	0.6	0.00293038	0.01564459	0.00966972	0.31069388	A_07_P040687	c_1284	cmr|Hypothetical protein yeeT [c_1284]
c_1286	-9.03	9.86	-3.18	11.45	0.0	8.27	0.2	0.00001133	0.00035103	0.00105818	0.10412500	A_07_P040696	c_1286	cmr|Hypothetical protein [c_1286]
c_1294	-3.48	7.74	-1.80	8.64	0.0	6.84	0.1	0.00014534	0.00167760	0.01920033	0.41984722	A_07_P040732	c_1294	cmr|Hypothetical protein [c_1294]
c_1323	+2.74	8.01	+1.45	7.29	0.1	8.74	0.0	0.00032449	0.00296332	0.03893928	0.49600324	A_07_P040769	c_1323	cmr|Cytochrome b561 homolog 2 [c_1323]
c_1406	-3.49	8.26	-1.80	9.16	0.1	7.36	0.4	0.00175340	0.01056472	0.01837907	0.41337143	A_07_P040862	c_1406	cmr|Hypothetical protein [c_1406]
c_1413	-4.06	9.67	-2.02	10.68	0.2	8.66	0.1	0.00017714	0.00193940	0.01185023	0.34678439	A_07_P040889	c_1413	cmr|Unknown protein encoded within prophage [c_1413]
c_1413	-2.57	10.14	-1.36	10.82	0.1	9.46	0.3	0.00223929	0.01266814	0.04720020	0.57606202	A_07_P040889	c_1413	cmr|Unknown protein encoded within prophage [c_1413]
c_1413	-3.68	9.78	-1.88	10.72	0.3	8.84	0.1	0.00044760	0.00372658	0.01584413	0.39160126	A_07_P040889	c_1413	cmr|Unknown protein encoded within prophage [c_1413]
c_1414	-2.99	8.83	-1.58	9.63	0.2	8.04	0.6	0.00991262	0.04113502	0.03041476	0.49062500	A_07_P040893	c_1414	cmr|Hypothetical protein [c_1414]
c_1414	-2.45	8.97	-1.30	9.62	0.1	8.33	0.1	0.00063490	0.00484533	0.05358295	0.60517217	A_07_P040893	c_1414	cmr|Hypothetical protein [c_1414]
c_1414	-2.01	9.02	-1.01	9.52	0.0	8.52	0.2	0.00515178	0.02433532	0.09142721	0.72551915	A_07_P040893	c_1414	cmr|Hypothetical protein [c_1414]
c_1414	-2.24	8.80	-1.16	9.38	0.0	8.22	0.1	0.00118492	0.00785490	0.06678100	0.65671455	A_07_P040893	c_1414	cmr|Hypothetical protein [c_1414]
c_1421	-11.90	9.55	-3.57	11.34	0.1	7.76	0.0	0.00000334	0.00019859	0.00040650	0.06213592	A_07_P040925	c_1421	cmr|Hypothetical protein [c_1421]
c_1436	-2.91	8.73	-1.54	9.50	0.2	7.96	0.3	0.00316710	0.01663205	0.03119982	0.49170170	A_07_P042220	c_1436	cmr|Probable lysozyme from lambdoid prophage Qin [c_1436]
c_1448	+11.89	10.84	+3.57	9.05	0.1	12.63	0.2	0.00000537	0.00024927	0.00249809	0.14354015	A_07_P041021	c_1448	cmr|Putative capsid assembly protein of prophage [c_1448]
c_1453	+6.09	10.94	+2.61	9.64	0.1	12.25	0.2	0.00004816	0.00078282	0.00978722	0.27033333	A_07_P038108	c_1453	cmr|Putative head-tail joining protein of prophage [c_1453]
c_1459	+2.00	8.32	+1.00	7.82	0.3	8.82	0.1	0.01115716	0.04526940	0.07630780	0.68690109	A_07_P038138	c_1459	cmr|Putative tail component of prophage [c_1459]
c_1461	+2.09	8.22	+1.06	7.69	0.2	8.76	0.0	0.00273176	0.01478982	0.06394881	0.63281584	A_07_P038146	c_1461	cmr|Putative tail fiber component M of prophage [c_1461]
c_1487	-2.38	10.35	-1.25	10.98	0.1	9.72	0.3	0.00297776	0.01582777	0.05558181	0.61366059	A_07_P038235	c_1487	cmr|Hypothetical protein [c_1487]
c_1496	-3.34	9.26	-1.74	10.13	0.5	8.39	0.3	0.00531492	0.02497852	0.02051893	0.43420699	A_07_P038279	c_1496	cmr|Unknown protein encoded by prophage [c_1496]
c_1499	+2.00	8.47	+1.00	7.97	0.0	8.97	0.2	0.00520615	0.02454795	0.07251207	0.67194232	A_07_P038295	c_1499	cmr|Putative capsid protein of prophage [c_1499]
c_1501	+2.63	8.87	+1.39	8.17	0.0	9.56	0.1	0.00045064	0.00374599	0.04177083	0.50861562	A_07_P038305	c_1501	cmr|Hypothetical protein [c_1501]
c_1506	-3.10	8.02	-1.63	8.83	0.0	7.20	0.3	0.00125088	0.00819212	0.02758892	0.48101883	A_07_P038330	c_1506	cmr|Unknown protein encoded by prophage [c_1506]
c_1514	-5.32	11.55	-2.41	12.75	0.0	10.34	0.1	0.00002420	0.00053203	0.00514101	0.23125714	A_07_P038334	c_1514	cmr|Hypothetical protein [c_1514]
c_1522	+12.21	9.47	+3.61	7.66	0.4	11.27	0.2	0.00005202	0.00082314	0.00236598	0.14382239	A_07_P038354	c_1522	cmr|Hypothetical protein [c_1522]
c_1523	+6.62	10.62	+2.73	9.26	0.1	11.99	0.2	0.00003677	0.00066014	0.00840447	0.25593810	A_07_P035459	c_1523	cmr|Unknown protein encoded by bacteriophage [c_1523]
c_1524	+5.43	9.34	+2.44	8.12	0.0	10.56	0.6	0.00136265	0.00872024	0.01151486	0.28594637	A_07_P038365	c_1524	cmr|Hypothetical protein [c_1524]
c_1525	+12.04	11.23	+3.59	9.44	0.0	13.03	0.2	0.00000470	0.00023240	0.00246888	0.14449814	A_07_P038369	c_1525	cmr|Hypothetical protein [c_1525]
c_1526	+9.60	9.39	+3.26	7.76	0.1	11.02	0.0	0.00000644	0.00027019	0.00386179	0.18424242	A_07_P038374	c_1526	cmr|Hypothetical protein [c_1526]
c_1527	+5.41	8.86	+2.44	7.65	0.3	10.08	0.1	0.00021694	0.00223672	0.01210048	0.29219325	A_07_P038377	c_1527	cmr|Hypothetical protein [c_1527]
c_1528	+8.51	9.20	+3.09	7.65	0.3	10.74	0.3	0.00008505	0.00116565	0.00509654	0.21397333	A_07_P038381	c_1528	cmr|Hypothetical protein [c_1528]
c_1530	-2.98	9.55	-1.57	10.33	0.0	8.76	0.5	0.00376543	0.01904978	0.02896913	0.48417197	A_07_P038389	c_1530	cmr|Hypothetical protein [c_1530]
c_1531	+11.98	9.85	+3.58	8.06	0.0	11.64	0.3	0.00001444	0.00039421	0.00243839	0.14432331	A_07_P038395	c_1531	cmr|Hypothetical protein [c_1531]
c_1536	-3.01	12.18	-1.59	12.98	0.1	11.38	0.2	0.00055291	0.00435904	0.02944741	0.48699580	A_07_P038406	c_1536	cmr|Putative recombination protein Bet of prophage [c_1536]
c_1538	+2.71	11.39	+1.44	10.67	0.0	12.11	0.1	0.00039838	0.00340501	0.03956428	0.49911859	A_07_P038419	c_1538	cmr|Hypothetical protein [c_1538]
c_1542	-46.74	13.12	-5.55	15.89	0.0	10.35	0.1	0.00000027	0.00009047	0.00000191	0.00375000	A_07_P038431	c_1542	cmr|Lambda ant-restriction protein [c_1542]
c_1544	-2.06	8.49	-1.04	9.01	0.0	7.97	0.1	0.00191942	0.01128006	0.08478341	0.70963849	A_07_P038443	c_1544	cmr|Hypothetical protein [c_1544]
c_1546	-4.07	12.41	-2.02	13.42	0.0	11.39	0.3	0.00023714	0.00238260	0.01175940	0.34998110	A_07_P038451	c_1546	cmr|Repressor protein [c_1546]
c_1547	-2.74	11.09	-1.45	11.82	0.1	10.36	0.2	0.00072865	0.00540616	0.03796811	0.52993794	A_07_P038456	c_1547	cmr|Hypothetical protein [c_1547]
c_1548	-2.75	10.57	-1.46	11.30	0.2	9.84	0.0	0.00065672	0.00499004	0.03844830	0.53427184	A_07_P038465	c_1548	cmr|Putative Regulatory protein CII of bacteriophage [c_1548]
c_1551	-5.23	12.85	-2.39	14.05	0.0	11.66	0.2	0.00005928	0.00090081	0.00560213	0.24568245	A_07_P038478	c_1551	cmr|Putative exclusion protein ren of prophage [c_1551]
c_1554	+6.58	9.29	+2.72	7.93	0.2	10.65	0.0	0.00002259	0.00051403	0.00864329	0.26068966	A_07_P038485	c_1554	cmr|Unknown protein encoded within prophage [c_1554]
c_1555	+3.64	8.28	+1.86	7.35	0.1	9.21	0.6	0.00542090	0.02536304	0.02454967	0.40685263	A_07_P038490	c_1555	cmr|Putative DNA N-6-adenine-methyltransferase of bacteriophage [c_1555]
c_1556	+14.65	9.22	+3.87	7.28	0.2	11.16	0.3	0.00001491	0.00040051	0.00168064	0.12306977	A_07_P038492	c_1556	cmr|Hypothetical protein [c_1556]
c_1565	+5.92	12.80	+2.57	11.51	0.2	14.08	0.0	0.00006106	0.00091644	0.01045986	0.27723906	A_07_P038514	c_1565	cmr|Partial tonB-like membrane protein encoded within prophage [c_1565]
c_1567	+2.96	9.64	+1.56	8.86	0.1	10.42	0.1	0.00026981	0.00258239	0.03385036	0.46790167	A_07_P038518	c_1567	cmr|Hypothetical protein [c_1567]
c_1570	+3.09	11.03	+1.63	10.21	0.4	11.84	0.0	0.00239982	0.01333149	0.03165523	0.45266122	A_07_P038532	c_1570	cmr|Putative DNA packaging protein of prophage [c_1570]
c_1571	+4.79	9.23	+2.26	8.10	0.0	10.36	0.1	0.00005122	0.00081372	0.01508194	0.33117155	A_07_P038538	c_1571	cmr|Putative capsid protein of prophage [c_1571]
c_1572	+25.59	10.19	+4.68	7.86	0.1	12.53	0.3	0.00000363	0.00020691	0.00047256	0.06584071	A_07_P038541	c_1572	cmr|Putative capsid assembly protein of prophage [c_1572]
c_1574	+13.26	10.19	+3.73	8.32	0.1	12.05	0.2	0.00000578	0.00025782	0.00201791	0.13292887	A_07_P038551	c_1574	cmr|Putative capsid protein of prophage [c_1574]
c_1575	+11.58	9.11	+3.53	7.34	0.2	10.87	0.1	0.00000730	0.00028727	0.00268229	0.15028470	A_07_P038556	c_1575	cmr|Putative capsid protein of prophage [c_1575]
c_1576	+8.83	9.22	+3.14	7.65	0.2	10.79	0.2	0.00003621	0.00065813	0.00465257	0.20234807	A_07_P038564	c_1576	cmr|Hypothetical protein [c_1576]
c_1577	+10.65	8.74	+3.41	7.03	0.1	10.44	0.0	0.00000460	0.00023228	0.00320694	0.16663366	A_07_P038569	c_1577	cmr|Putative head-tail joining protein of prophage [c_1577]
c_1579	+4.53	10.64	+2.18	9.55	0.1	11.73	0.8	0.00600228	0.02752691	0.01590574	0.33703903	A_07_P038580	c_1579	cmr|Putative tail component of prophage [c_1579]
c_1580	+24.47	9.33	+4.61	7.03	0.0	11.64	0.2	0.00000181	0.00016633	0.00050051	0.06852174	A_07_P038581	c_1580	cmr|Tail protein [c_1580]
c_1583	+7.22	9.32	+2.85	7.89	0.0	10.75	0.2	0.00002831	0.00057633	0.00705983	0.23903226	A_07_P038594	c_1583	cmr|Putative tail component of prophage [c_1583]
c_1585	+2.85	9.65	+1.51	8.89	0.2	10.40	0.2	0.00117138	0.00778480	0.03625953	0.48093513	A_07_P038605	c_1585	cmr|Putative tail component of prophage [c_1585]
c_1591	+3.06	9.52	+1.61	8.72	0.3	10.33	0.0	0.00121892	0.00802285	0.03228277	0.45789189	A_07_P038637	c_1591	cmr|Hypothetical protein [c_1591]
c_1593	+2.70	7.89	+1.43	7.17	0.2	8.60	0.1	0.00070403	0.00525566	0.03933117	0.49777331	A_07_P038645	c_1593	cmr|Hypothetical protein [c_1593]
c_1596	-2.44	9.06	-1.28	9.70	0.0	8.42	0.2	0.00104283	0.00712961	0.05328697	0.60356115	A_07_P038656	c_1596	cmr|Hypothetical protein [c_1596]
c_1605	-2.27	11.93	-1.18	12.52	0.1	11.34	0.1	0.00140321	0.00890456	0.06478659	0.65051020	A_07_P038687	c_1605	cmr|Hypothetical protein [c_1605]
c_1611	-2.70	8.22	-1.44	8.93	0.3	7.50	0.5	0.00986607	0.04101699	0.04027820	0.54479381	A_07_P038708	c_1611	cmr|Hypothetical protein [c_1611]
c_1640	-2.55	12.17	-1.35	12.85	0.1	11.50	0.3	0.00245507	0.01356706	0.04756606	0.57873261	A_07_P038726	c_1640	cmr|Putative Na [c_1640]
c_1657	+7.68	8.66	+2.94	7.19	0.1	10.13	0.2	0.00002131	0.00049411	0.00622269	0.22783721	A_07_P038777	c_1657	cmr|Protein ycgS [c_1657]
c_1660	-3.13	9.56	-1.64	10.38	0.0	8.74	0.1	0.00018403	0.00199406	0.02622332	0.47292096	A_07_P038788	c_1660	cmr|Hypothetical protein [c_1660]
c_1714	-8.09	10.75	-3.02	12.25	0.1	9.24	0.1	0.00000804	0.00029787	0.00143610	0.12631285	A_07_P038863	c_1714	cmr|Hypothetical protein [c_1714]
c_1752	-2.19	7.90	-1.13	8.47	0.0	7.34	0.1	0.00181297	0.01081601	0.07128493	0.66487559	A_07_P038909	c_1752	cmr|Hypothetical protein [c_1752]
c_1755	+2.57	9.09	+1.36	8.41	0.0	9.77	0.2	0.00077220	0.00564414	0.04302973	0.51283876	A_07_P038912	c_1755	cmr|Conserved hypothetical protein [c_1755]
c_1800	+5.46	9.31	+2.45	8.09	0.1	10.54	0.0	0.00002239	0.00051096	0.01216463	0.29284404	A_07_P034765	c_1800	cmr|Conserved hypothetical protein [c_1800]
c_1812	-3.02	9.58	-1.60	10.38	0.2	8.78	0.2	0.00113219	0.00759093	0.02811611	0.48167573	A_07_P034785	c_1812	cmr|Hypothetical protein [c_1812]
c_1825	+5.36	10.46	+2.42	9.25	0.3	11.67	0.1	0.00010609	0.00135459	0.01245363	0.29573152	A_07_P034801	c_1825	cmr|Hypothetical protein [c_1825]
c_1862	+2.32	7.63	+1.21	7.02	0.1	8.24	0.2	0.00277918	0.01497013	0.05190676	0.56476849	A_07_P042298	c_1862	cmr|Hypothetical protein [c_1862]
c_1889	-2.03	8.26	-1.02	8.77	0.1	7.74	0.2	0.00404219	0.02013275	0.08614266	0.71380526	A_07_P034909	c_1889	cmr|Hypothetical protein [c_1889]
c_1935	-3.60	8.72	-1.85	9.65	0.2	7.80	0.1	0.00024141	0.00240404	0.01743521	0.40427099	A_07_P034987	c_1935	cmr|Chaperone protein fimC precursor [c_1935]
c_1956	-3.65	11.00	-1.87	11.94	0.2	10.07	0.2	0.00059117	0.00460077	0.01602960	0.39248834	A_07_P035043	c_1956	cmr|Putative outer membrane protein yieC precursor [c_1956]
c_1989	-6.56	10.87	-2.71	12.23	0.1	9.51	0.2	0.00003123	0.00061413	0.00284172	0.17545098	A_07_P035078	c_1989	cmr|Putative acid shock protein [c_1989]
c_1994	+3.13	9.44	+1.65	8.62	0.1	10.26	0.0	0.00018037	0.00195982	0.03057482	0.44530065	A_07_P035084	c_1994	cmr|NAD [c_1994]
c_2032	+2.41	10.55	+1.27	9.92	0.0	11.19	0.1	0.00091344	0.00644609	0.04843432	0.54741565	A_07_P035130	c_2032	cmr|Hypothetical protein ydhH [c_2032]
c_2146	+3.25	9.10	+1.70	8.25	0.1	9.95	0.1	0.00017967	0.00195827	0.02862360	0.43880234	A_07_P042367	c_2146	cmr|Succinylglutamic semialdehyde dehydrogenase [c_2146]
c_2148	+5.05	8.40	+2.34	7.24	0.4	9.57	0.0	0.00043455	0.00364142	0.01340701	0.30814599	A_07_P035198	c_2148	cmr|Succinylornithine transaminase [c_2148]
c_2155	+9.37	9.89	+3.23	8.28	0.2	11.51	0.0	0.00001272	0.00037366	0.00421494	0.19179191	A_07_P042371	c_2155	cmr|Protein ynjB [c_2155]
c_2158	+6.86	9.73	+2.78	8.34	0.0	11.12	0.1	0.00001117	0.00034947	0.00792492	0.24854582	A_07_P041171	c_2158	cmr|Putative thiosulfate sulfurtransferase ynjE precursor [c_2158]
c_2225	-2.20	7.59	-1.13	8.16	0.2	7.02	0.1	0.00388351	0.01949807	0.06915015	0.65822249	A_07_P035300	c_2225	cmr|Hypothetical protein [c_2225]
c_2255	-28.72	9.92	-4.84	12.35	0.0	7.50	0.0	0.00000047	0.00009262	0.00001588	0.01136364	A_07_P035318	c_2255	cmr|Hypothetical protein [c_2255]
c_2348	+55.15	10.34	+5.79	7.45	0.4	13.23	0.1	0.00000348	0.00020221	0.00010163	0.02962963	A_07_P035389	c_2348	cmr|Outer membrane porin protein nmpC precursor [c_2348]
c_2393	+3.50	8.20	+1.81	7.30	0.2	9.10	0.2	0.00043122	0.00361892	0.02522040	0.41147150	A_07_P031420	c_2393	cmr|Hypothetical protein [c_2393]
c_2405	+2.59	8.13	+1.37	7.44	0.1	8.81	0.0	0.00047488	0.00388371	0.04227071	0.51036043	A_07_P031475	c_2405	cmr|Hypothetical protein [c_2405]
c_2406	+2.87	8.89	+1.52	8.13	0.0	9.65	0.2	0.00051159	0.00411787	0.03573552	0.47801189	A_07_P031476	c_2406	cmr|Hypothetical protein [c_2406]
c_2412	-2.58	9.43	-1.37	10.12	0.4	8.75	0.1	0.00401865	0.02006011	0.04496380	0.57320648	A_07_P031509	c_2412	cmr|Hypothetical protein [c_2412]
c_2418	-2.32	9.21	-1.22	9.82	0.3	8.60	0.2	0.00541862	0.02535992	0.06039888	0.63184053	A_07_P031531	c_2418	cmr|Prophage P4 integrase [c_2418]
c_2438	-3.34	8.06	-1.74	8.93	0.1	7.19	0.0	0.00014725	0.00169348	0.02129891	0.44064389	A_07_P031623	c_2438	cmr|Hypothetical protein [c_2438]
c_2450	+5.76	12.50	+2.53	11.24	0.0	13.77	0.1	0.00001824	0.00044319	0.01101626	0.28064725	A_07_P031648	c_2450	cmr|Hypothetical protein [c_2450]
c_2451	+14.98	11.70	+3.91	9.75	0.0	13.65	0.1	0.00000175	0.00016311	0.00161141	0.12315534	A_07_P031651	c_2451	cmr|Putative thioesterase [c_2451]
c_2452	+2.63	10.50	+1.39	9.80	0.0	11.20	0.2	0.00080954	0.00583846	0.04161395	0.50788372	A_07_P031658	c_2452	cmr|Hypothetical protein [c_2452]
c_2453	+4.60	8.77	+2.20	7.67	0.1	9.87	0.1	0.00006211	0.00092690	0.01617886	0.33917443	A_07_P031661	c_2453	cmr|Putative polyketide synthase [c_2453]
c_2454	+14.68	9.51	+3.88	7.57	0.0	11.45	0.0	0.00000168	0.00015927	0.00165714	0.12364929	A_07_P031667	c_2454	cmr|Hypothetical protein [c_2454]
c_2456	+3.26	9.68	+1.70	8.83	0.3	10.53	0.2	0.00113023	0.00758172	0.02899517	0.43809981	A_07_P031679	c_2456	cmr|Hypothetical protein [c_2456]
c_2457	+3.18	8.86	+1.67	8.03	0.1	9.70	0.2	0.00051860	0.00415407	0.02978913	0.43996248	A_07_P031685	c_2457	cmr|Putative amidase [c_2457]
c_2459	+7.74	8.92	+2.95	7.44	0.1	10.40	0.1	0.00001367	0.00038700	0.00616870	0.22638695	A_07_P031695	c_2459	cmr|Putative peptide synthetase [c_2459]
c_2460	+50.46	9.37	+5.66	6.54	0.2	12.20	0.1	0.00000083	0.00012615	0.00012068	0.02923077	A_07_P031697	c_2460	cmr|Putative polyketide synthase [c_2460]
c_2461	+30.50	10.13	+4.93	7.67	0.1	12.60	0.1	0.00000062	0.00010566	0.00030932	0.05180851	A_07_P031704	c_2461	cmr|Hypothetical protein [c_2461]
c_2463	+15.96	9.48	+4.00	7.49	0.1	11.48	0.1	0.00000292	0.00018679	0.00135734	0.11130208	A_07_P031708	c_2463	cmr|Putative transacylase [c_2463]
c_2464	+9.72	10.77	+3.28	9.13	0.0	12.41	0.0	0.00000434	0.00022321	0.00380653	0.18383436	A_07_P031712	c_2464	cmr|Putative acyl-coa dehydrogenase [c_2464]
c_2466	+26.52	10.97	+4.73	8.60	0.4	13.33	0.1	0.00000790	0.00029735	0.00043572	0.06293578	A_07_P031725	c_2466	cmr|Hypothetical protein [c_2466]
c_2467	+30.95	11.14	+4.95	8.66	0.1	13.61	0.1	0.00000081	0.00012560	0.00030742	0.05204301	A_07_P031728	c_2467	cmr|Putative 3-hydroxyacyl-CoA dehydrogenase [c_2467]
c_2467	+21.46	11.30	+4.42	9.09	0.4	13.51	0.3	0.00002849	0.00057729	0.00064787	0.07786260	A_07_P031728	c_2467	cmr|Putative 3-hydroxyacyl-CoA dehydrogenase [c_2467]
c_2467	+25.79	11.40	+4.69	9.06	0.3	13.75	0.1	0.00000613	0.00026402	0.00046049	0.06473214	A_07_P031728	c_2467	cmr|Putative 3-hydroxyacyl-CoA dehydrogenase [c_2467]
c_2467	+24.79	11.26	+4.63	8.94	0.3	13.58	0.2	0.00000455	0.00023012	0.00051702	0.06898305	A_07_P031728	c_2467	cmr|Putative 3-hydroxyacyl-CoA dehydrogenase [c_2467]
c_2467	+25.32	11.42	+4.66	9.09	0.4	13.75	0.1	0.00001030	0.00033778	0.00049670	0.06859649	A_07_P031728	c_2467	cmr|Putative 3-hydroxyacyl-CoA dehydrogenase [c_2467]
c_2468	+25.97	9.55	+4.70	7.20	0.1	11.90	0.3	0.00000289	0.00018580	0.00045287	0.06481818	A_07_P031733	c_2468	cmr|Putative polyketide synthase [c_2468]
c_2469	+8.29	10.74	+3.05	9.22	0.1	12.27	0.1	0.00001107	0.00034947	0.00536712	0.21666667	A_07_P031736	c_2469	cmr|Putative polyketide synthase [c_2469]
c_2470	+17.34	10.23	+4.12	8.17	0.2	12.28	0.2	0.00000659	0.00027019	0.00115981	0.10088398	A_07_P031741	c_2470	cmr|Putative peptide/polyketide synthase [c_2470]
c_2471	+5.60	8.00	+2.48	6.76	0.2	9.24	0.3	0.00013972	0.00163451	0.01123476	0.28300800	A_07_P031746	c_2471	cmr|Hypothetical protein [c_2471]
c_2472	+3.47	8.92	+1.80	8.02	0.2	9.82	0.1	0.00018883	0.00202661	0.02543699	0.41286598	A_07_P031751	c_2472	cmr|Transposase [c_2472]
c_2473	+2.45	7.84	+1.29	7.19	0.0	8.48	0.0	0.00054294	0.00430634	0.04690676	0.53944485	A_07_P031756	c_2473	cmr|Transposase [c_2473]
c_2481	-4.98	8.23	-2.32	9.39	0.0	7.07	0.2	0.00007895	0.00110461	0.00649200	0.26207692	A_07_P031773	c_2481	cmr|Hypothetical protein [c_2481]
c_2482	-5.24	8.53	-2.39	9.73	0.1	7.34	0.3	0.00010136	0.00131881	0.00559007	0.24583799	A_07_P031779	c_2482	cmr|Putative outer membrane receptor for iron compound [c_2482]
c_2484	-2.81	12.06	-1.49	12.80	0.0	11.32	0.1	0.00032184	0.00295285	0.03601054	0.51870997	A_07_P031782	c_2484	cmr|Hypothetical protein ybdM [c_2484]
c_2489	-2.73	7.74	-1.45	8.47	0.1	7.02	0.1	0.00057089	0.00446721	0.03865854	0.53577465	A_07_P031799	c_2489	cmr|Putative transferase [c_2489]
c_2492	+2.20	8.13	+1.14	7.56	0.0	8.70	0.2	0.00244404	0.01352513	0.05733676	0.59624174	A_07_P031808	c_2492	cmr|Putative carbohydrate kinase [c_2492]
c_2494	+3.07	7.97	+1.62	7.16	0.2	8.78	0.4	0.00380208	0.01920434	0.03198679	0.45533454	A_07_P031818	c_2494	cmr|Hypothetical protein [c_2494]
c_2496	-2.23	9.99	-1.16	10.57	0.0	9.41	0.2	0.00192176	0.01128543	0.06875508	0.65884358	A_07_P031827	c_2496	cmr|Hypothetical protein [c_2496]
c_2497	-4.03	9.88	-2.01	10.89	0.0	8.87	0.0	0.00005480	0.00084832	0.01203252	0.34759633	A_07_P031834	c_2497	cmr|Transposase [c_2497]
c_2500	-6.56	8.36	-2.71	9.72	0.1	7.01	0.3	0.00007057	0.00101790	0.00277439	0.17472000	A_07_P031846	c_2500	cmr|Hypothetical protein [c_2500]
c_2501	-2.19	8.97	-1.13	9.53	0.0	8.40	0.1	0.00134900	0.00865473	0.07175432	0.66688312	A_07_P031851	c_2501	cmr|Hypothetical protein [c_2501]
c_2512	+3.45	8.60	+1.79	7.70	0.2	9.49	0.0	0.00020897	0.00217593	0.02592353	0.41604485	A_07_P031888	c_2512	cmr|Transposase [c_2512]
c_2514	+10.35	9.43	+3.37	7.74	0.1	11.11	0.0	0.00000380	0.00020999	0.00333524	0.16830128	A_07_P031900	c_2514	cmr|Hypothetical protein [c_2514]
c_2520	+7.81	10.83	+2.97	9.34	0.1	12.31	0.0	0.00000863	0.00030658	0.00607597	0.22561321	A_07_P031907	c_2520	cmr|Conserved hypothetical protein [c_2520]
c_2524	+4.23	9.30	+2.08	8.26	0.2	10.34	0.0	0.00010975	0.00139127	0.01844703	0.36394737	A_07_P031927	c_2524	cmr|Hypothetical protein [c_2524]
c_2525	+3.73	9.04	+1.90	8.08	0.3	9.99	0.4	0.00165317	0.01010384	0.02293509	0.39855408	A_07_P031934	c_2525	cmr|Hypothetical protein [c_2525]
c_2528	-3.69	8.86	-1.88	9.80	0.1	7.91	0.0	0.00008777	0.00118848	0.01586700	0.39093897	A_07_P031949	c_2528	cmr|Hypothetical protein [c_2528]
c_2558	+8.11	11.07	+3.02	9.56	0.2	12.58	0.1	0.00001850	0.00044682	0.00554243	0.21599010	A_07_P031994	c_2558	cmr|Mannose-1-phosphate guanylyltransferase [c_2558]
c_2559	+3.05	11.73	+1.61	10.93	0.2	12.54	0.1	0.00069643	0.00521681	0.03221735	0.45778881	A_07_P031999	c_2559	cmr|Hypothetical protein [c_2559]
c_2561	+2.44	13.72	+1.28	13.08	0.1	14.37	0.2	0.00133706	0.00860965	0.04709731	0.54045190	A_07_P032006	c_2561	cmr|Hypothetical protein [c_2561]
c_2564	+2.97	11.42	+1.57	10.64	0.3	12.21	0.1	0.00167306	0.01020163	0.03380971	0.46775044	A_07_P032024	c_2564	cmr|Hypothetical protein [c_2564]
c_2662	-2.38	8.30	-1.25	8.93	0.1	7.68	0.2	0.00236909	0.01320315	0.05712335	0.61810997	A_07_P032204	c_2662	cmr|Hypothetical protein [c_2662]
c_2670	-3.16	9.28	-1.66	10.11	0.2	8.45	0.4	0.00277032	0.01494722	0.02435404	0.46030012	A_07_P032229	c_2670	cmr|Putative conserved protein [c_2670]
c_2727	-2.11	10.70	-1.08	11.24	0.2	10.16	0.2	0.00442791	0.02157630	0.07836065	0.68236173	A_07_P032290	c_2727	cmr|Hypothetical protein [c_2727]
c_2729	-4.50	8.55	-2.17	9.63	0.1	7.46	0.1	0.00004928	0.00079419	0.00885417	0.30304348	A_07_P032291	c_2729	cmr|Hypothetical protein [c_2729]
c_2895	-2.46	10.78	-1.30	11.43	0.0	10.13	0.3	0.00199551	0.01160276	0.05294906	0.60320550	A_07_P032532	c_2895	cmr|yapH homolog [c_2895]
c_2897	+2.71	7.60	+1.44	6.88	0.2	8.32	0.0	0.00084862	0.00608409	0.03888021	0.49565182	A_07_P032543	c_2897	cmr|Type 1 fimbriae Regulatory protein fimB [c_2897]
c_2911	-2.27	8.01	-1.18	8.60	0.0	7.42	0.3	0.00417344	0.02059106	0.06273882	0.64265452	A_07_P042574	c_2911	cmr|YfdX protein precursor [c_2911]
c_3030	-2.83	8.08	-1.50	8.83	0.1	7.33	0.0	0.00041400	0.00351373	0.03506161	0.51493470	A_07_P030168	c_3030	cmr|SinI homolog [c_3030]
c_3031	-6.08	8.69	-2.60	9.99	0.0	7.39	0.3	0.00007802	0.00109698	0.00352325	0.19810714	A_07_P030171	c_3031	cmr|SinH homolog [c_3031]
c_3033	-2.37	9.02	-1.25	9.64	0.2	8.40	0.2	0.00326618	0.01703302	0.05623793	0.61744073	A_07_P030181	c_3033	cmr|Probable GTP-binding protein engA [c_3033]
c_3053	-10.67	11.48	-3.42	13.19	0.2	9.77	0.0	0.00000668	0.00027038	0.00058943	0.07864407	A_07_P041244	c_3053	cmr|Protein yfhF [c_3053]
c_3064	-2.45	7.53	-1.29	8.18	0.1	6.88	0.1	0.00064646	0.00492163	0.05307482	0.60245854	A_07_P030207	c_3064	cmr|Hypothetical protein [c_3064]
c_3116	-6.88	10.64	-2.78	12.03	0.0	9.25	0.0	0.00001018	0.00033608	0.00244284	0.16577586	A_07_P030232	c_3116	cmr|Ribosomal large subunit pseudouridine synthase D [c_3116]
c_3140	-3.17	11.61	-1.66	12.44	0.1	10.78	0.1	0.00019405	0.00206432	0.02540777	0.46731308	A_07_P030260	c_3140	cmr|Protein yfjF [c_3140]
c_3156	+2.09	8.20	+1.07	7.67	0.1	8.73	0.1	0.00232649	0.01305298	0.06373920	0.63153556	A_07_P030311	c_3156	cmr|Putative tail fiber component K of prophage [c_3156]
c_3158	+4.06	8.05	+2.02	7.04	0.1	9.06	0.1	0.00009095	0.00121866	0.01980882	0.37665459	A_07_P030320	c_3158	cmr|Putative tail component of prophage [c_3158]
c_3159	+2.78	10.13	+1.47	9.39	0.5	10.86	0.2	0.00750758	0.03297971	0.03894754	0.49530695	A_07_P030328	c_3159	cmr|Putative tail component of prophage [c_3159]
c_3161	+4.98	8.06	+2.32	6.90	0.2	9.21	0.2	0.00010369	0.00133527	0.01410061	0.32034632	A_07_P030336	c_3161	cmr|Putative tail component of prophage [c_3161]
c_3163	+6.16	8.25	+2.62	6.94	0.2	9.56	0.3	0.00017135	0.00190249	0.00954586	0.26694494	A_07_P030350	c_3163	cmr|Putative tail component of prophage [c_3163]
c_3164	+19.65	9.13	+4.30	6.98	0.1	11.27	0.0	0.00000131	0.00014643	0.00081555	0.08503311	A_07_P030353	c_3164	cmr|Putative tail component of prophage [c_3164]
c_3165	+3.94	8.97	+1.98	7.98	0.6	9.96	0.0	0.00446113	0.02168450	0.02103659	0.38377752	A_07_P030358	c_3165	cmr|Putative tail fiber component Z of prophage [c_3165]
c_3176	+40.61	12.34	+5.34	9.67	0.1	15.01	0.0	0.00000042	0.00009262	0.00018102	0.03750000	A_07_P030411	c_3176	cmr|GnsB protein [c_3176]
c_3225	+3.18	8.62	+1.67	7.78	0.0	9.45	0.1	0.00017839	0.00195041	0.02962462	0.43918079	A_07_P030542	c_3225	cmr|Conserved hypothetical protein [c_3225]
c_3242	-6.61	8.34	-2.73	9.71	0.1	6.98	0.2	0.00004126	0.00070995	0.00273183	0.17627049	A_07_P030574	c_3242	cmr|Hypothetical protein [c_3242]
c_3243	-3.18	8.77	-1.67	9.60	0.1	7.94	0.3	0.00126359	0.00826163	0.02544715	0.46694639	A_07_P030576	c_3243	cmr|Hypothetical protein [c_3243]
c_3252	-32.89	10.81	-5.04	13.33	0.0	8.29	0.0	0.00000039	0.00009117	0.00000889	0.00777778	A_07_P041659	c_3252	cmr|Regulatory protein recX [c_3252]
c_3267	-2.04	7.39	-1.03	7.90	0.1	6.87	0.1	0.00260058	0.01422638	0.08631796	0.71375525	A_07_P030615	c_3267	cmr|FIRd-NAD [c_3267]
c_3270	-3.95	8.27	-1.98	9.26	0.0	7.28	0.2	0.00011020	0.00139359	0.01313453	0.36278947	A_07_P030625	c_3270	cmr|Hypothetical protein [c_3270]
c_3273	-13.58	12.17	-3.76	14.05	0.1	10.28	0.0	0.00000215	0.00017840	0.00022929	0.05013889	A_07_P030635	c_3273	cmr|Hypothetical protein ygjM [c_3273]
c_3274	-6.52	11.87	-2.71	13.22	0.0	10.52	0.2	0.00002597	0.00055176	0.00289571	0.17602317	A_07_P030642	c_3274	cmr|Hypothetical protein [c_3274]
c_3276	-2.37	8.30	-1.25	8.92	0.0	7.67	0.0	0.00067660	0.00510906	0.05800749	0.62254260	A_07_P030650	c_3276	cmr|Hypothetical protein [c_3276]
c_3292	+3.10	11.39	+1.63	10.58	0.3	12.21	0.0	0.00068324	0.00513948	0.03122142	0.45137741	A_07_P030663	c_3292	cmr|Potential molybdenum-pterin-binding-protein [c_3292]
c_3305	-2.33	7.90	-1.22	8.51	0.1	7.29	0.1	0.00100954	0.00695281	0.06085302	0.63406353	A_07_P030690	c_3305	cmr|Hypothetical protein [c_3305]
c_3307	+2.36	15.54	+1.24	14.92	0.1	16.15	0.2	0.00215910	0.01229844	0.05022040	0.55563598	A_07_P030691	c_3307	cmr|Putative conserved protein [c_3307]
c_3309	-3.16	10.53	-1.66	11.36	0.2	9.70	0.3	0.00131419	0.00848323	0.02621253	0.47326835	A_07_P030697	c_3309	cmr|Hypothetical protein [c_3309]
c_3379	-2.46	9.39	-1.30	10.04	0.1	8.74	0.3	0.00260693	0.01425120	0.05283854	0.60369376	A_07_P032655	c_3379	cmr|Hypothetical protein [c_3379]
c_3391	+2.17	7.80	+1.12	7.24	0.3	8.36	0.0	0.00695655	0.03101781	0.06133765	0.62182872	A_07_P032689	c_3391	cmr|Secreted protein Hcp [c_3391]
c_3391	+2.10	7.74	+1.07	7.20	0.0	8.28	0.2	0.00276527	0.01492508	0.06191946	0.62411012	A_07_P032689	c_3391	cmr|Secreted protein Hcp [c_3391]
c_3393	+2.43	7.69	+1.28	7.05	0.0	8.33	0.0	0.00058268	0.00454596	0.04727388	0.54050835	A_07_P032702	c_3393	cmr|Hypothetical protein [c_3393]
c_3394	-3.78	8.41	-1.92	9.37	0.1	7.45	0.0	0.00011482	0.00142792	0.01462525	0.38185738	A_07_P032706	c_3394	cmr|Hypothetical protein [c_3394]
c_3396	-4.29	8.77	-2.10	9.82	0.0	7.72	0.3	0.00026267	0.00255436	0.01002350	0.31625251	A_07_P032716	c_3396	cmr|Hypothetical protein [c_3396]
c_3397	-2.93	7.72	-1.55	8.49	0.0	6.94	0.1	0.00026044	0.00254204	0.03154027	0.49262897	A_07_P032722	c_3397	cmr|Hypothetical protein [c_3397]
c_3432	-3.58	11.21	-1.84	12.13	0.1	10.29	0.3	0.00076390	0.00559389	0.01687436	0.39711510	A_07_P032816	c_3432	cmr|Transposase [c_3432]
c_3506	+5.97	10.58	+2.58	9.29	0.1	11.86	0.1	0.00003130	0.00061413	0.01032393	0.27642857	A_07_P032900	c_3506	cmr|Hypothetical protein [c_3506]
c_3507	+14.42	9.41	+3.85	7.48	0.3	11.33	0.1	0.00000866	0.00030685	0.00175749	0.12577273	A_07_P032905	c_3507	cmr|Hypothetical protein [c_3507]
c_3508	+7.91	9.27	+2.98	7.78	0.0	10.76	0.1	0.00000763	0.00029300	0.00585112	0.22251208	A_07_P032907	c_3508	cmr|Hypothetical protein [c_3508]
c_3509	+3.36	7.65	+1.75	6.77	0.0	8.52	0.1	0.00012914	0.00154965	0.02719766	0.42396040	A_07_P032913	c_3509	cmr|Putative ATP-binding protein of ABC transport system [c_3509]
c_3513	-5.29	12.09	-2.40	13.29	0.1	10.89	0.2	0.00008948	0.00120520	0.00543763	0.24115493	A_07_P032930	c_3513	cmr|Putative conserved protein [c_3513]
c_3518	-3.57	8.51	-1.84	9.42	0.1	7.59	0.0	0.00010212	0.00132269	0.01728786	0.40263314	A_07_P032938	c_3518	cmr|Hypothetical protein [c_3518]
c_3557	-2.74	9.73	-1.45	10.46	0.1	9.01	0.0	0.00034744	0.00310252	0.03906822	0.53672775	A_07_P032997	c_3557	cmr|ShiA homolog [c_3557]
c_3560	-4.66	8.06	-2.22	9.17	0.1	6.95	0.0	0.00005378	0.00084214	0.00798971	0.29050808	A_07_P033008	c_3560	cmr|Unknown protein encoded by ISEc8 within prophage [c_3560]
c_3565	-2.57	10.75	-1.36	11.43	0.2	10.07	0.1	0.00129590	0.00841002	0.04617442	0.57377269	A_07_P033031	c_3565	cmr|Putative response regulator [c_3565]
c_3568	+3.58	11.10	+1.84	10.18	0.1	12.03	0.1	0.00014069	0.00164076	0.02419207	0.40390244	A_07_P033050	c_3568	cmr|Hypothetical protein [c_3568]
c_3579	-3.18	10.49	-1.67	11.32	0.0	9.66	0.0	0.00014151	0.00164696	0.02509782	0.46432432	A_07_P033108	c_3579	cmr|Hypothetical protein [c_3579]
c_3580	-11.33	11.53	-3.50	13.28	0.0	9.78	0.1	0.00000397	0.00021396	0.00051575	0.07381818	A_07_P033102	c_3580	cmr|Hypothetical protein [c_3580]
c_3581	-2.36	12.16	-1.24	12.78	0.0	11.54	0.1	0.00102187	0.00702132	0.05884718	0.62600676	A_07_P033112	c_3581	cmr|Hypothetical protein [c_3581]
c_3594	-13.22	10.78	-3.72	12.64	0.1	8.92	0.2	0.00000773	0.00029593	0.00025915	0.05164557	A_07_P033172	c_3594	cmr|Putative Transposase [c_3594]
c_3595	-8.30	10.67	-3.05	12.19	0.0	9.14	0.1	0.00000939	0.00032230	0.00134464	0.11960452	A_07_P033176	c_3595	cmr|Transposase [c_3595]
c_3596	-7.00	11.56	-2.81	12.96	0.1	10.16	0.2	0.00001808	0.00044140	0.00224212	0.15829596	A_07_P033185	c_3596	cmr|Hypothetical protein in IS [c_3596]
c_3597	-6.59	9.79	-2.72	11.15	0.0	8.43	0.1	0.00001181	0.00035886	0.00275089	0.17605691	A_07_P033190	c_3597	cmr|Transposase [c_3597]
c_3598	-5.23	9.39	-2.39	10.58	0.0	8.19	0.1	0.00002719	0.00056519	0.00561547	0.24558333	A_07_P033191	c_3598	cmr|Hypothetical protein [c_3598]
c_3598	-6.95	9.52	-2.80	10.92	0.1	8.12	0.0	0.00001139	0.00035149	0.00236280	0.16173913	A_07_P033191	c_3598	cmr|Hypothetical protein [c_3598]
c_3598	-9.22	9.22	-3.20	10.82	0.1	7.62	0.4	0.00009726	0.00128241	0.00092543	0.09979452	A_07_P033191	c_3598	cmr|Hypothetical protein [c_3598]
c_3598	-8.98	9.36	-3.17	10.94	0.0	7.77	0.2	0.00001118	0.00034947	0.00107533	0.10450617	A_07_P033191	c_3598	cmr|Hypothetical protein [c_3598]
c_3598	-9.81	9.17	-3.29	10.81	0.1	7.52	0.1	0.00000732	0.00028727	0.00078379	0.09073529	A_07_P033191	c_3598	cmr|Hypothetical protein [c_3598]
c_3601	-8.40	9.71	-3.07	11.25	0.1	8.18	0.2	0.00001220	0.00036519	0.00128620	0.11505682	A_07_P033207	c_3601	cmr|Hypothetical protein [c_3601]
c_3602	-4.06	11.93	-2.02	12.94	0.2	10.92	0.1	0.00016750	0.00187425	0.01169715	0.34944972	A_07_P033212	c_3602	cmr|Hypothetical protein [c_3602]
c_3604	-2.80	8.21	-1.48	8.95	0.0	7.46	0.1	0.00038054	0.00329914	0.03635099	0.52122951	A_07_P033222	c_3604	cmr|Hypothetical protein [c_3604]
c_3605	+4.18	11.05	+2.06	10.02	0.1	12.08	0.1	0.00008779	0.00118848	0.01880272	0.36728288	A_07_P033227	c_3605	cmr|Hypothetical protein [c_3605]
c_3606	+9.33	10.34	+3.22	8.73	0.1	11.95	0.3	0.00002946	0.00059394	0.00421939	0.19144092	A_07_P033231	c_3606	cmr|Hypothetical protein [c_3606]
c_3609	-3.00	8.74	-1.59	9.53	0.1	7.95	0.1	0.00032229	0.00295389	0.02964876	0.48776385	A_07_P033238	c_3609	cmr|Hypothetical protein [c_3609]
c_3612	-3.00	10.77	-1.59	11.56	0.2	9.97	0.4	0.00226157	0.01276227	0.02848514	0.48326509	A_07_P033247	c_3612	cmr|Transposase insC for insertion element IS2A/D/F/H/I/K [c_3612]
c_3614	-2.64	9.58	-1.40	10.28	0.1	8.88	0.1	0.00068910	0.00517863	0.04271405	0.56275314	A_07_P033257	c_3614	cmr|Hypothetical protein [c_3614]
c_3616	-2.72	10.98	-1.44	11.70	0.1	10.25	0.3	0.00221397	0.01256099	0.04044271	0.54608062	A_07_P033266	c_3616	cmr|Unknown in ISEc8 [c_3616]
c_3617	-2.44	8.35	-1.28	8.99	0.1	7.71	0.1	0.00090903	0.00642362	0.05361153	0.60506093	A_07_P036281	c_3617	cmr|Unknown in putative ISEc8 [c_3617]
c_3618	-9.05	9.60	-3.18	11.19	0.1	8.01	0.0	0.00000565	0.00025427	0.00102261	0.10320513	A_07_P036289	c_3618	cmr|Hypothetical protein [c_3618]
c_3618	-9.88	9.63	-3.30	11.29	0.1	7.98	0.0	0.00000530	0.00024923	0.00078379	0.09140741	A_07_P036289	c_3618	cmr|Hypothetical protein [c_3618]
c_3618	-7.69	9.38	-2.94	10.86	0.1	7.91	0.1	0.00000957	0.00032388	0.00171049	0.13465000	A_07_P036289	c_3618	cmr|Hypothetical protein [c_3618]
c_3618	-10.08	9.70	-3.33	11.37	0.1	8.04	0.1	0.00000467	0.00023240	0.00075140	0.08894737	A_07_P036289	c_3618	cmr|Hypothetical protein [c_3618]
c_3618	-8.77	9.29	-3.13	10.86	0.0	7.72	0.2	0.00001349	0.00038499	0.00115218	0.10927711	A_07_P036289	c_3618	cmr|Hypothetical protein [c_3618]
c_3620	-2.13	11.41	-1.09	11.95	0.1	10.86	0.0	0.00131028	0.00847887	0.07744601	0.67777098	A_07_P036293	c_3620	cmr|Hypothetical protein [c_3620]
c_3630	-3.52	9.44	-1.81	10.35	0.1	8.53	0.2	0.00027920	0.00265270	0.01804116	0.40987013	A_07_P036335	c_3630	cmr|Hypothetical protein yjhS precursor [c_3630]
c_3631	-2.94	9.47	-1.56	10.25	0.1	7.78	0.2	0.00060322	0.00466690	0.03109947	0.49159639	A_07_P036340	c_3631	cmr|Hypothetical protein [c_3631]
c_3634	+5.90	10.53	+2.56	9.25	0.0	11.81	0.1	0.00001572	0.00043046	0.01058054	0.27625207	A_07_P036349	c_3634	cmr|Hypothetical protein yjhT precursor [c_3634]
c_3635	+4.99	10.40	+2.32	9.24	0.2	11.56	0.1	0.00005382	0.00084214	0.01415206	0.31921203	A_07_P036354	c_3635	cmr|Hypothetical protein yjhA precursor [c_3635]
c_3636	+13.18	9.42	+3.72	7.56	0.0	11.28	0.1	0.00000240	0.00017963	0.00208968	0.13428571	A_07_P036358	c_3636	cmr|Hypothetical protein [c_3636]
c_3637	+3.01	8.74	+1.59	7.95	0.1	9.53	0.1	0.00113957	0.00762488	0.03302401	0.46339572	A_07_P036362	c_3637	cmr|Putative sialic acid transporter [c_3637]
c_3638	+7.32	9.37	+2.87	7.93	0.1	10.81	0.1	0.00001503	0.00040227	0.00682736	0.23520788	A_07_P036368	c_3638	cmr|Putative protein yhcl [c_3638]
c_3639	+11.92	9.91	+3.58	8.12	0.0	11.70	0.0	0.00000246	0.00017989	0.00253874	0.14429603	A_07_P036375	c_3639	cmr|N-acetylneuraminate lyase subunit [c_3639]
c_3641	-5.36	9.18	-2.42	10.39	0.2	7.97	0.1	0.00004881	0.00078969	0.00761006	0.22945245	A_07_P036380	c_3641	cmr|Unknown in ISEc8 [c_3641]
c_3646	-2.60	12.53	-1.38	13.21	0.3	11.84	0.3	0.00509020	0.02715543	0.04327172	0.56396523	A_07_P036409	c_3646	cmr|Hypothetical protein [c_3646]
c_3654	-3.23	8.28	-1.69	9.13	0.0	7.43	0.2	0.00047330	0.00387677	0.02326410	0.45107143	A_07_P036449	c_3654	cmr|Hypothetical protein yeeP [c_3654]

c_3655	+9.86	11.91	+3.30	10.26	0.1	13.56	0.1	0.00000621	0.00026588	0.00369538	0.18124611	A_07_P036451	c_3655	cmr|Antigen 43 precursor [c_3655]
c_3656	+2.13	7.87	+1.09	7.33	0.1	8.42	0.2	0.00418264	0.02061078	0.06413237	0.63343789	A_07_P036460	c_3656	cmr|Hypothetical protein [c_3656]
c_3658	-35.95	14.03	-5.17	16.62	0.0	11.45	0.0	0.00000035	0.00009117	0.00000572	0.00750000	A_07_P036466	c_3658	cmr|Hypothetical protein [c_3658]
c_3659	-49.34	9.69	-5.62	12.50	0.0	6.88	0.2	0.00000040	0.00009146	0.00000191	0.00500000	A_07_P036474	c_3659	cmr|Unknown protein encoded by ISEc8 [c_3659]
c_3660	-48.03	9.74	-5.59	12.53	0.0	6.95	0.1	0.00000027	0.00009047	0.00000127	0.00400000	A_07_P036480	c_3660	cmr|Unknown protein encoded by ISEc8 [c_3660]
c_3662	-2.37	11.71	-1.24	12.33	0.1	11.09	0.1	0.00089006	0.00630937	0.05808816	0.62255956	A_07_P036488	c_3662	cmr|Unknown protein encoded by ISEc8 [c_3662]
c_3663	-4.87	8.67	-2.28	9.82	0.1	7.53	0.2	0.00007089	0.00101926	0.00687119	0.26977556	A_07_P036493	c_3663	cmr|Hypothetical protein [c_3663]
c_3665	+2.26	11.30	+1.18	10.72	0.0	11.89	0.1	0.00123504	0.00811203	0.05436992	0.57955315	A_07_P036501	c_3665	cmr|Hypothetical protein [c_3665]
c_3666	-2.28	8.08	-1.19	8.68	0.0	7.49	0.1	0.00104263	0.00712961	0.06523056	0.65288620	A_07_P036509	c_3666	cmr|Hypothetical protein ykfF [c_3666]
c_3668	-2.61	8.97	-1.39	9.66	0.1	8.27	0.2	0.00136309	0.00872024	0.04452236	0.56988618	A_07_P036519	c_3668	cmr|Hypothetical protein yafZ [c_3668]
c_3670	-4.73	8.85	-2.24	9.97	0.0	7.73	0.2	0.00006553	0.00096786	0.00754700	0.28425837	A_07_P036530	c_3670	cmr|Hypothetical protein [c_3670]
c_3672	-3.03	9.12	-1.60	9.92	0.1	8.32	0.4	0.00204863	0.01184927	0.02919461	0.48485232	A_07_P036541	c_3672	cmr|Hypothetical protein [c_3672]
c_3675	-2.76	9.63	-1.47	10.36	0.0	8.89	0.2	0.00068017	0.00512982	0.03792492	0.53074667	A_07_P036560	c_3675	cmr|Hypothetical protein [c_3675]
c_3678	-2.10	8.72	-1.07	9.26	0.1	8.19	0.2	0.00408374	0.02028854	0.07766641	0.67894503	A_07_P036567	c_3678	cmr|Conserved hypothetical protein [c_3678]
c_3681	-10.84	11.97	-3.44	13.68	0.0	10.25	0.1	0.00000557	0.00025342	0.00055069	0.07672566	A_07_P036583	c_3681	cmr|Hypothetical protein [c_3681]
c_3682	-9.50	11.16	-3.25	12.78	0.0	9.54	0.2	0.00001168	0.00035700	0.00087970	0.09822695	A_07_P036586	c_3682	cmr|Hypothetical protein [c_3682]
c_3683	-34.22	12.32	-5.10	14.86	0.0	9.77	0.0	0.00000039	0.00009117	0.00000635	0.00666667	A_07_P036593	c_3683	cmr|Hypothetical protein [c_3683]
c_3686	+16.66	10.71	+4.06	8.68	0.1	12.74	0.1	0.00000274	0.00018406	0.00126524	0.10539683	A_07_P036604	c_3686	cmr|Hypothetical protein yrbH [c_3686]
c_3689	+16.21	10.05	+4.02	8.04	0.1	12.05	0.0	0.00000198	0.00017252	0.00132940	0.10958115	A_07_P036619	c_3689	cmr|3-deoxy-manno-octulosonate cytidylyltransferase [c_3689]
c_3693	+7.73	9.61	+2.95	8.14	0.2	11.09	0.2	0.00005910	0.00089898	0.00613758	0.22577103	A_07_P036636	c_3693	cmr|Hypothetical protein [c_3693]
c_3693	+9.97	10.32	+3.32	8.66	0.3	11.98	0.0	0.00001779	0.00043523	0.00373666	0.18270186	A_07_P036636	c_3693	cmr|Hypothetical protein [c_3693]
c_3693	+7.13	9.84	+2.83	8.42	0.0	11.25	0.5	0.00032740	0.00298128	0.00706047	0.23854077	A_07_P036636	c_3693	cmr|Hypothetical protein [c_3693]
c_3693	+10.44	10.10	+3.38	8.41	0.1	11.79	0.2	0.00000846	0.00030484	0.00330221	0.16770968	A_07_P036636	c_3693	cmr|Hypothetical protein [c_3693]
c_3693	+10.46	10.13	+3.39	8.44	0.1	11.83	0.1	0.00000741	0.00028894	0.00329205	0.16827922	A_07_P036636	c_3693	cmr|Hypothetical protein [c_3693]
c_3694	+6.33	11.98	+2.66	10.64	0.0	13.31	0.1	0.00001670	0.00042622	0.00936865	0.26867031	A_07_P036641	c_3694	cmr|Hypothetical protein [c_3694]
c_3695	+7.63	10.41	+2.93	8.95	0.2	11.88	0.1	0.00002591	0.00055176	0.00623412	0.22772622	A_07_P036649	c_3695	cmr|Hypothetical protein [c_3695]
c_3696	+2.46	13.22	+1.30	12.57	0.1	13.87	0.2	0.00189270	0.01116147	0.04656059	0.53782098	A_07_P036652	c_3696	cmr|Putative glycerol-3-phosphate cytidyltransferase [c_3696]
c_3751	+2.12	8.03	+1.09	7.49	0.2	8.57	0.2	0.00621278	0.02826178	0.06468496	0.63650000	A_07_P036738	c_3751	cmr|Hypothetical oxidoreductase ydfI [c_3751]
c_3771	-2.25	9.35	-1.17	9.94	0.1	8.77	0.1	0.00153200	0.00948854	0.06661077	0.65585991	A_07_P036801	c_3771	cmr|Putative iron compound-binding protein of ABC transporter [c_3771]
c_3774	-2.08	7.98	-1.06	8.51	0.0	7.45	0.0	0.00147564	0.00923022	0.08155678	0.69670646	A_07_P036820	c_3774	cmr|Ferric enterobactin transport ATP-binding protein fepC [c_3774]
c_3819	-8.69	14.74	-3.12	16.30	0.0	13.18	0.1	0.00000629	0.00026783	0.00116235	0.10958084	A_07_P036906	c_3819	cmr|Putative conserved protein [c_3819]
c_3873	+324.02	11.50	+8.34	7.33	0.1	15.67	0.5	0.00000088	0.00012806	0.00000064	0.00125000	A_07_P034083	c_3873	cmr|Putative conserved protein [c_3873]
c_3890	+9.08	9.50	+3.18	7.90	0.7	11.09	0.1	0.00078566	0.00571910	0.00435531	0.19424929	A_07_P034096	c_3890	cmr|PTS system, N-acetylgalactosamine-specific IID component [c_3890]
c_3891	+7.92	8.92	+2.99	7.43	0.3	10.41	0.0	0.00005046	0.00080580	0.00581872	0.22235437	A_07_P034105	c_3891	cmr|Putative phosphotransferase system enzyme subunit [c_3891]
c_3928	+3.54	7.81	+1.82	6.90	0.0	8.73	0.0	0.00009181	0.00122709	0.02478214	0.40941238	A_07_P034139	c_3928	cmr|Hypothetical protein [c_3928]
c_3976	+5.82	8.24	+2.54	6.97	0.1	9.51	0.2	0.00005425	0.00084354	0.01077236	0.27757774	A_07_P041300	c_3976	cmr|Hypothetical protein yhcI [c_3976]
c_4010	+2.45	9.37	+1.29	8.73	0.1	10.02	0.1	0.00092905	0.00652407	0.04700965	0.54023358	A_07_P034196	c_4010	cmr|Hypothetical protein [c_4010]
c_4014	-6.97	8.82	-2.80	10.22	0.1	7.42	0.2	0.00003540	0.00065256	0.00227579	0.15784141	A_07_P034207	c_4014	cmr|Hypothetical protein [c_4014]
c_4015	-2.24	8.52	-1.17	9.11	0.1	7.94	0.2	0.00188868	0.01115353	0.06829713	0.65927039	A_07_P034215	c_4015	cmr|Ribose transport system permease protein rbsC [c_4015]
c_4016	-5.16	8.70	-2.37	9.88	0.0	7.51	0.1	0.00002589	0.00055176	0.00570185	0.24527322	A_07_P034216	c_4016	cmr|Ribose transport ATP-binding protein rbsA [c_4016]
c_4020	+2.11	8.71	+1.08	8.17	0.1	9.25	0.1	0.00204066	0.01182053	0.06303290	0.62690461	A_07_P034233	c_4020	cmr|Hypothetical protein [c_4020]
c_4021	+3.28	8.60	+1.71	7.75	0.3	9.46	0.0	0.00061930	0.00474925	0.02862614	0.43841440	A_07_P034237	c_4021	cmr|Hypothetical protein [c_4021]
c_4021	+3.13	8.54	+1.65	7.72	0.0	9.37	0.2	0.00033802	0.00304380	0.03063516	0.44576710	A_07_P034237	c_4021	cmr|Hypothetical protein [c_4021]
c_4021	+3.18	8.71	+1.67	7.88	0.1	9.55	0.0	0.00016769	0.00187507	0.02943534	0.43927014	A_07_P034237	c_4021	cmr|Hypothetical protein [c_4021]
c_4021	+2.03	8.30	+1.02	7.79	0.1	8.81	0.1	0.00340795	0.01756292	0.06821329	0.65365186	A_07_P034237	c_4021	cmr|Hypothetical protein [c_4021]
c_4053	-2.94	11.10	-1.55	11.88	0.0	10.32	0.3	0.00083611	0.00600532	0.03098895	0.49281818	A_07_P041813	c_4053	cmr|Zn [c_4053]
c_4064	-2.59	8.63	-1.37	9.31	0.0	7.94	0.1	0.00048021	0.00391124	0.04570185	0.57241846	A_07_P034279	c_4064	cmr|Hypothetical protein [c_4064]
c_4074	-2.69	7.30	-1.43	8.02	0.1	6.59	0.1	0.00041062	0.00349068	0.04127858	0.55356899	A_07_P034295	c_4074	cmr|Hypothetical protein [c_4074]
c_4088	-2.24	8.82	-1.16	9.40	0.0	8.24	0.2	0.00299383	0.01590245	0.06792175	0.65887862	A_07_P034318	c_4088	cmr|Hypothetical protein [c_4088]
c_4206	-2.24	7.89	-1.16	8.48	0.3	7.31	0.1	0.00407831	0.02026799	0.06804433	0.65966133	A_07_P034456	c_4206	cmr|Hypothetical protein [c_4206]
c_4222	+3.07	10.80	+1.62	9.99	0.1	11.61	0.0	0.00022971	0.00234082	0.03159743	0.45306922	A_07_P034505	c_4222	cmr|Putative DNA processing protein [c_4222]
c_4223	+7.09	9.33	+2.83	7.91	0.0	10.74	0.0	0.00000925	0.00032221	0.00742060	0.24492662	A_07_P034506	c_4223	cmr|Putative conserved protein [c_4223]
c_4276	+2.02	10.50	+1.01	9.99	0.1	11.00	0.2	0.00531005	0.02496310	0.07056657	0.66209774	A_07_P034580	c_4276	cmr|Putative regulator [c_4276]
c_4277	+3.52	8.18	+1.82	7.27	0.3	9.09	0.2	0.00110803	0.00746025	0.02546176	0.41241770	A_07_P034581	c_4277	cmr|Putative phosphotransferase system enzyme subunit [c_4277]
c_4278	+4.52	8.97	+2.18	7.88	0.3	10.06	0.4	0.00114921	0.00766985	0.01655742	0.34300000	A_07_P034587	c_4278	cmr|Putative phosphotransferase system enzyme subunit [c_4278]
c_4279	+3.08	8.00	+1.62	7.18	0.1	8.81	0.1	0.00030374	0.00282296	0.03143293	0.45153285	A_07_P034593	c_4279	cmr|PTS system, galactitol-specific IIC component [c_4279]
c_4284	-2.20	10.66	-1.14	11.23	0.1	10.09	0.3	0.00576423	0.02661871	0.07108676	0.66539239	A_07_P034612	c_4284	cmr|Putative aldolase [c_4284]
c_4303	-2.14	7.65	-1.09	8.19	0.2	7.10	0.0	0.00301335	0.01597377	0.07452744	0.67125858	A_07_P034649	c_4303	cmr|Putative conserved protein [c_4303]
c_4352	-2.91	7.66	-1.54	8.43	0.0	6.89	0.1	0.00033631	0.00303844	0.03227261	0.49619141	A_07_P039574	c_4352	cmr|Hypothetical protein [c_4352]
c_4360	-4.49	9.08	-2.17	10.16	0.1	8.00	0.0	0.00004388	0.00074045	0.00879827	0.30178649	A_07_P039592	c_4360	cmr|Hypothetical protein [c_4360]
c_4374	-2.01	8.95	-1.01	9.45	0.3	8.44	0.1	0.00712095	0.03161655	0.09059134	0.72399492	A_07_P039629	c_4374	cmr|Hypothetical protein [c_4374]
c_4391	-2.02	8.34	-1.02	8.85	0.0	7.83	0.1	0.00193240	0.01132675	0.08835429	0.72075130	A_07_P039642	c_4391	cmr|Hypothetical protein [c_4391]
c_4422	-2.96	12.20	-1.57	12.98	0.2	11.41	0.2	0.00096952	0.00674548	0.03138338	0.49213147	A_07_P039723	c_4422	cmr|Hypothetical protein [c_4422]
c_4424	-2.22	7.77	-1.15	8.35	0.2	7.20	0.3	0.01042101	0.04290493	0.06717734	0.65691925	A_07_P039731	c_4424	cmr|Putative adhesin [c_4424]
c_4437	+2.58	7.44	+1.37	6.76	0.0	8.13	0.1	0.00054708	0.00432607	0.04271468	0.51179604	A_07_P039756	c_4437	cmr|Hypothetical protein [c_4437]
c_4437	+2.40	7.61	+1.26	6.98	0.1	8.25	0.0	0.00076119	0.00558185	0.04846735	0.54622047	A_07_P039756	c_4437	cmr|Hypothetical protein [c_4437]
c_4437	+2.10	7.52	+1.07	6.98	0.1	8.06	0.0	0.00154507	0.00955821	0.06248095	0.62656051	A_07_P039756	c_4437	cmr|Hypothetical protein [c_4437]
c_4437	+2.29	7.55	+1.20	6.95	0.1	8.15	0.2	0.00196152	0.01147840	0.05446202	0.57974983	A_07_P039756	c_4437	cmr|Hypothetical protein [c_4437]
c_4481	-2.18	10.33	-1.12	10.89	0.1	9.77	0.2	0.00300382	0.01595014	0.07071392	0.66387597	A_07_P039839	c_4481	cmr|Conserved hypothetical protein [c_4481]
c_4485	+4.76	8.57	+2.25	7.44	0.0	9.69	0.2	0.00011089	0.00139667	0.01518610	0.33114958	A_07_P039858	c_4485	cmr|Putative PTS enzyme-ii fructose [c_4485]
c_4486	+3.76	7.60	+1.91	6.65	0.2	8.56	0.1	0.00023755	0.00238308	0.02263592	0.39553829	A_07_P039865	c_4486	cmr|PTS system, fructose-like-2 IIB component 1 [c_4486]
c_4486	+4.30	7.73	+2.10	6.68	0.1	8.78	0.1	0.00006495	0.00096476	0.01803481	0.36032995	A_07_P039865	c_4486	cmr|PTS system, fructose-like-2 IIB component 1 [c_4486]
c_4486	+4.28	7.67	+2.10	6.63	0.0	8.72	0.2	0.00011061	0.00139657	0.01839304	0.36471033	A_07_P039865	c_4486	cmr|PTS system, fructose-like-2 IIB component 1 [c_4486]
c_4486	+2.91	7.50	+1.54	6.73	0.2	8.27	0.3	0.00149702	0.00932688	0.03479179	0.47349179	A_07_P039865	c_4486	cmr|PTS system, fructose-like-2 IIB component 1 [c_4486]
c_4486	+2.79	7.83	+1.48	7.09	0.2	8.57	0.3	0.00325328	0.01698829	0.03844449	0.49409796	A_07_P039865	c_4486	cmr|PTS system, fructose-like-2 IIB component 1 [c_4486]
c_4487	+5.47	8.38	+2.45	7.16	0.1	9.61	0.2	0.00004387	0.00074045	0.01201220	0.29185185	A_07_P039870	c_4487	cmr|Putative phosphotransferase system [c_4487]
c_4488	+3.12	7.63	+1.64	6.81	0.0	8.45	0.0	0.00016572	0.00185829	0.03044144	0.44459184	A_07_P039873	c_4488	cmr|Putative transcriptional Antiterminator [c_4488]
c_4493	-3.69	8.09	-1.88	9.03	0.0	7.15	0.1	0.00008725	0.00118419	0.01577744	0.39303797	A_07_P039886	c_4493	cmr|Hypothetical protein [c_4493]
c_4522	-2.59	7.80	-1.37	8.49	0.3	7.12	0.2	0.00338246	0.01748880	0.04434896	0.56859121	A_07_P040004	c_4522	cmr|Hypothetical protein [c_4522]
c_4523	-3.39	8.48	-1.76	9.36	0.2	7.60	0.3	0.00146720	0.00918111	0.02114329	0.43915567	A_07_P040008	c_4523	cmr|Conserved hypothetical protein [c_4523]
c_4529	+2.18	7.49	+1.12	6.93	0.1	8.05	0.2	0.00444828	0.02163537	0.06075648	0.61752744	A_07_P040039	c_4529	cmr|Hypothetical protein [c_4529]
c_4541	-2.18	9.04	-1.13	9.60	0.0	8.48	0.2	0.00207090	0.01195611	0.07115727	0.66526128	A_07_P040098	c_4541	cmr|Putative maturase-related protein [c_4541]
c_4580	-3.82	8.94	-1.93	9.91	0.2	7.98	0.2	0.00032233	0.00295389	0.01441629	0.37891486	A_07_P038963	c_4580	cmr|Hypothetical protein [c_4580]
c_4605	-3.04	8.13	-1.60	8.93	0.3	7.33	0.5	0.00787713	0.03426845	0.02914190	0.48448786	A_07_P039004	c_4605	cmr|Hypothetical protein [c_4605]
c_4630	-4.48	8.93	-2.16	10.01	0.0	7.85	0.1	0.00004134	0.00071049	0.00879446	0.30297593	A_07_P041901	c_4630	cmr|Probable tRNA modification GTPase trmE [c_4630]
c_4688	+3.80	8.23	+1.93	7.27	0.6	9.20	0.1	0.00418159	0.02061078	0.02243267	0.39505593	A_07_P039063	c_4688	cmr|Hypothetical protein [c_4688]
c_4748	-3.27	9.70	-1.71	10.56	0.0	8.85	0.1	0.00018956	0.00202942	0.02256415	0.45025349	A_07_P039154	c_4748	cmr|Hypothetical protein yigL [c_4748]
c_4758	+3.01	8.46	+1.59	7.66	0.3	9.26	0.1	0.00114432	0.00765015	0.03293382	0.46336908	A_07_P039181	c_4758	cmr|PTS system, glucose-specific IIBC component [c_4758]
c_4770	+2.17	7.88	+1.12	7.33	0.0	8.44	0.2	0.00236294	0.01318291	0.06015180	0.61336140	A_07_P039236	c_4770	cmr|Hypothetical protein [c_4770]
c_4775	+2.03	7.26	+1.02	6.75	0.2	7.77	0.1	0.00367911	0.01868515	0.06743458	0.64895477	A_07_P039252	c_4775	cmr|Hypothetical protein [c_4775]
c_4776	+7.70	9.21	+2.94	7.74	0.3	10.68	0.0	0.00003432	0.00064547	0.00613059	0.22657277	A_07_P039258	c_4776	cmr|Hypothetical protein [c_4776]
c_4777	+9.12	8.54	+3.19	6.94	0.0	10.13	0.0	0.00000495	0.00023989	0.00433435	0.19386364	A_07_P039263	c_4777	cmr|Putative conserved protein [c_4777]
c_4778	+4.27	8.47	+2.10	7.43	0.1	9.52	0.1	0.00006833	0.00099696	0.01793826	0.35931298	A_07_P039269	c_4778	cmr|Putative conserved protein [c_4778]
c_4779	+4.04	8.52	+2.01	7.51	0.1	9.52	0.1	0.00009560	0.00126806	0.02008702	0.37783751	A_07_P039274	c_4779	cmr|Putative transcriptional regulator [c_4779]
c_4811	-2.94	8.22	-1.56	9.00	0.1	7.44	0.1	0.00027443	0.00261702	0.03139609	0.49184080	A_07_P039303	c_4811	cmr|Hypothetical protein [c_4811]
c_4836	+5.92	11.36	+2.57	10.08	0.0	12.64	0.1	0.00001839	0.00044550	0.01053417	0.27734114	A_07_P039365	c_4836	cmr|Putative lipase [c_4836]
c_4837	-49.80	11.92	-5.64	14.74	0.0	9.10	0.2	0.00000039	0.00009117	0.00000127	0.00500000	A_07_P039369	c_4837	cmr|Hypothetical protein [c_4837]
c_4838	-2.67	12.65	-1.42	13.35	0.1	11.94	0.1	0.00049828	0.00402720	0.04113694	0.55261092	A_07_P039373	c_4838	cmr|Hypothetical protein [c_4838]
c_4839	-24.97	11.23	-4.64	13.55	0.1	8.91	0.1	0.00000094	0.00012946	0.00002731	0.01535714	A_07_P039376	c_4839	cmr|Hypothetical protein [c_4839]
c_4848	-3.22	8.50	-1.69	9.34	0.4	7.65	0.4	0.00536234	0.02515512	0.02263148	0.44988636	A_07_P039409	c_4848	cmr|Putative conserved protein [c_4848]
c_4924	-2.43	8.06	-1.28	8.70	0.0	7.42	0.0	0.00054909	0.00433542	0.05439342	0.60864961	A_07_P039536	c_4924	cmr|Putative hippuricase [c_4924]
c_4925	-2.59	8.36	-1.37	9.05	0.1	7.68	0.2	0.00145758	0.00913904	0.04579395	0.57175258	A_07_P039541	c_4925	cmr|Putative citrate permease [c_4925]
c_4928	-5.61	8.43	-2.49	9.67	0.1	7.19	0.3	0.00013227	0.00157046	0.00452617	0.21858896	A_07_P037396	c_4928	cmr|tRNA [c_4928]
c_4978	+2.70	7.45	+1.43	6.73	0.2	8.17	0.0	0.00098539	0.00682535	0.03915079	0.49628824	A_07_P037478	c_4978	cmr|Hypothetical protein [c_4978]
c_5008	+2.16	7.32	+1.11	6.76	0.0	7.87	0.2	0.00208399	0.01200525	0.06026931	0.61416181	A_07_P037543	c_5008	cmr|Hypothetical protein [c_5008]
c_5019	-6.99	8.56	-2.80	9.96	0.0	7.16	0.5	0.00044055	0.00368021	0.00251778	0.16940171	A_07_P035565	c_5019	cmr|Hypothetical protein yjbO [c_5019]
c_5029	-2.47	7.68	-1.31	8.33	0.0	7.03	0.1	0.00068321	0.00513948	0.05199251	0.60100587	A_07_P037590	c_5029	cmr|Putative transporter [c_5029]
c_5035	+3.10	8.28	+1.63	7.47	0.1	9.10	0.0	0.00028355	0.00267476	0.03113249	0.45091996	A_07_P037619	c_5035	cmr|Putative 2-oxoglutarate dehydrogenase [c_5035]
c_5058	-3.19	8.78	-1.68	9.62	0.2	7.95	0.2	0.00080787	0.00583178	0.02449632	0.46022673	A_07_P037663	c_5058	cmr|Putative transcriptional regulator [c_5058]
c_5061	-3.74	8.72	-1.90	9.68	0.7	7.77	0.3	0.00737661	0.03249897	0.01486344	0.38615512	A_07_P037671	c_5061	cmr|Hypothetical protein [c_5061]
c_5061	-3.07	8.55	-1.62	9.36	0.2	7.74	0.1	0.00037136	0.00324276	0.02734121	0.47882091	A_07_P037671	c_5061	cmr|Hypothetical protein [c_5061]
c_5062	+17.91	9.60	+4.16	7.52	0.1	11.68	0.1	0.00000233	0.00017963	0.00105246	0.09468571	A_07_P037679	c_5062	cmr|Putative symporter yjcG [c_5062]
c_5081	+2.54	7.61	+1.35	6.94	0.1	8.29	0.1	0.00067113	0.00507975	0.04420287	0.52207802	A_07_P037721	c_5081	cmr|Putative conserved protein [c_5081]
c_5088	-2.89	7.48	-1.53	8.24	0.0	6.71	0.1	0.00029959	0.00278936	0.03313643	0.50260116	A_07_P037733	c_5088	cmr|Hypothetical protein [c_5088]
c_5153	-4.88	9.62	-2.29	10.77	0.0	8.48	0.1	0.00004682	0.00076831	0.00689660	0.26942928	A_07_P037845	c_5153	cmr|Hypothetical protein [c_5153]
c_5154	-3.26	9.04	-1.70	9.89	0.1	8.19	0.2	0.00051159	0.00411787	0.02306847	0.45116770	A_07_P037850	c_5154	cmr|Hypothetical protein [c_5154]
c_5158	-2.01	8.35	-1.01	8.85	0.0	7.85	0.1	0.00207830	0.01199415	0.08989520	0.72357362	A_07_P037866	c_5158	cmr|Hypothetical protein [c_5158]
c_5166	-3.10	9.70	-1.63	10.52	0.0	8.88	0.1	0.00019518	0.00207030	0.02687373	0.47592801	A_07_P037902	c_5166	cmr|Partial Transposase [c_5166]
c_5211	-2.12	9.23	-1.09	9.78	0.0	8.69	0.2	0.00293546	0.01565028	0.07879002	0.68420849	A_07_P038078	c_5211	cmr|Hypothetical protein [c_5211]
c_5286	+2.69	8.27	+1.43	7.56	0.5	8.99	0.1	0.01093246	0.04462252	0.04137767	0.50696498	A_07_P036980	c_5286	cmr|Hypothetical protein [c_5286]
c_5295	-6.20	10.50	-2.63	11.82	0.0	9.19	0.2	0.00002770	0.00056928	0.00339748	0.19450909	A_07_P036994	c_5295	cmr|Hypothetical protein [c_5295]
c_5296	-12.85	12.28	-3.68	14.12	0.0	10.44	0.1	0.00000274	0.00018406	0.00029726	0.05258427	A_07_P037000	c_5296	cmr|Hypothetical protein [c_5296]
c_5297	-2.00	9.37	-1.00	9.87	0.2	8.88	0.1	0.00501894	0.02379351	0.09225102	0.72729094	A_07_P037001	c_5297	cmr|Hypothetical protein [c_5297]
c_5303	-2.02	8.39	-1.02	8.90	0.0	7.88	0.3	0.00738204	0.03250080	0.08534807	0.71133933	A_07_P037031	c_5303	cmr|Putative oxoacyl reductase [c_5303]
c_5321	-2.32	9.13	-1.21	9.73	0.2	8.52	0.1	0.00181752	0.01083904	0.06032330	0.63231025	A_07_P037042	c_5321	cmr|Putative conserved protein [c_5321]
c_5341	-138.61	11.38	-7.11	14.93	0.1	7.82	0.1	0.00000009	0.00007032	0.00000000	0.00000000	A_07_P042071	c_5341	cmr|Mg [c_5341]
c_5343	-40.46	9.66	-5.34	12.33	0.1	6.99	0.2	0.00000058	0.00010330	0.00000191	0.00300000	A_07_P037072	c_5343	cmr|Hypothetical protein [c_5343]
c_5349	+2.05	7.78	+1.03	7.26	0.0	8.29	0.2	0.00422334	0.02076584	0.06883130	0.65598063	A_07_P037091	c_5349	cmr|Carbamate kinase [c_5349]
c_5350	+2.65	7.34	+1.40	6.63	0.2	8.04	0.1	0.00098287	0.00681086	0.04147231	0.50694099	A_07_P037099	c_5350	cmr|Putative arginine deiminase [c_5350]
c_5356	-2.22	7.86	-1.15	8.43	0.1	7.28	0.2	0.00378822	0.01914050	0.06678417	0.65633583	A_07_P037109	c_5356	cmr|Hypothetical protein [c_5356]
c_5376	-2.85	10.45	-1.51	11.21	0.2	9.70	0.4	0.00295047	0.01571454	0.03309197	0.50289575	A_07_P037149	c_5376	cmr|Hypothetical protein [c_5376]
c_5416	+3.80	8.82	+1.92	7.86	0.2	9.78	0.1	0.00020051	0.00210910	0.02214748	0.39178652	A_07_P037232	c_5416	cmr|Hypothetical protein [c_5416]
c_5455	+2.41	8.40	+1.27	7.77	0.1	9.04	0.1	0.00100922	0.00695281	0.04845274	0.54644699	A_07_P037341	c_5455	cmr|Hypothetical protein [c_5455]
caiA	-8.49	11.86	-3.09	13.41	0.0	10.32	0.1	0.00000641	0.00027019	0.00119284	0.10982456	A_07_P010884	b0039	cmr|probable carnitine operon oxidoreductase [b0039]
caiC	+3.39	8.76	+1.76	7.87	0.1	9.64	0.1	0.00019839	0.00209346	0.02659362	0.42037149	A_07_P035675	c_0046	cmr|Probable crotonobetaine/carnitine-CoA ligase [c_0046]
caiF	+3.23	9.19	+1.69	8.34	0.0	10.03	0.0	0.00013340	0.00157799	0.02866870	0.43863946	A_07_P035426	c_0043	cmr|Transcriptional activatory protein caiF [c_0043]
caiF	+4.27	9.45	+2.10	8.41	0.2	10.50	0.0	0.00016607	0.00186089	0.01813770	0.36146835	A_07_P010261	b0034	cmr|transcriptional regulator of cai operon [b0034]
caiT	+2.30	6.89	+1.20	6.29	0.0	7.49	0.0	0.00078866	0.00573111	0.05250000	0.56964852	A_07_P010887	b0040	cmr|probable carnitine transporter [b0040]
carA	+4.92	8.85	+2.30	7.70	0.2	10.00	0.2	0.00014602	0.00168180	0.01440104	0.32297721	A_07_P010846	b0032	cmr|carbamoyl-phosphate synthetase, glutamine [b0032]
carB	+14.73	10.54	+3.88	8.60	0.2	12.48	0.1	0.00000607	0.00026402	0.00164253	0.12373206	A_07_P010855	b0033	cmr|carbamoyl-phosphate synthase large subunit [b0033]
cbl	-5.37	13.01	-2.42	14.23	0.0	11.80	0.3	0.00014354	0.00166295	0.00512767	0.23131805	A_07_P015271	b1987	cmr|transcriptional regulator cys regulon; accessory regulatory circuit affecting cysM [b1987]
cbpA	+6.50	8.68	+2.70	7.33	0.2	10.03	0.2	0.00005427	0.00084354	0.00887068	0.26055970	A_07_P001235	b1000	cmr|curved DNA-binding protein; functions closely related to DnaJ [b1000]
cbpM	+6.69	8.61	+2.74	7.23	0.0	9.98	0.0	0.00001080	0.00034789	0.00837525	0.25554264	A_07_P001226	b0999	cmr|orf, hypothetical protein [b0999]
cbrA	-3.08	7.88	-1.62	8.70	0.4	7.07	0.3	0.00411227	0.02037883	0.02611153	0.47252874	A_07_P017384	b3690	cmr|orf, hypothetical protein [b3690]
ccmD	+2.59	8.06	+1.37	7.37	0.0	8.74	0.2	0.00074546	0.00550491	0.04265943	0.51152323	A_07_P032305	c_2735	cmr|Heme exporter protein D [c_2735]
cdaR	-2.05	9.57	-1.04	10.08	0.0	9.05	0.2	0.00468484	0.02252160	0.08591654	0.71381003	A_07_P002772	b0162	cmr|orf, hypothetical protein [b0162]
cdd	-2.46	9.93	-1.30	10.58	0.0	9.28	0.1	0.00055230	0.00435638	0.05264164	0.60451495	A_07_P003479	b2143	cmr|cytidine/deoxycytidine deaminase [b2143]
cdh	-2.85	8.01	-1.51	8.76	0.1	7.25	0.3	0.00153508	0.00950012	0.03330729	0.50373679	A_07_P019370	b3918	cmr|CDP-diacylglycerol phosphotidylhydrolase [b3918]
cdsA	-2.72	11.10	-1.44	11.83	0.2	10.38	0.4	0.00411576	0.02038523	0.03776804	0.52902135	A_07_P002831	b0175	cmr|CDP-diglyceride synthetase [b0175]
cedA	-6.59	10.94	-2.72	12.30	0.1	9.58	0.2	0.00003247	0.00062646	0.00270897	0.17623967	A_07_P018702	b1731	cmr|orf, hypothetical protein [b1731]
chaC	-2.12	8.04	-1.08	8.58	0.2	7.49	0.0	0.00287276	0.01537868	0.07670478	0.67503633	A_07_P007044	b1218	cmr|cation transport regulator [b1218]
cheZ	-2.11	12.02	-1.08	12.56	0.1	11.48	0.0	0.00144516	0.00908649	0.07870935	0.68426284	A_07_P016084	b1881	cmr|chemotactic response; CheY protein phophatase; antagonist of CheY as switch regulator [b1881]
cld	-2.28	8.68	-1.19	9.27	0.0	8.08	0.3	0.00386477	0.01943373	0.06426004	0.64936457	A_07_P015457	b2027	cmr|regulator of length of O-antigen component of lipopolysaccharide chains [b2027]
clpA	+6.05	10.25	+2.60	8.95	0.0	11.55	0.1	0.00001521	0.00040444	0.01010925	0.27300172	A_07_P004862	b0882	cmr|ATP-binding component of serine protease [b0882]
clpB	+3.55	9.71	+1.83	8.79	0.1	10.62	0.3	0.00047732	0.00389176	0.02479040	0.40911950	A_07_P019845	b2592	cmr|heat shock protein [b2592]
clpP	+2.88	11.86	+1.52	11.09	0.1	12.62	0.0	0.00026388	0.00255503	0.03548717	0.47671502	A_07_P021300	b0437	cmr|ATP-dependent proteolytic subunit of clpA-clpP serine protease, heat shock protein F21.5 [b0437]
clpS	+5.29	10.74	+2.40	9.53	0.1	11.94	0.1	0.00003546	0.00065298	0.01279408	0.29930163	A_07_P004856	b0881	cmr|orf, hypothetical protein [b0881]
cmr	-2.70	8.02	-1.43	8.73	0.1	7.30	0.3	0.00134209	0.00862736	0.04062246	0.54803770	A_07_P004661	b0842	cmr|proton motive force efflux pump [b0842]
cmtA	-3.10	11.30	-1.63	12.11	0.4	10.48	0.0	0.00182618	0.01088243	0.02665396	0.47416949	A_07_P011811	b2933	cmr|PTS system, mannitol-specific enzyme II component, cryptic [b2933]
cnu	-2.59	7.77	-1.37	8.46	0.1	7.09	0.0	0.00044056	0.00368021	0.04573361	0.57236089	A_07_P020756	b1625	cmr|orf, hypothetical protein [b1625]
cobB	+2.60	8.91	+1.38	8.23	0.0	9.60	0.2	0.00097111	0.00675020	0.04209985	0.51025404	A_07_P052409	Z1761	cmr|putative nicotinic acid mononucleotide:5,6-dimethylbenzimidazole [Z1761]
cobS	-3.02	8.84	-1.59	9.63	0.1	8.04	0.3	0.00158501	0.00975927	0.02950076	0.48685535	A_07_P015292	b1992	cmr|cobalamin 5-phosphate synthase [b1992]
cobT	-2.29	9.91	-1.19	10.51	0.3	9.31	0.3	0.00722625	0.03196687	0.06482215	0.65045252	A_07_P015288	b1991	cmr|nicotinate-nucleotide dimethylbenzimidazole-P phophoribosyl transferase [b1991]
cobU	-2.22	9.50	-1.15	10.08	0.0	8.93	0.1	0.00104826	0.00715382	0.06876143	0.65810334	A_07_P015296	b1993	cmr|cobinamide kinase/cobinamide phosphate guanylyltransferase [b1993]
codA	+9.80	9.42	+3.29	7.77	0.0	11.07	0.1	0.00000464	0.00023240	0.00376588	0.18299383	A_07_P008378	b0337	cmr|cytosine deaminase [b0337]
codB	+4.50	7.94	+2.17	6.85	0.0	9.02	0.1	0.00004145	0.00071171	0.01670732	0.34429319	A_07_P031022	c_0455	cmr|Cytosine permease [c_0455]
codB	+2.39	7.82	+1.25	7.19	0.1	8.45	0.2	0.00177958	0.01067090	0.04971545	0.55355021	A_07_P008371	b0336	cmr|cytosine permease/transport [b0336]
corA	-5.26	10.83	-2.40	12.03	0.1	9.63	0.1	0.00003558	0.00065372	0.00548526	0.24190476	A_07_P050004	Z5333	cmr|Mg2+ transport, system I [Z5333]
corA	-6.10	10.93	-2.61	12.23	0.1	9.62	0.0	0.00001766	0.00043503	0.00346862	0.19786232	A_07_P039130	c_4737	cmr|Magnesium and cobalt transport protein corA [c_4737]
corA	-3.96	8.28	-1.99	9.28	0.1	7.29	0.5	0.00176388	0.01059944	0.01287221	0.35869027	A_07_P005948	b3816	cmr|Mg2+ transport, system I [b3816]
cpxR	+6.14	11.22	+2.62	9.91	0.0	12.53	0.2	0.00002569	0.00055176	0.00979484	0.27007005	A_07_P019340	b3912	cmr|transcriptional regulator in 2-component system [b3912]
cpxR	+7.51	11.80	+2.91	10.34	0.0	13.25	0.0	0.00000783	0.00029735	0.00660506	0.23263982	A_07_P041979	c_4864	cmr|Transcriptional regulatory protein cpxR [c_4864]
creD	-3.48	9.19	-1.80	10.09	0.1	8.29	0.1	0.00012107	0.00148250	0.01913999	0.41969359	A_07_P017684	b4400	cmr|tolerance to colicin E2 [b4400]
crp	-2.70	12.87	-1.43	13.59	0.0	12.15	0.1	0.00037399	0.00325950	0.04011242	0.54536269	A_07_P017815	b3357	cmr|cyclic AMP receptor protein [b3357]
csdA	-5.00	10.95	-2.32	12.11	0.0	9.79	0.1	0.00004638	0.00076454	0.00645516	0.26193299	A_07_P006050	b2810	cmr|orf, hypothetical protein [b2810]
csgA	+3.10	8.29	+1.63	7.48	0.3	9.11	0.3	0.00258821	0.01417346	0.03137640	0.45154479	A_07_P040743	c_1306	cmr|Major curlin subunit precursor [c_1306]
csgB	+2.07	7.62	+1.05	7.09	0.1	8.14	0.2	0.00402912	0.02009356	0.06473069	0.63655215	A_07_P001417	b1041	cmr|minor curlin subunit precursor, similar ro CsgA [b1041]
csiE	+8.99	8.59	+3.17	7.01	0.1	10.18	0.1	0.00000665	0.00027022	0.00444868	0.19729577	A_07_P019579	b2535	cmr|orf, hypothetical protein [b2535]
cspC	+6.26	14.52	+2.65	13.20	0.1	15.85	0.0	0.00002043	0.00047913	0.00943598	0.26767568	A_07_P015809	b1823	cmr|cold shock protein [b1823]
cspD	+107.94	11.31	+6.75	7.94	0.1	14.69	0.0	0.00000011	0.00007032	0.00001842	0.01000000	A_07_P004854	b0880	cmr|cold shock protein [b0880]
cspE	+3.79	12.57	+1.92	11.61	0.1	13.53	0.1	0.00011940	0.00146635	0.02196900	0.39082486	A_07_P035444	c_0714	cmr|Cold shock-like protein cspE [c_0714]
cspE	+3.82	12.39	+1.93	11.43	0.1	13.36	0.0	0.00008700	0.00118287	0.02167111	0.38815700	A_07_P009684	b0623	cmr|cold shock protein [b0623]
cstA	+38.48	11.45	+5.27	8.82	0.4	14.09	0.0	0.00000419	0.00022082	0.00020325	0.04050633	A_07_P016748	b0598	cmr|carbon starvation protein [b0598]
cusA	-2.12	8.59	-1.08	9.13	0.2	8.05	0.2	0.00597416	0.02741393	0.07488122	0.67328955	A_07_P031301	c_0661	cmr|Putative cation efflux system protein cusA [c_0661]
cusF	-3.78	9.24	-1.92	10.20	0.3	8.28	0.7	0.00991080	0.04113502	0.01560849	0.39130573	A_07_P016630	b0573	cmr|orf, hypothetical protein [b0573]
cutF	-2.06	11.23	-1.05	11.76	0.0	10.71	0.3	0.00887515	0.03764286	0.08088034	0.69431843	A_07_P051877	Z0204	cmr|copper homeostasis protein [Z0204]
cvpA	+2.36	12.22	+1.24	11.60	0.1	12.84	0.0	0.00088142	0.00626219	0.05011623	0.55526390	A_07_P013855	b2313	cmr|membrane protein required for colicin V production [b2313]
cvrA	-2.15	12.94	-1.10	13.49	0.2	12.39	0.2	0.00648401	0.02925890	0.07672320	0.67482123	A_07_P006906	b1191	cmr|orf, hypothetical protein [b1191]
cyaY	+2.67	10.43	+1.42	9.72	0.1	11.14	0.1	0.00051526	0.00413894	0.04039507	0.50156151	A_07_P005904	b3807	cmr|orf, hypothetical protein [b3807]
cyaY	+2.38	11.50	+1.25	10.87	0.1	12.12	0.1	0.00143828	0.00905049	0.05046811	0.55642157	A_07_P041940	c_4726	cmr|CyaY protein [c_4726]
cybC	+6.71	12.34	+2.75	10.97	0.2	13.72	0.0	0.00002371	0.00052555	0.00830983	0.25453307	A_07_P037063	c_5335	cmr|Soluble cytochrome b562 precursor [c_5335]
cybC	+7.73	11.18	+2.95	9.71	0.0	12.66	0.1	0.00000961	0.00032417	0.00613186	0.22608899	A_07_P003731	b4236	cmr|cytochrome b [b4236]
cydA	+82.79	13.43	+6.37	10.24	0.0	16.62	0.0	0.00000011	0.00007032	0.00003938	0.01631579	A_07_P010185	b0733	cmr|cytochrome d terminal oxidase, polypeptide subunit I [b0733]
cydB	+44.13	12.84	+5.46	10.11	0.1	15.58	0.1	0.00000029	0.00009047	0.00015307	0.03301370	A_07_P010187	b0734	cmr|cytochrome d terminal oxidase polypeptide subunit II [b0734]
cydC	+5.86	9.91	+2.55	8.63	0.2	11.18	0.0	0.00005402	0.00084294	0.01058626	0.27594371	A_07_P004879	b0886	cmr|ATP-binding component of cytochrome-related transport [b0886]
cyoA	+2.86	12.12	+1.51	11.37	0.0	12.88	0.1	0.00026186	0.00254965	0.03607787	0.48014370	A_07_P021271	b0432	cmr|cytochrome o ubiquinol oxidase subunit II [b0432]
cyoB	+14.77	15.06	+3.88	13.12	0.0	17.01	0.0	0.00000162	0.00015927	0.00161141	0.12256039	A_07_P021268	b0431	cmr|cytochrome o ubiquinol oxidase subunit I [b0431]
cyoC	+19.44	14.12	+4.28	11.98	0.0	16.26	0.0	0.00000093	0.00012946	0.00086192	0.08643312	A_07_P021261	b0430	cmr|cytochrome o ubiquinol oxidase subunit III [b0430]
cyoD	+9.14	9.31	+3.19	7.71	0.2	10.90	0.3	0.00004171	0.00071517	0.00445312	0.19693820	A_07_P051941	Z0532	cmr|cytochrome o ubiquinol oxidase subunit IV [Z0532]
cyoD	+12.89	12.30	+3.69	10.45	0.1	14.14	0.2	0.00000658	0.00027019	0.00212144	0.13522267	A_07_P021258	b0429	cmr|cytochrome o ubiquinol oxidase subunit IV [b0429]
cyoE	+7.05	11.18	+2.82	9.77	0.0	12.59	0.2	0.00002749	0.00056718	0.00733613	0.24367089	A_07_P021254	b0428	cmr|protoheme IX farnesyltransferase [b0428]
cysA	-4.10	9.16	-2.04	10.18	0.2	8.14	0.2	0.00015789	0.00178190	0.01177401	0.34909605	A_07_P013166	b2422	cmr|ATP-binding component of sulfate permease A protein; chromate resistance [b2422]
cysC	-2.51	9.78	-1.33	10.44	0.0	9.12	0.0	0.00048639	0.00394653	0.04979040	0.58939850	A_07_P002196	b2750	cmr|adenosine 5-phosphosulfate kinase [b2750]
cysH	-2.99	10.97	-1.58	11.76	0.0	10.17	0.1	0.00026013	0.00254204	0.02955158	0.48718325	A_07_P002256	b2762	cmr|3-phosphoadenosine 5-phosphosulfate reductase [b2762]
cysI	-4.81	11.60	-2.27	12.74	0.0	10.47	0.1	0.00005032	0.00080433	0.00711827	0.27400978	A_07_P002265	b2763	cmr|sulfite reductase, alpha subunit [b2763]
cysJ	-4.30	11.18	-2.10	12.23	0.0	10.13	0.1	0.00007071	0.00101790	0.01015434	0.31783300	A_07_P032576	c_3323	cmr|Sulfite reductase [c_3323]
cysM	-2.65	9.07	-1.40	9.77	0.1	8.37	0.1	0.00048411	0.00393484	0.04282012	0.56226856	A_07_P013164	b2421	cmr|cysteine synthase B, O-acetylserine sulfhydrolase B [b2421]
cysM	-3.44	9.85	-1.78	10.74	0.1	8.96	0.1	0.00016843	0.00187935	0.01945567	0.41960274	A_07_P030051	c_2955	cmr|Cysteine synthase B [c_2955]
cysN	-2.65	7.86	-1.40	8.56	0.2	7.16	0.1	0.00103023	0.00705826	0.04248539	0.56114933	A_07_P002205	b2751	cmr|ATP-sulfurylase [b2751]
cysN	-3.67	9.30	-1.87	10.24	0.1	8.36	0.0	0.00010003	0.00130806	0.01608613	0.39326087	A_07_P043152	Z4059	cmr|ATP-sulfurylase [Z4059]
cysN	-3.84	9.85	-1.94	10.83	0.1	8.88	0.1	0.00011432	0.00142293	0.01376397	0.37233677	A_07_P030710	c_3318	cmr|Sulfate adenylyltransferase subunit 1 [c_3318]
cysN	-3.85	9.36	-1.94	10.33	0.0	8.39	0.2	0.00020557	0.00215051	0.01376461	0.37171527	A_07_P056037	ECs3605	cmr|ATP-sulfurylase [ECs3605]
cysP	-4.40	11.63	-2.14	12.70	0.0	10.56	0.0	0.00003913	0.00068389	0.00941184	0.30870833	A_07_P013181	b2425	cmr|thiosulfate binding protein [b2425]
cysP	-6.59	12.41	-2.72	13.77	0.0	11.05	0.1	0.00001599	0.00041545	0.00277439	0.17542169	A_07_P030063	c_2959	cmr|Thiosulfate-binding protein precursor [c_2959]
cysP	-5.40	11.01	-2.43	12.23	0.0	9.79	0.1	0.00002674	0.00056124	0.00503430	0.22907514	A_07_P042814	Z3690	cmr|thiosulfate binding protein [Z3690]
cysQ	+2.14	9.26	+1.10	8.71	0.1	9.81	0.1	0.00172436	0.01042962	0.06107533	0.61996776	A_07_P042065	c_5313	cmr|CysQ protein [c_5313]
cysQ	+2.28	8.77	+1.19	8.17	0.1	9.36	0.1	0.00120077	0.00792330	0.05412411	0.57889266	A_07_P003624	b4214	cmr|affects pool of 3-phosphoadenosine-5-phosphosulfate in pathway of sulfite synthesis [b4214]
cysU	-3.92	11.98	-1.97	12.96	0.1	10.99	0.1	0.00007405	0.00105418	0.01313072	0.36332162	A_07_P013177	b2424	cmr|sulfate, thiosulfate transport system permease T protein [b2424]
cysU	-9.18	10.59	-3.20	12.19	0.0	8.99	0.2	0.00001271	0.00037366	0.00096735	0.10153333	A_07_P042582	c_2958	cmr|Sulfate transport system permease protein cysT [c_2958]
cysW	-17.31	10.67	-4.11	12.73	0.0	8.62	0.2	0.00000343	0.00020055	0.00008448	0.02714286	A_07_P013174	b2423	cmr|sulfate transport system permease W protein [b2423]
cytR	+2.24	10.36	+1.17	9.78	0.1	10.94	0.0	0.00120006	0.00792191	0.05499365	0.58304377	A_07_P019449	b3934	cmr|regulator for deo operon, udp, cdd, tsx, nupC, and nupG [b3934]
dadA	+19.78	10.47	+4.31	8.31	0.0	12.62	0.0	0.00000094	0.00012946	0.00082190	0.08457516	A_07_P006899	b1189	cmr|D-amino acid dehydrogenase subunit [b1189]
dadA	+10.74	10.29	+3.42	8.58	0.1	12.01	0.2	0.00001120	0.00034947	0.00317010	0.16526490	A_07_P038722	c_1638	cmr|D-amino acid dehydrogenase small subunit [c_1638]
dadX	+9.48	9.83	+3.25	8.21	0.0	11.45	0.1	0.00000446	0.00022815	0.00393483	0.18492537	A_07_P006905	b1190	cmr|alanine racemase 2, catabolic [b1190]
damX	-2.04	11.00	-1.03	11.52	0.0	10.48	0.1	0.00231369	0.01300027	0.08670859	0.71435897	A_07_P017966	b3388	cmr|putative membrane protein; interferes with cell division [b3388]
dapA	+2.12	11.30	+1.08	10.76	0.0	11.84	0.1	0.00190606	0.01121443	0.06269754	0.62633883	A_07_P013447	b2478	cmr|dihydrodipicolinate synthase [b2478]
dapB	+2.47	9.05	+1.31	8.39	0.0	9.70	0.2	0.00093685	0.00657300	0.04616997	0.53764793	A_07_P010842	b0031	cmr|dihydrodipicolinate reductase [b0031]
dapD	+4.38	8.95	+2.13	7.89	0.0	10.02	0.1	0.00004545	0.00075592	0.01742950	0.35407742	A_07_P041442	c_0201	cmr|2,3,4,5-tetrahydropyridine-2,6-dicarboxylate N-succinyltransferase [c_0201]
dapD	+2.83	8.22	+1.50	7.47	0.1	8.97	0.0	0.00032522	0.00296659	0.03636115	0.48187710	A_07_P002786	b0166	cmr|2,3,4,5-tetrahydropyridine-2-carboxylate N-succinyltransferase [b0166]
dapE	-6.18	10.64	-2.63	11.95	0.1	9.32	0.0	0.00001388	0.00039022	0.00332254	0.19091241	A_07_P013420	b2472	cmr|N-succinyl-diaminopimelate deacylase [b2472]
dapF	+2.01	10.58	+1.01	10.07	0.1	11.08	0.2	0.00366844	0.01865280	0.07069296	0.66209994	A_07_P005914	b3809	cmr|diaminopimelate epimerase [b3809]
dcd	-3.00	12.43	-1.58	13.22	0.0	11.63	0.0	0.00019740	0.00208724	0.02975292	0.48794792	A_07_P003102	b2065	cmr|2-deoxycytidine 5-triphosphate deaminase [b2065]
DCP_20_0	-2.08	9.94	-1.06	10.47	0.2	9.41	0.2	0.00581441	0.02678233	0.08158854	0.69659978	DCP_20_0	DCP_20_0	
DCP_20_9	-2.95	7.90	-1.56	8.68	0.0	7.12	0.1	0.00023647	0.00237743	0.03143928	0.49202783	DCP_20_9	DCP_20_9	
DCP_22_0	-2.54	10.58	-1.35	11.25	0.1	9.90	0.2	0.00146330	0.00916033	0.04844068	0.58128811	DCP_22_0	DCP_22_0	
DCP_22_0	-2.59	10.44	-1.37	11.12	0.1	9.75	0.2	0.00179042	0.01070582	0.04588415	0.57197150	DCP_22_0	DCP_22_0	
DCP_22_2	-2.65	10.21	-1.41	10.91	0.3	9.51	0.5	0.01009440	0.04178972	0.04349466	0.56500000	DCP_22_2	DCP_22_2	
DCP_22_2	-2.78	10.08	-1.47	10.82	0.3	9.34	0.2	0.00229729	0.01293121	0.03754446	0.52729706	DCP_22_2	DCP_22_2	
DCP_22_4	-2.53	10.32	-1.34	10.99	0.2	9.65	0.5	0.01092258	0.04459677	0.04908918	0.58505678	DCP_22_4	DCP_22_4	
DCP_22_4	-2.59	10.15	-1.37	10.84	0.1	9.47	0.2	0.00131780	0.00850307	0.04637386	0.57488976	DCP_22_4	DCP_22_4	
DCP_22_6	-3.01	10.39	-1.59	11.18	0.3	9.59	0.1	0.00177156	0.01062937	0.02979929	0.48819979	DCP_22_6	DCP_22_6	
DCP_22_6	-2.18	10.47	-1.12	11.03	0.1	9.91	0.3	0.00514856	0.02433241	0.07358232	0.67119351	DCP_22_6	DCP_22_6	
DCP_22_7	-3.00	9.64	-1.58	10.43	0.1	8.85	0.5	0.00436174	0.02130783	0.03061992	0.49091650	DCP_22_7	DCP_22_7	
dctA	+19.18	10.59	+4.26	8.46	0.1	12.72	0.0	0.00000120	0.00014031	0.00090320	0.08670732	A_07_P007574	b3528	cmr|uptake of C4-dicarboxylic acids [b3528]
dcuA	+5.85	10.66	+2.55	9.38	0.1	11.93	0.1	0.00002671	0.00056124	0.01076029	0.27817734	A_07_P011423	b4138	cmr|anaerobic dicarboxylate transport [b4138]
dcuB	-2.50	8.30	-1.32	8.96	0.0	7.64	0.2	0.00091982	0.00647948	0.04972815	0.58910459	A_07_P011354	b4123	cmr|anaerobic dicarboxylate transport [b4123]
dcuS	+2.23	10.19	+1.16	9.61	0.1	10.77	0.1	0.00140127	0.00890298	0.05560849	0.58640322	A_07_P011361	b4125	cmr|putative 2-component sensor protein [b4125]
dcyD	-3.47	11.82	-1.79	12.72	0.0	10.93	0.1	0.00011112	0.00139729	0.01870808	0.41778723	A_07_P014958	b1919	cmr|putative 1-aminocyclopropane-1-carboxylate deaminase [b1919]
ddlA	-3.65	9.89	-1.87	10.82	0.1	8.95	0.0	0.00010694	0.00135989	0.01631796	0.39403374	A_07_P021019	b0381	cmr|D-alanine-D-alanine ligase A [b0381]
deaD	-35.94	10.80	-5.17	13.39	0.0	8.22	0.1	0.00000046	0.00009262	0.00000572	0.00692308	A_07_P000534	b3162	cmr|inducible ATP-independent RNA helicase [b3162]
dedA	-3.22	9.99	-1.69	10.83	0.1	9.15	0.2	0.00050047	0.00404072	0.02372459	0.45662592	A_07_P032436	c_2862	cmr|DedA protein [c_2862]
degQ	+4.85	8.94	+2.28	7.80	0.0	10.08	0.1	0.00003275	0.00062948	0.01487233	0.32840112	A_07_P041306	c_3989	cmr|Protease degQ precursor [c_3989]
degS	-3.70	9.37	-1.89	10.31	0.1	8.42	0.1	0.00012109	0.00148250	0.01544779	0.39101286	A_07_P034177	c_3990	cmr|Protease degS precursor [c_3990]
deoB	+9.77	11.55	+3.29	9.91	0.1	13.20	0.2	0.00000934	0.00032222	0.00382749	0.18371951	A_07_P017598	b4383	cmr|phosphopentomutase [b4383]
deoC	-4.65	14.69	-2.22	15.80	0.0	13.58	0.1	0.00004544	0.00075592	0.00796939	0.29179070	A_07_P017589	b4381	cmr|2-deoxyribose-5-phosphate aldolase [b4381]
deoD	+2.17	11.16	+1.12	10.60	0.0	11.72	0.0	0.00107177	0.00727327	0.05853976	0.60356909	A_07_P017603	b4384	cmr|purine-nucleoside phosphorylase [b4384]
deoR	-8.63	10.18	-3.11	11.74	0.1	8.63	0.4	0.00006688	0.00098312	0.00117632	0.10894118	A_07_P042124	c_0925	cmr|Deoxyribose operon repressor [c_0925]
deoR	-10.79	10.63	-3.43	12.34	0.1	8.91	0.0	0.00000360	0.00020590	0.00060023	0.07941176	A_07_P004651	b0840	cmr|transcriptional repressor for deo operon, tsx, nupG [b0840]
der	-2.50	8.97	-1.32	9.63	0.1	8.31	0.1	0.00097418	0.00676552	0.04989456	0.58930233	A_07_P013612	b2511	cmr|putative GTP-binding factor [b2511]
dgoA	+2.15	7.85	+1.11	7.30	0.0	8.40	0.1	0.00173756	0.01049332	0.06064342	0.61677649	A_07_P017391	b3692	cmr|2-oxo-3-deoxygalactonate 6-phosphate aldolase and galactonate dehydratase [b3692]
dgt	+2.16	8.96	+1.11	8.40	0.0	9.51	0.1	0.00123161	0.00809287	0.05838288	0.60234600	A_07_P035898	c_0196	cmr|Deoxyguanosinetriphosphate triphosphohydrolase [c_0196]
dhaK	+20.35	10.08	+4.35	7.91	0.1	12.26	0.2	0.00000241	0.00017963	0.00076537	0.08368056	A_07_P006953	b1200	cmr|putative dihydroxyacetone kinase [b1200]
dhaL	+8.65	8.54	+3.11	6.98	0.1	10.09	0.1	0.00000781	0.00029735	0.00487360	0.20964481	A_07_P006948	b1199	cmr|putative dihydroxyacetone kinase [b1199]
dhaM	+2.22	7.39	+1.15	6.82	0.1	7.97	0.0	0.00111813	0.00751656	0.05611280	0.58856762	A_07_P006942	b1198	cmr|putative PTS system enzyme I [b1198]
diaA	+3.55	9.63	+1.83	8.72	0.1	10.55	0.0	0.00010347	0.00133412	0.02462652	0.40769716	A_07_P000466	b3149	cmr|orf, hypothetical protein [b3149]
dinB	-5.50	11.72	-2.46	12.95	0.1	10.49	0.0	0.00002706	0.00056519	0.00487043	0.22552941	A_07_P007867	b0231	cmr|damage-inducible protein P; putative tRNA synthetase [b0231]

dinF	-2.53	10.26	-1.34	10.93	0.0	9.59	0.2	0.00080911	0.00583808	0.04820567	0.57979374	A_07_P008863	b4044	cmr|DNA-damage-inducible protein F [b4044]
dinG	+7.17	11.62	+2.84	10.20	0.1	13.04	0.1	0.00001466	0.00039588	0.00720783	0.24144681	A_07_P004448	b0799	cmr|probably ATP-dependent helicase [b0799]
dinI	-15.45	12.97	-3.95	14.94	0.0	10.99	0.0	0.00000142	0.00015187	0.00013656	0.03909091	A_07_P001507	b1061	cmr|damage-inducible protein I [b1061]
dipZ	+2.74	8.40	+1.46	7.67	0.1	9.12	0.2	0.00069025	0.00518102	0.03870427	0.49501219	A_07_P011411	b4136	cmr|thiol:disulfide interchange protein; copper tolerance [b4136]
djlB	-3.58	11.86	-1.84	12.78	0.1	10.94	0.1	0.00013753	0.00161341	0.01716717	0.40041481	A_07_P009800	b0646	cmr|putative enzyme of polynucleotide modification [b0646]
dkgA	+4.23	9.03	+2.08	7.99	0.0	10.07	0.1	0.00005588	0.00086270	0.01844703	0.36349186	A_07_P012193	b3012	cmr|orf, hypothetical protein [b3012]
dkgB	-4.94	8.76	-2.30	9.91	0.1	7.61	0.4	0.00033107	0.00300596	0.00659807	0.26298734	A_07_P002965	b0207	cmr|putative aldose reductase [b0207]
dld	+2.79	9.06	+1.48	8.32	0.1	9.80	0.3	0.00257395	0.01411995	0.03794398	0.49127467	A_07_P032211	c_2664	cmr|D-lactate dehydrogenase [c_2664]
dld	+2.51	8.70	+1.33	8.04	0.4	9.37	0.2	0.00543106	0.02540304	0.04629764	0.53635762	A_07_P045443	Z3382	cmr|D-lactate dehydrogenase, FAD protein, NADH independent [Z3382]
dmsA	+10.26	10.49	+3.36	8.81	0.2	12.17	0.1	0.00001133	0.00035103	0.00341082	0.17156550	A_07_P033816	c_1031	cmr|Anaerobic dimethyl sulfoxide reductase chain A precursor [c_1031]
dmsA	+7.26	8.58	+2.86	7.15	0.1	10.01	0.2	0.00003453	0.00064547	0.00704649	0.23961123	A_07_P004916	b0894	cmr|anaerobic dimethyl sulfoxide reductase subunit A [b0894]
dmsB	+8.09	8.99	+3.02	7.49	0.1	10.50	0.1	0.00000935	0.00032222	0.00565930	0.21945813	A_07_P004924	b0895	cmr|anaerobic dimethyl sulfoxide reductase subunit B [b0895]
dmsC	+3.65	8.08	+1.87	7.15	0.1	9.02	0.1	0.00012741	0.00153683	0.02349149	0.40027056	A_07_P052033	Z1242	cmr|anaerobic dimethyl sulfoxide reductase subunit C [Z1242]
dmsC	+6.87	8.76	+2.78	7.37	0.0	10.15	0.1	0.00001288	0.00037639	0.00787729	0.24754491	A_07_P004926	b0896	cmr|anaerobic dimethyl sulfoxide reductase subunit C [b0896]
dmsD	+2.64	8.48	+1.40	7.78	0.1	9.18	0.2	0.00073718	0.00546433	0.04124809	0.50616524	A_07_P020586	b1591	cmr|putative oxidoreductase component [b1591]
dnaA	-4.30	13.48	-2.10	14.54	0.0	12.43	0.1	0.00004746	0.00077461	0.01004446	0.31628000	A_07_P005441	b3702	cmr|DNA biosynthesis; initiation of chromosome replication; can be transcription regulator [b3702]
dnaB	-2.38	9.25	-1.25	9.88	0.0	8.63	0.0	0.00064642	0.00492163	0.05807228	0.62281335	A_07_P008903	b4052	cmr|replicative DNA helicase; part of primosome [b4052]
dnaG	-11.91	11.12	-3.57	12.91	0.0	9.33	0.1	0.00000277	0.00018406	0.00040206	0.06267327	A_07_P000065	b3066	cmr|DNA biosynthesis; DNA primase [b3066]
dnaJ	+2.28	9.64	+1.19	9.05	0.1	10.24	0.0	0.00135886	0.00870378	0.05346418	0.57456655	A_07_P010766	b0015	cmr|chaperone with DnaK; heat shock protein [b0015]
dnaK	+3.93	11.67	+1.98	10.68	0.2	12.65	0.1	0.00014921	0.00170726	0.02057673	0.38112941	A_07_P010763	b0014	cmr|chaperone Hsp70; DNA biosynthesis; autoregulated heat shock proteins [b0014]
dnaN	-7.58	12.59	-2.92	14.05	0.0	11.13	0.2	0.00002431	0.00053302	0.00177020	0.13797030	A_07_P005436	b3701	cmr|DNA polymerase III, beta-subunit [b3701]
dnaQ	+2.07	8.90	+1.05	8.37	0.0	9.42	0.1	0.00193816	0.01135628	0.06522929	0.63786957	A_07_P051886	Z0241	cmr|DNA polymerase III, epsilon subunit [Z0241]
dnaT	-2.88	12.95	-1.52	13.71	0.0	12.18	0.1	0.00034271	0.00307622	0.03331364	0.50286673	A_07_P017509	b4362	cmr|DNA biosynthesis; primosomal protein i [b4362]
dnaX	+6.22	11.07	+2.64	9.76	0.1	12.39	0.1	0.00001569	0.00041046	0.00962716	0.26826549	A_07_P021457	b0470	cmr|DNA polymerase III, tau and gamma subunits; DNA elongation factor III [b0470]
dpiA	-3.44	9.73	-1.78	10.62	0.1	8.84	0.7	0.01017699	0.04205422	0.02003430	0.42914286	A_07_P009666	b0620	cmr|sequence similarity to Shigella regulator [b0620]
dpiB	-3.21	9.44	-1.68	10.28	0.4	8.60	0.1	0.00176741	0.01061336	0.02450711	0.45933333	A_07_P009663	b0619	cmr|putative sensor-type protein [b0619]
dppA	+3.18	8.22	+1.67	7.39	0.1	9.05	0.2	0.00028764	0.00270203	0.03001016	0.44198316	A_07_P007652	b3544	cmr|dipeptide transport protein [b3544]
dppD	+4.86	9.15	+2.28	8.01	0.3	10.29	0.1	0.00016856	0.00187949	0.01467670	0.32590973	A_07_P039583	c_4356	cmr|Dipeptide transport ATP-binding protein dppD [c_4356]
dppD	+3.94	8.46	+1.98	7.47	0.0	9.45	0.1	0.00006709	0.00098530	0.02056402	0.38134276	A_07_P007640	b3541	cmr|putative ATP-binding component of dipeptide transport system [b3541]
dps	+5.52	11.88	+2.47	10.64	0.1	13.11	0.3	0.00017567	0.00193279	0.01166349	0.28827316	A_07_P004515	b0812	cmr|global regulator, starvation conditions [b0812]
dsbA	+2.59	9.14	+1.37	8.45	0.1	9.83	0.0	0.00044896	0.00373401	0.04231199	0.51046743	A_07_P019081	b3860	cmr|protein disulfide isomerase I, essential for cytochrome c synthesis and formate-dependent reduction [b3860]
dsbB	-2.27	8.08	-1.18	8.67	0.1	7.49	0.2	0.00288715	0.01544525	0.06534362	0.65318730	A_07_P006878	b1185	cmr|reoxidizes DsbA protein following formation of disulfide bond in P-ring of flagella. [b1185]
dsbG	-2.03	9.52	-1.02	10.03	0.0	9.00	0.2	0.00337376	0.01746104	0.08565993	0.71280655	A_07_P016779	b0604	cmr|thiol:disulfide interchange protein [b0604]
dsdX	+7.50	8.40	+2.91	6.95	0.3	9.85	0.0	0.00003586	0.00065574	0.00652693	0.23144144	A_07_P014107	b2365	cmr|transport system permease [b2365]
dtd	+5.77	10.28	+2.53	9.02	0.3	11.54	0.2	0.00023278	0.00234931	0.01071583	0.27794069	A_07_P019212	b3887	cmr|orf, hypothetical protein [b3887]
dusA	-2.92	8.55	-1.55	9.33	0.1	7.78	0.1	0.00044086	0.00368021	0.03168064	0.49189349	A_07_P008886	b4049	cmr|orf, hypothetical protein [b4049]
dusC	-3.06	7.65	-1.61	8.46	0.1	6.84	0.0	0.00019204	0.00204431	0.02824822	0.48288817	A_07_P003464	b2140	cmr|putative regulator protein [b2140]
E1A_r60_a104	-2.00	9.82	-1.00	10.32	0.1	9.32	0.1	0.00305829	0.01616306	0.09084477	0.72454914	(+)E1A_r60_a104	E1A_r60_a104	
E1A_r60_a104	-2.91	8.92	-1.54	9.69	0.1	8.15	0.0	0.00033777	0.00304380	0.03249492	0.49766537	(+)E1A_r60_a104	E1A_r60_a104	
E1A_r60_a104	-2.23	9.99	-1.16	10.57	0.1	9.42	0.1	0.00117900	0.00781894	0.06844195	0.65905199	(+)E1A_r60_a104	E1A_r60_a104	
E1A_r60_a107	-2.37	8.66	-1.24	9.28	0.1	8.04	0.1	0.00094086	0.00658934	0.05825902	0.62311821	(+)E1A_r60_a107	E1A_r60_a107	
E1A_r60_a107	-3.33	9.03	-1.74	9.89	0.5	8.16	0.3	0.00429180	0.02103675	0.02080158	0.43435013	(+)E1A_r60_a107	E1A_r60_a107	
E1A_r60_a107	-2.34	8.38	-1.23	9.00	0.1	7.77	0.2	0.00172656	0.01043894	0.06066311	0.63250331	(+)E1A_r60_a107	E1A_r60_a107	
E1A_r60_a107	-2.63	8.58	-1.39	9.28	0.1	7.89	0.3	0.00330662	0.01719838	0.04360201	0.56546129	(+)E1A_r60_a107	E1A_r60_a107	
E1A_r60_a107	-2.42	8.98	-1.28	9.62	0.3	8.35	0.3	0.00759608	0.03324791	0.05293763	0.60351195	(+)E1A_r60_a107	E1A_r60_a107	
E1A_r60_a135	-3.75	8.99	-1.91	9.95	0.7	8.04	0.1	0.00728709	0.03218173	0.01500826	0.38799672	(+)E1A_r60_a135	E1A_r60_a135	
E1A_r60_a135	-2.78	9.56	-1.48	10.30	0.1	8.82	0.0	0.00035344	0.00313846	0.03727960	0.52592294	(+)E1A_r60_a135	E1A_r60_a135	
E1A_r60_a135	-2.58	8.98	-1.37	9.66	0.5	8.29	0.1	0.01089085	0.04452493	0.04425368	0.56922386	(+)E1A_r60_a135	E1A_r60_a135	
E1A_r60_a20	-2.20	9.84	-1.14	10.41	0.1	9.27	0.3	0.00641210	0.02902590	0.06807546	0.65874616	(+)E1A_r60_a20	E1A_r60_a20	
E1A_r60_a20	-2.85	10.12	-1.51	10.87	0.3	9.36	0.4	0.00543457	0.02540434	0.03286966	0.50048356	(+)E1A_r60_a20	E1A_r60_a20	
E1A_r60_a20	-3.04	9.84	-1.60	10.64	0.1	9.04	0.1	0.00036417	0.00320148	0.02809070	0.48229008	(+)E1A_r60_a20	E1A_r60_a20	
E1A_r60_a20	-2.65	9.71	-1.41	10.41	0.1	9.00	0.5	0.01126607	0.04562062	0.04270579	0.56311558	(+)E1A_r60_a20	E1A_r60_a20	
E1A_r60_a20	-4.09	9.50	-2.03	10.52	0.1	8.48	0.5	0.00174379	0.01051887	0.01208016	0.34642987	(+)E1A_r60_a20	E1A_r60_a20	
E1A_r60_a20	-2.51	9.86	-1.33	10.52	0.4	9.19	0.2	0.00783494	0.03412302	0.05038110	0.59194030	(+)E1A_r60_a20	E1A_r60_a20	
E1A_r60_a20	-2.92	10.04	-1.55	10.82	0.3	9.27	0.6	0.01224356	0.04864060	0.03020262	0.49072239	(+)E1A_r60_a20	E1A_r60_a20	
E1A_r60_a20	-4.09	9.58	-2.03	10.60	0.4	8.57	0.2	0.00127508	0.00830914	0.01189914	0.34628466	(+)E1A_r60_a20	E1A_r60_a20	
E1A_r60_a20	-2.46	10.23	-1.30	10.88	0.0	9.58	0.4	0.00632559	0.02872515	0.05205983	0.60134263	(+)E1A_r60_a20	E1A_r60_a20	
E1A_r60_a20	-2.51	9.84	-1.33	10.50	0.2	9.18	0.0	0.00143383	0.00903332	0.04917746	0.58478097	(+)E1A_r60_a20	E1A_r60_a20	
E1A_r60_a22	-2.22	10.89	-1.15	11.46	0.1	10.31	0.1	0.00189706	0.01117786	0.06890561	0.65788357	(+)E1A_r60_a22	E1A_r60_a22	
E1A_r60_a22	-2.31	10.64	-1.21	11.24	0.0	10.03	0.4	0.01015397	0.04198113	0.06175432	0.63712975	(+)E1A_r60_a22	E1A_r60_a22	
E1A_r60_a22	-3.07	10.69	-1.62	11.50	0.4	9.88	0.1	0.00237125	0.01321052	0.02782457	0.48245595	(+)E1A_r60_a22	E1A_r60_a22	
E1A_r60_a22	-2.31	10.56	-1.21	11.17	0.1	9.96	0.4	0.01128295	0.04567080	0.06224085	0.64005225	(+)E1A_r60_a22	E1A_r60_a22	
E1A_r60_a22	-3.21	10.37	-1.68	11.21	0.1	9.53	0.3	0.00099297	0.00687483	0.02494538	0.46259128	(+)E1A_r60_a22	E1A_r60_a22	
E1A_r60_a22	-2.65	10.63	-1.41	11.34	0.2	9.93	0.1	0.00097014	0.00674641	0.04293128	0.56325833	(+)E1A_r60_a22	E1A_r60_a22	
E1A_r60_a22	-2.30	10.66	-1.20	11.26	0.0	10.06	0.3	0.00360835	0.01842681	0.06328824	0.64367571	(+)E1A_r60_a22	E1A_r60_a22	
E1A_r60_a22	-3.50	10.37	-1.81	11.27	0.1	9.46	0.7	0.01219580	0.04848753	0.01939850	0.41894376	(+)E1A_r60_a22	E1A_r60_a22	
E1A_r60_a22	-2.89	10.49	-1.53	11.26	0.2	9.73	0.2	0.00161975	0.00994979	0.03364393	0.50494757	(+)E1A_r60_a22	E1A_r60_a22	
E1A_r60_a22	-2.15	10.79	-1.10	11.35	0.1	10.24	0.3	0.00845689	0.03626950	0.07606390	0.67467606	(+)E1A_r60_a22	E1A_r60_a22	
E1A_r60_a22	-2.48	10.83	-1.31	11.49	0.1	10.18	0.1	0.00092439	0.00650293	0.05194741	0.60092579	(+)E1A_r60_a22	E1A_r60_a22	
E1A_r60_a22	-2.14	10.69	-1.10	11.24	0.1	10.14	0.2	0.00288252	0.01542572	0.07726054	0.67765460	(+)E1A_r60_a22	E1A_r60_a22	
E1A_r60_a22	-2.38	10.61	-1.25	11.24	0.2	9.99	0.2	0.00335547	0.01737782	0.05826664	0.62277665	(+)E1A_r60_a22	E1A_r60_a22	
E1A_r60_a22	-3.57	10.27	-1.84	11.19	0.0	9.35	0.6	0.00535556	0.02513204	0.01715765	0.40078635	(+)E1A_r60_a22	E1A_r60_a22	
E1A_r60_a22	-2.20	10.67	-1.14	11.24	0.2	10.10	0.1	0.00293347	0.01564519	0.07093877	0.66519357	(+)E1A_r60_a22	E1A_r60_a22	
E1A_r60_a97	-2.43	8.17	-1.28	8.81	0.2	7.53	0.3	0.00708327	0.03150253	0.05419525	0.60816108	(+)E1A_r60_a97	E1A_r60_a97	
E1A_r60_n11	-4.54	7.92	-2.18	9.01	0.2	6.83	0.1	0.00016901	0.00188177	0.00825584	0.29540909	(+)E1A_r60_n11	E1A_r60_n11	
E1A_r60_n9	-2.12	7.41	-1.08	7.95	0.2	6.87	0.2	0.00693239	0.03092759	0.07771532	0.67861897	(+)E1A_r60_n9	E1A_r60_n9	
eaeH	-3.02	12.09	-1.60	12.89	0.1	11.29	0.3	0.00078421	0.00571336	0.02836509	0.48278919	A_07_P008177	b0297	cmr|attaching and effacing protein, pathogenesis factor [b0297]
ebgR	+3.15	9.45	+1.65	8.62	0.3	10.27	0.0	0.00092717	0.00651374	0.03052401	0.44497222	A_07_P041749	c_3832	cmr|Ebg operon repressor [c_3832]
eco	+2.92	13.17	+1.55	12.39	0.1	13.94	0.0	0.00026490	0.00255703	0.03455856	0.47148180	A_07_P032326	c_2750	cmr|Ecotin precursor [c_2750]
ECs0016	-3.31	12.57	-1.73	13.44	0.0	11.71	0.1	0.00020304	0.00212689	0.02239329	0.44797967	A_07_P058872	ECs0016	cmr|Gef protein [ECs0016]
ECs0031	+2.61	10.06	+1.38	9.37	0.2	10.75	0.4	0.00609359	0.02784025	0.04404281	0.52096920	A_07_P062890	ECs0031	cmr|putative FKBX-type 16KD peptidyl-prolyl cis-trans isomerase [ECs0031]
ECs0037	+3.53	9.36	+1.82	8.45	0.3	10.27	0.2	0.00127284	0.00829795	0.02527248	0.41189441	A_07_P058876	ECs0037	cmr|transcriptional regulator of cai operon [ECs0037]
ECs0052	-2.74	11.77	-1.45	12.50	0.0	11.05	0.1	0.00038477	0.00332667	0.03861217	0.53560352	A_07_P059413	ECs0052	cmr|CcdA-like protein [ECs0052]
ECs0077	+2.16	8.84	+1.11	8.28	0.0	9.39	0.2	0.00295943	0.01574747	0.06137386	0.62179537	A_07_P059441	ECs0077	cmr|3-isopropylmalate dehydrogenase [ECs0077]
ECs0132	-2.94	8.44	-1.56	9.21	0.1	7.66	0.3	0.00124940	0.00818921	0.03038300	0.49061538	A_07_P059452	ECs0132	cmr|hypothetical protein [ECs0132]
ECs0192	-3.54	10.61	-1.82	11.52	0.1	9.70	0.3	0.00074472	0.00550202	0.01797574	0.40956585	A_07_P059513	ECs0192	cmr|hypothetical protein [ECs0192]
ECs0230	-3.45	7.72	-1.79	8.61	0.5	6.82	0.0	0.00345821	0.01779285	0.01931911	0.41953103	A_07_P059608	ECs0230	cmr|hypothetical protein [ECs0230]
ECs0237	-2.85	10.09	-1.51	10.85	0.3	9.34	0.1	0.00148612	0.00928472	0.03436166	0.50892756	A_07_P059639	ECs0237	cmr|RhsG core protein with extension [ECs0237]
ECs0241	-2.76	10.67	-1.47	11.40	0.1	9.94	0.2	0.00104312	0.00712961	0.03684578	0.52308386	A_07_P053529	ECs0241	cmr|H repeat-associated protein [ECs0241]
ECs0269	-2.47	8.71	-1.30	9.36	0.0	8.06	0.4	0.00440816	0.02149338	0.05020135	0.59115183	A_07_P062927	ECs0269	cmr|gamma-glutamate kinase [ECs0269]
ECs0278	-6.30	11.30	-2.66	12.63	0.0	9.97	0.0	0.00001289	0.00037639	0.00309578	0.18532319	A_07_P053587	ECs0278	cmr|replication protein [ECs0278]
ECs0280	-3.29	9.09	-1.72	9.95	0.3	8.23	0.1	0.00080689	0.00582985	0.02282139	0.45138191	A_07_P053597	ECs0280	cmr|putative tail fiber protein [ECs0280]
ECs0290	-2.22	11.35	-1.15	11.93	0.1	10.78	0.2	0.00229564	0.01292654	0.07051258	0.66277612	A_07_P053647	ECs0290	cmr|hypothetical protein [ECs0290]
ECs0296	-4.53	9.54	-2.18	10.63	0.2	8.45	0.6	0.00265024	0.01443903	0.00892340	0.30343413	A_07_P053677	ECs0296	cmr|putative Ogr family transcription activator [ECs0296]
ECs0325	-2.30	8.33	-1.20	8.93	0.2	7.73	0.3	0.00659401	0.02970420	0.06335112	0.64389929	A_07_P053765	ECs0325	cmr|hypothetical protein [ECs0325]
ECs0326	-2.55	8.04	-1.35	8.72	0.0	7.36	0.1	0.00057712	0.00450930	0.04785061	0.58040062	A_07_P053768	ECs0326	cmr|hypothetical protein [ECs0326]
ECs0330	-2.57	8.90	-1.36	9.58	0.1	8.22	0.4	0.00635378	0.02882691	0.04378557	0.56644207	A_07_P053789	ECs0330	cmr|hypothetical protein [ECs0330]
ECs0332	+3.66	8.51	+1.87	7.57	0.3	9.44	0.5	0.00416807	0.02057988	0.02388783	0.40266595	A_07_P053796	ECs0332	cmr|hypothetical protein [ECs0332]
ECs0335	+3.17	7.75	+1.66	6.92	0.1	8.59	0.0	0.00016007	0.00180270	0.02948425	0.43916746	A_07_P053815	ECs0335	cmr|putative oxidoreductase [ECs0335]
ECs0350	-2.10	10.22	-1.07	10.76	0.3	9.69	0.2	0.01045233	0.04300011	0.07615218	0.67356180	A_07_P053843	ECs0350	cmr|putative adhesin [ECs0350]
ECs0357	+4.54	9.52	+2.18	8.43	0.0	10.61	0.0	0.00003477	0.00064621	0.01654217	0.34313570	A_07_P053871	ECs0357	cmr|choline dehydrogenase [ECs0357]
ECs0376	+2.32	7.41	+1.21	6.80	0.3	8.02	0.2	0.00758878	0.03323442	0.05314977	0.57275154	A_07_P053917	ECs0376	cmr|putative ATP binding protein of a transporter [ECs0376]
ECs0423	-5.81	9.63	-2.54	10.90	0.1	8.36	0.1	0.00002632	0.00055700	0.00403455	0.20894737	A_07_P053991	ECs0423	cmr|5-aminolevulinate dehydratase [ECs0423]
ECs0440	-2.78	7.95	-1.47	8.68	0.2	7.21	0.2	0.00120874	0.00796582	0.03645262	0.52126249	A_07_P054001	ECs0440	cmr|aroM protein [ECs0440]
ECs0443	-2.23	10.35	-1.15	10.92	0.1	9.77	0.1	0.00185885	0.01101870	0.06813389	0.65890663	A_07_P054012	ECs0443	cmr|hypothetical protein [ECs0443]
ECs0447	+2.64	8.42	+1.40	7.72	0.0	9.13	0.2	0.00080655	0.00582985	0.04110264	0.50556250	A_07_P062959	ECs0447	cmr|ATP-dependent dsDNA exonuclease [ECs0447]
ECs0449	-7.02	11.44	-2.81	12.85	0.0	10.04	0.2	0.00001657	0.00042559	0.00226308	0.15765487	A_07_P054018	ECs0449	cmr|positive response regulator for pho regulon PhoB [ECs0449]
ECs0477	-4.21	8.89	-2.07	9.92	0.0	7.85	0.1	0.00006145	0.00091973	0.01066247	0.32787109	A_07_P054057	ECs0477	cmr|putative oxidoreductase [ECs0477]
ECs0483	+7.29	9.27	+2.87	7.83	0.2	10.70	0.4	0.00024095	0.00240098	0.00691311	0.23660870	A_07_P062971	ECs0483	cmr|cytochrome o ubiquinol oxidase subunit IV [ECs0483]
ECs0541	+2.37	8.24	+1.24	7.62	0.3	8.86	0.1	0.00419900	0.02067198	0.05051004	0.55649405	A_07_P054092	ECs0541	cmr|hypothetical protein [ECs0541]
ECs0549	-3.02	8.99	-1.59	9.78	0.1	8.19	0.4	0.00224603	0.01269257	0.02789507	0.48208562	A_07_P054128	ECs0549	cmr|hypothetical protein [ECs0549]
ECs0551	-2.39	10.85	-1.26	11.48	0.1	10.22	0.0	0.00075855	0.00557260	0.05613377	0.61715782	A_07_P054138	ECs0551	cmr|hypothetical protein [ECs0551]
ECs0573	+3.06	7.72	+1.61	6.92	0.0	8.53	0.1	0.00020268	0.00212689	0.03178544	0.45287783	A_07_P054146	ECs0573	cmr|allantoinase [ECs0573]
ECs0679	-2.21	10.44	-1.15	11.01	0.0	9.87	0.0	0.00094235	0.00659689	0.07022167	0.66241462	A_07_P054223	ECs0679	cmr|minor lipoprotein [ECs0679]
ECs0694	+4.51	11.61	+2.17	10.52	0.2	12.69	0.3	0.00027695	0.00263619	0.01658791	0.34272966	A_07_P056560	ECs0694	cmr|putative periplasmic binding transport protein [ECs0694]
ECs0711	-2.84	10.06	-1.51	10.81	0.1	9.31	0.0	0.00041167	0.00349775	0.03479675	0.51200000	A_07_P056574	ECs0711	cmr|hypothetical protein [ECs0711]
ECs0737	+3.24	11.45	+1.69	10.60	0.1	12.30	0.2	0.00045594	0.00377804	0.02887830	0.43843780	A_07_P063012	ECs0737	cmr|putative carboxylase [ECs0737]
ECs0772	-2.32	10.29	-1.22	10.90	0.0	9.69	0.1	0.00094990	0.00662910	0.06173082	0.63730492	A_07_P056669	ECs0772	cmr|inner membrane protein TolQ [ECs0772]
ECs0778	-3.36	8.63	-1.75	9.50	0.1	7.75	0.1	0.00020884	0.00217593	0.02148310	0.44270942	A_07_P056671	ECs0778	cmr|quinolinate synthetase A protein [ECs0778]
ECs0805	+7.84	9.12	+2.97	7.63	0.0	10.60	0.3	0.00005388	0.00084214	0.00598514	0.22276596	A_07_P056700	ECs0805	cmr|hypothetical C4-type zinc finger protein TraR-family [ECs0805]
ECs0807	+2.45	8.50	+1.29	7.85	0.0	9.14	0.1	0.00076318	0.00559124	0.04702998	0.54007294	A_07_P056708	ECs0807	cmr|hypothetical protein [ECs0807]
ECs0809	-3.86	10.82	-1.95	11.80	0.1	9.85	0.2	0.00024396	0.00242329	0.01432038	0.37828859	A_07_P056720	ECs0809	cmr|exonuclease [ECs0809]
ECs0810	+3.46	9.05	+1.79	8.15	0.1	9.94	0.1	0.00011812	0.00145366	0.02594131	0.41590631	A_07_P056725	ECs0810	cmr|NinE protein [ECs0810]
ECs0819	+7.03	9.39	+2.81	7.99	0.1	10.80	0.2	0.00004461	0.00074958	0.00755335	0.24621118	A_07_P056765	ECs0819	cmr|endolysin [ECs0819]
ECs0820	+4.42	7.92	+2.14	6.85	0.1	9.00	0.2	0.00010702	0.00135989	0.01705285	0.34822309	A_07_P056766	ECs0820	cmr|putative endopeptidase [ECs0820]
ECs0825	-2.93	8.63	-1.55	9.41	0.2	7.86	0.0	0.00038642	0.00333358	0.03162221	0.49195652	A_07_P056791	ECs0825	cmr|putative terminase large subunit [ECs0825]
ECs0826	-2.03	7.61	-1.02	8.12	0.0	7.10	0.2	0.00365094	0.01861791	0.08640943	0.71413648	A_07_P056799	ECs0826	cmr|hypothetical protein [ECs0826]
ECs0835	+2.02	7.12	+1.01	6.61	0.1	7.63	0.1	0.00323704	0.01692654	0.06929433	0.65879831	A_07_P056842	ECs0835	cmr|putative minor tail protein [ECs0835]
ECs0837	+2.10	8.84	+1.07	8.30	0.1	9.37	0.2	0.00346425	0.01781811	0.06275915	0.62655675	A_07_P056851	ECs0837	cmr|putative tail length tape measure protein precursor [ECs0837]
ECs0840	+5.39	9.33	+2.43	8.11	0.2	10.55	0.2	0.00007588	0.00107529	0.01222688	0.29344512	A_07_P056866	ECs0840	cmr|putative tail assembly protein [ECs0840]
ECs0870	+2.26	7.93	+1.18	7.35	0.2	8.52	0.3	0.00870085	0.03706337	0.05777566	0.59922266	A_07_P056925	ECs0870	cmr|hypothetical protein [ECs0870]
ECs0878	+2.01	9.88	+1.01	9.38	0.0	10.39	0.1	0.00211648	0.01213470	0.06885226	0.65578342	A_07_P056933	ECs0878	cmr|putative enzyme [ECs0878]
ECs0901	+2.40	7.26	+1.26	6.62	0.1	7.89	0.1	0.00150658	0.00937163	0.04834413	0.54757554	A_07_P056937	ECs0901	cmr|hypothetical protein [ECs0901]
ECs0906	+4.65	11.10	+2.22	9.99	0.2	12.20	0.0	0.00008760	0.00118786	0.01592099	0.33645638	A_07_P056943	ECs0906	cmr|hypothetical protein [ECs0906]
ECs0950	-3.38	11.14	-1.76	12.02	0.1	10.26	0.2	0.00023233	0.00234924	0.02025597	0.43154263	A_07_P056991	ECs0950	cmr|hypothetical protein [ECs0950]
ECs0952	+2.35	8.76	+1.23	8.15	0.1	9.38	0.2	0.00252873	0.01390579	0.05138465	0.56258693	A_07_P057004	ECs0952	cmr|hypothetical protein [ECs0952]
ECs0955	+4.74	8.39	+2.25	7.27	0.1	9.52	0.1	0.00005592	0.00086270	0.01533155	0.33293793	A_07_P063047	ECs0955	cmr|putative dTDP-glucose enzyme [ECs0955]
ECs0981	+6.65	8.60	+2.73	7.23	0.2	9.96	0.1	0.00003040	0.00060429	0.00824949	0.25317739	A_07_P063053	ECs0981	cmr|anaerobic dimethyl sulfoxide reductase subunit C [ECs0981]
ECs1011	+5.69	9.85	+2.51	8.59	0.1	11.10	0.2	0.00005029	0.00080433	0.01120427	0.28269231	A_07_P063067	ECs1011	cmr|aspartate aminotransferase [ECs1011]
ECs1015	+14.76	9.98	+3.88	8.04	0.0	11.92	0.0	0.00000167	0.00015927	0.00164571	0.12338095	A_07_P063074	ECs1015	cmr|aminopeptidase N [ECs1015]
ECs1081	-5.42	8.89	-2.44	10.11	0.1	7.68	0.2	0.00004634	0.00076454	0.00505780	0.22882184	A_07_P057173	ECs1081	cmr|hypothetical protein [ECs1081]
ECs1092	+2.69	10.76	+1.43	10.05	0.3	11.48	0.2	0.00221684	0.01257272	0.04024898	0.50172605	A_07_P057221	ECs1092	cmr|putative lipoprotein Rz1 protein precursor [ECs1092]
ECs1101	-2.65	10.30	-1.41	11.00	0.3	9.59	0.1	0.00171824	0.01040141	0.04296557	0.56323897	A_07_P057264	ECs1101	cmr|hypothetical protein [ECs1101]
ECs1105	-3.34	12.11	-1.74	12.98	0.1	11.24	0.2	0.00032087	0.00294563	0.02195185	0.44826200	A_07_P057269	ECs1105	cmr|putative terminase small subunit [ECs1105]
ECs1106	+2.19	8.71	+1.13	8.15	0.0	9.28	0.1	0.00110177	0.00743837	0.05737297	0.59622442	A_07_P057274	ECs1106	cmr|putative terminase large subunit [ECs1106]
ECs1107	+2.69	7.95	+1.43	7.24	0.0	8.66	0.2	0.00105630	0.00718068	0.03986153	0.50006375	A_07_P057276	ECs1107	cmr|putative portal protein [ECs1107]
ECs1108	+19.68	10.21	+4.30	8.06	0.2	12.36	0.0	0.00000235	0.00017963	0.00077871	0.08455172	A_07_P057281	ECs1108	cmr|putative head-tail preconnector protein [ECs1108]
ECs1109	+21.40	9.88	+4.42	7.67	0.3	12.09	0.0	0.00000367	0.00020738	0.00066629	0.07828358	A_07_P057290	ECs1109	cmr|putative head decoration protein [ECs1109]
ECs1110	+12.26	8.65	+3.62	6.84	0.0	10.46	0.0	0.00000232	0.00017963	0.00239075	0.14421456	A_07_P057293	ECs1110	cmr|putative major head protein [ECs1110]
ECs1156	-7.64	9.50	-2.93	10.97	0.0	8.04	0.2	0.00001754	0.00043403	0.00172891	0.13542289	A_07_P061921	ECs1156	cmr|hypothetical protein [ECs1156]
ECs1160	-3.25	10.14	-1.70	10.99	0.0	9.29	0.5	0.00335107	0.01737166	0.02436357	0.45937725	A_07_P061927	ECs1160	cmr|putative integrase [ECs1160]
ECs1160	-2.80	9.85	-1.49	10.60	0.1	9.11	0.1	0.00039363	0.00338098	0.03610010	0.51952468	A_07_P061927	ECs1160	cmr|putative integrase [ECs1160]
ECs1160	-2.36	10.04	-1.24	10.66	0.1	9.42	0.4	0.00897856	0.03799959	0.05542937	0.61283708	A_07_P061927	ECs1160	cmr|putative integrase [ECs1160]
ECs1160	-2.38	9.83	-1.25	10.46	0.3	9.21	0.3	0.00666418	0.02999453	0.05559007	0.61332165	A_07_P061927	ECs1160	cmr|putative integrase [ECs1160]
ECs1161	-2.14	8.23	-1.10	8.78	0.1	7.68	0.0	0.00136182	0.00871917	0.07629764	0.67371284	A_07_P061935	ECs1161	cmr|putative excisionase [ECs1161]
ECs1163	+3.28	7.99	+1.71	7.13	0.0	8.84	0.0	0.00013214	0.00157032	0.02792937	0.43109804	A_07_P061944	ECs1163	cmr|hypothetical protein [ECs1163]
ECs1164	-2.83	7.85	-1.50	8.60	0.1	7.10	0.1	0.00034334	0.00307905	0.03544588	0.51434101	A_07_P061948	ECs1164	cmr|hypothetical protein [ECs1164]
ECs1170	+2.40	8.30	+1.26	7.66	0.2	8.93	0.3	0.00626438	0.02847393	0.04975419	0.55359011	A_07_P061980	ECs1170	cmr|C4-type zinc finger protein [ECs1170]
ECs1176	+2.24	11.05	+1.16	10.47	0.1	11.63	0.1	0.00129494	0.00840722	0.05553671	0.58682550	A_07_P062008	ECs1176	cmr|host-nuclease inhibitor protein Gam [ECs1176]
ECs1177	-6.09	12.32	-2.61	13.63	0.0	11.02	0.0	0.00001387	0.00039022	0.00349276	0.19780576	A_07_P062011	ECs1177	cmr|Kil protein [ECs1177]
ECs1181	-35.59	12.78	-5.15	15.36	0.1	10.20	0.1	0.00000047	0.00009262	0.00000572	0.00642857	A_07_P062031	ECs1181	cmr|putative anti-termination protein N [ECs1181]
ECs1182	-23.24	11.77	-4.54	14.04	0.0	9.50	0.2	0.00000159	0.00015888	0.00003684	0.01757576	A_07_P062036	ECs1182	cmr|hypothetical protein [ECs1182]
ECs1190	-2.26	7.82	-1.17	8.41	0.0	7.23	0.1	0.00114787	0.00766414	0.06631542	0.65623507	A_07_P062075	ECs1190	cmr|replication protein P [ECs1190]
ECs1194	-2.59	9.33	-1.37	10.02	0.0	8.64	0.0	0.00041639	0.00352830	0.04531949	0.57172276	A_07_P062091	ECs1194	cmr|hypothetical protein [ECs1194]
ECs1197	+6.99	8.43	+2.81	7.02	0.0	9.83	0.2	0.00002561	0.00055176	0.00751969	0.24562241	A_07_P062106	ECs1197	cmr|NinE protein [ECs1197]
ECs1199	+2.05	7.29	+1.03	6.78	0.0	7.81	0.1	0.00186648	0.01105148	0.06520833	0.63806091	A_07_P062119	ECs1199	cmr|putative antirepressor protein [ECs1199]
ECs1202	-3.05	10.50	-1.61	11.30	0.3	9.69	0.1	0.00169934	0.01032189	0.02784870	0.48234323	A_07_P062135	ECs1202	cmr|hypothetical protein [ECs1202]
ECs1205	-2.99	8.91	-1.58	9.70	0.1	8.12	0.1	0.00042844	0.00360716	0.03036903	0.49089322	A_07_P062148	ECs1205	cmr|Shiga toxin 2 subunit A [ECs1205]
ECs1219	-3.32	8.69	-1.73	9.56	0.0	7.83	0.0	0.00011753	0.00144794	0.02186992	0.44775033	A_07_P062208	ECs1219	cmr|putative small subunit terminase [ECs1219]
ECs1240	-2.18	9.49	-1.12	10.05	0.1	8.92	0.3	0.00771854	0.03369958	0.07350356	0.67086377	A_07_P062314	ECs1240	cmr|hypothetical protein [ECs1240]
ECs1249	-3.05	11.85	-1.61	12.66	0.2	11.05	0.1	0.00075926	0.00557283	0.02859629	0.48358754	A_07_P062357	ECs1249	cmr|hypothetical protein [ECs1249]
ECs1299	-2.72	10.97	-1.44	11.69	0.4	10.25	0.0	0.00408922	0.02030935	0.03936674	0.53988676	A_07_P062501	ECs1299	cmr|putative integrase [ECs1299]
ECs1300	+17.39	9.81	+4.12	7.75	0.2	11.87	0.2	0.00000924	0.00032221	0.00115600	0.10111111	A_07_P062509	ECs1300	cmr|putative membrane protein [ECs1300]
ECs1305	-3.30	12.89	-1.72	13.75	0.6	12.02	0.3	0.00867101	0.03696495	0.02202680	0.44804910	A_07_P062533	ECs1305	cmr|hypothetical protein [ECs1305]
ECs1310	+4.13	9.62	+2.05	8.59	0.0	10.64	0.0	0.00004956	0.00079780	0.01909426	0.37022167	A_07_P062546	ECs1310	cmr|IS30 transposase [ECs1310]
ECs1338	-3.03	9.79	-1.60	10.59	0.1	8.99	0.2	0.00054191	0.00430246	0.02892403	0.48444681	A_07_P062685	ECs1338	cmr|hypothetical protein [ECs1338]
ECs1339	-2.53	8.90	-1.34	9.57	0.3	8.23	0.3	0.00610202	0.02787065	0.04846989	0.58119573	A_07_P062686	ECs1339	cmr|hypothetical protein [ECs1339]
ECs1359	-3.13	8.07	-1.65	8.90	0.1	7.25	0.1	0.00018884	0.00202661	0.02637132	0.47450286	A_07_P060342	ECs1359	cmr|hypothetical protein [ECs1359]
ECs1363	-14.32	9.83	-3.84	11.75	0.1	7.91	0.3	0.00001779	0.00043523	0.00019944	0.04906250	A_07_P060356	ECs1363	cmr|hypothetical protein [ECs1363]
ECs1369	-2.43	7.73	-1.28	8.37	0.1	7.09	0.2	0.00139283	0.00886726	0.05450648	0.60861702	A_07_P060383	ECs1369	cmr|hypothetical protein [ECs1369]
ECs1375	-2.60	8.83	-1.38	9.52	0.0	8.14	0.2	0.00112006	0.00752635	0.04543128	0.57130192	A_07_P060402	ECs1375	cmr|hypothetical protein [ECs1375]
ECs1380	-9.11	9.91	-3.19	11.50	0.1	8.32	0.5	0.00013251	0.00157122	0.00092416	0.10034483	A_07_P060427	ECs1380	cmr|putative transposase OrfB protein of insertion sequence IS629 [ECs1380]
ECs1381	-3.12	9.82	-1.64	10.65	0.1	9.00	0.1	0.00026453	0.00255503	0.02639291	0.47380844	A_07_P060432	ECs1381	cmr|putative transposase OrfA protein of insertion sequence IS629 [ECs1381]
ECs1382	-2.22	7.84	-1.15	8.41	0.0	7.26	0.4	0.00742754	0.03266458	0.06623793	0.65670655	A_07_P060439	ECs1382	cmr|HecB-like protein [ECs1382]
ECs1384	+2.03	7.86	+1.02	7.35	0.0	8.37	0.0	0.00170269	0.01033706	0.06667302	0.64398773	A_07_P060446	ECs1384	cmr|hypothetical protein [ECs1384]
ECs1392	-6.03	8.75	-2.59	10.05	0.0	7.45	0.3	0.00010434	0.00134216	0.00370681	0.20124138	A_07_P060482	ECs1392	cmr|hypothetical protein [ECs1392]
ECs1394	-2.49	8.02	-1.32	8.68	0.2	7.37	0.1	0.00122189	0.00803675	0.05135607	0.60115242	A_07_P060493	ECs1394	cmr|hypothetical protein [ECs1394]
ECs1395	-2.41	7.69	-1.27	8.32	0.1	7.05	0.2	0.00269561	0.01461927	0.05403328	0.60764286	A_07_P060497	ECs1395	cmr|hypothetical protein [ECs1395]
ECs1445	+3.27	8.17	+1.71	7.31	0.0	9.02	0.1	0.00017246	0.00190945	0.02799035	0.43161606	A_07_P063092	ECs1445	cmr|hypothetical protein [ECs1445]
ECs1498	+2.62	9.01	+1.39	8.31	0.3	9.70	0.1	0.00268094	0.01454471	0.04226245	0.51065234	A_07_P063109	ECs1498	cmr|putative nicotinic acid mononucleotide:5,6-dimethylbenzimidazole [ECs1498]
ECs1511	-3.73	12.03	-1.90	12.98	0.1	11.08	0.3	0.00043975	0.00367873	0.01573869	0.39331746	A_07_P060633	ECs1511	cmr|hypothetical protein [ECs1511]
ECs1526	-8.76	8.39	-3.13	9.95	0.0	6.82	0.2	0.00001025	0.00033697	0.00109375	0.10564417	A_07_P060699	ECs1526	cmr|hypothetical protein [ECs1526]
ECs1537	-5.03	11.47	-2.33	12.64	0.1	10.31	0.9	0.00772128	0.03370219	0.00675368	0.26716080	A_07_P060748	ECs1537	cmr|hypothetical protein [ECs1537]
ECs1544	-3.21	8.74	-1.68	9.58	0.3	7.90	0.1	0.00124183	0.00814642	0.02428989	0.45963942	A_07_P060776	ECs1544	cmr|putative portal protein [ECs1544]
ECs1558	+2.21	8.54	+1.14	7.97	0.3	9.11	0.1	0.00680985	0.03049327	0.05949187	0.61178315	A_07_P060837	ECs1558	cmr|putative tail assembly protein [ECs1558]
ECs1567	-2.03	9.03	-1.02	9.54	0.2	8.52	0.0	0.00341048	0.01757023	0.08591590	0.71418163	A_07_P060877	ECs1567	cmr|hypothetical protein [ECs1567]
ECs1575	-2.37	7.94	-1.25	8.56	0.1	7.31	0.4	0.00774412	0.03378315	0.05790587	0.62230034	A_07_P060898	ECs1575	cmr|putative DNA binding protein [ECs1575]
ECs1595	-7.86	10.77	-2.97	12.25	0.3	9.28	0.8	0.00232236	0.01303965	0.00144881	0.12602210	A_07_P061000	ECs1595	cmr|hypothetical protein [ECs1595]
ECs1607	-10.52	13.01	-3.39	14.71	0.0	11.31	0.1	0.00000354	0.00020330	0.00065676	0.08338710	A_07_P061030	ECs1607	cmr|hypothetical protein [ECs1607]
ECs1608	+4.06	8.66	+2.02	7.65	0.1	9.67	0.2	0.00018841	0.00202485	0.01985582	0.37573317	A_07_P061034	ECs1608	cmr|isocitrate dehydrogenase [ECs1608]
ECs1610	+6.65	8.73	+2.73	7.37	0.3	10.10	0.3	0.00022493	0.00230251	0.00815866	0.25087891	A_07_P061042	ECs1610	cmr|excisionase [ECs1610]
ECs1623	+6.48	8.80	+2.70	7.45	0.4	10.15	0.2	0.00033781	0.00304380	0.00848895	0.25751445	A_07_P061089	ECs1623	cmr|putative endopeptidase [ECs1623]
ECs1638	+20.92	10.23	+4.39	8.04	0.1	12.43	0.1	0.00000137	0.00014976	0.00071900	0.08202899	A_07_P061143	ECs1638	cmr|minor tail protein [ECs1638]
ECs1639	+3.64	10.86	+1.86	9.93	0.0	11.80	0.2	0.00017976	0.00195827	0.02336382	0.39982609	A_07_P061148	ECs1639	cmr|minor tail protein [ECs1639]
ECs1640	+13.34	9.11	+3.74	7.24	0.1	10.98	0.1	0.00000259	0.00018134	0.00205348	0.13304527	A_07_P061154	ECs1640	cmr|major tail protein [ECs1640]
ECs1641	+13.94	9.07	+3.80	7.17	0.0	10.98	0.2	0.00000584	0.00025989	0.00180132	0.12548673	A_07_P061159	ECs1641	cmr|minor tail protein [ECs1641]
ECs1642	+3.80	7.81	+1.92	6.85	0.0	8.78	0.0	0.00006813	0.00099497	0.02212716	0.39186727	A_07_P061162	ECs1642	cmr|minor tail protein [ECs1642]
ECs1643	+3.44	9.75	+1.78	8.86	0.0	10.64	0.2	0.00019527	0.00207030	0.02610899	0.41689655	A_07_P061170	ECs1643	cmr|tail length tape measure protein precursor [ECs1643]
ECs1644	+3.44	9.60	+1.78	8.71	0.2	10.49	0.1	0.00037413	0.00325950	0.02612297	0.41669706	A_07_P061173	ECs1644	cmr|minor tail protein [ECs1644]
ECs1645	+2.62	9.39	+1.39	8.70	0.2	10.09	0.1	0.00114181	0.00763667	0.04150915	0.50699767	A_07_P061180	ECs1645	cmr|minor tail protein [ECs1645]
ECs1666	-7.12	9.40	-2.83	10.82	0.2	7.98	0.2	0.00003052	0.00060552	0.00218051	0.15747706	A_07_P058060	ECs1666	cmr|putative transposase OrfB protein of insertion sequence IS629 [ECs1666]
ECs1711	-3.83	8.44	-1.94	9.41	0.2	7.47	0.0	0.00025217	0.00248136	0.01408854	0.37594915	A_07_P058136	ECs1711	cmr|hypothetical protein [ECs1711]
ECs1724	+2.49	9.12	+1.32	8.47	0.0	9.78	0.0	0.00047510	0.00388371	0.04521151	0.53120149	A_07_P058147	ECs1724	cmr|hypothetical protein [ECs1724]
ECs1726	+3.96	11.24	+1.99	10.24	0.1	12.23	0.4	0.00104612	0.00714226	0.02096672	0.38294664	A_07_P063128	ECs1726	cmr|nitrate/nitrite response regulator protein NarL [ECs1726]
ECs1743	+312.18	12.95	+8.29	8.81	0.3	17.09	0.0	0.00000014	0.00007519	0.00000000	0.00000000	A_07_P063132	ECs1743	cmr|oligopeptide transport periplasmic binding protein [ECs1743]
ECs1751	+13.34	10.43	+3.74	8.56	0.0	12.30	0.0	0.00000192	0.00016889	0.00203061	0.13320833	A_07_P058156	ECs1751	cmr|hypothetical protein [ECs1751]
ECs1769	+2.74	8.21	+1.46	7.48	0.1	8.94	0.0	0.00036146	0.00318459	0.03855755	0.49393816	A_07_P058224	ECs1769	cmr|putative phage replication protein [ECs1769]
ECs1779	-2.31	8.54	-1.21	9.14	0.1	7.94	0.4	0.00769197	0.03359288	0.06296240	0.64243681	A_07_P058272	ECs1779	cmr|hypothetical lipoprotein [ECs1779]
ECs1780	-4.10	11.03	-2.03	12.04	0.0	10.01	0.5	0.00185562	0.01100785	0.01143547	0.34490421	A_07_P058276	ECs1780	cmr|putative DNA methylase [ECs1780]
ECs1795	-2.76	8.80	-1.46	9.53	0.2	8.07	0.2	0.00149592	0.00932371	0.03869792	0.53490781	A_07_P058326	ECs1795	cmr|putative portal protein [ECs1795]
ECs1798	-3.65	9.59	-1.87	10.52	0.2	8.65	0.6	0.00744681	0.03272189	0.01583587	0.39201258	A_07_P058341	ECs1798	cmr|hypothetical protein [ECs1798]
ECs1805	-2.03	9.41	-1.02	9.92	0.0	8.90	0.3	0.00732375	0.03230128	0.08827045	0.72081432	A_07_P058367	ECs1805	cmr|minor tail protein [ECs1805]
ECs1811	-2.45	7.88	-1.30	8.53	0.0	7.24	0.0	0.00054557	0.00431851	0.05307546	0.60203170	A_07_P058395	ECs1811	cmr|hypothetical protein [ECs1811]
ECs1827	-6.76	8.94	-2.76	10.32	0.0	7.56	0.1	0.00001432	0.00039410	0.00257177	0.17084388	A_07_P058453	ECs1827	cmr|IS630 hypothetical protein [ECs1827]
ECs1833	+111.74	11.07	+6.80	7.67	0.1	14.47	0.1	0.00000009	0.00007032	0.00001588	0.01000000	A_07_P058463	ECs1833	cmr|tryptophan synthase beta protein [ECs1833]
ECs1836	+3.78	9.96	+1.92	9.00	0.0	10.92	0.4	0.00069902	0.00523073	0.02250635	0.39414905	A_07_P058466	ECs1836	cmr|anthranilate synthase component I [ECs1836]
ECs1839	-3.45	8.67	-1.79	9.57	0.1	7.78	0.3	0.00074604	0.00550667	0.01888211	0.41988701	A_07_P058472	ECs1839	cmr|hypothetical protein [ECs1839]
ECs1839	-3.39	8.50	-1.76	9.38	0.0	7.62	0.1	0.00012413	0.00150779	0.02040968	0.43305930	A_07_P058472	ECs1839	cmr|hypothetical protein [ECs1839]
ECs1839	-3.68	8.75	-1.88	9.69	0.0	7.81	0.0	0.00007855	0.00110119	0.01585429	0.39123824	A_07_P058472	ECs1839	cmr|hypothetical protein [ECs1839]
ECs1839	-3.55	8.66	-1.83	9.57	0.0	7.75	0.1	0.00015686	0.00177542	0.01810023	0.41002878	A_07_P058472	ECs1839	cmr|hypothetical protein [ECs1839]
ECs1839	-4.48	8.62	-2.16	9.70	0.0	7.53	0.1	0.00004055	0.00070151	0.00886306	0.30203463	A_07_P058472	ECs1839	cmr|hypothetical protein [ECs1839]
ECs1889	-2.16	8.57	-1.11	9.12	0.0	8.01	0.2	0.00206717	0.01193892	0.07500635	0.67287749	A_07_P063152	ECs1889	cmr|hypothetical protein [ECs1889]
ECs1900	-4.96	11.68	-2.31	12.83	0.0	10.52	0.8	0.00443744	0.02159601	0.00694233	0.26987654	A_07_P058536	ECs1900	cmr|putative enzyme [ECs1900]
ECs1906	+6.74	9.27	+2.75	7.90	0.2	10.65	0.1	0.00002441	0.00053382	0.00811357	0.25047059	A_07_P058547	ECs1906	cmr|hypothetical protein [ECs1906]
ECs1930	-3.53	9.82	-1.82	10.73	0.0	8.91	0.4	0.00091902	0.00647676	0.01777503	0.40794461	A_07_P058575	ECs1930	cmr|hypothetical protein [ECs1930]
ECs1930	-5.92	9.86	-2.57	11.14	0.4	8.58	1.0	0.01103701	0.04494741	0.00381606	0.20297297	A_07_P058575	ECs1930	cmr|hypothetical protein [ECs1930]
ECs1930	-5.57	9.77	-2.48	11.01	0.8	8.53	0.4	0.00721182	0.03192993	0.00433880	0.21346875	A_07_P058575	ECs1930	cmr|hypothetical protein [ECs1930]
ECs1930	-3.44	9.28	-1.78	10.17	0.2	8.39	0.1	0.00036883	0.00323294	0.01926893	0.41959889	A_07_P058575	ECs1930	cmr|hypothetical protein [ECs1930]
ECs1948	-2.40	9.37	-1.26	10.00	0.1	8.73	0.4	0.00797493	0.03459833	0.05322091	0.60324694	A_07_P058616	ECs1948	cmr|hypothetical protein [ECs1948]
ECs1951	+4.30	8.23	+2.10	7.18	0.2	9.28	0.1	0.00016836	0.00187935	0.01802846	0.36066074	A_07_P058631	ECs1951	cmr|hypothetical protein [ECs1951]
ECs1975	-2.72	7.58	-1.44	8.30	0.1	6.86	0.1	0.00046715	0.00384264	0.03988059	0.54361905	A_07_P058702	ECs1975	cmr|hypothetical protein [ECs1975]
ECs1981	-2.30	10.73	-1.20	11.33	0.3	10.13	0.3	0.00916001	0.03859818	0.06411839	0.64834939	A_07_P058732	ECs1981	cmr|putative tail assembly chaperon [ECs1981]
ECs1990	-2.35	9.45	-1.23	10.06	0.1	8.83	0.3	0.00564515	0.02618655	0.05712271	0.61852820	A_07_P058780	ECs1990	cmr|putative host specificity protein [ECs1990]
ECs2019	-2.68	10.62	-1.42	11.33	0.4	9.90	0.0	0.00563256	0.02614359	0.04027630	0.54523646	A_07_P054232	ECs2019	cmr|hypothetical protein [ECs2019]
ECs2032	+2.64	8.67	+1.40	7.97	0.0	9.37	0.2	0.00105918	0.00719405	0.04139291	0.50636364	A_07_P054237	ECs2032	cmr|ribosomal-protein-serine N-acetyltransferase [ECs2032]
ECs2033	-2.84	8.91	-1.50	9.66	0.0	8.16	0.0	0.00024880	0.00245518	0.03523946	0.51418906	A_07_P063179	ECs2033	cmr|hypothetical protein [ECs2033]
ECs2049	-2.62	7.57	-1.39	8.27	0.1	6.88	0.1	0.00078883	0.00573111	0.04384527	0.56674877	A_07_P058915	ECs2049	cmr|hypothetical protein [ECs2049]
ECs2112	-2.32	7.95	-1.21	8.55	0.1	7.34	0.3	0.00482535	0.02304224	0.06201474	0.63856115	A_07_P054315	ECs2112	cmr|putative fimbrial chaperone protein precursor [ECs2112]
ECs2128	+3.49	9.93	+1.80	9.03	0.0	10.83	0.1	0.00012836	0.00154386	0.02500000	0.40957336	A_07_P063195	ECs2128	cmr|altronate oxidoreductase [ECs2128]
ECs2154	+2.35	7.85	+1.23	7.23	0.2	8.47	0.0	0.00142140	0.00899458	0.05094195	0.55890592	A_07_P054377	ECs2154	cmr|hypothetical protein [ECs2154]
ECs2161	-2.16	8.41	-1.11	8.96	0.1	7.86	0.1	0.00199667	0.01160412	0.07580602	0.67466930	A_07_P054412	ECs2161	cmr|putative host specificity protein [ECs2161]
ECs2169	-8.13	10.54	-3.02	12.05	0.6	9.03	0.1	0.00067398	0.00509417	0.00127287	0.11517241	A_07_P054447	ECs2169	cmr|hypothetical protein [ECs2169]
ECs2183	+3.71	8.96	+1.89	8.01	0.1	9.91	0.5	0.00282628	0.01516081	0.02364710	0.40118534	A_07_P054491	ECs2183	cmr|putative lipoprotein Rz1 precursor [ECs2183]
ECs2184	+4.31	8.10	+2.11	7.05	0.0	9.15	0.2	0.00007958	0.00110776	0.01807292	0.36063371	A_07_P054498	ECs2184	cmr|putative endopeptidase [ECs2184]
ECs2216	-2.01	7.83	-1.00	8.34	0.2	7.33	0.3	0.01223359	0.04861326	0.09220592	0.72729960	A_07_P054644	ECs2216	cmr|putative exonuclease [ECs2216]
ECs2234	+2.04	7.74	+1.03	7.22	0.0	8.25	0.0	0.00162109	0.00995416	0.06617696	0.64234895	A_07_P054691	ECs2234	cmr|putative host specificity protein [ECs2234]
ECs2277	-2.00	9.84	-1.00	10.34	0.1	9.35	0.0	0.00213939	0.01222990	0.09162475	0.72635448	A_07_P054803	ECs2277	cmr|hypothetical protein [ECs2277]
ECs2280	+2.87	12.61	+1.52	11.85	0.1	13.37	0.1	0.00037865	0.00328517	0.03561992	0.47768313	A_07_P054816	ECs2280	cmr|hypothetical protein [ECs2280]
ECs2297	+2.47	8.45	+1.31	7.80	0.3	9.11	0.0	0.00310103	0.01634504	0.04629510	0.53672312	A_07_P054856	ECs2297	cmr|putative oxidoreductase component [ECs2297]
ECs2319	+2.48	8.49	+1.31	7.84	0.3	9.15	0.1	0.00317358	0.01666050	0.04617632	0.53732446	A_07_P054872	ECs2319	cmr|mannose-6-phosphate isomerase [ECs2319]
ECs2356	+3.26	9.85	+1.70	9.00	0.0	10.70	0.1	0.00015915	0.00179363	0.02834667	0.43583008	A_07_P054895	ECs2356	cmr|hypothetical protein [ECs2356]
ECs2417	+2.33	8.98	+1.22	8.37	0.0	9.59	0.2	0.00138309	0.00882311	0.05183626	0.56439142	A_07_P054897	ECs2417	cmr|vitamin B12 transport [ECs2417]
ECs2431	+8.07	9.20	+3.01	7.69	0.2	10.70	0.1	0.00003647	0.00065813	0.00568725	0.22000000	A_07_P054909	ECs2431	cmr|hypothetical protein [ECs2431]
ECs2467	+6.79	9.84	+2.76	8.45	0.3	11.22	0.1	0.00006066	0.00091356	0.00794207	0.24760396	A_07_P063226	ECs2467	cmr|NADP-specific glutamate dehydrogenase [ECs2467]
ECs2471	+19.29	11.82	+4.27	9.69	0.2	13.96	0.1	0.00000306	0.00019257	0.00086954	0.08610063	A_07_P054922	ECs2471	cmr|hypothetical protein [ECs2471]
ECs2477	-3.93	9.02	-1.98	10.00	0.1	8.03	0.2	0.00015255	0.00173914	0.01325965	0.36496503	A_07_P054931	ECs2477	cmr|putative transposase OrfA protein of insertion sequence IS629 [ECs2477]

ECs2478	-3.88	8.17	-1.95	9.15	0.1	7.19	0.1	0.00012193	0.00148928	0.01393166	0.37494017	A_07_P054936	ECs2478	cmr|putative transposase OrfB protein of insertion sequence IS629 [ECs2478]
ECs2481	+2.18	7.68	+1.13	7.12	0.4	8.24	0.0	0.00941444	0.03949398	0.06127477	0.62159149	A_07_P054944	ECs2481	cmr|putative kinase [ECs2481]
ECs2495	-2.85	9.29	-1.51	10.04	0.1	8.53	0.3	0.00191568	0.01126632	0.03324378	0.50325962	A_07_P054951	ECs2495	cmr|hypothetical protein [ECs2495]
ECs2526	+6.51	8.68	+2.70	7.32	0.3	10.03	0.1	0.00006308	0.00093948	0.00867696	0.25971483	A_07_P054984	ECs2526	cmr|hypothetical protein [ECs2526]
ECs2555	-4.40	10.52	-2.14	11.59	0.2	9.45	0.4	0.00069650	0.00521681	0.00918636	0.30707006	A_07_P063252	ECs2555	cmr|protease II [ECs2555]
ECs2622	-2.21	7.56	-1.14	8.13	0.0	6.99	0.1	0.00154863	0.00956658	0.07091908	0.66540524	A_07_P055028	ECs2622	cmr|putative DNA-binding protein [ECs2622]
ECs2646	-2.28	9.49	-1.19	10.08	0.2	8.89	0.3	0.00478221	0.02288484	0.06278011	0.64224172	A_07_P055145	ECs2646	cmr|putative tail protein [ECs2646]
ECs2658	-3.52	9.96	-1.82	10.87	0.1	9.06	0.5	0.00262803	0.01434165	0.01908537	0.41907950	A_07_P055157	ECs2658	cmr|hypothetical protein [ECs2658]
ECs2700	-2.90	8.04	-1.54	8.81	0.0	7.28	0.1	0.00031707	0.00291758	0.03248031	0.49792600	A_07_P063287	ECs2700	cmr|hypothetical protein [ECs2700]
ECs2720	+3.58	8.77	+1.84	7.85	0.0	9.69	0.1	0.00013439	0.00158606	0.02400978	0.40171095	A_07_P055249	ECs2720	cmr|putative host specificity protein [ECs2720]
ECs2720	+3.13	8.70	+1.65	7.88	0.1	9.52	0.1	0.00029465	0.00275633	0.03028709	0.44357209	A_07_P055249	ECs2720	cmr|putative host specificity protein [ECs2720]
ECs2734	+3.21	10.48	+1.68	9.64	0.1	11.32	0.0	0.00014679	0.00168943	0.02900025	0.43775647	A_07_P055282	ECs2734	cmr|putative head completion protein [ECs2734]
ECs2771	-2.45	8.10	-1.30	8.75	0.3	7.46	0.1	0.00430199	0.02107899	0.05145198	0.60138085	A_07_P055443	ECs2771	cmr|hypothetical protein [ECs2771]
ECs2782	-7.07	8.75	-2.82	10.16	0.1	7.34	0.1	0.00001112	0.00034947	0.00225864	0.15875000	A_07_P055462	ECs2782	cmr|hypothetical protein [ECs2782]
ECs2792	-5.63	8.81	-2.49	10.06	0.0	7.56	0.1	0.00001871	0.00045100	0.00447154	0.21728395	A_07_P055475	ECs2792	cmr|hypothetical protein [ECs2792]
ECs2793	-3.63	8.67	-1.86	9.60	0.1	7.74	0.4	0.00127615	0.00831269	0.01664062	0.39695455	A_07_P055479	ECs2793	cmr|hypothetical protein [ECs2793]
ECs2795	-7.81	10.62	-2.96	12.10	0.1	9.14	0.1	0.00000951	0.00032326	0.00163618	0.13142857	A_07_P055487	ECs2795	cmr|putative transposase OrfB protein of insertion sequence IS629 [ECs2795]
ECs2825	+13.29	9.15	+3.73	7.28	0.2	11.01	0.2	0.00001236	0.00036795	0.00203252	0.13278008	A_07_P062811	ECs2825	cmr|N- [ECs2825]
ECs2827	+2.82	7.51	+1.50	6.76	0.1	8.26	0.1	0.00032402	0.00296307	0.03687881	0.48385000	A_07_P055553	ECs2827	cmr|phosphoribosyl-amp cyclohydrolase [ECs2827]
ECs2828	-2.12	8.98	-1.09	9.52	0.1	8.44	0.1	0.00188092	0.01111606	0.07615854	0.67323975	A_07_P055560	ECs2828	cmr|regulator of length of O-antigen component of lipopolysaccharide chains [ECs2828]
ECs2829	-4.15	8.71	-2.05	9.73	0.0	7.68	0.0	0.00005359	0.00084208	0.01134273	0.34408478	A_07_P055562	ECs2829	cmr|UDP-glucose 6-dehydrogenase [ECs2829]
ECs2835	+2.12	10.28	+1.08	9.74	0.1	10.82	0.2	0.00318154	0.01669672	0.06376651	0.63140881	A_07_P055581	ECs2835	cmr|phosphomannomutase [ECs2835]
ECs2845	-3.55	10.30	-1.83	11.22	0.0	9.39	0.1	0.00011089	0.00139667	0.01750889	0.40478708	A_07_P055631	ECs2845	cmr|putative glycosyl transferase [ECs2845]
ECs2885	-2.06	7.46	-1.04	7.98	0.2	6.93	0.1	0.00448461	0.02177843	0.08460239	0.70850000	A_07_P055701	ECs2885	cmr|putative transport protein [ECs2885]
ECs2905	-4.30	8.32	-2.10	9.38	0.1	7.27	0.1	0.00006060	0.00091356	0.00995998	0.31678788	A_07_P062818	ECs2905	cmr|hypothetical protein [ECs2905]
ECs2907	-2.08	8.99	-1.05	9.52	0.1	8.46	0.2	0.00427601	0.02097896	0.08271723	0.70242718	A_07_P055723	ECs2907	cmr|hydoxyethylthiazole kinase [ECs2907]
ECs2912	-2.05	9.97	-1.04	10.49	0.0	9.45	0.2	0.00427142	0.02096298	0.08618648	0.71341746	A_07_P055744	ECs2912	cmr|hypothetical protein [ECs2912]
ECs2923	+2.42	7.92	+1.27	7.28	0.4	8.56	0.0	0.00916167	0.03859818	0.04992378	0.55391121	A_07_P055776	ECs2923	cmr|interrupted molybdate metabolism regulator [ECs2923]
ECs2933	-2.74	9.38	-1.45	10.11	0.1	8.65	0.1	0.00038945	0.00335792	0.03851626	0.53474427	A_07_P055796	ECs2933	cmr|putative transposase OrfA protein of insertion sequence IS629 [ECs2933]
ECs2976	-2.57	9.68	-1.36	10.36	0.1	9.00	0.1	0.00088620	0.00628768	0.04673526	0.57439500	A_07_P052874	ECs2976	cmr|hypothetical protein [ECs2976]
ECs2978	-3.99	8.14	-2.00	9.14	0.1	7.14	0.3	0.00026608	0.00256374	0.01287602	0.35816254	A_07_P052883	ECs2978	cmr|hypothetical protein [ECs2978]
ECs2981	+2.76	7.24	+1.46	6.50	0.2	7.97	0.3	0.00254392	0.01397955	0.03819233	0.49206219	A_07_P052891	ECs2981	cmr|putative DNA methylase [ECs2981]
ECs2983	-2.48	7.28	-1.31	7.93	0.4	6.63	0.0	0.00724452	0.03203869	0.05072282	0.59462398	A_07_P052902	ECs2983	cmr|hypothetical protein [ECs2983]
ECs2998	-5.63	11.72	-2.49	12.97	0.0	10.47	0.0	0.00001736	0.00043241	0.00441756	0.21599379	A_07_P052976	ECs2998	cmr|Kil protein [ECs2998]
ECs3001	-2.05	11.56	-1.03	12.08	0.1	11.05	0.0	0.00199070	0.01159082	0.08480501	0.70944208	A_07_P052993	ECs3001	cmr|recombination protein Bet [ECs3001]
ECs3002	-3.63	10.13	-1.86	11.06	0.0	9.21	0.1	0.00011224	0.00140475	0.01685849	0.39733533	A_07_P053000	ECs3002	cmr|exonuclease [ECs3002]
ECs3003	+2.67	10.02	+1.42	9.31	0.1	10.72	0.1	0.00058709	0.00457583	0.04034934	0.50178515	A_07_P053004	ECs3003	cmr|hypothetical protein [ECs3003]
ECs3004	+2.47	8.38	+1.31	7.73	0.2	9.04	0.4	0.00830146	0.03577949	0.04777312	0.54424023	A_07_P053006	ECs3004	cmr|hypothetical protein [ECs3004]
ECs3005	+2.09	9.56	+1.06	9.03	0.3	10.09	0.2	0.01262762	0.04987975	0.07066756	0.66265039	A_07_P053011	ECs3005	cmr|hypothetical protein [ECs3005]
ECs3006	+3.61	7.69	+1.85	6.77	0.1	8.62	0.0	0.00011722	0.00144742	0.02376778	0.40150215	A_07_P053018	ECs3006	cmr|putative C4-type zinc finger protein [ECs3006]
ECs3020	+2.53	8.98	+1.34	8.31	0.5	9.65	0.0	0.00867517	0.03696495	0.04613186	0.53800000	A_07_P053058	ECs3020	cmr|D-lactate dehydrogenase [ECs3020]
ECs3029	-3.22	9.93	-1.69	10.78	0.6	9.09	0.3	0.00801408	0.03473944	0.02405805	0.45800484	A_07_P053079	ECs3029	cmr|putative isomerase [ECs3029]
ECs3042	+50.71	10.98	+5.66	8.15	0.1	13.81	0.8	0.00004993	0.00080150	0.00011878	0.02921875	A_07_P053100	ECs3042	cmr|galactose-binding transport protein [ECs3042]
ECs3098	+3.82	13.46	+1.93	12.50	0.1	14.43	0.1	0.00010699	0.00135989	0.02175622	0.38835601	A_07_P053126	ECs3098	cmr|ecotin, a serine protease inhibitor [ECs3098]
ECs3104	+2.53	10.45	+1.34	9.78	0.0	11.12	0.0	0.00046123	0.00380984	0.04401613	0.52104511	A_07_P053132	ECs3104	cmr|outer membrane protein C OmpC [ECs3104]
ECs3152	+2.16	8.74	+1.11	8.18	0.0	9.29	0.4	0.01001482	0.04153669	0.06143039	0.62196785	A_07_P053169	ECs3152	cmr|2-oxoglutarate decarboxylase [ECs3152]
ECs3160	+11.98	8.56	+3.58	6.77	0.1	10.35	0.4	0.00003177	0.00061949	0.00252033	0.14376812	A_07_P062826	ECs3160	cmr|NADH dehydrogenase I chain N [ECs3160]
ECs3163	+72.80	10.19	+6.19	7.10	0.1	13.29	0.1	0.00000015	0.00007760	0.00005716	0.02045455	A_07_P053173	ECs3163	cmr|NADH dehydrogenase I chain K [ECs3163]
ECs3167	+55.13	11.15	+5.78	8.26	0.1	14.05	0.1	0.00000022	0.00008757	0.00010290	0.02892857	A_07_P053176	ECs3167	cmr|NADH dehydrogenase I chain G [ECs3167]
ECs3169	+44.49	13.15	+5.48	10.41	0.2	15.89	0.1	0.00000103	0.00012946	0.00015180	0.03319444	A_07_P063350	ECs3169	cmr|NADH dehydrogenase I chain E [ECs3169]
ECs3179	+3.16	10.20	+1.66	9.37	0.0	11.03	0.2	0.00027025	0.00258336	0.02982660	0.43969101	A_07_P053181	ECs3179	cmr|hypothetical protein [ECs3179]
ECs3190	+2.59	7.52	+1.37	6.83	0.0	8.20	0.2	0.00070288	0.00524958	0.04247967	0.51131498	A_07_P053195	ECs3190	cmr|ATP-binding component of histidine transport [ECs3190]
ECs3196	+2.82	8.65	+1.49	7.90	0.0	9.40	0.0	0.00026145	0.00254717	0.03695313	0.48401830	A_07_P053197	ECs3196	cmr|amidophosphoribosyltransferase [ECs3196]
ECs3223	-2.98	8.47	-1.57	9.26	0.2	7.68	0.2	0.00093870	0.00657715	0.02966209	0.48747390	A_07_P053262	ECs3223	cmr|hypothetical protein [ECs3223]
ECs3236	-2.63	8.95	-1.40	9.65	0.4	8.25	0.3	0.00957824	0.03999996	0.04325838	0.56425849	A_07_P053303	ECs3236	cmr|putativa replication protein [ECs3236]
ECs3275	-3.03	9.67	-1.60	10.47	0.1	8.87	0.1	0.00037351	0.00325790	0.02880526	0.48400213	A_07_P053371	ECs3275	cmr|hypothetical protein [ECs3275]
ECs3296	-5.77	11.03	-2.53	12.30	0.1	9.77	0.2	0.00005143	0.00081548	0.00423590	0.21104430	A_07_P053399	ECs3296	cmr|thiosulfate binding protein [ECs3296]
ECs3306	-7.88	11.50	-2.98	12.99	0.0	10.01	0.2	0.00001679	0.00042622	0.00158918	0.13031250	A_07_P063373	ECs3306	cmr|N-acetylmuramoyl-l-alanine amidase I [ECs3306]
ECs3311	+2.72	7.53	+1.44	6.81	0.0	8.25	0.1	0.00033744	0.00304380	0.03894118	0.49562652	A_07_P053415	ECs3311	cmr|ethanolamine ammonia-lyase light chain [ECs3311]
ECs3382	-2.54	11.42	-1.35	12.10	0.1	10.75	0.1	0.00070691	0.00527472	0.04795287	0.57985407	A_07_P053440	ECs3382	cmr|putative anaerobic dimethyl sulfoxide reductase chain C [ECs3382]
ECs3398	-13.22	12.95	-3.73	14.81	0.0	11.08	0.2	0.00000661	0.00027019	0.00028328	0.05309524	A_07_P053458	ECs3398	cmr|putative ATP synthase beta subunit [ECs3398]
ECs3450	+3.62	9.66	+1.86	8.73	0.0	10.59	0.3	0.00074683	0.00550772	0.02430005	0.40441860	A_07_P053496	ECs3450	cmr|hypothetical protein [ECs3450]
ECs3484	-2.39	7.48	-1.26	8.11	0.2	6.85	0.0	0.00113913	0.00762488	0.05575076	0.61380420	A_07_P053513	ECs3484	cmr|hypothetical protein [ECs3484]
ECs3489	-3.15	11.61	-1.65	12.44	0.3	10.78	0.2	0.00115984	0.00772442	0.02597688	0.47117512	A_07_P055872	ECs3489	cmr|hypothetical protein [ECs3489]
ECs3492	-3.93	8.61	-1.97	9.60	0.0	7.62	0.2	0.00011296	0.00141151	0.01287792	0.35758377	A_07_P055876	ECs3492	cmr|hypothetical protein [ECs3492]
ECs3500	+2.35	10.90	+1.23	10.29	0.3	11.52	0.2	0.00646301	0.02919762	0.05411331	0.57917063	A_07_P055898	ECs3500	cmr|hypothetical membrane protein [ECs3500]
ECs3535	+3.17	8.43	+1.67	7.59	0.0	9.26	0.4	0.00140195	0.00890369	0.03004510	0.44167134	A_07_P055985	ECs3535	cmr|hypothetical protein [ECs3535]
ECs3559	+4.66	8.16	+2.22	7.05	0.2	9.27	0.1	0.00006877	0.00099883	0.01578570	0.33767663	A_07_P055986	ECs3559	cmr|interrupted glucitol/sorbitol-specific PTS system enzyme IIC component [ECs3559]
ECs3569	-3.68	7.96	-1.88	8.90	0.1	7.02	0.1	0.00010575	0.00135201	0.01578316	0.39255924	A_07_P055993	ECs3569	cmr|electron transport protein HydN [ECs3569]
ECs3585	+2.27	9.35	+1.18	8.75	0.1	9.94	0.2	0.00230035	0.01293914	0.05401740	0.57893125	A_07_P056003	ECs3585	cmr|hydrogenase isoenzyme HypD [ECs3585]
ECs3586	+2.38	11.32	+1.25	10.69	0.1	11.94	0.1	0.00118993	0.00787482	0.04941374	0.55292822	A_07_P056010	ECs3586	cmr|HypE protein [ECs3586]
ECs3627	-2.09	8.27	-1.06	8.81	0.2	7.74	0.2	0.00647556	0.02923750	0.08093814	0.69443597	A_07_P056098	ECs3627	cmr|hypothetical protein [ECs3627]
ECs3663	+14.35	12.45	+3.84	10.53	0.0	14.37	0.1	0.00000211	0.00017779	0.00170732	0.12387097	A_07_P056139	ECs3663	cmr|L-fuculokinase [ECs3663]
ECs3670	-3.74	8.96	-1.90	9.91	0.1	8.01	0.4	0.00076634	0.00560668	0.01514228	0.38827362	A_07_P056142	ECs3670	cmr|hypothetical protein [ECs3670]
ECs3683	+2.30	7.82	+1.20	7.22	0.2	8.43	0.0	0.00180169	0.01076503	0.05277376	0.57065247	A_07_P056146	ECs3683	cmr|prepilin peptidase dependent protein A [ECs3683]
ECs3718	-2.11	8.76	-1.08	9.30	0.1	8.22	0.2	0.00342212	0.01762443	0.07808943	0.68113019	A_07_P056180	ECs3718	cmr|type III secretion protein EprI [ECs3718]
ECs3727	-2.09	8.18	-1.06	8.71	0.1	7.65	0.0	0.00152449	0.00945346	0.08100927	0.69428960	A_07_P056224	ECs3727	cmr|type III secretion protein EivJ [ECs3727]
ECs3729	-3.11	8.30	-1.64	9.11	0.5	7.48	0.2	0.00616932	0.02812460	0.02638974	0.47429224	A_07_P056231	ECs3729	cmr|type III secretion protein EivI [ECs3729]
ECs3758	-2.34	8.97	-1.23	9.58	0.2	8.36	0.3	0.00474191	0.02272663	0.05854929	0.62368065	A_07_P056278	ECs3758	cmr|putative oxidoreductase Fe-S subunit [ECs3758]
ECs3764	-3.04	9.37	-1.60	10.17	0.2	8.57	0.1	0.00044254	0.00369035	0.02839876	0.48231931	A_07_P056285	ECs3764	cmr|ssDNA exonuclease RecJ [ECs3764]
ECs3771	+3.62	9.40	+1.86	8.47	0.2	10.33	0.0	0.00018778	0.00202082	0.02370681	0.40176534	A_07_P063449	ECs3771	cmr|putative oxidoreductase [ECs3771]
ECs3774	+11.70	9.49	+3.55	7.71	0.1	11.26	0.1	0.00000467	0.00023240	0.00260353	0.14744604	A_07_P063454	ECs3774	cmr|glycine decarboxylase [ECs3774]
ECs3778	+2.81	12.04	+1.49	11.29	0.0	12.78	0.0	0.00026789	0.00257334	0.03686547	0.48448247	A_07_P056295	ECs3778	cmr|2-octaprenyl-6-methoxyphenol 4-monoxygenase [ECs3778]
ECs3800	+9.72	8.47	+3.28	6.83	0.1	10.11	0.2	0.00001453	0.00039511	0.00383384	0.18346505	A_07_P056318	ECs3800	cmr|putative transporter [ECs3800]
ECs3801	+6.17	8.85	+2.63	7.53	0.1	10.16	0.2	0.00003576	0.00065463	0.00965257	0.26849823	A_07_P056323	ECs3801	cmr|hypothetical lipoprotein [ECs3801]
ECs3802	+3.08	7.55	+1.62	6.74	0.0	8.36	0.1	0.00018693	0.00201440	0.03129446	0.45160403	A_07_P056330	ECs3802	cmr|putative ABC transporter ATP-binding subunit [ECs3802]
ECs3812	-2.18	9.73	-1.13	10.29	0.0	9.16	0.0	0.00105085	0.00716524	0.07244601	0.66779274	A_07_P063456	ECs3812	cmr|agmatinase [ECs3812]
ECs3813	-3.62	7.46	-1.86	8.39	0.4	6.54	0.0	0.00137345	0.00876513	0.01667238	0.39651057	A_07_P056349	ECs3813	cmr|hypothetical lipoprotein [ECs3813]
ECs3844	-5.70	9.56	-2.51	10.82	0.0	8.31	0.0	0.00001648	0.00042417	0.00425877	0.21018809	A_07_P056361	ECs3844	cmr|hypothetical protein [ECs3844]
ECs3845	+2.81	7.98	+1.49	7.23	0.1	8.72	0.1	0.00055999	0.00439729	0.03706936	0.48352941	A_07_P056367	ECs3845	cmr|hypothetical protein [ECs3845]
ECs3846	-2.31	9.24	-1.21	9.84	0.1	8.64	0.1	0.00148683	0.00928549	0.06384909	0.64728912	A_07_P056371	ECs3846	cmr|hypothetical protein [ECs3846]
ECs3851	-3.33	8.62	-1.74	9.49	0.5	7.76	0.3	0.00451079	0.02188532	0.02208524	0.44750322	A_07_P056397	ECs3851	cmr|hypothetical protein [ECs3851]
ECs3853	-2.44	8.28	-1.29	8.93	0.0	7.64	0.0	0.00054594	0.00431923	0.05388211	0.60724409	A_07_P056407	ECs3853	cmr|putative transposase [ECs3853]
ECs3860	-3.06	9.32	-1.61	10.13	0.3	8.51	0.6	0.01136253	0.04588144	0.02870300	0.48435155	A_07_P056442	ECs3860	cmr|putative adherence factor [ECs3860]
ECs3869	-2.60	11.81	-1.38	12.50	0.1	11.12	0.1	0.00050294	0.00405648	0.04496189	0.57364668	A_07_P056468	ECs3869	cmr|hypothetical protein [ECs3869]
ECs3870	-2.10	9.36	-1.07	9.89	0.3	8.82	0.2	0.01111507	0.04514123	0.08130018	0.69526888	A_07_P056475	ECs3870	cmr|hypothetical protein [ECs3870]
ECs3874	+4.03	10.08	+2.01	9.07	0.2	11.08	0.1	0.00019042	0.00203528	0.01984502	0.37643373	A_07_P056483	ECs3874	cmr|hypothetical protein [ECs3874]
ECs3878	+4.78	11.93	+2.26	10.80	0.0	13.06	0.2	0.00007072	0.00101790	0.01511179	0.33090403	A_07_P056488	ECs3878	cmr|hydrogenase 2 maturation protease HybD [ECs3878]
ECs3913	-2.07	8.79	-1.05	9.31	0.1	8.26	0.1	0.00208071	0.01199877	0.08393356	0.70665775	A_07_P056506	ECs3913	cmr|putative ferrichrome-binding protein [ECs3913]
ECs3916	-2.53	8.18	-1.34	8.85	0.1	7.51	0.4	0.00441863	0.02153773	0.04674416	0.57405616	A_07_P056524	ECs3916	cmr|putative ABC-type iron-siderophore transport system ATP-binding protein [ECs3916]
ECs3930	-5.84	12.51	-2.55	13.78	0.1	11.24	0.0	0.00001899	0.00045650	0.00403011	0.20940594	A_07_P056534	ECs3930	cmr|hypothetical protein [ECs3930]
ECs3972	+2.06	7.57	+1.04	7.05	0.0	8.09	0.1	0.00173720	0.01049332	0.06420287	0.63373668	A_07_P063483	ECs3972	cmr|hypothetical protein [ECs3972]
ECs3995	+473.10	11.70	+8.89	7.25	0.2	16.14	0.1	0.00000006	0.00007032	0.00000000	0.00000000	A_07_P059659	ECs3995	cmr|putative kinase [ECs3995]
ECs4006	+3.86	9.08	+1.95	8.11	0.1	10.06	0.0	0.00007450	0.00105957	0.02123095	0.38553633	A_07_P059666	ECs4006	cmr|putative hydrolase [ECs4006]
ECs4013	+5.02	8.00	+2.33	6.84	0.2	9.16	0.2	0.00016227	0.00182090	0.01403074	0.31968162	A_07_P059671	ECs4013	cmr|N-acetylgalactosamine-specific PTS system enzyme IID component [ECs4013]
ECs4014	+7.92	8.60	+2.99	7.10	0.1	10.09	0.1	0.00001366	0.00038700	0.00594131	0.22377990	A_07_P059680	ECs4014	cmr|N-acetyl-galactosamine / galactosamine PTS system enzyme IIA component [ECs4014]
ECs4014	+4.79	8.89	+2.26	7.76	0.1	10.02	0.1	0.00004544	0.00075592	0.01514291	0.33066574	A_07_P059680	ECs4014	cmr|N-acetyl-galactosamine / galactosamine PTS system enzyme IIA component [ECs4014]
ECs4014	+8.66	8.70	+3.12	7.15	0.3	10.26	0.0	0.00003329	0.00063141	0.00493966	0.21075881	A_07_P059680	ECs4014	cmr|N-acetyl-galactosamine / galactosamine PTS system enzyme IIA component [ECs4014]
ECs4014	+5.40	8.98	+2.43	7.76	0.1	10.19	0.0	0.00002278	0.00051605	0.01245173	0.29613293	A_07_P059680	ECs4014	cmr|N-acetyl-galactosamine / galactosamine PTS system enzyme IIA component [ECs4014]
ECs4014	+7.44	9.01	+2.90	7.56	0.1	10.45	0.1	0.00002045	0.00047913	0.00665587	0.23338530	A_07_P059680	ECs4014	cmr|N-acetyl-galactosamine / galactosamine PTS system enzyme IIA component [ECs4014]
ECs4023	-32.17	11.79	-5.01	14.29	0.1	9.29	0.0	0.00000043	0.00009262	0.00000953	0.00789474	A_07_P059688	ECs4023	cmr|truncated putative fimbrial protein [ECs4023]
ECs4033	-2.48	7.96	-1.31	8.62	0.2	7.31	0.0	0.00139163	0.00886375	0.05153201	0.60142328	A_07_P059691	ECs4033	cmr|hypothetical protein [ECs4033]
ECs4052	+2.58	8.42	+1.37	7.74	0.1	9.10	0.1	0.00066985	0.00507658	0.04286903	0.51286474	A_07_P059701	ECs4052	cmr|argininosuccinate synthetase [ECs4052]
ECs4094	+3.38	7.95	+1.76	7.07	0.0	8.83	0.2	0.00028651	0.00269622	0.02675559	0.42124000	A_07_P059712	ECs4094	cmr|hypothetical protein [ECs4094]
ECs4098	+3.24	8.00	+1.69	7.15	0.0	8.85	0.0	0.00014040	0.00163986	0.02873539	0.43838178	A_07_P059716	ECs4098	cmr|N-acetylneuraminate lyase [ECs4098]
ECs4099	-2.56	9.58	-1.36	10.26	0.0	8.90	0.2	0.00075191	0.00553183	0.04647802	0.57482325	A_07_P059723	ECs4099	cmr|putative transcriptional repressor [ECs4099]
ECs4115	-4.38	8.13	-2.13	9.20	0.1	7.07	0.1	0.00007719	0.00108795	0.00949949	0.30900826	A_07_P058957	ECs4115	cmr|hypothetical protein [ECs4115]
ECs4150	+5.29	11.33	+2.40	10.13	0.0	12.53	0.1	0.00003385	0.00063751	0.01300877	0.30252585	A_07_P059737	ECs4150	cmr|hypothetical protein [ECs4150]
ECs4196	-2.82	9.69	-1.50	10.44	0.2	8.94	0.2	0.00169604	0.01030584	0.03629954	0.52096627	A_07_P063525	ECs4196	cmr|hypothetical protein [ECs4196]
ECs4237	+2.21	7.24	+1.14	6.67	0.1	7.81	0.1	0.00119286	0.00788759	0.05627541	0.58909574	A_07_P059765	ECs4237	cmr|hypothetical protein [ECs4237]
ECs4253	-2.91	9.17	-1.54	9.94	0.1	8.40	0.4	0.00385200	0.01938190	0.03077426	0.49238821	A_07_P063536	ECs4253	cmr|hypothetical protein [ECs4253]
ECs4260	+13.60	9.11	+3.77	7.23	0.2	10.99	0.2	0.00001050	0.00034033	0.00196964	0.13084388	A_07_P063542	ECs4260	cmr|positive regulator of mal regulon [ECs4260]
ECs4280	+3.95	11.17	+1.98	10.18	0.2	12.16	0.1	0.00022906	0.00233574	0.02076347	0.38233918	A_07_P059825	ECs4280	cmr|putative DNA processing chain A [ECs4280]
ECs4281	+5.00	10.27	+2.32	9.11	0.0	11.43	0.1	0.00004579	0.00075800	0.01414126	0.31988506	A_07_P059828	ECs4281	cmr|putative ATP-dependent DNA helicase [ECs4281]
ECs4282	+6.80	9.21	+2.77	7.83	0.1	10.59	0.0	0.00001229	0.00036634	0.00800813	0.24867850	A_07_P059833	ECs4282	cmr|putative ATP-dependent DNA helicase [ECs4282]
ECs4330	-2.22	8.54	-1.15	9.11	0.2	7.96	0.2	0.00262625	0.01434015	0.06837779	0.65883721	A_07_P059920	ECs4330	cmr|hypothetical membrane protein [ECs4330]
ECs4333	+3.63	7.55	+1.86	6.62	0.1	8.48	0.4	0.00091623	0.00646289	0.02399517	0.40232162	A_07_P059931	ECs4333	cmr|hypothetical protein [ECs4333]
ECs4334	+7.55	9.42	+2.92	7.96	0.1	10.87	0.0	0.00001328	0.00038273	0.00651296	0.23146727	A_07_P059939	ECs4334	cmr|hypothetical protein [ECs4334]
ECs4335	+5.45	8.61	+2.45	7.39	0.0	9.84	0.1	0.00002403	0.00053065	0.01216527	0.29241221	A_07_P059941	ECs4335	cmr|hypothetical protein [ECs4335]
ECs4337	+7.93	8.45	+2.99	6.96	0.0	9.95	0.1	0.00001135	0.00035103	0.00594258	0.22329356	A_07_P059955	ECs4337	cmr|hypothetical lipoprotein [ECs4337]
ECs4338	+6.89	8.44	+2.78	7.05	0.1	9.83	0.0	0.00001582	0.00041177	0.00786458	0.24813627	A_07_P059960	ECs4338	cmr|putative 3-oxoacyl- [ECs4338]
ECs4339	+8.36	8.04	+3.06	6.51	0.2	9.57	0.0	0.00001121	0.00034947	0.00529027	0.21521964	A_07_P059965	ECs4339	cmr|putative beta-hydroxydecanoyl-ACP dehydrase [ECs4339]
ECs4349	+3.68	9.21	+1.88	8.27	0.1	10.15	0.2	0.00020045	0.00210910	0.02325140	0.39876906	A_07_P059982	ECs4349	cmr|putative GntR family regulatory protein [ECs4349]
ECs4350	+3.92	7.81	+1.97	6.83	0.2	8.80	0.2	0.00028056	0.00265935	0.02093051	0.38317442	A_07_P059987	ECs4350	cmr|putative phosphotransferase system enzyme IIA [ECs4350]
ECs4351	+3.51	7.97	+1.81	7.06	0.1	8.87	0.3	0.00060841	0.00469780	0.02536966	0.41262397	A_07_P059994	ECs4351	cmr|putative phosphotransferase system enzyme IIB [ECs4351]
ECs4357	-2.08	10.63	-1.06	11.16	0.1	10.10	0.0	0.00197156	0.01150478	0.08115473	0.69515778	A_07_P060023	ECs4357	cmr|HicA-like protein [ECs4357]
ECs4367	+7.05	11.94	+2.82	10.53	0.1	13.35	0.0	0.00001415	0.00039205	0.00748539	0.24654812	A_07_P060029	ECs4367	cmr|universal stress protein UspA [ECs4367]
ECs4407	-2.91	9.26	-1.54	10.03	0.1	8.49	0.1	0.00027983	0.00265556	0.03221989	0.49683643	A_07_P060081	ECs4407	cmr|hypothetical protein [ECs4407]
ECs4475	+5.51	8.44	+2.46	7.21	0.1	9.67	0.0	0.00002074	0.00048521	0.01196646	0.29164087	A_07_P060156	ECs4475	cmr|mannitol-specific PTS system enzyme IIABC components [ECs4475]
ECs4503	-2.49	9.64	-1.31	10.29	0.1	8.98	0.1	0.00099989	0.00690783	0.05157139	0.60143704	A_07_P060202	ECs4503	cmr|lipopolysaccharide core biosynthesis protein WaaY [ECs4503]
ECs4503	-2.12	9.56	-1.08	10.10	0.1	9.02	0.0	0.00212350	0.01216607	0.07720465	0.67754181	A_07_P060202	ECs4503	cmr|lipopolysaccharide core biosynthesis protein WaaY [ECs4503]
ECs4503	-2.16	9.55	-1.11	10.10	0.1	8.99	0.1	0.00136898	0.00874373	0.07432419	0.67134825	A_07_P060202	ECs4503	cmr|lipopolysaccharide core biosynthesis protein WaaY [ECs4503]
ECs4505	-3.74	10.15	-1.90	11.10	0.2	9.20	0.3	0.00061711	0.00474404	0.01519944	0.38847403	A_07_P060214	ECs4505	cmr|lipopolysaccharide core biosynthesis protein RfaP [ECs4505]
ECs4506	-2.26	10.40	-1.17	10.99	0.1	9.81	0.2	0.00186748	0.01105321	0.06607914	0.65761694	A_07_P060217	ECs4506	cmr|glucosyltransferase I [ECs4506]
ECs4507	-2.25	10.51	-1.17	11.10	0.1	9.93	0.1	0.00176074	0.01059271	0.06542683	0.65277567	A_07_P060224	ECs4507	cmr|lipopolysaccharide core biosynthesis protein RfaQ [ECs4507]
ECs4519	-3.15	9.21	-1.66	10.04	0.1	8.38	0.4	0.00163901	0.01002510	0.02459223	0.46038050	A_07_P063587	ECs4519	cmr|putative alpha helix protein [ECs4519]
ECs4549	-2.64	8.12	-1.40	8.82	0.1	7.42	0.0	0.00051858	0.00415407	0.04330729	0.56396195	A_07_P057362	ECs4549	cmr|hypothetical protein [ECs4549]
ECs4551	-2.06	8.41	-1.05	8.93	0.2	7.89	0.0	0.00280415	0.01506775	0.08448869	0.70792443	A_07_P057374	ECs4551	cmr|hypothetical protein [ECs4551]
ECs4605	-2.31	8.68	-1.21	9.29	0.0	8.08	0.0	0.00078007	0.00569374	0.06291603	0.64238003	A_07_P057602	ECs4605	cmr|sensor histidine protein kinase UhpB [ECs4605]
ECs4615	+2.31	8.18	+1.21	7.58	0.0	8.78	0.2	0.00134129	0.00862635	0.05259146	0.57024793	A_07_P063607	ECs4615	cmr|putative transcriptional regulator [ECs4615]
ECs4630	-2.57	7.40	-1.36	8.08	0.0	6.72	0.2	0.00101168	0.00696452	0.04701918	0.57608560	A_07_P062852	ECs4630	cmr|hypothetical protein [ECs4630]
ECs4652	-3.44	10.26	-1.78	11.15	0.1	9.37	0.6	0.00782978	0.03411901	0.02049606	0.43430686	A_07_P057658	ECs4652	cmr|putative phosphatase [ECs4652]
ECs4666	-2.06	7.41	-1.04	7.93	0.1	6.89	0.0	0.00239234	0.01330919	0.08530170	0.71132945	A_07_P057700	ECs4666	cmr|putative fimbrial protein [ECs4666]
ECs4742	+3.75	8.98	+1.91	8.03	0.1	9.93	0.2	0.00028447	0.00267907	0.02263592	0.39597778	A_07_P057739	ECs4742	cmr|putative phosphatase [ECs4742]
ECs4746	-5.19	10.64	-2.37	11.83	0.0	9.45	0.2	0.00006522	0.00096672	0.00571710	0.24525886	A_07_P057749	ECs4746	cmr|Mg2+ transport protein CorA [ECs4746]
ECs4755	-2.57	10.36	-1.36	11.04	0.0	9.68	0.0	0.00042728	0.00360123	0.04670986	0.57498045	A_07_P057761	ECs4755	cmr|lysophospholipase L [ECs4755]
ECs4786	+6.09	8.96	+2.61	7.66	0.2	10.26	0.0	0.00003606	0.00065813	0.00994220	0.27128250	A_07_P063635	ECs4786	cmr|DNA polymerase I [ECs4786]
ECs4801	+2.07	7.32	+1.05	6.80	0.2	7.85	0.1	0.00308582	0.01628119	0.06517213	0.63850031	A_07_P057793	ECs4801	cmr|hypothetical membrane protein [ECs4801]
ECs4819	+4.18	11.33	+2.06	10.30	0.0	12.36	0.0	0.00004841	0.00078487	0.01864647	0.36468323	A_07_P057834	ECs4819	cmr|formate dehydrogenase-O iron-sulfur subunit [ECs4819]
ECs4820	+2.18	11.04	+1.13	10.48	0.1	11.61	0.0	0.00117242	0.00778841	0.05791667	0.59989474	A_07_P057838	ECs4820	cmr|formate dehydrogenase-O major subunit [ECs4820]
ECs4829	+2.20	8.41	+1.14	7.84	0.3	8.98	0.1	0.00496954	0.02360192	0.05818661	0.60110892	A_07_P057862	ECs4829	cmr|rhamnulose-phosphate aldolase [ECs4829]
ECs4832	+2.16	7.70	+1.11	7.14	0.0	8.25	0.1	0.00162972	0.00997925	0.05974276	0.61196487	A_07_P057869	ECs4832	cmr|positive regulator for rhaBAD operon [ECs4832]
ECs4847	+6.52	8.92	+2.70	7.57	0.2	10.27	0.0	0.00003206	0.00062191	0.00880272	0.26001876	A_07_P057876	ECs4847	cmr|hypothetical protein [ECs4847]
ECs4848	+5.84	10.25	+2.55	8.97	0.1	11.52	0.0	0.00001681	0.00042622	0.01080856	0.27760196	A_07_P057881	ECs4848	cmr|putative regulator [ECs4848]
ECs4866	-2.53	9.59	-1.34	10.26	0.1	8.92	0.1	0.00073673	0.00546356	0.04853214	0.58149924	A_07_P063657	ECs4866	cmr|hypothetical protein [ECs4866]
ECs4880	+2.00	8.62	+1.00	8.11	0.2	9.12	0.1	0.00389660	0.01953759	0.07028328	0.66141064	A_07_P063667	ECs4880	cmr|formate acetyltransferase 2 [ECs4880]
ECs4889	-2.56	8.23	-1.35	8.91	0.3	7.55	0.1	0.00186470	0.01104921	0.04769817	0.57899769	A_07_P057911	ECs4889	cmr|argininosuccinate lyase [ECs4889]
ECs4893	-2.24	11.47	-1.16	12.05	0.2	10.89	0.3	0.00674277	0.03026172	0.06623793	0.65712035	A_07_P057923	ECs4893	cmr|putative membrane transport / symporter protein [ECs4893]
ECs4893	-4.13	11.86	-2.05	12.88	0.3	10.83	0.4	0.00199570	0.01160276	0.01169906	0.34884470	A_07_P057923	ECs4893	cmr|putative membrane transport / symporter protein [ECs4893]
ECs4893	-3.50	11.57	-1.81	12.48	0.1	10.67	0.1	0.00020252	0.00212689	0.01905170	0.41892458	A_07_P057923	ECs4893	cmr|putative membrane transport / symporter protein [ECs4893]
ECs4893	-2.55	11.42	-1.35	12.10	0.1	10.75	0.4	0.00389123	0.01951690	0.04795097	0.58027671	A_07_P057923	ECs4893	cmr|putative membrane transport / symporter protein [ECs4893]
ECs4893	-2.34	11.37	-1.23	11.98	0.4	10.75	0.2	0.00835402	0.03591635	0.05866552	0.62449628	A_07_P057923	ECs4893	cmr|putative membrane transport / symporter protein [ECs4893]
ECs4896	-3.98	8.87	-1.99	9.86	0.1	7.87	0.1	0.00012751	0.00153683	0.01285124	0.35874113	A_07_P057927	ECs4896	cmr|tRNA [ECs4896]
ECs4921	+3.16	9.03	+1.66	8.20	0.0	9.86	0.4	0.00176755	0.01061336	0.03026613	0.44367784	A_07_P057952	ECs4921	cmr|endonuclease V [ECs4921]
ECs4949	-3.81	10.68	-1.93	11.65	0.1	9.72	0.3	0.00045526	0.00377438	0.01419970	0.37699831	A_07_P058017	ECs4949	cmr|hypothetical protein [ECs4949]
ECs4956	-3.36	8.84	-1.75	9.72	0.0	7.97	0.5	0.00347528	0.01786313	0.02216146	0.44674776	A_07_P061239	ECs4956	cmr|hypothetical protein [ECs4956]
ECs4994	-2.39	9.37	-1.26	10.00	0.4	8.74	0.2	0.00830842	0.03579851	0.05543890	0.61251228	A_07_P061427	ECs4994	cmr|hypothetical protein [ECs4994]
ECs5003	-2.32	7.55	-1.21	8.16	0.1	6.95	0.2	0.00147912	0.00924829	0.06099848	0.63390099	A_07_P061472	ECs5003	cmr|putative sorbitol-6-phosphate 2-dehydrogenase [ECs5003]
ECs5050	+2.93	9.93	+1.55	9.15	0.2	10.70	0.0	0.00042336	0.00357200	0.03423082	0.47027051	A_07_P061504	ECs5050	cmr|hypothetical protein [ECs5050]
ECs5051	+31.24	9.39	+4.97	6.90	0.2	11.87	0.1	0.00000200	0.00017318	0.00029599	0.05065217	A_07_P063704	ECs5051	cmr|acetyl-CoA synthetase [ECs5051]
ECs5057	-4.29	8.65	-2.10	9.70	0.4	7.60	0.4	0.00308857	0.01629028	0.01042492	0.32372781	A_07_P063709	ECs5057	cmr|cytochrome c-type biogenesis protein NrfF [ECs5057]
ECs5065	-4.02	7.51	-2.01	8.52	0.0	6.51	0.0	0.00005681	0.00087433	0.01203316	0.34697802	A_07_P061508	ECs5065	cmr|hypothetical protein [ECs5065]
ECs5129	-3.82	9.42	-1.93	10.39	0.0	8.45	0.4	0.00074689	0.00550772	0.01404916	0.37617347	A_07_P058973	ECs5129	cmr|suppressor of groEL [ECs5129]
ECs5165	+9.75	10.96	+3.28	9.32	0.0	12.61	0.1	0.00000549	0.00025298	0.00376143	0.18334365	A_07_P058980	ECs5165	cmr|hypothetical protein [ECs5165]
ECs5206	+4.74	9.67	+2.24	8.55	0.4	10.79	0.1	0.00061745	0.00474435	0.01530615	0.33284530	A_07_P063741	ECs5206	cmr|putative ATP-binding component of a transport system [ECs5206]
ECs5256	-2.24	9.30	-1.16	9.88	0.2	8.71	0.0	0.00166727	0.01017421	0.06668318	0.65616250	A_07_P061690	ECs5256	cmr|hypothetical protein [ECs5256]
ECs5261	-2.17	8.16	-1.12	8.72	0.1	7.60	0.0	0.00161498	0.00992827	0.07426512	0.67119977	A_07_P061712	ECs5261	cmr|putative ATP-dependent helicase [ECs5261]
ECs5282	+5.89	8.49	+2.56	7.21	0.1	9.77	0.1	0.00002338	0.00052279	0.01069296	0.27780528	A_07_P061751	ECs5282	cmr|D-mannonate oxidoreductase [ECs5282]
ECs5296	+5.38	8.25	+2.43	7.03	0.1	9.46	0.1	0.00003443	0.00064547	0.01251905	0.29550225	A_07_P061800	ECs5296	cmr|hypothetical protein [ECs5296]
ECs5314	-2.66	8.62	-1.41	9.33	0.1	7.92	0.1	0.00047781	0.00389373	0.04232025	0.56085017	A_07_P061848	ECs5314	cmr|hypothetical protein [ECs5314]
ECs5314	-2.37	8.92	-1.25	9.54	0.2	8.29	0.4	0.01082153	0.04428755	0.05832825	0.62216802	A_07_P061848	ECs5314	cmr|hypothetical protein [ECs5314]
ECs5337	+2.02	8.34	+1.02	7.83	0.2	8.84	0.0	0.00549369	0.02558952	0.06952744	0.65902468	A_07_P061859	ECs5337	cmr|putative activating enzyme [ECs5337]
ECs5338	+3.93	10.21	+1.97	9.22	0.1	11.19	0.3	0.00030417	0.00282365	0.02091908	0.38385781	A_07_P061864	ECs5338	cmr|hypothetical protein [ECs5338]
ECs5344	+2.48	8.82	+1.31	8.17	0.2	9.48	0.2	0.00164421	0.01005299	0.04680069	0.53940703	A_07_P061868	ECs5344	cmr|hypothetical protein [ECs5344]
ECs5345	+7.46	9.14	+2.90	7.69	0.1	10.59	0.1	0.00001119	0.00034947	0.00665904	0.23297778	A_07_P061871	ECs5345	cmr|putative lipoate-protein ligase A [ECs5345]
ECs5350	+3.83	8.10	+1.94	7.13	0.0	9.07	0.1	0.00008651	0.00117815	0.02141133	0.38525714	A_07_P062875	ECs5350	cmr|soluble lytic murein transglycosylase [ECs5350]
efp	-4.50	12.36	-2.17	13.45	0.0	11.28	0.1	0.00006067	0.00091356	0.00874174	0.30248352	A_07_P011467	b4147	cmr|elongation factor P [b4147]
EHEC-hlyD	-2.90	10.13	-1.53	10.90	0.1	9.36	0.4	0.00276473	0.01492508	0.03185213	0.49406897	A_07_P048869	ABH-0244088	asap|hemolysin transport protein [L7050]
elaA	+2.21	7.96	+1.14	7.39	0.0	8.53	0.2	0.00305331	0.01614216	0.05882431	0.60531373	A_07_P032393	c_2811	cmr|Protein elaA [c_2811]
elbB	+18.81	11.07	+4.23	8.95	0.1	13.19	0.1	0.00000149	0.00015540	0.00092416	0.08818182	A_07_P012521	b3209	cmr|sigma cross-reacting protein 27A [b3209]
emrA	-2.34	10.91	-1.23	11.53	0.0	10.30	0.0	0.00074458	0.00550202	0.06065041	0.63278993	A_07_P001896	b2685	cmr|multidrug resistance secretion protein [b2685]
emrD	-9.76	9.60	-3.29	11.24	0.0	7.96	0.1	0.00000562	0.00025427	0.00081237	0.09268116	A_07_P017297	b3673	cmr|2-module integral membrane pump; multidrug resistance [b3673]
emrE	-2.05	8.32	-1.04	8.84	0.1	7.80	0.2	0.00605638	0.02771044	0.08645452	0.71338574	A_07_P016482	b0543	cmr|methylviologen resistance [b0543]
emtA	-3.83	10.17	-1.94	11.14	0.2	9.21	0.1	0.00018395	0.00199406	0.01412030	0.37615905	A_07_P006916	b1193	cmr|murein transglycosylase E [b1193]
endA	-2.26	10.17	-1.18	10.75	0.1	9.58	0.1	0.00176301	0.01059944	0.06716463	0.65761194	A_07_P011868	b2945	cmr|DNA-specific endonuclease I [b2945]
eno	+6.44	10.71	+2.69	9.37	0.1	12.06	0.1	0.00001689	0.00042684	0.00896850	0.26148148	A_07_P002342	b2779	cmr|enolase [b2779]
entS	-2.38	7.14	-1.25	7.76	0.3	6.51	0.0	0.00295329	0.01572426	0.05750254	0.62008219	A_07_P016711	b0591	cmr|putative transport [b0591]
epd	+4.08	10.81	+2.03	9.80	0.2	11.83	0.2	0.00035933	0.00317256	0.01969385	0.37674362	A_07_P011783	b2927	cmr|D-erythrose 4-phosphate dehydrogenase [b2927]
eptA	+3.39	8.73	+1.76	7.85	0.0	9.61	0.2	0.00030232	0.00281137	0.02697091	0.42251741	A_07_P011307	b4114	cmr|orf, hypothetical protein [b4114]
eptB	+4.06	8.39	+2.02	7.38	0.0	9.40	0.1	0.00007382	0.00105267	0.01979992	0.37739709	A_07_P007657	b3546	cmr|orf, hypothetical protein [b3546]
EQC	-2.68	9.17	-1.42	9.88	0.1	8.46	0.2	0.00068708	0.00516592	0.04089240	0.55026496	(+)eQC-42	EQC	
EQC	-3.30	8.76	-1.72	9.62	0.0	7.90	0.1	0.00015744	0.00177863	0.02262386	0.45030341	(+)eQC-42	EQC	
era	-2.85	10.69	-1.51	11.44	0.1	9.93	0.1	0.00031345	0.00289486	0.03466590	0.51198874	A_07_P019734	b2566	cmr|GTP-binding protein [b2566]
erpA	-14.67	11.04	-3.88	12.98	0.1	9.10	0.0	0.00000186	0.00016832	0.00015752	0.04275862	A_07_P002744	b0156	cmr|orf, hypothetical protein [b0156]
essD	+7.28	8.98	+2.86	7.55	0.2	10.41	0.0	0.00003312	0.00063062	0.00692073	0.23635575	A_07_P016531	b0554	cmr|orf, hypothetical protein [b0554]
ETG10_234183	-4.77	12.18	-2.25	13.31	0.2	11.05	0.7	0.00339566	0.01752827	0.00766133	0.28650831	ETG10_234183	ETG10_234183	
ETG10_234183	-3.56	11.95	-1.83	12.86	0.2	11.03	0.5	0.00333475	0.01730464	0.01861471	0.41688478	ETG10_234183	ETG10_234183	
ETG10_234183	-2.40	11.58	-1.26	12.21	0.0	10.95	0.1	0.00095276	0.00664612	0.05632304	0.61622655	ETG10_234183	ETG10_234183	
etpM	-2.39	8.65	-1.26	9.28	0.0	8.02	0.0	0.00062124	0.00475805	0.05736535	0.61945130	A_07_P048838	ABH-0244073	asap|type II secretion protein [L7042]
eutA	+4.10	7.75	+2.03	6.74	0.1	8.77	0.3	0.00026982	0.00258239	0.01969830	0.37637136	A_07_P013311	b2451	cmr|orf, hypothetical protein [b2451]
eutC	+2.86	7.50	+1.52	6.74	0.1	8.26	0.1	0.00045306	0.00376202	0.03577299	0.47770144	A_07_P042830	Z3705	cmr|ethanolamine ammonia-lyase, light chain [Z3705]
eutD	+3.19	8.25	+1.67	7.42	0.4	9.09	0.1	0.00165555	0.01011055	0.03016070	0.44295709	A_07_P013350	b2458	cmr|ethanolamine utilization; homolog of Salmonella acetyl/butyryl P transferase [b2458]
eutQ	+4.45	8.60	+2.15	7.53	0.1	9.68	0.0	0.00004818	0.00078282	0.01713288	0.34940415	A_07_P013359	b2460	cmr|orf, hypothetical protein [b2460]
exuT	+6.36	8.58	+2.67	7.25	0.1	9.92	0.0	0.00001445	0.00039421	0.00923780	0.26637363	A_07_P000192	b3093	cmr|transport of hexuronates [b3093]
fabA	-2.44	8.43	-1.29	9.07	0.1	7.79	0.1	0.00116950	0.00777891	0.05333714	0.60369518	A_07_P033873	c_1090	cmr|3-hydroxydecanoyl- [c_1090]
fabB	+9.33	12.35	+3.22	10.74	0.0	13.96	0.2	0.00001049	0.00034033	0.00416921	0.19081395	A_07_P013901	b2323	cmr|3-oxoacyl- [b2323]
fabD	+4.36	12.71	+2.12	11.65	0.1	13.78	0.4	0.00058883	0.00458487	0.01771913	0.35719590	A_07_P001661	b1092	cmr|malonyl-CoA- [b1092]
fabF	-5.38	11.14	-2.43	12.35	0.1	9.93	0.4	0.00043672	0.00365532	0.00527376	0.23655271	A_07_P001676	b1095	cmr|3-oxoacyl- [b1095]
fabG	+4.46	7.61	+2.16	6.53	0.2	8.69	0.2	0.00022521	0.00230388	0.01699695	0.34843750	A_07_P046211	Z4865	cmr|putative beta-ketoacyl-ACP reductase [Z4865]
fabG	+4.22	7.80	+2.08	6.77	0.1	8.84	0.2	0.00021930	0.00225661	0.01849276	0.36302993	A_07_P059966	ECs4340	cmr|putative 3-oxoacyl- [ECs4340]
fabG	+5.77	12.34	+2.53	11.08	0.1	13.61	0.4	0.00026318	0.00255503	0.01055323	0.27691667	A_07_P001668	b1093	cmr|3-oxoacyl- [b1093]
fabH	+4.00	12.66	+2.00	11.66	0.1	13.66	0.0	0.00008101	0.00112081	0.01989710	0.37606242	A_07_P001656	b1091	cmr|3-oxoacyl- [b1091]
fabR	-2.46	12.52	-1.30	13.17	0.0	11.87	0.1	0.00087830	0.00624567	0.05170414	0.60076015	A_07_P008519	b3963	cmr|orf, hypothetical protein [b3963]
fabZ	-2.98	14.12	-1.58	14.90	0.0	13.33	0.0	0.00019508	0.00207030	0.03031186	0.49097737	A_07_P002859	b0180	cmr|(3R)-hydroxymyristol acyl carrier protein dehydratase [b0180]
fadH	+3.70	9.30	+1.89	8.35	0.8	10.24	0.1	0.01107315	0.04502470	0.02341209	0.39978308	A_07_P000131	b3081	cmr|putative NADPH dehydrogenase [b3081]
fadI	-4.11	11.19	-2.04	12.21	0.2	10.17	0.5	0.00203604	0.01179810	0.01168890	0.34986692	A_07_P053270	ECs3225	cmr|putative acyltransferase [ECs3225]
fadI	-3.83	10.70	-1.94	11.67	0.1	9.73	0.2	0.00021287	0.00220339	0.01398692	0.37514480	A_07_P014000	b2342	cmr|putative acyltransferase [b2342]
fadI	-3.80	10.90	-1.93	11.86	0.4	9.93	0.3	0.00162443	0.00995913	0.01406568	0.37597623	A_07_P042685	Z3605	cmr|putative acyltransferase [Z3605]
fadJ	+2.94	11.31	+1.56	10.53	0.0	12.09	0.0	0.00021040	0.00218894	0.03400025	0.46873905	A_07_P013995	b2341	cmr|putative enzyme [b2341]
fadK	-2.39	10.21	-1.26	10.84	0.1	9.58	0.4	0.00950469	0.03975606	0.05705158	0.61818307	A_07_P018557	b1701	cmr|putative ligase/synthetase [b1701]
fbaA	+21.99	11.90	+4.46	9.67	0.1	14.13	0.1	0.00000136	0.00014976	0.00061357	0.07546875	A_07_P011772	b2925	cmr|fructose-bisphosphate aldolase, class II [b2925]
fbaB	+5.92	10.80	+2.56	9.52	0.0	12.08	0.2	0.00003630	0.00065813	0.01045287	0.27752108	A_07_P003250	b2097	cmr|orf, hypothetical protein [b2097]
fbp	-3.31	13.93	-1.73	14.80	0.0	13.07	0.0	0.00013115	0.00156505	0.02195185	0.44768135	A_07_P003714	b4232	cmr|fructose-bisphosphatase [b4232]
fdnG	+2.34	9.43	+1.23	8.82	0.2	10.05	0.1	0.00192377	0.01129162	0.05181021	0.56449827	A_07_P009461	b1474	cmr|formate dehydrogenase-N, nitrate-inducible, alpha subunit [b1474]
fdoG	+3.02	11.19	+1.59	10.39	0.0	11.99	0.0	0.00018943	0.00202942	0.03242251	0.45863432	A_07_P047068	Z5436	cmr|formate dehydrogenase-O, major subunit [Z5436]
fdoG	+2.41	11.37	+1.27	10.74	0.1	12.01	0.1	0.00097938	0.00679265	0.04872713	0.54836312	A_07_P039391	c_5624	cmr|Formate dehydrogenase-O, major subunit [c_5624]
fdoG	+2.97	8.58	+1.57	7.79	0.0	9.37	0.1	0.00029680	0.00277317	0.03379002	0.46788918	A_07_P019246	b3894	cmr|formate dehydrogenase-O, major subunit [b3894]
fdoH	+3.35	9.53	+1.74	8.66	0.2	10.40	0.1	0.00034681	0.00310064	0.02722497	0.42396637	A_07_P019241	b3893	cmr|formate dehydrogenase-O, iron-sulfur subunit [b3893]
fdoH	+2.80	10.01	+1.49	9.26	0.0	10.75	0.2	0.00062226	0.00475805	0.03661649	0.48282245	A_07_P039386	c_4843	cmr|Formate dehydrogenase-O beta subunit [c_4843]
fdoH	+4.59	11.46	+2.20	10.36	0.0	12.56	0.1	0.00003800	0.00067369	0.01632114	0.34124834	A_07_P047064	Z5435	cmr|formate dehydrogenase-O, iron-sulfur subunit [Z5435]
fdoI	+7.34	10.11	+2.88	8.67	0.1	11.55	0.2	0.00002114	0.00049238	0.00685340	0.23558952	A_07_P019239	b3892	cmr|formate dehydrogenase, cytochrome B556 [b3892]
fecA	-3.06	9.63	-1.61	10.44	0.1	8.83	0.1	0.00035460	0.00314521	0.02791794	0.48195175	A_07_P003984	b4291	cmr|outer membrane receptor; citrate-dependent iron transport, outer membrane receptor [b4291]
fecB	-3.04	9.76	-1.60	10.56	0.1	8.95	0.1	0.00029936	0.00278880	0.02809832	0.48189542	A_07_P003977	b4290	cmr|citrate-dependent iron transport, periplasmic protein [b4290]
fecI	-2.38	8.64	-1.25	9.26	0.1	8.01	0.2	0.00145939	0.00914312	0.05643039	0.61697222	A_07_P003994	b4293	cmr|probable RNA polymerase sigma factor [b4293]
feoA	+2.04	8.25	+1.03	7.74	0.1	8.76	0.1	0.00229415	0.01292278	0.06598196	0.64203956	A_07_P018070	b3408	cmr|ferrous iron transport protein A [b3408]
fepA	-2.21	9.78	-1.14	10.36	0.1	9.21	0.1	0.00184541	0.01097122	0.07117124	0.66499703	A_07_P016680	b0584	cmr|outer membrane receptor for ferric enterobactin [b0584]
fepE	-10.62	11.73	-3.41	13.43	0.0	10.03	0.1	0.00000377	0.00020888	0.00064088	0.08270492	A_07_P031316	c_0674	cmr|Ferric enterobactin transport protein fepE [c_0674]
ffh	-5.55	13.19	-2.47	14.43	0.0	11.95	0.3	0.00010240	0.00132346	0.00475864	0.22364179	A_07_P019926	b2610	cmr|GTP-binding export factor binds to signal sequence, GTP and RNA [b2610]
fhlA	+2.20	7.93	+1.14	7.36	0.2	8.50	0.2	0.00366271	0.01863188	0.05869792	0.60440811	A_07_P002101	b2731	cmr|formate hydrogen-lyase transcriptional activator for fdhF, hyc and hyp operons [b2731]
fhuD	-2.85	7.72	-1.51	8.48	0.0	6.97	0.0	0.00024889	0.00245518	0.03476372	0.51247191	A_07_P002724	b0152	cmr|hydroxamate-dependent iron uptake, cytoplasmic membrane component [b0152]
fimD	-12.63	9.42	-3.66	11.25	0.0	7.60	0.1	0.00000342	0.00020055	0.00032076	0.05489130	A_07_P004111	b4317	cmr|outer membrane protein; export and assembly of type 1 fimbriae, interrupted [b4317]
fimF	-12.60	9.49	-3.66	11.31	0.2	7.66	0.0	0.00000426	0.00022159	0.00032965	0.05521277	A_07_P004120	b4318	cmr|fimbrial morphology [b4318]
fimF	-4.75	8.69	-2.25	9.82	0.1	7.57	0.0	0.00003648	0.00065813	0.00748920	0.28275779	A_07_P041401	c_5397	cmr|FimF protein precursor [c_5397]
fimG	-12.26	10.62	-3.62	12.43	0.1	8.81	0.0	0.00000278	0.00018406	0.00035442	0.05812500	A_07_P004124	b4319	cmr|fimbrial morphology [b4319]
fimH	-2.56	8.09	-1.36	8.77	0.1	7.41	0.3	0.00196984	0.01150478	0.04683244	0.57424455	A_07_P004128	b4320	cmr|minor fimbrial subunit, D-mannose specific adhesin [b4320]
fimI	-2.07	9.11	-1.05	9.64	0.1	8.59	0.0	0.00210706	0.01209391	0.08159870	0.69630894	A_07_P004101	b4315	cmr|fimbrial protein [b4315]

fkpB	+4.03	10.47	+2.01	9.46	0.0	11.47	0.1	0.00006022	0.00090997	0.01980310	0.37700121	A_07_P010829	b0028	cmr|probable FKBX-type 16KD peptidyl-prolyl cis-trans isomerase [b0028]
fldB	-2.93	10.04	-1.55	10.82	0.0	9.27	0.1	0.00022344	0.00229176	0.03218496	0.49678431	A_07_P006442	b2895	cmr|flavodoxin 2 [b2895]
flgK	-2.22	9.42	-1.15	9.99	0.1	8.84	0.1	0.00118648	0.00786195	0.06882241	0.65788707	A_07_P001612	b1082	cmr|flagellar biosynthesis, hook-filament junction protein 1 [b1082]
flgL	-2.85	8.43	-1.51	9.19	0.2	7.68	0.3	0.00199370	0.01159971	0.03448806	0.50984038	A_07_P001620	b1083	cmr|flagellar biosynthesis; hook-filament junction protein [b1083]
flhD	+2.67	8.54	+1.42	7.83	0.0	9.25	0.1	0.00051873	0.00415407	0.04053354	0.50169811	A_07_P016140	b1892	cmr|regulator of flagellar biosynthesis, acting on class 2 operons; transcriptional initiation factor [b1892]
fliA	-4.60	7.84	-2.20	8.94	0.1	6.74	0.2	0.00008925	0.00120512	0.00831555	0.29486486	A_07_P014973	b1922	cmr|flagellar biosynthesis; alternative sigma factor 28; regulation of flagellar operons [b1922]
fliE	-2.86	7.95	-1.52	8.71	0.3	7.19	0.1	0.00155235	0.00957690	0.03341082	0.50240688	A_07_P015049	b1937	cmr|flagellar biosynthesis; basal-body component, possibly at [b1937]
fliI	-2.25	12.58	-1.17	13.16	0.3	11.99	0.1	0.00404343	0.02013275	0.06717861	0.65652390	A_07_P044691	Z3031	cmr|flagellum-specific ATP synthase [Z3031]
fliI	-2.39	12.60	-1.26	13.23	0.4	11.98	0.1	0.00768753	0.03358281	0.05663935	0.61626123	A_07_P055201	ECs2680	cmr|flagellum-specific ATP synthase [ECs2680]
fliT	-2.38	8.94	-1.25	9.57	0.3	8.31	0.4	0.01156672	0.04646759	0.05480119	0.60974558	A_07_P014991	b1926	cmr|flagellar biosynthesis; repressor of class 3a and 3b operons [b1926]
fliY	-4.39	13.08	-2.14	14.15	0.0	12.01	0.0	0.00003945	0.00068718	0.00943407	0.30879418	A_07_P041209	c_2335	cmr|Cystine-binding periplasmic protein precursor [c_2335]
fliY	-5.81	11.67	-2.54	12.93	0.1	10.40	0.4	0.00017660	0.00193847	0.00405678	0.20872549	A_07_P014964	b1920	cmr|putative periplasmic binding transport protein [b1920]
flu	+5.38	10.91	+2.43	9.70	0.5	12.13	0.0	0.00089734	0.00634668	0.01175749	0.28968701	A_07_P015330	b2000	cmr|outer membrane fluffing protein, similar to adhesin [b2000]
fmt	-2.53	12.02	-1.34	12.69	0.0	11.35	0.1	0.00050019	0.00404054	0.04855246	0.58130038	A_07_P012874	b3288	cmr|10-formyltetrahydrofolate:L-methionyl-tRNA [b3288]
fnr	-8.50	13.79	-3.09	15.33	0.0	12.25	0.1	0.00000610	0.00026402	0.00123730	0.11325581	A_07_P014647	b1334	cmr|transcriptional regulation of aerobic, anaerobic respiration, osmotic balance [b1334]
focA	+6.52	11.15	+2.70	9.80	0.1	12.50	0.1	0.00001286	0.00037639	0.00871761	0.25945180	A_07_P004969	b0904	cmr|probable formate transporter [b0904]
focA	+120.94	13.45	+6.92	9.99	0.0	16.91	0.1	0.00000011	0.00007032	0.00001270	0.00909091	A_07_P040486	c_1239	cmr|F1C major fimbrial subunit precursor [c_1239]
focC	+15.24	10.13	+3.93	8.16	0.1	12.09	0.1	0.00000317	0.00019528	0.00153392	0.11955446	A_07_P040500	c_1241	cmr|F1C periplasmic chaperone [c_1241]
focD	+15.83	11.51	+3.98	9.52	0.3	13.50	0.1	0.00000798	0.00029787	0.00143039	0.11489796	A_07_P040505	c_1242	cmr|F1C fimbrial usher [c_1242]
focF	+19.76	11.05	+4.30	8.90	0.2	13.21	0.0	0.00000185	0.00016828	0.00080539	0.08453333	A_07_P040507	c_1243	cmr|F1C minor fimbrial subunit F precursor [c_1243]
focG	+15.59	11.29	+3.96	9.31	0.8	13.27	1.0	0.00380910	0.01922130	0.00148628	0.11758794	A_07_P040512	c_1244	cmr|F1C minor fimbrial subunit protein G presursor [c_1244]
focH	+14.79	10.76	+3.89	8.82	0.1	12.70	0.1	0.00000252	0.00018118	0.00163173	0.12350962	A_07_P040520	c_1245	cmr|F1C Putative fimbrial adhesin precursor [c_1245]
folB	-2.97	9.82	-1.57	10.60	0.1	9.03	0.2	0.00061160	0.00471780	0.03083524	0.49186424	A_07_P000021	b3058	cmr|putative kinase [b3058]
folD	-2.14	11.30	-1.10	11.85	0.1	10.75	0.1	0.00150882	0.00938185	0.07651677	0.67413542	A_07_P031265	c_0645	cmr|FolD bifunctional protein [c_0645]
folD	-2.39	10.05	-1.26	10.68	0.1	9.42	0.4	0.00902842	0.03816958	0.05649454	0.61724497	A_07_P016432	b0529	cmr|5,10-methylene-tetrahydrofolate dehydrogenase; 5,10-methylene-tetrahydrofolate cyclohydrolase [b0529]
folD	-2.21	12.91	-1.15	13.48	0.0	12.33	0.2	0.00221814	0.01257557	0.07016006	0.66302521	A_07_P043965	Z0684	cmr|5,10-methylene-tetrahydrofolate dehydrogenase 5,10-methylene-tetrahydrofolate cyclohydrolase [Z0684]
folE	+3.18	12.06	+1.67	11.23	0.0	12.89	0.0	0.00014962	0.00170945	0.02958968	0.43907634	A_07_P003527	b2153	cmr|GTP cyclohydrolase I [b2153]
folK	-2.24	8.45	-1.16	9.03	0.1	7.87	0.1	0.00129833	0.00842230	0.06895262	0.65793333	A_07_P002673	b0142	cmr|7,8-dihydro-6-hydroxymethylpterin- pyrophosphokinase [b0142]
fpr	+2.94	9.32	+1.55	8.54	0.1	10.10	0.1	0.00037872	0.00328517	0.03421494	0.47046288	A_07_P039438	c_4876	cmr|Ferredoxin--NADP reductase [c_4876]
fpr	+4.97	9.07	+2.31	7.91	0.1	10.22	0.1	0.00005859	0.00089563	0.01422510	0.32040057	A_07_P019396	b3924	cmr|ferredoxin-NADP reductase [b3924]
frdA	+2.57	14.83	+1.36	14.14	0.0	15.51	0.0	0.00040351	0.00344325	0.04291540	0.51302961	A_07_P011501	b4154	cmr|fumarate reductase, anaerobic, flavoprotein subunit [b4154]
frdB	+4.03	12.60	+2.01	11.59	0.1	13.60	0.1	0.00007887	0.00110461	0.01985328	0.37613718	A_07_P011497	b4153	cmr|fumarate reductase, anaerobic, iron-sulfur protein subunit [b4153]
frdC	+2.79	11.53	+1.48	10.79	0.2	12.27	0.0	0.00067780	0.00511569	0.03686738	0.48410342	A_07_P011491	b4152	cmr|fumarate reductase, anaerobic, membrane anchor polypeptide [b4152]
frdD	+2.92	9.84	+1.55	9.06	0.2	10.61	0.1	0.00043459	0.00364142	0.03454459	0.47169991	A_07_P011487	b4151	cmr|fumarate reductase, anaerobic, membrane anchor polypeptide [b4151]
frlD	+2.37	7.46	+1.25	6.84	0.1	8.09	0.0	0.00078349	0.00571074	0.04981072	0.55343684	A_07_P017900	b3374	cmr|putative kinase [b3374]
frmR	+3.94	10.28	+1.98	9.29	0.1	11.27	0.1	0.00009091	0.00121866	0.02072536	0.38253224	A_07_P020911	b0357	cmr|putative alpha helix chain [b0357]
frr	-4.46	12.99	-2.16	14.07	0.0	11.91	0.1	0.00004346	0.00073568	0.00911585	0.30536170	A_07_P059508	ECs0174	cmr|ribosome releasing factor [ECs0174]
frr	-3.77	13.69	-1.92	14.64	0.0	12.73	0.0	0.00007202	0.00103080	0.01452236	0.38043261	A_07_P049243	Z0183	cmr|ribosome releasing factor [Z0183]
frr	-3.33	13.51	-1.73	14.38	0.0	12.64	0.1	0.00012812	0.00154216	0.02164634	0.44490862	A_07_P035911	c_0208	cmr|Ribosome recycling factor [c_0208]
frr	-4.71	12.93	-2.24	14.05	0.0	11.82	0.0	0.00003014	0.00060327	0.00771341	0.28641509	A_07_P002818	b0172	cmr|ribosome releasing factor [b0172]
fruA	-2.44	8.91	-1.29	9.55	0.0	8.26	0.0	0.00055426	0.00436376	0.05424162	0.60824786	A_07_P000634	b2167	cmr|PTS system, fructose-specific transport protein [b2167]
fruA	-2.24	9.14	-1.17	9.72	0.1	8.55	0.4	0.00791826	0.03439987	0.06406250	0.64820051	A_07_P042526	c_2702	cmr|PTS system, fructose-specific IIBC component [c_2702]
fruR	+4.65	10.37	+2.22	9.26	0.1	11.48	0.0	0.00004175	0.00071517	0.01586192	0.33747297	A_07_P011076	b0080	cmr|transcriptional repressor of fru operon and others [b0080]
frvR	+2.32	7.66	+1.21	7.05	0.3	8.26	0.1	0.00539726	0.02527498	0.05312055	0.57322138	A_07_P047079	Z5440	cmr|putative frv operon regulatory protein [Z5440]
frwB	+2.02	7.37	+1.01	6.87	0.0	7.88	0.0	0.00183143	0.01090842	0.06817454	0.65367844	A_07_P019530	b3950	cmr|PTS system fructose-like IIB component 1 [b3950]
ftnA	+16.96	10.09	+4.08	8.05	0.0	12.14	0.0	0.00000120	0.00014031	0.00124047	0.10500000	A_07_P016193	b1905	cmr|cytoplasmic ferritin [b1905]
ftnB	+2.91	9.88	+1.54	9.11	0.1	10.65	0.1	0.00024819	0.00245286	0.03478150	0.47370242	A_07_P016180	b1902	cmr|ferritin-like protein [b1902]
ftsE	-14.82	10.62	-3.89	12.57	0.0	8.68	0.2	0.00000337	0.00019956	0.00015307	0.04228070	A_07_P007255	b3463	cmr|ATP-binding component of a membrane-associated complex involved in cell division [b3463]
ftsK	+3.38	10.54	+1.76	9.66	0.0	11.42	0.0	0.00011623	0.00144093	0.02676258	0.42092907	A_07_P004899	b0890	cmr|cell division protein [b0890]
ftsN	-3.30	11.24	-1.72	12.10	0.1	10.38	0.3	0.00061877	0.00474754	0.02187627	0.44729870	A_07_P019442	b3933	cmr|essential cell division protein [b3933]
ftsP	-2.15	9.15	-1.11	9.70	0.1	8.60	0.1	0.00134255	0.00862736	0.07491108	0.67317352	A_07_P012216	b3017	cmr|suppressor of ftsI [b3017]
ftsW	+2.87	8.97	+1.52	8.21	0.1	9.73	0.1	0.00032427	0.00296307	0.03546875	0.47687447	A_07_P011122	b0089	cmr|cell division; membrane protein involved in shape determination [b0089]
ftsX	-2.01	7.87	-1.01	8.37	0.0	7.37	0.1	0.00208212	0.01199888	0.09026105	0.72355906	A_07_P063559	ECs4311	cmr|cell division membrane protein FtsX [ECs4311]
ftsX	-2.38	8.14	-1.25	8.77	0.1	7.52	0.0	0.00079350	0.00575440	0.05730628	0.61966346	A_07_P052174	Z4836	cmr|cell division membrane protein [Z4836]
ftsX	-2.04	7.64	-1.03	8.16	0.1	7.13	0.1	0.00359783	0.01838386	0.08617315	0.71368227	A_07_P007248	b3462	cmr|cell division membrane protein [b3462]
ftsY	-8.48	12.69	-3.08	14.24	0.1	11.15	0.1	0.00000778	0.00029735	0.00126397	0.11502890	A_07_P007256	b3464	cmr|cell division membrane protein [b3464]
ftsZ	+2.96	13.53	+1.56	12.75	0.0	14.31	0.0	0.00021058	0.00218894	0.03387195	0.46778947	A_07_P002447	b0095	cmr|cell division; forms circumferential ring; tubulin-like GTP-binding protein and GTPase [b0095]
fucA	+10.52	11.68	+3.40	9.99	0.0	13.38	0.1	0.00000366	0.00020738	0.00322282	0.16690789	A_07_P005999	b2800	cmr|L-fuculose-1-phosphate aldolase [b2800]
fucI	+2.24	11.00	+1.17	10.42	0.1	11.59	0.1	0.00140029	0.00890035	0.05595020	0.58803738	A_07_P006006	b2802	cmr|L-fucose isomerase [b2802]
fucK	+6.53	9.85	+2.71	8.50	0.1	11.20	0.2	0.00003654	0.00065813	0.00876715	0.25994350	A_07_P006014	b2803	cmr|L-fuculokinase [b2803]
fucK	+14.64	12.66	+3.87	10.72	0.3	14.60	0.1	0.00001252	0.00037048	0.00168255	0.12263889	A_07_P043254	Z4120	cmr|L-fuculokinase [Z4120]
fucO	+10.18	10.44	+3.35	8.77	0.0	12.11	0.7	0.00051462	0.00413586	0.00377541	0.18289231	A_07_P005991	b2799	cmr|L-1,2-propanediol oxidoreductase [b2799]
fucR	+4.21	8.29	+2.07	7.26	0.1	9.33	0.1	0.00006676	0.00098312	0.01846291	0.36289638	A_07_P006024	b2805	cmr|positive regulator of the fuc operon [b2805]
fucU	+12.13	12.10	+3.60	10.30	0.1	13.90	0.0	0.00000260	0.00018134	0.00241171	0.14437262	A_07_P006018	b2804	cmr|protein of fucose operon [b2804]
fumA	+18.19	10.69	+4.19	8.60	0.0	12.79	0.2	0.00000281	0.00018406	0.00101118	0.09255814	A_07_P020691	b1612	cmr|fumarase A = fumarate hydratase Class I; aerobic isozyme [b1612]
fumC	+13.44	9.33	+3.75	7.46	0.0	11.21	0.1	0.00000225	0.00017963	0.00200013	0.13231092	A_07_P042312	c_2003	cmr|Fumarate hydratase class II [c_2003]
fumC	+19.97	9.48	+4.32	7.32	0.1	11.64	0.3	0.00000556	0.00025342	0.00079522	0.08517007	A_07_P020689	b1611	cmr|fumarase C= fumarate hydratase Class II; isozyme [b1611]
fur	-3.81	12.22	-1.93	13.18	0.0	11.25	0.2	0.00012518	0.00151602	0.01474657	0.38375207	A_07_P009932	b0683	cmr|negative regulator [b0683]
gabT	+2.48	9.05	+1.31	8.40	0.2	9.71	0.2	0.00244725	0.01353589	0.04718369	0.54026182	A_07_P020162	b2662	cmr|4-aminobutyrate aminotransferase activity [b2662]
galF	+2.15	10.01	+1.11	9.46	0.1	10.57	0.2	0.00384654	0.01936263	0.06010036	0.61363165	A_07_P015526	b2042	cmr|homolog of Salmonella UTP--glucose-1-P uridyltransferase, probably a UDP-gal transferase [b2042]
galK	+12.47	11.97	+3.64	10.15	0.1	13.79	0.1	0.00000373	0.00020748	0.00233041	0.14444882	A_07_P004240	b0757	cmr|galactokinase [b0757]
galM	+11.25	11.13	+3.49	9.38	0.1	12.87	0.1	0.00000472	0.00023240	0.00283600	0.15666667	A_07_P004231	b0756	cmr|galactose-1-epimerase [b0756]
galR	-2.19	10.81	-1.13	11.37	0.1	10.24	0.1	0.00124997	0.00818958	0.07282012	0.66733411	A_07_P006169	b2837	cmr|repressor of galETK operon [b2837]
galS	+3.13	9.63	+1.65	8.80	0.2	10.45	0.0	0.00048126	0.00391373	0.03084794	0.44762212	A_07_P032245	c_2685	cmr|Mgl repressor and galactose ultrainduction factor [c_2685]
galS	+2.37	8.54	+1.25	7.92	0.0	9.17	0.1	0.00075424	0.00554378	0.04946329	0.55269695	A_07_P003519	b2151	cmr|mgl repressor, galactose operon inducer [b2151]
galT	+3.38	10.60	+1.76	9.72	0.0	11.48	0.2	0.00039104	0.00336794	0.02710620	0.42295342	A_07_P004241	b0758	cmr|galactose-1-phosphate uridylyltransferase [b0758]
gapA	+20.28	10.88	+4.34	8.71	0.1	13.05	0.2	0.00000217	0.00017840	0.00076283	0.08398601	A_07_P015590	b1779	cmr|glyceraldehyde-3-phosphate dehydrogenase A [b1779]
garP	-2.06	7.90	-1.04	8.43	0.1	7.38	0.1	0.00189286	0.01116147	0.08376842	0.70640064	A_07_P000357	b3127	cmr|putative transport protein [b3127]
gcvH	+476.83	12.32	+8.90	7.88	0.0	16.77	0.0	0.00000002	0.00007032	0.00000000	0.00000000	A_07_P041711	c_3484	cmr|Glycine cleavage system H protein [c_3484]
gcvH	+283.91	11.56	+8.15	7.49	0.2	15.64	0.1	0.00000007	0.00007032	0.00000191	0.00250000	A_07_P006489	b2904	cmr|in glycine cleavage complex, carrier of aminomethyl moiety via covalently bound lipoyl cofactor [b2904]
gcvH	+273.63	12.62	+8.10	8.57	0.3	16.66	0.2	0.00000024	0.00009047	0.00000191	0.00272727	A_07_P041275	c_3484	cmr|Glycine cleavage system H protein [c_3484]
gcvP	+13.43	9.73	+3.75	7.86	0.1	11.60	0.0	0.00000234	0.00017963	0.00204332	0.13293388	A_07_P032879	c_3483	cmr|Glycine dehydrogenase [c_3483]
gcvP	+14.42	9.57	+3.85	7.65	0.2	11.50	0.1	0.00000389	0.00021124	0.00177210	0.12624434	A_07_P052779	Z4240	cmr|glycine decarboxylase, P protein of glycine cleavage [Z4240]
gcvP	+27.79	9.44	+4.80	7.04	0.0	11.84	0.0	0.00000052	0.00009532	0.00039444	0.06029126	A_07_P006481	b2903	cmr|glycine decarboxylase, P protein of glycine cleavage system [b2903]
gcvR	+6.17	9.09	+2.62	7.78	0.1	10.40	0.2	0.00004583	0.00075801	0.00959667	0.26789007	A_07_P013453	b2479	cmr|transcriptional regulation of gcv operon [b2479]
gcvT	+162.57	11.62	+7.34	7.95	0.0	15.29	0.3	0.00000029	0.00009047	0.00000762	0.00600000	A_07_P006491	b2905	cmr|aminomethyltransferase [b2905]
gdhA	+6.32	9.41	+2.66	8.08	0.0	10.74	0.0	0.00001306	0.00037778	0.00935531	0.26887737	A_07_P052546	Z2793	cmr|NADP-specific glutamate dehydrogenase [Z2793]
gdhA	+5.34	9.86	+2.42	8.65	0.3	11.07	0.2	0.00023155	0.00234711	0.01231961	0.29432473	A_07_P018851	b1761	cmr|NADP-specific glutamate dehydrogenase [b1761]
gef	-3.16	12.82	-1.66	13.65	0.0	11.99	0.1	0.00017351	0.00191971	0.02574886	0.46920139	A_07_P048482	Z0016	cmr|Gef protein interferes with membrane function when [Z0016]
gef	-3.01	10.57	-1.59	11.36	0.1	9.77	0.0	0.00021578	0.00222917	0.02947345	0.48691501	A_07_P048478	Z0016	cmr|Gef protein interferes with membrane function when [Z0016]
ghrA	+2.13	10.06	+1.09	9.52	0.2	10.61	0.0	0.00220295	0.01250298	0.06144563	0.62172237	A_07_P001376	b1033	cmr|putative dehydrogenase [b1033]
gidB	-5.47	9.81	-2.45	11.04	0.1	8.58	0.0	0.00002346	0.00052393	0.00478150	0.22272189	A_07_P005623	b3740	cmr|glucose-inhibited division; chromosome replication? [b3740]
glcB	+3.96	9.33	+1.98	8.33	0.1	10.32	0.1	0.00009221	0.00123129	0.02060658	0.38123384	A_07_P012016	b2976	cmr|malate synthase G [b2976]
glcB	+6.81	11.47	+2.77	10.09	0.1	12.85	0.2	0.00002597	0.00055176	0.00797828	0.24824111	A_07_P041726	c_3705	cmr|Malate synthase G [c_3705]
glcF	+12.21	9.38	+3.61	7.58	0.1	11.19	0.1	0.00000483	0.00023673	0.00236217	0.14414729	A_07_P012026	b2978	cmr|glycolate oxidase iron-sulfur subunit [b2978]
glcG	+72.20	10.10	+6.17	7.01	0.0	13.18	0.1	0.00000016	0.00007760	0.00005526	0.02023256	A_07_P012024	b2977	cmr|orf, hypothetical protein [b2977]
gldA	+20.87	12.93	+4.38	10.74	0.2	15.12	0.1	0.00000218	0.00017840	0.00071456	0.08211679	A_07_P019501	b3945	cmr|glycerol dehydrogenase, [b3945]
glgA	+17.90	11.50	+4.16	9.42	0.1	13.58	0.1	0.00000165	0.00015927	0.00107597	0.09625000	A_07_P018171	b3429	cmr|glycogen synthase [b3429]
glgB	+9.42	10.12	+3.24	8.50	0.1	11.74	0.2	0.00001339	0.00038387	0.00413935	0.19111437	A_07_P018189	b3432	cmr|1,4-alpha-glucan branching enzyme [b3432]
glgC	+21.19	10.78	+4.41	8.57	0.1	12.98	0.0	0.00000109	0.00013374	0.00064278	0.07784615	A_07_P018179	b3430	cmr|glucose-1-phosphate adenylyltransferase [b3430]
glgP	+7.08	8.87	+2.82	7.46	0.3	10.28	0.1	0.00005891	0.00089791	0.00729357	0.24276956	A_07_P018166	b3428	cmr|glycogen phosphorylase [b3428]
glgS	+9.21	10.39	+3.20	8.79	0.0	12.00	0.0	0.00000472	0.00023240	0.00420732	0.19200000	A_07_P012366	b3049	cmr|glycogen biosynthesis, rpoS dependent [b3049]
glgX	+23.67	12.15	+4.56	9.87	0.1	14.43	0.2	0.00000215	0.00017840	0.00054751	0.07065574	A_07_P018182	b3431	cmr|part of glycogen operon, a glycosyl hydrolase, debranching enzyme [b3431]
glk	-2.74	8.13	-1.46	8.86	0.1	7.40	0.2	0.00102928	0.00705795	0.03806275	0.53031858	A_07_P014224	b2388	cmr|glucokinase [b2388]
glmM	+2.04	10.84	+1.03	10.32	0.1	11.35	0.2	0.00533183	0.02504949	0.06670287	0.64348652	A_07_P000591	b3176	cmr|similar to phosphoglucomutases and phosphomannomutases [b3176]
glmU	-2.31	11.75	-1.21	12.35	0.1	11.14	0.1	0.00143516	0.00903805	0.06129065	0.63442472	A_07_P005571	b3730	cmr|N-acetyl glucosamine-1-phosphate uridyltransferase [b3730]
glmU	-3.13	12.63	-1.65	13.45	0.1	11.80	0.0	0.00017863	0.00195167	0.02641197	0.47361048	A_07_P052237	Z5228	cmr|N-acetyl glucosamine-1-phosphate uridyltransferase [Z5228]
glnA	-3.46	13.41	-1.79	14.30	0.0	12.51	0.1	0.00011433	0.00142293	0.01886941	0.42019802	A_07_P019129	b3870	cmr|glutamine synthetase [b3870]
glnB	+2.70	10.83	+1.43	10.12	0.1	11.55	0.0	0.00033814	0.00304380	0.03962081	0.49943155	A_07_P019668	b2553	cmr|regulatory protein P-II for glutamine synthetase [b2553]
glnD	+3.53	8.80	+1.82	7.89	0.1	9.71	0.1	0.00013741	0.00161341	0.02498349	0.40972917	A_07_P002791	b0167	cmr|protein PII; uridylyltransferase acts on regulator of glnA [b0167]
glnK	-2.42	7.77	-1.28	8.41	0.2	7.14	0.2	0.00258235	0.01415162	0.05430894	0.60856940	A_07_P021363	b0450	cmr|nitrogen regulatory protein P-II 2 [b0450]
glnS	-2.63	9.78	-1.40	10.48	0.2	9.08	0.1	0.00111171	0.00747664	0.04276867	0.56253133	A_07_P009919	b0680	cmr|glutamine tRNA synthetase [b0680]
gloA	+2.38	9.10	+1.25	8.47	0.2	9.72	0.1	0.00132396	0.00853580	0.04901232	0.54960826	A_07_P020890	b1651	cmr|lactoylglutathione lyase [b1651]
glpB	-3.10	11.15	-1.63	11.97	0.0	10.34	0.0	0.00017689	0.00193937	0.02704586	0.47790123	A_07_P041234	c_2783	cmr|Anaerobic glycerol-3-phosphate dehydrogenase subunit B [c_2783]
glpB	-2.51	10.35	-1.33	11.02	0.1	9.69	0.1	0.00084041	0.00603072	0.04994601	0.58946777	A_07_P000998	b2242	cmr|sn-glycerol-3-phosphate dehydrogenase [b2242]
glpE	-2.51	9.69	-1.33	10.36	0.0	9.03	0.0	0.00047351	0.00387677	0.05005843	0.58991018	A_07_P018153	b3425	cmr|protein of glp regulon [b3425]
glpF	+26.40	10.41	+4.72	8.05	0.1	12.77	0.1	0.00000071	0.00011547	0.00043382	0.06383178	A_07_P019412	b3927	cmr|facilitated diffusion of glycerol [b3927]
glpK	+3.23	10.24	+1.69	9.40	0.1	11.09	0.0	0.00015179	0.00173170	0.02894118	0.43896917	A_07_P019409	b3926	cmr|glycerol kinase [b3926]
glpQ	+2.58	8.31	+1.37	7.63	0.2	8.99	0.3	0.00409773	0.02033244	0.04362551	0.51797888	A_07_P000982	b2239	cmr|glycerophosphodiester phosphodiesterase, periplasmic [b2239]
glpT	+14.62	9.57	+3.87	7.63	0.1	11.50	0.0	0.00000287	0.00018535	0.00166984	0.12400943	A_07_P063326	ECs3125	cmr|sn-glycerol-3-phosphate permease [ECs3125]
glpT	+11.50	9.63	+3.52	7.87	0.1	11.39	0.0	0.00000328	0.00019644	0.00267848	0.15114695	A_07_P052656	Z3498	cmr|sn-glycerol-3-phosphate permease [Z3498]
glpT	+9.66	10.03	+3.27	8.39	0.2	11.67	0.1	0.00001193	0.00036114	0.00387830	0.18447130	A_07_P000990	b2240	cmr|sn-glycerol-3-phosphate permease [b2240]
gltA	+53.23	13.91	+5.73	11.04	0.1	16.78	0.0	0.00000033	0.00009117	0.00010861	0.02898305	A_07_P056596	ECs0745	cmr|citrate synthase [ECs0745]
gltA	+15.90	11.19	+3.99	9.19	0.2	13.18	0.0	0.00000271	0.00018406	0.00137767	0.11238342	A_07_P010118	b0720	cmr|citrate synthase [b0720]
gltA	+43.57	14.45	+5.45	11.72	0.1	17.17	0.0	0.00000032	0.00009117	0.00015625	0.03324324	A_07_P046286	Z0873	cmr|citrate synthase [Z0873]
gltD	+2.73	8.83	+1.45	8.11	0.1	9.56	0.0	0.00035091	0.00312664	0.03902185	0.49545161	A_07_P012545	b3213	cmr|glutamate synthase, small subunit [b3213]
gltI	+6.08	12.20	+2.60	10.89	0.1	13.50	0.1	0.00002286	0.00051643	0.00998920	0.27115517	A_07_P009841	b0655	cmr|putative periplasmic binding transport protein [b0655]
gltJ	+5.76	9.78	+2.53	8.52	0.0	11.04	0.1	0.00001812	0.00044149	0.01115663	0.28239550	A_07_P009836	b0654	cmr|glutamate/aspartate transport system permease [b0654]
gltK	+11.86	9.35	+3.57	7.56	0.1	11.13	0.4	0.00003180	0.00061949	0.00248349	0.14428044	A_07_P009831	b0653	cmr|glutamate/aspartate transport system permease [b0653]
gltL	+15.19	9.93	+3.93	7.97	0.1	11.89	0.0	0.00000173	0.00016209	0.00152312	0.11990000	A_07_P009830	b0652	cmr|ATP-binding protein of glutamate/aspartate transport system [b0652]
gltP	+2.83	8.57	+1.50	7.82	0.2	9.32	0.2	0.00149888	0.00933386	0.03676448	0.48396321	A_07_P009026	b4077	cmr|glutamate-aspartate symport protein [b4077]
gltX	-2.74	12.16	-1.45	12.88	0.1	11.43	0.1	0.00034416	0.00308214	0.03867505	0.53506151	A_07_P014274	b2400	cmr|glutamate tRNA synthetase, catalytic subunit [b2400]
glvG	-4.24	9.26	-2.08	10.30	0.4	8.22	0.2	0.00089223	0.00631620	0.01015371	0.31844622	A_07_P010606	ABE-0285077	asap|C-terminal fragment of predicted 6-phospho-beta-glucosidase (pseudogene) [b4556]
glyA	+4.27	11.28	+2.09	10.23	0.1	12.32	0.3	0.00023100	0.00234638	0.01831174	0.36447535	A_07_P019657	b2551	cmr|serine hydroxymethyltransferase [b2551]
glyQ	-2.06	11.57	-1.04	12.09	0.0	11.05	0.1	0.00192662	0.01130132	0.08327490	0.70412460	A_07_P007728	b3560	cmr|glycine tRNA synthetase, alpha subunit [b3560]
gmhA	+3.97	9.39	+1.99	8.40	0.0	10.38	0.1	0.00006465	0.00096109	0.02041095	0.37939787	A_07_P053536	ECs0249	cmr|phosphoheptose isomerase [ECs0249]
gmhA	+3.03	9.19	+1.60	8.39	0.2	9.99	0.1	0.00061317	0.00472758	0.03249111	0.45919210	A_07_P043366	Z0280	cmr|phosphoheptose isomerase [Z0280]
gmhA	+7.08	11.73	+2.82	10.32	0.0	13.14	0.0	0.00000921	0.00032221	0.00749492	0.24532225	A_07_P030845	c_0372	cmr|Phosphoheptose isomerase [c_0372]
gmk	-17.58	12.42	-4.14	14.48	0.0	10.35	0.2	0.00000246	0.00017989	0.00008638	0.02720000	A_07_P017181	b3648	cmr|guanylate kinase [b3648]
gnsA	+2.74	11.11	+1.45	10.39	0.1	11.84	0.1	0.00060988	0.00470682	0.03879446	0.49536091	A_07_P010343	ABE-0285038	asap|predicted regulator of phosphatidylethanolamine synthesis [b4517]
gnsB	+19.99	10.23	+4.32	8.07	0.5	12.39	0.0	0.00003283	0.00062948	0.00080285	0.08483221	A_07_P020394	b1550	cmr|orf, hypothetical protein [b1550]
gntK	-2.41	9.33	-1.27	9.96	0.1	8.69	0.0	0.00082791	0.00595734	0.05625127	0.61672702	A_07_P018213	b3437	cmr|gluconokinase 2, thermoresistant [b3437]
gntT	-2.00	7.35	-1.00	7.85	0.0	6.85	0.2	0.00366050	0.01863115	0.08867505	0.72224521	A_07_P018104	b3415	cmr|high-affinity transport of gluconate / gluconate permease [b3415]
gntX	+2.06	11.50	+1.04	10.98	0.1	12.02	0.0	0.00233688	0.01308389	0.06588796	0.64191832	A_07_P018091	b3413	cmr|orf, hypothetical protein [b3413]
gntY	-5.73	10.61	-2.52	11.87	0.1	9.35	0.1	0.00003088	0.00060993	0.00425368	0.21126183	A_07_P018096	b3414	cmr|orf, hypothetical protein [b3414]
gntY	-6.01	10.83	-2.59	12.12	0.1	9.54	0.2	0.00005150	0.00081576	0.00371253	0.20085911	A_07_P018096	b3414	cmr|orf, hypothetical protein [b3414]
gntY	-5.61	10.73	-2.49	11.97	0.1	9.49	0.2	0.00006747	0.00098907	0.00458016	0.22051988	A_07_P018096	b3414	cmr|orf, hypothetical protein [b3414]
gntY	-6.67	10.65	-2.74	12.02	0.1	9.28	0.2	0.00004427	0.00074459	0.00266895	0.17435685	A_07_P018096	b3414	cmr|orf, hypothetical protein [b3414]
gntY	-5.48	10.70	-2.46	11.93	0.1	9.47	0.3	0.00013972	0.00163451	0.00491425	0.22491279	A_07_P018096	b3414	cmr|orf, hypothetical protein [b3414]
gor	-3.13	11.07	-1.64	11.89	0.5	10.25	0.5	0.01258669	0.04974016	0.02725165	0.47885045	A_07_P007436	b3500	cmr|glutathione oxidoreductase [b3500]
gph	+6.53	10.07	+2.71	8.72	0.1	11.42	0.1	0.00002251	0.00051278	0.00870046	0.25992410	A_07_P017954	b3385	cmr|phosphoglycolate phosphatase [b3385]
gpmA	+3.24	7.85	+1.70	7.01	0.2	8.70	0.1	0.00039496	0.00339054	0.02912221	0.43791786	A_07_P004227	b0755	cmr|phosphoglyceromutase 1 [b0755]
gpp	+3.01	9.46	+1.59	8.67	0.1	10.26	0.1	0.00028392	0.00267666	0.03264926	0.46018800	A_07_P005780	b3779	cmr|guanosine pentaphosphatase; exopolyphosphatase [b3779]
gpsA	+6.04	11.27	+2.59	9.97	0.1	12.57	0.1	0.00002108	0.00049176	0.01005335	0.27242685	A_07_P016982	b3608	cmr|glycerol-3-phosphate dehydrogenase [b3608]
greA	-8.97	10.03	-3.17	11.62	0.1	8.45	0.2	0.00001756	0.00043403	0.00100673	0.10359477	A_07_P000616	b3181	cmr|transcription elongation factor: cleaves 3 nucleotide of paused mRNA [b3181]
greB	-2.22	11.02	-1.15	11.59	0.1	10.44	0.1	0.00129470	0.00840722	0.06859756	0.65893838	A_07_P018057	b3406	cmr|transcription elongation factor and transcript cleavage [b3406]
groL	+17.16	13.64	+4.10	11.59	0.0	15.69	0.0	0.00000131	0.00014643	0.00120363	0.10243243	A_07_P011446	b4143	cmr|GroEL, chaperone Hsp60, peptide-dependent ATPase, heat shock protein [b4143]
groS	+21.29	14.38	+4.41	12.17	0.1	16.59	0.0	0.00000100	0.00012946	0.00063707	0.07775194	A_07_P011443	b4142	cmr|GroES, 10 Kd chaperone binds to Hsp60 in pres. Mg-ATP, suppressing its ATPase activity [b4142]
grxA	-3.03	8.03	-1.60	8.83	0.1	7.23	0.1	0.00021400	0.00221369	0.02885607	0.48433902	A_07_P004696	b0849	cmr|glutaredoxin1 redox coenzyme for glutathione-dependent ribonucleotide reductase [b0849]
grxB	+18.20	10.17	+4.19	8.08	0.2	12.27	0.0	0.00000269	0.00018406	0.00100864	0.09286550	A_07_P001521	b1064	cmr|glutaredoxin 2 [b1064]
grxD	-2.34	12.58	-1.23	13.19	0.1	11.97	0.0	0.00073992	0.00547684	0.06041603	0.63160027	A_07_P018331	b1654	cmr|orf, hypothetical protein [b1654]
gshA	-4.35	10.15	-2.12	11.21	0.0	9.09	0.0	0.00004068	0.00070309	0.00968623	0.30995935	A_07_P001912	b2688	cmr|gamma-glutamate-cysteine ligase [b2688]
gshB	+2.83	11.43	+1.50	10.68	0.1	12.18	0.1	0.00031841	0.00292649	0.03661141	0.48316010	A_07_P011876	b2947	cmr|glutathione synthetase [b2947]
gsiA	+2.85	9.24	+1.51	8.49	0.2	10.00	0.0	0.00046372	0.00382438	0.03608422	0.47982264	A_07_P004599	b0829	cmr|putative ATP-binding component of a transport system [b0829]
gspD	-2.92	7.80	-1.55	8.57	0.0	7.03	0.2	0.00046454	0.00382719	0.03155170	0.49231913	A_07_P013055	b3325	cmr|putative general protein secretion protein [b3325]
gspL	-4.10	9.58	-2.04	10.60	0.1	8.56	0.1	0.00011136	0.00139929	0.01140689	0.34470250	A_07_P013091	b3333	cmr|putative general protein secretion protein [b3333]
gspM	-2.49	8.07	-1.31	8.72	0.1	7.41	0.1	0.00080569	0.00582985	0.05024517	0.59122571	A_07_P017699	b3334	cmr|putative general secretion [b3334]
gst	+11.30	10.41	+3.50	8.66	0.1	12.16	0.2	0.00000940	0.00032230	0.00288745	0.15784722	A_07_P020808	b1635	cmr|glutathionine S-transferase [b1635]
guaB	-2.17	9.30	-1.12	9.86	0.1	8.74	0.1	0.00140467	0.00890948	0.07249873	0.66788765	A_07_P013596	b2508	cmr|IMP dehydrogenase [b2508]
gutM	+2.33	7.53	+1.22	6.92	0.0	8.14	0.0	0.00069525	0.00521237	0.05087398	0.55854951	A_07_P001980	b2706	cmr|glucitol operon activator [b2706]
gyrA	-7.18	12.62	-2.84	14.04	0.0	11.20	0.1	0.00001257	0.00037059	0.00212398	0.15773585	A_07_P000942	b2231	cmr|DNA gyrase, subunit A, type II topoisomerase [b2231]
gyrB	-8.95	12.20	-3.16	13.78	0.0	10.62	0.0	0.00000504	0.00024251	0.00104548	0.10417722	A_07_P005429	b3699	cmr|DNA gyrase subunit B, type II topoisomerase, ATPase activity [b3699]
hchA	+8.24	11.21	+3.04	9.69	0.3	12.73	0.2	0.00006637	0.00097839	0.00542556	0.21570707	A_07_P015194	b1967	cmr|orf, hypothetical protein [b1967]
hda	-6.10	10.37	-2.61	11.68	0.0	9.07	0.2	0.00003919	0.00068397	0.00353214	0.19719858	A_07_P013536	b2496	cmr|putative DNA replication factor [b2496]
hdeA	+7.84	8.90	+2.97	7.41	0.1	10.38	0.1	0.00001199	0.00036220	0.00610010	0.22597647	A_07_P007483	b3510	cmr|orf, hypothetical protein [b3510]
hdhA	+3.97	10.10	+1.99	9.11	0.1	11.10	0.1	0.00012867	0.00154635	0.02046939	0.38003538	A_07_P035106	c_2011	cmr|7-alpha-hydroxysteroid dehydrogenase [c_2011]
hemB	-4.96	9.62	-2.31	10.77	0.0	8.46	0.0	0.00002596	0.00055176	0.00660569	0.26262626	A_07_P043796	Z0468	cmr|5-aminolevulinate dehydratase = porphobilinogen synthase [Z0468]
hemB	-5.15	9.64	-2.36	10.82	0.0	8.46	0.1	0.00003837	0.00067798	0.00583651	0.24635389	A_07_P020966	b0369	cmr|5-aminolevulinate dehydratase = porphobilinogen synthase [b0369]
hemC	+8.46	12.01	+3.08	10.47	0.1	13.55	0.1	0.00000820	0.00030062	0.00514482	0.21315789	A_07_P039097	c_4724	cmr|Porphobilinogen deaminase [c_4724]
hemC	+5.35	10.09	+2.42	8.88	0.1	11.30	0.0	0.00003520	0.00065109	0.01266832	0.29768657	A_07_P005892	b3805	cmr|porphobilinogen deaminase = hydroxymethylbilane synthase [b3805]
hemE	+2.51	8.85	+1.33	8.19	0.1	9.51	0.2	0.00171837	0.01040141	0.04567645	0.53466914	A_07_P008648	b3997	cmr|uroporphyrinogen decarboxylase [b3997]
hemF	-4.04	10.94	-2.02	11.95	0.0	9.93	0.5	0.00142933	0.00902664	0.01181085	0.34757009	A_07_P013240	b2436	cmr|coproporphyrinogen III oxidase [b2436]
hemH	-2.15	7.66	-1.11	8.21	0.0	7.10	0.0	0.00112978	0.00758172	0.07564850	0.67479320	A_07_P021484	b0475	cmr|ferrochelatase: final enzyme of heme biosynthesis [b0475]
hemN	+2.21	8.16	+1.14	7.59	0.0	8.73	0.1	0.00143372	0.00903332	0.05628684	0.58882392	A_07_P041357	c_4816	cmr|Oxygen-independent coproporphyrinogen III oxidase [c_4816]
hemX	+2.81	11.49	+1.49	10.75	0.1	12.24	0.1	0.00036542	0.00320871	0.03690485	0.48378851	A_07_P005885	b3803	cmr|uroporphyrinogen III methylase [b3803]
hemY	+2.14	10.35	+1.09	9.81	0.1	10.90	0.0	0.00209723	0.01205065	0.06097434	0.61934194	A_07_P005880	b3802	cmr|a late step of protoheme IX synthesis [b3802]
hepA	-13.46	9.82	-3.75	11.70	0.1	7.95	0.0	0.00000217	0.00017840	0.00024771	0.05200000	A_07_P010981	b0059	cmr|probable ATP-dependent RNA helicase [b0059]
hflB	-5.11	12.82	-2.35	13.99	0.0	11.64	0.0	0.00002412	0.00053180	0.00586001	0.24602667	A_07_P000601	b3178	cmr|degrades sigma32, integral membrane peptidase, cell division protein [b3178]
hflX	+2.05	11.91	+1.03	11.40	0.1	12.43	0.2	0.00370826	0.01881496	0.06758321	0.64998778	A_07_P011585	b4173	cmr|GTP - binding subunit of protease specific for phage lambda cII repressor [b4173]
hha	-3.45	10.25	-1.79	11.15	0.1	9.36	0.1	0.00013513	0.00159244	0.01935912	0.41866758	A_07_P021406	b0460	cmr|haemolysin expression modulating protein [b0460]
hinT	+2.25	11.23	+1.17	10.65	0.1	11.82	0.0	0.00145743	0.00913904	0.05485455	0.58313977	A_07_P001720	b1103	cmr|orf, hypothetical protein [b1103]
hisA	+8.23	9.34	+3.04	7.82	0.1	10.86	0.0	0.00000933	0.00032222	0.00537729	0.21652174	A_07_P015442	b2024	cmr|N- [b2024]
hisA	+9.34	9.28	+3.22	7.67	0.2	10.89	0.1	0.00001308	0.00037778	0.00413935	0.19055556	A_07_P051776	Z3186	cmr|N- [Z3186]
hisB	+12.18	9.29	+3.61	7.48	0.2	11.09	0.2	0.00001015	0.00033586	0.00244538	0.14365672	A_07_P015435	b2022	cmr|imidazoleglycerolphosphate dehydratase and histidinol-phosphate phosphatase [b2022]
hisC	+13.49	9.30	+3.75	7.43	0.1	11.18	0.1	0.00000402	0.00021605	0.00190295	0.12803419	A_07_P015429	b2021	cmr|histidinol-phosphate aminotransferase [b2021]
hisD	+27.57	10.23	+4.78	7.83	0.1	12.62	0.1	0.00000071	0.00011547	0.00039571	0.05990385	A_07_P015421	b2020	cmr|L-histidinal:NAD+ oxidoreductase; L-histidinol:NAD+ oxidoreductase [b2020]
hisF	+17.92	10.24	+4.16	8.16	0.6	12.32	0.0	0.00008279	0.00114139	0.00104929	0.09494253	A_07_P015446	b2025	cmr|imidazole glycerol phosphate synthase subunit in heterodimer with HisH = imidazole glycerol phsphate synthase holoenzyme [b2025]
hisG	+8.24	8.74	+3.04	7.22	0.0	10.27	0.0	0.00000610	0.00026402	0.00540714	0.21661578	A_07_P031978	c_2546	cmr|ATP phosphoribosyltransferase [c_2546]
hisG	+4.01	8.05	+2.00	7.04	0.2	9.05	0.2	0.00029149	0.00273170	0.02014482	0.37802145	A_07_P015420	b2019	cmr|ATP phosphoribosyltransferase [b2019]
hisH	+22.67	9.74	+4.50	7.49	0.1	11.99	0.0	0.00000108	0.00013374	0.00056339	0.07211382	A_07_P015440	b2023	cmr|glutamine amidotransferase subunit of heterodimer with HisF = imidazole glycerol phosphate synthase holoenzyme [b2023]
hisI	+2.58	7.63	+1.37	6.95	0.2	8.31	0.1	0.00088942	0.00630766	0.04250572	0.51123759	A_07_P044983	Z3188	cmr|phosphoribosyl-amp cyclohydrolase phosphoribosyl-ATP pyrophosphatase [Z3188]
hisI	+6.24	8.45	+2.64	7.13	0.2	9.78	0.1	0.00003684	0.00066063	0.00950584	0.26772809	A_07_P042478	c_2553	cmr|Histidine biosynthesis bifunctional protein hisIE [c_2553]
hisJ	+8.89	11.36	+3.15	9.78	0.0	12.93	0.0	0.00000489	0.00023837	0.00460493	0.20138889	A_07_P013834	b2309	cmr|histidine-binding periplasmic protein of high-affinity histidine transport system [b2309]
hisM	+4.43	9.41	+2.15	8.33	0.1	10.48	0.5	0.00162510	0.00995935	0.01703189	0.34824675	A_07_P013822	b2307	cmr|histidine transport, membrane protein M [b2307]
hisQ	+3.36	8.72	+1.75	7.84	0.0	9.59	0.1	0.00013566	0.00159753	0.02674987	0.42157157	A_07_P032423	c_2850	cmr|Histidine transport system permease protein hisQ [c_2850]
hlyC	+18.88	10.36	+4.24	8.24	0.1	12.48	0.1	0.00000142	0.00015187	0.00092480	0.08771084	A_07_P033053	c_3569	cmr|Hemolysin C [c_3569]
hmp	+7.05	8.96	+2.82	7.56	0.2	10.37	0.2	0.00004393	0.00074046	0.00749365	0.24579167	A_07_P019661	b2552	cmr|dihydropteridine reductase, ferrisiderophore reductase activity [b2552]
hmpA	+9.07	9.31	+3.18	7.72	0.4	10.90	0.0	0.00006571	0.00096957	0.00448044	0.19703911	A_07_P041601	c_3075	cmr|Flavohemoprotein [c_3075]
holC	+8.15	10.36	+3.03	8.84	0.2	11.87	0.0	0.00001552	0.00040864	0.00551575	0.21655860	A_07_P003845	b4259	cmr|DNA polymerase III, chi subunit [b4259]
holD	-2.33	11.68	-1.22	12.29	0.1	11.07	0.1	0.00091167	0.00643937	0.06091908	0.63391276	A_07_P017541	b4372	cmr|DNA polymerase III, psi subunit [b4372]
holE	+4.43	8.89	+2.15	7.82	0.1	9.96	0.0	0.00005386	0.00084214	0.01726372	0.35161708	A_07_P015890	b1842	cmr|DNA polymerase III, theta subunit [b1842]
holE	+3.38	9.16	+1.76	8.28	0.3	10.04	0.1	0.00097489	0.00676750	0.02750445	0.42579154	A_07_P035500	c_2252	cmr|DNA polymerase III, theta subunit [c_2252]
hpf	+54.17	12.97	+5.76	10.09	0.0	15.85	0.0	0.00000018	0.00008010	0.00010925	0.02819672	A_07_P012495	b3203	cmr|probable sigma-54 modulation protein [b3203]
hpt	-4.72	12.35	-2.24	13.47	0.0	11.23	0.2	0.00006086	0.00091430	0.00767213	0.28555556	A_07_P002588	b0125	cmr|hypoxanthine phosphoribosyltransferase [b0125]
hrpB	-3.19	10.08	-1.68	10.92	0.0	9.24	0.0	0.00014418	0.00166909	0.02474149	0.46043735	A_07_P035880	c_0181	cmr|ATP-dependent helicase hrpB [c_0181]
hscB	-5.11	10.24	-2.35	11.41	0.1	9.06	0.1	0.00003646	0.00065813	0.00600292	0.24805774	A_07_P019538	b2527	cmr|orf, hypothetical protein [b2527]
hslV	+4.25	11.82	+2.09	10.78	0.1	12.87	0.4	0.00061783	0.00474475	0.01840955	0.36457862	A_07_P019439	b3932	cmr|heat shock protein hslVU, proteasome-related peptidase subunit [b3932]
hspQ	-2.97	14.16	-1.57	14.94	0.1	13.37	0.0	0.00020482	0.00214405	0.03052846	0.49044898	A_07_P005278	b0966	cmr|orf, hypothetical protein [b0966]
hsrA	-2.27	9.70	-1.18	10.29	0.1	9.11	0.2	0.00232020	0.01303218	0.06531186	0.65328463	A_07_P005693	b3754	cmr|putative transport protein [b3754]
htpG	+8.15	9.49	+3.03	7.97	0.2	11.00	0.0	0.00001525	0.00040465	0.00552718	0.21646766	A_07_P021471	b0473	cmr|chaperone Hsp90, heat shock protein C 62.5 [b0473]
htrE	-2.22	7.78	-1.15	8.36	0.1	7.21	0.3	0.00652586	0.02941399	0.07018801	0.66249400	A_07_P049225	Z0150	cmr|putative fimbrial usher protein [Z0150]
htrG	-3.29	10.34	-1.72	11.20	0.1	9.48	0.2	0.00045938	0.00379654	0.02237868	0.44940051	A_07_P000008	b3055	cmr|orf, hypothetical protein [b3055]
hupA	+43.29	13.37	+5.44	10.65	0.0	16.09	0.0	0.00000027	0.00009047	0.00015625	0.03280000	A_07_P008662	b4000	cmr|DNA-binding protein HU-alpha [b4000]
hupB	+5.01	12.51	+2.32	11.35	0.1	13.68	0.1	0.00003842	0.00067808	0.01411712	0.31979856	A_07_P021314	b0440	cmr|DNA-binding protein HU-beta, NS1 [b0440]
hybA	+2.97	13.29	+1.57	12.50	0.1	14.07	0.1	0.00035235	0.00313416	0.03353214	0.46678161	A_07_P036723	c_3733	cmr|Hydrogenase-2 operon protein hybA precursor [c_3733]
hybA	+5.79	11.08	+2.53	9.81	0.1	12.34	0.2	0.00003946	0.00068718	0.01080602	0.27799020	A_07_P012111	b2996	cmr|hydrogenase-2 small subunit [b2996]
hybB	+15.50	11.94	+3.95	9.96	0.0	13.92	0.1	0.00000157	0.00015843	0.00145960	0.11606061	A_07_P012108	b2995	cmr|probable cytochrome Ni/Fe component of hydrogenase-2 [b2995]
hybC	+8.44	10.76	+3.08	9.22	0.0	12.30	0.1	0.00000625	0.00026673	0.00512068	0.21384615	A_07_P012105	b2994	cmr|probable large subunit, hydrogenase-2 [b2994]
hybD	+5.95	11.47	+2.57	10.18	0.2	12.76	0.1	0.00003896	0.00068231	0.01040968	0.27730964	A_07_P012100	b2993	cmr|probable processing element for hydrogenase-2 [b2993]
hybD	+4.63	12.28	+2.21	11.18	0.2	13.39	0.1	0.00009997	0.00130806	0.01591908	0.33686828	A_07_P045813	Z4347	cmr|probable processing element for hydrogenase-2 [Z4347]
hybE	+4.03	9.49	+2.01	8.49	0.0	10.50	0.2	0.00010202	0.00132269	0.02009527	0.37754177	A_07_P012094	b2992	cmr|member of hyb operon [b2992]
hybF	+4.08	11.17	+2.03	10.16	0.2	12.19	0.0	0.00010631	0.00135574	0.01938453	0.37446626	A_07_P012088	b2991	cmr|may modulate levels of hydrogenase-2 [b2991]
hybG	+2.53	10.11	+1.34	9.44	0.1	10.78	0.4	0.00514962	0.02433241	0.04615727	0.53789785	A_07_P012083	b2990	cmr|hydrogenase-2 operon protein: may effect maturation of large subunit of hydrogenase-2 [b2990]
hybO	+2.34	13.18	+1.23	12.56	0.0	13.79	0.1	0.00080022	0.00580047	0.05074822	0.55794693	A_07_P012116	b2997	cmr|putative hydrogenase subunit [b2997]
hycI	-2.22	8.35	-1.15	8.93	0.1	7.78	0.2	0.00243585	0.01348930	0.06806021	0.65900369	A_07_P002034	b2717	cmr|protease involved in processing C-terminal end of the large subunit of hydrogenase 3 [b2717]
hydN	-4.96	7.91	-2.31	9.07	0.0	6.76	0.0	0.00002737	0.00056692	0.00654726	0.26295918	A_07_P043108	Z4021	cmr|involved in electron transport from formate to [Z4021]
hydN	-5.59	8.34	-2.48	9.58	0.0	7.10	0.1	0.00002701	0.00056519	0.00459477	0.22054878	A_07_P002014	b2713	cmr|involved in electron transport from formate to hydrogen, Fe-S centers [b2713]
hydN	-3.31	8.52	-1.73	9.38	0.1	7.65	0.0	0.00014764	0.00169545	0.02239202	0.44852417	A_07_P030619	c_3269	cmr|Electron transport protein hydN [c_3269]
hypB	+2.92	12.97	+1.55	12.20	0.2	13.74	0.2	0.00144842	0.00909974	0.03489837	0.47406385	A_07_P041672	c_3287	cmr|Hydrogenase isoenzymes nickel incorporation protein hypB [c_3287]
hypB	+3.00	10.04	+1.58	9.24	0.1	10.83	0.1	0.00023444	0.00236134	0.03297320	0.46350893	A_07_P002083	b2727	cmr|guanine-nucleotide binding protein, functions as nickel donor for large subunit of hydrogenase 3 [b2727]
hypC	+3.62	12.68	+1.85	11.76	0.2	13.61	0.2	0.00026670	0.00256661	0.02400978	0.40213830	A_07_P002090	b2728	cmr|pleiotrophic effects on 3 hydrogenase isozymes [b2728]
hypD	+2.41	9.12	+1.27	8.49	0.1	9.75	0.5	0.01147914	0.04616284	0.05030043	0.55496146	A_07_P002091	b2729	cmr|pleiotrophic effects on 3 hydrogenase isozymes [b2729]
hypD	+2.64	9.84	+1.40	9.14	0.1	10.54	0.0	0.00043022	0.00361396	0.04107470	0.50561376	A_07_P043118	Z4038	cmr|pleiotrophic effects on 3 hydrogenase isozymes [Z4038]
hypE	+2.14	12.08	+1.10	11.53	0.0	12.63	0.0	0.00119125	0.00788028	0.06048590	0.61557207	A_07_P002097	b2730	cmr|plays structural role in maturation of all 3 hydrogenases [b2730]
hypE	+2.26	11.17	+1.18	10.58	0.0	11.76	0.1	0.00114671	0.00765966	0.05424479	0.57939620	A_07_P043125	Z4039	cmr|plays structural role in maturation of all [Z4039]
iaaA	+3.64	10.35	+1.86	9.42	0.1	11.29	0.0	0.00010023	0.00130845	0.02375508	0.40171858	A_07_P004591	b0828	cmr|putative asparaginase [b0828]
iap	-2.97	7.75	-1.57	8.54	0.1	6.96	0.4	0.00212631	0.01217773	0.03061865	0.49139653	A_07_P041682	c_3320	cmr|Alkaline phosphatase isozyme conversion protein precursor [c_3320]
ibpA	-2.89	9.17	-1.53	9.93	0.1	8.40	0.2	0.00051745	0.00415078	0.03290714	0.50057005	A_07_P017370	b3687	cmr|heat shock protein [b3687]
ibpB	-5.11	10.40	-2.35	11.57	0.1	9.22	0.2	0.00007051	0.00101790	0.00605437	0.24952880	A_07_P017361	b3686	cmr|heat shock protein [b3686]
icd	+5.50	10.45	+2.46	9.22	0.1	11.68	0.0	0.00002920	0.00059007	0.01207127	0.29193548	A_07_P006631	b1136	cmr|isocitrate dehydrogenase, specific for NADP+ [b1136]
icdA	+6.59	8.60	+2.72	7.24	0.0	9.96	0.0	0.00001092	0.00034947	0.00857660	0.25967308	A_07_P050489	Z1865	cmr|isocitrate dehydrogenase, specific for NADP+ [Z1865]
icdA	+51.01	14.35	+5.67	11.51	0.0	17.19	0.2	0.00000046	0.00009262	0.00011878	0.02968254	A_07_P038343	c_1517	cmr|Isocitrate dehydrogenase [c_1517]
iclR	+7.36	8.45	+2.88	7.01	0.1	9.89	0.0	0.00000951	0.00032326	0.00681148	0.23569231	A_07_P008735	b4018	cmr|repressor of aceBA operon [b4018]
iclR	+12.12	8.92	+3.60	7.12	0.1	10.72	0.0	0.00000260	0.00018134	0.00244220	0.14400749	A_07_P041378	c_4975	cmr|Acetate operon repressor [c_4975]
idnO	-3.51	11.57	-1.81	12.47	0.3	10.66	0.4	0.00309560	0.01632186	0.01924162	0.42016644	A_07_P003879	b4266	cmr|5-keto-D-gluconate 5-reductase [b4266]
ihfA	+2.28	12.05	+1.19	11.46	0.0	12.64	0.3	0.00498884	0.02366507	0.05430577	0.57965424	A_07_P018614	b1712	cmr|integration host factor [b1712]
ihfB	+2.41	9.41	+1.27	8.78	0.0	10.05	0.3	0.00235147	0.01313750	0.04959858	0.55303116	A_07_P005009	b0912	cmr|integration host factor [b0912]
ilvB	+2.88	10.78	+1.53	10.01	0.2	11.54	0.2	0.00105860	0.00719317	0.03540777	0.47646154	A_07_P017291	b3671	cmr|acetolactate synthase I,valine-sensitive, large subunit [b3671]
ilvD	+6.97	9.45	+2.80	8.04	0.0	10.85	0.1	0.00001083	0.00034789	0.00772104	0.24757637	A_07_P039071	c_4693	cmr|Dihydroxy-acid dehydratase [c_4693]
ilvD	+2.31	7.59	+1.21	6.99	0.0	8.20	0.2	0.00143640	0.00904225	0.05329649	0.57315574	A_07_P005736	b3771	cmr|dihydroxyacid dehydratase [b3771]
ilvM	+5.99	8.66	+2.58	7.37	0.0	9.95	0.0	0.00001395	0.00039090	0.01037729	0.27738540	A_07_P005727	b3769	cmr|acetolactate synthase II, valine insensitive, small subunit [b3769]
ilvN	+2.71	8.83	+1.44	8.11	0.0	9.54	0.0	0.00032422	0.00296307	0.03940549	0.49791332	A_07_P038986	c_4595	cmr|Acetolactate synthase isozyme I small subunit [c_4595]
imp	+2.78	10.65	+1.47	9.91	0.1	11.39	0.3	0.00224301	0.01268459	0.03812627	0.49161343	A_07_P010957	b0054	cmr|organic solvent tolerance [b0054]
inaA	-2.22	7.97	-1.15	8.55	0.0	7.40	0.2	0.00315374	0.01657844	0.06918636	0.65816918	A_07_P000971	b2237	cmr|pH-inducible protein involved in stress response [b2237]
infA	-2.15	10.79	-1.11	11.34	0.0	10.24	0.0	0.00113256	0.00759093	0.07505843	0.67257826	A_07_P004869	b0884	cmr|protein chain initiation factor IF-1 [b0884]
infB	-5.11	12.49	-2.35	13.66	0.0	11.31	0.1	0.00003132	0.00061413	0.00599276	0.24960317	A_07_P000561	b3168	cmr|protein chain initiation factor IF-2 [b3168]

infC	-5.89	14.35	-2.56	15.63	0.0	13.07	0.1	0.00001976	0.00046811	0.00388974	0.20481605	A_07_P018636	b1718	cmr|protein chain initiation factor IF-3 [b1718]
insA	-3.68	9.03	-1.88	9.97	0.1	8.09	0.3	0.00048655	0.00394653	0.01578760	0.39205047	A_07_P008028	b0265	cmr|IS1 protein InsA [b0265]
insA	-3.70	10.23	-1.89	11.17	0.0	9.28	0.3	0.00047702	0.00389132	0.01550940	0.39131410	A_07_P003998	b4294	cmr|IS1 protein InsA [b4294]
insB	-3.71	9.25	-1.89	10.19	0.3	8.30	0.4	0.00262984	0.01434655	0.01504764	0.38647635	A_07_P005381	b0988	cmr|IS1 protein InsB [b0988]
insB	-3.65	9.51	-1.87	10.45	0.0	8.58	0.3	0.00050643	0.00408045	0.01621761	0.39463679	A_07_P010675	b0021	cmr|IS1 protein InsB [b0021]
insC	-2.19	9.57	-1.13	10.14	0.1	9.01	0.3	0.00730235	0.03222202	0.06888021	0.65804005	A_07_P015315	b1997	cmr|IS2 hypothetical protein [b1997]
insD	-3.29	12.15	-1.72	13.01	0.2	11.30	0.2	0.00045353	0.00376202	0.02271786	0.45046599	A_07_P020927	b0361	cmr|IS2 hypothetical protein [b0361]
insG	-24.20	10.64	-4.60	12.94	0.0	8.34	0.1	0.00000081	0.00012560	0.00003366	0.01766667	A_07_P003924	b4278	cmr|IS4 hypothetical protein [b4278]
insI	+2.00	9.00	+1.00	8.51	0.1	9.50	0.0	0.00322340	0.01688264	0.07002350	0.66133773	A_07_P007989	b0256	cmr|IS30 transposase [b0256]
intO	-2.41	7.77	-1.27	8.41	0.2	7.14	0.1	0.00145677	0.00913904	0.05660950	0.61678893	A_07_P050808	Z2036	cmr|putative integrase for prophage CP-933O [Z2036]
intU	-3.07	10.40	-1.62	11.21	0.1	9.59	0.1	0.00040686	0.00346810	0.02806974	0.48245633	A_07_P044913	Z3130	cmr|putative integrase for prophage CP-933U [Z3130]
intW	-2.20	9.87	-1.14	10.44	0.0	9.30	0.0	0.00101851	0.00700236	0.07129764	0.66460036	A_07_P051282	Z1424	cmr|integrase for bacteriophage BP-933W [Z1424]
iraP	+5.69	11.57	+2.51	10.31	0.0	12.82	0.0	0.00001703	0.00042859	0.01147675	0.28590190	A_07_P021023	b0382	cmr|orf, hypothetical protein [b0382]
iroD	+2.17	9.35	+1.12	8.79	0.0	9.91	0.2	0.00236559	0.01319298	0.05991235	0.61250649	A_07_P040551	c_1252	cmr|Ferric enterochelin esterase [c_1252]
iroN	-3.18	9.25	-1.67	10.09	0.1	8.42	0.3	0.00076215	0.00558628	0.02435849	0.45983213	A_07_P040541	c_1250	cmr|Siderophore receptor IroN [c_1250]
iscA	-11.47	10.77	-3.52	12.53	0.1	9.01	0.3	0.00001571	0.00041046	0.00045605	0.06710280	A_07_P019544	b2528	cmr|putative regulator [b2528]
iscR	-7.71	10.08	-2.95	11.55	0.1	8.60	0.1	0.00000930	0.00032222	0.00170541	0.13492462	A_07_P019560	b2531	cmr|orf, hypothetical protein [b2531]
iscS	-5.30	11.53	-2.41	12.74	0.1	10.33	0.2	0.00005829	0.00089192	0.00538300	0.23940678	A_07_P019553	b2530	cmr|putative aminotransferase [b2530]
iscU	-4.89	11.64	-2.29	12.78	0.0	10.49	0.1	0.00003073	0.00060787	0.00688580	0.26967662	A_07_P019547	b2529	cmr|orf, hypothetical protein [b2529]
ispU	-2.04	11.41	-1.03	11.93	0.0	10.90	0.1	0.00197928	0.01153712	0.08527376	0.71185048	A_07_P002826	b0174	cmr|orf, hypothetical protein [b0174]
iucA	+2.63	8.20	+1.40	7.50	0.0	8.90	0.2	0.00064244	0.00489810	0.04135925	0.50713396	A_07_P036321	c_3627	cmr|IucA protein [c_3627]
iucD	+2.90	10.18	+1.54	9.41	0.1	10.95	0.3	0.00214008	0.01222990	0.03549733	0.47644501	A_07_P036310	c_3624	cmr|IucD protein [c_3624]
iutA	-2.58	8.25	-1.37	8.93	0.0	7.56	0.2	0.00085411	0.00611791	0.04574949	0.57210485	A_07_P036302	c_3623	cmr|IutA protein [c_3623]
katG	+12.32	11.12	+3.62	9.31	0.1	12.93	0.1	0.00000387	0.00021100	0.00235264	0.14412451	A_07_P019488	b3942	cmr|catalase; hydroperoxidase HPI [b3942]
kbaY	+2.01	9.17	+1.00	8.67	0.1	9.67	0.1	0.00244029	0.01350911	0.06955539	0.65889290	A_07_P000407	b3137	cmr|tagatose-bisphosphate aldolase 2 [b3137]
kbaZ	+4.78	8.14	+2.26	7.01	0.1	9.27	0.0	0.00003950	0.00068718	0.01519563	0.33089903	A_07_P000381	b3132	cmr|putative tagatose 6-phosphate kinase 2 [b3132]
kbl	+22.70	11.33	+4.50	9.07	0.1	13.58	0.5	0.00002333	0.00052262	0.00060658	0.07519685	A_07_P017030	b3617	cmr|2-amino-3-ketobutyrate CoA ligase [b3617]
kch	+3.10	8.82	+1.63	8.01	0.1	9.64	0.0	0.00027051	0.00258426	0.03130843	0.45139194	A_07_P007187	b1250	cmr|putative potassium channel protein [b1250]
kdgR	-2.43	9.66	-1.28	10.30	0.1	9.02	0.2	0.00217852	0.01239115	0.05337525	0.60369253	A_07_P015816	b1827	cmr|putative regulator [b1827]
kdpA	-2.73	8.67	-1.45	9.40	0.1	7.95	0.3	0.00184146	0.01095687	0.03950203	0.54127067	A_07_P010006	b0698	cmr|ATPase of high-affinity potassium transport system, A chain [b0698]
kdpE	+3.56	10.13	+1.83	9.22	0.2	11.05	0.3	0.00060028	0.00465408	0.02469766	0.40844538	A_07_P033368	c_0779	cmr|KDP operon transcriptional Regulatory protein kdpE [c_0779]
kdpE	+3.04	8.78	+1.60	7.98	0.2	9.58	0.0	0.00039977	0.00341321	0.03225927	0.45797115	A_07_P009988	b0694	cmr|regulator of kdp operon [b0694]
kdsB	-2.12	9.48	-1.08	10.02	0.2	8.94	0.0	0.00352364	0.01806454	0.07585112	0.67468927	A_07_P042155	c_1059	cmr|3-deoxy-manno-octulosonate cytidylyltransferase [c_1059]
kdsC	-2.50	12.91	-1.32	13.57	0.0	12.25	0.0	0.00049625	0.00401279	0.05066311	0.59480984	A_07_P012468	b3198	cmr|orf, hypothetical protein [b3198]
kefB	-2.64	8.47	-1.40	9.17	0.0	7.77	0.1	0.00038444	0.00332667	0.04279408	0.56239566	A_07_P017779	b3350	cmr|K+ efflux; NEM-activable K+/H+ antiporter [b3350]
kefG	-2.99	11.08	-1.58	11.87	0.1	10.29	0.0	0.00022682	0.00231589	0.03017213	0.49073347	A_07_P017784	b3351	cmr|putative NAD [b3351]
kpsC	+8.23	8.44	+3.04	6.92	0.0	9.96	0.2	0.00001547	0.00040806	0.00544970	0.21612091	A_07_P036625	c_3690	cmr|KpsC protein [c_3690]
kpsD	+5.71	11.58	+2.51	10.32	0.2	12.83	0.2	0.00010950	0.00138923	0.01097243	0.28043831	A_07_P036611	c_3688	cmr|KpsD protein [c_3688]
kpsE	+6.95	9.86	+2.80	8.46	0.1	11.26	0.2	0.00002352	0.00052457	0.00778963	0.24876268	A_07_P036606	c_3687	cmr|KpsE protein [c_3687]
ksgA	-2.96	11.70	-1.57	12.48	0.0	10.91	0.3	0.00108982	0.00736719	0.03091019	0.49206269	A_07_P010944	b0051	cmr|S-adenosylmethionine-6-N,N-adenosyl [b0051]
L7002	-3.69	12.51	-1.88	13.45	0.4	11.57	0.0	0.00128397	0.00835326	0.01559959	0.39170654	A_07_P048661	ABH-0243999	asap|unknown [L7002]
L7013	-3.75	8.78	-1.91	9.73	0.4	7.83	0.1	0.00080224	0.00580979	0.01501778	0.38760656	A_07_P048711	ABH-0244027	asap|transposase (partial) [L7013]
L7045	-5.54	9.75	-2.47	10.98	0.1	8.51	0.2	0.00007808	0.00109698	0.00474530	0.22368263	A_07_P051850	ABH-0244079	asap|hypothetical protein [L7045]
L7055	-2.76	10.99	-1.47	11.73	0.1	10.26	0.0	0.00030849	0.00285697	0.03793318	0.53039076	A_07_P048893	ABH-0244096	asap|replication protein [L7055]
L7073	-2.16	10.23	-1.11	10.78	0.1	9.67	0.1	0.00191636	0.01126632	0.07390689	0.67220682	A_07_P048969	ABH-0244142	asap|unknown [L7073]
L7079	-3.10	11.33	-1.63	12.15	0.0	10.52	0.0	0.00059778	0.00464073	0.02736979	0.47878889	A_07_P048996	ABH-0244148	asap|unknown [L7079]
L7082	-5.41	9.25	-2.44	10.46	0.3	8.03	0.3	0.00034580	0.00309337	0.00490917	0.22533528	A_07_P049011	ABH-0244151	asap|unknown [L7082]
lacA	+2.85	8.40	+1.51	7.65	0.1	9.16	0.2	0.00068098	0.00513060	0.03621824	0.48079258	A_07_P008405	b0342	cmr|thiogalactoside acetyltransferase [b0342]
lacA	+2.41	8.52	+1.27	7.88	0.1	9.15	0.2	0.00215891	0.01229844	0.04979167	0.55361582	A_07_P053944	ECs0395	cmr|thiogalactoside acetyltransferase [ECs0395]
lacI	+2.64	8.35	+1.40	7.65	0.1	9.05	0.0	0.00037134	0.00324276	0.04138783	0.50669518	A_07_P008419	b0345	cmr|transcriptional repressor of the lac operon [b0345]
lacY	+3.94	9.35	+1.98	8.36	0.5	10.34	0.2	0.00205680	0.01188584	0.02104802	0.38354167	A_07_P008407	b0343	cmr|galactoside permease [b0343]
lacZ	+23.17	9.32	+4.53	7.05	0.2	11.59	0.1	0.00000250	0.00018108	0.00056720	0.07201613	A_07_P031027	c_0459	cmr|Beta-galactosidase [c_0459]
lacZ	+10.25	8.51	+3.36	6.83	0.3	10.19	0.2	0.00003658	0.00065814	0.00348006	0.17393651	A_07_P008412	b0344	cmr|beta-D-galactosidase [b0344]
lamB	+350.33	12.05	+8.45	7.82	0.1	16.28	0.0	0.00000003	0.00007032	0.00000000	0.00000000	A_07_P008822	b4036	cmr|phage lambda receptor protein; maltose high-affinity receptor [b4036]
lamB	+358.11	11.57	+8.48	7.33	0.0	15.81	0.1	0.00000003	0.00007032	0.00000000	0.00000000	A_07_P037538	c_5006	cmr|Maltoporin precursor [c_5006]
lar	-2.78	9.16	-1.48	9.90	0.1	8.42	0.3	0.00130634	0.00846030	0.03749047	0.52700893	A_07_P014717	b1348	cmr|restriction alleviation and modification enhancement [b1348]
lasT	-2.63	9.25	-1.40	9.95	0.1	8.56	0.3	0.00252568	0.01389390	0.04244538	0.56109152	A_07_P052396	Z6006	cmr|orf, hypothetical protein [Z6006]
lasT	-2.36	11.14	-1.24	11.76	0.2	10.52	0.4	0.00921191	0.03878908	0.05886242	0.62532389	A_07_P037395	c_5490	cmr|Hypothetical tRNA/rRNA methyltransferase lasT [c_5490]
ldhA	+2.19	8.90	+1.13	8.34	0.2	9.47	0.2	0.00508959	0.02408491	0.06000000	0.61300454	A_07_P014878	b1380	cmr|fermentative D-lactate dehydrogenase, NAD-dependent [b1380]
leuB	+3.32	9.67	+1.73	8.81	0.0	10.54	0.1	0.00015630	0.00177268	0.02727769	0.42394867	A_07_P035745	c_0090	cmr|3-isopropylmalate dehydrogenase [c_0090]
leuE	-9.23	8.97	-3.21	10.57	0.1	7.37	0.2	0.00001994	0.00047056	0.00094703	0.10006711	A_07_P015685	b1798	cmr|orf, hypothetical protein [b1798]
lhr	-3.14	9.18	-1.65	10.01	0.0	8.36	0.3	0.00075887	0.00557260	0.02551321	0.46652729	A_07_P018330	b1653	cmr|member of ATP-dependent helicase superfamily II [b1653]
ligB	-2.88	8.07	-1.53	8.83	0.0	7.30	0.3	0.00192425	0.01129162	0.03201029	0.49554572	A_07_P017180	b3647	cmr|putative enzyme [b3647]
ligT	-2.38	10.57	-1.25	11.20	0.1	9.95	0.0	0.00082349	0.00592821	0.05732406	0.61943034	A_07_P002698	b0147	cmr|orf, hypothetical protein [b0147]
lipA	+2.29	12.86	+1.20	12.26	0.0	13.46	0.1	0.00104558	0.00714172	0.05306466	0.57301097	A_07_P009709	b0628	cmr|lipoate synthesis, sulfur insertion? [b0628]
lipB	-25.34	11.59	-4.66	13.93	0.0	9.26	0.5	0.00002324	0.00052262	0.00002033	0.01230769	A_07_P009718	b0630	cmr|protein of lipoate biosynthesis [b0630]
livK	-3.83	8.20	-1.94	9.17	0.1	7.23	0.1	0.00008052	0.00111496	0.01396087	0.37508532	A_07_P034548	c_4248	cmr|Leucine-specific binding protein precursor [c_4248]
lldD	+6.83	8.94	+2.77	7.56	0.5	10.33	0.2	0.00037473	0.00325950	0.00769817	0.24836066	A_07_P016968	b3605	cmr|L-lactate dehydrogenase [b3605]
lldP	+3.86	7.50	+1.95	6.52	0.1	8.47	0.0	0.00009677	0.00127819	0.02138148	0.38560137	A_07_P016959	b3603	cmr|L-lactate permease [b3603]
lldR	+7.60	8.81	+2.93	7.35	0.1	10.27	0.2	0.00002756	0.00056786	0.00627096	0.22801386	A_07_P016965	b3604	cmr|transcriptional regulator [b3604]
lolA	+4.41	9.87	+2.14	8.80	0.0	10.94	0.0	0.00004105	0.00070782	0.01727261	0.35134367	A_07_P004904	b0891	cmr|periplasmic protein effects translocation of lipoproteins from inner membrane to outer [b0891]
lon	+2.00	10.71	+1.00	10.21	0.1	11.21	0.1	0.00251691	0.01385532	0.07030869	0.66085970	A_07_P031164	c_0555	cmr|ATP-dependent protease La [c_0555]
lpcA	+6.39	10.88	+2.68	9.54	0.0	12.22	0.0	0.00001179	0.00035886	0.00909299	0.26316176	A_07_P007824	b0222	cmr|phosphoheptose isomerase [b0222]
lpp	+2.15	13.03	+1.10	12.47	0.1	13.58	0.0	0.00162713	0.00996789	0.05969068	0.61182943	A_07_P018436	b1677	cmr|murein lipoprotein [b1677]
lpxA	-4.33	11.30	-2.12	12.36	0.0	10.25	0.0	0.00004319	0.00073315	0.00983613	0.31348178	A_07_P002865	b0181	cmr|UDP-N-acetylglucosamine acetyltransferase; lipid A biosynthesis [b0181]
lpxB	-2.04	10.71	-1.03	11.22	0.1	10.19	0.0	0.00194770	0.01140373	0.08537602	0.71119577	A_07_P002870	b0182	cmr|tetraacyldisaccharide-1-P; lipid A biosynthesis, penultimate step [b0182]
lpxC	-6.57	14.40	-2.72	15.76	0.0	13.04	0.1	0.00001517	0.00040409	0.00277121	0.17592742	A_07_P002451	b0096	cmr|UDP-3-O-acyl N-acetylglucosamine deacetylase; lipid A biosynthesis [b0096]
lpxK	-6.49	9.81	-2.70	11.16	0.0	8.46	0.2	0.00003266	0.00062946	0.00279726	0.17407115	A_07_P005022	b0915	cmr|putative EC 1.2 enzyme [b0915]
lpxL	-4.88	11.47	-2.29	12.62	0.0	10.33	0.1	0.00004671	0.00076769	0.00694360	0.26926108	A_07_P001480	b1054	cmr|heat shock protein [b1054]
lpxP	-3.25	11.29	-1.70	12.14	0.0	10.44	0.1	0.00014448	0.00167009	0.02321265	0.45118519	A_07_P014174	b2378	cmr|putative heat shock protein [b2378]
lrhA	-5.43	11.68	-2.44	12.90	0.0	10.46	0.3	0.00009502	0.00126142	0.00490917	0.22599415	A_07_P013734	b2289	cmr|NADH dehydrogenase transcriptional regulator, LysR family [b2289]
lrp	+3.49	9.80	+1.80	8.90	0.1	10.71	0.1	0.00017103	0.00190160	0.02555005	0.41257436	A_07_P004893	b0889	cmr|regulator for leucine [b0889]
ltaE	+5.66	9.51	+2.50	8.26	0.0	10.77	0.2	0.00003345	0.00063226	0.01123920	0.28266773	A_07_P004805	b0870	cmr|putative arylsulfatase [b0870]
lysS	-6.21	11.06	-2.63	12.38	0.1	9.74	0.2	0.00002566	0.00055176	0.00324060	0.18757353	A_07_P006416	b2890	cmr|lysine tRNA synthetase, constitutive; suppressor of ColE1 mutation in primer RNA [b2890]
lysU	+7.10	10.83	+2.83	9.41	0.2	12.24	0.4	0.00018011	0.00195827	0.00723831	0.24144068	A_07_P011381	b4129	cmr|lysine tRNA synthetase, inducible; heat shock protein [b4129]
lyx	+2.42	7.16	+1.27	6.52	0.0	7.80	0.1	0.00065857	0.00499931	0.04792111	0.54513728	A_07_P016844	b3580	cmr|L-xylulose kinase, cryptic [b3580]
lyxK	+8.35	8.41	+3.06	6.87	0.1	9.94	0.1	0.00000710	0.00028183	0.00520770	0.21296104	A_07_P039671	c_4403	cmr|Cryptic L-xylulose kinase [c_4403]
maa	-9.02	9.42	-3.17	11.01	0.1	7.84	0.2	0.00001106	0.00034947	0.00100483	0.10407895	A_07_P021405	b0459	cmr|putative transferase [b0459]
macB	-2.05	8.69	-1.04	9.21	0.0	8.17	0.0	0.00162229	0.00995666	0.08492759	0.70971338	A_07_P004848	b0879	cmr|putative ATP-binding component of a transport system [b0879]
maeA	+6.48	9.22	+2.70	7.87	0.1	10.57	0.1	0.00002086	0.00048725	0.00879764	0.26035714	A_07_P009486	b1479	cmr|NAD-linked malate dehydrogenase [b1479]
maeB	+4.93	11.17	+2.30	10.02	0.2	12.32	0.2	0.00013744	0.00161341	0.01431529	0.32197143	A_07_P013371	b2463	cmr|putative multimodular enzyme [b2463]
malE	+72.51	11.94	+6.18	8.85	0.1	15.03	0.2	0.00000047	0.00009262	0.00006161	0.02108696	A_07_P008811	b4034	cmr|periplasmic maltose-binding protein; substrate recognition for transport and chemotaxis [b4034]
malF	+8.58	8.85	+3.10	7.30	0.0	10.40	0.3	0.00002820	0.00057601	0.00500064	0.21221024	A_07_P008806	b4033	cmr|part of maltose permease, periplasmic [b4033]
malG	+2.80	10.06	+1.48	9.32	0.0	10.81	0.0	0.00028054	0.00265935	0.03735899	0.48650124	A_07_P008801	b4032	cmr|part of maltose permease, inner membrane [b4032]
malK	+10.76	8.31	+3.43	6.59	0.0	10.02	0.1	0.00000353	0.00020330	0.00307546	0.16413559	A_07_P008816	b4035	cmr|ATP-binding component of transport system for maltose [b4035]
malM	+5.17	8.91	+2.37	7.72	0.1	10.09	0.2	0.00007951	0.00110776	0.01315739	0.30418502	A_07_P008827	b4037	cmr|periplasmic protein of mal regulon [b4037]
malP	+25.76	10.19	+4.69	7.85	0.1	12.53	0.1	0.00000121	0.00014031	0.00045414	0.06441441	A_07_P018111	b3417	cmr|maltodextrin phosphorylase [b3417]
malQ	+6.80	9.63	+2.77	8.25	0.1	11.01	0.4	0.00014915	0.00170726	0.00779154	0.24831984	A_07_P018108	b3416	cmr|4-alpha-glucanotransferase [b3416]
malT	+18.92	9.37	+4.24	7.25	0.0	11.49	0.0	0.00000101	0.00012946	0.00095020	0.08904762	A_07_P034438	c_4196	cmr|MalT Regulatory protein [c_4196]
malT	+10.52	8.87	+3.40	7.17	0.0	10.56	0.1	0.00000392	0.00021229	0.00322472	0.16645902	A_07_P018118	b3418	cmr|positive regulator of mal regulon [b3418]
malT	+11.43	9.20	+3.52	7.44	0.1	10.96	0.3	0.00002719	0.00056519	0.00275279	0.15314488	A_07_P052157	Z4774	cmr|positive regulator of mal regulon [Z4774]
malX	+3.68	9.75	+1.88	8.81	0.0	10.69	0.1	0.00011311	0.00141223	0.02322154	0.39869138	A_07_P020740	b1621	cmr|PTS system, maltose and glucose-specific II ABC [b1621]
malY	+2.60	9.17	+1.38	8.48	0.1	9.86	0.0	0.00062205	0.00475805	0.04179116	0.50846986	A_07_P042322	c_2014	cmr|MalY protein [c_2014]
manA	+2.59	8.89	+1.37	8.20	0.1	9.57	0.1	0.00074895	0.00551637	0.04274771	0.51180228	A_07_P020700	b1613	cmr|mannose-6-phosphate isomerase [b1613]
manA	+2.99	8.81	+1.58	8.02	0.1	9.60	0.1	0.00028283	0.00267294	0.03343178	0.46703638	A_07_P044397	Z2616	cmr|mannose-6-phosphate isomerase [Z2616]
manA	+2.61	8.81	+1.39	8.12	0.0	9.51	0.1	0.00042144	0.00355887	0.04204205	0.51033924	A_07_P035096	c_2005	cmr|Mannose-6-phosphate isomerase [c_2005]
manB	+2.88	12.70	+1.53	11.94	0.1	13.46	0.0	0.00035271	0.00313486	0.03520389	0.47615979	A_07_P031986	c_2557	cmr|Phosphomannomutase [c_2557]
manX	+19.40	10.43	+4.28	8.29	0.0	12.57	0.1	0.00000153	0.00015822	0.00086827	0.08651899	A_07_P015778	b1817	cmr|PTS enzyme IIAB, mannose-specific [b1817]
manY	+6.28	9.81	+2.65	8.48	0.1	11.13	0.1	0.00001424	0.00039271	0.00948361	0.26758065	A_07_P015784	b1818	cmr|PTS enzyme IIC, mannose-specific [b1818]
manZ	+5.89	9.84	+2.56	8.56	0.2	11.11	0.1	0.00003639	0.00065813	0.01054179	0.27707846	A_07_P015786	b1819	cmr|PTS enzyme IID, mannose-specific [b1819]
map	-2.16	8.96	-1.11	9.52	0.1	8.41	0.1	0.00210530	0.01208818	0.07290841	0.66775451	A_07_P002799	b0168	cmr|methionine aminopeptidase [b0168]
mchB	+2.37	9.03	+1.25	8.41	0.4	9.65	0.2	0.01162025	0.04663502	0.05273056	0.57096974	A_07_P040432	c_1227	cmr|MchB protein [c_1227]
mchD	-2.77	11.56	-1.47	12.30	0.4	10.83	0.1	0.00266784	0.01449358	0.03719766	0.52618149	A_07_P040445	c_1230	cmr|MchD protein [c_1230]
mchF	-2.37	10.53	-1.24	11.16	0.1	9.91	0.3	0.00533479	0.02504949	0.05836255	0.62211239	A_07_P040453	c_1232	cmr|Probable microcin H47 secretion ATP-binding protein [c_1232]
mdaB	+4.33	9.61	+2.11	8.55	0.2	10.66	0.1	0.00012614	0.00152533	0.01787919	0.35858599	A_07_P012273	b3028	cmr|modulator of drug activity B [b3028]
mdh	+19.83	12.39	+4.31	10.24	0.0	14.55	0.1	0.00000101	0.00012946	0.00079649	0.08472973	A_07_P012651	b3236	cmr|malate dehydrogenase [b3236]
mdlA	-2.17	7.92	-1.11	8.48	0.0	7.36	0.2	0.00265700	0.01445469	0.07236725	0.66824047	A_07_P021355	b0448	cmr|ATP-binding component of a transport system [b0448]
mdoD	+2.52	7.77	+1.33	7.10	0.2	8.43	0.0	0.00080682	0.00582985	0.04502985	0.53025430	A_07_P009213	b1424	cmr|putative glycoprotein [b1424]
mdtH	-3.03	9.46	-1.60	10.26	0.3	8.66	0.0	0.00092069	0.00648270	0.02869982	0.48481760	A_07_P001526	b1065	cmr|orf, hypothetical protein [b1065]
mdtI	+2.27	7.77	+1.18	7.18	0.1	8.36	0.2	0.00193889	0.01135633	0.05554116	0.58647887	A_07_P020626	b1599	cmr|possible chaperone [b1599]
mdtK	-4.42	10.94	-2.14	12.01	0.0	9.86	0.4	0.00053243	0.00424558	0.00958587	0.30989733	A_07_P018376	b1663	cmr|putative transport protein [b1663]
mdtL	-2.18	8.00	-1.12	8.56	0.3	7.44	0.1	0.00756993	0.03318881	0.07243775	0.66810779	A_07_P005475	b3710	cmr|putative transport protein [b3710]
melA	+4.77	9.20	+2.25	8.08	0.1	10.33	0.3	0.00026905	0.00257973	0.01487043	0.32882022	A_07_P011333	b4119	cmr|alpha-galactosidase [b4119]
menB	+8.51	9.98	+3.09	8.43	0.1	11.52	0.1	0.00000913	0.00032069	0.00500635	0.21188172	A_07_P001100	b2262	cmr|dihydroxynaphtoic acid synthetase [b2262]
menD	+2.59	8.72	+1.37	8.03	0.1	9.41	0.1	0.00077300	0.00564741	0.04262005	0.51183066	A_07_P001109	b2264	cmr|2-oxoglutarate decarboxylase; SHCHC synthase [b2264]
menD	+2.15	8.92	+1.11	8.37	0.2	9.48	0.2	0.00375176	0.01899281	0.06014482	0.61368762	A_07_P032389	c_2808	cmr|Menaquinone biosynthesis protein menD [c_2808]
metB	+2.58	8.90	+1.37	8.22	0.2	9.59	0.5	0.01208721	0.04818968	0.04609566	0.53797628	A_07_P019474	b3939	cmr|cystathionine gamma-synthase [b3939]
metH	+7.30	8.89	+2.87	7.46	0.3	10.33	0.0	0.00004711	0.00077097	0.00686293	0.23540305	A_07_P057969	ECs4937	cmr|B12-dependent homocysteine-N5-methyltetrahydrofolate transmethylase [ECs4937]
metH	+8.62	9.24	+3.11	7.69	0.0	10.80	0.1	0.00000714	0.00028183	0.00490790	0.20997283	A_07_P047219	Z5610	cmr|B12-dependent homocysteine-N5-methyltetrahydrofolate transmethylase, repressor of metE and [Z5610]
metH	+4.20	8.24	+2.07	7.20	0.1	9.27	0.2	0.00017912	0.00195567	0.01904345	0.36969174	A_07_P008740	b4019	cmr|B12-dependent homocysteine-N5-methyltetrahydrofolate transmethylase, repressor of metE and metF [b4019]
metH	+5.49	9.67	+2.46	8.44	0.1	10.90	0.1	0.00003034	0.00060429	0.01207127	0.29238462	A_07_P037467	c_4976	cmr|5-methyltetrahydrofolate--homocysteine methyltransferase [c_4976]
metJ	-2.21	9.92	-1.14	10.50	0.0	9.35	0.1	0.00134745	0.00865120	0.06982279	0.66102826	A_07_P035559	c_4891	cmr|Met repressor [c_4891]
metN	-7.15	10.24	-2.84	11.66	0.1	8.83	0.1	0.00001215	0.00036440	0.00219639	0.15647059	A_07_P002955	b0199	cmr|ATP-binding component of a transporter [b0199]
metR	-2.68	9.82	-1.42	10.53	0.3	9.11	0.1	0.00267088	0.01450510	0.04033092	0.54503863	A_07_P039160	c_4750	cmr|Transcriptional activator protein metR [c_4750]
mfd	+2.14	7.25	+1.09	6.71	0.1	7.80	0.1	0.00214956	0.01226631	0.06192200	0.62373640	A_07_P001771	b1114	cmr|transcription-repair coupling factor; mutation frequency decline [b1114]
mfd	+3.71	8.10	+1.89	7.16	0.1	9.05	0.3	0.00074911	0.00551637	0.02316184	0.39984649	A_07_P040806	c_1389	cmr|Transcription-repair coupling factor [c_1389]
mglA	+28.96	9.60	+4.86	7.17	0.1	12.03	0.5	0.00001419	0.00039205	0.00035887	0.05650000	A_07_P003507	b2149	cmr|ATP-binding component of methyl-galactoside transport and galactose taxis [b2149]
mglB	+16.75	9.36	+4.07	7.32	0.3	11.39	0.7	0.00026381	0.00255503	0.00124619	0.10436170	A_07_P003515	b2150	cmr|galactose-binding transport protein; receptor for galactose taxis [b2150]
mglB	+95.68	11.48	+6.58	8.19	0.0	14.77	0.0	0.00000010	0.00007032	0.00003239	0.01500000	A_07_P032239	c_2684	cmr|D-galactose-binding periplasmic protein precursor [c_2684]
mglB	+115.52	11.65	+6.85	8.22	0.1	15.08	0.2	0.00000020	0.00008010	0.00001524	0.01000000	A_07_P045485	Z3405	cmr|galactose-binding transport protein receptor for galactose taxis [Z3405]
mglC	+19.90	10.02	+4.31	7.86	0.0	12.18	0.1	0.00000104	0.00012946	0.00078697	0.08486301	A_07_P003504	b2148	cmr|methyl-galactoside transport and galactose taxis [b2148]
mgsA	+6.07	10.42	+2.60	9.12	0.2	11.72	0.0	0.00003651	0.00065813	0.00992505	0.27128472	A_07_P005264	b0963	cmr|methylglyoxal synthase [b0963]
mgtA	-115.69	10.95	-6.85	14.38	0.0	7.53	0.1	0.00000010	0.00007032	0.00000000	0.00000000	A_07_P003761	b4242	cmr|Mg2+ transport ATPase, P-type 1 [b4242]
miaA	+8.00	13.76	+3.00	12.26	0.0	15.26	0.0	0.00000649	0.00027019	0.00581364	0.22270073	A_07_P011571	b4171	cmr|delta [b4171]
miaB	-4.48	11.51	-2.16	12.59	0.1	10.43	0.1	0.00006873	0.00099883	0.00885925	0.30255965	A_07_P009866	b0661	cmr|orf, hypothetical protein [b0661]
minC	+4.71	11.12	+2.24	10.00	0.1	12.24	0.0	0.00003859	0.00068020	0.01552210	0.33430917	A_07_P006831	b1176	cmr|cell division inhibitor, inhibits ftsZ ring formation [b1176]
minD	+3.72	10.60	+1.89	9.65	0.2	11.54	0.2	0.00046283	0.00382050	0.02308816	0.39945055	A_07_P006827	b1175	cmr|cell division inhibitor, a membrane ATPase, activates minC [b1175]
minE	+11.57	12.86	+3.53	11.09	0.1	14.63	0.1	0.00000509	0.00024285	0.00267912	0.15064286	A_07_P006821	b1174	cmr|cell division topological specificity factor, reverses MinC inhibition of ftsZ ring formation [b1174]
mioC	-10.99	13.34	-3.46	15.07	0.0	11.61	0.1	0.00000410	0.00021832	0.00058117	0.07887931	A_07_P005634	b3742	cmr|initiation of chromosome replication [b3742]
mipA	+2.48	11.39	+1.31	10.73	0.1	12.04	0.1	0.00081058	0.00584327	0.04599022	0.53754269	A_07_P015602	b1782	cmr|orf, hypothetical protein [b1782]
mltB	-3.20	10.73	-1.68	11.57	0.1	9.89	0.6	0.00588536	0.02706167	0.02550877	0.46698837	A_07_P001954	b2701	cmr|membrane-bound lytic murein transglycosylase B [b2701]
mltD	+2.48	10.48	+1.31	9.82	0.2	11.13	0.3	0.00318579	0.01671343	0.04628239	0.53736726	A_07_P002982	b0211	cmr|transcriptional regulator for nitrite reductase [b0211]
mnmA	-4.95	11.46	-2.31	12.61	0.0	10.30	0.0	0.00002693	0.00056453	0.00665333	0.26385390	A_07_P006616	b1133	cmr|orf, hypothetical protein [b1133]
mnmE	-2.45	8.58	-1.29	9.22	0.1	7.93	0.3	0.00237585	0.01323150	0.05189977	0.60081618	A_07_P005456	b3706	cmr|GTP-binding protein in thiophene and furan oxidation [b3706]
mnmG	-5.24	10.14	-2.39	11.34	0.1	8.95	0.0	0.00003334	0.00063173	0.00565104	0.24509642	A_07_P005627	b3741	cmr|glucose-inhibited division; chromosome replication? [b3741]
mntH	-10.16	9.61	-3.35	11.28	0.2	7.93	0.1	0.00001106	0.00034947	0.00067454	0.08296875	A_07_P014241	b2392	cmr|putative transport system permease [b2392]
mntR	-2.16	11.82	-1.11	12.38	0.2	11.26	0.2	0.00417284	0.02059106	0.07416921	0.67148936	A_07_P004536	b0817	cmr|putative toxin [b0817]
moaB	+3.68	10.53	+1.88	9.59	0.1	11.47	0.2	0.00016811	0.00187842	0.02295414	0.39844542	A_07_P004361	b0782	cmr|molybdopterin biosynthesis, protein B [b0782]
moaC	+2.74	11.41	+1.46	10.69	0.3	12.14	0.3	0.00399032	0.01992503	0.03999492	0.49974603	A_07_P004368	b0783	cmr|molybdopterin biosynthesis, protein C [b0783]
MoaE	+2.41	11.00	+1.27	10.36	0.0	11.63	0.0	0.00059243	0.00460831	0.04773056	0.54454348	A_07_P041580	c_0867	cmr|Molybdopterin converting factor subunit 2 [c_0867]
mobA	+3.81	8.27	+1.93	7.30	0.4	9.23	0.0	0.00099746	0.00689682	0.02228913	0.39296753	A_07_P039291	c_4801	cmr|Molybdopterin-guanine dinucleotide biosynthesis protein A [c_4801]
modA	+2.00	9.18	+1.00	8.68	0.1	9.68	0.0	0.00210852	0.01209786	0.06965892	0.65908053	A_07_P041574	c_0840	cmr|Molybdate-binding periplasmic protein precursor [c_0840]
moeA	-2.59	11.21	-1.37	11.90	0.0	10.52	0.0	0.00040999	0.00348910	0.04560658	0.57213546	A_07_P004586	b0827	cmr|molybdopterin biosynthesis [b0827]
moeA	-2.08	9.40	-1.06	9.92	0.0	8.87	0.2	0.00421246	0.02072545	0.08064469	0.69342982	A_07_P051724	Z1050	cmr|molybdopterin biosynthesis [Z1050]
moeB	-2.74	8.96	-1.45	9.69	0.1	8.24	0.1	0.00050398	0.00406282	0.03888084	0.53555556	A_07_P004583	b0826	cmr|molybdopterin biosynthesis [b0826]
mokC	-2.96	12.49	-1.56	13.27	0.1	11.71	0.1	0.00037098	0.00324276	0.03047510	0.49059305	A_07_P010256	b0018	cmr|Gef protein interferes with membrane function when in excess [b0018]
mokC	-3.30	10.69	-1.72	11.55	0.1	9.83	0.0	0.00012964	0.00155330	0.02238694	0.44899363	A_07_P010779	b0018	cmr|Gef protein interferes with membrane function when in excess [b0018]
motB	-3.69	8.26	-1.88	9.20	0.0	7.32	0.1	0.00008884	0.00120061	0.01550686	0.39187801	A_07_P016121	b1889	cmr|enables flagellar motor rotation, linking torque machinery to cell wall [b1889]
mpl	-3.03	12.12	-1.60	12.92	0.0	11.32	0.0	0.00020054	0.00210910	0.02909997	0.48481481	A_07_P003716	b4233	cmr|putative ligase [b4233]
mraZ	-4.12	12.12	-2.04	13.15	0.0	11.10	0.1	0.00005417	0.00084354	0.01132749	0.34428571	A_07_P011082	b0081	cmr|orf, hypothetical protein [b0081]
mrcA	-2.48	10.04	-1.31	10.70	0.1	9.39	0.1	0.00060068	0.00465408	0.05157584	0.60104367	A_07_P018008	b3396	cmr|peptidoglycan synthetase; penicillin-binding protein 1A [b3396]
mrdA	-2.24	8.77	-1.17	9.35	0.1	8.18	0.4	0.01142855	0.04603687	0.06760861	0.65786774	A_07_P009743	b0635	cmr|cell elongation, e phase; peptidoglycan synthetase; penicillin-binding protein 2 [b0635]
msbA	-10.59	9.63	-3.40	11.33	0.0	7.93	0.0	0.00000320	0.00019528	0.00066184	0.08269841	A_07_P005017	b0914	cmr|ATP-binding transport protein; multicopy suppressor of htrB [b0914]
msrA	+5.16	8.97	+2.37	7.78	0.1	10.15	0.0	0.00003705	0.00066202	0.01333524	0.30694444	A_07_P003647	b4219	cmr|peptide methionine sulfoxide reductase [b4219]
msrB	+5.62	13.33	+2.49	12.09	0.1	14.58	0.0	0.00001884	0.00045351	0.01167238	0.28804075	A_07_P015584	b1778	cmr|orf, hypothetical protein [b1778]
msyB	+3.59	10.47	+1.85	9.55	0.1	11.39	0.0	0.00014850	0.00170287	0.02422447	0.40358730	A_07_P040763	c_1318	cmr|Acidic protein msyB [c_1318]
mtfA	+3.70	7.92	+1.89	6.98	0.1	8.86	0.3	0.00053309	0.00424558	0.02325965	0.39847661	A_07_P015234	b1976	cmr|orf, hypothetical protein [b1976]
mtgA	+3.09	9.36	+1.63	8.55	0.0	10.18	0.1	0.00024733	0.00245056	0.03131733	0.45110704	A_07_P012516	b3208	cmr|putative peptidoglycan enzyme [b3208]
mtlA	+5.18	8.43	+2.37	7.25	0.0	9.62	0.0	0.00002426	0.00053261	0.01340955	0.30775510	A_07_P049516	Z5023	cmr|PTS system, mannitol-specific enzyme IIABC components [Z5023]
mtlA	+6.27	8.69	+2.65	7.37	0.1	10.02	0.0	0.00001452	0.00039511	0.00952553	0.26685053	A_07_P039704	c_4416	cmr|PTS system, mannitol-specific IIABC component [c_4416]
mtlD	+3.98	9.10	+1.99	8.11	0.0	10.10	0.1	0.00007657	0.00108217	0.02036522	0.37944379	A_07_P039708	c_4417	cmr|Mannitol-1-phosphate 5-dehydrogenase [c_4417]
mtn	-2.70	12.88	-1.43	13.60	0.0	12.16	0.2	0.00069684	0.00521685	0.04000127	0.54479239	A_07_P002758	b0159	cmr|orf, hypothetical protein [b0159]
mtr	+4.37	8.13	+2.13	7.06	0.0	9.19	0.1	0.00004302	0.00073223	0.01747777	0.35414414	A_07_P000526	b3161	cmr|tryptophan-specific transport protein [b3161]
mtr	+8.96	9.54	+3.16	7.96	0.0	11.12	0.2	0.00000962	0.00032417	0.00456491	0.20019499	A_07_P041776	c_3914	cmr|Tryptophan-specific transport protein [c_3914]
mukB	+2.45	9.25	+1.29	8.60	0.1	9.90	0.2	0.00116850	0.00777552	0.04717480	0.54055313	A_07_P033851	c_1066	cmr|Cell division protein mukB [c_1066]
mukB	+3.51	9.91	+1.81	9.01	0.1	10.82	0.1	0.00012878	0.00154648	0.02485137	0.40969634	A_07_P005066	b0924	cmr|kinesin-like cell division protein involved in chromosome partitioning [b0924]
murD	+2.20	8.14	+1.14	7.58	0.1	8.71	0.2	0.00197188	0.01150478	0.05785633	0.59966425	A_07_P035759	c_0106	cmr|UDP-N-acetylmuramoylalanine--D-glutamate ligase [c_0106]
murI	-4.40	10.33	-2.14	11.40	0.1	9.26	0.1	0.00007133	0.00102376	0.00944868	0.30863071	A_07_P008540	b3967	cmr|glutamate racemase, required for biosynthesis of D-glutamate and peptidoglycan [b3967]
mutH	-2.84	8.22	-1.51	8.97	0.0	7.46	0.2	0.00092606	0.00650886	0.03418445	0.50821530	A_07_P041691	c_3426	cmr|DNA mismatch repair protein mutH [c_3426]
mutL	-2.14	9.63	-1.10	10.18	0.0	9.08	0.2	0.00212196	0.01216169	0.07607406	0.67438626	A_07_P011566	b4170	cmr|enzyme in methyl-directed mismatch repair [b4170]
mutM	-5.57	9.56	-2.48	10.80	0.1	8.32	0.1	0.00002169	0.00050073	0.00465765	0.22288754	A_07_P017118	b3635	cmr|formamidopyrimidine DNA glycosylase [b3635]
mutY	-3.26	11.93	-1.71	12.79	0.5	11.08	0.3	0.00547703	0.02552704	0.02344449	0.45289571	A_07_P011946	b2961	cmr|adenine glycosylase; G.C --> T.A transversions [b2961]
nac	-9.33	9.37	-3.22	10.98	0.1	7.76	0.1	0.00000808	0.00029787	0.00090701	0.10056338	A_07_P015279	b1988	cmr|nitrogen assimilation control protein [b1988]
nadA	-2.96	8.28	-1.56	9.06	0.2	7.50	0.2	0.00098073	0.00679905	0.03099403	0.49190524	A_07_P033430	c_0825	cmr|Quinolinate synthetase A [c_0825]
nadA	-3.65	8.28	-1.87	9.22	0.0	7.35	0.1	0.00009088	0.00121866	0.01600800	0.39257009	A_07_P046361	Z0919	cmr|quinolinate synthetase, A protein [Z0919]
nadE	+5.53	9.49	+2.47	8.26	0.0	10.73	0.5	0.00065785	0.00499623	0.01129065	0.28350877	A_07_P018749	b1740	cmr|NAD synthetase, prefers NH3 over glutamine [b1740]
nadR	+3.77	11.64	+1.91	10.69	0.3	12.60	0.1	0.00034026	0.00305885	0.02250191	0.39451002	A_07_P037369	c_5475	cmr|Transcriptional regulator nadR [c_5475]
nagA	+3.52	9.71	+1.82	8.80	0.2	10.61	0.0	0.00018938	0.00202942	0.02517149	0.41109959	A_07_P009903	b0677	cmr|N-acetylglucosamine-6-phosphate deacetylase [b0677]
nagC	+2.24	8.26	+1.16	7.68	0.1	8.84	0.1	0.00159624	0.00982073	0.05543826	0.58657258	A_07_P009896	b0676	cmr|transcriptional repressor of nag [b0676]
nagK	+4.39	10.22	+2.13	9.16	0.1	11.29	0.1	0.00006128	0.00091883	0.01742950	0.35362113	A_07_P001798	b1119	cmr|putative NAGC-like transcriptional regulator [b1119]
nagZ	+5.91	9.28	+2.56	8.00	0.0	10.56	0.0	0.00001471	0.00039645	0.01063453	0.27674380	A_07_P001737	b1107	cmr|orf, hypothetical protein [b1107]
nanA	+4.54	8.87	+2.18	7.78	0.4	9.97	0.1	0.00068033	0.00512982	0.01632241	0.34082228	A_07_P041797	c_3979	cmr|N-acetylneuraminate lyase subunit [c_3979]
nanA	+2.24	7.78	+1.17	7.20	0.1	8.36	0.2	0.00196191	0.01147840	0.05515053	0.58431359	A_07_P012599	b3225	cmr|N-acetylneuraminate lyase [b3225]
nanC	+2.76	8.47	+1.47	7.74	0.1	9.20	0.2	0.00072244	0.00536671	0.03799162	0.49108374	A_07_P004083	b4311	cmr|orf, hypothetical protein [b4311]
nanE	+3.18	8.22	+1.67	7.38	0.0	9.05	0.1	0.00025382	0.00249447	0.02955094	0.43891509	A_07_P012586	b3223	cmr|putative enzyme [b3223]
nanK	+2.72	7.39	+1.44	6.67	0.0	8.12	0.0	0.00032255	0.00295417	0.03904853	0.49539081	A_07_P012584	b3222	cmr|putative NAGC-like transcriptional regulator [b3222]
napA	+2.24	10.11	+1.16	9.53	0.1	10.69	0.0	0.00132836	0.00855713	0.05536395	0.58618023	A_07_P032316	c_2745	cmr|Periplasmic nitrate reductase precursor [c_2745]
napA	+2.29	10.22	+1.19	9.62	0.0	10.81	0.1	0.00093080	0.00653342	0.05328506	0.57342447	A_07_P032316	c_2745	cmr|Periplasmic nitrate reductase precursor [c_2745]
napA	+2.54	10.15	+1.35	9.48	0.0	10.82	0.1	0.00047233	0.00387253	0.04385417	0.51951843	A_07_P032316	c_2745	cmr|Periplasmic nitrate reductase precursor [c_2745]
napA	+2.27	10.40	+1.18	9.81	0.0	10.99	0.1	0.00143289	0.00903332	0.05422066	0.57953157	A_07_P032316	c_2745	cmr|Periplasmic nitrate reductase precursor [c_2745]
napB	+4.83	9.34	+2.27	8.20	0.1	10.48	0.1	0.00005447	0.00084566	0.01485010	0.32883263	A_07_P000804	b2203	cmr|cytochrome c-type protein [b2203]
napD	-4.08	9.99	-2.03	11.01	0.0	8.98	0.1	0.00005908	0.00089898	0.01189533	0.34681481	A_07_P000822	b2207	cmr|orf, hypothetical protein [b2207]
napF	-6.95	10.74	-2.80	12.13	0.1	9.34	0.2	0.00001978	0.00046811	0.00237297	0.16173160	A_07_P000827	b2208	cmr|ferredoxin-type protein: electron transfer [b2208]
napH	+3.70	9.28	+1.89	8.34	0.1	10.23	0.1	0.00013612	0.00160166	0.02264164	0.39519956	A_07_P000808	b2204	cmr|ferredoxin-type protein: electron transfer [b2204]
narI	+2.04	8.32	+1.03	7.81	0.1	8.83	0.0	0.00208880	0.01201539	0.06633321	0.64267692	A_07_P007089	b1227	cmr|nitrate reductase 1, cytochrome b [b1227]
narL	+4.85	11.35	+2.28	10.22	0.2	12.49	0.2	0.00010247	0.00132346	0.01473958	0.32684507	A_07_P052433	Z1996	cmr|pleiotrophic regulation of anaerobic respiration: response regulator [Z1996]
narQ	-2.32	8.91	-1.21	9.51	0.1	8.30	0.0	0.00105227	0.00716564	0.06119347	0.63508899	A_07_P013401	b2469	cmr|sensor for nitrate reductase system, protein histidine kinase [b2469]
narQ	-2.38	10.35	-1.25	10.98	0.1	9.73	0.2	0.00134790	0.00865120	0.05628874	0.61670842	A_07_P030109	c_2996	cmr|Nitrate/nitrite sensor protein narQ [c_2996]
narX	+2.20	11.38	+1.14	10.81	0.2	11.95	0.0	0.00197227	0.01150478	0.05744538	0.59618985	A_07_P007061	b1222	cmr|nitrate/nitrate sensor, histidine protein kinase acts on NarL regulator [b1222]
ndk	+3.86	10.85	+1.95	9.88	0.1	11.83	0.0	0.00008045	0.00111496	0.02128874	0.38525287	A_07_P013646	b2518	cmr|nucleoside diphosphate kinase [b2518]
nemA	+2.50	10.05	+1.32	9.39	0.0	10.71	0.1	0.00060490	0.00467758	0.04543953	0.53268801	A_07_P020882	b1650	cmr|N-ethylmaleimide reductase [b1650]
nfi	+4.13	9.38	+2.04	8.36	0.1	10.41	0.5	0.00110619	0.00745321	0.01950394	0.37493284	A_07_P037441	c_4955	cmr|Endonuclease V [c_4955]
nfi	+3.91	9.04	+1.97	8.05	0.0	10.02	0.1	0.00008519	0.00116635	0.02088859	0.38374562	A_07_P008655	b3998	cmr|endonuclease V [b3998]
nfi	+2.24	9.08	+1.16	8.50	0.3	9.66	0.2	0.00717955	0.03183181	0.05953951	0.61187337	A_07_P047202	Z5574	cmr|endonuclease V [Z5574]
nfo	+2.24	8.28	+1.16	7.70	0.1	8.86	0.1	0.00215340	0.01227927	0.05565993	0.58655288	A_07_P003556	b2159	cmr|endonuclease IV [b2159]
nhaA	-17.92	10.91	-4.16	13.00	0.0	8.83	0.2	0.00000234	0.00017963	0.00008194	0.02866667	A_07_P010784	b0019	cmr|Na+/H antiporter, pH dependent [b0019]
nhaB	+2.16	8.22	+1.11	7.66	0.2	8.77	0.2	0.00751171	0.03298867	0.06460556	0.63651439	A_07_P006885	b1186	cmr|Na+/H+ antiporter, pH independent [b1186]
nhaR	-3.58	9.49	-1.84	10.41	0.1	8.57	0.1	0.00013293	0.00157480	0.01744919	0.40400000	A_07_P035651	c_0025	cmr|Transcriptional activator protein nhaR [c_0025]
nikA	+2.52	7.67	+1.33	7.01	0.2	8.34	0.0	0.00134774	0.00865120	0.04526486	0.53103577	A_07_P007316	b3476	cmr|periplasmic binding protein for nickel [b3476]
nikD	+2.50	8.09	+1.32	7.42	0.1	8.75	0.2	0.00214551	0.01225405	0.04549352	0.53292411	A_07_P007331	b3479	cmr|ATP-binding protein of nickel transport system [b3479]
nikD	-2.56	10.12	-1.36	10.79	0.1	9.44	0.2	0.00094428	0.00659868	0.04704205	0.57591757	A_07_P059977	ECs4346	cmr|ATP-binding protein of nickel transport system [ECs4346]
nlpD	+3.22	16.08	+1.69	15.24	0.0	16.93	0.0	0.00013974	0.00163451	0.02880462	0.43816425	A_07_P002160	b2742	cmr|lipoprotein [b2742]
nlpI	-6.31	14.78	-2.66	16.11	0.0	13.45	0.1	0.00001626	0.00041974	0.00321202	0.18729630	A_07_P000538	b3163	cmr|putative control proteins [b3163]
nmpC	+88.32	11.59	+6.46	8.36	0.5	14.82	0.0	0.00000507	0.00024272	0.00003239	0.01457143	A_07_P038497	c_1560	cmr|Outer membrane porin protein nmpC precursor [c_1560]
nohA	+3.54	9.87	+1.82	8.95	0.0	10.78	0.1	0.00010013	0.00130829	0.02450140	0.40648051	A_07_P038523	c_1568	cmr|Prophage Qin DNA packaging protein NU1 homolog [c_1568]
norR	-2.14	7.89	-1.10	8.44	0.1	7.34	0.0	0.00184414	0.01096872	0.07697472	0.67665550	A_07_P001994	b2709	cmr|putative 2-component transcriptional regulator [b2709]
npr	+3.08	9.02	+1.62	8.21	0.2	9.83	0.1	0.00053552	0.00425823	0.03148056	0.45180492	A_07_P012510	b3206	cmr|phosphocarrier protein HPr-like NPr, nitrogen related, exchanges phosphate with Enzyme I, Hpr [b3206]
nrdA	+29.09	10.37	+4.86	7.93	0.1	12.80	0.0	0.00000059	0.00010477	0.00034172	0.05434343	A_07_P000956	b2234	cmr|ribonucleoside diphosphate reductase 1, alpha subunit, B1 [b2234]
nrdB	+6.56	10.20	+2.71	8.85	0.1	11.56	0.1	0.00001929	0.00046234	0.00864583	0.26026769	A_07_P000962	b2235	cmr|ribonucleoside-diphosphate reductase 1, beta subunit, B2 [b2235]
nrdD	+14.95	12.36	+3.90	10.41	0.1	14.31	0.0	0.00000182	0.00016679	0.00160633	0.12336585	A_07_P003742	b4238	cmr|anaerobic ribonucleoside-triphosphate reductase [b4238]
nrdD	+12.46	12.73	+3.64	10.91	0.0	14.55	0.3	0.00001569	0.00041046	0.00232597	0.14474308	A_07_P042066	c_5337	cmr|Anaerobic ribonucleoside-triphosphate reductase [c_5337]
nrdG	+5.51	9.64	+2.46	8.41	0.1	10.88	0.0	0.00002490	0.00054138	0.01203379	0.29192604	A_07_P003737	b4237	cmr|anaerobic ribonucleotide reductase activating protein [b4237]
nrdR	-4.27	13.22	-2.09	14.27	0.1	12.18	0.0	0.00004826	0.00078331	0.01019944	0.31861111	A_07_P021179	b0413	cmr|orf, hypothetical protein [b0413]
nudB	+2.04	10.87	+1.03	10.36	0.1	11.38	0.1	0.00292791	0.01563917	0.06768991	0.65061661	A_07_P016003	b1865	cmr|dATP pyrophosphohydrolase [b1865]
nudB	+2.21	10.75	+1.14	10.18	0.0	11.33	0.1	0.00118801	0.00786874	0.05673653	0.59156291	A_07_P016003	b1865	cmr|dATP pyrophosphohydrolase [b1865]
nudE	-5.88	9.93	-2.56	11.21	0.1	8.65	0.2	0.00006268	0.00093445	0.00392975	0.20623333	A_07_P018014	b3397	cmr|orf, hypothetical protein [b3397]
nudE	-4.61	10.47	-2.21	11.57	0.1	9.37	0.1	0.00005291	0.00083469	0.00826283	0.29432127	A_07_P059766	ECs4239	cmr|hypothetical protein [ECs4239]
nudE	-4.80	10.97	-2.26	12.10	0.0	9.83	0.0	0.00002836	0.00057633	0.00725610	0.27728155	A_07_P046016	Z4751	cmr|orf, hypothetical protein [Z4751]
nudF	-2.20	10.08	-1.14	10.65	0.1	9.51	0.4	0.01002079	0.04155053	0.06680196	0.65610106	A_07_P012303	b3034	cmr|orf, hypothetical protein [b3034]
nudI	+3.86	8.45	+1.95	7.47	0.1	9.42	0.0	0.00007997	0.00111032	0.02123920	0.38524194	A_07_P001045	b2251	cmr|orf, hypothetical protein [b2251]
nudJ	-3.75	10.75	-1.91	11.70	0.0	9.79	0.2	0.00023777	0.00238308	0.01558371	0.39193291	A_07_P006621	b1134	cmr|putative phosphohydrolase [b1134]
nuoA	+2.65	12.11	+1.41	11.40	0.0	12.81	0.1	0.00046695	0.00384264	0.04106898	0.50593897	A_07_P013728	b2288	cmr|NADH dehydrogenase I chain A [b2288]
nuoB	+2.50	12.78	+1.32	12.12	0.1	13.44	0.2	0.00107817	0.00730302	0.04572980	0.53489599	A_07_P013721	b2287	cmr|NADH dehydrogenase I chain B [b2287]
nuoC	+34.63	12.13	+5.11	9.58	0.0	14.69	0.0	0.00000038	0.00009117	0.00025025	0.04690476	A_07_P013718	b2286	cmr|NADH dehydrogenase I chain C, D [b2286]
nuoE	+31.97	14.09	+5.00	11.59	0.0	16.59	0.2	0.00000166	0.00015927	0.00028328	0.04955556	A_07_P042543	c_2826	cmr|NADH dehydrogenase I chain E [c_2826]
nuoE	+63.10	12.73	+5.98	9.74	0.0	15.72	0.1	0.00000016	0.00007760	0.00007495	0.02360000	A_07_P013712	b2285	cmr|NADH dehydrogenase I chain E [b2285]
nuoE	+23.27	12.36	+4.54	10.09	0.1	14.63	0.1	0.00000206	0.00017536	0.00054179	0.07108333	A_07_P052679	Z3544	cmr|NADH dehydrogenase I chain E [Z3544]
nuoF	+72.89	11.57	+6.19	8.48	0.1	14.67	0.0	0.00000023	0.00008900	0.00006161	0.02155556	A_07_P013708	b2284	cmr|NADH dehydrogenase I chain F [b2284]
nuoG	+35.10	9.88	+5.13	7.31	0.1	12.45	0.1	0.00000061	0.00010566	0.00023882	0.04641975	A_07_P013701	b2283	cmr|NADH dehydrogenase I chain G [b2283]
nuoG	+54.49	11.06	+5.77	8.18	0.0	13.94	0.0	0.00000018	0.00008010	0.00010544	0.02862069	A_07_P045556	Z3542	cmr|NADH dehydrogenase I chain G [Z3542]
nuoH	+107.87	10.90	+6.75	7.52	0.0	14.27	0.1	0.00000012	0.00007248	0.00001842	0.00966667	A_07_P013700	b2282	cmr|NADH dehydrogenase I chain H [b2282]
nuoI	+34.72	10.55	+5.12	7.99	0.7	13.11	0.0	0.00006808	0.00099497	0.00025470	0.04662791	A_07_P013694	b2281	cmr|NADH dehydrogenase I chain I [b2281]
nuoJ	+47.24	10.84	+5.56	8.06	0.3	13.62	0.1	0.00000173	0.00016209	0.00013720	0.03176471	A_07_P013686	b2280	cmr|NADH dehydrogenase I chain J [b2280]
nuoK	+55.19	9.92	+5.79	7.03	0.0	12.82	0.2	0.00000048	0.00009267	0.00009654	0.02867925	A_07_P001181	b2279	cmr|NADH dehydrogenase I chain K [b2279]
nuoK	+45.86	9.96	+5.52	7.20	0.0	12.72	0.3	0.00000176	0.00016311	0.00014672	0.03300000	A_07_P045553	Z3538	cmr|NADH dehydrogenase I chain K [Z3538]
nuoL	+26.30	9.83	+4.72	7.47	0.4	12.18	0.1	0.00000761	0.00029299	0.00042556	0.06320755	A_07_P001178	b2278	cmr|NADH dehydrogenase I chain L [b2278]

nuoM	+35.44	9.75	+5.15	7.18	0.2	12.32	0.1	0.00000104	0.00012946	0.00024073	0.04566265	A_07_P001174	b2277	cmr|NADH dehydrogenase I chain M [b2277]
nuoN	+14.34	8.64	+3.84	6.72	0.1	10.57	0.0	0.00000238	0.00017963	0.00175114	0.12646789	A_07_P051791	Z3534	cmr|NADH dehydrogenase I chain N [Z3534]
nuoN	+10.25	8.71	+3.36	7.03	0.2	10.39	0.0	0.00000867	0.00030685	0.00347180	0.17407643	A_07_P042539	c_2817	cmr|NADH dehydrogenase I chain N [c_2817]
nuoN	+8.39	8.71	+3.07	7.17	0.2	10.24	0.1	0.00002615	0.00055409	0.00518610	0.21374346	A_07_P001166	b2276	cmr|NADH dehydrogenase I chain N [b2276]
nupG	+6.83	10.38	+2.77	9.00	0.1	11.77	0.0	0.00001110	0.00034947	0.00794017	0.24803571	A_07_P011964	b2964	cmr|transport of nucleosides, permease protein [b2964]
nusA	-4.54	14.43	-2.18	15.52	0.1	13.34	0.1	0.00005076	0.00080800	0.00845020	0.29564444	A_07_P000566	b3169	cmr|transcription pausing; L factor [b3169]
nusB	-12.11	12.13	-3.60	13.93	0.0	10.33	0.1	0.00000307	0.00019257	0.00036966	0.05938776	A_07_P021194	b0416	cmr|transcription termination; L factor [b0416]
nusG	-2.51	8.92	-1.33	9.58	0.2	8.25	0.1	0.00139443	0.00887382	0.04917429	0.58518519	A_07_P008575	b3982	cmr|component in transcription antitermination [b3982]
obgE	-5.67	9.47	-2.50	10.72	0.1	8.21	0.3	0.00009742	0.00128285	0.00436801	0.21423676	A_07_P012391	b3183	cmr|putative GTP-binding factor [b3183]
ompC	+3.93	9.77	+1.97	8.79	0.1	10.76	0.1	0.00010131	0.00131881	0.02083333	0.38317757	A_07_P032359	c_2758	cmr|Outer membrane protein C precursor [c_2758]
ompC	+3.37	8.93	+1.75	8.05	0.1	9.80	0.2	0.00027910	0.00265270	0.02698361	0.42229622	A_07_P000863	b2215	cmr|outer membrane protein 1b [b2215]
ompC	+2.39	10.40	+1.25	9.77	0.1	11.02	0.1	0.00124280	0.00814936	0.04897294	0.54995007	A_07_P045517	Z3473	cmr|outer membrane protein 1b [Z3473]
ompF	+7.59	10.25	+2.92	8.79	0.1	11.71	0.0	0.00001007	0.00033383	0.00643483	0.23025000	A_07_P005092	b0929	cmr|outer membrane protein 1a [b0929]
ompT	+7.54	9.10	+2.91	7.64	0.3	10.55	0.0	0.00006511	0.00096619	0.00645516	0.22993213	A_07_P031283	c_0652	cmr|Protease VII precursor [c_0652]
ompW	+86.95	10.18	+6.44	6.96	0.0	13.40	0.1	0.00000011	0.00007032	0.00003430	0.01500000	A_07_P007216	b1256	cmr|putative outer membrane protein [b1256]
ompW	+264.62	12.67	+8.05	8.65	0.0	16.70	0.0	0.00000003	0.00007032	0.00000254	0.00307692	A_07_P038872	c_1722	cmr|Outer membrane protein W precursor [c_1722]
ompX	-16.00	13.32	-4.00	15.32	0.0	11.32	0.1	0.00000155	0.00015822	0.00012513	0.03716981	A_07_P004524	b0814	cmr|outer membrane protein X [b0814]
oppA	+315.13	12.27	+8.30	8.12	0.2	16.42	0.1	0.00000010	0.00007032	0.00000127	0.00200000	A_07_P007151	b1243	cmr|oligopeptide transport; periplasmic binding protein [b1243]
oppA	+349.11	13.18	+8.45	8.95	0.0	17.40	0.1	0.00000003	0.00007032	0.00000064	0.00111111	A_07_P052437	Z2019	cmr|oligopeptide transport periplasmic binding protein [Z2019]
oppB	+13.64	10.58	+3.77	8.70	0.0	12.47	0.1	0.00000257	0.00018134	0.00192708	0.12855932	A_07_P007157	b1244	cmr|oligopeptide transport permease protein [b1244]
oppC	+15.22	9.18	+3.93	7.21	0.0	11.14	0.4	0.00001836	0.00044549	0.00154535	0.11985222	A_07_P007165	b1245	cmr|homolog of Salmonella oligopeptide transport permease protein [b1245]
oppD	+58.96	11.45	+5.88	8.51	0.2	14.39	0.2	0.00000085	0.00012742	0.00008638	0.02666667	A_07_P007166	b1246	cmr|homolog of Salmonella ATP-binding protein of oligopeptide ABC transport system [b1246]
oppF	+25.53	9.84	+4.67	7.50	0.3	12.18	0.6	0.00007302	0.00104221	0.00050622	0.06811966	A_07_P007171	b1247	cmr|homolog of Salmonella ATP-binding protein of oligopeptide ABC transport system [b1247]
osmB	-4.36	9.16	-2.12	10.22	0.1	8.10	0.0	0.00005075	0.00080800	0.00969576	0.30963489	A_07_P014396	b1283	cmr|osmotically inducible lipoprotein [b1283]
osmE	+2.35	8.13	+1.23	7.52	0.1	8.75	0.1	0.00089097	0.00631227	0.05027185	0.55503506	A_07_P018744	b1739	cmr|activator of ntrL gene [b1739]
osmY	+2.40	8.73	+1.26	8.10	0.1	9.36	0.1	0.00135768	0.00869973	0.04835556	0.54731129	A_07_P017561	b4376	cmr|hyperosmotically inducible periplasmic protein [b4376]
pabA	-2.93	8.79	-1.55	9.56	0.0	8.01	0.3	0.00158261	0.00974826	0.03072853	0.49215666	A_07_P017829	b3360	cmr|p-aminobenzoate synthetase, component II [b3360]
pagB	+2.00	9.54	+1.00	9.04	0.1	10.04	0.2	0.00397609	0.01986659	0.07105882	0.66434086	A_07_P008756	b4023	cmr|orf, hypothetical protein [b4023]
panB	-2.80	10.54	-1.49	11.28	0.0	9.79	0.1	0.00036925	0.00323332	0.03616362	0.51996347	A_07_P002634	b0134	cmr|3-methyl-2-oxobutanoate hydroxymethyltransferase [b0134]
panB	-3.69	12.47	-1.88	13.41	0.0	11.53	0.1	0.00012395	0.00150701	0.01564787	0.39166932	A_07_P035825	c_0165	cmr|3-methyl-2-oxobutanoate hydroxymethyltransferase [c_0165]
panC	-2.00	9.61	-1.00	10.11	0.1	9.11	0.1	0.00279659	0.01504766	0.09048717	0.72352971	A_07_P049200	Z0144	cmr|pantothenate synthetase [Z0144]
panD	+2.07	11.15	+1.05	10.62	0.0	11.67	0.0	0.00152295	0.00945346	0.06445948	0.63547276	A_07_P002617	b0131	cmr|aspartate 1-decarboxylase [b0131]
panE	+2.00	8.89	+1.00	8.39	0.1	9.39	0.0	0.00323174	0.01690943	0.07010671	0.66093413	A_07_P021236	b0425	cmr|involved in thiamin biosynthesis, alternative pyrimidine biosynthesis [b0425]
papA	+32.04	9.74	+5.00	7.24	0.2	12.24	0.0	0.00000144	0.00015308	0.00028963	0.05010989	A_07_P033164	c_3592	cmr|PapA protein [c_3592]
papC	+11.21	9.71	+3.49	7.97	0.1	11.46	0.0	0.00000370	0.00020738	0.00288110	0.15804878	A_07_P033151	c_3590	cmr|PapC protein [c_3590]
papD_2	+7.64	10.12	+2.93	8.65	0.1	11.58	0.1	0.00001297	0.00037688	0.00627287	0.22755760	A_07_P037957	c_5185	cmr|PapD protein [c_5185]
papE	+10.00	10.37	+3.32	8.71	0.1	12.03	0.0	0.00000666	0.00027022	0.00361344	0.17833856	A_07_P033134	c_3585	cmr|PapE protein [c_3585]
papF	+3.25	8.80	+1.70	7.95	0.2	9.65	0.3	0.00107617	0.00729679	0.02913110	0.43721640	A_07_P033130	c_3584	cmr|PapF protein [c_3584]
papG	+2.59	10.14	+1.37	9.46	0.1	10.83	0.2	0.00151594	0.00941866	0.04295097	0.51228788	A_07_P033125	c_3583	cmr|PapG protein [c_3583]
papH	+9.24	11.23	+3.21	9.62	0.3	12.83	0.2	0.00004690	0.00076831	0.00424860	0.19221264	A_07_P033156	c_3591	cmr|PapH protein [c_3591]
papH_2	+12.26	9.43	+3.62	7.62	0.2	11.24	0.3	0.00001677	0.00042622	0.00240600	0.14458015	A_07_P037969	c_5187	cmr|PapH protein [c_5187]
papI_2	+7.37	8.51	+2.88	7.07	0.2	9.95	0.0	0.00002821	0.00057601	0.00672256	0.23415929	A_07_P037980	c_5189	cmr|PapI protein [c_5189]
papJ	+4.63	9.63	+2.21	8.52	0.0	10.73	0.1	0.00004566	0.00075668	0.01602579	0.33821716	A_07_P033147	c_3588	cmr|PapJ protein [c_3588]
papK	+5.64	10.20	+2.50	8.96	0.2	11.45	0.4	0.00038548	0.00332912	0.01116489	0.28215088	A_07_P033137	c_3586	cmr|PapK protein [c_3586]
papX	+2.75	11.59	+1.46	10.86	0.1	12.32	0.2	0.00094640	0.00661052	0.03854865	0.49462918	A_07_P033116	c_3582	cmr|PapX protein [c_3582]
pck	+17.28	15.09	+4.11	13.03	0.1	17.14	0.2	0.00000321	0.00019528	0.00116425	0.10071429	A_07_P018041	b3403	cmr|phosphoenolpyruvate carboxykinase [b3403]
pcnB	-2.00	11.63	-1.00	12.13	0.1	11.13	0.2	0.00392070	0.01962717	0.08864139	0.72234472	A_07_P002676	b0143	cmr|poly [b0143]
pdhR	+2.27	11.87	+1.18	11.28	0.1	12.46	0.1	0.00096111	0.00669841	0.05395833	0.57908657	A_07_P002527	b0113	cmr|transcriptional regulator for pyruvate dehydrogenase complex [b0113]
pdxJ	+5.65	9.32	+2.50	8.08	0.0	10.57	0.1	0.00001950	0.00046446	0.01138275	0.28446032	A_07_P019721	b2564	cmr|pyridoxine biosynthesis [b2564]
pepA	+2.66	12.48	+1.41	11.78	0.0	13.19	0.1	0.00038527	0.00332912	0.04086763	0.50424765	A_07_P003848	b4260	cmr|aminopeptidase A/I [b4260]
pepB	+9.94	10.21	+3.31	8.56	0.0	11.87	0.2	0.00000800	0.00029787	0.00368966	0.18153125	A_07_P013674	b2523	cmr|putative peptidase [b2523]
pepD	+17.33	10.82	+4.12	8.76	0.1	12.88	0.0	0.00000168	0.00015927	0.00116997	0.10065574	A_07_P007896	b0237	cmr|aminoacyl-histidine dipeptidase [b0237]
pepE	+3.22	7.81	+1.69	6.96	0.1	8.65	0.0	0.00017951	0.00195827	0.02878303	0.43825919	A_07_P008748	b4021	cmr|peptidase E, a dipeptidase where amino-terminal residue is aspartate [b4021]
pepN	+16.66	9.85	+4.06	7.82	0.1	11.88	0.0	0.00000163	0.00015927	0.00124365	0.10470588	A_07_P051734	Z1280	cmr|aminopeptidase N [Z1280]
pepT	+3.71	9.03	+1.89	8.09	0.1	9.98	0.2	0.00035283	0.00313486	0.02316184	0.39940854	A_07_P006587	b1127	cmr|putative peptidase T [b1127]
perM	-3.62	9.14	-1.86	10.07	0.1	8.21	0.0	0.00011652	0.00144104	0.01674860	0.39772247	A_07_P030120	c_3009	cmr|Putative permease perM [c_3009]
pfkB	+6.02	9.56	+2.59	8.26	0.1	10.85	0.1	0.00002767	0.00056928	0.01013910	0.27333904	A_07_P041160	c_2121	cmr|6-phosphofructokinase isozyme 2 [c_2121]
pfkB	+5.32	9.51	+2.41	8.31	0.1	10.72	0.5	0.00055975	0.00439729	0.01226499	0.29346505	A_07_P018663	b1723	cmr|6-phosphofructokinase II; suppressor of pfkA [b1723]
pflA	-4.39	12.63	-2.13	13.69	0.0	11.56	0.0	0.00003889	0.00068231	0.00951728	0.30894845	A_07_P004958	b0902	cmr|pyruvate formate lyase activating enzyme 1 [b0902]
pflB	+15.10	11.27	+3.92	9.32	0.1	13.23	0.6	0.00009677	0.00127819	0.00152503	0.11945274	A_07_P004961	b0903	cmr|formate acetyltransferase 1 [b0903]
pflD	+2.21	9.16	+1.14	8.59	0.1	9.73	0.1	0.00152342	0.00945346	0.05680831	0.59152778	A_07_P008458	b3951	cmr|formate acetyltransferase 2 [b3951]
pgaD	-4.61	9.12	-2.20	10.22	0.0	8.01	0.0	0.00003242	0.00062637	0.00825965	0.29487528	A_07_P001332	b1021	cmr|orf, hypothetical protein [b1021]
pgi	+3.25	9.07	+1.70	8.22	0.3	9.92	0.2	0.00154886	0.00956658	0.02898183	0.43874038	A_07_P008767	b4025	cmr|glucosephosphate isomerase [b4025]
pgk	+10.50	13.41	+3.39	11.71	0.1	15.10	0.0	0.00000385	0.00021100	0.00327045	0.16826797	A_07_P011776	b2926	cmr|phosphoglycerate kinase [b2926]
pgl	+7.94	9.43	+2.99	7.94	0.0	10.93	0.1	0.00001152	0.00035443	0.00594512	0.22285714	A_07_P004288	b0767	cmr|putative isomerase [b0767]
pheP	-2.82	8.51	-1.50	9.26	0.1	7.76	0.1	0.00035393	0.00314106	0.03595147	0.51880843	A_07_P016642	b0576	cmr|phenylalanine-specific transport system [b0576]
pheS	+5.51	11.49	+2.46	10.26	0.0	12.72	0.2	0.00004471	0.00075050	0.01186039	0.29085670	A_07_P018621	b1714	cmr|phenylalanine tRNA synthetase, alpha-subunit [b1714]
pheT	+2.77	10.25	+1.47	9.51	0.1	10.98	0.0	0.00033403	0.00302754	0.03790904	0.49122634	A_07_P018619	b1713	cmr|phenylalanine tRNA synthetase, beta-subunit [b1713]
phnH	+2.34	8.48	+1.23	7.87	0.1	9.09	0.4	0.00607614	0.02778472	0.05153582	0.56345833	A_07_P037761	c_5106	cmr|PhnH protein [c_5106]
phnI	-4.53	10.79	-2.18	11.88	0.1	9.70	0.6	0.00169363	0.01029914	0.00893229	0.30308190	A_07_P011231	b4099	cmr|phosphonate metabolism [b4099]
phnI	-2.85	9.88	-1.51	10.63	0.3	9.12	0.2	0.00189060	0.01116073	0.03340892	0.50285851	A_07_P041400	c_5105	cmr|PhnI protein [c_5105]
phnJ	+2.12	7.41	+1.09	6.86	0.2	7.95	0.2	0.00483696	0.02309067	0.06217988	0.62473516	A_07_P011230	b4098	cmr|phosphonate metabolism [b4098]
phnK	+2.38	7.77	+1.25	7.14	0.2	8.39	0.1	0.00224916	0.01270569	0.05022612	0.55530899	A_07_P011223	b4097	cmr|ATP-binding component of phosphonate transport [b4097]
phnK	-4.40	8.68	-2.14	9.75	0.4	7.61	0.1	0.00054782	0.00432759	0.00899835	0.30401288	A_07_P041390	c_5103	cmr|Phosphonates transport ATP-binding protein phnK [c_5103]
phoB	-9.63	11.42	-3.27	13.06	0.1	9.79	0.2	0.00001036	0.00033836	0.00085810	0.09650000	A_07_P031103	c_0508	cmr|Phosphate regulon transcriptional Regulatory protein phoB [c_0508]
phoB	-9.04	11.17	-3.18	12.76	0.0	9.58	0.2	0.00001365	0.00038700	0.00105755	0.10471698	A_07_P021109	b0399	cmr|positive response regulator for pho regulon, sensor is PhoR [b0399]
phoB	-7.84	11.43	-2.97	12.92	0.0	9.95	0.2	0.00001543	0.00040762	0.00164761	0.13167513	A_07_P043823	Z0497	cmr|positive response regulator for pho regulon, sensor [Z0497]
phoR	-3.26	8.23	-1.70	9.08	0.1	7.38	0.1	0.00022051	0.00226614	0.02341527	0.45288698	A_07_P021115	b0400	cmr|positive and negative sensor protein for pho regulon [b0400]
pinR	-2.48	8.55	-1.31	9.20	0.1	7.89	0.1	0.00093847	0.00657715	0.05074441	0.59443452	A_07_P014850	b1374	cmr|putative transposon resolvase [b1374]
pitA	-2.59	9.92	-1.37	10.61	0.1	9.24	0.1	0.00054476	0.00431420	0.04527693	0.57164395	A_07_P007401	b3493	cmr|low-affinity phosphate transport [b3493]
pitB	-2.34	8.23	-1.22	8.84	0.1	7.61	0.2	0.00203057	0.01177507	0.05975165	0.63051609	A_07_P012066	b2987	cmr|low-affinity phosphate transport [b2987]
pldB	-2.52	10.33	-1.33	10.99	0.1	9.66	0.3	0.00207902	0.01199415	0.04800051	0.57953988	A_07_P041944	c_4747	cmr|Lysophospholipase L2 [c_4747]
pldB	-2.25	10.01	-1.17	10.59	0.0	9.42	0.0	0.00091866	0.00647676	0.06777121	0.65863580	A_07_P046996	Z5346	cmr|lysophospholipase L [Z5346]
plsC	-2.55	10.31	-1.35	10.99	0.0	9.64	0.0	0.00041629	0.00352830	0.04731771	0.57704880	A_07_P036768	c_3758	cmr|1-acyl-sn-glycerol-3-phosphate acyltransferase [c_3758]
plsC	-2.54	8.59	-1.34	9.26	0.1	7.92	0.1	0.00056796	0.00444873	0.04788364	0.58035412	A_07_P012224	b3018	cmr|1-acyl-sn-glycerol-3-phosphate acyltransferase [b3018]
pmbA	+4.04	9.05	+2.01	8.04	0.2	10.05	0.2	0.00023934	0.00239252	0.01977833	0.37744242	A_07_P003729	b4235	cmr|maturation of antibiotic MccB17, see tld genes [b4235]
pntA	+11.69	9.20	+3.55	7.42	0.2	10.97	0.4	0.00006848	0.00099742	0.00268991	0.15017730	A_07_P020648	b1603	cmr|pyridine nucleotide transhydrogenase, alpha subunit [b1603]
pntB	+6.37	10.35	+2.67	9.01	0.2	11.68	0.1	0.00003750	0.00066782	0.00909934	0.26286239	A_07_P020644	b1602	cmr|pyridine nucleotide transhydrogenase, beta subunit [b1602]
pntB	+3.67	9.59	+1.87	8.65	0.0	10.52	0.1	0.00010220	0.00132269	0.02321900	0.39908297	A_07_P052517	Z2597	cmr|pyridine nucleotide transhydrogenase, beta subunit [Z2597]
pntB	+2.68	9.40	+1.42	8.69	0.2	10.11	0.2	0.00139901	0.00889583	0.04061547	0.50231736	A_07_P063202	ECs2308	cmr|pyridine nucleotide transhydrogenase beta subunit [ECs2308]
polA	+7.47	9.11	+2.90	7.66	0.2	10.56	0.0	0.00001507	0.00040227	0.00662792	0.23292411	A_07_P019099	b3863	cmr|DNA polymerase I, 3 --> 5 polymerase, 5 --> 3 and 3 --> 5 exonuclease [b3863]
polA	+5.69	9.16	+2.51	7.91	0.4	10.42	0.0	0.00019756	0.00208752	0.01096862	0.28125407	A_07_P052260	Z5398	cmr|DNA polymerase I, 3 --> 5 polymerase [Z5398]
polB	-4.32	10.60	-2.11	11.66	0.0	9.55	0.1	0.00004989	0.00080150	0.00996443	0.31629032	A_07_P010989	b0060	cmr|DNA polymerase II [b0060]
potA	-5.65	10.10	-2.50	11.35	0.0	8.85	0.2	0.00004552	0.00075592	0.00445376	0.21708978	A_07_P006583	b1126	cmr|ATP-binding component of spermidine/putrescine transport [b1126]
potC	-4.03	11.77	-2.01	12.77	0.0	10.76	0.1	0.00009773	0.00128464	0.01226118	0.34657092	A_07_P006571	b1124	cmr|spermidine/putrescine transport system permease [b1124]
potD	+3.02	12.81	+1.60	12.01	0.0	13.60	0.1	0.00020298	0.00212689	0.03234121	0.45830783	A_07_P052411	Z1762	cmr|spermidine/putrescine periplasmic transport protein [Z1762]
potD	+3.71	13.51	+1.89	12.56	0.0	14.45	0.4	0.00085531	0.00612371	0.02340384	0.40007600	A_07_P042212	c_1398	cmr|Spermidine/putrescine-binding periplasmic protein precursor [c_1398]
potD	+2.95	12.95	+1.56	12.17	0.0	13.73	0.0	0.00022104	0.00227013	0.03391260	0.46794040	A_07_P063111	ECs1499	cmr|spermidine/putrescine periplasmic transport protein [ECs1499]
potD	+5.20	13.04	+2.38	11.85	0.1	14.23	0.2	0.00007682	0.00108465	0.01298526	0.30242604	A_07_P001819	b1123	cmr|spermidine/putrescine periplasmic transport protein [b1123]
potE	+4.71	8.29	+2.24	7.17	0.2	9.41	0.1	0.00006856	0.00099768	0.01542556	0.33359890	A_07_P009978	b0692	cmr|putrescine transport protein [b0692]
ppa	-3.11	10.40	-1.63	11.21	0.1	9.58	0.1	0.00023400	0.00235854	0.02680323	0.47521396	A_07_P003684	b4226	cmr|inorganic pyrophosphatase [b4226]
ppc	+10.73	9.84	+3.42	8.13	0.1	11.55	0.0	0.00000448	0.00022850	0.00316311	0.16544850	A_07_P008482	b3956	cmr|phosphoenolpyruvate carboxylase [b3956]
ppdA	+2.40	7.92	+1.26	7.28	0.2	8.55	0.0	0.00162340	0.00995666	0.04902820	0.54939502	A_07_P043261	Z4143	cmr|prepilin peptidase dependent protein A [Z4143]
ppdA	+2.37	8.13	+1.25	7.50	0.1	8.75	0.1	0.00089450	0.00632947	0.05002223	0.55461268	A_07_P032800	c_3420	cmr|Prepilin peptidase dependent protein A precursor [c_3420]
ppiD	-2.46	9.82	-1.30	10.47	0.1	9.17	0.1	0.00062263	0.00475860	0.05271087	0.60442826	A_07_P021316	b0441	cmr|putative protease maturation protein [b0441]
ppk	+5.31	10.16	+2.41	8.95	0.2	11.36	0.1	0.00005028	0.00080433	0.01260226	0.29657698	A_07_P013561	b2501	cmr|polyphosphate kinase [b2501]
pps	+6.90	13.83	+2.79	12.44	0.0	15.22	0.1	0.00001047	0.00034033	0.00780234	0.24816162	A_07_P018562	b1702	cmr|phosphoenolpyruvate synthase [b1702]
pqiB	+2.91	11.79	+1.54	11.02	0.1	12.56	0.0	0.00031603	0.00290970	0.03463415	0.47210390	A_07_P005203	b0951	cmr|paraquat-inducible protein B [b0951]
prfB	-3.39	13.74	-1.76	14.62	0.0	12.86	0.0	0.00011010	0.00139340	0.02060340	0.43424364	A_07_P006421	b2891	cmr|peptide chain release factor RF-2 [b2891]
prfB	-3.45	14.18	-1.79	15.07	0.0	13.29	0.0	0.00010651	0.00135668	0.01928862	0.41944751	A_07_P032852	c_5622	cmr|Peptide chain release factor 2 [c_5622]
prfC	-2.55	10.50	-1.35	11.18	0.1	9.83	0.0	0.00044656	0.00371993	0.04754129	0.57932663	A_07_P017557	b4375	cmr|peptide chain release factor RF-3 [b4375]
prfH	-13.26	10.15	-3.73	12.01	0.0	8.28	0.2	0.00000787	0.00029735	0.00028138	0.05337349	A_07_P030853	c_0382	cmr|Peptide chain release factor homolog [c_0382]
prfH	-7.03	10.87	-2.81	12.28	0.2	9.46	0.1	0.00003027	0.00060404	0.00230437	0.15912281	A_07_P007894	b0236	cmr|probable peptide chain release factor [b0236]
prfH	-5.49	9.44	-2.46	10.67	0.1	8.21	0.1	0.00003515	0.00065097	0.00485455	0.22545723	A_07_P062922	ECs0263	cmr|probable peptide chain release factor [ECs0263]
prfH	-5.85	9.40	-2.55	10.67	0.0	8.12	0.2	0.00003283	0.00062948	0.00394118	0.20614618	A_07_P051892	Z0297	cmr|probable peptide chain release factor [Z0297]
priA	-2.21	8.55	-1.14	9.12	0.1	7.98	0.2	0.00409882	0.02033244	0.07043509	0.66244325	A_07_P019451	b3935	cmr|primosomal protein N [b3935]
priA	-4.02	9.76	-2.01	10.76	0.2	8.76	0.3	0.00080123	0.00580513	0.01178989	0.34760300	A_07_P039449	c_4888	cmr|Primosomal protein N [c_4888]
priB	-3.63	15.80	-1.86	16.73	0.0	14.87	0.1	0.00009012	0.00121169	0.01640434	0.39370427	A_07_P011721	b4201	cmr|primosomal replication protein N [b4201]
prkB	+2.09	8.60	+1.06	8.07	0.2	9.13	0.1	0.00496675	0.02360192	0.06511560	0.63834371	A_07_P052140	Z4716	cmr|probable phosphoribulokinase [Z4716]
prlC	+2.68	8.92	+1.42	8.20	0.0	9.63	0.2	0.00105221	0.00716564	0.03969449	0.49996000	A_07_P007426	b3498	cmr|oligopeptidase A [b3498]
prlC	+3.20	9.60	+1.68	8.76	0.0	10.44	0.2	0.00032484	0.00296476	0.02941629	0.43940228	A_07_P041321	c_4297	cmr|Oligopeptidase A [c_4297]
proB	-2.59	8.80	-1.37	9.48	0.1	8.11	0.3	0.00231201	0.01299546	0.04434769	0.56903830	A_07_P051897	Z0303	cmr|gamma-glutamate kinase [Z0303]
proP	-7.13	10.03	-2.83	11.45	0.1	8.61	0.0	0.00000966	0.00032448	0.00219068	0.15677273	A_07_P011292	b4111	cmr|low-affinity transport system; proline permease II [b4111]
proX	+3.30	8.35	+1.72	7.49	0.5	9.21	0.1	0.00339375	0.01752604	0.02839939	0.43621463	A_07_P001866	b2679	cmr|high-affinity transport system for glycine betaine and proline [b2679]
proX	+6.14	9.12	+2.62	7.81	0.2	10.43	0.4	0.00028293	0.00267294	0.00950711	0.26728571	A_07_P030557	c_3232	cmr|Glycine betaine-binding periplasmic protein precursor [c_3232]
prpR	-3.00	10.22	-1.59	11.01	0.2	9.43	0.5	0.00732441	0.03230128	0.02771786	0.48219890	A_07_P008344	b0330	cmr|regulator for prp operon [b0330]
prpR	-2.48	11.21	-1.31	11.87	0.3	10.56	0.4	0.01051764	0.04323490	0.04832825	0.58082443	A_07_P031011	c_0450	cmr|Propionate catabolism operon Regulatory protein [c_0450]
psd	+2.93	12.19	+1.55	11.42	0.0	12.97	0.1	0.00023554	0.00236959	0.03441057	0.47109565	A_07_P011531	b4160	cmr|phosphatidylserine decarboxylase; phospholipid synthesis [b4160]
pspB	-8.68	10.15	-3.12	11.71	0.1	8.59	0.4	0.00008713	0.00118364	0.00116489	0.10852071	A_07_P014506	b1305	cmr|phage shock protein [b1305]
pspC	-23.79	10.29	-4.57	12.57	0.0	8.00	0.2	0.00000197	0.00017252	0.00003366	0.01709677	A_07_P014514	b1306	cmr|phage shock protein: activates phage shock-protein expression [b1306]
pspD	-2.57	8.33	-1.36	9.01	0.0	7.65	0.1	0.00057962	0.00452429	0.04654472	0.57474510	A_07_P014520	b1307	cmr|phage shock protein [b1307]
pspE	+9.58	11.88	+3.26	10.25	0.1	13.51	0.1	0.00000951	0.00032326	0.00394436	0.18482143	A_07_P014522	b1308	cmr|phage shock protein [b1308]
pspE	+6.13	11.80	+2.62	10.49	0.1	13.10	0.2	0.00003891	0.00068231	0.00976372	0.27015817	A_07_P014522	b1308	cmr|phage shock protein [b1308]
pspF	+2.64	10.15	+1.40	9.45	0.0	10.85	0.2	0.00108926	0.00736651	0.04114964	0.50574551	A_07_P014500	b1303	cmr|psp operon transcriptional activator [b1303]
pspG	-3.69	9.26	-1.88	10.20	0.2	8.32	0.0	0.00015338	0.00174491	0.01587652	0.39056250	A_07_P058968	ECs5032	cmr|hypothetical protein [ECs5032]
pssA	+3.14	9.35	+1.65	8.52	0.1	10.17	0.0	0.00021753	0.00224136	0.03035760	0.44419145	A_07_P019829	b2585	cmr|phosphatidylserine synthase; phospholipid synthesis [b2585]
pstA	-4.03	10.06	-2.01	11.06	0.1	9.05	0.3	0.00023321	0.00235216	0.01206999	0.34677007	A_07_P005555	b3726	cmr|high-affinity phosphate-specific transport system [b3726]
pstC	-6.55	9.92	-2.71	11.27	0.1	8.56	0.0	0.00001387	0.00039022	0.00279154	0.17509960	A_07_P005556	b3727	cmr|high-affinity phosphate-specific transport system, cytoplasmic membrane component [b3727]
pstS	-7.11	9.10	-2.83	10.52	0.1	7.69	0.3	0.00007297	0.00104221	0.00218051	0.15820276	A_07_P005561	b3728	cmr|high-affinity phosphate-specific transport system; periplasmic phosphate-binding protein [b3728]
pta	+17.38	11.01	+4.12	8.96	0.1	13.07	0.0	0.00000139	0.00015045	0.00115091	0.10122905	A_07_P042552	c_2840	cmr|Phosphate acetyltransferase [c_2840]
pta	+12.13	9.81	+3.60	8.01	0.3	11.61	0.1	0.00001776	0.00043523	0.00243331	0.14456604	A_07_P013771	b2297	cmr|phosphotransacetylase [b2297]
pth	+2.73	10.72	+1.45	9.99	0.1	11.44	0.1	0.00037062	0.00324276	0.03895071	0.49494754	A_07_P006971	b1204	cmr|peptidyl-tRNA hydrolase [b1204]
ptrB	-4.66	8.28	-2.22	9.39	0.1	7.17	0.0	0.00005063	0.00080765	0.00797129	0.29118329	A_07_P015904	b1845	cmr|protease II [b1845]
ptrB	-8.37	9.99	-3.06	11.52	0.0	8.45	0.1	0.00000666	0.00027022	0.00128430	0.11554286	A_07_P035322	c_2256	cmr|Protease II [c_2256]
ptrB	-9.36	10.53	-3.23	12.14	0.2	8.92	0.5	0.00024503	0.00242982	0.00085239	0.09654676	A_07_P052572	Z2896	cmr|protease II [Z2896]
ptsA	+2.25	9.18	+1.17	8.59	0.1	9.76	0.1	0.00216240	0.01231280	0.05595655	0.58771181	A_07_P039491	c_4906	cmr|Phosphoenolpyruvate-protein phosphotransferase ptsA [c_4906]
ptsH	+2.84	11.72	+1.50	10.96	0.0	12.47	0.1	0.00027936	0.00265270	0.03648501	0.48230059	A_07_P013132	b2415	cmr|PTS system protein HPr [b2415]
ptsI	+10.82	13.29	+3.44	11.57	0.1	15.00	0.1	0.00000571	0.00025547	0.00304688	0.16428082	A_07_P013136	b2416	cmr|PEP-protein phosphotransferase system enzyme I [b2416]
ptsI	+10.12	13.77	+3.34	12.10	0.1	15.44	0.0	0.00000553	0.00025298	0.00350927	0.17484177	A_07_P013136	b2416	cmr|PEP-protein phosphotransferase system enzyme I [b2416]
ptsI	+14.03	13.10	+3.81	11.19	0.1	15.00	0.1	0.00000287	0.00018535	0.00184642	0.12806167	A_07_P013136	b2416	cmr|PEP-protein phosphotransferase system enzyme I [b2416]
ptsI	+11.11	13.90	+3.47	12.17	0.1	15.64	0.0	0.00000324	0.00019562	0.00290015	0.15799308	A_07_P013136	b2416	cmr|PEP-protein phosphotransferase system enzyme I [b2416]
ptsI	+10.72	13.87	+3.42	12.16	0.1	15.58	0.3	0.00002993	0.00060018	0.00313707	0.16463333	A_07_P013136	b2416	cmr|PEP-protein phosphotransferase system enzyme I [b2416]
ptsN	+5.55	11.77	+2.47	10.54	0.1	13.01	0.2	0.00006542	0.00096708	0.01158918	0.28733858	A_07_P012498	b3204	cmr|phosphotransferase system enzyme IIA, regulates N metabolism [b3204]
purA	-2.14	12.62	-1.10	13.16	0.1	12.07	0.3	0.00620851	0.02825868	0.07652884	0.67386465	A_07_P011601	b4177	cmr|adenylosuccinate synthetase [b4177]
purC	+8.20	9.68	+3.04	8.16	0.1	11.20	0.0	0.00000758	0.00029299	0.00546494	0.21563910	A_07_P013440	b2476	cmr|phosphoribosylaminoimidazole-succinocarboxamide synthetase = SAICAR synthetase [b2476]
purD	+18.96	10.08	+4.24	7.96	0.5	12.20	0.0	0.00002585	0.00055176	0.00096926	0.09029586	A_07_P008690	b4005	cmr|phosphoribosylglycinamide synthetase = GAR synthetase [b4005]
purF	+2.23	8.40	+1.16	7.82	0.1	8.98	0.1	0.00152214	0.00945346	0.05642213	0.58984728	A_07_P013849	b2312	cmr|amidophosphoribosyltransferase = PRPP amidotransferase [b2312]
purF	+2.93	9.50	+1.55	8.72	0.1	10.28	0.1	0.00035229	0.00313416	0.03434515	0.47060923	A_07_P032426	c_2856	cmr|Amidophosphoribosyltransferase [c_2856]
purH	+7.59	8.39	+2.92	6.93	0.1	9.85	0.2	0.00001684	0.00042624	0.00635607	0.22899314	A_07_P057962	ECs4929	cmr|phosphoribosylaminoimidazolecarboxamideformyltra nsferase [ECs4929]
purH	+24.45	10.00	+4.61	7.69	0.0	12.30	0.3	0.00000328	0.00019644	0.00050559	0.06862069	A_07_P037451	c_4964	cmr|Purine biosynthesis protein PurH [c_4964]
purH	+6.40	8.35	+2.68	7.02	0.1	9.69	0.3	0.00005469	0.00084744	0.00902693	0.26221402	A_07_P047211	Z5583	cmr|phosphoribosylaminoimidazolecarboxamide formyltransferase = AICAR formyltransferase IMP cyclohydrolase [Z5583]
purH	+8.45	8.65	+3.08	7.11	0.1	10.19	0.2	0.00001399	0.00039127	0.00518737	0.21323760	A_07_P008693	b4006	cmr|phosphoribosylaminoimidazolecarboxamide formyltransferase = AICAR formyltransferase; IMP cyclohydrolase [b4006]
purL	+11.11	9.35	+3.47	7.61	0.3	11.08	0.1	0.00002129	0.00049411	0.00287475	0.15825175	A_07_P019686	b2557	cmr|phosphoribosylformyl-glycineamide synthetase = FGAM synthetase [b2557]
purM	+3.43	9.95	+1.78	9.06	0.0	10.84	0.1	0.00011952	0.00146660	0.02579649	0.41442857	A_07_P013551	b2499	cmr|phosphoribosylaminoimidazole synthetase = AIR synthetase [b2499]
purN	+2.46	9.38	+1.30	8.73	0.1	10.04	0.1	0.00107848	0.00730302	0.04668445	0.53846154	A_07_P013559	b2500	cmr|phosphoribosylglycinamide formyltransferase 1 [b2500]
purR	-2.82	8.75	-1.49	9.50	0.0	8.00	0.1	0.00043008	0.00361396	0.03519563	0.51450325	A_07_P018351	b1658	cmr|transcriptional repressor for pur regulon, glyA, glnB, prsA, speA [b1658]
purT	+4.24	8.54	+2.09	7.49	0.0	9.58	0.2	0.00011051	0.00139635	0.01837907	0.36489281	A_07_P015921	b1849	cmr|phosphoribosylglycinamide formyltransferase 2 [b1849]
putA	+18.28	11.27	+4.19	9.17	0.2	13.36	0.2	0.00000600	0.00026402	0.00102515	0.09329480	A_07_P041092	c_1151	cmr|PutA protein [c_1151]
putA	+86.40	10.78	+6.43	7.57	0.2	14.00	0.2	0.00000051	0.00009483	0.00003493	0.01486486	A_07_P001304	b1014	cmr|proline dehydrogenase, P5C dehydrogenase [b1014]
putP	+19.46	10.72	+4.28	8.58	0.5	12.86	0.1	0.00003317	0.00063062	0.00087271	0.08587500	A_07_P001306	b1015	cmr|major sodium/proline symporter [b1015]
pykA	+9.88	10.79	+3.30	9.14	0.0	12.44	0.3	0.00003717	0.00066350	0.00388084	0.18403614	A_07_P015946	b1854	cmr|pyruvate kinase II, glucose stimulated [b1854]
pyrB	+18.04	10.14	+4.17	8.06	0.3	12.23	0.1	0.00000648	0.00027019	0.00096989	0.08982353	A_07_P003778	b4245	cmr|aspartate carbamoyltransferase, catalytic subunit [b4245]
pyrC	+2.98	8.43	+1.57	7.65	0.0	9.22	0.2	0.00053137	0.00424020	0.03360010	0.46731449	A_07_P001513	b1062	cmr|dihydro-orotase [b1062]
pyrD	+4.61	9.54	+2.20	8.43	0.1	10.64	0.1	0.00005263	0.00083108	0.01614901	0.33900000	A_07_P005175	b0945	cmr|dihydro-orotate dehydrogenase [b0945]
pyrG	+4.76	11.48	+2.25	10.36	0.1	12.61	0.1	0.00004282	0.00073125	0.01535442	0.33297521	A_07_P002348	b2780	cmr|CTP synthetase [b2780]
pyrH	-6.52	11.78	-2.70	13.13	0.0	10.43	0.0	0.00001098	0.00034947	0.00280805	0.17405512	A_07_P002814	b0171	cmr|uridylate kinase [b0171]
pyrI	+6.27	9.01	+2.65	7.68	0.1	10.33	0.2	0.00002725	0.00056519	0.00936928	0.26820000	A_07_P003774	b4244	cmr|aspartate carbamoyltransferase, regulatory subunit [b4244]
qor	+10.12	9.46	+3.34	7.79	0.1	11.13	0.1	0.00000506	0.00024271	0.00355246	0.17588050	A_07_P008897	b4051	cmr|quinone oxidoreductase [b4051]
quuD	-2.94	12.44	-1.56	13.22	0.1	11.66	0.2	0.00080723	0.00582985	0.03185976	0.49370079	A_07_P016524	b0551	cmr|orf, hypothetical protein [b0551]
raiA	+46.45	14.19	+5.54	11.42	0.1	16.96	0.0	0.00000038	0.00009117	0.00014418	0.03289855	A_07_P019869	b2597	cmr|putative yhbH sigma 54 modulator [b2597]
rarA	+2.34	10.50	+1.23	9.89	0.1	11.12	0.2	0.00139172	0.00886375	0.05159489	0.56332178	A_07_P004906	b0892	cmr|putative polynucleotide enzyme [b0892]
rarD	-4.46	8.69	-2.16	9.77	0.1	7.61	0.1	0.00005998	0.00090805	0.00902503	0.30361111	A_07_P005965	b3819	cmr|orf, hypothetical protein [b3819]
rbbA	+6.37	9.49	+2.67	8.15	0.1	10.82	0.2	0.00005314	0.00083747	0.00905234	0.26246777	A_07_P007367	b3486	cmr|putative ATP-binding component of a transport system, fragment 1 [b3486]
rbfA	-5.08	12.57	-2.34	13.74	0.0	11.40	0.3	0.00011178	0.00140345	0.00605564	0.24892950	A_07_P000559	b3167	cmr|ribosome-binding factor A [b3167]
rbn	+9.35	9.92	+3.22	8.31	0.1	11.54	0.2	0.00001733	0.00043241	0.00415079	0.19052478	A_07_P039358	c_4833	cmr|Ribonuclease BN [c_4833]
rbsB	+12.71	15.13	+3.67	13.29	0.0	16.96	0.1	0.00000246	0.00017989	0.00228404	0.14326693	A_07_P005677	b3751	cmr|D-ribose periplasmic binding protein [b3751]
rbsC	+21.08	10.95	+4.40	8.75	0.0	13.15	0.3	0.00000488	0.00023837	0.00072218	0.08121429	A_07_P005671	b3750	cmr|D-ribose high-affinity transport system [b3750]
rbsR	+2.19	10.17	+1.13	9.60	0.0	10.73	0.1	0.00119904	0.00792050	0.05795414	0.59988823	A_07_P005686	b3753	cmr|regulator for rbs operon [b3753]
rcsD	+6.77	9.95	+2.76	8.57	0.2	11.33	0.3	0.00007919	0.00110624	0.00803036	0.24887795	A_07_P000870	b2216	cmr|putative 2-component sensor protein [b2216]
rdoA	+3.00	10.52	+1.59	9.73	0.1	11.31	0.2	0.00038589	0.00333080	0.03308880	0.46389136	A_07_P019079	b3859	cmr|orf, hypothetical protein [b3859]
recA	-30.71	13.08	-4.94	15.55	0.0	10.61	0.0	0.00000044	0.00009262	0.00001207	0.00950000	A_07_P001941	b2699	cmr|DNA strand exchange and renaturation, DNA-dependent ATPase, DNA- and ATP-dependent coprotease [b2699]
recF	-5.21	10.91	-2.38	12.10	0.0	9.72	0.0	0.00002396	0.00052980	0.00563580	0.24578947	A_07_P005432	b3700	cmr|ssDNA and dsDNA binding, ATP binding [b3700]
recJ	-2.74	9.18	-1.45	9.90	0.1	8.45	0.0	0.00037116	0.00324276	0.03902503	0.53660262	A_07_P045645	Z4230	cmr|ssDNA exonuclease, 5 --> 3 specific [Z4230]
recJ	-3.16	11.33	-1.66	12.16	0.0	10.50	0.1	0.00022409	0.00229689	0.02527185	0.46535673	A_07_P032859	c_3472	cmr|Single-stranded-DNA-specific exonuclease recJ [c_3472]
recN	-2.98	13.89	-1.57	14.68	0.0	13.11	0.1	0.00020745	0.00216294	0.03044779	0.49065507	A_07_P019956	b2616	cmr|protein used in recombination and DNA repair [b2616]
recR	+3.31	11.18	+1.73	10.32	0.1	12.04	0.3	0.00118954	0.00787482	0.02802973	0.43180039	A_07_P021466	b0472	cmr|recombination and repair [b0472]
recX	-20.77	10.25	-4.38	12.44	0.0	8.06	0.1	0.00000096	0.00012946	0.00004573	0.02057143	A_07_P063405	ECs3555	cmr|regulatory protein OraA [ECs3555]
recX	-23.37	10.42	-4.55	12.69	0.0	8.15	0.2	0.00000210	0.00017779	0.00003684	0.01812500	A_07_P001938	b2698	cmr|regulator, OraA protein [b2698]
recX	-13.48	9.69	-3.75	11.56	0.1	7.81	0.1	0.00000308	0.00019269	0.00024771	0.05270270	A_07_P052735	Z4001	cmr|regulator, OraA protein [Z4001]
relA	+2.37	12.92	+1.24	12.30	0.0	13.54	0.1	0.00070860	0.00528303	0.04950711	0.55279433	A_07_P002368	b2784	cmr|(p)ppGpp synthetase I/GTP pyrophosphokinase [b2784]
rfaF	+2.03	8.97	+1.02	8.46	0.0	9.48	0.1	0.00233025	0.01306025	0.06872142	0.65533010	A_07_P017044	b3620	cmr|ADP-heptose--lps heptosyltransferase II; lipopolysaccharide core biosynthesis [b3620]
rfaG	-2.10	10.94	-1.07	11.47	0.0	10.40	0.0	0.00130884	0.00847300	0.08042746	0.69269694	A_07_P049582	Z5055	cmr|glucosyltransferase I lipopolysaccharide core biosynthesis [Z5055]
rfaG	-2.22	11.56	-1.15	12.13	0.1	10.98	0.2	0.00234815	0.01312861	0.06859248	0.65929182	A_07_P039815	c_4455	cmr|Lipopolysaccharide core biosynthesis protein rfaG [c_4455]
rfaH	-7.83	8.73	-2.97	10.22	0.0	7.25	0.1	0.00000797	0.00029787	0.00159616	0.12887179	A_07_P019019	b3842	cmr|transcriptional activator affecting biosynthesis of lipopolysaccharide core, F pilin, and haemolysin [b3842]
rfaI	-13.88	12.13	-3.79	14.03	0.0	10.23	0.0	0.00000190	0.00016889	0.00020198	0.04818182	A_07_P039805	c_4453	cmr|Lipopolysaccharide 1,3-galactosyltransferase [c_4453]
rfaP	-5.14	11.64	-2.36	12.83	0.0	10.46	0.1	0.00003621	0.00065813	0.00593941	0.24803714	A_07_P039808	c_4454	cmr|Lipopolysaccharide core biosynthesis protein rfaP [c_4454]
rfaP	-5.79	11.93	-2.53	13.20	0.2	10.67	0.0	0.00004039	0.00069965	0.00419334	0.21092652	A_07_P039808	c_4454	cmr|Lipopolysaccharide core biosynthesis protein rfaP [c_4454]
rfaP	-6.51	11.45	-2.70	12.80	0.0	10.09	0.4	0.00018496	0.00199636	0.00287284	0.17531008	A_07_P039808	c_4454	cmr|Lipopolysaccharide core biosynthesis protein rfaP [c_4454]
rfaP	-5.51	11.99	-2.46	13.22	0.0	10.76	0.1	0.00001958	0.00046563	0.00469258	0.22253012	A_07_P039808	c_4454	cmr|Lipopolysaccharide core biosynthesis protein rfaP [c_4454]
rfaP	-5.85	11.75	-2.55	13.02	0.0	10.47	0.3	0.00010580	0.00135201	0.00408346	0.20805825	A_07_P039808	c_4454	cmr|Lipopolysaccharide core biosynthesis protein rfaP [c_4454]
rfaQ	-2.20	10.85	-1.14	11.42	0.0	10.28	0.2	0.00223856	0.01266814	0.06980310	0.66123947	A_07_P039820	c_4456	cmr|Lipopolysaccharide core biosynthesis glycosyl transferase rfaQ [c_4456]
rfaY	-2.23	7.28	-1.16	7.86	0.3	6.70	0.1	0.00604479	0.02768639	0.06820249	0.65876074	A_07_P017067	b3625	cmr|lipopolysaccharide core biosynthesis [b3625]
rffA	+3.22	8.53	+1.69	7.69	0.1	9.38	0.1	0.00026352	0.00255503	0.02943725	0.43888258	A_07_P005832	b3791	cmr|putative regulator [b3791]
rffC	+2.36	7.94	+1.24	7.32	0.4	8.56	0.1	0.00922577	0.03883704	0.05300877	0.57280027	A_07_P005826	b3790	cmr|orf, hypothetical protein [b3790]
rffD	+2.60	7.96	+1.38	7.27	0.1	8.65	0.1	0.00052792	0.00421696	0.04204776	0.51001541	A_07_P005811	b3787	cmr|UDP-N-acetyl-D-mannosaminuronic acid dehydrogenase; synthesis of enterobacterial common antigen [b3787]
rffE	+2.77	9.96	+1.47	9.23	0.1	10.70	0.1	0.00036419	0.00320148	0.03807673	0.49137705	A_07_P005810	b3786	cmr|UDP-N-acetyl glucosamine -2-epimerase; synthesis of enterobacterial common antigen [b3786]
rffG	+2.68	8.08	+1.42	7.37	0.0	8.79	0.2	0.00094398	0.00659868	0.04033283	0.50197628	A_07_P005816	b3788	cmr|dTDP-glucose 4,6-dehydratase [b3788]
rffH	+2.87	7.65	+1.52	6.89	0.0	8.41	0.0	0.00023823	0.00238593	0.03576220	0.47796265	A_07_P005823	b3789	cmr|glucose-1-phosphate thymidylyltransferase [b3789]
rffM	+2.41	7.73	+1.27	7.09	0.1	8.36	0.3	0.00267989	0.01454402	0.04821837	0.54654428	A_07_P005860	b3794	cmr|probable UDP-N-acetyl-D-mannosaminuronic acid transferase; synthesis of enterobacterial common antigen [b3794]
rhaD	-2.12	7.63	-1.08	8.18	0.1	7.09	0.1	0.00158101	0.00974591	0.07754002	0.67821667	A_07_P019286	b3902	cmr|rhamnulose-phosphate aldolase [b3902]
rhaS	+4.22	7.85	+2.08	6.81	0.3	8.89	0.2	0.00055828	0.00439043	0.01842670	0.36400251	A_07_P019305	b3905	cmr|positive regulator for rhaBAD operon [b3905]
rhaS	+2.07	7.48	+1.05	6.96	0.2	8.01	0.2	0.00804459	0.03483332	0.06669017	0.64375843	A_07_P047099	Z5449	cmr|positive regulator for rhaBAD operon [Z5449]
rhlB	+4.36	9.37	+2.13	8.31	0.0	10.44	0.1	0.00005321	0.00083747	0.01762259	0.35616175	A_07_P005782	b3780	cmr|putative ATP-dependent RNA helicase [b3780]
rho	-2.64	10.39	-1.40	11.09	0.1	9.69	0.2	0.00138977	0.00885851	0.04336954	0.56430579	A_07_P005791	b3783	cmr|transcription termination factor Rho; polarity suppressor [b3783]
rhsA	-2.04	7.38	-1.03	7.90	0.1	6.87	0.0	0.00201883	0.01171561	0.08702490	0.71546736	A_07_P016907	b3593	cmr|rhsA protein in rhs element [b3593]
rhsC	-2.20	8.06	-1.14	8.63	0.2	7.49	0.1	0.00326420	0.01702834	0.07148755	0.66558250	A_07_P010017	b0700	cmr|rhsC protein in rhs element [b0700]
ribA	-2.10	10.81	-1.07	11.35	0.1	10.28	0.2	0.00315696	0.01658983	0.08010163	0.69102466	A_07_P058480	ECs1850	cmr|GTP cyclohydrolase II [ECs1850]
ribA	-2.55	8.67	-1.35	9.35	0.2	8.00	0.1	0.00117719	0.00781023	0.04802718	0.57941762	A_07_P014370	b1277	cmr|GTP cyclohydrolase II [b1277]
ribD	-4.25	10.62	-2.09	11.67	0.0	9.58	0.2	0.00012758	0.00153683	0.01031123	0.32146535	A_07_P021185	b0414	cmr|bifunctional pyrimidine deaminase/reductase in pathway of riboflavin synthesis [b0414]
ribE	-5.98	12.61	-2.58	13.90	0.0	11.32	0.0	0.00001457	0.00039551	0.00374301	0.20112628	A_07_P021187	b0415	cmr|riboflavin synthase, beta chain [b0415]
ribE	-5.94	12.76	-2.57	14.04	0.0	11.47	0.1	0.00002228	0.00050974	0.00381733	0.20235690	A_07_P021187	b0415	cmr|riboflavin synthase, beta chain [b0415]
ribE	-6.05	12.55	-2.60	13.85	0.0	11.25	0.1	0.00002149	0.00049767	0.00362233	0.19802083	A_07_P021187	b0415	cmr|riboflavin synthase, beta chain [b0415]
ribE	-6.75	12.39	-2.75	13.77	0.0	11.02	0.1	0.00001206	0.00036323	0.00257558	0.17037815	A_07_P021187	b0415	cmr|riboflavin synthase, beta chain [b0415]
ribE	-6.50	12.50	-2.70	13.85	0.0	11.15	0.1	0.00001666	0.00042622	0.00289761	0.17546154	A_07_P021187	b0415	cmr|riboflavin synthase, beta chain [b0415]
rihB	+2.32	7.96	+1.21	7.35	0.1	8.57	0.1	0.00083389	0.00599213	0.05168636	0.56392931	A_07_P053101	ECs3054	cmr|hypothetical protein [ECs3054]
rihB	+3.14	8.32	+1.65	7.49	0.1	9.14	0.1	0.00019831	0.00209346	0.03036395	0.44387187	A_07_P045486	Z3419	cmr|orf, hypothetical protein [Z3419]
rimI	-6.95	10.86	-2.80	12.26	0.0	9.46	0.2	0.00002324	0.00052262	0.00232025	0.15951965	A_07_P017550	b4373	cmr|acyltransferase for 30S ribosomal subunit protein S18; acetylation of N-terminal alanine [b4373]
rimJ	+10.90	11.29	+3.45	9.56	0.1	13.01	0.1	0.00000548	0.00025298	0.00303290	0.16408935	A_07_P001534	b1066	cmr|acetylation of N-terminal alanine of 30S ribosomal subunit protein S5 [b1066]
rimM	-4.57	16.19	-2.19	17.28	0.0	15.09	0.1	0.00004379	0.00074045	0.00834223	0.29448430	A_07_P019918	b2608	cmr|orf, hypothetical protein [b2608]
rlmN	-2.64	10.59	-1.40	11.29	0.1	9.89	0.1	0.00042940	0.00361139	0.04267975	0.56324392	A_07_P013641	b2517	cmr|orf, hypothetical protein [b2517]
rlpA	+3.23	9.98	+1.69	9.14	0.1	10.83	0.2	0.00029837	0.00278451	0.02898374	0.43834774	A_07_P009735	b0633	cmr|a minor lipoprotein [b0633]
rlpB	-2.34	10.42	-1.23	11.03	0.1	9.80	0.1	0.00094751	0.00661532	0.06024263	0.63188541	A_07_P044023	Z0788	cmr|a minor lipoprotein [Z0788]
rlpB	-2.47	12.64	-1.31	13.30	0.1	11.99	0.1	0.00060532	0.00467850	0.05170224	0.60118168	A_07_P009773	b0641	cmr|a minor lipoprotein [b0641]
rluA	-4.80	9.26	-2.26	10.39	0.2	8.13	0.0	0.00008978	0.00120814	0.00739075	0.28038554	A_07_P010976	b0058	cmr|orf, hypothetical protein [b0058]
rluB	-3.46	9.66	-1.79	10.56	0.0	8.77	0.1	0.00017163	0.00190367	0.01890244	0.41915493	A_07_P014329	b1269	cmr|orf, hypothetical protein [b1269]
rluD	-4.54	10.09	-2.18	11.18	0.3	8.99	0.5	0.00158161	0.00974591	0.00837907	0.29446429	A_07_P042940	Z3888	cmr|suppressor of ftsH mutation [Z3888]
rluD	-3.73	8.18	-1.90	9.13	0.0	7.23	0.3	0.00033801	0.00304380	0.01521596	0.38826580	A_07_P019852	b2594	cmr|suppressor of ftsH mutation [b2594]
rluD	-3.56	10.12	-1.83	11.04	0.2	9.21	0.2	0.00043597	0.00365102	0.01801829	0.40994220	A_07_P053505	ECs3457	cmr|suppressor of ftsH mutation [ECs3457]
rluE	-10.01	10.22	-3.32	11.88	0.0	8.55	0.3	0.00003795	0.00067356	0.00068407	0.08284615	A_07_P006629	b1135	cmr|orf, hypothetical protein [b1135]
rmf	+264.10	12.83	+8.04	8.80	0.1	16.85	0.0	0.00000003	0.00007032	0.00000254	0.00285714	A_07_P005212	b0953	cmr|ribosome modulation factor [b0953]
rnc	-7.85	11.42	-2.97	12.91	0.0	9.94	0.1	0.00001104	0.00034947	0.00159489	0.12943299	A_07_P019740	b2567	cmr|RNase III, ds RNA [b2567]
rnd	-2.15	8.07	-1.11	8.62	0.1	7.51	0.2	0.00295988	0.01574747	0.07422701	0.67162644	A_07_P015715	b1804	cmr|RNase D, processes tRNA precursor [b1804]
rne	+5.92	8.73	+2.56	7.45	0.1	10.01	0.5	0.00069412	0.00520636	0.00984184	0.27041885	A_07_P001621	b1084	cmr|RNase E, membrane attachment, mRNA turnover, maturation 5S RNA [b1084]
rnhB	-5.81	10.92	-2.54	12.18	0.1	9.65	0.1	0.00003273	0.00062948	0.00407711	0.20840909	A_07_P002875	b0183	cmr|RNase HII, degrades RNA of DNA-RNA hybrids [b0183]
rnk	+3.54	9.86	+1.82	8.95	0.2	10.77	0.1	0.00033638	0.00303844	0.02500826	0.40928274	A_07_P016807	b0610	cmr|regulator of nucleoside diphosphate kinase [b0610]
rnpA	-2.89	14.14	-1.53	14.90	0.0	13.37	0.0	0.00024012	0.00239420	0.03331999	0.50248084	A_07_P005448	b3704	cmr|RNase P, protein component; protein C5; processes tRNA, 4.5S RNA [b3704]
rnt	-2.31	8.72	-1.21	9.32	0.1	8.11	0.0	0.00077895	0.00568819	0.06200457	0.63887435	A_07_P018322	b1652	cmr|RNase T, degrades tRNA [b1652]
rob	+4.07	12.14	+2.03	11.12	0.1	13.15	0.1	0.00008172	0.00112858	0.01942200	0.37427173	A_07_P017663	b4396	cmr|right origin-binding protein [b4396]
rpe	+6.63	10.53	+2.73	9.16	0.1	11.89	0.1	0.00002373	0.00052555	0.00832063	0.25436893	A_07_P017959	b3386	cmr|D-ribulose-5-phosphate 3-epimerase [b3386]
rph	-4.70	11.75	-2.23	12.87	0.1	10.64	0.2	0.00008507	0.00116565	0.00780551	0.28645688	A_07_P017160	b3643	cmr|RNase PH [b3643]
rph	-3.47	12.03	-1.80	12.92	0.1	11.13	0.0	0.00010635	0.00135574	0.01924543	0.41966759	A_07_P041332	c_4467	cmr|Ribonuclease PH [c_4467]
rpiR	+15.72	9.67	+3.97	7.68	0.1	11.66	0.4	0.00001674	0.00042622	0.00141578	0.11489691	A_07_P037745	c_5095	cmr|Protein rpiR [c_5095]
rplA	-2.07	14.91	-1.05	15.43	0.1	14.39	0.0	0.00161664	0.00993456	0.08413745	0.70761752	A_07_P008585	b3984	cmr|50S ribosomal subunit protein L1, regulates synthesis of L1 and L11 [b3984]
rplC	-3.03	15.97	-1.60	16.77	0.0	15.17	0.0	0.00017505	0.00192996	0.02907520	0.48491525	A_07_P013027	b3320	cmr|50S ribosomal subunit protein L3 [b3320]
rplD	-2.65	16.73	-1.40	17.44	0.1	16.03	0.0	0.00040639	0.00346600	0.04318153	0.56372305	A_07_P013022	b3319	cmr|50S ribosomal subunit protein L4, regulates expression of S10 operon [b3319]
rplF	-5.46	15.40	-2.45	16.62	0.1	14.17	0.0	0.00002234	0.00051049	0.00476054	0.22240356	A_07_P012955	b3305	cmr|50S ribosomal subunit protein L6 [b3305]

rplI	-12.99	13.86	-3.70	15.71	0.0	12.01	0.1	0.00000295	0.00018751	0.00028900	0.05290698	A_07_P011735	b4203	cmr|50S ribosomal subunit protein L9 [b4203]
rplJ	-2.43	14.66	-1.28	15.30	0.0	14.02	0.1	0.00062185	0.00475805	0.05468686	0.60933475	A_07_P008588	b3985	cmr|50S ribosomal subunit protein L10 [b3985]
rplL	-2.62	15.31	-1.39	16.00	0.0	14.61	0.2	0.00073808	0.00546585	0.04492569	0.57364964	A_07_P008595	b3986	cmr|50S ribosomal subunit protein L7/L12 [b3986]
rplM	-4.97	16.01	-2.31	17.17	0.0	14.86	0.4	0.00026424	0.00255503	0.00643737	0.26188630	A_07_P012630	b3231	cmr|50S ribosomal subunit protein L13 [b3231]
rplO	-2.84	16.24	-1.51	16.99	0.1	15.48	0.2	0.00053780	0.00427318	0.03435595	0.50932203	A_07_P012932	b3301	cmr|50S ribosomal subunit protein L15 [b3301]
rplP	-3.59	15.01	-1.84	15.93	0.1	14.09	0.1	0.00016091	0.00180958	0.01676575	0.39753012	A_07_P012994	b3313	cmr|50S ribosomal subunit protein L16 [b3313]
rplR	-7.84	15.58	-2.97	17.06	0.1	14.09	0.3	0.00003651	0.00065813	0.00159108	0.12979275	A_07_P012950	b3304	cmr|50S ribosomal subunit protein L18 [b3304]
rplS	-57.82	11.28	-5.85	14.21	0.0	8.35	0.2	0.00000037	0.00009117	0.00000064	0.00333333	A_07_P019906	b2606	cmr|50S ribosomal subunit protein L19 [b2606]
rplT	-13.89	12.99	-3.80	14.89	0.1	11.09	0.1	0.00000280	0.00018406	0.00020389	0.04791045	A_07_P018627	b1716	cmr|50S ribosomal subunit protein L20, and regulator [b1716]
rplU	-2.59	14.27	-1.37	14.96	0.0	13.58	0.0	0.00039793	0.00340481	0.04581682	0.57158479	A_07_P012406	b3186	cmr|50S ribosomal subunit protein L21 [b3186]
rplV	-3.33	16.13	-1.74	17.00	0.0	15.26	0.0	0.00011521	0.00143166	0.02175622	0.44600260	A_07_P013002	b3315	cmr|50S ribosomal subunit protein L22 [b3315]
rplW	-2.60	16.19	-1.38	16.88	0.0	15.50	0.1	0.00041717	0.00352927	0.04516324	0.57296535	A_07_P013020	b3318	cmr|50S ribosomal subunit protein L23 [b3318]
rplX	-2.97	14.73	-1.57	15.52	0.0	13.95	0.3	0.00121587	0.00800615	0.03108994	0.49193970	A_07_P012973	b3309	cmr|50S ribosomal subunit protein L24 [b3309]
rplY	-3.56	12.52	-1.83	13.44	0.1	11.61	0.0	0.00011537	0.00143243	0.01761941	0.40674487	A_07_P000722	b2185	cmr|50S ribosomal subunit protein L25 [b2185]
rplY	-3.44	12.33	-1.78	13.22	0.1	11.44	0.0	0.00013328	0.00157770	0.01969195	0.42353825	A_07_P035511	c_2722	cmr|50S ribosomal protein L25 [c_2722]
rpmA	-7.69	15.06	-2.94	16.53	0.1	13.59	0.1	0.00000854	0.00030484	0.00169842	0.13505051	A_07_P012404	b3185	cmr|50S ribosomal subunit protein L27 [b3185]
rpmB	-8.82	13.88	-3.14	15.45	0.0	12.31	0.1	0.00000614	0.00026402	0.00109693	0.10530488	A_07_P017127	b3637	cmr|50S ribosomal subunit protein L28 [b3637]
rpmC	-14.11	15.18	-3.82	17.09	0.0	13.27	0.3	0.00001294	0.00037688	0.00019881	0.04968254	A_07_P012987	b3312	cmr|50S ribosomal subunit protein L29 [b3312]
rpmD	-2.89	15.58	-1.53	16.34	0.0	14.81	0.2	0.00041061	0.00349068	0.03259400	0.49821359	A_07_P012940	b3302	cmr|50S ribosomal subunit protein L30 [b3302]
rpmE	-15.71	14.41	-3.97	16.40	0.0	12.42	0.1	0.00000157	0.00015843	0.00013021	0.03796296	A_07_P019460	b3936	cmr|50S ribosomal subunit protein L31 [b3936]
rpmG	-19.82	12.82	-4.31	14.98	0.0	10.67	0.1	0.00000096	0.00012946	0.00005145	0.02076923	A_07_P017122	b3636	cmr|50S ribosomal subunit protein L33 [b3636]
rpmI	-2.31	13.82	-1.21	14.43	0.0	13.22	0.0	0.00075337	0.00553994	0.06264736	0.64213542	A_07_P018633	b1717	cmr|50S ribosomal subunit protein A [b1717]
rpoA	-6.45	14.36	-2.69	15.70	0.0	13.01	0.2	0.00003704	0.00066202	0.00289888	0.17486590	A_07_P012909	b3295	cmr|RNA polymerase, alpha subunit [b3295]
rpoB	+5.32	11.01	+2.41	9.80	0.0	12.21	0.1	0.00003125	0.00061413	0.01272421	0.29811012	A_07_P008600	b3987	cmr|RNA polymerase, beta subunit [b3987]
rpoC	+2.54	10.66	+1.35	9.99	0.1	11.33	0.3	0.00239874	0.01333065	0.04382431	0.51955572	A_07_P008605	b3988	cmr|RNA polymerase, beta prime subunit [b3988]
rpoD	-9.19	14.00	-3.20	15.60	0.0	12.40	0.3	0.00004291	0.00073189	0.00094131	0.10013514	A_07_P000070	b3067	cmr|RNA polymerase, sigma [b3067]
rpoE	-2.80	12.53	-1.49	13.28	0.0	11.79	0.1	0.00034383	0.00308094	0.03654281	0.52160471	A_07_P019770	b2573	cmr|RNA polymerase, sigma-E factor; heat shock and oxidative stress [b2573]
rpoH	-11.68	10.87	-3.55	12.64	0.0	9.10	0.3	0.00002461	0.00053658	0.00040904	0.06192308	A_07_P007245	b3461	cmr|RNA polymerase, sigma [b3461]
rpoN	+6.82	10.62	+2.77	9.24	0.0	12.01	0.1	0.00001245	0.00036986	0.00803735	0.24860511	A_07_P012490	b3202	cmr|RNA polymerase, sigma [b3202]
rpoZ	-5.77	13.41	-2.53	14.68	0.0	12.15	0.0	0.00001616	0.00041859	0.00414190	0.20900641	A_07_P017187	b3649	cmr|RNA polymerase, omega subunit [b3649]
rppH	-3.51	12.45	-1.81	13.35	0.1	11.54	0.2	0.00030947	0.00286300	0.01818407	0.41015759	A_07_P006135	b2830	cmr|putative invasion protein [b2830]
rpsA	-5.97	13.94	-2.58	15.23	0.0	12.65	0.3	0.00013968	0.00163451	0.00374619	0.20061224	A_07_P005002	b0911	cmr|30S ribosomal subunit protein S1 [b0911]
rpsB	-9.42	13.71	-3.24	15.33	0.0	12.09	0.1	0.00000806	0.00029787	0.00091845	0.10041667	A_07_P002803	b0169	cmr|30S ribosomal subunit protein S2 [b0169]
rpsC	-10.20	15.65	-3.35	17.32	0.0	13.97	0.1	0.00000414	0.00021972	0.00071011	0.08534351	A_07_P012998	b3314	cmr|30S ribosomal subunit protein S3 [b3314]
rpsE	-3.62	16.69	-1.86	17.62	0.0	15.76	0.0	0.00008563	0.00116924	0.01650152	0.39543379	A_07_P012943	b3303	cmr|30S ribosomal subunit protein S5 [b3303]
rpsF	-4.31	15.91	-2.11	16.96	0.1	14.86	0.0	0.00004806	0.00078246	0.00998412	0.31564257	A_07_P011720	b4200	cmr|30S ribosomal subunit protein S6 [b4200]
rpsG	-7.56	15.42	-2.92	16.88	0.0	13.96	0.6	0.00044832	0.00373061	0.00180386	0.13990148	A_07_P017731	b3341	cmr|30S ribosomal subunit protein S7, initiates assembly [b3341]
rpsH	-3.22	15.71	-1.69	16.55	0.1	14.87	0.1	0.00022248	0.00228337	0.02391832	0.45755772	A_07_P012957	b3306	cmr|30S ribosomal subunit protein S8, and regulator [b3306]
rpsI	-20.66	12.29	-4.37	14.47	0.1	10.10	0.0	0.00000102	0.00012946	0.00004700	0.02000000	A_07_P012625	b3230	cmr|30S ribosomal subunit protein S9 [b3230]
rpsK	-2.07	14.70	-1.05	15.23	0.1	14.18	0.1	0.00186577	0.01105145	0.08298971	0.70360258	A_07_P012916	b3297	cmr|30S ribosomal subunit protein S11 [b3297]
rpsL	-3.91	15.89	-1.97	16.88	0.1	14.91	0.0	0.00006683	0.00098312	0.01315549	0.36273205	A_07_P017738	b3342	cmr|30S ribosomal subunit protein S12 [b3342]
rpsN	-2.96	15.72	-1.56	16.50	0.0	14.94	0.2	0.00067059	0.00507827	0.03025407	0.49054583	A_07_P012963	b3307	cmr|30S ribosomal subunit protein S14 [b3307]
rpsP	-3.55	16.51	-1.83	17.42	0.1	15.59	0.1	0.00017124	0.00190249	0.01813326	0.40959828	A_07_P019921	b2609	cmr|30S ribosomal subunit protein S16 [b2609]
rpsQ	-29.87	14.39	-4.90	16.84	0.0	11.94	0.0	0.00000047	0.00009262	0.00001207	0.00904762	A_07_P012981	b3311	cmr|30S ribosomal subunit protein S17 [b3311]
rpsR	-5.09	15.79	-2.35	16.96	0.0	14.62	0.0	0.00002332	0.00052262	0.00599848	0.24918206	A_07_P011729	b4202	cmr|30S ribosomal subunit protein S18 [b4202]
rpsR	-4.70	15.87	-2.23	16.99	0.0	14.75	0.0	0.00003323	0.00063116	0.00779980	0.28691589	A_07_P052364	Z5811	cmr|30S ribosomal subunit protein S18 [Z5811]
rpsT	-17.42	12.57	-4.12	14.64	0.0	10.51	0.0	0.00000114	0.00013852	0.00008257	0.02826087	A_07_P010803	b0023	cmr|30S ribosomal subunit protein S20 [b0023]
rpsU	-2.89	16.73	-1.53	17.49	0.0	15.96	0.1	0.00023851	0.00238617	0.03309197	0.50241080	A_07_P000059	b3065	cmr|30S ribosomal subunit protein S21 [b3065]
rrmJ	-3.46	12.11	-1.79	13.00	0.0	11.21	0.4	0.00199268	0.01159805	0.01890434	0.41860759	A_07_P000609	b3179	cmr|cell division protein [b3179]
rsd	+3.80	10.15	+1.93	9.19	0.1	11.12	0.1	0.00008949	0.00120520	0.02194550	0.39084842	A_07_P008637	b3995	cmr|putative transcriptional regulator [b3995]
rseA	-2.05	14.03	-1.03	14.55	0.0	13.52	0.1	0.00171829	0.01040141	0.08631479	0.71410405	A_07_P019761	b2572	cmr|sigma-E factor, negative regulatory protein [b2572]
rseP	-2.00	12.24	-1.00	12.74	0.0	11.74	0.1	0.00294835	0.01570856	0.08977896	0.72486154	A_07_P002836	b0176	cmr|orf, hypothetical protein [b0176]
rsmE	+2.93	10.92	+1.55	10.14	0.1	11.70	0.3	0.00100717	0.00694263	0.03426702	0.47035745	A_07_P011872	b2946	cmr|orf, hypothetical protein [b2946]
rsuA	-6.08	11.71	-2.60	13.01	0.1	10.41	0.5	0.00035555	0.00315017	0.00361344	0.19822300	A_07_P000715	b2183	cmr|16S pseudouridylate 516 synthase [b2183]
rsxA	-25.42	10.54	-4.67	12.88	0.0	8.21	0.2	0.00000158	0.00015880	0.00002160	0.01259259	A_07_P020768	b1627	cmr|orf, hypothetical protein [b1627]
rsxB	-13.20	9.87	-3.72	11.73	0.0	8.01	0.2	0.00000416	0.00022003	0.00027566	0.05292683	A_07_P020774	b1628	cmr|orf, hypothetical protein [b1628]
rsxC	-4.37	9.70	-2.13	10.76	0.0	8.63	0.1	0.00004644	0.00076475	0.00967162	0.31012220	A_07_P020776	b1629	cmr|putative membrane protein [b1629]
rsxD	-2.41	8.14	-1.27	8.78	0.1	7.51	0.0	0.00078192	0.00570461	0.05484947	0.60985169	A_07_P020785	b1630	cmr|orf, hypothetical protein [b1630]
rumB	-3.90	9.09	-1.96	10.07	0.1	8.11	0.0	0.00007063	0.00101790	0.01334667	0.36544348	A_07_P004749	b0859	cmr|putative enzyme [b0859]
rumB	-3.07	8.94	-1.62	9.75	0.1	8.13	0.2	0.00039685	0.00340023	0.02800813	0.48297919	A_07_P056960	ECs0939	cmr|putative enzyme [ECs0939]
rumB	-3.05	9.20	-1.61	10.00	0.1	8.39	0.2	0.00065310	0.00496738	0.02738758	0.47856826	A_07_P046615	Z1086	cmr|putative enzyme [Z1086]
rusA	+4.22	7.93	+2.08	6.89	0.0	8.96	0.2	0.00017190	0.00190459	0.01837398	0.36525253	A_07_P016520	b0550	cmr|endodeoxyribonuclease RUS [b0550]
rutB	-3.21	10.93	-1.68	11.78	0.0	10.09	0.1	0.00018740	0.00201810	0.02443979	0.45971326	A_07_P001287	b1011	cmr|putative synthetase [b1011]
rutR	-4.52	8.86	-2.18	9.95	0.1	7.77	0.2	0.00012940	0.00155163	0.00860709	0.29980088	A_07_P001300	b1013	cmr|putative tet operon regulator [b1013]
ruvA	-4.75	11.62	-2.25	12.74	0.0	10.49	0.1	0.00005118	0.00081372	0.00735201	0.28026634	A_07_P015985	b1861	cmr|Holliday junction helicase subunit B; branch migration; repair [b1861]
ruvB	-3.72	11.37	-1.90	12.32	0.0	10.42	0.0	0.00007704	0.00108686	0.01514609	0.38773984	A_07_P015980	b1860	cmr|Holliday junction helicase subunit A; branch migration; repair [b1860]
ruvC	-2.41	8.02	-1.27	8.66	0.0	7.39	0.2	0.00185209	0.01099520	0.05439405	0.60822443	A_07_P015994	b1863	cmr|Holliday junction nuclease; resolution of structures; repair [b1863]
ruvC	-3.01	9.01	-1.59	9.80	0.1	8.21	0.3	0.00136882	0.00874373	0.02835556	0.48314935	A_07_P041203	c_2277	cmr|Crossover junction endodeoxyribonuclease ruvC [c_2277]
rzpD	+8.52	10.50	+3.09	8.95	0.4	12.04	0.2	0.00018499	0.00199636	0.00504192	0.21224599	A_07_P010301	b0556	cmr|bacteriophage lambda endopeptidase homolog [b0556]
rzpD	+3.46	8.86	+1.79	7.96	0.2	9.75	0.4	0.00174155	0.01050937	0.02594195	0.41549339	A_07_P016543	b0556	cmr|bacteriophage lambda endopeptidase homolog [b0556]
sad	-2.68	9.31	-1.42	10.02	0.2	8.60	0.1	0.00141014	0.00893412	0.04147929	0.55437182	A_07_P020272	b1525	cmr|putative aldehyde dehydrogenase [b1525]
sapA	-2.36	9.79	-1.24	10.41	0.0	9.17	0.0	0.00065028	0.00494827	0.05896278	0.62512458	A_07_P014455	b1294	cmr|homolog of Salmonella peptide transport periplasmic protein [b1294]
sapD	+2.61	8.27	+1.38	7.58	0.0	8.96	0.0	0.00039170	0.00336992	0.04213034	0.50983859	A_07_P014440	b1291	cmr|putative ATP-binding protein of peptide transport system [b1291]
sat	-12.35	10.36	-3.63	12.17	0.1	8.55	0.1	0.00000277	0.00018406	0.00033791	0.05600000	A_07_P036297	c_3619	cmr|Aecreted auto transpoter toxin [c_3619]
sbcB	+3.68	9.21	+1.88	8.27	0.2	10.15	0.6	0.00643344	0.02911415	0.02362805	0.40129450	A_07_P044967	Z3173	cmr|exonuclease I, 3 --> 5 specific deoxyribophosphodiesterase [Z3173]
sbcB	+4.59	9.73	+2.20	8.63	0.1	10.83	0.3	0.00026372	0.00255503	0.01610645	0.33946452	A_07_P055542	ECs2813	cmr|exonuclease I [ECs2813]
sbcB	+5.92	11.02	+2.57	9.74	0.0	12.30	0.2	0.00003061	0.00060619	0.01044334	0.27773649	A_07_P031971	c_2539	cmr|Exodeoxyribonuclease I [c_2539]
sbcC	+2.55	8.70	+1.35	8.03	0.1	9.38	0.1	0.00071852	0.00534863	0.04365473	0.51793519	A_07_P021099	b0397	cmr|ATP-dependent dsDNA exonuclease [b0397]
sbcC	+2.49	8.73	+1.32	8.07	0.3	9.39	0.1	0.00436749	0.02132808	0.04726690	0.54082122	A_07_P051929	Z0495	cmr|ATP-dependent dsDNA exonuclease [Z0495]
sbcC	+2.33	8.83	+1.22	8.22	0.0	9.44	0.1	0.00081989	0.00590502	0.05126080	0.56162143	A_07_P021099	b0397	cmr|ATP-dependent dsDNA exonuclease [b0397]
sbmA	-2.37	9.40	-1.24	10.03	0.1	8.78	0.1	0.00089127	0.00631227	0.05799352	0.62281719	A_07_P020999	b0377	cmr|sensitivity to microcin B17, possibly envelop protein [b0377]
sbp	-8.96	10.71	-3.16	12.30	0.1	9.13	0.3	0.00002716	0.00056519	0.00101944	0.10354839	A_07_P019364	b3917	cmr|periplasmic sulfate-binding protein [b3917]
sdhA	+19.53	12.16	+4.29	10.01	0.1	14.30	0.1	0.00000135	0.00014976	0.00081618	0.08453947	A_07_P010135	b0723	cmr|succinate dehydrogenase, flavoprotein subunit [b0723]
sdhA	+15.49	10.38	+3.95	8.40	0.2	12.36	0.0	0.00000404	0.00021622	0.00144817	0.11573604	A_07_P051706	Z0877	cmr|succinate dehydrogenase, flavoprotein subunit [Z0877]
sdhA	+13.82	10.46	+3.79	8.57	0.0	12.36	0.1	0.00000234	0.00017963	0.00186230	0.12859649	A_07_P063016	ECs0748	cmr|succinate dehydrogenase flavoprotein subunit [ECs0748]
sdhB	+11.50	12.08	+3.52	10.32	0.1	13.84	0.3	0.00002511	0.00054532	0.00276740	0.15341549	A_07_P010139	b0724	cmr|succinate dehydrogenase, iron sulfur protein [b0724]
sdhC	+63.49	12.80	+5.99	9.81	0.1	15.79	0.2	0.00000078	0.00012461	0.00007431	0.02387755	A_07_P010122	b0721	cmr|succinate dehydrogenase, cytochrome b556 [b0721]
sdhD	+48.28	12.70	+5.59	9.91	0.1	15.50	0.1	0.00000045	0.00009262	0.00013021	0.03059701	A_07_P010127	b0722	cmr|succinate dehydrogenase, hydrophobic subunit [b0722]
sdiA	-3.82	10.19	-1.93	11.16	0.1	9.23	0.1	0.00014188	0.00164851	0.01415841	0.37653716	A_07_P014943	b1916	cmr|transcriptional regulator of ftsQAZ gene cluster [b1916]
sdiA	-3.27	8.45	-1.71	9.31	0.1	7.60	0.0	0.00014761	0.00169545	0.02316502	0.45249380	A_07_P035369	c_2330	cmr|Regulatory protein sdiA [c_2330]
secA	+3.13	9.11	+1.65	8.29	0.0	9.94	0.1	0.00023856	0.00238617	0.03045287	0.44434662	A_07_P002461	b0098	cmr|preprotein translocase; secretion protein [b0098]
secB	+3.99	11.02	+1.99	10.03	0.1	12.02	0.2	0.00023457	0.00236134	0.02038808	0.37942080	A_07_P016988	b3609	cmr|protein export; molecular chaperone; may bind to signel sequence [b3609]
secE	-13.77	12.74	-3.78	14.63	0.0	10.85	0.2	0.00000527	0.00024857	0.00022612	0.05014085	A_07_P008570	b3981	cmr|preprotein translocase [b3981]
secG	-3.17	11.58	-1.67	12.41	0.1	10.75	0.0	0.00018080	0.00196311	0.02525407	0.46557377	A_07_P000590	b3175	cmr|protein export - membrane protein [b3175]
selD	+8.42	10.69	+3.07	9.15	0.0	12.23	0.1	0.00000657	0.00027019	0.00517848	0.21398950	A_07_P018866	b1764	cmr|selenophosphate synthase, H [b1764]
sfaB	+12.61	9.04	+3.66	7.21	0.1	10.87	0.1	0.00000406	0.00021673	0.00225546	0.14204000	A_07_P040481	c_1238	cmr|Putative F1C and S fimbrial switch Regulatory [c_1238]
sfaC	+7.14	8.36	+2.84	6.94	0.1	9.78	0.0	0.00000980	0.00032816	0.00736344	0.24406316	A_07_P040480	c_1237	cmr|Putative F1C and S fimbrial switch Regulatory [c_1237]
sfaD	+7.30	9.24	+2.87	7.81	0.0	10.68	0.3	0.00007073	0.00101790	0.00681974	0.23546053	A_07_P040493	c_1240	cmr|Putative minor F1C fimbrial subunit precursor [c_1240]
sfsA	+2.98	10.81	+1.57	10.02	0.0	11.60	0.4	0.00192798	0.01130507	0.03367696	0.46796999	A_07_P035869	c_0179	cmr|Sugar fermentation stimulation protein A [c_0179]
sgbH	+8.47	8.39	+3.08	6.85	0.1	9.93	0.2	0.00001228	0.00036634	0.00514164	0.21358839	A_07_P016850	b3581	cmr|probable 3-hexulose 6-phosphate synthase [b3581]
sgbU	+7.17	8.48	+2.84	7.06	0.2	9.90	0.1	0.00001941	0.00046369	0.00714939	0.24000000	A_07_P039678	c_4405	cmr|Putative hexulose-6-phosphate isomerase [c_4405]
sgcA	+2.96	8.42	+1.57	7.64	0.3	9.20	0.2	0.00218269	0.01241033	0.03430323	0.47044425	A_07_P004040	b4302	cmr|putative PTS system enzyme II A component [b4302]
sgcA	+3.25	8.33	+1.70	7.48	0.2	9.18	0.0	0.00037769	0.00327983	0.02869157	0.43856311	A_07_P004040	b4302	cmr|putative PTS system enzyme II A component [b4302]
sgcA	+3.21	8.25	+1.68	7.41	0.1	9.10	0.1	0.00018430	0.00199457	0.02909807	0.43839234	A_07_P004040	b4302	cmr|putative PTS system enzyme II A component [b4302]
sgcA	+2.08	8.29	+1.05	7.76	0.2	8.81	0.1	0.00387684	0.01948196	0.06587779	0.64221672	A_07_P004040	b4302	cmr|putative PTS system enzyme II A component [b4302]
sgcA	+2.64	8.39	+1.40	7.69	0.3	9.09	0.2	0.00459047	0.02220852	0.04293191	0.51283763	A_07_P004040	b4302	cmr|putative PTS system enzyme II A component [b4302]
sgcB	+2.84	7.68	+1.51	6.93	0.0	8.44	0.1	0.00035587	0.00315116	0.03642912	0.48196639	A_07_P010684	ABE-0285086	asap|predicted enzyme IIB component of PTS [b4565]
sgcC	+2.26	7.65	+1.18	7.06	0.0	8.24	0.1	0.00119934	0.00792050	0.05398374	0.57896458	A_07_P004046	b4304	cmr|putative PTS system enzyme IIC component [b4304]
sgcE	+2.21	8.20	+1.14	7.63	0.1	8.77	0.2	0.00308437	0.01628119	0.05836954	0.60260328	A_07_P004033	b4301	cmr|putative epimerase [b4301]
sgcX	+7.89	8.23	+2.98	6.74	0.0	9.72	0.2	0.00001485	0.00039976	0.00589812	0.22268585	A_07_P004054	b4305	cmr|putative lyase/synthase [b4305]
sirA	-2.29	10.47	-1.20	11.07	0.1	9.87	0.2	0.00190163	0.01119642	0.06432419	0.64876361	A_07_P007289	b3470	cmr|orf, hypothetical protein [b3470]
sitB	-2.60	8.31	-1.38	9.00	0.1	7.62	0.0	0.00056368	0.00442179	0.04508956	0.57295400	A_07_P038673	c_1599	cmr|SitB protein [c_1599]
sitC	-4.57	8.31	-2.19	9.41	0.2	7.22	0.1	0.00015296	0.00174253	0.00859311	0.29997783	A_07_P038667	c_1598	cmr|SitC protein [c_1598]
skp	+14.75	13.14	+3.88	11.20	0.0	15.08	0.4	0.00002119	0.00049283	0.00167873	0.12408451	A_07_P002847	b0178	cmr|histone-like protein, located in outer membrane or nucleoid [b0178]
slmA	-3.40	11.37	-1.77	12.25	0.2	10.48	0.1	0.00026714	0.00256768	0.02073996	0.43479361	A_07_P017148	b3641	cmr|putative transcriptional regulator [b3641]
slpA	+2.84	10.46	+1.51	9.70	0.5	11.21	0.1	0.00482288	0.02303744	0.03706745	0.48390547	A_07_P051860	Z0033	cmr|probable FKBX-type 16KD peptidyl-prolyl cis-trans isomerase [Z0033]
slt	+4.52	8.60	+2.18	7.51	0.3	9.69	0.2	0.00045930	0.00379654	0.01646596	0.34291005	A_07_P017645	b4392	cmr|soluble lytic murein transglycosylase [b4392]
slt	+4.63	8.40	+2.21	7.29	0.0	9.50	0.1	0.00004523	0.00075578	0.01590003	0.33737197	A_07_P017645	b4392	cmr|soluble lytic murein transglycosylase [b4392]
slt	+2.82	8.52	+1.50	7.77	0.0	9.27	0.3	0.00105343	0.00717044	0.03649644	0.48204698	A_07_P017645	b4392	cmr|soluble lytic murein transglycosylase [b4392]
slt	+3.43	8.37	+1.78	7.48	0.1	9.25	0.4	0.00213394	0.01220812	0.02649581	0.41924623	A_07_P017645	b4392	cmr|soluble lytic murein transglycosylase [b4392]
slt	+4.34	8.57	+2.12	7.51	0.1	9.63	0.1	0.00006997	0.00101347	0.01779345	0.35732143	A_07_P051845	Z5994	cmr|soluble lytic murein transglycosylase [Z5994]
slt	+3.92	8.71	+1.97	7.73	0.1	9.70	0.1	0.00013993	0.00163551	0.02105056	0.38314451	A_07_P017645	b4392	cmr|soluble lytic murein transglycosylase [b4392]
slyD	-3.13	11.28	-1.65	12.10	0.1	10.45	0.1	0.00026114	0.00254676	0.02614266	0.47254879	A_07_P017773	b3349	cmr|FKBP-type peptidyl-prolyl cis-trans isomerase [b3349]
smg	+7.83	9.79	+2.97	8.30	0.1	11.27	0.2	0.00002571	0.00055176	0.00597243	0.22281991	A_07_P034260	c_4045	cmr|Protein smg [c_4045]
smg	+4.58	9.26	+2.19	8.17	0.2	10.36	0.0	0.00006985	0.00101264	0.01624301	0.34006649	A_07_P012853	b3284	cmr|orf, hypothetical protein [b3284]
smg	+4.57	11.44	+2.19	10.34	0.3	12.53	0.2	0.00028876	0.00271092	0.01643610	0.34274172	A_07_P045982	Z4655	cmr|orf, hypothetical protein [Z4655]
smp	+2.22	10.33	+1.15	9.75	0.0	10.91	0.0	0.00091274	0.00644402	0.05585556	0.58743487	A_07_P037364	c_5472	cmr|Protein smp precursor [c_5472]
smpA	-12.22	11.45	-3.61	13.26	0.1	9.65	0.1	0.00000323	0.00019540	0.00035887	0.05824742	A_07_P019965	b2617	cmr|small membrane protein A [b2617]
smpB	-5.13	11.92	-2.36	13.10	0.0	10.74	0.1	0.00003818	0.00067554	0.00593178	0.24837766	A_07_P019980	b2620	cmr|small protein B [b2620]
sodA	+2.11	9.83	+1.08	9.30	0.0	10.37	0.2	0.00235409	0.01314751	0.06318852	0.62805556	A_07_P041367	c_4859	cmr|Superoxide dismutase [c_4859]
sodA	+2.05	8.39	+1.04	7.87	0.3	8.90	0.0	0.00685252	0.03063453	0.06853404	0.65433596	A_07_P019316	b3908	cmr|superoxide dismutase, manganese [b3908]
sodB	+12.35	13.05	+3.63	11.24	0.1	14.87	0.1	0.00000495	0.00023989	0.00237741	0.14396154	A_07_P018342	b1656	cmr|superoxide dismutase, iron [b1656]
sodB	+9.14	13.39	+3.19	11.80	0.0	14.99	0.1	0.00000760	0.00029299	0.00428798	0.19288571	A_07_P042333	c_2050	cmr|Superoxide dismutase [c_2050]
sodC	+2.65	9.37	+1.41	8.67	0.2	10.08	0.0	0.00115138	0.00768106	0.04045605	0.50152756	A_07_P020863	b1646	cmr|superoxide dismutase precursor [b1646]
sohA	-2.33	11.13	-1.22	11.74	0.1	10.52	0.1	0.00112532	0.00755844	0.06059896	0.63267241	A_07_P000370	b3129	cmr|putative protease; htrA suppressor protein [b3129]
soxR	-3.69	8.46	-1.88	9.41	0.1	7.52	0.2	0.00032764	0.00298169	0.01636814	0.39464012	A_07_P008959	b4063	cmr|redox-sensing activator of soxS [b4063]
speB	-3.48	9.24	-1.80	10.14	0.0	8.34	0.0	0.00009606	0.00127307	0.01902312	0.41888112	A_07_P011831	b2937	cmr|agmatinase [b2937]
speD	+2.46	10.23	+1.30	9.58	0.2	10.88	0.1	0.00122205	0.00803675	0.04623095	0.53716605	A_07_P002565	b0120	cmr|S-adenosylmethionine decarboxylase [b0120]
speE	+2.07	11.08	+1.05	10.56	0.0	11.61	0.1	0.00205369	0.01187413	0.06349657	0.63032156	A_07_P002568	b0121	cmr|spermidine synthase = putrescine aminopropyltransferase [b0121]
speF	+31.28	10.03	+4.97	7.55	0.4	12.52	0.2	0.00001184	0.00035909	0.00033092	0.05371134	A_07_P009981	b0693	cmr|ornithine decarboxylase isozyme, inducible [b0693]
speG	-3.29	10.68	-1.72	11.54	0.1	9.82	0.0	0.00018617	0.00200758	0.02261496	0.45069620	A_07_P020551	b1584	cmr|spermidine N1-acetyltransferase [b1584]
srlA	+7.29	8.50	+2.87	7.06	0.0	9.93	0.0	0.00000853	0.00030484	0.00705158	0.23926724	A_07_P001960	b2702	cmr|PTS system, glucitol/sorbitol-specific IIC component, one of two [b2702]
srlA	+7.00	9.01	+2.81	7.61	0.2	10.42	0.2	0.00005698	0.00087614	0.00757304	0.24634298	A_07_P041662	c_3256	cmr|PTS system, glucitol/sorbitol-specific IIC2 component [c_3256]
srlA_1	+5.37	8.01	+2.42	6.80	0.0	9.23	0.1	0.00002662	0.00056107	0.01247459	0.29533835	A_07_P043096	Z4005	cmr|PTS system, glucitol/sorbitol-specific IIC component, one of [Z4005]
srlB	+9.41	8.20	+3.23	6.58	0.2	9.81	0.1	0.00001661	0.00042603	0.00408981	0.18994100	A_07_P001966	b2704	cmr|PTS system, glucitol/sorbitol-specific enzyme IIA component [b2704]
srlE	+6.80	8.01	+2.76	6.63	0.1	9.39	0.3	0.00007400	0.00105418	0.00786903	0.24778000	A_07_P030595	c_3257	cmr|PTS system, glucitol/sorbitol-specific IIBC component [c_3257]
srlE	+8.67	8.39	+3.12	6.83	0.0	9.94	0.2	0.00001405	0.00039160	0.00488631	0.20961853	A_07_P001964	b2703	cmr|PTS system, glucitol/sorbitol-specific IIB component and second of two IIC components [b2703]
srlE_1	+5.89	9.39	+2.56	8.11	0.1	10.67	0.3	0.00009775	0.00128464	0.01055767	0.27657238	A_07_P043105	Z4007	cmr|PTS system, glucitol/sorbitol-specific IIB component and second [Z4007]
srlR	+2.12	10.41	+1.08	9.87	0.1	10.95	0.1	0.00219722	0.01247948	0.06285124	0.62668144	A_07_P041666	c_3261	cmr|Glucitol operon repressor [c_3261]
srlR	+2.02	10.52	+1.01	10.01	0.1	11.02	0.3	0.00818628	0.03535933	0.07242124	0.67228774	A_07_P001981	b2707	cmr|regulator for gut [b2707]
srmB	+2.03	8.91	+1.02	8.40	0.0	9.42	0.1	0.00253197	0.01391879	0.06861662	0.65472727	A_07_P030217	c_3100	cmr|ATP-dependent RNA helicase srmB [c_3100]
ssb	-8.19	12.36	-3.03	13.87	0.0	10.84	0.2	0.00001444	0.00039421	0.00143674	0.12566667	A_07_P008940	b4059	cmr|ssDNA-binding protein [b4059]
sseA	+13.97	11.91	+3.80	10.01	0.1	13.82	0.1	0.00000354	0.00020330	0.00188707	0.12806034	A_07_P013661	b2521	cmr|putative thiosulfate sulfurtransferase [b2521]
sspA	-18.36	12.53	-4.20	14.63	0.0	10.43	0.2	0.00000430	0.00022258	0.00006479	0.02428571	A_07_P012616	b3229	cmr|regulator of transcription; stringent starvation protein A [b3229]
sspB	-11.94	13.38	-3.58	15.17	0.0	11.60	0.1	0.00000385	0.00021100	0.00039507	0.06220000	A_07_P012612	b3228	cmr|stringent starvation protein B [b3228]
sstT	+2.08	8.23	+1.06	7.70	0.1	8.76	0.1	0.00214893	0.01226631	0.06356644	0.63061752	A_07_P000173	b3089	cmr|putative transport protein [b3089]
sthA	+2.32	13.78	+1.22	13.17	0.1	14.39	0.1	0.00131183	0.00848030	0.05274136	0.57069416	A_07_P008514	b3962	cmr|putative oxidoreductase [b3962]
stpA	-7.17	9.80	-2.84	11.22	0.1	8.38	0.1	0.00001581	0.00041177	0.00211700	0.15796209	A_07_P020199	b2669	cmr|DNA-binding protein; H-NS-like protein; chaperone activity; RNA splicing? [b2669]
sucA	+465.07	11.82	+8.86	7.39	0.0	16.25	0.0	0.00000002	0.00007032	0.00000000	0.00000000	A_07_P010149	b0726	cmr|2-oxoglutarate dehydrogenase [b0726]
sucB	+190.56	13.10	+7.57	9.32	0.0	16.89	0.0	0.00000005	0.00007032	0.00000762	0.00666667	A_07_P010151	b0727	cmr|2-oxoglutarate dehydrogenase [b0727]
sucC	+215.93	12.20	+7.75	8.32	0.1	16.07	0.3	0.00000042	0.00009262	0.00000572	0.00600000	A_07_P010159	b0728	cmr|succinyl-CoA synthetase, beta subunit [b0728]
sucD	+112.44	11.35	+6.81	7.94	0.0	14.75	0.1	0.00000009	0.00007032	0.00001588	0.00961538	A_07_P010165	b0729	cmr|succinyl-CoA synthetase, alpha subunit [b0729]
sufS	+2.42	7.58	+1.27	6.95	0.0	8.22	0.1	0.00078968	0.00573466	0.04816502	0.54633285	A_07_P018452	b1680	cmr|orf, hypothetical protein [b1680]
sugE	-4.17	11.68	-2.06	12.71	0.1	10.65	0.1	0.00011609	0.00144033	0.01087525	0.33311284	A_07_P035585	c_5236	cmr|SugE protein [c_5236]
sugE	-3.50	9.51	-1.81	10.42	0.1	8.61	0.0	0.00010302	0.00132941	0.01866425	0.41740057	A_07_P048623	Z5755	cmr|suppresses groEL, may be chaperone [Z5755]
sugE	-3.22	10.43	-1.69	11.27	0.2	9.58	0.0	0.00052132	0.00417053	0.02396913	0.45741818	A_07_P010653	b4148	cmr|suppresses groEL, may be chaperone [b4148]
sugE	-12.70	8.94	-3.67	10.77	0.1	7.11	0.2	0.00000515	0.00024400	0.00031123	0.05444444	A_07_P011472	b4148	cmr|suppresses groEL, may be chaperone [b4148]
surA	+3.68	11.15	+1.88	10.21	0.0	12.09	0.1	0.00009370	0.00124911	0.02320440	0.39970460	A_07_P010951	b0053	cmr|survival protein [b0053]
tadA	-6.05	8.99	-2.60	10.29	0.0	7.69	0.3	0.00014068	0.00164076	0.00356390	0.19826855	A_07_P019700	b2559	cmr|putative deaminase [b2559]
talA	+3.22	8.40	+1.69	7.55	0.3	9.24	0.2	0.00117096	0.00778480	0.02935086	0.43925856	A_07_P013377	b2464	cmr|transaldolase A [b2464]
talC	+3.02	11.07	+1.59	10.27	0.1	11.87	0.1	0.00022003	0.00226266	0.03259654	0.46026906	A_07_P039490	c_4905	cmr|Fructose-6-phosphate aldolase 2 [c_4905]
tatB	-4.03	13.41	-2.01	14.41	0.0	12.40	0.0	0.00005325	0.00083747	0.01205729	0.34703839	A_07_P018999	b3838	cmr|orf, hypothetical protein [b3838]
tatC	-2.01	7.88	-1.00	8.39	0.1	7.38	0.2	0.00515862	0.02435303	0.08762386	0.71851562	A_07_P019003	b3839	cmr|orf, hypothetical protein [b3839]
tdcA	+19.15	10.59	+4.26	8.46	0.2	12.72	0.2	0.00000588	0.00026063	0.00088796	0.08629630	A_07_P000316	b3118	cmr|transcriptional activator of tdc operon [b3118]
tdcB	+45.05	9.87	+5.49	7.12	0.1	12.62	0.1	0.00000037	0.00009117	0.00015117	0.03352113	A_07_P000312	b3117	cmr|threonine dehydratase, catabolic [b3117]
tdcC	+181.02	10.99	+7.50	7.24	0.3	14.74	0.2	0.00000039	0.00009117	0.00000762	0.00631579	A_07_P000306	b3116	cmr|anaerobically inducible L-threonine, L-serine permease [b3116]
tdcD	+51.82	9.12	+5.70	6.27	0.1	11.97	0.3	0.00000191	0.00016889	0.00010925	0.02866667	A_07_P000305	b3115	cmr|putative kinase [b3115]
tdcD	+429.64	11.60	+8.75	7.23	0.0	15.97	0.3	0.00000011	0.00007032	0.00000000	0.00000000	A_07_P045889	Z4467	cmr|putative kinase [Z4467]
tdcE	+30.11	10.81	+4.91	8.35	0.0	13.26	0.0	0.00000048	0.00009267	0.00033600	0.05397959	A_07_P034076	c_3872	cmr|Keto-acid formate acetyltransferase [c_3872]
tdcE	+15.16	10.10	+3.92	8.14	0.1	12.06	0.4	0.00002003	0.00047140	0.00156377	0.12068627	A_07_P000296	b3114	cmr|probable formate acetyltransferase 3 [b3114]
tdcF	+21.40	10.83	+4.42	8.62	0.2	13.04	0.2	0.00000369	0.00020738	0.00066311	0.07849624	A_07_P000294	b3113	cmr|orf, hypothetical protein [b3113]
tdh	+12.11	10.49	+3.60	8.69	0.0	12.29	0.4	0.00003467	0.00064547	0.00234756	0.14437500	A_07_P017024	b3616	cmr|threonine dehydrogenase [b3616]
tehA	+2.08	8.11	+1.05	7.58	0.0	8.64	0.0	0.00148077	0.00925499	0.06400534	0.63297739	A_07_P009237	b1429	cmr|tellurite resistance [b1429]
tehB	+3.81	9.47	+1.93	8.51	0.1	10.44	0.0	0.00010545	0.00135201	0.02160760	0.38746014	A_07_P009242	b1430	cmr|tellurite resistance [b1430]
terB	-2.04	7.50	-1.03	8.01	0.0	6.98	0.1	0.00178732	0.01069540	0.08704459	0.71525574	A_07_P046904	Z1173	cmr|putative phage inhibition, colicin resistance and tellurite [Z1173]
tesB	-2.25	10.19	-1.17	10.77	0.1	9.60	0.1	0.00113553	0.00760757	0.06636623	0.65632538	A_07_P021371	b0452	cmr|acyl-CoA thioesterase II [b0452]
tfaR	-4.60	9.15	-2.20	10.25	0.0	8.05	0.4	0.00048115	0.00391373	0.00834223	0.29514607	A_07_P014841	b1373	cmr|orf, hypothetical protein [b1373]
tgt	+8.03	10.53	+3.01	9.03	0.0	12.04	0.1	0.00001004	0.00033349	0.00572599	0.21987805	A_07_P021144	b0406	cmr|tRNA-guanine transglycosylase [b0406]
thiC	+2.67	8.17	+1.42	7.46	0.1	8.88	0.0	0.00045674	0.00378148	0.03994601	0.50032617	A_07_P063676	ECs4917	cmr|thiamin biosynthesis protein ThiC [ECs4917]
thiD	-6.43	9.88	-2.69	11.22	0.2	8.53	0.4	0.00017484	0.00192910	0.00286649	0.17560311	A_07_P003278	b2103	cmr|phosphomethylpyrimidine kinase [b2103]
thiF	+2.04	7.59	+1.03	7.07	0.0	8.10	0.0	0.00168419	0.01025762	0.06630018	0.64314849	A_07_P008624	b3992	cmr|thiamin biosynthesis, thiazole moiety [b3992]
thiG	+2.26	6.88	+1.17	6.29	0.2	7.47	0.2	0.00354737	0.01817438	0.05496062	0.58308625	A_07_P008618	b3991	cmr|thiamin biosynthesis, thiazole moiety [b3991]
thiG	+2.64	8.10	+1.40	7.40	0.0	8.80	0.3	0.00187273	0.01108015	0.04165714	0.50801704	A_07_P010631	b3991	cmr|thiamin biosynthesis, thiazole moiety [b3991]
thiI	-4.63	10.31	-2.21	11.42	0.1	9.21	0.1	0.00005465	0.00084744	0.00810023	0.29116438	A_07_P021226	b0423	cmr|putative oxidoreductase [b0423]
thiK	+3.21	11.09	+1.68	10.25	0.0	11.93	0.0	0.00014834	0.00170225	0.02905996	0.43823755	A_07_P001731	b1106	cmr|putative beta-glucosidase [b1106]
thiM	-5.49	8.95	-2.46	10.18	0.0	7.72	0.5	0.00056688	0.00444249	0.00491870	0.22446377	A_07_P003284	b2104	cmr|hydoxyethylthiazole kinase [b2104]
thiQ	+2.31	7.39	+1.21	6.79	0.2	8.00	0.0	0.00188047	0.01111606	0.05265053	0.57049553	A_07_P011020	b0066	cmr|putative ATP-binding component of a transport system [b0066]
thrA	+4.92	8.32	+2.30	7.18	0.1	9.47	0.2	0.00010479	0.00134567	0.01437246	0.32279601	A_07_P010704	b0002	cmr|aspartokinase I, homoserine dehydrogenase I [b0002]
thrB	+6.86	8.66	+2.78	7.27	0.2	10.05	0.1	0.00003527	0.00065109	0.00774771	0.24792683	A_07_P010710	b0003	cmr|homoserine kinase [b0003]
thrC	+9.16	10.24	+3.19	8.65	0.0	11.84	0.0	0.00000471	0.00023240	0.00430767	0.19321937	A_07_P035606	c_0005	cmr|Threonine synthase [c_0005]
thyA	+3.46	9.46	+1.79	8.56	0.1	10.35	0.1	0.00017668	0.00193847	0.02568915	0.41397134	A_07_P006119	b2827	cmr|thymidylate synthetase [b2827]
tig	-2.79	13.01	-1.48	13.75	0.0	12.27	0.2	0.00087368	0.00621562	0.03672383	0.52276673	A_07_P021295	b0436	cmr|trigger factor; a molecular chaperone involved in cell division [b0436]
tilS	-2.73	8.68	-1.45	9.41	0.0	7.96	0.1	0.00038974	0.00335855	0.03887513	0.53594571	A_07_P002899	b0188	cmr|cell cycle protein [b0188]
tktA	+12.53	12.04	+3.65	10.22	0.0	13.86	0.0	0.00000231	0.00017963	0.00233359	0.14407843	A_07_P032946	c_3520	cmr|Transketolase 1 [c_3520]
tktA	+4.06	8.35	+2.02	7.34	0.3	9.36	0.2	0.00069041	0.00518102	0.01992315	0.37610312	A_07_P011821	b2935	cmr|transketolase 1 isozyme [b2935]
tldD	+2.89	9.54	+1.53	8.78	0.4	10.31	0.1	0.00223612	0.01265927	0.03537856	0.47647562	A_07_P034184	c_3999	cmr|TldD protein [c_3999]
tnaA	+179.42	10.38	+7.49	6.64	0.2	14.12	0.4	0.00000087	0.00012765	0.00000762	0.00705882	A_07_P057646	ECs4645	cmr|tryptophanase [ECs4645]
tnaA	+109.04	11.95	+6.77	8.56	0.1	15.33	0.0	0.00000013	0.00007343	0.00001778	0.01000000	A_07_P005461	b3708	cmr|tryptophanase [b3708]
tnaA	+214.62	10.80	+7.75	6.93	0.1	14.68	0.0	0.00000007	0.00007032	0.00000635	0.00625000	A_07_P049911	Z5203	cmr|tryptophanase [Z5203]
tnaB	+19.04	9.65	+4.25	7.52	0.3	11.78	0.0	0.00000785	0.00029735	0.00082952	0.08480519	A_07_P041909	c_4632	cmr|Low affinity tryptophan permease [c_4632]
tnaB	+5.90	8.30	+2.56	7.02	0.3	9.58	0.1	0.00010168	0.00132087	0.01028773	0.27592845	A_07_P005466	b3709	cmr|low affinity tryptophan permease [b3709]
tolB	+5.08	10.81	+2.34	9.64	0.2	11.98	0.2	0.00012214	0.00148956	0.01366616	0.31273256	A_07_P010216	b0740	cmr|periplasmic protein involved in the tonb-independent uptake of group A colicins [b0740]
tolC	+3.80	10.58	+1.93	9.61	0.0	11.54	0.1	0.00009694	0.00127929	0.02169207	0.38809091	A_07_P012308	b3035	cmr|outer membrane channel; specific tolerance to colicin E1; segregation of daughter chromosomes [b3035]
tolQ	-2.86	10.00	-1.51	10.76	0.0	9.24	0.0	0.00025487	0.00250325	0.03438389	0.50877820	A_07_P046359	Z0905	cmr|inner membrane protein, membrane-spanning, maintains integrity of [Z0905]
tolQ	-3.06	10.89	-1.61	11.70	0.1	10.08	0.2	0.00034556	0.00309291	0.02829840	0.48269772	A_07_P010205	b0737	cmr|inner membrane protein, membrane-spanning, maintains integrity of cell envelope; tolerance to group A colicins [b0737]
tolR	-2.31	10.85	-1.21	11.45	0.1	10.24	0.2	0.00188172	0.01111664	0.06103976	0.63391161	A_07_P010209	b0738	cmr|putative inner membrane protein, involved in the tonB-independent uptake of group A colicins [b0738]
topB	+2.29	8.75	+1.19	8.15	0.0	9.34	0.3	0.00404054	0.02013275	0.05607660	0.58858000	A_07_P018861	b1763	cmr|DNA topoisomerase III [b1763]
torD	-2.48	7.77	-1.31	8.42	0.0	7.11	0.3	0.00196940	0.01150478	0.05183244	0.60092047	A_07_P061916	ECs1153	cmr|part of trimethylamine-N-oxide oxidoreductase [ECs1153]
torD	-2.22	7.88	-1.15	8.46	0.1	7.31	0.1	0.00142095	0.00899458	0.06790714	0.65914303	A_07_P051271	Z1416	cmr|part of trimethylamine-N-oxide oxidoreductase [Z1416]
torS	-4.70	12.09	-2.23	13.20	0.1	10.97	0.6	0.00256864	0.01410065	0.00798844	0.29113426	A_07_P001197	b0993	cmr|sensor protein torS [b0993]
tpiA	+4.66	13.06	+2.22	11.95	0.0	14.17	0.1	0.00003445	0.00064547	0.01575140	0.33740136	A_07_P019375	b3919	cmr|triosephosphate isomerase [b3919]
tpx	+6.85	10.99	+2.78	9.60	0.0	12.38	0.2	0.00002360	0.00052497	0.00786077	0.24851406	A_07_P014605	b1324	cmr|thiol peroxidase [b1324]
treB	+3.70	8.52	+1.89	7.58	0.1	9.46	0.0	0.00010372	0.00133527	0.02300940	0.39896476	A_07_P003751	b4240	cmr|PTS system enzyme II, trehalose specific [b4240]
treC	+2.17	8.32	+1.12	7.76	0.2	8.87	0.1	0.00269889	0.01463197	0.05975864	0.61172952	A_07_P003746	b4239	cmr|trehalase 6-P hydrolase [b4239]
treR	-2.13	11.82	-1.09	12.36	0.1	11.27	0.1	0.00185054	0.01099016	0.07709794	0.67698271	A_07_P003760	b4241	cmr|repressor of treA,B,C [b4241]
trkH	-2.26	8.99	-1.18	9.58	0.1	8.40	0.2	0.00216784	0.01233929	0.06428608	0.64921103	A_07_P019052	b3849	cmr|potassium uptake, requires TrkE [b3849]
trmA	-4.24	8.89	-2.08	9.93	0.1	7.85	0.1	0.00009164	0.00122584	0.01058943	0.32690196	A_07_P047177	Z5526	cmr|tRNA [Z5526]
trmD	-10.14	15.15	-3.34	16.82	0.0	13.48	0.2	0.00001506	0.00040227	0.00067454	0.08232558	A_07_P019915	b2607	cmr|tRNA methyltransferase; tRNA [b2607]
trmI	+4.94	12.61	+2.31	11.46	0.0	13.76	0.0	0.00002849	0.00057729	0.01440358	0.32257468	A_07_P011943	b2960	cmr|orf, hypothetical protein [b2960]
trmJ	-14.81	13.09	-3.89	15.04	0.0	11.15	0.5	0.00004277	0.00073122	0.00017530	0.04451613	A_07_P019564	b2532	cmr|putative ATP synthase beta subunit [b2532]
trpA	+2.91	10.76	+1.54	9.99	0.1	11.54	0.2	0.00076036	0.00557834	0.03482470	0.47347150	A_07_P007238	b1260	cmr|tryptophan synthase, alpha protein [b1260]
trpB	+99.57	10.97	+6.64	7.65	0.1	14.29	0.1	0.00000012	0.00007248	0.00002795	0.01375000	A_07_P014291	b1261	cmr|tryptophan synthase, beta protein [b1261]
trpB	+119.11	11.76	+6.90	8.31	0.0	15.21	0.1	0.00000009	0.00007032	0.00001270	0.00869565	A_07_P038879	c_1726	cmr|Tryptophan synthase beta chain [c_1726]
trpB	+74.57	11.04	+6.22	7.93	0.1	14.15	0.0	0.00000018	0.00008010	0.00005399	0.02073171	A_07_P044133	Z2550	cmr|tryptophan synthase, beta protein [Z2550]
trpC	+81.31	11.32	+6.35	8.15	0.1	14.50	0.1	0.00000019	0.00008010	0.00004192	0.01692308	A_07_P014296	b1262	cmr|N- [b1262]
trpD	+65.78	11.10	+6.04	8.08	0.3	14.12	0.1	0.00000119	0.00014031	0.00007241	0.02375000	A_07_P038884	c_1729	cmr|Anthranilate synthase component II [c_1729]
trpD	+95.96	10.22	+6.58	6.93	0.1	13.52	0.0	0.00000016	0.00007760	0.00002795	0.01333333	A_07_P014304	b1263	cmr|anthranilate synthase component II, glutamine amidotransferase and phosphoribosylanthranilate transferase [b1263]
trpE	+19.62	9.22	+4.29	7.07	0.3	11.37	0.3	0.00002175	0.00050136	0.00088224	0.08627329	A_07_P038886	c_1730	cmr|Anthranilate synthase component I [c_1730]
trpE	+2.15	10.11	+1.11	9.56	0.3	10.67	0.0	0.00576660	0.02661871	0.06256542	0.62700827	A_07_P014306	b1264	cmr|anthranilate synthase component I [b1264]
trpR	+4.92	9.11	+2.30	7.96	0.0	10.26	0.1	0.00003501	0.00064930	0.01450394	0.32298444	A_07_P017646	b4393	cmr|regulator for trp operon and aroH; trp aporepressor [b4393]
truA	-3.03	9.19	-1.60	9.99	0.1	8.39	0.1	0.00030037	0.00279494	0.02902503	0.48459173	A_07_P063352	ECs3202	cmr|pseudouridylate synthase I [ECs3202]
truA	-3.47	9.25	-1.79	10.15	0.1	8.35	0.4	0.00140965	0.00893412	0.02010290	0.43002717	A_07_P013879	b2318	cmr|pseudouridylate synthase I [b2318]
truA	-3.21	9.24	-1.68	10.08	0.1	8.40	0.1	0.00022670	0.00231589	0.02411141	0.45791315	A_07_P042559	c_2863	cmr|tRNA pseudouridine synthase A [c_2863]
truA	-3.27	9.38	-1.71	10.24	0.0	8.53	0.2	0.00026874	0.00257833	0.02283346	0.45105395	A_07_P052682	Z3580	cmr|pseudouridylate synthase I [Z3580]
truC	-2.78	9.61	-1.47	10.35	0.1	8.87	0.1	0.00049096	0.00397620	0.03724721	0.52640934	A_07_P002405	b2791	cmr|orf, hypothetical protein [b2791]
trxA	-6.36	14.00	-2.67	15.33	0.0	12.66	0.1	0.00001562	0.00041043	0.00310340	0.18507576	A_07_P057729	ECs4714	cmr|thioredoxin 1 [ECs4714]
trxA	-9.91	13.45	-3.31	15.10	0.0	11.79	0.3	0.00001762	0.00043473	0.00071456	0.08522727	A_07_P005789	b3781	cmr|thioredoxin 1 [b3781]
trxA	-6.70	13.97	-2.74	15.35	0.1	12.60	0.2	0.00003673	0.00066007	0.00261560	0.17230126	A_07_P035554	c_4701	cmr|Thioredoxin 1 [c_4701]
trxB	+3.46	12.58	+1.79	11.69	0.1	13.48	0.2	0.00024011	0.00239420	0.02577807	0.41455567	A_07_P033812	c_1025	cmr|Thioredoxin reductase [c_1025]
tsf	-7.32	12.70	-2.87	14.14	0.0	11.26	0.0	0.00000857	0.00030533	0.00202045	0.15293269	A_07_P002806	b0170	cmr|protein chain elongation factor EF-Ts [b0170]
tsgA	-4.76	7.84	-2.25	8.97	0.1	6.72	0.0	0.00003382	0.00063751	0.00747396	0.28286058	A_07_P017847	b3364	cmr|putative transport [b3364]
ttcA	-2.07	8.77	-1.05	9.29	0.3	8.24	0.1	0.00721997	0.03194804	0.08362805	0.70559486	A_07_P014700	b1344	cmr|orf, hypothetical protein [b1344]
ttdR	-3.53	9.80	-1.82	10.71	0.1	8.89	0.3	0.00068967	0.00518045	0.01791032	0.40985465	A_07_P000033	b3060	cmr|putative transcriptional regulator LYSR-type [b3060]
tuf	+3.36	16.55	+1.75	15.67	0.1	17.42	0.1	0.00017170	0.00190367	0.02682292	0.42103689	A_07_P059760	ECs4190	cmr|protein chain elongation factor EF-Tu [ECs4190]
tuf	+3.18	17.00	+1.67	16.17	0.0	17.83	0.0	0.00014154	0.00164696	0.02953570	0.43951796	A_07_P046005	Z4697	cmr|protein chain elongation factor EF-Tu [Z4697]
tufA	+2.85	16.81	+1.51	16.05	0.0	17.56	0.1	0.00025655	0.00251502	0.03594004	0.47911939	A_07_P017725	b3339	cmr|protein chain elongation factor EF-Tu [b3339]
typA	-4.02	10.27	-2.01	11.27	0.1	9.27	0.1	0.00010178	0.00132106	0.01243521	0.34960714	A_07_P019135	b3871	cmr|putative GTP-binding factor [b3871]
tyrA	-2.66	7.83	-1.41	8.53	0.1	7.12	0.1	0.00050239	0.00405418	0.04196265	0.55846154	A_07_P019876	b2600	cmr|chorismate mutase-T and prephenate dehydrogenase [b2600]
tyrB	+2.72	8.18	+1.44	7.46	0.3	8.90	0.2	0.00240375	0.01334436	0.03970719	0.49972022	A_07_P037596	c_5031	cmr|Aromatic-amino-acid aminotransferase [c_5031]
tyrB	+2.44	7.67	+1.29	7.02	0.1	8.31	0.2	0.00180659	0.01078199	0.04776613	0.54455467	A_07_P008914	b4054	cmr|tyrosine aminotransferase, tyrosine repressible [b4054]
tyrS	+3.04	8.29	+1.60	7.49	0.1	9.09	0.1	0.00023970	0.00239420	0.03209858	0.45651310	A_07_P020819	b1637	cmr|tyrosine tRNA synthetase [b1637]
ubiC	+2.02	13.07	+1.01	12.57	0.0	13.58	0.1	0.00246513	0.01360835	0.06934642	0.65849819	A_07_P008838	b4039	cmr|chorismate lyase [b4039]
ubiE	-4.45	13.62	-2.16	14.70	0.0	12.54	0.3	0.00023075	0.00234535	0.00879510	0.30233624	A_07_P018975	b3833	cmr|2-octaprenyl-6-methoxy-1,4-benzoquinone --> 2-octaprenyl-3-methyl-6-methoxy-1,4-benzoquinone [b3833]
ubiF	-2.20	9.18	-1.14	9.75	0.1	8.61	0.0	0.00134066	0.00862580	0.07025152	0.66229940	A_07_P044036	Z0811	cmr|orf, hypothetical protein [Z0811]
ubiH	+3.47	12.11	+1.79	11.21	0.1	13.01	0.0	0.00011076	0.00139667	0.02532203	0.41227508	A_07_P045655	Z4244	cmr|2-octaprenyl-6-methoxyphenol--> 2-octaprenyl-6-methoxy-1, 4-benzoquinone [Z4244]
ubiH	+2.99	12.06	+1.58	11.27	0.0	12.85	0.1	0.00024337	0.00242045	0.03318534	0.46441778	A_07_P032895	c_3489	cmr|2-octaprenyl-6-methoxyphenol hydroxylase [c_3489]

ucpA	+3.75	8.76	+1.91	7.80	0.2	9.71	0.1	0.00014181	0.00164851	0.02248158	0.39503348	A_07_P013188	b2426	cmr|putative oxidoreductase [b2426]
ucpA	+6.06	11.56	+2.60	10.26	0.1	12.86	0.3	0.00011673	0.00144253	0.00998603	0.27153713	A_07_P041240	c_2960	cmr|Oxidoreductase ucpA [c_2960]
ugd	-2.12	8.93	-1.08	9.47	0.1	8.39	0.4	0.01072023	0.04397586	0.07569233	0.67480181	A_07_P042482	c_2555	cmr|UDP-glucose 6-dehydrogenase [c_2555]
ugd	-3.08	8.78	-1.62	9.60	0.1	7.97	0.0	0.00025117	0.00247311	0.02746189	0.47933481	A_07_P044992	Z3190	cmr|UDP-glucose 6-dehydrogenase [Z3190]
ugpB	+2.10	8.16	+1.07	7.62	0.1	8.69	0.1	0.00171688	0.01040141	0.06334350	0.62919874	A_07_P018281	b3453	cmr|sn-glycerol 3-phosphate transport system; periplasmic binding protein [b3453]
ugpC	+5.79	8.77	+2.53	7.50	0.0	10.03	0.1	0.00001709	0.00042882	0.01099276	0.28050243	A_07_P018267	b3450	cmr|ATP-binding component of sn-glycerol 3-phosphate transport system [b3450]
uhpA	-2.14	8.24	-1.10	8.79	0.1	7.69	0.1	0.00168545	0.01026132	0.07547574	0.67439841	A_07_P017284	b3669	cmr|response regulator, positive activator of uhpT transcription [b3669]
uhpB	-2.01	8.03	-1.01	8.53	0.2	7.52	0.1	0.00498562	0.02365876	0.09175686	0.72703573	A_07_P038979	c_4592	cmr|Sensor protein uhpB [c_4592]
uhpB	-2.32	8.60	-1.21	9.21	0.0	7.99	0.1	0.00110634	0.00745321	0.06098006	0.63412814	A_07_P049867	Z5158	cmr|sensor histidine protein kinase phosphorylates UhpA [Z5158]
uhpT	-11.97	8.33	-3.58	10.12	0.0	6.54	0.2	0.00000521	0.00024641	0.00040269	0.06215686	A_07_P017270	b3666	cmr|hexose phosphate transport protein [b3666]
uhpT	-15.24	8.58	-3.93	10.54	0.0	6.61	0.2	0.00000278	0.00018406	0.00013910	0.03910714	A_07_P038972	c_4590	cmr|Hexose phosphate transport protein [c_4590]
ulaA	+3.34	8.00	+1.74	7.13	0.0	8.87	0.2	0.00029862	0.00278521	0.02734566	0.42416749	A_07_P011684	b4193	cmr|orf, hypothetical protein [b4193]
ulaB	+3.63	7.86	+1.86	6.93	0.0	8.79	0.1	0.00009842	0.00129131	0.02348514	0.40059588	A_07_P011689	b4194	cmr|orf, hypothetical protein [b4194]
ulaC	+3.52	8.43	+1.82	7.52	0.2	9.34	0.4	0.00144305	0.00907687	0.02514863	0.41115265	A_07_P011693	b4195	cmr|putative PTS system enzyme II A component [b4195]
ulaD	+4.99	9.86	+2.32	8.70	0.3	11.02	0.2	0.00017418	0.00192438	0.01411014	0.32010086	A_07_P011698	b4196	cmr|probable hexulose-6-phosphate synthase [b4196]
umuC	-7.86	11.07	-2.98	12.56	0.1	9.58	0.2	0.00001924	0.00046180	0.00152630	0.12647368	A_07_P006875	b1184	cmr|SOS mutagenesis and repair [b1184]
umuD	-5.75	11.17	-2.52	12.44	0.1	9.91	0.4	0.00031350	0.00289486	0.00420605	0.21089172	A_07_P006868	b1183	cmr|SOS mutagenesis; error-prone repair; processed to UmuD; forms complex with UmuC [b1183]
ung	-2.35	10.48	-1.23	11.10	0.1	9.87	0.2	0.00213231	0.01220764	0.05773628	0.62132604	A_07_P041614	c_3105	cmr|Uracil-DNA glycosylase [c_3105]
upp	+3.35	11.65	+1.74	10.78	0.2	12.52	0.1	0.00027021	0.00258336	0.02704586	0.42285005	A_07_P013547	b2498	cmr|uracil phosphoribosyltransferase [b2498]
usg	-3.69	10.77	-1.88	11.71	0.0	9.83	0.1	0.00012370	0.00150616	0.01555196	0.39176000	A_07_P032443	c_2864	cmr|USG-1 protein [c_2864]
usg	-3.35	10.18	-1.74	11.06	0.1	9.31	0.0	0.00013122	0.00156505	0.02144627	0.44252949	A_07_P013884	b2319	cmr|putative PTS system enzyme II A component [b2319]
uspA	+18.87	13.09	+4.24	10.97	0.0	15.21	0.1	0.00000154	0.00015822	0.00094385	0.08898204	A_07_P007413	b3495	cmr|universal stress protein; broad regulatory function? [b3495]
uspA	+6.08	11.44	+2.60	10.14	0.0	12.74	0.4	0.00025879	0.00253067	0.00970084	0.26889085	A_07_P049374	Z4895	cmr|universal stress protein broad regulatory function? [Z4895]
uspA	+16.74	12.63	+4.07	10.60	0.1	14.67	0.2	0.00000638	0.00027019	0.00119919	0.10260870	A_07_P034623	c_4293	cmr|Universal stress protein A [c_4293]
uspB	-2.24	8.11	-1.16	8.69	0.0	7.53	0.2	0.00241954	0.01342731	0.06649962	0.65517522	A_07_P007406	b3494	cmr|orf, hypothetical protein [b3494]
uspD	+4.66	9.59	+2.22	8.48	0.0	10.70	0.0	0.00003292	0.00062963	0.01585302	0.33774019	A_07_P019392	b3923	cmr|putative regulator [b3923]
uspE	+2.48	12.53	+1.31	11.87	0.1	13.18	0.1	0.00073767	0.00546535	0.04600356	0.53729970	A_07_P014645	b1333	cmr|orf, hypothetical protein [b1333]
uspF	+75.82	12.40	+6.24	9.28	0.1	15.52	0.2	0.00000030	0.00009047	0.00005399	0.02023810	A_07_P014857	b1376	cmr|putative filament protein [b1376]
uup	-2.10	10.19	-1.07	10.73	0.1	9.66	0.2	0.00301615	0.01597786	0.07801321	0.68084257	A_07_P005192	b0949	cmr|putative ATP-binding component of a transport system [b0949]
uvrA	-11.08	12.89	-3.47	14.63	0.0	11.16	0.0	0.00000298	0.00018867	0.00056212	0.07695652	A_07_P008933	b4058	cmr|excision nuclease subunit A [b4058]
uvrB	-17.33	12.77	-4.12	14.82	0.0	10.71	0.0	0.00000118	0.00014031	0.00008257	0.02708333	A_07_P033475	c_0860	cmr|Excinuclease ABC subunit B [c_0860]
uvrB	-20.36	11.85	-4.35	14.02	0.1	9.68	0.3	0.00000425	0.00022159	0.00004764	0.01973684	A_07_P004347	b0779	cmr|DNA repair; excision nuclease subunit B [b0779]
uvrD	-2.32	12.77	-1.21	13.38	0.1	12.16	0.1	0.00119933	0.00792050	0.06287093	0.64233615	A_07_P005932	b3813	cmr|DNA-dependent ATPase I and helicase II [b3813]
uvrY	-2.60	9.76	-1.38	10.44	0.0	9.07	0.0	0.00038275	0.00331645	0.04535252	0.57168135	A_07_P014931	b1914	cmr|putative 2-component transcriptional regulator [b1914]
uxaA	+9.78	8.54	+3.29	6.89	0.3	10.18	0.4	0.00015651	0.00177268	0.00392785	0.18514970	A_07_P000185	b3091	cmr|altronate hydrolase [b3091]
uxaA	+21.00	10.01	+4.39	7.82	0.1	12.21	0.1	0.00000099	0.00012946	0.00067327	0.07851852	A_07_P034058	c_3849	cmr|Altronate hydrolase [c_3849]
uxaB	+2.02	7.79	+1.01	7.29	0.2	8.30	0.1	0.00330527	0.01719703	0.06900851	0.65647734	A_07_P020252	b1521	cmr|altronate oxidoreductase [b1521]
uxaB	+3.08	10.04	+1.62	9.23	0.0	10.85	0.1	0.00017618	0.00193694	0.03123603	0.45117431	A_07_P052450	Z2184	cmr|altronate oxidoreductase [Z2184]
uxaC	+68.10	10.36	+6.09	7.32	0.0	13.41	0.2	0.00000026	0.00009047	0.00006606	0.02212766	A_07_P000188	b3092	cmr|uronate isomerase [b3092]
uxuA	+2.09	12.04	+1.07	11.51	0.3	12.58	0.1	0.00557238	0.02589481	0.06631479	0.64289409	A_07_P004138	b4322	cmr|mannonate hydrolase [b4322]
uxuB	+2.64	9.59	+1.40	8.89	0.1	10.29	0.1	0.00044247	0.00369035	0.04088669	0.50408771	A_07_P037204	c_5403	cmr|D-mannonate oxidoreductase [c_5403]
uxuB	+5.47	8.43	+2.45	7.20	0.1	9.65	0.0	0.00002361	0.00052497	0.01214050	0.29271057	A_07_P047511	Z5921	cmr|mannonate oxidoreductase [Z5921]
waaD	-2.27	7.57	-1.18	8.16	0.1	6.98	0.3	0.00387920	0.01948761	0.06366489	0.64625403	A_07_P049559	Z5050	cmr|putative LPS biosynthesis enzyme [Z5050]
waaP	-4.45	10.11	-2.15	11.19	0.2	9.04	0.2	0.00013408	0.00158449	0.00928862	0.30658281	A_07_P049579	Z5054	cmr|putative LPS biosynthesis enzyme [Z5054]
waaQ	-2.13	10.92	-1.09	11.46	0.1	10.37	0.0	0.00173052	0.01045887	0.07591019	0.67445260	A_07_P049589	Z5056	cmr|putative LPS biosynthesis enzyme [Z5056]
waaY	-2.23	9.67	-1.16	10.25	0.1	9.09	0.2	0.00304944	0.01612713	0.06923971	0.65788171	A_07_P049567	Z5052	cmr|putative LPS biosynthesis protein [Z5052]
wbdN	-3.95	10.33	-1.98	11.32	0.1	9.34	0.0	0.00006177	0.00092352	0.01288110	0.35704225	A_07_P045051	Z3204	cmr|glycosyl transferase [Z3204]
wrbA	+4.50	9.78	+2.17	8.69	0.1	10.86	0.1	0.00008297	0.00114285	0.01652566	0.34369881	A_07_P001254	b1004	cmr|trp repressor binding protein; affects association of trp repressor and operator [b1004]
wzb	-2.05	8.24	-1.03	8.76	0.1	7.73	0.0	0.00232508	0.01305026	0.08654217	0.71336126	A_07_P003081	b2061	cmr|probable protein-tyrosine-phosphatase [b2061]
wzxE	+2.05	10.27	+1.04	9.75	0.1	10.79	0.0	0.00263587	0.01437301	0.06640307	0.64295818	A_07_P005838	b3792	cmr|putative cytochrome [b3792]
wzyE	+4.92	8.78	+2.30	7.63	0.2	9.93	0.3	0.00019685	0.00208281	0.01440866	0.32223011	A_07_P005852	b3793	cmr|TDP-Fuc4NAc:lipidII transferase; synthesis of enterobacterial common antigen [b3793]
wzzB	-3.44	11.26	-1.78	12.15	0.0	10.37	0.6	0.00636541	0.02886436	0.02029980	0.43189189	A_07_P031985	c_2554	cmr|Chain length determinant protein [c_2554]
wzzB	-2.46	8.99	-1.30	9.64	0.2	8.34	0.3	0.00537504	0.02520089	0.04998857	0.58952809	A_07_P044990	Z3189	cmr|regulator of length of O-antigen component of [Z3189]
xdhA	+11.08	9.88	+3.47	8.15	0.3	11.62	0.3	0.00004725	0.00077251	0.00304688	0.16372014	A_07_P006300	b2866	cmr|orf, hypothetical protein [b2866]
xerC	+4.15	8.47	+2.05	7.44	0.3	9.50	0.1	0.00026409	0.00255503	0.01884718	0.36724010	A_07_P049986	Z5328	cmr|site-specific recombinase, acts on cer sequence of [Z5328]
xerC	+3.40	8.25	+1.77	7.37	0.2	9.14	0.0	0.00020240	0.00212689	0.02665650	0.42094283	A_07_P005923	b3811	cmr|site-specific recombinase, acts on cer sequence of ColE1, effects chromosome segregation at cell division [b3811]
xerC	+3.71	8.73	+1.89	7.78	0.0	9.67	0.1	0.00012431	0.00150779	0.02270706	0.39590255	A_07_P039112	c_4732	cmr|Integrase/recombinase xerC [c_4732]
xerC	+2.55	8.57	+1.35	7.90	0.0	9.25	0.4	0.00673249	0.03023285	0.04620871	0.53730428	A_07_P057731	ECs4741	cmr|site-specific recombinase XerC [ECs4741]
xerD	-4.65	11.57	-2.22	12.68	0.0	10.46	0.0	0.00003287	0.00062948	0.00805069	0.29004577	A_07_P006436	b2894	cmr|site-specific recombinase [b2894]
xisE	-2.79	9.74	-1.48	10.49	0.1	9.00	0.4	0.00260009	0.01422638	0.03663618	0.52199095	A_07_P006656	b1141	cmr|orf, hypothetical protein [b1141]
xisW	-2.01	8.47	-1.01	8.97	0.0	7.96	0.0	0.00175761	0.01058198	0.08947536	0.72463992	A_07_P051290	Z1425	cmr|putative excisionase for bacteriophage BP-933W [Z1425]
xylA	+2.61	8.52	+1.39	7.83	0.1	9.21	0.2	0.00096958	0.00674548	0.04171684	0.50835139	A_07_P007753	b3565	cmr|D-xylose isomerase [b3565]
xylG	+5.69	8.59	+2.51	7.34	0.0	9.85	0.1	0.00002521	0.00054604	0.01131352	0.28363057	A_07_P007761	b3567	cmr|putative ATP-binding protein of xylose transport system [b3567]
xylH	+2.96	8.16	+1.56	7.38	0.0	8.94	0.0	0.00020608	0.00215299	0.03369157	0.46776014	A_07_P007770	b3568	cmr|putative xylose transport, membrane component [b3568]
yaaA	+2.31	9.17	+1.21	8.57	0.1	9.77	0.1	0.00109570	0.00740376	0.05217670	0.56692202	A_07_P010722	b0006	cmr|orf, hypothetical protein [b0006]
yaaH	-3.36	8.34	-1.75	9.21	0.1	7.46	0.1	0.00020627	0.00215353	0.02094322	0.43672848	A_07_P010741	b0010	cmr|orf, hypothetical protein [b0010]
yaaY	-3.78	8.39	-1.92	9.35	0.0	7.44	0.2	0.00027500	0.00261922	0.01441502	0.37951505	A_07_P010806	b0024	cmr|orf, hypothetical protein [b0024]
yabI	-3.71	8.30	-1.89	9.25	0.1	7.36	0.2	0.00034713	0.00310175	0.01625889	0.39381538	A_07_P035723	c_0081	cmr|Hypothetical protein yabI [c_0081]
yabI	-4.62	8.16	-2.21	9.26	0.1	7.05	0.1	0.00003979	0.00069074	0.00826791	0.29383747	A_07_P035723	c_0081	cmr|Hypothetical protein yabI [c_0081]
yabI	-3.61	8.05	-1.85	8.97	0.0	7.12	0.3	0.00037656	0.00327367	0.01681911	0.39759760	A_07_P035723	c_0081	cmr|Hypothetical protein yabI [c_0081]
yabI	-3.35	8.20	-1.74	9.07	0.0	7.33	0.1	0.00013143	0.00156640	0.02128112	0.44085526	A_07_P035723	c_0081	cmr|Hypothetical protein yabI [c_0081]
yabI	-3.45	8.26	-1.79	9.16	0.0	7.37	0.2	0.00019599	0.00207651	0.01916857	0.41973574	A_07_P035723	c_0081	cmr|Hypothetical protein yabI [c_0081]
yacF	-2.65	9.86	-1.41	10.56	0.1	9.16	0.3	0.00265329	0.01444445	0.04274517	0.56269231	A_07_P002479	b0102	cmr|orf, hypothetical protein [b0102]
yacL	+3.26	13.25	+1.70	12.40	0.1	14.11	0.0	0.00019486	0.00207010	0.02871761	0.43853540	A_07_P002559	b0119	cmr|orf, hypothetical protein [b0119]
yadC	-16.14	11.63	-4.01	13.64	0.0	9.63	0.1	0.00000247	0.00017989	0.00011560	0.03568627	A_07_P035826	c_0166	cmr|Hypothetical fimbrial-like protein yadC precursor [c_0166]
yadG	-2.87	9.82	-1.52	10.58	0.0	9.06	0.2	0.00054368	0.00430783	0.03319741	0.50304139	A_07_P002599	b0127	cmr|putative ATP-binding component of a transport system [b0127]
yadH	-2.65	8.53	-1.40	9.23	0.1	7.83	0.1	0.00056348	0.00442179	0.04243140	0.56137815	A_07_P049187	Z0139	cmr|orf, hypothetical protein [Z0139]
yadH	-2.85	8.58	-1.51	9.34	0.0	7.83	0.1	0.00042158	0.00355887	0.03397231	0.50793922	A_07_P002602	b0128	cmr|orf, hypothetical protein [b0128]
yadP	+3.12	11.01	+1.64	10.19	0.3	11.83	0.1	0.00074358	0.00549878	0.03095338	0.44873849	A_07_P035875	c_0180	cmr|2-5 RNA ligase [c_0180]
yadS	-2.08	8.33	-1.06	8.86	0.1	7.80	0.2	0.00562691	0.02612506	0.07898946	0.68480727	A_07_P002749	b0157	cmr|orf, hypothetical protein [b0157]
yaeJ	-2.30	9.47	-1.20	10.07	0.0	8.87	0.3	0.00593083	0.02723892	0.06304560	0.64286917	A_07_P002914	b0191	cmr|orf, hypothetical protein [b0191]
yaeQ	-4.90	9.31	-2.29	10.46	0.1	8.17	0.6	0.00129353	0.00840501	0.00704141	0.27238329	A_07_P002906	b0190	cmr|orf, hypothetical protein [b0190]
yafE	+2.23	7.76	+1.16	7.18	0.0	8.34	0.1	0.00153497	0.00950012	0.05620744	0.58916778	A_07_P041461	c_0247	cmr|Hypothetical protein yafE [c_0247]
yafJ	+3.20	10.71	+1.68	9.87	0.0	11.54	0.1	0.00019186	0.00204384	0.02927083	0.43847764	A_07_P007830	b0223	cmr|putative amidotransferase [b0223]
yafK	-4.56	10.12	-2.19	11.22	0.2	9.03	0.3	0.00030560	0.00283522	0.00866425	0.30112583	A_07_P007831	b0224	cmr|orf, hypothetical protein [b0224]
yafP	-2.87	9.72	-1.52	10.48	0.1	8.96	0.1	0.00031496	0.00290492	0.03413046	0.50789225	A_07_P007881	b0234	cmr|orf, hypothetical protein [b0234]
yafX	-2.70	8.92	-1.43	9.64	0.2	8.20	0.1	0.00116579	0.00776078	0.03972815	0.54295139	A_07_P007946	b0248	cmr|orf, hypothetical protein [b0248]
yahA	-4.70	8.61	-2.23	9.73	0.1	7.50	0.3	0.00019071	0.00203700	0.00777185	0.28655738	A_07_P008268	b0315	cmr|orf, hypothetical protein [b0315]
yahI	+2.42	8.11	+1.27	7.48	0.3	8.75	0.1	0.00367681	0.01867948	0.04925178	0.55150782	A_07_P008306	b0323	cmr|putative kinase [b0323]
yahM	-8.28	9.60	-3.05	11.12	0.1	8.08	0.4	0.00012208	0.00148956	0.00138529	0.12252809	A_07_P008330	b0327	cmr|orf, hypothetical protein [b0327]
yahN	+2.56	8.40	+1.35	7.72	0.3	9.08	0.2	0.00311265	0.01639533	0.04506034	0.53021674	A_07_P008332	b0328	cmr|putative cytochrome subunit of dehydrogenase [b0328]
yaiA	-6.04	12.00	-2.60	13.29	0.1	10.70	0.2	0.00004558	0.00075621	0.00360391	0.19839161	A_07_P021060	b0389	cmr|orf, hypothetical protein [b0389]
yaiE	+2.00	10.61	+1.00	10.11	0.0	11.11	0.1	0.00270593	0.01466008	0.07029027	0.66108124	A_07_P021069	b0391	cmr|orf, hypothetical protein [b0391]
yaiI	+3.57	10.23	+1.84	9.32	0.5	11.15	0.0	0.00280370	0.01506775	0.02491044	0.40981191	A_07_P021047	b0387	cmr|orf, hypothetical protein [b0387]
yajC	+5.81	9.66	+2.54	8.39	0.3	10.93	0.3	0.00025312	0.00248913	0.01057736	0.27662791	A_07_P021148	b0407	cmr|orf, hypothetical protein [b0407]
yajK	-3.21	9.17	-1.68	10.01	0.0	8.33	0.1	0.00015515	0.00176235	0.02410950	0.45842995	A_07_P043862	Z0526	cmr|putative oxidoreductase [Z0526]
ybaA	-2.89	8.16	-1.53	8.92	0.2	7.39	0.3	0.00165493	0.01011055	0.03202363	0.49526523	A_07_P021388	b0456	cmr|orf, hypothetical protein [b0456]
ybaB	+2.04	11.71	+1.03	11.19	0.0	12.22	0.0	0.00166797	0.01017455	0.06646468	0.64315919	A_07_P021461	b0471	cmr|orf, hypothetical protein [b0471]
ybaC	-18.37	10.31	-4.20	12.41	0.1	8.21	0.2	0.00000232	0.00017963	0.00007368	0.02636364	A_07_P031201	c_0596	cmr|Acetyl esterase [c_0596]
ybaL	-3.38	10.40	-1.76	11.28	0.1	9.52	0.1	0.00014082	0.00164105	0.02067518	0.43517380	A_07_P021498	b0478	cmr|putative transport protein [b0478]
ybaQ	-18.22	10.19	-4.19	12.29	0.1	8.10	0.0	0.00000120	0.00014031	0.00007114	0.02604651	A_07_P021524	b0483	cmr|orf, hypothetical protein [b0483]
ybaR	-2.00	9.95	-1.00	10.45	0.2	9.45	0.1	0.00709529	0.03152676	0.08986979	0.72410952	A_07_P031208	c_0604	cmr|Copper-transporting P-type ATPase [c_0604]
ybaV	+3.89	10.54	+1.96	9.56	0.3	11.52	0.1	0.00035221	0.00313416	0.02107533	0.38315242	A_07_P021322	b0442	cmr|orf, hypothetical protein [b0442]
ybaY	-2.58	10.08	-1.37	10.76	0.1	9.39	0.2	0.00110448	0.00745030	0.04505462	0.57297254	A_07_P021380	b0453	cmr|glycoprotein/polysaccharide metabolism [b0453]
ybbB	-2.26	8.50	-1.17	9.08	0.1	7.91	0.1	0.00111118	0.00747627	0.06608867	0.65729627	A_07_P016305	b0503	cmr|putative capsule anchoring protein [b0503]
ybbJ	-2.51	8.97	-1.33	9.63	0.1	8.31	0.4	0.00604585	0.02768639	0.04960937	0.58814006	A_07_P043936	Z0641	cmr|orf, hypothetical protein [Z0641]
ybbJ	-2.65	10.56	-1.41	11.26	0.0	9.86	0.1	0.00051753	0.00415078	0.04243077	0.56184188	A_07_P031218	c_0609	cmr|Hypothetical protein ybbJ [c_0609]
ybbQ	+2.11	7.81	+1.07	7.27	0.1	8.35	0.0	0.00208997	0.01201770	0.06261941	0.62635324	A_07_P031226	c_0624	cmr|2-hydroxy-3-oxopropionate reductase [c_0624]
ybbT	+2.48	8.38	+1.31	7.72	0.3	9.03	0.1	0.00258766	0.01417346	0.04738440	0.54137881	A_07_P031224	c_0620	cmr|Ureidoglycolate hydrolase [c_0620]
ybbW	+3.99	8.64	+2.00	7.64	0.2	9.63	0.0	0.00010224	0.00132269	0.02007812	0.37812201	A_07_P016343	b0511	cmr|putative transport protein [b0511]
ybcJ	-2.52	9.62	-1.33	10.28	0.1	8.95	0.1	0.00060608	0.00468214	0.04882749	0.58326252	A_07_P016429	b0528	cmr|orf, hypothetical protein [b0528]
ybcK	-2.00	7.74	-1.00	8.24	0.2	7.24	0.0	0.00348943	0.01791833	0.08955475	0.72491003	A_07_P016486	b0544	cmr|orf, hypothetical protein [b0544]
ybdD	+8.14	9.03	+3.03	7.52	0.0	10.55	0.1	0.00001024	0.00033697	0.00557800	0.21683951	A_07_P010317	ABE-0285033	asap|conserved protein [b4512]
ybdM	-3.07	7.84	-1.62	8.64	0.1	7.03	0.2	0.00062870	0.00480266	0.02725800	0.47842809	A_07_P016765	b0601	cmr|orf, hypothetical protein [b0601]
ybdN	-6.30	10.54	-2.66	11.87	0.0	9.21	0.2	0.00003467	0.00064547	0.00317645	0.18730337	A_07_P016768	b0602	cmr|orf, hypothetical protein [b0602]
ybeA	-6.69	11.85	-2.74	13.22	0.0	10.47	0.1	0.00001295	0.00037688	0.00265752	0.17433333	A_07_P009747	b0636	cmr|orf, hypothetical protein [b0636]
ybeB	-5.18	13.06	-2.37	14.24	0.0	11.87	0.0	0.00002209	0.00050688	0.00568979	0.24542466	A_07_P009752	b0637	cmr|orf, hypothetical protein [b0637]
ybeJ	+4.71	12.26	+2.23	11.14	0.1	13.38	0.4	0.00035553	0.00315017	0.01542365	0.33401651	A_07_P044035	Z0805	cmr|putative periplasmic binding transport protein [Z0805]
ybeL	+3.49	11.28	+1.80	10.38	0.3	12.18	0.0	0.00054323	0.00430647	0.02565866	0.41390369	A_07_P009784	b0643	cmr|putative alpha helical protein [b0643]
ybeY	+5.55	10.76	+2.47	9.52	0.1	12.00	0.1	0.00002666	0.00056107	0.01184959	0.29150000	A_07_P009856	b0659	cmr|orf, hypothetical protein [b0659]
ybeZ	+5.08	10.88	+2.34	9.71	0.0	12.05	0.1	0.00002837	0.00057633	0.01381479	0.31567489	A_07_P009865	b0660	cmr|putative ATP-binding protein in pho regulon [b0660]
ybfA	-10.45	10.89	-3.39	12.58	0.0	9.19	0.1	0.00000368	0.00020738	0.00066374	0.08228346	A_07_P010014	b0699	cmr|orf, hypothetical protein [b0699]
ybfD	-2.98	8.56	-1.58	9.35	0.1	7.77	0.2	0.00070225	0.00524738	0.03022104	0.49051546	A_07_P010047	b0706	cmr|putative DNA ligase [b0706]
ybfE	-5.59	11.26	-2.48	12.50	0.1	10.02	0.3	0.00011380	0.00141969	0.00465955	0.22230303	A_07_P009941	b0685	cmr|orf, hypothetical protein [b0685]
ybfL	-2.36	10.65	-1.24	11.27	0.4	10.03	0.1	0.00915906	0.03859818	0.05909172	0.62606999	A_07_P010041	b0705	cmr|putative receptor protein [b0705]
ybgA	-4.63	9.69	-2.21	10.80	0.1	8.58	0.3	0.00021131	0.00219159	0.00802401	0.29041379	A_07_P033376	c_0786	cmr|Hypothetical protein ybgA [c_0786]
ybgC	-3.72	10.67	-1.90	11.62	0.1	9.72	0.2	0.00017992	0.00195827	0.01533918	0.39014540	A_07_P010198	b0736	cmr|orf, hypothetical protein [b0736]
ybgE	+6.55	8.89	+2.71	7.54	0.1	10.25	0.1	0.00001672	0.00042622	0.00861662	0.26038388	A_07_P010194	b0735	cmr|orf, hypothetical protein [b0735]
ybgI	+3.38	12.04	+1.76	11.16	0.1	12.92	0.1	0.00019438	0.00206638	0.02707381	0.42286706	A_07_P010066	b0710	cmr|orf, hypothetical protein [b0710]
ybgO	+2.52	7.59	+1.33	6.93	0.1	8.26	0.1	0.00064641	0.00492163	0.04467416	0.52685393	A_07_P010097	b0716	cmr|orf, hypothetical protein [b0716]
ybhC	+2.06	7.82	+1.04	7.30	0.0	8.34	0.1	0.00213545	0.01221232	0.06614710	0.64245527	A_07_P051991	Z0943	cmr|putative pectinesterase [Z0943]
ybhF	+3.19	9.17	+1.67	8.33	0.1	10.00	0.0	0.00018500	0.00199636	0.02939787	0.43954416	A_07_P004422	b0794	cmr|putative ATP-binding component of a transport system [b0794]
ybhG	+2.86	9.71	+1.51	8.95	0.1	10.47	0.0	0.00037308	0.00325597	0.03593623	0.47947458	A_07_P004426	b0795	cmr|putative membrane protein [b0795]
ybhN	-2.88	9.55	-1.53	10.32	0.0	8.79	0.1	0.00028726	0.00270003	0.03331364	0.50334933	A_07_P004392	b0788	cmr|orf, hypothetical protein [b0788]
ybhQ	+2.06	10.30	+1.04	9.78	0.1	10.82	0.3	0.00787293	0.03425964	0.06659870	0.64366483	A_07_P004406	b0791	cmr|orf, hypothetical protein [b0791]
ybhR	+2.57	7.65	+1.36	6.97	0.0	8.33	0.1	0.00044387	0.00369944	0.04294271	0.51257771	A_07_P004412	b0792	cmr|orf, hypothetical protein [b0792]
ybhR	+2.30	8.08	+1.20	7.48	0.3	8.68	0.0	0.00507532	0.02403904	0.05454522	0.58024324	A_07_P046575	Z1012	cmr|orf, hypothetical protein [Z1012]
ybiB	+2.64	11.82	+1.40	11.12	0.1	12.52	0.0	0.00048109	0.00391373	0.04121761	0.50618565	A_07_P004455	b0800	cmr|putative enzyme [b0800]
ybiB	+2.11	9.89	+1.08	9.35	0.1	10.42	0.1	0.00204682	0.01184594	0.06183054	0.62361307	A_07_P046583	Z1021	cmr|putative enzyme [Z1021]
ybiB	+3.91	11.20	+1.97	10.21	0.1	12.18	0.1	0.00012551	0.00151881	0.02074441	0.38243560	A_07_P041584	c_0884	cmr|Hypothetical protein ybiB [c_0884]
ybiC	+6.89	9.26	+2.79	7.86	0.1	10.65	0.2	0.00003038	0.00060429	0.00771850	0.24800000	A_07_P042097	c_0885	cmr|Hypothetical oxidoreductase ybiC [c_0885]
ybiC	+2.76	9.23	+1.47	8.49	0.0	9.96	0.0	0.00029102	0.00272890	0.03841209	0.49408497	A_07_P004457	b0801	cmr|putative dehydrogenase [b0801]
ybiH	+2.82	7.71	+1.50	6.96	0.2	8.46	0.4	0.00644410	0.02914177	0.03749174	0.48702145	A_07_P004432	b0796	cmr|putative transcriptional regulator [b0796]
ybiJ	-10.60	9.25	-3.41	10.95	0.2	7.54	0.3	0.00003112	0.00061325	0.00062309	0.08175000	A_07_P004463	b0802	cmr|orf, hypothetical protein [b0802]
ybiO	-4.95	9.45	-2.31	10.60	0.1	8.30	0.0	0.00003464	0.00064547	0.00646024	0.26146530	A_07_P004491	b0808	cmr|putative transport protein [b0808]
ybiR	-3.57	9.62	-1.84	10.54	0.1	8.70	0.1	0.00011828	0.00145366	0.01769944	0.40739766	A_07_P004542	b0818	cmr|orf, hypothetical protein [b0818]
ybiS	-5.86	9.96	-2.55	11.23	0.0	8.68	0.1	0.00002003	0.00047140	0.00397929	0.20745033	A_07_P004547	b0819	cmr|orf, hypothetical protein [b0819]
ybiS	-7.94	11.46	-2.99	12.96	0.1	9.97	0.1	0.00000818	0.00030062	0.00150661	0.12617021	A_07_P042114	c_0905	cmr|Protein ybiS precursor [c_0905]
ybiT	-13.98	10.33	-3.81	12.23	0.1	8.43	0.2	0.00000536	0.00024927	0.00020071	0.04861538	A_07_P004555	b0820	cmr|putative ATP-binding component of a transport system [b0820]
ybjG	-44.27	10.66	-5.47	13.40	0.0	7.93	0.1	0.00000029	0.00009047	0.00000191	0.00428571	A_07_P004660	b0841	cmr|orf, hypothetical protein [b0841]
ybjL	-13.79	9.98	-3.79	11.87	0.1	8.09	0.1	0.00000319	0.00019528	0.00022104	0.04971429	A_07_P004688	b0847	cmr|putative transport protein [b0847]
ybjM	-4.41	9.01	-2.14	10.08	0.1	7.94	0.2	0.00010556	0.00135201	0.00927782	0.30751579	A_07_P004692	b0848	cmr|orf, hypothetical protein [b0848]
ybjP	+2.75	9.25	+1.46	8.52	0.1	9.99	0.2	0.00074344	0.00549878	0.03845846	0.49387439	A_07_P052022	Z1095	cmr|putative enzyme [Z1095]
ybjT	+7.58	8.55	+2.92	7.09	0.0	10.01	0.1	0.00000850	0.00030484	0.00653582	0.23071749	A_07_P004800	b0869	cmr|putative dTDP-glucose enzyme [b0869]
ybjT	+4.79	8.28	+2.26	7.15	0.1	9.41	0.2	0.00012396	0.00150701	0.01503430	0.33104895	A_07_P052027	Z1103	cmr|putative dTDP-glucose enzyme [Z1103]
ybjX	-9.00	10.25	-3.17	11.83	0.0	8.66	0.1	0.00000618	0.00026501	0.00103404	0.10369427	A_07_P004839	b0877	cmr|putative enzyme [b0877]
ybjX	-10.40	11.93	-3.38	13.62	0.0	10.25	0.2	0.00000898	0.00031671	0.00062500	0.08132231	A_07_P042136	c_1012	cmr|Hypothetical protein ybjX [c_1012]
ycaD	-2.72	8.10	-1.45	8.82	0.1	7.38	0.0	0.00052178	0.00417189	0.03952807	0.54115652	A_07_P033821	c_1037	cmr|Hypothetical protein ycaD [c_1037]
ycaI	-2.17	8.30	-1.11	8.86	0.0	7.74	0.4	0.01129006	0.04567080	0.07058117	0.66302506	A_07_P033841	c_1052	cmr|Hypothetical protein ycaI [c_1052]
ycaQ	-2.39	7.77	-1.26	8.40	0.1	7.14	0.3	0.00365987	0.01863115	0.05503239	0.61102257	A_07_P005026	b0916	cmr|orf, hypothetical protein [b0916]
ycbF	-3.80	11.49	-1.93	12.46	0.1	10.53	0.3	0.00045331	0.00376202	0.01504192	0.38696078	A_07_P005167	b0944	cmr|putative chaperone [b0944]
ycbG	+3.21	9.22	+1.68	8.38	0.0	10.06	0.3	0.00100251	0.00691416	0.02969639	0.43941729	A_07_P005226	b0956	cmr|putative dehydrogenase [b0956]
ycbJ	+4.69	9.51	+2.23	8.39	0.0	10.62	0.1	0.00004903	0.00079170	0.01564914	0.33612551	A_07_P005042	b0919	cmr|orf, hypothetical protein [b0919]
ycbK	+2.36	12.56	+1.24	11.94	0.1	13.18	0.1	0.00090368	0.00638869	0.04984311	0.55340621	A_07_P005076	b0926	cmr|orf, hypothetical protein [b0926]
ycbQ	-4.77	8.51	-2.25	9.64	0.4	7.39	0.3	0.00125779	0.00823393	0.00766832	0.28609005	A_07_P005137	b0938	cmr|putative fimbrial-like protein [b0938]
ycbZ	-2.34	8.48	-1.23	9.10	0.1	7.87	0.4	0.00895165	0.03790606	0.05663554	0.61664592	A_07_P005221	b0955	cmr|putative ATP-dependent protease [b0955]
yccX	-2.10	13.37	-1.07	13.91	0.1	12.84	0.1	0.00169576	0.01030584	0.08035442	0.69282585	A_07_P005289	b0968	cmr|orf, hypothetical protein [b0968]
ycdT	-2.63	11.21	-1.39	11.91	0.1	10.52	0.2	0.00110532	0.00745275	0.04365981	0.56527961	A_07_P041100	c_1164	cmr|Hypothetical protein ycdT [c_1164]
ycdT	-8.68	10.53	-3.12	12.08	0.0	8.97	0.0	0.00000567	0.00025427	0.00112551	0.10739394	A_07_P001352	b1025	cmr|orf, hypothetical protein [b1025]
ycdZ	-10.55	9.21	-3.40	10.91	0.1	7.51	0.1	0.00000567	0.00025427	0.00064405	0.08243902	A_07_P001391	b1036	cmr|orf, hypothetical protein [b1036]
yceD	-2.41	13.61	-1.27	14.24	0.0	12.98	0.1	0.00083166	0.00597887	0.05530234	0.61186226	A_07_P001644	b1088	cmr|orf, hypothetical protein [b1088]
yceF	+2.16	7.68	+1.11	7.13	0.0	8.24	0.1	0.00142226	0.00899643	0.05968369	0.61215635	A_07_P001639	b1087	cmr|orf, hypothetical protein [b1087]
yceH	+3.48	8.62	+1.80	7.72	0.2	9.52	0.1	0.00018831	0.00202485	0.02537792	0.41233230	A_07_P040780	c_1334	cmr|Hypothetical protein yceH [c_1334]
yceH	+3.12	8.62	+1.64	7.80	0.1	9.44	0.0	0.00019037	0.00203528	0.03064405	0.44548476	A_07_P001540	b1067	cmr|orf, hypothetical protein [b1067]
yceH	+2.80	8.28	+1.49	7.54	0.2	9.02	0.1	0.00058868	0.00458487	0.03701156	0.48438071	A_07_P052072	Z1704	cmr|orf, hypothetical protein [Z1704]
yceM	+3.77	10.62	+1.92	9.66	0.0	11.57	0.2	0.00013121	0.00156505	0.02208968	0.39164414	A_07_P001544	b1068	cmr|putative virulence factor [b1068]
ycfD	-2.62	9.67	-1.39	10.37	0.1	8.98	0.0	0.00039731	0.00340147	0.04397866	0.56754098	A_07_P006595	b1128	cmr|orf, hypothetical protein [b1128]
ycfJ	-6.40	9.82	-2.68	11.16	0.1	8.48	0.3	0.00011736	0.00144794	0.00309515	0.18599237	A_07_P001753	b1110	cmr|orf, hypothetical protein [b1110]
ycfL	+4.75	9.76	+2.25	8.63	0.3	10.88	0.5	0.00139864	0.00889583	0.01506733	0.33131285	A_07_P001721	b1104	cmr|orf, hypothetical protein [b1104]
ycfM	+2.37	11.25	+1.24	10.62	0.2	11.87	0.1	0.00203251	0.01178195	0.04968686	0.55362350	A_07_P001730	b1105	cmr|orf, hypothetical protein [b1105]
ycfP	+4.53	14.10	+2.18	13.01	0.1	15.19	0.1	0.00006535	0.00096691	0.01657901	0.34299606	A_07_P001743	b1108	cmr|orf, hypothetical protein [b1108]
ycfQ	-11.50	10.68	-3.52	12.44	0.1	8.92	0.2	0.00000825	0.00030135	0.00043953	0.06528302	A_07_P001759	b1111	cmr|orf, hypothetical protein [b1111]
ycfS	+2.12	8.37	+1.08	7.83	0.0	8.91	0.0	0.00129946	0.00842614	0.06167302	0.62282232	A_07_P001770	b1113	cmr|orf, hypothetical protein [b1113]
ycfZ	-2.33	7.51	-1.22	8.12	0.0	6.90	0.2	0.00167382	0.01020233	0.05976880	0.63027461	A_07_P001806	b1121	cmr|homolog of virulence factor [b1121]
ycgK	+5.35	10.59	+2.42	9.38	0.1	11.80	0.2	0.00006775	0.00099132	0.01240346	0.29543116	A_07_P006843	b1178	cmr|orf, hypothetical protein [b1178]
ycgL	-2.13	11.84	-1.09	12.39	0.0	11.29	0.1	0.00131352	0.00848236	0.07636433	0.67354622	A_07_P006850	b1179	cmr|orf, hypothetical protein [b1179]
ycgM	+2.05	9.05	+1.04	8.53	0.1	9.57	0.1	0.00332156	0.01726467	0.06692772	0.64526026	A_07_P006851	b1180	cmr|putative isomerase [b1180]
ycgN	+2.14	8.27	+1.10	7.73	0.1	8.82	0.1	0.00262684	0.01434015	0.06216781	0.62501277	A_07_P006860	b1181	cmr|orf, hypothetical protein [b1181]
ycgR	-2.17	7.75	-1.12	8.31	0.1	7.19	0.2	0.00298935	0.01588401	0.07333333	0.66969838	A_07_P006924	b1194	cmr|orf, hypothetical protein [b1194]
ycgZ	+5.89	9.42	+2.56	8.14	0.2	10.70	0.1	0.00005568	0.00086111	0.01052973	0.27768844	A_07_P042226	c_1607	cmr|Hypothetical protein ycgZ [c_1607]
ycgZ	-2.03	11.11	-1.02	11.62	0.1	10.60	0.2	0.00496156	0.02359252	0.08880843	0.72258398	A_07_P006771	b1164	cmr|orf, hypothetical protein [b1164]
ychA	+4.48	10.40	+2.16	9.31	0.2	11.48	0.1	0.00009456	0.00125632	0.01680069	0.34531332	A_07_P007024	b1214	cmr|orf, hypothetical protein [b1214]
ychF	-3.14	9.37	-1.65	10.20	0.1	8.55	0.1	0.00031809	0.00292520	0.02601308	0.47128884	A_07_P006968	b1203	cmr|putative GTP-binding protein [b1203]
ychH	+2.43	10.58	+1.28	9.94	0.0	11.22	0.1	0.00075091	0.00552704	0.04768547	0.54442350	A_07_P006978	b1205	cmr|orf, hypothetical protein [b1205]
ychM	-4.95	8.57	-2.31	9.72	0.0	7.41	0.1	0.00003571	0.00065450	0.00655551	0.26262087	A_07_P038791	c_1664	cmr|Putative sulfate transporter ychM [c_1664]
ychM	-2.47	8.93	-1.31	9.59	0.2	8.28	0.1	0.00178119	0.01067090	0.05167619	0.60132299	A_07_P050776	Z1977	cmr|orf, hypothetical protein [Z1977]
ychM	-2.23	8.84	-1.16	9.42	0.4	8.26	0.1	0.00859256	0.03673126	0.06897993	0.65699940	A_07_P006981	b1206	cmr|orf, hypothetical protein [b1206]
ychN	+2.33	9.06	+1.22	8.45	0.3	9.67	0.1	0.00339872	0.01753833	0.05169525	0.56363573	A_07_P007047	b1219	cmr|orf, hypothetical protein [b1219]
ychN	+2.48	9.05	+1.31	8.39	0.2	9.70	0.0	0.00141469	0.00895934	0.04666159	0.53859238	A_07_P050787	Z1994	cmr|orf, hypothetical protein [Z1994]
ychO	-2.77	8.21	-1.47	8.94	0.2	7.48	0.0	0.00101714	0.00699909	0.03706872	0.52577477	A_07_P007051	b1220	cmr|putative factor [b1220]
yciB	-2.38	10.85	-1.25	11.48	0.0	10.22	0.1	0.00086116	0.00614603	0.05696392	0.61851034	A_07_P007207	b1254	cmr|orf, hypothetical protein [b1254]
yciI	+17.30	10.33	+4.11	8.27	0.1	12.39	0.4	0.00002155	0.00049821	0.00113122	0.10005618	A_07_P007191	b1251	cmr|orf, hypothetical protein [b1251]
yciI	+14.20	10.50	+3.83	8.59	0.1	12.42	0.0	0.00000191	0.00016889	0.00177464	0.12529148	A_07_P050796	Z2029	cmr|orf, hypothetical protein [Z2029]
yciK	+5.42	11.45	+2.44	10.23	0.1	12.67	0.1	0.00003307	0.00063062	0.01226435	0.29389650	A_07_P014337	b1271	cmr|putative oxidoreductase [b1271]
yciM	-4.42	11.04	-2.14	12.12	0.4	9.97	0.0	0.00057612	0.00450368	0.00901042	0.30376874	A_07_P014385	b1280	cmr|putative heat shock protein [b1280]
yciO	-3.51	8.60	-1.81	9.50	0.0	7.69	0.2	0.00023209	0.00234886	0.01888338	0.41932299	A_07_P044117	Z2543	cmr|orf, hypothetical protein [Z2543]
yciO	-6.88	10.11	-2.78	11.51	0.0	8.72	0.1	0.00001740	0.00043278	0.00244538	0.16523605	A_07_P038891	c_1732	cmr|Protein yciO [c_1732]
yciO	-4.71	9.26	-2.24	10.38	0.0	8.14	0.2	0.00007159	0.00102646	0.00775343	0.28654930	A_07_P014319	b1267	cmr|orf, hypothetical protein [b1267]
yciQ	-2.10	7.52	-1.07	8.06	0.0	6.99	0.4	0.00991275	0.04113502	0.07590193	0.67476002	A_07_P014324	b1268	cmr|orf, hypothetical protein [b1268]
yciW	-2.91	12.18	-1.54	12.95	0.3	11.41	0.4	0.00386669	0.01943713	0.03240727	0.49729045	A_07_P014418	b1287	cmr|putative oxidoreductase [b1287]
ycjD	-2.09	9.62	-1.06	10.15	0.1	9.09	0.3	0.00838398	0.03601567	0.08260798	0.70225702	A_07_P014428	b1289	cmr|orf, hypothetical protein [b1289]
ycjF	+3.50	7.97	+1.81	7.06	0.3	8.87	0.1	0.00100037	0.00690783	0.02545986	0.41281153	A_07_P042267	c_1794	cmr|Hypothetical protein ycjF [c_1794]
ycjF	+2.37	8.01	+1.24	7.39	0.2	8.64	0.1	0.00236808	0.01320221	0.05057673	0.55683916	A_07_P048458	Z2456	cmr|orf, hypothetical protein [Z2456]
ycjX	+2.61	8.11	+1.39	7.42	0.2	8.81	0.2	0.00210336	0.01208146	0.04264799	0.51177591	A_07_P041136	c_1793	cmr|Hypothetical protein ycjX [c_1793]
ycjX	-2.69	11.65	-1.43	12.36	0.0	10.93	0.0	0.00033039	0.00300324	0.04099911	0.55122792	A_07_P048461	Z2458	cmr|putative EC 2.1 enzymes [Z2458]
ydaE	-3.07	8.80	-1.62	9.61	0.2	7.99	0.5	0.00491990	0.02341563	0.02806275	0.48286339	A_07_P010386	ABE-0285047	asap|Rac prophage; conserved protein [b4526]
ydaL	-2.40	9.73	-1.26	10.36	0.4	9.10	0.2	0.01074830	0.04406802	0.05515561	0.61195913	A_07_P014679	b1340	cmr|orf, hypothetical protein [b1340]
ydaQ	-3.65	9.45	-1.87	10.39	0.1	8.52	0.4	0.00131174	0.00848030	0.01703951	0.39861813	A_07_P048430	Z2414	cmr|unknown protein encoded within prophage CP-933R [Z2414]
ydbH	+2.72	8.11	+1.44	7.39	0.1	8.83	0.2	0.00066263	0.00502528	0.03924162	0.49703942	A_07_P014885	b1381	cmr|orf, hypothetical protein [b1381]
ydbJ	+3.11	10.61	+1.64	9.79	0.1	11.42	0.1	0.00020578	0.00215127	0.03072409	0.44623616	A_07_P010409	ABE-0285050	asap|predicted protein [b4529]
ydbK	+4.71	8.90	+2.24	7.78	0.1	10.02	0.1	0.00005959	0.00090378	0.01564787	0.33655738	A_07_P014866	b1378	cmr|putative oxidoreductase, Fe-S subunit [b1378]
ydcH	+12.58	9.35	+3.65	7.52	0.0	11.18	0.0	0.00000238	0.00017963	0.00223196	0.14112450	A_07_P009222	b1426	cmr|orf, hypothetical protein [b1426]
ydcI	-2.18	8.63	-1.12	9.20	0.1	8.07	0.1	0.00171833	0.01040141	0.07135544	0.66474556	A_07_P009204	b1422	cmr|putative transcriptional regulator LYSR-type [b1422]
ydcM	-2.00	9.17	-1.00	9.67	0.1	8.67	0.0	0.00198967	0.01158911	0.09008321	0.72397652	A_07_P009252	b1432	cmr|putative virulence protein [b1432]
ydcN	-2.09	8.79	-1.06	9.32	0.1	8.26	0.2	0.00464017	0.02238200	0.07936801	0.68657692	A_07_P009265	b1434	cmr|orf, hypothetical protein [b1434]
ydcY	+4.04	11.97	+2.02	10.96	0.0	12.98	0.1	0.00005887	0.00089791	0.01981707	0.37635706	A_07_P009325	b1446	cmr|orf, hypothetical protein [b1446]
yddA	-2.50	7.49	-1.32	8.15	0.3	6.83	0.3	0.00466280	0.02245607	0.04864075	0.58147304	A_07_P009570	b1496	cmr|putative ATP-binding component of a transport system [b1496]
yddV	-2.47	8.15	-1.31	8.80	0.1	7.50	0.3	0.00453243	0.02197680	0.05211128	0.60149560	A_07_P009536	b1490	cmr|orf, hypothetical protein [b1490]
ydeA	-4.00	8.53	-2.00	9.53	0.2	7.53	0.3	0.00047693	0.00389132	0.01267975	0.35521352	A_07_P020286	b1528	cmr|putative resistance / regulatory protein [b1528]
ydeN	+6.50	9.25	+2.70	7.90	0.2	10.60	0.0	0.00002719	0.00056519	0.00884972	0.26042991	A_07_P009578	b1498	cmr|putative sulfatase [b1498]
ydfG	+2.97	8.48	+1.57	7.69	0.0	9.26	0.1	0.00024307	0.00241906	0.03346354	0.46706560	A_07_P020342	b1539	cmr|putative oxidoreductase [b1539]
ydfH	-2.95	8.88	-1.56	9.66	0.1	8.09	0.3	0.00126514	0.00826488	0.03107406	0.49218310	A_07_P052445	Z2157	cmr|orf, hypothetical protein [Z2157]
ydfH	-2.20	9.59	-1.14	10.16	0.1	9.02	0.1	0.00131305	0.00848236	0.07016006	0.66262747	A_07_P020348	b1540	cmr|orf, hypothetical protein [b1540]
ydfM	-7.41	8.77	-2.89	10.21	0.1	7.32	0.3	0.00003819	0.00067554	0.00188453	0.14544118	A_07_P031275	c_0649	cmr|Tail fiber assembly protein homolog from lambdoid [c_0649]
ydfX	-2.56	9.63	-1.36	10.31	0.3	8.95	0.3	0.00484213	0.02310837	0.04523628	0.57250804	A_07_P020485	b1568	cmr|orf, hypothetical protein [b1568]
ydgH	+3.85	9.75	+1.95	8.78	0.2	10.73	0.1	0.00024425	0.00242467	0.02138656	0.38525172	A_07_P020654	b1604	cmr|orf, hypothetical protein [b1604]
ydgJ	+3.68	8.73	+1.88	7.79	0.2	9.66	0.1	0.00021660	0.00223471	0.02321710	0.39948634	A_07_P035111	c_2016	cmr|Hypothetical oxidoreductase ydgJ [c_2016]
ydgK	-5.99	8.74	-2.58	10.03	0.1	7.45	0.2	0.00004661	0.00076682	0.00379573	0.20257627	A_07_P020765	b1626	cmr|orf, hypothetical protein [b1626]
ydhF	+2.69	9.53	+1.43	8.82	0.1	10.25	0.2	0.00088532	0.00628424	0.04027312	0.50162975	A_07_P020869	b1647	cmr|orf, hypothetical protein [b1647]
ydhI	-4.24	8.10	-2.08	9.14	0.2	7.06	0.1	0.00011821	0.00145366	0.01053862	0.32597250	A_07_P020850	b1643	cmr|orf, hypothetical protein [b1643]
ydhJ	-2.17	7.68	-1.12	8.24	0.2	7.12	0.3	0.00954741	0.03989238	0.07384972	0.67207514	A_07_P020852	b1644	cmr|putative membrane protein [b1644]
ydhJ	-2.00	7.76	-1.00	8.26	0.2	7.26	0.2	0.00842513	0.03615297	0.09141514	0.72578921	A_07_P020852	b1644	cmr|putative membrane protein [b1644]
ydhJ	-2.31	8.08	-1.21	8.68	0.1	7.47	0.3	0.00359877	0.01838386	0.05994919	0.63048764	A_07_P020852	b1644	cmr|putative membrane protein [b1644]
ydhJ	-2.20	7.88	-1.14	8.45	0.1	7.31	0.1	0.00140286	0.00890456	0.07032330	0.66257929	A_07_P020852	b1644	cmr|putative membrane protein [b1644]
ydhO	+3.36	8.75	+1.75	7.88	0.0	9.62	0.1	0.00014961	0.00170945	0.02669271	0.42109218	A_07_P018336	b1655	cmr|putative lipoprotein [b1655]
ydhS	-3.30	9.12	-1.72	9.98	0.5	8.26	0.4	0.01112162	0.04515183	0.02240346	0.44761421	A_07_P018392	b1668	cmr|orf, hypothetical protein [b1668]
ydhT	+3.02	8.18	+1.60	7.38	0.2	8.98	0.1	0.00071062	0.00529486	0.03261751	0.46015233	A_07_P018399	b1669	cmr|orf, hypothetical protein [b1669]
ydhU	+5.54	9.03	+2.47	7.79	0.0	10.26	0.0	0.00001980	0.00046811	0.01184959	0.29104524	A_07_P018401	b1670	cmr|orf, hypothetical protein [b1670]
ydhV	+13.96	9.51	+3.80	7.61	0.1	11.41	0.0	0.00000268	0.00018406	0.00186865	0.12847162	A_07_P018419	b1673	cmr|orf, hypothetical protein [b1673]
ydhW	+4.18	8.36	+2.06	7.33	0.1	9.39	0.0	0.00005128	0.00081384	0.01888338	0.36749073	A_07_P018411	b1672	cmr|orf, hypothetical protein [b1672]
ydhX	+8.73	8.66	+3.13	7.10	0.2	10.22	0.1	0.00001131	0.00035103	0.00478214	0.20741047	A_07_P018406	b1671	cmr|putative oxidoreductase, Fe-S subunit [b1671]
ydhY	+4.02	9.63	+2.01	8.62	0.3	10.63	0.0	0.00024011	0.00239420	0.02020897	0.37832342	A_07_P018421	b1674	cmr|putative oxidoreductase, Fe-S subunit [b1674]
ydiA	+8.85	8.70	+3.15	7.12	0.1	10.27	0.1	0.00000607	0.00026402	0.00463859	0.20229917	A_07_P018569	b1703	cmr|orf, hypothetical protein [b1703]
ydiI	+5.24	9.78	+2.39	8.59	0.1	10.98	0.0	0.00003344	0.00063226	0.01309896	0.30372607	A_07_P018484	b1686	cmr|orf, hypothetical protein [b1686]
ydiJ	+4.14	9.76	+2.05	8.73	0.3	10.78	0.4	0.00127891	0.00832378	0.01911585	0.37018450	A_07_P018489	b1687	cmr|putative oxidase [b1687]
ydiT	-3.47	7.88	-1.80	8.78	0.4	6.99	0.2	0.00255231	0.01401589	0.01852642	0.41549858	A_07_P018551	b1700	cmr|orf, hypothetical protein [b1700]
ydiV	-3.16	9.21	-1.66	10.04	0.1	8.38	0.0	0.00017410	0.00192438	0.02560213	0.46761021	A_07_P018587	b1707	cmr|orf, hypothetical protein [b1707]
ydiZ	+2.83	9.79	+1.50	9.04	0.0	10.54	0.0	0.00024834	0.00245286	0.03670033	0.48352301	A_07_P018667	b1724	cmr|orf, hypothetical protein [b1724]
ydjA	+20.05	11.47	+4.33	9.31	0.2	13.63	0.1	0.00000283	0.00018517	0.00074187	0.08225352	A_07_P044447	Z2798	cmr|orf, hypothetical protein [Z2798]
ydjA	+35.52	10.98	+5.15	8.40	0.1	13.55	0.1	0.00000049	0.00009267	0.00024073	0.04621951	A_07_P035242	c_2168	cmr|Protein ydjA [c_2168]
ydjA	+32.65	10.80	+5.03	8.29	0.1	13.32	0.1	0.00000068	0.00011376	0.00025978	0.04647727	A_07_P035242	c_2168	cmr|Protein ydjA [c_2168]
ydjA	+32.08	10.75	+5.00	8.25	0.1	13.25	0.2	0.00000101	0.00012946	0.00026867	0.04752809	A_07_P035242	c_2168	cmr|Protein ydjA [c_2168]
ydjA	+34.57	10.87	+5.11	8.31	0.1	13.42	0.1	0.00000064	0.00010827	0.00025152	0.04658824	A_07_P035242	c_2168	cmr|Protein ydjA [c_2168]
ydjA	+28.68	10.93	+4.84	8.51	0.2	13.35	0.2	0.00000240	0.00017963	0.00036331	0.05663366	A_07_P018872	b1765	cmr|orf, hypothetical protein [b1765]
ydjA	+38.33	11.25	+5.26	8.62	0.1	13.88	0.1	0.00000053	0.00009579	0.00019245	0.03935065	A_07_P035242	c_2168	cmr|Protein ydjA [c_2168]
ydjE	-4.04	8.15	-2.01	9.16	0.1	7.14	0.5	0.00168795	0.01026858	0.01178100	0.34864662	A_07_P041186	c_2173	cmr|Hypothetical metabolite transport protein ydjE [c_2173]

ydjE	-3.38	7.93	-1.76	8.81	0.0	7.05	0.1	0.00015895	0.00179261	0.02101817	0.43771164	A_07_P018893	b1769	cmr|putative transport protein [b1769]
ydjF	-4.40	9.94	-2.14	11.01	0.1	8.87	0.2	0.00010058	0.00131088	0.00932990	0.30730126	A_07_P018898	b1770	cmr|putative DEOR-type transcriptional regulator [b1770]
ydjH	+2.04	7.85	+1.03	7.33	0.0	8.36	0.2	0.00277932	0.01497013	0.06802401	0.65303049	A_07_P018910	b1772	cmr|putative kinase [b1772]
ydjI	+2.09	7.90	+1.07	7.37	0.1	8.43	0.0	0.00178231	0.01067351	0.06291413	0.62651486	A_07_P018915	b1773	cmr|putative aldolase [b1773]
ydjM	-4.80	8.27	-2.26	9.40	0.1	7.14	0.1	0.00003967	0.00068943	0.00718941	0.27607317	A_07_P018690	b1728	cmr|orf, hypothetical protein [b1728]
ydjN	-14.67	9.96	-3.88	11.90	0.0	8.02	0.1	0.00000244	0.00017989	0.00017340	0.04475410	A_07_P018691	b1729	cmr|part of a kinase [b1729]
ydjN	-7.34	9.33	-2.88	10.76	0.0	7.89	0.2	0.00002225	0.00050974	0.00195312	0.15000000	A_07_P042351	c_2128	cmr|Hypothetical symporter ydjN [c_2128]
ydjR	-2.24	7.71	-1.16	8.30	0.0	7.13	0.1	0.00102437	0.00703346	0.06851880	0.65898595	A_07_P041169	c_2141	cmr|Hypothetical protein ydjR [c_2141]
ydjY	+2.88	8.69	+1.53	7.92	0.4	9.45	0.3	0.00668506	0.03005413	0.03676448	0.48355890	A_07_P018802	b1751	cmr|orf, hypothetical protein [b1751]
yeaC	+2.67	12.15	+1.42	11.44	0.1	12.86	0.1	0.00057230	0.00447604	0.04046684	0.50126672	A_07_P015578	b1777	cmr|orf, hypothetical protein [b1777]
yeaD	+6.39	9.49	+2.68	8.16	0.2	10.83	0.3	0.00009624	0.00127430	0.00880907	0.25971910	A_07_P035263	c_2185	cmr|Unknown protein from 2D-page [c_2185]
yeaD	+6.55	9.61	+2.71	8.26	0.3	10.97	0.1	0.00007938	0.00110728	0.00865346	0.25950476	A_07_P035263	c_2185	cmr|Unknown protein from 2D-page [c_2185]
yeaD	+6.96	9.96	+2.80	8.56	0.1	11.36	0.0	0.00001748	0.00043398	0.00763338	0.24728395	A_07_P035263	c_2185	cmr|Unknown protein from 2D-page [c_2185]
yeaD	+6.57	9.67	+2.72	8.31	0.0	11.03	0.0	0.00001155	0.00035443	0.00865282	0.25998092	A_07_P035263	c_2185	cmr|Unknown protein from 2D-page [c_2185]
yeaD	+5.41	8.97	+2.44	7.75	0.0	10.19	0.1	0.00002466	0.00053709	0.01234947	0.29459091	A_07_P015593	b1780	cmr|orf, hypothetical protein [b1780]
yeaD	+6.86	9.93	+2.78	8.54	0.0	11.32	0.1	0.00001710	0.00042882	0.00786014	0.24899396	A_07_P035263	c_2185	cmr|Unknown protein from 2D-page [c_2185]
yeaJ	-5.77	8.93	-2.53	10.20	0.1	7.67	0.1	0.00002982	0.00059893	0.00411077	0.20877419	A_07_P015623	b1786	cmr|orf, hypothetical protein [b1786]
'yeaJ	-2.52	9.51	-1.33	10.18	0.1	8.85	0.5	0.00900886	0.03809711	0.04903392	0.58528431	A_07_P044476	Z2826	cmr|orf Unknown function [Z2826]
'yeaJ	-3.59	8.99	-1.84	9.91	0.1	8.07	0.4	0.00144733	0.00909652	0.01812691	0.41004310	A_07_P044476	Z2826	cmr|orf Unknown function [Z2826]
'yeaJ	-2.36	9.59	-1.24	10.21	0.0	8.97	0.1	0.00070134	0.00524305	0.05895706	0.62548518	A_07_P044476	Z2826	cmr|orf Unknown function [Z2826]
'yeaJ	-3.73	9.34	-1.90	10.29	0.3	8.39	0.1	0.00048953	0.00396659	0.01543636	0.39135266	A_07_P044476	Z2826	cmr|orf Unknown function [Z2826]
'yeaJ	-2.79	9.47	-1.48	10.21	0.0	8.73	0.3	0.00154881	0.00956658	0.03683626	0.52342058	A_07_P044476	Z2826	cmr|orf Unknown function [Z2826]
yeaL	-4.45	8.89	-2.15	9.97	0.2	7.81	0.1	0.00009161	0.00122584	0.00893864	0.30264516	A_07_P015638	b1789	cmr|orf, hypothetical protein [b1789]
yeaT	-2.92	7.72	-1.55	8.50	0.0	6.95	0.1	0.00035922	0.00317256	0.03160124	0.49211672	A_07_P015689	b1799	cmr|putative transcriptional regulator LYSR-type [b1799]
yebB	-3.70	10.73	-1.89	11.67	0.3	9.78	0.3	0.00155126	0.00957391	0.01610455	0.39310078	A_07_P035344	c_2276	cmr|Hypothetical protein yebB [c_2276]
yebC	-2.00	12.22	-1.00	12.72	0.1	11.72	0.1	0.00322479	0.01688430	0.08985582	0.72473873	A_07_P015999	b1864	cmr|orf, hypothetical protein [b1864]
yebE	-19.50	10.48	-4.29	12.63	0.0	8.34	0.2	0.00000223	0.00017963	0.00005780	0.02275000	A_07_P015906	b1846	cmr|orf, hypothetical protein [b1846]
yebF	-29.05	14.00	-4.86	16.43	0.0	11.57	0.1	0.00000051	0.00009514	0.00001588	0.01086957	A_07_P015914	b1847	cmr|orf, hypothetical protein [b1847]
yebG	-7.96	14.53	-2.99	16.03	0.0	13.04	0.0	0.00000660	0.00027019	0.00149708	0.12809783	A_07_P015916	b1848	cmr|orf, hypothetical protein [b1848]
yebK	+5.81	8.85	+2.54	7.58	0.1	10.12	0.0	0.00001780	0.00043523	0.01097180	0.28087805	A_07_P015941	b1853	cmr|orf, hypothetical protein [b1853]
yebN	-8.04	10.22	-3.01	11.72	0.1	8.71	0.2	0.00001704	0.00042859	0.00146786	0.12697802	A_07_P015797	b1821	cmr|orf, hypothetical protein [b1821]
yebO	-11.82	9.54	-3.56	11.32	0.0	7.76	0.1	0.00000342	0.00020055	0.00040904	0.06133333	A_07_P015812	b1825	cmr|orf, hypothetical protein [b1825]
yebR	+3.69	8.95	+1.88	8.01	0.1	9.89	0.2	0.00024820	0.00245286	0.02301258	0.39858086	A_07_P015837	b1832	cmr|orf, hypothetical protein [b1832]
yebY	+4.64	10.48	+2.22	9.38	0.0	11.59	0.2	0.00007065	0.00101790	0.01585175	0.33817073	A_07_P015874	b1839	cmr|orf, hypothetical protein [b1839]
yecA	+2.46	12.07	+1.30	11.42	0.1	12.72	0.0	0.00068141	0.00513060	0.04629510	0.53711864	A_07_P014920	b1908	cmr|orf, hypothetical protein [b1908]
yecC	-3.32	8.64	-1.73	9.50	0.1	7.77	0.1	0.00021092	0.00218906	0.02207762	0.44850323	A_07_P014947	b1917	cmr|putative ATP-binding component of a transport system [b1917]
yecF	-2.24	8.56	-1.16	9.15	0.0	7.98	0.2	0.00219844	0.01248185	0.06804497	0.65926154	A_07_P014940	b1915	cmr|orf, hypothetical protein [b1915]
yecI	+2.87	10.29	+1.52	9.53	0.0	11.05	0.1	0.00027373	0.00261189	0.03573425	0.47840136	A_07_P035358	c_2315	cmr|Ferritin-like protein 2 [c_2315]
yecR	-4.37	7.85	-2.13	8.91	0.0	6.78	0.1	0.00005756	0.00088323	0.00952998	0.30872428	A_07_P016190	b1904	cmr|orf, hypothetical protein [b1904]
yecS	-4.81	9.20	-2.27	10.33	0.1	8.07	0.1	0.00004485	0.00075204	0.00724657	0.27759124	A_07_P014955	b1918	cmr|putative transport system permease protein [b1918]
yecS	-4.18	9.45	-2.06	10.48	0.0	8.42	0.2	0.00009669	0.00127819	0.01092797	0.33407767	A_07_P042434	c_2332	cmr|Hypothetical amino-acid ABC transporter permease protein yecS [c_2332]
yedE	+21.52	11.02	+4.43	8.81	0.1	13.23	0.1	0.00000131	0.00014643	0.00065231	0.07780303	A_07_P035376	c_2344	cmr|Hypothetical protein yedE [c_2344]
yedF	+20.90	10.61	+4.39	8.41	0.2	12.80	0.3	0.00000899	0.00031671	0.00071900	0.08143885	A_07_P015012	b1930	cmr|orf, hypothetical protein [b1930]
yedJ	-2.58	8.17	-1.37	8.85	0.0	7.49	0.2	0.00131212	0.00848030	0.04622777	0.57398265	A_07_P052607	Z3055	cmr|orf, hypothetical protein [Z3055]
yedJ	-5.89	10.04	-2.56	11.31	0.0	8.76	0.2	0.00003023	0.00060399	0.00388974	0.20550336	A_07_P041216	c_2381	cmr|Hypothetical protein yedJ [c_2381]
yedJ	-4.52	9.13	-2.18	10.21	0.0	8.04	0.1	0.00005392	0.00084214	0.00869474	0.30151982	A_07_P015168	b1962	cmr|orf, hypothetical protein [b1962]
yedR	-3.48	8.11	-1.80	9.01	0.1	7.21	0.2	0.00031566	0.00290966	0.01873984	0.41790368	A_07_P015174	b1963	cmr|orf, hypothetical protein [b1963]
yedU	+6.43	11.09	+2.69	9.75	0.4	12.43	0.2	0.00026036	0.00254204	0.00871316	0.25981061	A_07_P042474	c_2385	cmr|Protein yedU [c_2385]
yedW	-2.67	8.13	-1.41	8.84	0.1	7.43	0.1	0.00047099	0.00387016	0.04209540	0.55975507	A_07_P015203	b1969	cmr|putative 2-component transcriptional regulator [b1969]
yedZ	+2.17	7.64	+1.12	7.08	0.1	8.19	0.0	0.00135361	0.00867719	0.05818661	0.60150361	A_07_P015218	b1972	cmr|orf, hypothetical protein [b1972]
yeeA	+3.07	8.05	+1.62	7.24	0.1	8.86	0.1	0.00041245	0.00350252	0.03165269	0.45303636	A_07_P031967	c_2536	cmr|Hypothetical protein yeeA [c_2536]
yeeD	-4.11	10.34	-2.04	11.36	0.1	9.32	0.8	0.00858305	0.03670060	0.01210493	0.34650909	A_07_P015390	b2012	cmr|orf, hypothetical protein [b2012]
yeeF	-2.67	7.98	-1.42	8.69	0.0	7.27	0.1	0.00048751	0.00395227	0.04162093	0.55438240	A_07_P015400	b2014	cmr|putative amino acid/amine transport protein [b2014]
yeeO	-7.02	8.34	-2.81	9.75	0.0	6.93	0.2	0.00003314	0.00063062	0.00226245	0.15831111	A_07_P015266	b1985	cmr|orf, hypothetical protein [b1985]
yeeY	-3.29	9.56	-1.72	10.42	0.1	8.70	0.2	0.00025056	0.00246861	0.02223260	0.44760870	A_07_P015404	b2015	cmr|putative transcriptional regulator LYSR-type [b2015]
yeeZ	-3.53	12.17	-1.82	13.08	0.0	11.26	0.1	0.00009867	0.00129314	0.01784743	0.40901019	A_07_P015407	b2016	cmr|putative enzyme of sugar metabolism [b2016]
yefM	-2.64	13.81	-1.40	14.51	0.1	13.11	0.0	0.00039255	0.00337353	0.04301956	0.56300914	A_07_P015411	b2017	cmr|orf, hypothetical protein [b2017]
yegB	-2.33	7.95	-1.22	8.55	0.2	7.34	0.1	0.00301436	0.01597377	0.05992187	0.63062166	A_07_P042496	c_2603	cmr|Hypothetical transport protein yegB [c_2603]
yegD	-6.58	8.62	-2.72	9.98	0.1	7.26	0.3	0.00005775	0.00088534	0.00279662	0.17472222	A_07_P003121	b2069	cmr|putative heat shock protein [b2069]
yegL	-2.52	10.57	-1.33	11.24	0.2	9.91	0.3	0.00380749	0.01921934	0.04945058	0.58714178	A_07_P003144	b2073	cmr|orf, hypothetical protein [b2073]
yegM	-2.02	7.75	-1.01	8.26	0.0	7.25	0.0	0.00174533	0.01052412	0.08932101	0.72413491	A_07_P032088	c_2600	cmr|Hypothetical protein yegM precursor [c_2600]
yegQ	-10.07	10.00	-3.33	11.66	0.1	8.33	0.0	0.00000453	0.00023009	0.00075648	0.08888060	A_07_P003182	b2081	cmr|orf, hypothetical protein [b2081]
yegS	-2.17	9.57	-1.12	10.13	0.2	9.01	0.1	0.00339411	0.01752604	0.07177655	0.66630307	A_07_P032127	c_2614	cmr|Hypothetical protein yegS [c_2614]
yegU	+2.41	8.42	+1.27	7.79	0.3	9.06	0.1	0.00395948	0.01980872	0.04891197	0.54965739	A_07_P003256	b2099	cmr|orf, hypothetical protein [b2099]
yegX	-2.72	8.62	-1.44	9.34	0.1	7.90	0.1	0.00047237	0.00387253	0.03987932	0.54407279	A_07_P051783	Z3266	cmr|orf, hypothetical protein [Z3266]
yehD	+5.65	7.85	+2.50	6.60	0.0	9.10	0.0	0.00001713	0.00042882	0.01149390	0.28587678	A_07_P003318	b2111	cmr|putative fimbrial-like protein [b2111]
yehL	-5.27	7.90	-2.40	9.10	0.0	6.70	0.1	0.00002435	0.00053313	0.00531949	0.23792614	A_07_P003356	b2119	cmr|orf, hypothetical protein [b2119]
yehL	-5.57	9.08	-2.48	10.32	0.1	7.84	0.2	0.00007940	0.00110728	0.00469449	0.22195195	A_07_P041221	c_2649	cmr|Hypothetical protein yehL [c_2649]
yehP	-3.87	10.66	-1.95	11.63	0.6	9.68	0.0	0.00497182	0.02360564	0.01356961	0.36961938	A_07_P003369	b2121	cmr|orf, hypothetical protein [b2121]
yehS	-2.85	9.21	-1.51	9.96	0.0	8.45	0.1	0.00026434	0.00255503	0.03429370	0.50935849	A_07_P003385	b2124	cmr|orf, hypothetical protein [b2124]
yehT	+2.03	9.19	+1.02	8.67	0.3	9.70	0.1	0.00621165	0.02826178	0.07006098	0.66089874	A_07_P003390	b2125	cmr|orf, hypothetical protein [b2125]
yehX	-2.01	7.73	-1.01	8.24	0.0	7.23	0.1	0.00200328	0.01162967	0.09034299	0.72384733	A_07_P032191	c_2659	cmr|Hypothetical ABC transporter ATP-binding protein yehX [c_2659]
yehZ	-2.13	8.45	-1.09	8.99	0.1	7.90	0.2	0.00456604	0.02211250	0.07734883	0.67805122	A_07_P032198	c_2661	cmr|Hypothetical protein yehZ precursor [c_2661]
yeiA	+4.46	9.88	+2.16	8.80	0.1	10.95	0.0	0.00004911	0.00079227	0.01674606	0.34464052	A_07_P003500	b2147	cmr|putative oxidoreductase [b2147]
yeiA	+2.82	9.94	+1.50	9.19	0.3	10.69	0.1	0.00222126	0.01257968	0.03703887	0.48433555	A_07_P003500	b2147	cmr|putative oxidoreductase [b2147]
yeiA	+3.61	10.02	+1.85	9.09	0.3	10.94	0.0	0.00060208	0.00466268	0.02415841	0.40376858	A_07_P003500	b2147	cmr|putative oxidoreductase [b2147]
yeiA	+3.87	9.57	+1.95	8.60	0.2	10.55	0.1	0.00023248	0.00234924	0.02132749	0.38551091	A_07_P003500	b2147	cmr|putative oxidoreductase [b2147]
yeiA	+3.55	9.90	+1.83	8.99	0.2	10.82	0.1	0.00031017	0.00286751	0.02486280	0.40945607	A_07_P003500	b2147	cmr|putative oxidoreductase [b2147]
yeiC	+19.66	8.75	+4.30	6.60	0.3	10.89	0.2	0.00000648	0.00027019	0.00083206	0.08451613	A_07_P032264	c_2701	cmr|Hypothetical sugar kinase yeiC [c_2701]
yeiQ	+12.10	9.64	+3.60	7.84	0.0	11.44	0.1	0.00000295	0.00018751	0.00247205	0.14414815	A_07_P000660	b2172	cmr|putative oxidoreductase [b2172]
yeiT	+10.84	9.68	+3.44	7.96	0.2	11.40	0.0	0.00000734	0.00028727	0.00306847	0.16431973	A_07_P003491	b2146	cmr|putative oxidoreductase [b2146]
yeiW	-2.42	8.10	-1.28	8.74	0.1	7.46	0.1	0.00081209	0.00585149	0.05381098	0.60687679	A_07_P010506	ABE-0285016	asap|conserved protein [b4502]
yejE	+3.75	7.79	+1.91	6.84	0.1	8.74	0.0	0.00007840	0.00110010	0.02247586	0.39537430	A_07_P032268	c_2716	cmr|Hypothetical ABC transporter permease protein yejE [c_2716]
yejE	+2.98	7.79	+1.58	7.00	0.1	8.58	0.1	0.00025627	0.00251460	0.03317962	0.46475089	A_07_P000694	b2179	cmr|putative transport system permease protein [b2179]
yejG	-7.98	12.07	-3.00	13.57	0.0	10.57	0.0	0.00000645	0.00027019	0.00150025	0.12631016	A_07_P000703	b2181	cmr|orf, hypothetical protein [b2181]
yejL	-2.38	12.00	-1.25	12.63	0.1	11.38	0.0	0.00070870	0.00528303	0.05699441	0.61798898	A_07_P000733	b2187	cmr|orf, hypothetical protein [b2187]
yejM	-2.16	8.59	-1.11	9.15	0.2	8.04	0.1	0.00402918	0.02009356	0.07254129	0.66749854	A_07_P000736	b2188	cmr|putative sulfatase [b2188]
yejO	-2.85	8.57	-1.51	9.32	0.2	7.81	0.1	0.00085937	0.00613879	0.03476753	0.51204864	A_07_P000743	b2190	cmr|putative ATP-binding component of a transport system [b2190]
yfaA	-2.43	7.77	-1.28	8.41	0.2	7.13	0.2	0.00277378	0.01495299	0.05402566	0.60799142	A_07_P000938	b2230	cmr|orf, hypothetical protein [b2230]
yfaE	+6.62	9.31	+2.73	7.95	0.2	10.67	0.0	0.00003459	0.00064547	0.00845592	0.25700772	A_07_P000966	b2236	cmr|orf, hypothetical protein [b2236]
yfaW	+2.90	7.80	+1.53	7.03	0.0	8.57	0.1	0.00033459	0.00302754	0.03520262	0.47655202	A_07_P001021	b2247	cmr|putative racemase [b2247]
yfaZ	-2.35	7.62	-1.23	8.24	0.0	7.01	0.0	0.00072007	0.00535764	0.05971989	0.63060362	A_07_P001040	b2250	cmr|orf, hypothetical protein [b2250]
yfbB	+3.41	8.72	+1.77	7.83	0.0	9.60	0.3	0.00061435	0.00472975	0.02641197	0.41834004	A_07_P001104	b2263	cmr|putative enzyme [b2263]
yfbM	+2.42	8.03	+1.27	7.39	0.1	8.67	0.0	0.00083966	0.00602809	0.04843750	0.54705882	A_07_P001148	b2272	cmr|orf, hypothetical protein [b2272]
yfbQ	-2.14	8.69	-1.10	9.24	0.1	8.15	0.1	0.00155052	0.00957309	0.07573806	0.67482739	A_07_P013738	b2290	cmr|putative aminotransferase [b2290]
yfbV	-2.00	9.94	-1.00	10.44	0.0	9.44	0.0	0.00183193	0.01090842	0.08973895	0.72528234	A_07_P013765	b2295	cmr|orf, hypothetical protein [b2295]
yfcZ	-2.12	12.36	-1.09	12.90	0.1	11.82	0.1	0.00197180	0.01150478	0.07609883	0.67384702	A_07_P014002	b2343	cmr|orf, hypothetical protein [b2343]
yfdY	+2.05	9.70	+1.04	9.18	0.1	10.22	0.0	0.00175234	0.01056236	0.06543826	0.63911911	A_07_P014168	b2377	cmr|orf, hypothetical protein [b2377]
yfdZ	+3.86	8.80	+1.95	7.82	0.0	9.77	0.4	0.00068123	0.00513060	0.02178100	0.38835787	A_07_P014179	b2379	cmr|putative aminotransferase [b2379]
yfeD	-3.36	9.37	-1.75	10.25	0.1	8.50	0.2	0.00037450	0.00325950	0.02079586	0.43480744	A_07_P042786	Z3663	cmr|orf, hypothetical protein [Z3663]
yfeD	-5.22	9.25	-2.38	10.44	0.0	8.06	0.4	0.00047585	0.00388777	0.00565803	0.24472527	A_07_P030017	c_2935	cmr|Hypothetical protein yfeD [c_2935]
yfeN	-2.98	10.85	-1.58	11.63	0.2	10.06	0.0	0.00063194	0.00482505	0.03033791	0.49089414	A_07_P013096	b2408	cmr|putative sugar hydrolase [b2408]
yffB	-2.31	11.20	-1.21	11.81	0.0	10.60	0.1	0.00085981	0.00613919	0.06182419	0.63743287	A_07_P013411	b2471	cmr|orf, hypothetical protein [b2471]
yfgC	+2.69	8.66	+1.43	7.95	0.0	9.37	0.1	0.00078572	0.00571910	0.03973577	0.49968051	A_07_P013530	b2494	cmr|orf, hypothetical protein [b2494]
yfgC	+3.45	9.82	+1.79	8.92	0.2	10.71	0.2	0.00047399	0.00387863	0.02618394	0.41682508	A_07_P042608	c_3011	cmr|Hypothetical protein yfgC precursor [c_3011]
yfgD	+4.09	8.44	+2.03	7.42	0.1	9.45	0.2	0.00018433	0.00199457	0.01953252	0.37502439	A_07_P013532	b2495	cmr|putative oxidoreductase [b2495]
yfgG	+3.15	10.46	+1.66	9.63	0.1	11.29	0.0	0.00026388	0.00255503	0.03019817	0.44309413	A_07_P013578	b2504	cmr|orf, hypothetical protein [b2504]
yfgI	-3.60	10.90	-1.85	11.82	0.2	9.97	0.4	0.00143012	0.00902801	0.01773183	0.40754745	A_07_P013587	b2506	cmr|putative membrane protein [b2506]
yfhA	+2.02	9.09	+1.01	8.58	0.2	9.59	0.0	0.00487081	0.02322412	0.06944804	0.65866867	A_07_P019671	b2554	cmr|putative 2-component transcriptional regulator [b2554]
yfhB	-7.30	9.87	-2.87	11.30	0.1	8.43	0.0	0.00001102	0.00034947	0.00205983	0.15516746	A_07_P019703	b2560	cmr|orf, hypothetical protein [b2560]
yfhM	+2.07	8.39	+1.05	7.86	0.2	8.92	0.2	0.00427821	0.02098320	0.06598641	0.64168623	A_07_P030191	c_3043	cmr|Hypothetical lipoprotein yfhM precursor [c_3043]
yfiB	+3.43	9.31	+1.78	8.42	0.1	10.20	0.1	0.00021077	0.00218894	0.02603976	0.41621320	A_07_P019901	b2605	cmr|putative outer membrane protein [b2605]
yfiD	+4.84	11.34	+2.28	10.20	0.1	12.48	0.2	0.00007173	0.00102756	0.01467543	0.32634181	A_07_P019799	b2579	cmr|putative formate acetyltransferase [b2579]
yfiF	+5.39	10.78	+2.43	9.56	0.1	11.99	0.1	0.00002743	0.00056718	0.01246253	0.29549699	A_07_P019806	b2581	cmr|orf, hypothetical protein [b2581]
yfiH	-3.77	8.97	-1.92	9.92	0.1	8.01	0.0	0.00007984	0.00110942	0.01469195	0.38296358	A_07_P019847	b2593	cmr|orf, hypothetical protein [b2593]
yfiQ	+10.64	9.88	+3.41	8.17	0.0	11.58	0.2	0.00000612	0.00026402	0.00312055	0.16486577	A_07_P019821	b2584	cmr|orf, hypothetical protein [b2584]
yfjF	-3.26	11.45	-1.70	12.30	0.1	10.60	0.1	0.00018474	0.00199636	0.02320884	0.45166873	A_07_P019968	b2618	cmr|orf, hypothetical protein [b2618]
yfjG	-4.01	9.96	-2.00	10.96	0.0	8.96	0.5	0.00124026	0.00813949	0.01213923	0.34560579	A_07_P019973	b2619	cmr|orf, hypothetical protein [b2619]
yfjW	-2.24	8.95	-1.16	9.53	0.2	8.37	0.2	0.00285575	0.01529803	0.06725927	0.65690447	A_07_P020076	b2642	cmr|orf, hypothetical protein [b2642]
ygaA	-4.87	8.90	-2.28	10.04	0.1	7.76	0.2	0.00005875	0.00089714	0.00690422	0.26905941	A_07_P030601	c_3263	cmr|Hypothetical sigma-54-dependent transcriptional regulator ygaA [c_3263]
ygaD	+2.77	9.61	+1.47	8.88	0.0	10.34	0.2	0.00078658	0.00572006	0.03747015	0.48714286	A_07_P001946	b2700	cmr|orf, hypothetical protein [b2700]
ygaF	+2.50	10.35	+1.32	9.69	0.1	11.01	0.3	0.00204442	0.01183794	0.04526169	0.53139448	A_07_P043074	Z3958	cmr|orf, hypothetical protein [Z3958]
ygaF	+2.48	10.24	+1.31	9.58	0.1	10.90	0.2	0.00163799	0.01002272	0.04653646	0.53793686	A_07_P030517	c_3208	cmr|Hypothetical protein ygaF [c_3208]
ygaV	+6.22	9.20	+2.64	7.88	0.2	10.52	0.1	0.00004863	0.00078769	0.00939469	0.26746835	A_07_P020189	b2667	cmr|orf, hypothetical protein [b2667]
ygaV	+2.68	8.18	+1.42	7.47	0.1	8.89	0.2	0.00085785	0.00613593	0.03994792	0.49995231	A_07_P041648	c_3216	cmr|Hypothetical transcriptional regulator ygaV [c_3216]
ygaW	-13.50	11.10	-3.76	12.97	0.1	9.22	0.2	0.00000834	0.00030273	0.00026232	0.05162500	A_07_P001825	b2670	cmr|orf, hypothetical protein [b2670]
ygaZ	-2.43	9.86	-1.28	10.50	0.2	9.22	0.1	0.00140512	0.00890948	0.05303417	0.60286643	A_07_P001881	b2682	cmr|orf, hypothetical protein [b2682]
ygbE	-2.08	8.87	-1.06	9.40	0.0	8.34	0.1	0.00244771	0.01353589	0.08117315	0.69493747	A_07_P030701	c_3316	cmr|Hypothetical protein ygbE [c_3316]
ygbF	-2.09	8.98	-1.06	9.51	0.1	8.44	0.2	0.00443461	0.02159558	0.07855373	0.68366501	A_07_P002216	b2754	cmr|orf, hypothetical protein [b2754]
ygbJ	+8.24	9.39	+3.04	7.87	0.1	10.91	0.0	0.00000670	0.00027042	0.00541159	0.21569620	A_07_P030675	c_3297	cmr|Hypothetical oxidoreductase ygbJ [c_3297]
ygbJ	+2.96	8.10	+1.57	7.32	0.1	8.88	0.1	0.00038466	0.00332667	0.03376270	0.46833480	A_07_P002127	b2736	cmr|putative dehydrogenase [b2736]
ygbK	+5.00	8.70	+2.32	7.54	0.2	9.86	0.1	0.00006080	0.00091422	0.01407393	0.32020231	A_07_P002131	b2737	cmr|orf, hypothetical protein [b2737]
ygcG	-2.84	9.64	-1.50	10.39	0.1	8.89	0.1	0.00032676	0.00297891	0.03526867	0.51366327	A_07_P032628	c_3342	cmr|Hypothetical protein ygcG [c_3342]
ygcI	-2.97	8.11	-1.57	8.89	0.1	7.32	0.1	0.00034761	0.00310252	0.03080412	0.49186613	A_07_P002234	b2757	cmr|orf, hypothetical protein [b2757]
ygcP	-2.17	10.26	-1.12	10.82	0.0	9.70	0.2	0.00208480	0.01200551	0.07413745	0.67197467	A_07_P002288	b2768	cmr|putative anti-terminator regulatory protein [b2768]
ygdD	-2.14	10.71	-1.10	11.26	0.2	10.17	0.2	0.00582680	0.02682374	0.07554560	0.67425737	A_07_P006031	b2807	cmr|orf, hypothetical protein [b2807]
ygdE	-3.01	8.79	-1.59	9.58	0.1	7.99	0.2	0.00061409	0.00472975	0.02874111	0.48395722	A_07_P006026	b2806	cmr|orf, hypothetical protein [b2806]
ygdH	+6.31	10.18	+2.66	8.85	0.1	11.51	0.0	0.00001727	0.00043168	0.00945567	0.26775180	A_07_P002424	b2795	cmr|orf, hypothetical protein [b2795]
ygdL	-4.74	10.38	-2.24	11.50	0.1	9.26	0.1	0.00003757	0.00066784	0.00762957	0.28600000	A_07_P032656	c_3383	cmr|Hypothetical protein ygdL [c_3383]
ygdQ	-3.40	7.99	-1.77	8.87	0.1	7.11	0.1	0.00012696	0.00153291	0.02052591	0.43377181	A_07_P006141	b2832	cmr|putative transport protein [b2832]
ygeK	-3.57	10.98	-1.84	11.90	0.2	10.07	0.4	0.00180250	0.01076579	0.01682800	0.39721139	A_07_P006257	b2855	cmr|putative 2-component transcriptional regulator [b2855]
ygeR	-13.72	9.88	-3.78	11.77	0.0	7.99	0.1	0.00000275	0.00018406	0.00022104	0.05043478	A_07_P006295	b2865	cmr|putative lipoprotein [b2865]
ygeT	+12.09	9.06	+3.60	7.26	0.1	10.85	0.0	0.00000330	0.00019671	0.00249428	0.14384615	A_07_P032831	c_3445	cmr|Xanthine dehydrogenase, FAD binding subunit [c_3445]
ygeV	+2.66	8.27	+1.41	7.57	0.3	8.98	0.1	0.00189155	0.01116147	0.04064469	0.50228414	A_07_P006312	b2869	cmr|putative transcriptional regulator [b2869]
ygfA	-3.61	9.30	-1.85	10.23	0.1	8.37	0.1	0.00012294	0.00149808	0.01639609	0.39470948	A_07_P006525	b2912	cmr|putative ligase [b2912]
ygfM	+2.11	7.63	+1.07	7.09	0.1	8.16	0.0	0.00149146	0.00930330	0.06215320	0.62526518	A_07_P041709	c_3458	cmr|Hypothetical protein ygfM [c_3458]
ygfX	-4.06	11.03	-2.02	12.04	0.0	10.01	0.1	0.00006970	0.00101135	0.01189977	0.34566421	A_07_P006446	b2896	cmr|orf, hypothetical protein [b2896]
yggD	-2.95	8.37	-1.56	9.15	0.0	7.59	0.1	0.00029740	0.00277716	0.03115600	0.49199599	A_07_P011793	b2929	cmr|putative transcriptional regulator [b2929]
yggD	-3.62	9.60	-1.86	10.53	0.0	8.67	0.2	0.00019654	0.00208096	0.01654789	0.39594225	A_07_P032921	c_3512	cmr|Hypothetical protein yggD [c_3512]
yggG	+2.07	10.54	+1.05	10.01	0.1	11.06	0.1	0.00258242	0.01415162	0.06520452	0.63842040	A_07_P032951	c_3521	cmr|Putative metalloprotease yggG [c_3521]
yggL	+3.47	12.93	+1.79	12.03	0.0	13.82	0.3	0.00067395	0.00509417	0.02594195	0.41507114	A_07_P011938	b2959	cmr|orf, hypothetical protein [b2959]
yggP	-3.09	8.08	-1.63	8.90	0.1	7.27	0.1	0.00029928	0.00278880	0.02660887	0.47497732	A_07_P011806	b2932	cmr|orf, hypothetical protein [b2932]
yggS	-2.84	8.67	-1.50	9.42	0.1	7.92	0.0	0.00036005	0.00317569	0.03496316	0.51396825	A_07_P011899	b2951	cmr|orf, hypothetical protein [b2951]
yggX	+2.74	10.68	+1.45	9.96	0.1	11.41	0.1	0.00034342	0.00307905	0.03830602	0.49312347	A_07_P011954	b2962	cmr|orf, hypothetical protein [b2962]
yghB	-11.26	10.95	-3.49	12.70	0.0	9.21	0.0	0.00000276	0.00018406	0.00051639	0.07324324	A_07_P012180	b3009	cmr|orf, hypothetical protein [b3009]
yghD	-2.92	8.57	-1.55	9.34	0.0	7.79	0.2	0.00046673	0.00384264	0.03158283	0.49231683	A_07_P036669	c_3699	cmr|Putative general secretion pathway protein M-type yghD [c_3699]
yghU	+3.90	8.43	+1.96	7.45	0.1	9.41	0.1	0.00008507	0.00116565	0.02091908	0.38341094	A_07_P012078	b2989	cmr|orf, hypothetical protein [b2989]
yghU	+4.11	8.98	+2.04	7.96	0.1	10.00	0.3	0.00047666	0.00389132	0.01962779	0.37639464	A_07_P041281	c_3726	cmr|Hypothetical GST-like protein yghU [c_3726]
yghZ	+5.50	9.99	+2.46	8.76	0.1	11.22	0.1	0.00003183	0.00061949	0.01196900	0.29125193	A_07_P012136	b3001	cmr|putative reductase [b3001]
ygiB	+2.91	11.98	+1.54	11.21	0.2	12.75	0.4	0.00330094	0.01718583	0.03490028	0.47368103	A_07_P012316	b3037	cmr|orf, hypothetical protein [b3037]
ygiC	+2.60	8.48	+1.38	7.79	0.3	9.17	0.1	0.00167957	0.01023342	0.04214685	0.50964670	A_07_P012323	b3038	cmr|putative synthetase/amidase [b3038]
ygiF	-4.91	11.28	-2.30	12.43	0.0	10.14	0.1	0.00002834	0.00057633	0.00680640	0.26790000	A_07_P000003	b3054	cmr|orf, hypothetical protein [b3054]
ygiF	-3.09	12.73	-1.63	13.54	0.0	11.91	0.2	0.00032038	0.00294290	0.02665396	0.47470588	A_07_P041286	c_3802	cmr|Hypothetical protein ygiF [c_3802]
ygiH	-2.27	8.61	-1.19	9.21	0.0	8.02	0.2	0.00178959	0.01070491	0.06386115	0.64699485	A_07_P000026	b3059	cmr|orf, hypothetical protein [b3059]
ygiN	+29.94	11.18	+4.90	8.72	0.1	13.63	0.0	0.00000069	0.00011461	0.00032901	0.05395833	A_07_P012278	b3029	cmr|orf, hypothetical protein [b3029]
ygiN	+2.10	11.89	+1.07	11.35	0.3	12.42	0.0	0.00842487	0.03615297	0.06546557	0.63898946	A_07_P036791	c_3769	cmr|Protein ygiN [c_3769]
ygiP	-2.78	10.12	-1.48	10.86	0.0	9.39	0.2	0.00061528	0.00473461	0.03645071	0.52170909	A_07_P036891	c_3810	cmr|Hypothetical transcriptional regulator ygiP [c_3810]
ygjH	-2.87	12.44	-1.52	13.19	0.0	11.68	0.3	0.00151374	0.00940872	0.03431466	0.50918944	A_07_P000096	b3074	cmr|putative tRNA synthetase [b3074]
ygjN	-2.00	9.04	-1.00	9.54	0.1	8.54	0.1	0.00243126	0.01347806	0.09115155	0.72442706	A_07_P000142	b3083	cmr|orf, hypothetical protein [b3083]
ygjP	-6.15	9.16	-2.62	10.47	0.1	7.85	0.1	0.00002282	0.00051615	0.00349848	0.19741935	A_07_P000154	b3085	cmr|orf, hypothetical protein [b3085]
ygjP	-15.94	10.34	-3.99	12.34	0.0	8.34	0.1	0.00000188	0.00016889	0.00011941	0.03615385	A_07_P034044	c_3843	cmr|Hypothetical protein ygjP [c_3843]
ygjQ	-4.40	7.89	-2.14	8.96	0.0	6.82	0.2	0.00007811	0.00109698	0.00937436	0.30812109	A_07_P041292	c_3844	cmr|Hypothetical protein ygjQ [c_3844]
ygjQ	-2.87	8.21	-1.52	8.97	0.3	7.45	0.2	0.00172413	0.01042962	0.03403582	0.50744318	A_07_P000160	b3086	cmr|orf, hypothetical protein [b3086]
ygjR	+7.50	9.51	+2.91	8.06	0.1	10.97	0.1	0.00000999	0.00033316	0.00666857	0.23279379	A_07_P000165	b3087	cmr|orf, hypothetical protein [b3087]
ygjU	+2.70	9.16	+1.43	8.44	0.1	9.87	0.2	0.00096379	0.00671411	0.04014228	0.50079239	A_07_P034047	c_3847	cmr|Hypothetical symporter ygjU [c_3847]
ygjV	+2.59	7.82	+1.37	7.13	0.0	8.50	0.1	0.00055339	0.00436067	0.04243140	0.51112471	A_07_P034052	c_3848	cmr|Hypothetical protein ygjV [c_3848]
yhaG	+2.05	9.30	+1.04	8.78	0.2	9.82	0.0	0.00337716	0.01747289	0.06650851	0.64318796	A_07_P034086	c_3883	cmr|D-galactarate dehydratase [c_3883]
yhaG	+3.52	9.32	+1.82	8.41	0.2	10.23	0.2	0.00033094	0.00300596	0.02494347	0.40992693	A_07_P045896	Z4480	cmr|putative hydrolase [Z4480]
yhaJ	+2.11	9.60	+1.08	9.06	0.0	10.14	0.1	0.00149966	0.00933386	0.06241171	0.62626514	A_07_P000254	b3105	cmr|putative transcriptional regulator LYSR-type [b3105]
yhaR	+14.39	12.08	+3.85	10.15	0.1	14.00	0.4	0.00004321	0.00073315	0.00175432	0.12611872	A_07_P034071	c_3871	cmr|TdcF protein [c_3871]
yhaV	-2.52	10.04	-1.33	10.71	0.0	9.37	0.1	0.00053410	0.00424957	0.04945058	0.58669932	A_07_P041771	c_3885	cmr|Hypothetical protein yhaV [c_3885]
yhbC	-4.07	13.17	-2.03	14.18	0.0	12.16	0.2	0.00010565	0.00135201	0.01195630	0.34602941	A_07_P000572	b3170	cmr|orf, hypothetical protein [b3170]
yhbE	-4.44	9.13	-2.15	10.20	0.1	8.05	0.0	0.00004093	0.00070661	0.00922383	0.30701903	A_07_P012400	b3184	cmr|orf, hypothetical protein [b3184]
yhbJ	+3.96	9.23	+1.98	8.24	0.1	10.22	0.0	0.00007108	0.00102103	0.02029408	0.37856635	A_07_P012503	b3205	cmr|orf, hypothetical protein [b3205]
yhbO	+2.70	8.09	+1.43	7.37	0.0	8.80	0.1	0.00036345	0.00319849	0.03994029	0.50065287	A_07_P000486	b3153	cmr|orf, hypothetical protein [b3153]
yhbY	-2.37	10.14	-1.24	10.76	0.1	9.52	0.1	0.00108019	0.00731149	0.05760353	0.62074606	A_07_P000614	b3180	cmr|orf, hypothetical protein [b3180]
yhcB	+3.35	12.47	+1.74	11.60	0.0	13.34	0.1	0.00012996	0.00155599	0.02689787	0.42179283	A_07_P012640	b3233	cmr|orf, hypothetical protein [b3233]
yhcH	+4.01	8.06	+2.00	7.06	0.0	9.06	0.2	0.00018209	0.00197571	0.02018928	0.37840476	A_07_P034165	c_3975	cmr|Hypothetical protein yhcH [c_3975]
yhcH	+2.71	8.03	+1.44	7.31	0.3	8.75	0.2	0.00190611	0.01121443	0.03974339	0.49937749	A_07_P045957	Z4579	cmr|orf, hypothetical protein [Z4579]
yhcI	+3.52	7.93	+1.82	7.02	0.2	8.83	0.0	0.00035830	0.00316911	0.02497967	0.41009385	A_07_P051815	Z4580	cmr|putative NAGC-like transcriptional regulator [Z4580]
yhcJ	+5.24	8.57	+2.39	7.38	0.2	9.77	0.2	0.00010473	0.00134567	0.01268229	0.29757079	A_07_P041791	c_3977	cmr|Hypothetical protein yhcJ [c_3977]
yhcJ	+6.46	8.57	+2.69	7.22	0.1	9.91	0.2	0.00002594	0.00055176	0.00892403	0.26066790	A_07_P041304	c_3977	cmr|Hypothetical protein yhcJ [c_3977]
yhcK	-2.25	10.01	-1.17	10.60	0.0	9.42	0.1	0.00115845	0.00772167	0.06646723	0.65567669	A_07_P045969	Z4584	cmr|putative FADA-type transcriptional regulator [Z4584]
yhcK	-2.62	9.53	-1.39	10.22	0.0	8.83	0.1	0.00051354	0.00412930	0.04365981	0.56574486	A_07_P034168	c_3980	cmr|Hypothetical transcriptional regulator yhcK [c_3980]
yhcO	-2.99	8.05	-1.58	8.84	0.1	7.26	0.1	0.00028318	0.00267294	0.03016959	0.49119959	A_07_P012666	b3239	cmr|orf, hypothetical protein [b3239]
yhcR	-3.00	7.62	-1.59	8.41	0.1	6.82	0.2	0.00053293	0.00424558	0.02933562	0.48616842	A_07_P035544	c_3997	cmr|Hypothetical protein yhcR [c_3997]
yhcR	-5.73	8.02	-2.52	9.27	0.0	6.76	0.1	0.00001774	0.00043523	0.00422764	0.21130159	A_07_P048607	Z4601	cmr|orf, hypothetical protein [Z4601]
yhdE	+3.43	9.38	+1.78	8.49	0.2	10.27	0.0	0.00027301	0.00260657	0.02627350	0.41782828	A_07_P012715	b3248	cmr|orf, hypothetical protein [b3248]
yhdH	+10.93	10.25	+3.45	8.53	0.3	11.98	0.0	0.00001500	0.00040227	0.00302020	0.16396552	A_07_P034191	c_4009	cmr|Protein yhdH [c_4009]
yhdH	+6.24	8.77	+2.64	7.45	0.2	10.09	0.1	0.00003242	0.00062637	0.00941629	0.26759928	A_07_P012736	b3253	cmr|putative dehydrogenase [b3253]
yhdT	-2.03	10.50	-1.02	11.02	0.1	9.99	0.1	0.00215041	0.01226667	0.08700203	0.71565308	A_07_P012753	b3257	cmr|orf, hypothetical protein [b3257]
yhdZ	+6.14	8.70	+2.62	7.39	0.1	10.01	0.0	0.00002365	0.00052523	0.00987805	0.27094077	A_07_P012823	b3271	cmr|putative ATP-binding component of a transport system [b3271]
yheF	-3.41	8.16	-1.77	9.04	0.1	7.28	0.1	0.00019858	0.00209402	0.02038554	0.43313090	A_07_P034326	c_4096	cmr|Probable general secretion pathway protein D precursor [c_4096]
yheM	-2.34	8.19	-1.23	8.81	0.0	7.58	0.1	0.00076636	0.00560668	0.06015434	0.63180120	A_07_P017750	b3344	cmr|orf, hypothetical protein [b3344]
yheN	-2.90	9.37	-1.54	10.14	0.0	8.61	0.0	0.00023160	0.00234711	0.03283473	0.50092054	A_07_P052135	Z4703	cmr|orf, hypothetical protein [Z4703]
yheN	-3.28	10.26	-1.72	11.12	0.3	9.40	0.1	0.00074765	0.00551078	0.02278392	0.45120755	A_07_P017753	b3345	cmr|orf, hypothetical protein [b3345]
yheS	-2.58	11.10	-1.37	11.78	0.0	10.41	0.0	0.00039972	0.00341321	0.04614901	0.57390995	A_07_P017786	b3352	cmr|putative ATP-binding component of a transport system [b3352]
yheV	-2.01	10.50	-1.01	11.00	0.0	9.99	0.2	0.00553132	0.02574958	0.09094512	0.72461538	A_07_P010582	ABE-0285072	asap|predicted protein [b4551]
yhfC	-6.32	8.16	-2.66	9.49	0.0	6.83	0.3	0.00006023	0.00090997	0.00318979	0.18669145	A_07_P041829	c_4139	cmr|Hypothetical protein yhfC [c_4139]
yhfS	-3.55	10.70	-1.83	11.61	0.0	9.78	0.0	0.00008942	0.00120520	0.01769754	0.40795022	A_07_P017907	b3376	cmr|orf, hypothetical protein [b3376]
yhgA	-2.51	8.13	-1.33	8.79	0.2	7.47	0.2	0.00211309	0.01211969	0.05030297	0.59146378	A_07_P052151	Z4766	cmr|orf, hypothetical protein [Z4766]
yhgF	+4.38	10.57	+2.13	9.50	0.0	11.63	0.2	0.00011252	0.00140710	0.01753938	0.35493573	A_07_P018065	b3407	cmr|orf, hypothetical protein [b3407]
yhgH	+2.05	7.72	+1.03	7.21	0.0	8.24	0.1	0.00217591	0.01238077	0.06726562	0.64772477	A_07_P041831	c_4190	cmr|Hypothetical protein yhgH [c_4190]
yhgH	+2.60	11.78	+1.38	11.09	0.0	12.47	0.1	0.00045133	0.00374973	0.04232597	0.51024502	A_07_P041315	c_4190	cmr|Hypothetical protein yhgH [c_4190]
yhhA	+2.50	8.78	+1.32	8.12	0.1	9.44	0.1	0.00067004	0.00507658	0.04519245	0.53137416	A_07_P018259	b3448	cmr|orf, hypothetical protein [b3448]
yhhG	+2.07	7.35	+1.05	6.83	0.1	7.88	0.1	0.00222034	0.01257968	0.06589367	0.64157699	A_07_P034573	c_4274	cmr|Nickel responsive regulator [c_4274]
yhhI	-4.38	10.77	-2.13	11.84	0.5	9.71	0.3	0.00242836	0.01346675	0.00905043	0.30381663	A_07_P007358	b3484	cmr|putative receptor [b3484]
yhhJ	+3.77	8.28	+1.91	7.33	0.2	9.24	0.0	0.00019187	0.00204384	0.02216717	0.39169473	A_07_P007361	b3485	cmr|putative transporter [b3485]
yhhS	-2.64	8.20	-1.40	8.90	0.1	7.50	0.1	0.00059388	0.00461503	0.04314850	0.56422757	A_07_P007303	b3473	cmr|putative transport [b3473]
yhhU	-2.18	10.50	-1.12	11.06	0.1	9.93	0.2	0.00250093	0.01378662	0.07226626	0.66809160	A_07_P041853	c_4268	cmr|4-phosphopantetheinyl transferase acpT [c_4268]
yhhX	+3.09	9.67	+1.63	8.86	0.3	10.48	0.1	0.00136589	0.00873191	0.03161903	0.45296633	A_07_P018230	b3440	cmr|putative regulator [b3440]
yhiD	-3.20	14.30	-1.68	15.14	0.0	13.46	0.1	0.00015447	0.00175596	0.02441629	0.45982057	A_07_P007475	b3508	cmr|putative transport ATPase [b3508]
yhiN	-7.15	10.34	-2.84	11.76	0.1	8.92	0.1	0.00001209	0.00036323	0.00220592	0.15644144	A_07_P007396	b3492	cmr|orf, hypothetical protein [b3492]
yhiP	+3.77	10.42	+1.91	9.46	0.0	11.37	0.3	0.00047251	0.00387253	0.02248539	0.39465998	A_07_P007419	b3496	cmr|putative transport protein [b3496]
yhiQ	-2.15	8.10	-1.10	8.65	0.1	7.54	0.2	0.00329875	0.01718011	0.07447536	0.67155785	A_07_P007422	b3497	cmr|orf, hypothetical protein [b3497]
yhiR	-5.11	12.39	-2.35	13.57	0.0	11.22	0.2	0.00006313	0.00093948	0.00599975	0.24857895	A_07_P007435	b3499	cmr|orf, hypothetical protein [b3499]
yhjJ	-3.08	9.12	-1.63	9.93	0.0	8.31	0.2	0.00036331	0.00319849	0.02714558	0.47805369	A_07_P049426	Z4941	cmr|orf, hypothetical protein [Z4941]
yhjJ	-2.92	9.88	-1.55	10.65	0.0	9.11	0.2	0.00038434	0.00332667	0.03151296	0.49269116	A_07_P039556	c_4339	cmr|Protein yhjJ precursor [c_4339]
yhjN	+4.15	10.13	+2.05	9.10	0.1	11.16	0.1	0.00005806	0.00088921	0.01883829	0.36752169	A_07_P039562	c_4344	cmr|Cyclic di-GMP binding protein precursor [c_4344]
yhjQ	-4.94	8.69	-2.30	9.85	0.1	7.54	0.2	0.00007474	0.00106206	0.00653900	0.26329923	A_07_P007604	b3534	cmr|orf, hypothetical protein [b3534]
yhjR	-2.21	8.81	-1.14	9.38	0.0	8.24	0.2	0.00324869	0.01697555	0.06865917	0.65832521	A_07_P007606	b3535	cmr|orf, hypothetical protein [b3535]
yiaC	-3.25	9.95	-1.70	10.80	0.1	9.10	0.2	0.00052103	0.00417039	0.02302591	0.45201995	A_07_P007676	b3550	cmr|orf, hypothetical protein [b3550]
yiaC	-2.30	9.43	-1.20	10.03	0.1	8.83	0.2	0.00280947	0.01508605	0.06133956	0.63409718	A_07_P039621	c_4368	cmr|Hypothetical acetyltransferase yiaC [c_4368]
yiaK	+7.61	9.15	+2.93	7.69	0.0	10.61	0.2	0.00002277	0.00051605	0.00627541	0.22712644	A_07_P007803	b3575	cmr|putative dehydrogenase [b3575]
yiaL	+2.99	7.98	+1.58	7.19	0.2	8.77	0.3	0.00240058	0.01333149	0.03350737	0.46684956	A_07_P039647	c_4397	cmr|Hypothetical protein yiaL [c_4397]
yiaN	+5.19	7.60	+2.38	6.41	0.0	8.79	0.0	0.00002263	0.00051412	0.01328697	0.30628111	A_07_P039665	c_4400	cmr|Hypothetical protein yiaN [c_4400]
yibF	+3.61	8.42	+1.85	7.49	0.0	9.34	0.1	0.00012641	0.00152739	0.02373793	0.40186022	A_07_P016905	b3592	cmr|putative S-transferase [b3592]
yibN	-2.62	11.03	-1.39	11.73	0.1	10.34	0.2	0.00107396	0.00728496	0.04356580	0.56545754	A_07_P017000	b3611	cmr|orf, hypothetical protein [b3611]
yibP	-2.58	9.86	-1.37	10.55	0.1	9.18	0.1	0.00062211	0.00475805	0.04642912	0.57512195	A_07_P049541	Z5040	cmr|putative membrane protein [Z5040]
yibP	-2.15	10.29	-1.11	10.85	0.1	9.74	0.2	0.00390144	0.01954942	0.07307609	0.66890116	A_07_P039761	c_4439	cmr|Hypothetical protein yibP [c_4439]
yicC	-3.31	8.95	-1.73	9.81	0.4	8.09	0.2	0.00228206	0.01285927	0.02169144	0.44525424	A_07_P017163	b3644	cmr|putative alpha helix protein [b3644]
yicC	-2.64	9.33	-1.40	10.03	0.1	8.63	0.3	0.00280264	0.01506775	0.04154217	0.55380186	A_07_P052202	Z5069	cmr|putative alpha helix protein [Z5069]
yicE	-2.03	8.03	-1.02	8.54	0.1	7.52	0.1	0.00304821	0.01612601	0.08563262	0.71295611	A_07_P017211	b3654	cmr|putative transport protein [b3654]
yicG	-2.81	7.75	-1.49	8.50	0.0	7.00	0.2	0.00056565	0.00443510	0.03562436	0.51503214	A_07_P017173	b3646	cmr|orf, hypothetical protein [b3646]
yicJ	+2.35	8.12	+1.23	7.51	0.1	8.74	0.1	0.00137138	0.00875546	0.05157774	0.56352533	A_07_P017227	b3657	cmr|putative permease [b3657]
yicR	-2.11	11.29	-1.08	11.83	0.1	10.76	0.1	0.00170739	0.01035884	0.07892403	0.68461708	A_07_P017134	b3638	cmr|DNA repair protein [b3638]
yidB	-5.22	9.64	-2.38	10.83	0.1	8.45	0.2	0.00007560	0.00107226	0.00564850	0.24566298	A_07_P005425	b3698	cmr|orf, hypothetical protein [b3698]
yidC	-4.00	12.29	-2.00	13.29	0.1	11.29	0.1	0.00008558	0.00116924	0.01228659	0.34604651	A_07_P005451	b3705	cmr|60 KD inner-membrane protein [b3705]
yidD	-3.98	13.15	-1.99	14.15	0.1	12.16	0.3	0.00026955	0.00258239	0.01276994	0.35710480	A_07_P021528	ABE-0285078	asap|predicted protein [b4557]
yidF	+2.35	9.32	+1.23	8.70	0.2	9.93	0.1	0.00265504	0.01444899	0.05069423	0.55774284	A_07_P041897	c_4598	cmr|Hypothetical protein yidF [c_4598]
yidF	+6.18	9.38	+2.63	8.07	0.0	10.69	0.0	0.00001300	0.00037693	0.00980628	0.26991259	A_07_P017305	b3674	cmr|putative transcriptional regulator [b3674]
yidF	+2.45	8.32	+1.29	7.68	0.1	8.97	0.2	0.00153183	0.00948854	0.04779281	0.54407086	A_07_P052222	Z5169	cmr|putative transcriptional regulator [Z5169]
yidS	-4.72	7.92	-2.24	9.04	0.0	6.80	0.2	0.00006527	0.00096672	0.00762703	0.28658711	A_07_P039007	c_4611	cmr|Hypothetical protein yidS precursor [c_4611]
yidS	-3.34	7.87	-1.74	8.74	0.1	7.00	0.3	0.00063858	0.00487104	0.02212779	0.44664103	A_07_P051822	Z5186	cmr|orf, hypothetical protein [Z5186]
yieF	+4.26	11.76	+2.09	10.71	0.2	12.80	0.1	0.00011640	0.00144104	0.01841146	0.36415829	A_07_P005486	b3713	cmr|orf, hypothetical protein [b3713]
yieF	+2.69	11.78	+1.43	11.07	0.3	12.49	0.1	0.00145871	0.00914250	0.03955729	0.49943063	A_07_P005486	b3713	cmr|orf, hypothetical protein [b3713]
yieF	+3.18	11.78	+1.67	10.94	0.2	12.61	0.2	0.00088336	0.00627318	0.02981072	0.43986879	A_07_P005486	b3713	cmr|orf, hypothetical protein [b3713]
yieG	-4.32	7.90	-2.11	8.96	0.1	6.85	0.1	0.00005935	0.00090106	0.00997396	0.31595573	A_07_P005491	b3714	cmr|putative membrane / transport protein [b3714]
yieG	-6.48	8.68	-2.70	10.03	0.1	7.34	0.1	0.00002334	0.00052262	0.00284235	0.17480469	A_07_P039030	c_4637	cmr|Hypothetical protein yieG [c_4637]
yieH	-3.27	10.58	-1.71	11.44	0.0	9.73	0.2	0.00023070	0.00234535	0.02317137	0.45149752	A_07_P049923	Z5210	cmr|putative phosphatase [Z5210]
yieH	-3.91	10.10	-1.97	11.08	0.0	9.12	0.1	0.00007617	0.00107840	0.01334794	0.36484375	A_07_P039031	c_4638	cmr|Hypothetical protein yieH [c_4638]
yifK	+4.02	8.75	+2.01	7.75	0.2	9.76	0.1	0.00014552	0.00167812	0.01996634	0.37646707	A_07_P041930	c_4717	cmr|Probable transport protein yifK [c_4717]
yifL	+3.39	10.08	+1.76	9.20	0.0	10.96	0.1	0.00012425	0.00150779	0.02629954	0.41739919	A_07_P010621	ABE-0285079	asap|predicted lipoprotein [b4558]
yigB	+2.12	9.06	+1.08	8.52	0.1	9.60	0.2	0.00272556	0.01476135	0.06292302	0.62620733	A_07_P049994	Z5329	cmr|putative phosphatase [Z5329]
yigB	+2.92	8.81	+1.55	8.04	0.0	9.58	0.3	0.00149408	0.00931596	0.03450711	0.47200695	A_07_P039117	c_4733	cmr|Hypothetical protein yigB [c_4733]
yigI	-7.10	9.62	-2.83	11.04	0.1	8.21	0.3	0.00004591	0.00075849	0.00217734	0.15944186	A_07_P005970	b3820	cmr|orf, hypothetical protein [b3820]

yigM	-2.80	8.05	-1.49	8.79	0.1	7.30	0.0	0.00033181	0.00301100	0.03650152	0.52148820	A_07_P018941	b3827	cmr|orf, hypothetical protein [b3827]
yigP	-3.41	12.93	-1.77	13.81	0.0	12.04	0.0	0.00010767	0.00136707	0.01995363	0.42799728	A_07_P018976	b3834	cmr|orf, hypothetical protein [b3834]
yigZ	-2.46	10.44	-1.30	11.09	0.1	9.79	0.4	0.00673769	0.03024757	0.05218750	0.60193407	A_07_P041354	c_4795	cmr|Hypothetical protein yigZ [c_4795]
yihD	+57.91	10.37	+5.86	7.44	0.3	13.30	0.0	0.00000130	0.00014643	0.00009273	0.02807692	A_07_P019075	b3858	cmr|orf, hypothetical protein [b3858]
yihI	+3.61	8.22	+1.85	7.30	0.1	9.15	0.1	0.00011197	0.00140397	0.02391832	0.40231838	A_07_P019108	b3866	cmr|orf, hypothetical protein [b3866]
yihT	+2.25	7.47	+1.17	6.89	0.0	8.06	0.1	0.00126308	0.00826163	0.05549987	0.58683009	A_07_P039333	c_4827	cmr|Hypothetical protein yihT [c_4827]
yihX	+6.74	10.44	+2.75	9.06	0.1	11.82	0.0	0.00001153	0.00035443	0.00812309	0.25027397	A_07_P019205	b3885	cmr|putative phosphatase [b3885]
yihY	+2.23	7.81	+1.16	7.23	0.1	8.39	0.1	0.00197782	0.01153289	0.05572726	0.58686957	A_07_P019210	b3886	cmr|tRNA processing exoribonuclease BN [b3886]
yiiD	+3.47	8.81	+1.80	7.91	0.2	9.71	0.3	0.00096774	0.00673864	0.02572917	0.41419223	A_07_P019216	b3888	cmr|putative acetyltransferase [b3888]
yiiD	+5.84	9.73	+2.55	8.46	0.3	11.01	0.2	0.00014870	0.00170389	0.01047764	0.27724370	A_07_P041956	c_4835	cmr|Hypothetical protein yiiD [c_4835]
yiiQ	+4.72	8.60	+2.24	7.48	0.1	9.72	0.0	0.00004310	0.00073284	0.01545097	0.33368999	A_07_P019377	b3920	cmr|orf, hypothetical protein [b3920]
yiiS	+7.16	9.07	+2.84	7.65	0.1	10.49	0.0	0.00001394	0.00039090	0.00723450	0.24182590	A_07_P039426	c_4874	cmr|Hypothetical protein yiiS [c_4874]
yiiS	+6.97	8.51	+2.80	7.11	0.0	9.91	0.2	0.00002183	0.00050245	0.00760861	0.24698969	A_07_P019386	b3922	cmr|orf, hypothetical protein [b3922]
yiiS	+3.84	8.65	+1.94	7.68	0.3	9.62	0.3	0.00113729	0.00761611	0.02170478	0.38787741	A_07_P047106	Z5467	cmr|orf, hypothetical protein [Z5467]
yiiT	+6.12	10.22	+2.61	8.92	0.1	11.53	0.1	0.00002024	0.00047565	0.00992188	0.27166957	A_07_P047111	Z5468	cmr|putative regulator [Z5468]
yiiT	+5.50	10.20	+2.46	8.97	0.0	11.42	0.2	0.00003758	0.00066784	0.01191883	0.29093023	A_07_P039431	c_4875	cmr|Hypothetical protein yiiT [c_4875]
yiiU	+2.41	12.47	+1.27	11.84	0.1	13.11	0.0	0.00087051	0.00620147	0.04843814	0.54667384	A_07_P019420	b3928	cmr|orf, hypothetical protein [b3928]
yiiX	-2.42	9.73	-1.27	10.37	0.1	9.09	0.1	0.00092517	0.00650555	0.05448869	0.60885025	A_07_P052282	Z5492	cmr|orf, hypothetical protein [Z5492]
yiiX	-2.83	9.04	-1.50	9.79	0.0	8.29	0.0	0.00026444	0.00255503	0.03553163	0.51511050	A_07_P041984	c_4890	cmr|Hypothetical protein yiiX precursor [c_4890]
yiiX	-2.52	7.77	-1.33	8.44	0.0	7.11	0.3	0.00215837	0.01229844	0.04797129	0.57963162	A_07_P019465	b3937	cmr|orf, hypothetical protein [b3937]
yijD	-2.06	10.03	-1.04	10.55	0.0	9.51	0.1	0.00177996	0.01067090	0.08304433	0.70330823	A_07_P052298	Z5525	cmr|orf, hypothetical protein [Z5525]
yijD	-2.84	9.98	-1.50	10.73	0.0	9.22	0.1	0.00027919	0.00265270	0.03526169	0.51403704	A_07_P008525	b3964	cmr|orf, hypothetical protein [b3964]
yijF	-4.01	12.81	-2.01	13.81	0.2	11.80	0.1	0.00014344	0.00166295	0.01215955	0.34555957	A_07_P039476	c_4902	cmr|Hypothetical protein yijF precursor [c_4902]
yijF	-2.93	13.33	-1.55	14.11	0.4	12.56	0.2	0.00300981	0.01597063	0.03222116	0.49636986	A_07_P019499	b3944	cmr|orf, hypothetical protein [b3944]
yjaA	-8.67	9.50	-3.12	11.06	0.1	7.94	0.2	0.00001695	0.00042762	0.00116235	0.10892857	A_07_P008699	b4011	cmr|orf, hypothetical protein [b4011]
yjaB	-2.25	12.84	-1.17	13.43	0.1	12.26	0.3	0.00382888	0.01930258	0.06768166	0.65817171	A_07_P008704	b4012	cmr|orf, hypothetical protein [b4012]
yjbG	-2.90	11.51	-1.53	12.28	0.1	10.74	0.3	0.00128475	0.00835485	0.03364774	0.50452381	A_07_P008783	b4028	cmr|orf, hypothetical protein [b4028]
yjbJ	-2.91	9.61	-1.54	10.38	0.0	8.84	0.0	0.00023779	0.00238308	0.03281885	0.50116392	A_07_P008870	b4045	cmr|orf, hypothetical protein [b4045]
yjbJ	-5.06	10.82	-2.34	11.99	0.1	9.65	0.0	0.00002780	0.00057043	0.00612741	0.25057143	A_07_P037555	c_5016	cmr|Protein yjbJ [c_5016]
yjbQ	+8.15	9.58	+3.03	8.06	0.2	11.09	0.3	0.00010056	0.00131088	0.00553544	0.21625310	A_07_P008923	b4056	cmr|orf, hypothetical protein [b4056]
yjcB	-13.32	9.80	-3.74	11.66	0.1	7.93	0.4	0.00003444	0.00064547	0.00028392	0.05258824	A_07_P008942	b4060	cmr|orf, hypothetical protein [b4060]
yjcB	-9.22	9.77	-3.20	11.37	0.1	8.17	0.0	0.00000791	0.00029735	0.00098260	0.10245033	A_07_P035567	c_5050	cmr|Hypothetical protein yjcB [c_5050]
yjcH	+8.16	10.05	+3.03	8.54	0.1	11.57	0.1	0.00001465	0.00039588	0.00548653	0.21595000	A_07_P037683	c_5063	cmr|Hypothetical protein yjcH [c_5063]
yjcH	+9.03	10.45	+3.17	8.87	0.0	12.04	0.2	0.00001032	0.00033778	0.00445630	0.19652661	A_07_P037683	c_5063	cmr|Hypothetical protein yjcH [c_5063]
yjcH	+6.25	10.06	+2.64	8.74	0.1	11.38	0.3	0.00013415	0.00158449	0.00938834	0.26777174	A_07_P037683	c_5063	cmr|Hypothetical protein yjcH [c_5063]
yjcH	+3.03	10.08	+1.60	9.28	0.1	10.88	0.0	0.00021626	0.00223270	0.03239012	0.45858813	A_07_P047279	Z5667	cmr|orf, hypothetical protein [Z5667]
yjcH	+6.90	10.18	+2.79	8.79	0.1	11.58	0.1	0.00001464	0.00039588	0.00793890	0.24848907	A_07_P037683	c_5063	cmr|Hypothetical protein yjcH [c_5063]
yjcH	+8.19	10.28	+3.03	8.76	0.0	11.79	0.0	0.00000608	0.00026402	0.00546113	0.21603015	A_07_P037683	c_5063	cmr|Hypothetical protein yjcH [c_5063]
yjcO	-2.37	9.40	-1.25	10.03	0.0	8.78	0.2	0.00160046	0.00984285	0.05652820	0.61675676	A_07_P009034	b4078	cmr|orf, hypothetical protein [b4078]
yjcO	-3.31	9.53	-1.73	10.39	0.1	8.66	0.5	0.00382251	0.01927663	0.02297447	0.45213750	A_07_P009034	b4078	cmr|orf, hypothetical protein [b4078]
yjcO	-2.20	9.75	-1.14	10.32	0.1	9.18	0.0	0.00145015	0.00910331	0.07175622	0.66650737	A_07_P009034	b4078	cmr|orf, hypothetical protein [b4078]
yjcO	-2.59	9.56	-1.37	10.25	0.0	8.88	0.1	0.00049446	0.00400242	0.04552210	0.57153110	A_07_P009034	b4078	cmr|orf, hypothetical protein [b4078]
yjcW	+3.33	9.90	+1.74	9.03	0.2	10.77	0.1	0.00036132	0.00318459	0.02783664	0.43008832	A_07_P037736	c_5093	cmr|D-allose transport ATP-binding protein alsA [c_5093]
yjdB	+3.90	7.99	+1.96	7.01	0.2	8.97	0.0	0.00017791	0.00194647	0.02096164	0.38329849	A_07_P037782	c_5119	cmr|Hypothetical protein yjdB [c_5119]
yjdC	+3.89	9.01	+1.96	8.04	0.0	9.99	0.7	0.00614440	0.02803982	0.02156885	0.38764840	A_07_P011409	b4135	cmr|orf, hypothetical protein [b4135]
yjdJ	+3.07	8.54	+1.62	7.73	0.0	9.35	0.2	0.00042875	0.00360786	0.03168699	0.45229374	A_07_P047348	Z5729	cmr|orf, hypothetical protein [Z5729]
yjdJ	+2.50	8.69	+1.32	8.03	0.3	9.35	0.2	0.00429023	0.02103562	0.04651677	0.53810434	A_07_P011374	b4127	cmr|orf, hypothetical protein [b4127]
yjdN	-3.25	8.55	-1.70	9.40	0.1	7.70	0.1	0.00028131	0.00266319	0.02304560	0.45128109	A_07_P011271	b4107	cmr|orf, hypothetical protein [b4107]
yjeI	+2.41	10.90	+1.27	10.27	0.0	11.53	0.1	0.00100261	0.00691416	0.04862170	0.54756795	A_07_P011455	b4144	cmr|orf, hypothetical protein [b4144]
yjeK	-2.26	8.84	-1.18	9.43	0.1	8.25	0.1	0.00101788	0.00700111	0.06542048	0.65354061	A_07_P011461	b4146	cmr|orf, hypothetical protein [b4146]
yjeP	+2.11	8.61	+1.08	8.07	0.1	9.15	0.1	0.00218653	0.01242767	0.06240663	0.62661352	A_07_P036943	c_5245	cmr|Hypothetical protein yjeP precursor [c_5245]
yjeS	-13.38	9.63	-3.74	11.50	0.0	7.76	0.3	0.00001171	0.00035727	0.00027248	0.05296296	A_07_P011550	b4166	cmr|orf, hypothetical protein [b4166]
yjfN	+10.05	10.52	+3.33	8.85	0.0	12.18	0.2	0.00000687	0.00027524	0.00354992	0.17630915	A_07_P011660	b4188	cmr|orf, hypothetical protein [b4188]
yjfO	+14.67	12.48	+3.87	10.55	0.1	14.42	0.1	0.00000318	0.00019528	0.00168064	0.12364486	A_07_P035589	c_5277	cmr|Hypothetical lipoprotein yjfO precursor [c_5277]
yjfO	+14.12	10.76	+3.82	8.85	0.1	12.67	0.1	0.00000259	0.00018134	0.00177846	0.12500000	A_07_P011661	b4189	cmr|orf, hypothetical protein [b4189]
yjfO	+13.79	11.22	+3.79	9.33	0.1	13.12	0.1	0.00000251	0.00018118	0.00191311	0.12817021	A_07_P010658	b4189	cmr|orf, hypothetical protein [b4189]
yjfO	+10.73	10.81	+3.42	9.10	0.2	12.52	0.1	0.00001064	0.00034402	0.00313580	0.16511706	A_07_P048630	Z5796	cmr|orf, hypothetical protein [Z5796]
yjgA	-8.94	12.38	-3.16	13.96	0.0	10.80	0.1	0.00000643	0.00027019	0.00107215	0.10484472	A_07_P037054	c_5332	cmr|Hypothetical protein yjgA [c_5332]
yjgA	-8.01	12.20	-3.00	13.71	0.0	10.70	0.0	0.00000679	0.00027353	0.00149835	0.12682796	A_07_P003722	b4234	cmr|putative alpha helix protein [b4234]
yjgF	+110.93	12.08	+6.79	8.68	0.2	15.48	0.1	0.00000027	0.00009047	0.00001651	0.00962963	A_07_P037066	c_5342	cmr|Protein yjgF [c_5342]
yjgF	+37.72	10.37	+5.24	7.75	0.1	12.99	0.0	0.00000037	0.00009117	0.00019563	0.03948718	A_07_P003767	b4243	cmr|orf, hypothetical protein [b4243]
yjgP	-3.80	10.26	-1.92	11.22	0.1	9.29	0.3	0.00036499	0.00320669	0.01434832	0.37839196	A_07_P003851	b4261	cmr|orf, hypothetical protein [b4261]
yjgQ	-4.61	11.04	-2.21	12.15	0.0	9.94	0.0	0.00003565	0.00065418	0.00825394	0.29601367	A_07_P003856	b4262	cmr|orf, hypothetical protein [b4262]
yjgR	+2.67	8.58	+1.42	7.88	0.5	9.29	0.1	0.00753978	0.03308425	0.04210493	0.50992308	A_07_P003863	b4263	cmr|orf, hypothetical protein [b4263]
yjgR	+8.29	9.26	+3.05	7.74	0.0	10.79	0.3	0.00003867	0.00068020	0.00540904	0.21614213	A_07_P037116	c_5364	cmr|Hypothetical protein yjgR [c_5364]
yjgZ	-3.21	7.74	-1.68	8.58	0.3	6.90	0.1	0.00142501	0.00900660	0.02378747	0.45727717	A_07_P003917	b4277	cmr|orf, hypothetical protein [b4277]
yjhH	+3.35	8.28	+1.74	7.41	0.2	9.15	0.2	0.00060056	0.00465408	0.02731707	0.42414201	A_07_P004016	b4298	cmr|putative lyase/synthase [b4298]
yjhQ	+2.16	8.14	+1.11	7.58	0.2	8.70	0.0	0.00338434	0.01749279	0.06035950	0.61468305	A_07_P004061	b4307	cmr|orf, hypothetical protein [b4307]
yjhQ	+2.01	8.41	+1.00	7.91	0.1	8.91	0.0	0.00208579	0.01200683	0.06830793	0.65416058	A_07_P004061	b4307	cmr|orf, hypothetical protein [b4307]
yjhR	-3.06	11.74	-1.61	12.55	0.1	10.94	0.2	0.00039615	0.00339887	0.02780170	0.48259096	A_07_P004068	b4308	cmr|putative frameshift suppressor [b4308]
yjhU	-2.99	9.58	-1.58	10.37	0.1	8.79	0.1	0.00032994	0.00300092	0.02995363	0.48869430	A_07_P004004	b4295	cmr|orf, hypothetical protein [b4295]
yjiD	-2.42	9.09	-1.28	9.73	0.0	8.45	0.1	0.00070062	0.00524016	0.05474530	0.60955446	A_07_P004159	b4326	cmr|orf, hypothetical protein [b4326]
yjiG	+7.58	8.91	+2.92	7.45	0.0	10.37	0.2	0.00001382	0.00039022	0.00636242	0.22869863	A_07_P004174	b4329	cmr|orf, hypothetical protein [b4329]
yjiH	+3.60	7.92	+1.85	6.99	0.1	8.84	0.1	0.00017705	0.00193940	0.02394118	0.40184435	A_07_P004179	b4330	cmr|orf, hypothetical protein [b4330]
yjiH	+2.07	7.34	+1.05	6.82	0.0	7.87	0.0	0.00159544	0.00981961	0.06466781	0.63672921	A_07_P037230	c_5410	cmr|Hypothetical protein yjiH [c_5410]
yjiL	+13.34	10.47	+3.74	8.60	0.2	12.34	0.0	0.00000681	0.00027353	0.00208270	0.13438525	A_07_P004196	b4334	cmr|putative enzyme [b4334]
yjiM	+17.34	9.70	+4.12	7.64	0.1	11.76	0.3	0.00000967	0.00032448	0.00109121	0.09706215	A_07_P004201	b4335	cmr|orf, hypothetical protein [b4335]
yjiP	-2.87	9.72	-1.52	10.48	0.2	8.96	0.5	0.00622849	0.02832506	0.03356136	0.50418893	A_07_P004217	b4338	cmr|orf, hypothetical protein [b4338]
yjiX	+2.72	9.38	+1.44	8.66	0.1	10.10	0.0	0.00042084	0.00355645	0.03882685	0.49537277	A_07_P017462	b4353	cmr|orf, hypothetical protein [b4353]
yjjI	+4.58	10.60	+2.19	9.51	0.0	11.70	0.4	0.00072461	0.00537873	0.01614774	0.33942590	A_07_P017585	b4380	cmr|orf, hypothetical protein [b4380]
yjjK	+3.33	9.90	+1.73	9.03	0.1	10.77	0.0	0.00015728	0.00177863	0.02751905	0.42559921	A_07_P017637	b4391	cmr|putative ATP-binding component of a transport system [b4391]
yjjK	+3.40	9.91	+1.76	9.03	0.0	10.79	0.1	0.00013097	0.00156505	0.02612868	0.41636640	A_07_P017637	b4391	cmr|putative ATP-binding component of a transport system [b4391]
yjjK	+2.81	9.67	+1.49	8.92	0.0	10.41	0.0	0.00025731	0.00252041	0.03728404	0.48592715	A_07_P017637	b4391	cmr|putative ATP-binding component of a transport system [b4391]
yjjK	+2.44	9.76	+1.28	9.11	0.0	10.40	0.3	0.00238542	0.01327539	0.04710112	0.54010197	A_07_P017637	b4391	cmr|putative ATP-binding component of a transport system [b4391]
yjjM	+2.33	7.83	+1.22	7.22	0.3	8.44	0.1	0.00597726	0.02742014	0.05313643	0.57300000	A_07_P017484	b4357	cmr|orf, hypothetical protein [b4357]
yjjN	-2.10	7.67	-1.07	8.21	0.0	7.14	0.2	0.00246713	0.01361462	0.08094385	0.69410675	A_07_P017488	b4358	cmr|putative oxidoreductase [b4358]
yjjN	-2.34	7.49	-1.22	8.10	0.1	6.87	0.2	0.00149103	0.00930330	0.05937500	0.62780390	A_07_P017488	b4358	cmr|putative oxidoreductase [b4358]
yjjQ	-2.52	7.22	-1.33	7.89	0.2	6.56	0.1	0.00085792	0.00613593	0.04927718	0.58552453	A_07_P042086	c_5444	cmr|Hypothetical protein yjjQ [c_5444]
yjjQ	-2.88	7.53	-1.52	8.29	0.0	6.76	0.1	0.00030821	0.00285608	0.03381606	0.50608365	A_07_P017521	b4365	cmr|putative regulator [b4365]
yjjX	-3.67	10.52	-1.88	11.46	0.2	9.58	0.6	0.00544180	0.02540548	0.01692264	0.39647321	A_07_P017655	b4394	cmr|orf, hypothetical protein [b4394]
ykfJ	-14.63	11.16	-3.87	13.10	0.0	9.23	0.1	0.00000220	0.00017938	0.00015816	0.04220339	A_07_P007889	b0235	cmr|orf, hypothetical protein [b0235]
ykgF	+2.22	8.53	+1.15	7.96	0.0	9.11	0.1	0.00135330	0.00867719	0.05665142	0.59106693	A_07_P008226	b0307	cmr|orf, hypothetical protein [b0307]
ykgJ	-4.99	8.26	-2.32	9.42	0.3	7.10	0.1	0.00021535	0.00222616	0.00658854	0.26327411	A_07_P008131	b0288	cmr|putative ferredoxin [b0288]
ykgL	-2.62	7.88	-1.39	8.57	0.1	7.18	0.1	0.00043337	0.00363502	0.04454141	0.56966694	A_07_P043583	Z0363	cmr|orf, hypothetical protein [Z0363]
ykiA	-2.28	7.58	-1.19	8.17	0.2	6.98	0.3	0.00509450	0.02409365	0.06461382	0.64918953	A_07_P021074	b0392	cmr|orf, hypothetical protein [b0392]
ylaB	-2.32	11.16	-1.21	11.76	0.1	10.55	0.3	0.00356919	0.01826240	0.06221418	0.64019608	A_07_P021391	b0457	cmr|orf, hypothetical protein [b0457]
ylaC	-17.67	11.77	-4.14	13.84	0.0	9.70	0.1	0.00000147	0.00015430	0.00008257	0.02765957	A_07_P021396	b0458	cmr|orf, hypothetical protein [b0458]
ylbA	-2.79	11.89	-1.48	12.63	0.3	11.15	0.5	0.01100143	0.04481409	0.03635226	0.52077343	A_07_P016365	b0515	cmr|orf, hypothetical protein [b0515]
ylbA	+2.28	7.81	+1.19	7.21	0.1	8.40	0.3	0.00398146	0.01988707	0.05559832	0.58668901	A_07_P031240	c_0629	cmr|Hypothetical protein ylbA [c_0629]
yliJ	+2.34	10.18	+1.22	9.57	0.2	10.79	0.1	0.00179234	0.01071324	0.05244919	0.56948966	A_07_P004644	b0838	cmr|putative transferase [b0838]
ymcE	+5.31	9.52	+2.41	8.31	0.0	10.72	0.1	0.00002644	0.00055821	0.01285188	0.30020772	A_07_P001189	b0991	cmr|suppresses fabA and ts growth mutation [b0991]
ymdF	-3.45	12.20	-1.79	13.09	0.0	11.30	0.1	0.00017632	0.00193718	0.01982724	0.42586630	A_07_P010350	ABE-0285039	asap|conserved protein [b4518]
ymfA	-2.33	9.09	-1.22	9.70	0.0	8.48	0.2	0.00143162	0.00903332	0.06005208	0.63114820	A_07_P040833	c_1397	cmr|Hypothetical protein ymfA [c_1397]
ymfC	-13.49	12.64	-3.75	14.52	0.0	10.76	0.2	0.00000387	0.00021100	0.00023628	0.05095890	A_07_P050485	Z1864	cmr|orf, hypothetical protein [Z1864]
ymfE	+2.33	7.73	+1.22	7.12	0.0	8.34	0.3	0.00552863	0.02574469	0.05487043	0.58291498	A_07_P006641	b1138	cmr|orf, hypothetical protein [b1138]
ymfM	-2.42	9.34	-1.28	9.98	0.4	8.71	0.0	0.00461663	0.02229577	0.05466019	0.60946884	A_07_P006694	b1148	cmr|orf, hypothetical protein [b1148]
ymgA	+9.62	8.31	+3.27	6.68	0.1	9.94	0.1	0.00000952	0.00032326	0.00395770	0.18489614	A_07_P006777	b1165	cmr|orf, hypothetical protein [b1165]
ymgB	+13.87	10.55	+3.79	8.65	0.2	12.44	0.1	0.00000533	0.00024927	0.00187246	0.12817391	A_07_P038703	c_1609	cmr|Hypothetical protein ymgB [c_1609]
ymgD	-2.99	8.66	-1.58	9.45	0.3	7.87	0.4	0.00435791	0.02130114	0.02872015	0.48412206	A_07_P006807	b1171	cmr|orf, hypothetical protein [b1171]
ymgG	-2.31	13.07	-1.21	13.67	0.0	12.46	0.2	0.00174142	0.01050937	0.06127350	0.63508229	A_07_P006812	b1172	cmr|orf, hypothetical protein [b1172]
ymjA	+3.65	8.29	+1.87	7.36	0.3	9.22	0.1	0.00052907	0.00422398	0.02393610	0.40218783	A_07_P014457	b1295	cmr|orf, hypothetical protein [b1295]
ynaI	-4.06	10.96	-2.02	11.97	0.1	9.95	0.1	0.00011215	0.00140465	0.01176385	0.34945283	A_07_P014632	b1330	cmr|orf, hypothetical protein [b1330]
ynaJ	-2.45	9.31	-1.29	9.95	0.1	8.66	0.1	0.00080634	0.00582985	0.05284807	0.60336476	A_07_P014640	b1332	cmr|orf, hypothetical protein [b1332]
ynaK	-3.04	9.27	-1.60	10.08	0.3	8.47	0.5	0.00793023	0.03443287	0.02913872	0.48494715	A_07_P014801	b1365	cmr|orf, hypothetical protein [b1365]
ynbC	-3.19	8.10	-1.67	8.93	0.4	7.26	0.2	0.00275949	0.01490408	0.02472180	0.46061538	A_07_P009144	b1410	cmr|orf, hypothetical protein [b1410]
ynbE	+2.47	8.42	+1.30	7.76	0.2	9.07	0.2	0.00208822	0.01201539	0.04688262	0.53995611	A_07_P014888	b1382	cmr|orf, hypothetical protein [b1382]
ynbE	+3.63	8.03	+1.86	7.10	0.0	8.96	0.3	0.00039233	0.00337350	0.02389926	0.40242781	A_07_P034809	c_1829	cmr|Hypothetical protein ynbE [c_1829]
ynbE	-3.35	8.50	-1.74	9.37	0.7	7.63	0.0	0.00961277	0.04011225	0.02136751	0.44148294	A_07_P048215	Z2327	cmr|orf, hypothetical protein [Z2327]
yncC	+2.42	7.86	+1.28	7.22	0.2	8.49	0.0	0.00105148	0.00716564	0.04838097	0.54720546	A_07_P009341	b1450	cmr|orf, hypothetical protein [b1450]
yneH	-5.73	9.74	-2.52	11.00	0.1	8.49	0.0	0.00002969	0.00059702	0.00425877	0.21084906	A_07_P020270	b1524	cmr|putative glutaminase [b1524]
yneI	-5.98	8.80	-2.58	10.09	0.0	7.51	0.2	0.00003482	0.00064639	0.00372650	0.20092466	A_07_P035026	c_1948	cmr|Aldehyde-dehydrogenase like protein yneI [c_1948]
yneJ	-2.44	11.30	-1.29	11.94	0.0	10.65	0.5	0.00939886	0.03944965	0.05279726	0.60366013	A_07_P020279	b1526	cmr|putative transcriptional regulator LYSR-type [b1526]
ynfB	-3.70	14.20	-1.89	15.14	0.0	13.25	0.1	0.00010565	0.00135201	0.01541286	0.39138710	A_07_P020550	b1583	cmr|orf, hypothetical protein [b1583]
ynfD	+2.61	9.92	+1.39	9.22	0.1	10.61	0.2	0.00097747	0.00678238	0.04199441	0.51054826	A_07_P020563	b1586	cmr|orf, hypothetical protein [b1586]
ynfE	+10.61	8.58	+3.41	6.88	0.3	10.28	0.3	0.00005408	0.00084295	0.00327109	0.16775244	A_07_P020568	b1587	cmr|putative oxidoreductase, major subunit [b1587]
ynfE	+23.09	9.52	+4.53	7.25	0.0	11.78	0.0	0.00000072	0.00011611	0.00054306	0.07066116	A_07_P042301	c_1977	cmr|Putative dimethyl sulfoxide reductase chain ynfE precursor [c_1977]
ynfF	+13.00	10.39	+3.70	8.54	0.3	12.24	0.1	0.00001435	0.00039421	0.00214812	0.13637097	A_07_P020575	b1588	cmr|putative oxidoreductase, major subunit [b1588]
ynfG	+33.16	9.85	+5.05	7.32	0.3	12.37	0.0	0.00000201	0.00017324	0.00025915	0.04689655	A_07_P020576	b1589	cmr|putative oxidoreductase, Fe-S subunit [b1589]
ynfH	+4.67	9.30	+2.22	8.19	0.2	10.41	0.2	0.00013253	0.00157122	0.01583397	0.33824966	A_07_P020582	b1590	cmr|putative DMSO reductase anchor subunit [b1590]
ynfL	-2.95	10.89	-1.56	11.67	0.2	10.10	0.2	0.00106584	0.00723611	0.03123603	0.49128871	A_07_P020609	b1595	cmr|putative transcriptional regulator LYSR-type [b1595]
ynfM	-6.02	11.20	-2.59	12.49	0.4	9.90	0.6	0.00202157	0.01172718	0.00370363	0.20176471	A_07_P020611	b1596	cmr|putative transport protein [b1596]
yniA	+7.96	8.92	+2.99	7.42	0.0	10.41	0.1	0.00000713	0.00028183	0.00586636	0.22201923	A_07_P018674	b1725	cmr|orf, hypothetical protein [b1725]
yniC	-2.50	10.26	-1.32	10.92	0.1	9.60	0.1	0.00087236	0.00620908	0.04986979	0.58945195	A_07_P018685	b1727	cmr|putative phosphatase [b1727]
ynjA	+3.64	8.83	+1.86	7.90	0.1	9.76	0.1	0.00013197	0.00157032	0.02350864	0.40012973	A_07_P018811	b1753	cmr|orf, hypothetical protein [b1753]
ynjB	+7.57	8.56	+2.92	7.10	0.0	10.03	0.1	0.00001003	0.00033349	0.00636623	0.22831435	A_07_P018816	b1754	cmr|orf, hypothetical protein [b1754]
ynjC	+2.95	8.89	+1.56	8.11	0.0	9.67	0.3	0.00171500	0.01040100	0.03411395	0.46948427	A_07_P018825	b1755	cmr|putative transport system permease protein [b1755]
ynjD	+4.11	9.06	+2.04	8.04	0.2	10.08	0.1	0.00015339	0.00174491	0.01944550	0.37426650	A_07_P035219	c_2157	cmr|Hypothetical ABC transporter ATP-binding protein ynjD [c_2157]
ynjE	+3.87	9.08	+1.95	8.11	0.4	10.06	0.0	0.00061596	0.00473754	0.02156885	0.38720639	A_07_P018832	b1757	cmr|putative thiosulfate sulfur transferase [b1757]
yoaB	+2.88	8.79	+1.52	8.03	0.0	9.55	0.2	0.00046300	0.00382050	0.03568216	0.47811064	A_07_P015736	b1809	cmr|orf, hypothetical protein [b1809]
yoaH	-2.25	10.09	-1.17	10.68	0.1	9.51	0.1	0.00121567	0.00800615	0.06600673	0.65731183	A_07_P015748	b1811	cmr|orf, hypothetical protein [b1811]
yobA	+2.67	11.57	+1.42	10.86	0.1	12.28	0.1	0.00042765	0.00360242	0.03997015	0.49983320	A_07_P015882	b1841	cmr|orf, hypothetical protein [b1841]
yobB	+3.70	8.98	+1.89	8.04	0.1	9.92	0.0	0.00008430	0.00115817	0.02283664	0.39772124	A_07_P015892	b1843	cmr|orf, hypothetical protein [b1843]
yodA	+2.21	10.20	+1.15	9.63	0.1	10.77	0.2	0.00260551	0.01424838	0.05659108	0.59082891	A_07_P031409	c_2391	cmr|Hypothetical protein yodA [c_2391]
yoeB	-3.22	11.31	-1.68	12.15	0.0	10.47	0.3	0.00058336	0.00454897	0.02450394	0.45982122	A_07_P010484	ABE-0285060	asap|toxin of the YoeB-YefM toxin-antitoxin system [b4539]
yohK	-2.00	7.46	-1.00	7.96	0.0	6.96	0.1	0.00289781	0.01549700	0.08963923	0.72522097	A_07_P003473	b2142	cmr|putative seritonin transporter [b2142]
yohM	-2.52	10.19	-1.34	10.85	0.3	9.52	0.2	0.00333099	0.01730256	0.04836001	0.58076278	A_07_P045159	Z3274	cmr|orf, hypothetical protein [Z3274]
ypfG	-7.13	9.72	-2.83	11.14	0.0	8.30	0.5	0.00036855	0.00323256	0.00216972	0.15962617	A_07_P042601	c_2991	cmr|Hypothetical protein ypfG precursor [c_2991]
ypfG	-3.66	9.58	-1.87	10.51	0.1	8.64	0.3	0.00066166	0.00502036	0.01678227	0.39732331	A_07_P013386	b2466	cmr|orf, hypothetical protein [b2466]
yphE	-4.63	12.72	-2.21	13.82	0.0	11.61	0.5	0.00092300	0.00649607	0.00801131	0.29062212	A_07_P019639	b2547	cmr|putative ATP-binding component of a transport system [b2547]
yphF	-2.45	7.89	-1.29	8.53	0.2	7.24	0.1	0.00184092	0.01095687	0.05299289	0.60283237	A_07_P019645	b2548	cmr|putative LACI-type transcriptional regulator [b2548]
ypjC	-2.68	8.30	-1.42	9.01	0.1	7.59	0.2	0.00126964	0.00828397	0.04149009	0.55357627	A_07_P020112	b2650	cmr|orf, hypothetical protein [b2650]
ypjD	-2.03	9.68	-1.02	10.19	0.3	9.17	0.2	0.01168793	0.04685886	0.08783473	0.71912116	A_07_P019932	b2611	cmr|orf, hypothetical protein [b2611]
yqaA	-5.82	9.52	-2.54	10.79	0.1	8.25	0.3	0.00009310	0.00124213	0.00404535	0.20881967	A_07_P001920	b2689	cmr|orf, hypothetical protein [b2689]
yqaB	-11.46	10.89	-3.52	12.64	0.0	9.13	0.1	0.00000434	0.00022321	0.00049225	0.07110092	A_07_P030582	c_3247	cmr|Hypothetical protein yqaB [c_3247]
yqaB	-7.37	10.70	-2.88	12.15	0.0	9.26	0.3	0.00004996	0.00080150	0.00198869	0.15125604	A_07_P001923	b2690	cmr|putative phosphatase [b2690]
yqaE	-2.03	7.72	-1.02	8.23	0.0	7.21	0.2	0.00308395	0.01628119	0.08606834	0.71394099	A_07_P020182	b2666	cmr|orf, hypothetical protein [b2666]
yqcA	+3.93	9.67	+1.97	8.68	0.1	10.66	0.0	0.00007900	0.00110461	0.02063643	0.38133803	A_07_P002396	b2790	cmr|orf, hypothetical protein [b2790]
yqfA	+3.87	9.90	+1.95	8.93	0.1	10.88	0.1	0.00009804	0.00128741	0.02124238	0.38485616	A_07_P006461	b2899	cmr|putative oxidoreductase [b2899]
yqfE	-2.58	12.64	-1.37	13.33	0.2	11.96	0.1	0.00105556	0.00717873	0.04612551	0.57407115	A_07_P006538	b2915	cmr|orf, hypothetical protein [b2915]
yqgE	+4.49	10.84	+2.17	9.76	0.1	11.93	0.0	0.00005608	0.00086392	0.01670732	0.34474443	A_07_P011885	b2948	cmr|orf, hypothetical protein [b2948]
yqhA	-3.61	8.81	-1.85	9.74	0.0	7.89	0.0	0.00008169	0.00112858	0.01689024	0.39689552	A_07_P012144	b3002	cmr|orf, hypothetical protein [b3002]
yqhD	+6.00	10.92	+2.59	9.63	0.2	12.21	0.0	0.00003016	0.00060327	0.01020325	0.27412969	A_07_P012186	b3011	cmr|putative oxidoreductase [b3011]
yqiC	-2.16	7.33	-1.11	7.88	0.4	6.77	0.0	0.00861748	0.03680780	0.07491806	0.67285225	A_07_P012342	b3042	cmr|orf, hypothetical protein [b3042]
yqjA	-7.90	11.35	-2.98	12.84	0.0	9.86	0.1	0.00000789	0.00029735	0.00155932	0.12853403	A_07_P000202	b3095	cmr|orf, hypothetical protein [b3095]
yqjA	-10.68	10.86	-3.42	12.57	0.0	9.16	0.2	0.00000805	0.00029787	0.00056085	0.07745614	A_07_P041768	c_3853	cmr|Hypothetical protein yqjA [c_3853]
yqjB	-3.39	10.51	-1.76	11.39	0.1	9.63	0.0	0.00019092	0.00203786	0.02056847	0.43408847	A_07_P000206	b3096	cmr|orf, hypothetical protein [b3096]
yqjC	+3.85	10.59	+1.94	9.62	0.1	11.56	0.0	0.00007493	0.00106375	0.02136052	0.38566514	A_07_P000212	b3097	cmr|orf, hypothetical protein [b3097]
yqjF	-2.15	8.16	-1.11	8.71	0.1	7.61	0.1	0.00162297	0.00995666	0.07607724	0.67403489	A_07_P034067	c_3859	cmr|Hypothetical protein yqjF [c_3859]
yraM	+2.96	9.56	+1.57	8.78	0.2	10.34	0.1	0.00061811	0.00474475	0.03350673	0.46725421	A_07_P000458	b3147	cmr|putative glycosylase [b3147]
yraN	+2.46	9.39	+1.30	8.74	0.2	10.03	0.1	0.00127173	0.00829415	0.04690231	0.53978801	A_07_P000461	b3148	cmr|orf, hypothetical protein [b3148]
yraP	+5.60	10.47	+2.49	9.23	0.0	11.72	0.0	0.00001816	0.00044199	0.01163745	0.28808176	A_07_P000475	b3150	cmr|putative periplasmic protein [b3150]
yraQ	-3.03	8.54	-1.60	9.34	0.1	7.74	0.1	0.00024815	0.00245286	0.02878049	0.48410256	A_07_P000479	b3151	cmr|orf, hypothetical protein [b3151]
yraR	-2.01	8.10	-1.01	8.60	0.1	7.59	0.1	0.00233100	0.01306025	0.08910315	0.72385965	A_07_P045936	Z4511	cmr|orf, hypothetical protein [Z4511]
yraR	-2.29	9.00	-1.19	9.59	0.0	8.40	0.0	0.00078631	0.00572006	0.06444487	0.64873402	A_07_P000481	b3152	cmr|orf, hypothetical protein [b3152]
yrbD	-3.18	10.97	-1.67	11.80	0.0	10.13	0.0	0.00014328	0.00166233	0.02484184	0.46175915	A_07_P012441	b3193	cmr|orf, hypothetical protein [b3193]
yrbE	-9.85	10.30	-3.30	11.95	0.1	8.65	0.0	0.00000421	0.00022113	0.00079649	0.09153285	A_07_P012449	b3194	cmr|orf, hypothetical protein [b3194]
yrbF	-4.25	10.68	-2.09	11.73	0.2	9.64	0.2	0.00021229	0.00220037	0.01040523	0.32375494	A_07_P012451	b3195	cmr|putative ATP-binding component of a transport system [b3195]
yrbK	+3.07	12.62	+1.62	11.81	0.0	13.42	0.2	0.00032381	0.00296307	0.03169334	0.45197464	A_07_P012474	b3199	cmr|orf, hypothetical protein [b3199]
yrdB	-2.19	10.01	-1.13	10.58	0.3	9.45	0.1	0.00694979	0.03099647	0.07273247	0.66808635	A_07_P012835	b3280	cmr|orf, hypothetical protein [b3280]
yrdD	+2.59	9.07	+1.37	8.38	0.0	9.76	0.2	0.00102928	0.00705795	0.04252731	0.51110687	A_07_P012850	b3283	cmr|putative DNA topoisomerase [b3283]
yrfD	+2.00	7.92	+1.00	7.42	0.1	8.42	0.0	0.00283633	0.01520438	0.06942327	0.65883062	A_07_P034394	c_4165	cmr|Hypothetical protein yrfD [c_4165]
yrfF	-2.69	9.87	-1.43	10.59	0.0	9.16	0.2	0.00085218	0.00610680	0.04026423	0.54601206	A_07_P034400	c_4169	cmr|Putative membrane protein igaA homolog [c_4169]
yrfF	-2.08	9.23	-1.06	9.76	0.0	8.70	0.2	0.00385423	0.01938688	0.07931466	0.68649258	A_07_P018019	b3398	cmr|putative dehydrogenase [b3398]
yrhB	-3.20	9.36	-1.68	10.20	0.1	8.52	0.3	0.00088116	0.00626219	0.02488504	0.46201651	A_07_P018247	b3446	cmr|orf, hypothetical protein [b3446]
ysaA	-2.39	8.50	-1.26	9.13	0.0	7.87	0.1	0.00084127	0.00603417	0.05697218	0.61817367	A_07_P007793	b3573	cmr|orf, hypothetical protein [b3573]
ytfH	-4.05	10.26	-2.02	11.27	0.2	9.25	0.3	0.00055626	0.00437668	0.01212907	0.34594203	A_07_P003611	b4212	cmr|orf, hypothetical protein [b4212]
ytfH	-5.17	9.75	-2.37	10.94	0.0	8.57	0.3	0.00013160	0.00156730	0.00581110	0.24727027	A_07_P035591	c_5311	cmr|Hypothetical protein ytfH [c_5311]
ytfL	-3.05	8.78	-1.61	9.59	0.1	7.98	0.1	0.00025635	0.00251460	0.02837970	0.48251620	A_07_P003641	b4218	cmr|putative transport protein [b4218]
ytfQ	+11.85	10.11	+3.57	8.32	0.2	11.89	0.7	0.00049479	0.00400307	0.00248666	0.14393382	A_07_P037048	c_5325	cmr|ABC transporter Periplasmic binding protein ytfQ precursor [c_5325]
ytfQ	+4.02	8.57	+2.01	7.57	0.0	9.57	0.3	0.00024508	0.00242982	0.02024454	0.37853919	A_07_P003689	b4227	cmr|putative LACI-type transcriptional regulator [b4227]
Z0040	+4.10	9.42	+2.04	8.41	0.3	10.44	0.2	0.00039695	0.00340023	0.01941565	0.37460784	A_07_P048486	Z0040	cmr|transcriptional regulator of cai operon [Z0040]
Z0056	-3.07	11.88	-1.62	12.69	0.1	11.07	0.2	0.00047195	0.00387253	0.02710747	0.47845291	A_07_P049148	Z0056	cmr|putative antitoxin of gyrase inhibiting toxin-antitoxin system [Z0056]
Z0202	-3.09	10.92	-1.63	11.74	0.0	10.11	0.1	0.00024376	0.00242279	0.02732914	0.47914254	A_07_P049248	Z0202	cmr|orf Unknown function [Z0202]
Z0309	-2.94	12.79	-1.56	13.56	0.0	12.01	0.1	0.00026520	0.00255839	0.03123984	0.49085828	A_07_P043395	Z0309	cmr|putative cI repressor protein for prophage CP-933H [Z0309]
Z0312	-4.57	13.03	-2.19	14.12	0.0	11.93	0.1	0.00003731	0.00066523	0.00844131	0.29599109	A_07_P043406	Z0312	cmr|partial O replication protein for prophage CP-933H [Z0312]
Z0325	-2.77	11.23	-1.47	11.96	0.0	10.49	0.0	0.00028937	0.00271509	0.03794779	0.53012422	A_07_P043462	Z0325	cmr|unknown protein encoded in prophage CP-933I [Z0325]
Z0374	+2.95	7.71	+1.56	6.93	0.0	8.49	0.1	0.00025812	0.00252573	0.03377731	0.46812500	A_07_P043615	Z0374	cmr|orf Unknown function [Z0374]
Z0390	-3.16	9.82	-1.66	10.65	0.5	8.99	0.1	0.00618030	0.02815471	0.02522294	0.46609155	A_07_P043643	Z0390	cmr|orf Unknown function [Z0390]
Z0397	-2.54	9.95	-1.35	10.63	0.2	9.28	0.2	0.00321062	0.01683251	0.04666667	0.57534847	A_07_P043675	Z0397	cmr|orf Unknown function [Z0397]
Z0476	-3.05	12.26	-1.61	13.07	0.3	11.46	0.2	0.00120421	0.00794261	0.02894372	0.48426142	A_07_P048504	Z0476	cmr|orf Unknown function [Z0476]
Z0491	-2.24	10.45	-1.17	11.03	0.3	9.87	0.2	0.00692285	0.03089380	0.06645262	0.65594357	A_07_P043817	Z0491	cmr|orf Unknown function [Z0491]
Z0666	+3.43	7.70	+1.78	6.81	0.0	8.59	0.2	0.00021261	0.00220221	0.02630589	0.41707956	A_07_P043951	Z0666	cmr|orf Unknown function [Z0666]
Z0948	-2.27	8.10	-1.19	8.69	0.2	7.51	0.1	0.00192168	0.01128543	0.06611979	0.65677603	A_07_P046383	Z0948	cmr|unknown protein encoded by prophage CP-933K [Z0948]
Z0948	-2.94	7.90	-1.56	8.68	0.3	7.12	0.0	0.00082889	0.00596162	0.03080221	0.49233503	A_07_P046383	Z0948	cmr|unknown protein encoded by prophage CP-933K [Z0948]
Z0950	+3.10	8.46	+1.63	7.65	0.1	9.28	0.2	0.00059361	0.00461503	0.03115091	0.45077206	A_07_P046393	Z0950	cmr|unknown protein encoded by prophage CP-933K [Z0950]
Z0952	-2.18	10.97	-1.13	11.53	0.1	10.40	0.3	0.00713060	0.03165047	0.07276486	0.66760490	A_07_P046403	Z0952	cmr|putative Bet recombination protein of prophage CP-933K [Z0952]
Z0960	+10.32	9.42	+3.37	7.73	0.1	11.10	0.1	0.00000513	0.00024400	0.00329395	0.16783172	A_07_P046435	Z0960	cmr|putative lysozyme protein R of prophage CP-933K [Z0960]
Z0964	-2.83	8.51	-1.50	9.26	0.0	7.76	0.1	0.00037722	0.00327757	0.03508194	0.51475303	A_07_P046446	Z0964	cmr|putative DNA packaging protein of prophage CP-933K [Z0964]
Z0965	-2.19	9.86	-1.13	10.43	0.1	9.30	0.1	0.00154727	0.00956658	0.07105183	0.66546104	A_07_P046451	Z0965	cmr|unknown protein encoded by prophage CP-933K [Z0965]
Z0966	-2.08	8.39	-1.06	8.92	0.1	7.86	0.1	0.00178057	0.01067090	0.08200902	0.69829638	A_07_P046460	Z0966	cmr|unknown protein encoded by prophage CP-933K [Z0966]
Z0966	-2.10	8.42	-1.07	8.96	0.1	7.88	0.1	0.00209251	0.01202792	0.07919715	0.68623005	A_07_P046460	Z0966	cmr|unknown protein encoded by prophage CP-933K [Z0966]
Z0966	-2.26	8.39	-1.18	8.98	0.0	7.80	0.3	0.00461149	0.02227779	0.06326728	0.64387847	A_07_P046460	Z0966	cmr|unknown protein encoded by prophage CP-933K [Z0966]
Z0966	-2.01	8.56	-1.01	9.06	0.1	8.05	0.1	0.00214586	0.01225405	0.08829522	0.72064282	A_07_P046460	Z0966	cmr|unknown protein encoded by prophage CP-933K [Z0966]
Z0968	-2.09	8.77	-1.06	9.30	0.1	8.24	0.1	0.00322907	0.01690110	0.08053417	0.69285792	A_07_P046469	Z0968	cmr|unknown protein encoded by prophage CP-933K [Z0968]
Z0971	+3.31	7.61	+1.73	6.75	0.0	8.47	0.1	0.00015590	0.00176961	0.02737678	0.42423228	A_07_P046483	Z0971	cmr|putative tail component of prophage CP-933K [Z0971]
Z0975	+2.15	8.51	+1.11	7.95	0.1	9.06	0.4	0.01174252	0.04702981	0.06155297	0.62200899	A_07_P046501	Z0975	cmr|putative tail component of prophage CP-933K [Z0975]
Z0977	+3.24	9.19	+1.69	8.35	0.3	10.04	0.2	0.00182026	0.01085124	0.02971100	0.43922066	A_07_P046512	Z0977	cmr|putative tail component of prophage CP-933K [Z0977]
Z0979	+2.12	8.33	+1.09	7.79	0.0	8.87	0.2	0.00233964	0.01309465	0.06286141	0.62638608	A_07_P046522	Z0979	cmr|putative tail component of prophage CP-933K [Z0979]
Z1046	+2.94	7.52	+1.56	6.74	0.0	8.30	0.0	0.00020287	0.00212689	0.03405615	0.46909886	A_07_P046587	Z1046	cmr|orf Unknown function [Z1046]
Z1051	+4.67	10.65	+2.22	9.53	0.1	11.76	0.1	0.00004689	0.00076831	0.01586509	0.33708502	A_07_P046593	Z1051	cmr|orf Unknown function [Z1051]
Z1098	-2.18	11.47	-1.13	12.03	0.1	10.91	0.1	0.00189920	0.01118634	0.07277439	0.66730344	A_07_P046651	Z1098	cmr|orf Unknown function [Z1098]
Z1099	+2.15	8.97	+1.10	8.42	0.1	9.52	0.2	0.00283107	0.01518131	0.06146723	0.62154143	A_07_P046659	Z1099	cmr|orf, hypothetical protein [Z1099]
Z1121	+13.75	9.76	+3.78	7.86	0.3	11.65	0.1	0.00001095	0.00034947	0.00189278	0.12789700	A_07_P046679	Z1121	cmr|orf Unknown function [Z1121]
Z1125	-3.17	10.72	-1.66	11.55	0.5	9.89	0.3	0.00488252	0.02327288	0.02524454	0.46594373	A_07_P046692	Z1125	cmr|orf Unknown function [Z1125]
Z1129	-4.75	13.04	-2.25	14.17	0.3	11.92	0.3	0.00045892	0.00379654	0.00772866	0.28630588	A_07_P046708	Z1129	cmr|putative helicase [Z1129]
Z1131	-3.33	8.91	-1.73	9.78	0.0	8.05	0.1	0.00017485	0.00192910	0.02151359	0.44275817	A_07_P046719	Z1131	cmr|unknown protein encoded in ISEc8 [Z1131]
Z1133	+3.97	9.77	+1.99	8.77	0.1	10.76	0.1	0.00008688	0.00118218	0.02025025	0.37819692	A_07_P046726	Z1133	cmr|partial putative transposase [Z1133]
Z1160	-3.77	9.60	-1.91	10.55	0.2	8.64	0.5	0.00199849	0.01160615	0.01428163	0.37853535	A_07_P046840	Z1160	cmr|unknown in ISEc8 [Z1160]
Z1161	-2.28	8.92	-1.19	9.51	0.0	8.32	0.3	0.00375102	0.01899281	0.06488186	0.65063694	A_07_P046841	Z1161	cmr|unknown in ISEc8 [Z1161]
Z1191	-2.01	8.33	-1.00	8.84	0.3	7.83	0.2	0.01105142	0.04498284	0.08612424	0.71402844	A_07_P046966	Z1191	cmr|orf Unknown function [Z1191]
Z1199	-3.35	9.42	-1.75	10.29	0.1	8.54	0.6	0.00553502	0.02575919	0.02210048	0.44723650	A_07_P050887	Z1199	cmr|unknown in IS [Z1199]
Z1200	-2.82	7.77	-1.49	8.51	0.2	7.02	0.1	0.00096008	0.00669418	0.03514418	0.51470698	A_07_P050894	Z1200	cmr|orf Unknown function [Z1200]
Z1210	-2.00	7.82	-1.00	8.32	0.1	7.32	0.1	0.00357874	0.01830530	0.08883067	0.72239153	A_07_P050942	Z1210	cmr|putative histone [Z1210]
Z1217	-3.05	8.08	-1.61	8.88	0.0	7.27	0.2	0.00035716	0.00316085	0.02786712	0.48213187	A_07_P050973	Z1217	cmr|putative DNA repair protein, RADC family [Z1217]
Z1335	-3.53	10.57	-1.82	11.49	0.1	9.66	0.3	0.00094329	0.00659868	0.01892149	0.41839888	A_07_P051076	Z1335	cmr|unknown protein encoded by cryptic prophage CP-933M [Z1335]
Z1340	-5.14	9.40	-2.36	10.58	0.4	8.22	0.1	0.00042530	0.00358650	0.00572599	0.24497283	A_07_P051102	Z1340	cmr|unknown protein encoded by cryptic prophage CP-933M [Z1340]
Z1363	-4.14	8.77	-2.05	9.80	0.4	7.75	0.0	0.00078316	0.00571074	0.01108994	0.33837209	A_07_P051187	Z1363	cmr|unknown protein encoded by cryptic prophage CP-933M [Z1363]
Z1368	-2.64	8.14	-1.40	8.85	0.1	7.44	0.2	0.00130476	0.00845662	0.04228976	0.56091828	A_07_P051210	Z1368	cmr|unknown protein encoded by cryptic prophage CP-933M [Z1368]
Z1428	+3.75	8.20	+1.91	7.24	0.1	9.15	0.1	0.00012160	0.00148756	0.02226690	0.39301570	A_07_P048529	Z1428	cmr|unknown protein encoded by bacteriophage BP-933W [Z1428]
Z1429	-3.89	8.15	-1.96	9.13	0.0	7.17	0.1	0.00010986	0.00139154	0.01359375	0.36900000	A_07_P051303	Z1429	cmr|unknown protein encoded by bacteriophage BP-933W [Z1429]
Z1433	+2.99	9.48	+1.58	8.69	0.2	10.27	0.2	0.00079108	0.00574219	0.03321710	0.46444938	A_07_P051325	Z1433	cmr|unknown protein encoded by bacteriophage BP-933W [Z1433]
Z1434	+2.75	8.23	+1.46	7.50	0.3	8.97	0.0	0.00110247	0.00743993	0.03777693	0.48991763	A_07_P051326	Z1434	cmr|unknown protein encoded by bacteriophage BP-933W [Z1434]
Z1444	-2.60	12.79	-1.38	13.48	0.1	12.10	0.1	0.00057896	0.00452142	0.04544652	0.57103751	A_07_P051356	Z1444	cmr|putative serine/threonine kinase encoded by bacteriophage BP-933W [Z1444]
Z1454	+6.30	8.64	+2.66	7.31	0.3	9.97	0.0	0.00008370	0.00115089	0.00924860	0.26619744	A_07_P051405	Z1454	cmr|putative DNA N-6-adenine-methyltransferase of bacteriophage BP-933W [Z1454]
Z1473	+4.23	8.23	+2.08	7.19	0.3	9.27	0.2	0.00059679	0.00463535	0.01855691	0.36338308	A_07_P051457	Z1473	cmr|putative endopeptidase Rz of bacteriophage BP-933W [Z1473]
Z1475	-3.60	8.96	-1.85	9.89	0.3	8.04	0.5	0.00415981	0.02056266	0.01640180	0.39424427	A_07_P051463	Z1475	cmr|putative terminase small subunit of bacteriophage BP-933W [Z1475]
Z1660	-3.13	8.46	-1.65	9.29	0.0	7.64	0.1	0.00025742	0.00252041	0.02573107	0.46942063	A_07_P050081	Z1660	cmr|transposase for IS629 [Z1660]
Z1775	-2.69	11.90	-1.43	12.61	0.0	11.19	0.0	0.00032724	0.00298128	0.04084032	0.55050514	A_07_P050153	Z1775	cmr|unknown protein encoded by prophage CP-933N [Z1775]
Z1778	-2.31	11.74	-1.21	12.35	0.1	11.14	0.1	0.00103111	0.00706123	0.06304560	0.64245307	A_07_P050169	Z1778	cmr|unknown protein encoded by prophage CP-933N [Z1778]
Z1779	-2.23	7.57	-1.15	8.14	0.1	6.99	0.2	0.00270323	0.01465051	0.06780488	0.65855645	A_07_P050172	Z1779	cmr|unknown protein encoded by prophage CP-933N [Z1779]
Z1806	-2.88	8.68	-1.52	9.44	0.0	7.92	0.5	0.00443637	0.02159601	0.03165587	0.49199408	A_07_P050281	Z1806	cmr|unknown protein encoded by prophage CP-933N [Z1806]
Z1836	-3.36	8.56	-1.75	9.44	0.3	7.69	0.4	0.00311860	0.01642116	0.02016895	0.43027100	A_07_P050384	Z1836	cmr|unknown protein encoded by prophage CP-933C [Z1836]
Z1852	-4.20	10.62	-2.07	11.65	0.3	9.58	0.4	0.00093781	0.00657676	0.01044970	0.32385827	A_07_P050455	Z1852	cmr|putative holin protein of prophage CP-933C [Z1852]
Z1856	-2.97	7.80	-1.57	8.59	0.0	7.02	0.3	0.00077167	0.00564293	0.02990091	0.48884735	A_07_P050473	Z1856	cmr|unknown protein encoded by prophage CP-933C [Z1856]
Z1866	+2.00	7.47	+1.00	6.97	0.1	7.97	0.1	0.00339130	0.01752303	0.07107215	0.66407122	A_07_P050491	Z1866	cmr|putative integrase of prophage CP-933X [Z1866]
Z1867	+8.40	9.10	+3.07	7.57	0.1	10.64	0.1	0.00000983	0.00032848	0.00523247	0.21341969	A_07_P050497	Z1867	cmr|putative integrase of prophage CP-933X [Z1867]
Z1868	-3.06	7.76	-1.61	8.56	0.6	6.95	0.0	0.01143027	0.04603687	0.02770897	0.48257743	A_07_P050504	Z1868	cmr|putative replication protein O of prophage CP-933X [Z1868]
Z1871	-3.27	8.47	-1.71	9.32	0.1	7.62	0.7	0.01130337	0.04571086	0.02396214	0.45783981	A_07_P050509	Z1871	cmr|unknown protein encoded by prophage CP-933X [Z1871]
Z1871	-2.49	8.42	-1.31	9.07	0.0	7.76	0.5	0.00957151	0.03998246	0.04766451	0.57903549	A_07_P050509	Z1871	cmr|unknown protein encoded by prophage CP-933X [Z1871]
Z1871	-3.17	8.13	-1.66	8.96	0.2	7.30	0.4	0.00333154	0.01730256	0.02417810	0.45862651	A_07_P050509	Z1871	cmr|unknown protein encoded by prophage CP-933X [Z1871]
Z1871	-3.25	7.94	-1.70	8.79	0.1	7.09	0.1	0.00017549	0.00193212	0.02302591	0.45258427	A_07_P050509	Z1871	cmr|unknown protein encoded by prophage CP-933X [Z1871]
Z1891	+14.29	10.18	+3.84	8.26	0.1	12.10	0.2	0.00000426	0.00022159	0.00177401	0.12581081	A_07_P050583	Z1891	cmr|putative tail component of prophage CP-933X [Z1891]
Z1893	+2.88	10.69	+1.53	9.92	0.1	11.45	0.1	0.00039814	0.00340481	0.03513974	0.47611015	A_07_P050588	Z1893	cmr|putative tail component of prophage CP-933X [Z1893]
Z1894	+11.94	9.19	+3.58	7.40	0.1	10.97	0.2	0.00000536	0.00024927	0.00250318	0.14330909	A_07_P050594	Z1894	cmr|putative tail component of prophage CP-933X [Z1894]
Z1895	+7.86	8.48	+2.97	6.99	0.3	9.97	0.8	0.00247159	0.01363444	0.00572154	0.22024450	A_07_P050599	Z1895	cmr|putative tail component of prophage CP-933X [Z1895]
Z1896	+2.54	7.79	+1.34	7.12	0.3	8.46	0.0	0.00243432	0.01348555	0.04466781	0.52717391	A_07_P050602	Z1896	cmr|putative tail component of prophage CP-933X [Z1896]

Z1898	+5.20	10.31	+2.38	9.12	0.4	11.50	0.0	0.00046787	0.00384652	0.01294207	0.30186667	A_07_P050610	Z1898	cmr|putative tail component of prophage CP-933X [Z1898]
Z1915	+3.23	8.62	+1.69	7.77	0.0	9.47	0.1	0.00022634	0.00231393	0.02874111	0.43804453	A_07_P050656	Z1915	cmr|putative tail protein [Z1915]
Z1957	-7.97	9.94	-2.99	11.44	0.1	8.44	0.1	0.00001162	0.00035586	0.00151423	0.12613757	A_07_P050726	Z1957	cmr|transposase for IS629 [Z1957]
Z2005	-3.46	12.34	-1.79	13.24	0.2	11.45	0.0	0.00023142	0.00234711	0.01932609	0.41910468	A_07_P050791	Z2005	cmr|orf Unknown function [Z2005]
Z2047	-2.31	7.79	-1.21	8.40	0.4	7.19	0.1	0.01083098	0.04430319	0.06243394	0.64161880	A_07_P050858	Z2047	cmr|unknown protein encoded by prophage CP-933O [Z2047]
Z2049	+2.09	8.22	+1.06	7.68	0.0	8.75	0.0	0.00143325	0.00903332	0.06264355	0.62619683	A_07_P047614	Z2049	cmr|unknown protein encoded by prophage CP-933O [Z2049]
Z2074	-3.06	11.17	-1.61	11.98	0.1	10.36	0.0	0.00021775	0.00224211	0.02812500	0.48130435	A_07_P047687	Z2074	cmr|putative IS encoded protein within CP-933O [Z2074]
Z2076	-2.07	11.19	-1.05	11.71	0.1	10.67	0.3	0.00894989	0.03790606	0.08048336	0.69279934	A_07_P047700	Z2076	cmr|unknown protein encoded by prophage CP-933O [Z2076]
Z2080	-9.35	11.39	-3.23	13.00	0.1	9.77	0.1	0.00000602	0.00026402	0.00093369	0.10000000	A_07_P047717	Z2080	cmr|putative IS encoded protein within CP-933O [Z2080]
Z2096	-4.88	9.32	-2.29	10.47	0.8	8.18	0.4	0.00896545	0.03795432	0.00737297	0.28038647	A_07_P047778	Z2096	cmr|unknown protein encoded within prophage CP-933O [Z2096]
Z2099	-2.32	8.63	-1.21	9.24	0.0	8.03	0.4	0.00802999	0.03479884	0.06128430	0.63477632	A_07_P047791	Z2099	cmr|unknown protein encoded within prophage CP-933O [Z2099]
Z2115	-2.44	11.51	-1.28	12.15	0.3	10.87	0.0	0.00340604	0.01755883	0.05406568	0.60713980	A_07_P047851	Z2115	cmr|unknown protein encoded within prophage CP-933O [Z2115]
Z2119	-2.79	8.95	-1.48	9.69	0.0	8.21	0.1	0.00033473	0.00302754	0.03654789	0.52120471	A_07_P047868	Z2119	cmr|unknown protein encoded within prophage CP-933O [Z2119]
Z2133	+2.73	7.74	+1.45	7.01	0.1	8.46	0.1	0.00054284	0.00430634	0.03873857	0.49504870	A_07_P047906	Z2133	cmr|putative capsid assembly protein of prophage CP-933O [Z2133]
Z2136	+7.32	8.71	+2.87	7.28	0.4	10.15	0.0	0.00019178	0.00204384	0.00675686	0.23431718	A_07_P047913	Z2136	cmr|partial putative major capsid protein of prophage [Z2136]
Z2142	-4.24	11.49	-2.09	12.54	0.2	10.45	0.5	0.00230006	0.01293914	0.01084731	0.33290448	A_07_P047939	Z2142	cmr|putative tail component of prophage CP-933O [Z2142]
Z2144	-3.40	10.59	-1.77	11.48	0.3	9.71	0.2	0.00110061	0.00743373	0.02074568	0.43433511	A_07_P047949	Z2144	cmr|putative tail component of prophage CP-933O [Z2144]
Z2181	-2.10	7.92	-1.07	8.45	0.2	7.38	0.1	0.00355803	0.01821120	0.07876778	0.68439294	A_07_P048026	Z2181	cmr|orf Unknown function [Z2181]
Z2199	-2.29	8.72	-1.19	9.31	0.1	8.12	0.0	0.00133913	0.00861948	0.06461001	0.64956577	A_07_P048039	Z2199	cmr|orf, hypothetical protein [Z2199]
Z2201	-2.58	7.68	-1.37	8.37	0.1	7.00	0.1	0.00072185	0.00536584	0.04526613	0.57196629	A_07_P048050	Z2201	cmr|putative fimbrial chaperone protein [Z2201]
Z2249	-4.07	10.32	-2.02	11.33	0.2	9.30	0.2	0.00023214	0.00234886	0.01178608	0.34814259	A_07_P048081	Z2249	cmr|orf Unknown function [Z2249]
Z2274	-2.46	7.29	-1.30	7.94	0.2	6.64	0.2	0.00388476	0.01949807	0.05174670	0.60081121	A_07_P048560	Z2274	cmr|orf Unknown function [Z2274]
Z2290	-2.53	9.37	-1.34	10.03	0.3	8.70	0.3	0.00618767	0.02818013	0.04818026	0.57993119	A_07_P052464	Z2290	cmr|orf, hypothetical protein [Z2290]
Z2297	-2.16	10.95	-1.11	11.51	0.1	10.40	0.0	0.00176850	0.01061506	0.07406504	0.67170507	A_07_P048153	Z2297	cmr|orf Unknown function [Z2297]
Z2298	-3.24	9.03	-1.70	9.88	0.1	8.18	0.1	0.00016883	0.00188113	0.02356707	0.45470588	A_07_P048160	Z2298	cmr|orf Unknown function [Z2298]
Z2309	-3.26	10.61	-1.70	11.46	0.2	9.76	0.0	0.00040741	0.00347035	0.02316946	0.45201983	A_07_P048162	Z2309	cmr|orf Unknown function [Z2309]
Z2318	-2.27	9.38	-1.18	9.97	0.0	8.79	0.1	0.00097375	0.00676551	0.06642594	0.65609159	A_07_P048190	Z2318	cmr|putative phosphatidate cytidiltransferase [Z2318]
Z2360	+15.65	9.68	+3.97	7.69	0.5	11.66	0.1	0.00004999	0.00080150	0.00141705	0.11441026	A_07_P048290	Z2360	cmr|putative capsid protein of prophage CP-933R [Z2360]
Z2361	+20.94	10.57	+4.39	8.37	0.0	12.76	0.1	0.00000101	0.00012946	0.00068280	0.07904412	A_07_P048291	Z2361	cmr|putative capsid assembly protein of prophage CP-933R [Z2361]
Z2391	+2.75	7.66	+1.46	6.93	0.2	8.38	0.5	0.01117959	0.04532019	0.03978532	0.49950558	A_07_P048371	Z2391	cmr|unknown protein encoded within prophage CP-933R [Z2391]
Z2394	-2.56	8.98	-1.35	9.66	0.1	8.31	0.4	0.00669880	0.03010731	0.04760417	0.57874903	A_07_P048386	Z2394	cmr|unknown protein encoded within prophage CP-933R [Z2394]
Z2446	+5.51	9.38	+2.46	8.15	0.0	10.61	0.1	0.00002966	0.00059702	0.01189914	0.29090062	A_07_P048452	Z2446	cmr|orf Unknown function [Z2446]
Z2511	-2.59	7.59	-1.37	8.27	0.3	6.90	0.3	0.00455836	0.02208209	0.04411204	0.56833061	A_07_P044103	Z2511	cmr|orf Unknown function [Z2511]
Z2519	+2.81	8.30	+1.49	7.55	0.3	9.05	0.1	0.00188092	0.01111606	0.03704268	0.48398340	A_07_P044110	Z2519	cmr|orf Unknown function [Z2519]
Z2519	+2.85	8.59	+1.51	7.84	0.2	9.35	0.2	0.00072050	0.00535832	0.03615282	0.48032911	A_07_P044110	Z2519	cmr|orf Unknown function [Z2519]
Z2519	+3.57	8.78	+1.84	7.87	0.1	9.70	0.1	0.00013471	0.00158871	0.02419842	0.40358051	A_07_P044110	Z2519	cmr|orf Unknown function [Z2519]
Z2519	+3.43	8.63	+1.78	7.74	0.2	9.52	0.1	0.00028452	0.00267907	0.02628620	0.41760848	A_07_P044110	Z2519	cmr|orf Unknown function [Z2519]
Z2519	+2.89	8.46	+1.53	7.69	0.1	9.23	0.1	0.00051701	0.00415078	0.03522358	0.47601717	A_07_P044110	Z2519	cmr|orf Unknown function [Z2519]
Z2546	+5.70	10.27	+2.51	9.01	0.0	11.52	0.0	0.00001616	0.00041859	0.01138275	0.28491256	A_07_P044121	Z2546	cmr|anthranilate synthase component I [Z2546]
Z2547	+78.24	10.63	+6.29	7.49	0.2	13.78	0.1	0.00000031	0.00009117	0.00004446	0.01750000	A_07_P044129	Z2547	cmr|anthranilate synthase component I [Z2547]
Z2560	-2.37	8.99	-1.25	9.61	0.0	8.37	0.4	0.00602109	0.02760512	0.05520389	0.61206338	A_07_P044137	Z2560	cmr|orf Unknown function [Z2560]
Z2561	-4.65	8.98	-2.22	10.09	0.1	7.87	0.2	0.00008551	0.00116924	0.00804560	0.29052752	A_07_P044143	Z2561	cmr|putative transposase [Z2561]
Z2581	+2.10	8.53	+1.07	7.99	0.0	9.07	0.2	0.00323716	0.01692654	0.06474339	0.63627965	A_07_P044386	Z2581	cmr|putative oxidoreductase component [Z2581]
Z2622	+2.02	7.57	+1.01	7.06	0.2	8.07	0.1	0.00496808	0.02360192	0.06989647	0.66053421	A_07_P044415	Z2622	cmr|partial beta-D-glucuronidase [Z2622]
Z2664	+3.09	9.47	+1.63	8.66	0.0	10.28	0.1	0.00026702	0.00256768	0.03138084	0.45119635	A_07_P044420	Z2664	cmr|orf Unknown function [Z2664]
Z2754	+6.34	9.46	+2.66	8.12	0.4	10.79	0.1	0.00024821	0.00245286	0.00890307	0.26053903	A_07_P044434	Z2754	cmr|orf, hypothetical protein [Z2754]
Z2756	-3.29	10.51	-1.72	11.37	0.2	9.65	0.1	0.00041677	0.00352927	0.02287792	0.45136591	A_07_P052541	Z2756	cmr|putative phosphatase [Z2756]
Z2804	-3.59	9.04	-1.84	9.96	0.1	8.12	0.2	0.00037460	0.00325950	0.01797066	0.41063861	A_07_P044458	Z2804	cmr|unknown protein encoded within IS629 [Z2804]
Z2806	-5.84	8.21	-2.54	9.48	0.2	6.94	0.2	0.00006208	0.00092690	0.00407076	0.20876221	A_07_P044461	Z2806	cmr|putative transposase [Z2806]
Z2810	+2.01	7.57	+1.01	7.07	0.2	8.08	0.0	0.00589823	0.02711293	0.07021024	0.66111842	A_07_P044469	Z2810	cmr|putative kinase [Z2810]
Z2992	-2.31	9.41	-1.21	10.01	0.2	8.81	0.3	0.00699202	0.03114951	0.06017022	0.63154667	A_07_P044645	Z2992	cmr|putative tail assembly protein of prophage CP-933T [Z2992]
Z2992	-2.08	9.46	-1.06	9.99	0.0	8.93	0.2	0.00266545	0.01448562	0.08300877	0.70338536	A_07_P044645	Z2992	cmr|putative tail assembly protein of prophage CP-933T [Z2992]
Z2992	-2.08	9.55	-1.06	10.08	0.1	9.02	0.0	0.00170315	0.01033706	0.08222497	0.69937871	A_07_P044645	Z2992	cmr|putative tail assembly protein of prophage CP-933T [Z2992]
Z2994	-5.78	8.98	-2.53	10.24	1.0	7.71	0.0	0.00796442	0.03456223	0.00412792	0.20897106	A_07_P044649	Z2994	cmr|unknown protein encoded by prophage CP-933T [Z2994]
Z3058	-2.32	8.45	-1.22	9.05	0.0	7.84	0.4	0.00573883	0.02652733	0.06132177	0.63432983	A_07_P044705	Z3058	cmr|putative outer membrane protein [Z3058]
Z3077	-2.03	8.48	-1.02	8.99	0.1	7.97	0.1	0.00292837	0.01563917	0.08803290	0.71999481	A_07_P044736	Z3077	cmr|putative tail fiber component J of prophage [Z3077]
Z3108	-3.24	8.10	-1.70	8.95	0.0	7.26	0.4	0.00238414	0.01327294	0.02294334	0.45209011	A_07_P044835	Z3108	cmr|unknown protein encoded within prophage CP-933U [Z3108]
Z3144	-5.17	8.83	-2.37	10.02	0.0	7.65	0.3	0.00016033	0.00180431	0.00584667	0.24612299	A_07_P044923	Z3144	cmr|orf Unknown function [Z3144]
Z3159	-6.61	8.83	-2.72	10.19	0.1	7.47	0.3	0.00006774	0.00099132	0.00274962	0.17669388	A_07_P044945	Z3159	cmr|putative outer membrane receptor for iron compound [Z3159]
Z3162	-4.87	9.25	-2.28	10.39	0.1	8.11	0.2	0.00011107	0.00139729	0.00705158	0.27210784	A_07_P044951	Z3162	cmr|IS629 transposase [Z3162]
Z3272	-2.16	8.06	-1.11	8.62	0.2	7.50	0.0	0.00257215	0.01411500	0.07494665	0.67272520	A_07_P045154	Z3272	cmr|orf Unknown function [Z3272]
Z3297	-6.73	9.65	-2.75	11.02	0.0	8.27	0.4	0.00015650	0.00177268	0.00255526	0.17046610	A_07_P045211	Z3297	cmr|putative transposase for IS629 [Z3297]
Z3311	+2.01	7.14	+1.01	6.63	0.0	7.64	0.3	0.00684311	0.03061948	0.07236408	0.67215339	A_07_P045236	Z3311	cmr|putative tail fiber protein of prophage CP-933V [Z3311]
Z3313	-3.62	7.89	-1.85	8.81	0.7	6.96	0.1	0.00685062	0.03063453	0.01690739	0.39670641	A_07_P052645	Z3313	cmr|putative tail component of prophage CP-933V [Z3313]
Z3314	-2.16	9.85	-1.11	10.41	0.2	9.30	0.1	0.00366138	0.01863115	0.07486026	0.67348571	A_07_P045250	Z3314	cmr|putative tail component of prophage CP-933V [Z3314]
Z3345	-2.59	10.23	-1.37	10.92	0.2	9.55	0.2	0.00204731	0.01184594	0.04536522	0.57138400	A_07_P045326	Z3345	cmr|putative antitermination protein Q for prophage CP-933V [Z3345]
Z3349	+3.21	7.46	+1.68	6.62	0.1	8.30	0.1	0.00024910	0.00245579	0.02920414	0.43789524	A_07_P045345	Z3349	cmr|putative DNA methyltransferase encoded within prophage CP-933V [Z3349]
Z3357	-2.16	8.05	-1.11	8.60	0.1	7.49	0.3	0.00425830	0.02090508	0.07271659	0.66833041	A_07_P045373	Z3357	cmr|putative regulatory protein CII of prophage CP-933V [Z3357]
Z3364	-4.39	11.96	-2.14	13.02	0.0	10.89	0.0	0.00004116	0.00070900	0.00948234	0.30908903	A_07_P045400	Z3364	cmr|putative host killing protein Kil of prophage [Z3364]
Z3366	-2.20	13.22	-1.14	13.79	0.0	12.65	0.1	0.00108449	0.00733741	0.07120173	0.66449318	A_07_P045406	Z3366	cmr|putative recombination protein Bet of prophage CP-933V [Z3366]
Z3367	-3.41	10.02	-1.77	10.90	0.1	9.13	0.6	0.00573842	0.02652733	0.02110391	0.43891678	A_07_P045415	Z3367	cmr|putative exonuclease of prophage CP-933V [Z3367]
Z3390	-2.22	10.20	-1.15	10.78	0.3	9.62	0.1	0.00423035	0.02079385	0.06727960	0.65669560	A_07_P045451	Z3390	cmr|putative hydroxylase [Z3390]
Z3392	-2.78	10.43	-1.47	11.17	0.3	9.70	0.4	0.00785007	0.03417298	0.03540333	0.51419742	A_07_P045464	Z3392	cmr|putative isomerase-decarboxylase [Z3392]
Z3603	-3.53	8.75	-1.82	9.66	0.3	7.84	0.6	0.00544210	0.02540548	0.01729675	0.40224520	A_07_P042677	Z3603	cmr|orf, hypothetical protein [Z3603]
Z3618	-2.44	8.86	-1.29	9.51	0.0	8.22	0.1	0.00060040	0.00465408	0.05349530	0.60461594	A_07_P042718	Z3618	cmr|orf Unknown function [Z3618]
Z3657	-3.21	10.57	-1.68	11.41	0.1	9.73	0.4	0.00227837	0.01284308	0.02503049	0.46362353	A_07_P042781	Z3657	cmr|orf, hypothetical protein [Z3657]
Z3673	-4.22	11.32	-2.08	12.36	0.0	10.28	0.0	0.00004505	0.00075371	0.01062373	0.32731898	A_07_P042804	Z3673	cmr|putative transcriptional regulator LYSR-type [Z3673]
Z3699	-2.25	9.82	-1.17	10.41	0.4	9.24	0.0	0.00878758	0.03733178	0.06696583	0.65648194	A_07_P042822	Z3699	cmr|orf Unknown function [Z3699]
Z3767	-2.14	7.41	-1.10	7.96	0.1	6.86	0.1	0.00222077	0.01257968	0.07423463	0.67130959	A_07_P042845	Z3767	cmr|orf Unknown function [Z3767]
Z3783	-5.15	11.43	-2.36	12.61	0.1	10.25	0.3	0.00021077	0.00218894	0.00583460	0.24693548	A_07_P042860	Z3783	cmr|putative dimethyl sulfoxide reductase subunit C [Z3783]
Z3799	-19.27	12.94	-4.27	15.07	0.0	10.80	0.6	0.00005595	0.00086270	0.00006034	0.02317073	A_07_P042878	Z3799	cmr|putative ATP synthase beta subunit [Z3799]
Z3799	-11.35	12.95	-3.50	14.70	0.0	11.20	0.1	0.00000440	0.00022561	0.00049162	0.07166667	A_07_P042878	Z3799	cmr|putative ATP synthase beta subunit [Z3799]
Z3799	-14.91	13.06	-3.90	15.01	0.0	11.11	0.4	0.00001974	0.00046811	0.00016070	0.04216667	A_07_P042878	Z3799	cmr|putative ATP synthase beta subunit [Z3799]
Z3799	-10.38	13.63	-3.38	15.31	0.1	11.94	0.0	0.00000550	0.00025298	0.00065930	0.08304000	A_07_P042878	Z3799	cmr|putative ATP synthase beta subunit [Z3799]
Z3799	-13.64	13.07	-3.77	14.96	0.1	11.19	0.4	0.00002645	0.00055821	0.00024835	0.05144737	A_07_P042878	Z3799	cmr|putative ATP synthase beta subunit [Z3799]
Z3866	+2.33	7.82	+1.22	7.21	0.0	8.43	0.0	0.00072265	0.00536671	0.05097624	0.55889276	A_07_P042932	Z3866	cmr|orf Unknown function [Z3866]
Z3923	-6.63	10.02	-2.73	11.39	0.1	8.66	0.0	0.00001402	0.00039147	0.00277058	0.17659919	A_07_P042966	Z3923	cmr|orf Unknown function [Z3923]
Z3924	-10.93	10.43	-3.45	12.16	0.0	8.71	0.2	0.00000821	0.00030062	0.00055005	0.07732143	A_07_P042971	Z3924	cmr|partial putative transposase [Z3924]
Z3925	-6.12	9.22	-2.61	10.53	0.1	7.92	0.1	0.00002323	0.00052262	0.00356771	0.19778169	A_07_P042976	Z3925	cmr|partial putative transposase [Z3925]
Z3931	+2.77	10.76	+1.47	10.03	0.1	11.50	0.1	0.00051332	0.00412930	0.03799098	0.49147905	A_07_P042986	Z3931	cmr|unknown protein encoded by prophage CP-933Y [Z3931]
Z3934	-2.62	10.29	-1.39	10.99	0.2	9.60	0.3	0.00411615	0.02038523	0.04221481	0.56086920	A_07_P043004	Z3934	cmr|unknown protein encoded by prophage CP-933Y [Z3934]
Z3974	+3.58	8.28	+1.84	7.36	0.3	9.20	0.0	0.00055956	0.00439729	0.02445249	0.40609705	A_07_P043090	Z3974	cmr|orf Unknown function [Z3974]
Z4065	-5.99	9.59	-2.58	10.88	0.4	8.29	0.0	0.00023775	0.00238308	0.00358930	0.19828070	A_07_P043170	Z4065	cmr|orf hypothetical protein [Z4065]
Z4127	-3.12	9.21	-1.64	10.03	0.0	8.39	0.2	0.00041747	0.00352993	0.02594131	0.47107266	A_07_P043257	Z4127	cmr|orf, hypothetical protein [Z4127]
Z4191	-2.57	8.09	-1.36	8.78	0.2	7.41	0.2	0.00168657	0.01026417	0.04577490	0.57196825	A_07_P045584	Z4191	cmr|type III secretion apparatus protein [Z4191]
Z4237	+3.59	9.26	+1.84	8.34	0.1	10.18	0.0	0.00012509	0.00151602	0.02431847	0.40429778	A_07_P052774	Z4237	cmr|putative oxidoreductase [Z4237]
Z4268	+12.89	8.63	+3.69	6.78	0.3	10.47	0.1	0.00001937	0.00046342	0.00210493	0.13471545	A_07_P045673	Z4268	cmr|orf hypothetical protein [Z4268]
Z4269	+8.45	8.87	+3.08	7.33	0.1	10.41	0.0	0.00000604	0.00026402	0.00509654	0.21340426	A_07_P045678	Z4269	cmr|orf hypothetical protein [Z4269]
Z4270	+4.93	7.75	+2.30	6.60	0.1	8.90	0.1	0.00004527	0.00075578	0.01440993	0.32180142	A_07_P045685	Z4270	cmr|putative ATP-binding protein of ABC transport system [Z4270]
Z4315	-2.92	9.15	-1.55	9.92	0.1	8.37	0.0	0.00027464	0.00261738	0.03227134	0.49665689	A_07_P045721	Z4315	cmr|unknown protein encoded by ISEc8 [Z4315]
Z4324	-2.78	8.55	-1.48	9.29	0.4	7.81	0.4	0.00981572	0.04086162	0.03508829	0.51436685	A_07_P045757	Z4324	cmr|putative transposase [Z4324]
Z4332	-2.22	9.33	-1.15	9.91	0.2	8.76	0.2	0.00439947	0.02145764	0.06897929	0.65739104	A_07_P045787	Z4332	cmr|putative cytotoxin [Z4332]
Z4338	-2.38	7.39	-1.25	8.01	0.1	6.76	0.3	0.00315817	0.01659066	0.05767086	0.62104651	A_07_P045796	Z4338	cmr|unknown protein encoded by ISEc8 [Z4338]
Z4340	-2.99	11.84	-1.58	12.63	0.0	11.05	0.1	0.00023061	0.00234535	0.02990346	0.48838174	A_07_P045803	Z4340	cmr|unknown protein encoded by ISEc8 [Z4340]
Z4343	+2.89	10.23	+1.53	9.46	0.2	10.99	0.2	0.00110833	0.00746025	0.03532012	0.47650386	A_07_P045808	Z4343	cmr|orf, hypothetical protein [Z4343]
Z4382	-2.76	8.57	-1.46	9.30	0.0	7.84	0.0	0.00029237	0.00273664	0.03801512	0.53012400	A_07_P045826	Z4382	cmr|putative iron compound-binding protein of ABC transporter [Z4382]
Z4385	-2.15	8.50	-1.10	9.05	0.0	7.95	0.0	0.00117500	0.00780071	0.07548717	0.67411798	A_07_P045844	Z4385	cmr|putative ATP-binding protein of ABC transporter family [Z4385]
Z4400	-7.15	12.51	-2.84	13.93	0.1	11.09	0.2	0.00003155	0.00061713	0.00216717	0.16018779	A_07_P045859	Z4400	cmr|orf Unknown function [Z4400]
Z4400	-6.07	13.15	-2.60	14.45	0.0	11.85	0.0	0.00001417	0.00039205	0.00353023	0.19779359	A_07_P045859	Z4400	cmr|orf Unknown function [Z4400]
Z4400	-7.15	12.78	-2.84	14.20	0.0	11.36	0.0	0.00000915	0.00032069	0.00217734	0.15870370	A_07_P045859	Z4400	cmr|orf Unknown function [Z4400]
Z4400	-14.02	13.30	-3.81	15.21	1.0	11.40	0.1	0.00142814	0.00902275	0.00025089	0.05064103	A_07_P045859	Z4400	cmr|orf Unknown function [Z4400]
Z4400	-5.26	13.12	-2.39	14.32	0.0	11.93	0.1	0.00003295	0.00062963	0.00545795	0.24137640	A_07_P045859	Z4400	cmr|orf Unknown function [Z4400]
Z4487	+5.63	7.93	+2.49	6.68	0.0	9.17	0.1	0.00002721	0.00056519	0.01141197	0.28473851	A_07_P045901	Z4487	cmr|putative phosphotransferase system enzyme subunit [Z4487]
Z4488	+5.27	8.90	+2.40	7.70	0.1	10.10	0.0	0.00003468	0.00064547	0.01312055	0.30377941	A_07_P045910	Z4488	cmr|putative phosphotransferase system enzyme subunit [Z4488]
Z4501	-43.14	11.60	-5.43	14.32	0.0	8.89	0.1	0.00000036	0.00009117	0.00000191	0.00333333	A_07_P045934	Z4501	cmr|orf hypothetical protein [Z4501]
Z4799	+2.56	10.75	+1.36	10.07	0.2	11.43	0.4	0.00899363	0.03805312	0.04645706	0.53780882	A_07_P046075	Z4799	cmr|putative DNA processing protein [Z4799]
Z4801	+5.90	9.04	+2.56	7.76	0.2	10.32	0.0	0.00002926	0.00059064	0.01050877	0.27760067	A_07_P046078	Z4801	cmr|putative ATP-dependent DNA helicase [Z4801]
Z4803	+4.15	8.17	+2.05	7.14	0.1	9.20	0.2	0.00015748	0.00177863	0.01897866	0.36888889	A_07_P046088	Z4803	cmr|putative ATP-dependent DNA helicase [Z4803]
Z4858	+3.65	7.69	+1.87	6.75	0.1	8.62	0.2	0.00028709	0.00270003	0.02385988	0.40262594	A_07_P046176	Z4858	cmr|putative enzyme [Z4858]
Z4859	+8.25	9.44	+3.04	7.92	0.2	10.96	0.0	0.00001332	0.00038273	0.00538681	0.21635204	A_07_P046184	Z4859	cmr|orf hypothetical protein [Z4859]
Z4860	+4.72	8.72	+2.24	7.60	0.1	9.84	0.1	0.00007976	0.00110927	0.01551067	0.33452055	A_07_P046186	Z4860	cmr|orf Unknown function [Z4860]
Z4862	+10.69	8.54	+3.42	6.83	0.0	10.25	0.0	0.00000312	0.00019442	0.00311484	0.16511785	A_07_P046200	Z4862	cmr|orf Unknown function [Z4862]
Z4863	+9.39	8.48	+3.23	6.87	0.1	10.10	0.1	0.00000695	0.00027756	0.00410379	0.19002941	A_07_P046205	Z4863	cmr|putative 3-oxoacyl- [Z4863]
Z4864	+5.78	8.39	+2.53	7.13	0.2	9.66	0.2	0.00014265	0.00165628	0.01076220	0.27777049	A_07_P046210	Z4864	cmr|orf Unknown function [Z4864]
Z4874	+4.14	9.22	+2.05	8.20	0.7	10.25	0.1	0.00671012	0.03014959	0.01930005	0.37329238	A_07_P046227	Z4874	cmr|putative regulator [Z4874]
Z4875	+3.25	7.91	+1.70	7.06	0.2	8.76	0.2	0.00060286	0.00466639	0.02887195	0.43876448	A_07_P046232	Z4875	cmr|putative phosphotransferase system enzyme subunit [Z4875]
Z4876	+3.29	8.11	+1.72	7.25	0.3	8.97	0.3	0.00175494	0.01056991	0.02853214	0.43782651	A_07_P046239	Z4876	cmr|putative phosphotransferase system enzyme subunit [Z4876]
Z4883	-2.23	10.50	-1.16	11.08	0.1	9.92	0.1	0.00122702	0.00806608	0.06741616	0.65640074	A_07_P049368	Z4883	cmr|orf Unknown function [Z4883]
Z4945	+3.49	8.92	+1.80	8.02	0.2	9.82	0.0	0.00033479	0.00302754	0.02547447	0.41219938	A_07_P049434	Z4945	cmr|orf Unknown function [Z4945]
Z4950	-2.16	10.37	-1.11	10.93	0.2	9.82	0.1	0.00396114	0.01981073	0.07214939	0.66818824	A_07_P049440	Z4950	cmr|orf Unknown function [Z4950]
Z4982	-2.14	11.50	-1.10	12.05	0.1	10.95	0.2	0.00424086	0.02083900	0.07767658	0.67865705	A_07_P048615	Z4982	cmr|putative membrane permeability altering protein [Z4982]
Z4999	-2.84	7.95	-1.50	8.70	0.1	7.20	0.3	0.00165772	0.01011988	0.03399962	0.50786528	A_07_P049484	Z4999	cmr|orf Unknown function [Z4999]
Z5220	-2.25	7.74	-1.17	8.32	0.1	7.15	0.1	0.00130523	0.00845662	0.06814660	0.65862492	A_07_P049946	Z5220	cmr|putative fimbrial protein [Z5220]
Z5339	-2.08	9.97	-1.05	10.49	0.1	9.44	0.1	0.00232722	0.01305298	0.08182355	0.69747158	A_07_P050021	Z5339	cmr|orf Unknown function [Z5339]
Z5522	-2.32	11.96	-1.21	12.56	0.1	11.35	0.1	0.00126510	0.00826488	0.06247078	0.64157860	A_07_P047170	Z5522	cmr|putative hippuricase [Z5522]
Z5523	-4.42	11.64	-2.15	12.72	0.1	10.57	0.3	0.00026220	0.00255135	0.00928227	0.30701681	A_07_P047173	Z5523	cmr|putative citrate permease [Z5523]
Z5613	+2.38	8.21	+1.25	7.58	0.3	8.83	0.1	0.00468662	0.02252189	0.05018293	0.55561181	A_07_P047225	Z5613	cmr|putative L-sorbose-1-P-reductase [Z5613]
Z5618	-2.07	7.56	-1.05	8.09	0.1	7.03	0.2	0.00312010	0.01642355	0.08203887	0.69817297	A_07_P047247	Z5618	cmr|D-glucitol-6-phosphate dehydrogenase [Z5618]
Z5839	+5.05	9.46	+2.33	8.29	0.1	10.63	0.1	0.00004407	0.00074207	0.01400407	0.31953623	A_07_P052376	Z5839	cmr|putative ATP-binding component of ABC transporter [Z5839]
Z5852	-38.72	13.71	-5.27	16.35	0.0	11.08	0.1	0.00000034	0.00009117	0.00000254	0.00363636	A_07_P047387	Z5852	cmr|orf Unknown function [Z5852]
Z5886	-2.19	10.55	-1.13	11.11	0.0	9.98	0.1	0.00132011	0.00851447	0.07110709	0.66518717	A_07_P047426	Z5886	cmr|orf Unknown function [Z5886]
Z5894	-2.90	9.15	-1.53	9.92	0.2	8.39	0.2	0.00087152	0.00620590	0.03216527	0.49696762	A_07_P047460	Z5894	cmr|orf Unknown function [Z5894]
Z5940	+3.25	8.08	+1.70	7.23	0.0	8.93	0.2	0.00026124	0.00254676	0.02894499	0.43860443	A_07_P047549	Z5940	cmr|orf Unknown function [Z5940]
Z5954	-2.58	8.79	-1.36	9.47	0.1	8.11	0.5	0.00788290	0.03427461	0.04296939	0.56282030	A_07_P047598	Z5954	cmr|orf Unknown function [Z5954]
Z5987	+2.74	8.68	+1.45	7.95	0.0	9.41	0.2	0.00055434	0.00436376	0.03805386	0.49148482	A_07_P050868	Z5987	cmr|orf Unknown function [Z5987]
Z5988	+9.12	9.24	+3.19	7.64	0.0	10.83	0.1	0.00000750	0.00029103	0.00439469	0.19545198	A_07_P050871	Z5988	cmr|putative lipoate-protein ligase A [Z5988]
Z5988	+8.49	8.72	+3.09	7.18	0.2	10.26	0.2	0.00004330	0.00073378	0.00500699	0.21134048	A_07_P050871	Z5988	cmr|putative lipoate-protein ligase A [Z5988]
Z5988	+7.67	9.02	+2.94	7.55	0.1	10.49	0.1	0.00000953	0.00032326	0.00626016	0.22814815	A_07_P050871	Z5988	cmr|putative lipoate-protein ligase A [Z5988]
Z5988	+9.29	9.19	+3.22	7.58	0.1	10.79	0.1	0.00000835	0.00030273	0.00425305	0.19186246	A_07_P050871	Z5988	cmr|putative lipoate-protein ligase A [Z5988]
Z5988	+7.86	9.17	+2.97	7.68	0.1	10.66	0.1	0.00001350	0.00038499	0.00595401	0.22266033	A_07_P050871	Z5988	cmr|putative lipoate-protein ligase A [Z5988]
Z6030	+3.39	8.15	+1.76	7.27	0.3	9.03	0.2	0.00100178	0.00691416	0.02679497	0.42101796	A_07_P044196	Z6030	cmr|putative tail component of cryptic prophage CP-933P [Z6030]
Z6074	+2.72	13.20	+1.45	12.48	0.0	13.93	0.1	0.00035862	0.00317016	0.03854929	0.49423453	A_07_P044326	Z6074	cmr|unknown protein encoded by cryptic prophage CP-933P [Z6074]
zntA	+3.32	9.44	+1.73	8.58	0.0	10.31	0.0	0.00013107	0.00156505	0.02724975	0.42393281	A_07_P034561	c_4262	cmr|Lead, cadmium, zinc and mercury transporting ATPase [c_4262]
zntR	-3.27	11.13	-1.71	11.99	0.1	10.28	0.1	0.00022475	0.00230221	0.02302782	0.45149440	A_07_P052121	Z4662	cmr|putative transcriptional regulator [Z4662]
znuC	-2.62	9.10	-1.39	9.80	0.0	8.40	0.1	0.00041696	0.00352927	0.04432482	0.56967347	A_07_P015968	b1858	cmr|putative ATP-binding component of a transport system [b1858]
zraP	-2.24	9.38	-1.16	9.97	0.4	8.80	0.2	0.01038637	0.04277348	0.06730882	0.65616718	A_07_P008674	b4002	cmr|orf, hypothetical protein [b4002]
zraP	-2.38	9.32	-1.25	9.95	0.1	8.70	0.2	0.00196819	0.01150478	0.05620935	0.61755757	A_07_P047207	Z5578	cmr|orf, hypothetical protein [Z5578]
zupT	+2.03	9.17	+1.02	8.66	0.1	9.68	0.1	0.00235130	0.01313750	0.06843559	0.65458688	A_07_P012331	b3040	cmr|orf, hypothetical protein [b3040]
zwf	+2.27	9.33	+1.18	8.74	0.0	9.92	0.1	0.00099705	0.00689682	0.05409616	0.57938095	A_07_P015938	b1852	cmr|glucose-6-phosphate dehydrogenase [b1852]
