# Supplementary material for: Cellular Response to Ciprofloxacin in Low-Level Quinolone-Resistant Escherichia coli
Source: Front Microbiol. 2017 Jul 19;8:1370. doi: 10.3389/fmicb.2017.01370 (PMC5516121; doi:10.3389/fmicb.2017.01370)
Supplement: Supplementary file 5 [file Table_4.DOC]

**Supplementary Table 4. Proteomic Supplementary Data**

**Proteomic Approach Method**

***Sample Preparation***

Cultures were started from single colonies and grown overnight in 25 ml of LB. These cells were diluted 1:100 and grown to cell concentrations of 4x10^8^ cells/ml (OD_600nm_=0.4, exponential phase) for treatment. Strains were incubated at 1 μg/mL ciprofloxacin for 60 minutes. Approximately 10^9^ cells (5 ml) were taken for protein isolation (Proteomic Service, Biomedicine Institute of Seville).

***In-solution Protein Digestion***

Approximately 100 µg of proteins for each sample were reduced with 10 mM DTT for 30 min at 37 °C and alkylated with 25mM iodoacetamide for 30 min in the dark at room temperature. Trypsin digestion was carried out overnight at 37 °C in a 1:100 enzyme:protein ratio in 50 mmol/L ammonium bicarbonate. After digestion, the peptide solution was acidified with 1% TFA and desalted using HLB 1cc (10 mg) tips (Oasis; waters). Peptides were eluted with 50% ACN and 0.1% TFA.

***LC-MS/MS***

Digested peptides were analyzed by LC-MS/MS using a ThermoScientific Easy nLC-1000 in tandem with a Q-Exactive plus Orbitrap mass spectrometer. 10 μL of each sample was subject to a 120-minute gradient (0% to 45% buffer B; buffer A: 0.1% formic acid in water; buffer B: 0.1% formic acid in acetonitrile) on a 2 cm Acclaim 100 PepMap Nanoviper C18 trapping column in tandem with a New Objective PicoChip reverse-phase analytical LC column. For data dependent analysis, the top 15 most abundant ions were analyzed for MS/MS analysis while +1 ions were excluded from MS/MS analysis. Additionally, a dynamic exclusion of 30 seconds was applied to prevent continued re-analysis of abundant peptides. For the analysis, a resolution of 60,000 was used for full scans that ranged from 350 to 2000 m/z and a resolution of 30,000 was used for MS/MS analysis. For data analysis, raw data files corresponding to samples were searched using Proteome Discoverer 1.4.1.14’s SEQUEST search algorithm using the reviewed, non-redundant *E. coli K12* complete proteome retrieved from UniprotKB. Parameters were set as follows: event detector mass precision = 2ppm; spectrum selector minimum precursor mass = 350Da, maximum precursor mass = 5000Da; maximum collision energy = 1000; input data digestion enzyme = trypsin (full) with maximum missed cleavage sites = 2; precursor mass tolerance = 10ppm with fragment mass tolerance =0.01Da; dynamic modifications to peptides = oxidation of methionine (+15.995Da), static modifications to peptides = carbamidomethylation of cysteine (+57.021Da). During data processing, the ‘Precursor Ion Area Detector’ node of Proteome Discoverer 1.4.1.14’s SEQUEST workflow editor was implemented to determine the relative extracted ion chromatogram for each protein identified from the raw data. Searched results were filtered using a minimum of 2 medium confidence peptides per protein.

**Table**. Selected proteins related to ROS modulation detected by LC-MS/MS.

| **Gene** | **Protein** | **Strains** | | | | | | | |
| --- | --- | --- | --- | --- | --- | --- | --- | --- | --- |
|  |  | **ATCC 25922** | | **EC14** | | **EC19** | | **EC24** | |
|  |  | Score | Coverage (%) | Score | Coverage (%) | Score | Coverage (%) | Score | Coverage (%) |
| *sdhB* | Succinate dehydrogenase iron-sulfur subunit | ND | ND | 0 | 94,12 | ND | ND | 0 | 99,16 |
| *sdhA* | Succinate dehydrogenase flavoprotein subunit | ND | ND | 0 | 87,93 | 0 | 90,99 | 7,28 | 87,24 |
| *atpA* | ATP synthase subunit alpha | ND | ND | 0 | 84,02 | ND | ND | 2,16 | 75,63 |
| *atpD* | ATP synthase subunit delta | ND | ND | 2,8 | 93,7 | 0 | 79,8 | 5,93 | 84,13 |
| *nuoG* | NADH-quinone oxidoreductase subunit G | ND | ND | 15,06 | 83,37 | ND | ND | 0 | 79,19 |
| *sucA* | 2-oxoglutarate dehydrogenase E1 component | ND | ND | 0 | 73,10 | ND | ND | 0 | 78,35 |
| *sucC* | Succinate--CoA ligase [ADP-forming] subunit beta | 8,26 | 89,18 | 34,94 | 93,56 | 22,9 | 86,86 | 15,96 | 87,63 |
| *gnd* | 6-phosphogluconate dehydrogenase | ND | ND | 0 | 83.33 | ND | ND | 0 | 87,39 |
| *ahpC* | Alkyl hydroperoxide reductase subunit C | 0 | 65,24 | 15,5 | 84,49 | 0 | 93,58 | 5,32 | 78,08 |
| *ahpF* | Alkyl hydroperoxide reductase subunit F | 0 | 76,39 | 34,31 | 84,45 | 4,95 | 81,57 | 17,11 | 85,45 |

ND: Not Detected
